# Syndemic geographic patterns of cancer risk in a health-deprived area of England

**DOI:** 10.1016/j.puhip.2024.100552

**Published:** 2024-10-25

**Authors:** Catherine Jones, Thomas Keegan, Andy Knox, Alison Birtle, Jessica A. Mendes, Kelly Heys, Peter Atkinson, Luigi Sedda

**Affiliations:** a University Hospitals of Morecambe Bay NHS Foundation Trust, Kendal, LA9 7RG, UK; b Lancaster Medical School, Lancaster University, Lancaster, LA1 4YG, UK; c Lancaster Ecology and Epidemiology Group (LEEG), Lancaster University, Lancaster, LA1 4YG, UK; d NHS Lancashire and South Cumbria Integrated Care Board, Preston, PR1 8XB, UK; e Rosemere Cancer Centre, Lancashire Teaching Hospitals, Preston, PR 29HT, UK; f Centre for Tropical Medicine and Global Health, University of Oxford, Oxford, OX3 7LG, UK; g Science and Technology, Lancaster University, Lancaster, LA1 4YG, UK

**Keywords:** Synchronic diseases, Geospatial analyses, Joint modelling, Left-censoring, Variable selection, Common cancers, North West of England, Morecambe Bay

## Abstract

**Objectives:**

This study aims to analyse the geographical co-occurrence of cancers and their individual and shared risk factors in a highly deprived area of the North West of England to aid the identification of potential interventions.

**Study design:**

An ecological study design was employed and applied at postcode sector level in the Morecambe Bay region.

**Methods:**

A novel spatial joint modelling framework designed to account for large frequencies of left-censored cancer data was employed. Nine cancer types (breast, colorectal, gynaecology, haematology, head and neck, lung, skin, upper gastrointestinal, urology) alongside demographic, behavioural factors and socio-economic variables were included in the model. Explanatory factors were selected by employing an accelerated failure model with lognormal distribution. Post-processing included principal components analysis and hierarchical clustering to delineate geographic areas with similar spatial risk patterns of different cancer types.

**Results:**

15,506 cancers were diagnosed from 2017 to 2022, with the highest incidence in skin, breast and urology cancers. Factors such as age, ethnicity, frailty and comorbidities were associated with cancer risk for most of the cancer types. A positive geographical association was found mostly between the colorectal, haematology, upper GI, urology and head and neck cancer types. That is, these cancers had their largest risk in the same areas, similarly to their lowest risk values. The spatial distribution of the risk and cumulative risk of the cancer types revealed regional variations, with five clusters identified based on cancer type risk, demographic and socio-economic characteristics. Rural areas were the least affected by cancer and the urban area of Barrow-in-Furness was the area with the highest cancer risk, three times greater than the risk in the surrounding rural areas.

**Conclusions:**

This study emphasizes the utility of joint disease mapping by geographically identifying common or shared factors that, if targeted, could lead to reduced risk of multiple cancers simultaneously. The findings suggest the need for tailored public health interventions, considering specific risk factors and socio-economic disparities. Policymakers can utilize the spatial patterns identified to allocate resources effectively and implement targeted cancer prevention programmes.

## 1 Introduction

Geographic mapping of disease is an essential public health tool. It can be used to identify spatial patterns of disease risk, incidence, prevalence and survival as well as differences in disease diagnosis, burden and mortality across one or more regions [1]. Moreover, maps can be used for prevention and control programmes, prioritisation of intervention and services management, for example, by targeting high risk communities, but also to investigate the aetiology of a disease [2].

The advantages of disease mapping can be enhanced by employing syndemic frameworks where multiple diseases within a population that share common risk factors are modelled and mapped jointly [1]. A geographic syndemic framework is composed of two or more geographically co-occurring diseases that interact with each other and the environment (in the ecological sense) [3]. This framework requires shared risk factors or common components such as those defining the spatial variation of the diseases, and allows other risk factors or components to exist at the individual disease level (further details on syndemic joint modelling frameworks are provided in Supplementary Information S1).

Providing accurate and advanced public health tools and information to tackle cancer morbidity and mortality are particularly needed in the areas at most risk for cancer occurrence. The North West of England is one of these areas. Historically, in the scientific literature, the North West has been identified as one of the regions most affected by cancer in England (Supplementary Information S2). Within the North West, Morecambe Bay and South Cumbria, the focus of the present study, have been recognized as priority areas for public health initiatives due to poor health outcomes for some portions of their populations. Morecambe, a town of almost 33,000 inhabitants within the Morecambe Bay and South Cumbria area, was a case study in a recent report on coastal towns [4]. The research found that 20 % of people smoke in Morecambe (16.6 % nationally). Further, residents have high rates of hospital admission for alcohol-related harm, and are more likely to have hypertension or depression than the national average, with a quarter having a limiting, long-term illness or disability, significantly greater than the national average. In addition, Morecambe has worse values for all emergency hospital admission indicators, and higher standardised mortality ratios for all ages. According to the same study, rates for lung cancer, peripheral artery disease, COPD, dementia, stroke, coronary heart disease, kidney disease, epilepsy and diabetes are greater than the national averages. Finally, deprivation rates are significantly worse than the England average.

Given the above context, the research questions that we aimed to answer were.

(i) What are the most important cancers, in terms of incidence, in the Morecambe Bay region?
(ii) What are the most prevalent risk factors?
(iii) How do cancers cluster geographically and associate to each other?
(iv) How can the identification of risk from geographically co-occurrent cancers inform cancer interventions?

To answer these questions, we applied an ecological study design and geospatial methods to analyse cancer incidence from the Morecambe Bay Region for the 2017–2022 period.

## 2 Methods

### 2.1 Study area

**Fig. 1:**
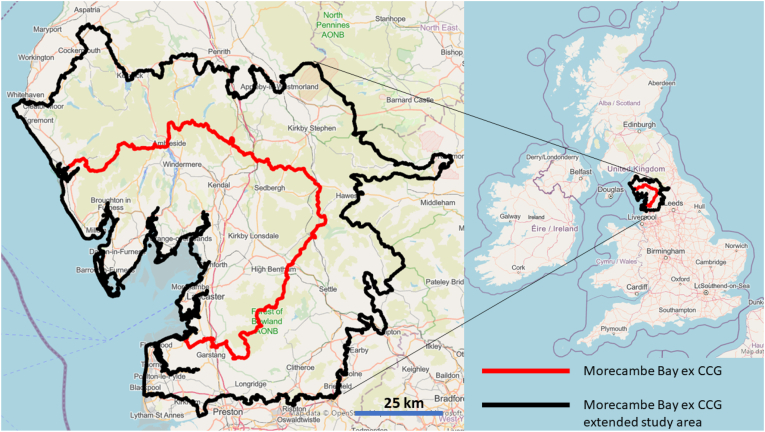
Morecambe Bay ex CCG (red border) and study area (black border). Basemap from openstreetmap under the Open Database licence (https://www.openstreetmap.org/copyright), Microsoft, Facebook Inc and its affiliates, and Esri Community Maps contributors. Map layer by Esri. (For interpretation of the references to color in this figure legend, the reader is referred to the Web version of this article.)

The study area is Morecambe Bay in the North West of England. The Morecambe Bay study area was defined using the limits of the Morecambe Bay ex Clinical Commissioning Group (CCG, Fig. 1, red border) to simplify data acquisition and homogenisation. CCGs formed the lowest level of the health geography hierarchy in England which was introduced by the Health and Social Care Act 2012, but abolished by the Health and Care Act 2022 (hence, use of the prefix ‘ex’). Since patients were recruited from a larger region (Fig. 1, black border), the study area was extended to include these patients.

In 2012, Morecambe Bay ex CCG had a population of 334,287. The Index of Multiple Deprivation of 2019 ranked Morecambe Bay as CCG 99th out of 191, but 53rd for its proportion of Lower Super Output Areas (LSOAs) in the 10 % most deprived LSOAs in England (https://www.gov.uk/government/statistics/english-indices-of-deprivation-2019). A lower super output area typically contains between 400 and 1200 households and a resident population of between 1000 and 3000 residents (https://www.ons.gov.uk/methodology/geography/ukgeographies/censusgeographies/census2021geographies).

The age distribution of Morecambe Bay ex CCG is reported in Supplementary Fig. S1.

### 2.2 Study design and data collection

An ecological study design was used for this research. The primary outcome is the 6-months new cancer diagnosis count by cancer type from January 2017 to December 2022 (for a total of 12 temporal measurements). The data were extracted in August 2023. The geographic unit of analysis is the postcode sector which includes the first part of the postcode (postcode district or outward code), the single space, and the first character of the second part of the postcode (inward code) (https://www.ons.gov.uk/methodology/geography/ukgeographies/postalgeography), for a total of 75 postcode sectors. The same data were also provided at Local Authority district level, for a total of 13 districts. The data were provided by the University Hospitals of Morecambe Bay Trust for the Morecambe Bay ex CCG extended study area (Fig. 1). The patients included were those that were referred to the trust through the Somerset Cancer Registry (SCR), maintained at the Taunton and Somerset NHS Foundation Trust, Crown Industrial Estate, Taunton, Somerset, UK (https://www.somersetft.nhs.uk/somerset-cancer-register/).

Cancers were classified into different types by their anatomic location (site) [5]. Nine cancer types were considered in this study: lung, skin, breast, colorectal, haematology, upper gastrointestinal (upper GI), urology, head and neck and gynaecology (a brief overview on these cancers is provided in Supplementary Information S3). Sarcoma, brain and central nervous system cancers were excluded because they were fully censored, and imputation of values was not possible with the statistical model used here. The ICD10 Codes used to identify each cancer by its tumour site are reported in Supplementary Table S1.

### 2.3 Censored cancers

The postcode sector and Local Authority district count data used here, of cancer new diagnosis by type, were censored, with values less than or equal to 5 but greater than 0 replaced by the words ‘less than or equal to 5’, for confidentiality reasons. Therefore, the number of people with a specific cancer type was known in each postcode sector and local authority and set to a range when below or equal to 5 and greater than 0, to remove patients' identifiability. The level of censoring for each cancer type is provided in Supplementary Table S2. Censoring was reduced by integration of postcode sector and Local Authority data, as described in Supplementary Information S4. The remaining censored locations were inputted during the inferential process by making statistical assumptions on the distribution of the missing data (see Inference and prediction section below).

### 2.4 Exposure and predictor variables

Demographic, socio-economic and behavioural factors aggregated by postcode sector and provided as counts of patients by cancer type were obtained from the University Hospital of Morecambe Bay Trust. Additional variables from public datasets and available at postcode (i.e. crime), Local Authority (i.e. UK Census 2011) and LSOA (i.e. the Place-Based Longitudinal Data resource) were geographically merged with the cancer dataset. The full list of the 783 variables included in the analyses is available in Supplementary Table S3, and additional information about them is provided in Supplementary Information S5. We do not assume causation between any of these 783 variables and cancer types, but rather evaluate associations that are significant for mapping the individual and joint cancer risk. All the variables considered in this analysis have been linked to deprivation and/or cancers by other studies (e.g. Ref. [6]). In addition, we decided to use granular, fine ethnicity groups to account for potential differences in risk factor prevalence among ethnic minority groups that can be informative for targeted interventions [7].

### 2.5 Descriptive statistics

Due to the high proportion of censoring in the cancer data, it was not possible to provide statistics to describe the demographic (age, sex, ethnicity), socio-economic and clinical characteristics (comorbidities and frailty) of the real study population. Post-analyses statistics were provided as crude cancer rates by cancer type, and age-adjusted rates (age-specific incidence rate) averaged by postcode sector and 6 months period. We considered two age groups, 0–50 and 50+ because the level of censoring did not support creating multiple age thresholds. A single age threshold was used in other cancer research [8,9]. The standard errors of the incidence and age-adjusted incidence rates were calculated using the Poisson approximation method [10].

### 2.6 Variable selection

Using a Bayesian joint model for variables selection would have required exceptionally lengthy computations. For example, a single model with 20 predictors and 10,000 MCMC iterations requires around 200 h of computation. During variable selection, thousands of models need to be tested with different numbers of variables (potentially modelling all 783 variables) and this would have caused millions of hours of computation using a Xubuntu system 16GiB system memory, and Intel(R) Core(TM) i7-4790 CPU @ 3.60 GHz. For this reason, we employed a deterministic method that can account for censored and clustered data within the entire cancer dataset. The stepwise selection method applied to an accelerated failure model with lognormal distribution [11] is described in Supplementary Information S5.

### 2.7 Inference and prediction

The joint modelling of two or more cancers allows the identification of shared and divergent trends among the cancers in terms of geographic patterns and risk factors. Rather than treating a cancer as a proxy for unmeasured risk factors affecting another cancer, the proposed model treats the different cancer types symmetrically and assumes that the area-specific relative risks of each cancer depend on a shared latent component plus additional latent components specific to one or other cancer types [12,13]. The joint model employed in this study is, thus the shared component model [14] for Poisson distributed data. The shared component is represented by the spatial covariance (i.e. a function representing the variation in the cancer types in space) which is assumed to vary independently from the risk factors considered in the model. During the maximum likelihood estimation process the joint model inputs values to the censored records within the estimated censored ranges (which can differ among postcode sectors, see Supplementary Table S2) and observed cancer counts are modelled simultaneously with the censored data. A full description of the model is provided in Supplementary Information S6.

Inference and predictions were made at the LSOA level. The outcome variables were the nine censored cancers and the predictors were the selected important variables that represent the frequency of that condition or characteristic in the cancer diagnosis in each LSOA unit. The joint modelling outputs mapped at LSOA level were.

1) cancer risk by type for all ages, over 50 years old and 50 years old or under;
2) cancer cumulative risk by number of cancers for all ages;
3) geographical correlation (co-regionalisation) between pairs of cancer types;
4) geographic clusters of cancers.

All the outcomes are given in terms of model posterior means [15]. Uncertainty is represented by the posterior standard deviation of the outcome or parameter of interest, with smaller values indicating greater certainty in the inference of the parameter or the prediction of the outcome. To facilitate comparisons, all maps are shown on the same scale [16]. Finally, we conducted a mediational analysis [17] to examine the effects of including each of the potential mediating variables in the individual-level cancer type generalised linear models on the odds ratios associated to each selected variable.

### 2.8 Model performance and validation

Model performance was assessed for each individual cancer type through the mean error and mean squared error difference between the observed and predicted outcomes. To account for the complex parameterisation of the joint model and to evaluate the global and individual (i.e. the individual models for each cancer type of the joint model) predictive accuracy we used two information criteria: Deviance Information Criterion (DIC) and Watanabe-Akaike Information Criterion (WAIC). Both measures are designed for Bayesian analyses. However, the WAIC averages over the posterior distribution rather than conditioning on a point estimate as in the DIC [18]. The WAIC often produces values with small differences between models with similar structure. Therefore, we decided to report both the WAIC and DIC estimates to provide evidence of consensus between the two statistics.

The joint model was compared with nine independent (one for each cancer type) spatial linear models optimised via stochastic approximation of the expectation-maximization algorithm (SAEM) as proposed in Ref. [19]. We used the same variables employed in the joint model as predictors. The spatial covariance function for each model (or cancer type) was estimated through manual fitting [20]. To evaluate the predictive capability of the independent spatial linear models, we compared the errors between the predicted and real total counts of each cancer type.

Finally, the robustness of the joint model was assessed using cross-validation, by leaving out 10 % of the data for each cancer type. Validation assessment was undertaken by measuring the Root Mean Square Error and the Mean Squared Deviation Ratio [21].

### 2.9 Clustering

Principal components analysis (PCA) was used to identify co-regionalisation between cancers and geographic cancer clusters. PCA was employed on the posterior predictions of the latent variable for each cancer type. Based on the first two principal components (explaining the greatest variability) a centroid hierarchical cluster analysis [22] was employed to cluster the LSOAs based on similarity of the cancer patterns.

### 2.10 Software

All the analyses were performed in the R-cran software [23] using various packages and the authors’ written codes for model inference, predictions, and mapping.

## 3 Results

Between January 1, 2017 and December 31, 2022, the University Hospitals of Morecambe Bay Trust recorded 15,506 cancer diagnoses in the Morecambe Bay ex CCG extended area. There were 4599 skin, 2450 urology, 2076 breast, 1606 colorectal, 1535 lung, 1039 upper GI, 992 haematology, 670 gynaecology, and 539 head and neck cancers (Supplementary Table S4). Male breast cancer was 0.7 % of the breast cancer total. Our model predictions were accurate with 15,243 estimated cancers (an error of 1.7 %) (Supplementary Table S4). However, the accuracy was reduced for upper GI (9.2 % underestimation) and, to a lesser extent, relatively high for lung cancer (2.3 % overestimation). Rates for breast, colorectal, haematology and urology cancers were larger in Morecambe Bay ex CCG extended area than in the North West and England as a whole (Supplementary Table S4).

As expected, all cancer types had a larger value of incidence in the over 50 years old group compared to the 50 years old and under group, with around a 20-fold difference for lung and upper GI; around 15-fold for skin and urology; and 11-fold for colorectal cancers. The largest incidence was in skin cancer, followed by urology, breast, colorectal and lung (all above 1 new case per 1000 people per postcode sector every 6-months) (Supplementary Table S5). Geographically, four postcode sectors experienced all 12 cancer types between 2017 and 2022; 72 % of postcode sectors had at least 10 cancer types during the same period (Supplementary Table S6).

Variable selection reduced the initial 783 variables to 23. These variables can be grouped into four domains: demographic (age, frailty, ethnicity and comorbidities), behavioural (smoking status), socio-economic (employment) and time. Despite the absence of the gender variable and excluding time, all the remaining domains are commonly associated with incident diagnoses of cancer [24]. Summary statistics for these 22 variables (time excluded) are provided in Supplementary Table S7.

Seventeen out of 23 factors were risk factors for one or more cancer types, with comorbidities such as ‘chronic kidney disease’ and ‘COVID19’ being statistically significant factors for six out of nine cancer types – associated to higher risk of diagnoses for five and four cancer types, respectively (Table 1). ‘Age above 80’, ‘depression’ and ‘congestive heart failure’ were risk factors and protective factors for the same number of cancer types; while being ‘fit’ or ‘self employed with one parent working’ or ‘northern Irish’ were mostly protective factors.

**Table 1:**
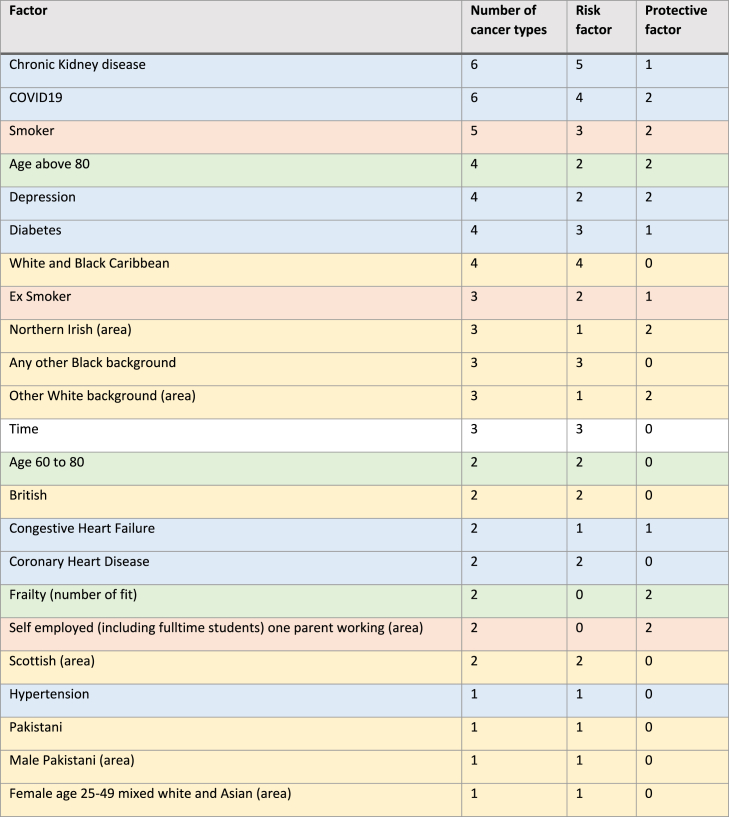
Summary of the number of associations between selected variables and cancer types (comorbidities in blue, ethnicity in yellow, behavioural in orange, and age and frailty in green). Note that the term ‘Protective factor’ does not have a biological or medical meaning, but rather is an epidemiological term to represent a negative association between the factor and the likelihood to be diagnosed with cancer. Time accounts for the presence of temporal trends. When Time is a risk factor, it means that from 2017 to 2022 there was an increase in cancer risk for those cancers that had time as important factor (breast, colorectal and urology).

The odds ratios and relative credible intervals of the statistically significant variables for each cancer type are provided in Supplementary Information S7 and further results and analyses in Supplementary Information S8. Credible intervals obtained from Bayesian frameworks account for the uncertainty originating from zero-inflated variables and their lack of statistical power, in addition to uncertainty deriving from model priors and parameterisation [25]. As described in Supplementary Information S8, it is likely that some associations are proxies for personal or areal-related risk factors not captured in this analysis. For example, the association between ‘any other black background’ and skin cancer is likely to represent the characteristics of areas where this group lives than a larger incidence of skin cancer in this minority group [26].

Risk and co-regionalisation maps are shown in Supplementary Information S9 and S10. Guidance on interpretation of these maps is provided in Supplementary Information S12. In Supplementary Information S9, the maps for risk of a cancer type in the Morecambe Bay ex CCG extended study area and more specifically for three regions (Morecambe and Lancaster, Barrow in Furness and Kendal) are presented alongside their uncertainties. In these maps, areas indicated with low uncertainty risk are those for which the prediction is more accurate. LSOAs without colour are those where cancers were not recorded during 2017–2022. For all cancer types, the North Cumbria and Forest of Bowland areas were generally at low risk, apart from Bentham (in the latter) although this area was associated with large uncertainty. Locally, Morecambe and Barrow-in-Furness suffer the highest incidence of cancers, especially in areas such as Vickerstown (Barrow) and Torrisholme (Morecambe), which had all cancer types in the top ten of the areas ranked risks.

When adjusted by age (Supplementary Information S10), one LSOA (located in Westgate/White Lund in Morecambe) was in the top ten LSOAs for the risk of six of the cancer types in the under 50 years old group. For the over 50 years old group, two LSOA areas were consistently in the top ten LSOAs for the risk of nine cancer types: Bowerham south and Freehold West in Lancaster.

**Fig. 2:**
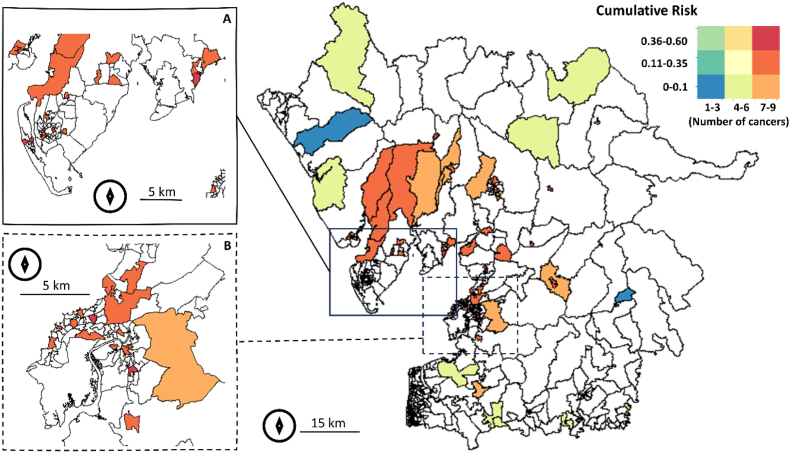
Cumulative risk for the number of cancer types for the Morecambe Bay ex CCG extended area (main) zoomed to (A) Barrow-in-Furness and (B) Morecambe and Lancaster. White polygons show no cases of cancer types during 2017–2022. Map created in R (sf and sp packages).

Fig. 2 presents the cumulative risk for the number of cancer types, with the maximum found in urban areas. Areas such as the Forest of Bowland, Yorkshire borders and Windermere West exhibit the presence of most cancer types, but with low cumulative risk (less than or equal to 10 % or, in lay terms, one in ten people was likely to get one of the nine cancer types during the 2017–2022 period). With the same number of cancer types, some areas in Morecambe, Lancaster and Barrow-in-Furness had up to six times more cumulative risk than Windemere West, Burton in Lonsdale or Quernmore.

**Fig. 3:**
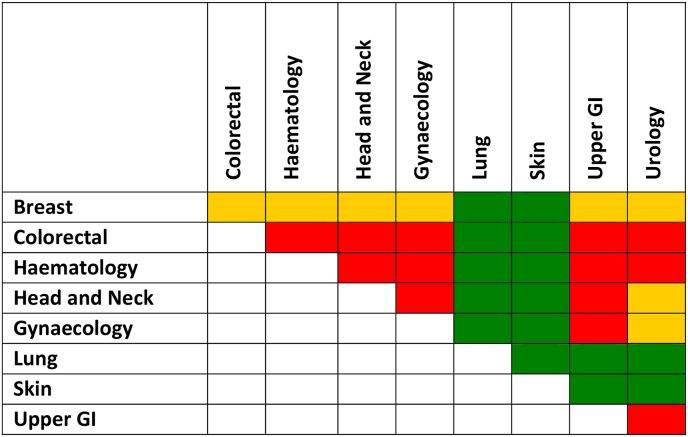
Median correlation between posterior samples of cancer types in the Morecambe Bay ex CCG extended area. **Orange = no correlation**; **green = negative correlation** (areas with high incidence in one cancer type have low incidence in the other cancer type); and **red = positive correlation** (both cancer types have high incidence in the same area). The correlation threshold is arbitrary: negative correlation is lower than −0.3; positive correlation is above 0.3 and no correlation (or weak correlation) is between −0.3 and 0.3. (For interpretation of the references to color in this figure legend, the reader is referred to the Web version of this article.)

In terms of co-regionalisation, lung and skin cancer types were negatively correlated (spatial patterns not coincident) between themselves and between each of them and the rest of the cancer types (Fig. 3). Apart from the negative associations with the lung and skin cancer types, breast cancer had no significant associations with any other cancer type. A positive association was mostly found between the colorectal, haematology, upper GI, urology and head and neck cancer types.

Spatially, most of the associations were homogeneous over the region, but not all. For example, between the colorectal, haematology, head and neck, gynaecology, upper GI and urology cancer types some areas exhibited no association (e.g. Windemere), while being contiguous with areas with positive association (e.g. Windemere West) (Supplementary Information S11).

**Fig. 4:**
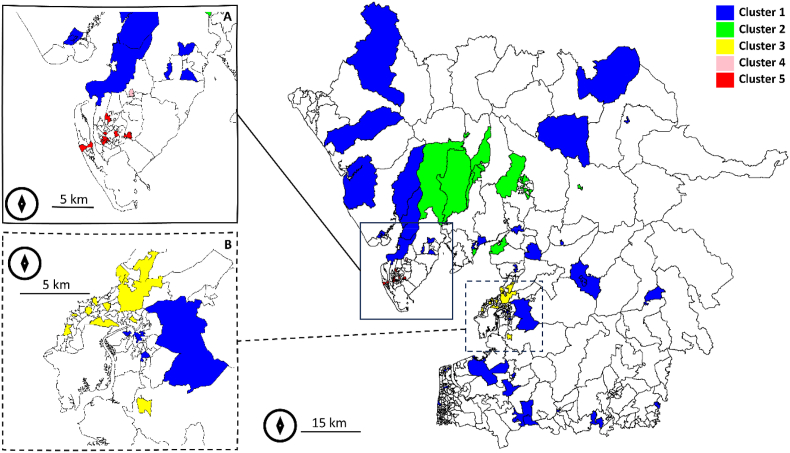
Cancer type risk clustering by LSOA for the Morecambe Bay ex CCG extended area (main) zoomed to (A) Barrow-in-Furness and (B) Morecambe and Lancaster. White polygons were not clustered due to the absence of cases of cancer types during 2017–2022. Map created in R (sf and sp packages).

As described in the methods section, the first two principal components from the PCA of the posteriors for the nine cancer types were employed in conjunction with a centroid hierarchical clustering to cluster the LSOAs in the Morecambe Bay ex CCG extended area. The first two principal components explained 91 % of the variance (64 % PC1 and 27 % PC2). The centroid hierarchical clustering identified five clusters (Fig. 4).

• Cluster 1 (rural). Large rural LSOAs with large, but generally younger population. Low level of comorbidities and smoking. Generally cancer risk is low.
• Cluster 2 (Windemere East to Coniston). Similar to Cluster 1, but with an older population and larger proportion of British ethnic group individuals compared to other clusters. High number of cancers with low-to-moderate risk.
• Cluster 3 (Morecambe and Lancaster). This urban cluster is characterised by a high level of unemployment, and a high proportion of chronic diseases and mental health conditions. High number of cancers with moderate-to-high risk.
• Cluster 4 (Dalton-in-Furness). This isolated cluster presents a high level of comorbidities in a generally younger population than the study area as a whole. High number of cancers with moderate risk.
• Cluster 5 (Barrow-in-Furness). This is the cluster with the highest cancer incidence. The local population is affected by a high level of comorbidities, lower population density and a high level of smoking. High number of cancers with moderate-to-high risk.

Model validation results indicate a precise joint model with a mean squared error ranging from around 10 cases for lung cancer type to 30 cases for upper GI cancer type, over the six years’ time period (Supplementary Information S13). In comparison with individual independent spatial linear models, the joint cancer count predictions were in general more accurate than in the independent models, but not for skin cancer where the independent model error was only 4 % (7 % in the joint model) (Supplementary Table S4). It is important to highlight that the largest accuracy in the independent models was achieved for the cancers in the dataset with the largest availability of data (and, therefore, the smallest number of censored records).

## 4 Discussion

This is the first population-based study to investigate spatial patterns in multiple cancers in the Morecambe Bay area and quantify how the different cancers contribute to the cumulative risk in its geographic unit.

*What are the most important cancers, in terms of incidence, in the Morecambe Bay region?*

The rates for the breast, colorectal and urology cancer types in the Morecambe Bay ex CCG extended area were above the rates for England and the North West for the 2017 and 2016–2018 periods, respectively (however, see ‘Limitations’ for potential screening effects). The major difference was in urology cancer type with 14 new cases per 100,000 people per year more than in the North West and 24 new cases more than in England. In contrast, the rates for the lung, skin and upper GI cancer types were generally below the North West and England rates. Between these, skin cancer had 25 new cases per 100,000 people per year fewer than the rest of the North West. The incidence rates were driven by the over 50 years old population since the incidence rates for the general population and those for the over 50s had the same ranking of cancer types. In the under 50 years old population, skin cancer type remained the most common cancer followed by breast cancer instead of urology, the second cancer for incidence in the over 50s. Also, in the under 50 group the least common cancer type was upper GI instead of head and neck cancer whose incidence was the lowest in the over 50 group.

*How do cancers cluster geographically and associate to each other?*

The nine cancer types considered in this research were co-regionalised in three quarters of the Morecambe Bay ex CCG extended area for the LSOAs where cancers were found between 2017 and 2022. In fact, 19 % of the LSOAs had only one cancer type during the study period. Apart from co-occurring, cancers tend to associate geographically with positive associations (co-occurring with high risk) or negative associations (where one cancer has high risk, the other has low risk) [27].

Spatial heterogeneity was confirmed by the presence of geographic clusters for cancers risk. Although the clustering analysis was affected by a level of uncertainty due to the use of only two principal components, a clear pattern emerged: the cancer risk for Barrow-in-Furness (identified cluster 5) was three times greater than for the surrounding rural areas (cluster 1) and higher than Morecambe and Lancaster urban centres (cluster 3).

*What are the prevalent risk factors?*

The use of a large number of candidate factors (783), and the selection of 23 of them, indicates that individual-level characteristics and area-level socio-economic characteristics were far from providing a comprehensive explanation for the observed spatial heterogeneities in cancer types [28].

Chronic Kidney Disease and COVID19 were the most important factors (speculatively, COVID19 may have increased the cancer diagnosis rate once people were hospitalised), both being associated to six of the nine cancer types, followed by ‘current smoker’ associated to five of the nine cancer types included in the joint modelling. Overall, comorbidities (seven risk factors) and ethnicities (eight risk factors) were the most important factors associated to cancer type counts. Extended discussion on risk factors is provided in Supplementary Information S14.

*How identified syndemic geographic patterns can inform cancer interventions?*

A positive spatial correlation can support the theory of shared environmental aetiology [29], although differences in risk factors selected for each individual cancer type suggest that this is not the only relevant component [2]. Upper GI and gynaecology, upper GI and head and neck, and upper GI and urology correlations were the most widespread positive associations. This is likely attributable to the fact that these cancers share risk factors such as alcohol consumption, ethnicity, obesity and smoking, but also infectious diseases (https://www.cancerresearchuk.org/about-cancer/type). This reinforces the ‘integrating intervention’ paradigm, where interventions are designed to reduce and eliminate multiple cancers instead of tackling individual cancers especially when the common risk factors are identified [30,31]. The latter was a designed-in goal of this research, which aimed not only to select the risk factors for each cancer type, but also to find areas with correlated residuals (or in other words residual patterns of co-occurrence) [32] which are indicative of the presence of factors or biological processes associated to both cancers, but not considered in the linear fixed effect (or trend) of the model [33].

Policymakers may potentially use our spatial results, as well as the joint distribution modelling approach demonstrated here, to aid studies and analysis for resource allocation and education in holistic public health interventions and programmes targeted to reduce the burden from geographically correlated and co-regionalised cancer types [27]. The results may aid policymakers to take action on common risk factors, deploy further research for hidden common risk factors or prioritise cancers that are on the rise (as we found for the breast, colorectal and urology cancers) [12]. Some cancer types were found not to be associated to others or were negatively associated (breast, skin, lung) meaning that cancer type-specific interventions may be the most appropriate, and have greater impact, for these cancer types.

Most of the factors associated to cancer risk by type were grouped by ethnicity and comorbidities, which may help health commissioners and policymakers to consider health equality in different geographical regions and reduce health inequities in ethnic minority groups [24], but also to potentially include comorbidities in screening programmes [34]. This complies with the goals set by the NHS Long Term Plan (https://www.longtermplan.nhs.uk/areas-of-work/cancer/#:~:text=Our%20NHS%20Long%20Term%20Plan,more%20following%20their%20cancer%20diagnosis) which aims by 2028 to reach a target of 55,000 people each year who will survive for five years or more following their cancer diagnosis. The Plan set up the actions to improve and extend screening (including lung screening pilots) and reach ethnic minority backgrounds. However, focusing only on individual factors may not be beneficial if the healthcare system is not improved as well [35] and, in this sense, our maps of cumulative risk versus number of cancer types may inform on the necessity for expertise and facilities in response to the complexity of local cancer type dynamics.

Identifying regional and subregional inequalities is essential for the distribution of resources [36]. Some cancers were localised. Therefore, while the rates may be low generally, some communities may experience larger than expected risks. This requires further investigation into the causes, and possible interventions. In the long-term, reducing the socioeconomic variation in incidence should have a substantial impact on the burden of cancer [9] and fulfil the goal of universal health coverage by targeting the most vulnerable members of society first [37]. Mobile services such as ‘stop smoking services’, ‘drink aware’ and ‘low dose CT scan’ (the latter used in US) could be successful in reaching hard-to-reach populations and improving their health [38]. These interventions will benefit non-cancer diseases also (such as diabetes and heart disease) due to the common risk factors shared between cancers and comorbidities. It is also essential to enhance health literacy and reduce patients' misconceptions about cancer screening in high-risk areas [39,40].

### 4.1 Limitations

The following limitations affected the present study.

• Each cancer type is effectively a collection of different cancers (Supplementary Table S1) which may present different detection and under-detection rates (e.g., non-melanoma skin cancer versus melanoma skin cancer). For example, prostate cancer has the greatest incidence of urological cancers, but urothelial cancer may present the largest mortality threat.
• Late notification in the cancer registry is a potential issue for any cancer database. However, we have not found significant evidence of late notifications, as the number of records for the latest period (July–December 2022) is comparable to those recorded in the same periods of 2017, 2018, 2019, and 2020. The only exception is 2021, where there were 17 fewer records in 2022, representing a minor difference of 4 %. Generally, late notifications can introduce biases by affecting the accuracy of incidence rates and temporal trend estimations.
• Data on cancer stage at diagnosis were not available at the time of the analyses and therefore, the relative proportion of early-stage cancers compared to late-stage cancers is not known in our dataset. This means that some incidence differences may be affected by early/late diagnosis and screening uptake instead of the other risk factors considered in this analysis [41]. The potential effect of inflated rates due to cancer screening programmes for breast, cervical and bowel cancer (the three cancer screenings active in England) cannot be determined. However, regional and national data (Supplementary Table S8) suggest no significant difference between and within (tested by test of two proportions) England, the North West and Lancashire, in cancer rates and the percentage of early stage cancers obtained from screening for breast and cervical cancers.
• Undiagnosed levels were not considered, but are likely to be clustered in space [42] and, therefore could have biased the relative cancer type geographical pattern.
• Border effects: some patients within the extended portion of the Morecambe Bay ex CCG may have chosen to go to a different CCG if they lived closer to it. This could have created a potential bias in the most peripheral areas of the study region.
• Identification of important factors was carried out for all cancers together. While this promotes shared risk factors, it may reduce the significance of risk factors for less common cancers.
• Some factors may be affected by reverse causation (e.g., depression and cancer) although this analysis focused on new diagnosis instead of cancer prevalence, which should have reduced this risk.
• Some maps show high levels of uncertainty (standard errors), often based on very small numbers and, therefore, interpretation should be done cautiously. As described by Ref. [43], mapping and interpreting cancer incidence rates faces three major hurdles: the presence of unreliable rates that occur for sparsely populated areas and/or rare cancers, the visual bias caused by the aggregation of health data within administrative units of widely different sizes and shapes [44], and the mismatch of spatial supports for cancer rates and explanatory variables that prevent their direct use in correlation analysis [45].
• Postcode sector of patient residence is related to the last known address and, therefore, local factors may or may not be involved in the development of the cancer since cancers have a complex aetiology and long latency [1,46].

Despite the limitations posed by sparse and censored data as detailed above, which introduced significant uncertainties in certain areas, these uncertainties were largely mitigated by the robust joint model developed here and designed to reduce them.

## 5 Conclusions

Striking geographical, socioeconomic, behavioural and demographic variations were observed in Morecambe Bay ex CCG extended area in relation to nine cancer types during the 2017–2022 period. The joint distribution model provided a richer and more accurate perspective on spatial variation in disease risk than a standard spatial linear disease model by quantifying the different geographic levels of association between cancers, and between cancers and explanatory factors. Importantly, the joint analysis (i) identified three out of nine cancer types, namely breast, colorectal and urology, with incidence rates above the regional and national rates, (ii) underlined the associations of different cancer types with ethnicity, comorbidities and socio-economic conditions that are intrinsically dependent on the local geography (crime, mental health, demographics) and (iii) highlighted the heterogeneity in cancer risk and cancers cumulative risk which resulted in an urban-rural divide, with the risk in Barrow in Furness threefold higher than the surrounding rural areas or similarly deprived locations such as Morecambe. The results illustrate how joint distribution modelling and mapping can help to quantify the spatial distribution of the cancer burden for different cancers in an area of interest to inform etiologic debate about the specific causes of disease, aid policy formulation and inform possible resource allocation. For example, the approach demonstrated here could be used to help policymakers consider comorbidities in screening programmes and/or develop integrated interventions for cancers. As such, this study calls for, and provides the roadmap towards, more geographic-specific cancer and multi-cancer analyses to better control these diseases within at-risk communities.

## Software

The joint modelling analysis was performed using a modified version of the MCMC software written by Arnab Hazra available at https://github.com/arnabstatswithR/Arsenic-contamination-mapping.

## Contributors

LS, AK, 10.13039/100024877AB, 10.13039/100014294KH and 10.13039/100006131PA secured the funding for this study. LS is the project lead and the guarantor of this study. LS, AK and AB conceived the study. CJ acquired and manipulated the cancer data with input from KH. JAM obtained the ecological open source data, and led the ethical approval. LS conducted the literature review, designed the statistical analysis plan, with methodological input from PA and TK. LS performed statistical analyses and prepared the maps, tables and figures. All authors contributed to the interpretation of the findings. LS drafted the whole manuscript. All authors read and commented on earlier drafts, contributed to revision of the manuscript, approved the final version of the manuscript, and had final responsibility for the decision to submit for publication.

## Declaration of competing interest

The authors declare that they have no conflict of interest.
