# Supplementary material for: Syndemic geographic patterns of cancer risk in a health-deprived area of England
Source: Public Health Pract (Oxf). 2024 Oct 25;8:100552. doi: 10.1016/j.puhip.2024.100552 (PMC11564078; doi:10.1016/j.puhip.2024.100552)
Supplement: Multimedia component 1 [file mmc1.pdf]

Syndemic Geographic Patterns of Cancer Risk in a Health-Deprived Area of England

Supplementary files (omnibus)

To navigate between Supplementary Files please use the Bookmarks tab.

Supplementary Information S1: Syndemic frameworks and joint modelling

Cancers are an ideal candidate disease complex to be analysed by syndemic frameworks due to the shared risk factors of different cancer types (socio-demographic, anthropometric and lifestyle factors), and their common empirical geographical patterns (1, 2). Methodologically, syndemic frameworks can be analysed by employing joint models whose joint component can include the risk factors affecting the average risk (named fixed effects) of the cancers and/or variables influencing the variability of the cancers (named random effects, as for example, spatial, temporal and spatial–temporal autocorrelations) caused by the dependency and association between measurements in space and/or time (3). It has been shown in other studies that joint modelling results in better performance than individual models with an independent set of random effects (4, 5). Shared random effects can identify geographic areas with unmeasured or unknown risk factors that are common for different cancers among multiple population groups for gender, ethnicity and age distributions (4).

Given the complexity of relationships between demographic, behavioural and socio-economic conditions, and cancer morbidity measures, evaluation of geographical risk must consider the local scale factors influencing cancer dynamics. Variation within the study region, apart from revealing important local differences in risk can, in turn, provide aetiological clues. In the present study, we developed a Bayesian hierarchical joint spatial analysis for left-censored counts of newly diagnosed cancer types (censored to remove patients' identifiability), provided for each postcode sector, to investigate the variation in each cancer risk, joint cancer risk and cancers' correlations, across the Morecambe Bay region in the North West of England.

References

1. Jahan F, Duncan EW, Cramb SM, Baade PD, Mengersen KL. Multivariate Bayesian meta-analysis: joint modelling of multiple cancer types using summary statistics. Int J Health Geogr. 2020;19(1):42.
2. Gomez-Rubio V, Palmi-Perales F, Lopez-Abente G, Ramis-Prieto R, Fernandez-Navarro P. Bayesian joint spatio-temporal analysis of multiple diseases. Sort-Stat Oper Res T. 2019;43(1):51-74.
3. Król A, Ferrer L, Pignon J-P, Proust-Lima C, Ducreux M, Bouché O, et al. Joint model for left-censored longitudinal data, recurrent events and terminal event: Predictive abilities of tumor burden for cancer evolution with application to the FFCD 2000–05 trial. Biometrics. 2016;72(3):907-16.
4. Wah W, Ahern S, Earnest A. A systematic review of Bayesian spatial-temporal models on cancer incidence and mortality. Int J Public Health. 2020;65(5):673-82.

5. Kouame RMA, Edi AVC, Cain RJ, Weetman D, Donnelly MJ, Sedda L. Joint spatial modelling of malaria incidence and vector's abundance shows heterogeneity in malaria-vector geographical relationships. Journal of Applied Ecology. 2023.

Supplementary Information S2: Cancers in the North West of England

The North West of England had the highest oral cancer incidence rates in the period 1990-1999 (1); the highest uveal melanoma admission rates between 1999-2010 (2) with Lancashire also experiencing the highest cutaneous melanoma; the second highest glioblastoma (a fast growing type of brain tumour) incidence and the lowest survival in 2007-2011 (3); higher incidence (2008-2010) and mortality (2009-2011) rates, compared to England, of head and neck cancer in the Merseyside and Cheshire region (4); and finally the lowest incidence of basal cell carcinoma (the most common skin cancer) between 2004 and 2010 (5). Compared with London, the North West of England had a higher incidence rate for liver cancer subtypes hepatocellular carcinoma and intrahepatic cholangiocarcinoma between 2008 and 2018 (6); while compared to the whole UK, the North of England had the highest rates of lung cancer in non-smokers for men and women during the period 1998 to 2007 (7).

References

1. Conway DI, Stockton DL, Warnakulasuriya KA, Ogden G, Macpherson LM. Incidence of oral and oropharyngeal cancer in United Kingdom (1990-1999) -- recent trends and regional variation. Oral Oncol. 2006;42(6):586-92.
2. Keenan TD, Yeates D, Goldacre MJ. Uveal melanoma in England: trends over time and geographical variation. Br J Ophthalmol. 2012;96(11):1415-9.
3. Brodbelt A, Greenberg D, Winters T, Williams M, Vernon S, Collins VP, National Cancer Information Network Brain Tumour G. Glioblastoma in England: 2007-2011. Eur J Cancer. 2015;51(4):533-42.
4. Taib BG, Oakley J, Dailey Y, Hodge I, Wright P, du Plessis R, et al. Socioeconomic deprivation and the burden of head and neck cancer-Regional variations of incidence and mortality in Merseyside and Cheshire, North West, England. Clin Otolaryngol. 2018;43(3):846-53.
5. Musah A, Gibson JE, Leonardi-Bee J, Cave MR, Ander EL, Bath-Hextall F. Regional variations of basal cell carcinoma incidence in the U.K. using The Health Improvement Network database (2004-10). Br J Dermatol. 2013;169(5):1093-9.
6. Liao W, Coupland CAC, Innes H, Jepsen P, Matthews PC, Campbell C, et al. Disparities in care and outcomes for primary liver cancer in England during 2008-2018: a cohort study of 8.52 million primary care population using the QResearch database. EClinicalMedicine. 2023;59:101969.
7. Rait G, Horsfall L. Twenty-year sociodemographic trends in lung cancer in non-smokers: A UK-based cohort study of 3.7 million people. Cancer Epidemiol. 2020;67:101771.

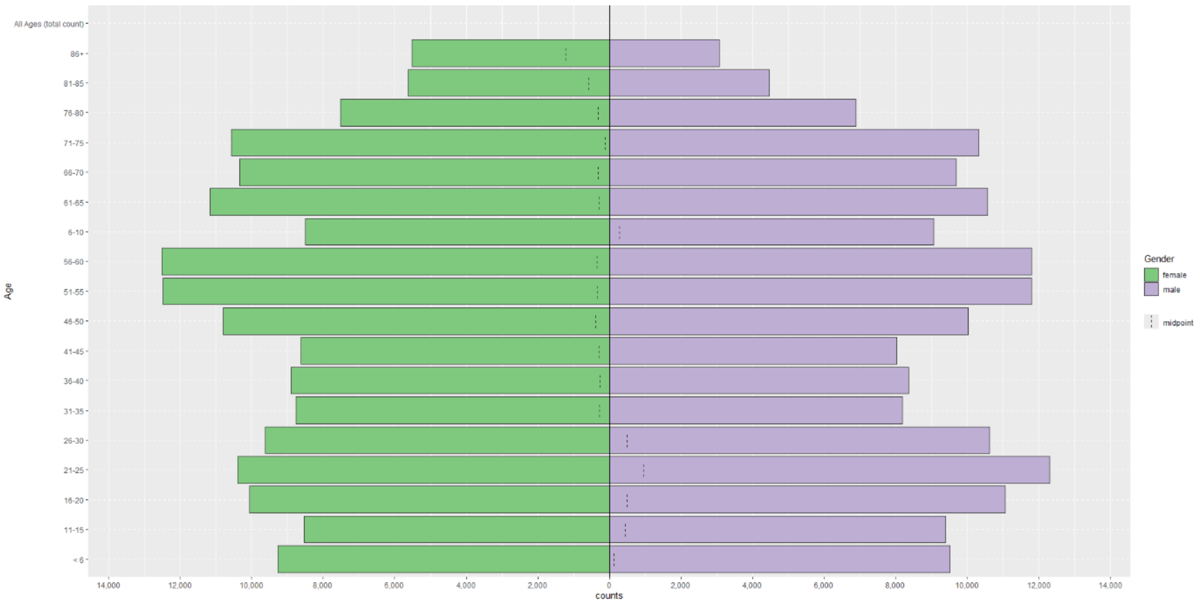

Supplementary Figure S1. Age distribution in Morecambe Bay ex CCG. This age pyramid illustrates the distribution of the population across different age groups, separated by gender. The horizontal axis represents the counts of the total population, while the vertical axis represents age groups in 5-year intervals. Purple bars (right side of the pyramid) represent the male population within each age group; green bars (left side of the pyramid) represent the female population within each age group.

Supplementary Information S3: Cancer burden

Lung cancer contributes to the largest number of cancer deaths in the world (1) while skin cancer is the most common in the general population (2), although prostate cancer is the most common in men, and breast cancer in female (3). After lung cancer, colorectal cancer is the second highest cause of cancer deaths in the United Kingdom (4). Haematological cancers are the third largest cancer killers in the UK[1] and increasingly important worldwide, with 1.3 million new cases in 2020 and 700,000 deaths (5). Within the upper GI cancers, oesophageal cancer in the UK has one of the highest age-standardised rates within the oesophageal cancers world wide (6). Head and neck cancer, a diverse group of more than 30 different subsites, is the 6th most common cancer worldwide and one of the most debilitating, although it constitutes only 3% of all cancers in the UK (7). Finally, every year in the UK, more than 21 000 women (5% of all cancers) are diagnosed with a gynaecological cancer and around 8000 die[2] (8).

References

1. Rait G, Horsfall L. Twenty-year sociodemographic trends in lung cancer in non-smokers: A UK-based cohort study of 3.7 million people. Cancer Epidemiol. 2020;67:101771.
2. Saleh GM, Desai P, Collin JR, Ives A, Jones T, Hussain B. Incidence of eyelid basal cell carcinoma in England: 2000-2010. Br J Ophthalmol. 2017;101(2):209-12.
3. Arnold M, Morgan E, Rumgay H, Mafra A, Singh D, Laversanne M, et al. Current and future burden of breast cancer: Global statistics for 2020 and 2040. Breast. 2022;66:15-23.
4. Lal N, Singh HK, Majeed A, Pawa N. The impact of socioeconomic deprivation on the uptake of colorectal cancer screening in London. J Med Screen. 2020:969141320916206.
5. Chen L, Zheng Y, Yu K, Chen S, Wang W, Gale RP, et al. Changing causes of death in persons with haematological cancers 1975-2016. Leukemia. 2022;36(7):1850-60.
6. Arnold M, Abnet CC, Neale RE, Vignat J, Giovannucci EL, McGlynn KA, Bray F. Global Burden of 5 Major Types of Gastrointestinal Cancer. Gastroenterology. 2020;159(1):335-49 e15.
7. Taib BG, Oakley J, Dailey Y, Hodge I, Wright P, du Plessis R, et al. Socioeconomic deprivation and the burden of head and neck cancer-Regional variations of incidence and mortality in Merseyside and Cheshire, North West, England. Clin Otolaryngol. 2018;43(3):846-53.
8. Knapp S, Henderson L, Duncan T, Gray T. Gynaecological cancers. InnovAiT: Education and inspiration for general practice. 2021;14(7):449-57.

---

[1] https://bloodcancer.org.uk/news/blood-cancer-facts/#

[2] https://www.macmillan.org.uk/about-us/what-we-do/research/cancer-statistics-fact-sheet#

Supplementary Table S1: Cancer type categorization

ICD10 Code	ICD10 Group	ICD10 Code Description	TumourSite
C50	C50	Malignant neoplasm of breast	Breast
C500	C50	Malignant neoplasm of nipple and areola	Breast
C501	C50	Malignant neoplasm of central portion of breast	Breast
C502	C50	Malignant neoplasm of upper-inner quadrant of breast	Breast
C503	C50	Malignant neoplasm of lower-inner quadrant of breast	Breast
C504	C50	Malignant neoplasm of upper-outer quadrant of breast	Breast
C505	C50	Malignant neoplasm of lower-outer quadrant of breast	Breast
C506	C50	Malignant neoplasm of axillary tail of breast	Breast
C508	C50	Malignant neoplasm, overlapping lesion of breast	Breast
C509	C50	Malignant neoplasm of breast, unspecified	Breast
D05	D05	Carcinoma in situ of breast	Breast
D050	D05	Lobular carcinoma in situ	Breast
D051	D05	Intraductal carcinoma in situ	Breast
D057	D05	Other carcinoma in situ of breast	Breast
D059	D05	Carcinoma in situ of breast, unspecified	Breast
C17	C17	Malignant neoplasm of small intestine	Colorectal
C170	C17	Malignant neoplasm of small intestine, duodenum	Colorectal
C171	C17	Malignant neoplasm of small intestine, jejunum	Colorectal
C172	C17	Malignant neoplasm of small intestine, ileum	Colorectal
C173	C17	Malignant neoplasm of small intestine, Meckel's diverticulum	Colorectal
C178	C17	Malignant neoplasm overlapping lesion of small intestine	Colorectal
C179	C17	Malignant neoplasm of small intestine, unspecified	Colorectal
C18	C18	Malignant neoplasm of colon	Colorectal
C180	C18	Malignant neoplasm of caecum	Colorectal
C181	C18	Malignant neoplasm of appendix	Colorectal
C182	C18	Malignant neoplasm of ascending colon	Colorectal
C183	C18	Malignant neoplasm of hepatic flexure	Colorectal
C184	C18	Malignant neoplasm of transverse colon	Colorectal
C185	C18	Malignant neoplasm of splenic flexure	Colorectal
C186	C18	Malignant neoplasm of descending colon	Colorectal
C187	C18	Malignant neoplasm of sigmoid colon	Colorectal
C188	C18	Malignant neoplasm overlapping lesion of colon	Colorectal

C189	C18	Malignant neoplasm of colon, unspecified	Colorectal
C19X	C19	Malignant neoplasm of rectosigmoid junction	Colorectal
C20X	C20	Malignant neoplasm of rectum	Colorectal
C21	C21	Malignant neoplasm of anus and anal canal	Colorectal
C210	C21	Malignant neoplasm of anus, unspecified	Colorectal
C211	C21	Malignant neoplasm of anal canal	Colorectal
C212	C21	Malignant neoplasm of cloacogenic zone	Colorectal
C218	C21	Malig neo, overlapping lesion of rectum, anus and anal canal	Colorectal
C26	C26	Malignant neoplasm of other and ill-defined digestive organs	Colorectal
C260	C26	Malignant neoplasm of intestinal tract, part unsp	Colorectal
C261	C26	Malignant neoplasm of spleen	Colorectal
C268	C26	Malignant neoplasm, overlapping lesion of digestive system	Colorectal
C269	C26	Malignant neoplasm of ill-def sites within digestive system	Colorectal
C784	C78	Secondary malignant neoplasm of small intestine	Colorectal
C785	C78	Secondary malignant neoplasm of large intest & rectum	Colorectal
C788	C78	Secondary malignant neoplasm of other & unsp digestive orgs	Colorectal
C51	C51	Malignant neoplasm of vulva	Gynaecology
C510	C51	Malignant neoplasm of labium majus	Gynaecology
C511	C51	Malignant neoplasm of labium minus	Gynaecology
C512	C51	Malignant neoplasm of clitoris	Gynaecology
C518	C51	Malignant neoplasm of overlapping lesion of vulva	Gynaecology
C519	C51	Malignant neoplasm of vulva, unspecified	Gynaecology
C52X	C52	Malignant neoplasm of vagina	Gynaecology
C53	C53	Malignant neoplasm of cervix uteri	Gynaecology
C530	C53	Malignant neoplasm of endocervix	Gynaecology
C531	C53	Malignant neoplasm of exocervix	Gynaecology
C538	C53	Malignant neoplasm, overlapping lesion of cervix uteri	Gynaecology
C539	C53	Malignant neoplasm of cervix uteri, unsp	Gynaecology
C54	C54	Malignant neoplasm of corpus uteri	Gynaecology
C540	C54	Malignant neoplasm of isthmus uteri	Gynaecology
C541	C54	Malignant neoplasm of endometrium	Gynaecology
C542	C54	Malignant neoplasm of myometrium	Gynaecology
C543	C54	Malignant neoplasm of fundus uteri	Gynaecology

C548	C54	Malignant neoplasm overlapping lesion of corpus uteri	Gynaecology
C549	C54	Malignant neoplasm of corpus uteri, unsp	Gynaecology
C55X	C55	Malignant neoplasm of uterus, part unspecified	Gynaecology
C56X	C56	Malignant neoplasm of ovary	Gynaecology
C57	C57	Malignant neoplasm of other and unspec female genital orgs	Gynaecology
C570	C57	Malignant neoplasm of fallopian tube	Gynaecology
C571	C57	Malignant neoplasm of broad ligament	Gynaecology
C572	C57	Malignant neoplasm of round ligament	Gynaecology
C573	C57	Malignant neoplasm of parametrium	Gynaecology
C574	C57	Malignant neoplasm of uterine adnexa, unsp	Gynaecology
C577	C57	Malignant neoplasm of other specified female genital organs	Gynaecology
C578	C57	Malignant neoplasm, overlapping lesion female genital organs	Gynaecology
C579	C57	Malignant neoplasm of female genital organ, unspecified	Gynaecology
C58X	C58	Malignant neoplasm of placenta	Gynaecology
C796	C79	Secondary malignant neoplasm of ovary	Gynaecology
C81	C81	Hodgkin lymphoma	Haematology
C810	C81	Nodular lymphocyte predominant Hodgkin lymphoma	Haematology
C811	C81	Nodular sclerosis classical Hodgkin lymphoma	Haematology
C812	C81	Mixed cellularity classical Hodgkin lymphoma	Haematology
C813	C81	Lymphocyte depleted classical Hodgkin lymphoma	Haematology
C814	C81	Lymphocyte-rich classical Hodgkin lymphoma	Haematology
C817	C81	Other classical Hodgkin lymphoma	Haematology
C819	C81	Hodgkin lymphoma, unspecified	Haematology
C82	C82	Follicular lymphoma	Haematology
C820	C82	Follicular lymphoma grade I	Haematology
C821	C82	Follicular lymphoma grade II	Haematology
C822	C82	Follicular lymphoma grade III, unspecified	Haematology
C823	C82	Follicular lymphoma grade IIIa	Haematology
C824	C82	Follicular lymphoma grade IIIb	Haematology
C825	C82	Diffuse follicle centre lymphoma	Haematology
C826	C82	Cutaneous follicle centre lymphoma	Haematology
C827	C82	Other types of follicular lymphoma	Haematology
C829	C82	Follicular lymphoma, unspecified	Haematology

C83	C83	Non-follicular lymphoma	Haematology
C830	C83	Small cell B-cell lymphoma	Haematology
C831	C83	Mantle cell lymphoma	Haematology
C832	C83	Diffuse non-Hodgkin mixed sml & lge cell (diffuse) lymphoma	Haematology
C833	C83	Diffuse large B-cell lymphoma	Haematology
C834	C83	Diffuse non-Hodgkin's immunoblastic (diffuse) lymphoma	Haematology
C835	C83	Lymphoblastic (diffuse) lymphoma	Haematology
C836	C83	Diffuse non-Hodgkin's lymphoma undifferentiated (diffuse)	Haematology
C837	C83	Burkitt lymphoma	Haematology
C838	C83	Other non-follicular lymphoma	Haematology
C839	C83	Non-follicular (diffuse) lymphoma, unspecified	Haematology
C84	C84	Mature T/NK-cell lymphomas	Haematology
C840	C84	Peripheral and cutaneous T-cell lymphomas, mycosis fungoides	Haematology
C841	C84	SezÚry disease	Haematology
C842	C84	Peripheral and cutaneous T-cell lymphomas, T-zone lymphoma	Haematology
C843	C84	Periph & cutan T-cell lymphomas, lymphoepithelioid lymphoma	Haematology
C844	C84	Peripheral T-cell lymphoma, not elsewhere classified	Haematology
C845	C84	Other mature T/NK-cell lymphomas	Haematology
C846	C84	Anaplastic large cell lymphoma, ALK-positive	Haematology
C847	C84	Anaplastic large cell lymphoma, ALK-negative	Haematology
C848	C84	Cutaneous T-cell lymphoma, unspecified	Haematology
C849	C84	Mature T/NK-cell lymphoma, unspecified	Haematology
C85	C85	Other and unspecified types of non-Hodgkin lymphoma	Haematology
C850	C85	Oth & unspec types of non-Hodgkin lymphoma, lymphosarcoma	Haematology
C851	C85	Oth & unsp types non-Hodgkin B-cell lymphoma, unsp	Haematology
C852	C85	Mediastinal (thymic) large B-cell lymphoma	Haematology
C857	C85	Other specified types of non-Hodgkin lymphoma	Haematology
C859	C85	Non-Hodgkin lymphoma, unspecified	Haematology
C86	C86	Other specified types of T/NK-cell lymphoma	Haematology
C860	C86	Extranodal NK/T-cell lymphoma, nasal type	Haematology
C861	C86	Hepatosplenic T-cell lymphoma	Haematology
C862	C86	Enteropathy-type (intestinal) T-cell lymphoma	Haematology
C863	C86	Subcutaneous panniculitis-like T-cell lymphoma	Haematology

C864	C86	Blastic NK-cell lymphoma	Haematology
C865	C86	Angioimmunoblastic T-cell lymphoma	Haematology
C866	C86	Primary cutaneous CD30-positive T-cell proliferations	Haematology
C88	C88	Malignant immunoproliferative diseases	Haematology
C880	C88	Waldenstrom macroglobulinaemia	Haematology
C881	C88	Alpha heavy chain disease	Haematology
C882	C88	Other heavy chain disease	Haematology
C883	C88	Malignant immunoproliferative small intestinal disease	Haematology
C884	C88	Extranodal marginal zone B-cell lymphoma of mucosa-associated lymphoid tissue [MALT-lymphoma]	Haematology
C887	C88	Other malignant immunoproliferative diseases	Haematology
C889	C88	Malignant immunoproliferative disease, unspecified	Haematology
C90	C90	Multiple myeloma and malignant plasma cell neoplasms	Haematology
C900	C90	Multiple myeloma	Haematology
C901	C90	Plasma cell leukaemia	Haematology
C902	C90	Extramedullary plasmacytoma	Haematology
C903	C90	Solitary plasmacytoma	Haematology
C91	C91	Lymphoid leukaemia	Haematology
C910	C91	Acute lymphoblastic leukaemia [ALL]	Haematology
C911	C91	Chronic lymphocytic leukaemia of B-cell type	Haematology
C912	C91	Subacute lymphocytic leukaemia	Haematology
C913	C91	Prolymphocytic leukaemia of B-cell type	Haematology
C914	C91	Hairy-cell leukaemia	Haematology
C915	C91	Adult T-cell lymphoma/leukaemia (HTLV-1-associated)	Haematology
C916	C91	Prolymphocytic leukaemia of T-cell type	Haematology
C917	C91	Other lymphoid leukaemia	Haematology
C918	C91	Mature B-cell leukaemia Burkitt-type	Haematology
C919	C91	Lymphoid leukaemia, unspecified	Haematology
C92	C92	Myeloid leukaemia	Haematology
C920	C92	Acute myeloblastic leukaemia [AML]	Haematology
C921	C92	Chronic myeloid leukaemia [CML], BCR/ABL-positive	Haematology
C922	C92	Atypical chronic myeloid leukaemia, BCR/ABL-negative	Haematology
C923	C92	Myeloid sarcoma	Haematology
C924	C92	Acute promyelocytic leukaemia [PML]	Haematology

C925	C92	Acute myelomonocytic leukaemia	Haematology
C926	C92	Acute myeloid leukaemia with 11q23-abnormality	Haematology
C927	C92	Other myeloid leukaemia	Haematology
C928	C92	Acute myeloid leukaemia with multilineage dysplasia	Haematology
C929	C92	Myeloid leukaemia, unspecified	Haematology
C93	C93	Monocytic leukaemia	Haematology
C930	C93	Acute monoblastic/monocytic leukaemia	Haematology
C931	C93	Chronic myelomonocytic leukaemia	Haematology
C932	C93	Subacute monocytic leukaemia	Haematology
C933	C93	Juvenile myelomonocytic leukaemia	Haematology
C937	C93	Other monocytic leukaemia	Haematology
C939	C93	Monocytic leukaemia, unspecified	Haematology
C94	C94	Other leukaemias of specified cell type	Haematology
C940	C94	Acute erythroid leukaemia	Haematology
C941	C94	Chronic erythraemia	Haematology
C942	C94	Acute megakaryoblastic leukaemia	Haematology
C943	C94	Mast cell leukaemia	Haematology
C944	C94	Acute panmyelosis with myelofibrosis	Haematology
C945	C94	Acute myelofibrosis	Haematology
C946	C94	Myelodysplastic and myeloproliferative disease, not elsewhere classified	Haematology
C947	C94	Other specified leukaemias	Haematology
C95	C95	Leukaemia of unspecified cell type	Haematology
C950	C95	Acute leukaemia of unsp cell type	Haematology
C951	C95	Chronic leukaemia unsp cell type	Haematology
C952	C95	Subacute leukaemia unsp cell type	Haematology
C957	C95	Other leukaemia unspecified cell type	Haematology
C959	C95	Leukaemia, unspecified	Haematology
C96	C96	Oth & unspec malig neop lymphoid, haematapoietic & rel tiss	Haematology
C960	C96	Multifocal and multisystemic (disseminated) Langerhans-cell histiocytosis [Letterer-Siwe disease]	Haematology
C961	C96	Malignant histiocytosis	Haematology
C962	C96	Malignant mast cell tumour	Haematology
C963	C96	True histiocytic lymphoma	Haematology
C964	C96	Sarcoma of dendritic cells (accessory cells)	Haematology

C965	C96	Multifocal and unisystemic Langerhans-cell histiocytosis	Haematology
C966	C96	Unifocal Langerhans-cell histiocytosis	Haematology
C967	C96	Oth spec malig neop lymphoid h'poietic & related tissue	Haematology
C968	C96	Histiocytic sarcoma	Haematology
C969	C96	Malig neop lymphoid haematopoietic and related tissue unspec	Haematology
C00	C00	Malignant neoplasm of lip	Head and Neck
C000	C00	Malignant neoplasm of external upper lip	Head and Neck
C001	C00	Malignant neoplasm of external lower lip	Head and Neck
C002	C00	Malignant neoplasm of external lip, unspecified	Head and Neck
C003	C00	Malignant neoplasm of upper lip, inner aspect	Head and Neck
C004	C00	Malignant neoplasm of lower lip, inner aspect	Head and Neck
C005	C00	Malignant neoplasm of lip, unspecified, inner aspect	Head and Neck
C006	C00	Malignant neoplasm of commissure of lip	Head and Neck
C008	C00	Malignant neoplasm of overlapping lesion of lip	Head and Neck
C009	C00	Malignant neoplasm of lip, unspecified	Head and Neck
C01X	C01	Malignant neoplasm of base of tongue	Head and Neck
C02	C02	Malignant neoplasm of other and unspecified parts of tongue	Head and Neck
C020	C02	Malignant neoplasm of dorsal surface tongue	Head and Neck
C021	C02	Malignant neoplasm of border of tongue	Head and Neck
C022	C02	Malignant neoplasm of ventral surface of tongue	Head and Neck
C023	C02	Malignant neo of anterior two-thirds of tongue, part uns	Head and Neck
C024	C02	Malignant neoplasm of lingual tonsil	Head and Neck
C028	C02	Malignant neoplasm of overlapping lesion of tongue	Head and Neck
C029	C02	Malignant neoplasm of tongue, unspecified	Head and Neck
C03	C03	Malignant neoplasm of gum	Head and Neck
C030	C03	Malignant neoplasm of upper gum	Head and Neck
C031	C03	Malignant neoplasm of lower gum	Head and Neck
C039	C03	Malignant neoplasm of gum unspecified	Head and Neck
C04	C04	Malignant neoplasm of floor of mouth	Head and Neck
C040	C04	Malignant neoplasm of floor of anterior floor of mouth	Head and Neck
C041	C04	Malignant neoplasm of lateral floor of mouth	Head and Neck
C048	C04	Malignant neoplasm, overlapping lesion of floor of mouth	Head and Neck
C049	C04	Malignant neoplasm of floor of mouth, floor of mouth, unspec	Head and Neck

C05	C05	Malignant neoplasm of palate	Head and Neck
C050	C05	Malignant neoplasm of hard palate	Head and Neck
C051	C05	Malignant neoplasm of soft palate	Head and Neck
C052	C05	Malignant neoplasm of uvula	Head and Neck
C058	C05	Malignant neoplasm, overlapping lesion of palate	Head and Neck
C059	C05	Malignant neoplasm of palate, unspecified	Head and Neck
C06	C06	Malignant neoplasm of other and unspecified parts of mouth	Head and Neck
C060	C06	Malignant neoplasm cheek mucosa	Head and Neck
C061	C06	Malignant neoplasm of vestibule of mouth	Head and Neck
C062	C06	Malignant neoplasm of retromolar area	Head and Neck
C068	C06	Malignant neoplasm, overlap les of oth & unsp part of mouth	Head and Neck
C069	C06	Malignant neoplasm of part of mouth, unspecified	Head and Neck
C07X	C07	Malignant neoplasm of parotid gland	Head and Neck
C08	C08	Maligt neoplasm of oth and unspec major saliv glands	Head and Neck
C080	C08	Malignant neoplasm of submandibular gland	Head and Neck
C081	C08	Malignant neoplasm of sublingual gland	Head and Neck
C088	C08	Malignant neoplasm, overlapping lesion of major saliv gland	Head and Neck
C089	C08	Malignant neoplasm of major salivary gland, unspecified	Head and Neck
C09	C09	Malignant neoplasm of tonsil	Head and Neck
C090	C09	Malignant neoplasm tonsillar fossa	Head and Neck
C091	C09	Malig neo of tonsillar pillar (anterior)(posterior)	Head and Neck
C098	C09	Malignant neoplasm of overlapping lesion of tonsil	Head and Neck
C099	C09	Malignant neoplasm of tonsil unspecified	Head and Neck
C10	C10	Malignant neoplasm of oropharynx	Head and Neck
C100	C10	Malignant neoplasm of vallecula	Head and Neck
C101	C10	Malignant neoplasm of anterior surface of epiglottis	Head and Neck
C102	C10	Malignant neoplasm of lateral wall of oropharynx	Head and Neck
C103	C10	Malignant neoplasm of posterior wall of oropharynx	Head and Neck
C104	C10	Malignant neoplasm of branchial cleft	Head and Neck
C108	C10	Malignant neoplasm overlapping lesion of oropharynx	Head and Neck
C109	C10	Malignant neoplasm of oropharynx unspecified	Head and Neck
C11	C11	Malignant neoplasm of nasopharynx	Head and Neck
C110	C11	Malignant neoplasm of superior wall of nasopharynx	Head and Neck

C111	C11	Malignant neoplasm of posterior wall of nasopharynx	Head and Neck
C112	C11	Malignant neoplasm of lateral wall of nasopharynx	Head and Neck
C113	C11	Malignant neoplasm of anterior wall of nasopharynx	Head and Neck
C118	C11	Malignant neoplasm overlapping lesion of nasopharynx	Head and Neck
C119	C11	Malignant neoplasm of nasopharynx unspecified	Head and Neck
C12X	C12	Malignant neoplasm of pyriform sinus	Head and Neck
C13	C13	Malignant neoplasm of hypopharynx	Head and Neck
C130	C13	Malignant neoplasm of hypopharynx, postcricoid region	Head and Neck
C131	C13	Malig neoplasm aryepiglottic fold, hypopharyngeal aspect	Head and Neck
C132	C13	Malignant neoplasm posterior wall of hypopharynx	Head and Neck
C138	C13	Malignant neoplasm overlapping lesion of hypopharynx	Head and Neck
C139	C13	Malignant neoplasm of hypopharynx unspecified	Head and Neck
C14	C14	Mal neo oth ill-def sites lip/oral cavity/pharynx	Head and Neck
C140	C14	Malignant neoplasm of pharynx, unsp	Head and Neck
C142	C14	Malignant neoplasm of Waldeyer's ring	Head and Neck
C148	C14	Malig neo, overlapping lesion of lip, oral cavity & pharynx	Head and Neck
C30	C30	Malignant neoplasm of nasal cavity and middle ear	Head and Neck
C300	C30	Malignant neoplasm of nasal cavity	Head and Neck
C301	C30	Malignant neoplasm of middle ear	Head and Neck
C31	C31	Malignant neoplasm of accessory sinuses	Head and Neck
C310	C31	Malignant neoplasm of maxillary sinus	Head and Neck
C311	C31	Malignant neoplasm of ethmoidal sinus	Head and Neck
C312	C31	Malignant neoplasm of frontal sinus	Head and Neck
C313	C31	Malignant neoplasm of sphenoidal sinus	Head and Neck
C318	C31	Malignant neoplasm, overlapping lesion accessory sinuses	Head and Neck
C319	C31	Malignant neoplasm of accessory sinus, unsp	Head and Neck
C32	C32	Malignant neoplasm of larynx	Head and Neck
C320	C32	Malignant neoplasm of glottis	Head and Neck
C320A	C32	Vocal cords, true	Head and Neck
C320B	C32	Anterior commissure	Head and Neck
C320C	C32	Posterior commissure	Head and Neck
C321	C32	Malignant neoplasm of supraglottis	Head and Neck
C321A	C32	Suprahyoid epiglottis (tip, laryngeal surface)	Head and Neck

C321B	C32	Aryepiglottic fold, laryngeal aspect	Head and Neck
C321C	C32	Arytenoid	Head and Neck
C321D	C32	Infrahyoid epiglottis	Head and Neck
C321E	C32	False cords	Head and Neck
C322	C32	Malignant neoplasm of subglottis	Head and Neck
C323	C32	Malignant neoplasm of laryngeal cartilage	Head and Neck
C323A	C32	Arytenoid cartilage	Head and Neck
C323B	C32	Cricoid cartilage	Head and Neck
C323C	C32	Thyroid cartilage	Head and Neck
C328	C32	Malignant neoplasm, overlapping lesion of larynx	Head and Neck
C329	C32	Malignant neoplasm of larynx, unspecified	Head and Neck
C73X	C73	Malignant neoplasm of thyroid gland	Head and Neck
C770	C77	Sec & uns malig neoplasm of lymph nodes of head, face & neck	Head and Neck
C339A	C33	Tracheal stoma	Lung
C33X	C33	Malignant neoplasm of trachea	Lung
C34	C34	Malignant neoplasm of bronchus and lung	Lung
C340	C34	Malignant neoplasm of main bronchus	Lung
C341	C34	Malignant neoplasm of upper lobe, bronchus or lung	Lung
C342	C34	Malignant neoplasm of middle lobe, bronchus or lung	Lung
C343	C34	Malignant neoplasm of lower lobe, bronchus or lung	Lung
C348	C34	Malignant neoplasm of overlap les of bronchus & lung	Lung
C349	C34	Malignant neoplasm of bronchus or lung, unspec	Lung
C37X	C37	Malignant neoplasm of thymus	Lung
C38	C38	Malignant neoplasm of heart, mediastinum and pleura	Lung
C380	C38	Malignant neoplasm of heart, mediastinum & pleura, heart	Lung
C381	C38	Malignant neoplasm of anterior mediastinum	Lung
C382	C38	Malignant neoplasm of posterior mediastinum	Lung
C383	C38	Malig neo heart, mediastinum & pleura,mediastinum,part unsp	Lung
C384	C38	Malignant neoplasm of pleura	Lung
C388	C38	Malig neo, overlapping lesion of heart, mediastinum & pleura	Lung
C39	C39	Malig neo oth + ill-def sites resp sys + intrathorac orgs	Lung
C390	C39	Malignant neoplasm of upper respiratory tract, part unsp	Lung
C398	C39	Malignant neoplasm, overlap lesion of resp & intrathor orgs	Lung

C399	C39	Malignant neoplasm of ill-def sites within the resp sys	Lung
C45	C45	Mesothelioma	Lung
C450	C45	Mesothelioma of pleura	Lung
C451	C45	Mesothelioma of peritoneum	Lung
C452	C45	Mesothelioma of pericardium	Lung
C457	C45	Mesothelioma of other sites	Lung
C459	C45	Mesothelioma, unspecified	Lung
C78	C78	Sec malignant neoplasm of respiratory and digestive organs	Lung
C780	C78	Secondary malignant neoplasm of lung	Lung
C781	C78	Secondary malignant neoplasm of mediastinum	Lung
C782	C78	Secondary malignant neoplasm of pleura	Lung
C783	C78	Secondary malignant neoplasm of oth & unsp respiratory orgs	Lung
C43	C43	Malignant melanoma of skin	Skin
C430	C43	Malignant melanoma of lip	Skin
C431	C43	Malignant melanoma of eyelid, including canthus	Skin
C432	C43	Malignant melanoma of ear and ext auricular canal	Skin
C433	C43	Malignant melanoma of other and unspecified parts of face	Skin
C434	C43	Malignant melanoma of scalp and neck	Skin
C435	C43	Malignant melanoma of trunk	Skin
C436	C43	Malignant melanoma of upper limb, including shoulder	Skin
C437	C43	Malignant melanoma of lower limb, including hip	Skin
C438	C43	Malignant melanoma of skin	Skin
C439	C43	Malignant melanoma of skin, unsp	Skin
C44	C44	Other malignant neoplasms of skin	Skin
C440	C44	Other malignant neoplasms of skin of lip	Skin
C441	C44	Other malignant neoplasms of skin of eyelid, incl canthus	Skin
C442	C44	Oth malignant neoplasms of skin of ear & ext auricular canal	Skin
C443	C44	Oth malignant neoplasm of skin of oth & unsp parts of face	Skin
C444	C44	Other malignant neoplasms of skin of scalp and neck	Skin
C445	C44	Other malignant neoplasms of skin of trunk	Skin
C446	C44	Oth malignant neoplasms of skin of upper limb, incl shoulder	Skin
C447	C44	Other malignant neoplasms of skin of lower limb, incl hip	Skin
C448	C44	Other malignant neoplasms, overlapping lesion of skin	Skin

C449	C44	Other malignant neoplasms of skin, unspecified	Skin
C792	C79	Secondary malignant neoplasm of skin	Skin
C15	C15	Malignant neoplasm of oesophagus	Upper GI
C150	C15	Malignant neoplasm of cervical part of oesophagus	Upper GI
C151	C15	Malignant neoplasm of thoracic part of oesophagus	Upper GI
C152	C15	Malignant neo of abdominal part of oesophagus	Upper GI
C153	C15	Malignant neoplasm of upper third of oesophagus	Upper GI
C154	C15	Malignant neoplasm of middle third of oesophagus	Upper GI
C155	C15	Malignant neoplasm of lower third of oesophagus	Upper GI
C158	C15	Malignant neoplasm overlapping lesion of oesophagus	Upper GI
C159	C15	Malignant neoplasm of oesophagus unspecified	Upper GI
C16	C16	Malignant neoplasm of stomach	Upper GI
C160	C16	Malignant neoplasm of cardia of stomach	Upper GI
C161	C16	Malignant neoplasm of fundus of stomach	Upper GI
C162	C16	Malignant neoplasm of body of stomach	Upper GI
C163	C16	Malignant neoplasm of pyloric antrum	Upper GI
C164	C16	Malignant neoplasm of pylorus	Upper GI
C165	C16	Malignant neoplasm of lesser curvature of stomach, unsp	Upper GI
C166	C16	Malignant neoplasm of greater curvature of stomach, unsp	Upper GI
C168	C16	Malignant neoplasm overlapping lesion of stomach	Upper GI
C169	C16	Malignant neoplasm of stomach, unspecified	Upper GI
C22	C22	Malignant neoplasm of liver and intrahepatic bile ducts	Upper GI
C220	C22	Malignant neoplasm, liver cell carcinoma	Upper GI
C221	C22	Malignant neoplasm, intrahep bile duct carcinoma	Upper GI
C222	C22	Malignant neoplasm, hepatoblastoma	Upper GI
C223	C22	Malignant neoplasm, angiosarcoma of liver	Upper GI
C224	C22	Malignant neoplasm, other sarcomas of liver	Upper GI
C227	C22	Malignant neoplasm, oth spec carcinomas of liver	Upper GI
C229	C22	Malignant neoplasm, liver, unspecified	Upper GI
C23X	C23	Malignant neoplasm of gallbladder	Upper GI
C24	C24	Maligt neoplasm of other and unspec parts biliary tract	Upper GI
C240	C24	Malignant neoplasm of extrahepatic bile duct	Upper GI
C241	C24	Malignant neoplasm of Ampulla of Vater	Upper GI

C248	C24	Malignant neoplasm overlapping lesion of biliary tract	Upper GI
C249	C24	Malignant neoplasm of biliary tract, unspecified	Upper GI
C25	C25	Malignant neoplasm of pancreas	Upper GI
C250	C25	Malignant neoplasm of head of pancreas	Upper GI
C251	C25	Malignant neoplasm of body of pancreas	Upper GI
C252	C25	Malignant neoplasm of tail of pancreas	Upper GI
C253	C25	Malignant neoplasm of pancreatic duct	Upper GI
C254	C25	Malignant neoplasm of endocrine pancreas	Upper GI
C257	C25	Malignant neoplasm of other parts of pancreas	Upper GI
C258	C25	Malignant neoplasm, overlapping lesion of pancreas	Upper GI
C259	C25	Malignant neoplasm of pancreas, unspecified	Upper GI
C787	C78	Secondary malignant neoplasm of liver and intrahepatic bile duct	Upper GI
C60	C60	Malignant neoplasm of penis	Urology
C600	C60	Malignant neoplasm of prepuce	Urology
C601	C60	Malignant neoplasm of glans penis	Urology
C602	C60	Malignant neoplasm of body of penis	Urology
C608	C60	Malignant neoplasm, overlapping lesion of penis	Urology
C609	C60	Malignant neoplasm of penis, unspecified	Urology
C61X	C61	Malignant neoplasm of prostate	Urology
C62	C62	Malignant neoplasm of testis	Urology
C620	C62	Malignant neoplasm of undescended testis	Urology
C621	C62	Malignant neoplasm of descended testis	Urology
C629	C62	Malignant neoplasm of testis, unspecified	Urology
C63	C63	Malignant neoplasm of other and unspec male genital organs	Urology
C630	C63	Malignant neoplasm of epididymis	Urology
C631	C63	Malignant neoplasm of spermatic cord	Urology
C632	C63	Malignant neoplasm of scrotum	Urology
C637	C63	Malignant neoplasm of other specified male genital orgs	Urology
C638	C63	Malignant neoplasm, overlapping lesion male genital orgs	Urology
C639	C63	Malignant neoplasm of male genital organ, unspecified	Urology
C64X	C64	Malignant neoplasm of kidney, except renal pelvis	Urology
C65X	C65	Malignant neoplasm of renal pelvis	Urology
C66X	C66	Malignant neoplasm of ureter	Urology

C67	C67	Malignant neoplasm of bladder	Urology
C670	C67	Malignant neoplasm of trigone of bladder	Urology
C671	C67	Malignant neoplasm of dome of bladder	Urology
C672	C67	Malignant neoplasm of lateral wall of bladder	Urology
C673	C67	Malignant neoplasm of anterior wall of bladder	Urology
C674	C67	Malignant neoplasm of posterior wall of bladder	Urology
C675	C67	Malignant neoplasm of bladder neck	Urology
C676	C67	Malignant neoplasm of ureteric orifice	Urology
C677	C67	Malignant neoplasm of urachus	Urology
C678	C67	Malignant neoplasm, overlapping lesion of bladder	Urology
C679	C67	Malignant neoplasm of bladder, unspecified	Urology
C68	C68	Malignant neoplasm of other and unspecified urinary organs	Urology
C680	C68	Malignant neoplasm of urethra	Urology
C681	C68	Malignant neoplasm of paraurethral gland	Urology
C688	C68	Malignant neoplasm of overlapping lesion urinary organs	Urology
C689	C68	Malignant neoplasm of urinary organ, unspecified	Urology
C790	C79	Secondary malignant neoplasm of kidney & renal pelvis	Urology
C791	C79	Secondary malignant neoplasm of oth & uns urinary organs	Urology

Supplementary Table S2. Censoring correction by cancer type. The preliminary adjustment reduced the number of censored records (one record containing the number of diagnoses for a cancer type in a postcode sector during a 6 months period) to up 86% (for cancer type 'others' not considered in this study). Head and neck cancer censored records reduced by 84% (from 366 censored records to only 57), but breast and colorectal of less than 25%.

Cancer	N. of records	Censored records (%)	Censored records after correction (%)	Mean number of diagnoses for uncensored records	Mean number of diagnoses for uncensored records after correction	% records with censored values between 1 and 2	% records with censored values between 1 and 3	% records with censored values between 1 and 4	% records with censored values between 1 and 5	Sum of diagnoses from uncensored records before correction	Sum of diagnoses from uncensored records after correction	Number of censored records after correction
Brain/CNS	7	100	100	NA	NA	0	0	0	100	0	0	7
Breast	606	81.35	77.72	7.77	6.67	45.54	21.29	2.97	7.92	878	900	471
Colorectal	588	92.18	85.71	6.93	4.25	59.52	19.22	0	6.97	319	357	504
Gynaecology	406	99.51	43.84	6	1.04	37.19	0	0	6.65	12	238	178
Haematology	479	97.49	62.84	6.58	1.38	55.53	0.42	0	6.89	79	245	301
Head and Neck	366	100	15.57	NA	1	8.2	0	0	7.38	0	309	57
Lung	531	88.14	66.1	7.25	3.19	35.4	21.66	0.38	8.66	457	574	351
Other	266	99.62	13.91	6	1.02	1.88	0	0	12.03	6	234	37
Sarcoma	54	100	100	NA	NA	0	0	0	100	0	0	54
Skin	669	50.82	45.59	11.66	10.7	17.19	16.14	4.93	7.32	3837	3893	305
Upper GI	498	98.19	64.66	6.33	1.27	46.79	8.03	0	9.84	57	224	322
Urology	640	75.62	74.69	7.73	7.49	42.97	22.34	3.91	5.47	1206	1214	478

Supplementary Information S4: Censoring reduction

The postcode sector and Local Authority district count data used here, of cancer new diagnosis by type, were censored, with values less than or equal to 5 replaced by the words 'less than or equal to 5'. However, because zero was not included in the censoring, it was more appropriate to describe the censoring as interval censoring, with the censored values being larger than 0 and smaller than 6 (1). This means that the dataset contains nonignorable or 'missing not random' values (2). In other words, the missing data isn't just randomly scattered throughout the dataset, but instead, there's a pattern.

Before any statistical analyses started, the number of censored data was reduced by combining the Local Authority (LA) with the postcode sector (PS) data. The following rules were used (for each cancer type and time period):

a) if within an LA (containing multiple PS) only one PS was censored, then the number of patients in the censored PS was obtained by the difference of the number of diagnosis in the LA minus the sum of patients in the non-censored PS belonging to that LA;

b) if the number of diagnosis in the LA (or the residual number of diagnosis after deducting diagnosis from non-censored PS) was equal to the number of censored PS, then each censored PS was associated with one diagnosis;

c) if the number of diagnosis in the LA (or the residual number of diagnosis after deducting diagnosis from non-censored PS) is equal to five times the number of censored PS, then each censored PS was associated with five diagnoses.

In all the other cases, the number of censored patients in each PS and time period, was converted to 1 to 2, 1 to 3, 1 to 4 and 1 to 5 censoring level depending on the total number or residual number of diagnoses at the LA unit level. For example, if an LA had nine censored diagnosis and three censored PS, each censored PS will have a censoring level of 1 to 5 (since it is still possible for one PS to have 5 diagnoses). However, if the LA had 5 diagnoses, then the three censored PS will be censored 1 to 3 since none of them can have all five or four diagnoses.

References

1. Wong GYC, Yu Q. Generalized MLE of a Joint Distribution Function with Multivariate Interval-Censored Data. Journal of Multivariate Analysis. 1999;69(2):155-66.

2. Leacy FP, Floyd S, Yates TA, White IR. Analyses of Sensitivity to the Missing-at-Random Assumption Using Multiple Imputation With Delta Adjustment: Application to a Tuberculosis/HIV Prevalence Survey With Incomplete HIV-Status Data. Am J Epidemiol. 2017;185(4):304-15.

Supplementary Table S3: List of variables used in the study

ID	ID relative to data source	***Aggregated data from Hospital Information (linked primary and secondary data)***	Spatial Resolution
1	1	Month	Post-code sector
2	2	Year	Post-code sector
3	3	CancerCount (censored)	Post-code sector
4	4	PostcodeSector	Post-code sector
5	5	CancerSite	Post-code sector
6	6	AgeUnder18	Post-code sector
7	7	Age18-40	Post-code sector
8	8	Age40-60	Post-code sector
9	9	Age60-80	Post-code sector
10	10	AgeMoreThan80	Post-code sector
11	11	Male	Post-code sector
12	12	Female	Post-code sector
13	13	CKDStage1	Post-code sector
14	14	CKDStage2	Post-code sector
15	15	CKDStage3	Post-code sector
16	16	CKDStage4	Post-code sector
17	17	CKDStage5	Post-code sector
18	18	DiabetesType1	Post-code sector
19	19	DiabetesType2	Post-code sector
20	20	Diabetes	Post-code sector
21	21	CKD	Post-code sector
22	22	CongestiveHeartFailure	Post-code sector
23	23	COPD	Post-code sector
24	24	CoronaryHeartDisease	Post-code sector
25	25	Covid19	Post-code sector
26	26	Depression	Post-code sector
27	27	Hypertensive	Post-code sector
28	28	FrailtyFit	Post-code sector
29	29	FrailtyMild	Post-code sector
30	30	FrailtyModerate	Post-code sector
31	31	FrailtySevere	Post-code sector
32	32	UnknownSmoker	Post-code sector
33	33	NeverSmoker	Post-code sector
34	34	ExSmoker	Post-code sector
35	35	CurrentSmoker	Post-code sector

36	36	Anxiety	Post-code sector
37	37	African	Post-code sector
38	38	British	Post-code sector
39	39	AnyotherWhitebackground	Post-code sector
40	40	AnyotherBlackbackground	Post-code sector
41	41	AnyotherAsianbackground	Post-code sector
42	42	Anyothermixedbackground	Post-code sector
43	43	Anyotherethnicgroup	Post-code sector
44	44	English-ethniccategory2001census	Post-code sector
45	45	Irish	Post-code sector
46	46	Scottish-ethniccategory2001census	Post-code sector
47	47	Chinese	Post-code sector
48	48	Indian	Post-code sector
49	49	Italian-ethniccategory2001census	Post-code sector
50	50	WhiteandBlackAfrican	Post-code sector
51	51	WhiteandBlackCaribbean	Post-code sector
52	52	WhiteandAsian	Post-code sector
53	53	OthermixedWhite-ethniccategory2001census	Post-code sector
54	54	OthWhiteEuropean/Europeanunsp/MixedEuropean2001census	Post-code sector
55	55	OtherWhiteorWhiteunspecifiedethniccategory2001census	Post-code sector
56	56	Polish-ethniccategory2001census	Post-code sector
57	57	Pakistani	Post-code sector
58	58	Filipino-ethniccategory2001census	Post-code sector
59	59	Welsh-ethniccategory2001census	Post-code sector
60	60	Bangladeshi	Post-code sector
61	61	EastAfricanAsian-ethniccategory2001census	Post-code sector
62	62	Caribbean	Post-code sector
63	63	ChineseandWhite-ethniccategory2001census	Post-code sector
64	64	NorthernIrish-ethniccategory2001census	Post-code sector
65	65	Notknown	Post-code sector
66	66	Notstated	Post-code sector
		Geography	
67	1	Longitude	Post-code centroid
68	2	Latitude	Post-code centroid
69	3	LSOA	LSOA
70	4	Local Authority	LA
71	5	Total Area	LSOA
		Crime	

72	1	Criminaldamageandarson	Post-code
73	2	Vehiclecrime	Post-code
74	3	Violenceandsexualoffences	Post-code
75	4	Anti-socialbehaviour	Post-code
76	5	Publicorder	Post-code
77	6	Burglary	Post-code
78	7	Shoplifting	Post-code
79	8	Drugs	Post-code
80	9	Bicycletheft	Post-code
81	10	Theftfromtheperson	Post-code
82	11	Robbery	Post-code
83	12	Possessionofweapons	Post-code
84	13	Othercrime	Post-code
85	14	Othertheft	Post-code
		Census 2011	
86	1	Sex_Allpersons;Age_Allcategories_Age;EthnicGroup_White_Total;measures_Value	LA
87	2	Sex_Allpersons;Age_Allcategories_Age;EthnicGroup_White_English/Welsh/Scottish/NorthernIrish/British;measures_Value	LA
88	3	Sex_Allpersons;Age_Allcategories_Age;EthnicGroup_White_Irish;measures_Value	LA
89	4	Sex_Allpersons;Age_Allcategories_Age;EthnicGroup_White_GypsyorIrishTraveller;measures_Value	LA
90	5	Sex_Allpersons;Age_Allcategories_Age;EthnicGroup_White_OtherWhite;measures_Value	LA
91	6	Sex_Allpersons;Age_Allcategories_Age;EthnicGroup_Mixed/multipleethnicgroup_Total;measures_Value	LA
92	7	Sex_Allpersons;Age_Allcategories_Age;EthnicGroup_Mixed/multipleethnicgroup_WhiteandBlackCaribbean;measures_Value	LA
93	8	Sex_Allpersons;Age_Allcategories_Age;EthnicGroup_Mixed/multipleethnicgroup_WhiteandBlackAfrican;measures_Value	LA
94	9	Sex_Allpersons;Age_Allcategories_Age;EthnicGroup_Mixed/multipleethnicgroup_WhiteandAsian;measures_Value	LA
95	10	Sex_Allpersons;Age_Allcategories_Age;EthnicGroup_Mixed/multipleethnicgroup_OtherMixed;measures_Value	LA
96	11	Sex_Allpersons;Age_Allcategories_Age;EthnicGroup_Asian/AsianBritish_Total;measures_Value	LA
97	12	Sex_Allpersons;Age_Allcategories_Age;EthnicGroup_Asian/AsianBritish_Indian;measures_Value	LA
98	13	Sex_Allpersons;Age_Allcategories_Age;EthnicGroup_Asian/AsianBritish_Pakistani;measures_Value	LA
99	14	Sex_Allpersons;Age_Allcategories_Age;EthnicGroup_Asian/AsianBritish_Bangladeshi;measures_Value	LA
100	15	Sex_Allpersons;Age_Allcategories_Age;EthnicGroup_Asian/AsianBritish_Chinese;measures_Value	LA
101	16	Sex_Allpersons;Age_Allcategories_Age;EthnicGroup_Asian/AsianBritish_OtherAsian;measures_Value	LA
102	17	Sex_Allpersons;Age_Allcategories_Age;EthnicGroup_Black/African/Caribbean/BlackBritish_Total;measures_Value	LA
103	18	Sex_Allpersons;Age_Allcategories_Age;EthnicGroup_Black/African/Caribbean/BlackBritish_African;measures_Value	LA
104	19	Sex_Allpersons;Age_Allcategories_Age;EthnicGroup_Black/African/Caribbean/BlackBritish_Caribbean;measures_Value	LA
105	20	Sex_Allpersons;Age_Allcategories_Age;EthnicGroup_Black/African/Caribbean/BlackBritish_OtherBlack;measures_Value	LA
106	21	Sex_Allpersons;Age_Allcategories_Age;EthnicGroup_Otherethnicgroup_Total;measures_Value	LA
107	22	Sex_Allpersons;Age_Allcategories_Age;EthnicGroup_Otherethnicgroup_Arab;measures_Value	LA
108	23	Sex_Allpersons;Age_Allcategories_Age;EthnicGroup_Otherethnicgroup_Anyotherethnicgroup;measures_Value	LA

109	24	Sex_Allpersons;Age_Age0to24;EthnicGroup_Allcategories_Ethnicgroup;measures_Value	LA
110	25	Sex_Allpersons;Age_Age0to24;EthnicGroup_White_Total;measures_Value	LA
111	26	Sex_Allpersons;Age_Age0to24;EthnicGroup_White_English/Welsh/Scottish/NorthernIrish/British;measures_Value	LA
112	27	Sex_Allpersons;Age_Age0to24;EthnicGroup_White_Irish;measures_Value	LA
113	28	Sex_Allpersons;Age_Age0to24;EthnicGroup_White_GypsyorIrishTraveller;measures_Value	LA
114	29	Sex_Allpersons;Age_Age0to24;EthnicGroup_White_OtherWhite;measures_Value	LA
115	30	Sex_Allpersons;Age_Age0to24;EthnicGroup_Mixed/multipleethnicgroup_Total;measures_Value	LA
116	31	Sex_Allpersons;Age_Age0to24;EthnicGroup_Mixed/multipleethnicgroup_WhiteandBlackCaribbean;measures_Value	LA
117	32	Sex_Allpersons;Age_Age0to24;EthnicGroup_Mixed/multipleethnicgroup_WhiteandBlackAfrican;measures_Value	LA
118	33	Sex_Allpersons;Age_Age0to24;EthnicGroup_Mixed/multipleethnicgroup_WhiteandAsian;measures_Value	LA
119	34	Sex_Allpersons;Age_Age0to24;EthnicGroup_Mixed/multipleethnicgroup_OtherMixed;measures_Value	LA
120	35	Sex_Allpersons;Age_Age0to24;EthnicGroup_Asian/AsianBritish_Total;measures_Value	LA
121	36	Sex_Allpersons;Age_Age0to24;EthnicGroup_Asian/AsianBritish_Indian;measures_Value	LA
122	37	Sex_Allpersons;Age_Age0to24;EthnicGroup_Asian/AsianBritish_Pakistani;measures_Value	LA
123	38	Sex_Allpersons;Age_Age0to24;EthnicGroup_Asian/AsianBritish_Bangladeshi;measures_Value	LA
124	39	Sex_Allpersons;Age_Age0to24;EthnicGroup_Asian/AsianBritish_Chinese;measures_Value	LA
125	40	Sex_Allpersons;Age_Age0to24;EthnicGroup_Asian/AsianBritish_OtherAsian;measures_Value	LA
126	41	Sex_Allpersons;Age_Age0to24;EthnicGroup_Black/African/Caribbean/BlackBritish_Total;measures_Value	LA
127	42	Sex_Allpersons;Age_Age0to24;EthnicGroup_Black/African/Caribbean/BlackBritish_African;measures_Value	LA
128	43	Sex_Allpersons;Age_Age0to24;EthnicGroup_Black/African/Caribbean/BlackBritish_Caribbean;measures_Value	LA
129	44	Sex_Allpersons;Age_Age0to24;EthnicGroup_Black/African/Caribbean/BlackBritish_OtherBlack;measures_Value	LA
130	45	Sex_Allpersons;Age_Age0to24;EthnicGroup_Otherethnicgroup_Total;measures_Value	LA
131	46	Sex_Allpersons;Age_Age0to24;EthnicGroup_Otherethnicgroup_Arab;measures_Value	LA
132	47	Sex_Allpersons;Age_Age0to24;EthnicGroup_Otherethnicgroup_Anyotherethnicgroup;measures_Value	LA
133	48	Sex_Allpersons;Age_Age25to49;EthnicGroup_Allcategories_Ethnicgroup;measures_Value	LA
134	49	Sex_Allpersons;Age_Age25to49;EthnicGroup_White_Total;measures_Value	LA
135	50	Sex_Allpersons;Age_Age25to49;EthnicGroup_White_English/Welsh/Scottish/NorthernIrish/British;measures_Value	LA
136	51	Sex_Allpersons;Age_Age25to49;EthnicGroup_White_Irish;measures_Value	LA
137	52	Sex_Allpersons;Age_Age25to49;EthnicGroup_White_GypsyorIrishTraveller;measures_Value	LA
138	53	Sex_Allpersons;Age_Age25to49;EthnicGroup_White_OtherWhite;measures_Value	LA
139	54	Sex_Allpersons;Age_Age25to49;EthnicGroup_Mixed/multipleethnicgroup_Total;measures_Value	LA
140	55	Sex_Allpersons;Age_Age25to49;EthnicGroup_Mixed/multipleethnicgroup_WhiteandBlackCaribbean;measures_Value	LA
141	56	Sex_Allpersons;Age_Age25to49;EthnicGroup_Mixed/multipleethnicgroup_WhiteandBlackAfrican;measures_Value	LA
142	57	Sex_Allpersons;Age_Age25to49;EthnicGroup_Mixed/multipleethnicgroup_WhiteandAsian;measures_Value	LA
143	58	Sex_Allpersons;Age_Age25to49;EthnicGroup_Mixed/multipleethnicgroup_OtherMixed;measures_Value	LA
144	59	Sex_Allpersons;Age_Age25to49;EthnicGroup_Asian/AsianBritish_Total;measures_Value	LA
145	60	Sex_Allpersons;Age_Age25to49;EthnicGroup_Asian/AsianBritish_Indian;measures_Value	LA
146	61	Sex_Allpersons;Age_Age25to49;EthnicGroup_Asian/AsianBritish_Pakistani;measures_Value	LA

147	62	Sex_Allpersons;Age_Age25to49;EthnicGroup_Asian/AsianBritish_Bangladeshi;measures_Value	LA
148	63	Sex_Allpersons;Age_Age25to49;EthnicGroup_Asian/AsianBritish_Chinese;measures_Value	LA
149	64	Sex_Allpersons;Age_Age25to49;EthnicGroup_Asian/AsianBritish_OtherAsian;measures_Value	LA
150	65	Sex_Allpersons;Age_Age25to49;EthnicGroup_Black/African/Caribbean/BlackBritish_Total;measures_Value	LA
151	66	Sex_Allpersons;Age_Age25to49;EthnicGroup_Black/African/Caribbean/BlackBritish_African;measures_Value	LA
152	67	Sex_Allpersons;Age_Age25to49;EthnicGroup_Black/African/Caribbean/BlackBritish_Caribbean;measures_Value	LA
153	68	Sex_Allpersons;Age_Age25to49;EthnicGroup_Black/African/Caribbean/BlackBritish_OtherBlack;measures_Value	LA
154	69	Sex_Allpersons;Age_Age25to49;EthnicGroup_Otherethnicgroup_Total;measures_Value	LA
155	70	Sex_Allpersons;Age_Age25to49;EthnicGroup_Otherethnicgroup_Arab;measures_Value	LA
156	71	Sex_Allpersons;Age_Age25to49;EthnicGroup_Otherethnicgroup_Anyotherethnicgroup;measures_Value	LA
157	72	Sex_Allpersons;Age_Age50to64;EthnicGroup_Allcategories_Ethnicgroup;measures_Value	LA
158	73	Sex_Allpersons;Age_Age50to64;EthnicGroup_White_Total;measures_Value	LA
159	74	Sex_Allpersons;Age_Age50to64;EthnicGroup_White_English/Welsh/Scottish/NorthernIrish/British;measures_Value	LA
160	75	Sex_Allpersons;Age_Age50to64;EthnicGroup_White_Irish;measures_Value	LA
161	76	Sex_Allpersons;Age_Age50to64;EthnicGroup_White_GypsyorIrishTraveller;measures_Value	LA
162	77	Sex_Allpersons;Age_Age50to64;EthnicGroup_White_OtherWhite;measures_Value	LA
163	78	Sex_Allpersons;Age_Age50to64;EthnicGroup_Mixed/multipleethnicgroup_Total;measures_Value	LA
164	79	Sex_Allpersons;Age_Age50to64;EthnicGroup_Mixed/multipleethnicgroup_WhiteandBlackCaribbean;measures_Value	LA
165	80	Sex_Allpersons;Age_Age50to64;EthnicGroup_Mixed/multipleethnicgroup_WhiteandBlackAfrican;measures_Value	LA
166	81	Sex_Allpersons;Age_Age50to64;EthnicGroup_Mixed/multipleethnicgroup_WhiteandAsian;measures_Value	LA
167	82	Sex_Allpersons;Age_Age50to64;EthnicGroup_Mixed/multipleethnicgroup_OtherMixed;measures_Value	LA
168	83	Sex_Allpersons;Age_Age50to64;EthnicGroup_Asian/AsianBritish_Total;measures_Value	LA
169	84	Sex_Allpersons;Age_Age50to64;EthnicGroup_Asian/AsianBritish_Indian;measures_Value	LA
170	85	Sex_Allpersons;Age_Age50to64;EthnicGroup_Asian/AsianBritish_Pakistani;measures_Value	LA
171	86	Sex_Allpersons;Age_Age50to64;EthnicGroup_Asian/AsianBritish_Bangladeshi;measures_Value	LA
172	87	Sex_Allpersons;Age_Age50to64;EthnicGroup_Asian/AsianBritish_Chinese;measures_Value	LA
173	88	Sex_Allpersons;Age_Age50to64;EthnicGroup_Asian/AsianBritish_OtherAsian;measures_Value	LA
174	89	Sex_Allpersons;Age_Age50to64;EthnicGroup_Black/African/Caribbean/BlackBritish_Total;measures_Value	LA
175	90	Sex_Allpersons;Age_Age50to64;EthnicGroup_Black/African/Caribbean/BlackBritish_African;measures_Value	LA
176	91	Sex_Allpersons;Age_Age50to64;EthnicGroup_Black/African/Caribbean/BlackBritish_Caribbean;measures_Value	LA
177	92	Sex_Allpersons;Age_Age50to64;EthnicGroup_Black/African/Caribbean/BlackBritish_OtherBlack;measures_Value	LA
178	93	Sex_Allpersons;Age_Age50to64;EthnicGroup_Otherethnicgroup_Total;measures_Value	LA
179	94	Sex_Allpersons;Age_Age50to64;EthnicGroup_Otherethnicgroup_Arab;measures_Value	LA
180	95	Sex_Allpersons;Age_Age50to64;EthnicGroup_Otherethnicgroup_Anyotherethnicgroup;measures_Value	LA
181	96	Sex_Allpersons;Age_Age65andover;EthnicGroup_Allcategories_Ethnicgroup;measures_Value	LA
182	97	Sex_Allpersons;Age_Age65andover;EthnicGroup_White_Total;measures_Value	LA
183	98	Sex_Allpersons;Age_Age65andover;EthnicGroup_White_English/Welsh/Scottish/NorthernIrish/British;measures_Value	LA
184	99	Sex_Allpersons;Age_Age65andover;EthnicGroup_White_Irish;measures_Value	LA

185	100	Sex_Allpersons;Age_Age65andover;EthnicGroup_White_GypsyorIrishTraveller;measures_Value	LA
186	101	Sex_Allpersons;Age_Age65andover;EthnicGroup_White_OtherWhite;measures_Value	LA
187	102	Sex_Allpersons;Age_Age65andover;EthnicGroup_Mixed/multipleethnicgroup_Total;measures_Value	LA
188	103	Sex_Allpersons;Age_Age65andover;EthnicGroup_Mixed/multipleethnicgroup_WhiteandBlackCaribbean;measures_Value	LA
189	104	Sex_Allpersons;Age_Age65andover;EthnicGroup_Mixed/multipleethnicgroup_WhiteandBlackAfrican;measures_Value	LA
190	105	Sex_Allpersons;Age_Age65andover;EthnicGroup_Mixed/multipleethnicgroup_WhiteandAsian;measures_Value	LA
191	106	Sex_Allpersons;Age_Age65andover;EthnicGroup_Mixed/multipleethnicgroup_OtherMixed;measures_Value	LA
192	107	Sex_Allpersons;Age_Age65andover;EthnicGroup_Asian/AsianBritish_Total;measures_Value	LA
193	108	Sex_Allpersons;Age_Age65andover;EthnicGroup_Asian/AsianBritish_Indian;measures_Value	LA
194	109	Sex_Allpersons;Age_Age65andover;EthnicGroup_Asian/AsianBritish_Pakistani;measures_Value	LA
195	110	Sex_Allpersons;Age_Age65andover;EthnicGroup_Asian/AsianBritish_Bangladeshi;measures_Value	LA
196	111	Sex_Allpersons;Age_Age65andover;EthnicGroup_Asian/AsianBritish_Chinese;measures_Value	LA
197	112	Sex_Allpersons;Age_Age65andover;EthnicGroup_Asian/AsianBritish_OtherAsian;measures_Value	LA
198	113	Sex_Allpersons;Age_Age65andover;EthnicGroup_Black/African/Caribbean/BlackBritish_Total;measures_Value	LA
199	114	Sex_Allpersons;Age_Age65andover;EthnicGroup_Black/African/Caribbean/BlackBritish_African;measures_Value	LA
200	115	Sex_Allpersons;Age_Age65andover;EthnicGroup_Black/African/Caribbean/BlackBritish_Caribbean;measures_Value	LA
201	116	Sex_Allpersons;Age_Age65andover;EthnicGroup_Black/African/Caribbean/BlackBritish_OtherBlack;measures_Value	LA
202	117	Sex_Allpersons;Age_Age65andover;EthnicGroup_Otherethnicgroup_Total;measures_Value	LA
203	118	Sex_Allpersons;Age_Age65andover;EthnicGroup_Otherethnicgroup_Arab;measures_Value	LA
204	119	Sex_Allpersons;Age_Age65andover;EthnicGroup_Otherethnicgroup_Anyotherethnicgroup;measures_Value	LA
205	120	Sex_Males;Age_Allcategories_Age;EthnicGroup_Allcategories_Ethnicgroup;measures_Value	LA
206	121	Sex_Males;Age_Allcategories_Age;EthnicGroup_White_Total;measures_Value	LA
207	122	Sex_Males;Age_Allcategories_Age;EthnicGroup_White_English/Welsh/Scottish/NorthernIrish/British;measures_Value	LA
208	123	Sex_Males;Age_Allcategories_Age;EthnicGroup_White_Irish;measures_Value	LA
209	124	Sex_Males;Age_Allcategories_Age;EthnicGroup_White_GypsyorIrishTraveller;measures_Value	LA
210	125	Sex_Males;Age_Allcategories_Age;EthnicGroup_White_OtherWhite;measures_Value	LA
211	126	Sex_Males;Age_Allcategories_Age;EthnicGroup_Mixed/multipleethnicgroup_Total;measures_Value	LA
212	127	Sex_Males;Age_Allcategories_Age;EthnicGroup_Mixed/multipleethnicgroup_WhiteandBlackCaribbean;measures_Value	LA
213	128	Sex_Males;Age_Allcategories_Age;EthnicGroup_Mixed/multipleethnicgroup_WhiteandBlackAfrican;measures_Value	LA
214	129	Sex_Males;Age_Allcategories_Age;EthnicGroup_Mixed/multipleethnicgroup_WhiteandAsian;measures_Value	LA
215	130	Sex_Males;Age_Allcategories_Age;EthnicGroup_Mixed/multipleethnicgroup_OtherMixed;measures_Value	LA
216	131	Sex_Males;Age_Allcategories_Age;EthnicGroup_Asian/AsianBritish_Total;measures_Value	LA
217	132	Sex_Males;Age_Allcategories_Age;EthnicGroup_Asian/AsianBritish_Indian;measures_Value	LA
218	133	Sex_Males;Age_Allcategories_Age;EthnicGroup_Asian/AsianBritish_Pakistani;measures_Value	LA
219	134	Sex_Males;Age_Allcategories_Age;EthnicGroup_Asian/AsianBritish_Bangladeshi;measures_Value	LA
220	135	Sex_Males;Age_Allcategories_Age;EthnicGroup_Asian/AsianBritish_Chinese;measures_Value	LA
221	136	Sex_Males;Age_Allcategories_Age;EthnicGroup_Asian/AsianBritish_OtherAsian;measures_Value	LA
222	137	Sex_Males;Age_Allcategories_Age;EthnicGroup_Black/African/Caribbean/BlackBritish_Total;measures_Value	LA

223	138	Sex_Males;Age_Allcategories_Age;EthnicGroup_Black/African/Caribbean/BlackBritish_African;measures_Value	LA
224	139	Sex_Males;Age_Allcategories_Age;EthnicGroup_Black/African/Caribbean/BlackBritish_Caribbean;measures_Value	LA
225	140	Sex_Males;Age_Allcategories_Age;EthnicGroup_Black/African/Caribbean/BlackBritish_OtherBlack;measures_Value	LA
226	141	Sex_Males;Age_Allcategories_Age;EthnicGroup_Otherethnicgroup_Total;measures_Value	LA
227	142	Sex_Males;Age_Allcategories_Age;EthnicGroup_Otherethnicgroup_Arab;measures_Value	LA
228	143	Sex_Males;Age_Allcategories_Age;EthnicGroup_Otherethnicgroup_Anyotherethnicgroup;measures_Value	LA
229	144	Sex_Males;Age_Age0to24;EthnicGroup_Allcategories_Ethnicgroup;measures_Value	LA
230	145	Sex_Males;Age_Age0to24;EthnicGroup_White_Total;measures_Value	LA
231	146	Sex_Males;Age_Age0to24;EthnicGroup_White_English/Welsh/Scottish/NorthernIrish/British;measures_Value	LA
232	147	Sex_Males;Age_Age0to24;EthnicGroup_White_Irish;measures_Value	LA
233	148	Sex_Males;Age_Age0to24;EthnicGroup_White_GypsyorIrishTraveller;measures_Value	LA
234	149	Sex_Males;Age_Age0to24;EthnicGroup_White_OtherWhite;measures_Value	LA
235	150	Sex_Males;Age_Age0to24;EthnicGroup_Mixed/multipleethnicgroup_Total;measures_Value	LA
236	151	Sex_Males;Age_Age0to24;EthnicGroup_Mixed/multipleethnicgroup_WhiteandBlackCaribbean;measures_Value	LA
237	152	Sex_Males;Age_Age0to24;EthnicGroup_Mixed/multipleethnicgroup_WhiteandBlackAfrican;measures_Value	LA
238	153	Sex_Males;Age_Age0to24;EthnicGroup_Mixed/multipleethnicgroup_WhiteandAsian;measures_Value	LA
239	154	Sex_Males;Age_Age0to24;EthnicGroup_Mixed/multipleethnicgroup_OtherMixed;measures_Value	LA
240	155	Sex_Males;Age_Age0to24;EthnicGroup_Asian/AsianBritish_Total;measures_Value	LA
241	156	Sex_Males;Age_Age0to24;EthnicGroup_Asian/AsianBritish_Indian;measures_Value	LA
242	157	Sex_Males;Age_Age0to24;EthnicGroup_Asian/AsianBritish_Pakistani;measures_Value	LA
243	158	Sex_Males;Age_Age0to24;EthnicGroup_Asian/AsianBritish_Bangladeshi;measures_Value	LA
244	159	Sex_Males;Age_Age0to24;EthnicGroup_Asian/AsianBritish_Chinese;measures_Value	LA
245	160	Sex_Males;Age_Age0to24;EthnicGroup_Asian/AsianBritish_OtherAsian;measures_Value	LA
246	161	Sex_Males;Age_Age0to24;EthnicGroup_Black/African/Caribbean/BlackBritish_Total;measures_Value	LA
247	162	Sex_Males;Age_Age0to24;EthnicGroup_Black/African/Caribbean/BlackBritish_African;measures_Value	LA
248	163	Sex_Males;Age_Age0to24;EthnicGroup_Black/African/Caribbean/BlackBritish_Caribbean;measures_Value	LA
249	164	Sex_Males;Age_Age0to24;EthnicGroup_Black/African/Caribbean/BlackBritish_OtherBlack;measures_Value	LA
250	165	Sex_Males;Age_Age0to24;EthnicGroup_Otherethnicgroup_Total;measures_Value	LA
251	166	Sex_Males;Age_Age0to24;EthnicGroup_Otherethnicgroup_Arab;measures_Value	LA
252	167	Sex_Males;Age_Age0to24;EthnicGroup_Otherethnicgroup_Anyotherethnicgroup;measures_Value	LA
253	168	Sex_Males;Age_Age25to49;EthnicGroup_Allcategories_Ethnicgroup;measures_Value	LA
254	169	Sex_Males;Age_Age25to49;EthnicGroup_White_Total;measures_Value	LA
255	170	Sex_Males;Age_Age25to49;EthnicGroup_White_English/Welsh/Scottish/NorthernIrish/British;measures_Value	LA
256	171	Sex_Males;Age_Age25to49;EthnicGroup_White_Irish;measures_Value	LA
257	172	Sex_Males;Age_Age25to49;EthnicGroup_White_GypsyorIrishTraveller;measures_Value	LA
258	173	Sex_Males;Age_Age25to49;EthnicGroup_White_OtherWhite;measures_Value	LA
259	174	Sex_Males;Age_Age25to49;EthnicGroup_Mixed/multipleethnicgroup_Total;measures_Value	LA
260	175	Sex_Males;Age_Age25to49;EthnicGroup_Mixed/multipleethnicgroup_WhiteandBlackCaribbean;measures_Value	LA

261	176	Sex_Males;Age_Age25to49;EthnicGroup_Mixed/multipleethnicgroup_WhiteandBlackAfrican;measures_Value	LA
262	177	Sex_Males;Age_Age25to49;EthnicGroup_Mixed/multipleethnicgroup_WhiteandAsian;measures_Value	LA
263	178	Sex_Males;Age_Age25to49;EthnicGroup_Mixed/multipleethnicgroup_OtherMixed;measures_Value	LA
264	179	Sex_Males;Age_Age25to49;EthnicGroup_Asian/AsianBritish_Total;measures_Value	LA
265	180	Sex_Males;Age_Age25to49;EthnicGroup_Asian/AsianBritish_Indian;measures_Value	LA
266	181	Sex_Males;Age_Age25to49;EthnicGroup_Asian/AsianBritish_Pakistani;measures_Value	LA
267	182	Sex_Males;Age_Age25to49;EthnicGroup_Asian/AsianBritish_Bangladeshi;measures_Value	LA
268	183	Sex_Males;Age_Age25to49;EthnicGroup_Asian/AsianBritish_Chinese;measures_Value	LA
269	184	Sex_Males;Age_Age25to49;EthnicGroup_Asian/AsianBritish_OtherAsian;measures_Value	LA
270	185	Sex_Males;Age_Age25to49;EthnicGroup_Black/African/Caribbean/BlackBritish_Total;measures_Value	LA
271	186	Sex_Males;Age_Age25to49;EthnicGroup_Black/African/Caribbean/BlackBritish_African;measures_Value	LA
272	187	Sex_Males;Age_Age25to49;EthnicGroup_Black/African/Caribbean/BlackBritish_Caribbean;measures_Value	LA
273	188	Sex_Males;Age_Age25to49;EthnicGroup_Black/African/Caribbean/BlackBritish_OtherBlack;measures_Value	LA
274	189	Sex_Males;Age_Age25to49;EthnicGroup_Otherethnicgroup_Total;measures_Value	LA
275	190	Sex_Males;Age_Age25to49;EthnicGroup_Otherethnicgroup_Arab;measures_Value	LA
276	191	Sex_Males;Age_Age25to49;EthnicGroup_Otherethnicgroup_Anyotherethnicgroup;measures_Value	LA
277	192	Sex_Males;Age_Age50to64;EthnicGroup_Allcategories_Ethnicgroup;measures_Value	LA
278	193	Sex_Males;Age_Age50to64;EthnicGroup_White_Total;measures_Value	LA
279	194	Sex_Males;Age_Age50to64;EthnicGroup_White_English/Welsh/Scottish/NorthernIrish/British;measures_Value	LA
280	195	Sex_Males;Age_Age50to64;EthnicGroup_White_Irish;measures_Value	LA
281	196	Sex_Males;Age_Age50to64;EthnicGroup_White_GypsyorIrishTraveller;measures_Value	LA
282	197	Sex_Males;Age_Age50to64;EthnicGroup_White_OtherWhite;measures_Value	LA
283	198	Sex_Males;Age_Age50to64;EthnicGroup_Mixed/multipleethnicgroup_Total;measures_Value	LA
284	199	Sex_Males;Age_Age50to64;EthnicGroup_Mixed/multipleethnicgroup_WhiteandBlackCaribbean;measures_Value	LA
285	200	Sex_Males;Age_Age50to64;EthnicGroup_Mixed/multipleethnicgroup_WhiteandBlackAfrican;measures_Value	LA
286	201	Sex_Males;Age_Age50to64;EthnicGroup_Mixed/multipleethnicgroup_WhiteandAsian;measures_Value	LA
287	202	Sex_Males;Age_Age50to64;EthnicGroup_Mixed/multipleethnicgroup_OtherMixed;measures_Value	LA
288	203	Sex_Males;Age_Age50to64;EthnicGroup_Asian/AsianBritish_Total;measures_Value	LA
289	204	Sex_Males;Age_Age50to64;EthnicGroup_Asian/AsianBritish_Indian;measures_Value	LA
290	205	Sex_Males;Age_Age50to64;EthnicGroup_Asian/AsianBritish_Pakistani;measures_Value	LA
291	206	Sex_Males;Age_Age50to64;EthnicGroup_Asian/AsianBritish_Bangladeshi;measures_Value	LA
292	207	Sex_Males;Age_Age50to64;EthnicGroup_Asian/AsianBritish_Chinese;measures_Value	LA
293	208	Sex_Males;Age_Age50to64;EthnicGroup_Asian/AsianBritish_OtherAsian;measures_Value	LA
294	209	Sex_Males;Age_Age50to64;EthnicGroup_Black/African/Caribbean/BlackBritish_Total;measures_Value	LA
295	210	Sex_Males;Age_Age50to64;EthnicGroup_Black/African/Caribbean/BlackBritish_African;measures_Value	LA
296	211	Sex_Males;Age_Age50to64;EthnicGroup_Black/African/Caribbean/BlackBritish_Caribbean;measures_Value	LA
297	212	Sex_Males;Age_Age50to64;EthnicGroup_Black/African/Caribbean/BlackBritish_OtherBlack;measures_Value	LA
298	213	Sex_Males;Age_Age50to64;EthnicGroup_Otherethnicgroup_Total;measures_Value	LA

299	214	Sex_Males;Age_Age50to64;EthnicGroup_Otherethnicgroup_Arab;measures_Value	LA
300	215	Sex_Males;Age_Age50to64;EthnicGroup_Otherethnicgroup_Anyotherethnicgroup;measures_Value	LA
301	216	Sex_Males;Age_Age65andover;EthnicGroup_Allcategories_Ethnicgroup;measures_Value	LA
302	217	Sex_Males;Age_Age65andover;EthnicGroup_White_Total;measures_Value	LA
303	218	Sex_Males;Age_Age65andover;EthnicGroup_White_English/Welsh/Scottish/NorthernIrish/British;measures_Value	LA
304	219	Sex_Males;Age_Age65andover;EthnicGroup_White_Irish;measures_Value	LA
305	220	Sex_Males;Age_Age65andover;EthnicGroup_White_GypsyorIrishTraveller;measures_Value	LA
306	221	Sex_Males;Age_Age65andover;EthnicGroup_White_OtherWhite;measures_Value	LA
307	222	Sex_Males;Age_Age65andover;EthnicGroup_Mixed/multipleethnicgroup_Total;measures_Value	LA
308	223	Sex_Males;Age_Age65andover;EthnicGroup_Mixed/multipleethnicgroup_WhiteandBlackCaribbean;measures_Value	LA
309	224	Sex_Males;Age_Age65andover;EthnicGroup_Mixed/multipleethnicgroup_WhiteandBlackAfrican;measures_Value	LA
310	225	Sex_Males;Age_Age65andover;EthnicGroup_Mixed/multipleethnicgroup_WhiteandAsian;measures_Value	LA
311	226	Sex_Males;Age_Age65andover;EthnicGroup_Mixed/multipleethnicgroup_OtherMixed;measures_Value	LA
312	227	Sex_Males;Age_Age65andover;EthnicGroup_Asian/AsianBritish_Total;measures_Value	LA
313	228	Sex_Males;Age_Age65andover;EthnicGroup_Asian/AsianBritish_Indian;measures_Value	LA
314	229	Sex_Males;Age_Age65andover;EthnicGroup_Asian/AsianBritish_Pakistani;measures_Value	LA
315	230	Sex_Males;Age_Age65andover;EthnicGroup_Asian/AsianBritish_Bangladeshi;measures_Value	LA
316	231	Sex_Males;Age_Age65andover;EthnicGroup_Asian/AsianBritish_Chinese;measures_Value	LA
317	232	Sex_Males;Age_Age65andover;EthnicGroup_Asian/AsianBritish_OtherAsian;measures_Value	LA
318	233	Sex_Males;Age_Age65andover;EthnicGroup_Black/African/Caribbean/BlackBritish_Total;measures_Value	LA
319	234	Sex_Males;Age_Age65andover;EthnicGroup_Black/African/Caribbean/BlackBritish_African;measures_Value	LA
320	235	Sex_Males;Age_Age65andover;EthnicGroup_Black/African/Caribbean/BlackBritish_Caribbean;measures_Value	LA
321	236	Sex_Males;Age_Age65andover;EthnicGroup_Black/African/Caribbean/BlackBritish_OtherBlack;measures_Value	LA
322	237	Sex_Males;Age_Age65andover;EthnicGroup_Otherethnicgroup_Total;measures_Value	LA
323	238	Sex_Males;Age_Age65andover;EthnicGroup_Otherethnicgroup_Arab;measures_Value	LA
324	239	Sex_Males;Age_Age65andover;EthnicGroup_Otherethnicgroup_Anyotherethnicgroup;measures_Value	LA
325	240	Sex_Females;Age_Allcategories_Age;EthnicGroup_Allcategories_Ethnicgroup;measures_Value	LA
326	241	Sex_Females;Age_Allcategories_Age;EthnicGroup_White_Total;measures_Value	LA
327	242	Sex_Females;Age_Allcategories_Age;EthnicGroup_White_English/Welsh/Scottish/NorthernIrish/British;measures_Value	LA
328	243	Sex_Females;Age_Allcategories_Age;EthnicGroup_White_Irish;measures_Value	LA
329	244	Sex_Females;Age_Allcategories_Age;EthnicGroup_White_GypsyorIrishTraveller;measures_Value	LA
330	245	Sex_Females;Age_Allcategories_Age;EthnicGroup_White_OtherWhite;measures_Value	LA
331	246	Sex_Females;Age_Allcategories_Age;EthnicGroup_Mixed/multipleethnicgroup_Total;measures_Value	LA
332	247	Sex_Females;Age_Allcategories_Age;EthnicGroup_Mixed/multipleethnicgroup_WhiteandBlackCaribbean;measures_Value	LA
333	248	Sex_Females;Age_Allcategories_Age;EthnicGroup_Mixed/multipleethnicgroup_WhiteandBlackAfrican;measures_Value	LA
334	249	Sex_Females;Age_Allcategories_Age;EthnicGroup_Mixed/multipleethnicgroup_WhiteandAsian;measures_Value	LA
335	250	Sex_Females;Age_Allcategories_Age;EthnicGroup_Mixed/multipleethnicgroup_OtherMixed;measures_Value	LA
336	251	Sex_Females;Age_Allcategories_Age;EthnicGroup_Asian/AsianBritish_Total;measures_Value	LA

337	252	Sex_Females;Age_Allcategories_Age;EthnicGroup_Asian/AsianBritish_Indian;measures_Value	LA
338	253	Sex_Females;Age_Allcategories_Age;EthnicGroup_Asian/AsianBritish_Pakistani;measures_Value	LA
339	254	Sex_Females;Age_Allcategories_Age;EthnicGroup_Asian/AsianBritish_Bangladeshi;measures_Value	LA
340	255	Sex_Females;Age_Allcategories_Age;EthnicGroup_Asian/AsianBritish_Chinese;measures_Value	LA
341	256	Sex_Females;Age_Allcategories_Age;EthnicGroup_Asian/AsianBritish_OtherAsian;measures_Value	LA
342	257	Sex_Females;Age_Allcategories_Age;EthnicGroup_Black/African/Caribbean/BlackBritish_Total;measures_Value	LA
343	258	Sex_Females;Age_Allcategories_Age;EthnicGroup_Black/African/Caribbean/BlackBritish_African;measures_Value	LA
344	259	Sex_Females;Age_Allcategories_Age;EthnicGroup_Black/African/Caribbean/BlackBritish_Caribbean;measures_Value	LA
345	260	Sex_Females;Age_Allcategories_Age;EthnicGroup_Black/African/Caribbean/BlackBritish_OtherBlack;measures_Value	LA
346	261	Sex_Females;Age_Allcategories_Age;EthnicGroup_Otherethnicgroup_Total;measures_Value	LA
347	262	Sex_Females;Age_Allcategories_Age;EthnicGroup_Otherethnicgroup_Arab;measures_Value	LA
348	263	Sex_Females;Age_Allcategories_Age;EthnicGroup_Otherethnicgroup_Anyotherethnicgroup;measures_Value	LA
349	264	Sex_Females;Age_Age0to24;EthnicGroup_Allcategories_Ethnicgroup;measures_Value	LA
350	265	Sex_Females;Age_Age0to24;EthnicGroup_White_Total;measures_Value	LA
351	266	Sex_Females;Age_Age0to24;EthnicGroup_White_English/Welsh/Scottish/NorthernIrish/British;measures_Value	LA
352	267	Sex_Females;Age_Age0to24;EthnicGroup_White_Irish;measures_Value	LA
353	268	Sex_Females;Age_Age0to24;EthnicGroup_White_GypsyorIrishTraveller;measures_Value	LA
354	269	Sex_Females;Age_Age0to24;EthnicGroup_White_OtherWhite;measures_Value	LA
355	270	Sex_Females;Age_Age0to24;EthnicGroup_Mixed/multipleethnicgroup_Total;measures_Value	LA
356	271	Sex_Females;Age_Age0to24;EthnicGroup_Mixed/multipleethnicgroup_WhiteandBlackCaribbean;measures_Value	LA
357	272	Sex_Females;Age_Age0to24;EthnicGroup_Mixed/multipleethnicgroup_WhiteandBlackAfrican;measures_Value	LA
358	273	Sex_Females;Age_Age0to24;EthnicGroup_Mixed/multipleethnicgroup_WhiteandAsian;measures_Value	LA
359	274	Sex_Females;Age_Age0to24;EthnicGroup_Mixed/multipleethnicgroup_OtherMixed;measures_Value	LA
360	275	Sex_Females;Age_Age0to24;EthnicGroup_Asian/AsianBritish_Total;measures_Value	LA
361	276	Sex_Females;Age_Age0to24;EthnicGroup_Asian/AsianBritish_Indian;measures_Value	LA
362	277	Sex_Females;Age_Age0to24;EthnicGroup_Asian/AsianBritish_Pakistani;measures_Value	LA
363	278	Sex_Females;Age_Age0to24;EthnicGroup_Asian/AsianBritish_Bangladeshi;measures_Value	LA
364	279	Sex_Females;Age_Age0to24;EthnicGroup_Asian/AsianBritish_Chinese;measures_Value	LA
365	280	Sex_Females;Age_Age0to24;EthnicGroup_Asian/AsianBritish_OtherAsian;measures_Value	LA
366	281	Sex_Females;Age_Age0to24;EthnicGroup_Black/African/Caribbean/BlackBritish_Total;measures_Value	LA
367	282	Sex_Females;Age_Age0to24;EthnicGroup_Black/African/Caribbean/BlackBritish_African;measures_Value	LA
368	283	Sex_Females;Age_Age0to24;EthnicGroup_Black/African/Caribbean/BlackBritish_Caribbean;measures_Value	LA
369	284	Sex_Females;Age_Age0to24;EthnicGroup_Black/African/Caribbean/BlackBritish_OtherBlack;measures_Value	LA
370	285	Sex_Females;Age_Age0to24;EthnicGroup_Otherethnicgroup_Total;measures_Value	LA
371	286	Sex_Females;Age_Age0to24;EthnicGroup_Otherethnicgroup_Arab;measures_Value	LA
372	287	Sex_Females;Age_Age0to24;EthnicGroup_Otherethnicgroup_Anyotherethnicgroup;measures_Value	LA
373	288	Sex_Females;Age_Age25to49;EthnicGroup_Allcategories_Ethnicgroup;measures_Value	LA
374	289	Sex_Females;Age_Age25to49;EthnicGroup_White_Total;measures_Value	LA

375	290	Sex_Females;Age_Age25to49;EthnicGroup_White_English/Welsh/Scottish/NorthernIrish/British;measures_Value	LA
376	291	Sex_Females;Age_Age25to49;EthnicGroup_White_Irish;measures_Value	LA
377	292	Sex_Females;Age_Age25to49;EthnicGroup_White_GypsyorIrishTraveller;measures_Value	LA
378	293	Sex_Females;Age_Age25to49;EthnicGroup_White_OtherWhite;measures_Value	LA
379	294	Sex_Females;Age_Age25to49;EthnicGroup_Mixed/multipleethnicgroup_Total;measures_Value	LA
380	295	Sex_Females;Age_Age25to49;EthnicGroup_Mixed/multipleethnicgroup_WhiteandBlackCaribbean;measures_Value	LA
381	296	Sex_Females;Age_Age25to49;EthnicGroup_Mixed/multipleethnicgroup_WhiteandBlackAfrican;measures_Value	LA
382	297	Sex_Females;Age_Age25to49;EthnicGroup_Mixed/multipleethnicgroup_WhiteandAsian;measures_Value	LA
383	298	Sex_Females;Age_Age25to49;EthnicGroup_Mixed/multipleethnicgroup_OtherMixed;measures_Value	LA
384	299	Sex_Females;Age_Age25to49;EthnicGroup_Asian/AsianBritish_Total;measures_Value	LA
385	300	Sex_Females;Age_Age25to49;EthnicGroup_Asian/AsianBritish_Indian;measures_Value	LA
386	301	Sex_Females;Age_Age25to49;EthnicGroup_Asian/AsianBritish_Pakistani;measures_Value	LA
387	302	Sex_Females;Age_Age25to49;EthnicGroup_Asian/AsianBritish_Bangladeshi;measures_Value	LA
388	303	Sex_Females;Age_Age25to49;EthnicGroup_Asian/AsianBritish_Chinese;measures_Value	LA
389	304	Sex_Females;Age_Age25to49;EthnicGroup_Asian/AsianBritish_OtherAsian;measures_Value	LA
390	305	Sex_Females;Age_Age25to49;EthnicGroup_Black/African/Caribbean/BlackBritish_Total;measures_Value	LA
391	306	Sex_Females;Age_Age25to49;EthnicGroup_Black/African/Caribbean/BlackBritish_African;measures_Value	LA
392	307	Sex_Females;Age_Age25to49;EthnicGroup_Black/African/Caribbean/BlackBritish_Caribbean;measures_Value	LA
393	308	Sex_Females;Age_Age25to49;EthnicGroup_Black/African/Caribbean/BlackBritish_OtherBlack;measures_Value	LA
394	309	Sex_Females;Age_Age25to49;EthnicGroup_Otherethnicgroup_Total;measures_Value	LA
395	310	Sex_Females;Age_Age25to49;EthnicGroup_Otherethnicgroup_Arab;measures_Value	LA
396	311	Sex_Females;Age_Age25to49;EthnicGroup_Otherethnicgroup_Anyotherethnicgroup;measures_Value	LA
397	312	Sex_Females;Age_Age50to64;EthnicGroup_Allcategories_Ethnicgroup;measures_Value	LA
398	313	Sex_Females;Age_Age50to64;EthnicGroup_White_Total;measures_Value	LA
399	314	Sex_Females;Age_Age50to64;EthnicGroup_White_English/Welsh/Scottish/NorthernIrish/British;measures_Value	LA
400	315	Sex_Females;Age_Age50to64;EthnicGroup_White_Irish;measures_Value	LA
401	316	Sex_Females;Age_Age50to64;EthnicGroup_White_GypsyorIrishTraveller;measures_Value	LA
402	317	Sex_Females;Age_Age50to64;EthnicGroup_White_OtherWhite;measures_Value	LA
403	318	Sex_Females;Age_Age50to64;EthnicGroup_Mixed/multipleethnicgroup_Total;measures_Value	LA
404	319	Sex_Females;Age_Age50to64;EthnicGroup_Mixed/multipleethnicgroup_WhiteandBlackCaribbean;measures_Value	LA
405	320	Sex_Females;Age_Age50to64;EthnicGroup_Mixed/multipleethnicgroup_WhiteandBlackAfrican;measures_Value	LA
406	321	Sex_Females;Age_Age50to64;EthnicGroup_Mixed/multipleethnicgroup_WhiteandAsian;measures_Value	LA
407	322	Sex_Females;Age_Age50to64;EthnicGroup_Mixed/multipleethnicgroup_OtherMixed;measures_Value	LA
408	323	Sex_Females;Age_Age50to64;EthnicGroup_Asian/AsianBritish_Total;measures_Value	LA
409	324	Sex_Females;Age_Age50to64;EthnicGroup_Asian/AsianBritish_Indian;measures_Value	LA
410	325	Sex_Females;Age_Age50to64;EthnicGroup_Asian/AsianBritish_Pakistani;measures_Value	LA
411	326	Sex_Females;Age_Age50to64;EthnicGroup_Asian/AsianBritish_Bangladeshi;measures_Value	LA
412	327	Sex_Females;Age_Age50to64;EthnicGroup_Asian/AsianBritish_Chinese;measures_Value	LA

413	328	Sex_Females;Age_Age50to64;EthnicGroup_Asian/AsianBritish_OtherAsian;measures_Value	LA
414	329	Sex_Females;Age_Age50to64;EthnicGroup_Black/African/Caribbean/BlackBritish_Total;measures_Value	LA
415	330	Sex_Females;Age_Age50to64;EthnicGroup_Black/African/Caribbean/BlackBritish_African;measures_Value	LA
416	331	Sex_Females;Age_Age50to64;EthnicGroup_Black/African/Caribbean/BlackBritish_Caribbean;measures_Value	LA
417	332	Sex_Females;Age_Age50to64;EthnicGroup_Black/African/Caribbean/BlackBritish_OtherBlack;measures_Value	LA
418	333	Sex_Females;Age_Age50to64;EthnicGroup_Otherethnicgroup_Total;measures_Value	LA
419	334	Sex_Females;Age_Age50to64;EthnicGroup_Otherethnicgroup_Arab;measures_Value	LA
420	335	Sex_Females;Age_Age50to64;EthnicGroup_Otherethnicgroup_Anyotherethnicgroup;measures_Value	LA
421	336	Sex_Females;Age_Age65andover;EthnicGroup_Allcategories_Ethnicgroup;measures_Value	LA
422	337	Sex_Females;Age_Age65andover;EthnicGroup_White_Total;measures_Value	LA
423	338	Sex_Females;Age_Age65andover;EthnicGroup_White_English/Welsh/Scottish/NorthernIrish/British;measures_Value	LA
424	339	Sex_Females;Age_Age65andover;EthnicGroup_White_Irish;measures_Value	LA
425	340	Sex_Females;Age_Age65andover;EthnicGroup_White_GypsyorIrishTraveller;measures_Value	LA
426	341	Sex_Females;Age_Age65andover;EthnicGroup_White_OtherWhite;measures_Value	LA
427	342	Sex_Females;Age_Age65andover;EthnicGroup_Mixed/multipleethnicgroup_Total;measures_Value	LA
428	343	Sex_Females;Age_Age65andover;EthnicGroup_Mixed/multipleethnicgroup_WhiteandBlackCaribbean;measures_Value	LA
429	344	Sex_Females;Age_Age65andover;EthnicGroup_Mixed/multipleethnicgroup_WhiteandBlackAfrican;measures_Value	LA
430	345	Sex_Females;Age_Age65andover;EthnicGroup_Mixed/multipleethnicgroup_WhiteandAsian;measures_Value	LA
431	346	Sex_Females;Age_Age65andover;EthnicGroup_Mixed/multipleethnicgroup_OtherMixed;measures_Value	LA
432	347	Sex_Females;Age_Age65andover;EthnicGroup_Asian/AsianBritish_Total;measures_Value	LA
433	348	Sex_Females;Age_Age65andover;EthnicGroup_Asian/AsianBritish_Indian;measures_Value	LA
434	349	Sex_Females;Age_Age65andover;EthnicGroup_Asian/AsianBritish_Pakistani;measures_Value	LA
435	350	Sex_Females;Age_Age65andover;EthnicGroup_Asian/AsianBritish_Bangladeshi;measures_Value	LA
436	351	Sex_Females;Age_Age65andover;EthnicGroup_Asian/AsianBritish_Chinese;measures_Value	LA
437	352	Sex_Females;Age_Age65andover;EthnicGroup_Asian/AsianBritish_OtherAsian;measures_Value	LA
438	353	Sex_Females;Age_Age65andover;EthnicGroup_Black/African/Caribbean/BlackBritish_Total;measures_Value	LA
439	354	Sex_Females;Age_Age65andover;EthnicGroup_Black/African/Caribbean/BlackBritish_African;measures_Value	LA
440	355	Sex_Females;Age_Age65andover;EthnicGroup_Black/African/Caribbean/BlackBritish_Caribbean;measures_Value	LA
441	356	Sex_Females;Age_Age65andover;EthnicGroup_Black/African/Caribbean/BlackBritish_OtherBlack;measures_Value	LA
442	357	Sex_Females;Age_Age65andover;EthnicGroup_Otherethnicgroup_Total;measures_Value	LA
443	358	Sex_Females;Age_Age65andover;EthnicGroup_Otherethnicgroup_Arab;measures_Value	LA
444	359	Sex_Females;Age_Age65andover;EthnicGroup_Otherethnicgroup_Anyotherethnicgroup;measures_Value	LA
445	360	Familystatus_Allcategories_Familystatusbynumberofparentsworking;Economicactivity_Economicallyactive_Total;measures_Value	LA
446	361	Familystatus_Allcategories_Familystatusbynumberofparentsworking;Economicactivity_Economicallyactive_Inemployment_Total;measures_Value	LA
447	362	Familystatus_Allcategories_Familystatusbynumberofparentsworking;Economicactivity_Economicallyactive_Inemployment_Employee(includingfull-timestudents);measures_Value	LA
448	363	Familystatus_Allcategories_Familystatusbynumberofparentsworking;Economicactivity_Economicallyactive_Inemployment_Self-employed(includingfull-timestudents);measures_Value	LA
449	364	Familystatus_Allcategories_Familystatusbynumberofparentsworking;Economicactivity_Economicallyactive_Unemployed(includingfull-timestudents);measures_Value	LA
450	365	Familystatus_Allcategories_Familystatusbynumberofparentsworking;Economicactivity_Economicallyinactive_Total;measures_Value	LA

451	366	Familystatus_Allcategories_Familystatusbynumberofparentsworking;Economicactivity_Economicallyinactive_Retired;measures_Value	LA
452	367	Familystatus_Allcategories_Familystatusbynumberofparentsworking;Economicactivity_Economicallyinactive_Student(includingfull-timestudents);measures_Value	LA
453	368	Familystatus_Allcategories_Familystatusbynumberofparentsworking;Economicactivity_Economicallyinactive_Lookingafterhomeorfamily;measures_Value	LA
454	369	Familystatus_Allcategories_Familystatusbynumberofparentsworking;Economicactivity_Economicallyinactive_Long-termsickordisabled;measures_Value	LA
455	370	Familystatus_Allcategories_Familystatusbynumberofparentsworking;Economicactivity_Economicallyinactive_Other;measures_Value	LA
456	371	Familystatus_Couplefamily_Total;Economicactivity_Allcategories_Economicactivity;measures_Value	LA
457	372	Familystatus_Couplefamily_Total;Economicactivity_Economicallyactive_Total;measures_Value	LA
458	373	Familystatus_Couplefamily_Total;Economicactivity_Economicallyactive_Inemployment_Total;measures_Value	LA
459	374	Familystatus_Couplefamily_Total;Economicactivity_Economicallyactive_Inemployment_Employee(includingfull-timestudents);measures_Value	LA
460	375	Familystatus_Couplefamily_Total;Economicactivity_Economicallyactive_Inemployment_Self-employed(includingfull-timestudents);measures_Value	LA
461	376	Familystatus_Couplefamily_Total;Economicactivity_Economicallyactive_Unemployed(includingfull-timestudents);measures_Value	LA
462	377	Familystatus_Couplefamily_Total;Economicactivity_Economicallyinactive_Total;measures_Value	LA
463	378	Familystatus_Couplefamily_Total;Economicactivity_Economicallyinactive_Retired;measures_Value	LA
464	379	Familystatus_Couplefamily_Total;Economicactivity_Economicallyinactive_Student(includingfull-timestudents);measures_Value	LA
465	380	Familystatus_Couplefamily_Total;Economicactivity_Economicallyinactive_Lookingafterhomeorfamily;measures_Value	LA
466	381	Familystatus_Couplefamily_Total;Economicactivity_Economicallyinactive_Long-termsickordisabled;measures_Value	LA
467	382	Familystatus_Couplefamily_Total;Economicactivity_Economicallyinactive_Other;measures_Value	LA
468	383	Familystatus_Couplefamily_Bothparentsworking;Economicactivity_Allcategories_Economicactivity;measures_Value	LA
469	384	Familystatus_Couplefamily_Bothparentsworking;Economicactivity_Economicallyactive_Total;measures_Value	LA
470	385	Familystatus_Couplefamily_Bothparentsworking;Economicactivity_Economicallyactive_Inemployment_Total;measures_Value	LA
471	386	Familystatus_Couplefamily_Bothparentsworking;Economicactivity_Economicallyactive_Inemployment_Employee(includingfull-timestudents);measures_Value	LA
472	387	Familystatus_Couplefamily_Bothparentsworking;Economicactivity_Economicallyactive_Inemployment_Self-employed(includingfull-timestudents);measures_Value	LA
473	388	Familystatus_Couplefamily_Bothparentsworking;Economicactivity_Economicallyactive_Unemployed(includingfull-timestudents);measures_Value	LA
474	389	Familystatus_Couplefamily_Bothparentsworking;Economicactivity_Economicallyinactive_Total;measures_Value	LA
475	390	Familystatus_Couplefamily_Bothparentsworking;Economicactivity_Economicallyinactive_Retired;measures_Value	LA
476	391	Familystatus_Couplefamily_Bothparentsworking;Economicactivity_Economicallyinactive_Student(includingfull-timestudents);measures_Value	LA
477	392	Familystatus_Couplefamily_Bothparentsworking;Economicactivity_Economicallyinactive_Lookingafterhomeorfamily;measures_Value	LA
478	393	Familystatus_Couplefamily_Bothparentsworking;Economicactivity_Economicallyinactive_Long-termsickordisabled;measures_Value	LA
479	394	Familystatus_Couplefamily_Bothparentsworking;Economicactivity_Economicallyinactive_Other;measures_Value	LA
480	395	Familystatus_Couplefamily_Oneparentworking;Economicactivity_Allcategories_Economicactivity;measures_Value	LA
481	396	Familystatus_Couplefamily_Oneparentworking;Economicactivity_Economicallyactive_Total;measures_Value	LA
482	397	Familystatus_Couplefamily_Oneparentworking;Economicactivity_Economicallyactive_Inemployment_Total;measures_Value	LA
483	398	Familystatus_Couplefamily_Oneparentworking;Economicactivity_Economicallyactive_Inemployment_Employee(includingfull-timestudents);measures_Value	LA
484	399	Familystatus_Couplefamily_Oneparentworking;Economicactivity_Economicallyactive_Inemployment_Self-employed(includingfull-timestudents);measures_Value	LA
485	400	Familystatus_Couplefamily_Oneparentworking;Economicactivity_Economicallyactive_Unemployed(includingfull-timestudents);measures_Value	LA
486	401	Familystatus_Couplefamily_Oneparentworking;Economicactivity_Economicallyinactive_Total;measures_Value	LA
487	402	Familystatus_Couplefamily_Oneparentworking;Economicactivity_Economicallyinactive_Retired;measures_Value	LA
488	403	Familystatus_Couplefamily_Oneparentworking;Economicactivity_Economicallyinactive_Student(includingfull-timestudents);measures_Value	LA

489	404	Familystatus_Couplefamily_Oneparentworking;Economicactivity_Economicallyinactive_Lookingafterhomeorfamily;measures_Value	LA
490	405	Familystatus_Couplefamily_Oneparentworking;Economicactivity_Economicallyinactive_Long-termsickordisabled;measures_Value	LA
491	406	Familystatus_Couplefamily_Oneparentworking;Economicactivity_Economicallyinactive_Other;measures_Value	LA
492	407	Familystatus_Couplefamily_Noparentsworking;Economicactivity_Allcategories_Economicactivity;measures_Value	LA
493	408	Familystatus_Couplefamily_Noparentsworking;Economicactivity_Economicallyactive_Total;measures_Value	LA
494	409	Familystatus_Couplefamily_Noparentsworking;Economicactivity_Economicallyactive_Inemployment_Total;measures_Value	LA
495	410	Familystatus_Couplefamily_Noparentsworking;Economicactivity_Economicallyactive_Inemployment_Employee(includingfull-timestudents);measures_Value	LA
496	411	Familystatus_Couplefamily_Noparentsworking;Economicactivity_Economicallyactive_Inemployment_Self-employed(includingfull-timestudents);measures_Value	LA
497	412	Familystatus_Couplefamily_Noparentsworking;Economicactivity_Economicallyactive_Unemployed(includingfull-timestudents);measures_Value	LA
498	413	Familystatus_Couplefamily_Noparentsworking;Economicactivity_Economicallyinactive_Total;measures_Value	LA
499	414	Familystatus_Couplefamily_Noparentsworking;Economicactivity_Economicallyinactive_Retired;measures_Value	LA
500	415	Familystatus_Couplefamily_Noparentsworking;Economicactivity_Economicallyinactive_Student(includingfull-timestudents);measures_Value	LA
501	416	Familystatus_Couplefamily_Noparentsworking;Economicactivity_Economicallyinactive_Lookingafterhomeorfamily;measures_Value	LA
502	417	Familystatus_Couplefamily_Noparentsworking;Economicactivity_Economicallyinactive_Long-termsickordisabled;measures_Value	LA
503	418	Familystatus_Couplefamily_Noparentsworking;Economicactivity_Economicallyinactive_Other;measures_Value	LA
504	419	Familystatus_Loneparentfamily_Total;Economicactivity_Allcategories_Economicactivity;measures_Value	LA
505	420	Familystatus_Loneparentfamily_Total;Economicactivity_Economicallyactive_Total;measures_Value	LA
506	421	Familystatus_Loneparentfamily_Total;Economicactivity_Economicallyactive_Inemployment_Total;measures_Value	LA
507	422	Familystatus_Loneparentfamily_Total;Economicactivity_Economicallyactive_Inemployment_Employee(includingfull-timestudents);measures_Value	LA
508	423	Familystatus_Loneparentfamily_Total;Economicactivity_Economicallyactive_Inemployment_Self-employed(includingfull-timestudents);measures_Value	LA
509	424	Familystatus_Loneparentfamily_Total;Economicactivity_Economicallyactive_Unemployed(includingfull-timestudents);measures_Value	LA
510	425	Familystatus_Loneparentfamily_Total;Economicactivity_Economicallyinactive_Total;measures_Value	LA
511	426	Familystatus_Loneparentfamily_Total;Economicactivity_Economicallyinactive_Retired;measures_Value	LA
512	427	Familystatus_Loneparentfamily_Total;Economicactivity_Economicallyinactive_Student(includingfull-timestudents);measures_Value	LA
513	428	Familystatus_Loneparentfamily_Total;Economicactivity_Economicallyinactive_Lookingafterhomeorfamily;measures_Value	LA
514	429	Familystatus_Loneparentfamily_Total;Economicactivity_Economicallyinactive_Long-termsickordisabled;measures_Value	LA
515	430	Familystatus_Loneparentfamily_Total;Economicactivity_Economicallyinactive_Other;measures_Value	LA
516	431	Familystatus_Loneparentfamily_Parentworking;Economicactivity_Allcategories_Economicactivity;measures_Value	LA
517	432	Familystatus_Loneparentfamily_Parentworking;Economicactivity_Economicallyactive_Total;measures_Value	LA
518	433	Familystatus_Loneparentfamily_Parentworking;Economicactivity_Economicallyactive_Inemployment_Total;measures_Value	LA
519	434	Familystatus_Loneparentfamily_Parentworking;Economicactivity_Economicallyactive_Inemployment_Employee(includingfull-timestudents);measures_Value	LA
520	435	Familystatus_Loneparentfamily_Parentworking;Economicactivity_Economicallyactive_Inemployment_Self-employed(includingfull-timestudents);measures_Value	LA
521	436	Familystatus_Loneparentfamily_Parentworking;Economicactivity_Economicallyactive_Unemployed(includingfull-timestudents);measures_Value	LA
522	437	Familystatus_Loneparentfamily_Parentworking;Economicactivity_Economicallyinactive_Total;measures_Value	LA
523	438	Familystatus_Loneparentfamily_Parentworking;Economicactivity_Economicallyinactive_Retired;measures_Value	LA
524	439	Familystatus_Loneparentfamily_Parentworking;Economicactivity_Economicallyinactive_Student(includingfull-timestudents);measures_Value	LA
525	440	Familystatus_Loneparentfamily_Parentworking;Economicactivity_Economicallyinactive_Lookingafterhomeorfamily;measures_Value	LA
526	441	Familystatus_Loneparentfamily_Parentworking;Economicactivity_Economicallyinactive_Long-termsickordisabled;measures_Value	LA

527	442	Familystatus_Loneparentfamily_Parentworking;Economicactivity_Economicallyinactive_Other;measures_Value	LA
528	443	Familystatus_Loneparentfamily_Parentnotworking;Economicactivity_Allcategories_Economicactivity;measures_Value	LA
529	444	Familystatus_Loneparentfamily_Parentnotworking;Economicactivity_Economicallyactive_Total;measures_Value	LA
530	445	Familystatus_Loneparentfamily_Parentnotworking;Economicactivity_Economicallyactive_Inemployment_Total;measures_Value	LA
531	446	Familystatus_Loneparentfamily_Parentnotworking;Economicactivity_Economicallyactive_Inemployment_Employee(includingfull-timestudents);measures_Value	LA
532	447	Familystatus_Loneparentfamily_Parentnotworking;Economicactivity_Economicallyactive_Inemployment_Self-employed(includingfull-timestudents);measures_Value	LA
533	448	Familystatus_Loneparentfamily_Parentnotworking;Economicactivity_Economicallyactive_Unemployed(includingfull-timestudents);measures_Value	LA
534	449	Familystatus_Loneparentfamily_Parentnotworking;Economicactivity_Economicallyinactive_Total;measures_Value	LA
535	450	Familystatus_Loneparentfamily_Parentnotworking;Economicactivity_Economicallyinactive_Retired;measures_Value	LA
536	451	Familystatus_Loneparentfamily_Parentnotworking;Economicactivity_Economicallyinactive_Student(includingfull-timestudents);measures_Value	LA
537	452	Familystatus_Loneparentfamily_Parentnotworking;Economicactivity_Economicallyinactive_Lookingafterhomeorfamily;measures_Value	LA
538	453	Familystatus_Loneparentfamily_Parentnotworking;Economicactivity_Economicallyinactive_Long-termsickordisabled;measures_Value	LA
539	454	Familystatus_Loneparentfamily_Parentnotworking;Economicactivity_Economicallyinactive_Other;measures_Value	LA
540	455	disability/health/care_Allcategories_Long-termhealthproblemordisability;measures_Value	LA
541	456	disability/health/care_Allcategories_Long-termhealthproblemordisability;measures_Percent	LA
542	457	disability/health/care_Day-to-dayactivitieslimitedalot;measures_Value	LA
543	458	disability/health/care_Day-to-dayactivitieslimitedalot;measures_Percent	LA
544	459	disability/health/care_Day-to-dayactivitieslimitedalittle;measures_Value	LA
545	460	disability/health/care_Day-to-dayactivitieslimitedalittle;measures_Percent	LA
546	461	disability/health/care_Day-to-dayactivitiesnotlimited;measures_Value	LA
547	462	disability/health/care_Day-to-dayactivitiesnotlimited;measures_Percent	LA
548	463	disability/health/care_Day-to-dayactivitieslimitedalot_Age16to64;measures_Value	LA
549	464	disability/health/care_Day-to-dayactivitieslimitedalot_Age16to64;measures_Percent	LA
550	465	disability/health/care_Day-to-dayactivitieslimitedalittle_Age16to64;measures_Value	LA
551	466	disability/health/care_Day-to-dayactivitieslimitedalittle_Age16to64;measures_Percent	LA
552	467	disability/health/care_Day-to-dayactivitiesnotlimited_Age16to64;measures_Value	LA
553	468	disability/health/care_Day-to-dayactivitiesnotlimited_Age16to64;measures_Percent	LA
554	469	disability/health/care_Verygoodhealth;measures_Value	LA
555	470	disability/health/care_Verygoodhealth;measures_Percent	LA
556	471	disability/health/care_Goodhealth;measures_Value	LA
557	472	disability/health/care_Goodhealth;measures_Percent	LA
558	473	disability/health/care_Fairhealth;measures_Value	LA
559	474	disability/health/care_Fairhealth;measures_Percent	LA
560	475	disability/health/care_Badhealth;measures_Value	LA
561	476	disability/health/care_Badhealth;measures_Percent	LA
562	477	disability/health/care_Verybadhealth;measures_Value	LA
563	478	disability/health/care_Verybadhealth;measures_Percent	LA
564	479	disability/health/care_Providesnounpaidcare;measures_Value	LA

565	480	disability/health/care_Providesnounpaidcare;measures_Percent	LA
566	481	disability/health/care_Provides1to19hoursunpaidcareaweek;measures_Value	LA
567	482	disability/health/care_Provides1to19hoursunpaidcareaweek;measures_Percent	LA
568	483	disability/health/care_Provides20to49hoursunpaidcareaweek;measures_Value	LA
569	484	disability/health/care_Provides20to49hoursunpaidcareaweek;measures_Percent	LA
570	485	disability/health/care_Provides50ormorehoursunpaidcareaweek;measures_Value	LA
571	486	disability/health/care_Provides50ormorehoursunpaidcareaweek;measures_Percent	LA
572	487	HouseholdComposition_Allcategories_Householdcomposition;EthnicGroupofHRP_White_Total;measures_Value	LA
573	488	HouseholdComposition_Allcategories_Householdcomposition;EthnicGroupofHRP_White_English/Welsh/Scottish/NorthernIrish/British;measures_Value	LA
574	489	HouseholdComposition_Allcategories_Householdcomposition;EthnicGroupofHRP_White_Irish;measures_Value	LA
575	490	HouseholdComposition_Allcategories_Householdcomposition;EthnicGroupofHRP_White_OtherWhite;measures_Value	LA
576	491	HouseholdComposition_Allcategories_Householdcomposition;EthnicGroupofHRP_Mixed/multipleethnicgroup;measures_Value	LA
577	492	HouseholdComposition_Allcategories_Householdcomposition;EthnicGroupofHRP_Asian/AsianBritish;measures_Value	LA
578	493	HouseholdComposition_Allcategories_Householdcomposition;EthnicGroupofHRP_Black/African/Caribbean/BlackBritish;measures_Value	LA
579	494	HouseholdComposition_Allcategories_Householdcomposition;EthnicGroupofHRP_Otherethnicgroup;measures_Value	LA
580	495	HouseholdComposition_Onepersonhousehold;EthnicGroupofHRP_Allcategories_EthnicgroupofHRP;measures_Value	LA
581	496	HouseholdComposition_Onepersonhousehold;EthnicGroupofHRP_White_Total;measures_Value	LA
582	497	HouseholdComposition_Onepersonhousehold;EthnicGroupofHRP_White_English/Welsh/Scottish/NorthernIrish/British;measures_Value	LA
583	498	HouseholdComposition_Onepersonhousehold;EthnicGroupofHRP_White_Irish;measures_Value	LA
584	499	HouseholdComposition_Onepersonhousehold;EthnicGroupofHRP_White_OtherWhite;measures_Value	LA
585	500	HouseholdComposition_Onepersonhousehold;EthnicGroupofHRP_Mixed/multipleethnicgroup;measures_Value	LA
586	501	HouseholdComposition_Onepersonhousehold;EthnicGroupofHRP_Asian/AsianBritish;measures_Value	LA
587	502	HouseholdComposition_Onepersonhousehold;EthnicGroupofHRP_Black/African/Caribbean/BlackBritish;measures_Value	LA
588	503	HouseholdComposition_Onepersonhousehold;EthnicGroupofHRP_Otherethnicgroup;measures_Value	LA
589	504	HouseholdComposition_Onefamilyonly_Total;EthnicGroupofHRP_Allcategories_EthnicgroupofHRP;measures_Value	LA
590	505	HouseholdComposition_Onefamilyonly_Total;EthnicGroupofHRP_White_Total;measures_Value	LA
591	506	HouseholdComposition_Onefamilyonly_Total;EthnicGroupofHRP_White_English/Welsh/Scottish/NorthernIrish/British;measures_Value	LA
592	507	HouseholdComposition_Onefamilyonly_Total;EthnicGroupofHRP_White_Irish;measures_Value	LA
593	508	HouseholdComposition_Onefamilyonly_Total;EthnicGroupofHRP_White_OtherWhite;measures_Value	LA
594	509	HouseholdComposition_Onefamilyonly_Total;EthnicGroupofHRP_Mixed/multipleethnicgroup;measures_Value	LA
595	510	HouseholdComposition_Onefamilyonly_Total;EthnicGroupofHRP_Asian/AsianBritish;measures_Value	LA
596	511	HouseholdComposition_Onefamilyonly_Total;EthnicGroupofHRP_Black/African/Caribbean/BlackBritish;measures_Value	LA
597	512	HouseholdComposition_Onefamilyonly_Total;EthnicGroupofHRP_Otherethnicgroup;measures_Value	LA
598	513	HouseholdComposition_Onefamilyonly_Allaged65andover;EthnicGroupofHRP_Allcategories_EthnicgroupofHRP;measures_Value	LA
599	514	HouseholdComposition_Onefamilyonly_Allaged65andover;EthnicGroupofHRP_White_Total;measures_Value	LA
600	515	HouseholdComposition_Onefamilyonly_Allaged65andover;EthnicGroupofHRP_White_English/Welsh/Scottish/NorthernIrish/British;measures_Value	LA
601	516	HouseholdComposition_Onefamilyonly_Allaged65andover;EthnicGroupofHRP_White_Irish;measures_Value	LA
602	517	HouseholdComposition_Onefamilyonly_Allaged65andover;EthnicGroupofHRP_White_OtherWhite;measures_Value	LA

603	518	HouseholdComposition_Onefamilyonly_Allaged65andover;EthnicGroupofHRP_Mixed/multipleethnicgroup;measures_Value	LA
604	519	HouseholdComposition_Onefamilyonly_Allaged65andover;EthnicGroupofHRP_Asian/AsianBritish;measures_Value	LA
605	520	HouseholdComposition_Onefamilyonly_Allaged65andover;EthnicGroupofHRP_Black/African/Caribbean/BlackBritish;measures_Value	LA
606	521	HouseholdComposition_Onefamilyonly_Allaged65andover;EthnicGroupofHRP_Otherethnicgroup;measures_Value	LA
607	522	HouseholdComposition_Onefamilyonly_Married,same-sexcivilpartnershiporcohabitingcouple;EthnicGroupofHRP_Allcategories_EthnicgroupofHRP;measures_Value	LA
608	523	HouseholdComposition_Onefamilyonly_Married,same-sexcivilpartnershiporcohabitingcouple;EthnicGroupofHRP_White_Total;measures_Value	LA
609	524	HouseholdComposition_Onefamilyonly_Married,same-sexcivilpartnershiporcohabitingcouple;EthnicGroupofHRP_White_English/Welsh/Scottish/NorthernIrish/British;measures_Value	LA
610	525	HouseholdComposition_Onefamilyonly_Married,same-sexcivilpartnershiporcohabitingcouple;EthnicGroupofHRP_White_Irish;measures_Value	LA
611	526	HouseholdComposition_Onefamilyonly_Married,same-sexcivilpartnershiporcohabitingcouple;EthnicGroupofHRP_White_OtherWhite;measures_Value	LA
612	527	HouseholdComposition_Onefamilyonly_Married,same-sexcivilpartnershiporcohabitingcouple;EthnicGroupofHRP_Mixed/multipleethnicgroup;measures_Value	LA
613	528	HouseholdComposition_Onefamilyonly_Married,same-sexcivilpartnershiporcohabitingcouple;EthnicGroupofHRP_Asian/AsianBritish;measures_Value	LA
614	529	HouseholdComposition_Onefamilyonly_Married,same-sexcivilpartnershiporcohabitingcouple;EthnicGroupofHRP_Black/African/Caribbean/BlackBritish;measures_Value	LA
615	530	HouseholdComposition_Onefamilyonly_Married,same-sexcivilpartnershiporcohabitingcouple;EthnicGroupofHRP_Otherethnicgroup;measures_Value	LA
616	531	HouseholdComposition_Onefamilyonly_Loneparent;EthnicGroupofHRP_Allcategories_EthnicgroupofHRP;measures_Value	LA
617	532	HouseholdComposition_Onefamilyonly_Loneparent;EthnicGroupofHRP_White_Total;measures_Value	LA
618	533	HouseholdComposition_Onefamilyonly_Loneparent;EthnicGroupofHRP_White_English/Welsh/Scottish/NorthernIrish/British;measures_Value	LA
619	534	HouseholdComposition_Onefamilyonly_Loneparent;EthnicGroupofHRP_White_Irish;measures_Value	LA
620	535	HouseholdComposition_Onefamilyonly_Loneparent;EthnicGroupofHRP_White_OtherWhite;measures_Value	LA
621	536	HouseholdComposition_Onefamilyonly_Loneparent;EthnicGroupofHRP_Mixed/multipleethnicgroup;measures_Value	LA
622	537	HouseholdComposition_Onefamilyonly_Loneparent;EthnicGroupofHRP_Asian/AsianBritish;measures_Value	LA
623	538	HouseholdComposition_Onefamilyonly_Loneparent;EthnicGroupofHRP_Black/African/Caribbean/BlackBritish;measures_Value	LA
624	539	HouseholdComposition_Onefamilyonly_Loneparent;EthnicGroupofHRP_Otherethnicgroup;measures_Value	LA
625	540	HouseholdComposition_Otherhouseholdtypes;EthnicGroupofHRP_Allcategories_EthnicgroupofHRP;measures_Value	LA
626	541	HouseholdComposition_Otherhouseholdtypes;EthnicGroupofHRP_White_Total;measures_Value	LA
627	542	HouseholdComposition_Otherhouseholdtypes;EthnicGroupofHRP_White_English/Welsh/Scottish/NorthernIrish/British;measures_Value	LA
628	543	HouseholdComposition_Otherhouseholdtypes;EthnicGroupofHRP_White_Irish;measures_Value	LA
629	544	HouseholdComposition_Otherhouseholdtypes;EthnicGroupofHRP_White_OtherWhite;measures_Value	LA
630	545	HouseholdComposition_Otherhouseholdtypes;EthnicGroupofHRP_Mixed/multipleethnicgroup;measures_Value	LA
631	546	HouseholdComposition_Otherhouseholdtypes;EthnicGroupofHRP_Asian/AsianBritish;measures_Value	LA
632	547	HouseholdComposition_Otherhouseholdtypes;EthnicGroupofHRP_Black/African/Caribbean/BlackBritish;measures_Value	LA
633	548	HouseholdComposition_Otherhouseholdtypes;EthnicGroupofHRP_Otherethnicgroup;measures_Value	LA
634	549	Age_Allcategories_Age;livingarrangements_Livinginacouple_Total;measures_Value	LA
635	550	Age_Allcategories_Age;livingarrangements_Livinginacouple_Marriedorinaregisteredsame-sexcivilpartnership;measures_Value	LA
636	551	Age_Allcategories_Age;livingarrangements_Livinginacouple_Cohabiting;measures_Value	LA
637	552	Age_Allcategories_Age;livingarrangements_Notlivinginacouple_Total;measures_Value	LA
638	553	Age_Allcategories_Age;livingarrangements_Notlivinginacouple_Single(nevermarriedorneverregisteredasame-sexcivilpartnership);measures_Value	LA
639	554	Age_Allcategories_Age;livingarrangements_Notlivinginacouple_Marriedorinaregisteredsame-sexcivilpartnership;measures_Value	LA
640	555	Age_Allcategories_Age;livingarrangements_Notlivinginacouple_Separated(butstilllegallymarriedorstilllegallyinasame-sexcivilpartnership);measures_Value	LA

641	556	Age_Allcategories_Age;livingarrangements_Notlivinginacouple_Divorcedorformerlyinasame-sexcivilpartnershipwhichisnowlegallydissolved;measures_Value	LA
642	557	Age_Allcategories_Age;livingarrangements_Notlivinginacouple_Widowedorsurvivingpartnerfromasame-sexcivilpartnership;measures_Value	LA
643	558	Age_Age24andunder;livingarrangements_Allcategories_Livingarrangements;measures_Value	LA
644	559	Age_Age24andunder;livingarrangements_Livinginacouple_Total;measures_Value	LA
645	560	Age_Age24andunder;livingarrangements_Livinginacouple_Marriedorinaregisteredsame-sexcivilpartnership;measures_Value	LA
646	561	Age_Age24andunder;livingarrangements_Livinginacouple_Cohabiting;measures_Value	LA
647	562	Age_Age24andunder;livingarrangements_Notlivinginacouple_Total;measures_Value	LA
648	563	Age_Age24andunder;livingarrangements_Notlivinginacouple_Single(nevermarriedorneverregisteredasame-sexcivilpartnership);measures_Value	LA
649	564	Age_Age24andunder;livingarrangements_Notlivinginacouple_Marriedorinaregisteredsame-sexcivilpartnership;measures_Value	LA
650	565	Age_Age24andunder;livingarrangements_Notlivinginacouple_Separated(butstilllegallymarriedorstilllegallyinasame-sexcivilpartnership);measures_Value	LA
651	566	Age_Age24andunder;livingarrangements_Notlivinginacouple_Divorcedorformerlyinasame-sexcivilpartnershipwhichisnowlegallydissolved;measures_Value	LA
652	567	Age_Age24andunder;livingarrangements_Notlivinginacouple_Widowedorsurvivingpartnerfromasame-sexcivilpartnership;measures_Value	LA
653	568	Age_Age25to34;livingarrangements_Allcategories_Livingarrangements;measures_Value	LA
654	569	Age_Age25to34;livingarrangements_Livinginacouple_Total;measures_Value	LA
655	570	Age_Age25to34;livingarrangements_Livinginacouple_Marriedorinaregisteredsame-sexcivilpartnership;measures_Value	LA
656	571	Age_Age25to34;livingarrangements_Livinginacouple_Cohabiting;measures_Value	LA
657	572	Age_Age25to34;livingarrangements_Notlivinginacouple_Total;measures_Value	LA
658	573	Age_Age25to34;livingarrangements_Notlivinginacouple_Single(nevermarriedorneverregisteredasame-sexcivilpartnership);measures_Value	LA
659	574	Age_Age25to34;livingarrangements_Notlivinginacouple_Marriedorinaregisteredsame-sexcivilpartnership;measures_Value	LA
660	575	Age_Age25to34;livingarrangements_Notlivinginacouple_Separated(butstilllegallymarriedorstilllegallyinasame-sexcivilpartnership);measures_Value	LA
661	576	Age_Age25to34;livingarrangements_Notlivinginacouple_Divorcedorformerlyinasame-sexcivilpartnershipwhichisnowlegallydissolved;measures_Value	LA
662	577	Age_Age25to34;livingarrangements_Notlivinginacouple_Widowedorsurvivingpartnerfromasame-sexcivilpartnership;measures_Value	LA
663	578	Age_Age35to49;livingarrangements_Allcategories_Livingarrangements;measures_Value	LA
664	579	Age_Age35to49;livingarrangements_Livinginacouple_Total;measures_Value	LA
665	580	Age_Age35to49;livingarrangements_Livinginacouple_Marriedorinaregisteredsame-sexcivilpartnership;measures_Value	LA
666	581	Age_Age35to49;livingarrangements_Livinginacouple_Cohabiting;measures_Value	LA
667	582	Age_Age35to49;livingarrangements_Notlivinginacouple_Total;measures_Value	LA
668	583	Age_Age35to49;livingarrangements_Notlivinginacouple_Single(nevermarriedorneverregisteredasame-sexcivilpartnership);measures_Value	LA
669	584	Age_Age35to49;livingarrangements_Notlivinginacouple_Marriedorinaregisteredsame-sexcivilpartnership;measures_Value	LA
670	585	Age_Age35to49;livingarrangements_Notlivinginacouple_Separated(butstilllegallymarriedorstilllegallyinasame-sexcivilpartnership);measures_Value	LA
671	586	Age_Age35to49;livingarrangements_Notlivinginacouple_Divorcedorformerlyinasame-sexcivilpartnershipwhichisnowlegallydissolved;measures_Value	LA
672	587	Age_Age35to49;livingarrangements_Notlivinginacouple_Widowedorsurvivingpartnerfromasame-sexcivilpartnership;measures_Value	LA
673	588	Age_Age50to64;livingarrangements_Allcategories_Livingarrangements;measures_Value	LA
674	589	Age_Age50to64;livingarrangements_Livinginacouple_Total;measures_Value	LA
675	590	Age_Age50to64;livingarrangements_Livinginacouple_Marriedorinaregisteredsame-sexcivilpartnership;measures_Value	LA
676	591	Age_Age50to64;livingarrangements_Livinginacouple_Cohabiting;measures_Value	LA
677	592	Age_Age50to64;livingarrangements_Notlivinginacouple_Total;measures_Value	LA
678	593	Age_Age50to64;livingarrangements_Notlivinginacouple_Single(nevermarriedorneverregisteredasame-sexcivilpartnership);measures_Value	LA

679	594	Age_Age50to64;livingarrangements_Notlivinginacouple_Marriedorinaregisteredsame-sexcivilpartnership;measures_Value	LA
680	595	Age_Age50to64;livingarrangements_Notlivinginacouple_Separated(butstilllegallymarriedorstilllegallyinasame-sexcivilpartnership);measures_Value	LA
681	596	Age_Age50to64;livingarrangements_Notlivinginacouple_Divorcedorformerlyinasame-sexcivilpartnershipwhichisnowlegallydissolved;measures_Value	LA
682	597	Age_Age50to64;livingarrangements_Notlivinginacouple_Widowedorsurvivingpartnerfromasame-sexcivilpartnership;measures_Value	LA
683	598	Age_Age65andover;livingarrangements_Allcategories_Livingarrangements;measures_Value	LA
684	599	Age_Age65andover;livingarrangements_Livinginacouple_Total;measures_Value	LA
685	600	Age_Age65andover;livingarrangements_Livinginacouple_Marriedorinaregisteredsame-sexcivilpartnership;measures_Value	LA
686	601	Age_Age65andover;livingarrangements_Livinginacouple_Cohabiting;measures_Value	LA
687	602	Age_Age65andover;livingarrangements_Notlivinginacouple_Total;measures_Value	LA
688	603	Age_Age65andover;livingarrangements_Notlivinginacouple_Single(nevermarriedorneverregisteredasame-sexcivilpartnership);measures_Value	LA
689	604	Age_Age65andover;livingarrangements_Notlivinginacouple_Marriedorinaregisteredsame-sexcivilpartnership;measures_Value	LA
690	605	Age_Age65andover;livingarrangements_Notlivinginacouple_Separated(butstilllegallymarriedorstilllegallyinasame-sexcivilpartnership);measures_Value	LA
691	606	Age_Age65andover;livingarrangements_Notlivinginacouple_Divorcedorformerlyinasame-sexcivilpartnershipwhichisnowlegallydissolved;measures_Value	LA
692	607	Age_Age65andover;livingarrangements_Notlivinginacouple_Widowedorsurvivingpartnerfromasame-sexcivilpartnership;measures_Value	LA
693	608	RuralUrban	LA
694	609	Qualifications_Allcategories_Highestlevelofqualification;measures_Value	LA
695	610	Qualifications_Allcategories_Highestlevelofqualification;measures_Percent	LA
696	611	Qualifications_Noqualifications;measures_Value	LA
697	612	Qualifications_Noqualifications;measures_Percent	LA
698	613	Qualifications_Highestlevelofqualification_Level1qualifications;measures_Value	LA
699	614	Qualifications_Highestlevelofqualification_Level1qualifications;measures_Percent	LA
700	615	Qualifications_Highestlevelofqualification_Level2qualifications;measures_Value	LA
701	616	Qualifications_Highestlevelofqualification_Level2qualifications;measures_Percent	LA
702	617	Qualifications_Highestlevelofqualification_Apprenticeship;measures_Value	LA
703	618	Qualifications_Highestlevelofqualification_Apprenticeship;measures_Percent	LA
704	619	Qualifications_Highestlevelofqualification_Level3qualifications;measures_Value	LA
705	620	Qualifications_Highestlevelofqualification_Level3qualifications;measures_Percent	LA
706	621	Qualifications_Highestlevelofqualification_Level4qualificationsandabove;measures_Value	LA
707	622	Qualifications_Highestlevelofqualification_Level4qualificationsandabove;measures_Percent	LA
708	623	Qualifications_Highestlevelofqualification_Otherqualifications;measures_Value	LA
709	624	Qualifications_Highestlevelofqualification_Otherqualifications;measures_Percent	LA
710	625	Qualifications_Schoolchildrenandfull-timestudents_Age16to17;measures_Value	LA
711	626	Qualifications_Schoolchildrenandfull-timestudents_Age16to17;measures_Percent	LA
712	627	Qualifications_Schoolchildrenandfull-timestudents_Age18andover;measures_Value	LA
713	628	Qualifications_Schoolchildrenandfull-timestudents_Age18andover;measures_Percent	LA
714	629	Qualifications_Full-timestudents_Age18to74_Economicallyactive_Inemployment;measures_Value	LA
715	630	Qualifications_Full-timestudents_Age18to74_Economicallyactive_Inemployment;measures_Percent	LA
716	631	Qualifications_Full-timestudents_Age18to74_Economicallyactive_Unemployed;measures_Value	LA

717	632	Qualifications_Full-timestudents_Age18to74_Economicallyactive_Unemployed;measures_Percent	LA
718	633	Qualifications_Full-timestudents_Age18to74_Economicallyinactive;measures_Value	LA
719	634	Qualifications_Full-timestudents_Age18to74_Economicallyinactive;measures_Percent	LA
720	635	Age_Allcategories_Age;ResidenceType_Livesinahousehold;measures_Value	LA
721	636	Age_Allcategories_Age;ResidenceType_Livesinacommunalestablishment;measures_Value	LA
722	637	Age_Age0to15;ResidenceType_Allcategories_Residencetype;measures_Value	LA
723	638	Age_Age0to15;ResidenceType_Livesinahousehold;measures_Value	LA
724	639	Age_Age0to15;ResidenceType_Livesinacommunalestablishment;measures_Value	LA
725	640	Age_Age16to24;ResidenceType_Allcategories_Residencetype;measures_Value	LA
726	641	Age_Age16to24;ResidenceType_Livesinahousehold;measures_Value	LA
727	642	Age_Age16to24;ResidenceType_Livesinacommunalestablishment;measures_Value	LA
728	643	Age_Age25to34;ResidenceType_Allcategories_Residencetype;measures_Value	LA
729	644	Age_Age25to34;ResidenceType_Livesinahousehold;measures_Value	LA
730	645	Age_Age25to34;ResidenceType_Livesinacommunalestablishment;measures_Value	LA
731	646	Age_Age35to49;ResidenceType_Allcategories_Residencetype;measures_Value	LA
732	647	Age_Age35to49;ResidenceType_Livesinahousehold;measures_Value	LA
733	648	Age_Age35to49;ResidenceType_Livesinacommunalestablishment;measures_Value	LA
734	649	Age_Age50to64;ResidenceType_Allcategories_Residencetype;measures_Value	LA
735	650	Age_Age50to64;ResidenceType_Livesinahousehold;measures_Value	LA
736	651	Age_Age50to64;ResidenceType_Livesinacommunalestablishment;measures_Value	LA
737	652	Age_Age65andover;ResidenceType_Allcategories_Residencetype;measures_Value	LA
738	653	Age_Age65andover;ResidenceType_Livesinahousehold;measures_Value	LA
739	654	Age_Age65andover;ResidenceType_Livesinacommunalestablishment;measures_Value	LA
		Deprivation	
740	1	IMDSCORE	LSOA
741	2	RANKOFIMDSCORE(where1ismostdeprived)	LSOA
742	3	IMDDecile_1isMostDeprived	LSOA
		Place based longitudinal data	
743	1	Employment and Support Allowance claimants	LSOA
744	2	Distance to nearest Gambling Outlet (minutes)	LSOA
745	3	Distance to nearest Fast Food Outlet (minutes)	LSOA
746	4	Distance to nearest Pubs/Bars/Nightclub (minutes)	LSOA
747	5	Distance to nearest Off licence (minutes)	LSOA
748	6	Distance to nearest Tobacconists/Vape Store (minutes)	LSOA
749	7	Distance to nearest GP Practice (minutes)	LSOA
750	8	Distance to nearest Hospital (minutes)	LSOA
751	9	Distance to nearest Dentist (minutes)	LSOA
752	10	Distance to nearest Pharmacy (minutes)	LSOA

753	11	Distance to nearest Leisure Centre (minutes)	LSOA
754	12	Distance to nearest Blue space (minutes)	LSOA
755	13	NVDI value indicating Passive Green Space	LSOA
756	14	Annual mean Nitrogen Dioxide (µgm³)	LSOA
757	15	Annual mean Particulate Matter (µgm³)	LSOA
758	16	Annual mean Sulpur Dioxide (µgm³)	LSOA
759	17	Health Domain Score	LSOA
760	18	Green/Bluespace Domain Score	LSOA
761	19	Air quality Domain Score	LSOA
762	20	Retail Domain Score	LSOA
763	21	Access to Healthy Assets and Hazards index score	LSOA
764	22	AHAH Index ranked	LSOA
765	23	AHAH Index percentiles	LSOA
766	24	SmallAreaMentalHealthIndex_index.2011	LSOA
767	25	SmallAreaMentalHealthIndex_decile2011	LSOA
768	26	SmallAreaMentalHealthIndex_index.2012	LSOA
769	27	SmallAreaMentalHealthIndex_decile2012	LSOA
770	28	SmallAreaMentalHealthIndex_index.2013	LSOA
771	29	SmallAreaMentalHealthIndex_decile2013	LSOA
772	30	SmallAreaMentalHealthIndex_index.2014	LSOA
773	31	SmallAreaMentalHealthIndex_decile2014	LSOA
774	32	SmallAreaMentalHealthIndex_index.2015	LSOA
775	33	SmallAreaMentalHealthIndex_decile2015	LSOA
776	34	SmallAreaMentalHealthIndex_index.2016	LSOA
777	35	SmallAreaMentalHealthIndex_decile2016	LSOA
778	36	SmallAreaMentalHealthIndex_index.2017	LSOA
779	37	SmallAreaMentalHealthIndex_decile2017	LSOA
780	38	SmallAreaMentalHealthIndex_index.2018	LSOA
781	39	SmallAreaMentalHealthIndex_decile2018	LSOA
782	40	SmallAreaMentalHealthIndex_index.2019	LSOA
783	41	SmallAreaMentalHealthIndex_decile2019	LSOA

Supplementary Information S5: Variable linkage and selection

Variable selection considered all the 783 variables listed in Supplementary Table S3. The variables acquired by linking primary and secondary data are:

- cancer diagnosis
- time of diagnosis
- post-code sector
- age group
- gender;
- ethnicity (African, any other Asian background, any other black background, any other ethnic group, any other mixed background, any other white background, Asian and Chinese, British, British Asian, Caribbean, Chinese, Chinese and white, English, Filipino, Indian, Irish, Italian, Northern-Irish, not known, other mixed white, other white or white unspecified, other white European, Pakistani, Polish, Scottish, Welsh, white and Asian, white and black African, white and black Caribbean);
- frailty score (fit, mild, moderate and severe);
- smoking information (never smoked, ex smoker, current smoker, unknown);
- co-morbidities (chronic kidney disease and stage, chronic obstructive pulmonary disease, congestive heart failure, coronary heart disease, depression, anxiety, hypertension, diabetes type and COVID19).

These primary and secondary data variables were recorded at the same time of the cancer diagnosis and aggregated every six months during the period 2017-2022. In addition, geographic-level conditions were extracted for each local authority without further manipulation from the following datasets:

- police crime reports (from https://data.police.uk/), for the same period of the cancer incidences (2017-2022);
- household data was obtained from the Office of National Statistics,

Census of 2011 (https://www.nomisweb.co.uk/sources/census_2011);

- the index of multiple deprivation from the Ministry for Housing, Communities, and Local Government, and relative to 2019 (https://www.gov.uk/government/statistics/english-indices-of-deprivation-2019);
- place-based longitudinal data resource which includes health, social and behavioral variables (https://pldr.org/) relative to the 2020 update. Network distances in minutes to the nearest healthy asset or hazard as provided by PBLDR are calculated from the open source software Routino and by considering the quickest route through the transport network (1).

The Index of Multiple Deprivation (IMD) is the official measure of relative deprivation for LSOAs in England. Every LSOA in England is ranked from 1 (most deprived or poor) to 5 (quintile) or 10 (decile) (least deprived or most affluent), but there is no definitive cut-off below which an area is considered 'deprived' (2).

The linkage between primary (the GPs of the Morecambe Bay exCCG area) and secondary data (obtained from the University Hospitals of Morecambe Bay NHS Foundation Trust, including cancer diagnosis) data was performed by the data provider (University Hospitals of Morecambe Bay Trust). Linkage between linked primary and secondary data, and data from publicly available datasets (crime, census, IMD, PBLDR) was done by geographically merging the linked primary and secondary data at post-code sector level with the public dataset available at post-code, Local Authority or Lower Super Output Area levels. This is a common operation in geographic health analyses (3-5). Population estimates were required for calculating cancer risk and rates. These were obtained at LSOA level from the 2011 Census for England and Wales.

A stepwise selection method was applied to an accelerated failure model with lognormal distribution. The accelerated failure model allows the inclusion of censored data as the outcome (6), while cancer type and postcode sector were used as clusters for robust variance computation (to account for correlation within each cancer type). For the cancer counts C, the accelerated failure model is:

$$\log C_{i,j} = \beta_0 + \boldsymbol{\beta}_1 \mathbf{X}_{i,j} + u_i + \delta\varepsilon_{i,j}$$

where β_0 is the intercept, $\boldsymbol{\beta}_1$ is a vector of regression coefficients, u is the random effect, δ is scale parameter and, ε is the error distribution assumed to have a normal distribution. The subscript i refers to post-code sector at a given time period, and j to the cancer type.

The accelerated failure model was estimated using the maximum likelihood estimation method. The model's performance was assessed using the Bayesian Information Criterion (BIC) and the best model was chosen as the one with the lowest BIC. The described model is mostly used in parametric mixed survival problems with left truncated data or Tobit regression, where the time to occurrence of an event was substituted here with the number of patients with a cancer type. The use of the proposed method for non-survival data has been described elsewhere (7).

References

1. Green MA, Daras K, Davies A, Barr B, Singleton A. Developing an openly accessible multi-dimensional small area index of 'Access to Healthy Assets and Hazards' for Great Britain, 2016. Health Place. 2018;54:11-9.
2. Rogers SN, Staunton A, Girach R, Langton S, Lowe D. Audit of the two-week pathway for patients with suspected cancer of the head and neck and the influence of socioeconomic status. Br J Oral Maxillofac Surg. 2019;57(5):419-24.
3. McNally RJ, Alston RD, Cairns DP, Eden OB, Birch JM. Geographical and ecological analyses of childhood acute leukaemias and lymphomas in north-west England. Br J Haematol. 2003;123(1):60-5.
4. Manda SO, Feltbower RG, Gilthorpe MS. Investigating spatio-temporal similarities in the epidemiology of childhood leukaemia and diabetes. Eur J Epidemiol. 2009;24(12):743-52.
5. Wheeler BW, Kothencz G, Pollard AS. Geography of non-melanoma skin cancer and ecological associations with environmental risk factors in England. Br J Cancer. 2013;109(1):235-41.
6. Kalbfleisch JD, Prentice RL. The statistical analysis of failure time data: John Wiley & Sons; 2011.
7. Kong S, Nan B. Semiparametric approach to regression with a covariate subject to a detection limit. Biometrika. 2016;103(1):161-74.

Supplementary Information S6: Inference

The proposed model allowed for shared local space dependence, in addition to cancer specific components. Similar frameworks have been proposed elsewhere for cancers and other diseases (1), although none of them are censored cancer-specific. This allowed to investigate spatial inequalities in cancer, especially between urban and rural areas.

The log of the number of cases observed in each postcode sector was the dependent variable, and the individual and ecological variables obtained from the variable selection step were the independent variables. The joint model is spatially explicit (by modelling the spatial autocorrelation common to all the cancer types) and Bayesian, and for these reasons adapted to stabilise risk estimates based on small numbers at postcode sector level (2). The resulting relative risk estimate for each postcode sector is a form of smoothed average of the observed risk and the mean relative risk in the neighbouring postcode sectors conditioned to a set of prior assumptions.

The common shared component is the spatial dependence, which results from common factors interacting with the cancers, but not represented by independent variables. If spatial dependence is disregarded, inferences are likely to be biased (3).

Since the cancer counts are censored, the joint model's maximum likelihood estimation method integrates the censored data into the likelihood function. Therefore, the observed cancers' counts are simultaneously modelled with the censored data.

Within this approach the method first models the complete response values, including both the observed and the unobserved values, and then models the censored values conditioned on the complete response values (4). To facilitate the computation of the spatial dependence structure the data were modelled using a log Gaussian process (4). The main advantage of the likelihood-based approach is that it utilizes all the information available (5).

Formally, let $Y_{j,s}$ represent the observed (including censored) number of cases of cancer type j at postcode sector s. The joint modelling follows the multivariate spatial Bayesian log-Gaussian model for left-censored data proposed by Sahoo and colleagues (6) adapted to Poisson (Po) processes (7) and to account for censoring levels varying among locations. Y follows a Poisson distribution with mean $E_{j,s}\theta_{j,s}$:

$$Y_{j,s}|\theta_{j,s} \sim Po\left(E_{j,s}\theta_{j,s}\right)$$

where E is the expected number of cases and θ is the relative risk modelled using a Poisson log-linear model within a multivariate spatial Bayesian framework:

$$\log\left(\theta_{j,s}\right) = \boldsymbol{X}_s + \mathrm{z}_{j,s} + \varepsilon_{j,s} \text{ with } j=1,2, \ldots, \mathrm{J}$$

where **X** is the (full rank) design matrix containing the covariate values (obtained from the variable selection step described above and common to all cancers), β are unknown regression coefficients, z is a multivariate spatial zero mean Gaussian process, at every location s and for each joint process j with non-singular covariance matrix $\boldsymbol{\Sigma}$:

$$\boldsymbol{\Sigma} = \sigma^2\boldsymbol{\Psi} \; and \; \boldsymbol{\Psi} = \left(\nu^2\mathbf{I} + \mathbf{K}(\phi)\right), \;\; \nu^2 = \frac{\tau^2}{\sigma^2}$$

where $\mathbf{K}$ is the exponential spatial correlation matrix controlling the spatial smoothness of the cancers, ν is the ratio between the spatial variance τ^2 and the total variance σ^2, and **I** is the identity matrix. These parameters are common for all cancer types. Finally, ε is the multivariate nugget effect (pure error term), zero-mean normally distributed with covariance $(1-\nu^2)\boldsymbol{\Sigma}$.

The data of size n consist of m exact observations and n-m (censored) interval observations at locations s^*. Therefore, Y_j^c is left-censored with censoring level u_{j,s^*}, with u_{j,s^*} being one of the following integer intervals: $[\![1,2]\!]$, $[\![1,3]\!]$, $[\![1,4]\!]$ and $[\![1,5]\!]$, depending on the cancer type and location.

The likelihood for censored spatial data of cancer j, in its generic form, is:

$$\mathcal{L}_j(\theta) = \int_{u_j}^{\square} Normal_J\left(y_j; \beta_j\mathbf{X}, \boldsymbol{\Sigma}\right)du_j$$

The set of parameters and hyperparameters are:

$$\Theta = \{\boldsymbol{\beta}, \boldsymbol{\Sigma}, \phi, \upsilon, \varepsilon, \mathbf{Y}^c, \mathbf{Y}^0\}$$

where $\mathbf{Y}^c$ is the set of censored cancer observations and $\mathbf{Y}^0$ the set of cancers to be predicted. Inference on these parameters is based on Markov chain Monte Carlo (MCMC) sampling. The regression coefficients $\boldsymbol{\beta}$ are assumed to have normal priors with zero mean and ten standard deviations; the covariance $\boldsymbol{\Sigma}$ follows an Inverse-Wishart

distribution with scale 0.01 and degrees of freedom 0.01**I**; the spatial range parameter ϕ is uniformely distributed between 0 and 50 km (approximately half of the maximum distance between two postcode-sectors); the ratio of spatial to total variance υ is uniformely distributed between its natural limits of 0 and 1; finally the pure error term ε is normally distributed with mean zero and variance $\upsilon\mathbf{\Sigma}_s \otimes \mathbf{\Sigma}$, with $\mathbf{\Sigma}_s$ been the spatial covariance. Censored values are inputted at every iteration from a Truncated-Normal distribution for each cancer type. In practice, by using conventional equations the truncated-normal distribution probability at each censored value is used to estimate the inputted binomial value (8).

Θ relative posteriors and full MCMC algorithm are described elsewhere (6).

We ran the MCMC for 100,000 iterations and discarded the first 20,000 iterations. The remaining 80,000 iterations were thinned by keeping one of every fifty. Convergence was assessed by a visual examination of trace plots. Results were based on posterior sample sizes sufficient to give Monte Carlo standard errors less than 5% of the posterior standard deviation for the parameters of interest (9)

Uncertainty is represented by the posterior standard deviation of the outcome or parameter of interest, with smaller values indicating more certainty in the inference of the parameter or the prediction of the outcome. Uncertainty enables a better understanding of the information produced and more informed decision making based on this information. For example, large uncertainties may be dependent on the vaguely defined or non-informative assumptions made for the model, the absence of model components, and/or the lack of predictive capacity from the employed risk factors. As stressed by Roberts and colleagues (10), ignoring and/or not understanding uncertainty information can result in misinterpretation of model outputs, substandard decision making, or disregard of important information due to its large uncertainty.

References

1. Manda SO, Feltbower RG, Gilthorpe MS. Investigating spatio-temporal similarities in the epidemiology of childhood leukaemia and diabetes. Eur J Epidemiol. 2009;24(12):743-52.
2. Jarup L, Best N, Toledano MB, Wakefield J, Elliott P. Geographical epidemiology of prostate cancer in Great Britain. Int J Cancer. 2002;97(5):695-9.
3. Nogueira MC, Fayer VA, Correa CSL, Guerra MR, Stavola B, Dos-Santos-Silva I, et al. Inequities in access to mammographic screening in Brazil. Cad Saude Publica. 2019;35(6):e00099817.
4. Qu T, Li B, Sally Chan Mp, Albarracin D. Bias correction for nonignorable missing counts of areal HIV new diagnosis. Stat. 2023;12(1).
5. Leung K-M, Elashoff RM, Afifi AA. Censoring issues in survival analysis. Annual review of public health. 1997;18(1):83-104.
6. Sahoo I, Hazra A. Contamination mapping in Bangladesh using a multivariate spatial Bayesian model for left-censored data. arXiv preprint arXiv:210615730. 2021.
7. Gomez-Rubio V, Palmi-Perales F, Lopez-Abente G, Ramis-Prieto R, Fernandez-Navarro P. Bayesian joint spatio-temporal analysis of multiple diseases. Sort-Stat Oper Res T. 2019;43(1):51-74.

8. Horgan JM. Probability with R: an introduction with computer science applications: John Wiley & Sons; 2019.
9. Best N, Hansell AL. Geographic variations in risk: adjusting for unmeasured confounders through joint modeling of multiple diseases. Epidemiology. 2009;20(3):400-10.
10. Roberts J, Cramb S, Baade P, Mengersen K. Communicating statistical outputs through health maps. 2016.

Supplementary Table S4. Predicted and real total number of diagnosed cancers, and relative rates, in the study area between 2017 and 2022. 95%CRL is the lower limit of the 95% credible interval for the model-predicated cancer counts; 95%CRU is the upper limit of the 95% credible interval for the model-predicated cancer counts. * data from the Office of National Statistics for the year 2017. ** data from Cancer Research UK for the period 2016-2018.

	Cancer counts							Rates x 100,000 per year			
**Cancer type**	**Real**	**Individual model predicted**	**Individual model Error (%)**	**Joint Model predicted**	**Joint model Error (%)**	**95%CRL**	**95%CRU**	**Joint Model**	**Real**	**North West***	**England****
Breast	2076	2537	22.2	2011	-3.1	1389	2181	100.26	103.5	95	83
Colorectal	1606	946	-41.1	1680	4.6	822	2200	83.76	80.07	72	69
Gynaecology	670	335	-50.0	708	5.7	490	994	35.3	33.4	36	32
Haematology	992	429	-56.8	947	-4.5	590	1131	47.21	49.46	48	47
Head and Neck	539	338	-37.3	519	-3.7	391	931	25.88	26.87	28	18
Lung	1535	948	-38.2	1570	2.3	1135	2009	78.28	76.53	92	76
Skin	4599	4413	-4.0	4276	-7.0	3276	4922	213.19	229.29	254	257
Upper GI	1039	420	-59.6	943	-9.2	628	1145	47.02	51.8	61	50
Urology	2450	1882	-23.2	2589	5.7	1999	2714	129.08	122.15	108	98

Supplementary Table S5. Descriptive statistics for model-based cancer counts by cancer type per postcode sector and 6 months period. Incidence refers to 1000 people. SD = standard deviation, SEI = standard error incidence, SEI50 standard error for incidence in 50 years old and under, SEIO50 = standard error for incidence in over 50 years old. Standard errors were estimated using a Poisson approximation method (see methods).

Cancer type	Mean	SD	Incidence	Incidence for 50 and under	Incidence for over 50	SEI	SEI50	SEIO50
Breast	2.79	1.57	1.65	0.57	2.84	1.51	0.93	4.49
Colorectal	2.33	0.8	1.38	0.24	2.65	1.21	0.52	6.13
Gynaecology	0.98	0.47	0.58	0.16	0.82	0.46	0.35	1.34
Haematology	1.31	0.51	0.78	0.15	1.33	0.55	0.41	1.84
Head and Neck	0.95	0.45	0.56	0.14	0.69	0.42	0.35	1.28
Lung	2.18	1.02	1.29	0.11	2.37	1.03	0.41	3.82
Skin	9.55	6.06	5.68	0.81	12.41	5.34	1.28	17.77
Upper GI	1.31	0.46	0.78	0.07	1.43	0.49	0.21	1.94
Urology	3.59	2.07	2.13	0.32	4.53	1.87	0.71	6.17

Supplementary Table S6. Number of cancer types in postcode sectors

**Number of cancer types in each postcode sector**	**Number of postcode sectors**	**Range in the proportion of the population in postcode sectors respect to the overall in the Morecambe Bay ex CCG extended area.**
12	4	0.83% - 2.30%
11	30	0.49% - 3.26%
10	20	0.26% - 2.53%
9	2	0.22% - 0.63%
8	1	1.90%
6	1	2.38%
4	2	1.02% - 1.07%
2	1	1.40%
1	14	0.2% - 2.73%

Supplementary Table S7. Variable selection method: summary statistics for selected candidate variables for joint modelling. SD = standard deviation; min = minimum, max = maximum, 25PER = 25th percentile or first quartile (the value below which 25% of the data falls in a dataset), 75PER = 75th percentile or third quartile (the value below which 75% of the data falls in a dataset).

**Risk Factor (number of people with)**	**Mean**	**SD**	**Min**	**25PER**	**75PER**	**Max**
Age 60 to 80	7.9	5.8	0	4	11	42
Age above 80	4.3	3.7	0	2	5	21
Any other Black background	0.0014	0.037	0	0	0	1
British	8.9	7.3	0	4	12	45
Chronic Kidney Disease	4.7	3.6	0	2	6	24
Congestive Hearth Failure	1.4	1.3	0	0	2	10
Coronary Heart Disease	14	11	0	6	20	54
COVID19	0.73	1.4	0	0	1	8
Depression	6.3	4.4	0	3	8	26
Diabetes	5.6	4.1	0	3	7	25
Ex Smoker	0.84	0.96	0	0	1	6
Female age 25-49 mixed white and Asian (area)	3.9	5.1	0	0	8	20
Frailty (number of fit)	5.9	4.2	0	3	7	24
Hypertension	11	8.1	0	5	15	45
Self employed (including fulltime students) one parent working (area)	30	20	0	15	45	110
Male Pakistani (area)	12	29	0	0	9	220
Northern Irish (area)	0.0014	0.037	0	0	0	1
Other White background (area)	0.0055	0.074	0	0	0	1
Pakistani	0.0028	0.053	0	0	0	1
Scottish (area)	0.022	0.15	0	0	0	1
Smoker	0.89	1	0	0	1	5
White and Black Caribbean	0.0014	0.037	0	0	0	1

Supplementary Information S7: Statistically significant risk factors by cancer type

Joint modelling: statistically significant risk factors for Breast cancer

Risk factor	OR	Low CI	Upp CI
Chronic Kidney Disease	1.14	1.07	1.21
Congestive Heart Failure	1.08	1.04	1.13
COVID19	1.11	1.02	1.20
Depression	1.00	1.00	1.01
Diabetes	1.07	1.02	1.13
Age above 80	1.24	1.04	1.50
Time	1.34	1.09	1.61

Joint modelling: statistically significant risk factors for Colorectal cancer

Risk factor	OR	Low CI	Upp CI
Coronary Hearth Disease	1.04	1.02	1.06
COVID19	1.06	1.01	1.10
Depression	1.10	1.03	1.13
Diabetes	1.31	1.20	1.44
Hypertension	1.06	1.00	1.14
Age 60 to 80	5.14	2.30	11.39
Age above 80	0.73	0.59	0.95
Ex Smoker	1.23	1.10	1.37
Self employed (including fulltime students) one parent working (area)	0.95	0.91	0.99
British	1.52	1.26	1.81
Any other Black background	1.09	1.03	1.16
Other White background (area)	0.89	0.83	0.95
Pakistani	1.11	1.03	1.19
Scottish (area)	1.19	1.11	1.28
White and Black Caribbean (area)	1.18	1.03	1.36
Time	1.08	1.02	1.15

Joint modelling: statistically significant risk factors for Gynaecology cancer

Risk factor	OR	Low CI	Upp CI
Chronic Kidney Disease	1.00	1.00	1.00
Depression	0.91	0.84	1.00
Diabetes	1.04	1.01	1.07
Frailty (number of fit)	0.89	0.83	0.95
Age above 80	1.26	1.17	1.34
Smoker	0.92	0.84	0.99
Other White background (area)	1.01	1.00	1.02

Joint modelling: statistically significant risk factors for Haematology cancer

**Risk factor**	**OR**	**Low CI**	**Upp CI**
Chronic Kidney Disease	1.04	1.01	1.06
Covid19	1.16	1.03	1.31
Smoker	1.12	1.01	1.23
White and Black Caribbean	1.11	1.01	1.22

Joint modelling: statistically significant risk factors for Head and Neck cancer

**Risk factor**	**OR**	**Low CI**	**Upp CI**
Chronic Kidney Disease	0.98	0.96	1.00
COVID19	1.16	1.04	1.30
Smoker	1.12	1.02	1.24
White and Black Caribbean	1.11	1.01	1.21
Northern Irish (area)	0.90	0.82	0.98

Joint modelling: statistically significant risk factors for Lung cancer

**Risk factor**	**Odd ratios (OR)**	**Low 95% Confidence interval**	**Upper 95% Confidence interval**
Congestive Heart Failure	0.35	0.14	0.87
Chronic Kidney Disease	1.28	1.16	1.41
Coronary Heart Disease	1.18	1.11	1.26
Smoker	1.05	1.03	1.07
Self employed (including fulltime students) one parent working (area)	0.92	0.86	0.98
Female age 25-49 mixed white and Asian (area)	11.53	2.81	55.79
Male Pakistani (area)	1.07	1.00	1.14
Northern Irish (area)	1.30	1.02	1.69

Joint modelling: statistically significant risk factors for Skin cancer

Risk factor	OR	Low CI	Upp CI
Ex Smoker	0.69	0.55	0.87
Any other Black background	8.23	3.44	18.54

Joint modelling: statistically significant risk factors for Upper GI cancer

**Risk factor**	**OR**	**Low CI**	**Upp CI**
Chronic Kidney Disease	1.00	1.00	1.00
Covid19	0.98	0.97	0.99
Depression	0.90	0.83	0.99
Frailty (number of fit)	0.89	0.83	0.96
Smoker	0.91	0.84	0.99
Northern Irish (area)	0.97	0.95	1.00

Joint modelling: statistically significant risk factors for Urology cancer

Risk factor	OR	Low CI	Upp CI
COVID19	0.95	0.90	1.00
Diabetes	0.69	0.67	0.80
Age 60 to 80	1.20	1.12	1.28
Age above 80	0.73	0.58	0.93
Ex Smoker	1.45	1.24	1.70
British	1.49	1.24	1.79
Any other Black background	1.09	1.02	1.16
Other White background (area)	0.89	0.82	0.95
White and Black Caribbean	1.19	1.04	1.37
Scottish (area)	1.20	1.12	1.28
Time	1.15	1.06	1.26

Supplementary Information S8: Further results on risk factors

Increasing cancer incidence over time was found for the breast, colorectal and urology cancer types, with breast cancer types increasing faster than the other two cancer types. Being a current smoker or ex-smoker is usually associated to an increased risk of cancer diagnosis. However, 'smoking' was found protective for upper GI and gynaecology cancer types. Apart from breast cancer, all the other cancer types were associated to one or more variables belonging to the ethnicity categorisation. Fifteen times they appeared as a risk factor and only four times as protective. Large risks were found for 'female age 25-49 mixed white and Asian' for lung cancer type and for 'any other black background' for skin cancer type. 'White and black Caribbean' and 'any other black background' were the most common risk factors appearing statistically significant for four and three cancer types, respectively (Table 1 main text).

By employing a Poisson generalised linear model for any selected important factor (listed in Table 1 of main text) as the outcome and each one of the remaining 783 variables considered in this study as a predictor, we found that the selected variables were mostly associated to higher values of ecological variables such as Small Area Mental Health Index (meaning worst mental health conditions), unemployment, criminality (shoplifting, criminal damage) and lone parenting (Supplementary Table below). The Small Area Mental Health Index takes into consideration depression rates, incapacity benefit and employment support allowance for mental illness, prescription of antidepressant, and mental health related hospital attendances. In the mediation analysis, the risk factors of only two cancer types mediated other factors. For breast cancer the effect of diabetes was weakly influenced by 'age over 60' (coefficient of the average causal mediation effects for the variable 0.23, *p-value* = 0.04) and also by the 'Scottish ethnicity' (coefficient -0.15, *p-value* = 0.04); for urology cancer, the 'COVID19' variable was weakly influenced by 'never smoker' (coefficient -0.09, *p-value* = 0.02) and 'Scottish ethnicity' (coefficient 0.32, *p-value* = 0.02).

Supplementary Table. Largest statistically significant associations between selected important variables (selected variable) and other variables in the dataset.

Selected variable	Correlated variable	Regression coefficient	P-value
Age 60 to 80	Small Area Mental Health Index (higher values: worst)	0.066	<0.001
Age above 80	Small Area Mental Health Index (higher values: worst)	0.063	<0.001
Any other Black background	Not living in a couple	0.649	0.006
British	Small Area Mental Health Index (higher values: worst)	0.095	<0.001
Chronic Kidney Disease	Distance to nearest Tobacconists/Vape Store (minutes)	-0.015	<0.001
Congestive Hearth Failure	Small Area Mental Health Index (higher values: worst)	0.086	<0.001
Coronary Heart Disease	Small Area Mental Health Index (higher values: worst)	0.076	<0.001
COVID19	Shoplifting	0.063	0.001
Depression	Small Area Mental Health Index (higher values: worst)	0.065	<0.001
Diabetes	Small Area Mental Health Index (higher values: worst)	0.063	<0.001
Ex Smoker	Distance to nearest GP Practice (minutes)	-0.036	<0.001
Frailty (number of fit)	Small Area Mental Health Index (higher values: worst)	0.052	<0.001
Hypertension	Small Area Mental Health Index (higher values: worst)	0.073	<0.001
Female age 25-49 mixed white and Asian (area)	Cohabitating	-0.055	<0.001
Northern Irish (area)	Asian, Asian British	0.298	0.006
Other White background (area)	Criminal damage and arson	0.778	0.009
Pakistani	Shoplifting	0.007	0.006
Self employed (including fulltime students) one parent working (area)	Shoplifting	0.011	<0.001
Scottish (area)	Loneparenting	0.057	0.006
Smoker	Small Area Mental Health Index (higher values: worst)	0.093	<0.001
White and Black Caribbean	Small Area Mental Health Index (higher values: worst)	1.751	0.010

Supplementary Information S9: Cancer type risk mapping and relative standard errors

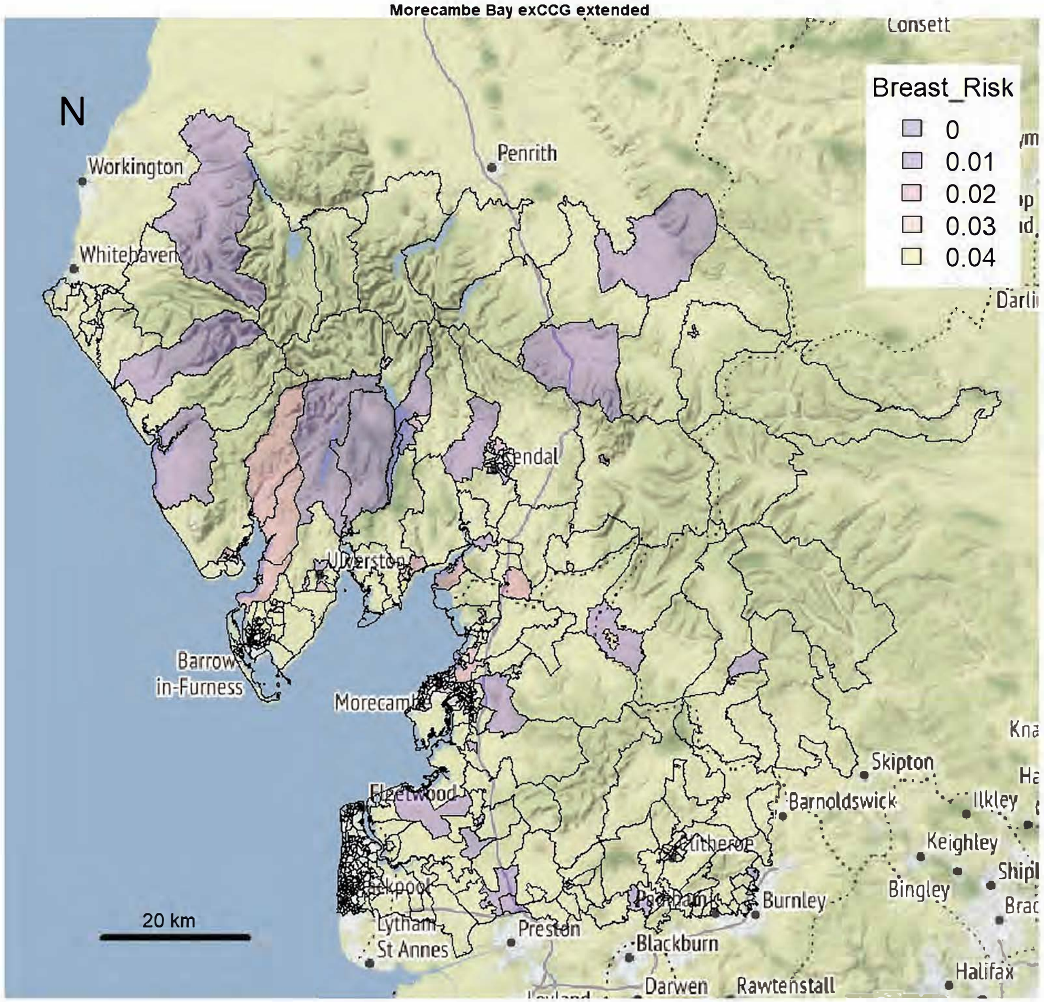

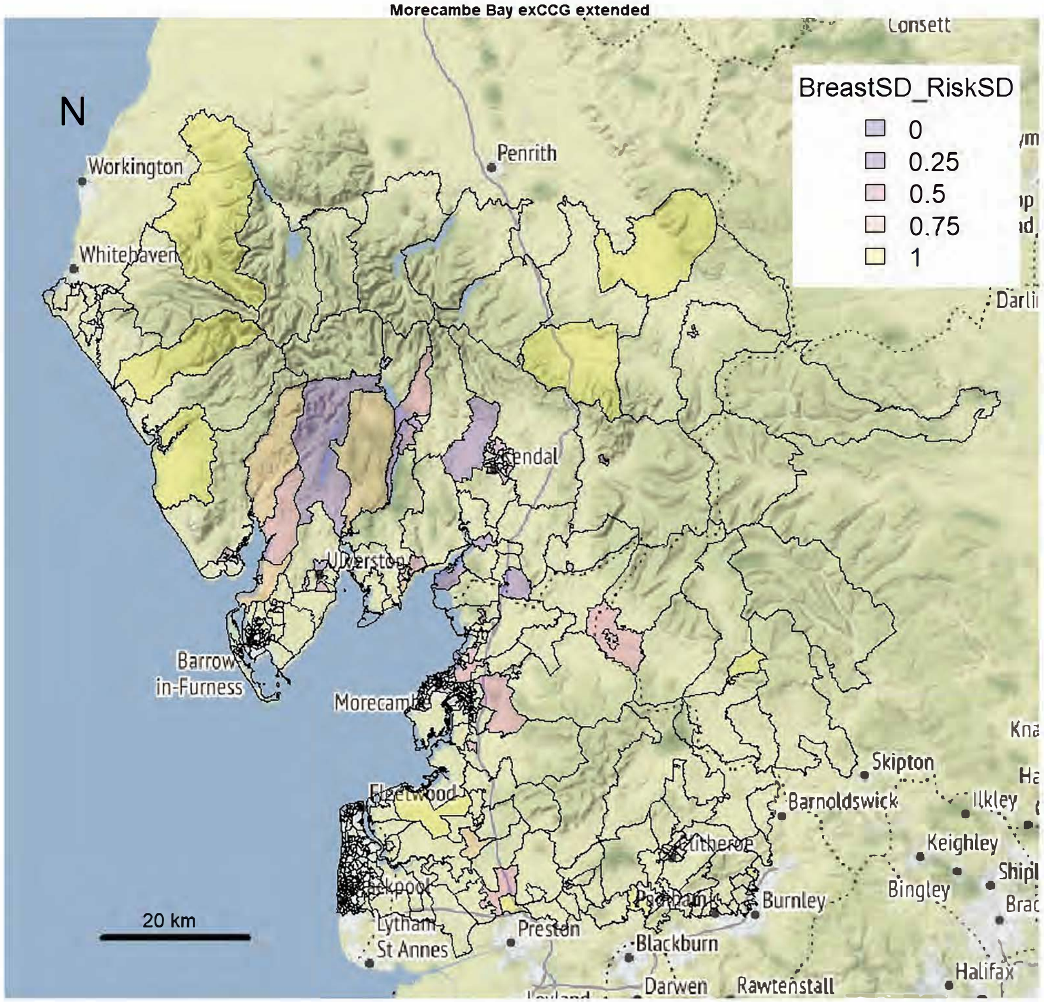

Morecambe Bay exCCG extended

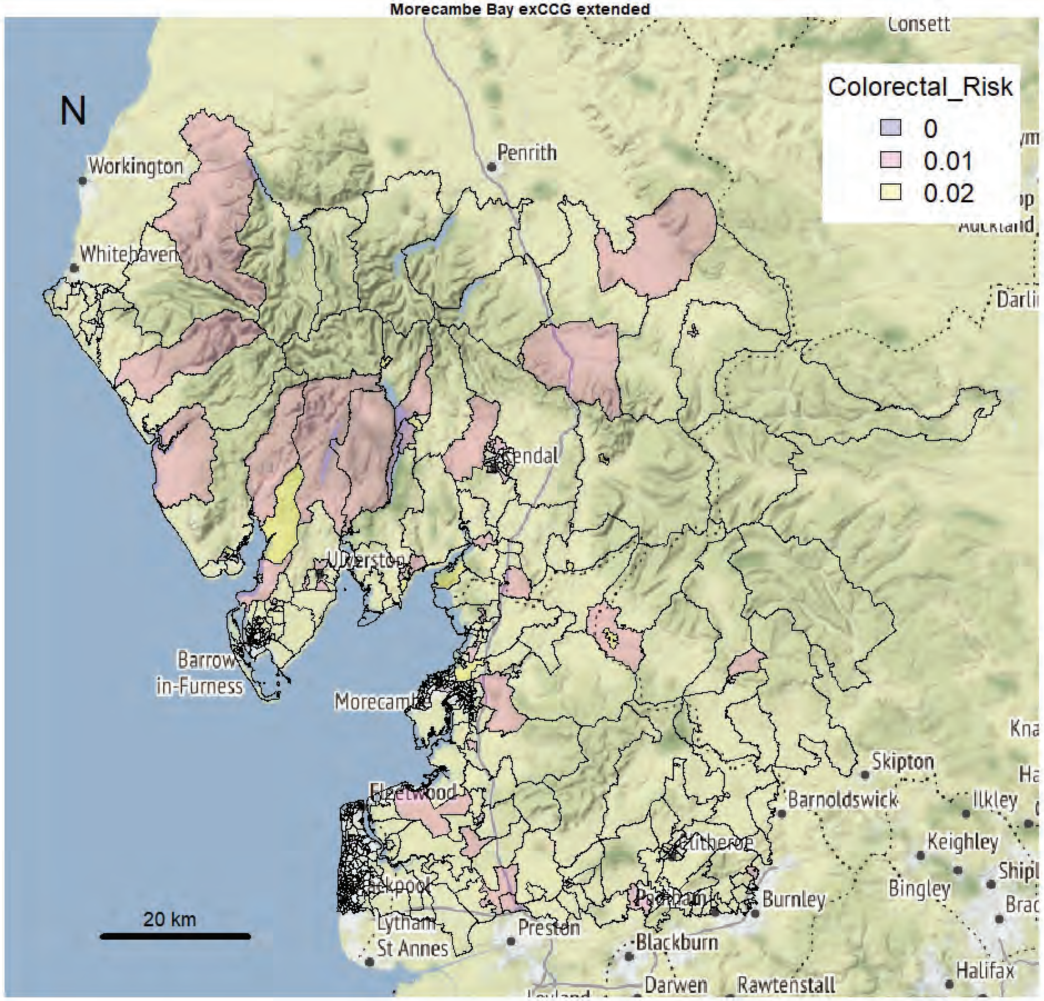

Morecambe Bay exCCG extended

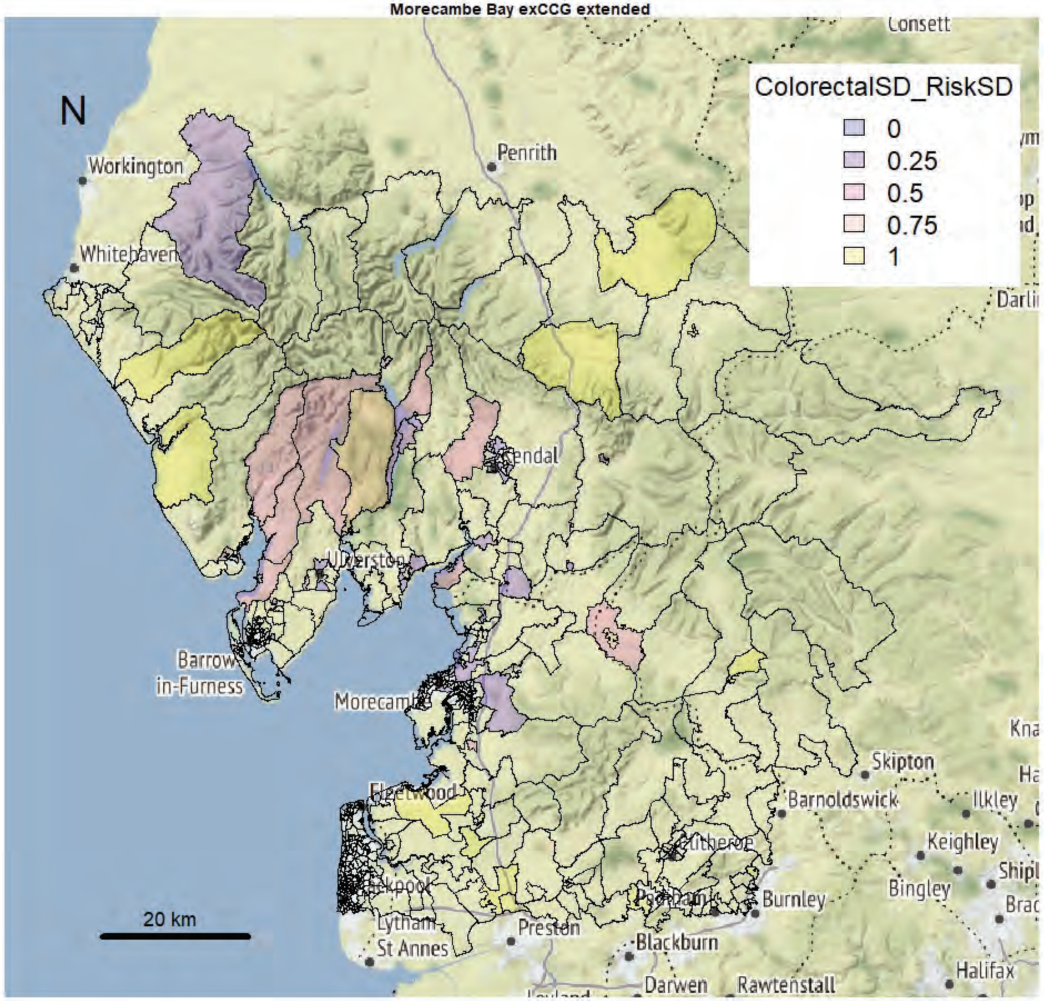

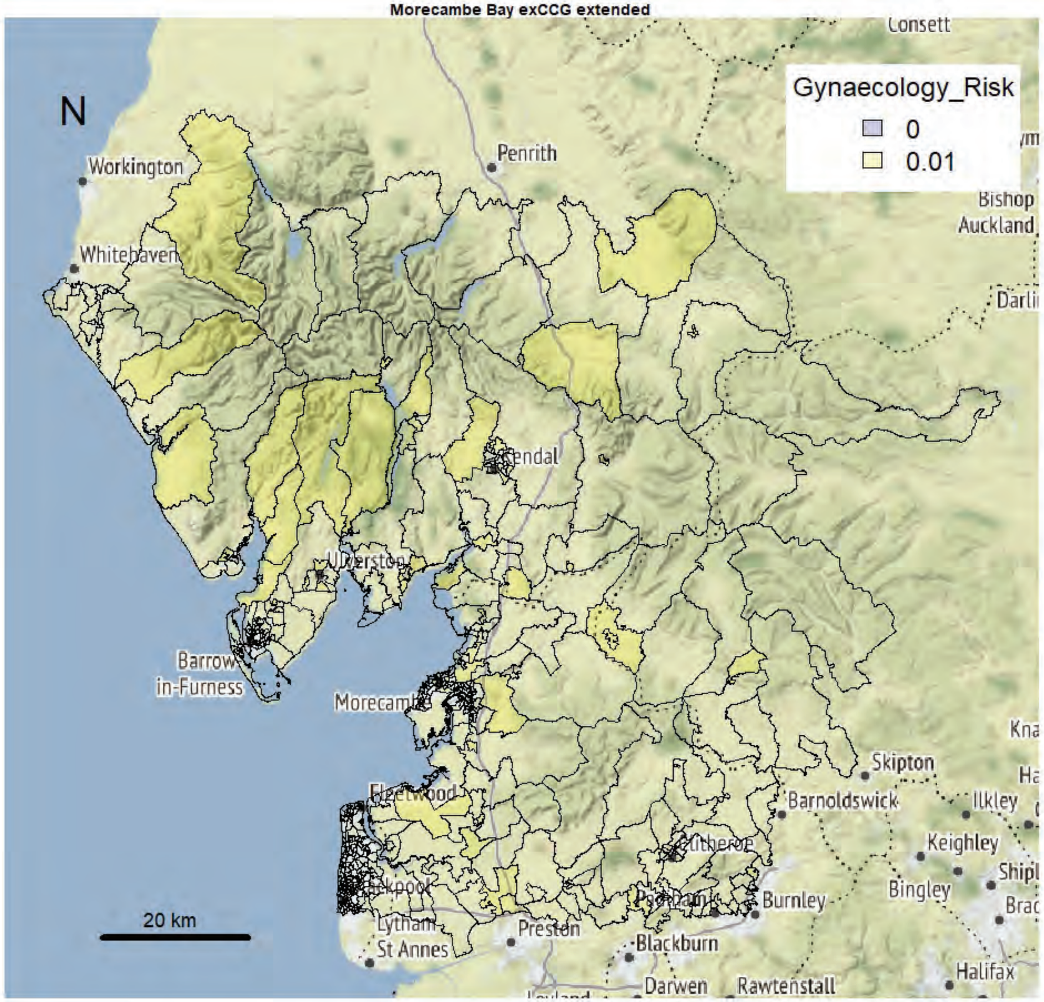
Morecambe Bay exCCG extended
N
Gynaecology_Risk
0
0.01
20 km
Workington
Penrith
Whitehaven
Kendal
Ulverston
Barrow-in-Furness
Morecambe
Fleetwood
Blackpool
Lytham St Annes
Preston
Blackburn
Darwen
Rawtenstall
Burnley
Clitheroe
Skipton
Barnoldswick
Ilkley
Keighley
Bingley
Halifax
Consett
Bishop Auckland

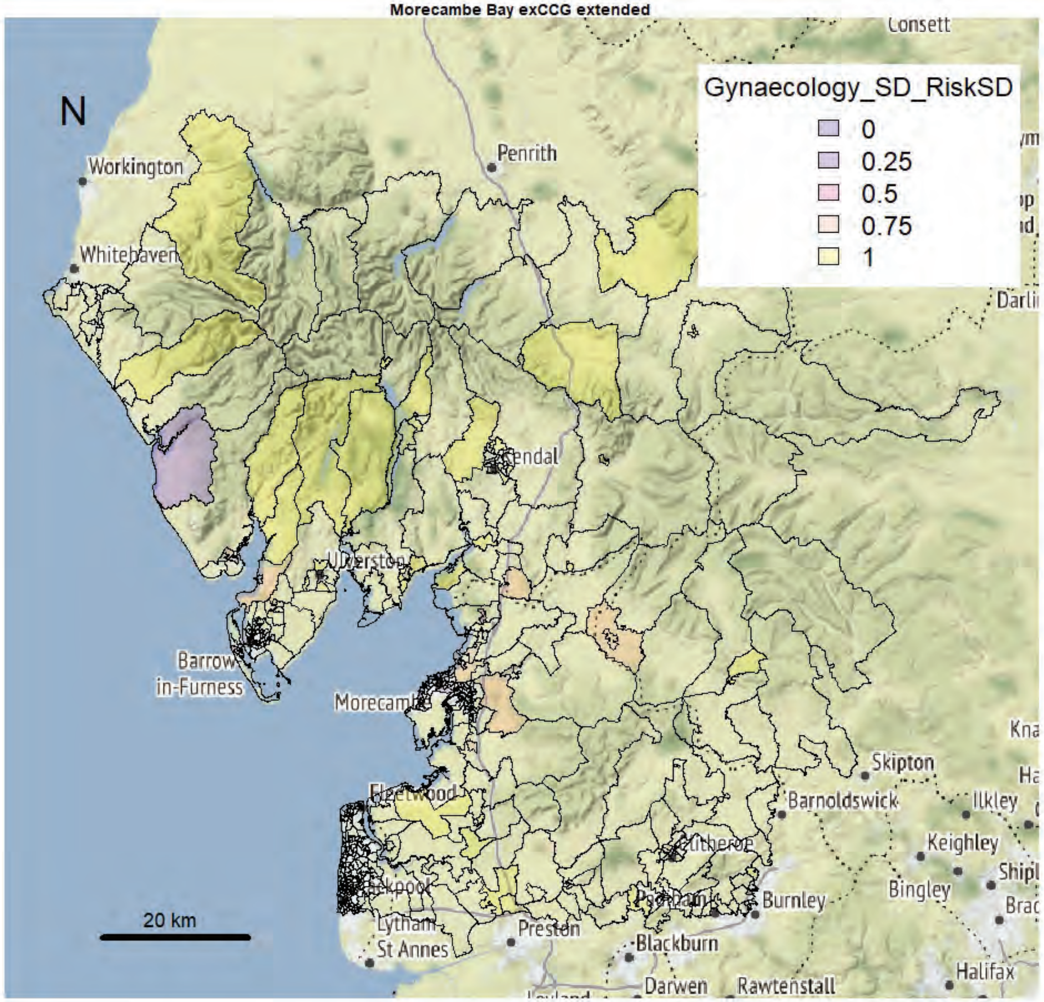
Morecambe Bay exCCG extended
N
Gynaecology_SD_RiskSD
0
0.25
0.5
0.75
1
20 km
Workington
Penrith
Whitehaven
Kendal
Ulverston
Barrow-in-Furness
Morecambe
Fleetwood
Blackpool
Lytham St Annes
Preston
Blackburn
Darwen
Rawtenstall
Burnley
Clitheroe
Skipton
Barnoldswick
Ilkley
Keighley
Bingley
Halifax
Consett

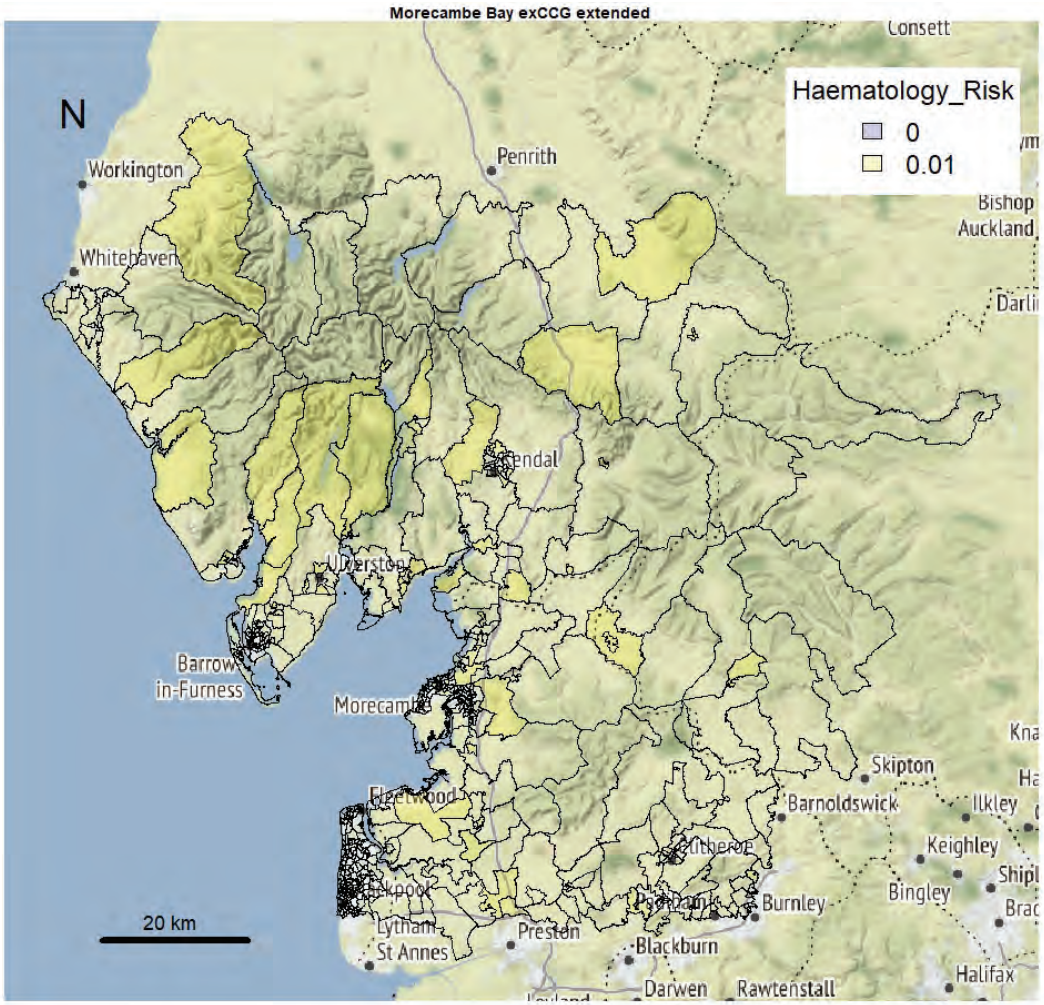
Morecambe Bay exCCG extended
Haematology_Risk
0
0.01
N
20 km
Workington
Whitehaven
Penrith
Consett
Bishop Auckland
Kendal
Ulverston
Barrow-in-Furness
Morecambe
Fleetwood
Blackpool
Lytham St Annes
Preston
Blackburn
Darwen
Rawtenstall
Clitheroe
Burnley
Skipton
Barnoldswick
Ilkley
Keighley
Bingley
Halifax

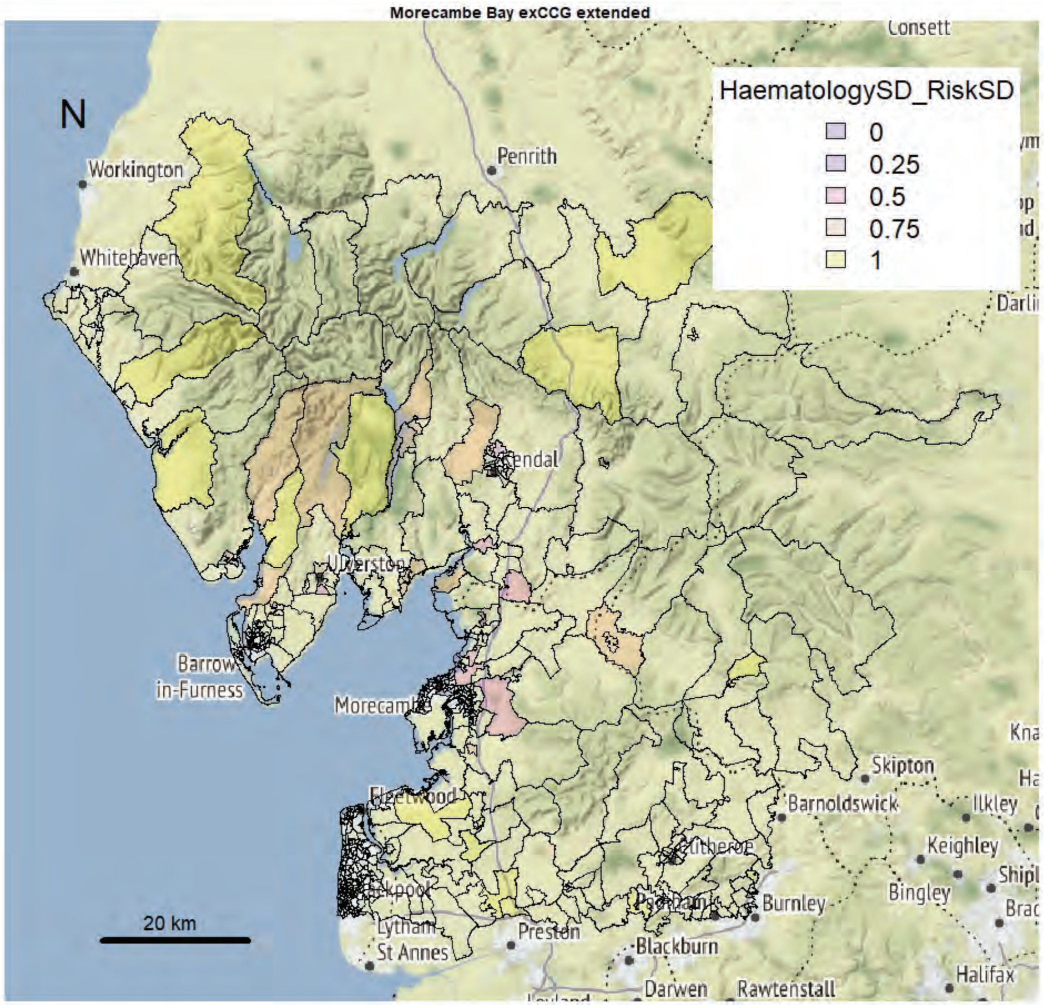
Morecambe Bay exCCG extended
HaematologySD_RiskSD
0
0.25
0.5
0.75
1
N
20 km
Workington
Whitehaven
Penrith
Consett
Kendal
Ulverston
Barrow-in-Furness
Morecambe
Fleetwood
Blackpool
Lytham St Annes
Preston
Blackburn
Darwen
Rawtenstall
Clitheroe
Burnley
Skipton
Barnoldswick
Ilkley
Keighley
Bingley
Halifax

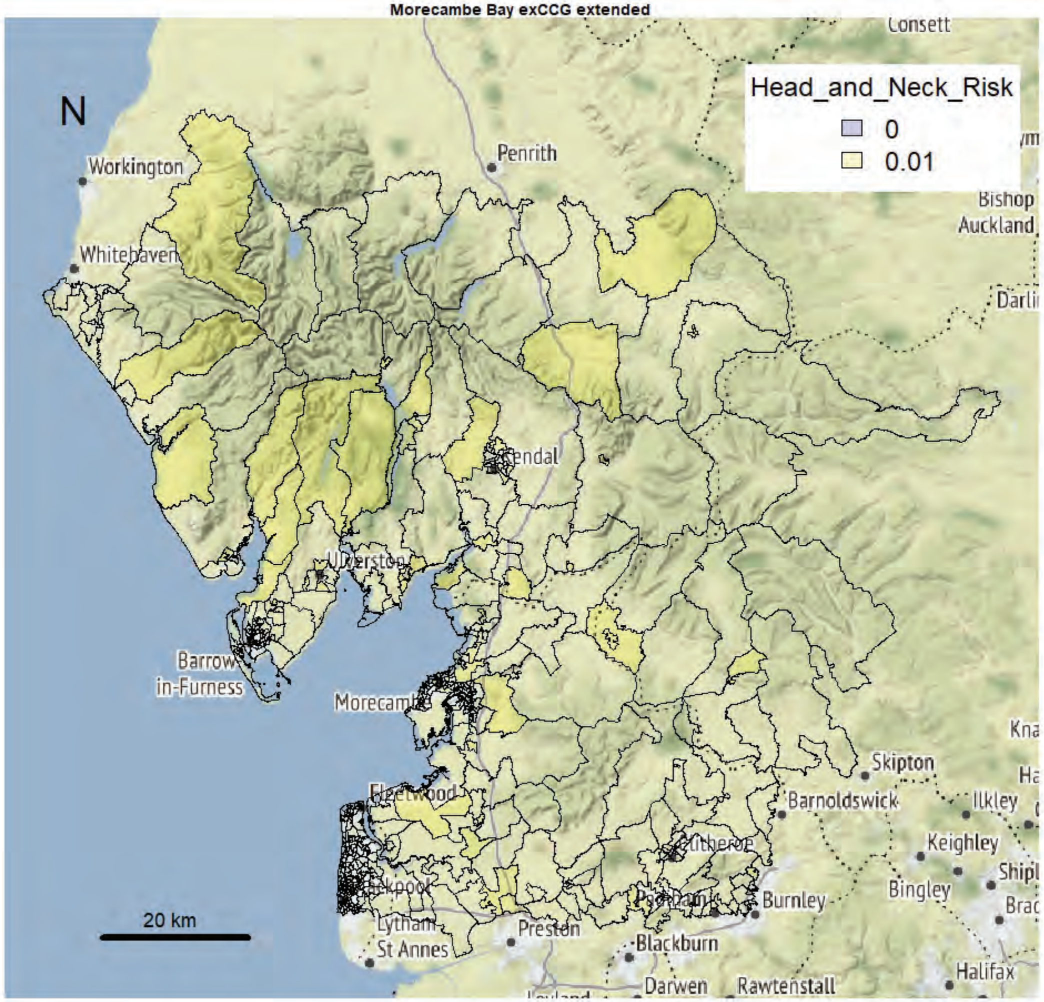
Morecambe Bay exCCG extended
N
Head_and_Neck_Risk
0
0.01
20 km
Workington
Penrith
Whitehaven
Kendal
Ulverston
Barrow-in-Furness
Morecambe
Fleetwood
Blackpool
Lytham St Annes
Preston
Blackburn
Darwen
Rawtenstall
Burnley
Clitheroe
Skipton
Barnoldswick
Ilkley
Keighley
Bingley
Halifax
Consett
Bishop Auckland

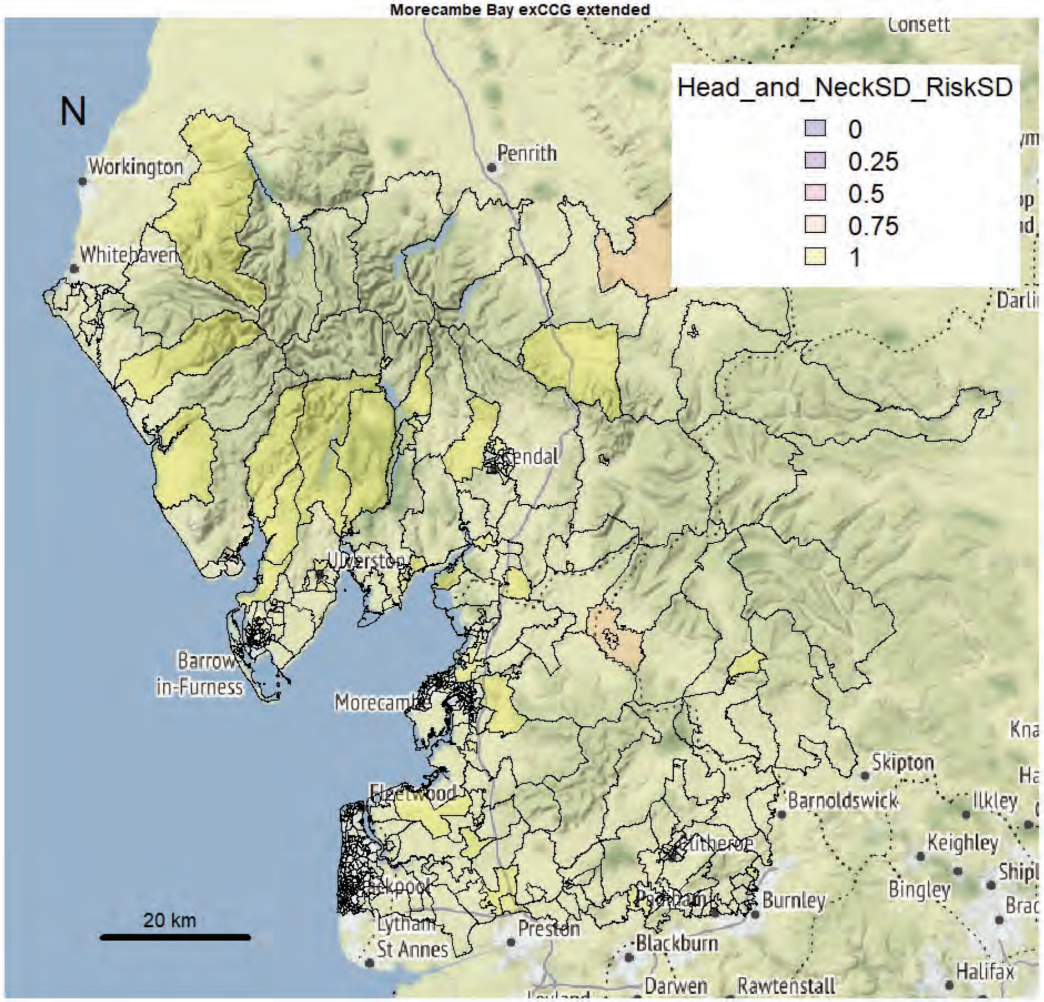
Morecambe Bay exCCG extended
N
Head_and_NeckSD_RiskSD
0
0.25
0.5
0.75
1
20 km
Workington
Penrith
Whitehaven
Kendal
Ulverston
Barrow-in-Furness
Morecambe
Fleetwood
Blackpool
Lytham St Annes
Preston
Blackburn
Darwen
Rawtenstall
Burnley
Clitheroe
Skipton
Barnoldswick
Ilkley
Keighley
Bingley
Halifax
Consett

Morecambe Bay exCCG extended

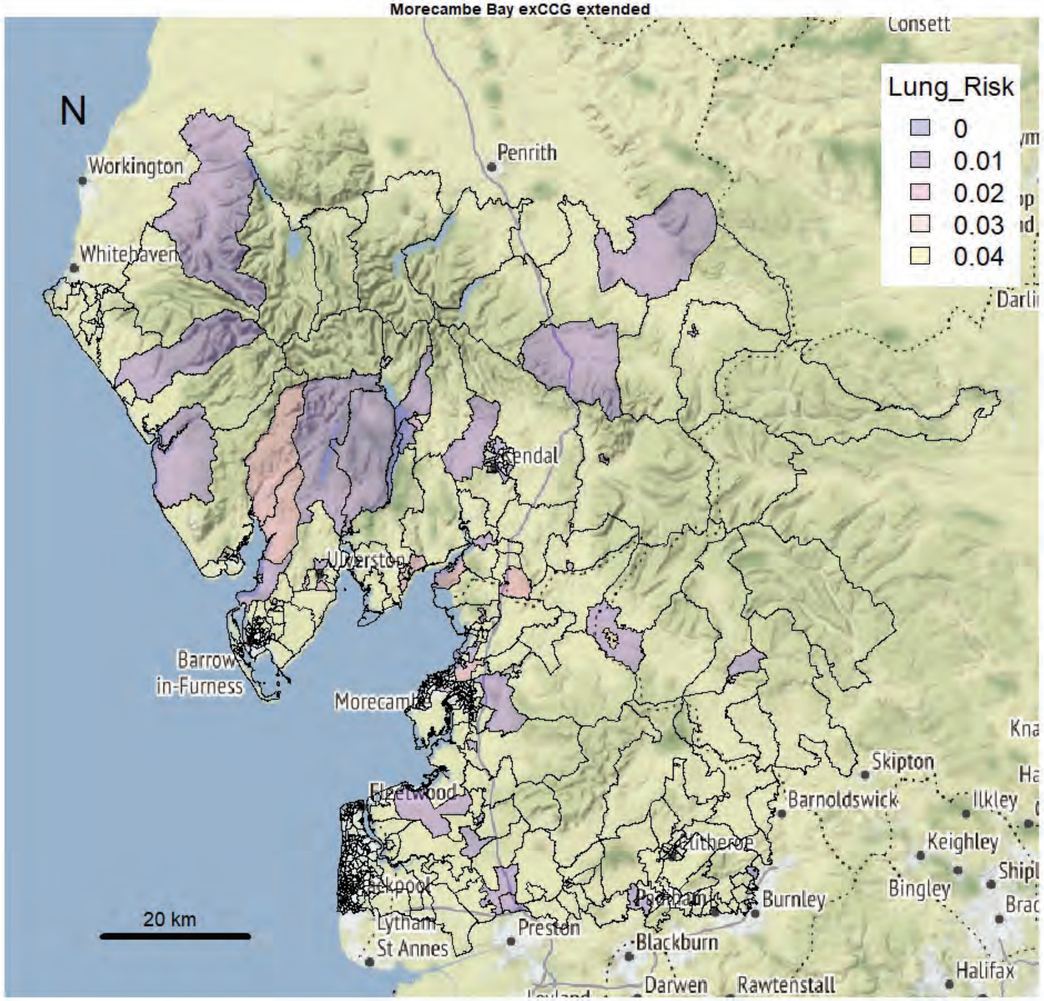

Morecambe Bay exCCG extended

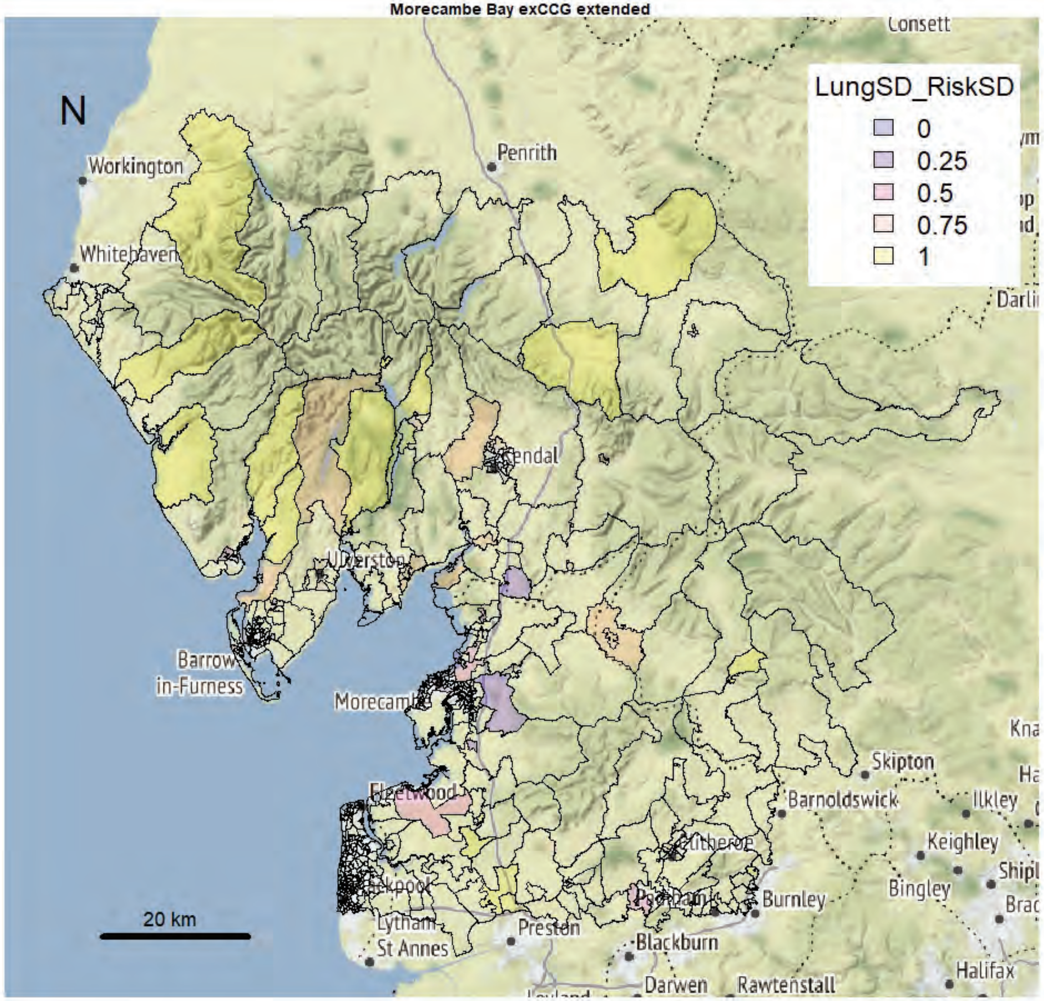

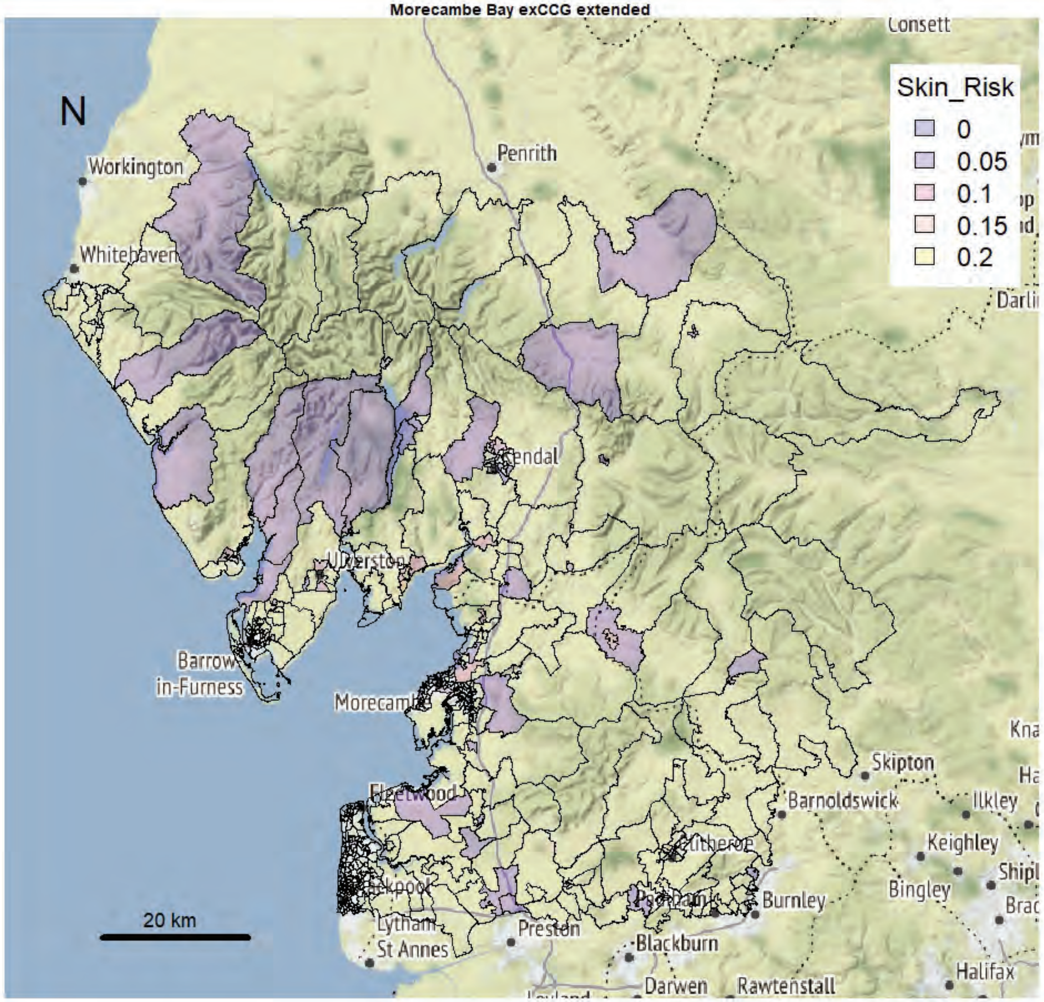
Morecambe Bay exCCG extended
N
Skin_Risk
0
0.05
0.1
0.15
0.2
Workington
Penrith
Whitehaven
Kendal
Barrow-in-Furness
Morecambe
Fleetwood
Blackpool
Lytham St Annes
Preston
Blackburn
Darwen
Rawtenstall
Burnley
Skipton
Barnoldswick
Ilkley
Keighley
Bingley
Halifax
Consett
20 km

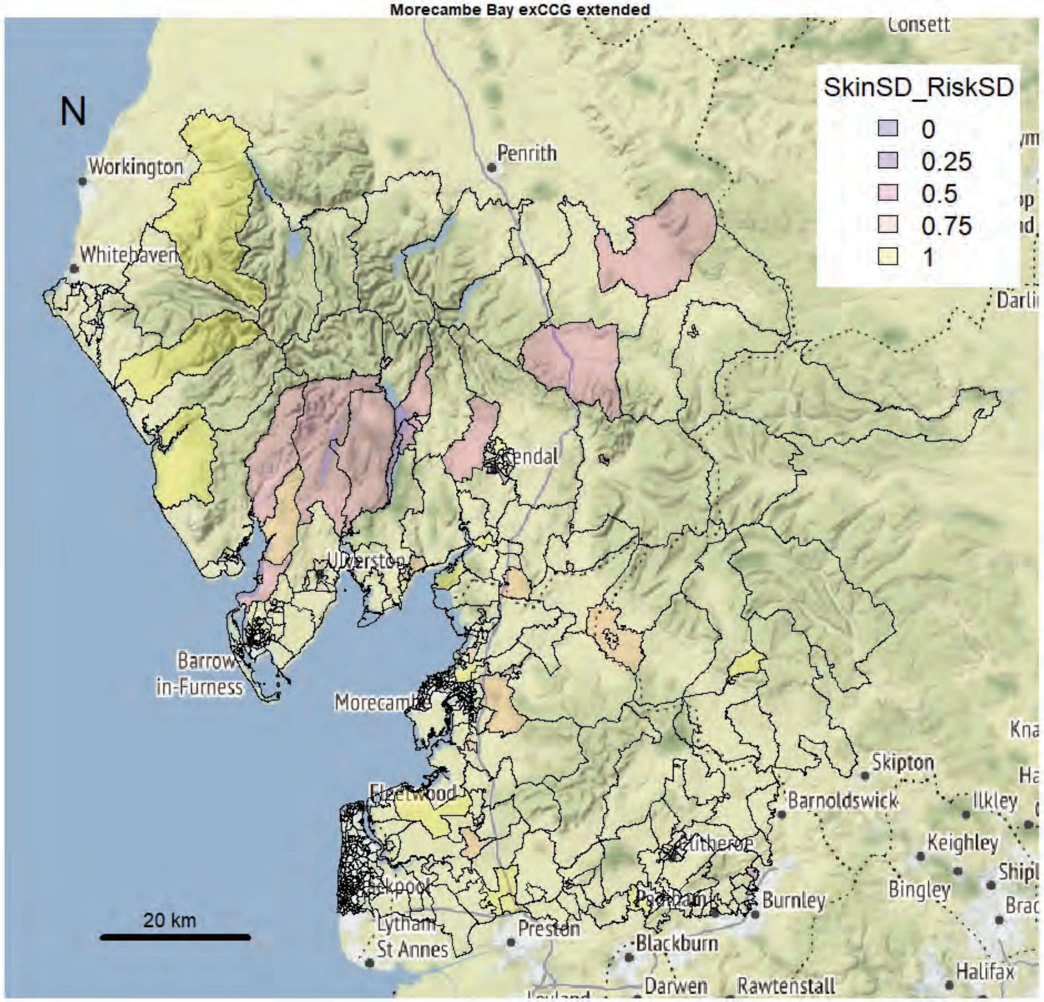
Morecambe Bay exCCG extended
N
SkinSD_RiskSD
0
0.25
0.5
0.75
1
Workington
Penrith
Whitehaven
Kendal
Barrow-in-Furness
Morecambe
Fleetwood
Blackpool
Lytham St Annes
Preston
Blackburn
Darwen
Rawtenstall
Burnley
Skipton
Barnoldswick
Ilkley
Keighley
Bingley
Halifax
Consett
20 km

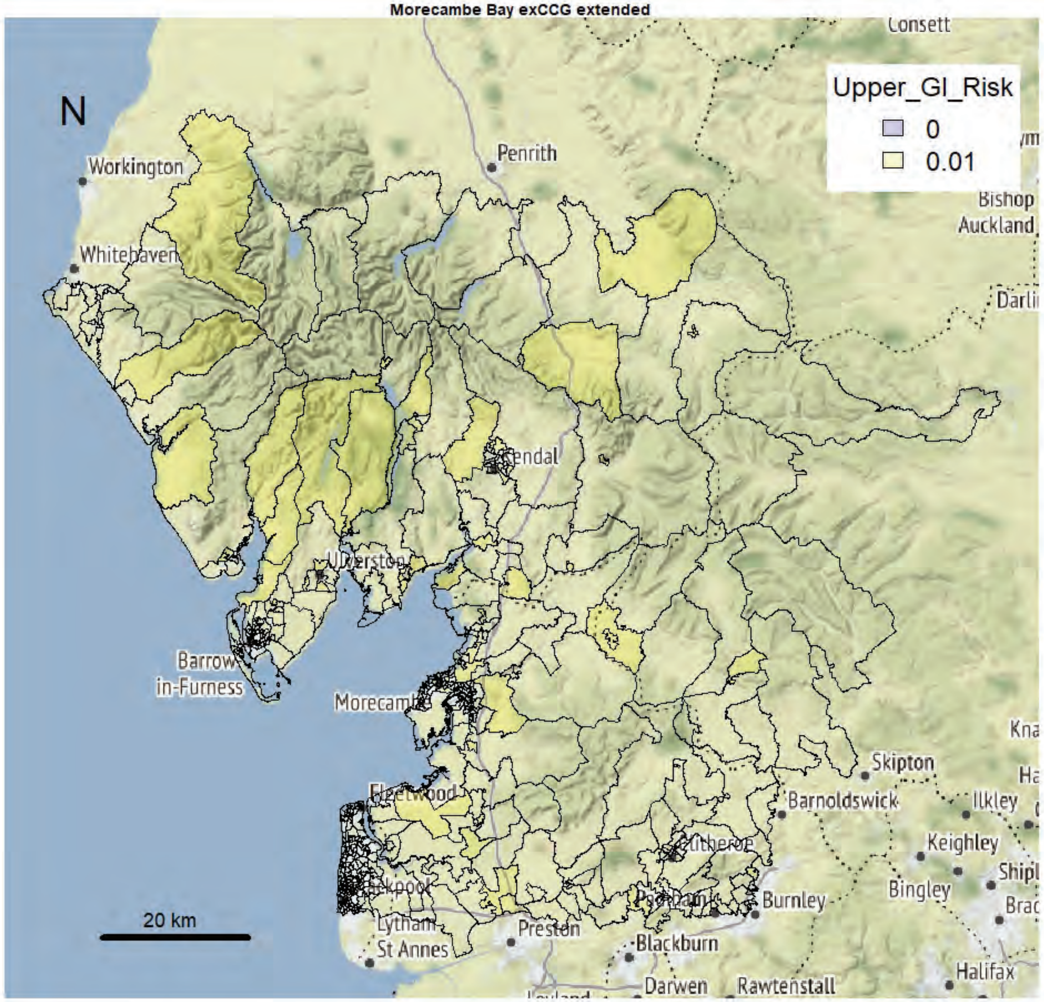
Morecambe Bay exCCG extended
Upper_GI_Risk
0
0.01
N
20 km
Workington
Whitehaven
Penrith
Kendal
Barrow-in-Furness
Morecambe
Fleetwood
Blackpool
Lytham St Annes
Preston
Blackburn
Darwen
Rawtenstall
Burnley
Skipton
Barnoldswick
Ilkley
Keighley
Bingley
Halifax
Consett
Bishop Auckland

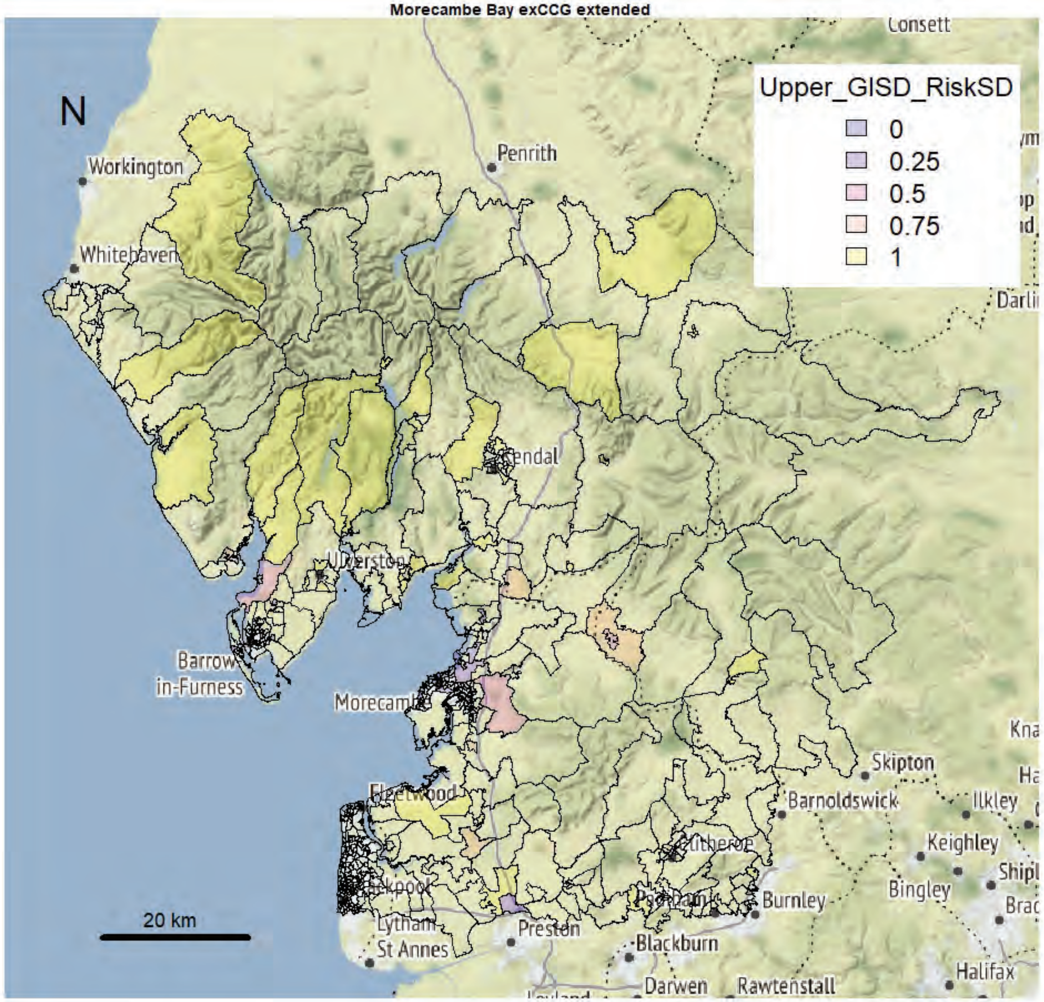
Morecambe Bay exCCG extended
Upper_GISD_RiskSD
0
0.25
0.5
0.75
1
N
20 km
Workington
Whitehaven
Penrith
Kendal
Barrow-in-Furness
Morecambe
Fleetwood
Blackpool
Lytham St Annes
Preston
Blackburn
Darwen
Rawtenstall
Burnley
Skipton
Barnoldswick
Ilkley
Keighley
Bingley
Halifax
Consett

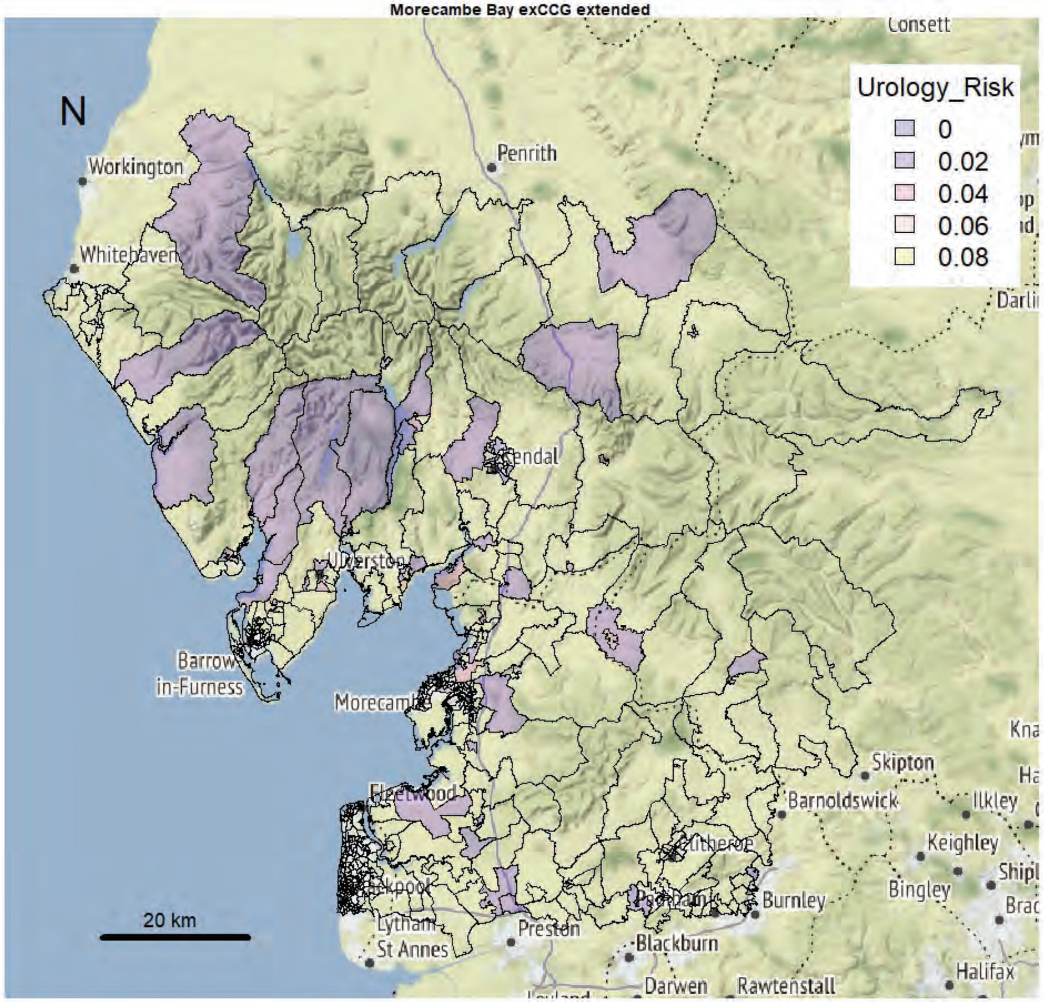
Morecambe Bay exCCG extended
Urology_Risk
0
0.02
0.04
0.06
0.08
N
20 km
Workington
Whitehaven
Penrith
Kendal
Ulverston
Barrow-in-Furness
Morecambe
Fleetwood
Blackpool
Lytham St Annes
Preston
Blackburn
Darwen
Rawtenstall
Burnley
Clitheroe
Skipton
Barnoldswick
Ilkley
Keighley
Bingley
Halifax
Consett

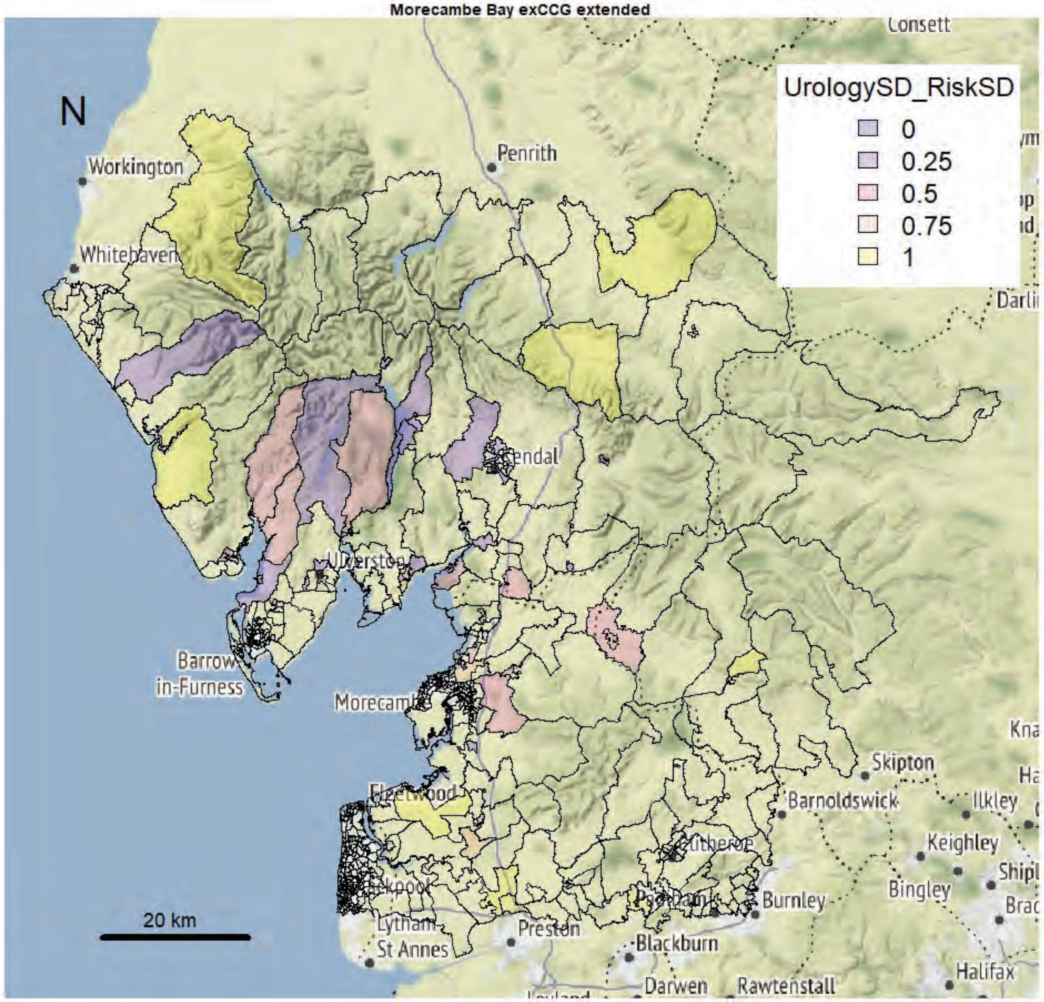
Morecambe Bay exCCG extended
UrologySD_RiskSD
0
0.25
0.5
0.75
1
N
20 km
Workington
Whitehaven
Penrith
Kendal
Ulverston
Barrow-in-Furness
Morecambe
Fleetwood
Blackpool
Lytham St Annes
Preston
Blackburn
Darwen
Rawtenstall
Burnley
Clitheroe
Skipton
Barnoldswick
Ilkley
Keighley
Bingley
Halifax
Consett

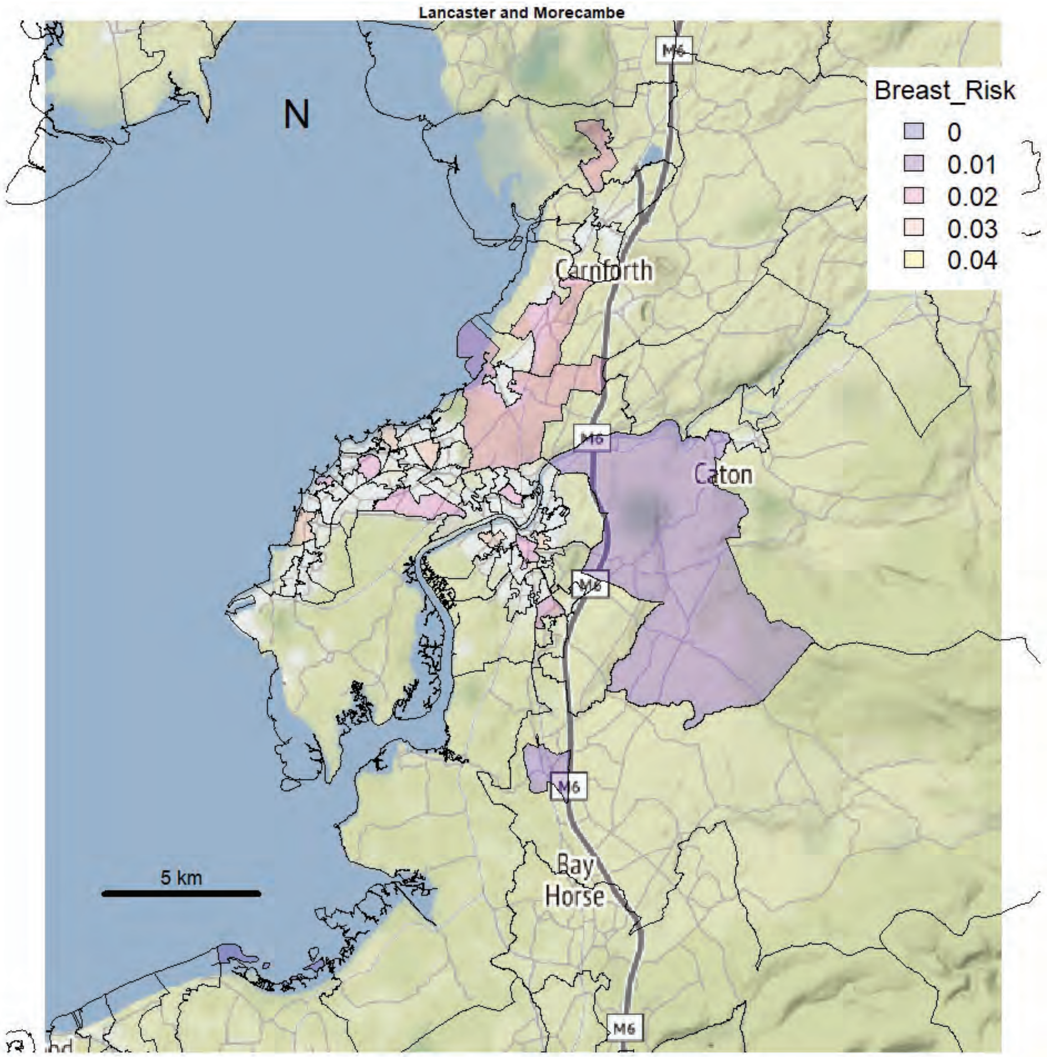
Lancaster and Morecambe
N
Breast_Risk
0
0.01
0.02
0.03
0.04
Carnforth
Caton
Bay
Horse
M6
5 km

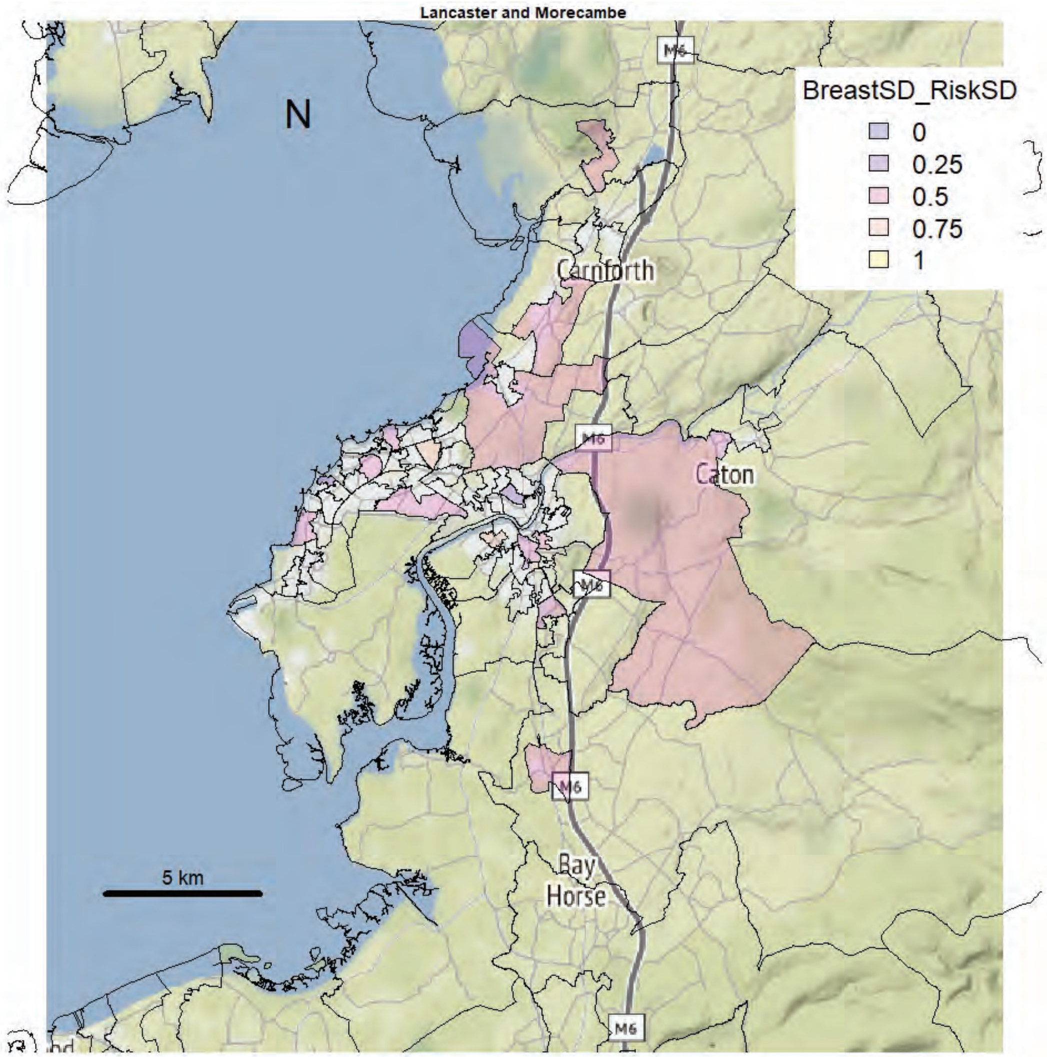
Lancaster and Morecambe
N
BreastSD_RiskSD
0
0.25
0.5
0.75
1
Carnforth
Caton
Bay
Horse
M6
5 km

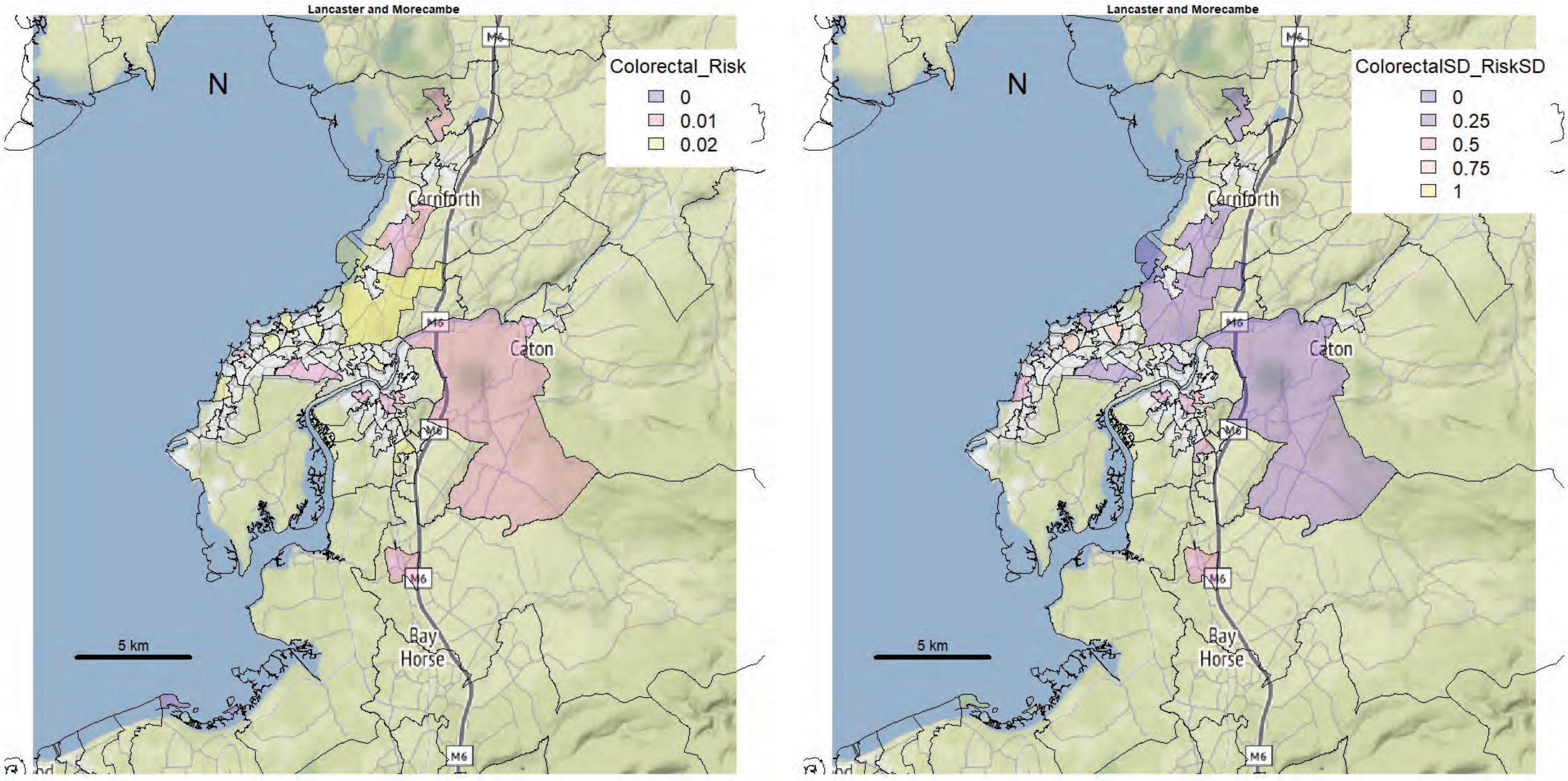

Lancaster and Morecambe
Colorectal_Risk
0
0.01
0.02
N
Carnforth
Caton
Bay Horse
M6
5 km
Lancaster and Morecambe
ColorectalSD_RiskSD
0
0.25
0.5
0.75
1
N
Carnforth
Caton
Bay Horse
M6
5 km

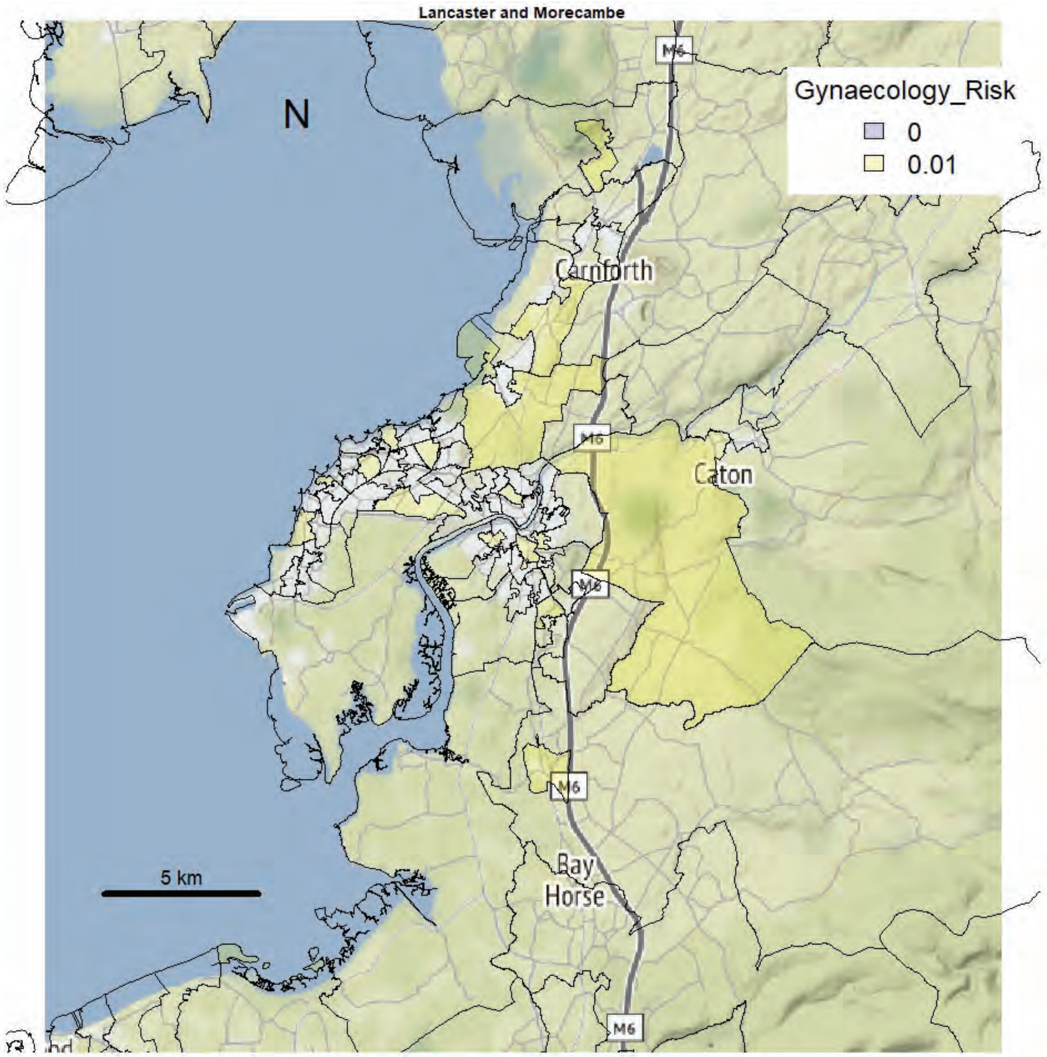
Lancaster and Morecambe
Gynaecology_Risk
0
0.01
N
Carnforth
Caton
Bay
Horse
M6
5 km
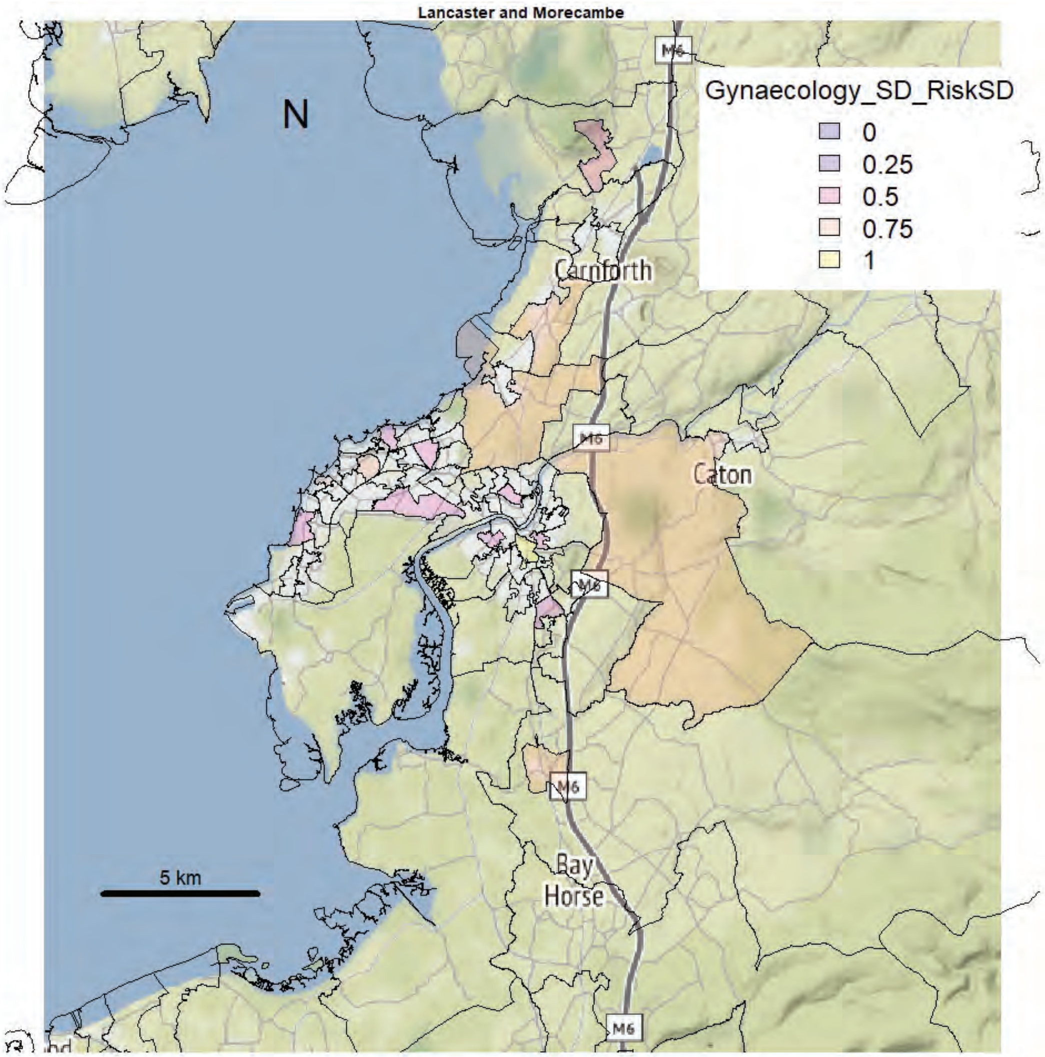
Lancaster and Morecambe
Gynaecology_SD_RiskSD
0
0.25
0.5
0.75
1
N
Carnforth
Caton
Bay
Horse
M6
5 km

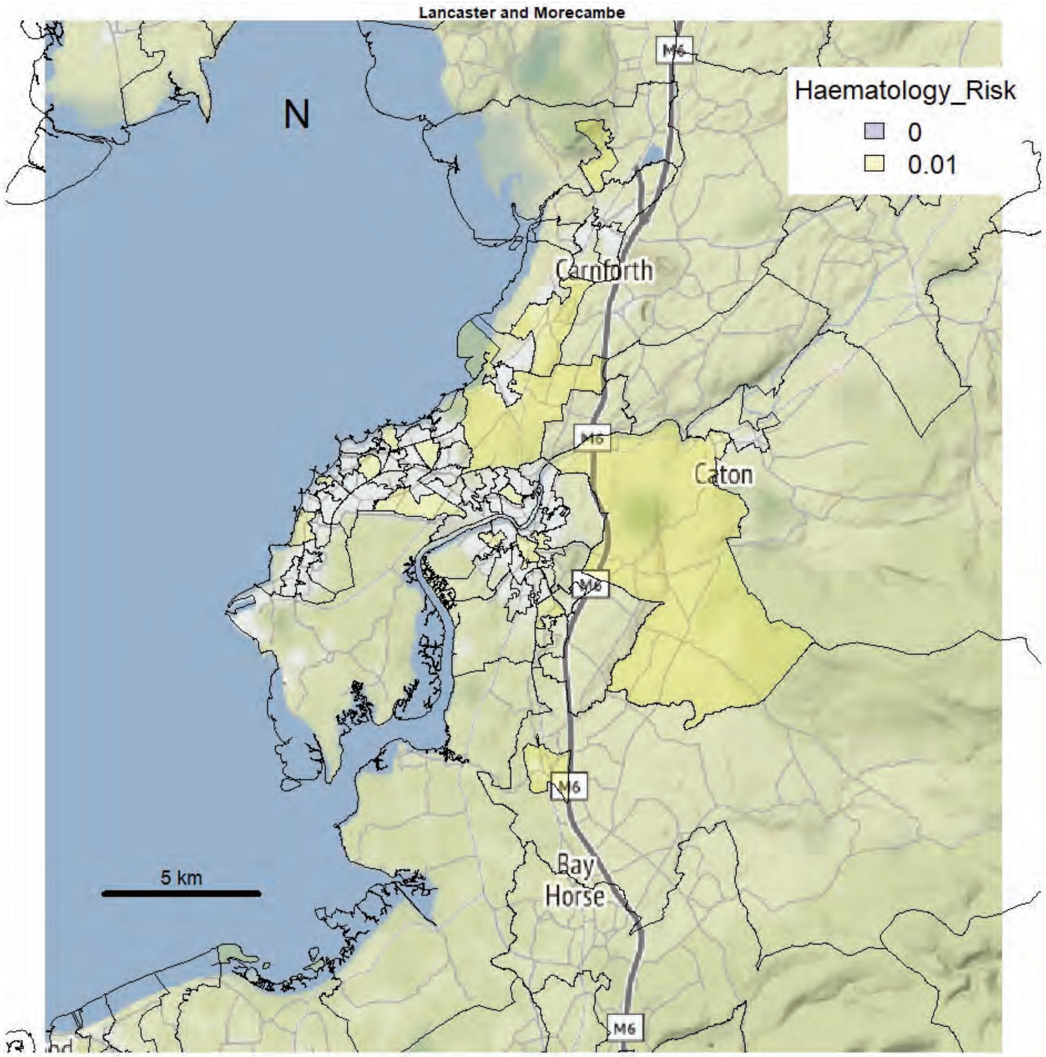
Lancaster and Morecambe
Haematology_Risk
0
0.01
N
Carnforth
Caton
Bay
Horse
M6
5 km

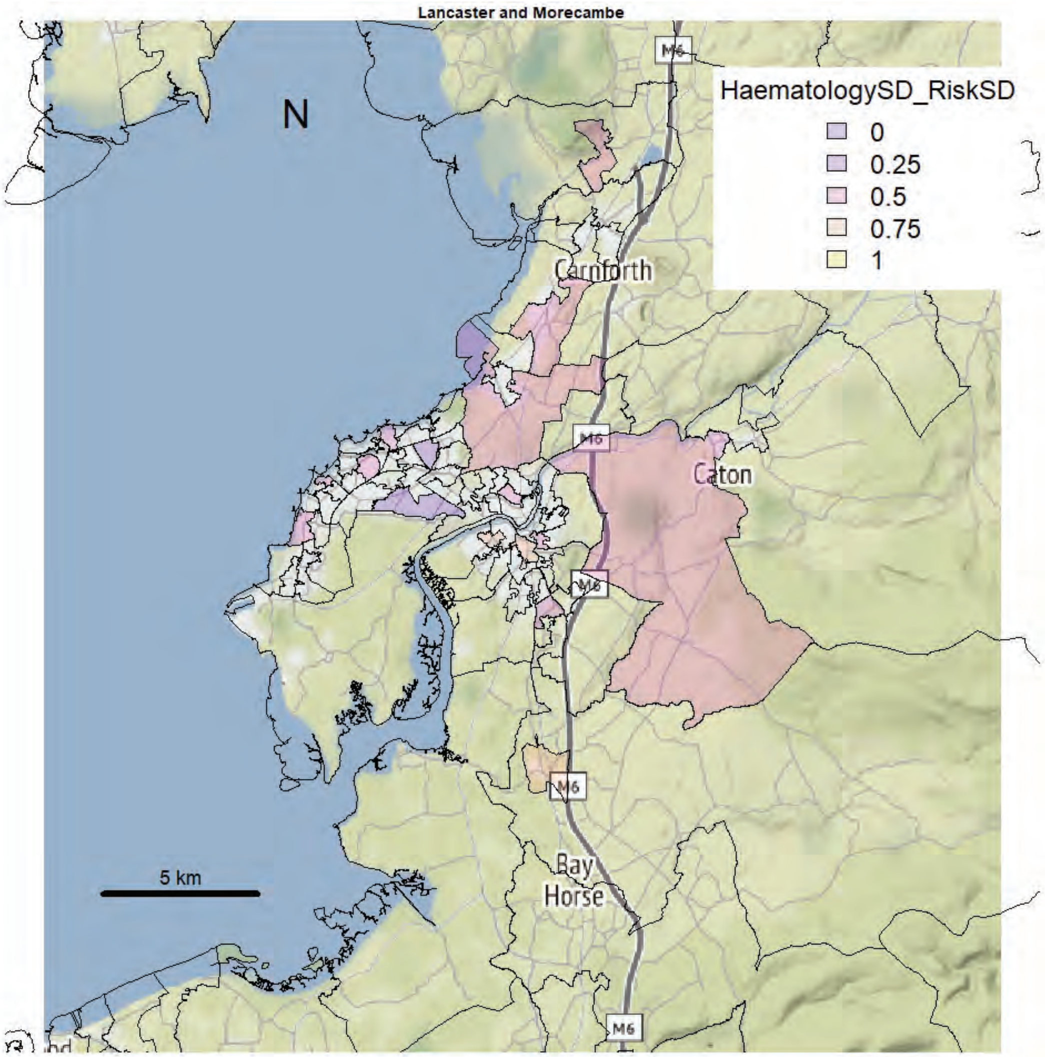
Lancaster and Morecambe
HaematologySD_RiskSD
0
0.25
0.5
0.75
1
N
Carnforth
Caton
Bay
Horse
M6
5 km

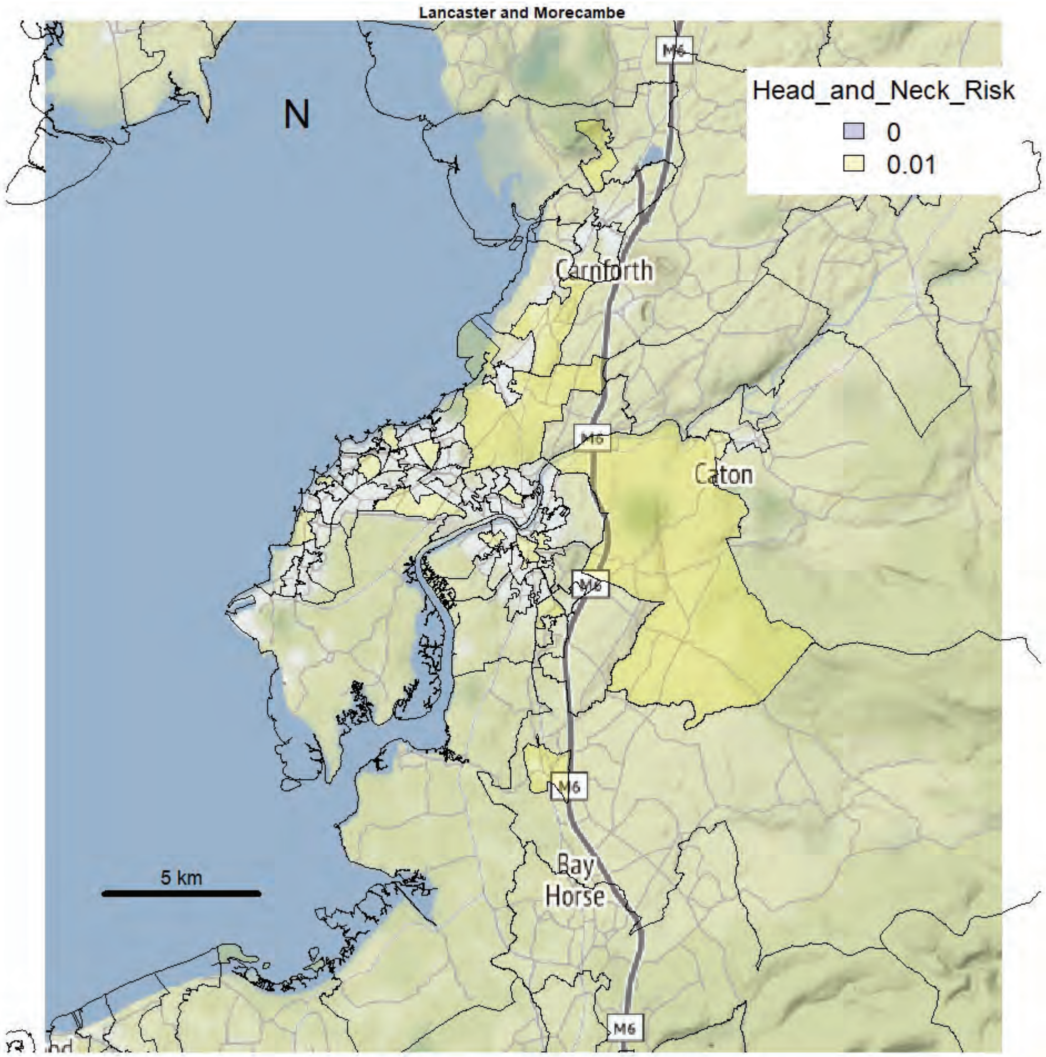

Lancaster and Morecambe
Head_and_Neck_Risk
0
0.01
N
Carnforth
Caton
Bay
Horse
M6
5 km

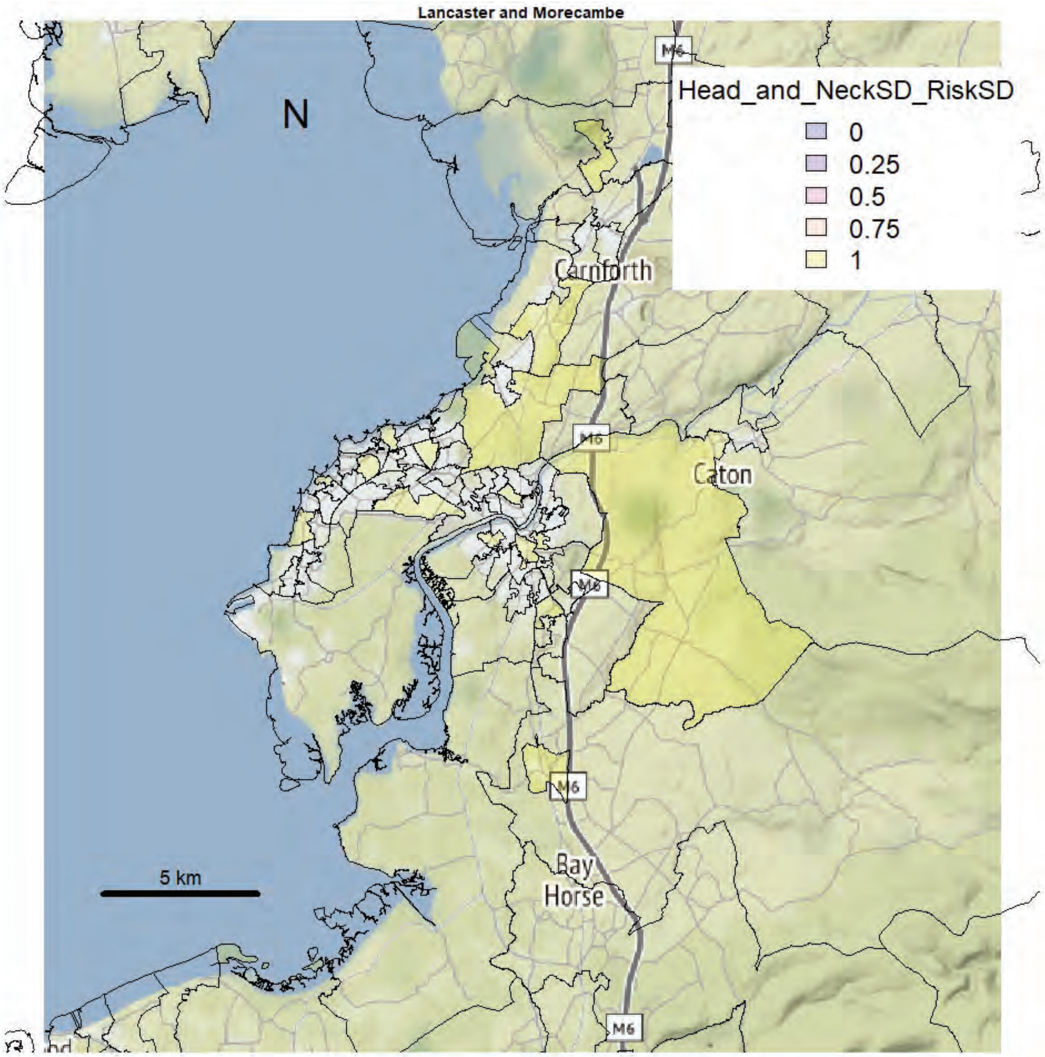

Lancaster and Morecambe
Head_and_NeckSD_RiskSD
0
0.25
0.5
0.75
1
N
Carnforth
Caton
Bay
Horse
M6
5 km

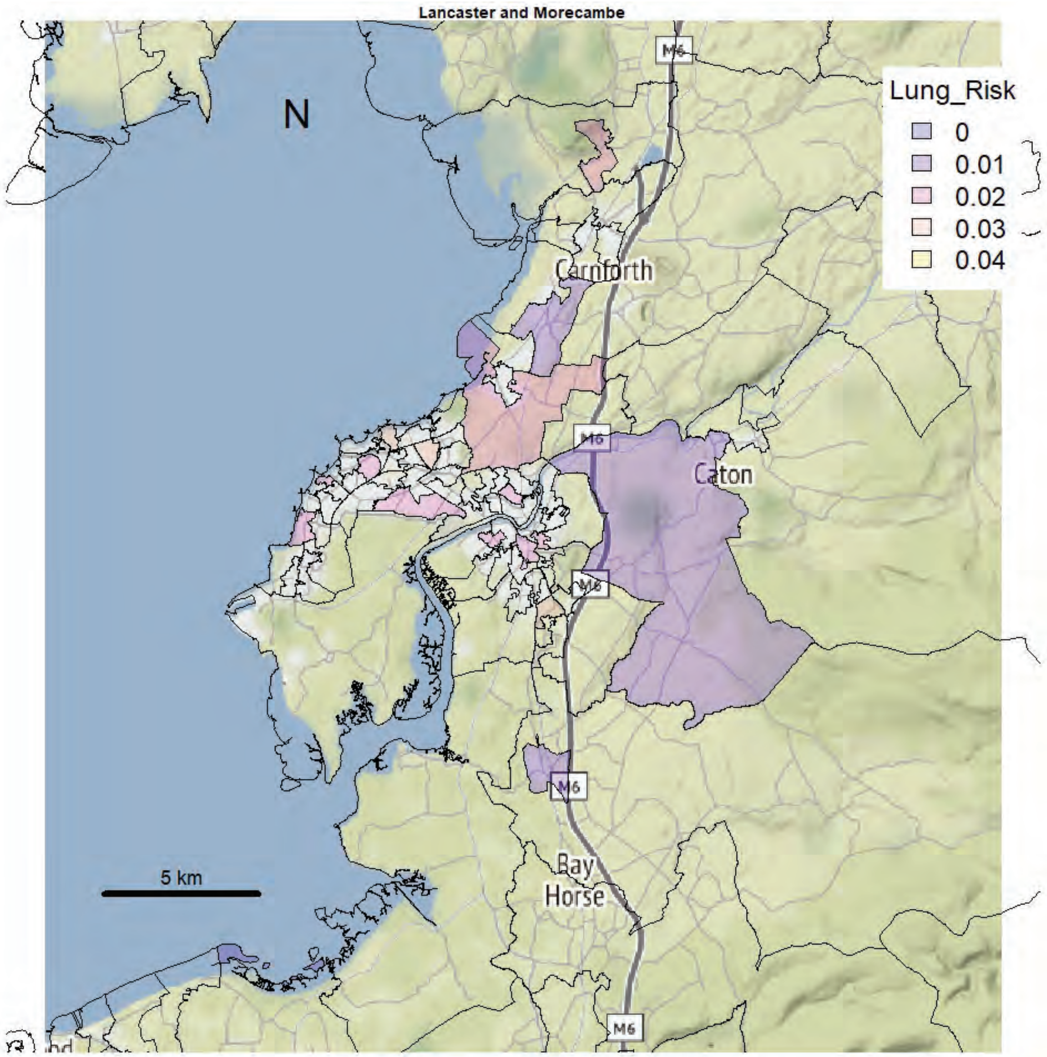
Lancaster and Morecambe
N
Lung_Risk
0
0.01
0.02
0.03
0.04
Carnforth
Caton
Bay Horse
M6
5 km

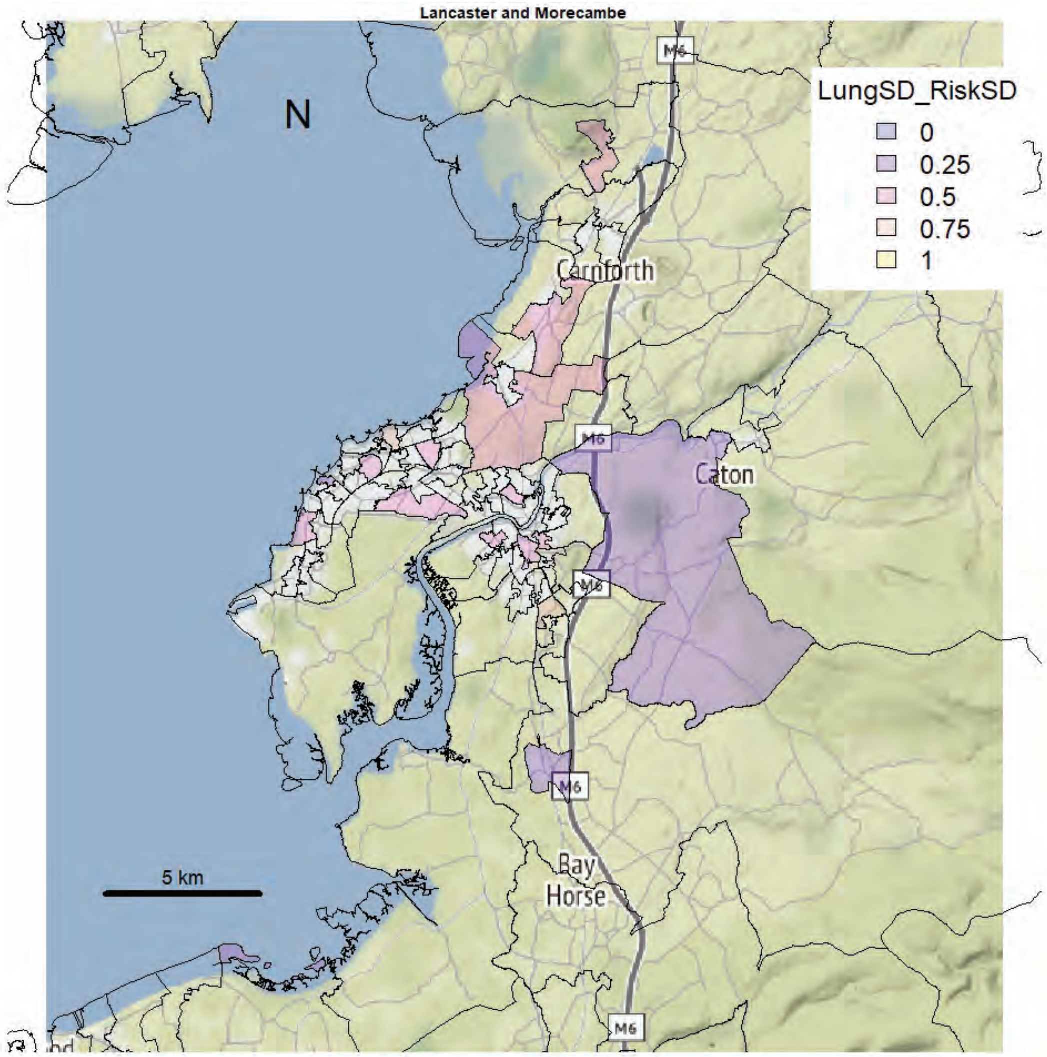
Lancaster and Morecambe
N
LungSD_RiskSD
0
0.25
0.5
0.75
1
Carnforth
Caton
Bay Horse
M6
5 km

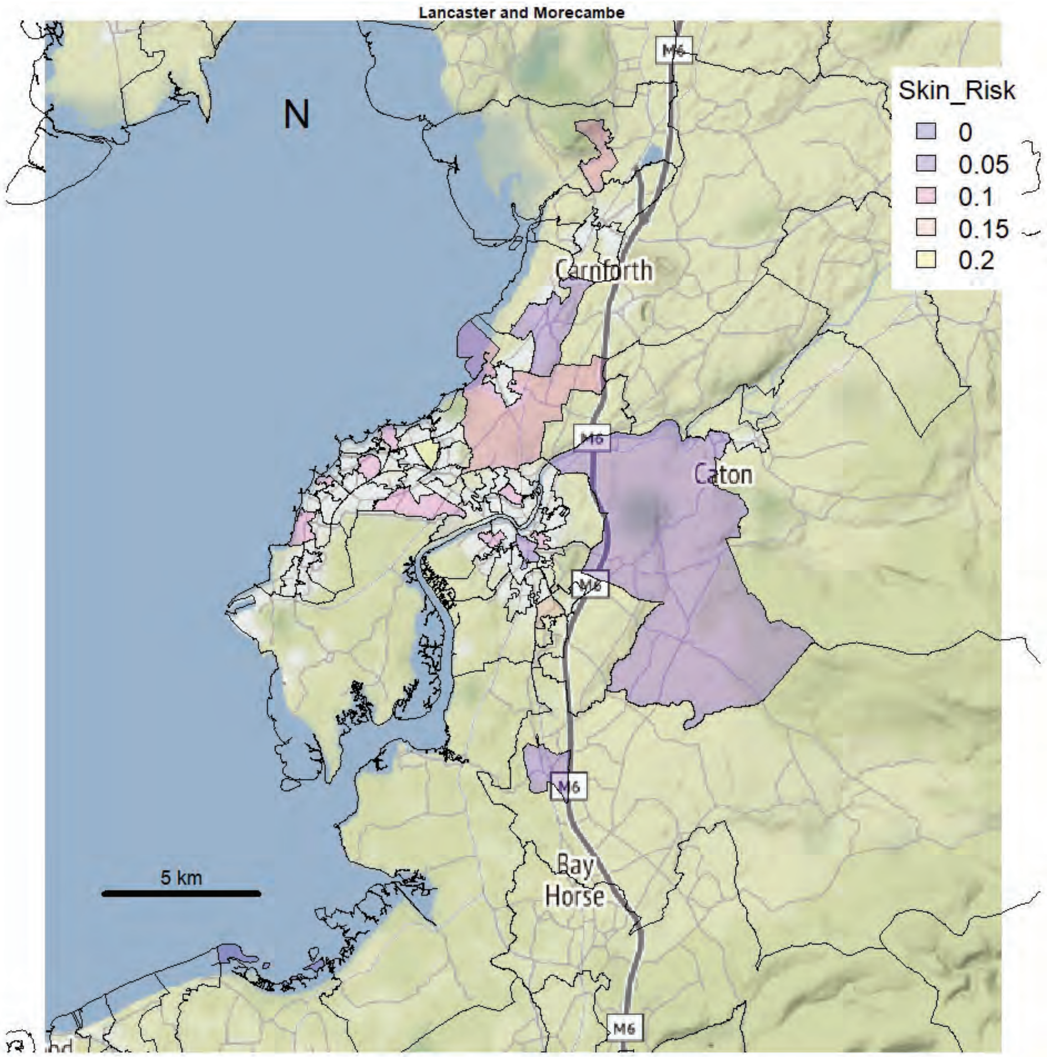
Lancaster and Morecambe
N
Skin_Risk
0
0.05
0.1
0.15
0.2
Carnforth
Caton
M6
Bay Horse
5 km

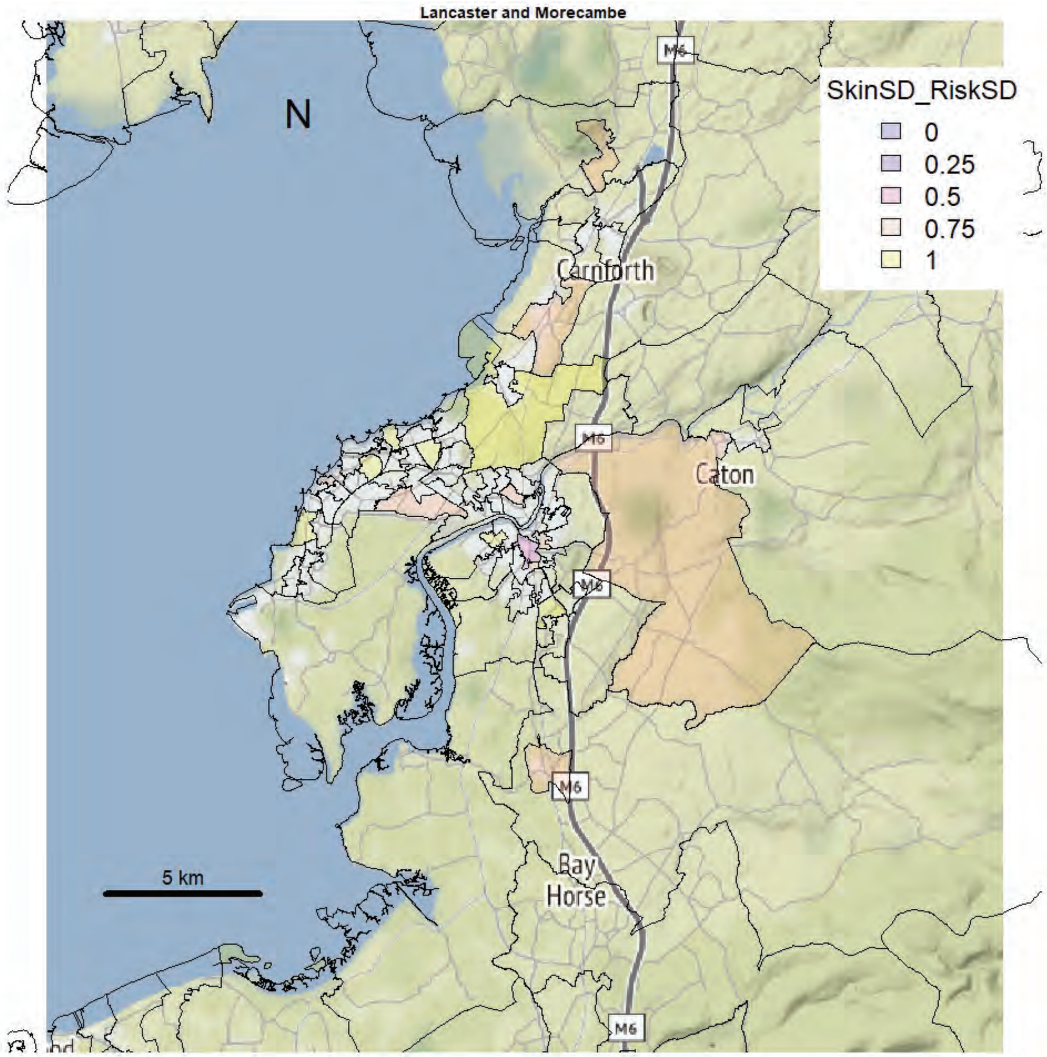
Lancaster and Morecambe
N
SkinSD_RiskSD
0
0.25
0.5
0.75
1
Carnforth
Caton
M6
Bay Horse
5 km

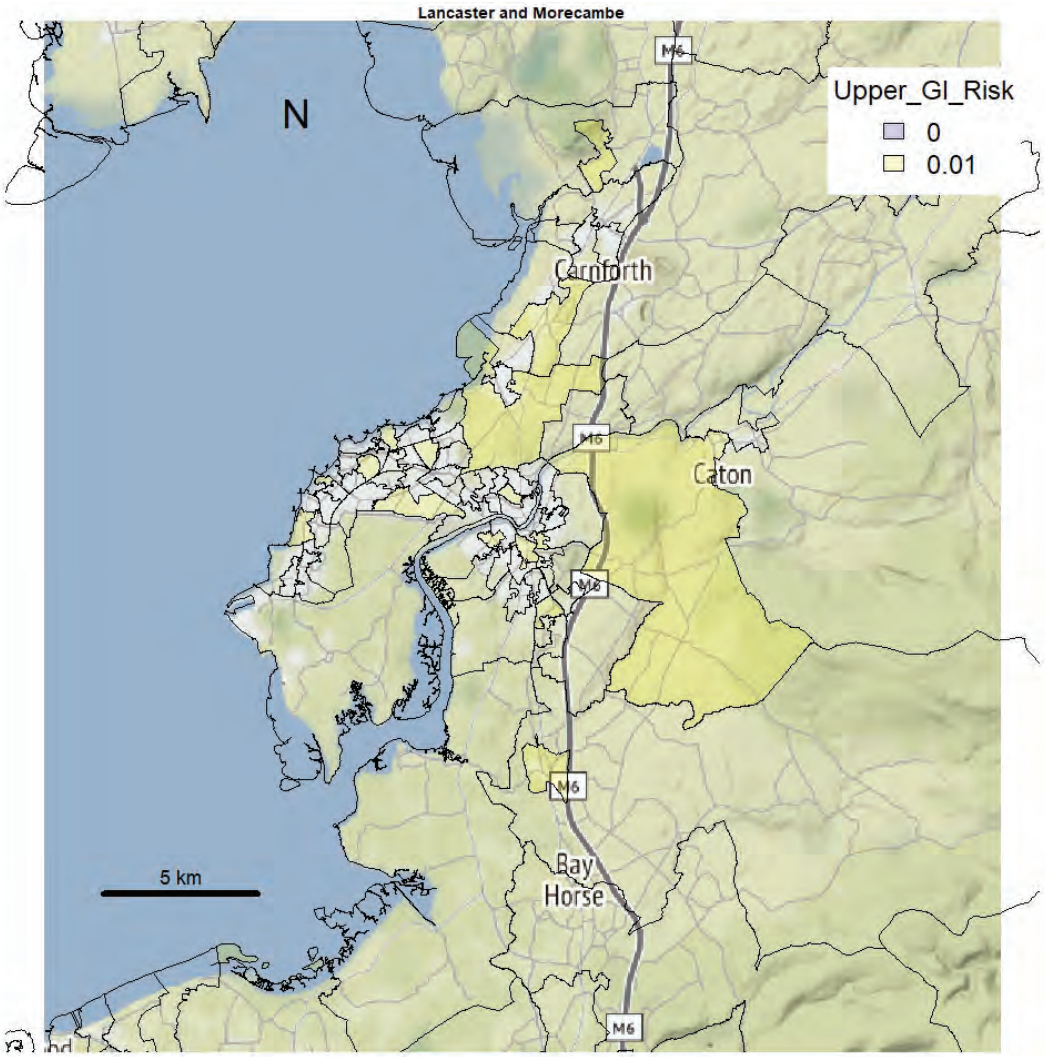
Lancaster and Morecambe
N
Upper_GI_Risk
0
0.01
Carnforth
Caton
Bay Horse
M6
5 km

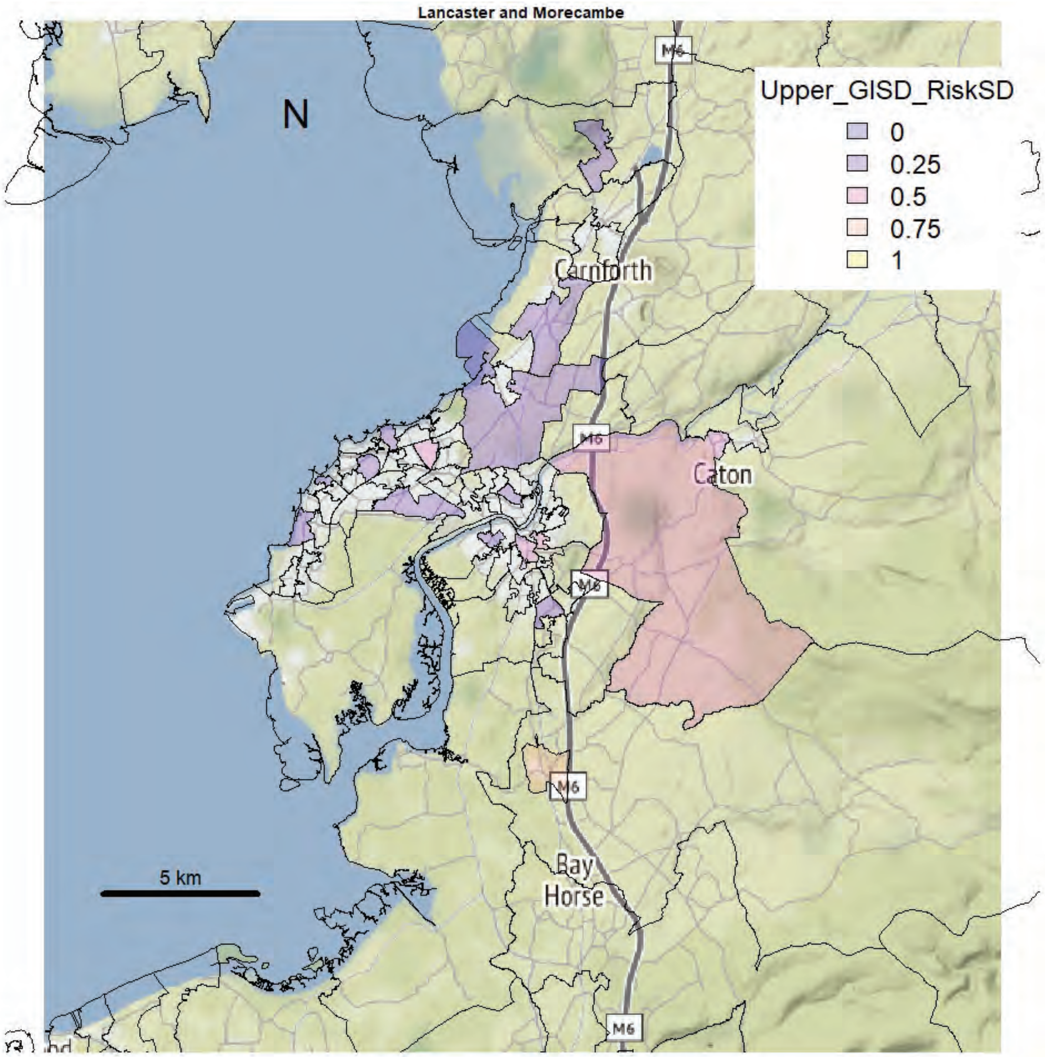
Lancaster and Morecambe
N
Upper_GISD_RiskSD
0
0.25
0.5
0.75
1
Carnforth
Caton
Bay Horse
M6
5 km

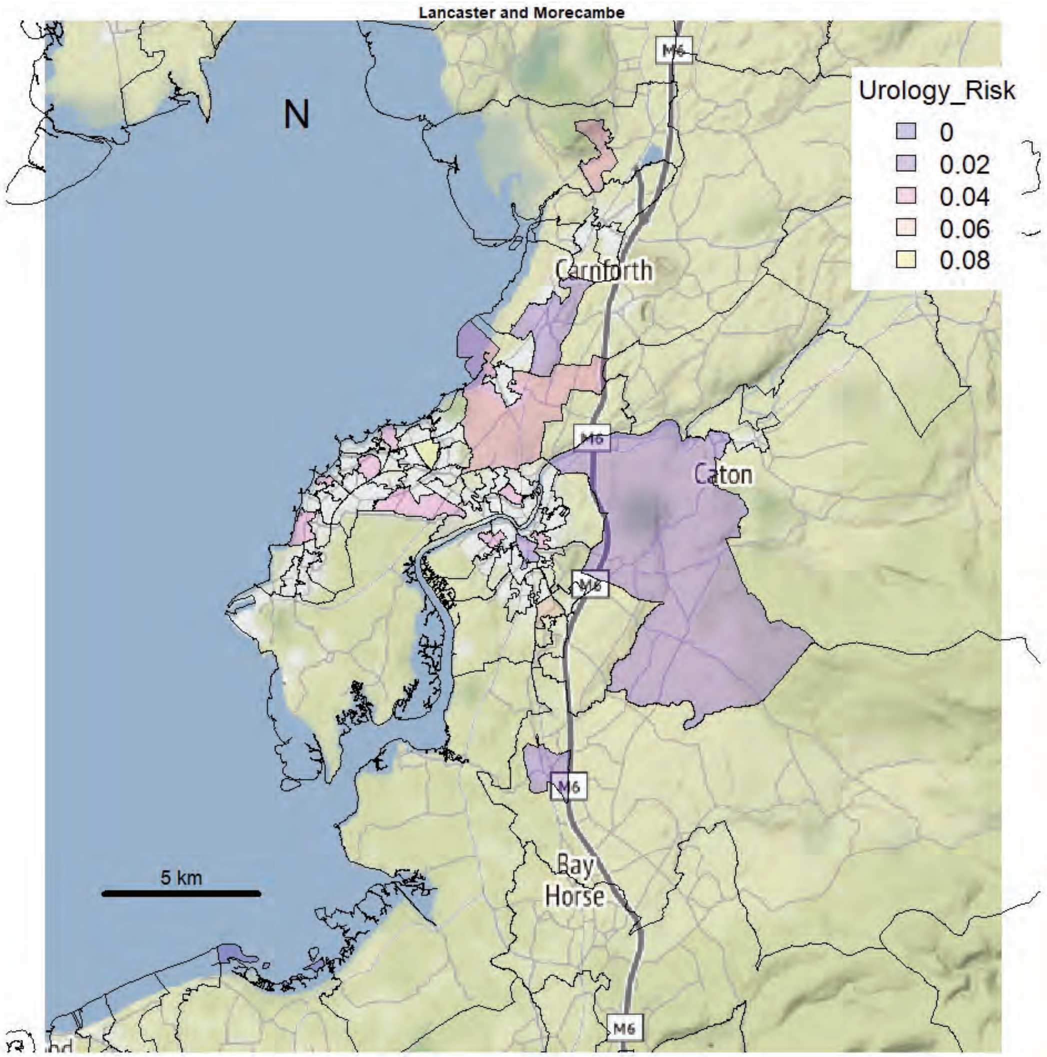
Lancaster and Morecambe
N
Urology_Risk
0
0.02
0.04
0.06
0.08
Carnforth
Caton
Bay
Horse
M6
5 km

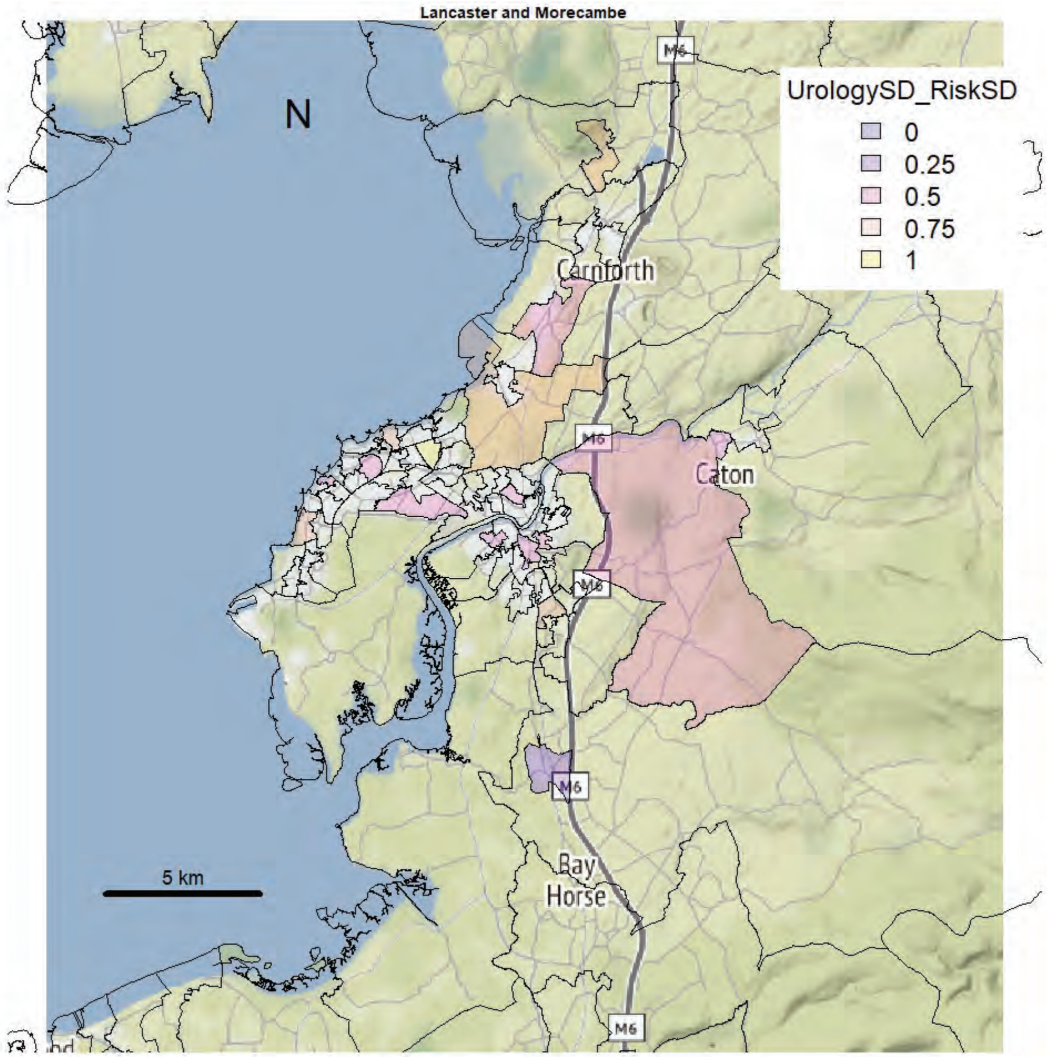
Lancaster and Morecambe
N
UrologySD_RiskSD
0
0.25
0.5
0.75
1
Carnforth
Caton
Bay
Horse
M6
5 km

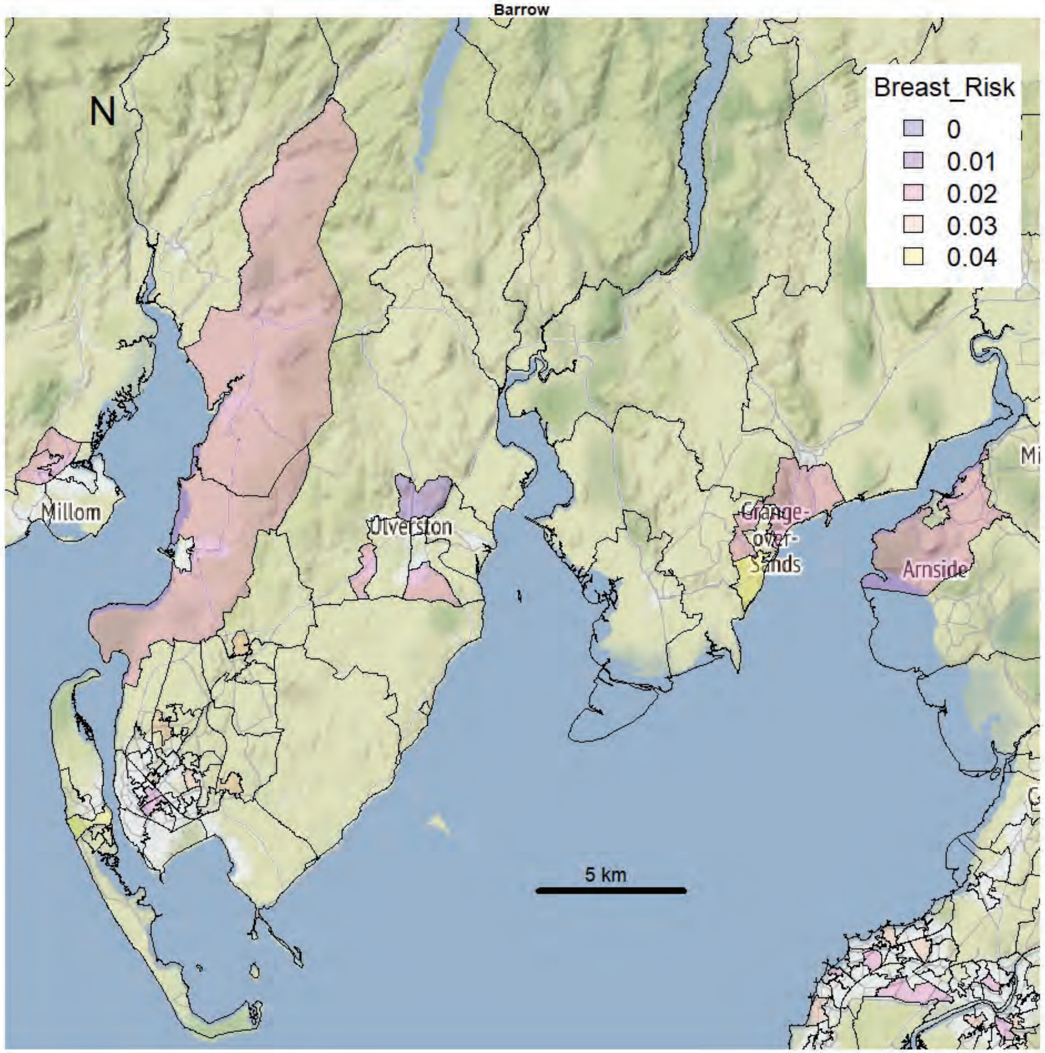
Barrow
N
Breast_Risk
0
0.01
0.02
0.03
0.04
Millom
Ulverston
Grange-over-Sands
Arnside
5 km

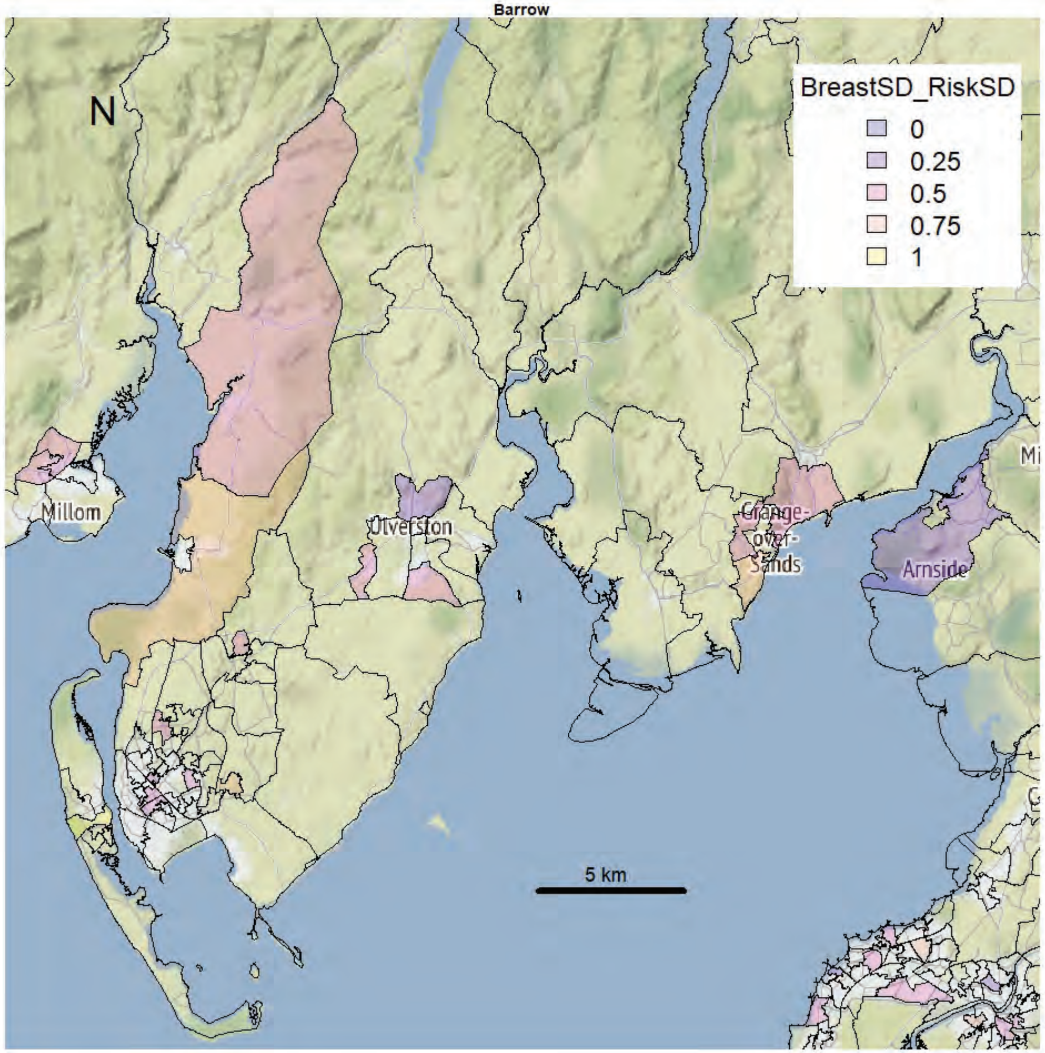
Barrow
N
BreastSD_RiskSD
0
0.25
0.5
0.75
1
Millom
Ulverston
Grange-over-Sands
Arnside
5 km

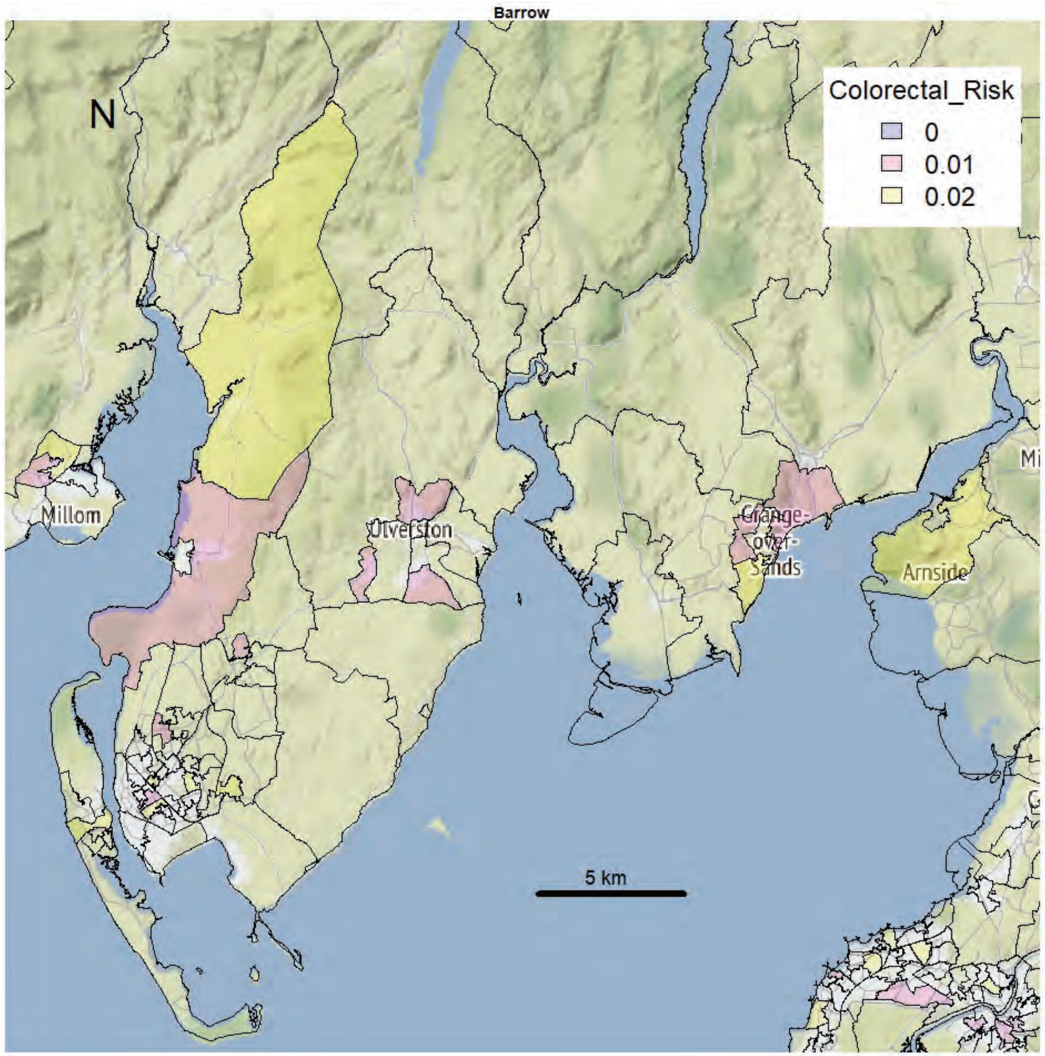
Barrow
N
Colorectal_Risk
0
0.01
0.02
Millom
Ulverston
Grange-over-Sands
Arnside
5 km

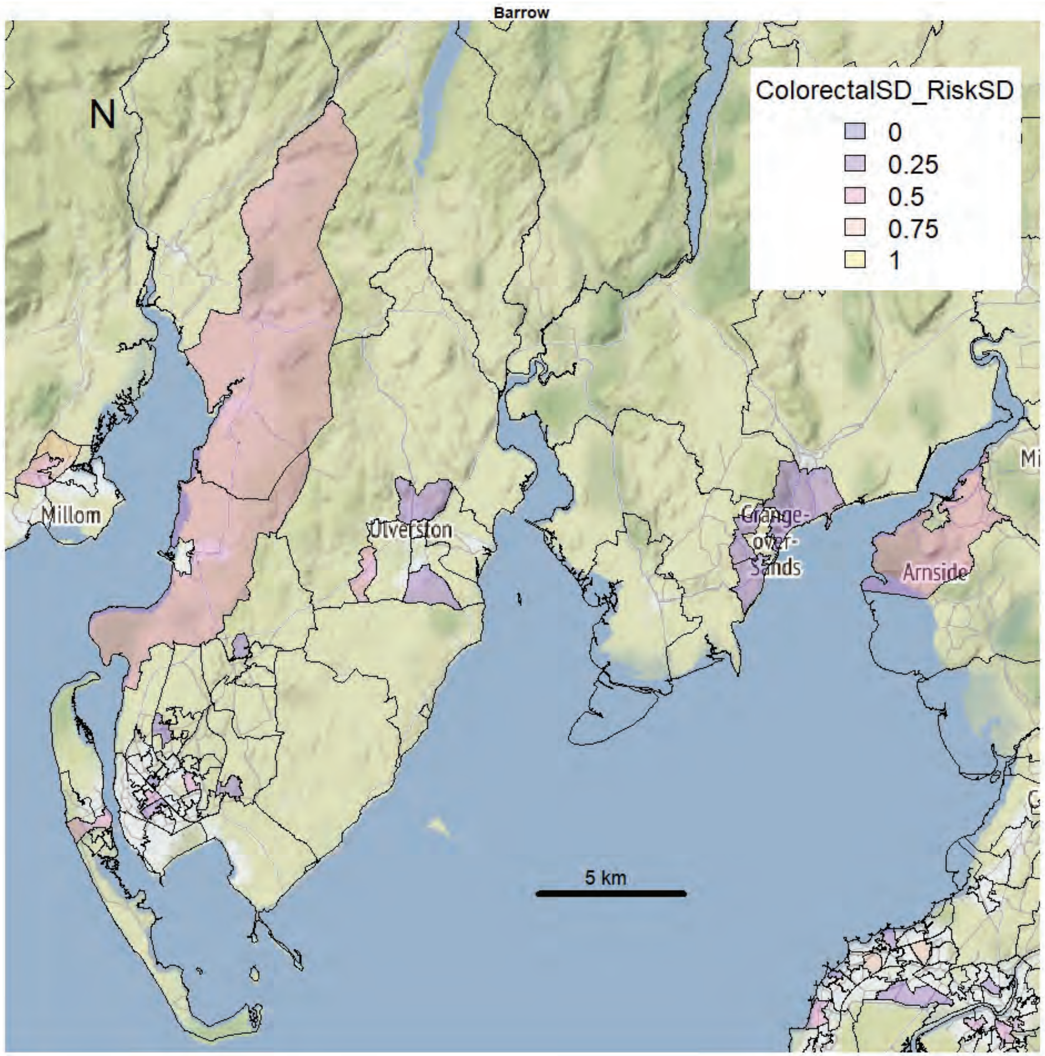
Barrow
N
ColorectalSD_RiskSD
0
0.25
0.5
0.75
1
Millom
Ulverston
Grange-over-Sands
Arnside
5 km

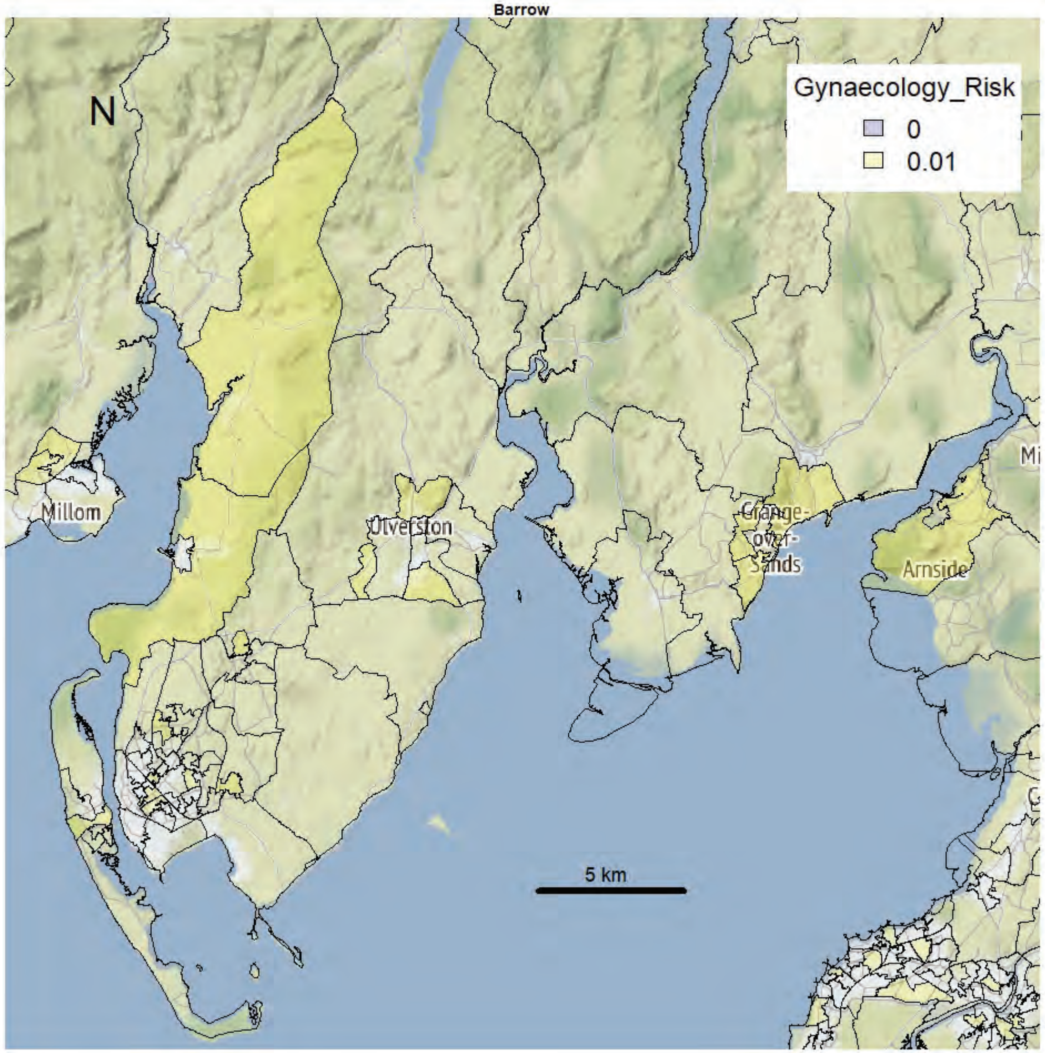
Barrow
Gynaecology_Risk
0
0.01
N
Millom
Ulverston
Grange-over-Sands
Arnside
5 km

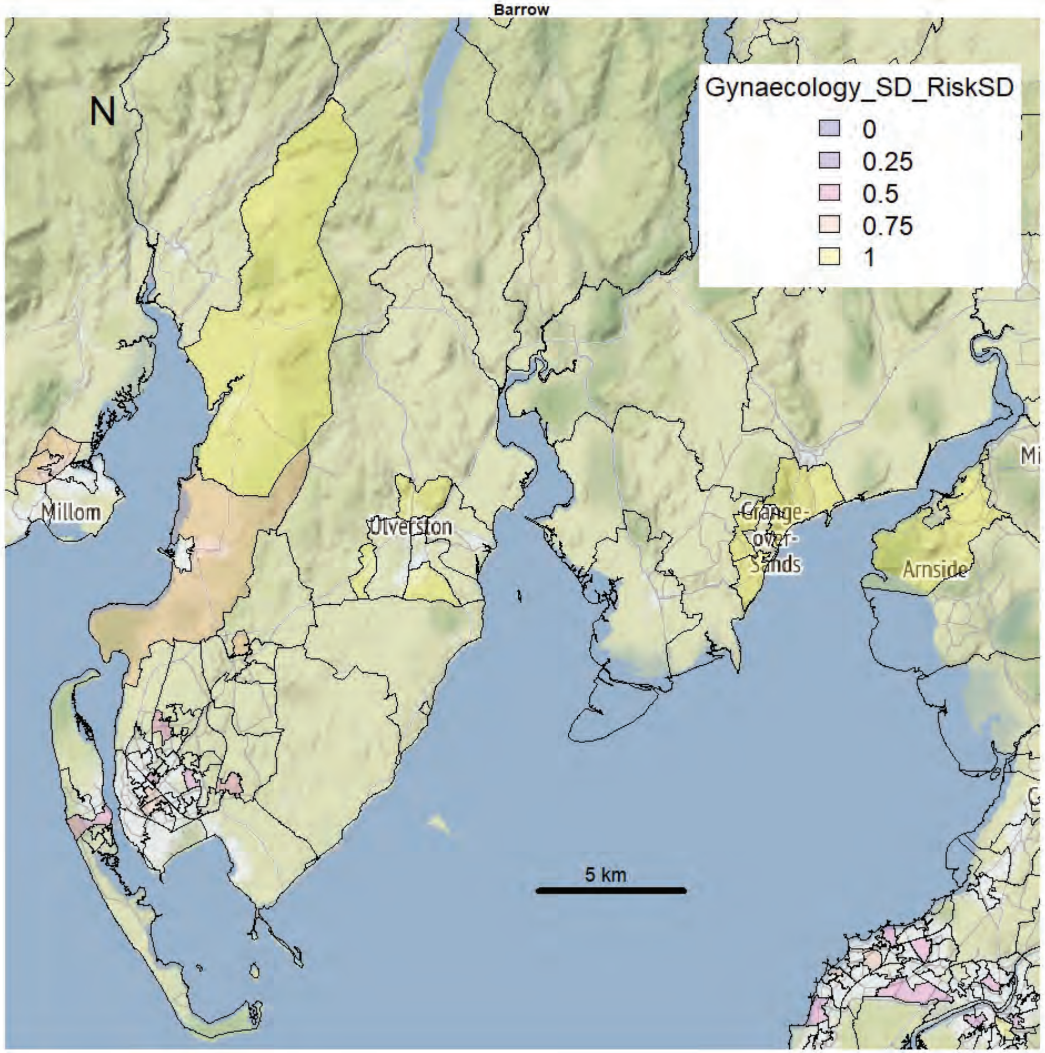
Barrow
Gynaecology_SD_RiskSD
0
0.25
0.5
0.75
1
N
Millom
Ulverston
Grange-over-Sands
Arnside
5 km

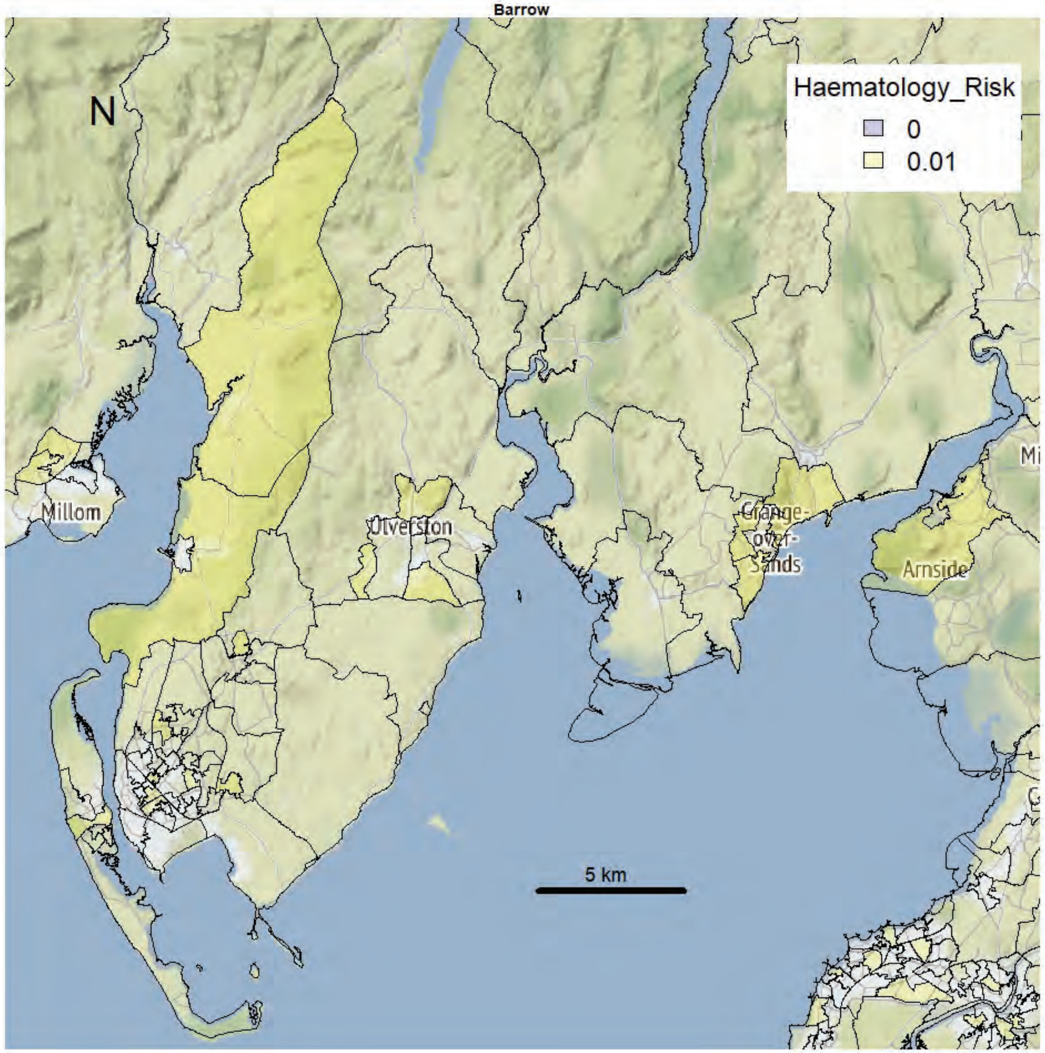
Barrow
N
Haematology_Risk
0
0.01
Millom
Ulverston
Grange-over-Sands
Arnside
5 km

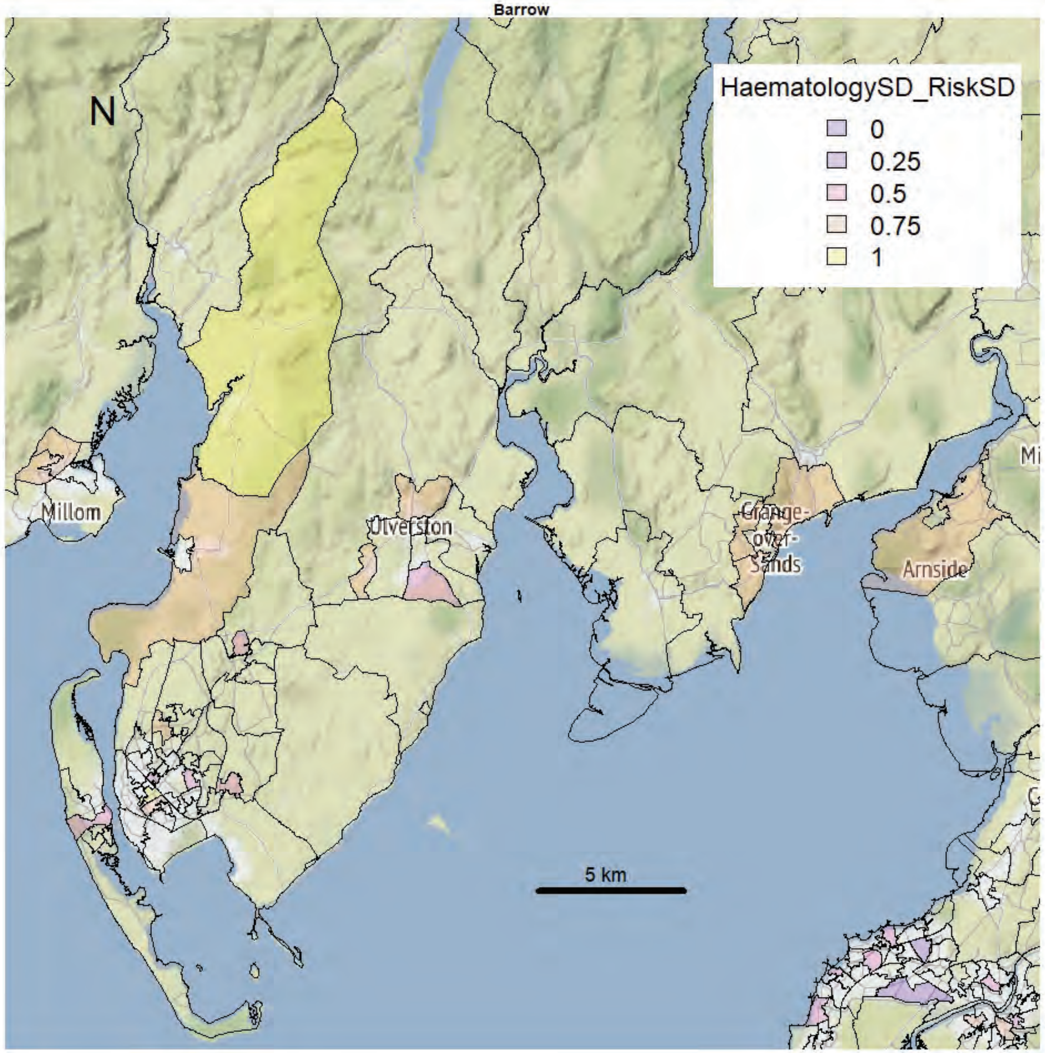
Barrow
N
HaematologySD_RiskSD
0
0.25
0.5
0.75
1
Millom
Ulverston
Grange-over-Sands
Arnside
5 km

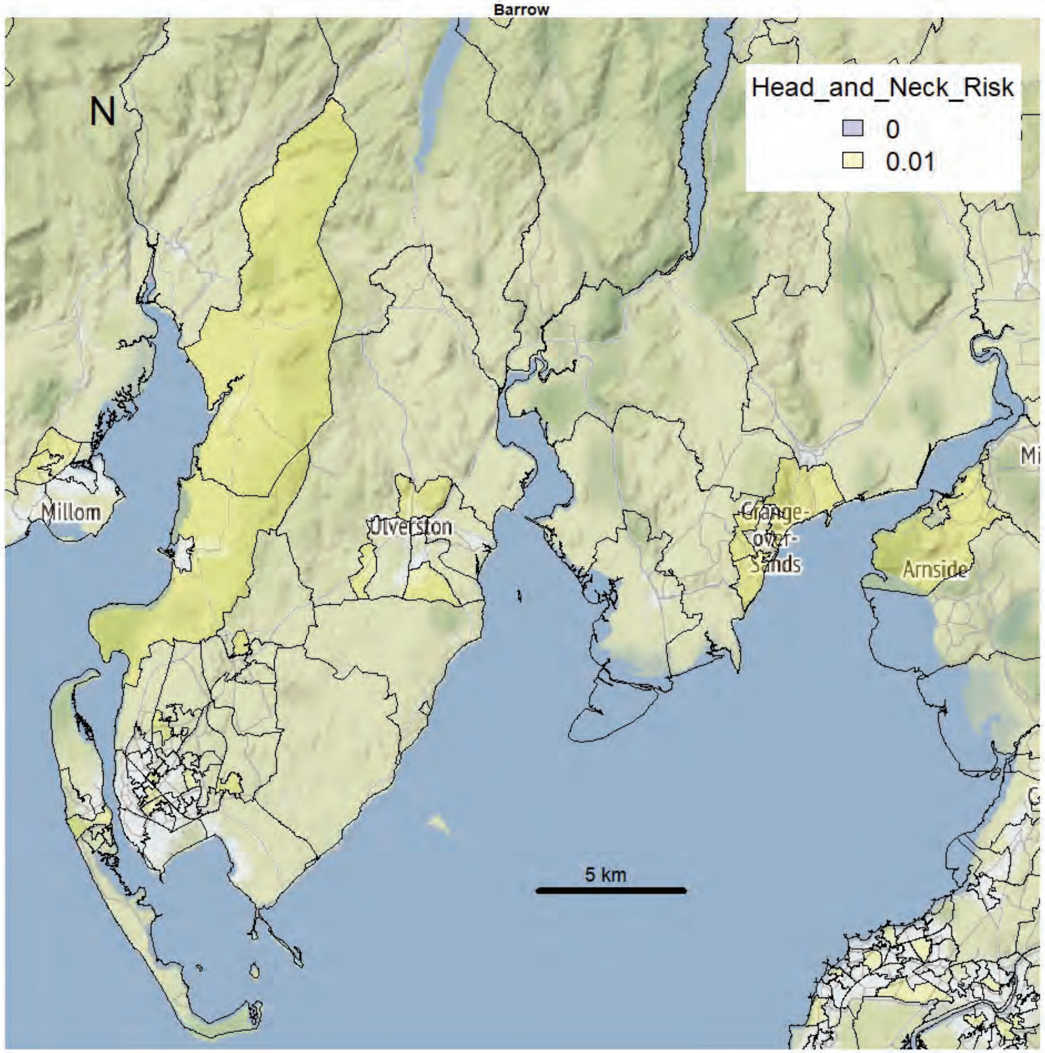
Barrow
N
Head_and_Neck_Risk
0
0.01
Millom
Ulverston
Grange-over-Sands
Arnside
5 km

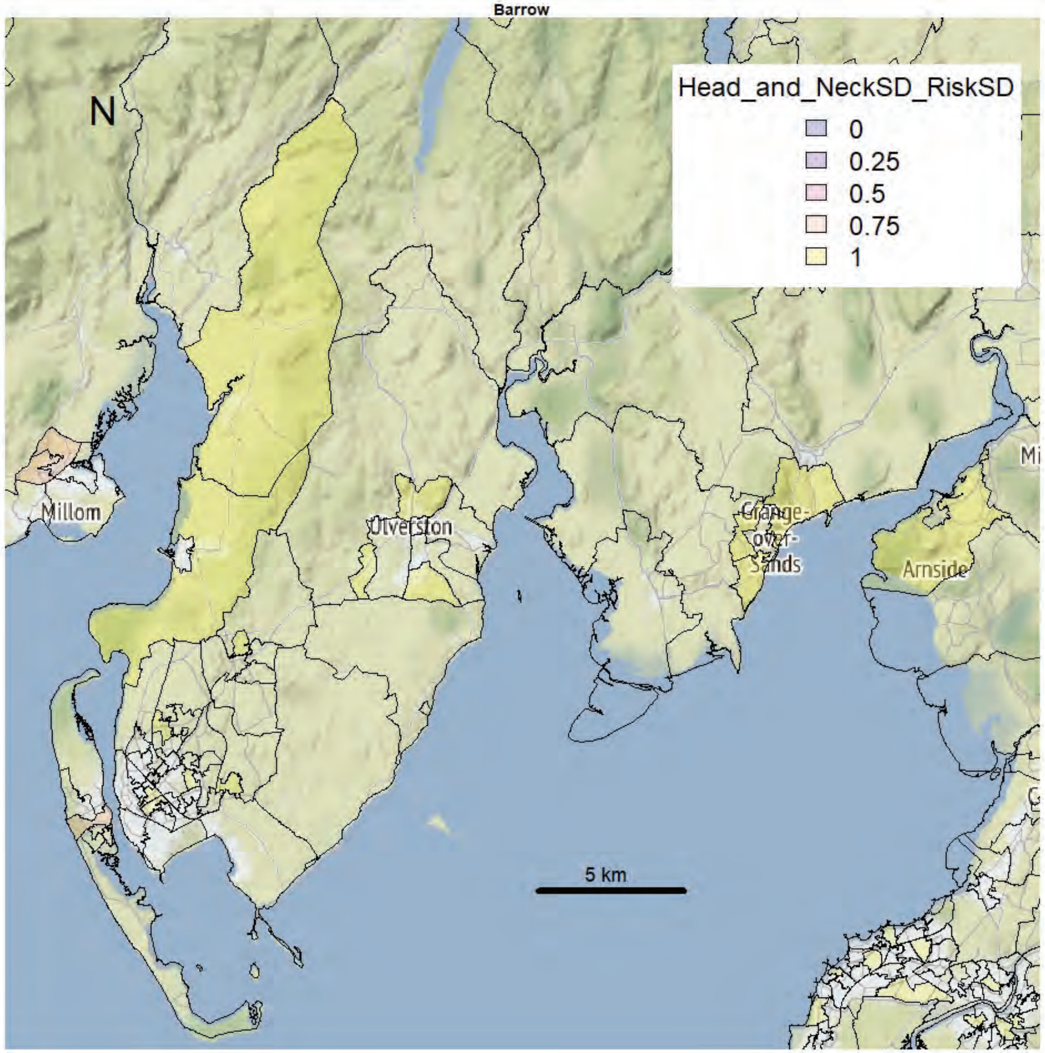
Barrow
N
Head_and_NeckSD_RiskSD
0
0.25
0.5
0.75
1
Millom
Ulverston
Grange-over-Sands
Arnside
5 km

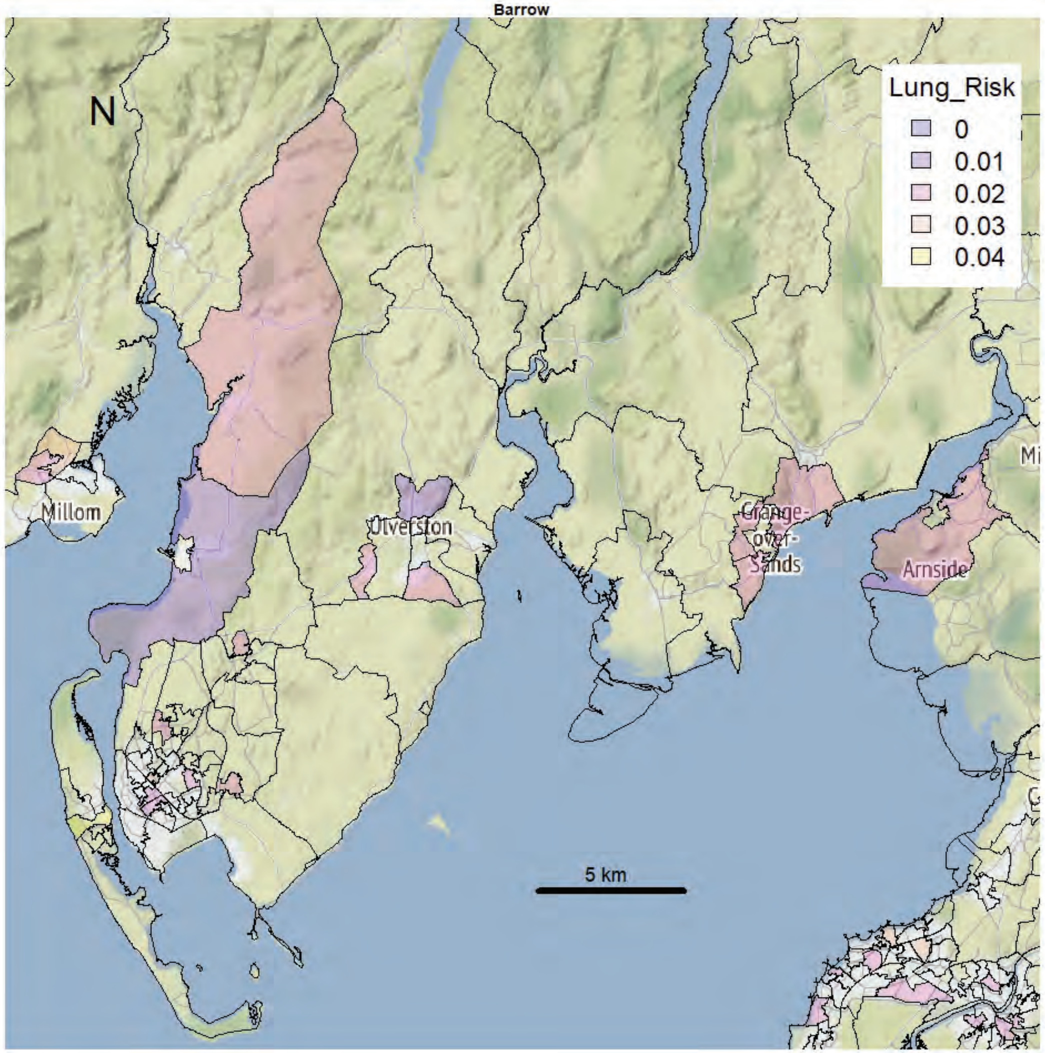
Barrow
N
Lung_Risk
0
0.01
0.02
0.03
0.04
Millom
Ulverston
Grange-over-Sands
Arnside
5 km

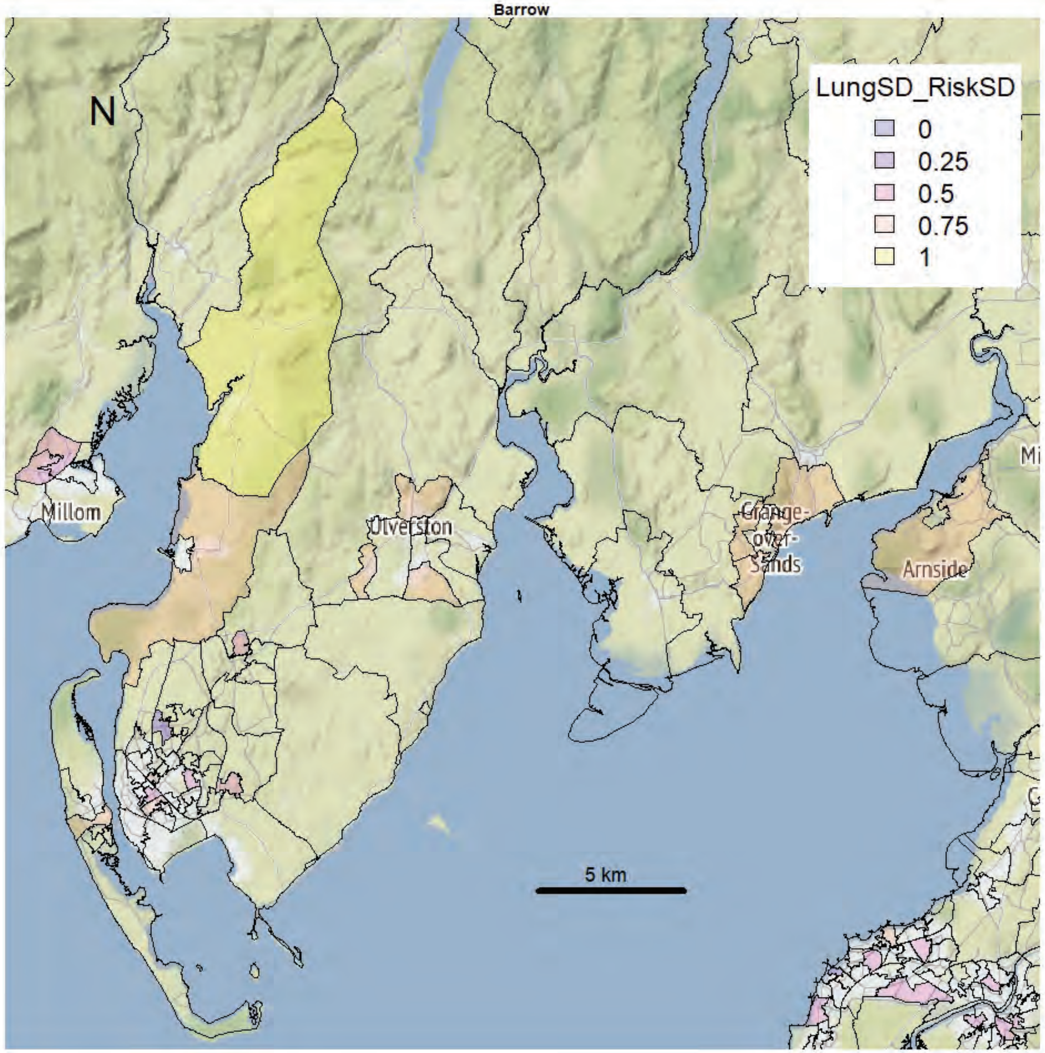
Barrow
N
LungSD_RiskSD
0
0.25
0.5
0.75
1
Millom
Ulverston
Grange-over-Sands
Arnside
5 km

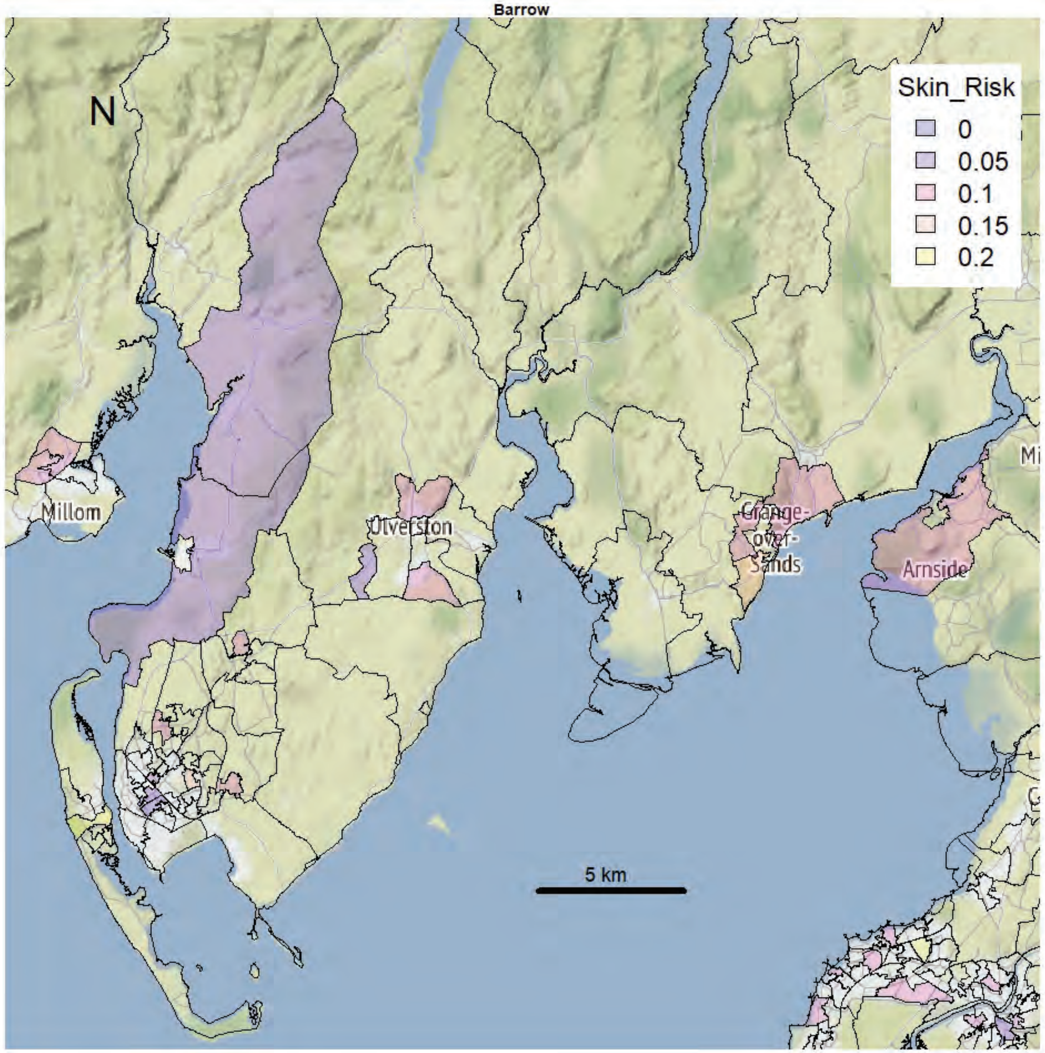
Barrow
N
Skin_Risk
0
0.05
0.1
0.15
0.2
Millom
Ulverston
Grange-over-Sands
Arnside
5 km

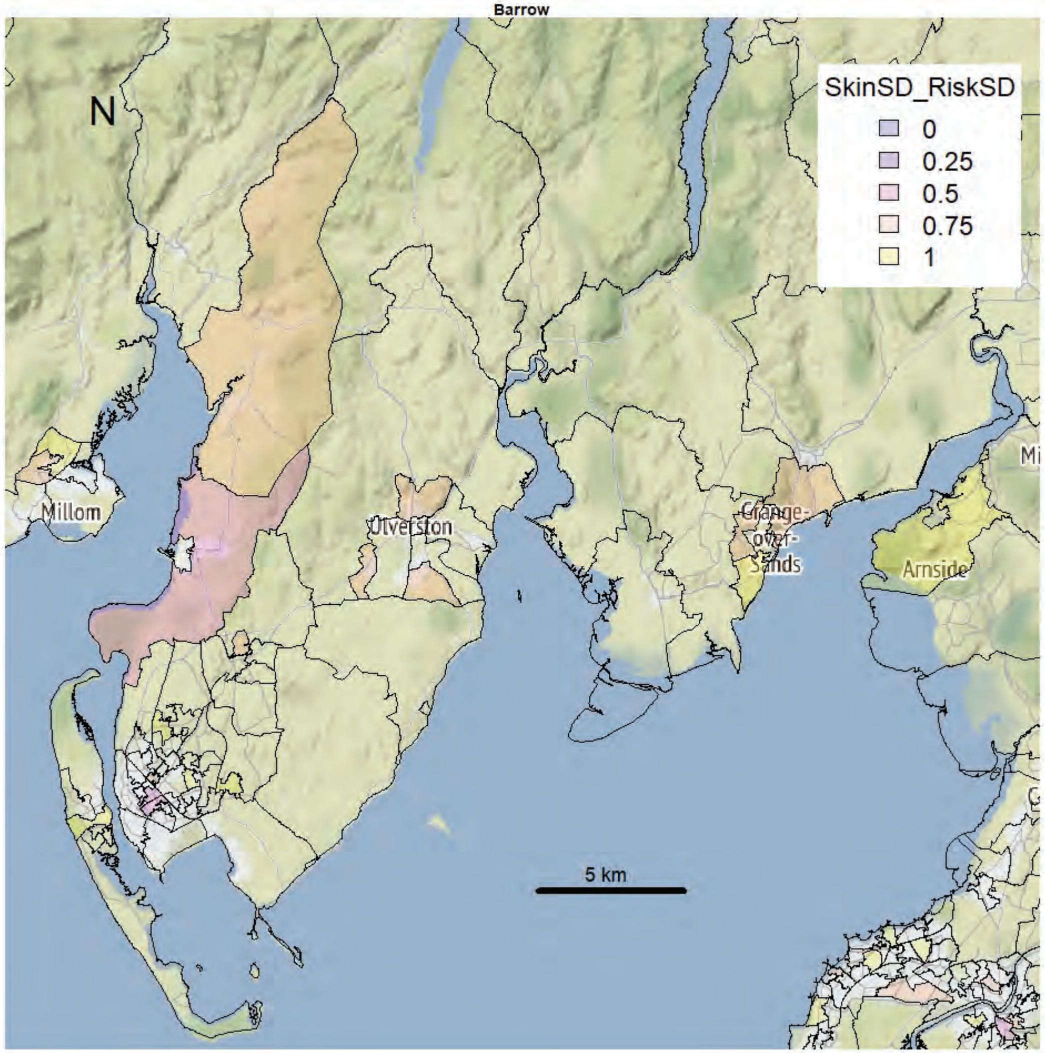
Barrow
N
SkinSD_RiskSD
0
0.25
0.5
0.75
1
Millom
Ulverston
Grange-over-Sands
Arnside
5 km

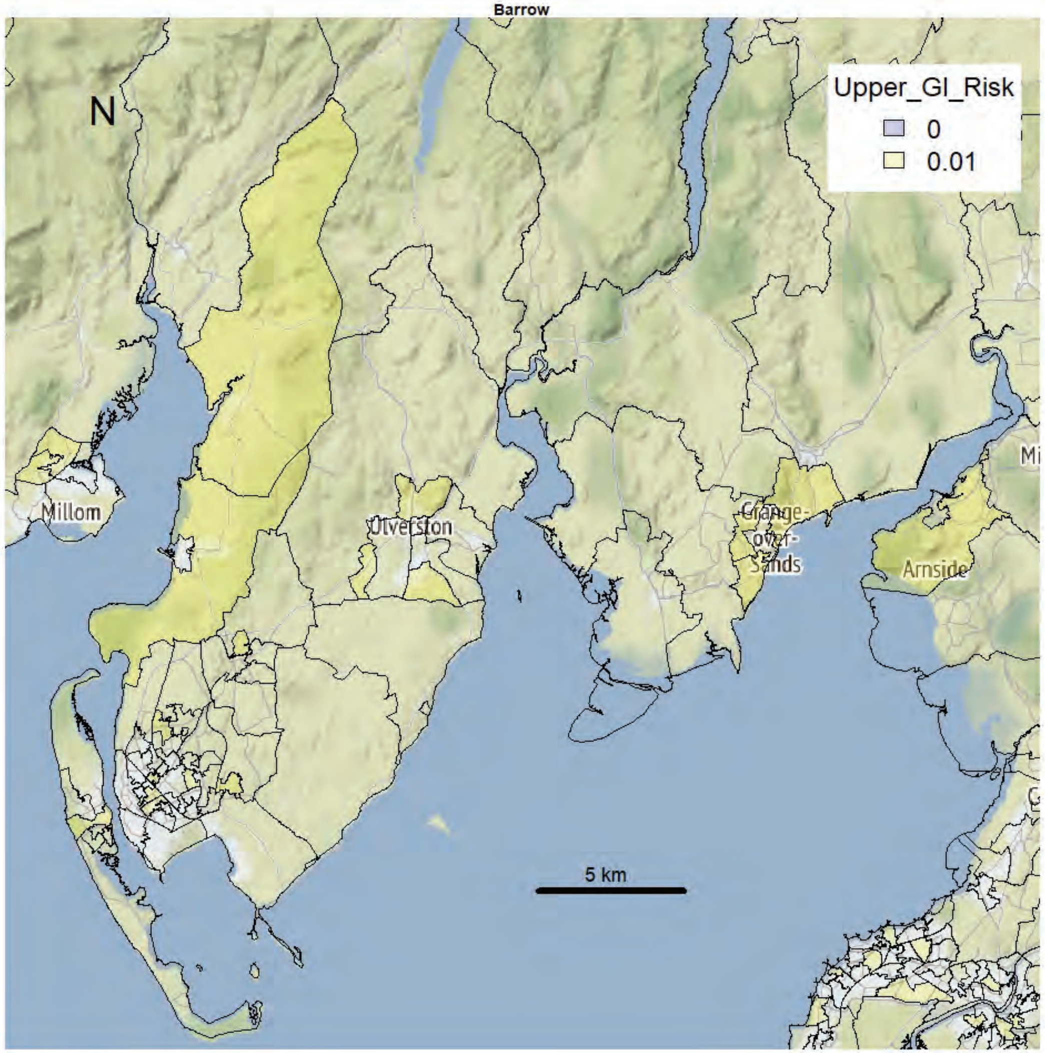

Barrow
N
Upper_GI_Risk
0
0.01
Millom
Ulverston
Grange-over-Sands
Arnside
5 km

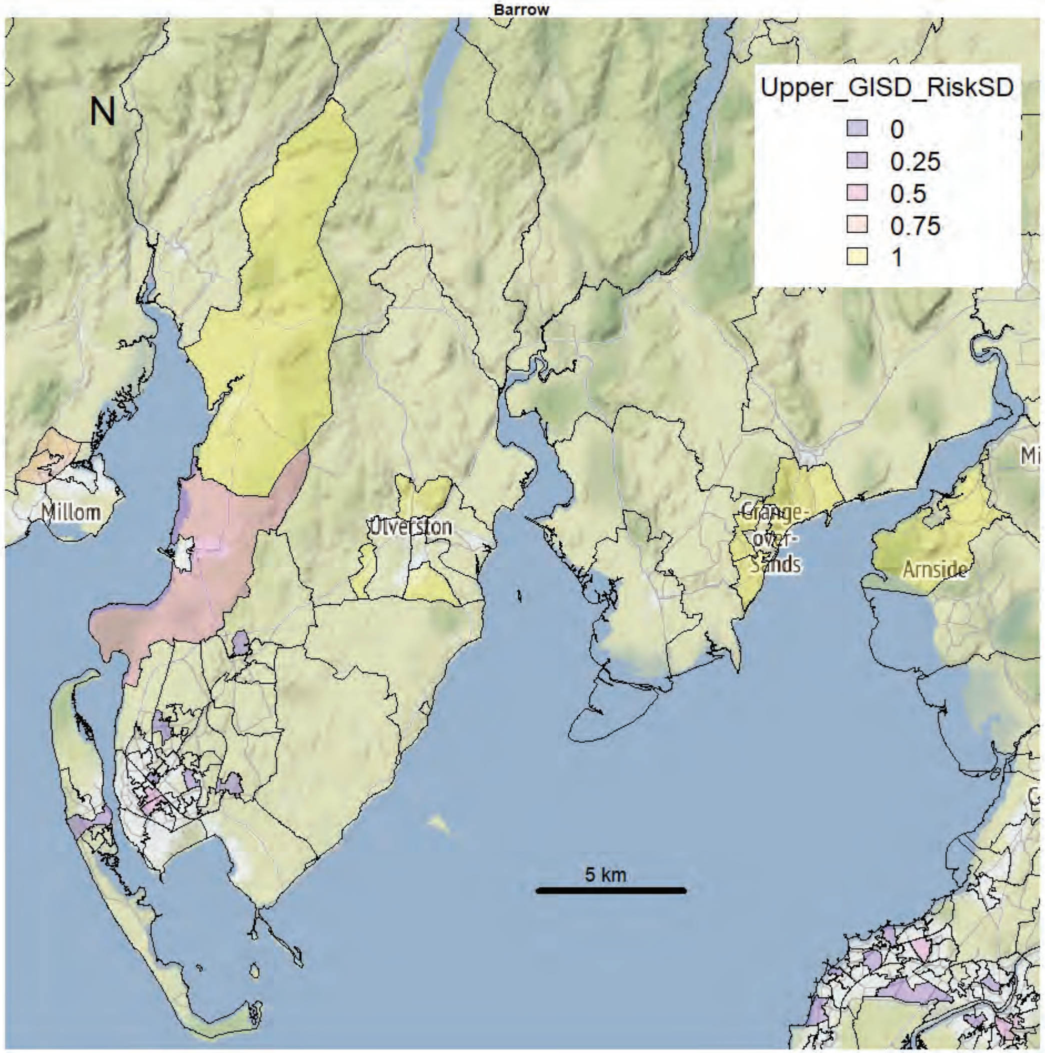

Barrow
N
Upper_GISD_RiskSD
0
0.25
0.5
0.75
1
Millom
Ulverston
Grange-over-Sands
Arnside
5 km

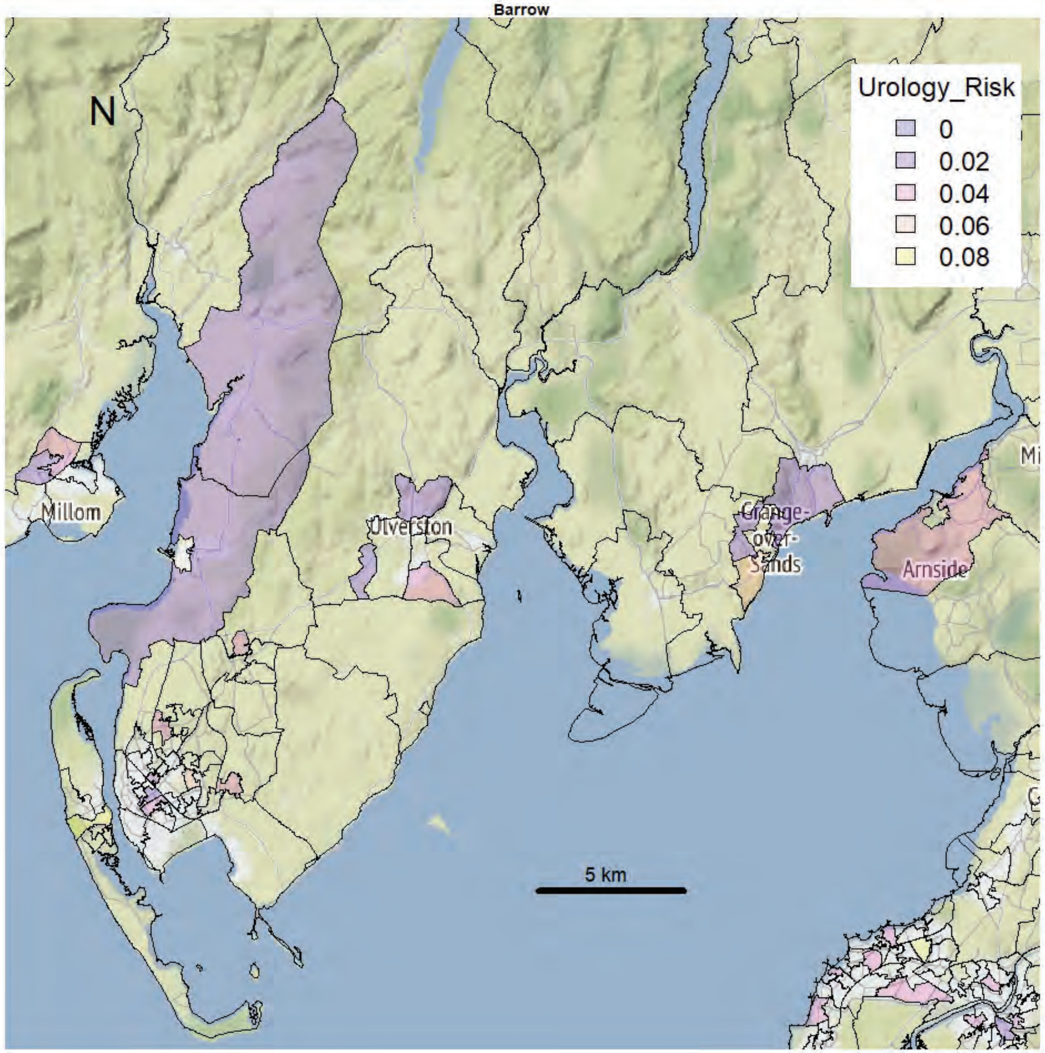
Barrow
N
Urology_Risk
0
0.02
0.04
0.06
0.08
Millom
Ulverston
Grange-over-Sands
Arnside
5 km

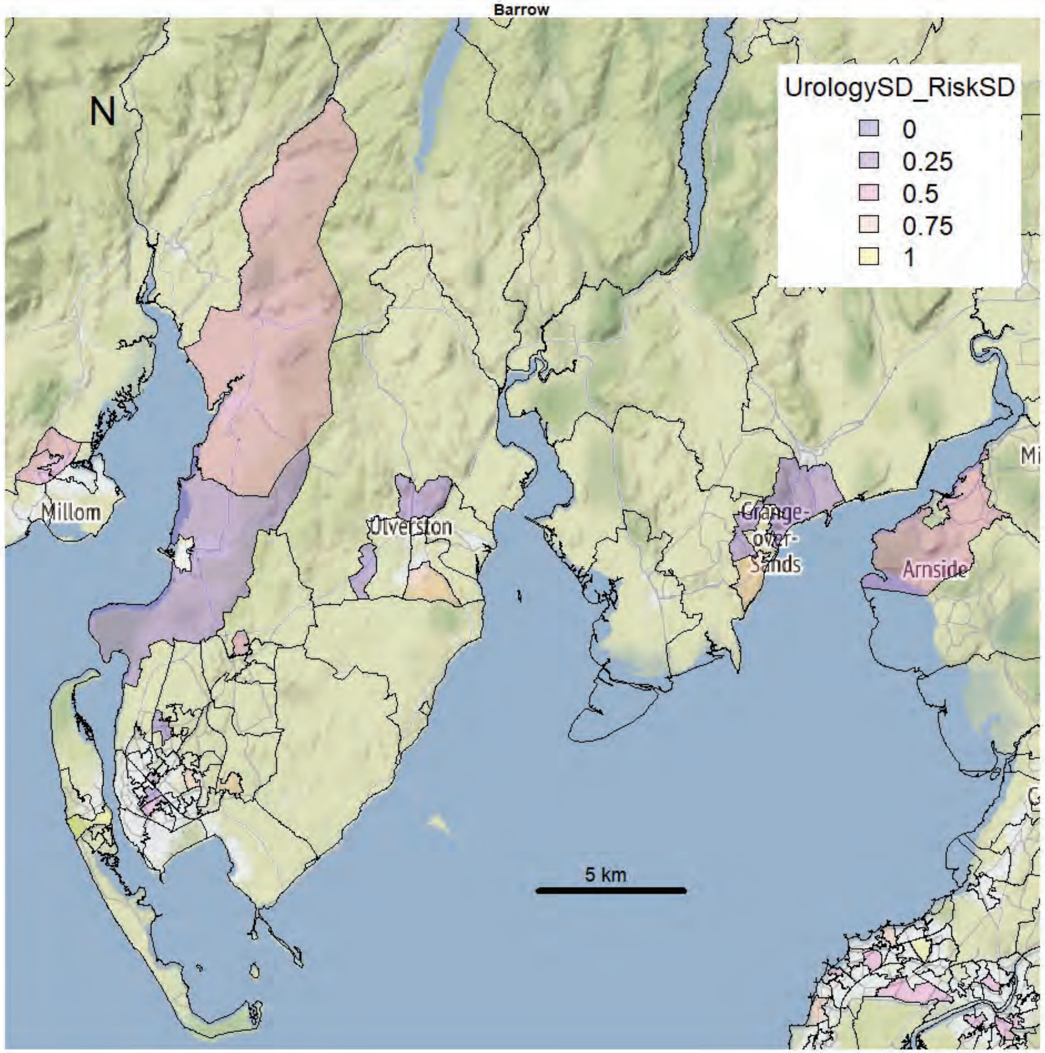
Barrow
N
UrologySD_RiskSD
0
0.25
0.5
0.75
1
Millom
Ulverston
Grange-over-Sands
Arnside
5 km

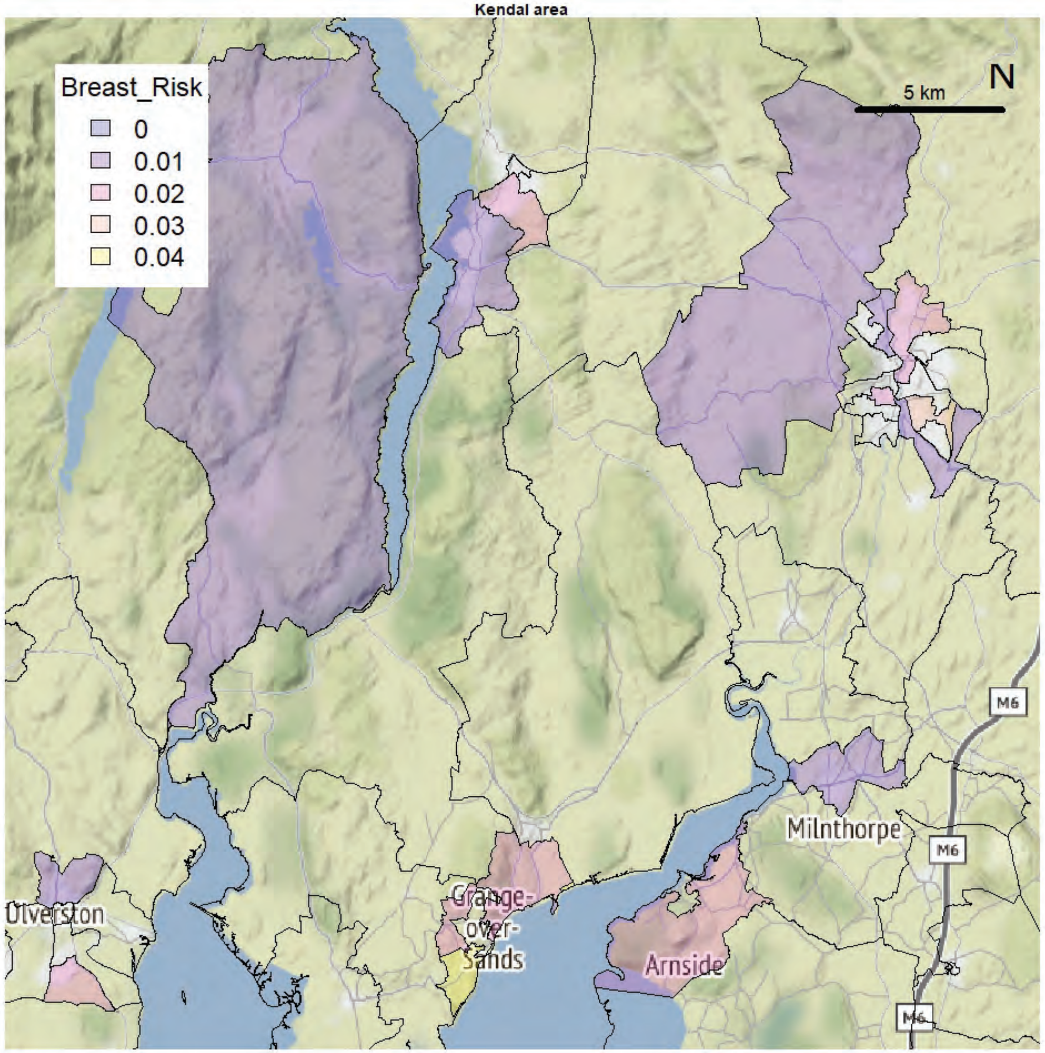
Kendal area
Breast_Risk
0
0.01
0.02
0.03
0.04
5 km
N
M6
Milnthorpe
Ulverston
Grange-over-Sands
Arnside

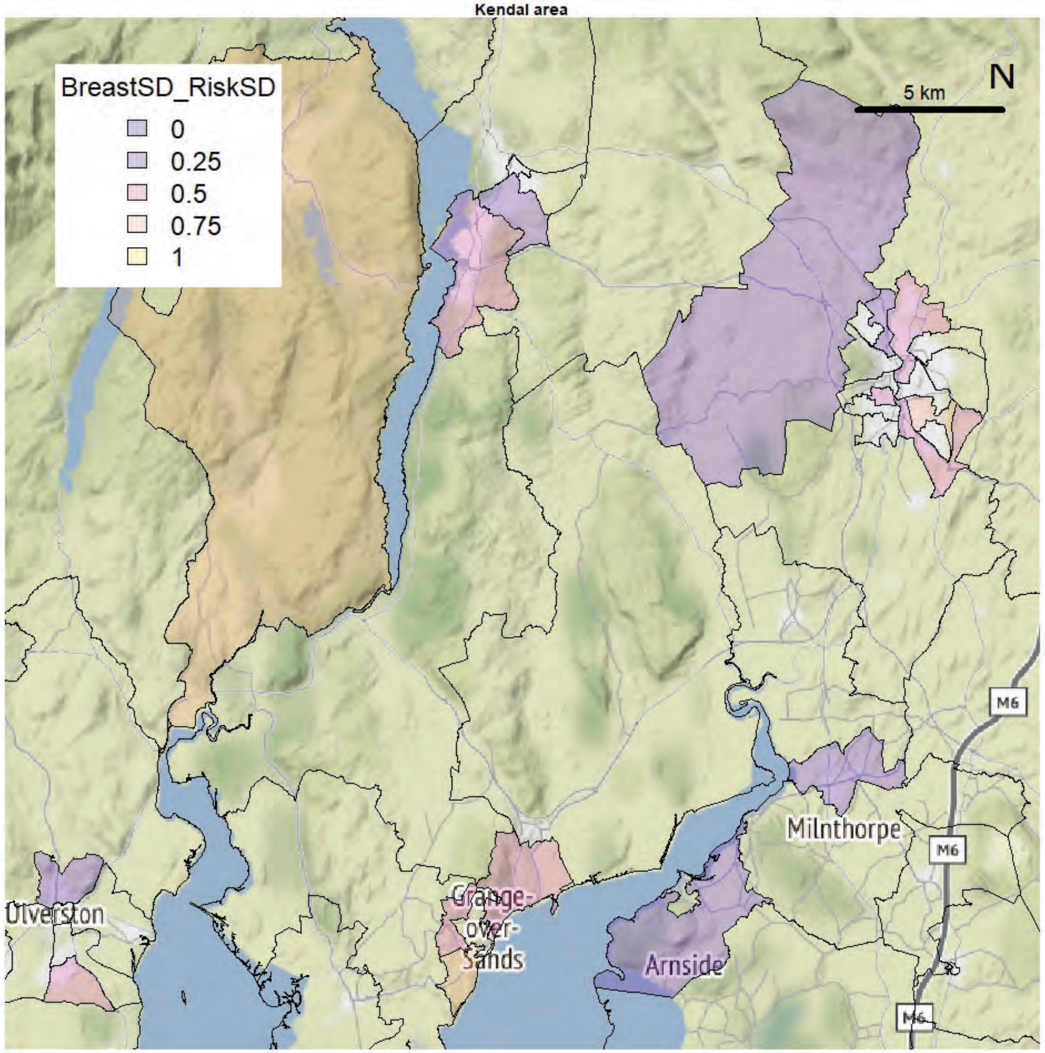
Kendal area
BreastSD_RiskSD
0
0.25
0.5
0.75
1
5 km
N
M6
Milnthorpe
Ulverston
Grange-over-Sands
Arnside

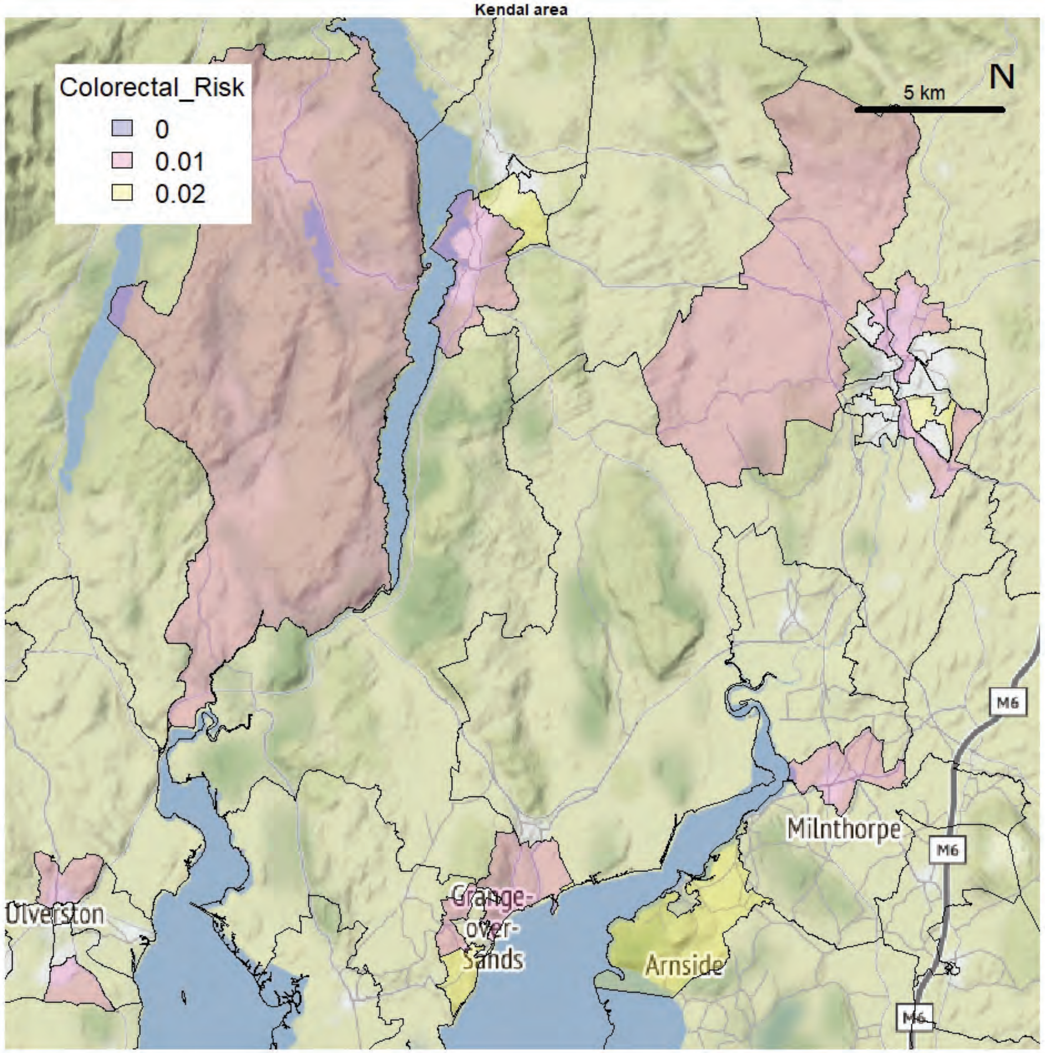
Kendal area
Colorectal_Risk
0
0.01
0.02
5 km
N
Ulverston
Grange-over-Sands
Arnside
Milnthorpe
M6

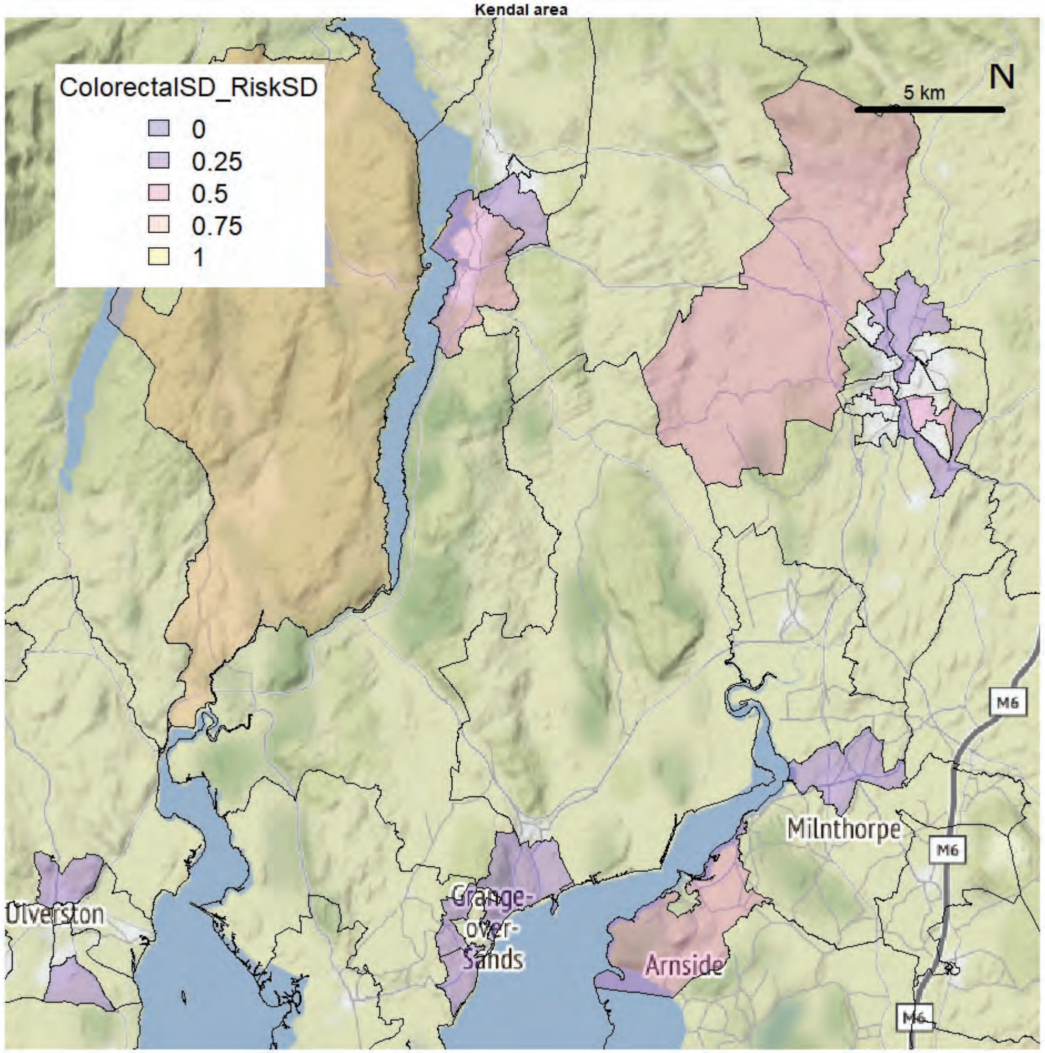
Kendal area
ColorectalSD_RiskSD
0
0.25
0.5
0.75
1
5 km
N
Ulverston
Grange-over-Sands
Arnside
Milnthorpe
M6

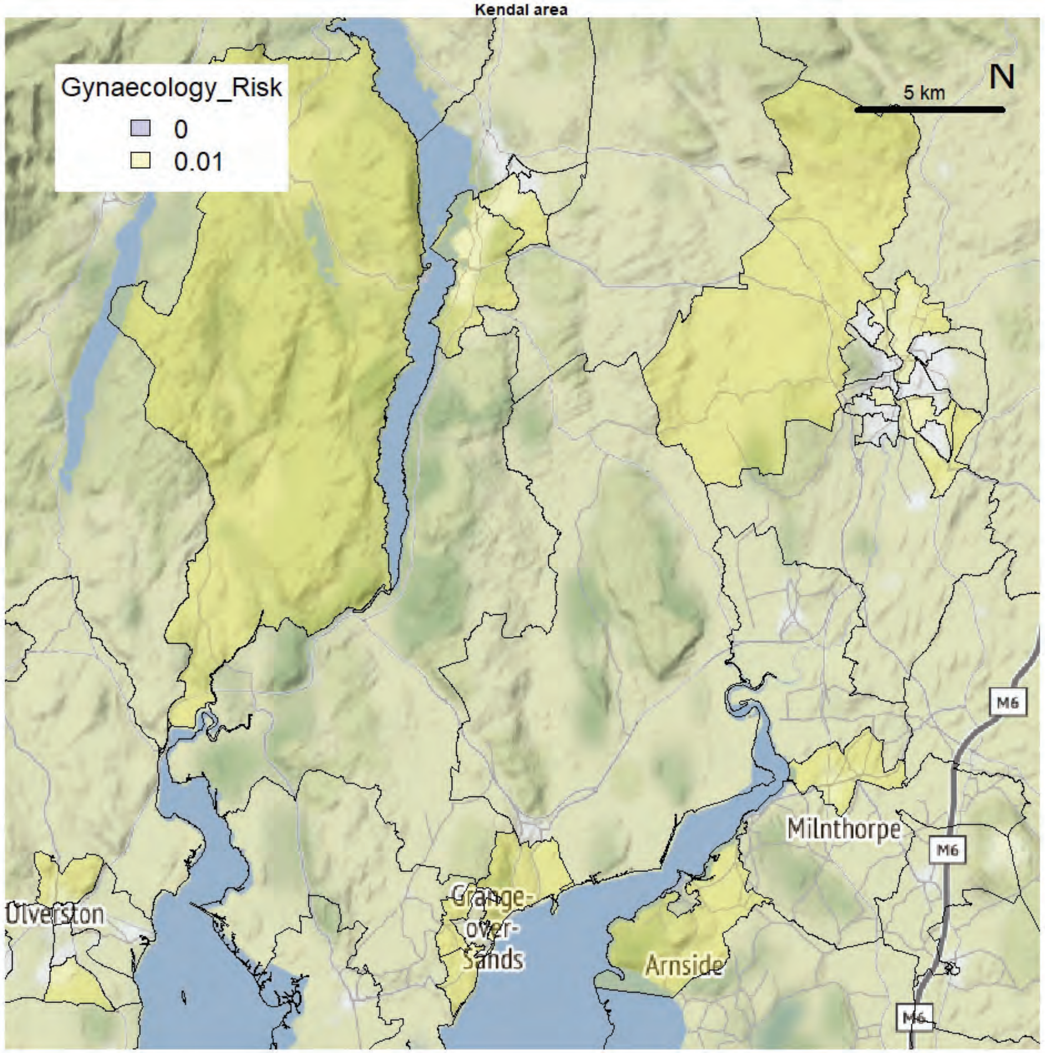
Kendal area
Gynaecology_Risk
0
0.01
5 km
N
M6
Milnthorpe
Ulverston
Grange-over-Sands
Arnside

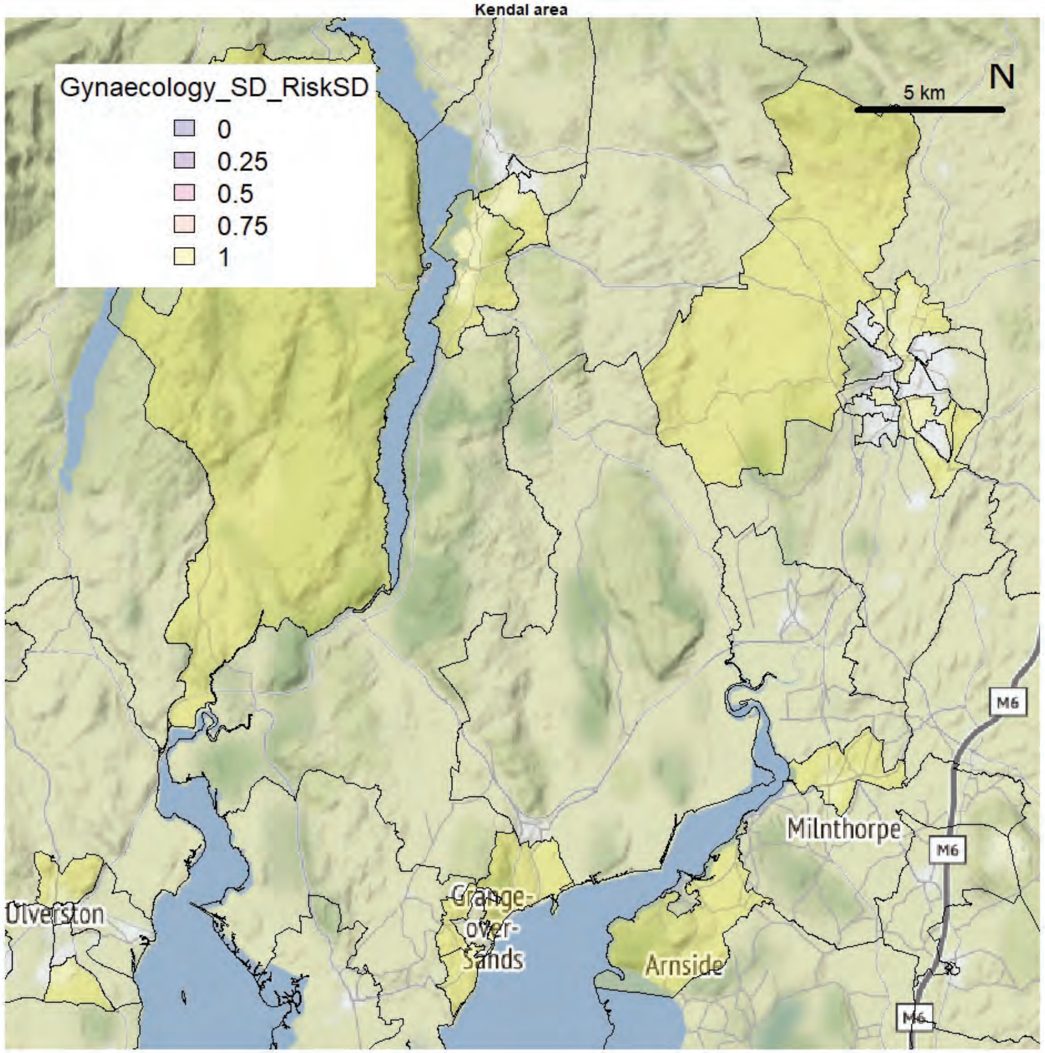
Kendal area
Gynaecology_SD_RiskSD
0
0.25
0.5
0.75
1
5 km
N
M6
Milnthorpe
Ulverston
Grange-over-Sands
Arnside

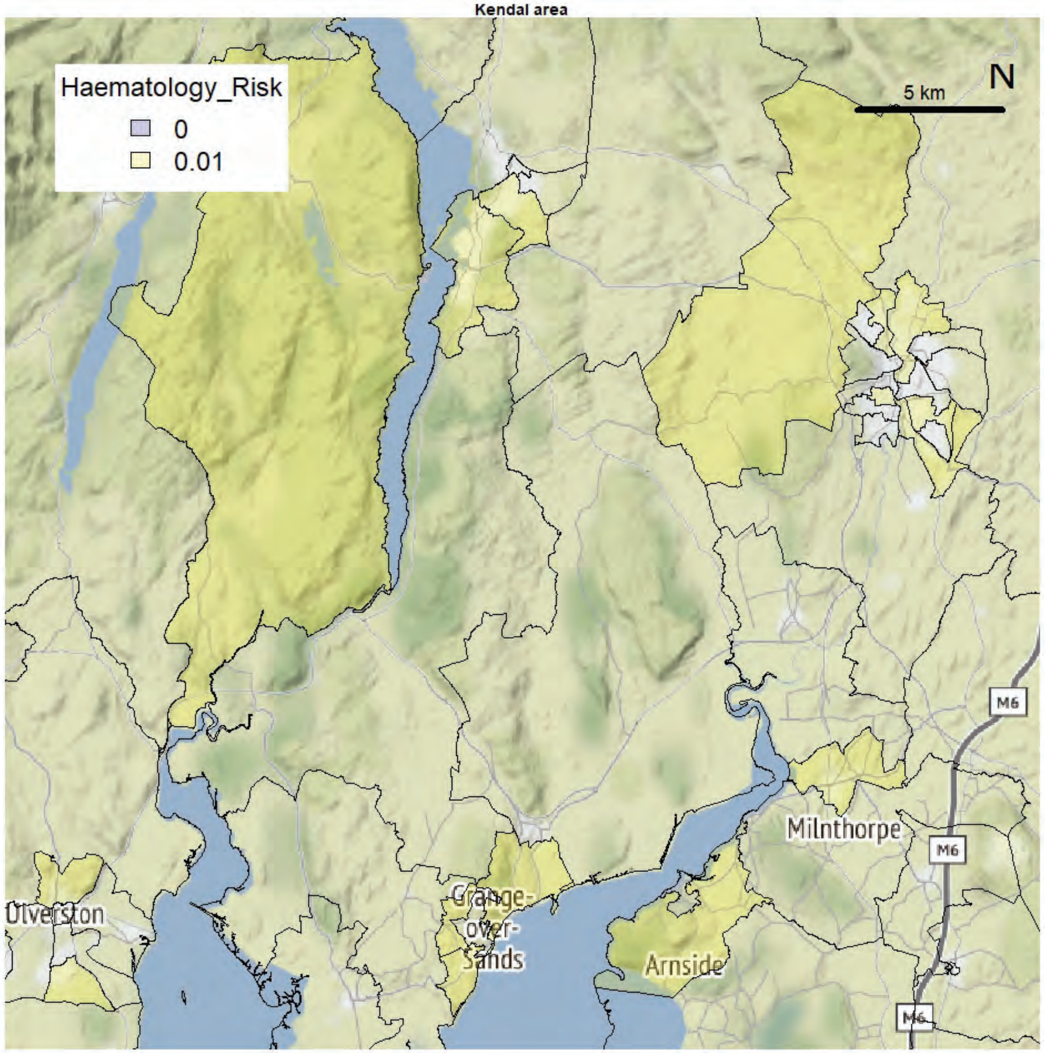

Kendal area
Haematology_Risk
0
0.01
5 km
N
M6
M6
M6
Milnthorpe
Ulverston
Grange-
over-
Sands
Arnside

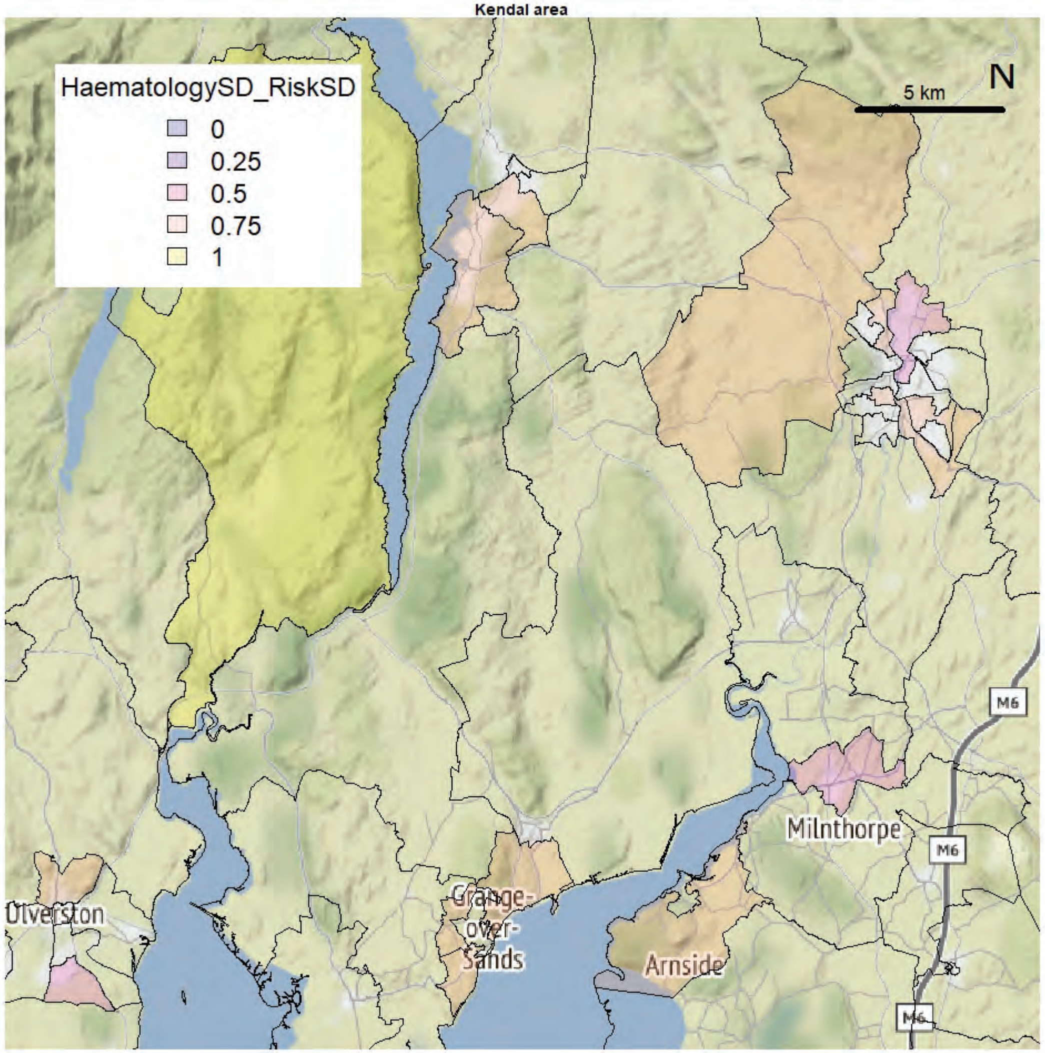

Kendal area
HaematologySD_RiskSD
0
0.25
0.5
0.75
1
5 km
N
M6
M6
M6
Milnthorpe
Ulverston
Grange-
over-
Sands
Arnside

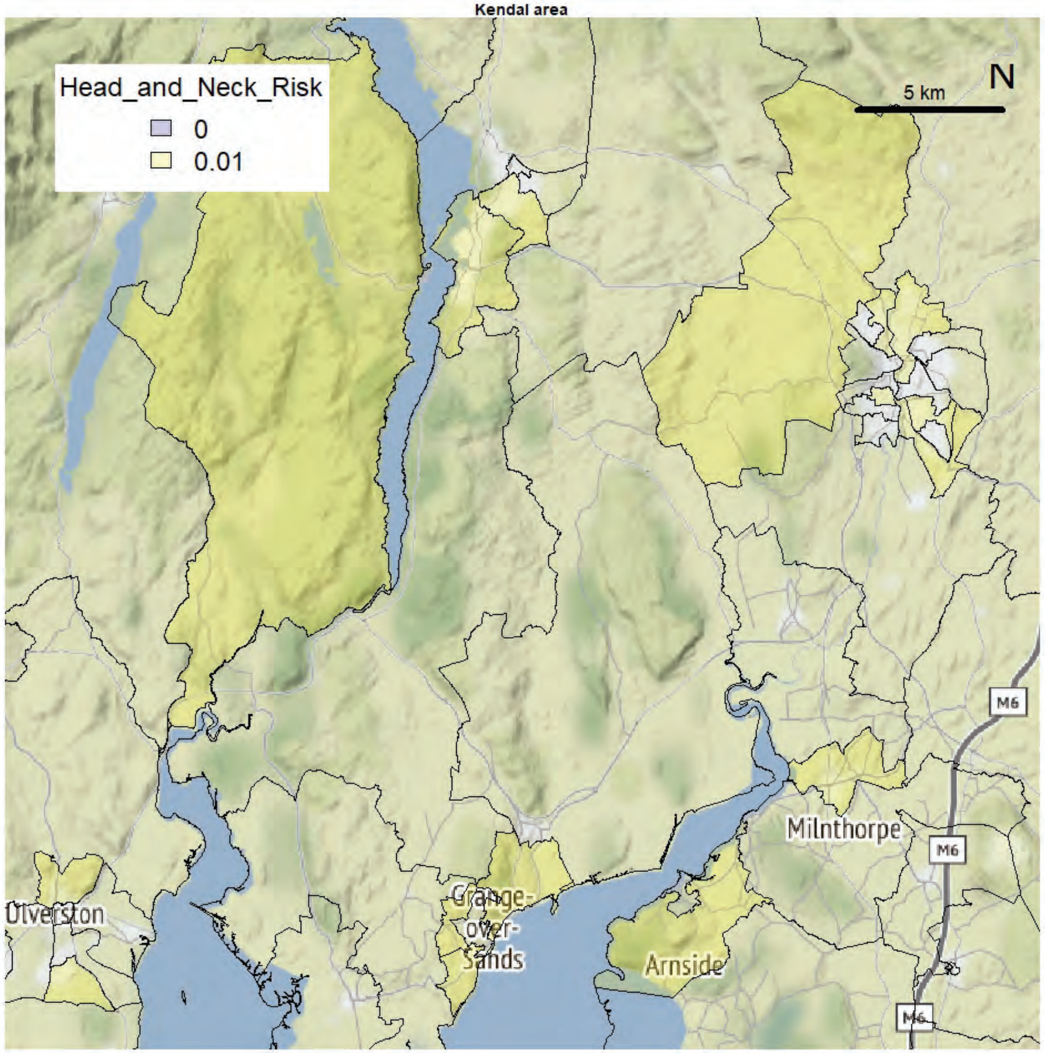

Kendal area
Head_and_Neck_Risk
0
0.01
5 km
N
M6
Milnthorpe
Ulverston
Grange-over-Sands
Arnside

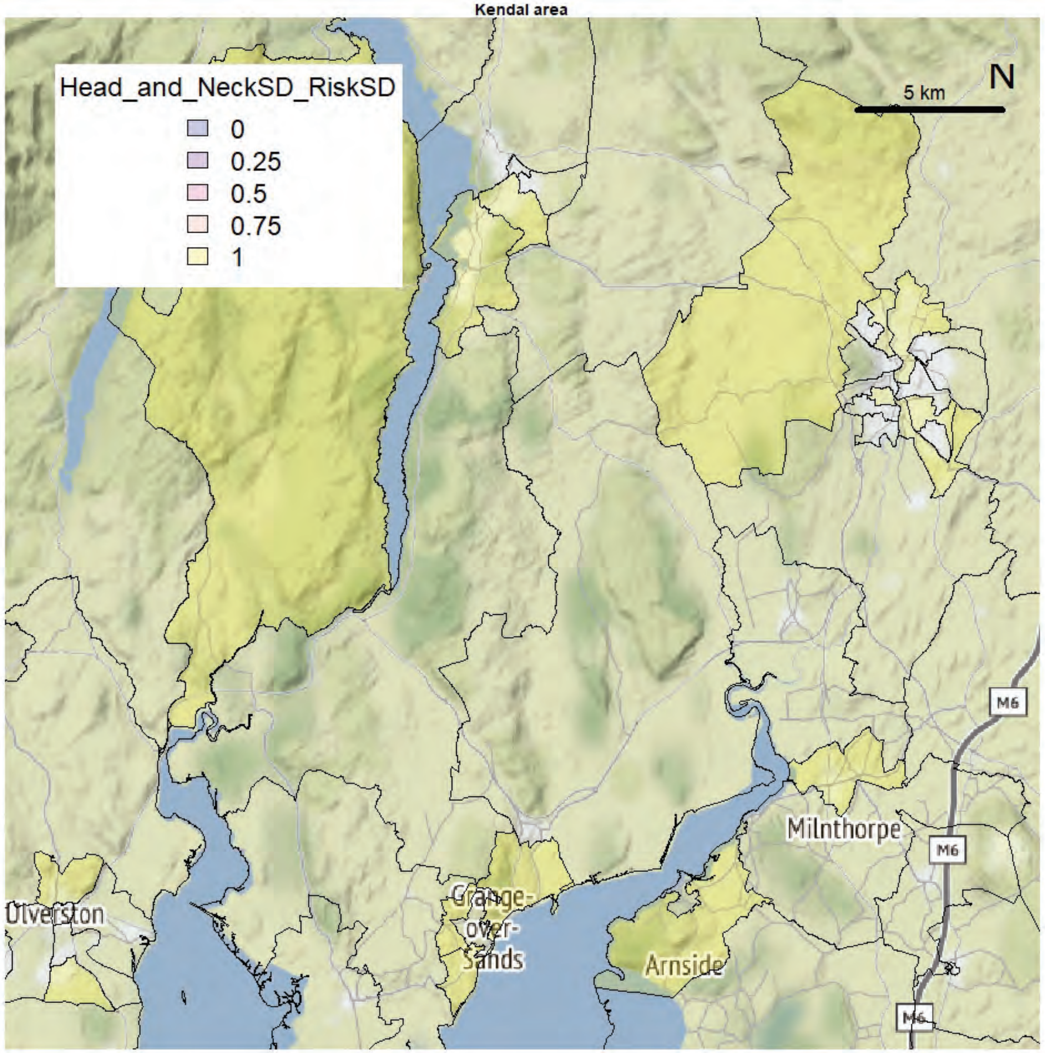

Kendal area
Head_and_NeckSD_RiskSD
0
0.25
0.5
0.75
1
5 km
N
M6
Milnthorpe
Ulverston
Grange-over-Sands
Arnside

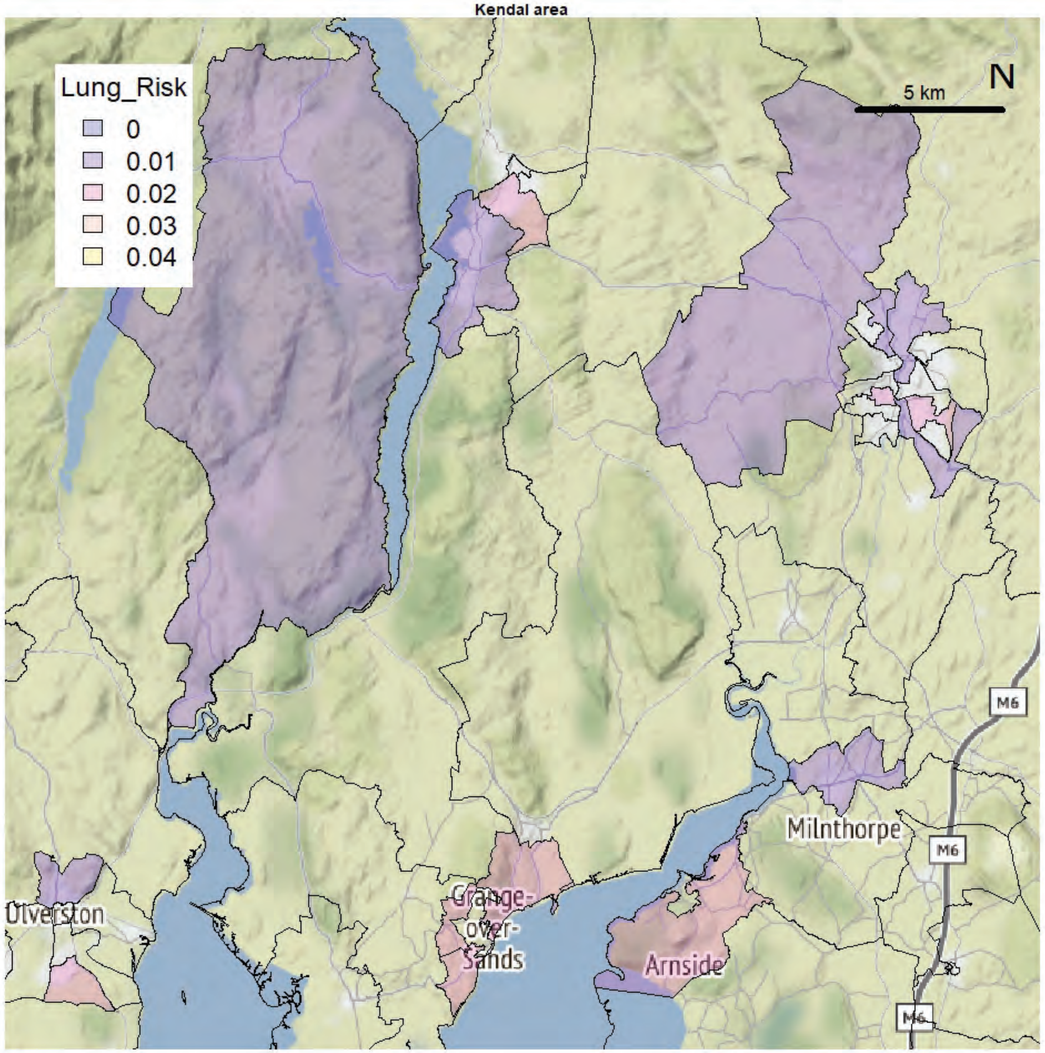
Kendal area
Lung_Risk
0
0.01
0.02
0.03
0.04
5 km
N
M6
Milnthorpe
Ulverston
Grange-over-Sands
Arnside

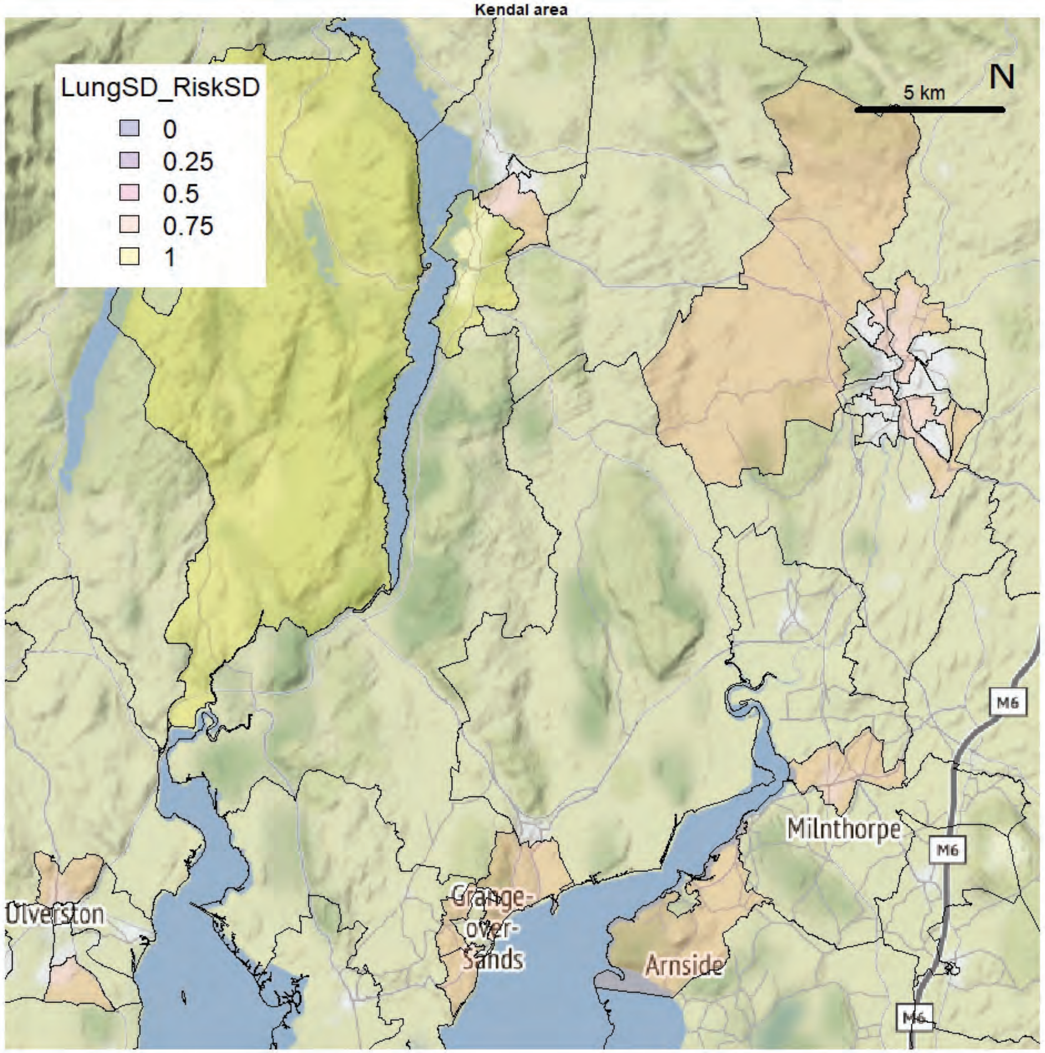
Kendal area
LungSD_RiskSD
0
0.25
0.5
0.75
1
5 km
N
M6
Milnthorpe
Ulverston
Grange-over-Sands
Arnside

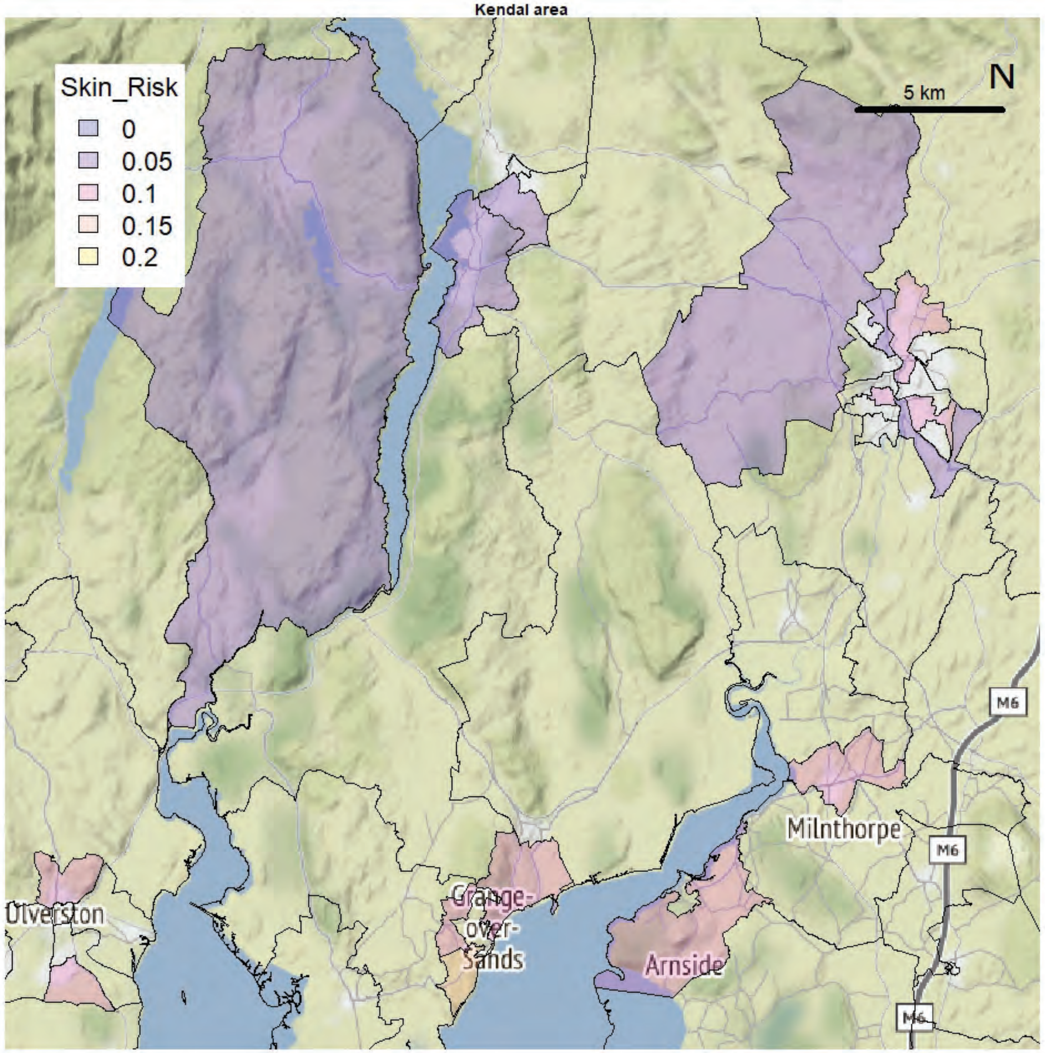
Kendal area
Skin_Risk
0
0.05
0.1
0.15
0.2
5 km
N
M6
M6
M6
Milnthorpe
Ulverston
Grange-over-Sands
Arnside

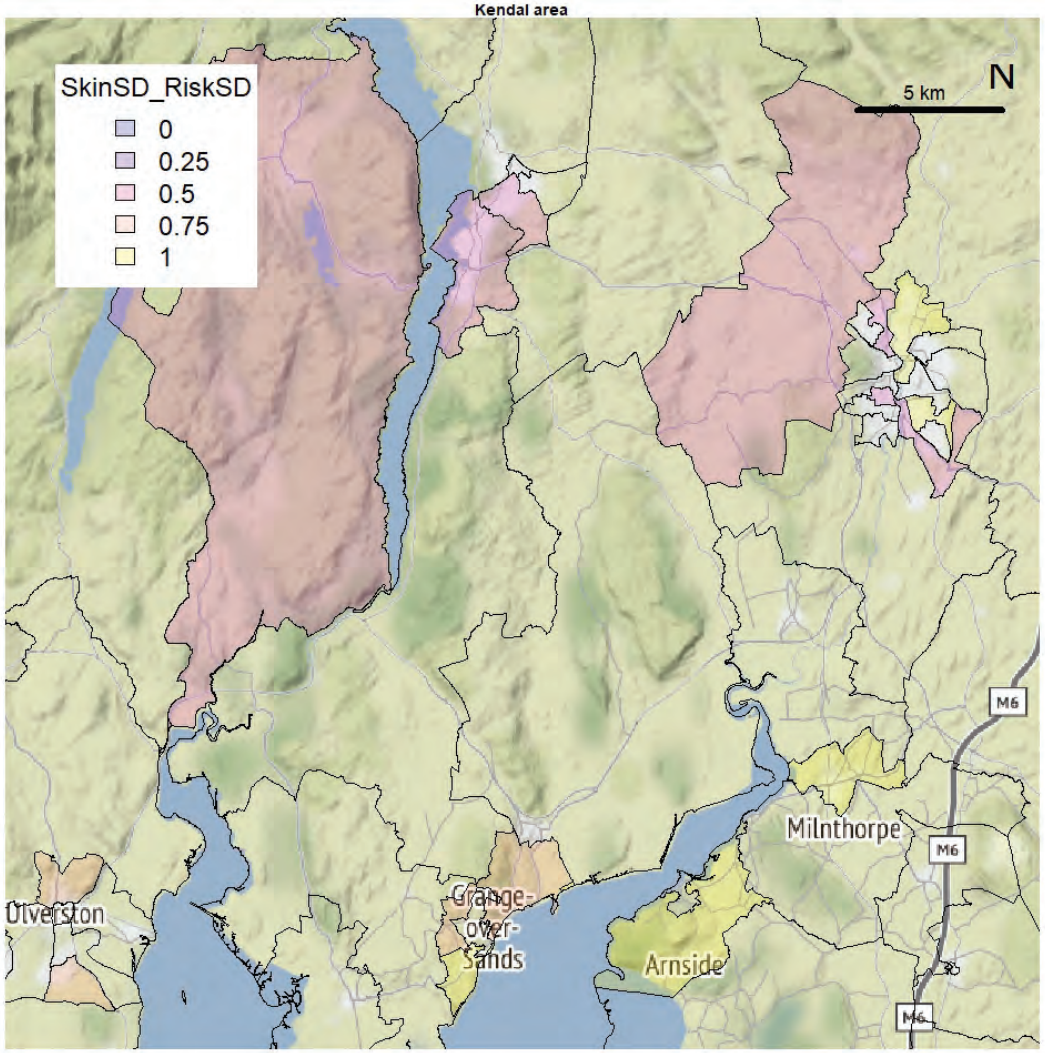
Kendal area
SkinSD_RiskSD
0
0.25
0.5
0.75
1
5 km
N
M6
M6
M6
Milnthorpe
Ulverston
Grange-over-Sands
Arnside

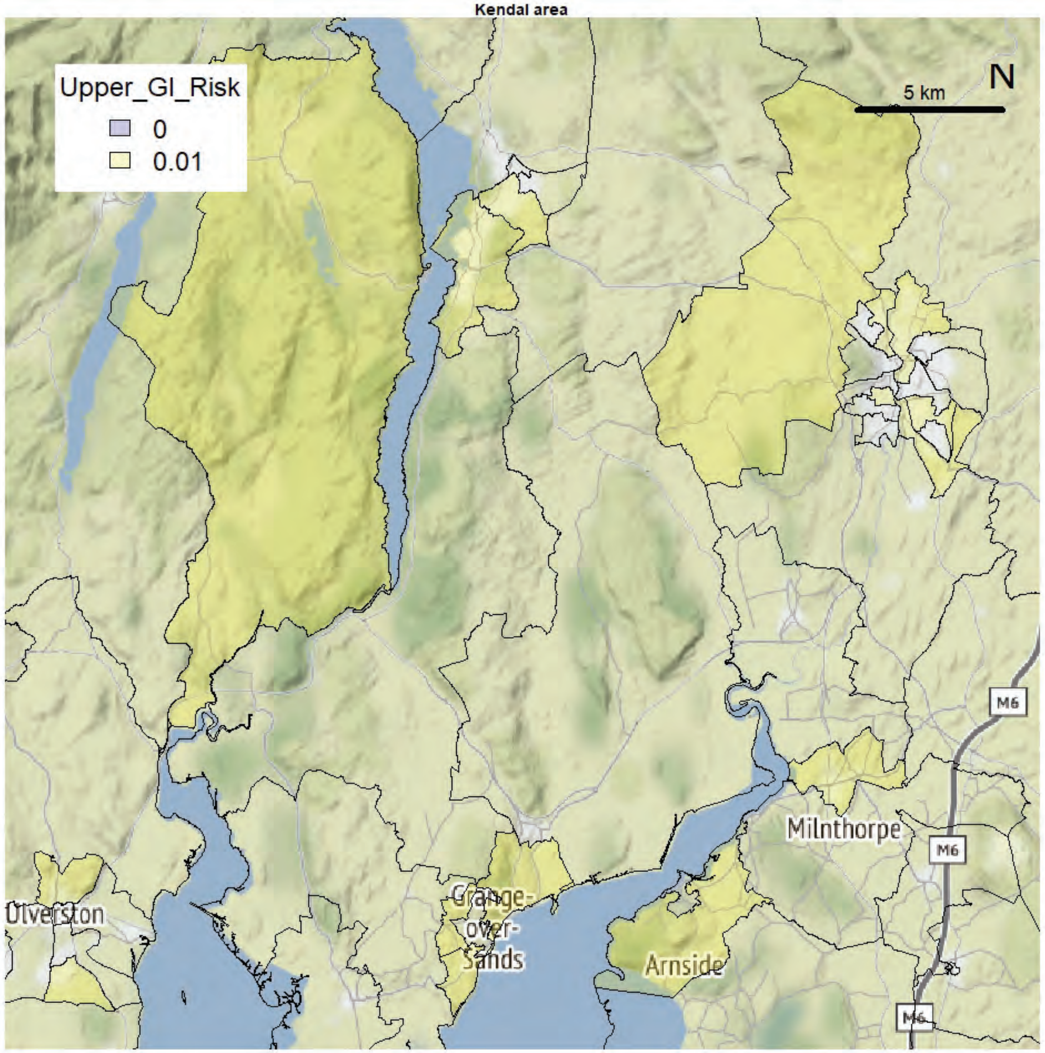

Kendal area
Upper_GI_Risk
0
0.01
5 km
N
M6
Milnthorpe
Ulverston
Grange-over-Sands
Arnside

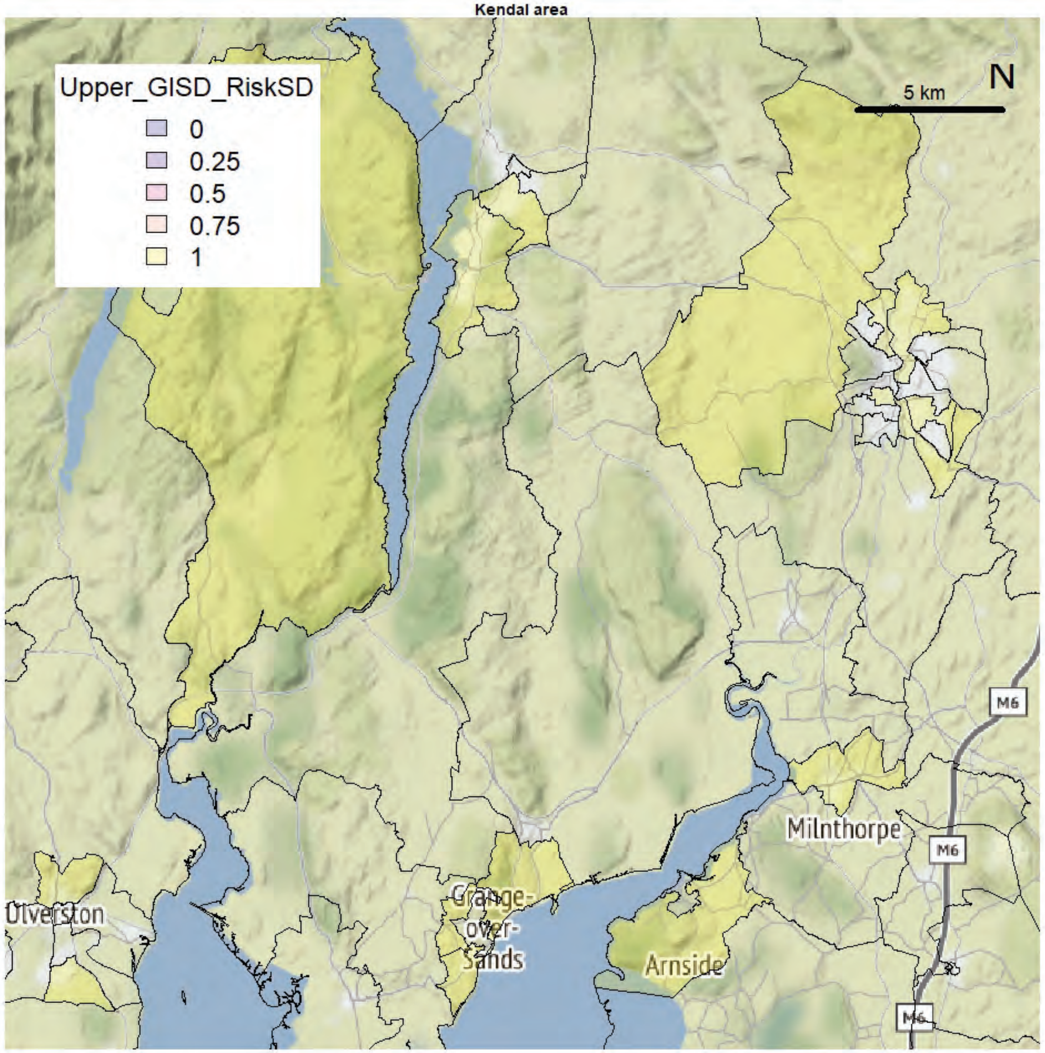

Kendal area
Upper_GISD_RiskSD
0
0.25
0.5
0.75
1
5 km
N
M6
Milnthorpe
Ulverston
Grange-over-Sands
Arnside

Kendal area

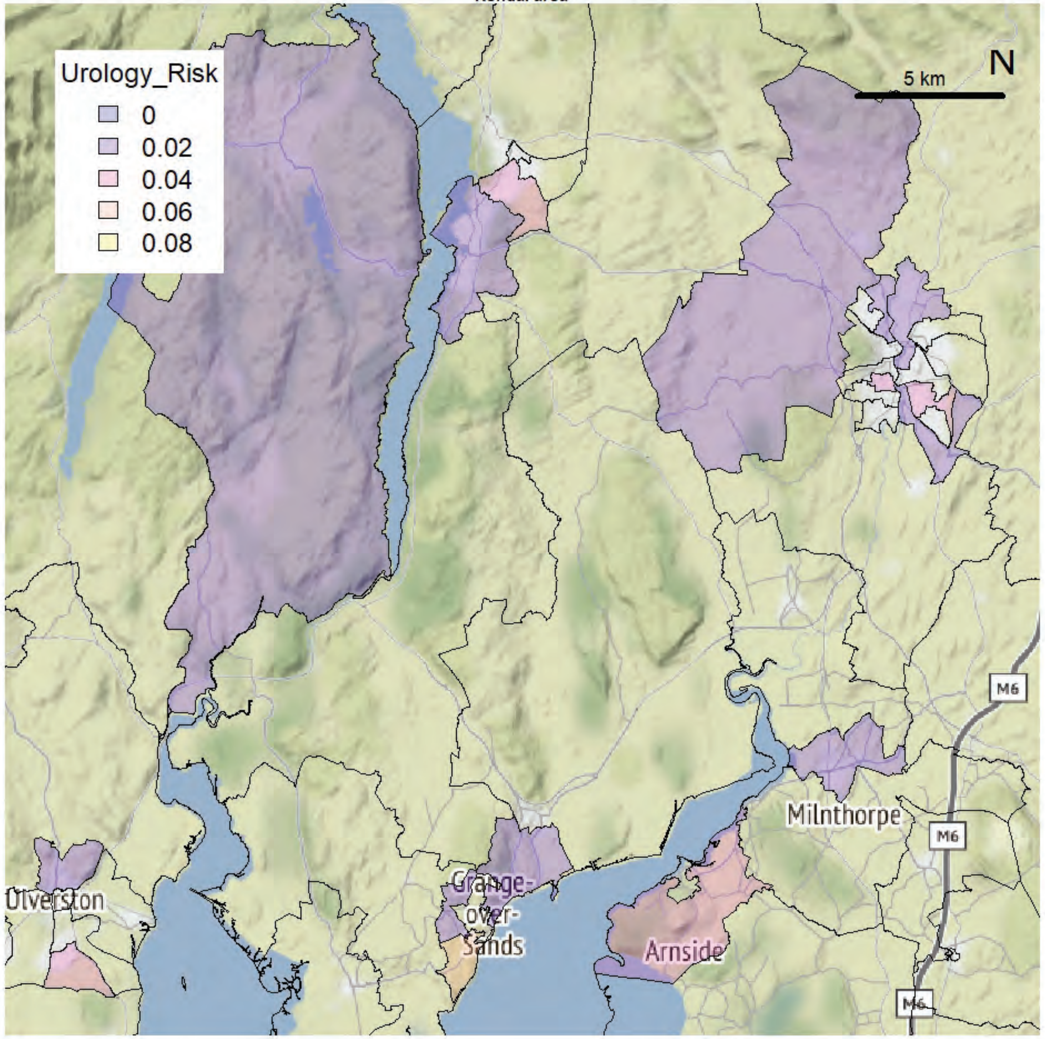

Kendal area

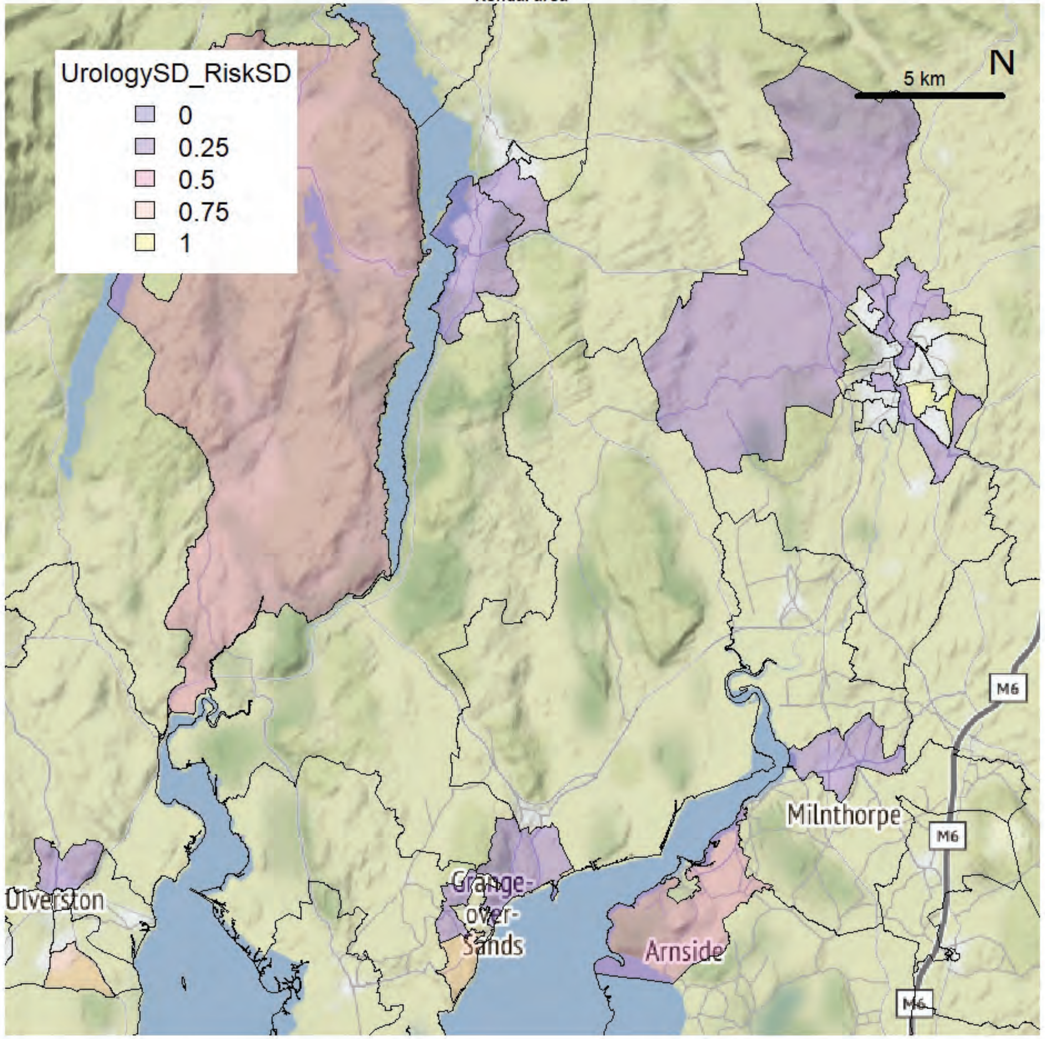

Supplementary Information S10: Cancer type risk mapping by age groups (under 50 and 50 years old and over).

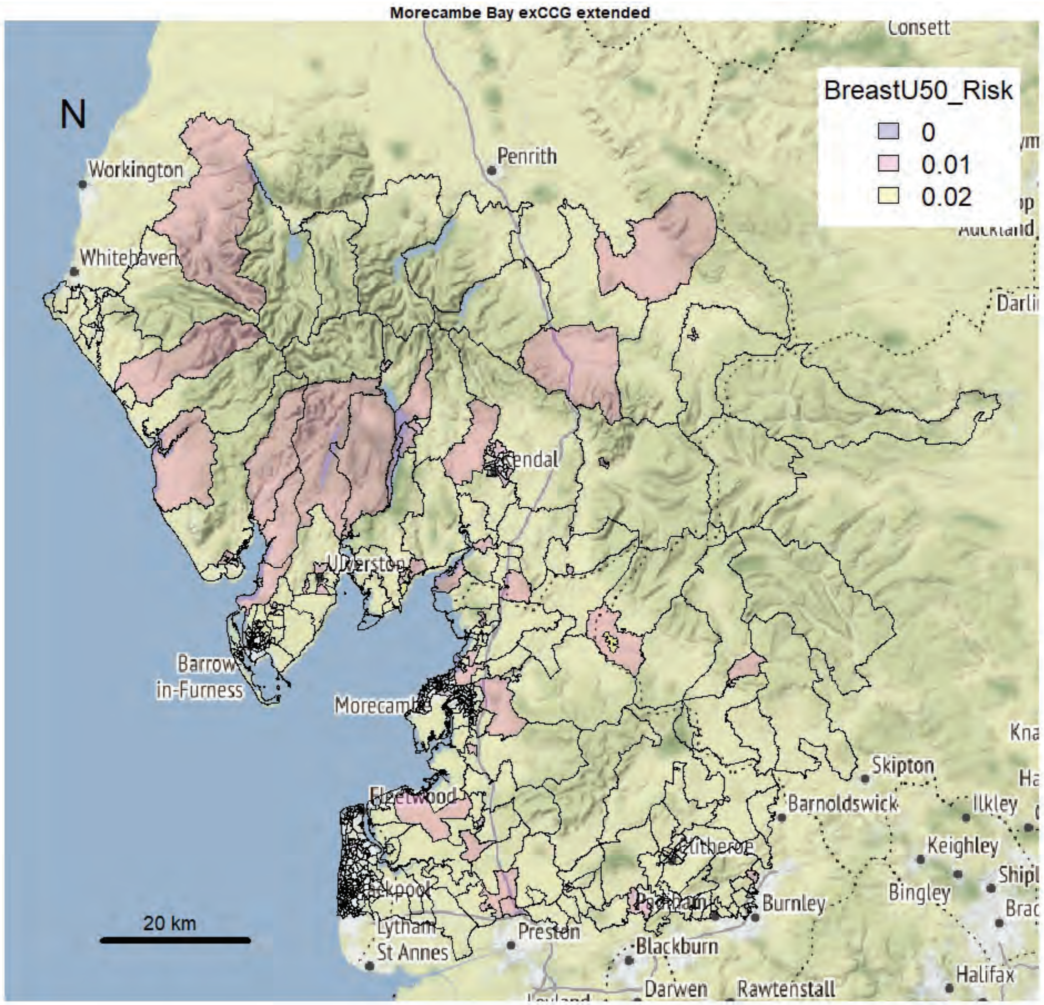

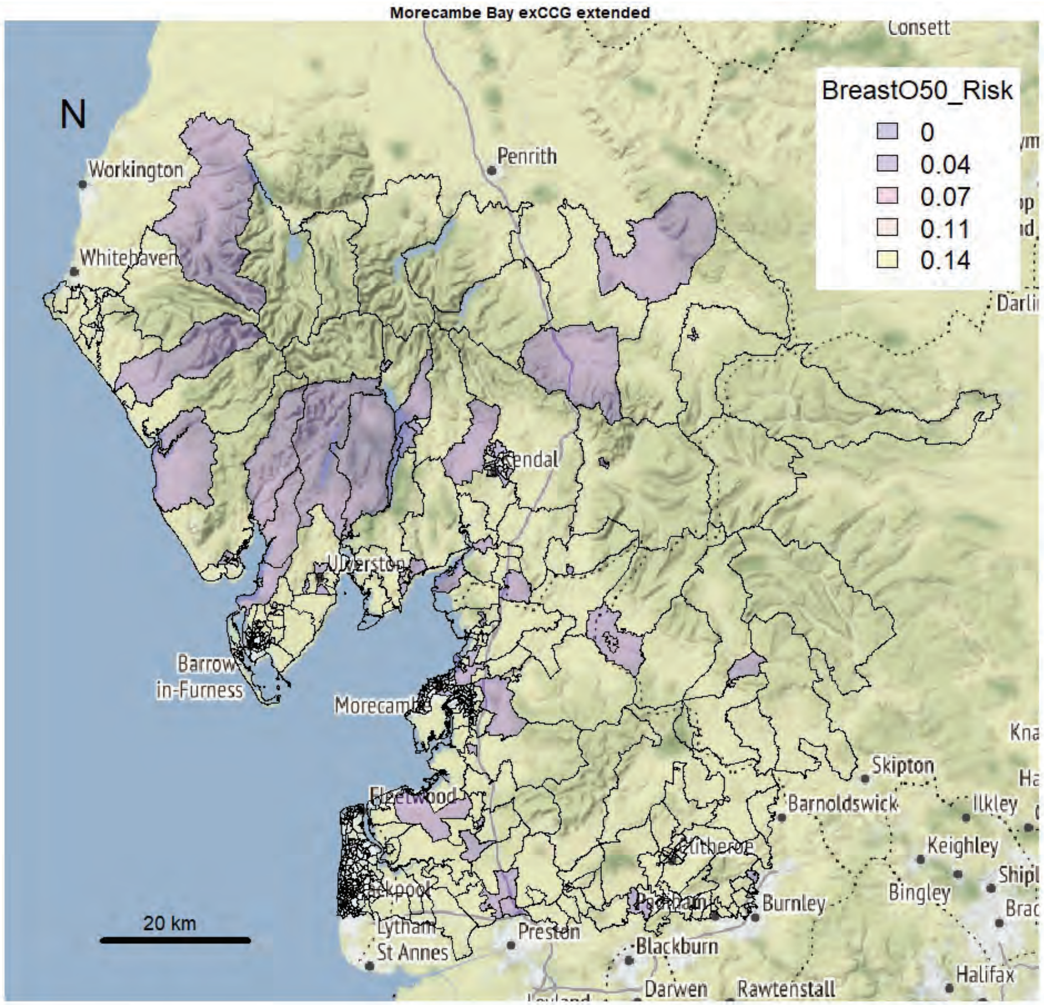

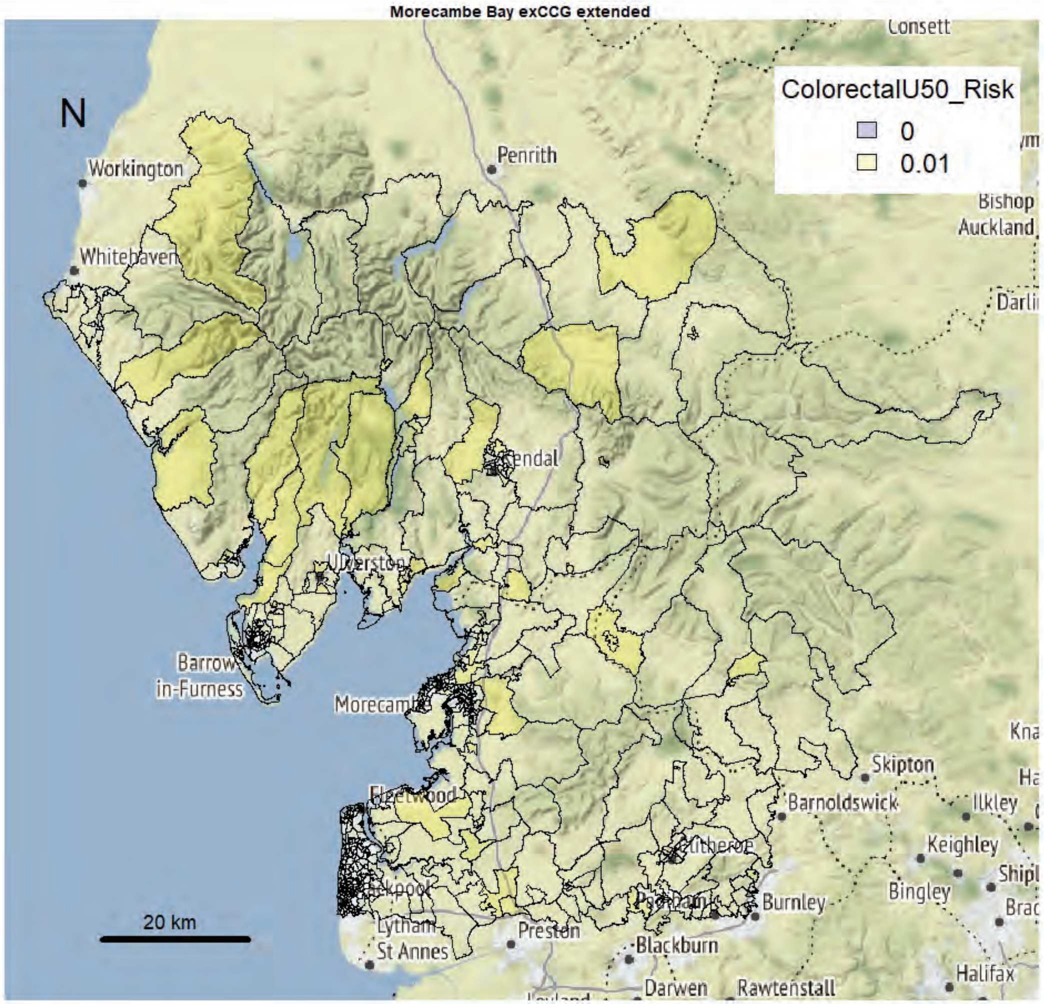
Morecambe Bay exCCG extended
ColorectalU50_Risk
0
0.01
N
20 km
Workington
Whitehaven
Penrith
Kendal
Ulverston
Barrow-in-Furness
Morecambe
Fleetwood
Blackpool
Lytham St Annes
Preston
Blackburn
Darwen
Rawtenstall
Burnley
Clitheroe
Skipton
Barnoldswick
Ilkley
Keighley
Bingley
Halifax
Consett
Bishop Auckland

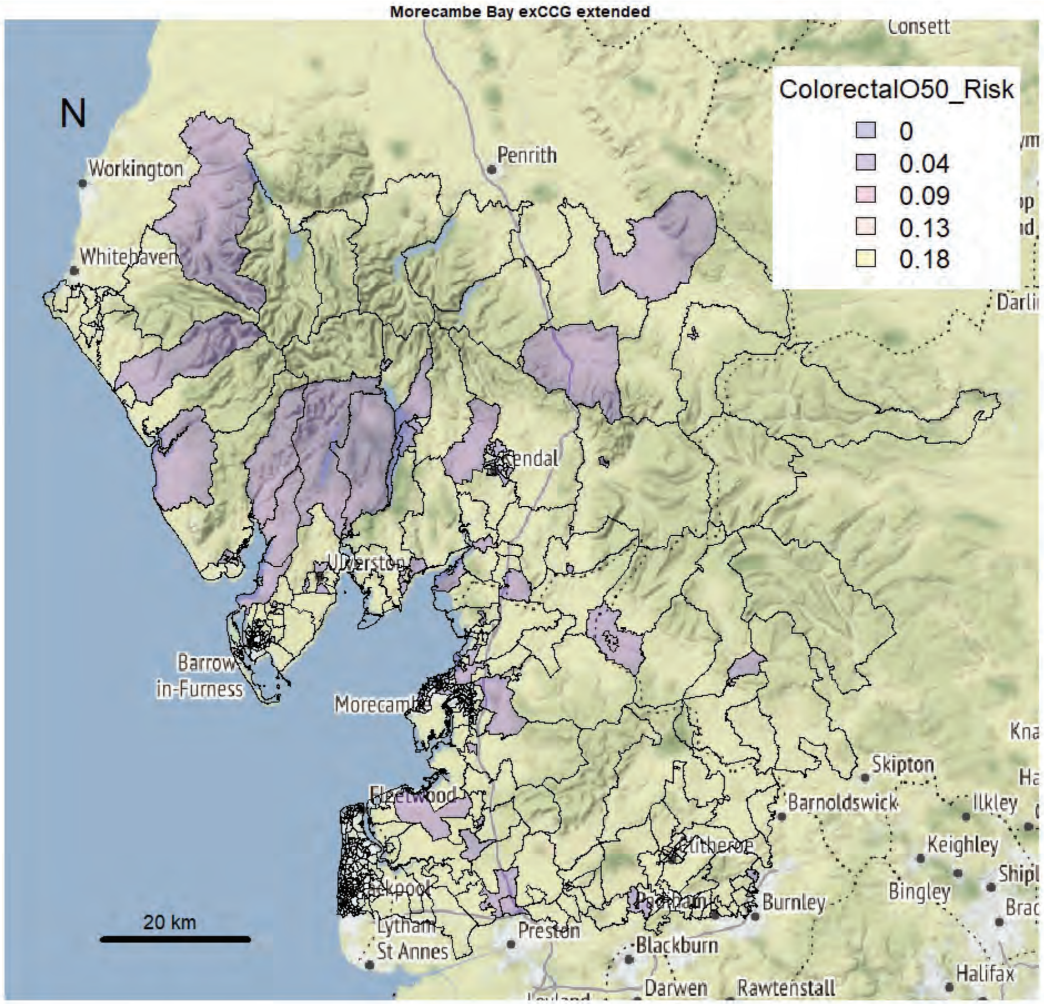
Morecambe Bay exCCG extended
ColorectalO50_Risk
0
0.04
0.09
0.13
0.18
N
20 km
Workington
Whitehaven
Penrith
Kendal
Ulverston
Barrow-in-Furness
Morecambe
Fleetwood
Blackpool
Lytham St Annes
Preston
Blackburn
Darwen
Rawtenstall
Burnley
Clitheroe
Skipton
Barnoldswick
Ilkley
Keighley
Bingley
Halifax
Consett

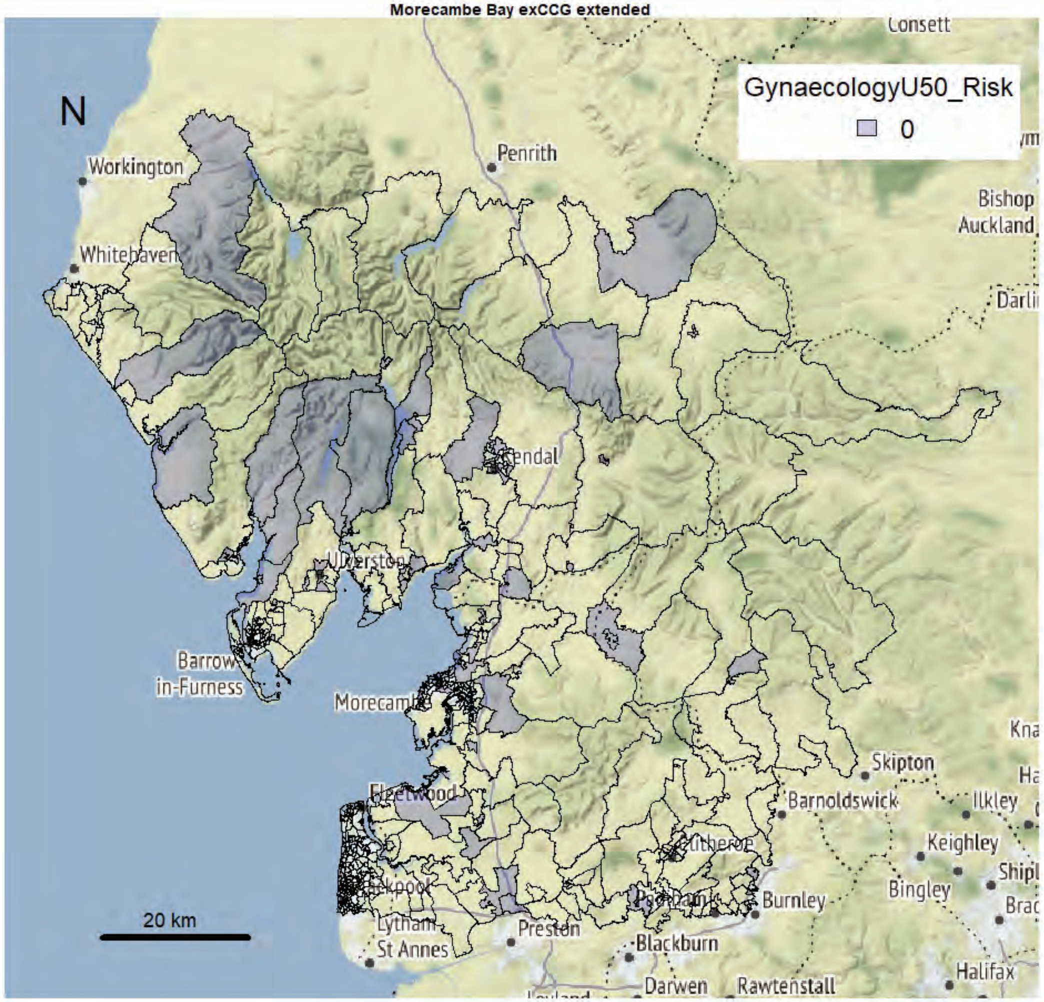
Morecambe Bay exCCG extended
GynaecologyU50_Risk
0
N
20 km

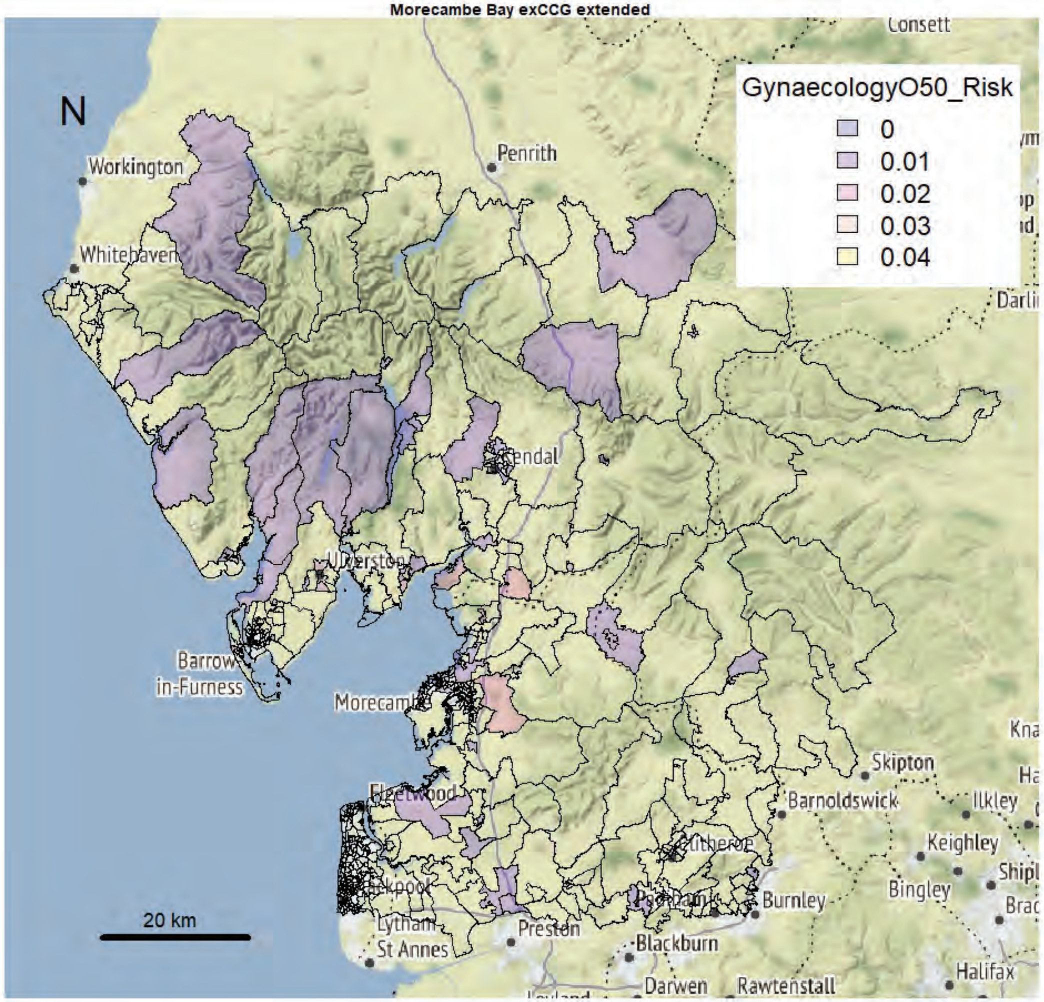
Morecambe Bay exCCG extended
GynaecologyO50_Risk
0
0.01
0.02
0.03
0.04
N
20 km

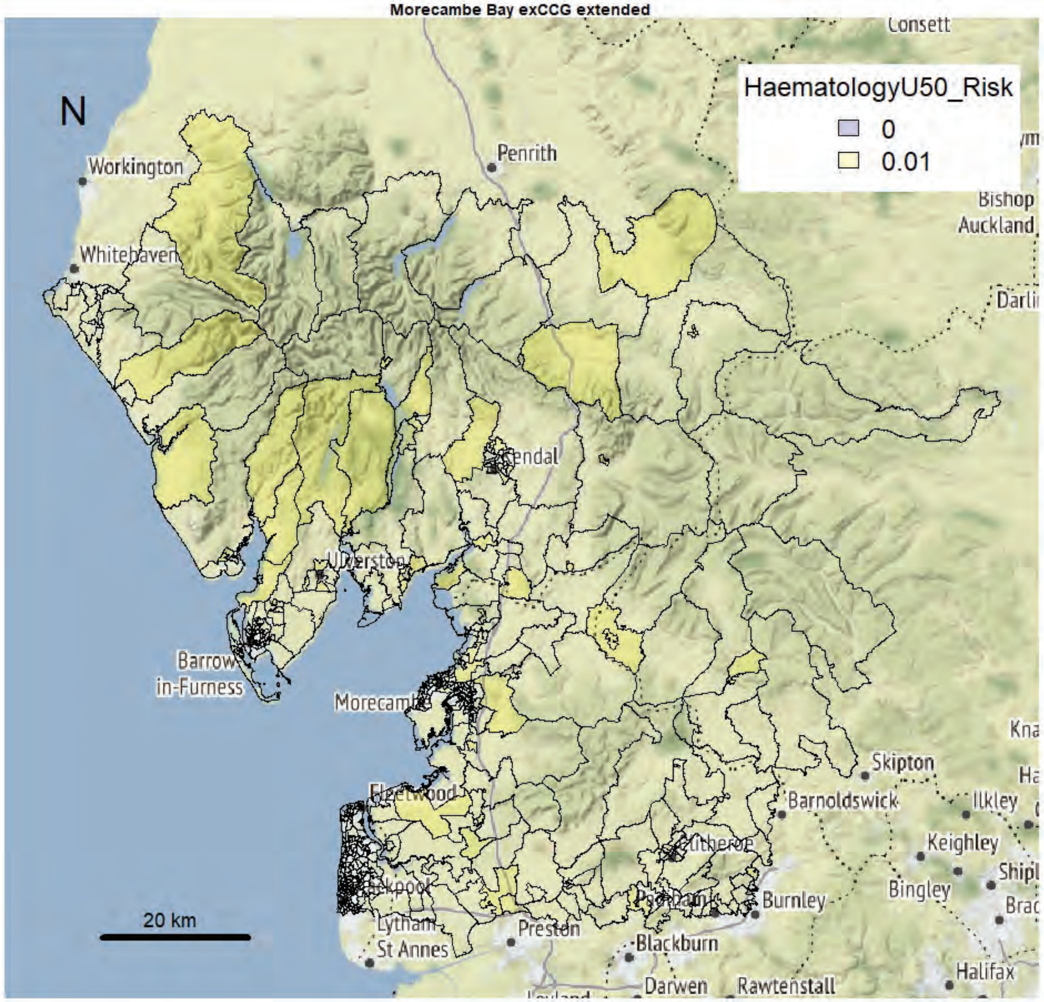
Morecambe Bay exCCG extended
HaematologyU50_Risk
0
0.01
N
20 km
Workington
Penrith
Whitehaven
Bishop Auckland
Kendal
Ulverston
Barrow in-Furness
Fleetwood
Skipton
Barnoldswick
Ilkley
Keighley
Bingley
Burnley
Preston
Blackburn
Darwen
Rawtenstall
Halifax
Lytham St Annes
Consett

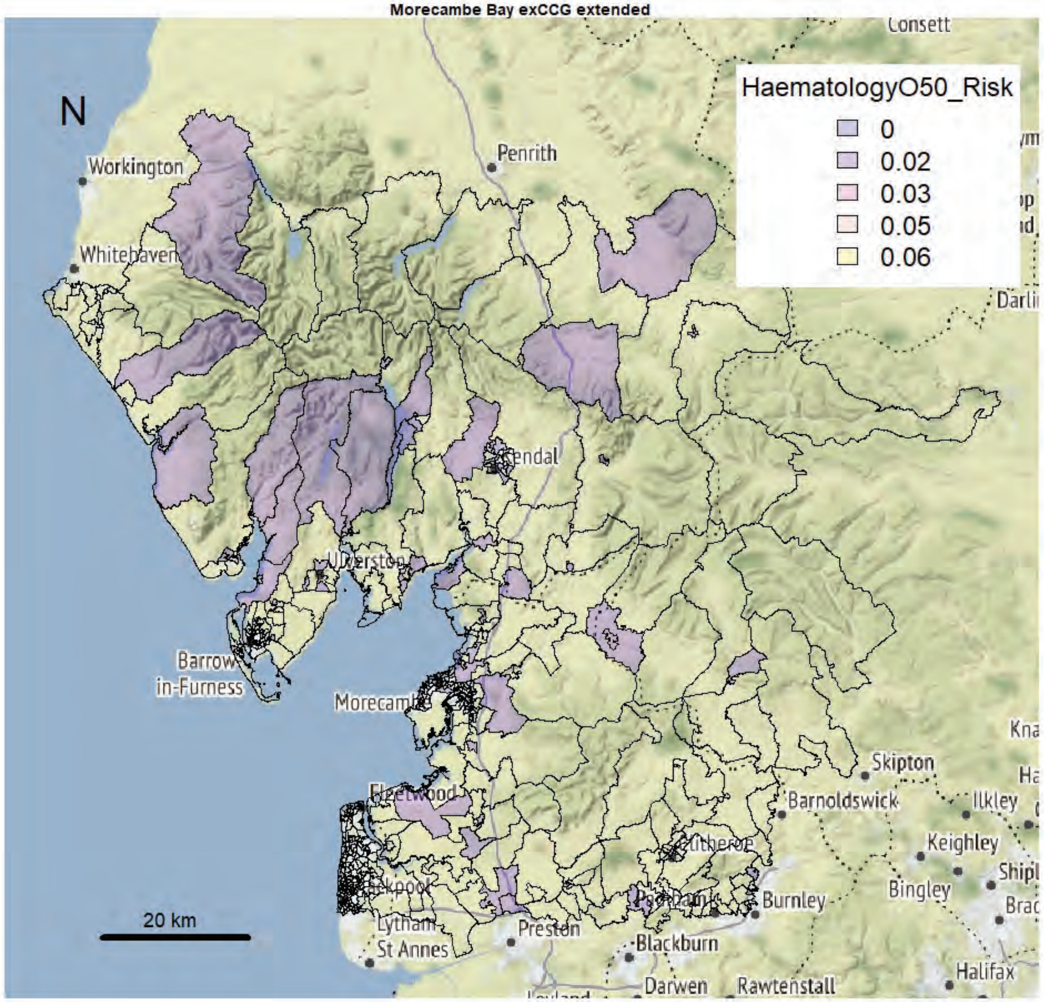
Morecambe Bay exCCG extended
HaematologyO50_Risk
0
0.02
0.03
0.05
0.06
N
20 km
Workington
Penrith
Whitehaven
Kendal
Ulverston
Barrow in-Furness
Fleetwood
Skipton
Barnoldswick
Ilkley
Keighley
Bingley
Burnley
Preston
Blackburn
Darwen
Rawtenstall
Halifax
Lytham St Annes
Consett

Morecambe Bay exCCG extended

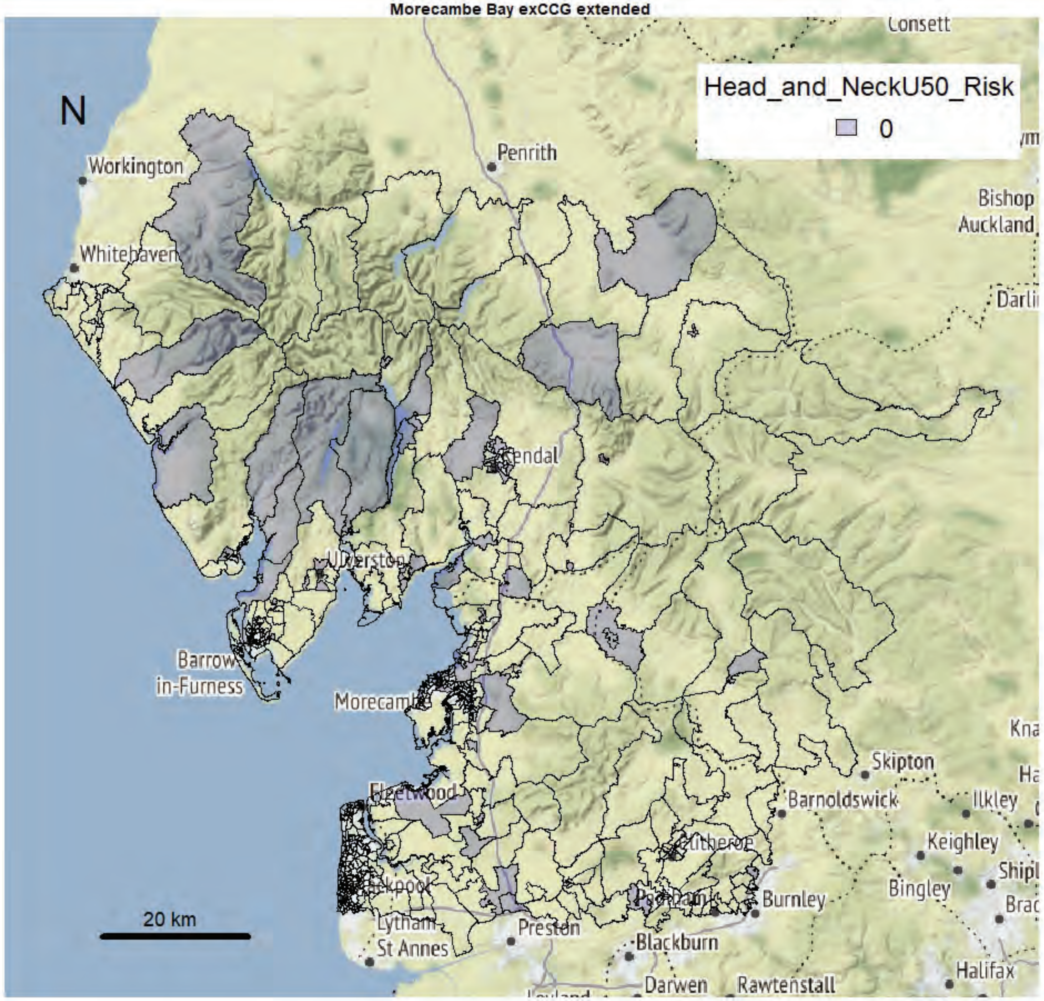

Morecambe Bay exCCG extended

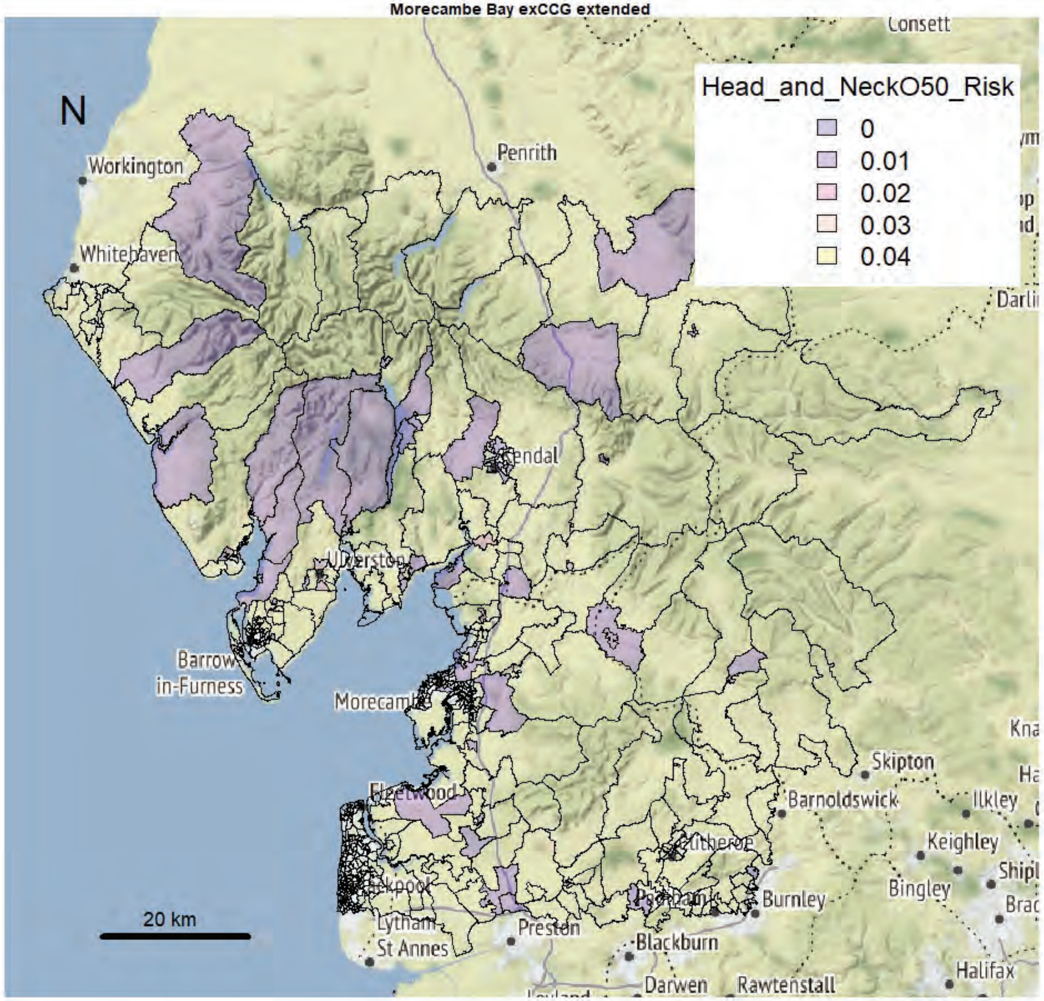

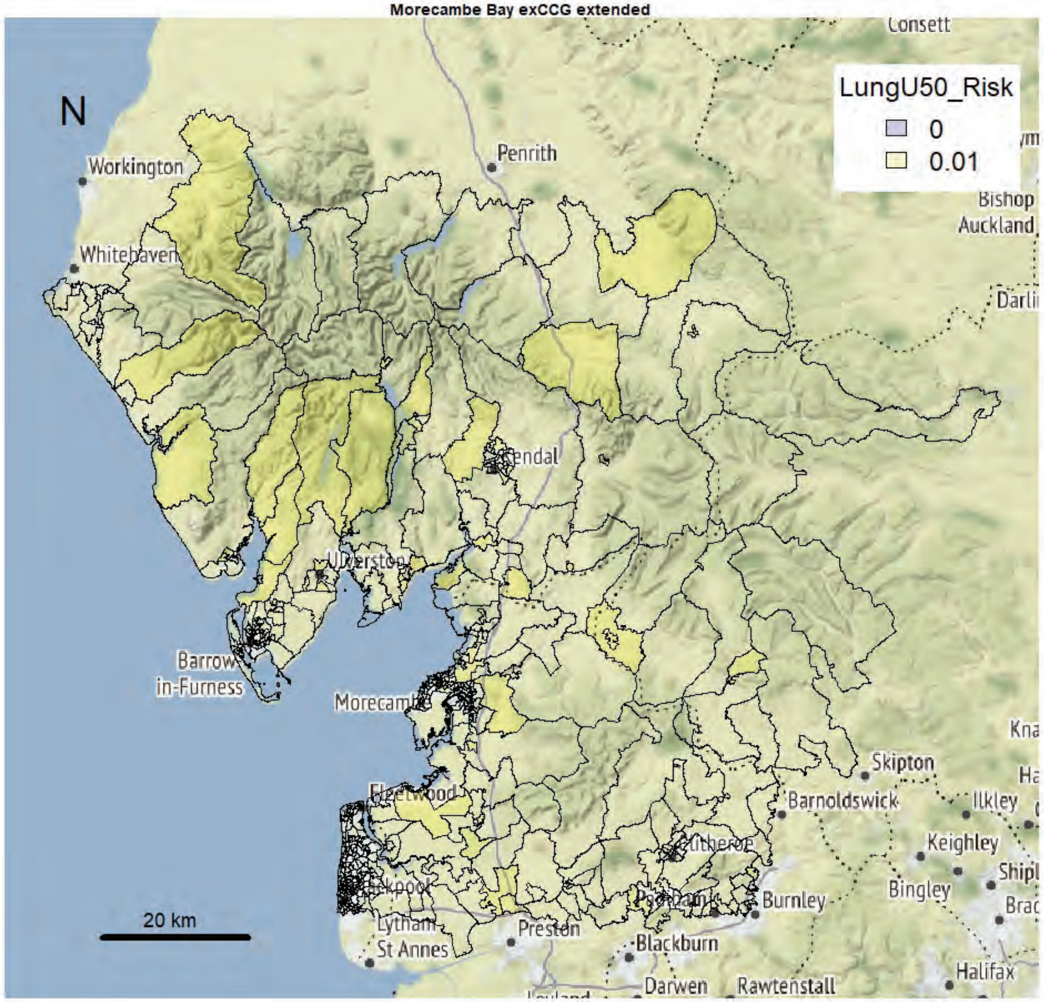

Morecambe Bay exCCG extended
N
LungU50_Risk
0
0.01
20 km

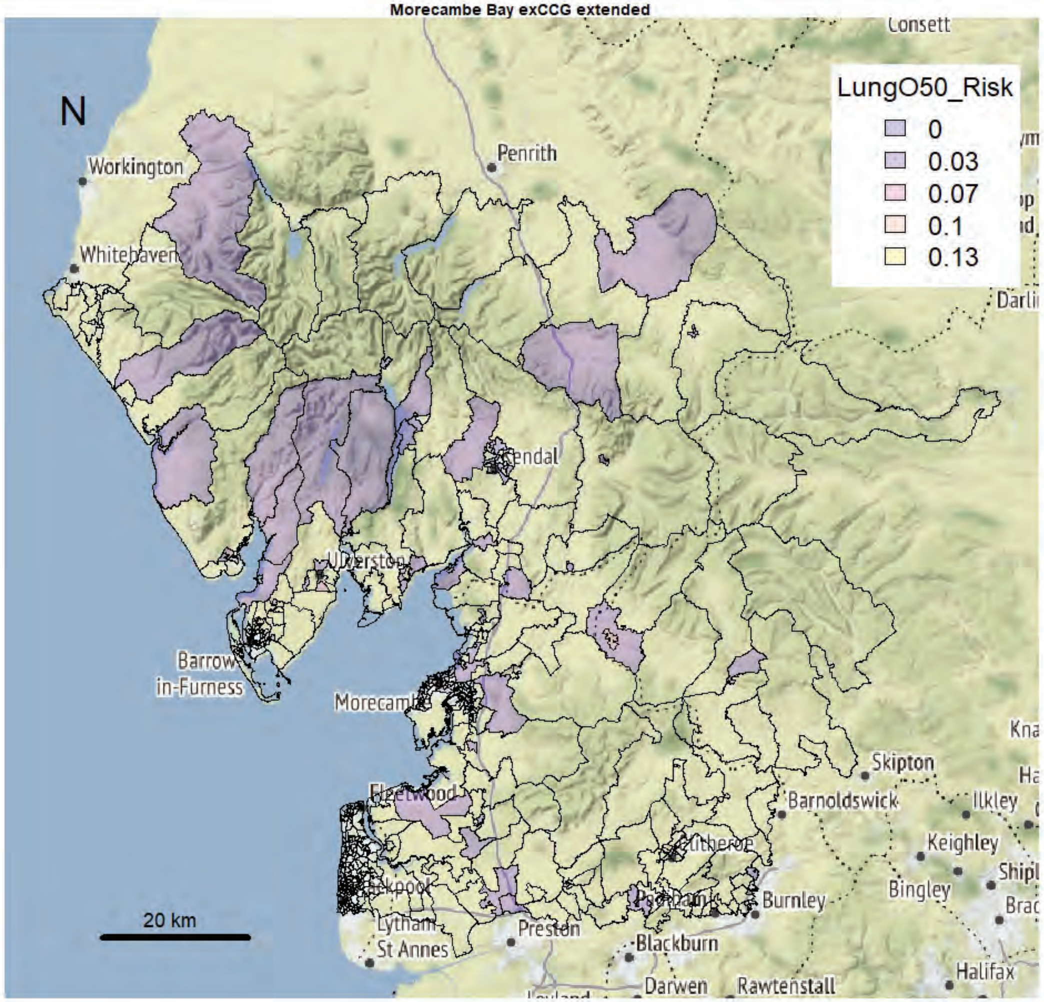

Morecambe Bay exCCG extended
N
LungO50_Risk
0
0.03
0.07
0.1
0.13
20 km

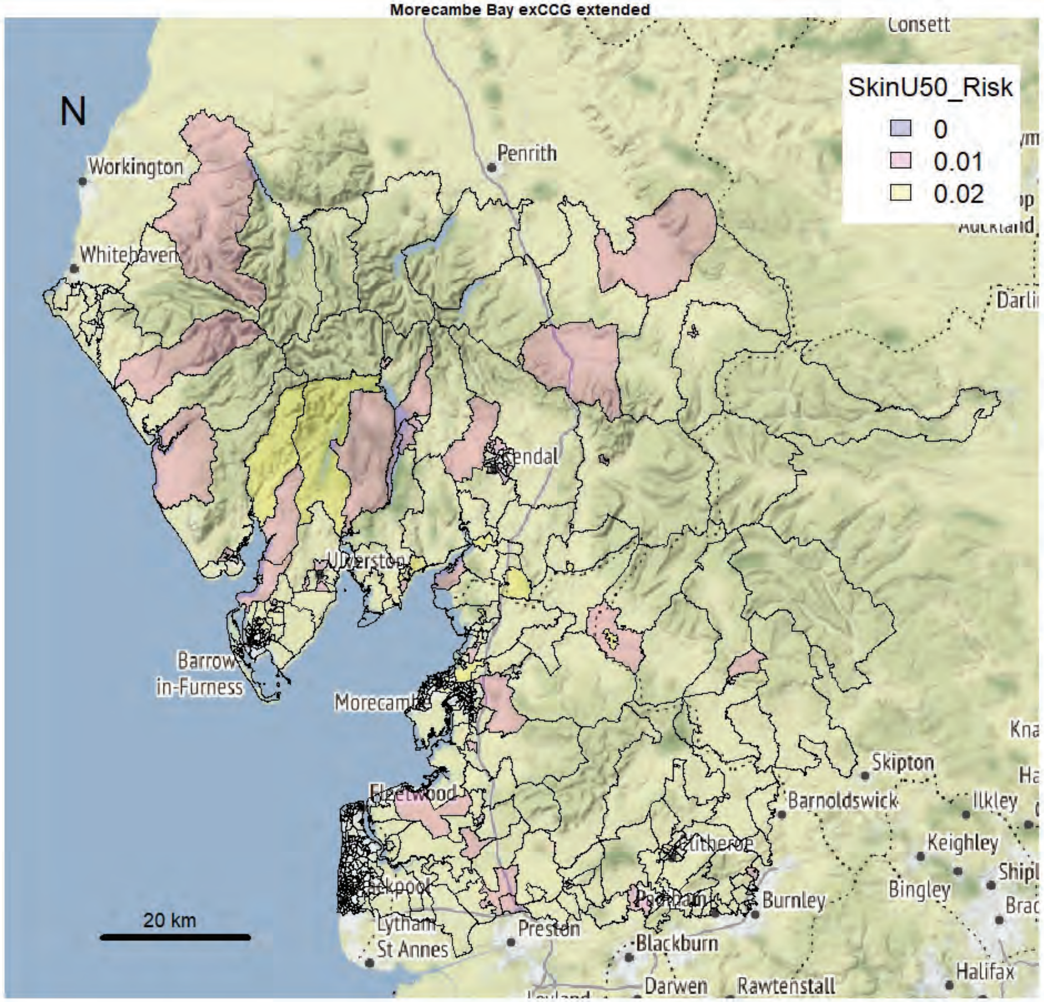
Morecambe Bay exCCG extended
N
SkinU50_Risk
0
0.01
0.02
Consett
Workington
Penrith
Whitehaven
Kendal
Ulverston
Barrow-in-Furness
Morecambe
Fleetwood
Skipton
Barnoldswick
Ilkley
Keighley
Bingley
Clitheroe
Blackpool
Burnley
Lytham St Annes
Preston
Blackburn
Darwen
Rawtenstall
Halifax
20 km

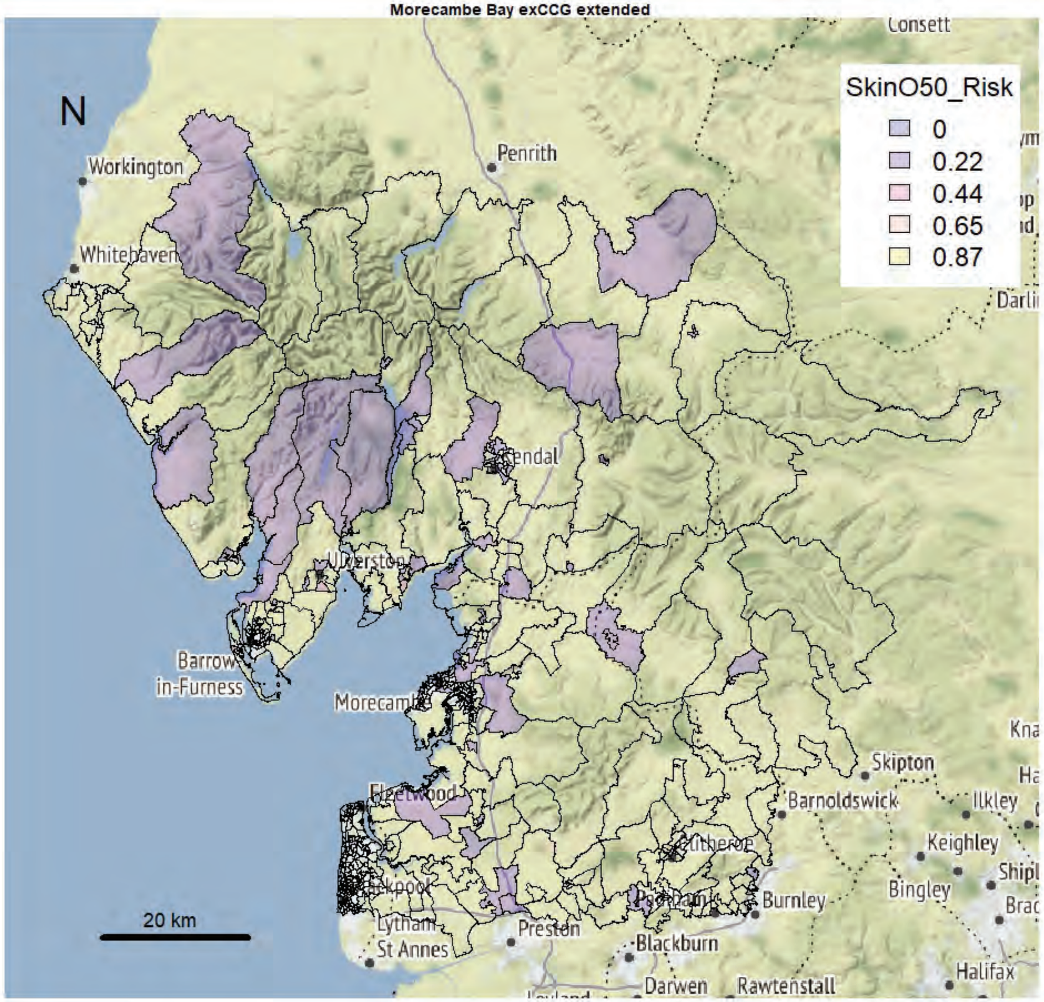
Morecambe Bay exCCG extended
N
SkinO50_Risk
0
0.22
0.44
0.65
0.87
Consett
Workington
Penrith
Whitehaven
Kendal
Ulverston
Barrow-in-Furness
Morecambe
Fleetwood
Skipton
Barnoldswick
Ilkley
Keighley
Bingley
Clitheroe
Blackpool
Burnley
Lytham St Annes
Preston
Blackburn
Darwen
Rawtenstall
Halifax
20 km

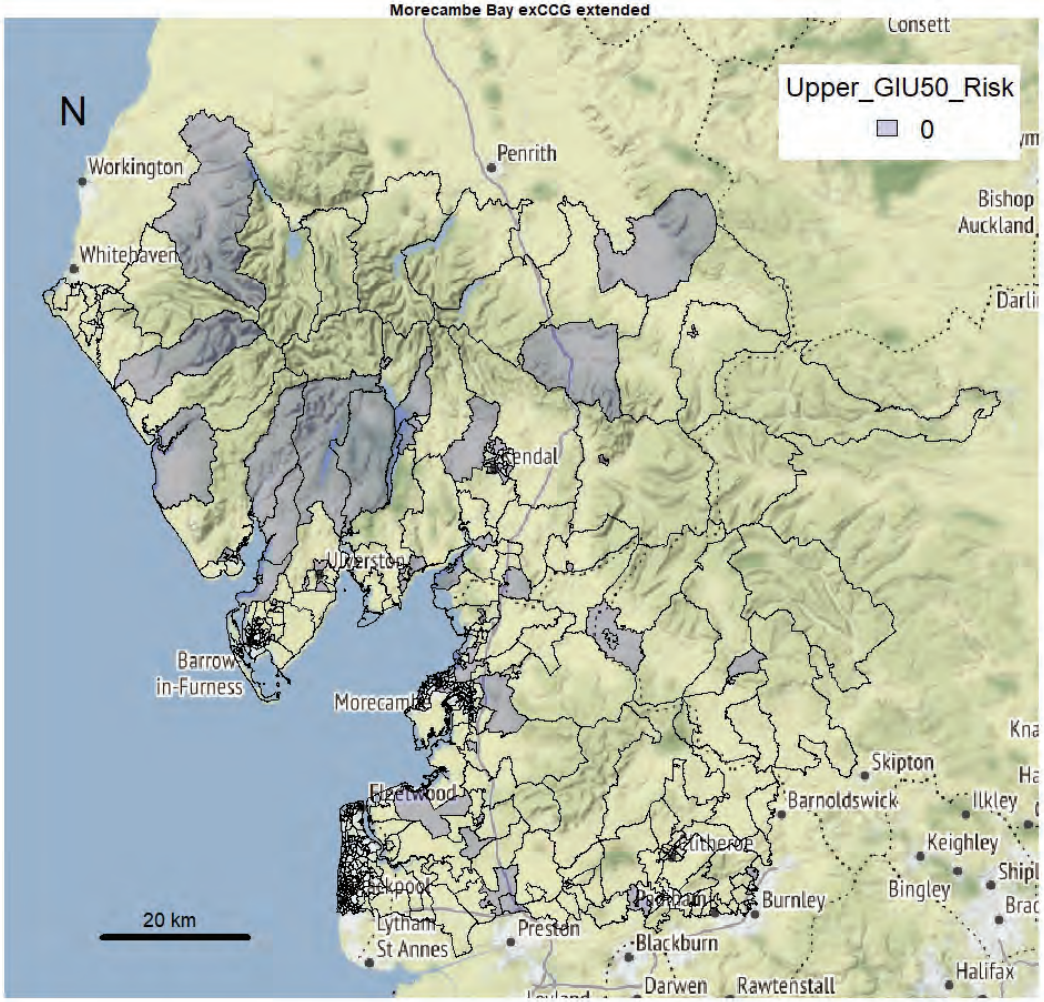

Morecambe Bay exCCG extended
N
Upper_GIU50_Risk
0
20 km
Workington
Penrith
Whitehaven
Kendal
Ulverston
Barrow-in-Furness
Morecambe
Fleetwood
Blackpool
Lytham St Annes
Preston
Blackburn
Darwen
Rawtenstall
Burnley
Skipton
Barnoldswick
Ilkley
Keighley
Bingley
Halifax
Consett
Bishop Auckland

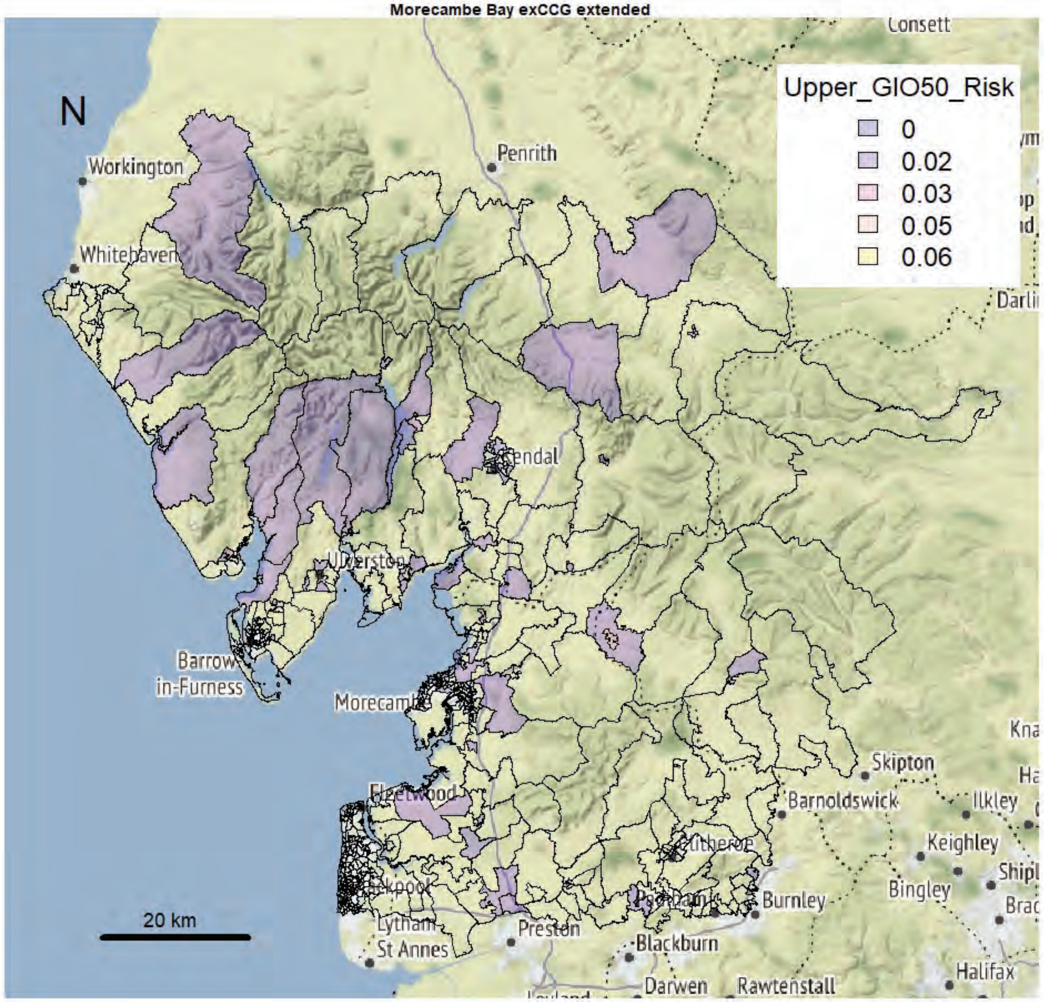

Morecambe Bay exCCG extended
N
Upper_GIO50_Risk
0
0.02
0.03
0.05
0.06
20 km
Workington
Penrith
Whitehaven
Kendal
Ulverston
Barrow-in-Furness
Morecambe
Fleetwood
Blackpool
Lytham St Annes
Preston
Blackburn
Darwen
Rawtenstall
Burnley
Skipton
Barnoldswick
Ilkley
Keighley
Bingley
Halifax
Consett

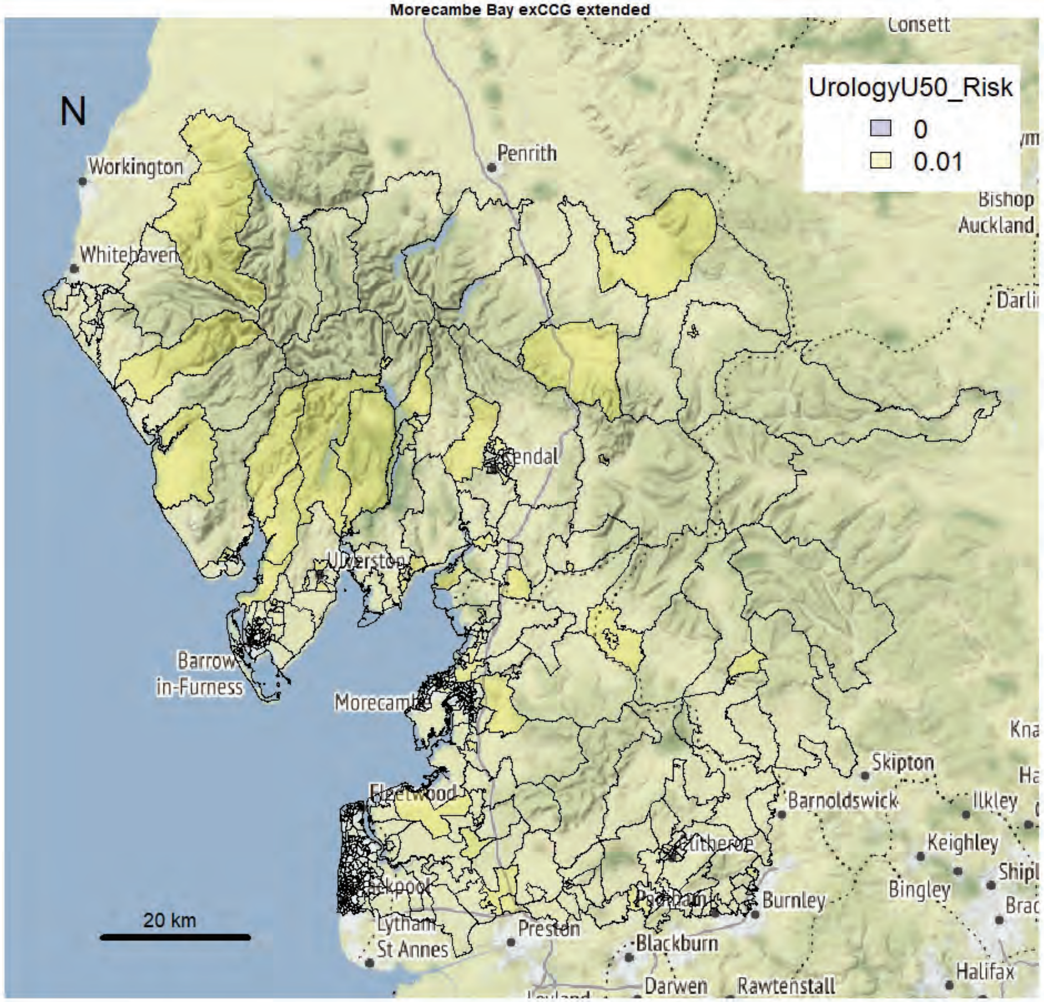
Morecambe Bay exCCG extended
UrologyU50_Risk
0
0.01
N
20 km
Workington
Whitehaven
Penrith
Consett
Bishop Auckland
Kendal
Ulverston
Barrow-in-Furness
Morecambe
Fleetwood
Blackpool
Lytham St Annes
Preston
Blackburn
Darwen
Rawtenstall
Burnley
Clitheroe
Skipton
Barnoldswick
Ilkley
Keighley
Bingley
Halifax

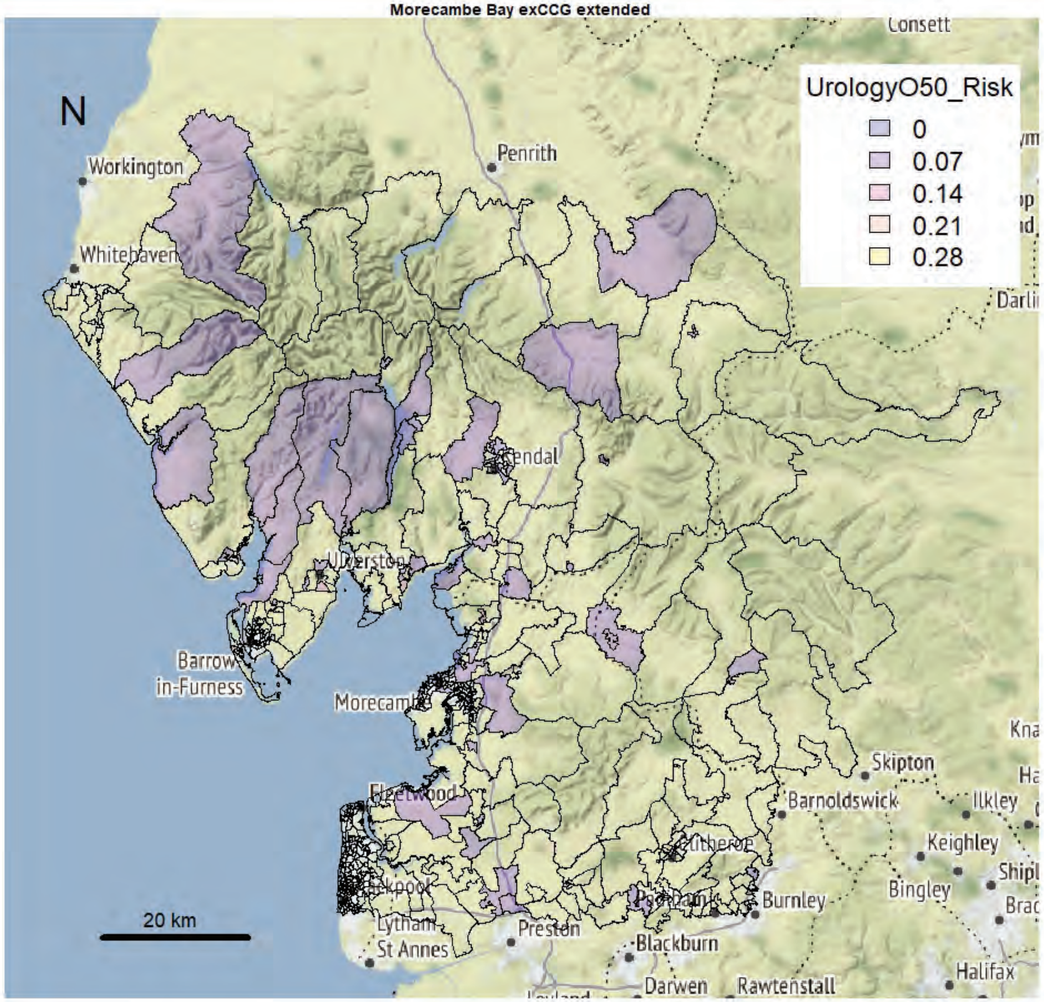
Morecambe Bay exCCG extended
UrologyO50_Risk
0
0.07
0.14
0.21
0.28
N
20 km
Workington
Whitehaven
Penrith
Consett
Kendal
Ulverston
Barrow-in-Furness
Morecambe
Fleetwood
Blackpool
Lytham St Annes
Preston
Blackburn
Darwen
Rawtenstall
Burnley
Clitheroe
Skipton
Barnoldswick
Ilkley
Keighley
Bingley
Halifax

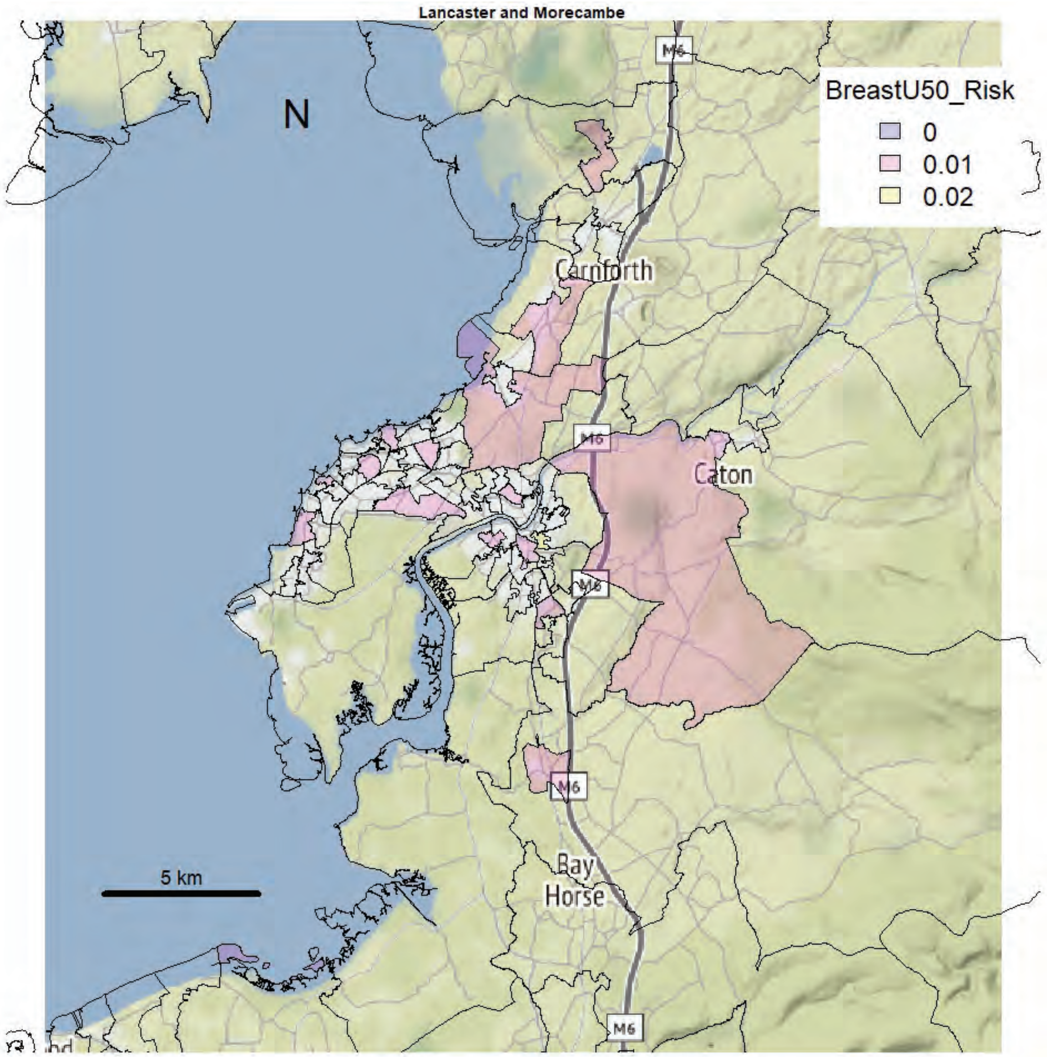
Lancaster and Morecambe
N
BreastU50_Risk
0
0.01
0.02
M6
Carnforth
Caton
Bay
Horse
5 km

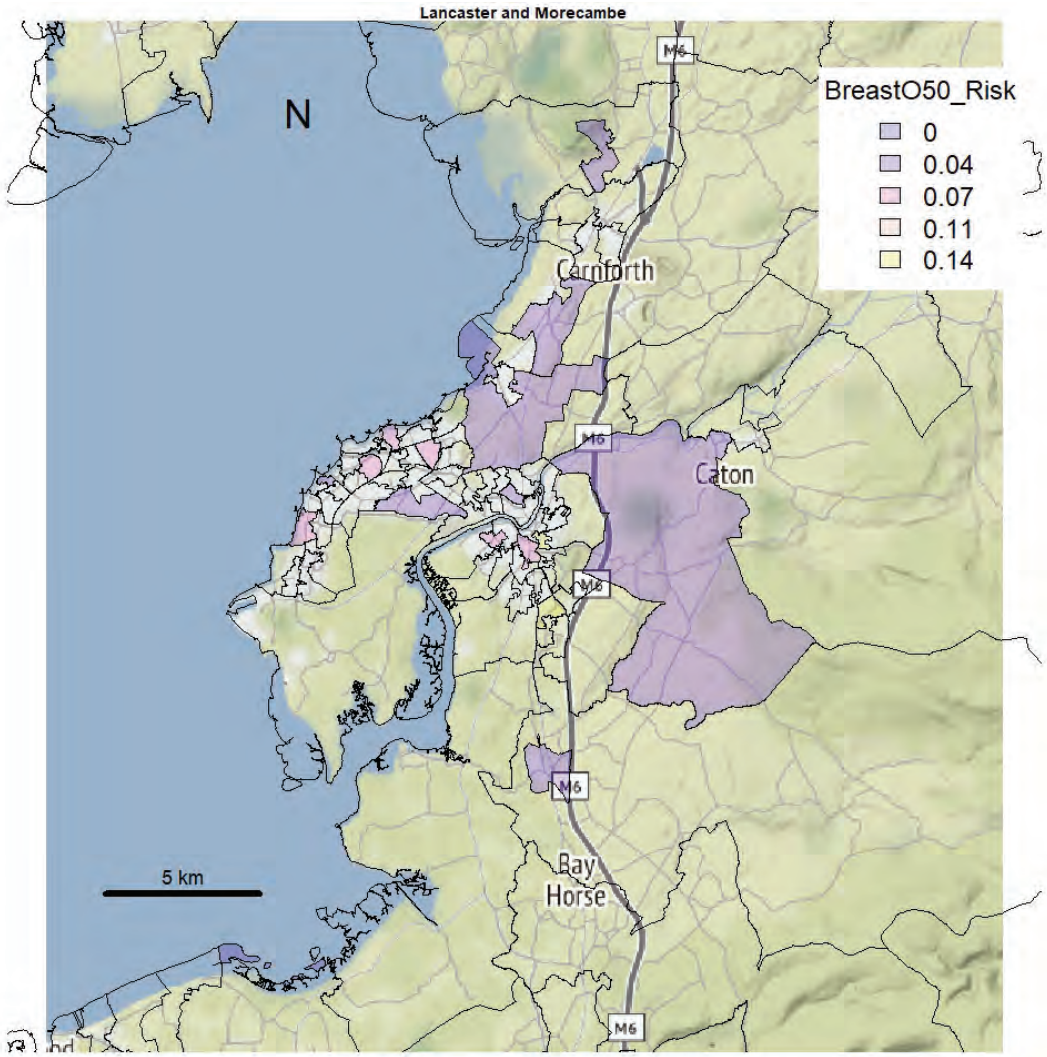
Lancaster and Morecambe
N
BreastO50_Risk
0
0.04
0.07
0.11
0.14
M6
Carnforth
Caton
Bay
Horse
5 km

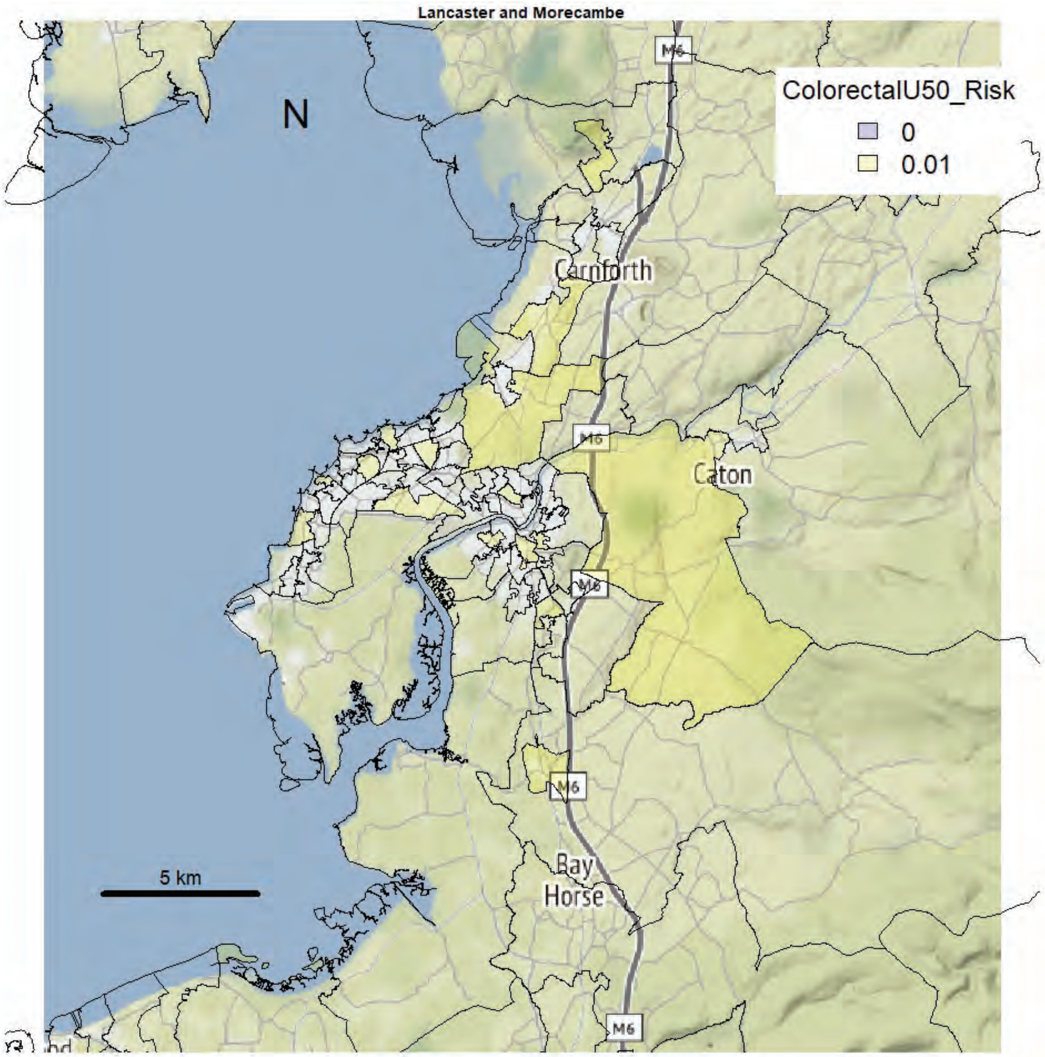
Lancaster and Morecambe
ColorectalU50_Risk
0
0.01
N
Carnforth
Caton
Bay
Horse
M6
5 km

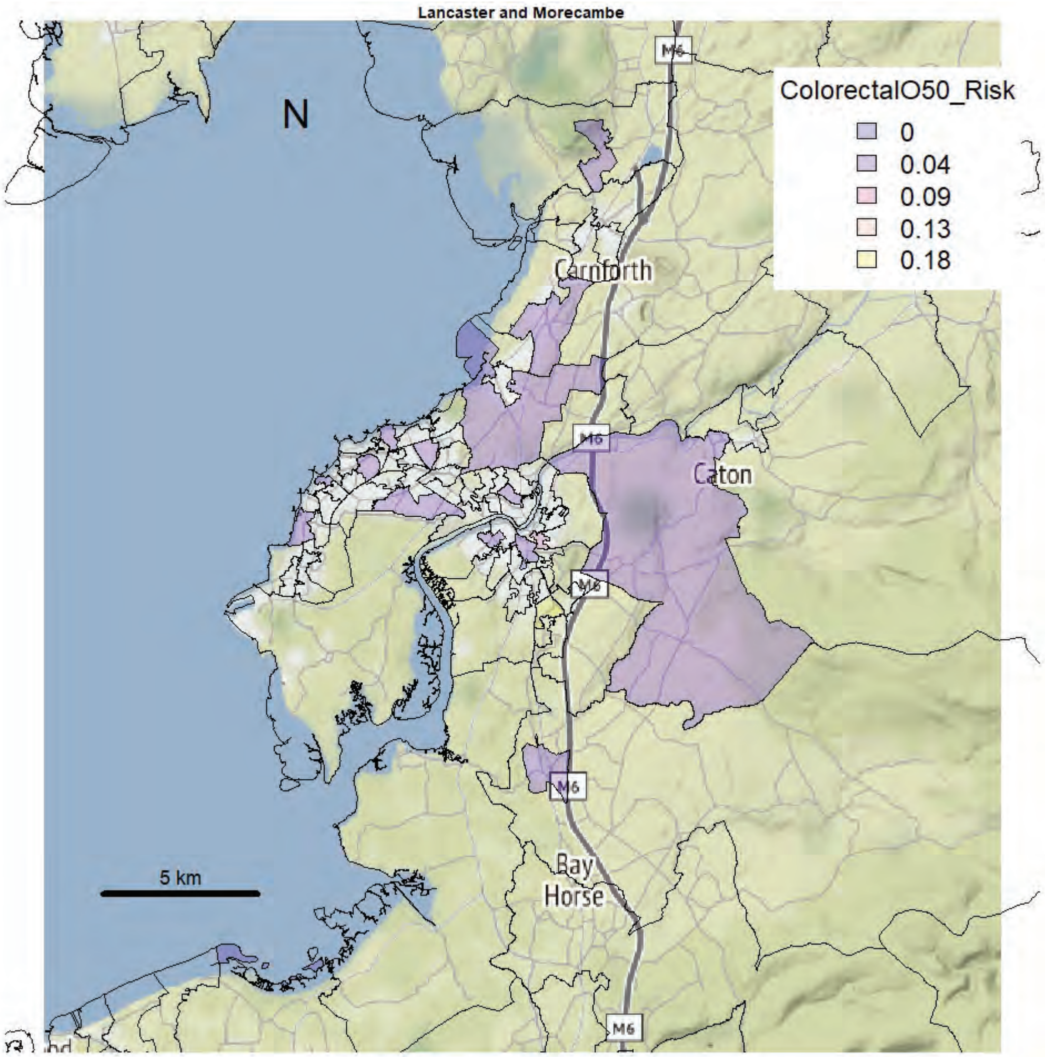
Lancaster and Morecambe
ColorectalO50_Risk
0
0.04
0.09
0.13
0.18
N
Carnforth
Caton
Bay
Horse
M6
5 km

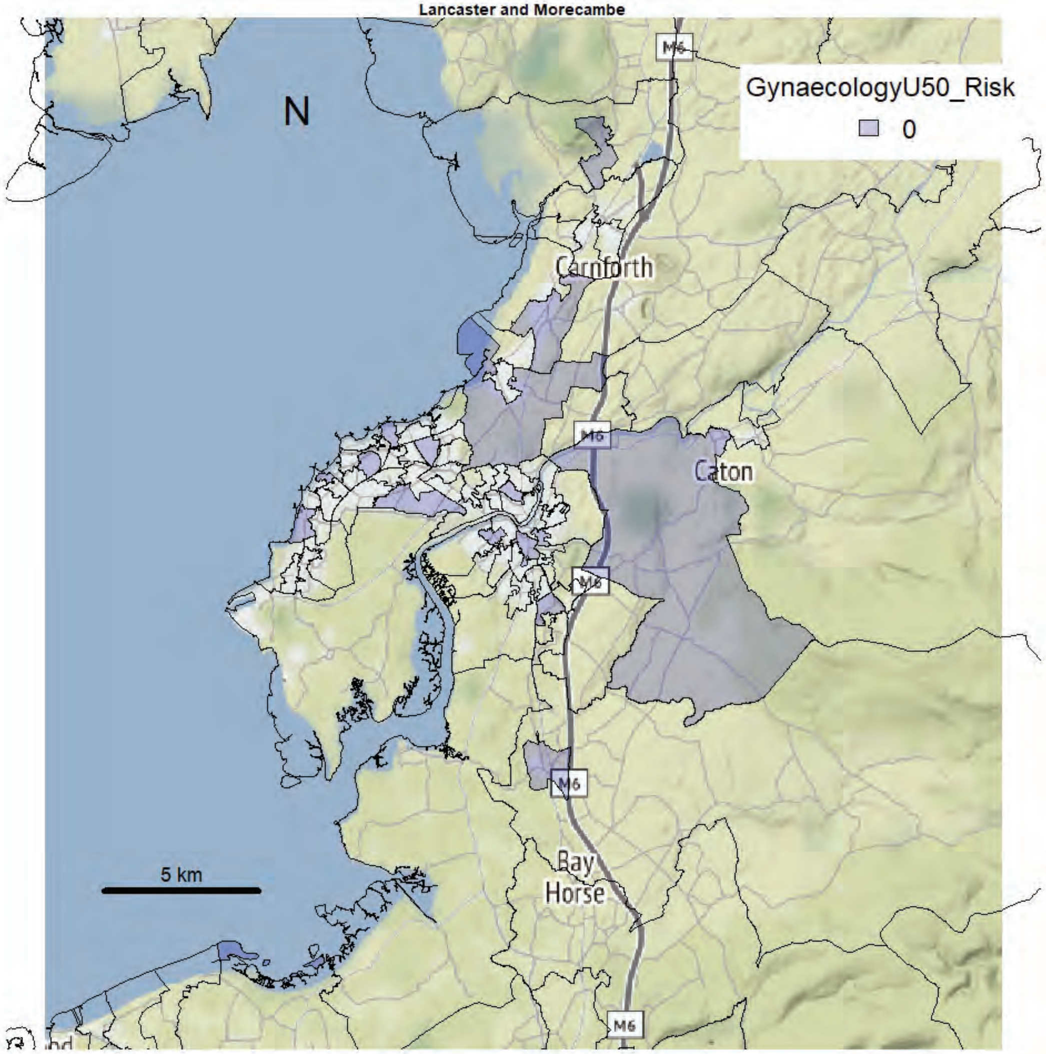
Lancaster and Morecambe
GynaecologyU50_Risk
0
N
Carnforth
Caton
Bay Horse
M6
5 km

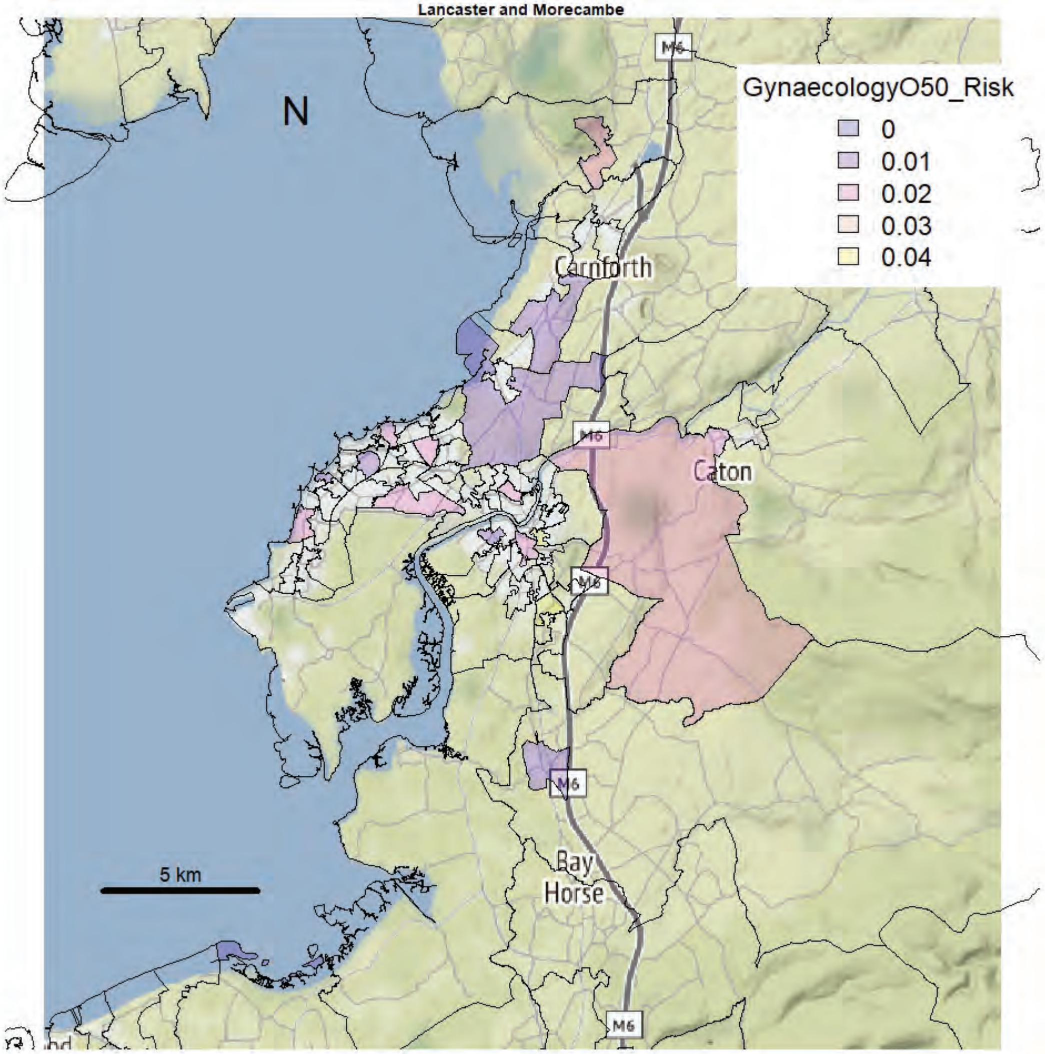
Lancaster and Morecambe
GynaecologyO50_Risk
0
0.01
0.02
0.03
0.04
N
Carnforth
Caton
Bay Horse
M6
5 km

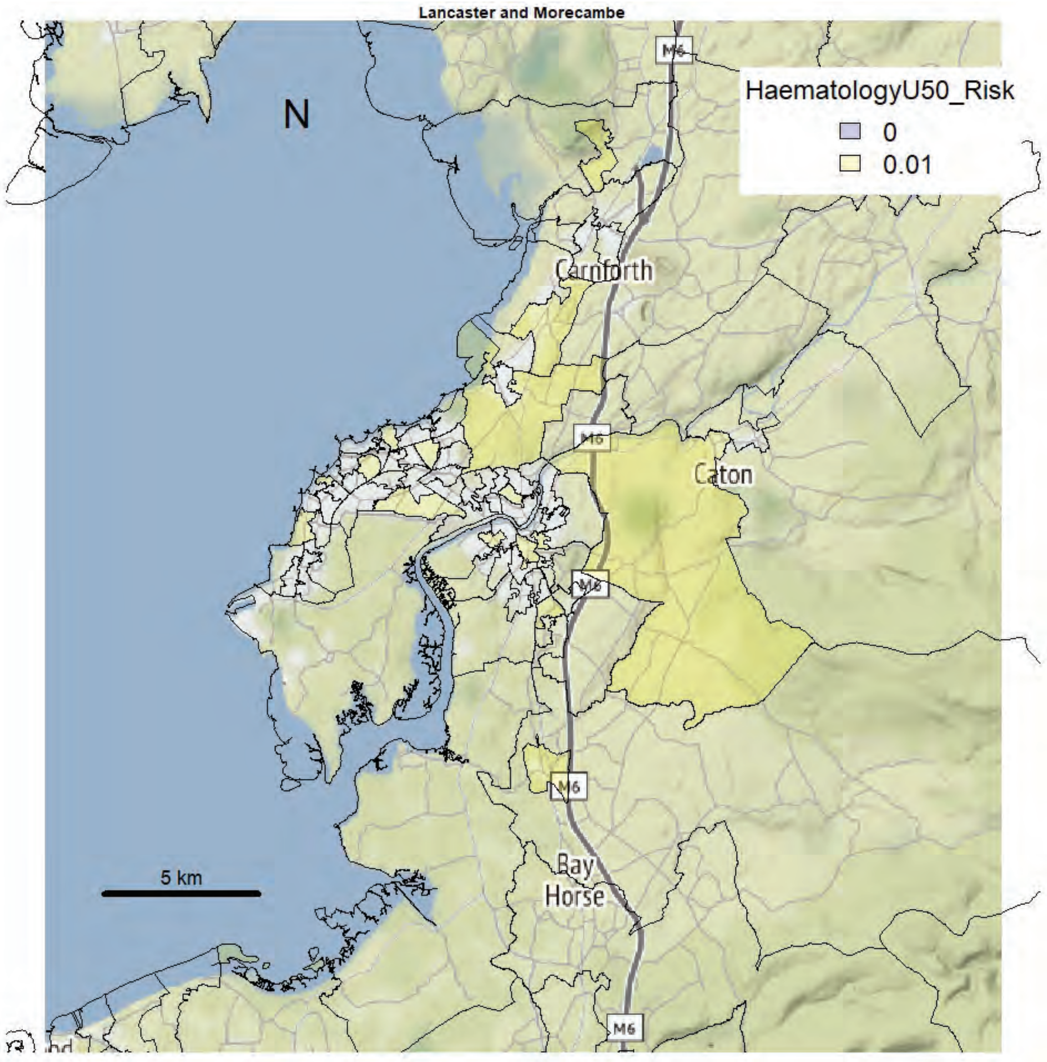
Lancaster and Morecambe
HaematologyU50_Risk
0
0.01
N
Carnforth
Caton
Bay Horse
M6
5 km

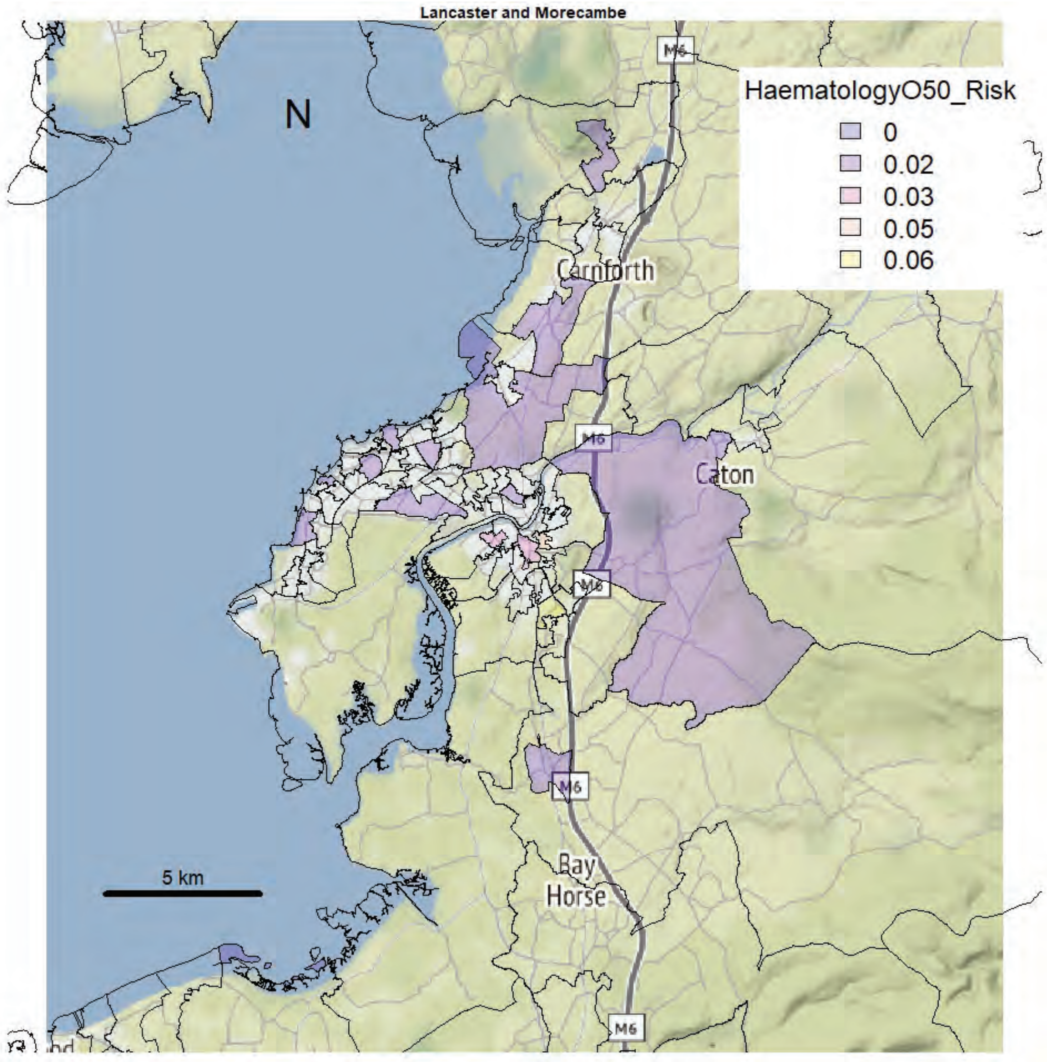
Lancaster and Morecambe
HaematologyO50_Risk
0
0.02
0.03
0.05
0.06
N
Carnforth
Caton
Bay Horse
M6
5 km

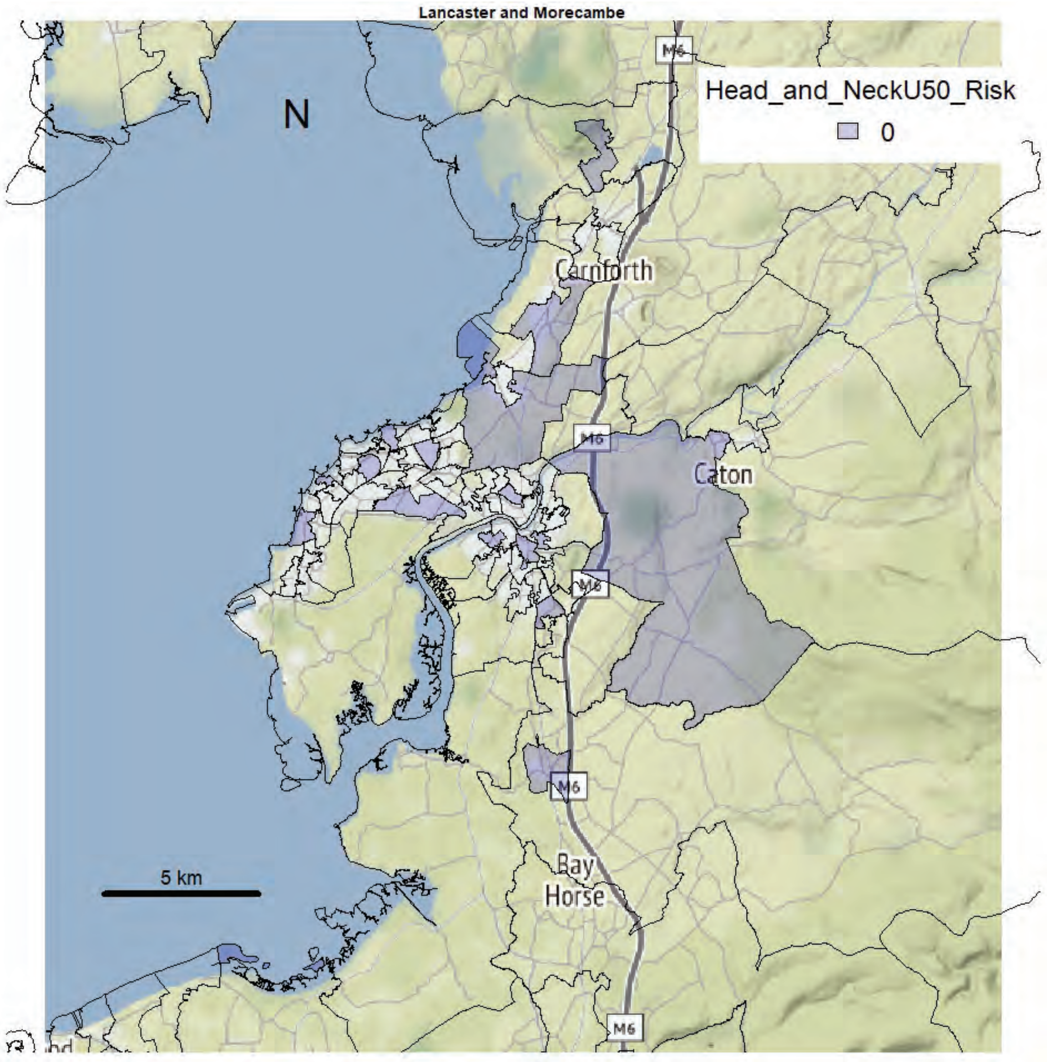
Lancaster and Morecambe
Head_and_NeckU50_Risk
0
N
Carnforth
Caton
Bay Horse
M6
5 km

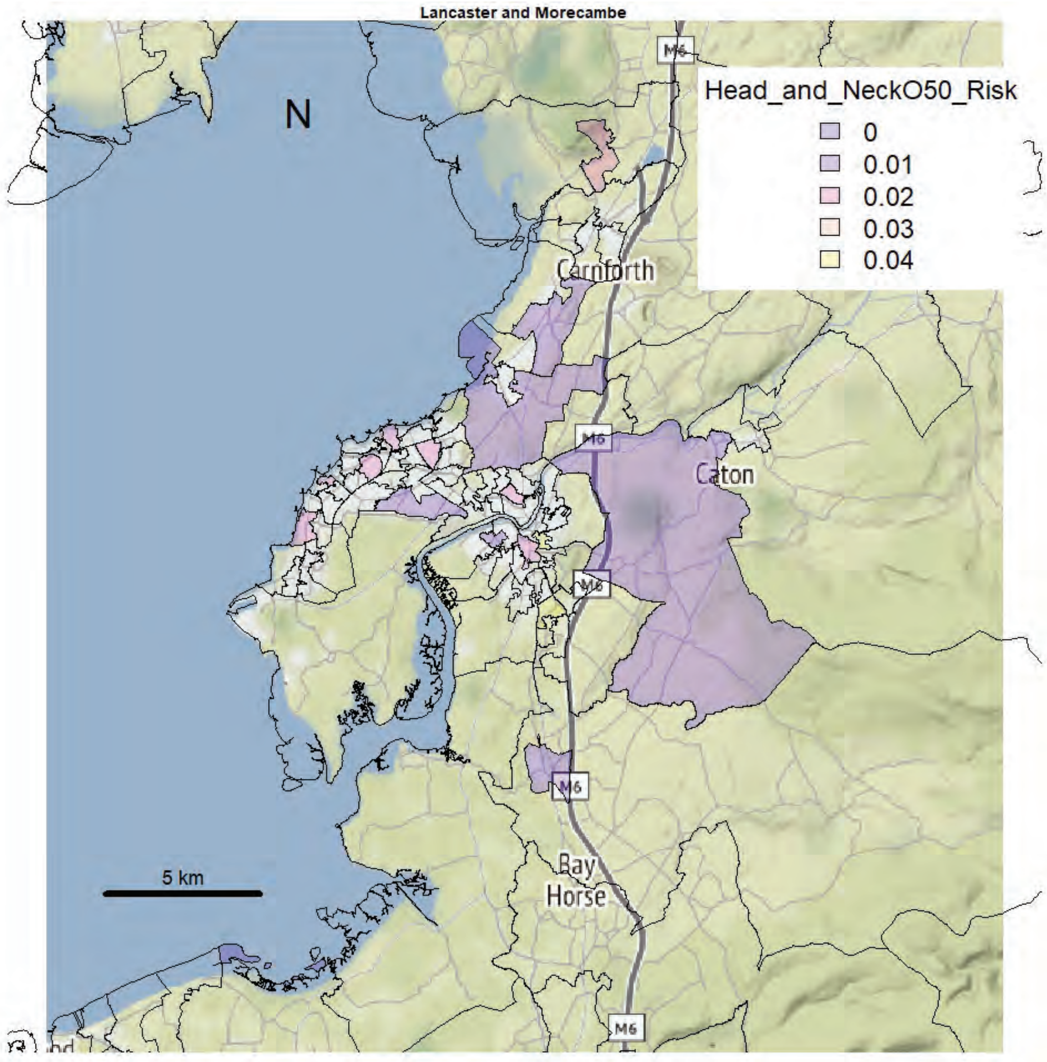
Lancaster and Morecambe
Head_and_NeckO50_Risk
0
0.01
0.02
0.03
0.04
N
Carnforth
Caton
Bay Horse
M6
5 km

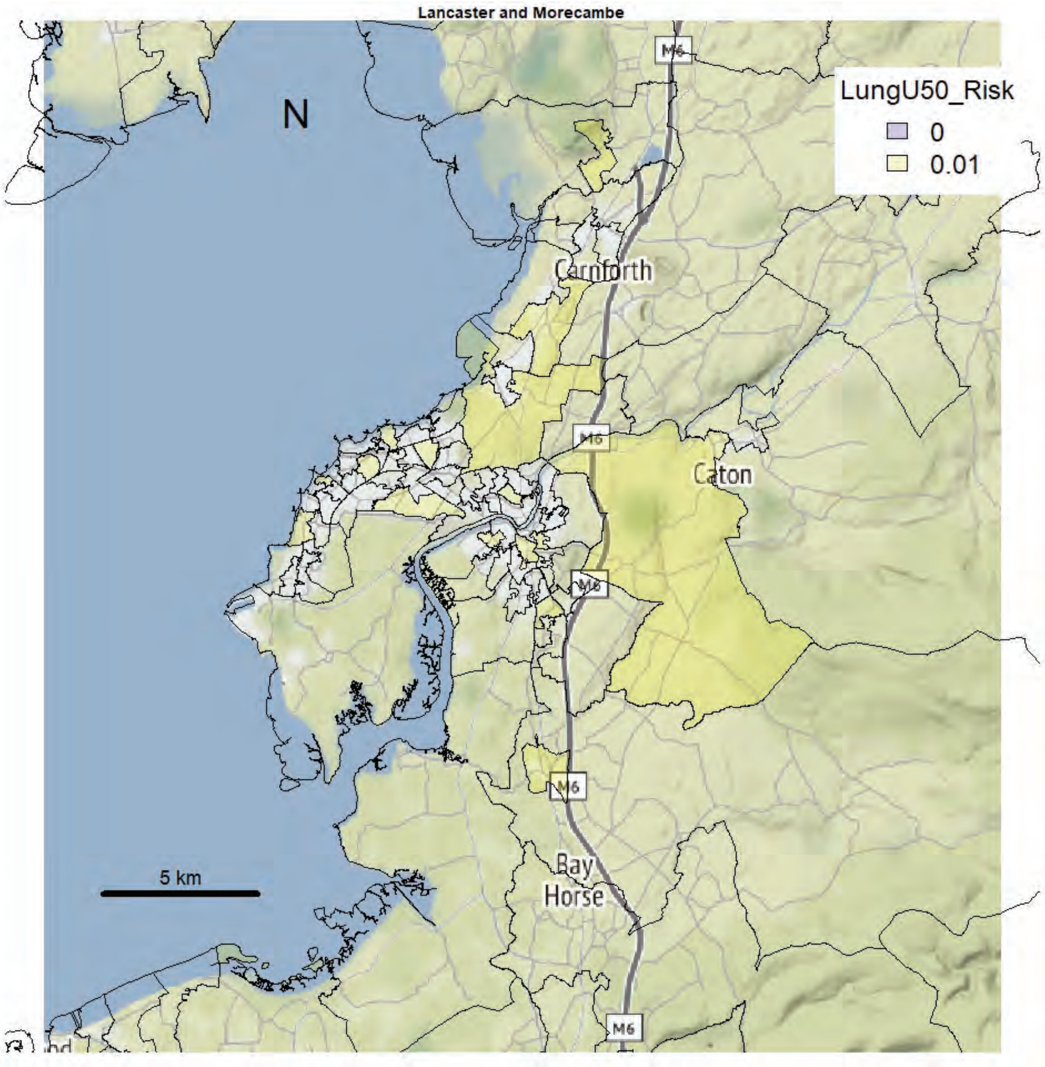
Lancaster and Morecambe
LungU50_Risk
0
0.01
N
Carnforth
Caton
M6
Bay
Horse
5 km

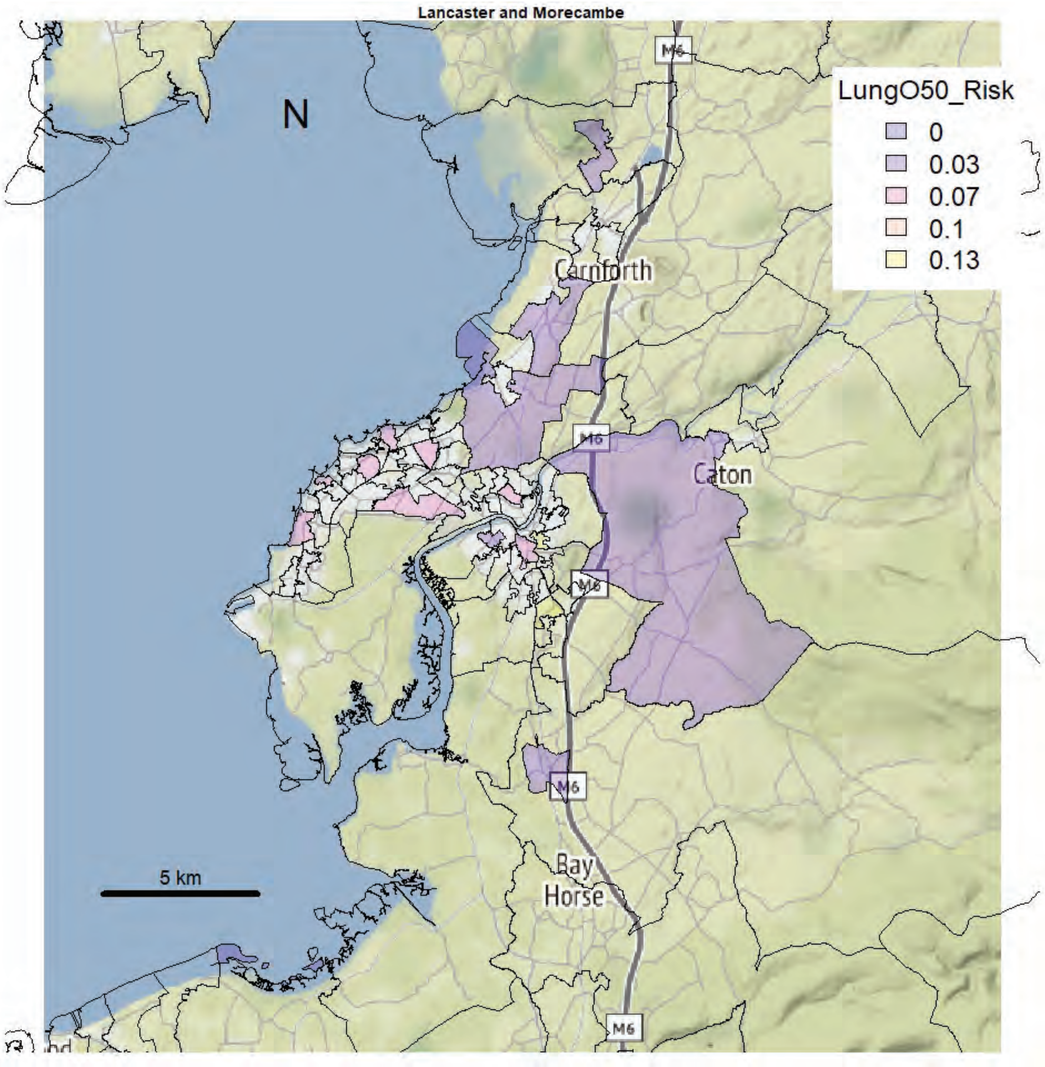
Lancaster and Morecambe
LungO50_Risk
0
0.03
0.07
0.1
0.13
N
Carnforth
Caton
M6
Bay
Horse
5 km

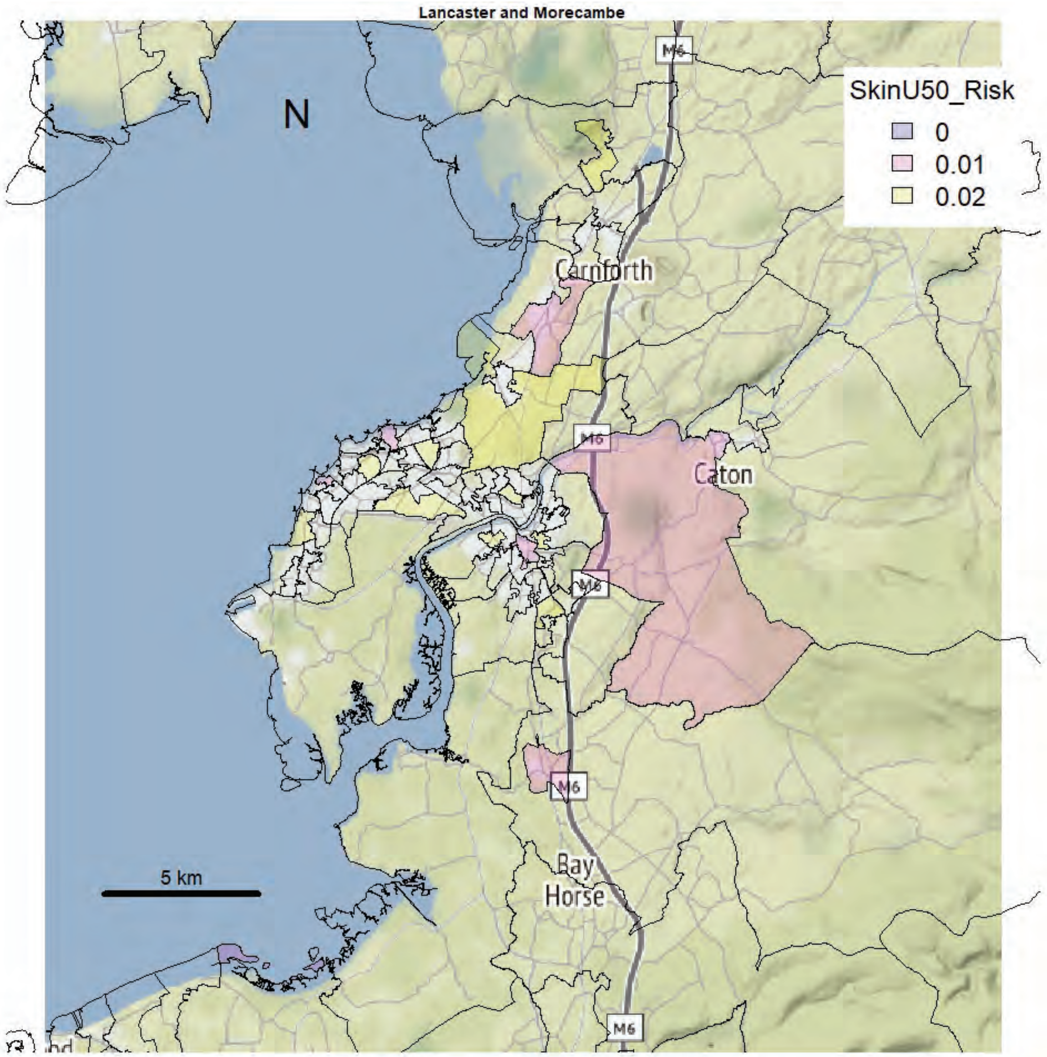
Lancaster and Morecambe
SkinU50_Risk
0
0.01
0.02
N
Carnforth
Caton
Bay Horse
M6
5 km

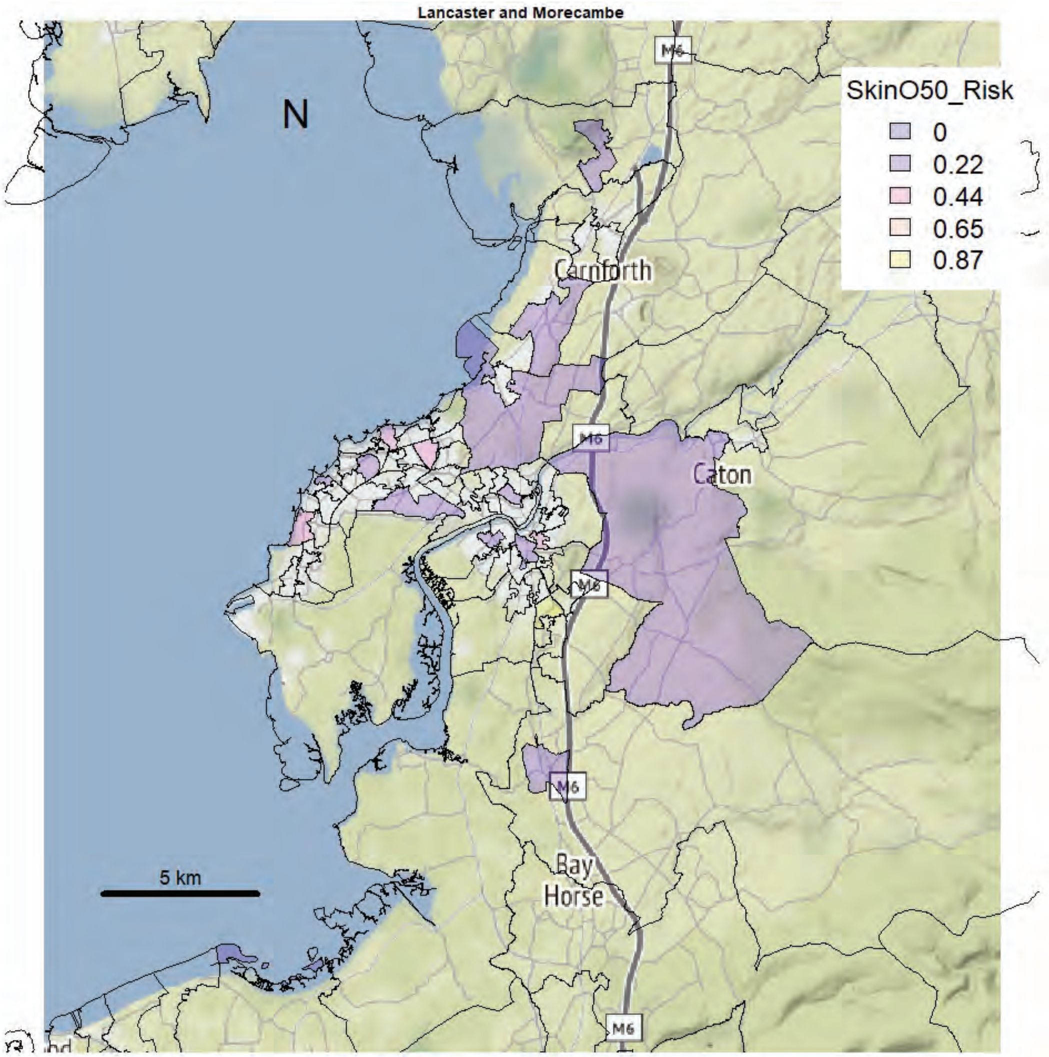
Lancaster and Morecambe
SkinO50_Risk
0
0.22
0.44
0.65
0.87
N
Carnforth
Caton
Bay Horse
M6
5 km

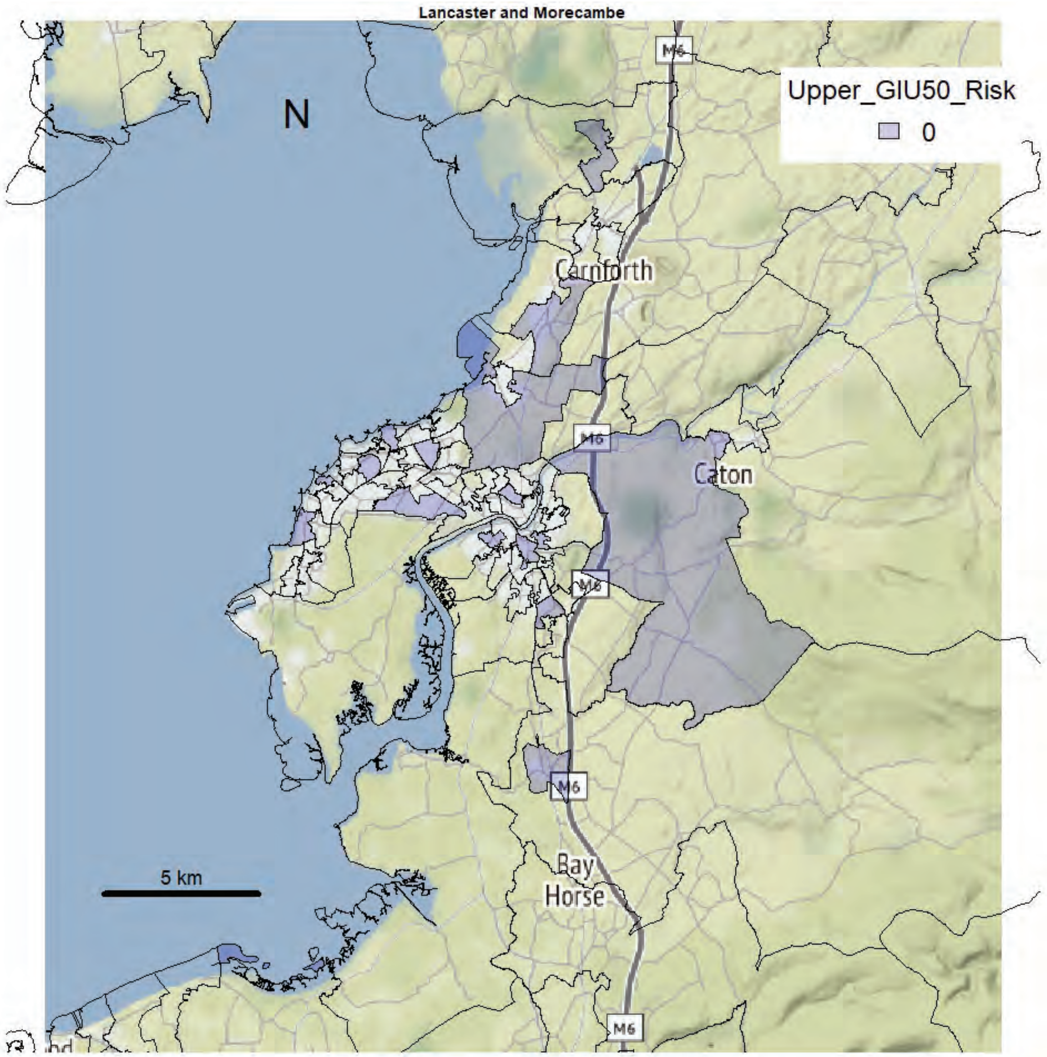
Lancaster and Morecambe
Upper_GIU50_Risk
0
N
Carnforth
Caton
Bay
Horse
M6
5 km

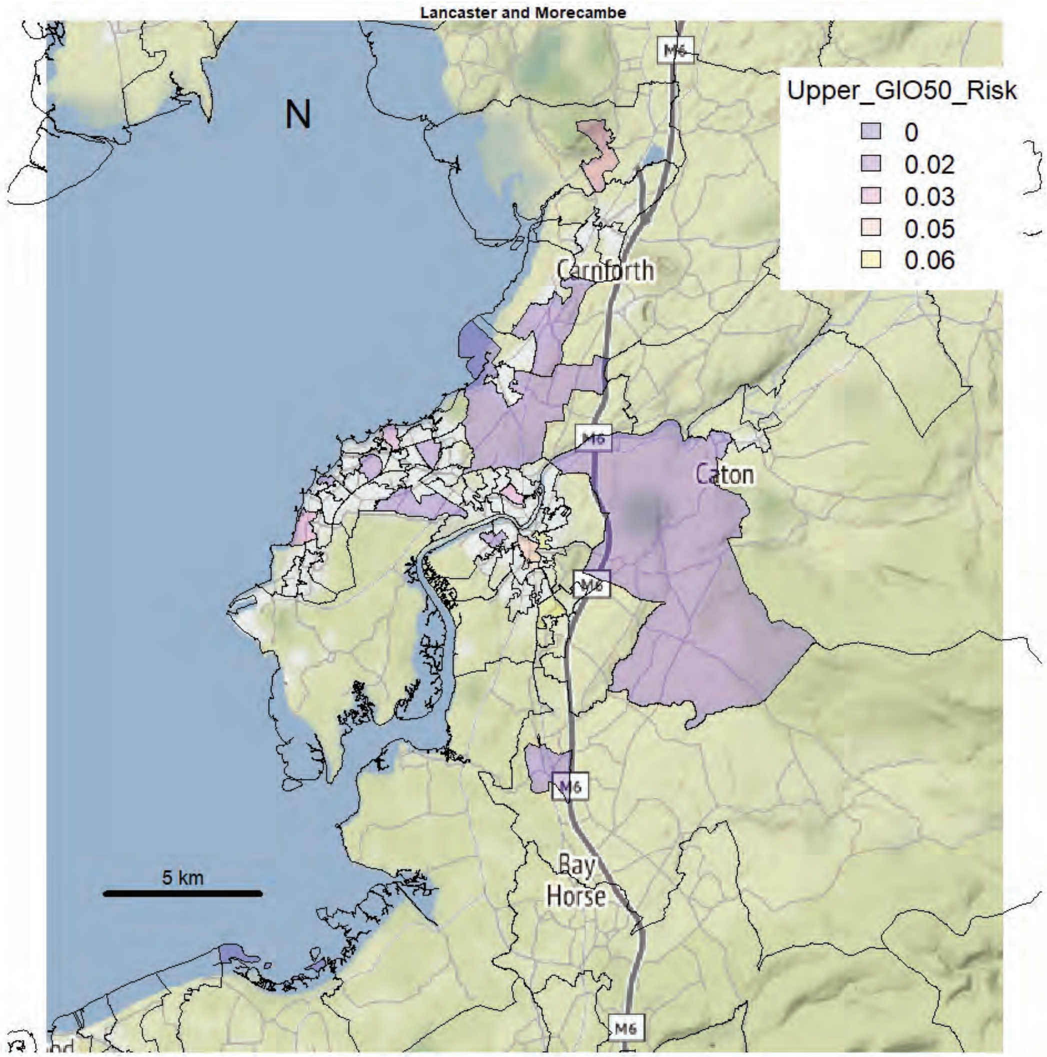
Lancaster and Morecambe
Upper_GIO50_Risk
0
0.02
0.03
0.05
0.06
N
Carnforth
Caton
Bay
Horse
M6
5 km

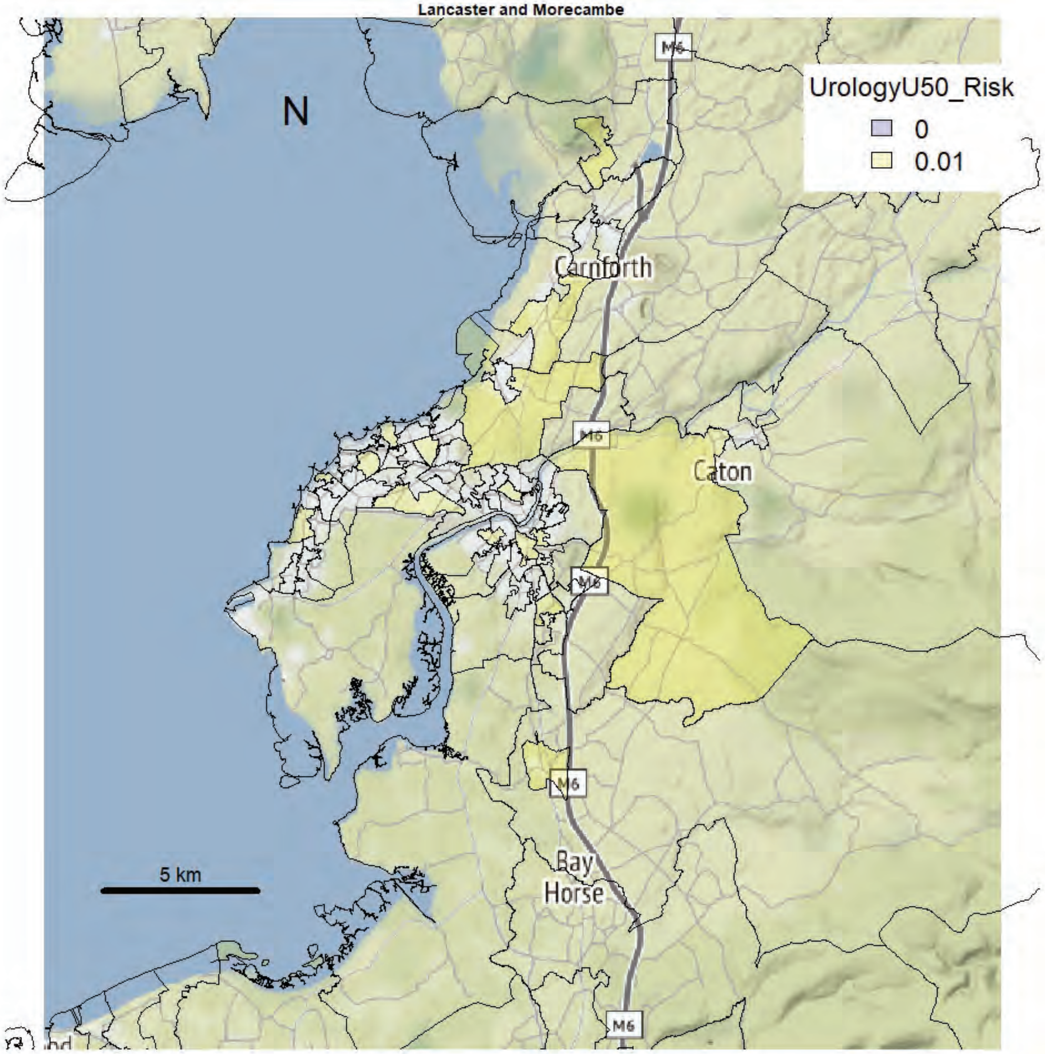
Lancaster and Morecambe
UrologyU50_Risk
0
0.01
N
Carnforth
Caton
Bay Horse
M6
5 km

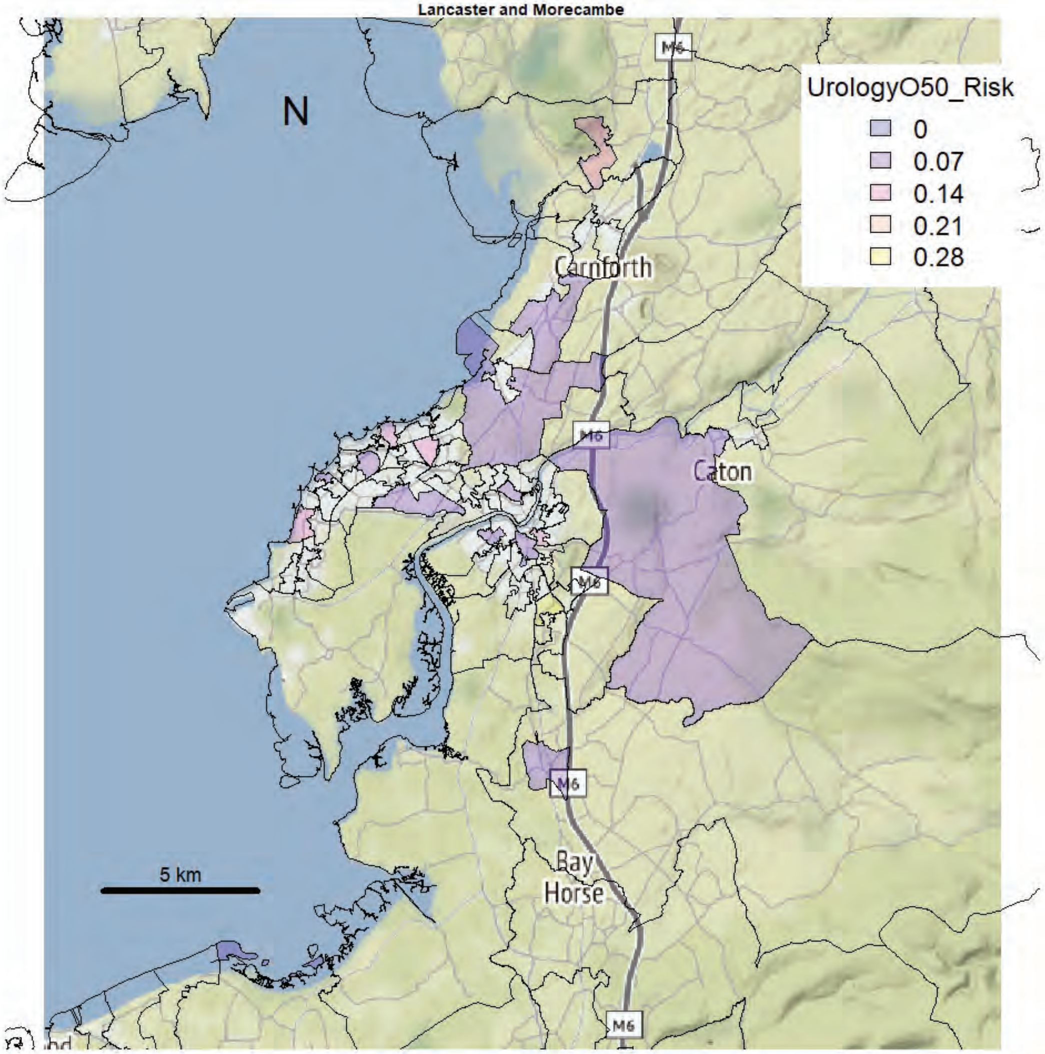
Lancaster and Morecambe
UrologyO50_Risk
0
0.07
0.14
0.21
0.28
N
Carnforth
Caton
Bay Horse
M6
5 km

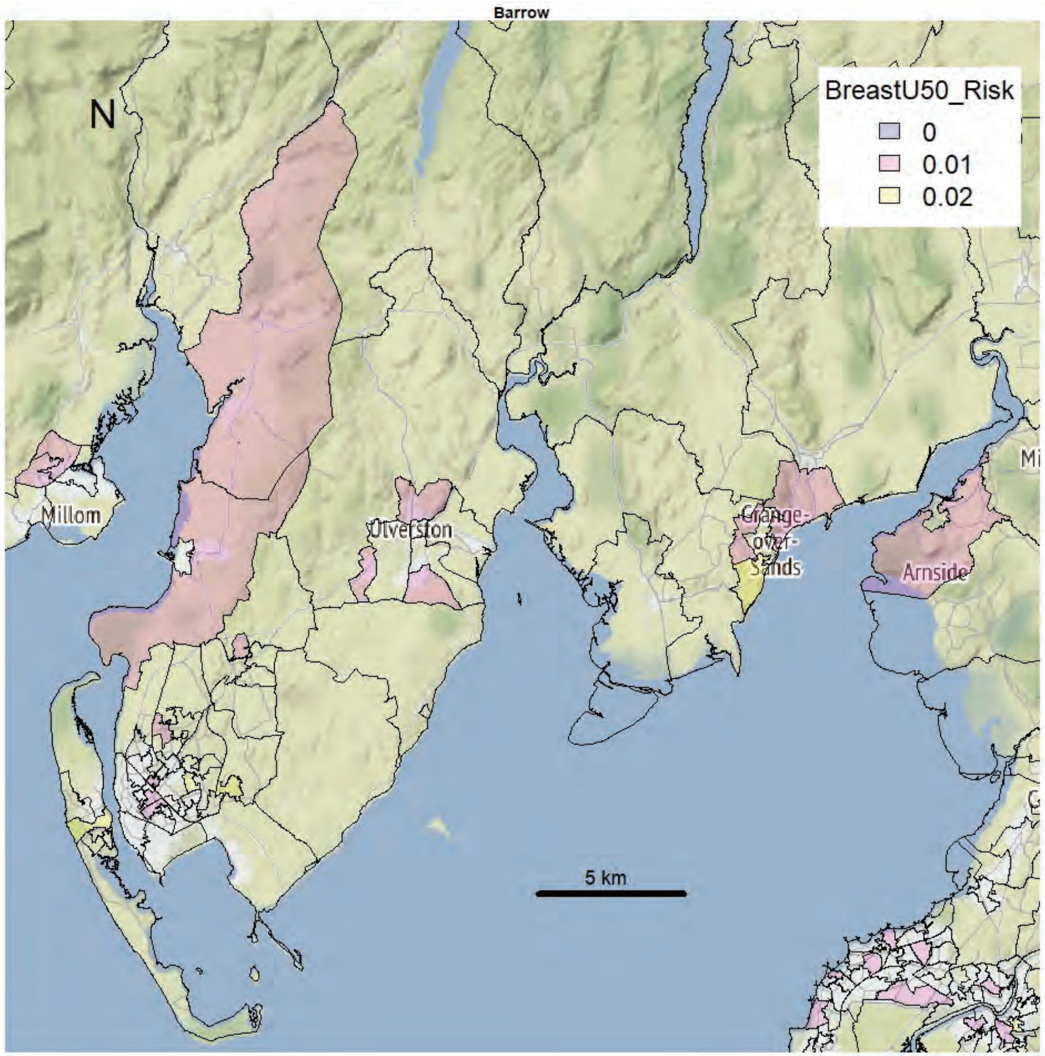
Barrow
N
BreastU50_Risk
0
0.01
0.02
Millom
Ulverston
Grange-over-Sands
Arnside
5 km

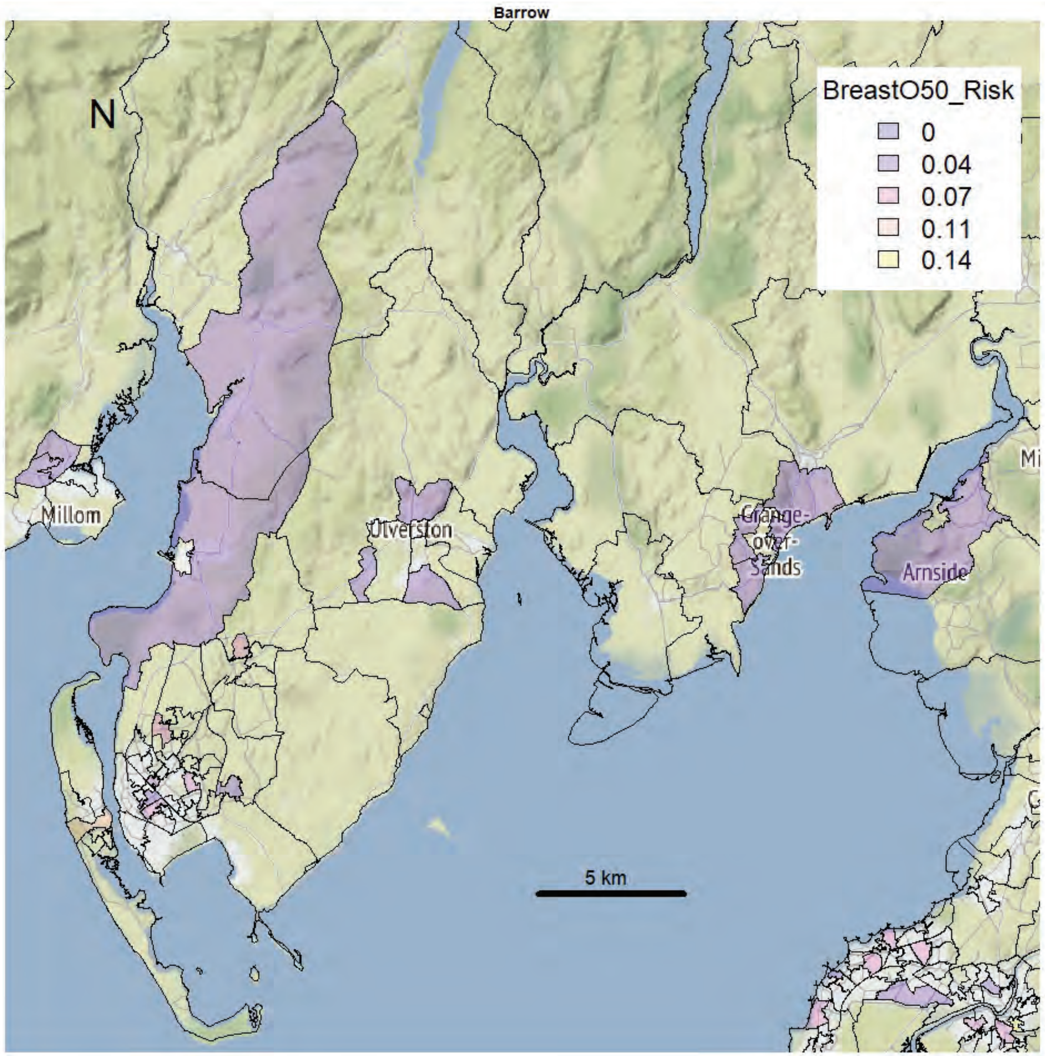
Barrow
N
BreastO50_Risk
0
0.04
0.07
0.11
0.14
Millom
Ulverston
Grange-over-Sands
Arnside
5 km

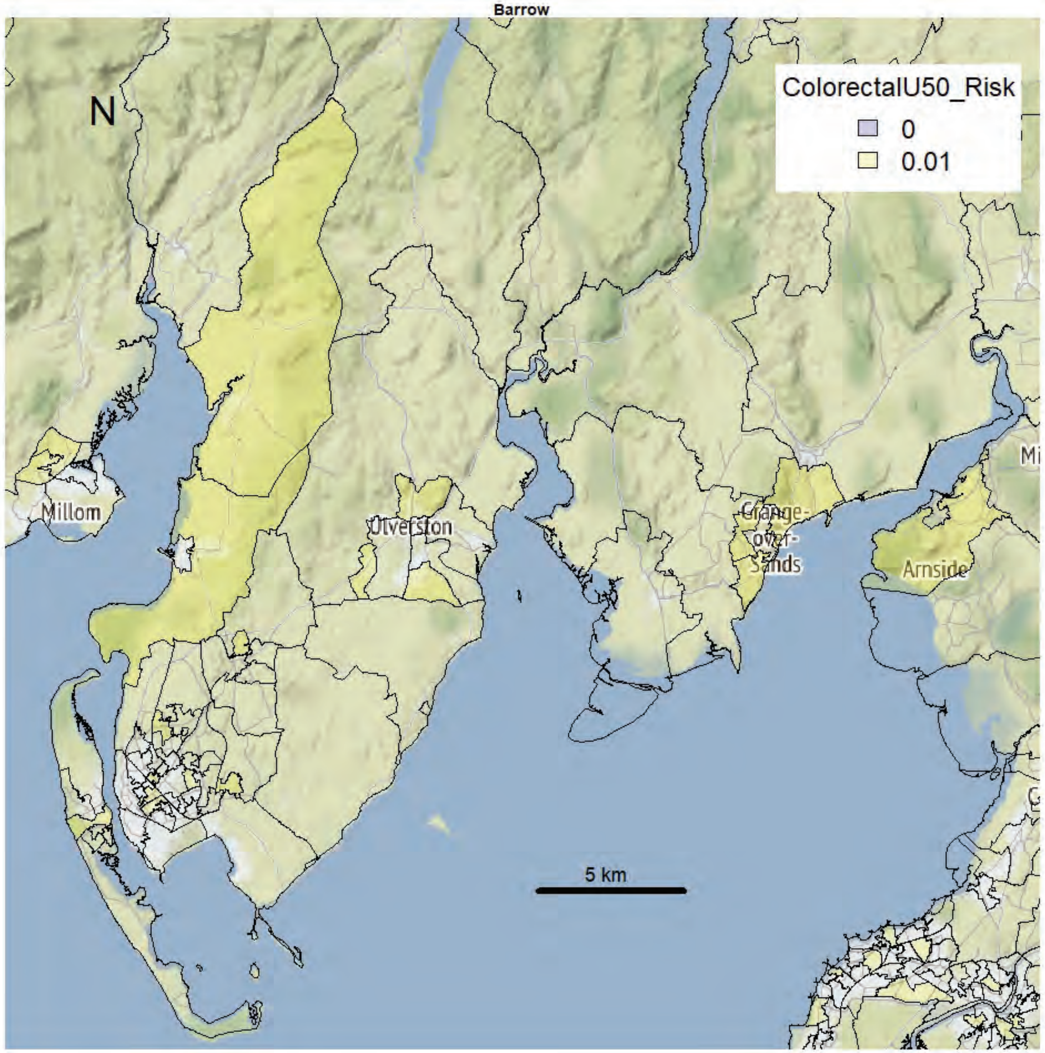
Barrow
N
ColorectalU50_Risk
0
0.01
Millom
Ulverston
Grange-over-Sands
Arnside
5 km

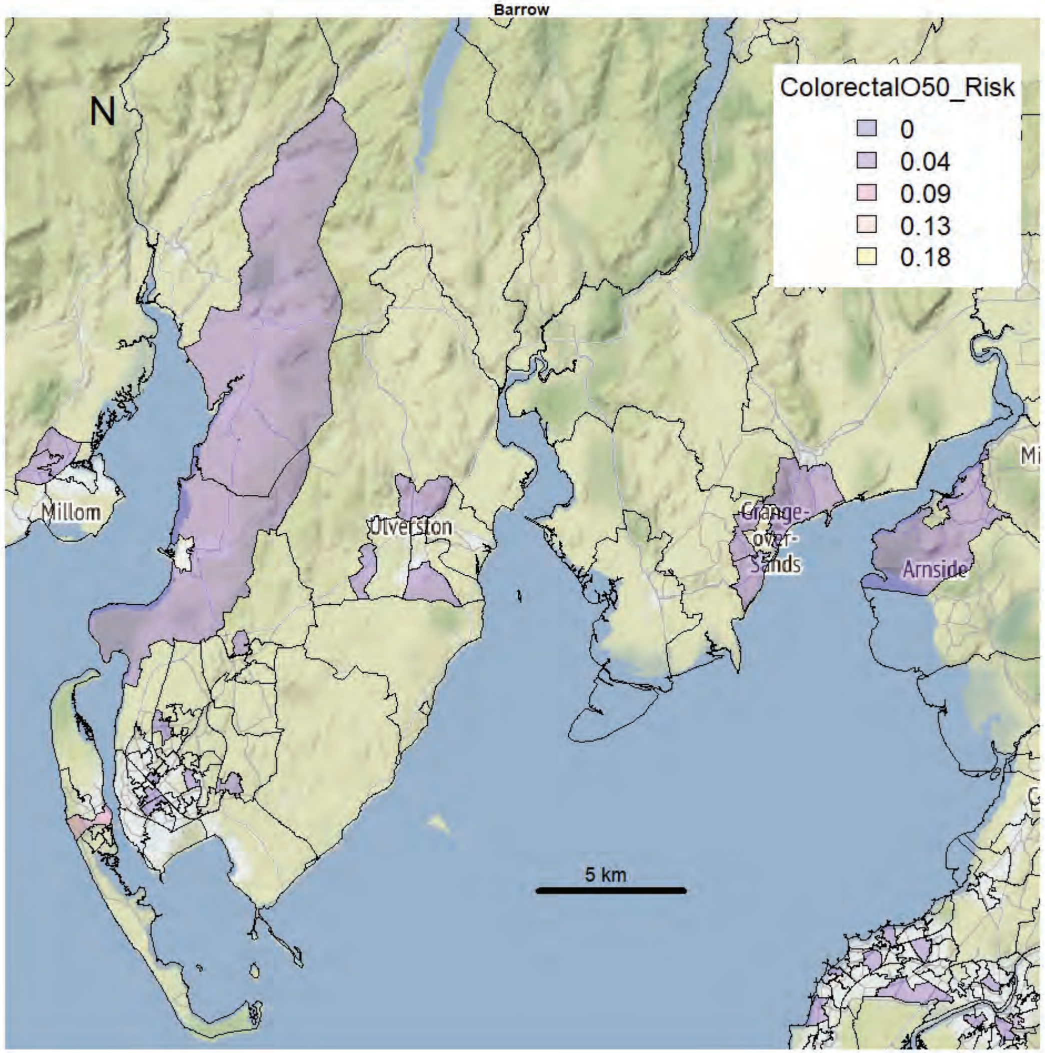
Barrow
N
ColorectalO50_Risk
0
0.04
0.09
0.13
0.18
Millom
Ulverston
Grange-over-Sands
Arnside
5 km

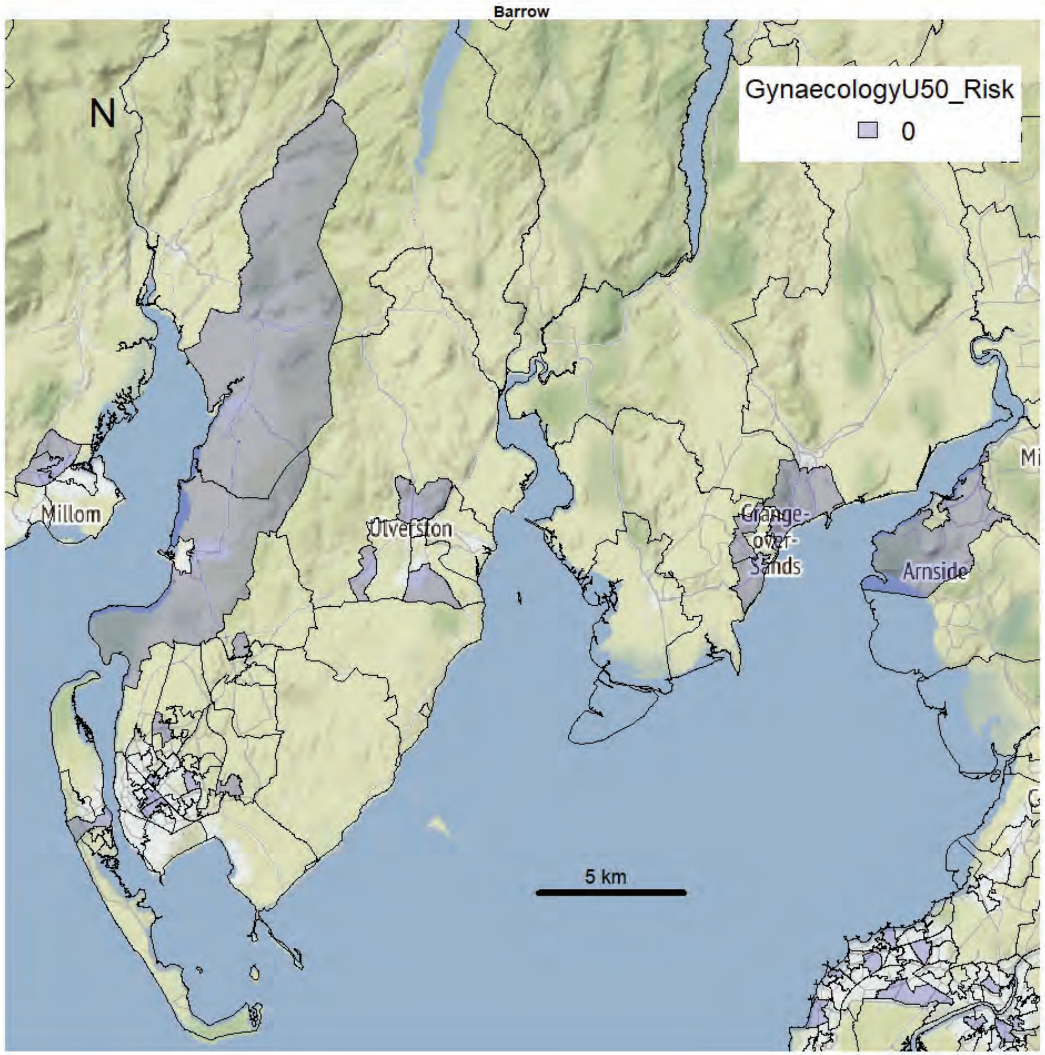
Barrow
N
GynaecologyU50_Risk
0
Millom
Ulverston
Grange-over-Sands
Arnside
5 km

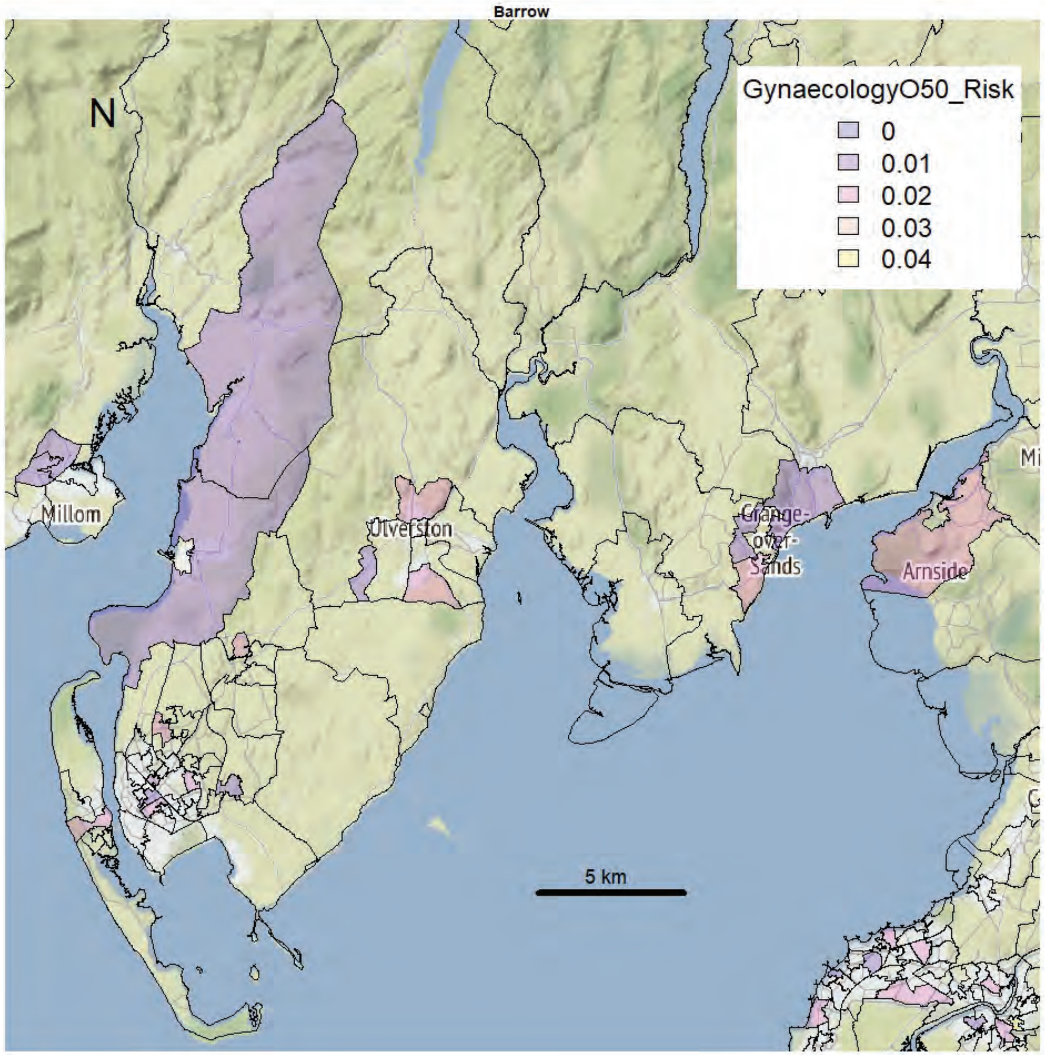
Barrow
N
GynaecologyO50_Risk
0
0.01
0.02
0.03
0.04
Millom
Ulverston
Grange-over-Sands
Arnside
5 km

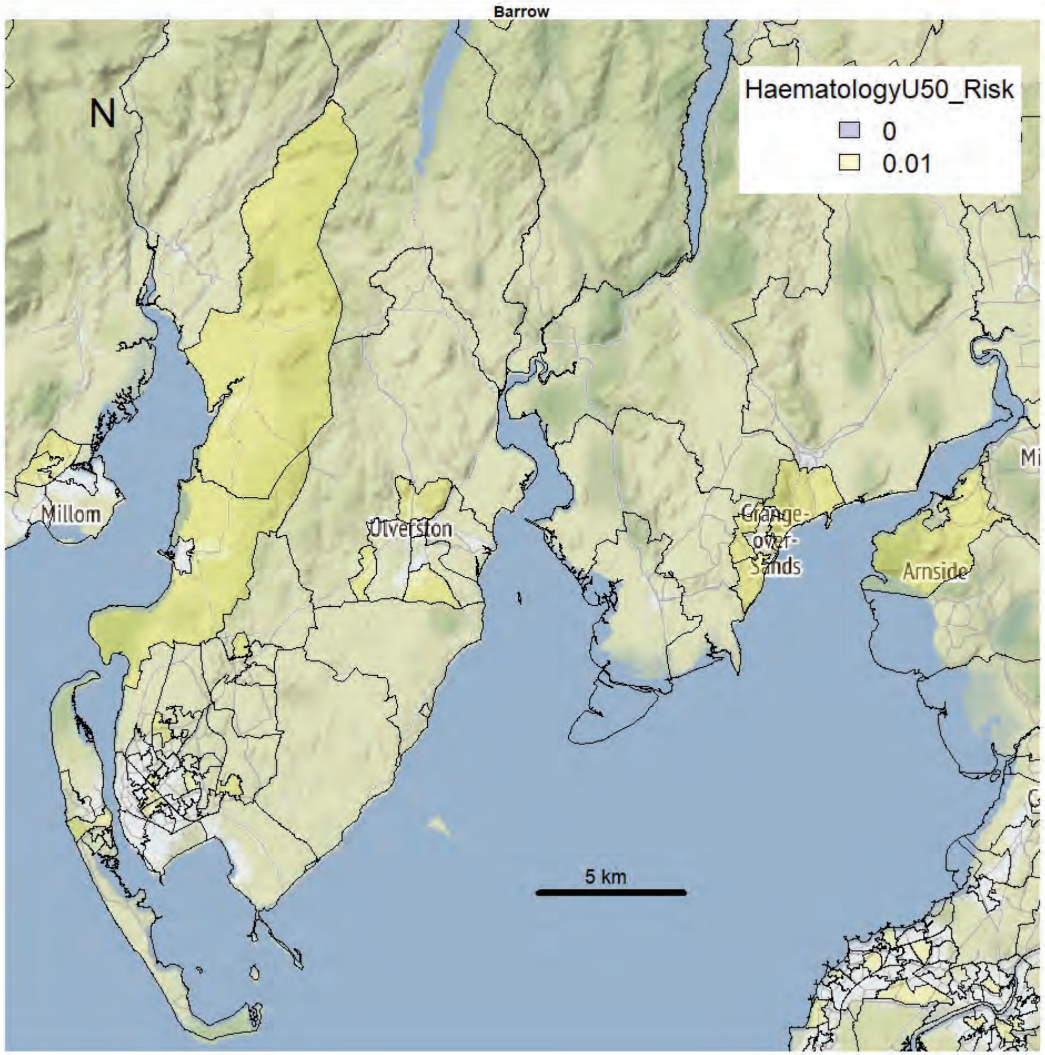
Barrow
N
HaematologyU50_Risk
0
0.01
Millom
Ulverston
Grange-over-Sands
Arnside
5 km

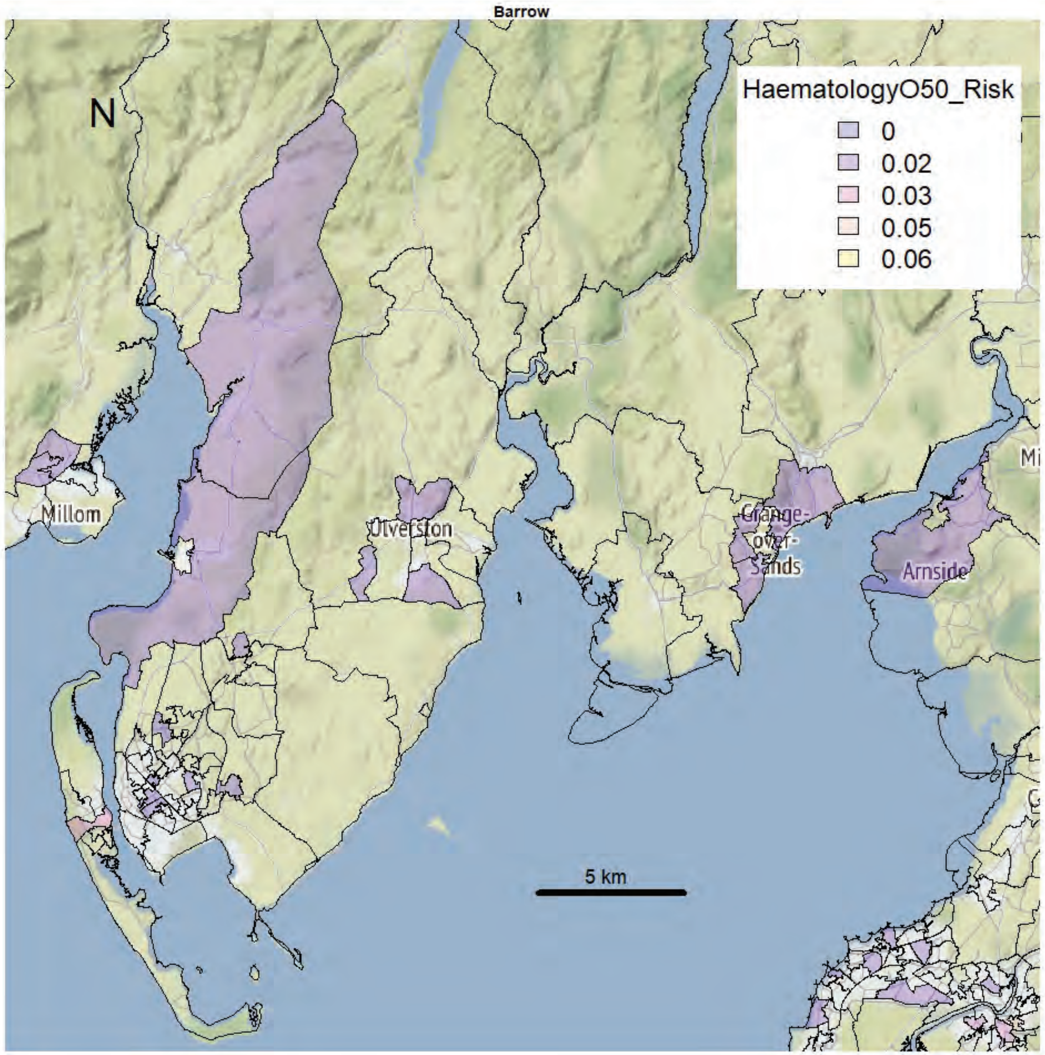
Barrow
N
HaematologyO50_Risk
0
0.02
0.03
0.05
0.06
Millom
Ulverston
Grange-over-Sands
Arnside
5 km

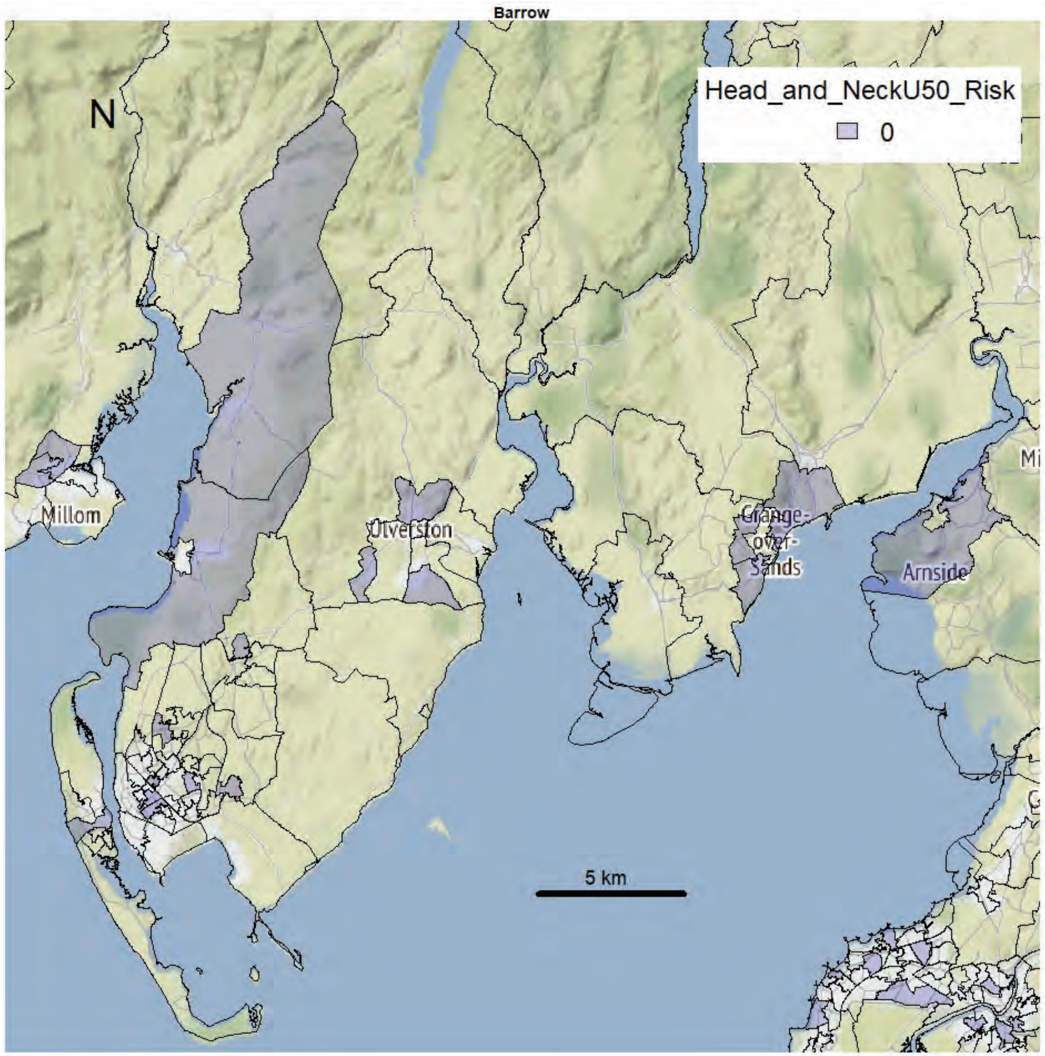

Barrow
N
Head_and_NeckU50_Risk
0
Millom
Ulverston
Grange-over-Sands
Arnside
5 km

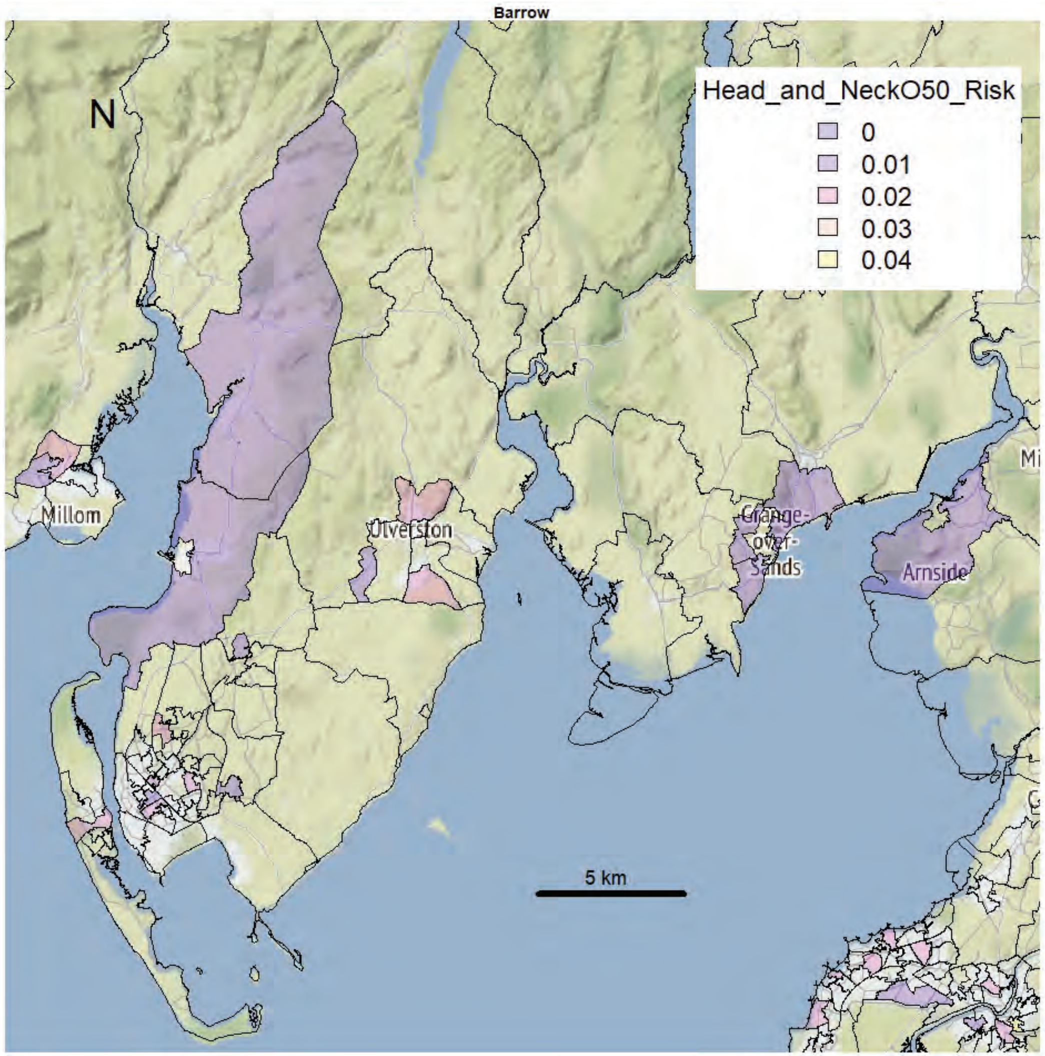

Barrow
N
Head_and_NeckO50_Risk
0
0.01
0.02
0.03
0.04
Millom
Ulverston
Grange-over-Sands
Arnside
5 km

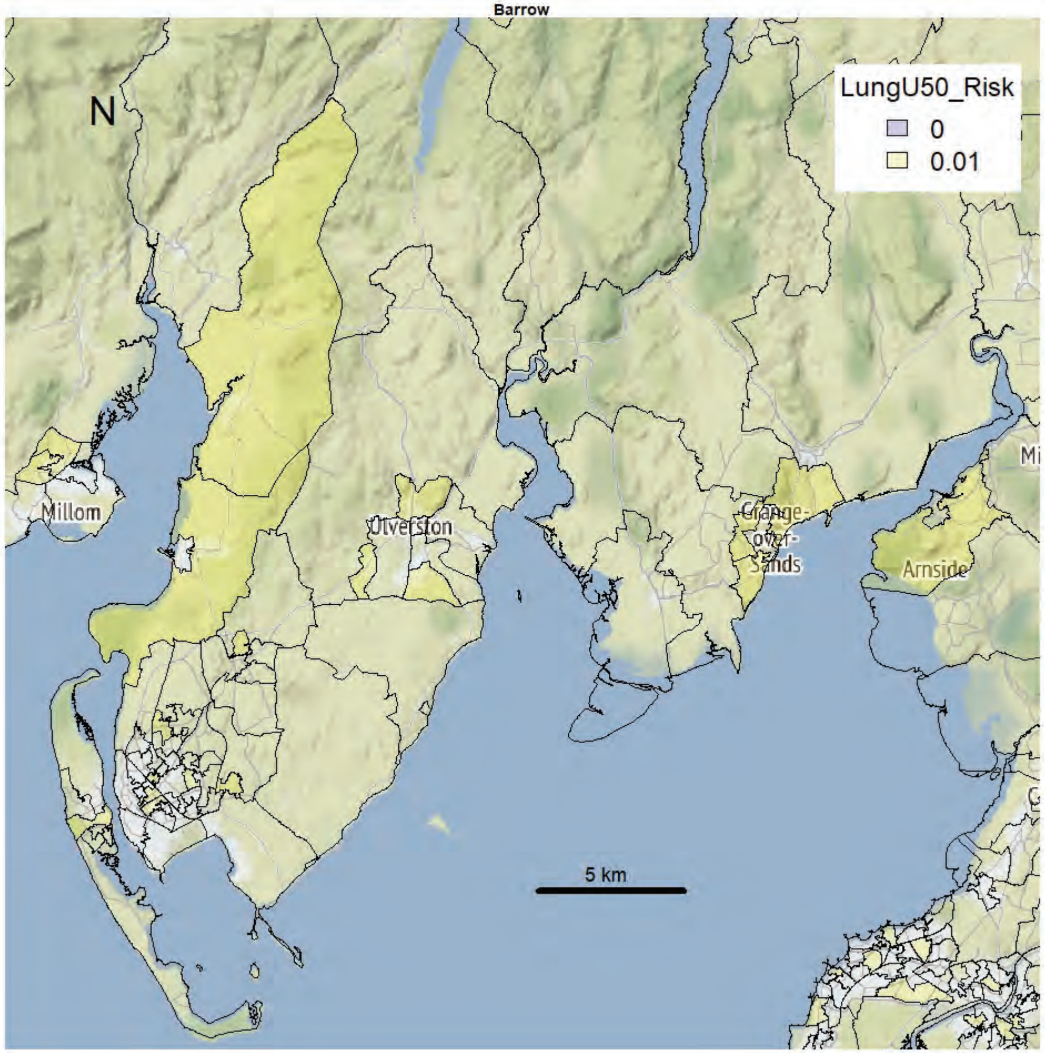
Barrow
N
LungU50_Risk
0
0.01
Millom
Ulverston
Grange-over-Sands
Arnside
5 km

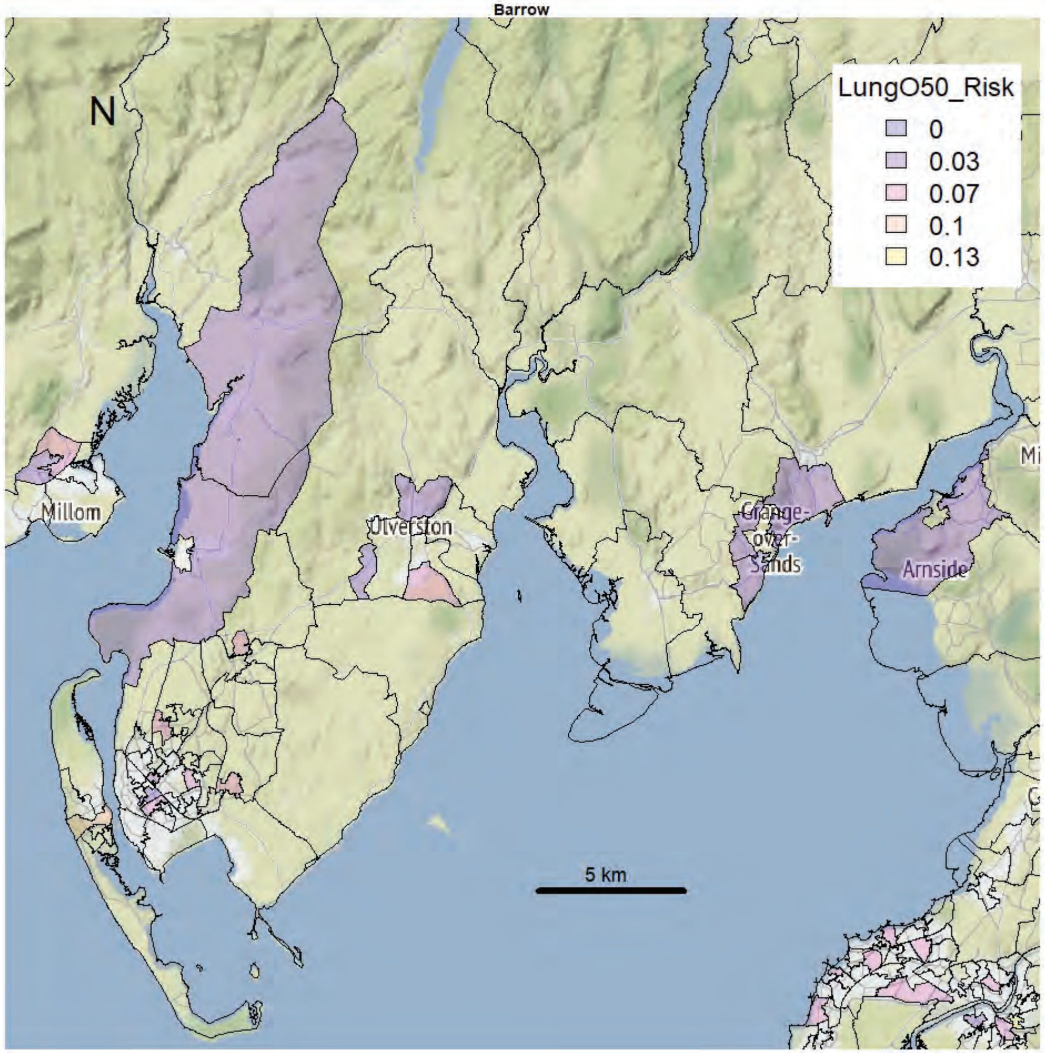
Barrow
N
LungO50_Risk
0
0.03
0.07
0.1
0.13
Millom
Ulverston
Grange-over-Sands
Arnside
5 km

Barrow

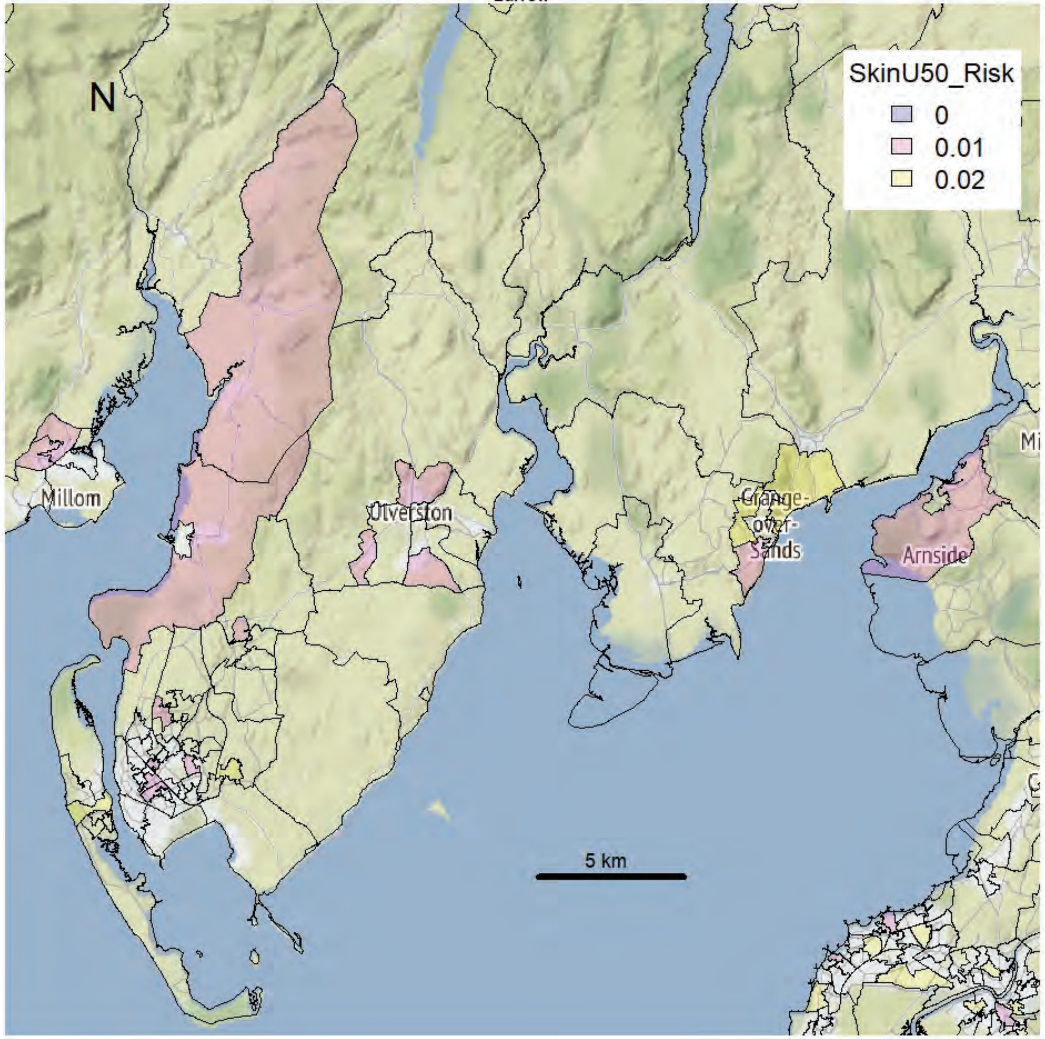

Barrow

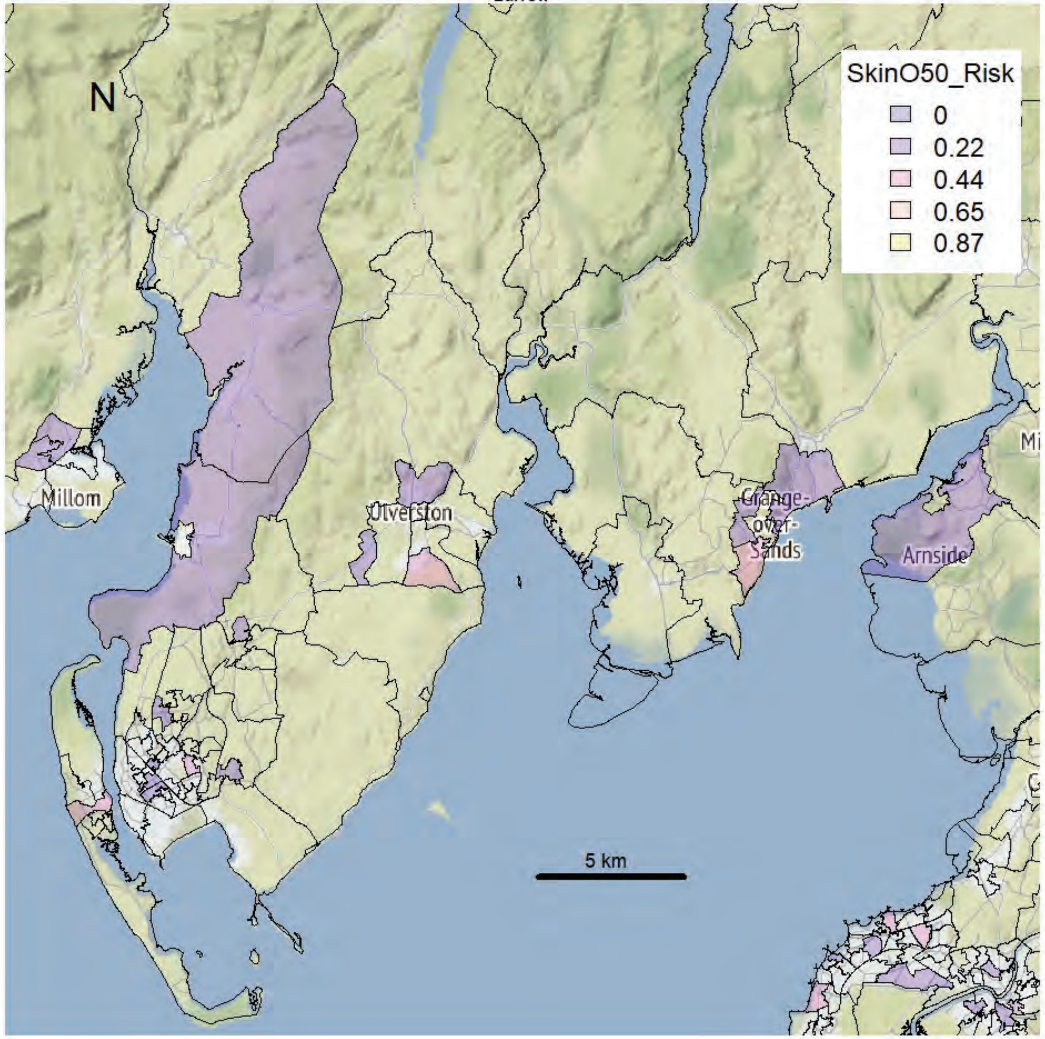

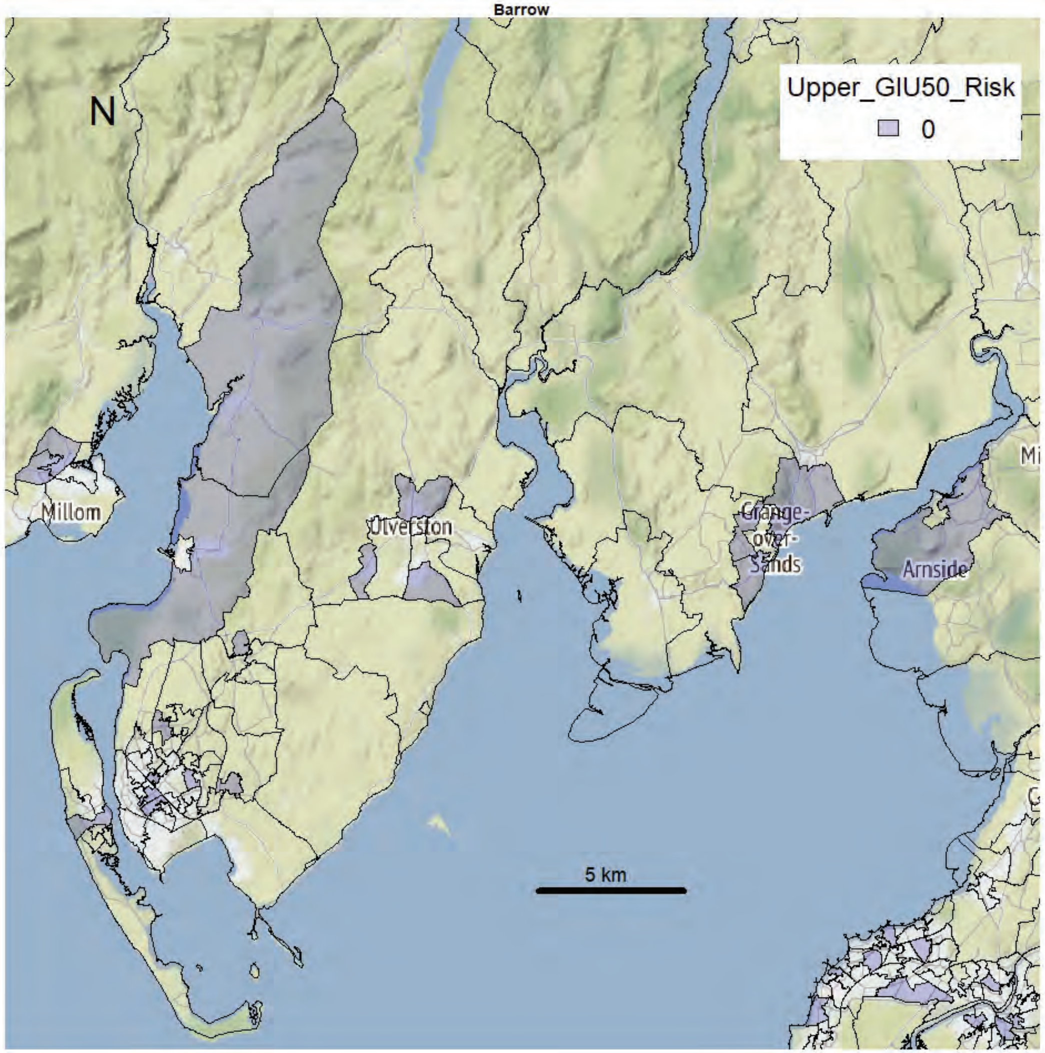
Barrow
N
Upper_GIU50_Risk
0
Millom
Ulverston
Grange-over-Sands
Arnside
5 km

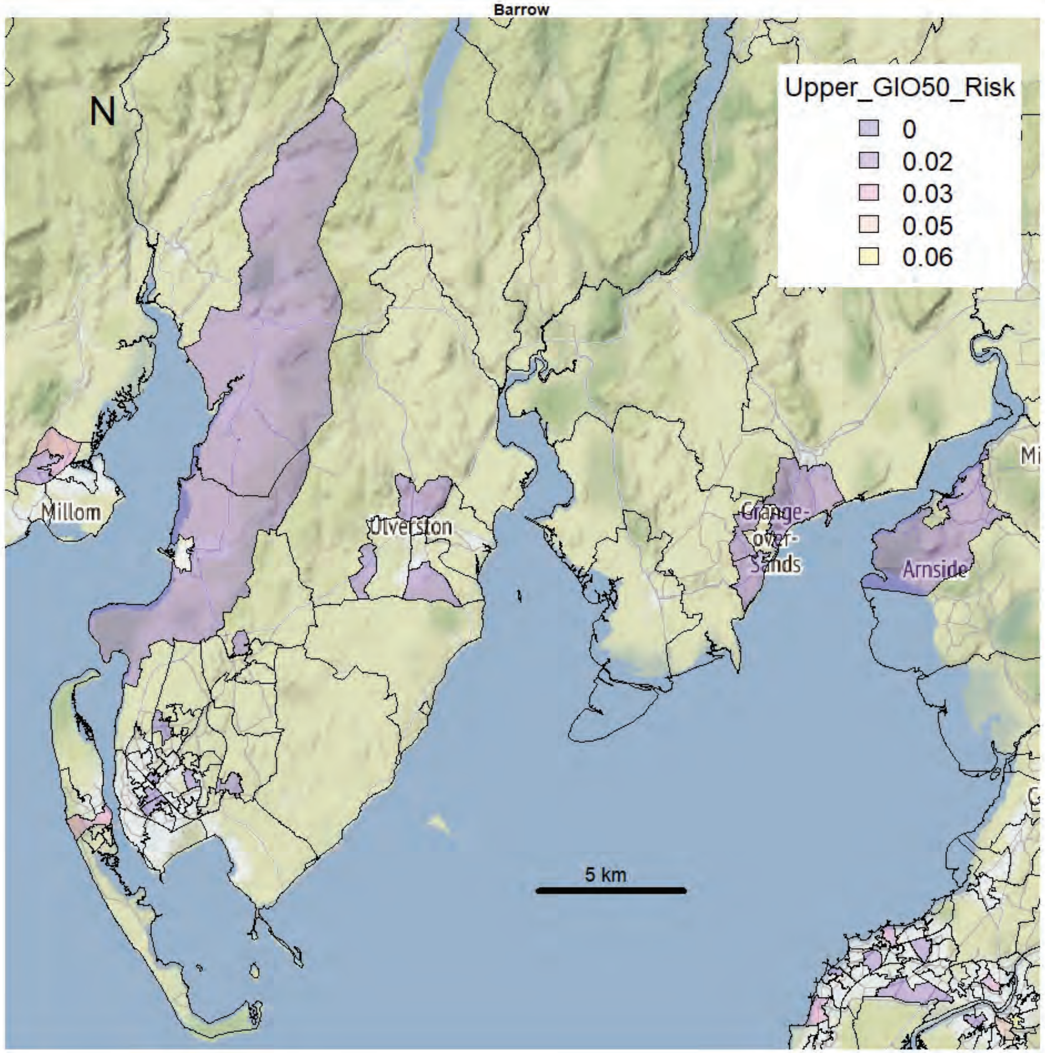
Barrow
N
Upper_GIO50_Risk
0
0.02
0.03
0.05
0.06
Millom
Ulverston
Grange-over-Sands
Arnside
5 km

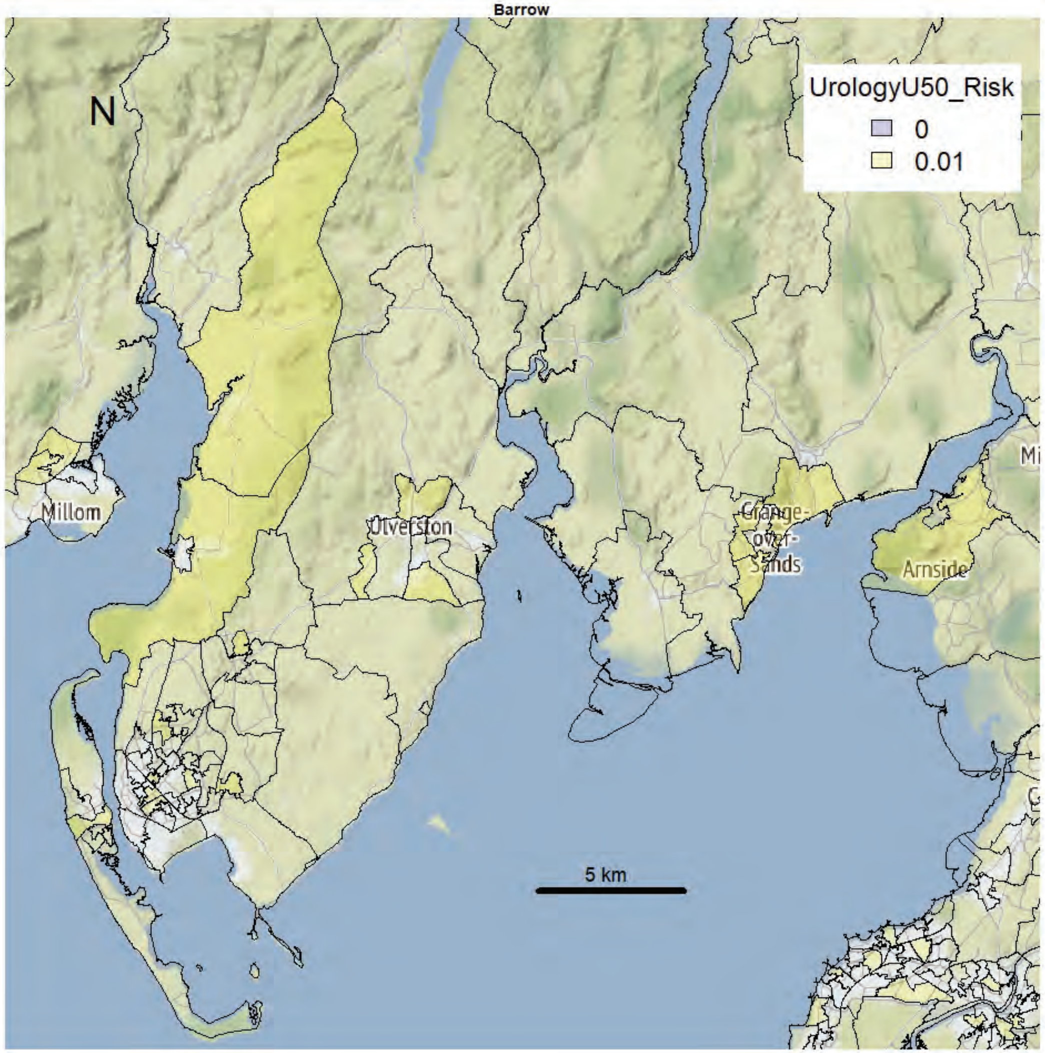
Barrow
N
UrologyU50_Risk
0
0.01
Millom
Ulverston
Grange-over-Sands
Arnside
5 km

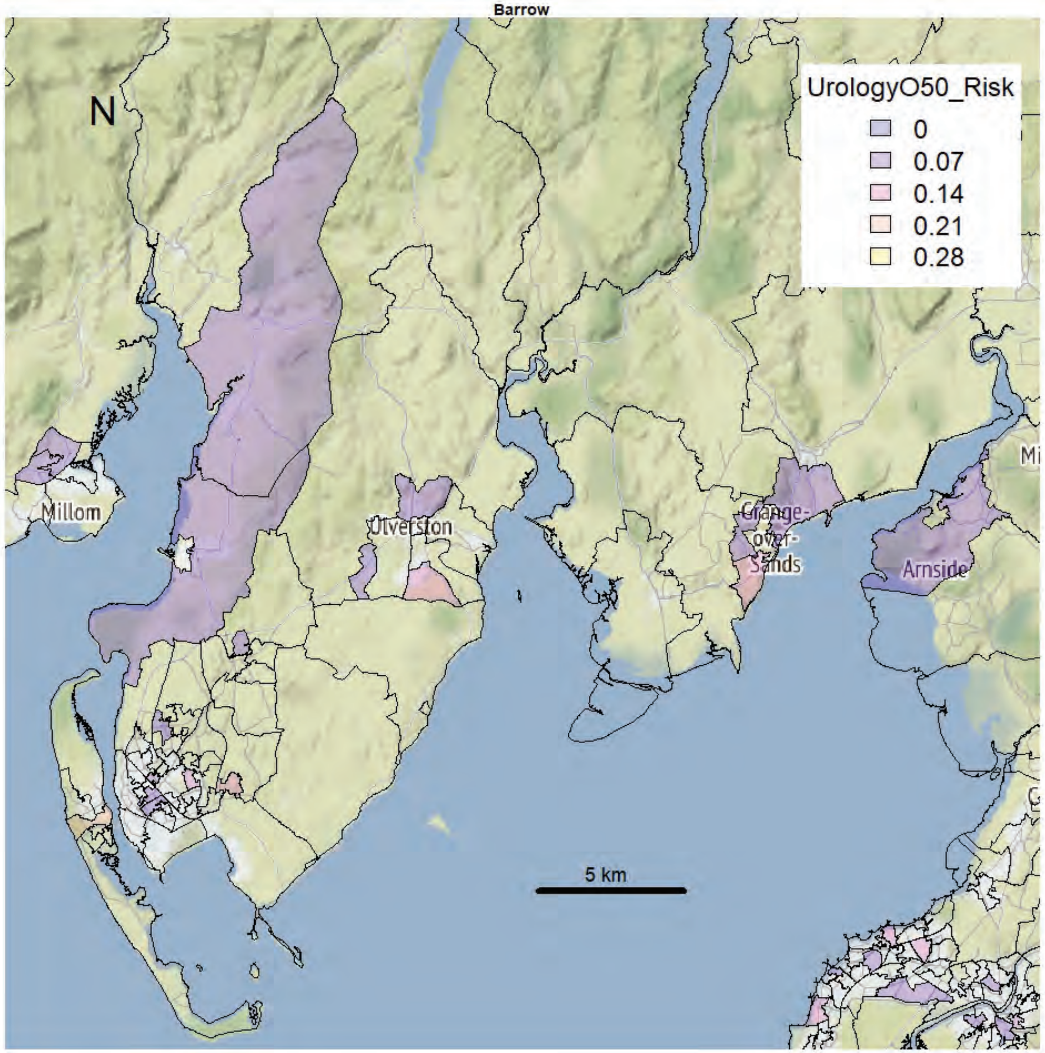
Barrow
N
UrologyO50_Risk
0
0.07
0.14
0.21
0.28
Millom
Ulverston
Grange-over-Sands
Arnside
5 km

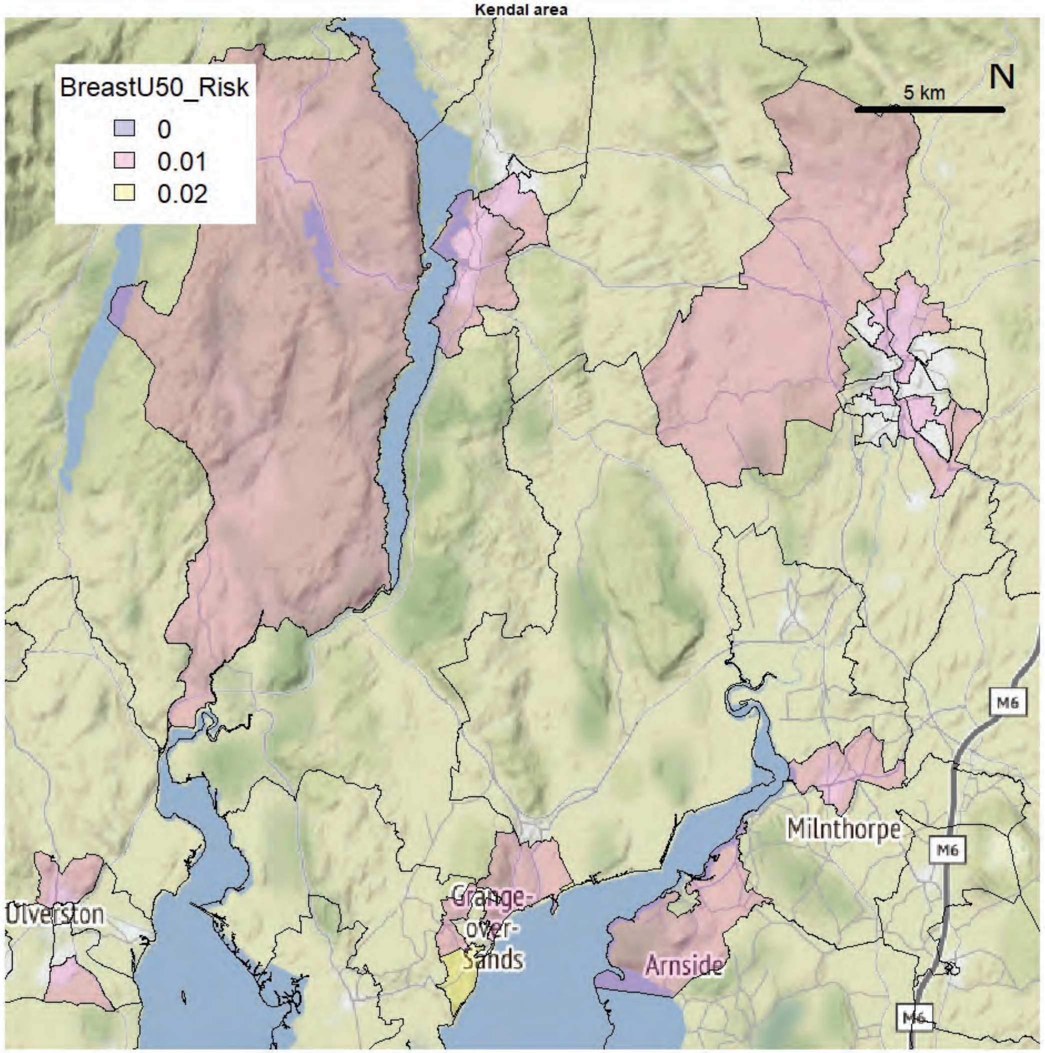
Kendal area
BreastU50_Risk
0
0.01
0.02
5 km
N
M6
Milnthorpe
Ulverston
Grange-over-Sands
Arnside

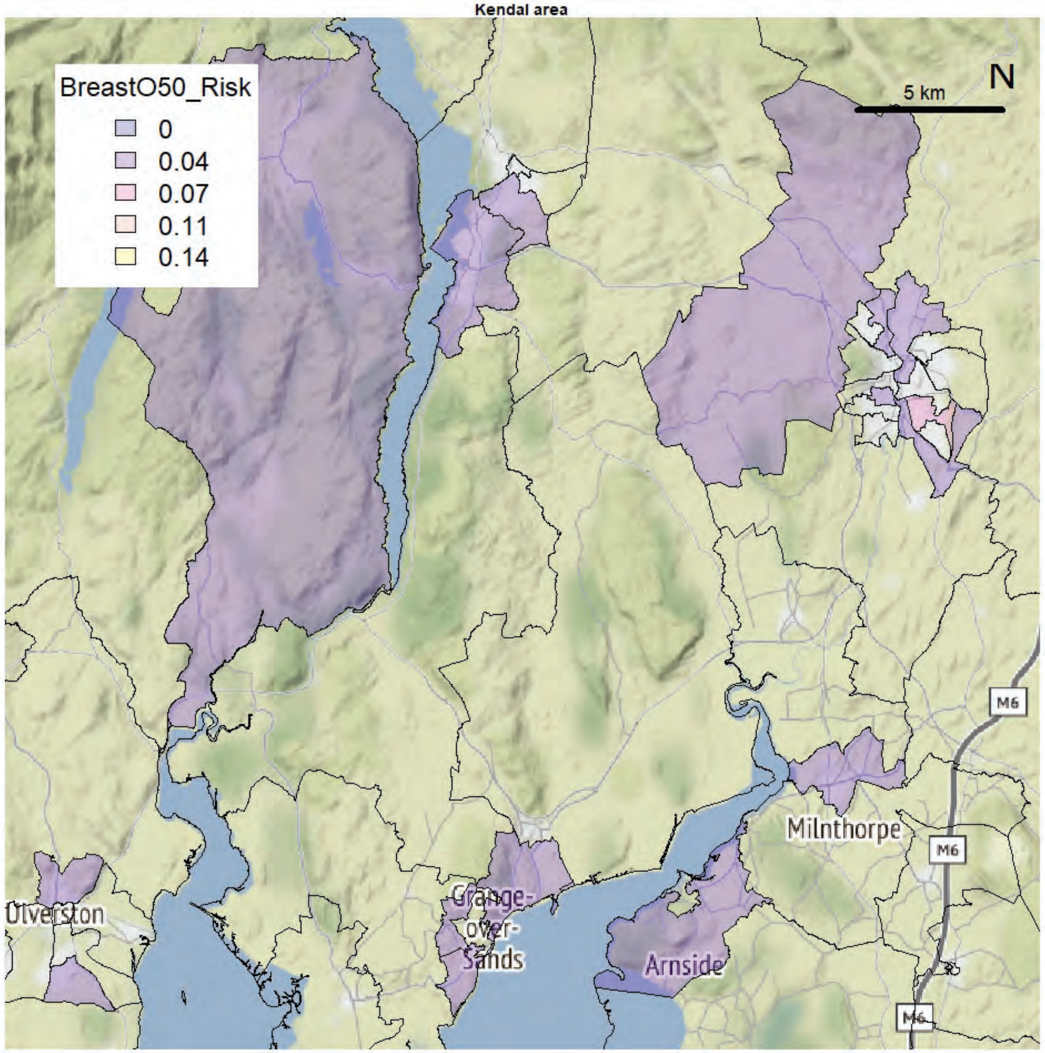
Kendal area
BreastO50_Risk
0
0.04
0.07
0.11
0.14
5 km
N
M6
Milnthorpe
Ulverston
Grange-over-Sands
Arnside

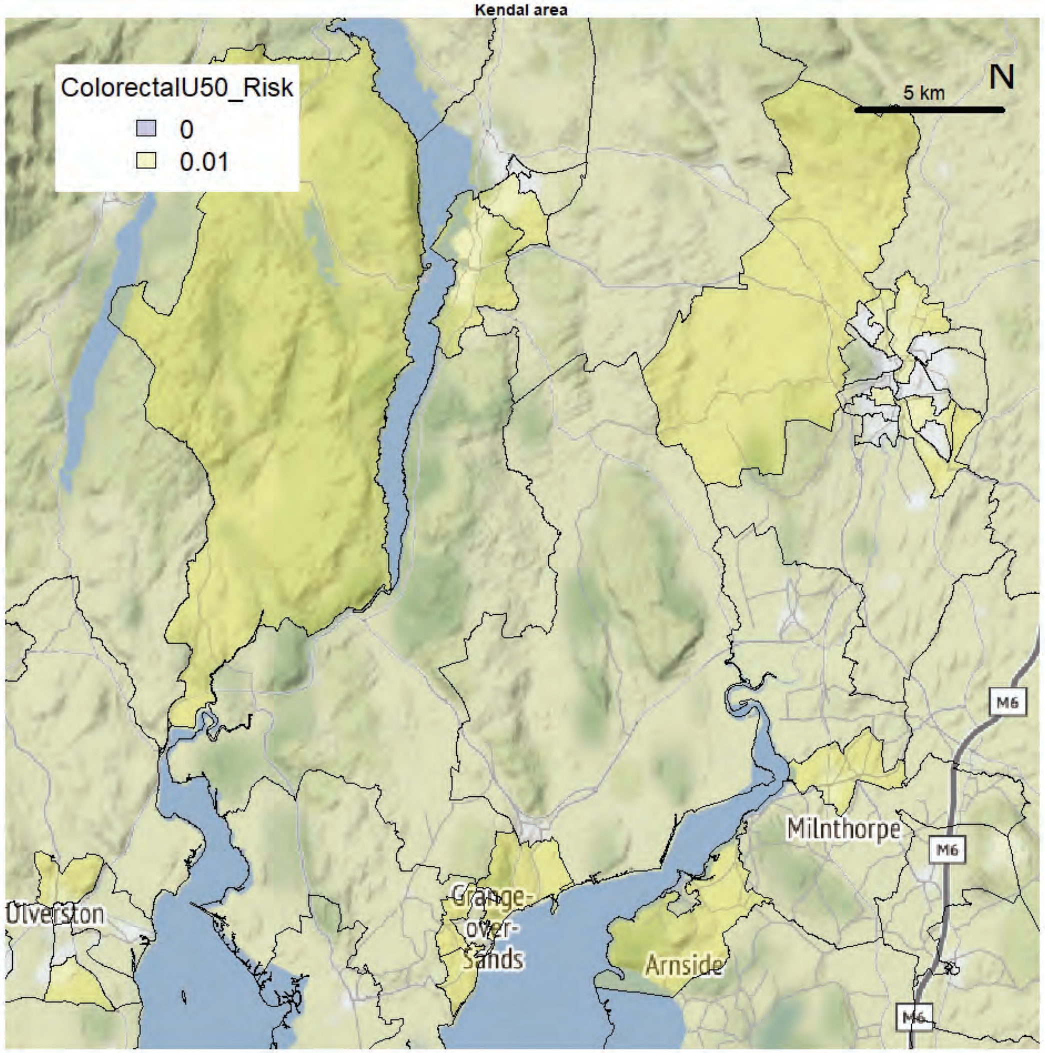
Kendal area
ColorectalU50_Risk
0
0.01
5 km
N
M6
M6
M6
Milnthorpe
Ulverston
Grange-
over-
Sands
Arnside

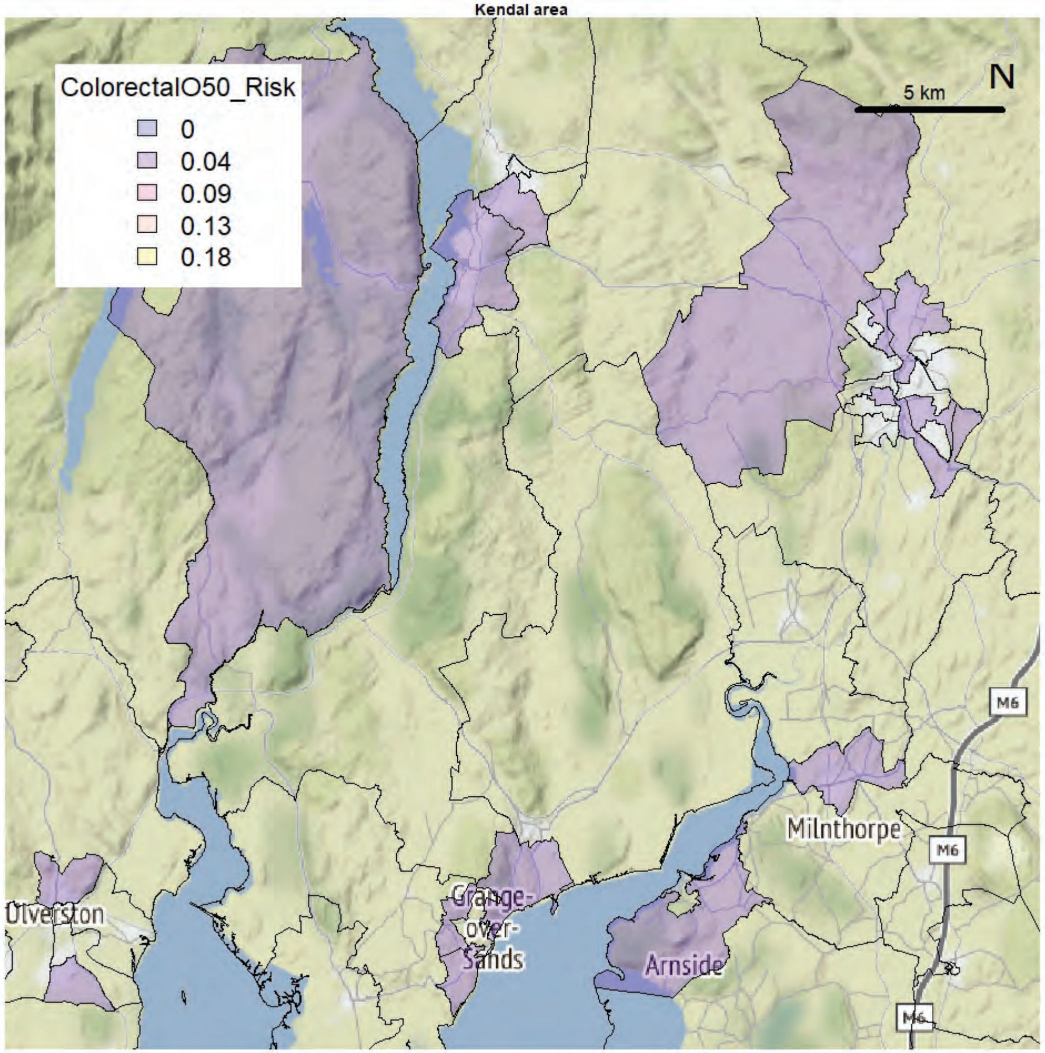
Kendal area
ColorectalO50_Risk
0
0.04
0.09
0.13
0.18
5 km
N
M6
M6
M6
Milnthorpe
Ulverston
Grange-
over-
Sands
Arnside

Kendal area

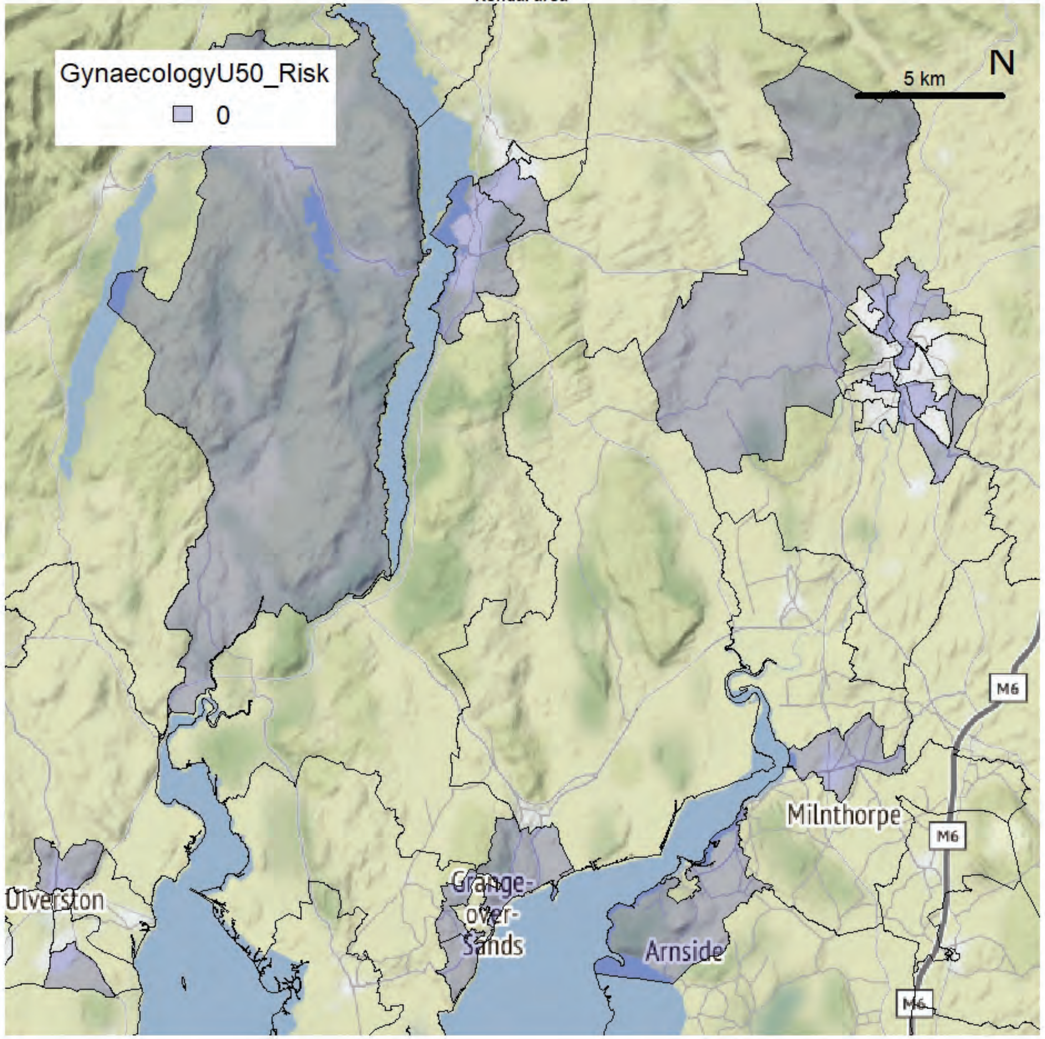

Kendal area

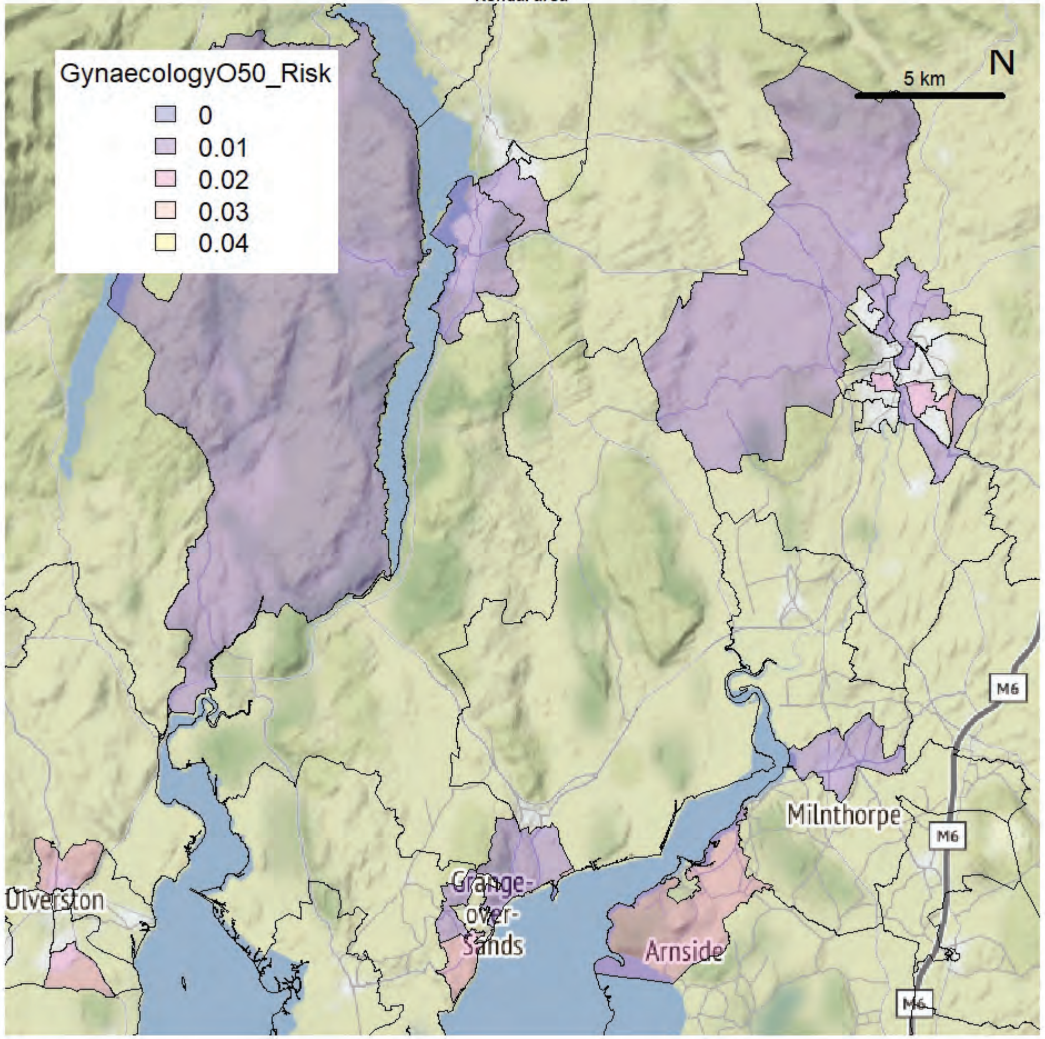

Kendal area

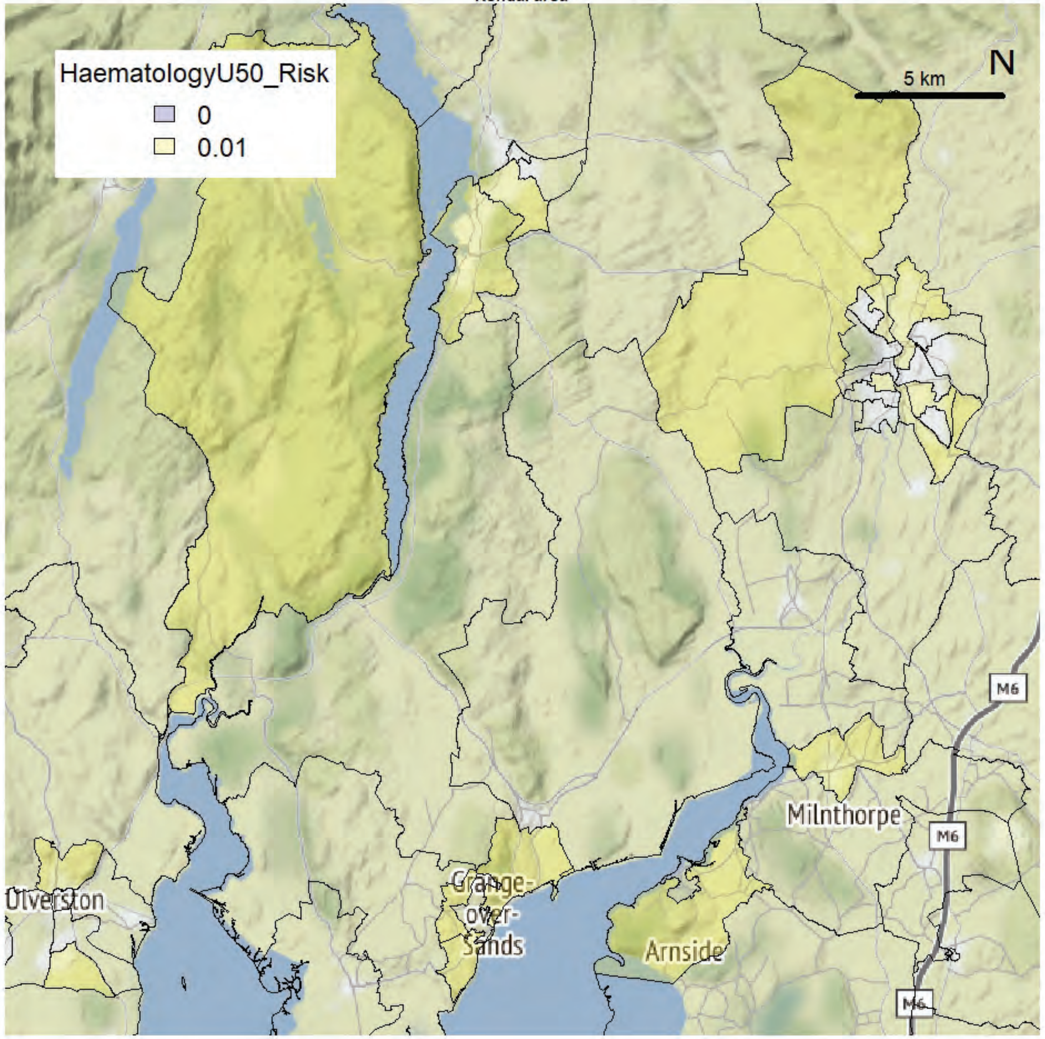

Kendal area

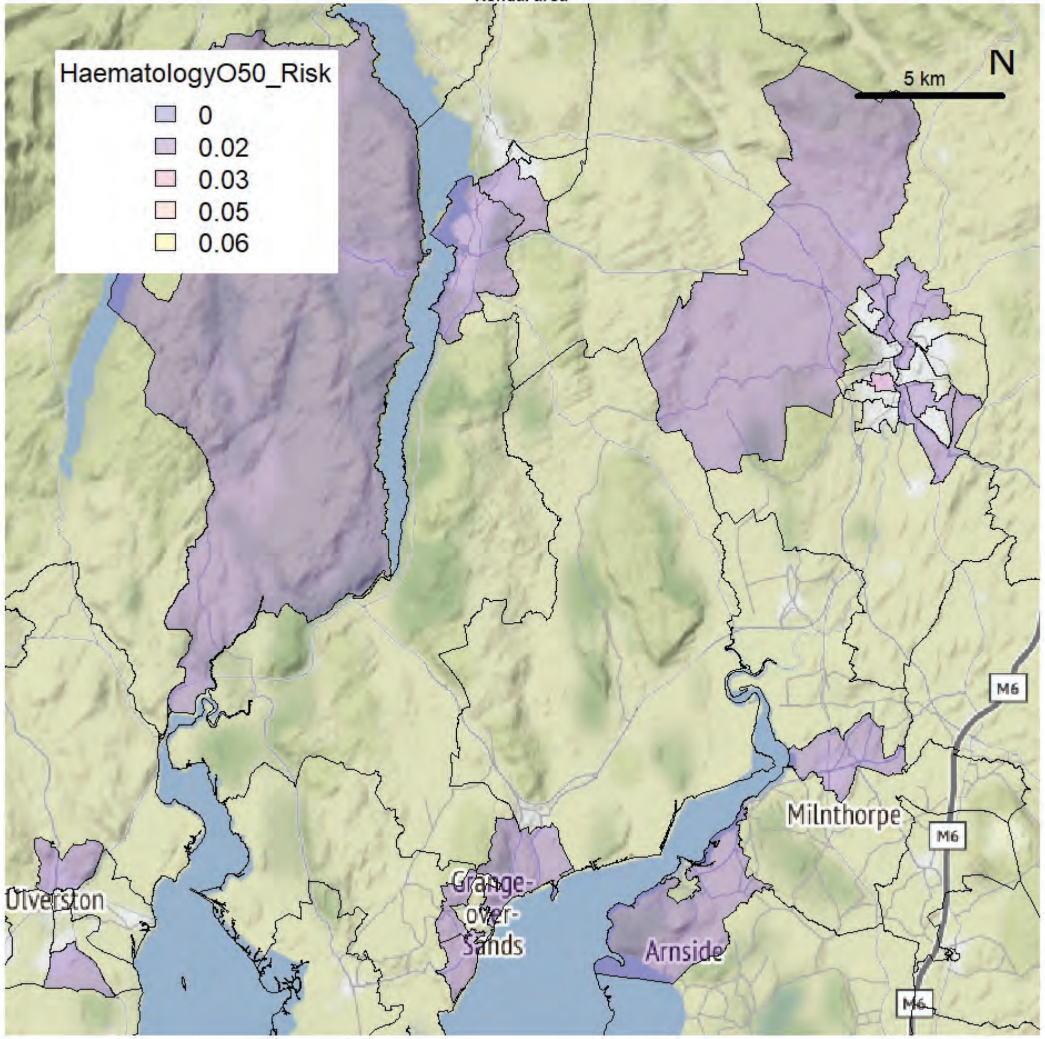

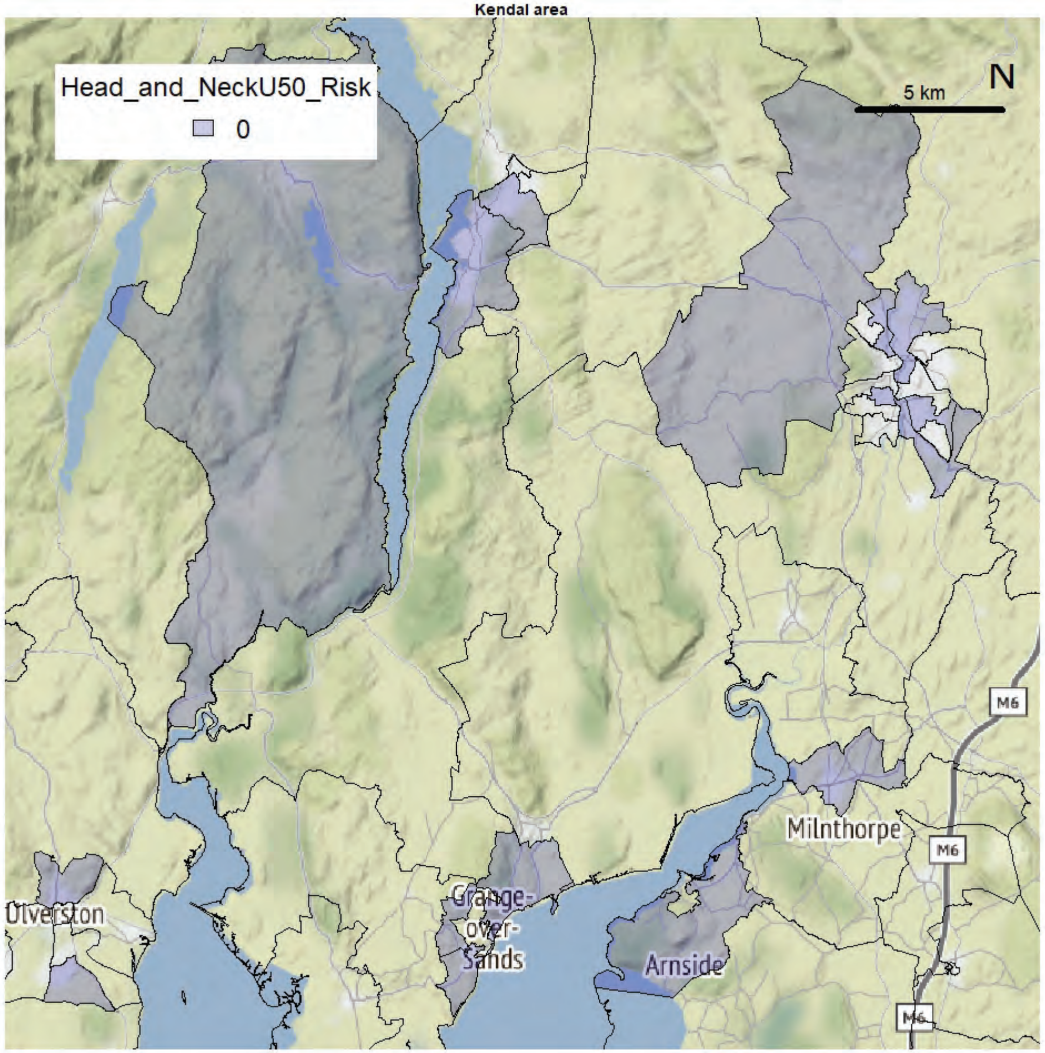
Kendal area
Head_and_NeckU50_Risk
0
5 km
N
M6
Milnthorpe
Ulverston
Grange-over-Sands
Arnside

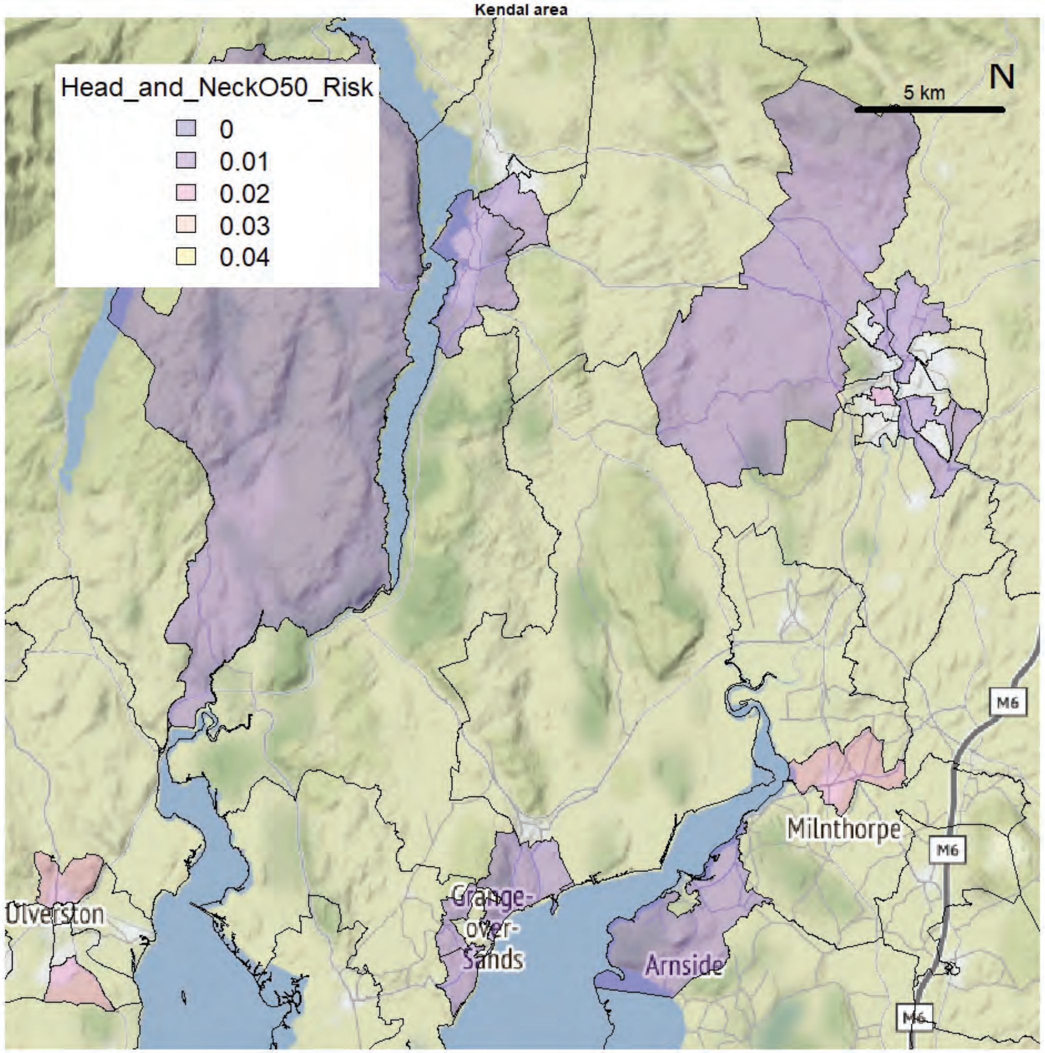
Kendal area
Head_and_NeckO50_Risk
0
0.01
0.02
0.03
0.04
5 km
N
M6
Milnthorpe
Ulverston
Grange-over-Sands
Arnside

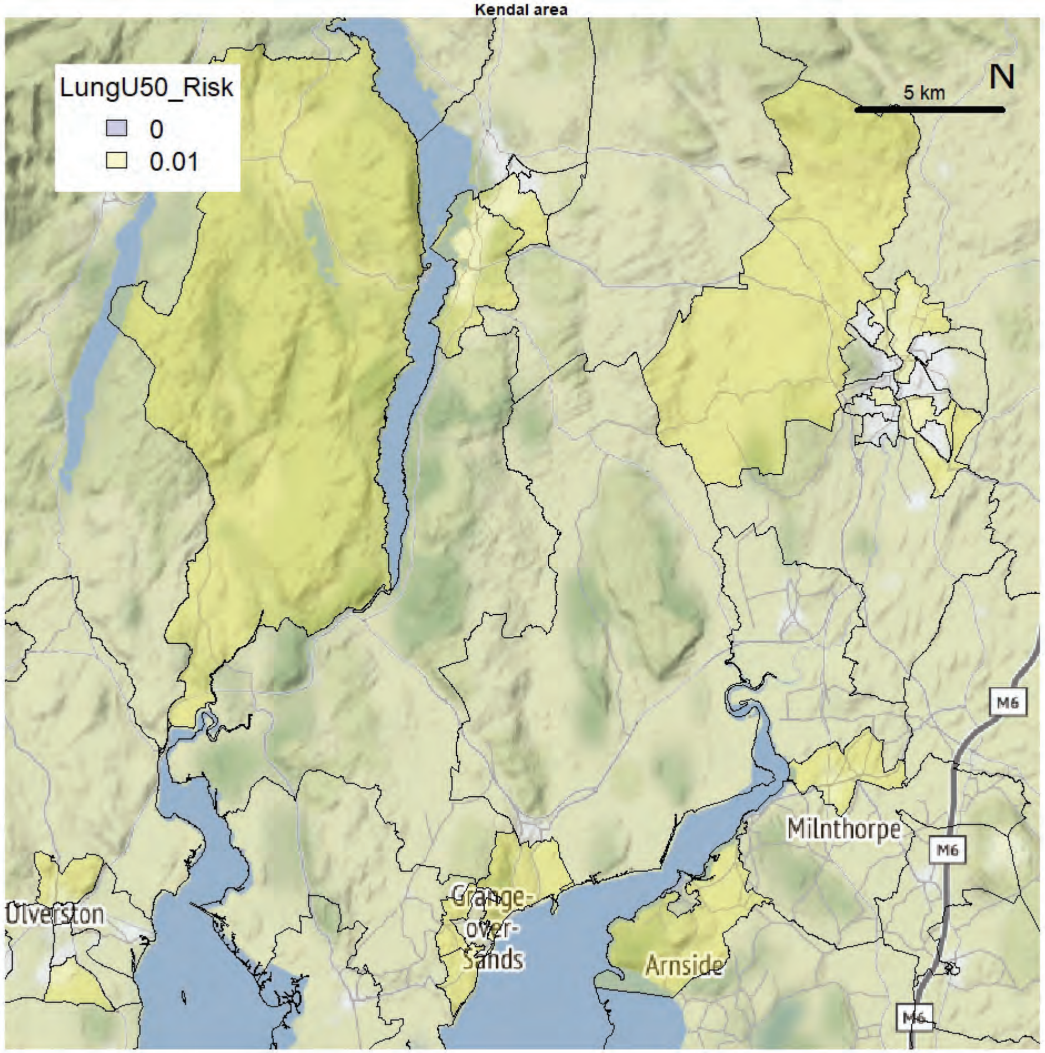
Kendal area
LungU50_Risk
0
0.01
5 km
N
M6
Milnthorpe
Ulverston
Grange-over-Sands
Arnside

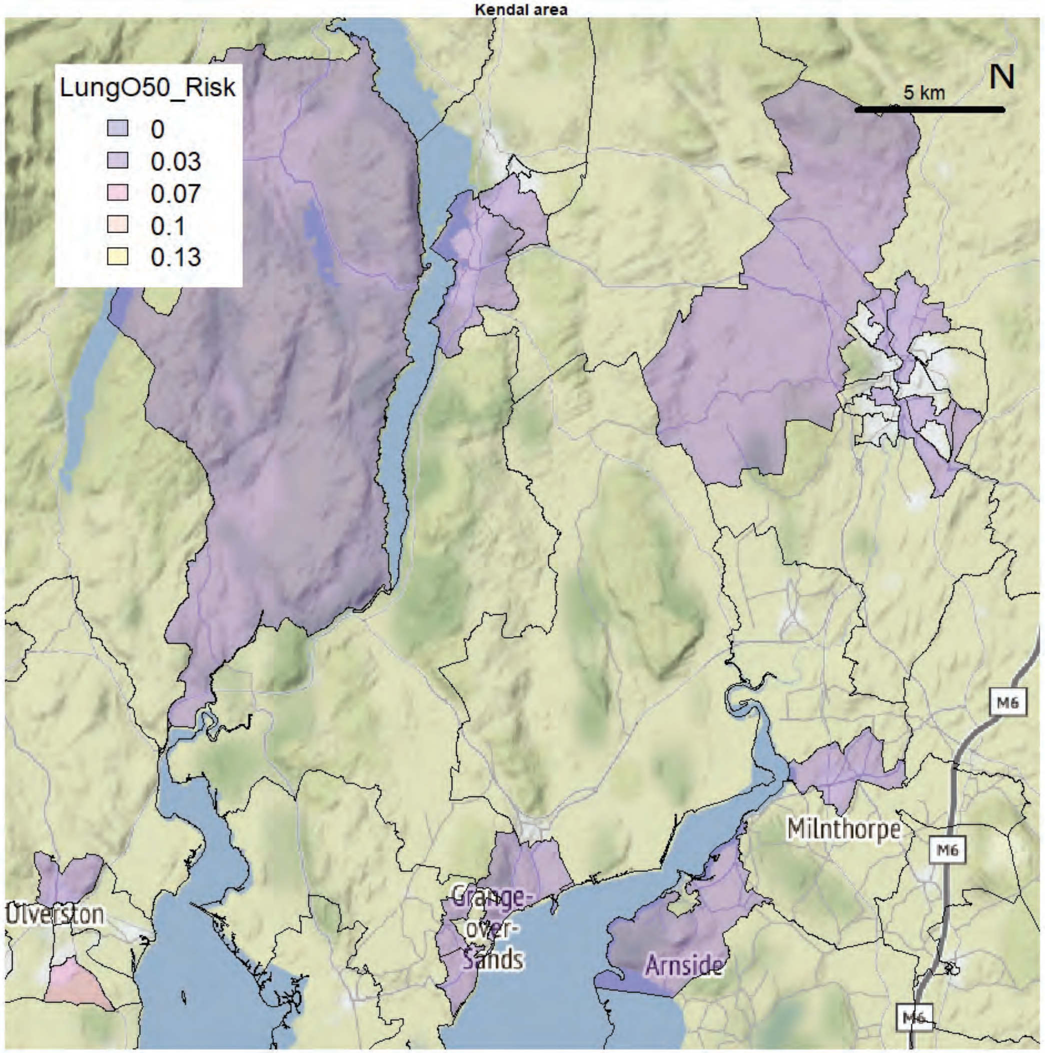
Kendal area
LungO50_Risk
0
0.03
0.07
0.1
0.13
5 km
N
M6
Milnthorpe
Ulverston
Grange-over-Sands
Arnside

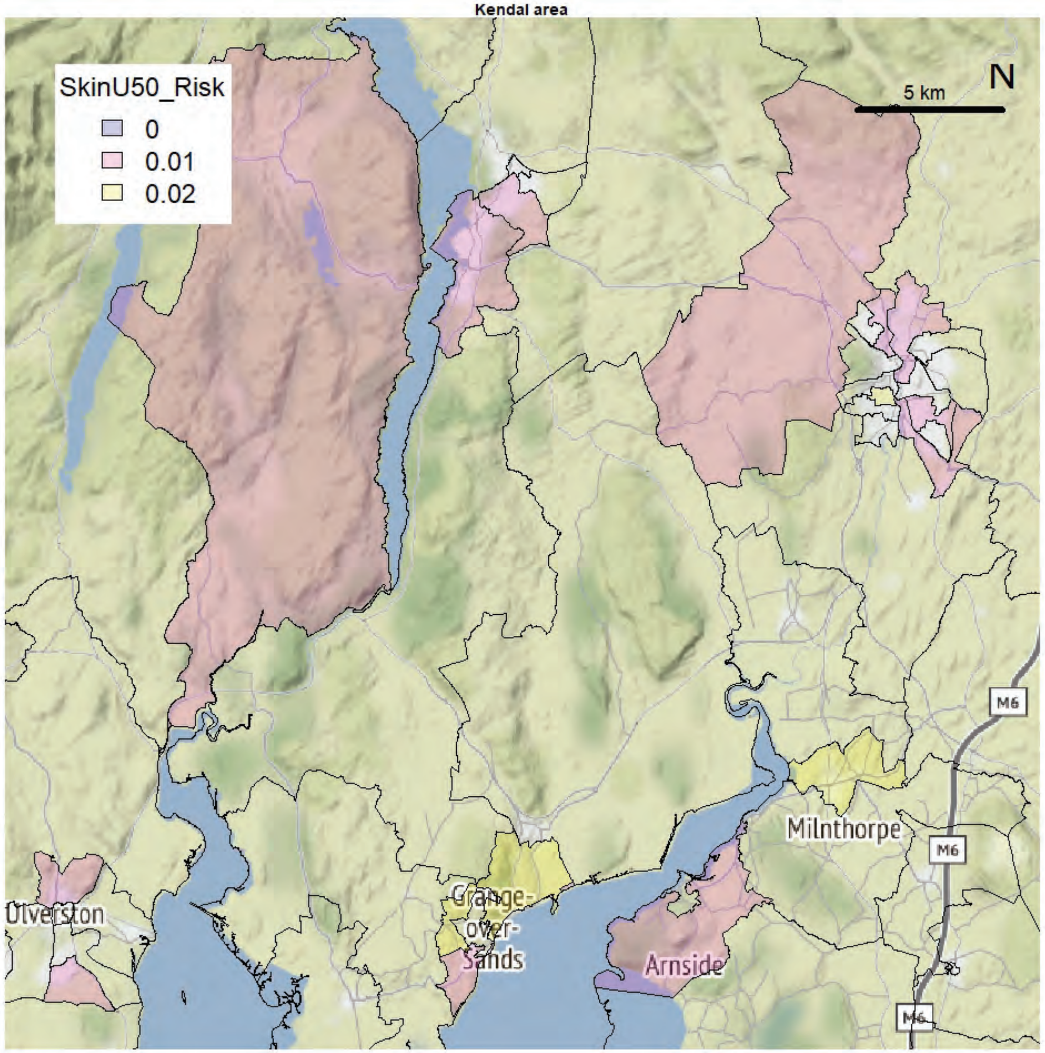
Kendal area
SkinU50_Risk
0
0.01
0.02
5 km
N
M6
Milnthorpe
M6
Ulverston
Grange-
over-
Sands
Arnside
M6

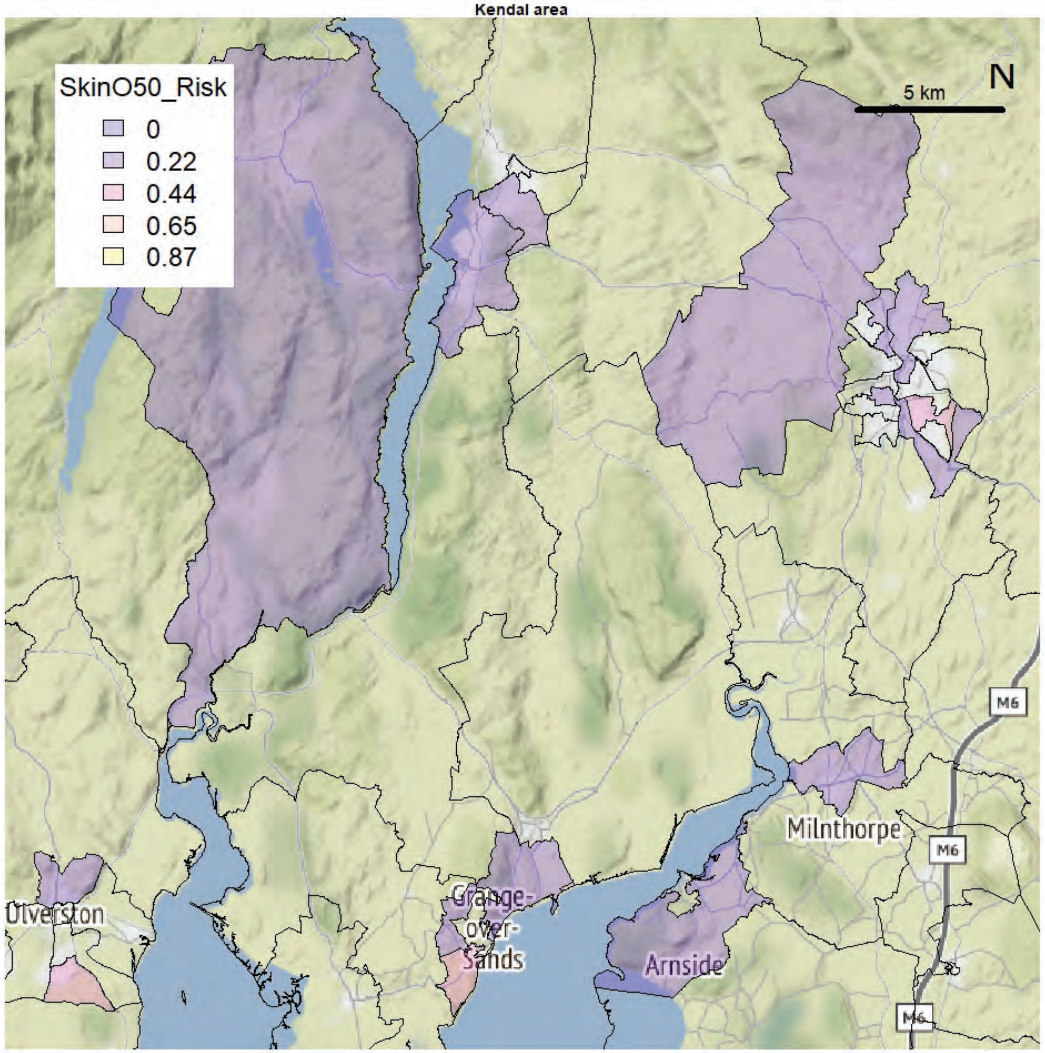
Kendal area
SkinO50_Risk
0
0.22
0.44
0.65
0.87
5 km
N
M6
Milnthorpe
M6
Ulverston
Grange-
over-
Sands
Arnside
M6

Kendal area

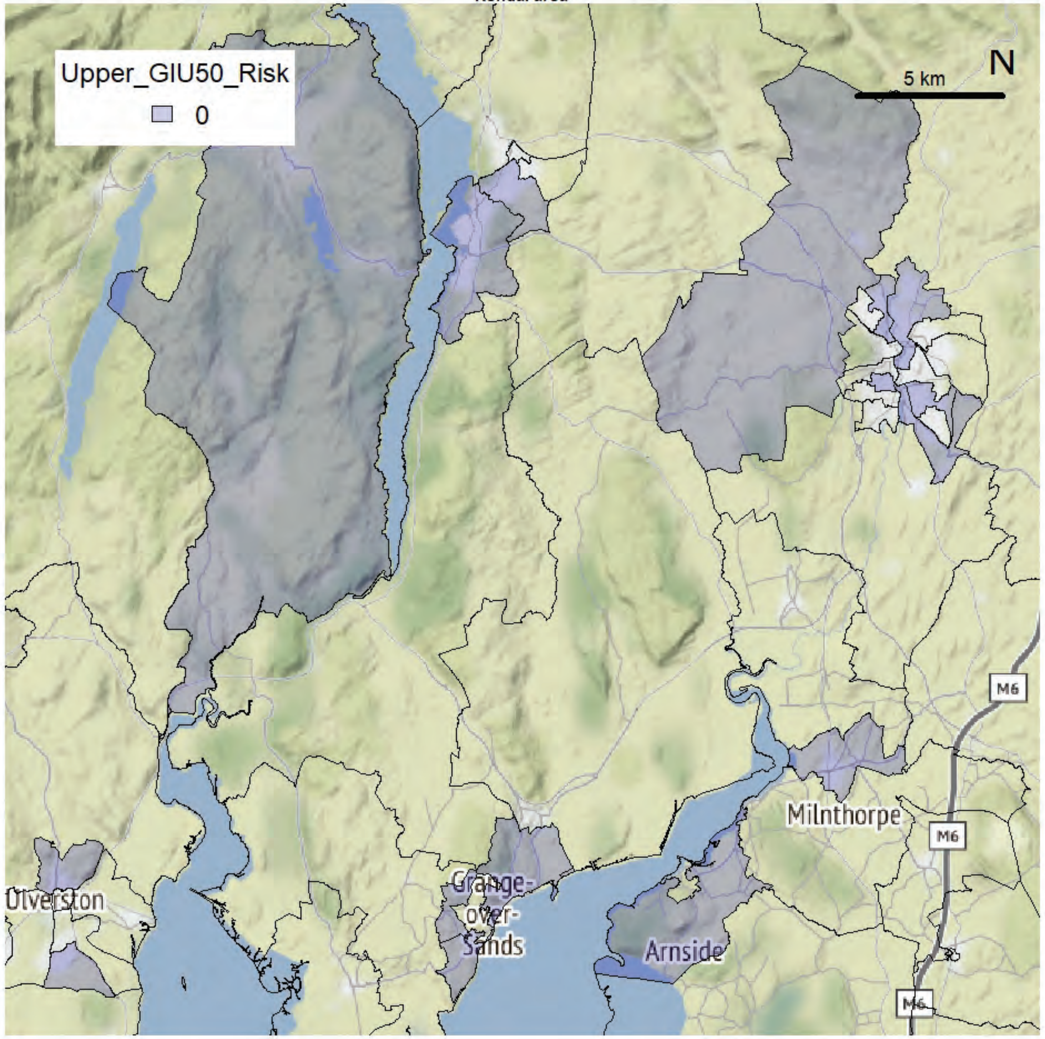

Kendal area

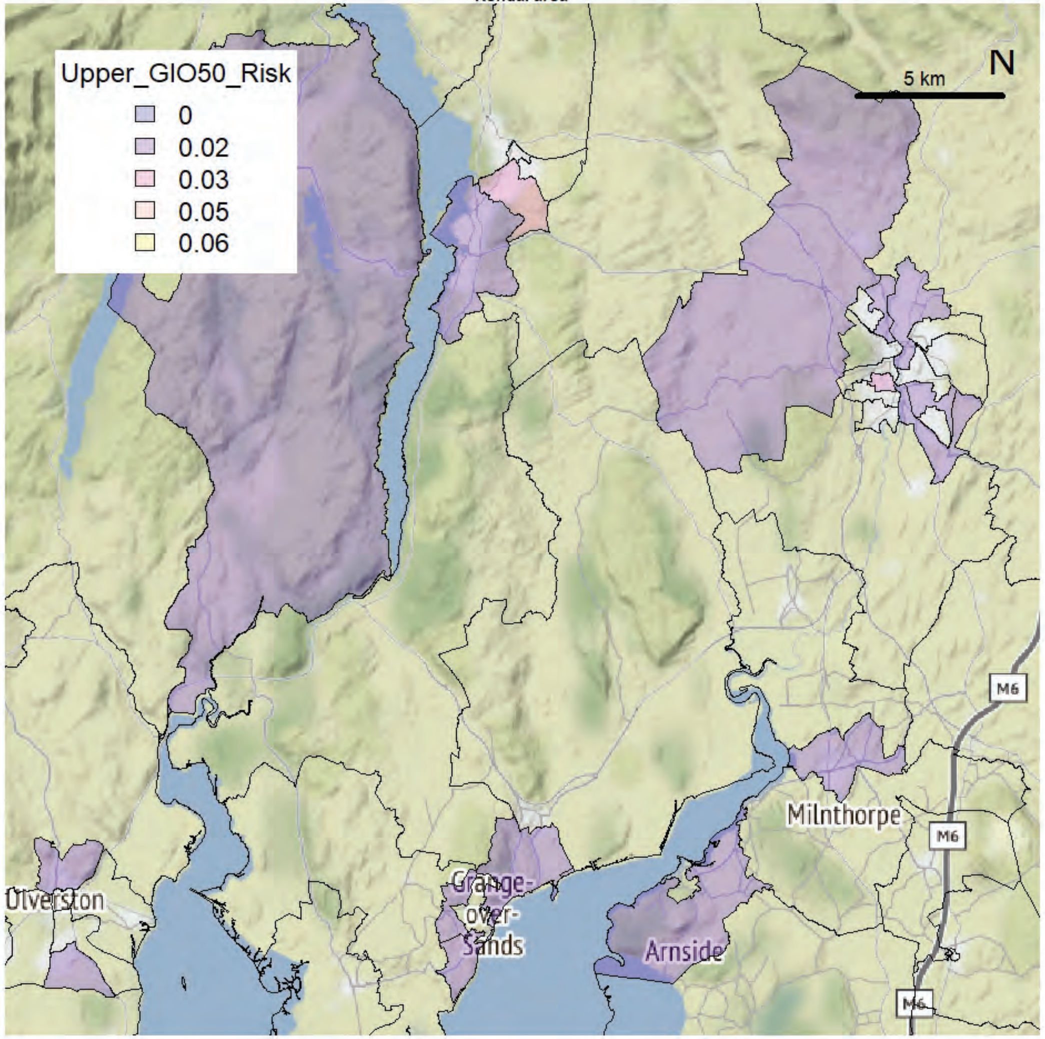

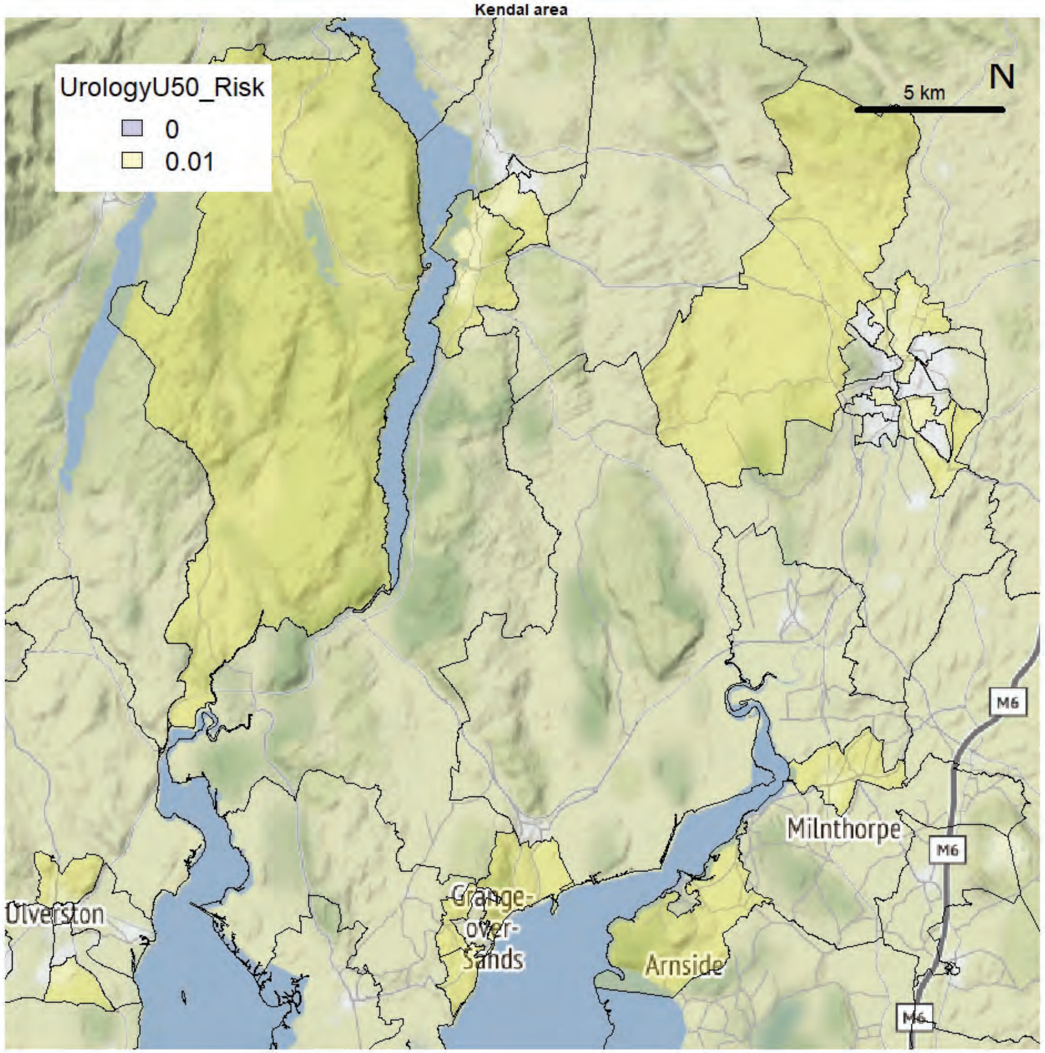
Kendal area
UrologyU50_Risk
0
0.01
5 km
N
M6
Milnthorpe
Ulverston
Grange-over-Sands
Arnside

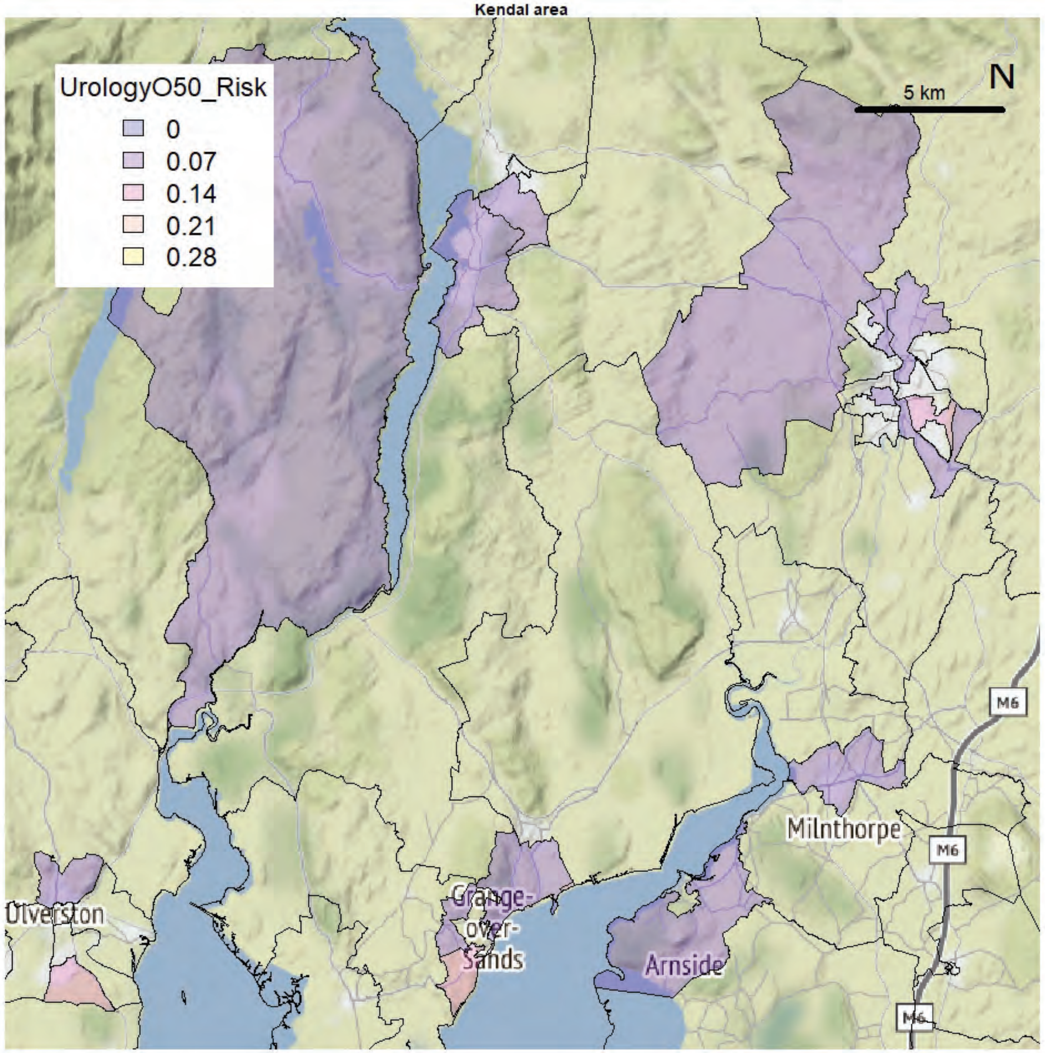
Kendal area
UrologyO50_Risk
0
0.07
0.14
0.21
0.28
5 km
N
M6
Milnthorpe
Ulverston
Grange-over-Sands
Arnside

Supplementary Information S11: Corregionalisation between pairs of cancer types

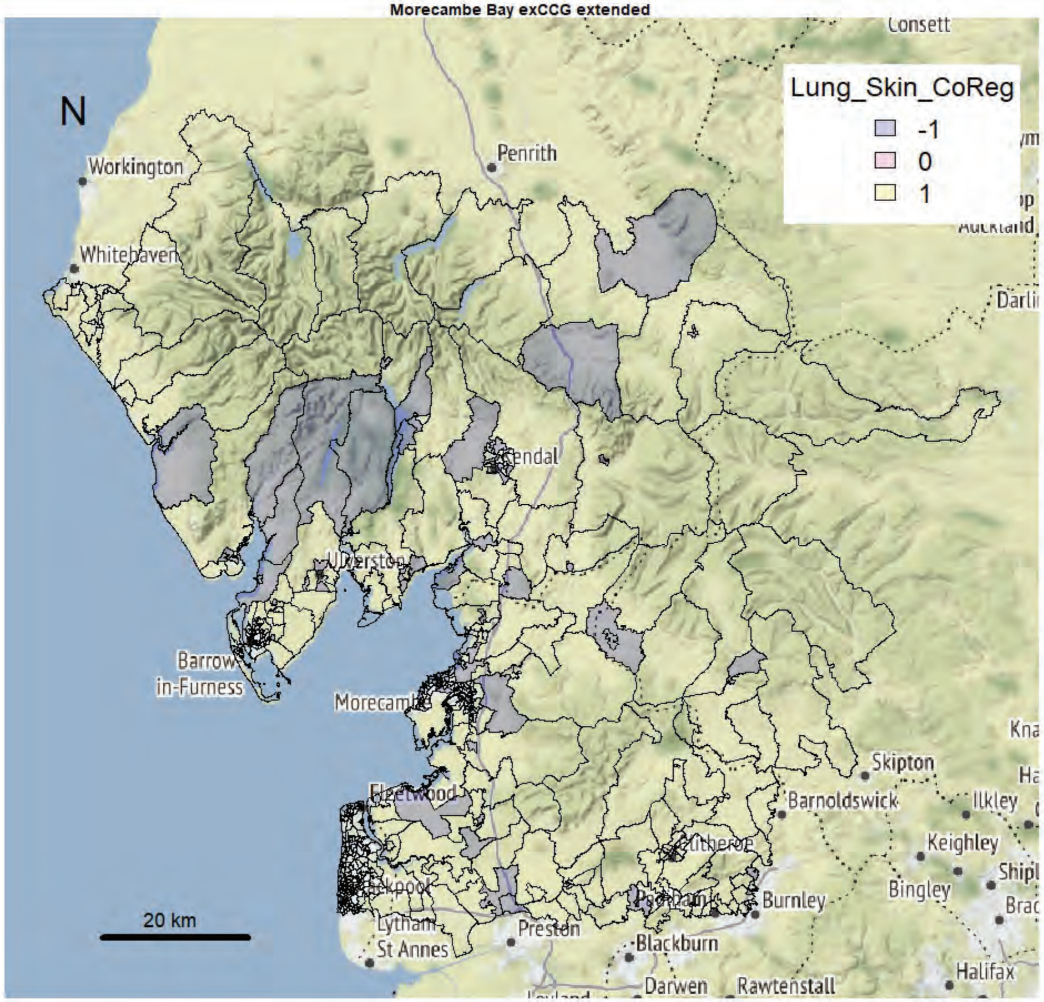

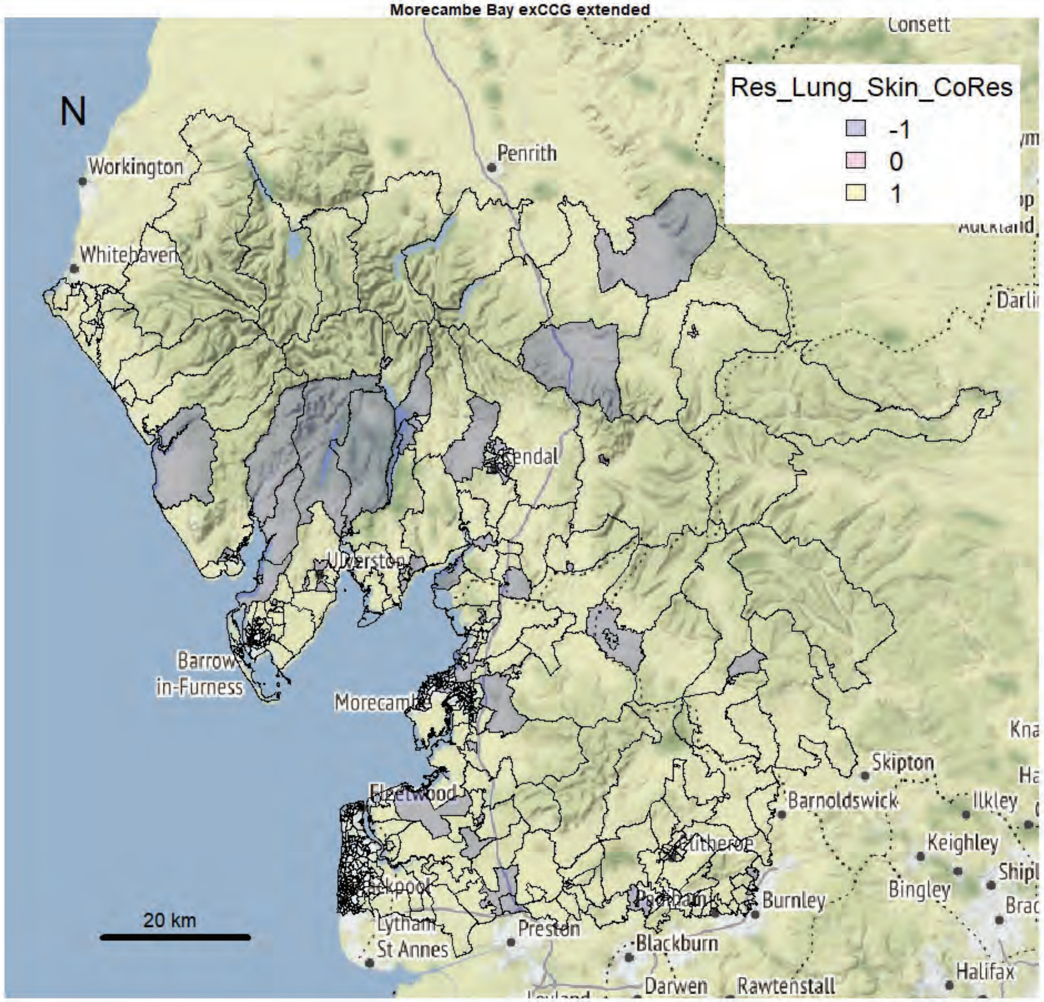

Morecambe Bay exCCG extended

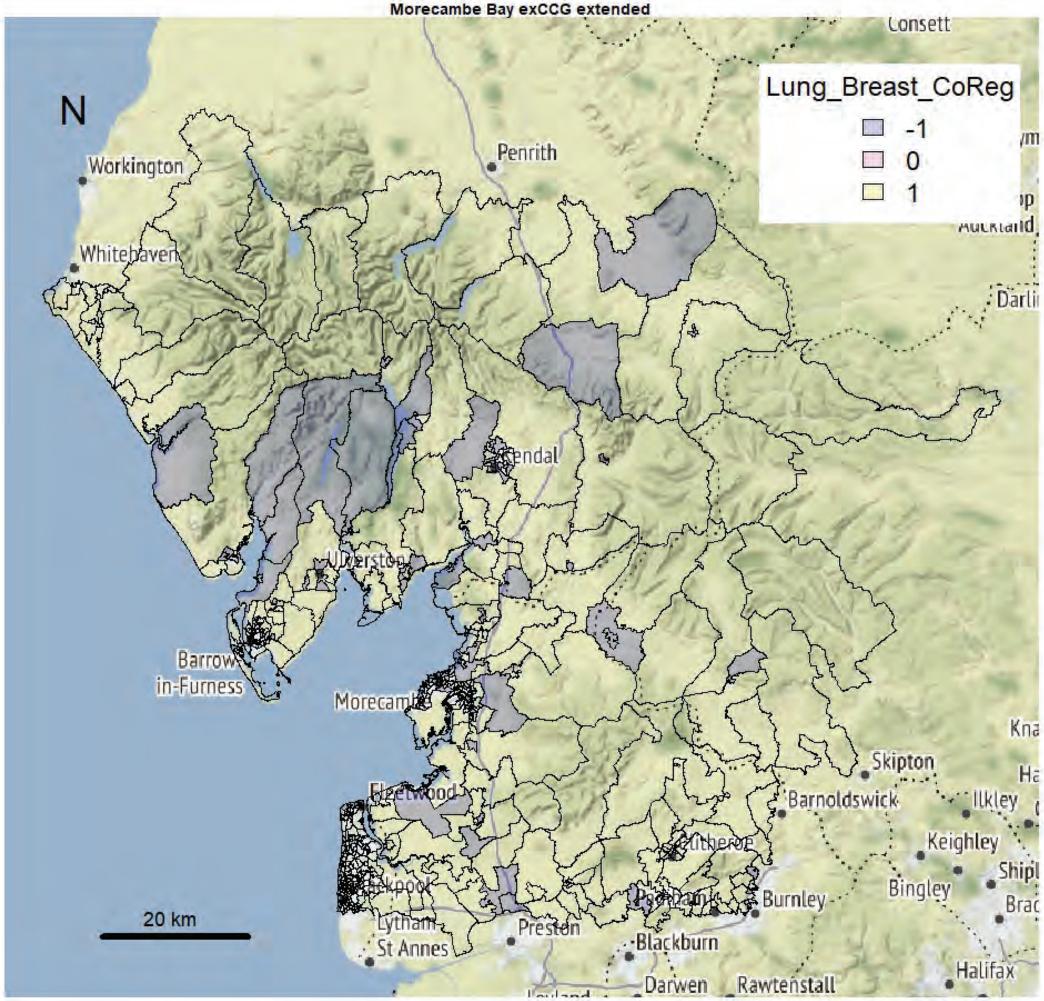

Morecambe Bay exCCG extended

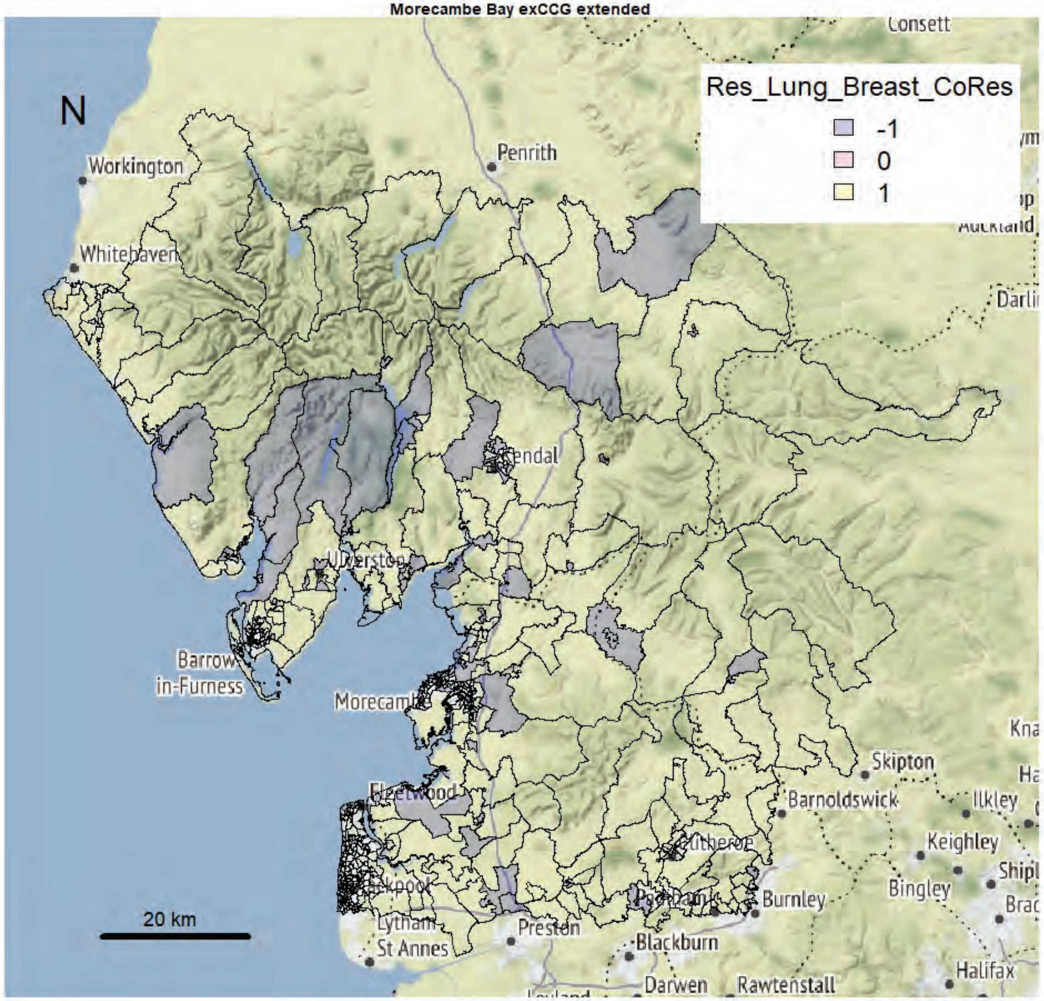

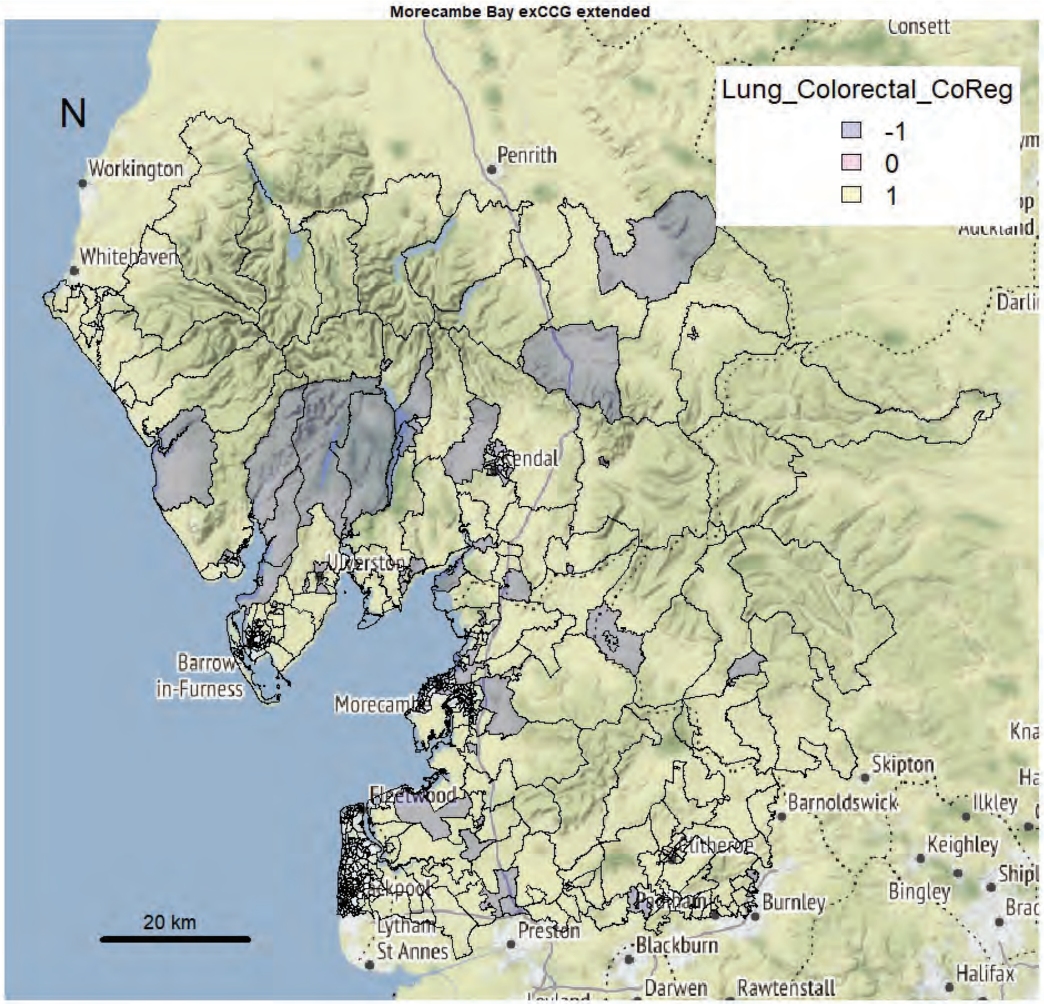
Morecambe Bay exCCG extended
Lung_Colorectal_CoReg
-1
0
1
N
20 km
Consett
Workington
Penrith
Whitehaven
Kendal
Ulverston
Barrow-in-Furness
Morecambe
Fleetwood
Skipton
Barnoldswick
Ilkley
Keighley
Bingley
Clitheroe
Burnley
Padiham
Preston
Blackburn
Lytham St Annes
Darwen
Rawtenstall
Halifax

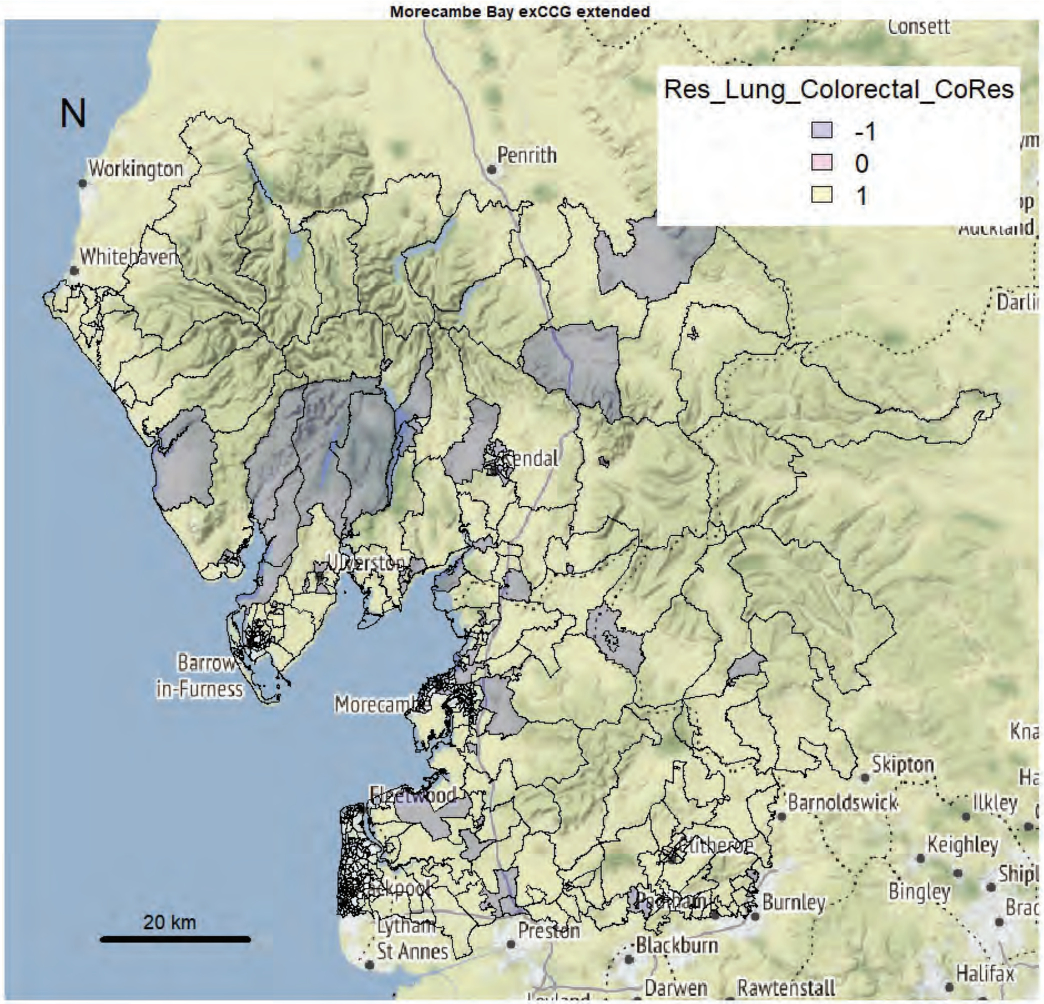
Morecambe Bay exCCG extended
Res_Lung_Colorectal_CoRes
-1
0
1
N
20 km
Consett
Workington
Penrith
Whitehaven
Kendal
Ulverston
Barrow-in-Furness
Morecambe
Fleetwood
Skipton
Barnoldswick
Ilkley
Keighley
Bingley
Clitheroe
Burnley
Padiham
Preston
Blackburn
Lytham St Annes
Darwen
Rawtenstall
Halifax

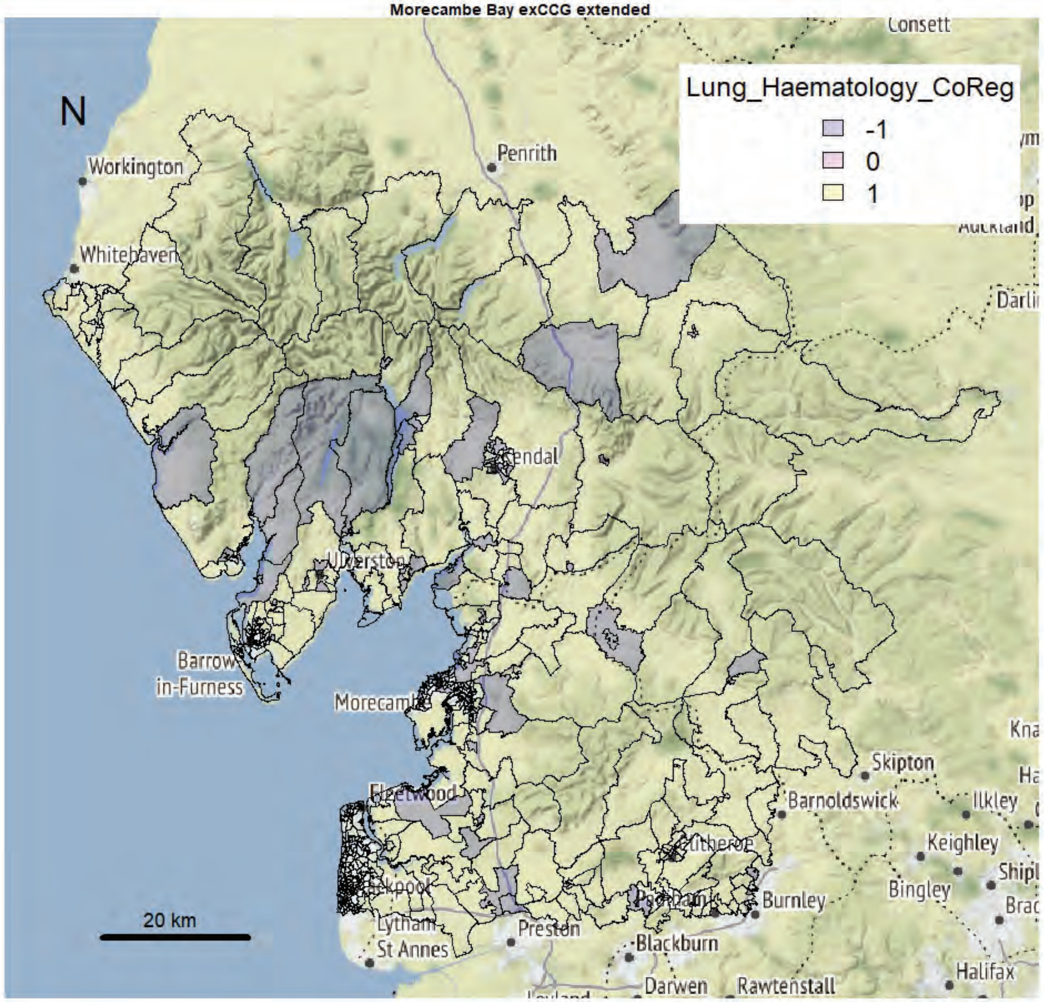
Morecambe Bay exCCG extended
N
Lung_Haematology_CoReg
-1
0
1
20 km
Workington
Penrith
Whitehaven
Kendal
Ulverston
Barrow-in-Furness
Morecambe
Fleetwood
Blackpool
Lytham St Annes
Preston
Blackburn
Burnley
Skipton
Barnoldswick
Ilkley
Keighley
Bingley
Halifax
Darwen
Rawtenstall
Consett

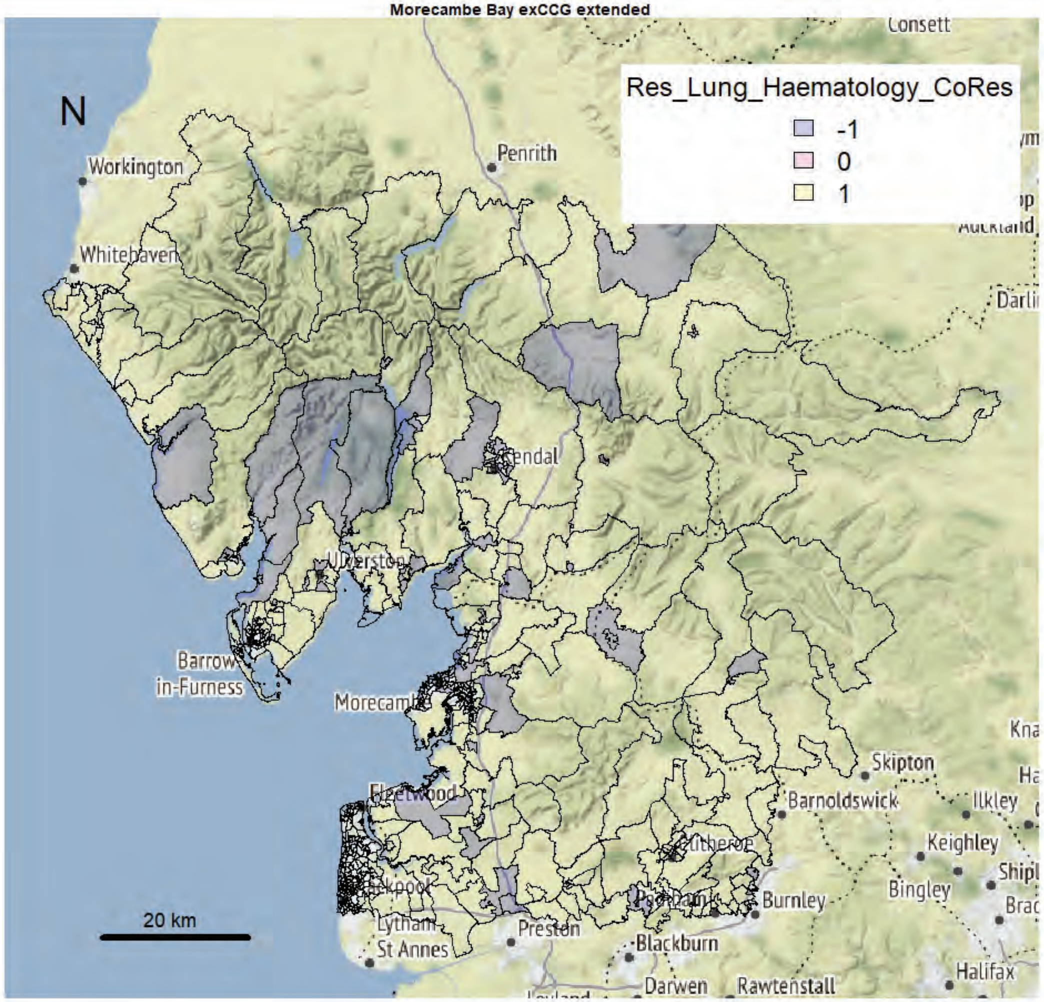
Morecambe Bay exCCG extended
N
Res_Lung_Haematology_CoRes
-1
0
1
20 km
Workington
Penrith
Whitehaven
Kendal
Ulverston
Barrow-in-Furness
Morecambe
Fleetwood
Blackpool
Lytham St Annes
Preston
Blackburn
Burnley
Skipton
Barnoldswick
Ilkley
Keighley
Bingley
Halifax
Darwen
Rawtenstall
Consett

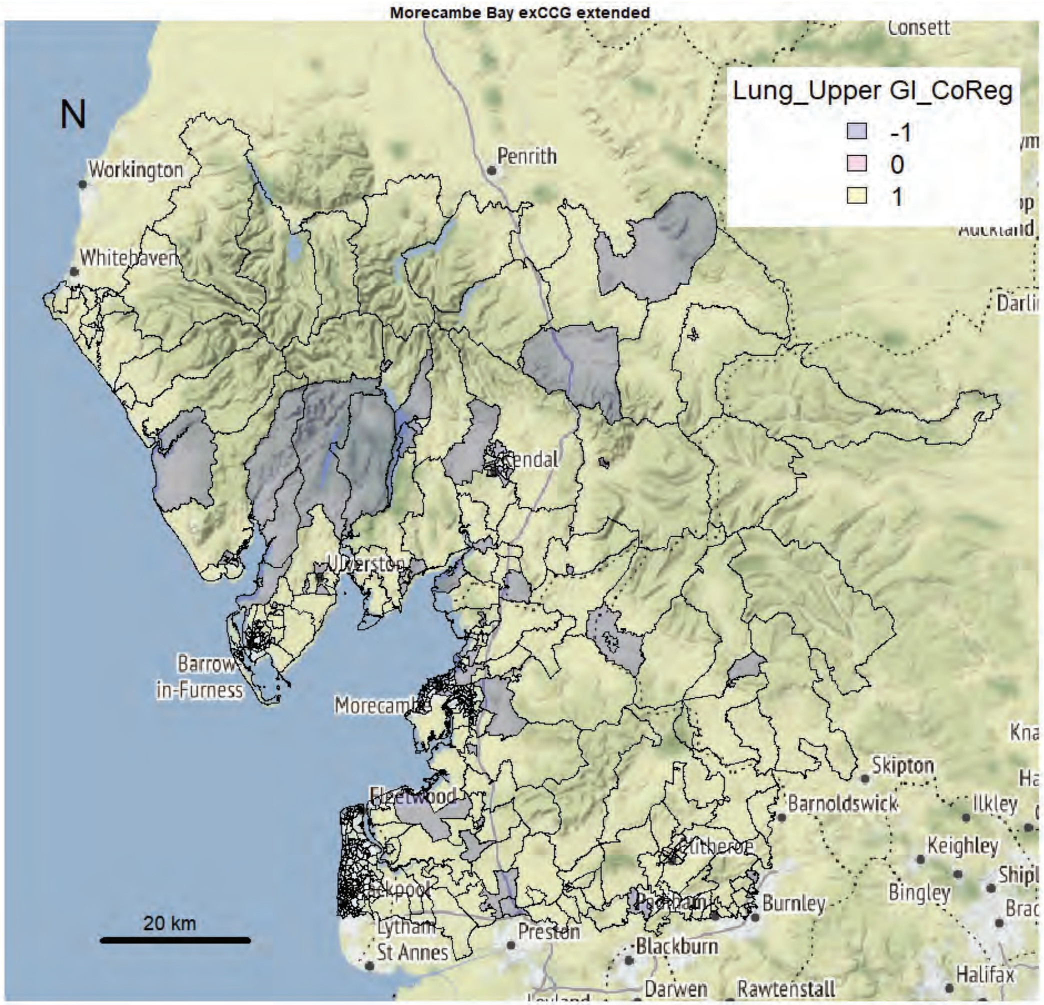
Morecambe Bay exCCG extended
Lung_Upper GI_CoReg
-1
0
1
N
20 km
Consett
Workington
Penrith
Whitehaven
Darli
Kendal
Ulverston
Barrow-in-Furness
Morecamb
Fleetwood
Skipton
Barnoldswick
Ilkley
Keighley
Bingley
Burnley
Preston
Blackburn
Lytham St Annes
Darwen
Rawtenstall
Halifax

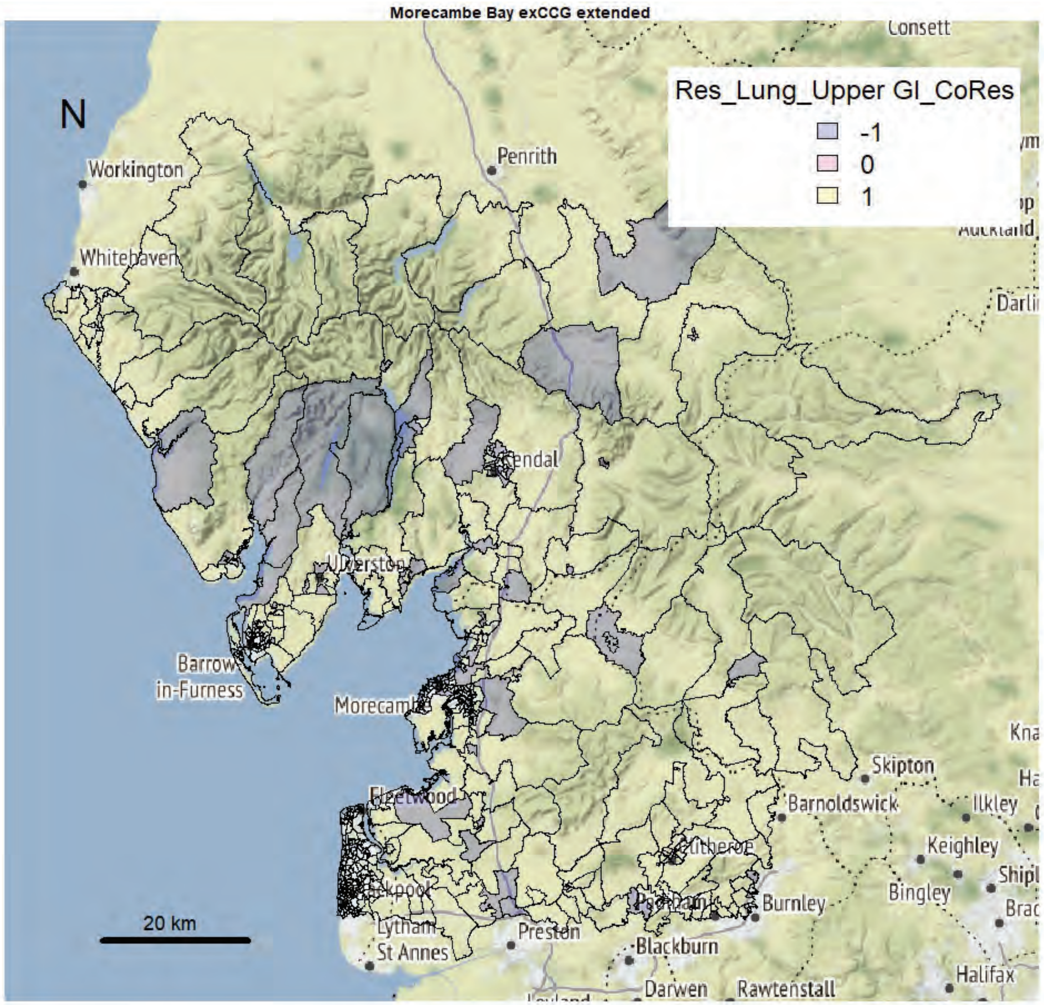
Morecambe Bay exCCG extended
Res_Lung_Upper GI_CoRes
-1
0
1
N
20 km
Consett
Workington
Penrith
Whitehaven
Darli
Kendal
Ulverston
Barrow-in-Furness
Morecamb
Fleetwood
Skipton
Barnoldswick
Ilkley
Keighley
Bingley
Burnley
Preston
Blackburn
Lytham St Annes
Darwen
Rawtenstall
Halifax

Morecambe Bay exCCG extended

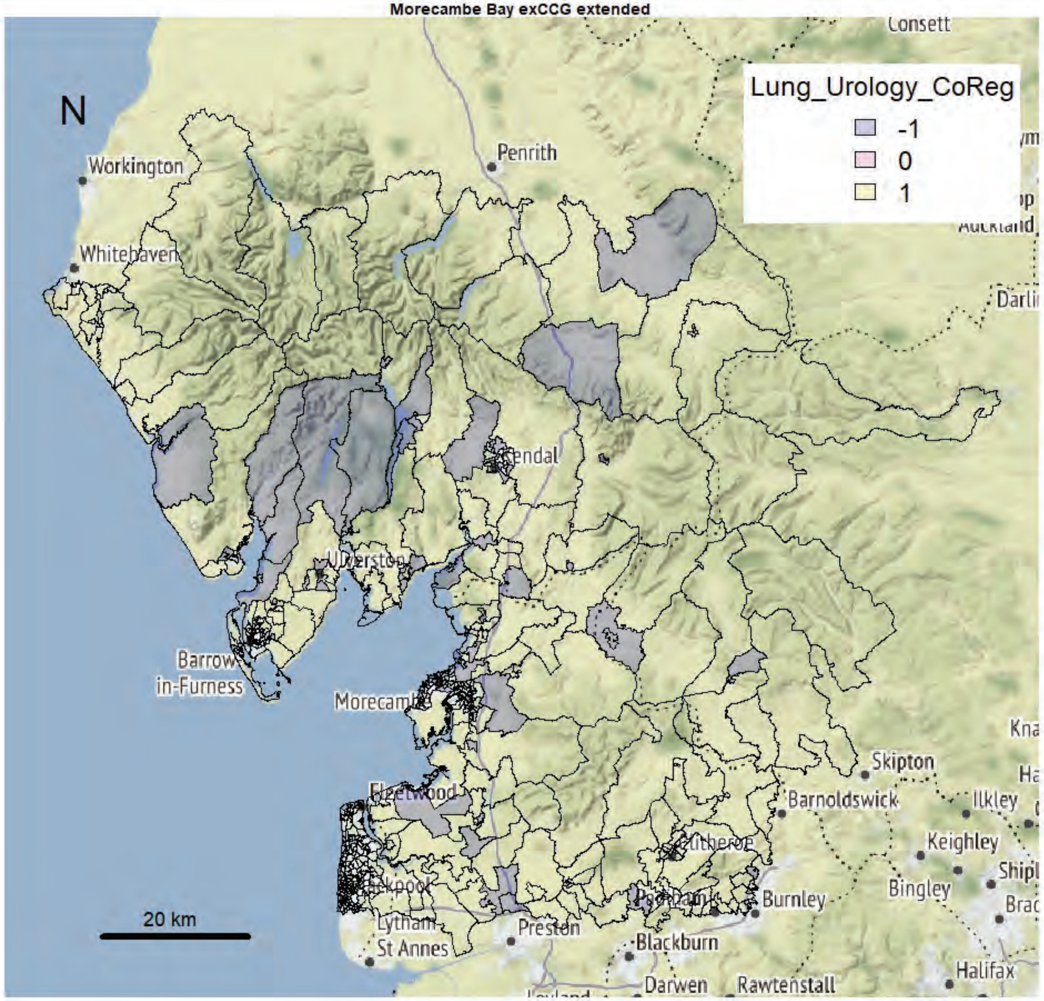

Morecambe Bay exCCG extended

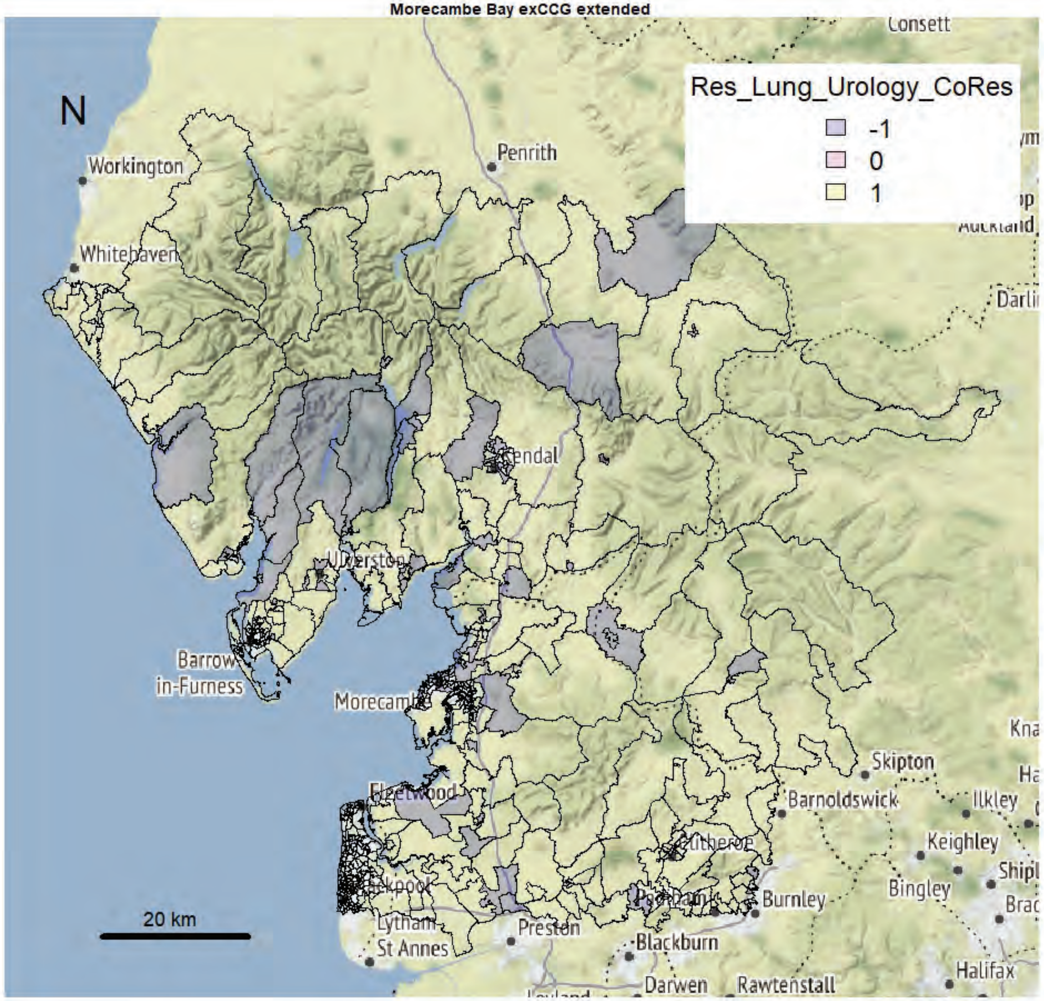

Morecambe Bay exCCG extended

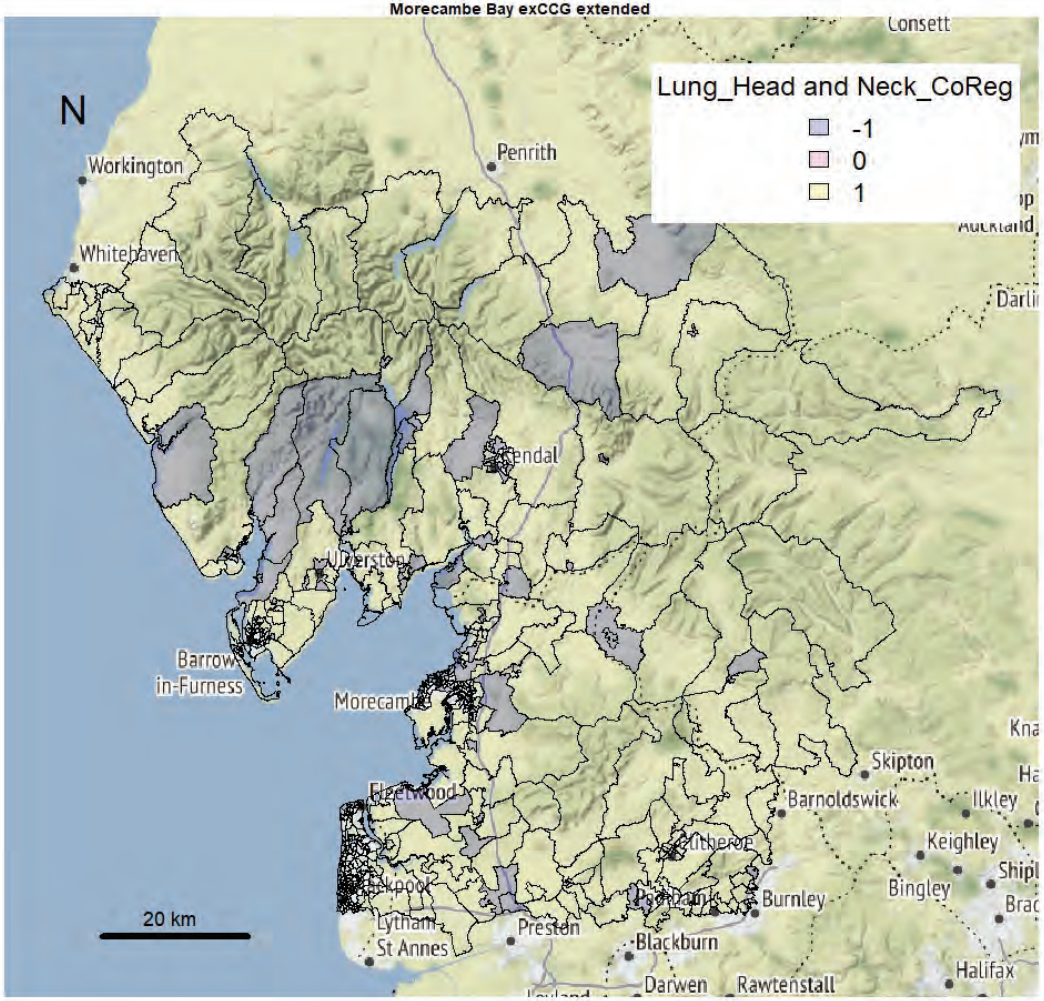

Morecambe Bay exCCG extended

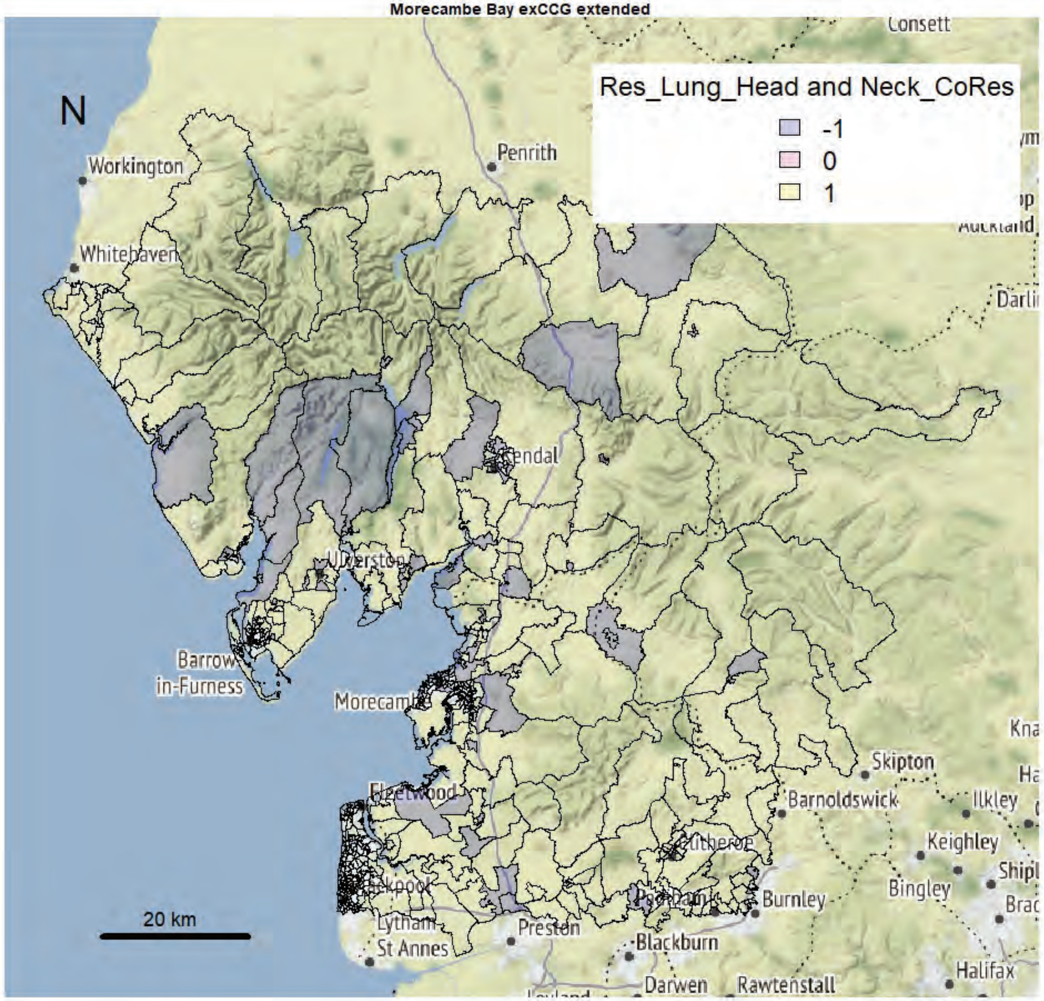

Morecambe Bay exCCG extended

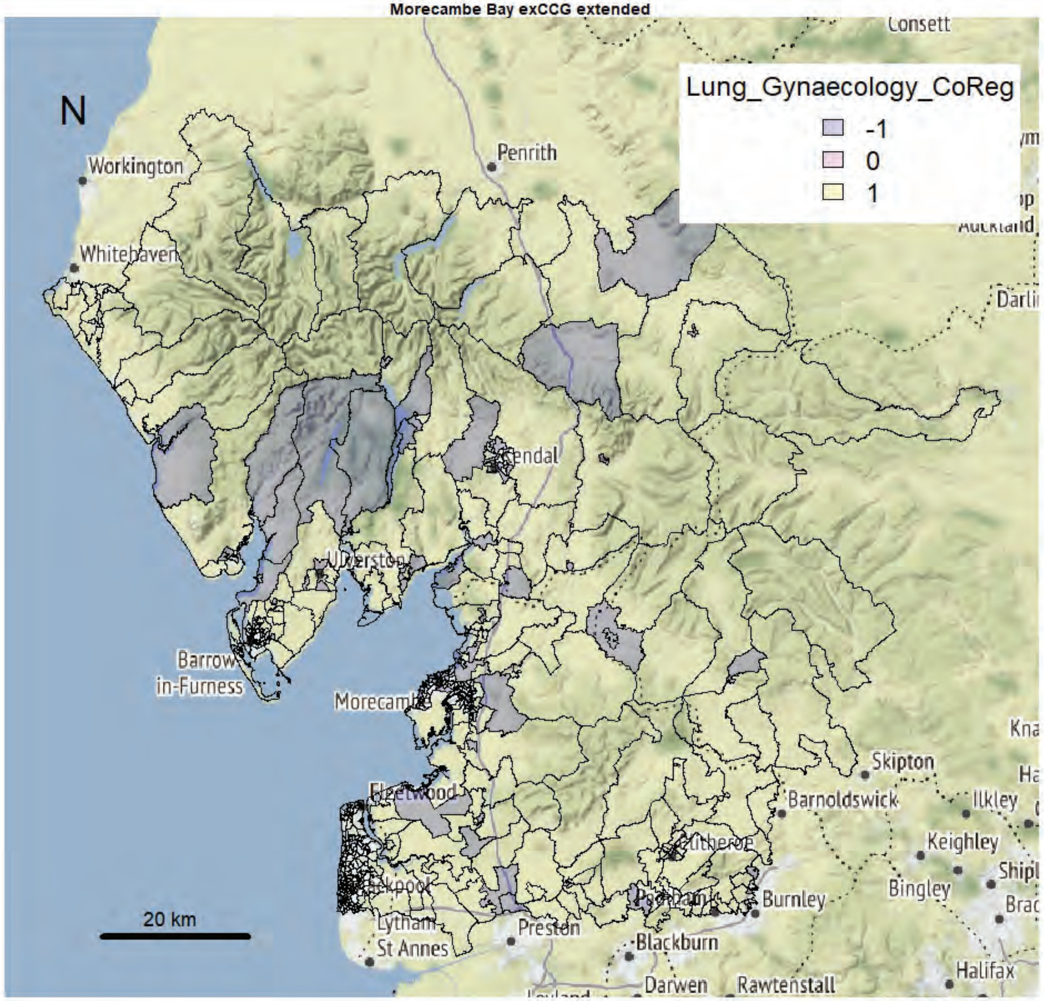

Morecambe Bay exCCG extended

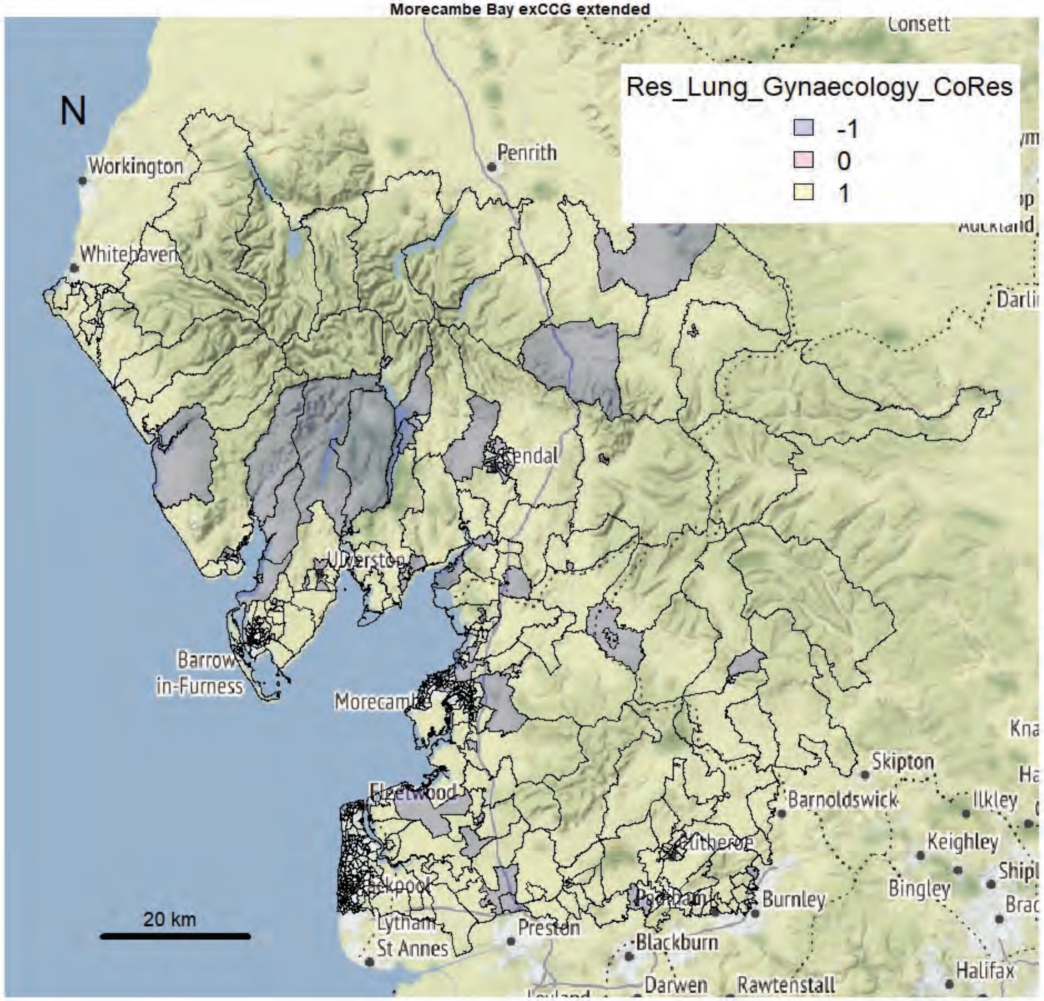

Morecambe Bay exCCG extended

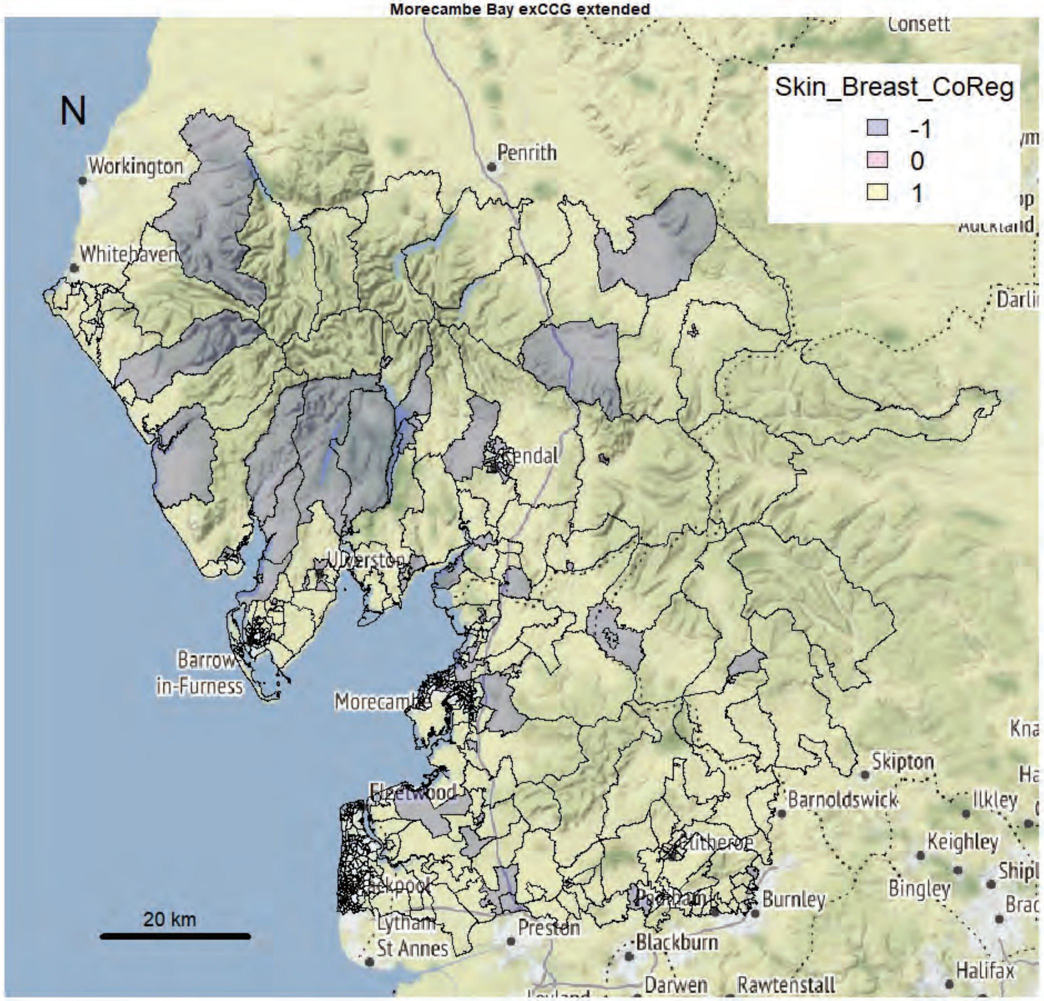

Morecambe Bay exCCG extended

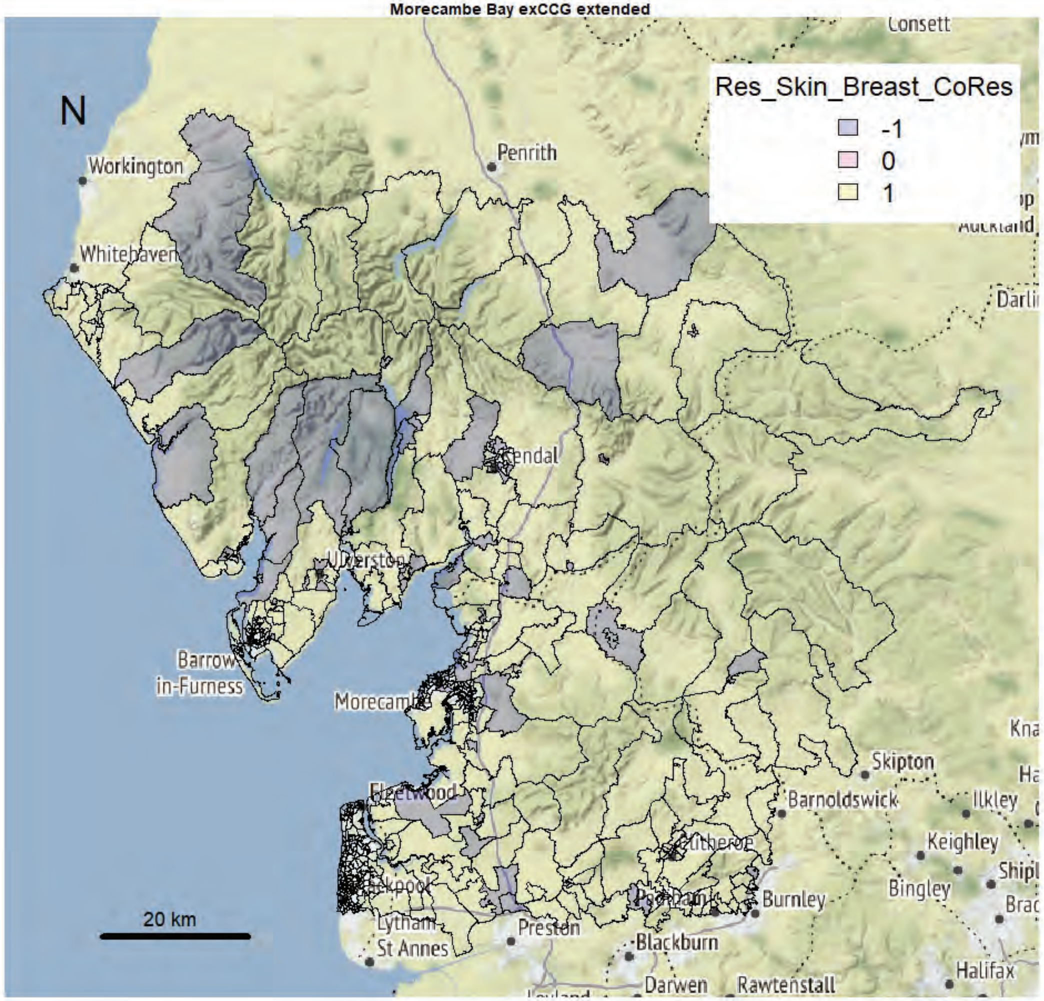

Morecambe Bay exCCG extended

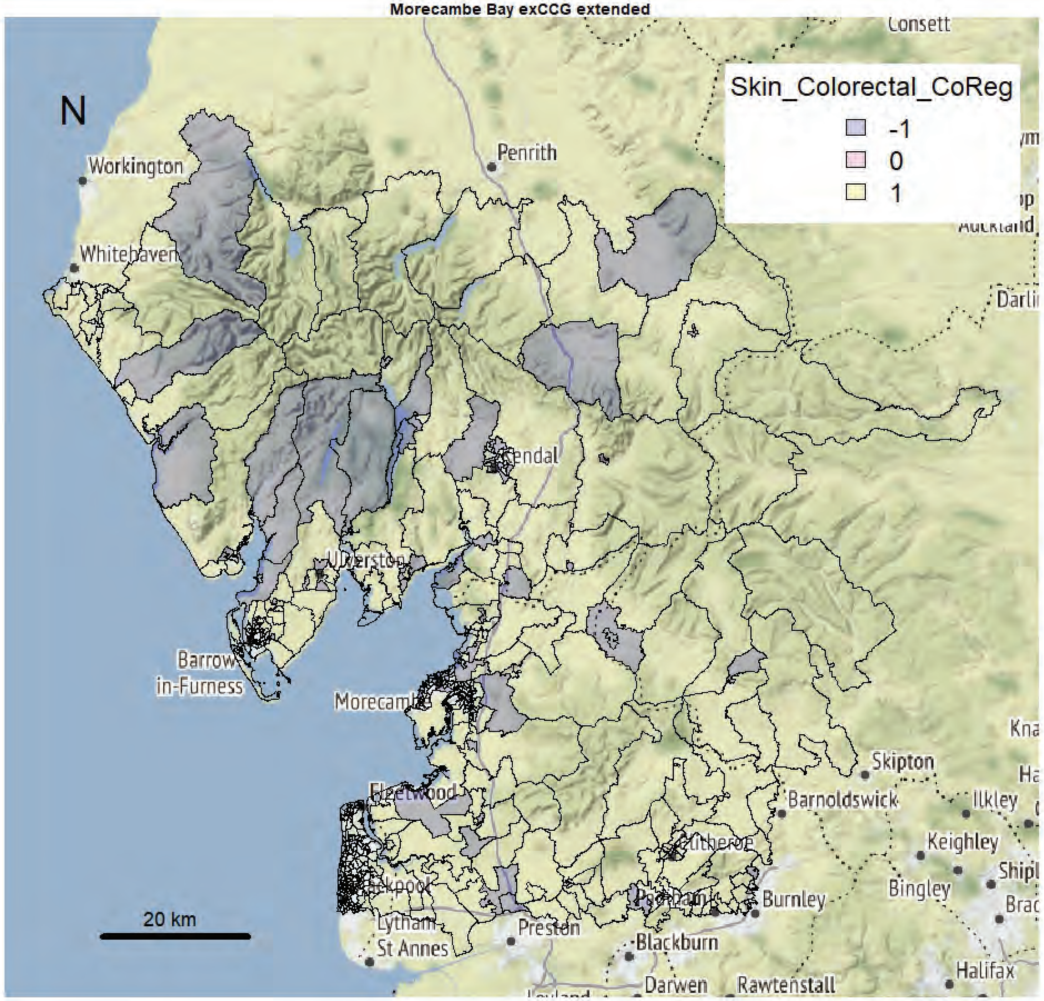

Morecambe Bay exCCG extended

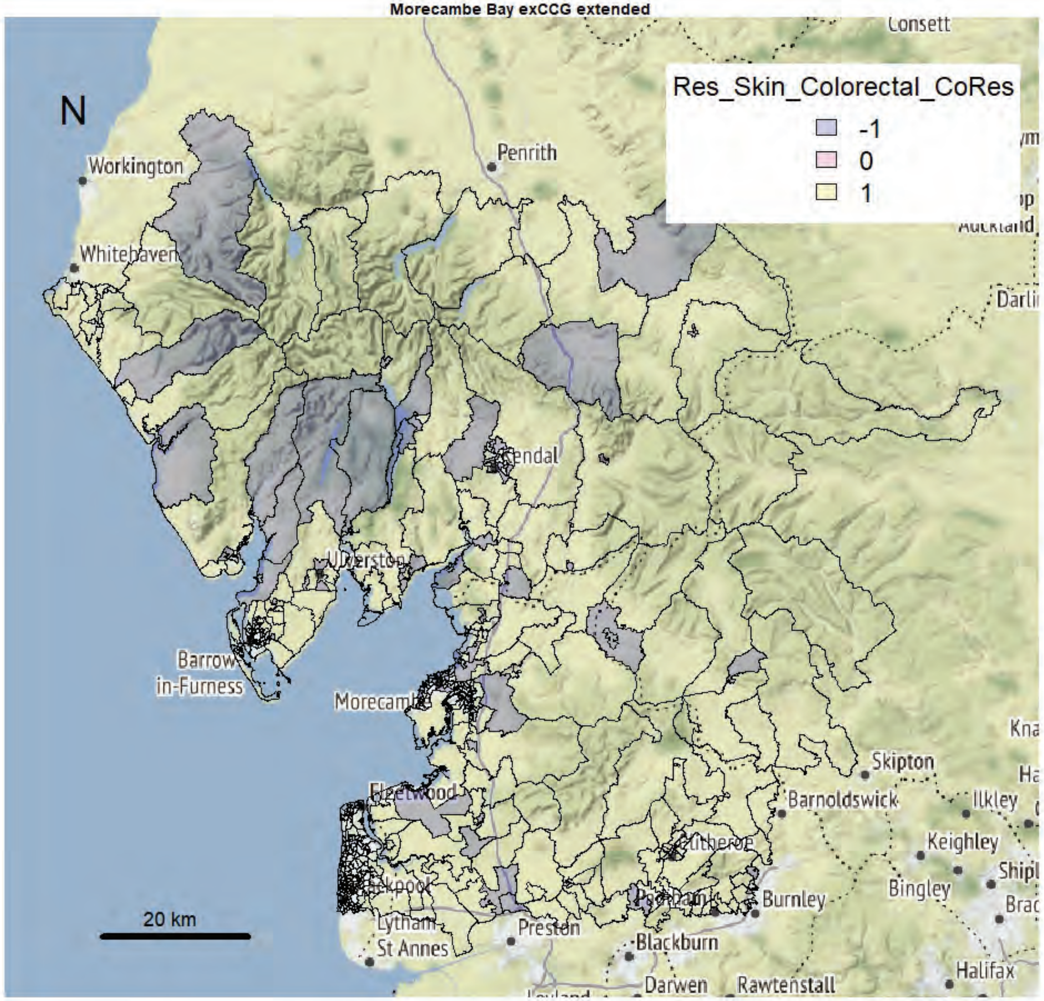

Morecambe Bay exCCG extended

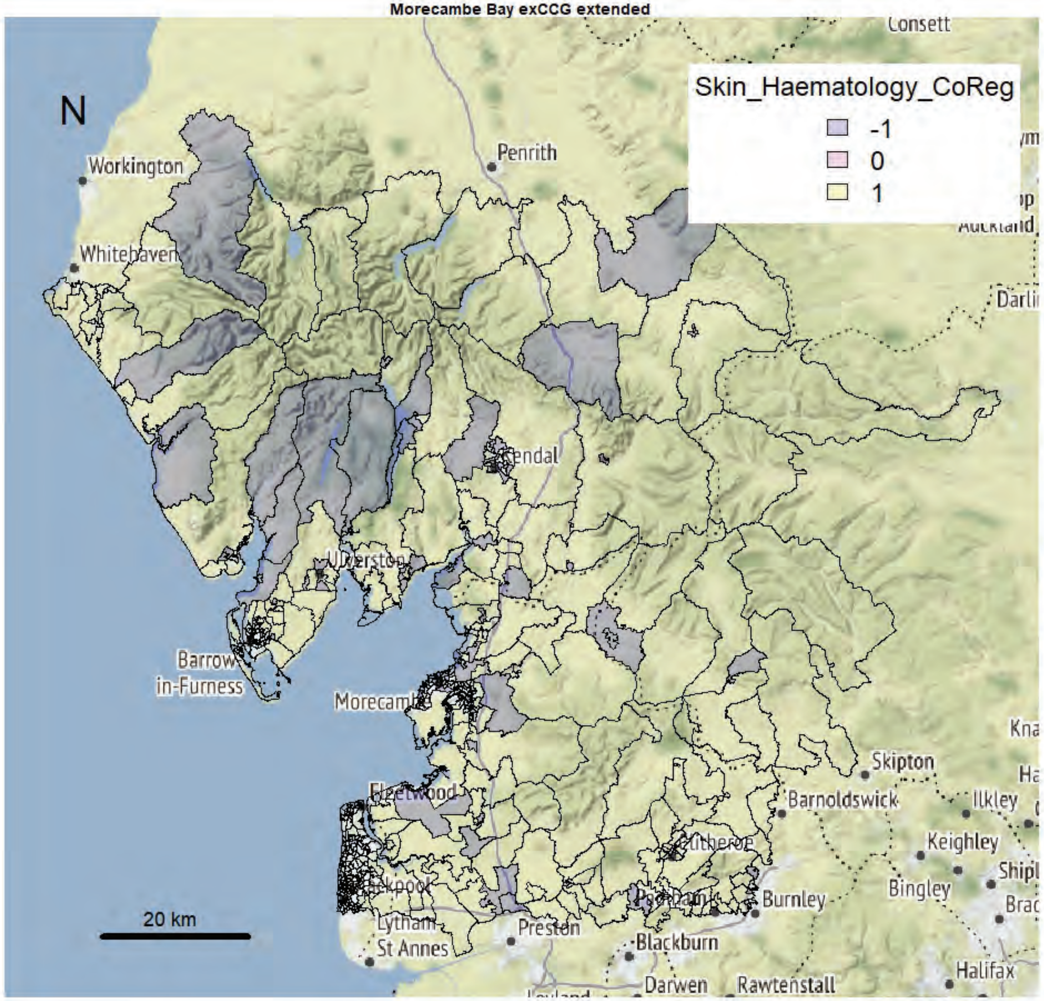

Morecambe Bay exCCG extended

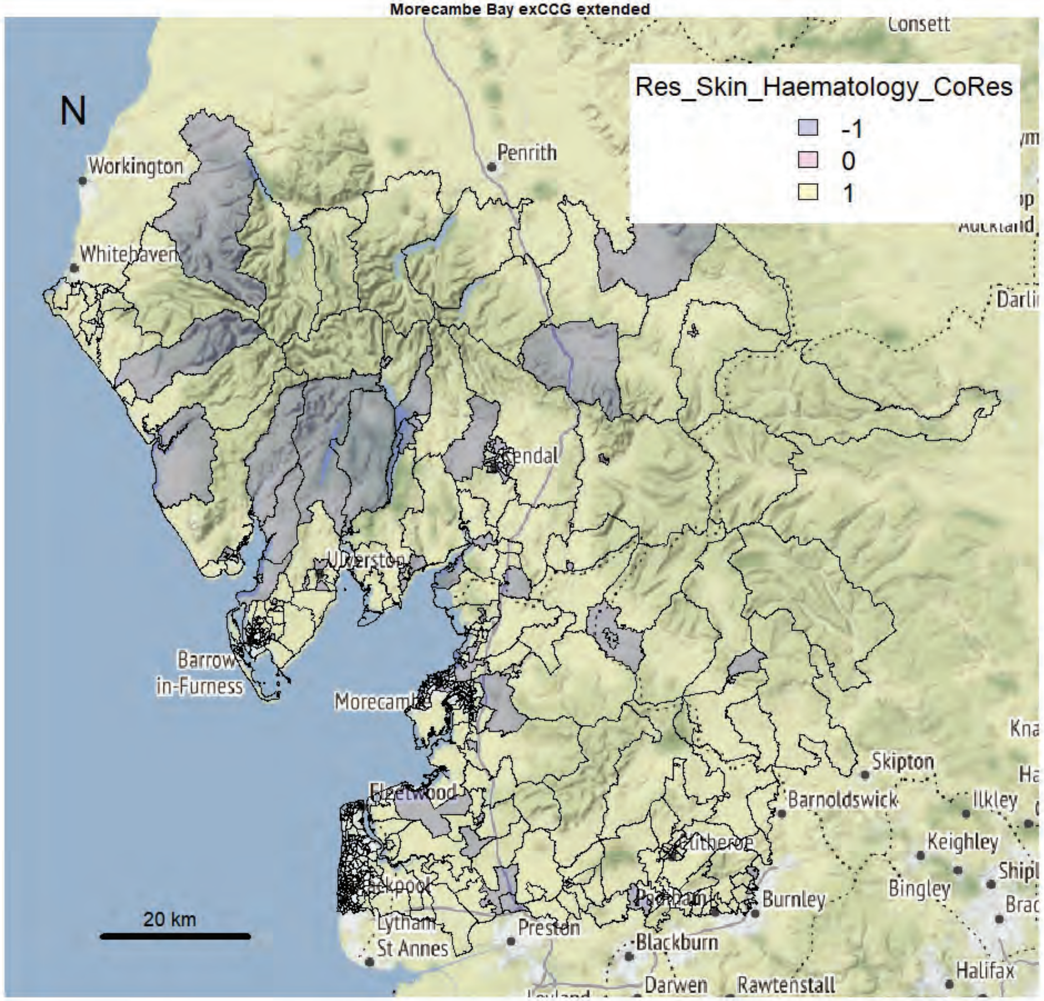

Morecambe Bay exCCG extended

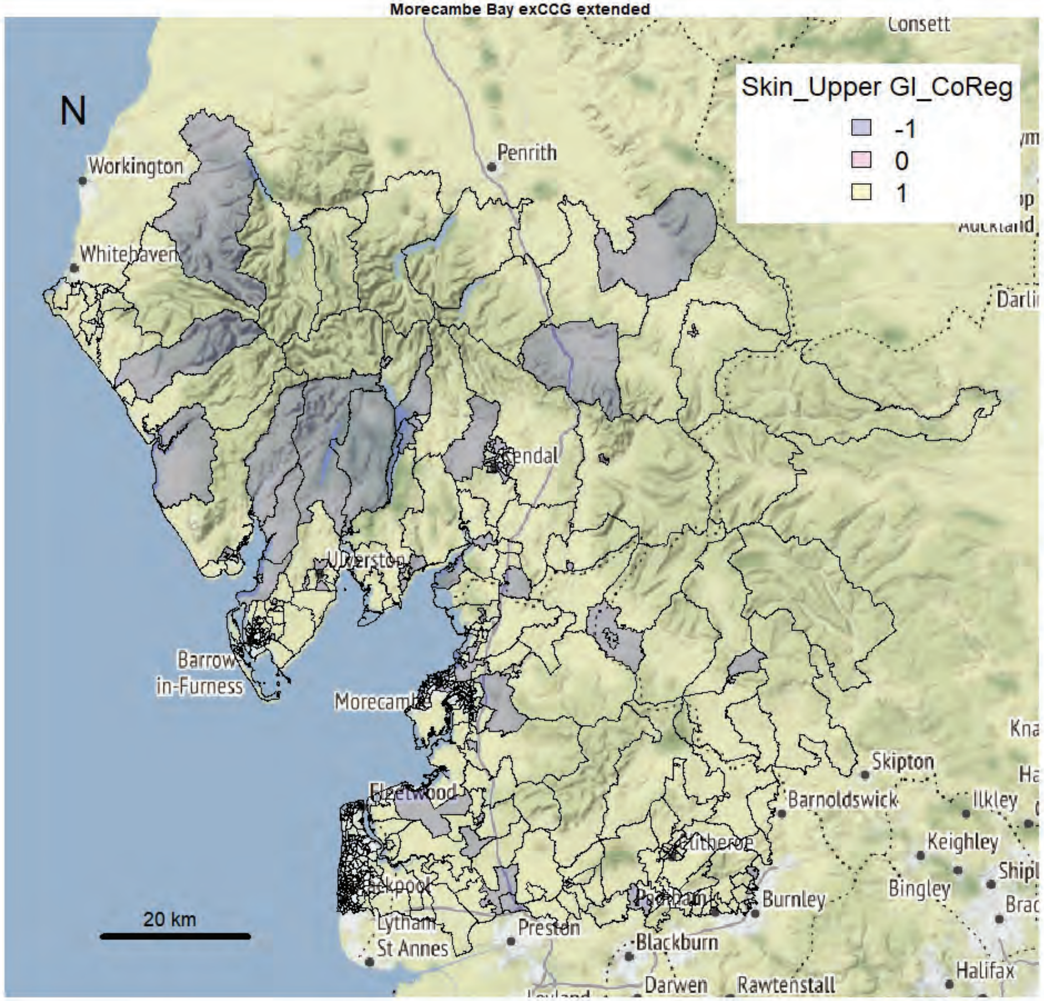

Morecambe Bay exCCG extended

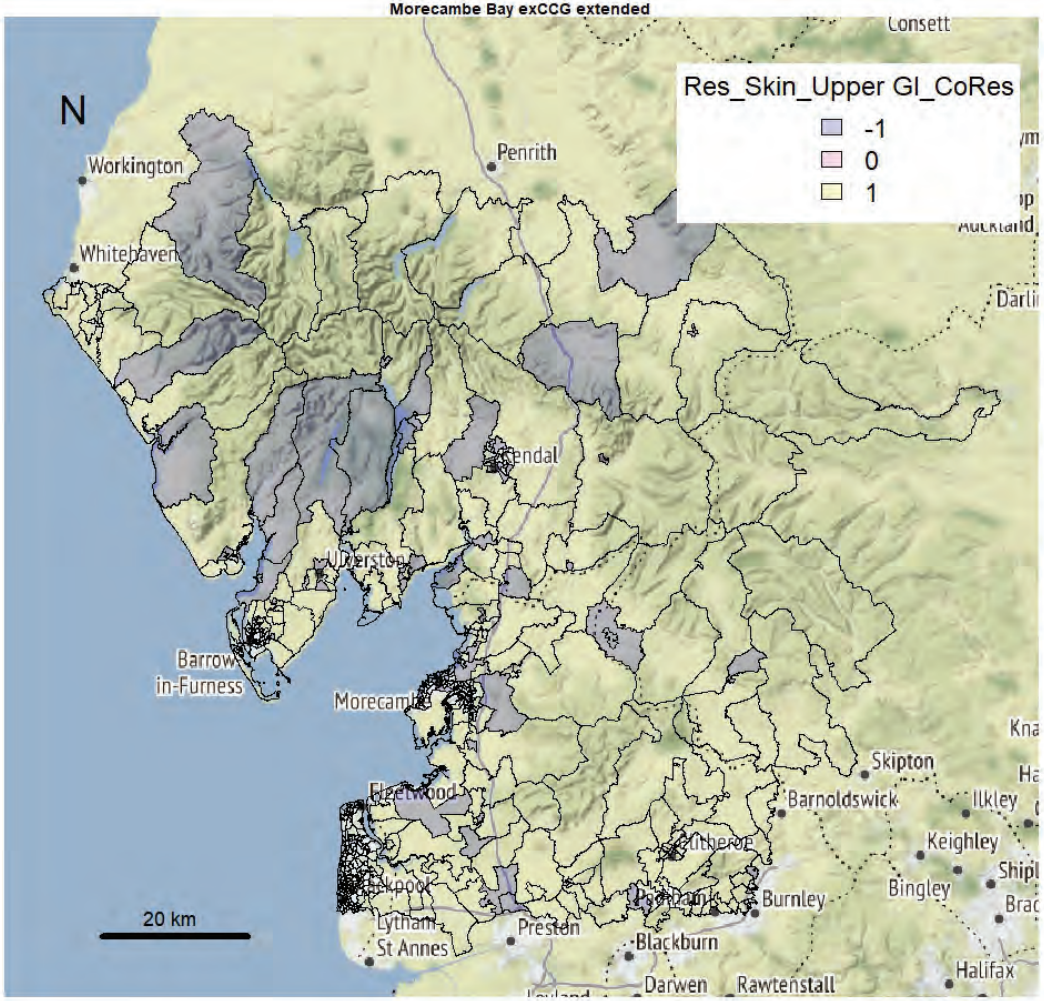

Morecambe Bay exCCG extended

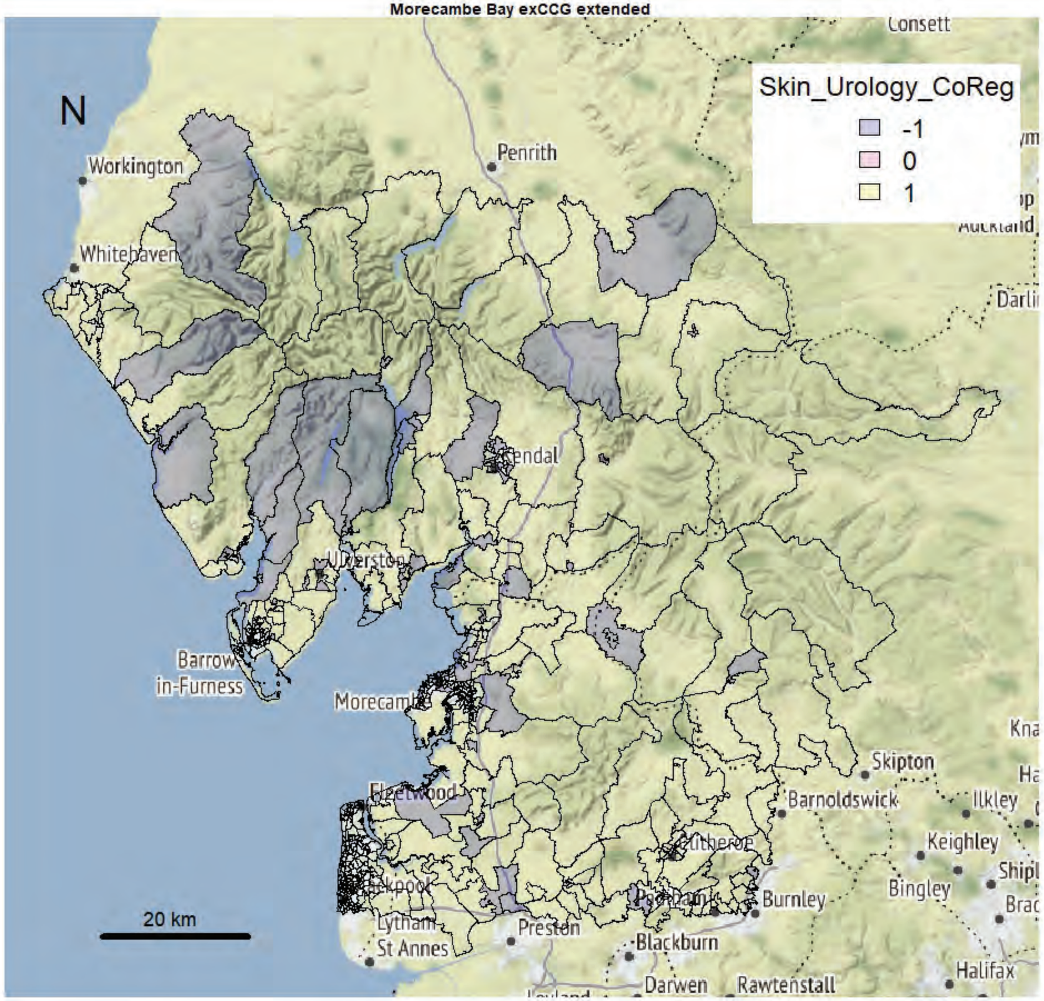

Morecambe Bay exCCG extended

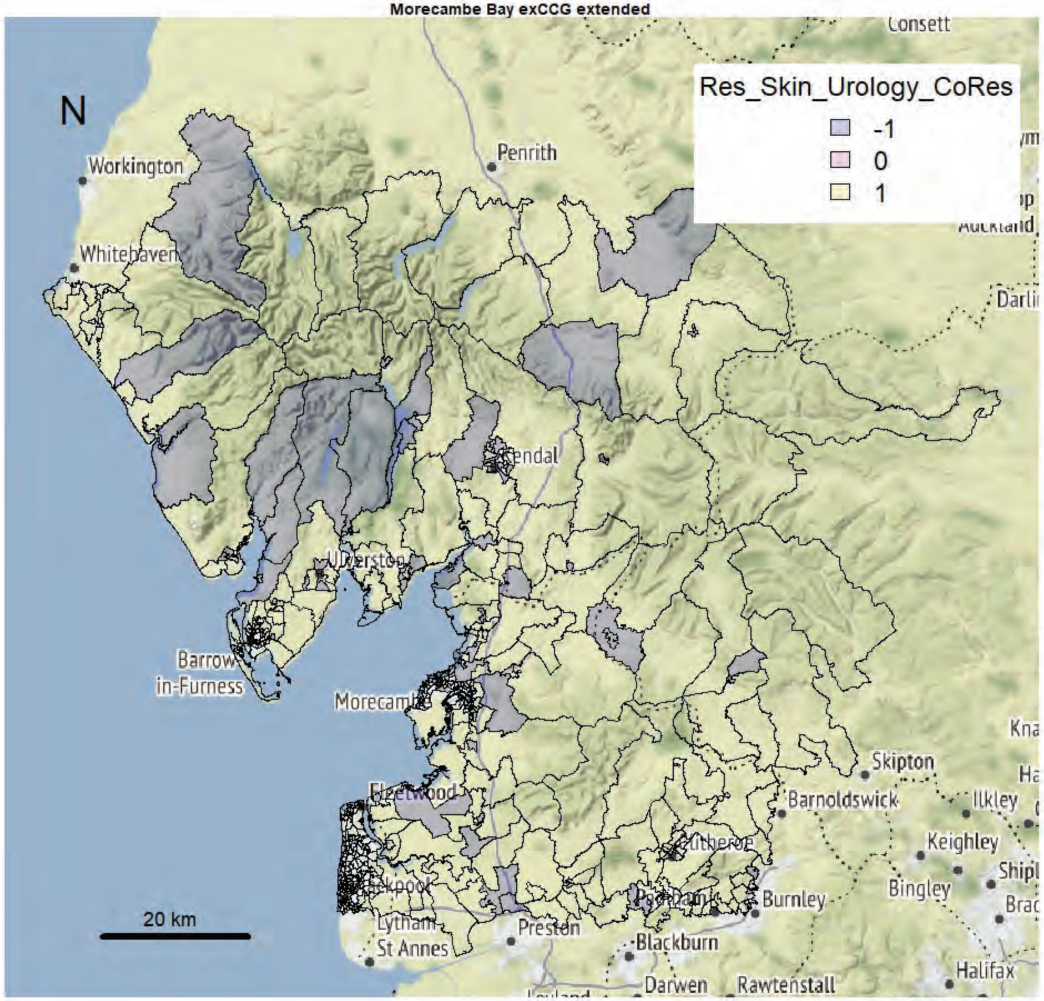

Morecambe Bay exCCG extended

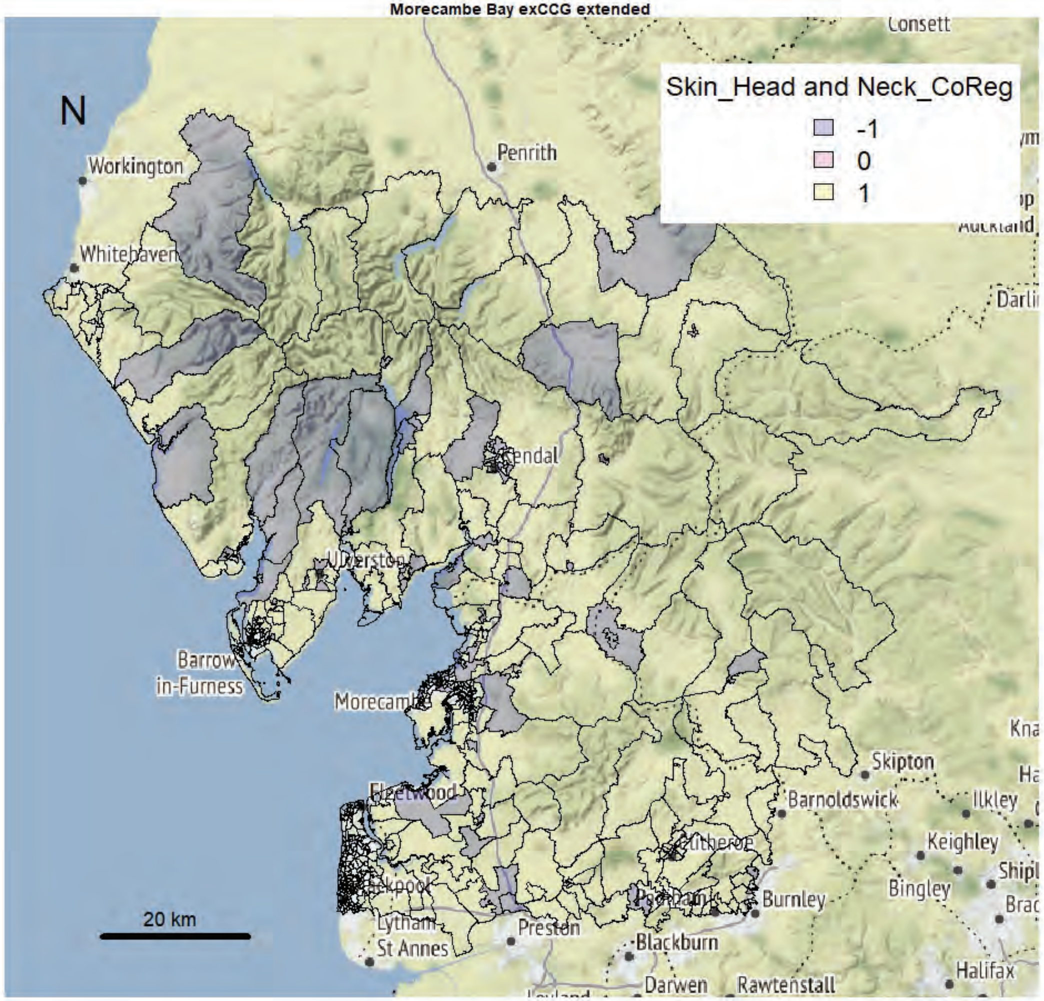

Morecambe Bay exCCG extended

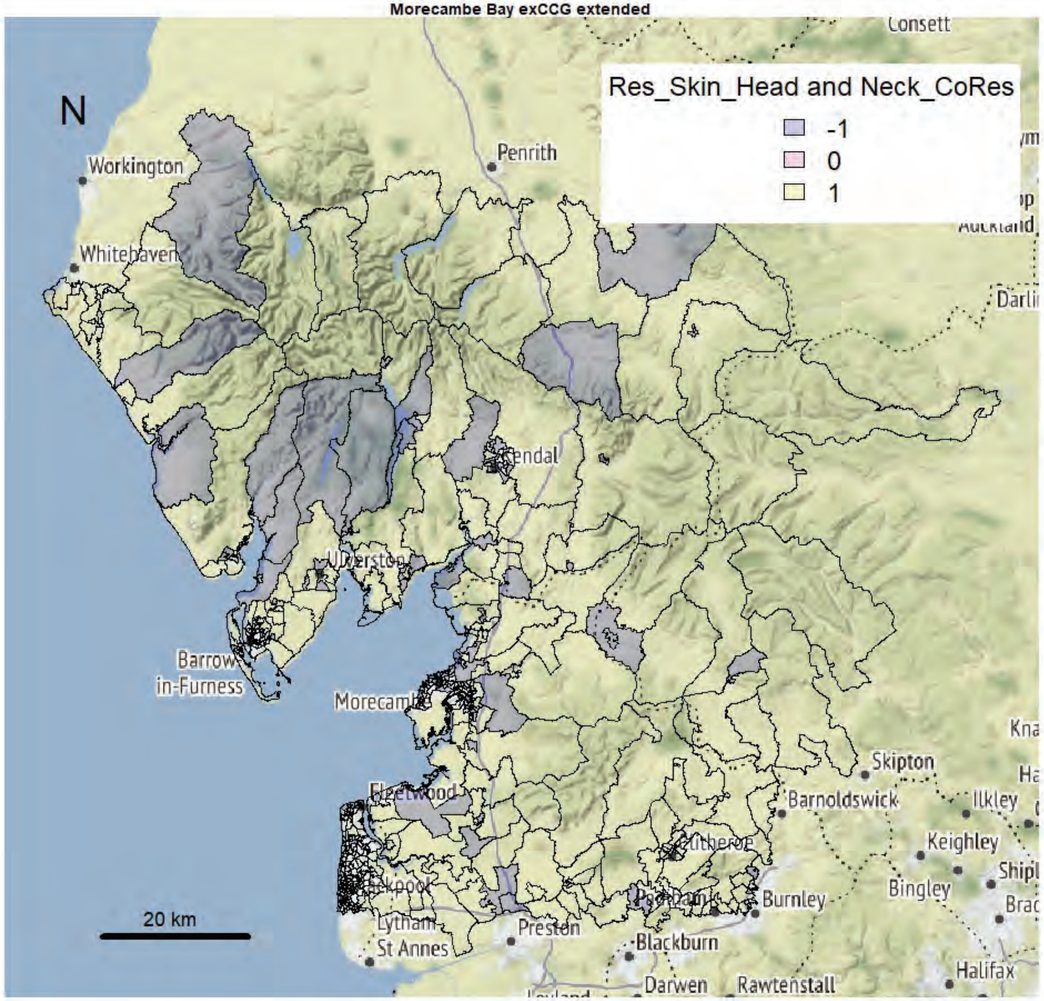

Morecambe Bay exCCG extended

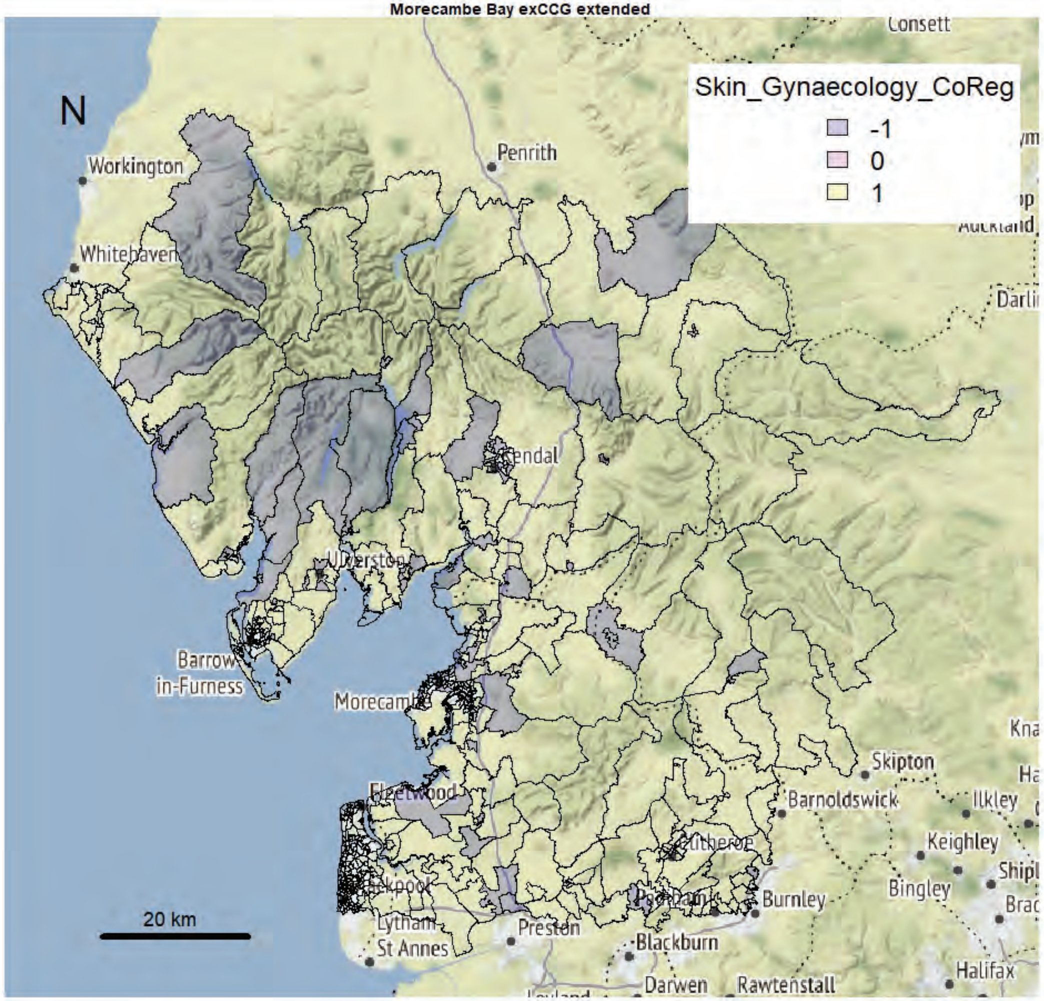

Morecambe Bay exCCG extended

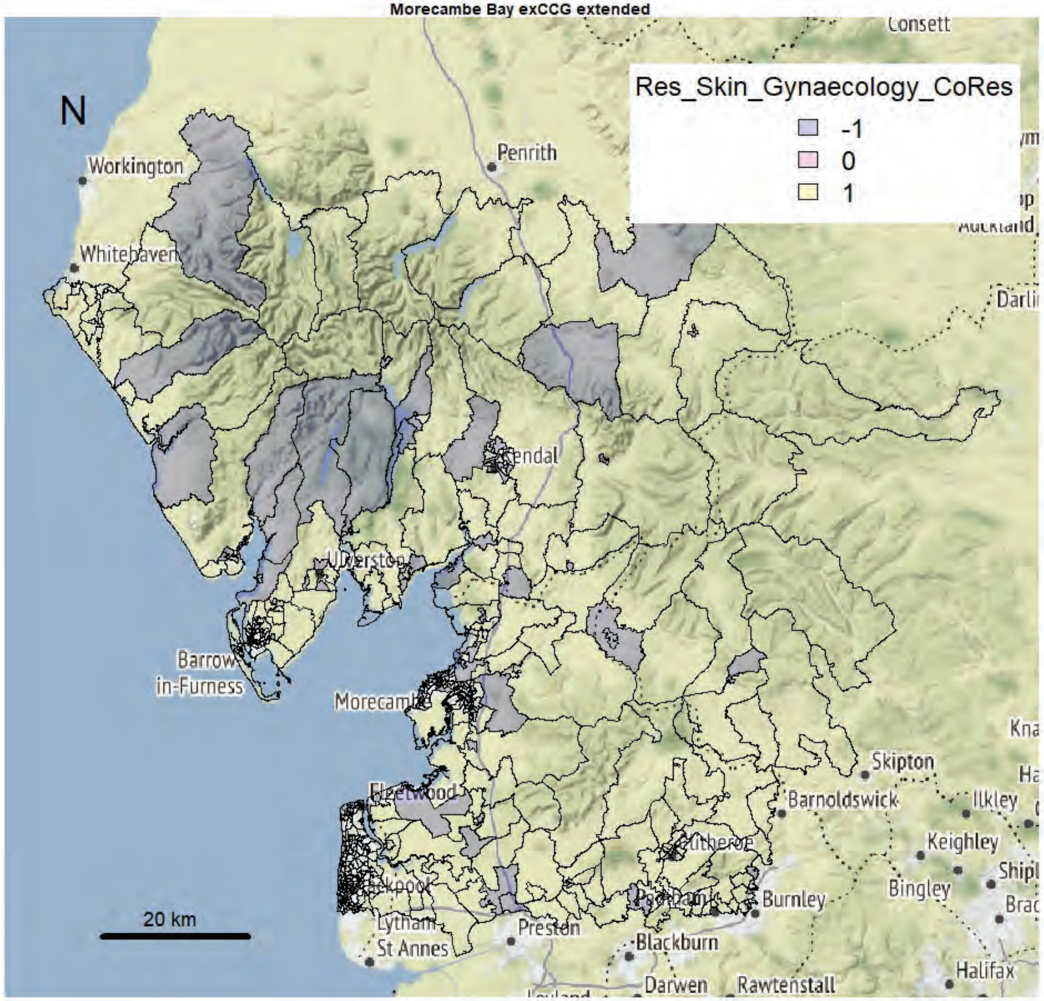

Morecambe Bay exCCG extended

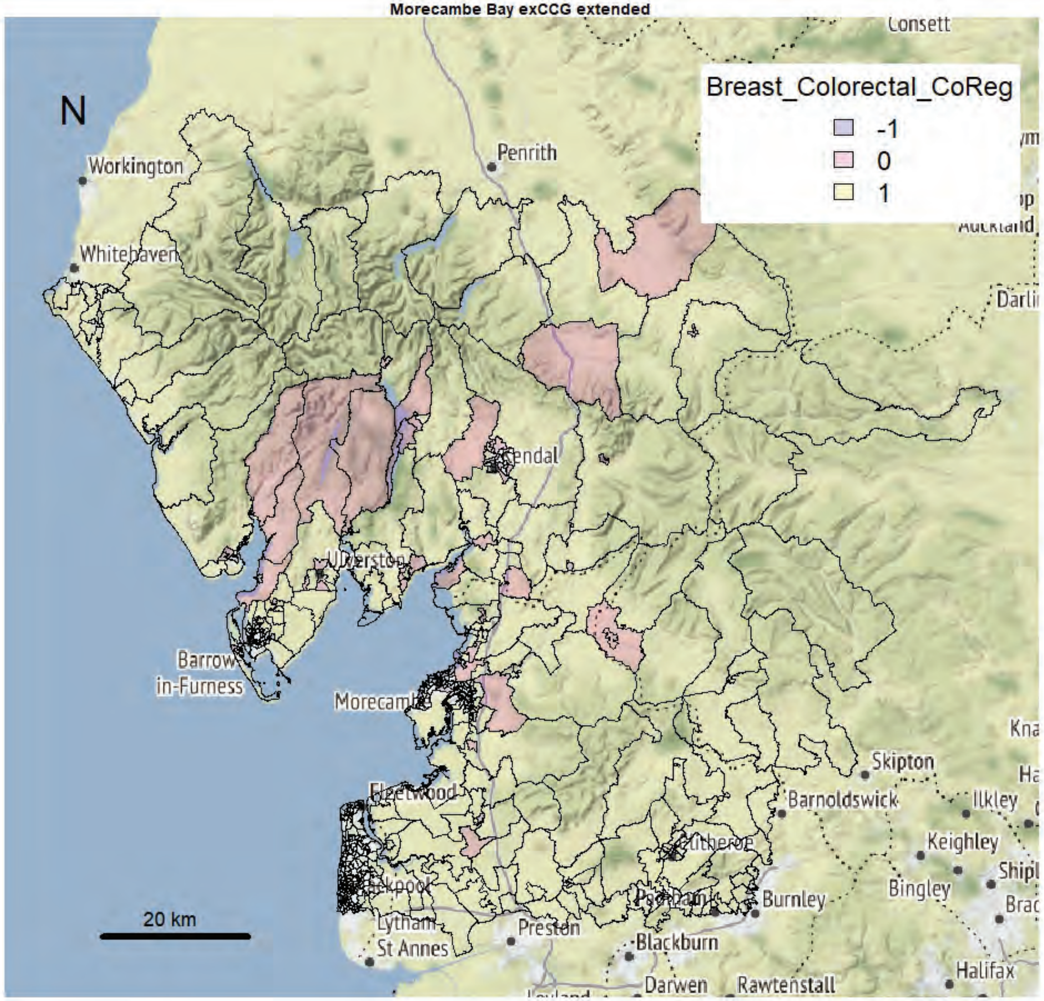

Morecambe Bay exCCG extended

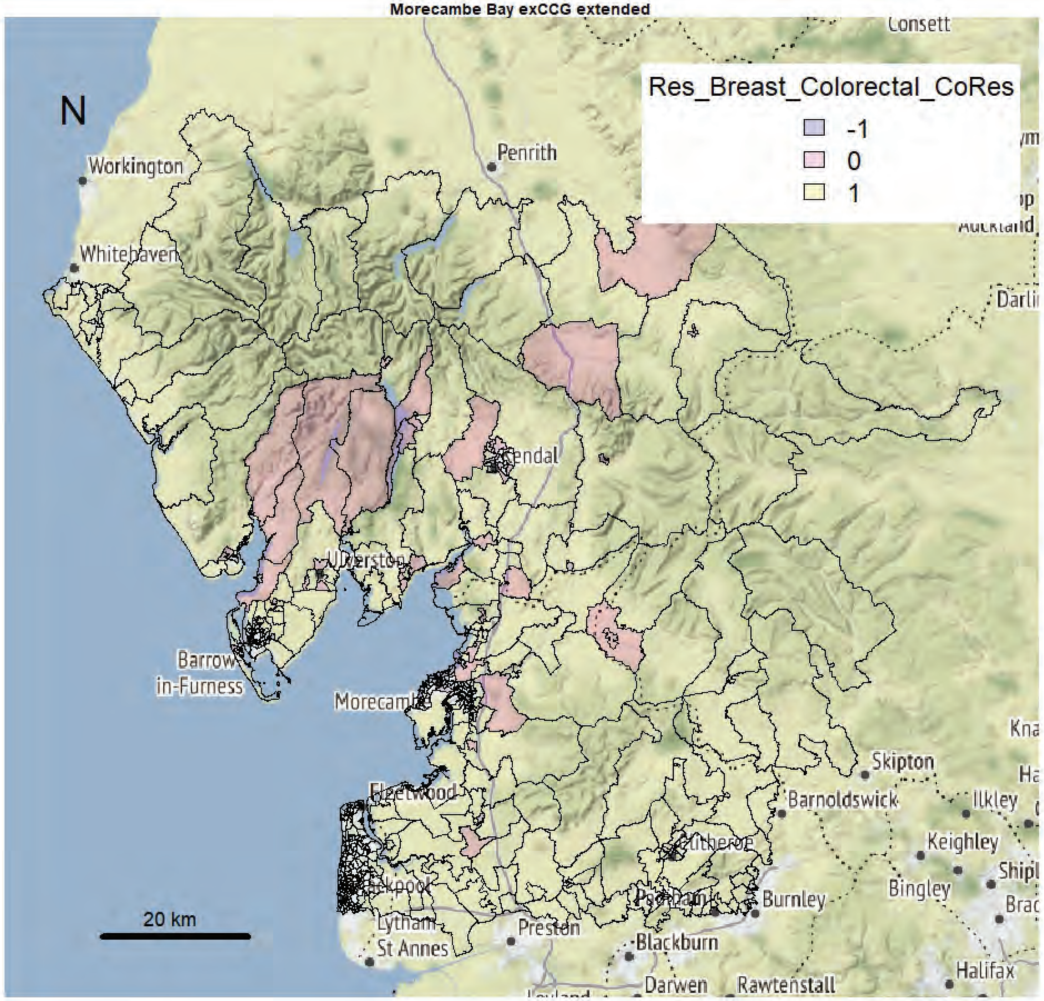

Morecambe Bay exCCG extended

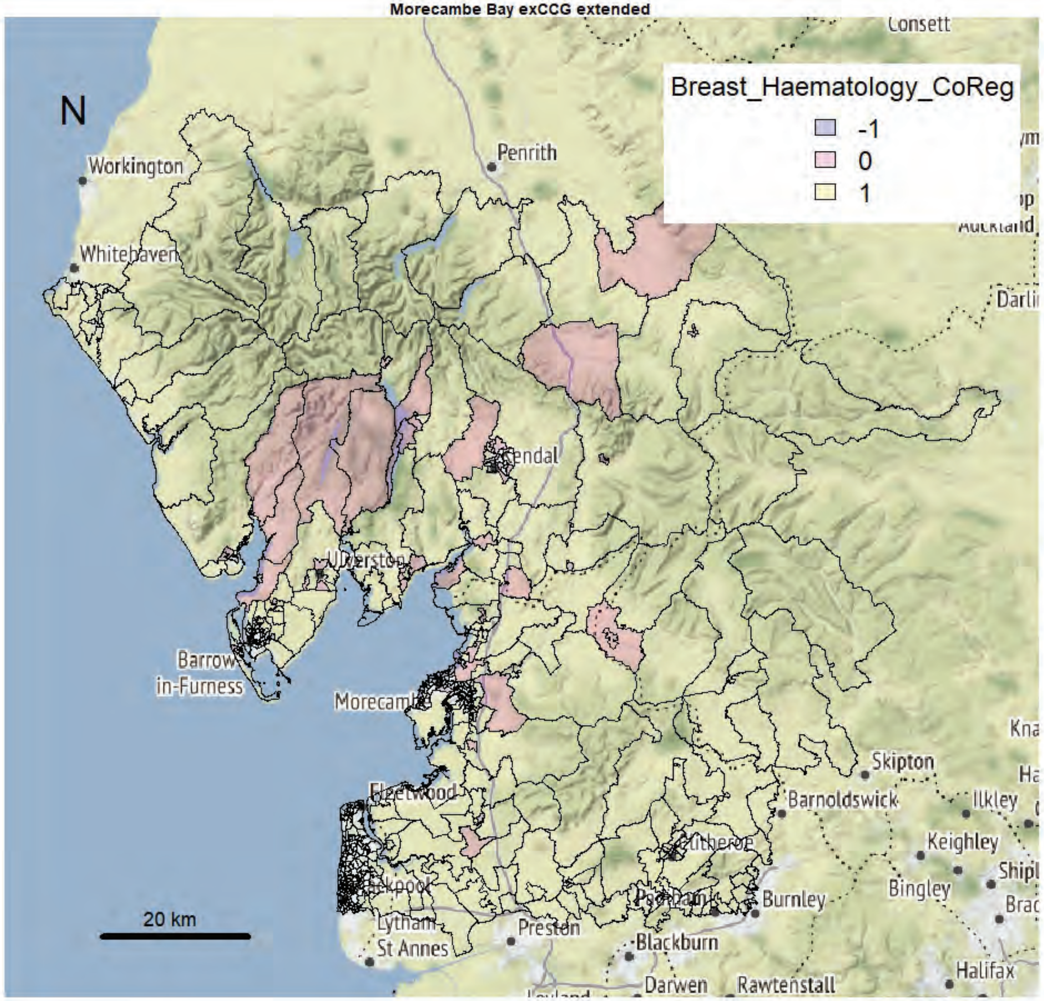

Morecambe Bay exCCG extended

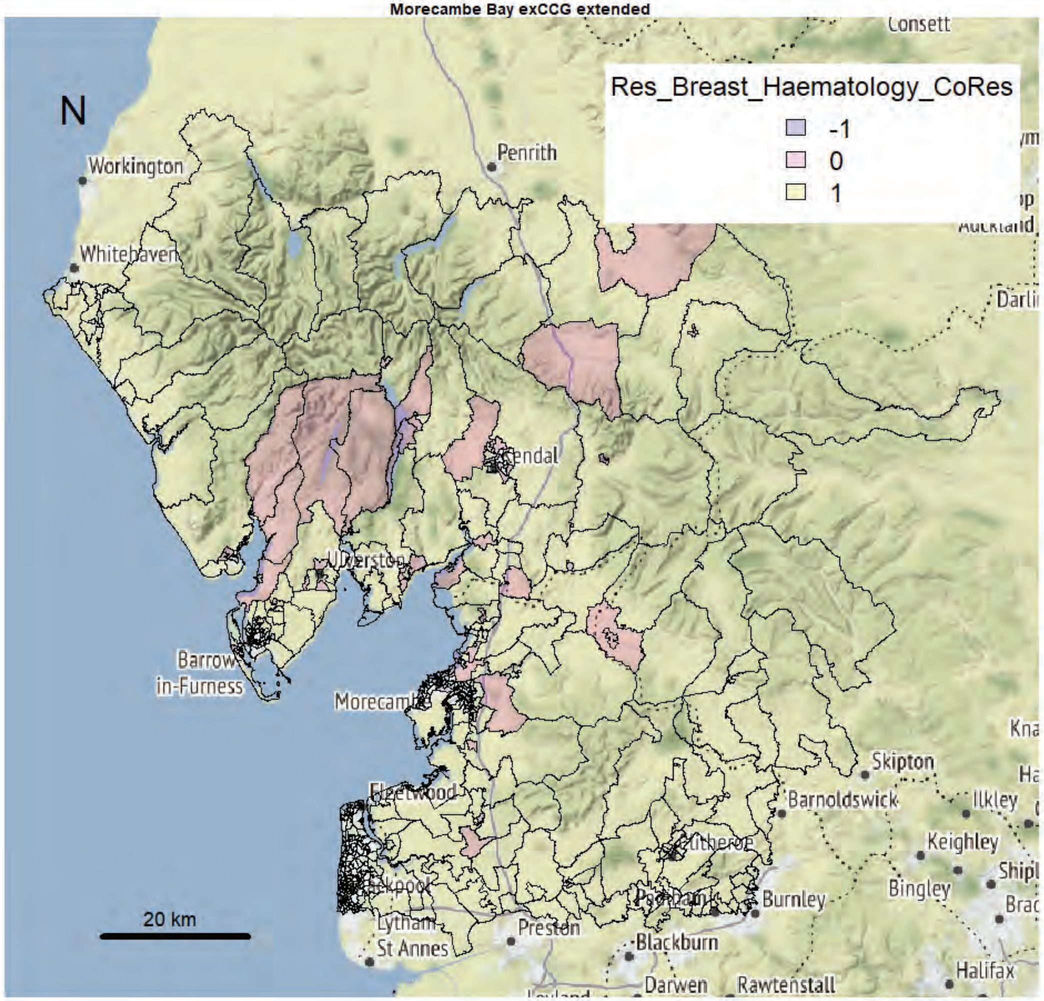

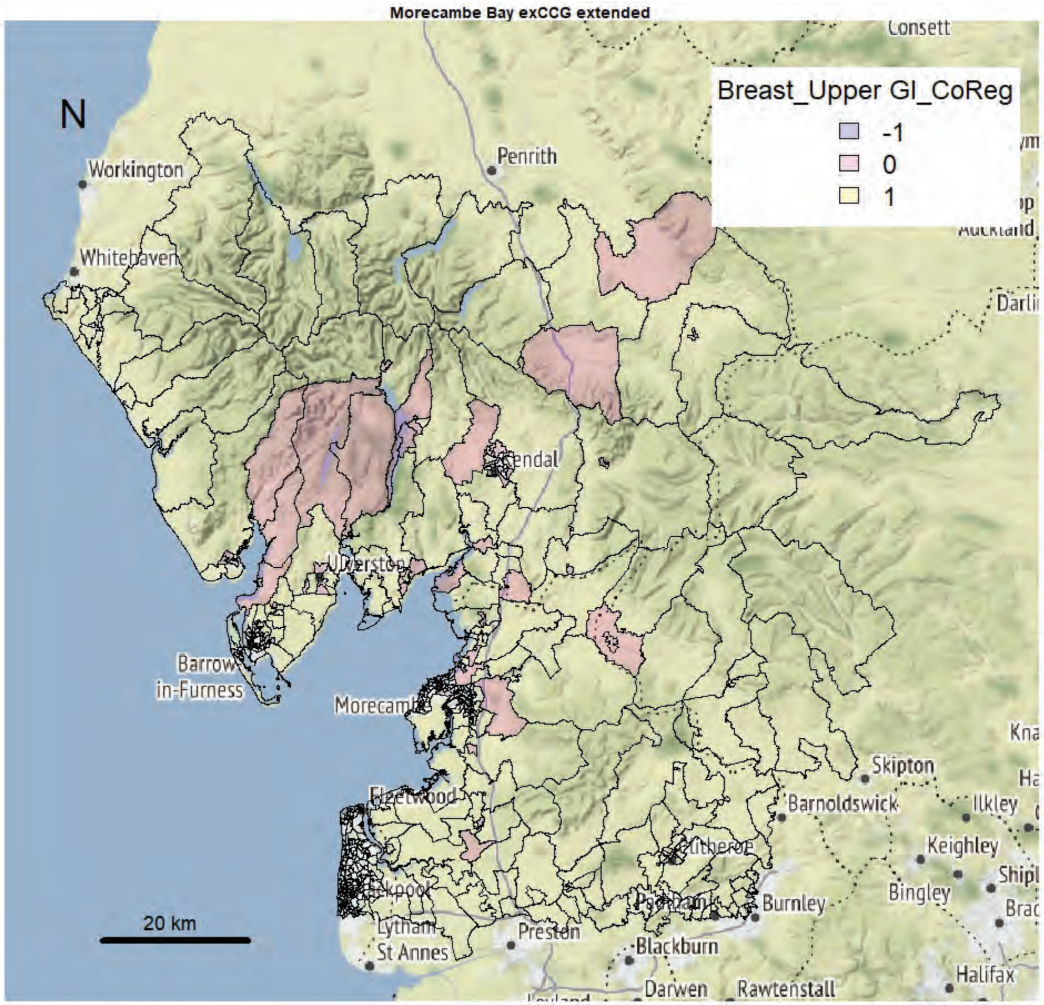
Morecambe Bay exCCG extended
Breast_Upper GI_CoReg
-1
0
1
N
20 km
Workington
Penrith
Whitehaven
Kendal
Ulverston
Barrow-in-Furness
Morecambe
Fleetwood
Blackpool
Lytham St Annes
Preston
Blackburn
Darwen
Rawtenstall
Burnley
Clitheroe
Skipton
Barnoldswick
Ilkley
Keighley
Bingley
Halifax
Consett

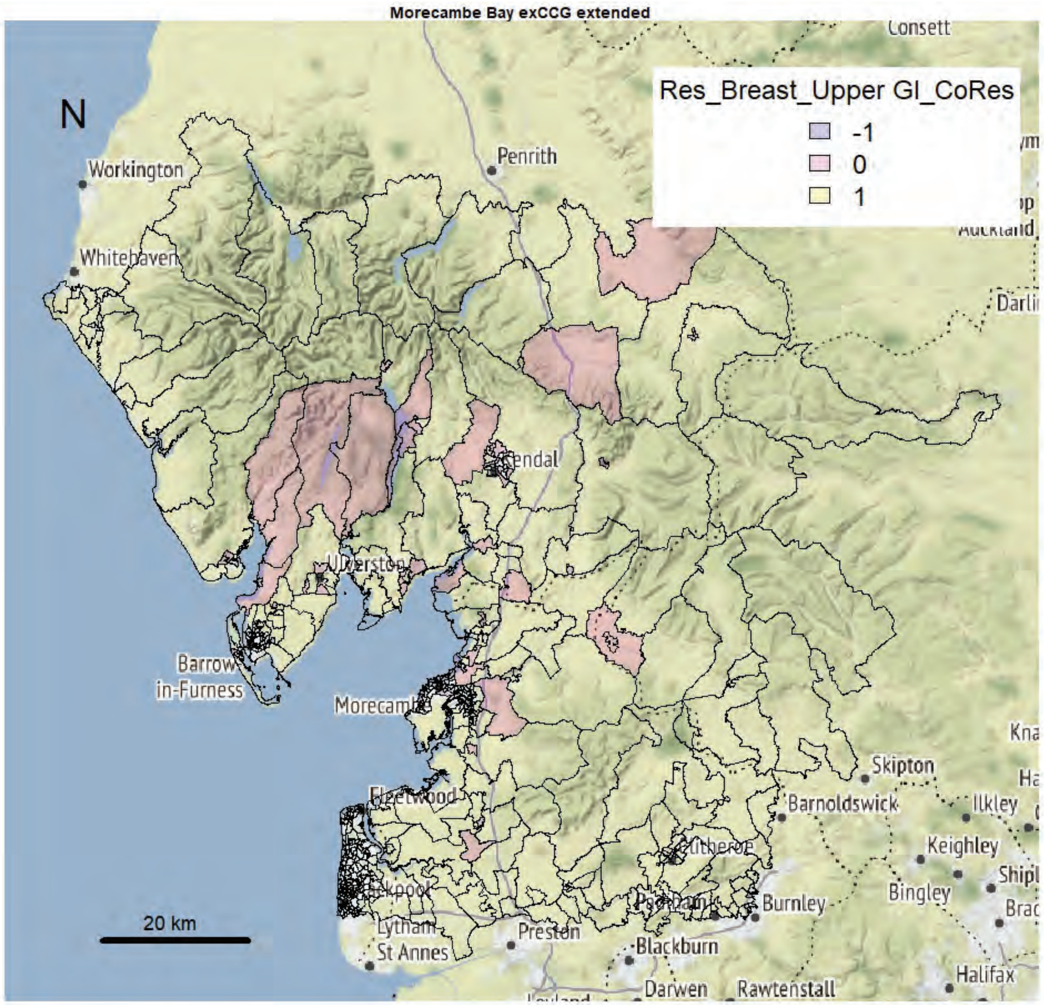
Morecambe Bay exCCG extended
Res_Breast_Upper GI_CoRes
-1
0
1
N
20 km
Workington
Penrith
Whitehaven
Kendal
Ulverston
Barrow-in-Furness
Morecambe
Fleetwood
Blackpool
Lytham St Annes
Preston
Blackburn
Darwen
Rawtenstall
Burnley
Clitheroe
Skipton
Barnoldswick
Ilkley
Keighley
Bingley
Halifax
Consett

Morecambe Bay exCCG extended

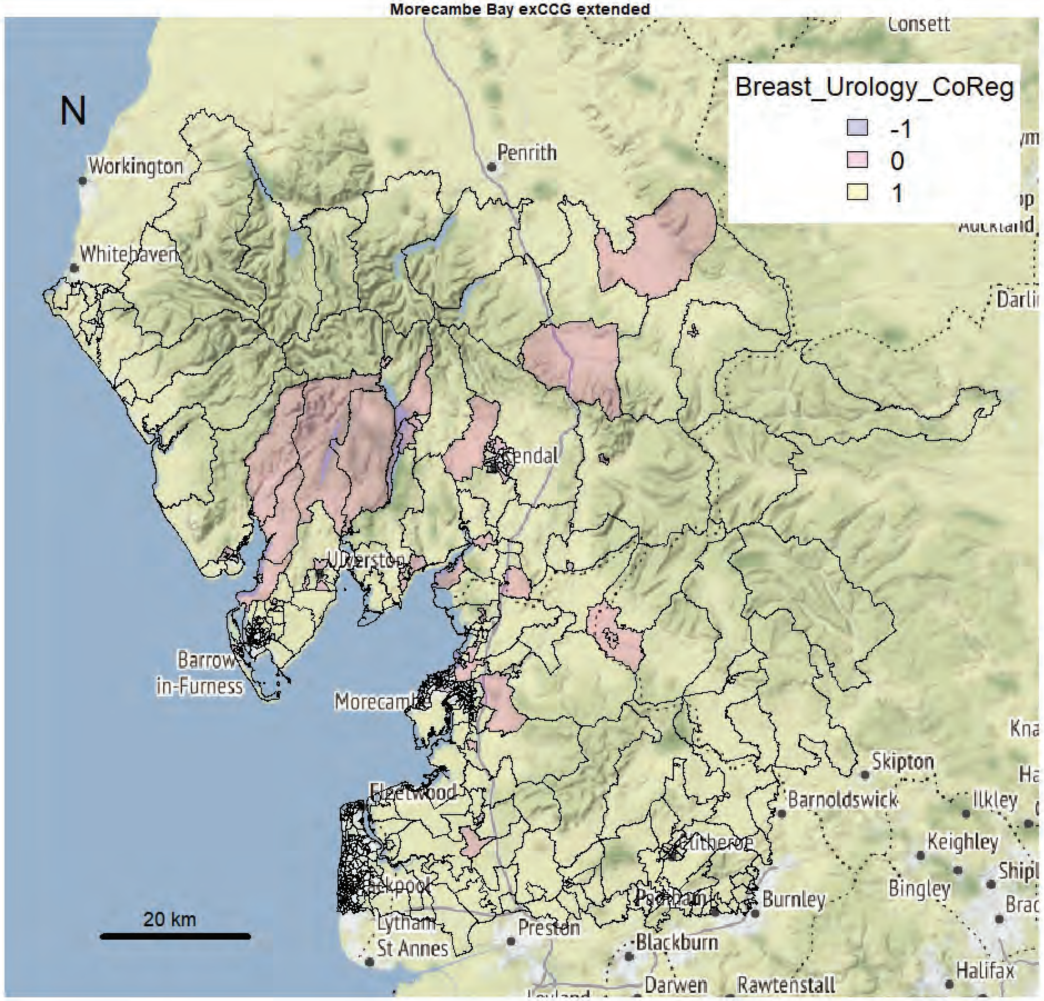

Morecambe Bay exCCG extended

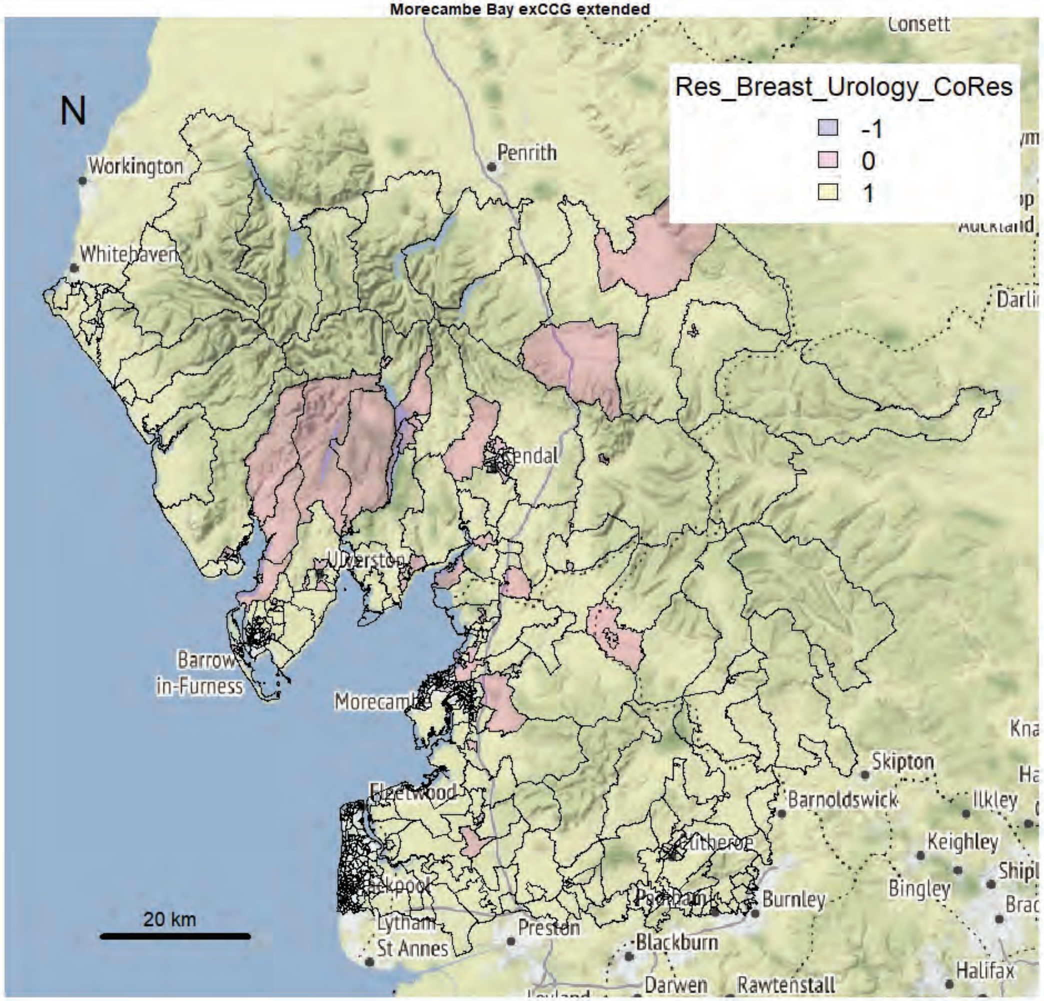

Morecambe Bay exCCG extended

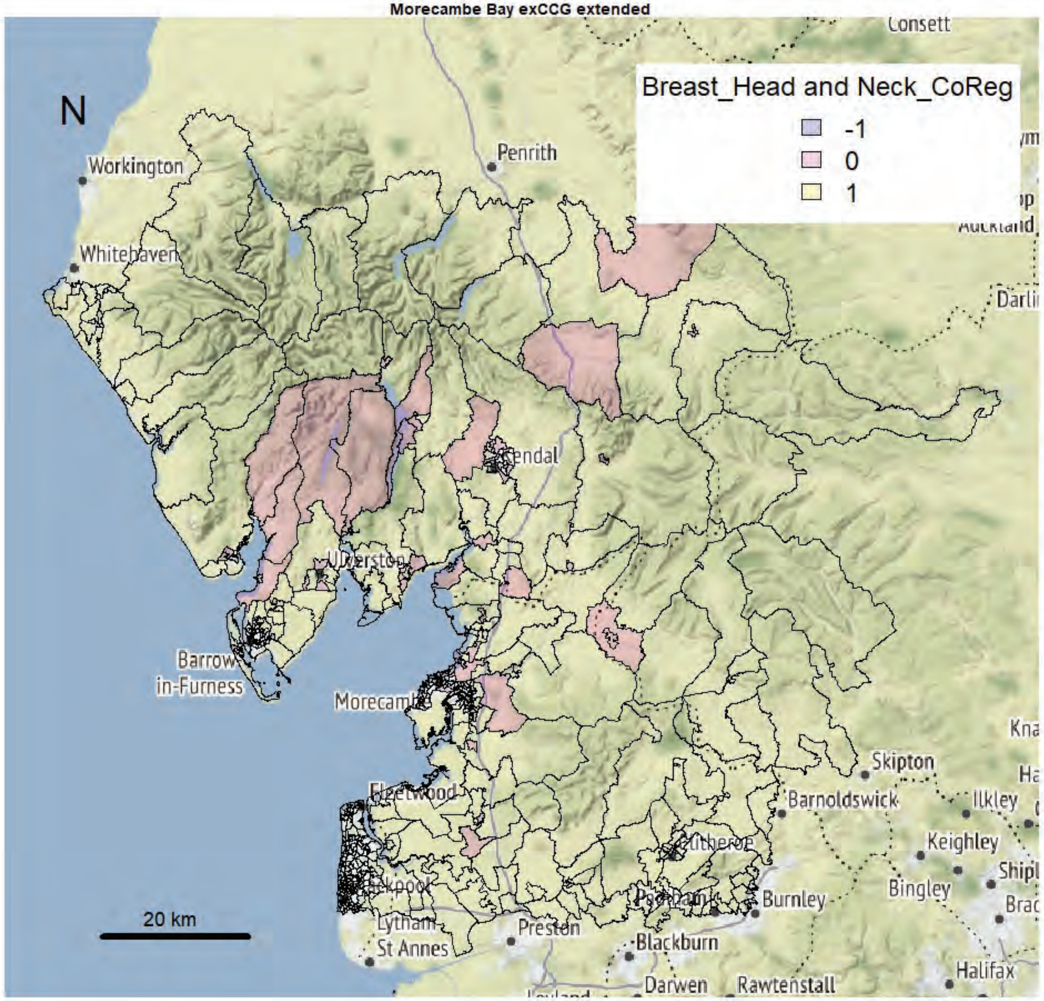

Morecambe Bay exCCG extended

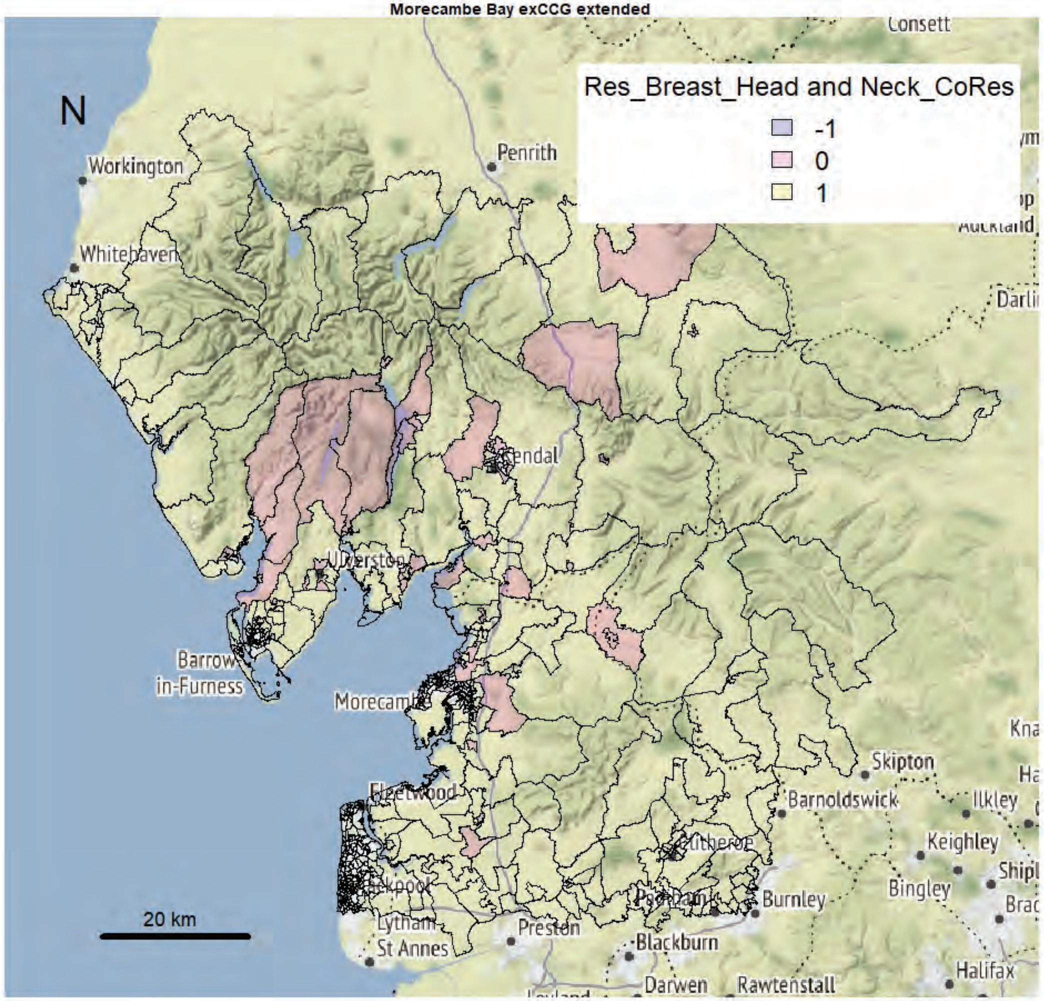

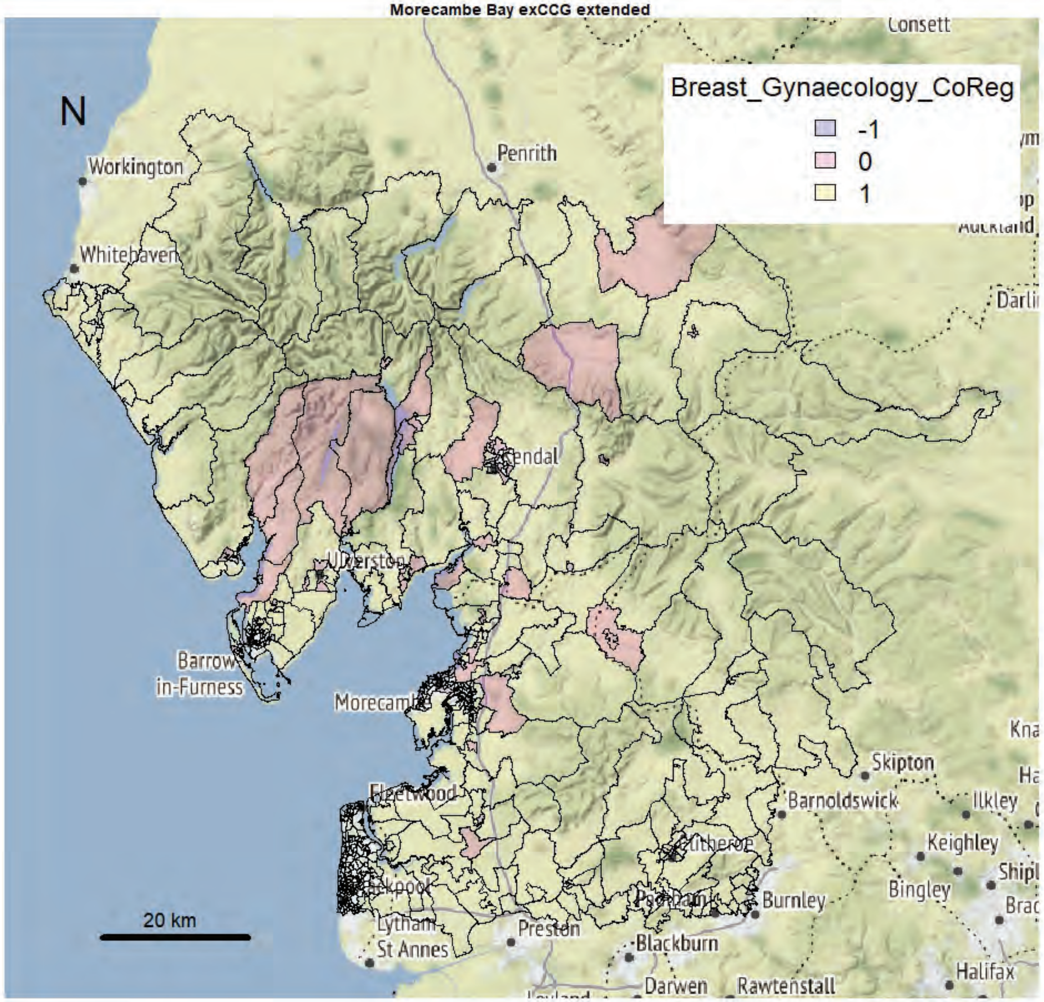
Morecambe Bay exCCG extended
Breast_Gynaecology_CoReg
-1
0
1
N
20 km
Workington
Penrith
Whitehaven
Kendal
Ulverston
Barrow-in-Furness
Morecambe
Fleetwood
Blackpool
Lytham St Annes
Preston
Blackburn
Darwen
Rawtenstall
Clitheroe
Padiham
Burnley
Skipton
Barnoldswick
Ilkley
Keighley
Bingley
Halifax
Consett

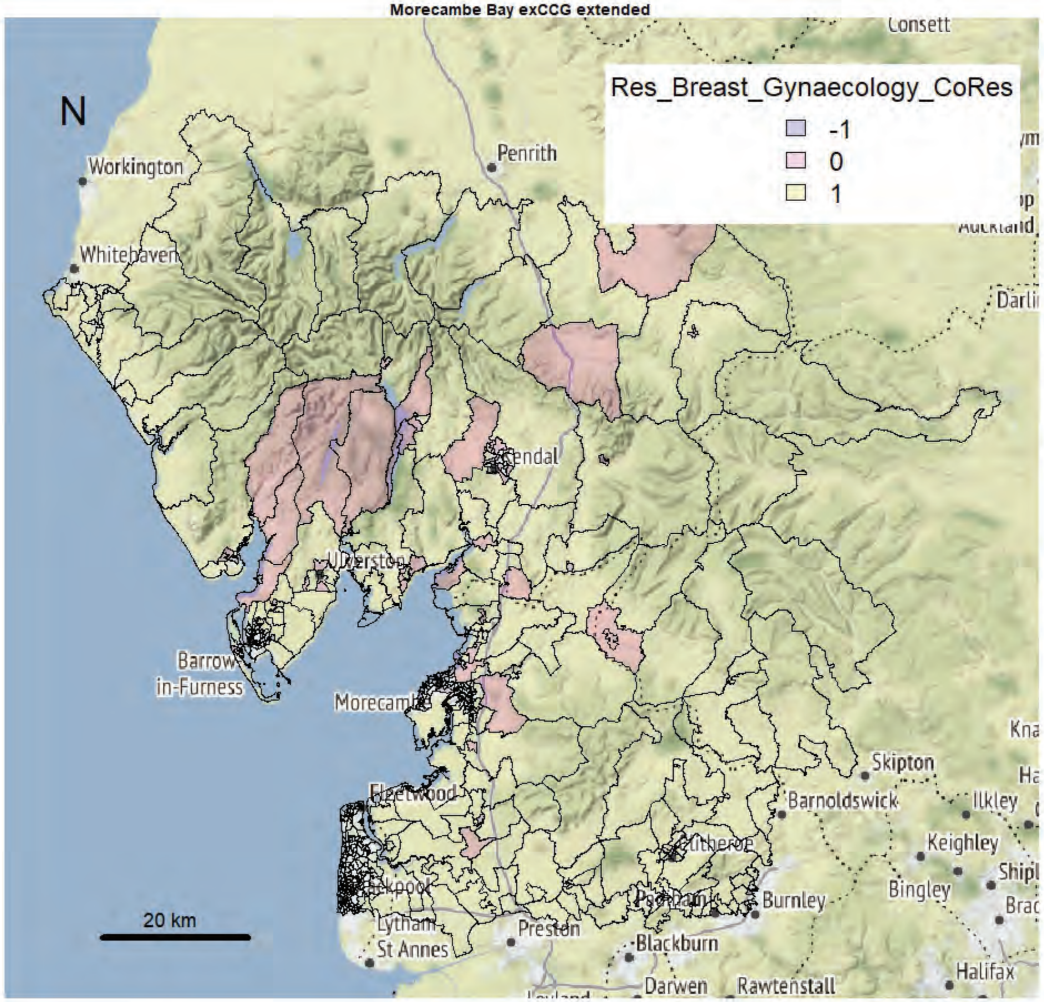
Morecambe Bay exCCG extended
Res_Breast_Gynaecology_CoRes
-1
0
1
N
20 km
Workington
Penrith
Whitehaven
Kendal
Ulverston
Barrow-in-Furness
Morecambe
Fleetwood
Blackpool
Lytham St Annes
Preston
Blackburn
Darwen
Rawtenstall
Clitheroe
Padiham
Burnley
Skipton
Barnoldswick
Ilkley
Keighley
Bingley
Halifax
Consett

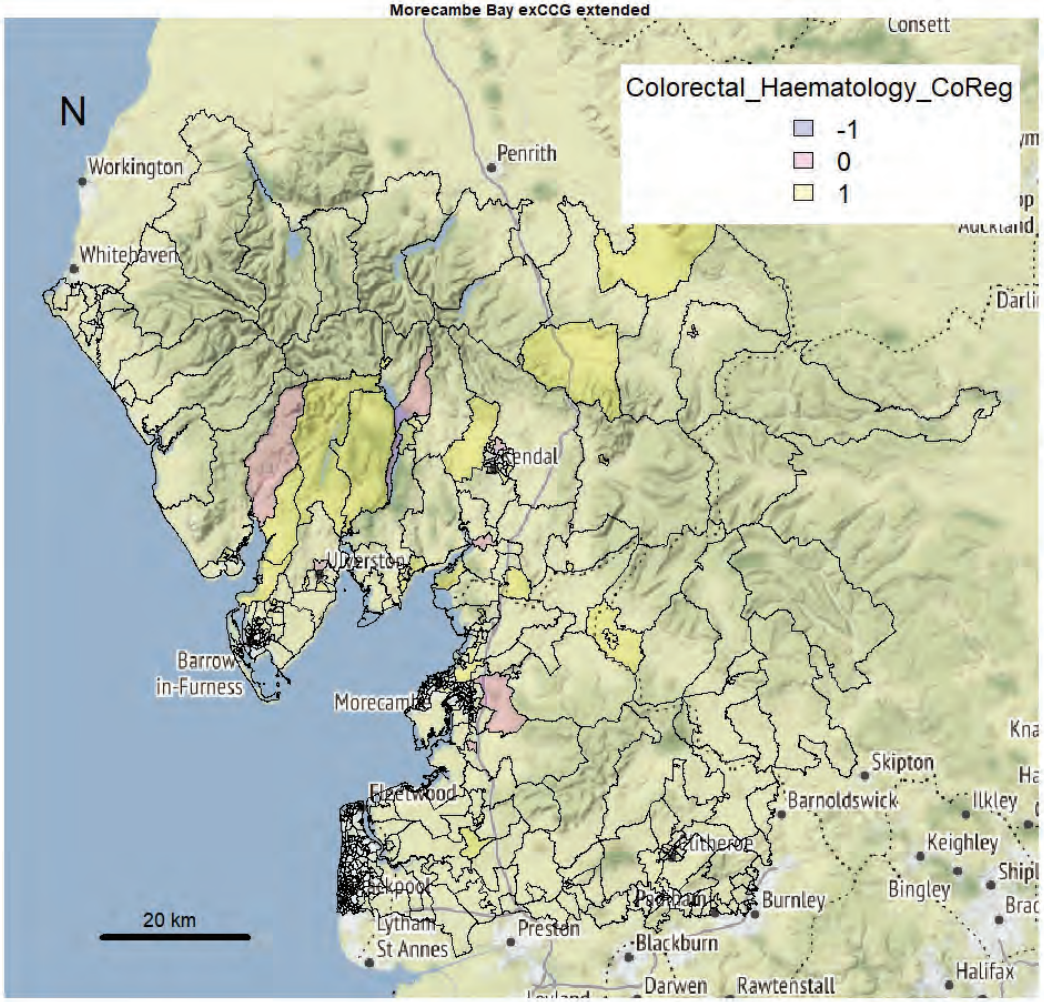
Morecambe Bay exCCG extended
Colorectal_Haematology_CoReg
-1
0
1
N
Workington
Penrith
Whitehaven
Kendal
Ulverston
Barrow-in-Furness
Morecambe
Skipton
Barnoldswick
Ilkley
Keighley
Fleetwood
Clitheroe
Blackpool
Padiham
Burnley
Bingley
Lytham St Annes
Preston
Blackburn
Darwen
Rawtenstall
Halifax
Consett
20 km

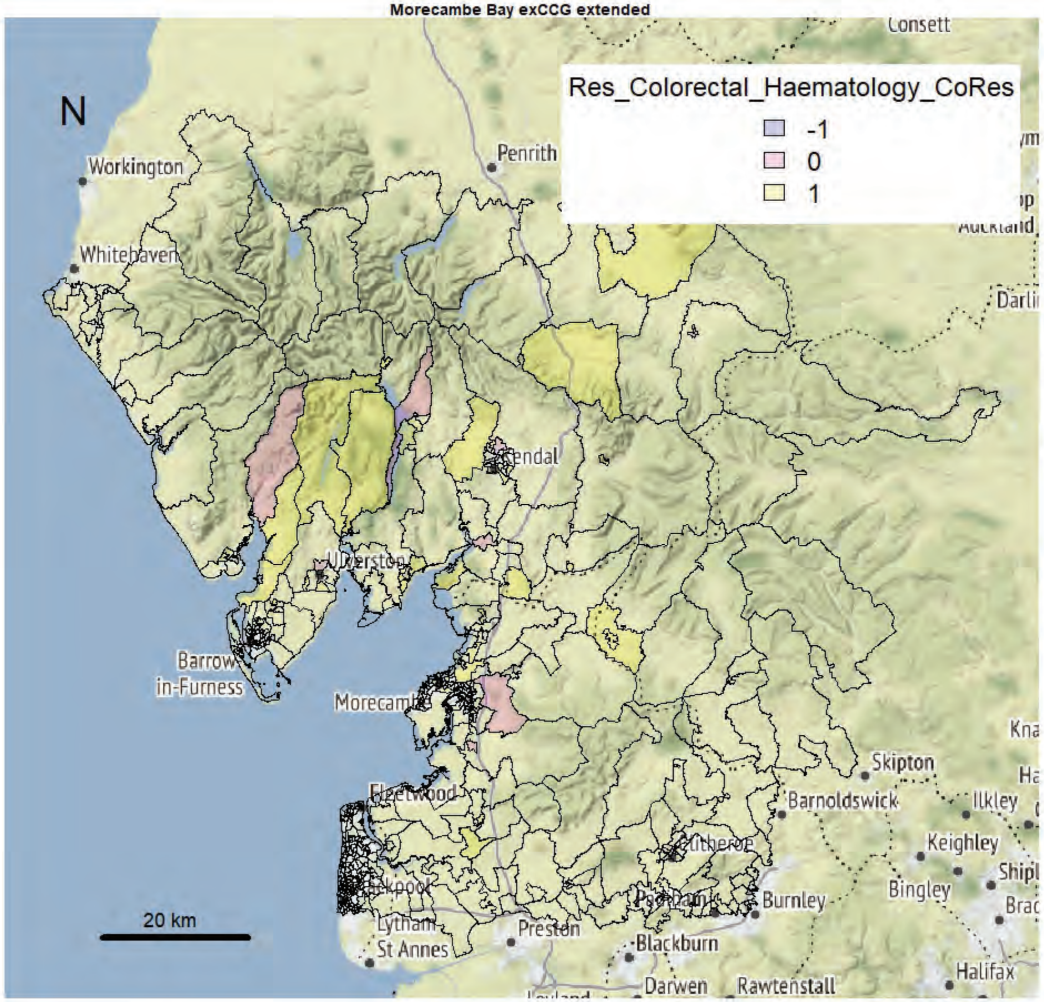
Morecambe Bay exCCG extended
Res_Colorectal_Haematology_CoRes
-1
0
1
N
Workington
Penrith
Whitehaven
Kendal
Ulverston
Barrow-in-Furness
Morecambe
Skipton
Barnoldswick
Ilkley
Keighley
Fleetwood
Clitheroe
Blackpool
Padiham
Burnley
Bingley
Lytham St Annes
Preston
Blackburn
Darwen
Rawtenstall
Halifax
Consett
20 km

Morecambe Bay exCCG extended

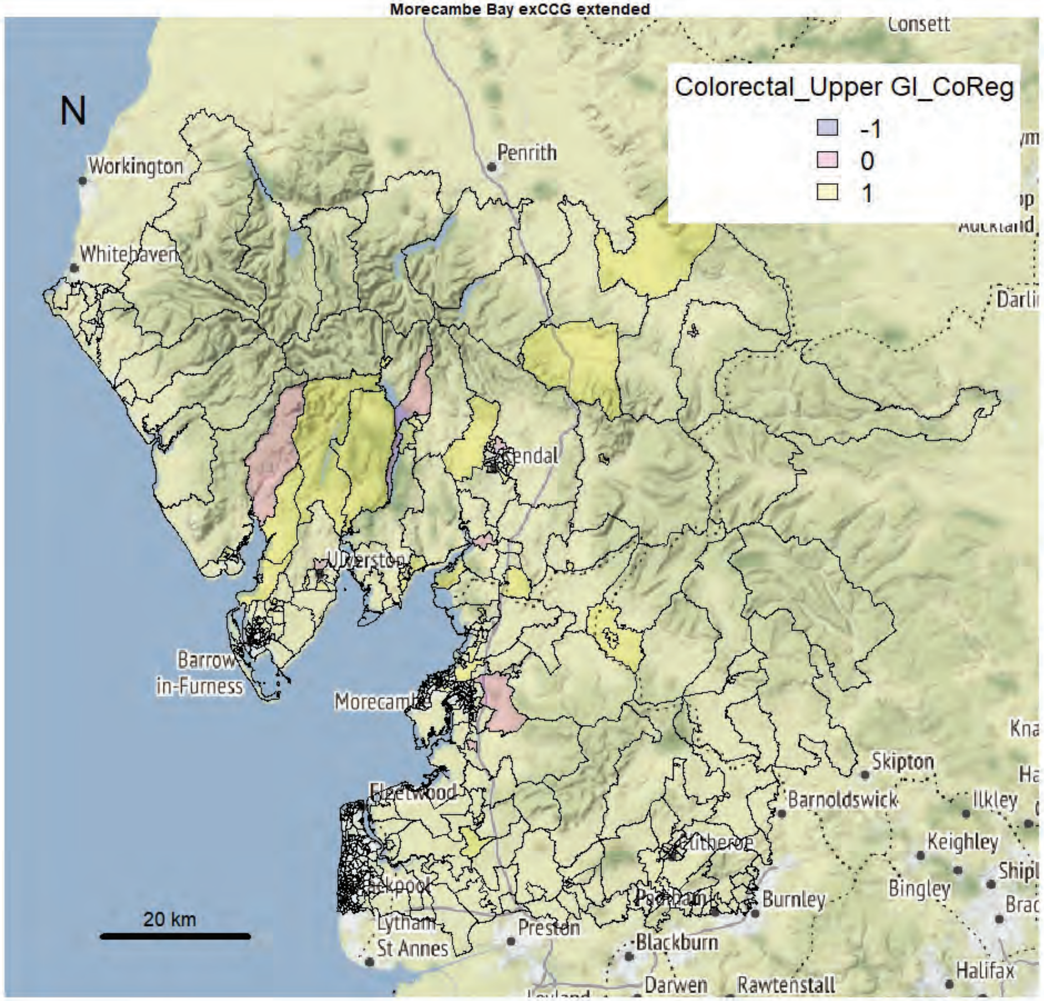

Morecambe Bay exCCG extended

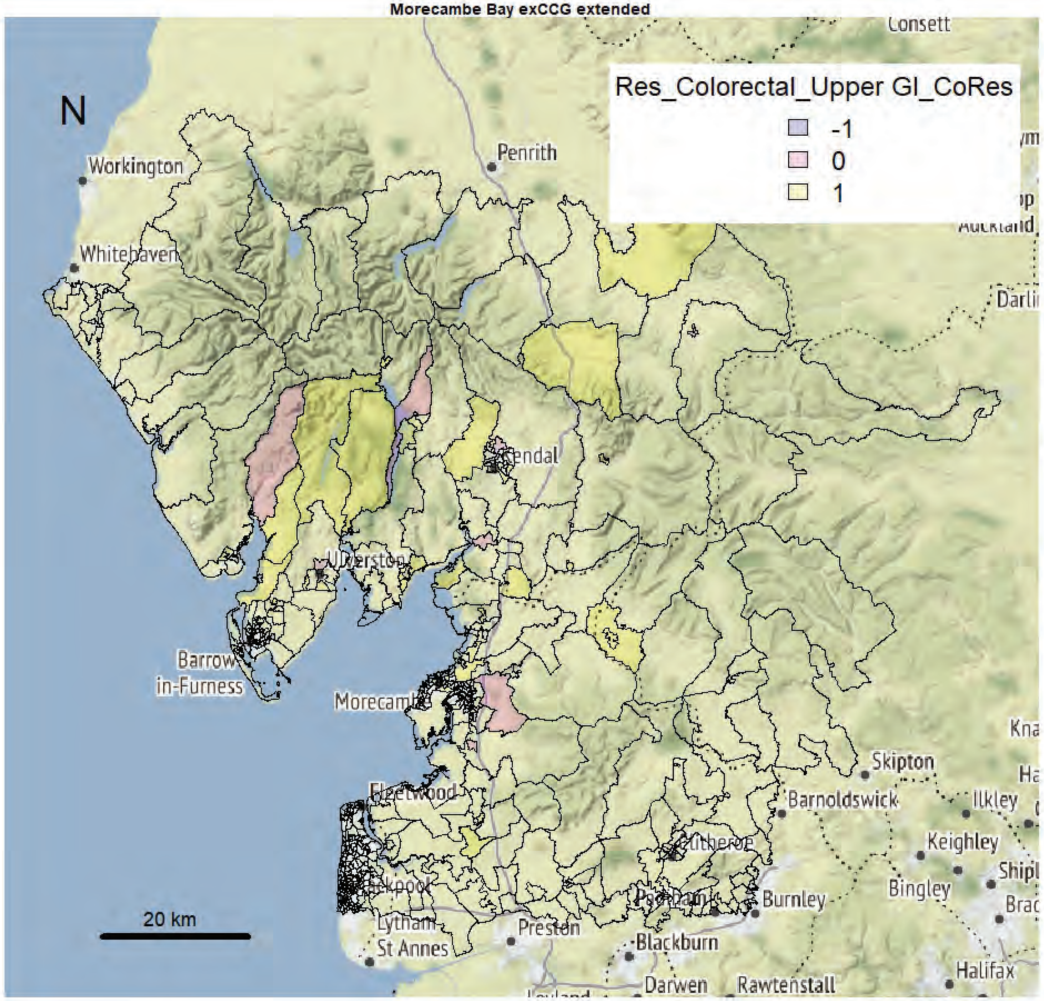

Morecambe Bay exCCG extended

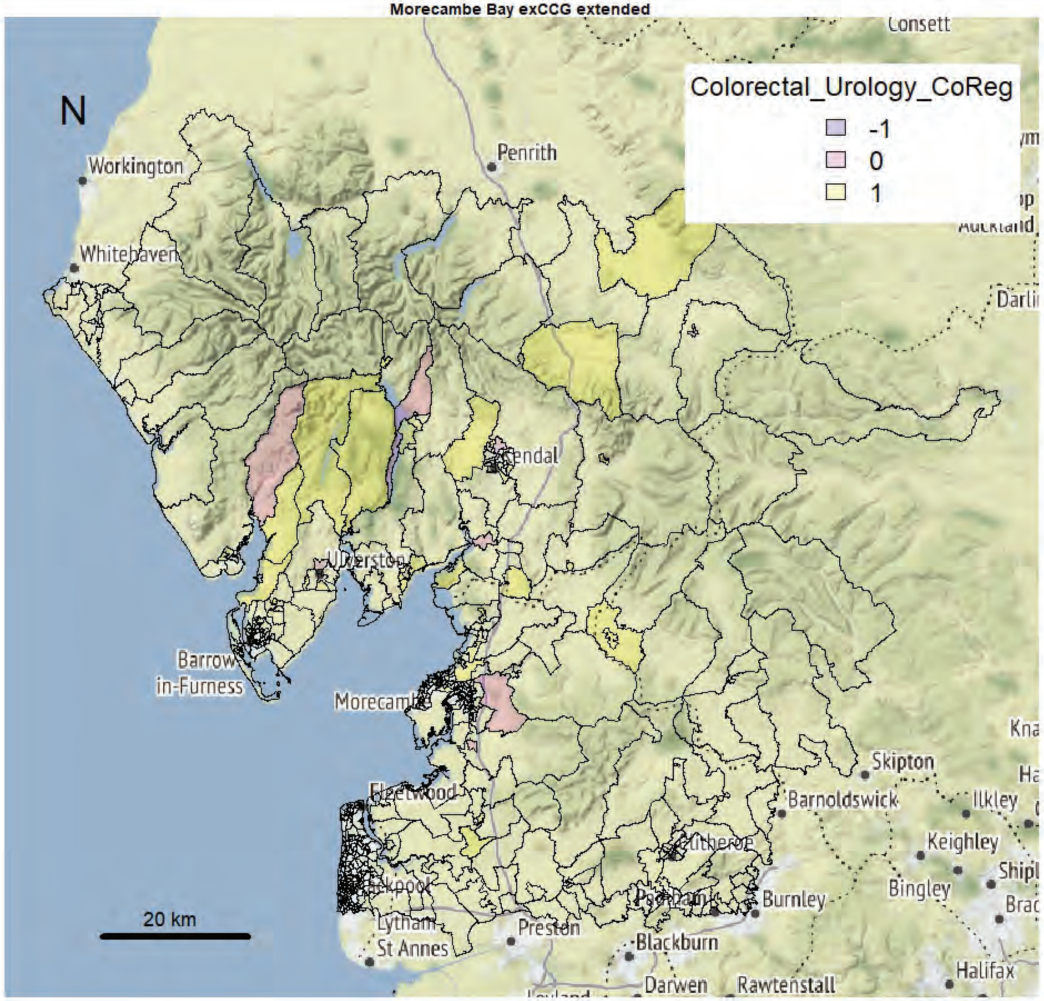

Morecambe Bay exCCG extended

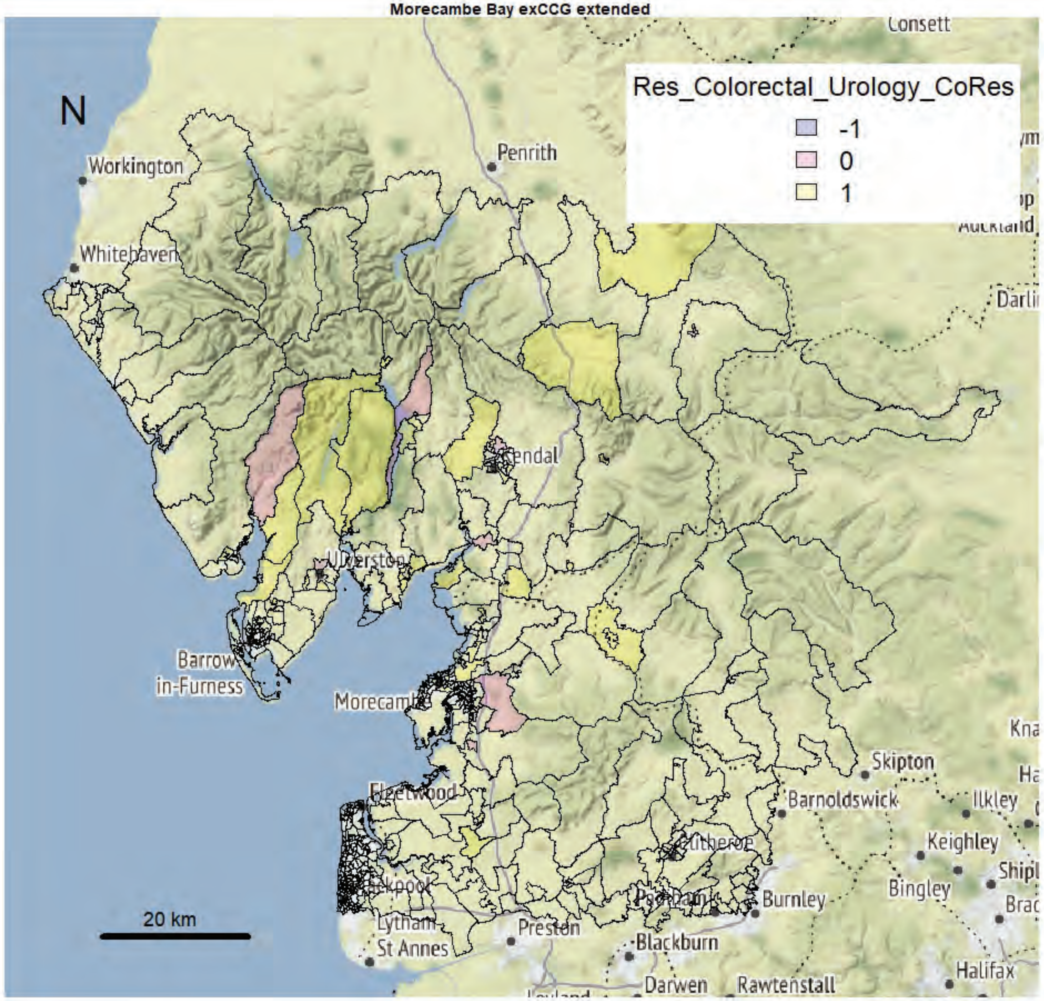

Morecambe Bay exCCG extended

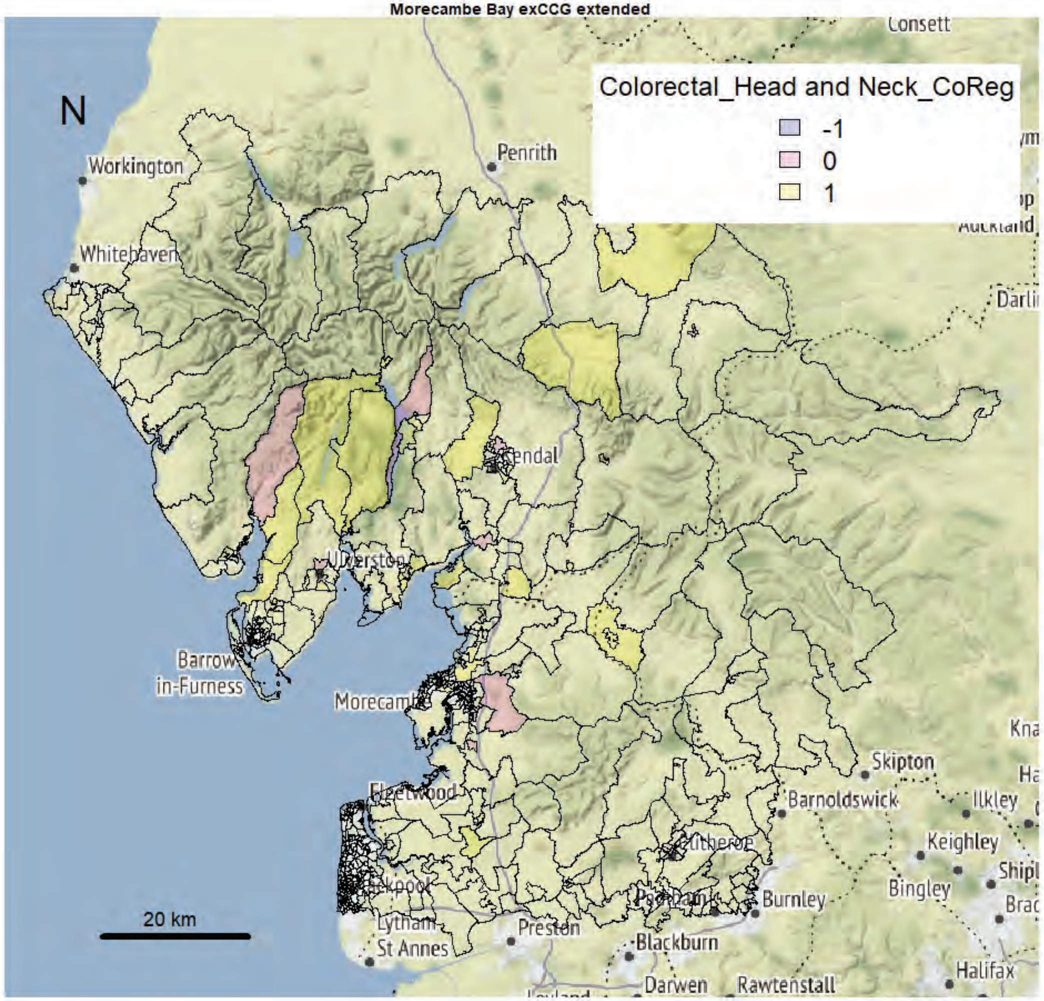

Morecambe Bay exCCG extended

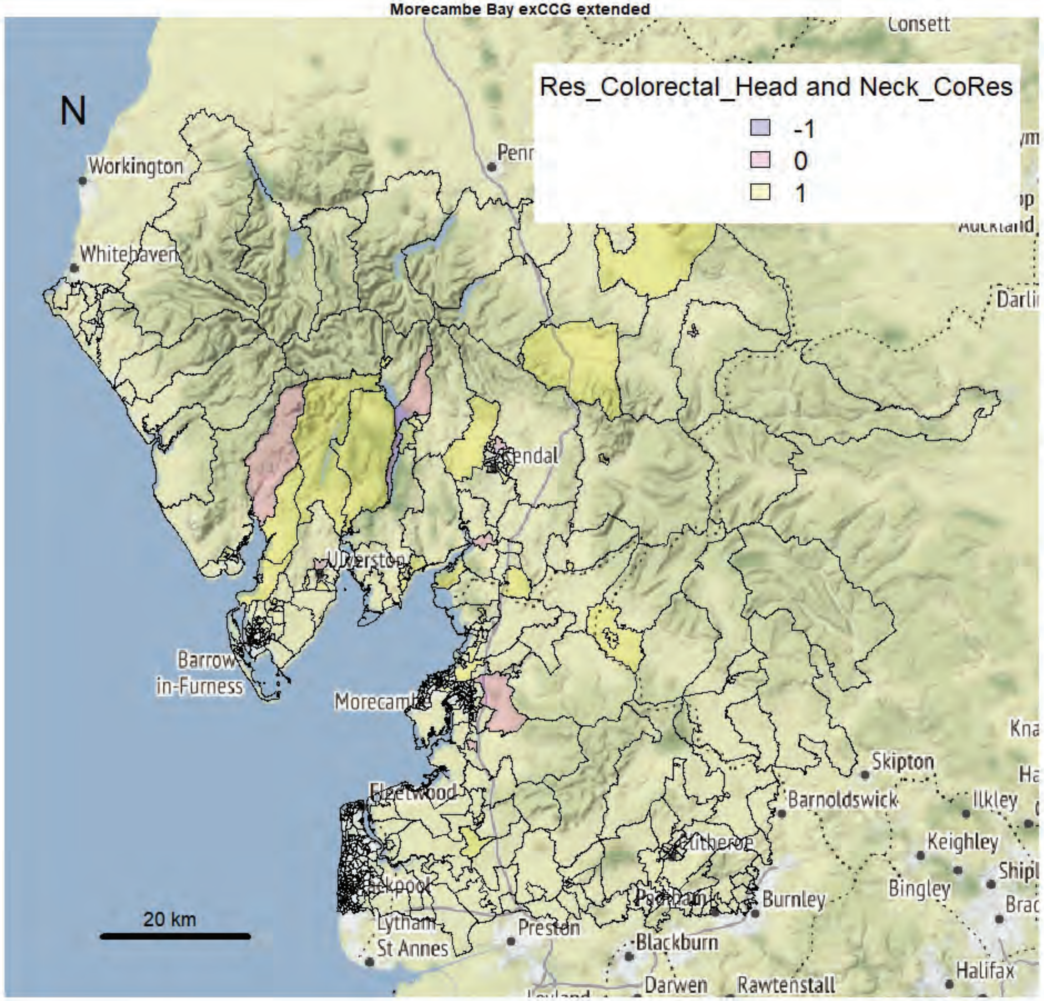

Morecambe Bay exCCG extended

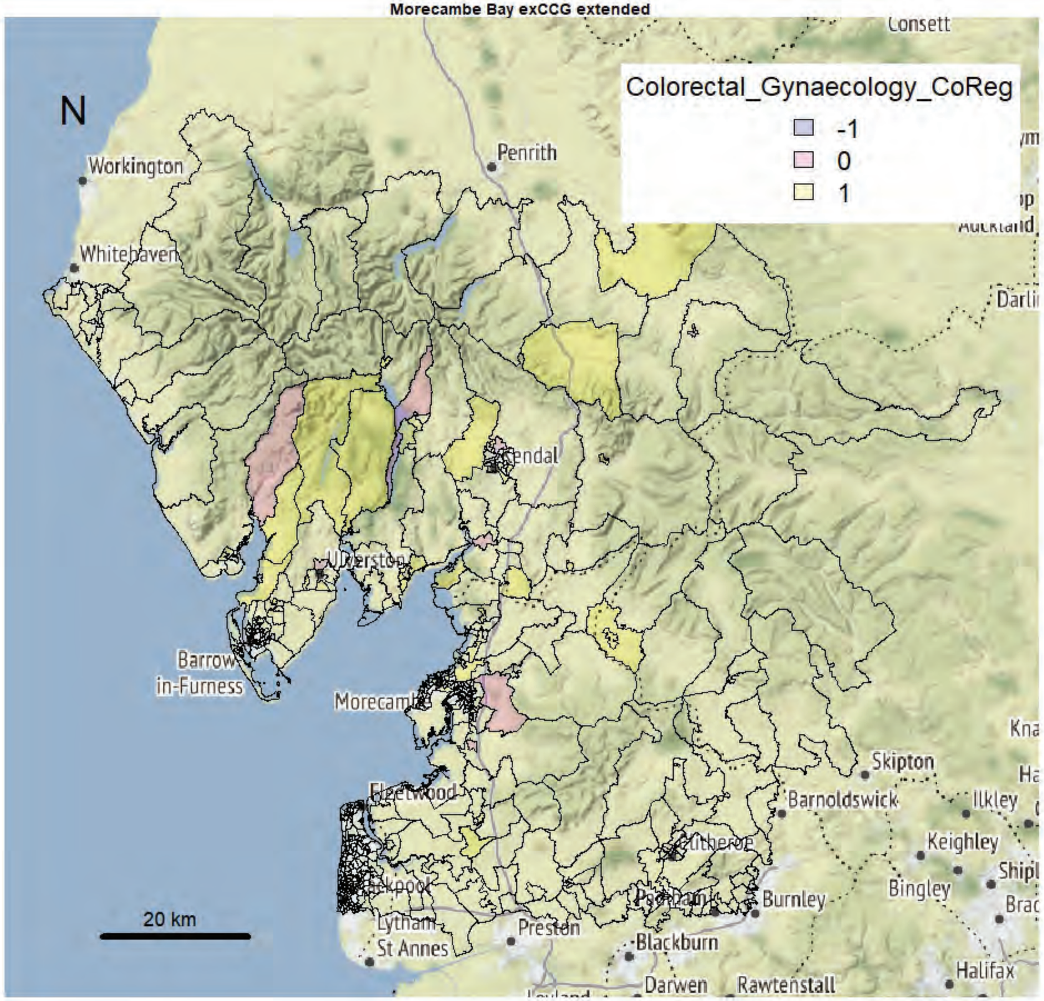

Morecambe Bay exCCG extended

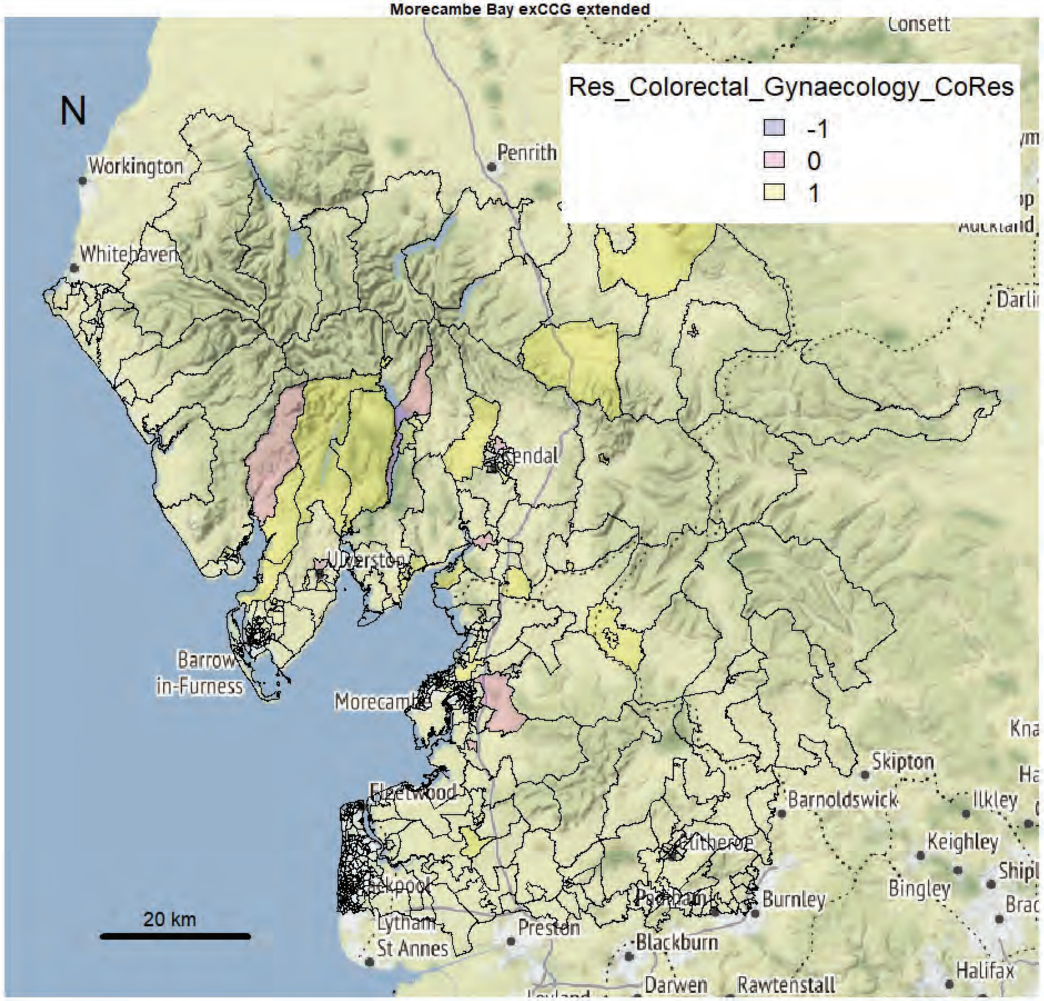

Morecambe Bay exCCG extended

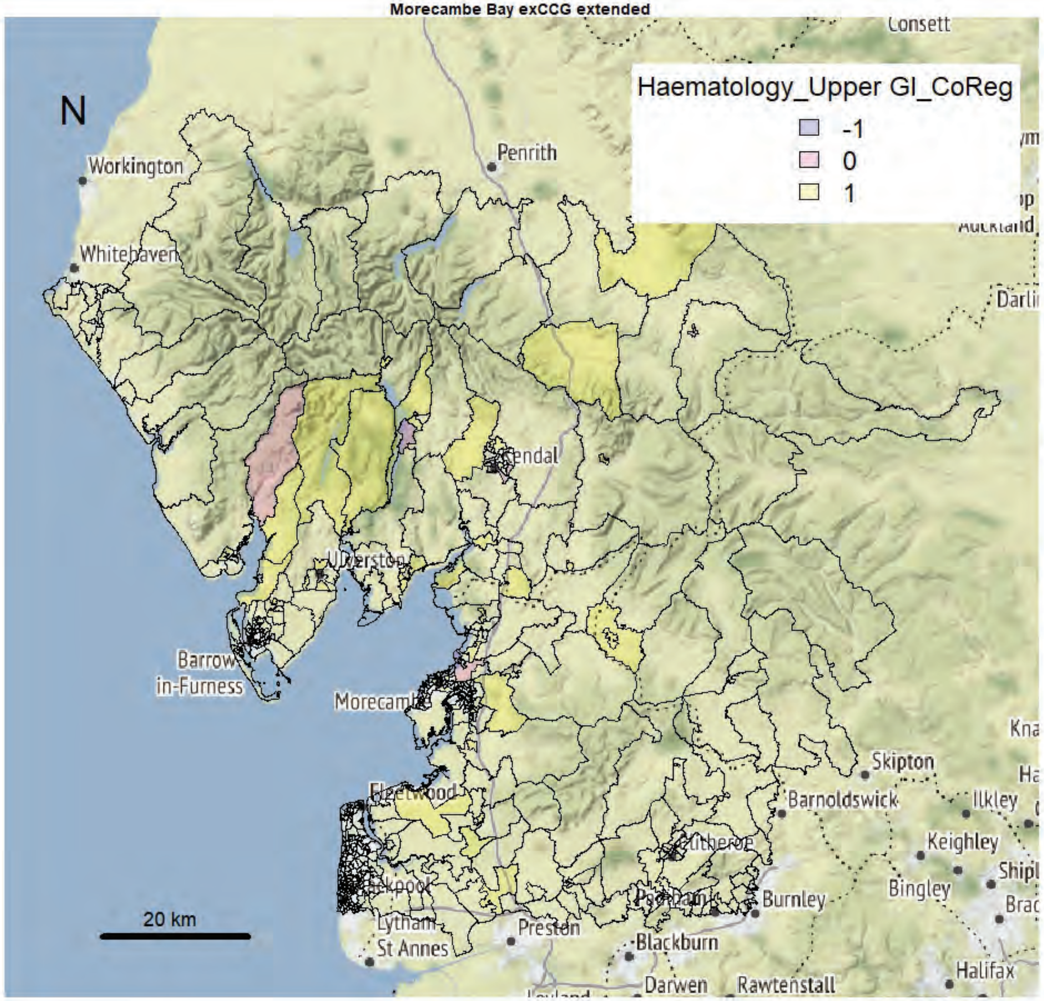

Morecambe Bay exCCG extended

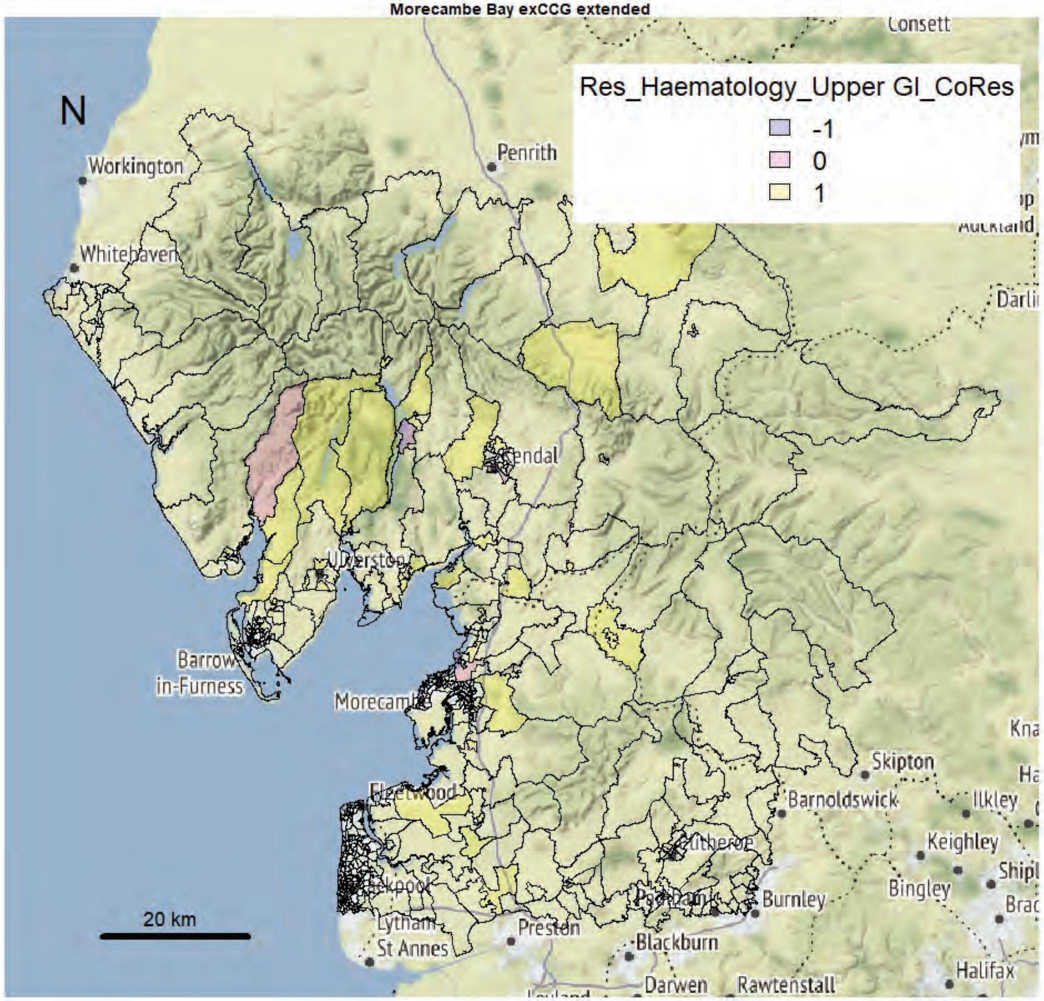

Morecambe Bay exCCG extended

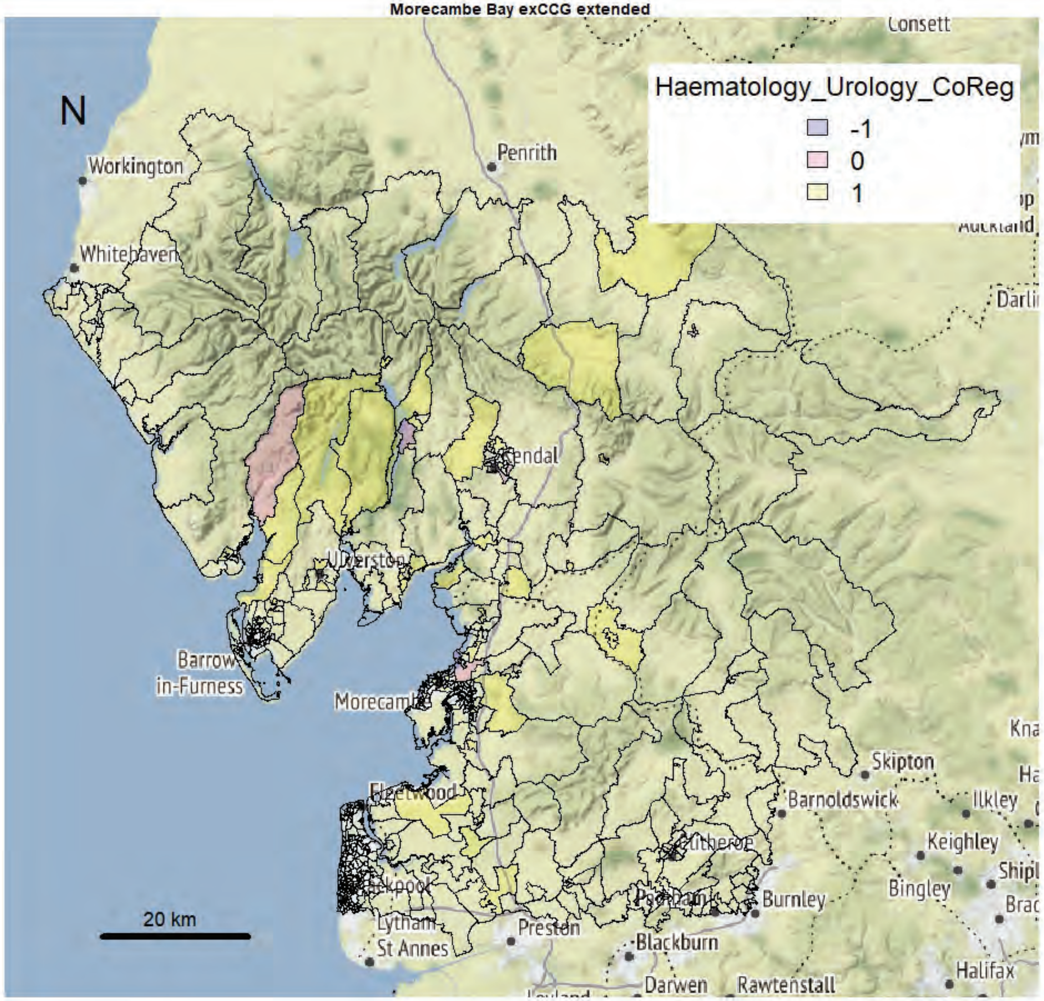

Morecambe Bay exCCG extended

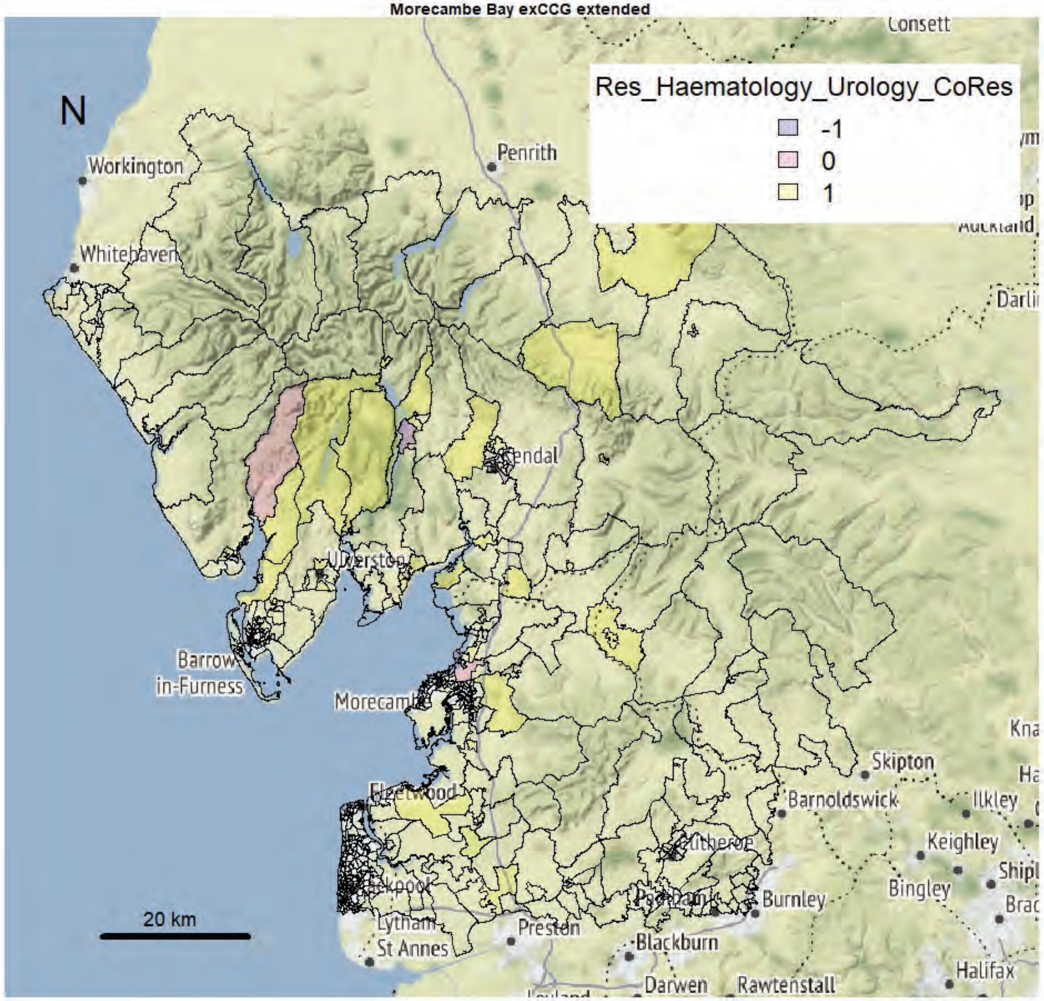

Morecambe Bay exCCG extended

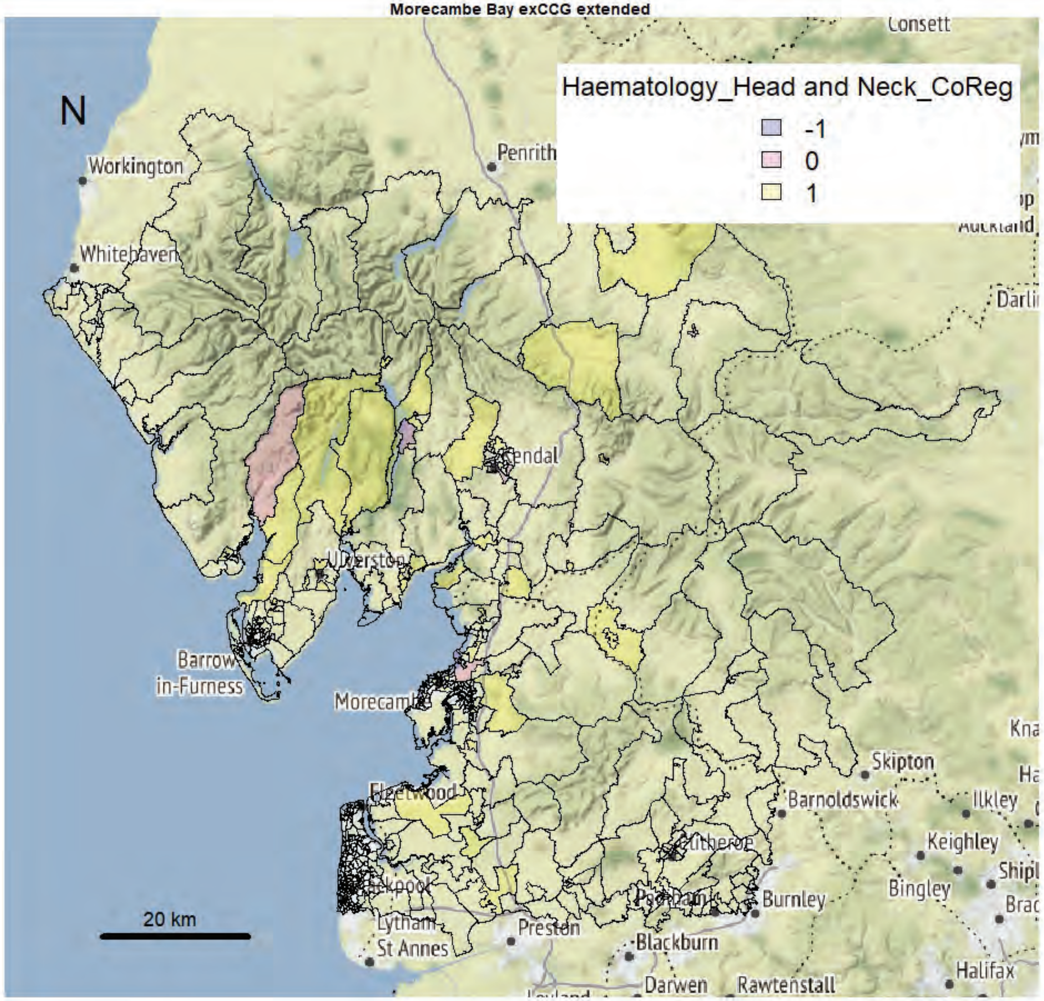

Morecambe Bay exCCG extended

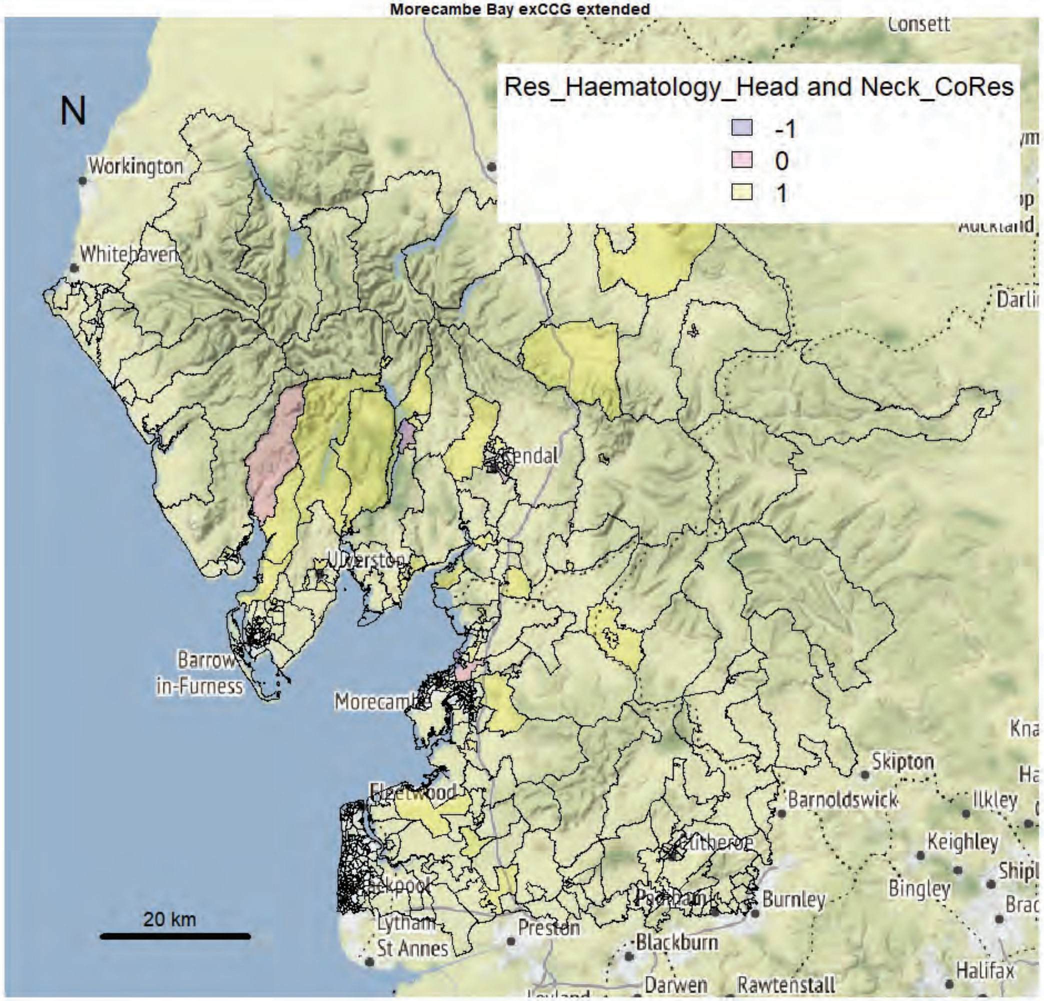

Morecambe Bay exCCG extended

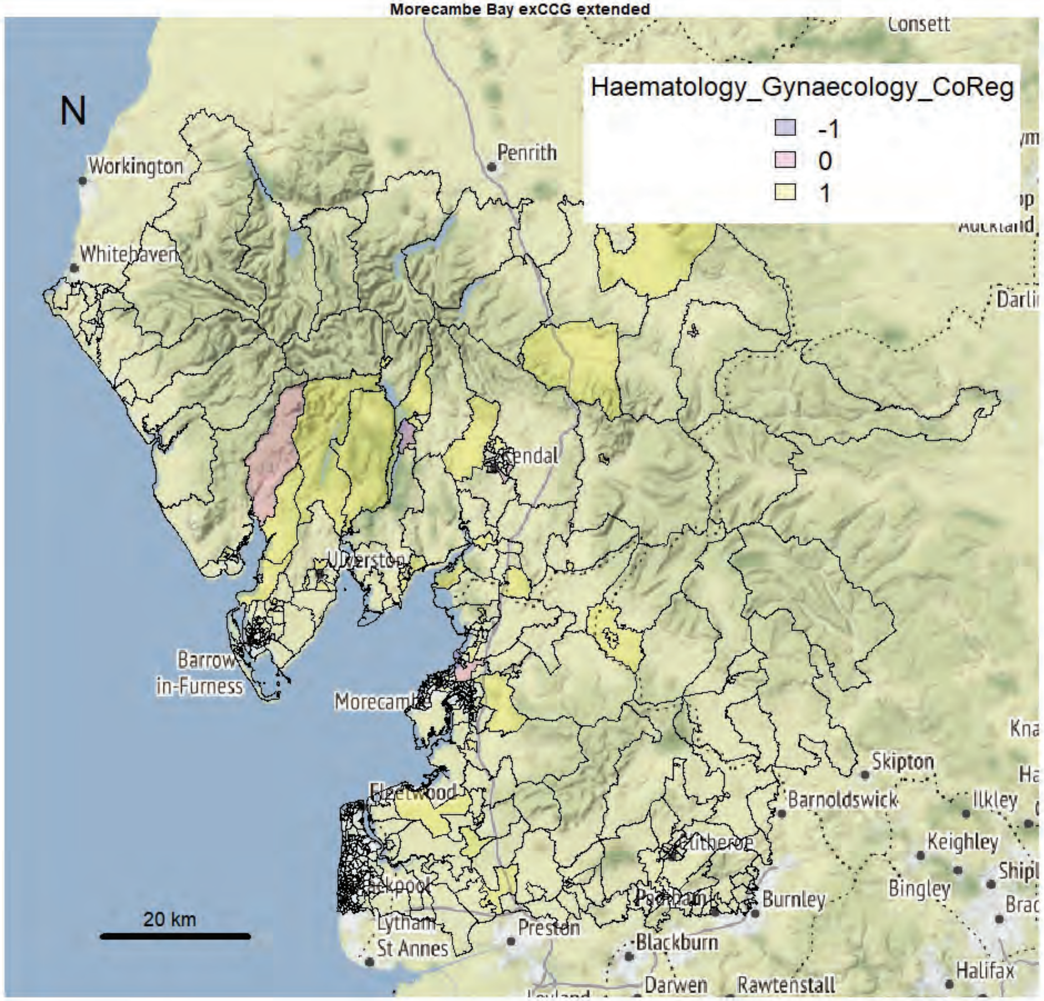

Morecambe Bay exCCG extended

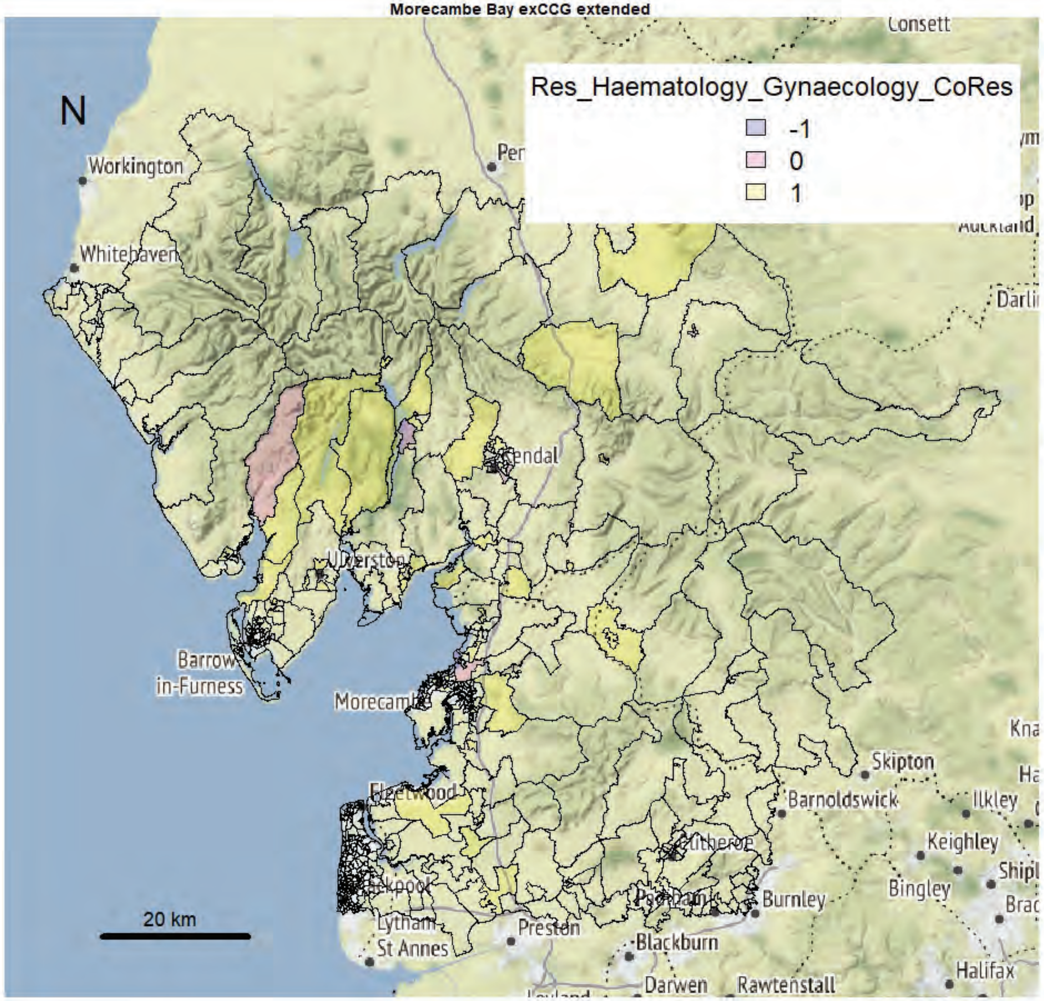

Morecambe Bay exCCG extended

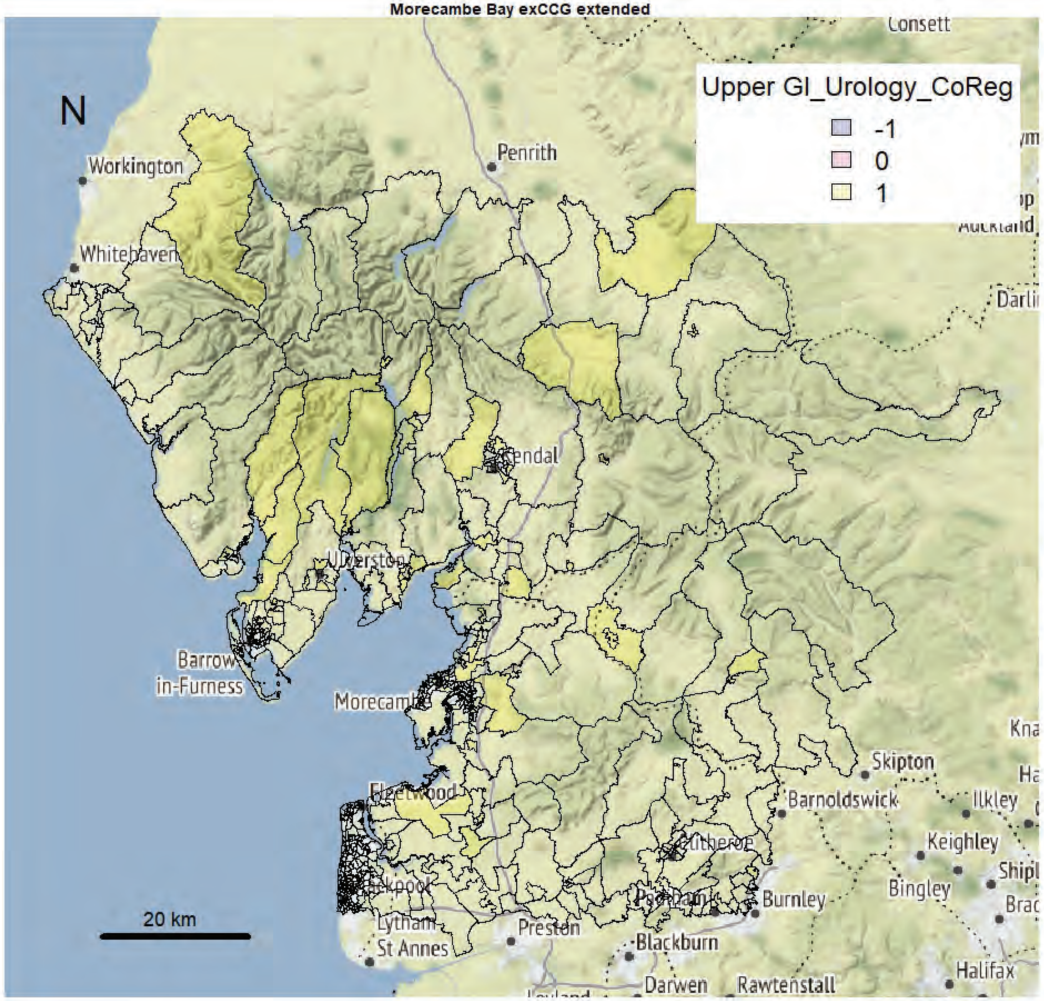

Morecambe Bay exCCG extended

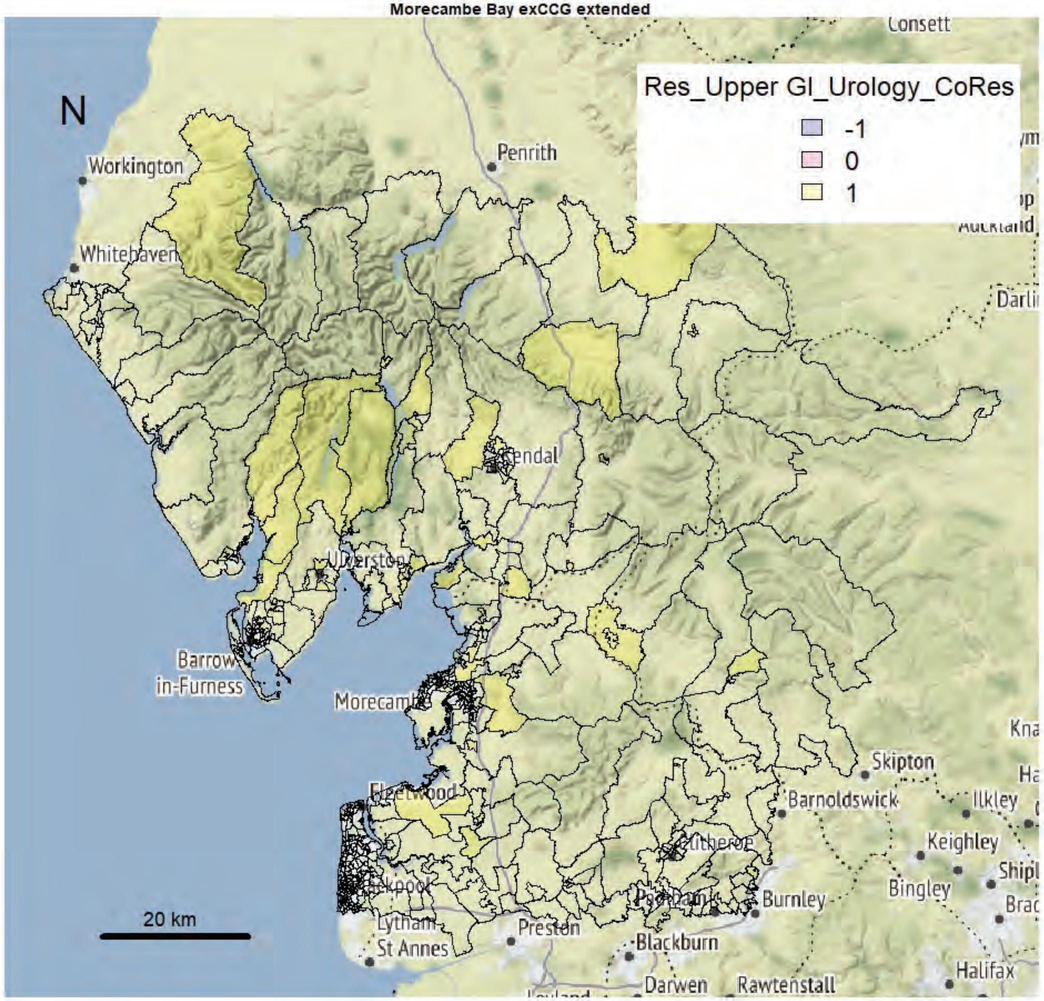

Morecambe Bay exCCG extended

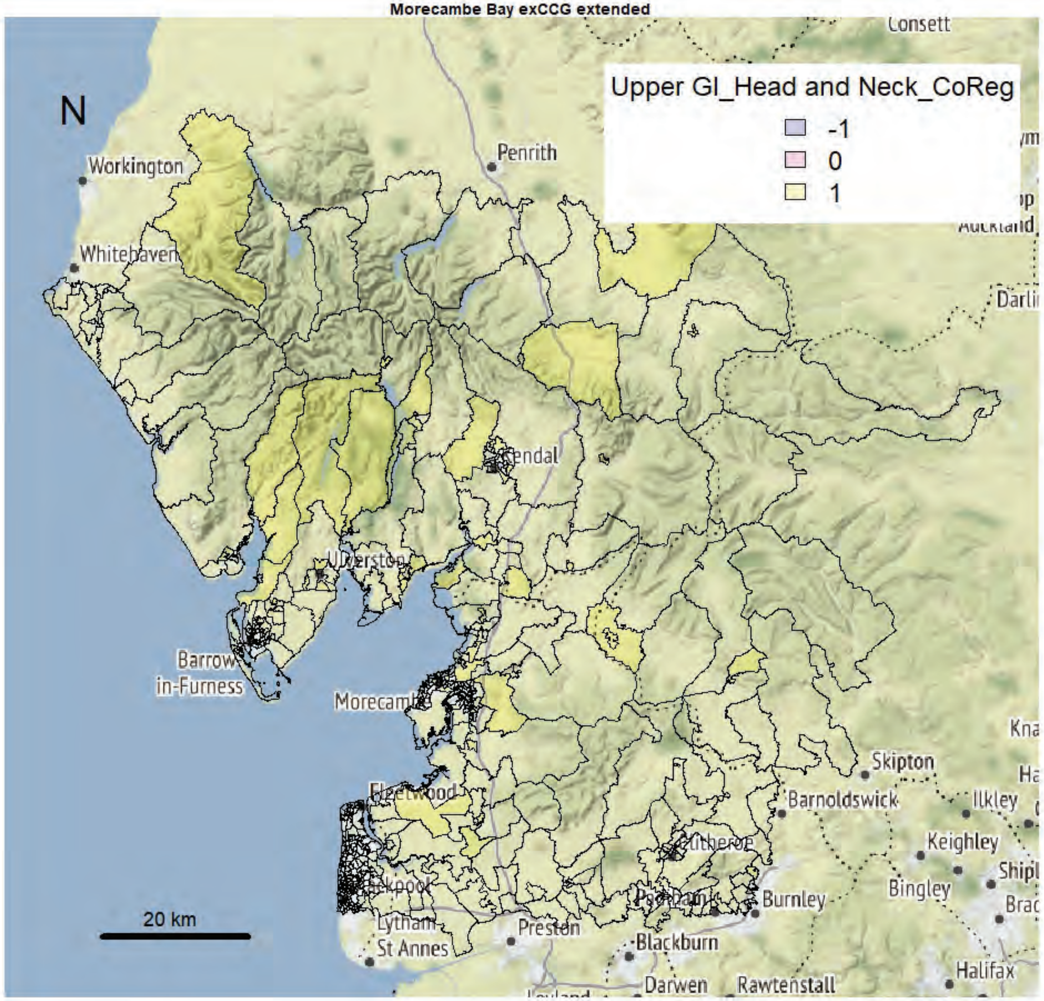

Morecambe Bay exCCG extended

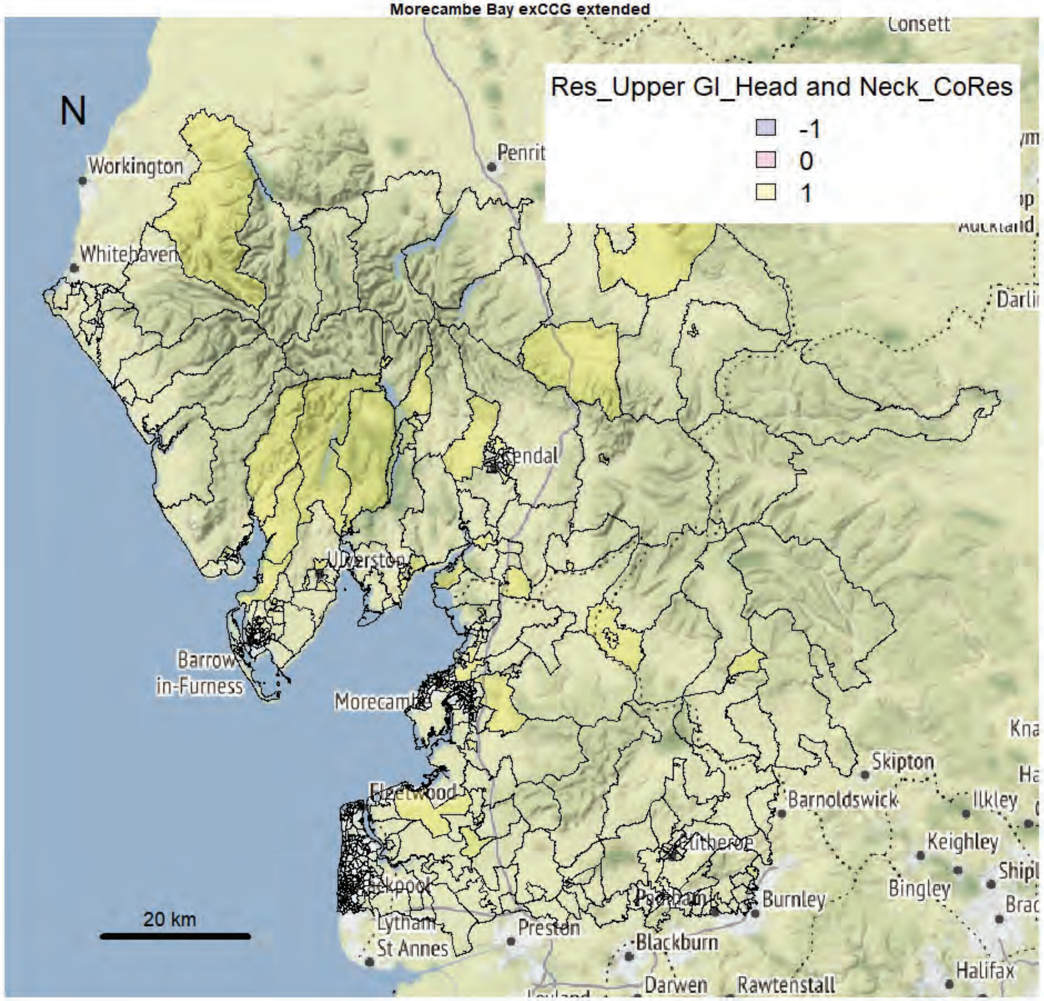

Morecambe Bay exCCG extended

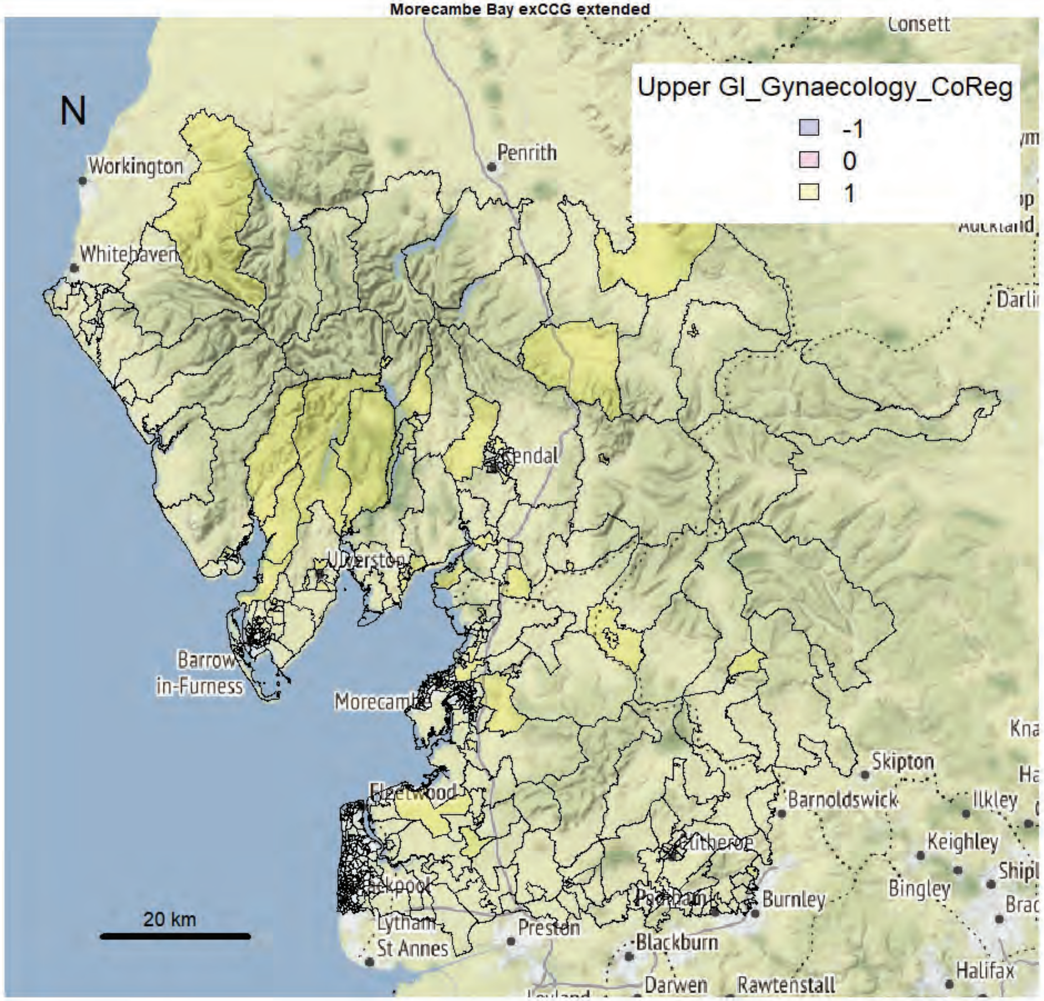

Morecambe Bay exCCG extended

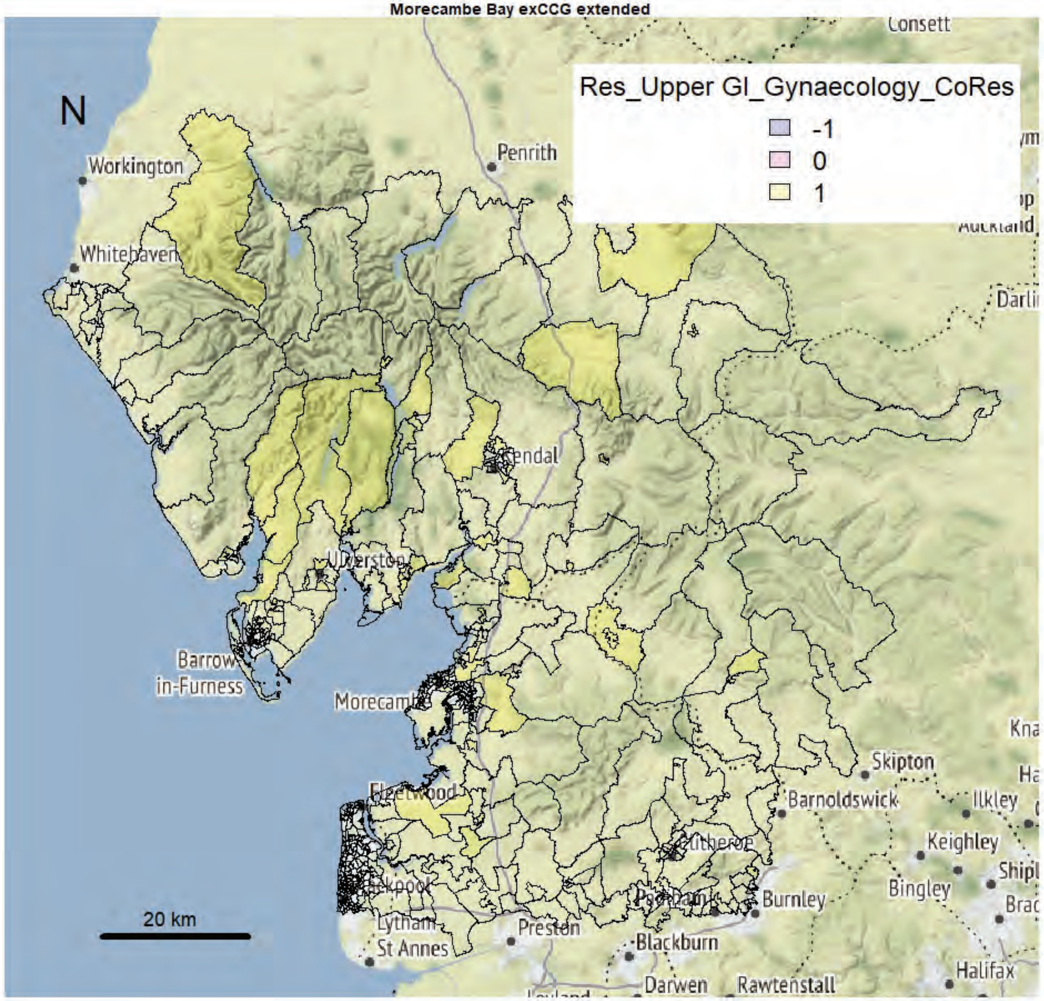

Morecambe Bay exCCG extended

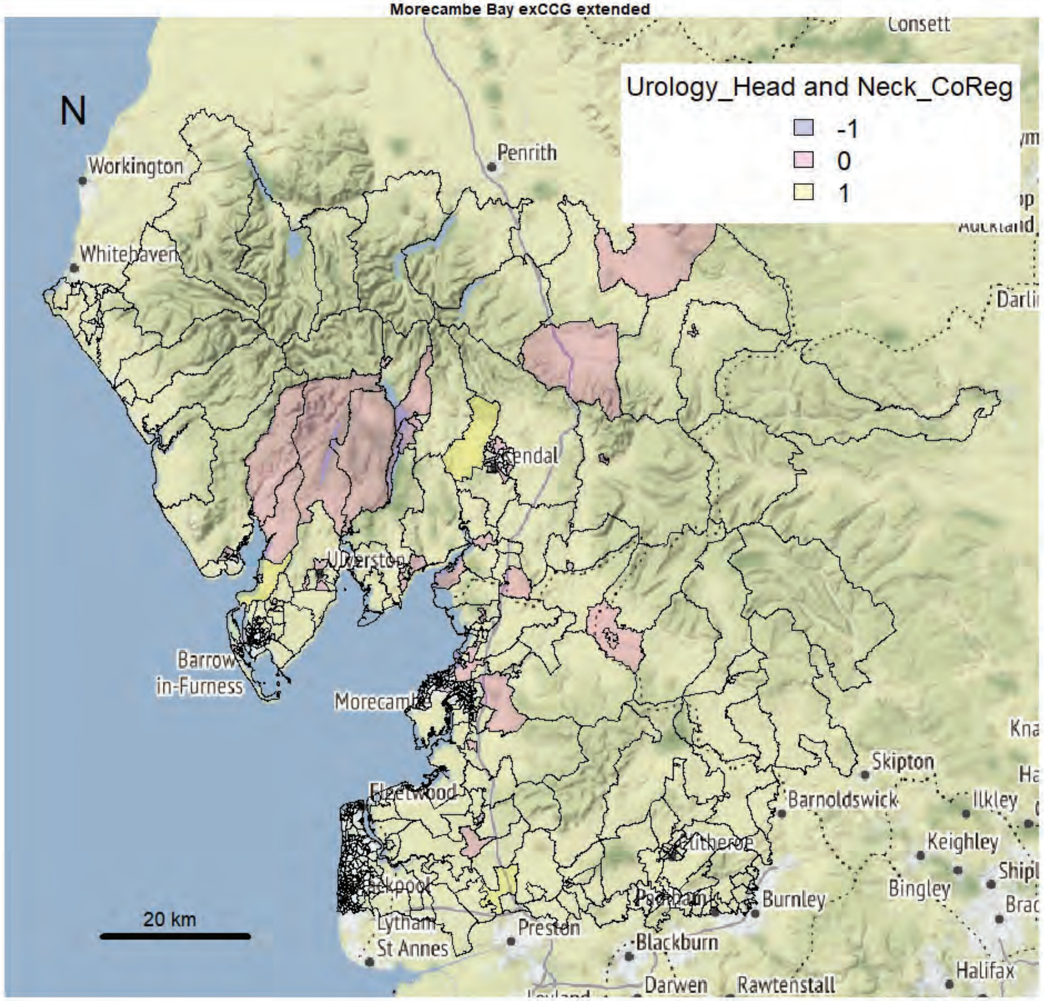

Morecambe Bay exCCG extended

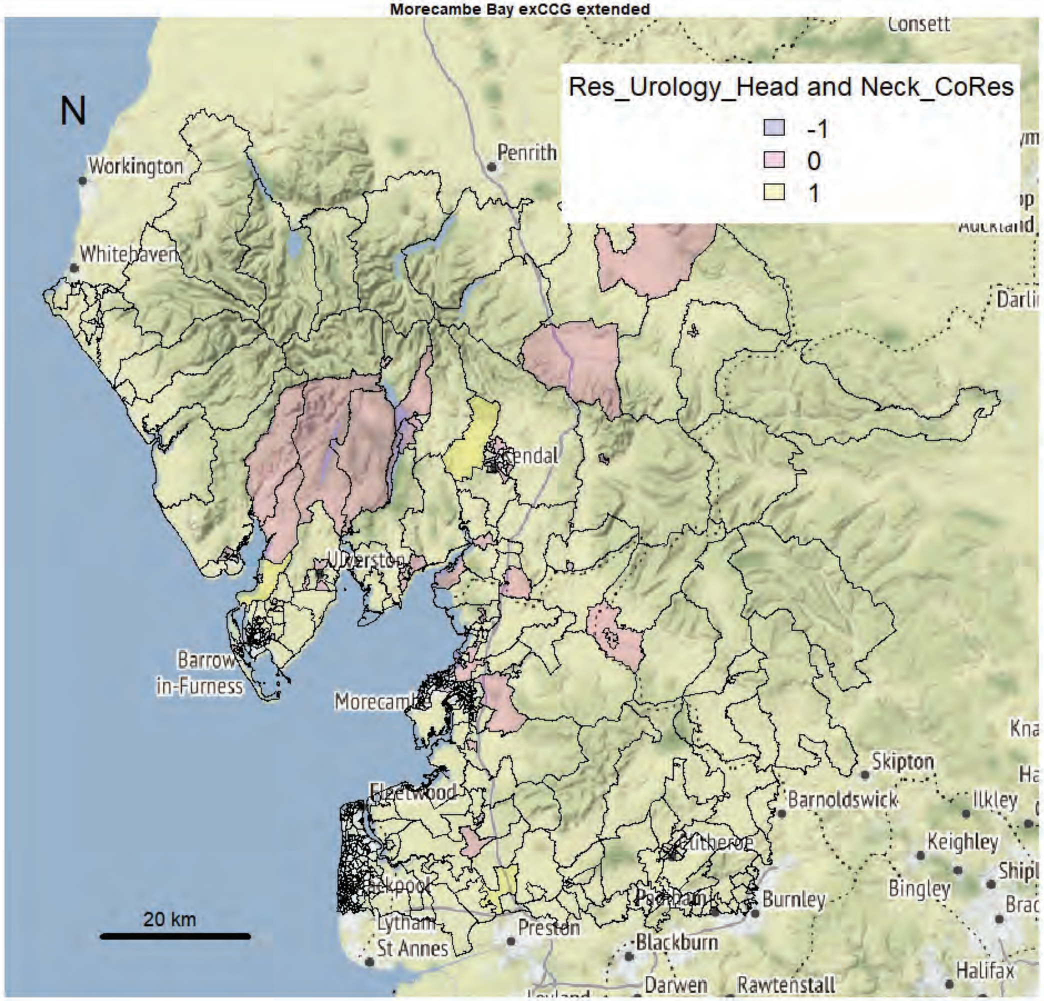

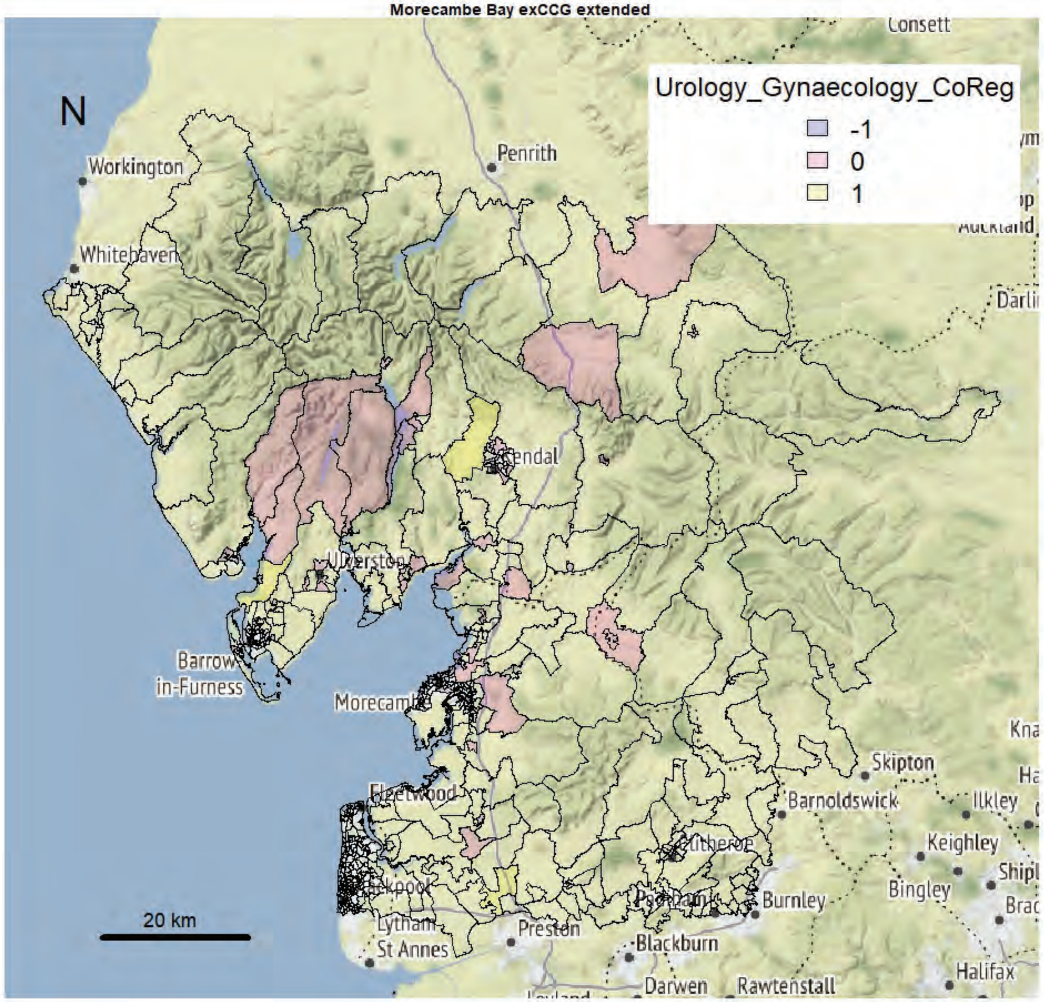
Morecambe Bay exCCG extended
Urology_Gynaecology_CoReg
-1
0
1
N
20 km
Workington
Whitehaven
Penrith
Kendal
Ulverston
Barrow-in-Furness
Morecambe
Fleetwood
Blackpool
Lytham St Annes
Preston
Blackburn
Darwen
Rawtenstall
Burnley
Clitheroe
Padiham
Skipton
Barnoldswick
Ilkley
Keighley
Bingley
Halifax
Consett

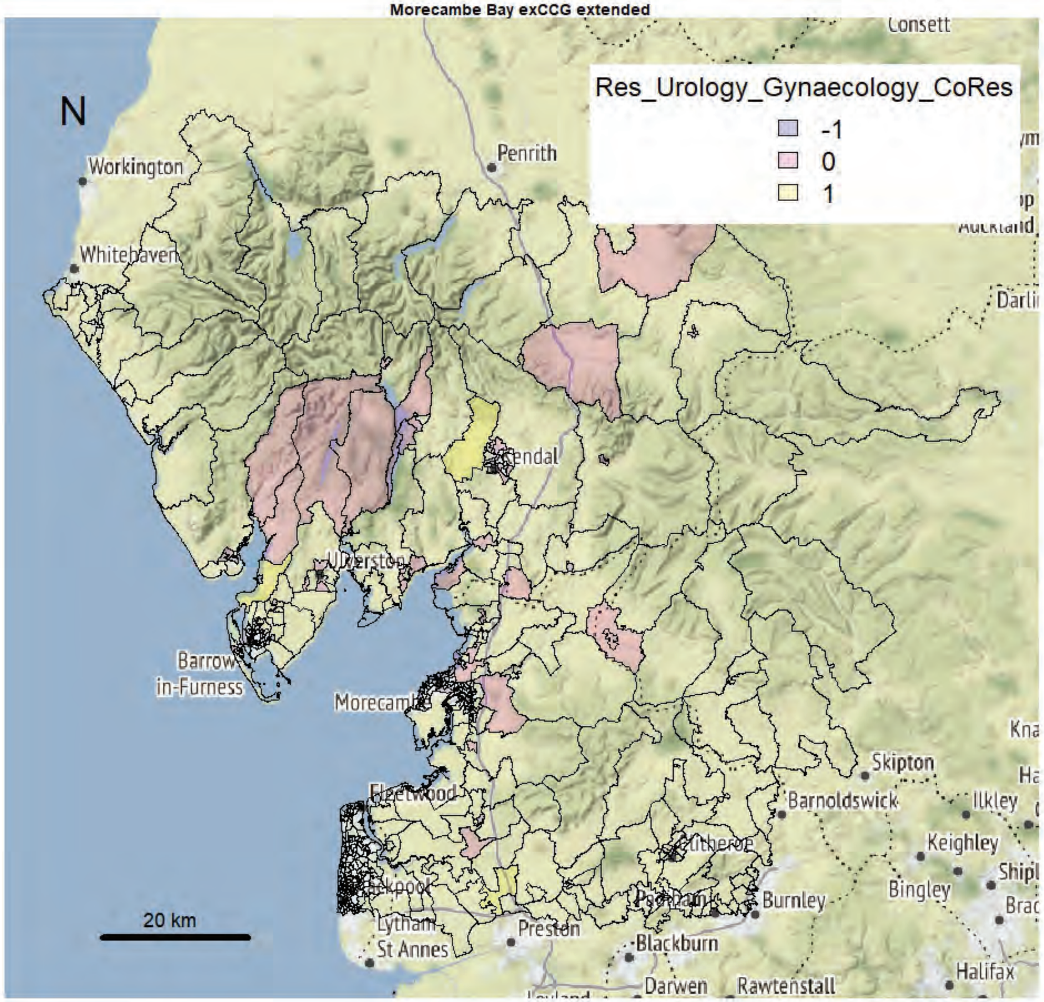
Morecambe Bay exCCG extended
Res_Urology_Gynaecology_CoRes
-1
0
1
N
20 km
Workington
Whitehaven
Penrith
Kendal
Ulverston
Barrow-in-Furness
Morecambe
Fleetwood
Blackpool
Lytham St Annes
Preston
Blackburn
Darwen
Rawtenstall
Burnley
Clitheroe
Padiham
Skipton
Barnoldswick
Ilkley
Keighley
Bingley
Halifax
Consett

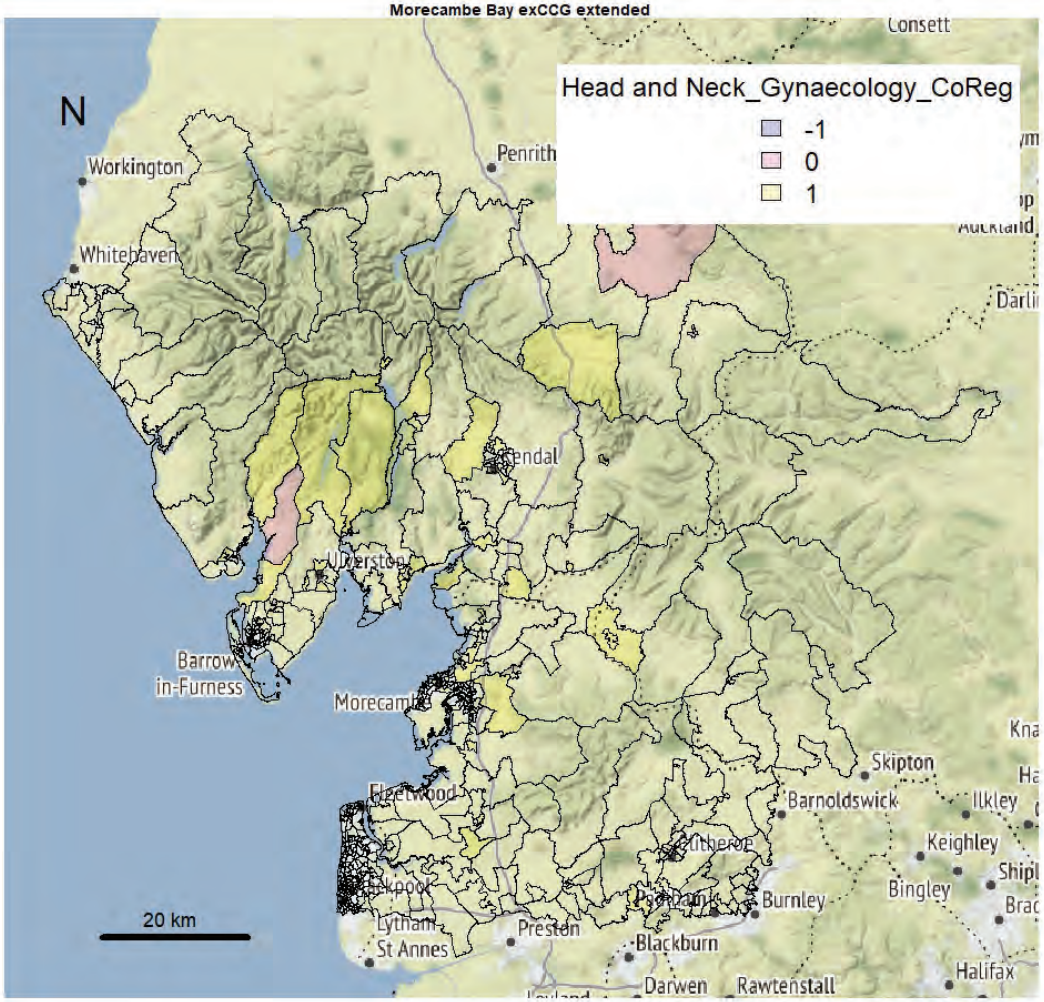
Morecambe Bay exCCG extended
N
Head and Neck_Gynaecology_CoReg
-1
0
1
20 km
Workington
Penrith
Whitehaven
Kendal
Ulverston
Barrow-in-Furness
Morecambe
Fleetwood
Blackpool
Lytham St Annes
Preston
Blackburn
Darwen
Rawtenstall
Clitheroe
Padiham
Burnley
Barnoldswick
Skipton
Ilkley
Keighley
Bingley
Halifax
Consett

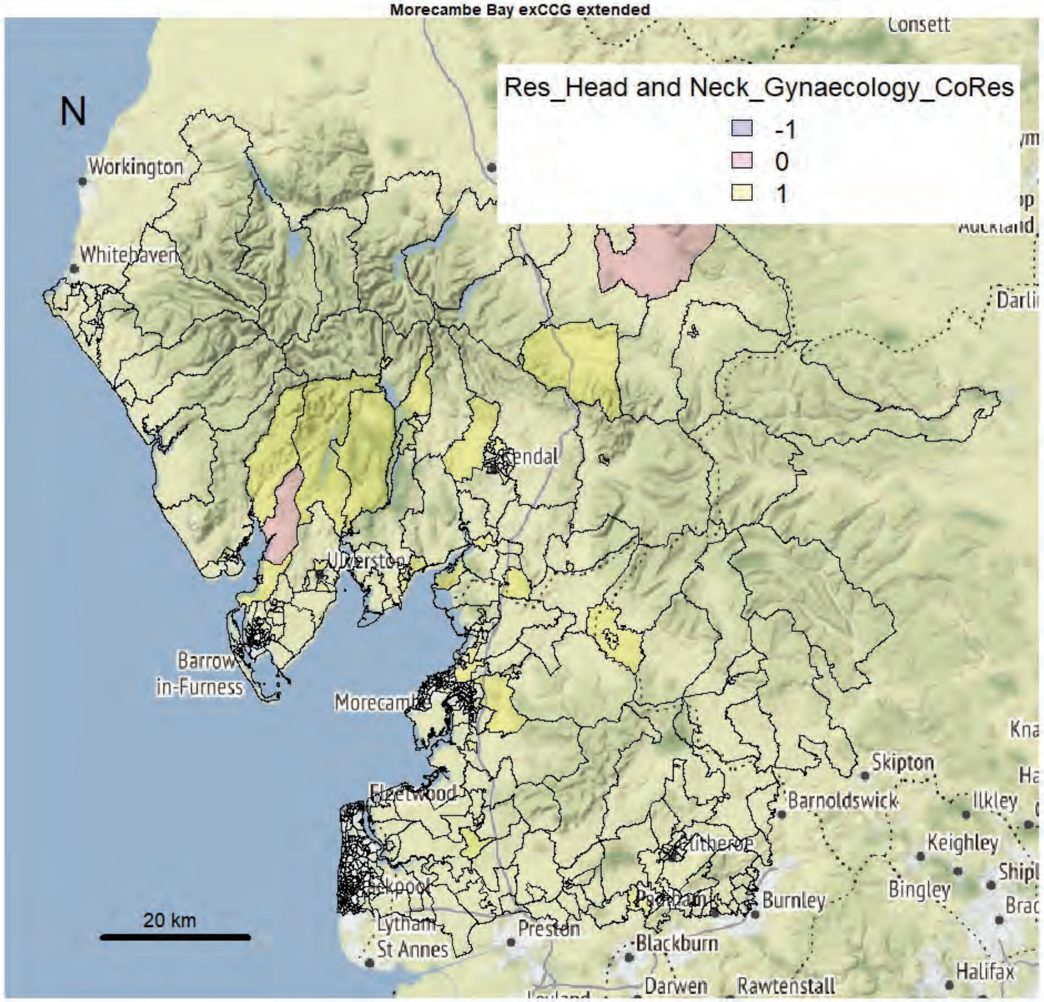
Morecambe Bay exCCG extended
N
Res_Head and Neck_Gynaecology_CoRes
-1
0
1
20 km
Workington
Whitehaven
Kendal
Ulverston
Barrow-in-Furness
Morecambe
Fleetwood
Blackpool
Lytham St Annes
Preston
Blackburn
Darwen
Rawtenstall
Clitheroe
Padiham
Burnley
Barnoldswick
Skipton
Ilkley
Keighley
Bingley
Halifax
Consett

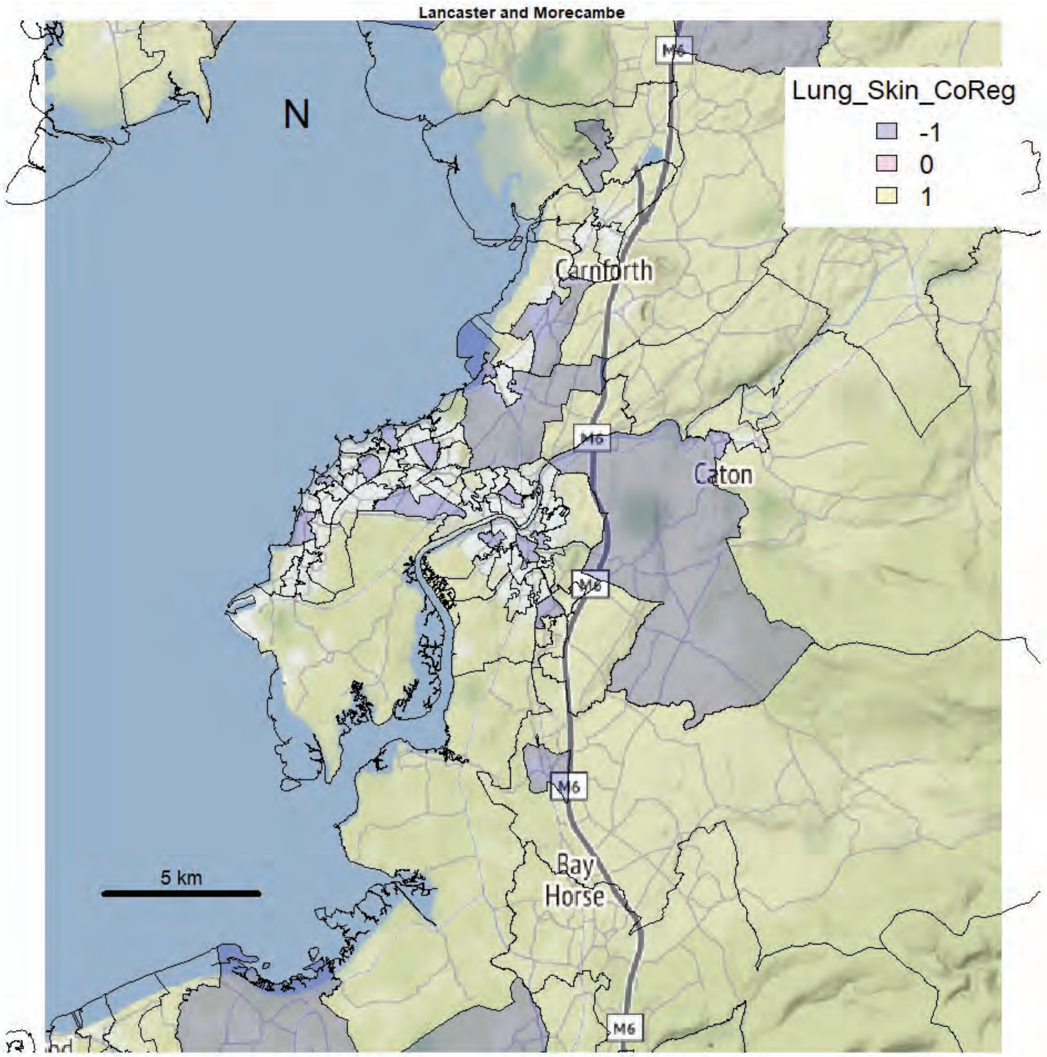
Lancaster and Morecambe
N
Lung_Skin_CoReg
-1
0
1
Carnforth
Caton
Bay
Horse
M6
5 km

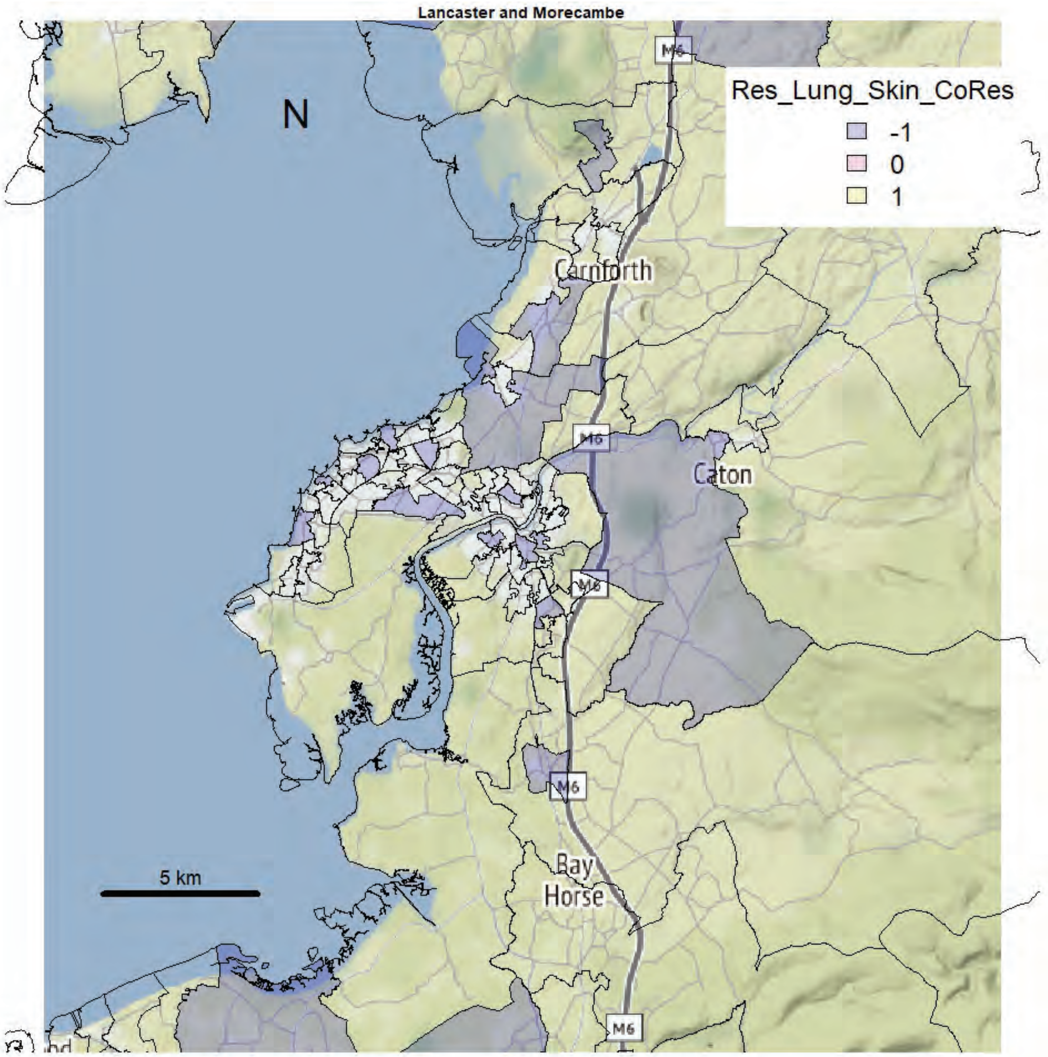
Lancaster and Morecambe
N
Res_Lung_Skin_CoRes
-1
0
1
Carnforth
Caton
Bay
Horse
M6
5 km

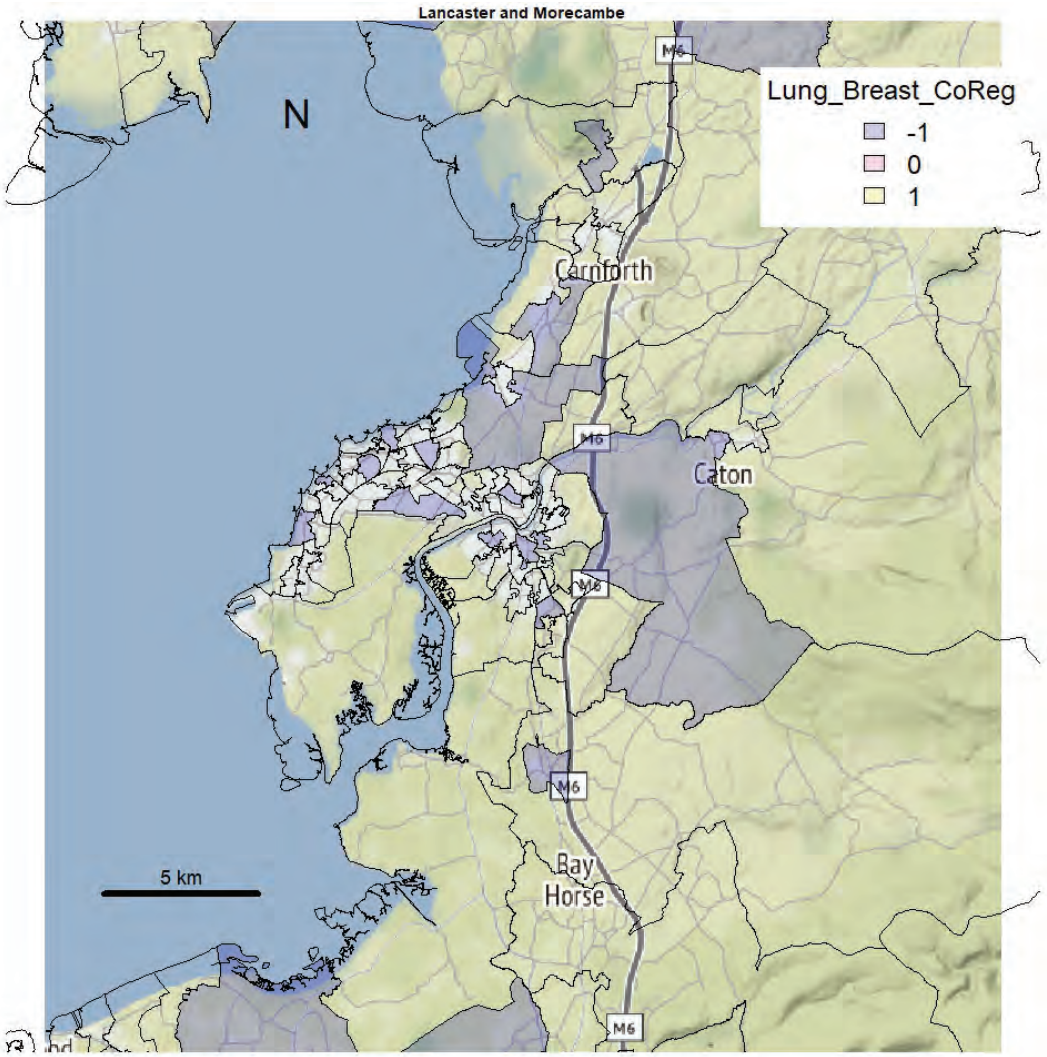
Lancaster and Morecambe
N
Lung_Breast_CoReg
-1
0
1
Carnforth
Caton
Bay Horse
M6
5 km

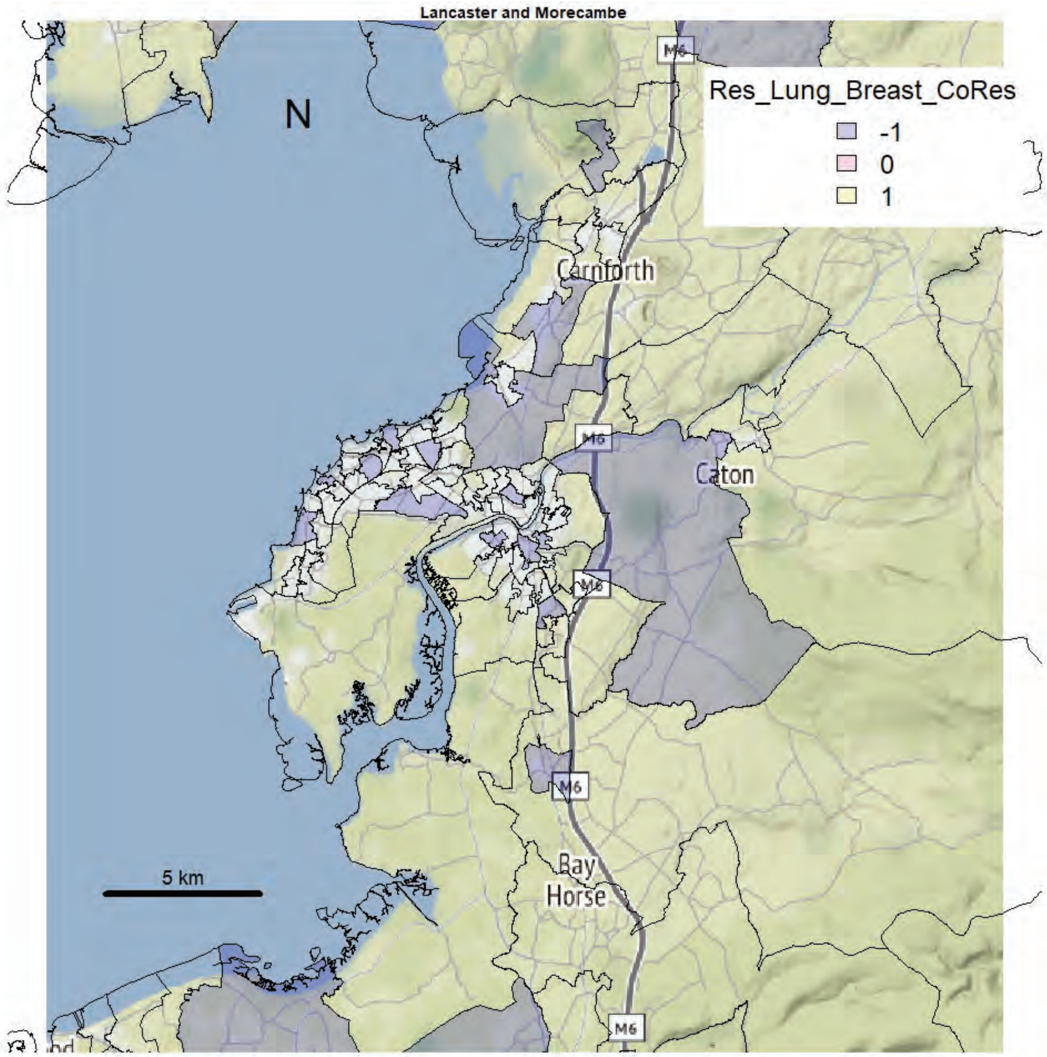
Lancaster and Morecambe
N
Res_Lung_Breast_CoRes
-1
0
1
Carnforth
Caton
Bay Horse
M6
5 km

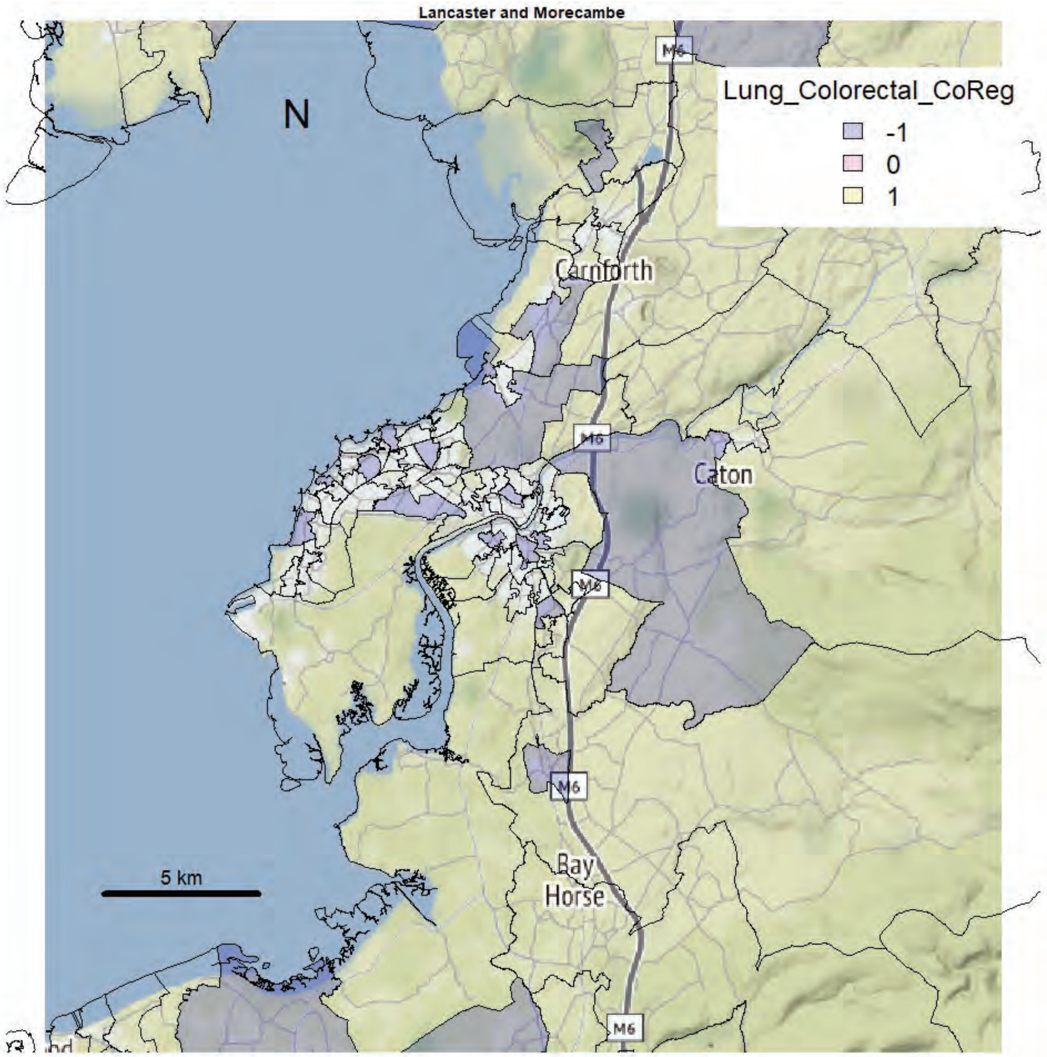
Lancaster and Morecambe
Lung_Colorectal_CoReg
-1
0
1
N
Carnforth
Caton
Bay
Horse
M6
5 km

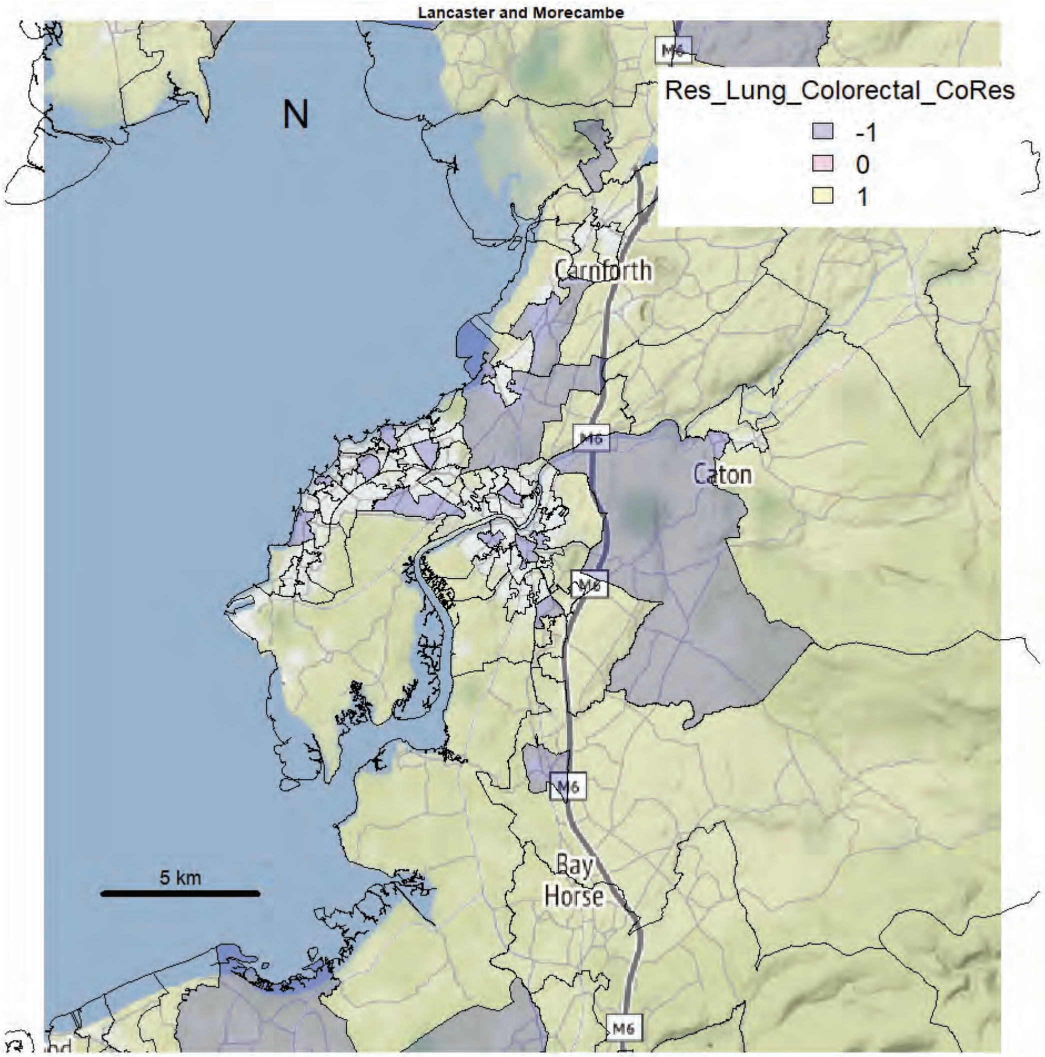
Lancaster and Morecambe
Res_Lung_Colorectal_CoRes
-1
0
1
N
Carnforth
Caton
Bay
Horse
M6
5 km

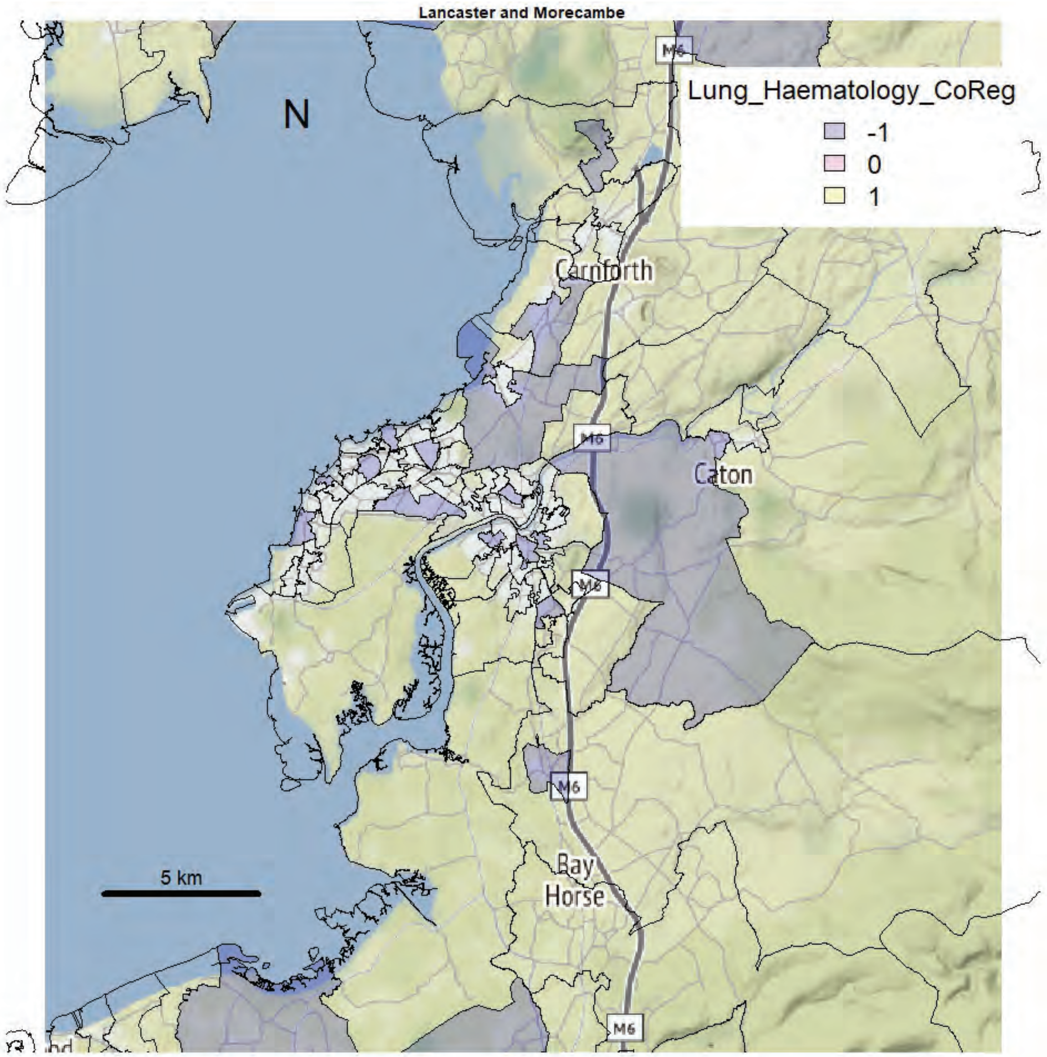
Lancaster and Morecambe
Lung_Haematology_CoReg
-1
0
1
N
Carnforth
Caton
Bay
Horse
M6
5 km

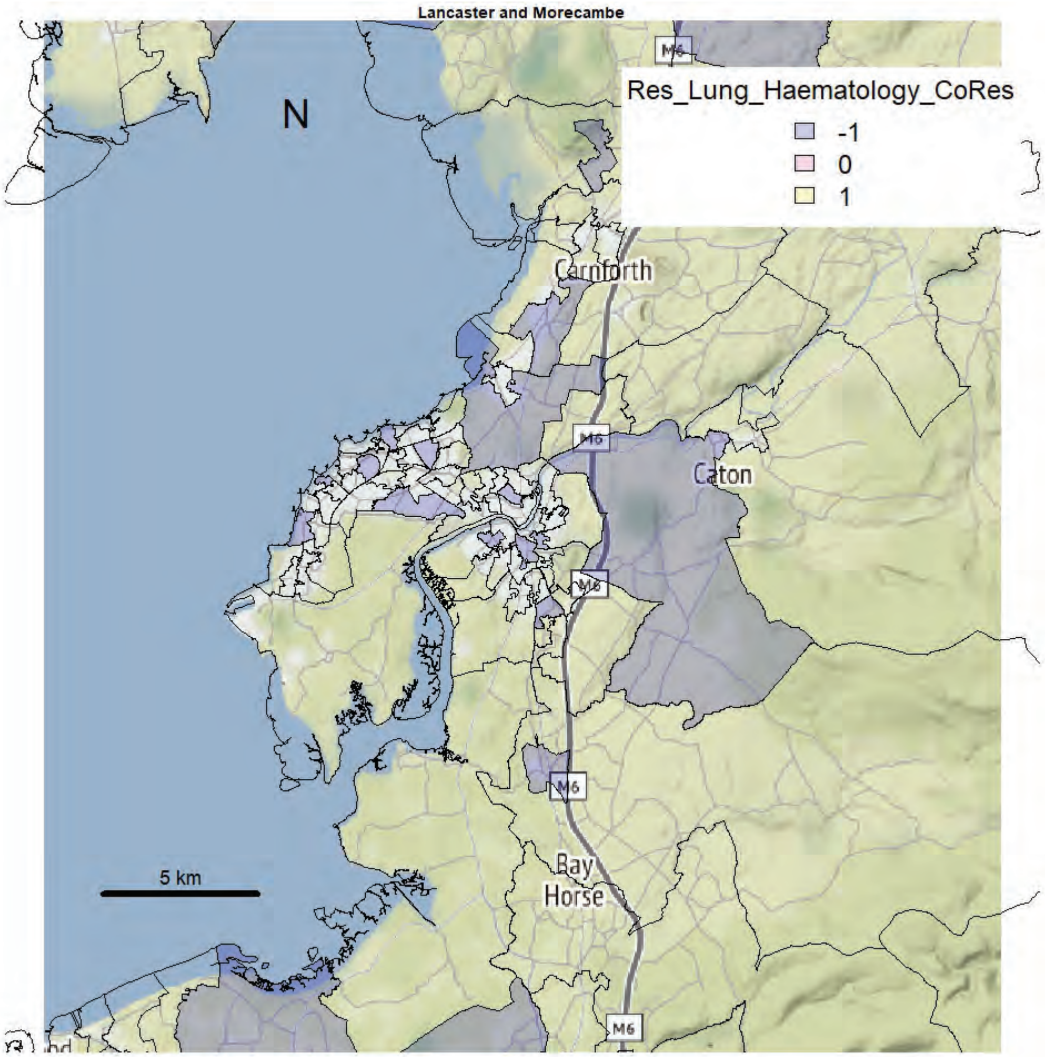
Lancaster and Morecambe
Res_Lung_Haematology_CoRes
-1
0
1
N
Carnforth
Caton
Bay
Horse
M6
5 km

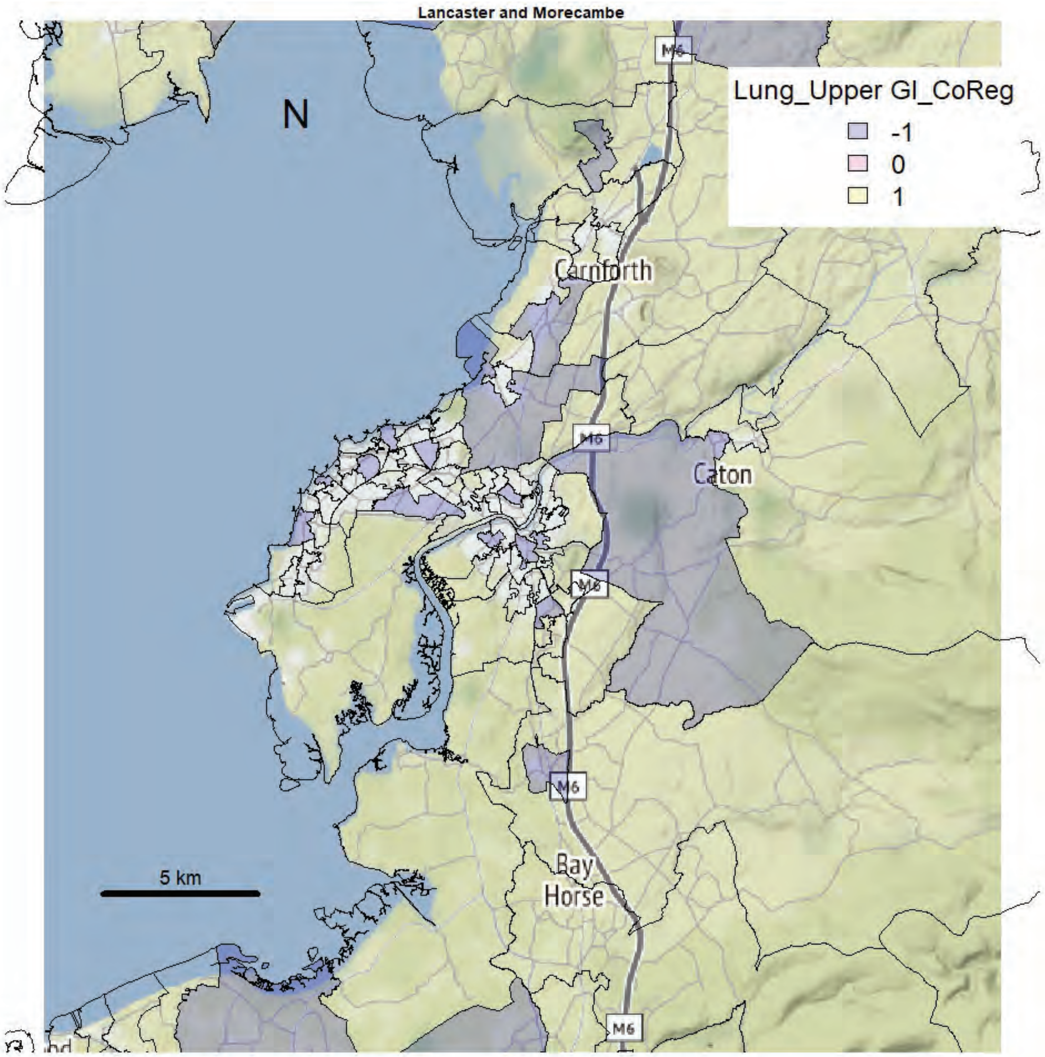
Lancaster and Morecambe
N
Lung_Upper GI_CoReg
-1
0
1
Carnforth
Caton
Bay
Horse
M6
5 km

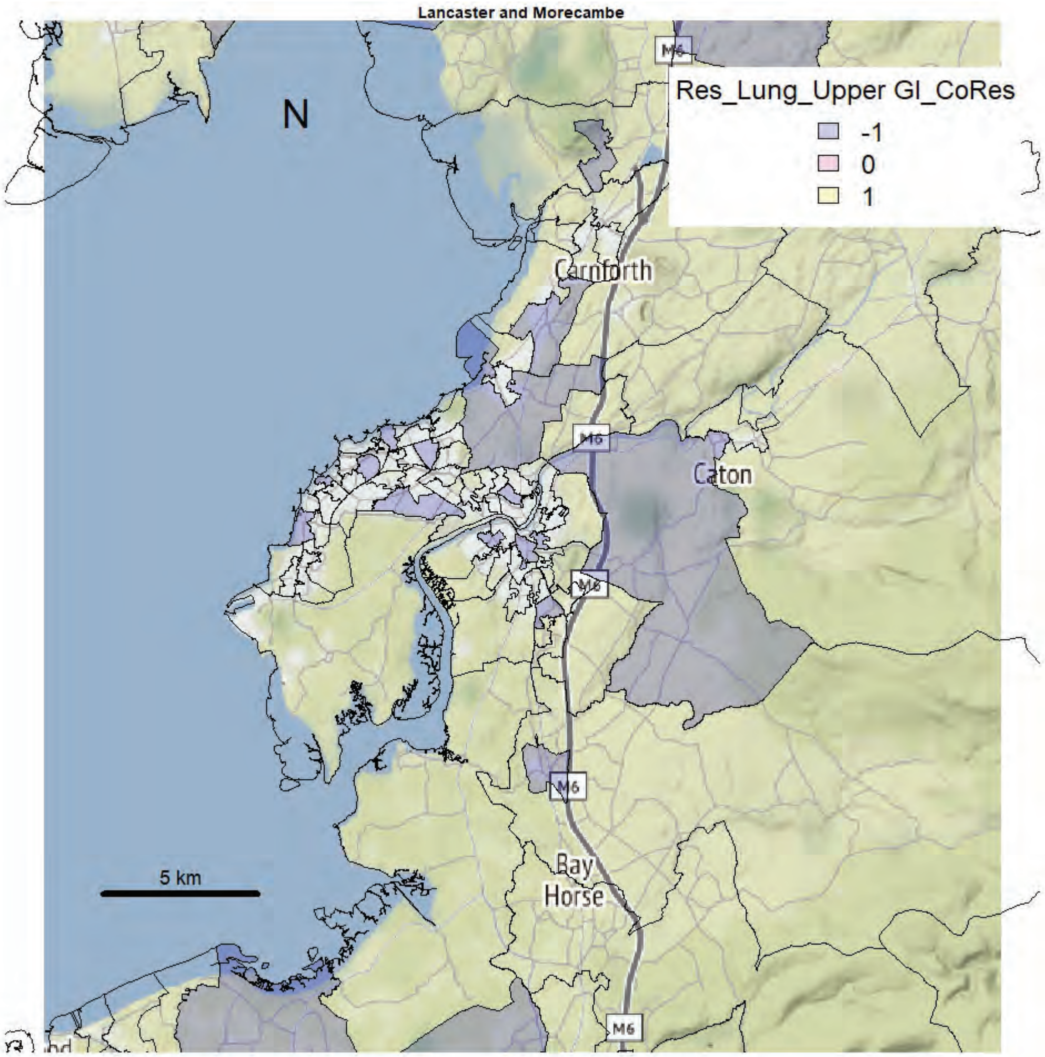
Lancaster and Morecambe
N
Res_Lung_Upper GI_CoRes
-1
0
1
Carnforth
Caton
Bay
Horse
M6
5 km

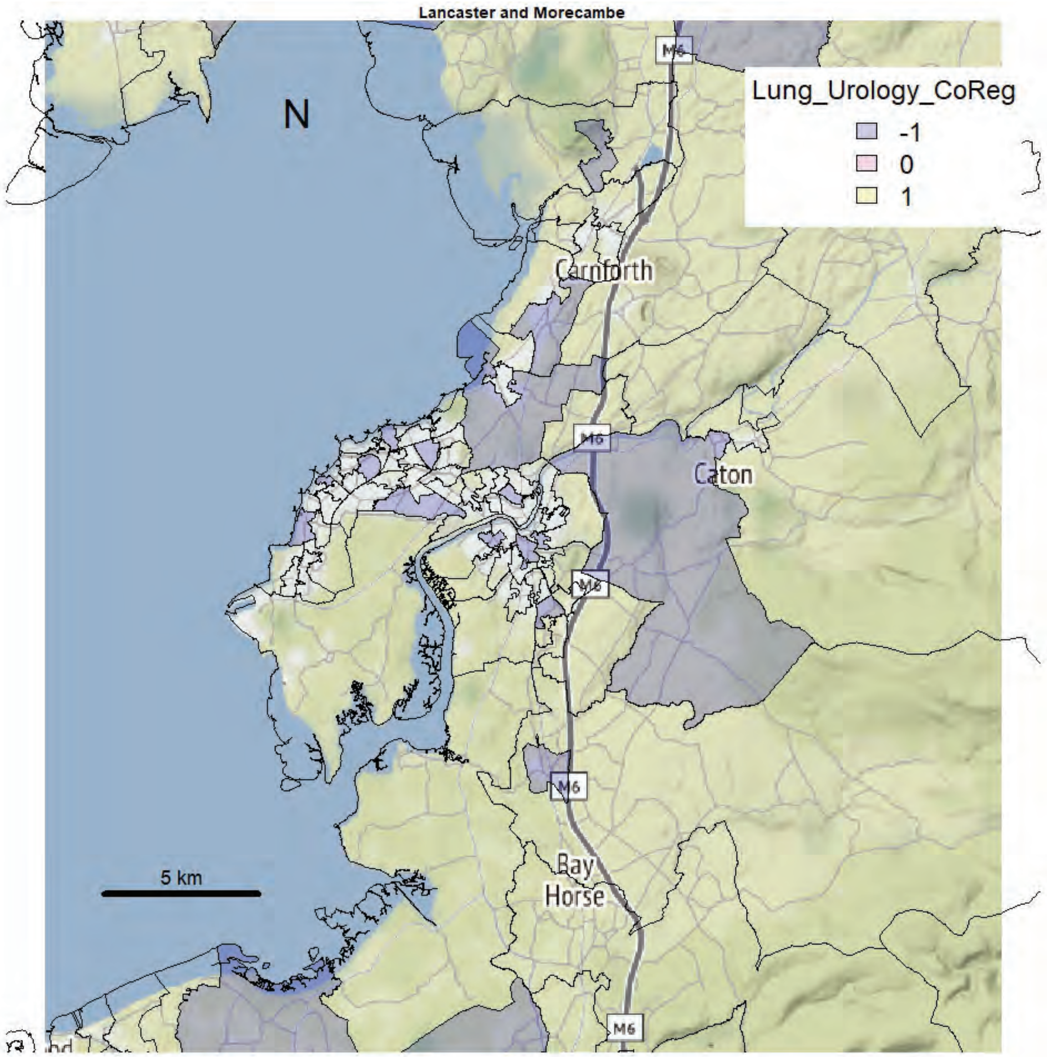

Lancaster and Morecambe
Lung_Urology_CoReg
-1
0
1
N
Carnforth
Caton
Bay
Horse
M6
5 km

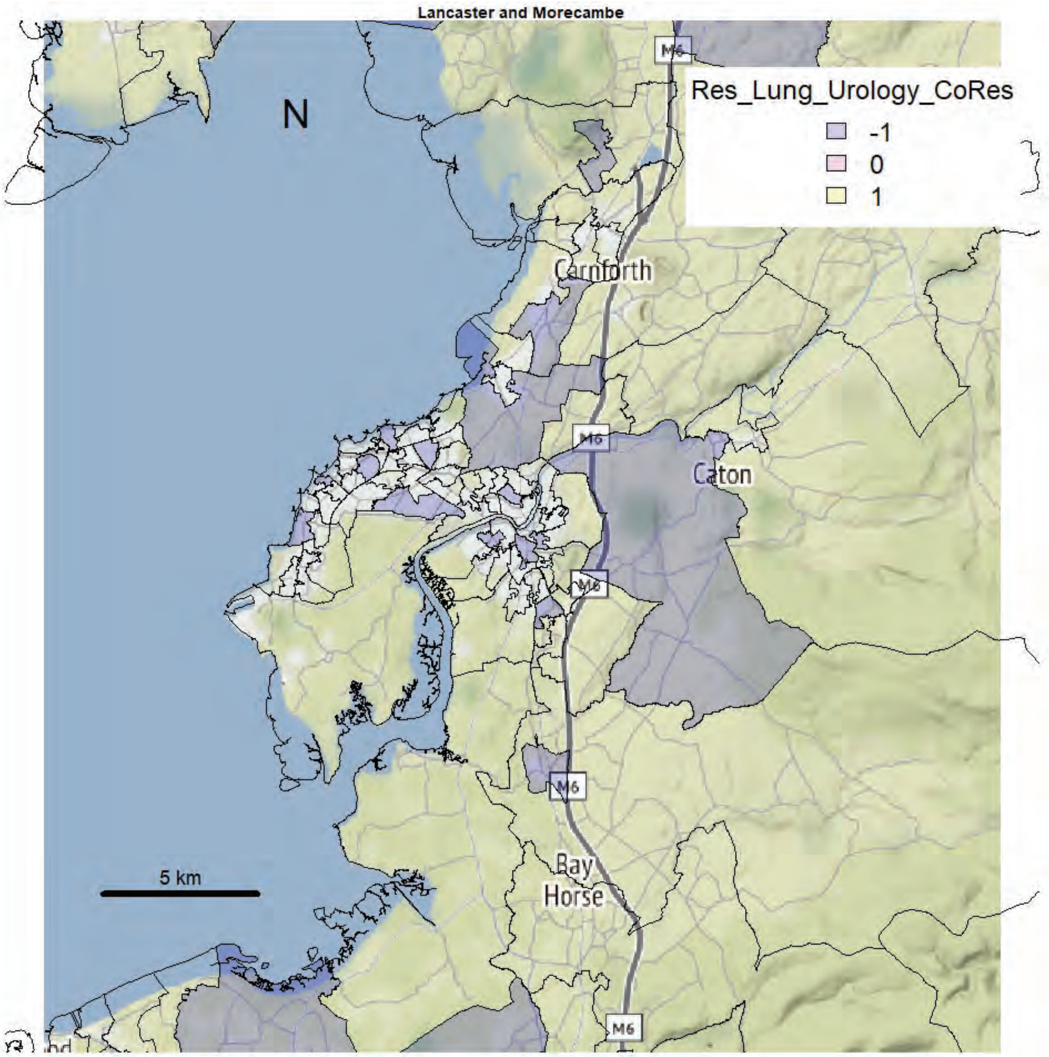

Lancaster and Morecambe
Res_Lung_Urology_CoRes
-1
0
1
N
Carnforth
Caton
Bay
Horse
M6
5 km

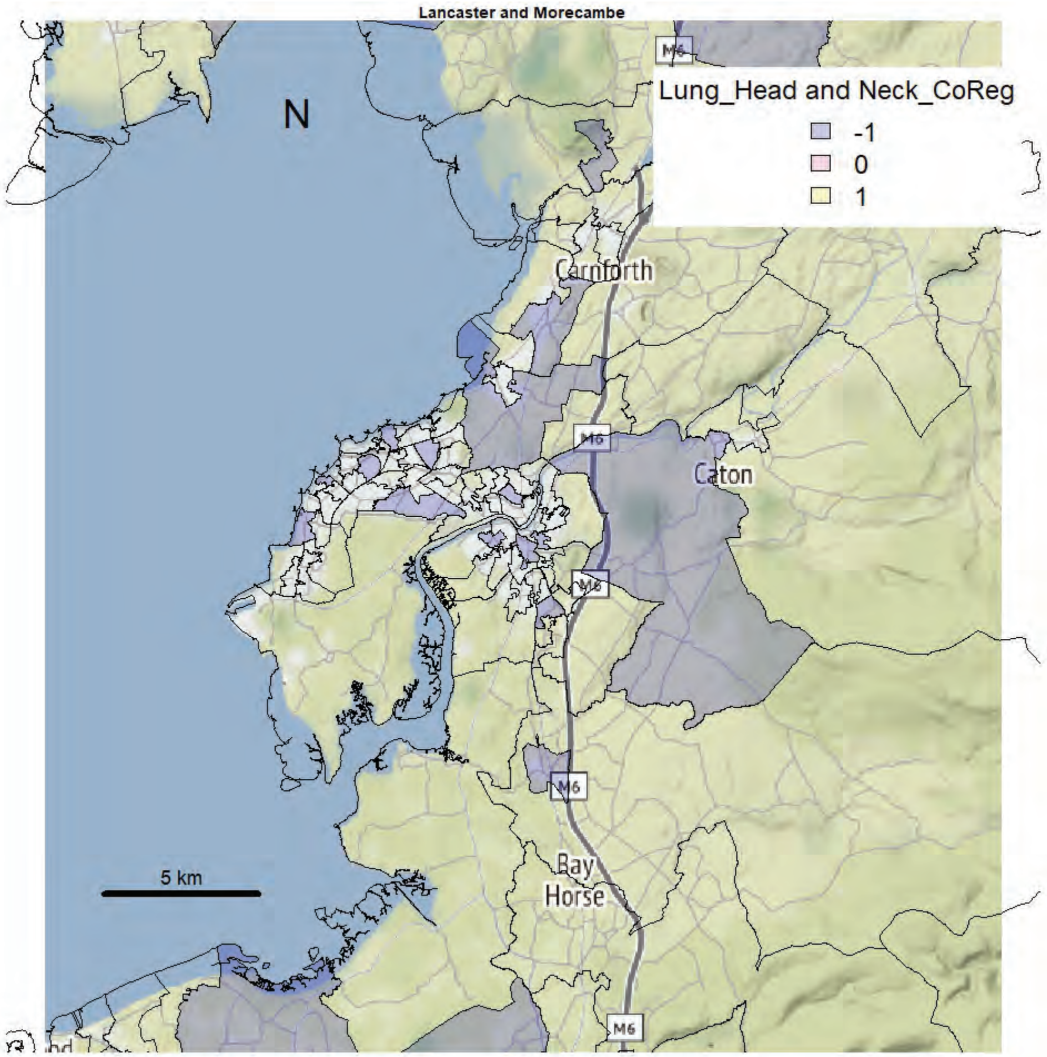
Lancaster and Morecambe
Lung_Head and Neck_CoReg
-1
0
1
N
Carnforth
Caton
Bay
Horse
M6
5 km

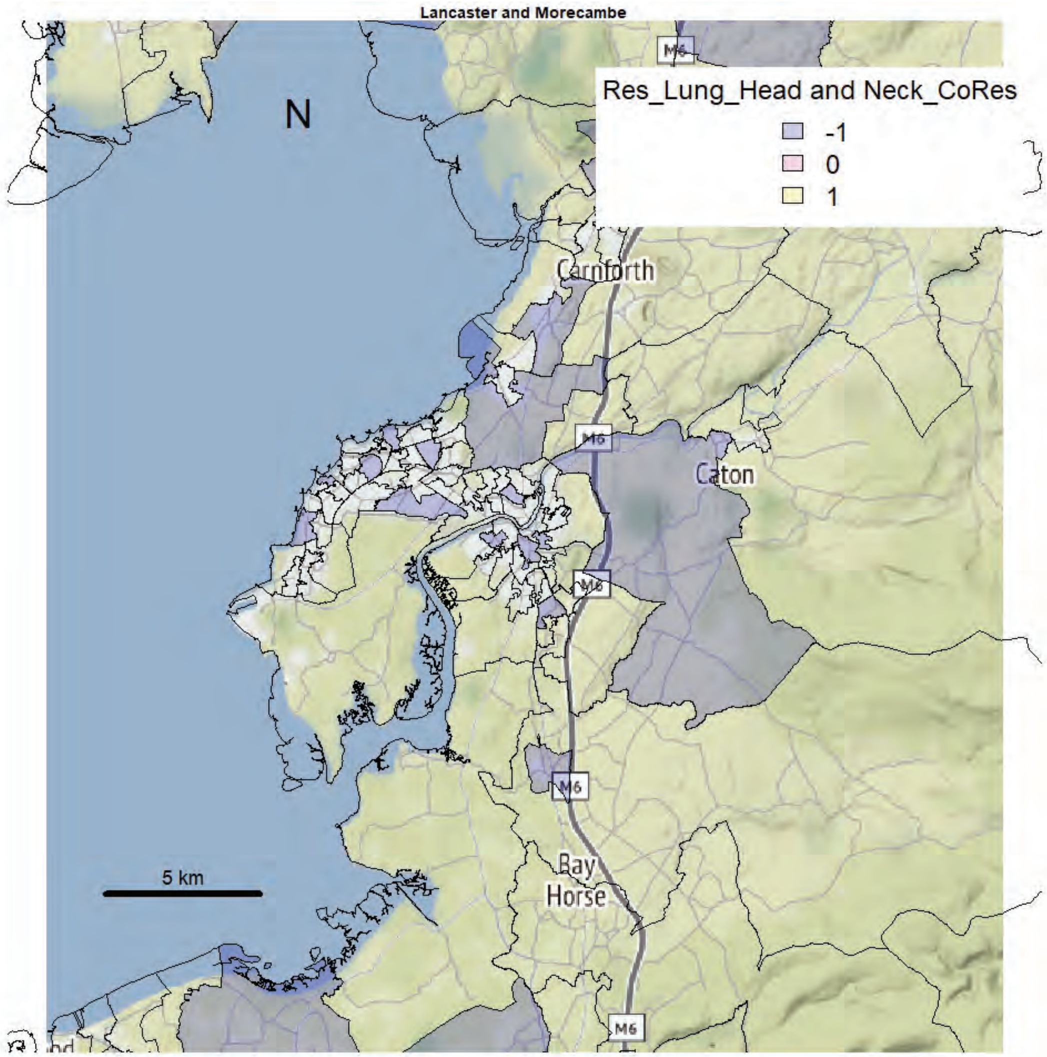
Lancaster and Morecambe
Res_Lung_Head and Neck_CoRes
-1
0
1
N
Carnforth
Caton
Bay
Horse
M6
5 km

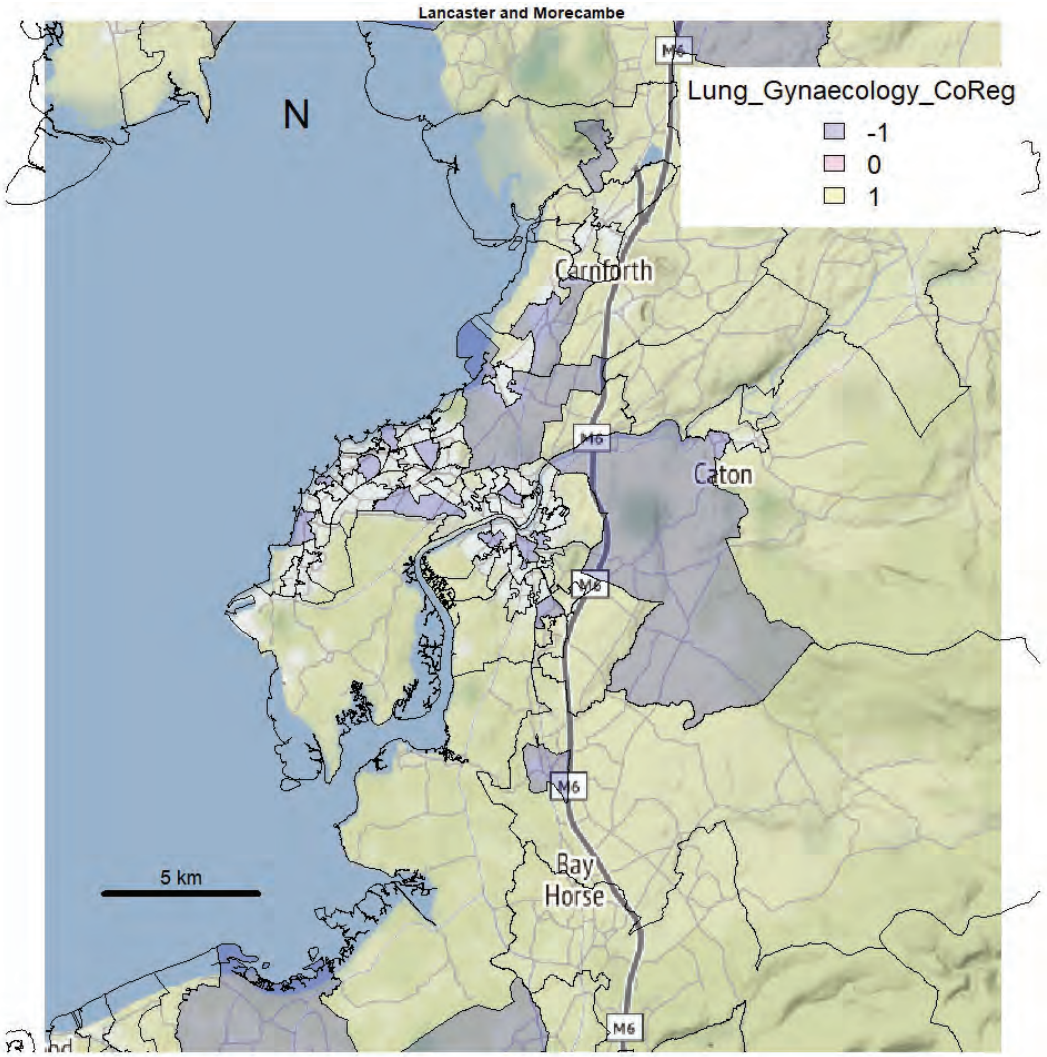
Lancaster and Morecambe
Lung_Gynaecology_CoReg
-1
0
1
N
Carnforth
Caton
Bay
Horse
M6
5 km

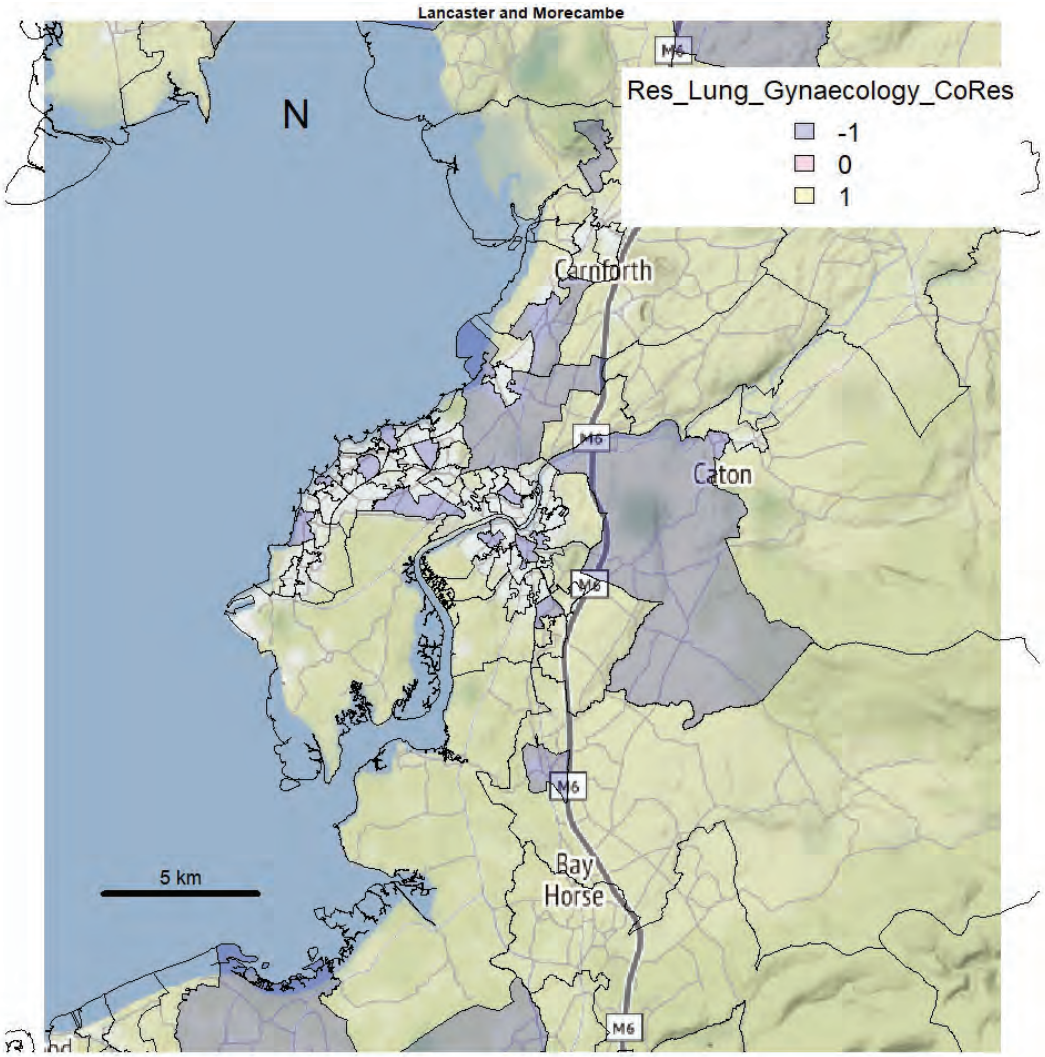
Lancaster and Morecambe
Res_Lung_Gynaecology_CoRes
-1
0
1
N
Carnforth
Caton
Bay
Horse
M6
5 km

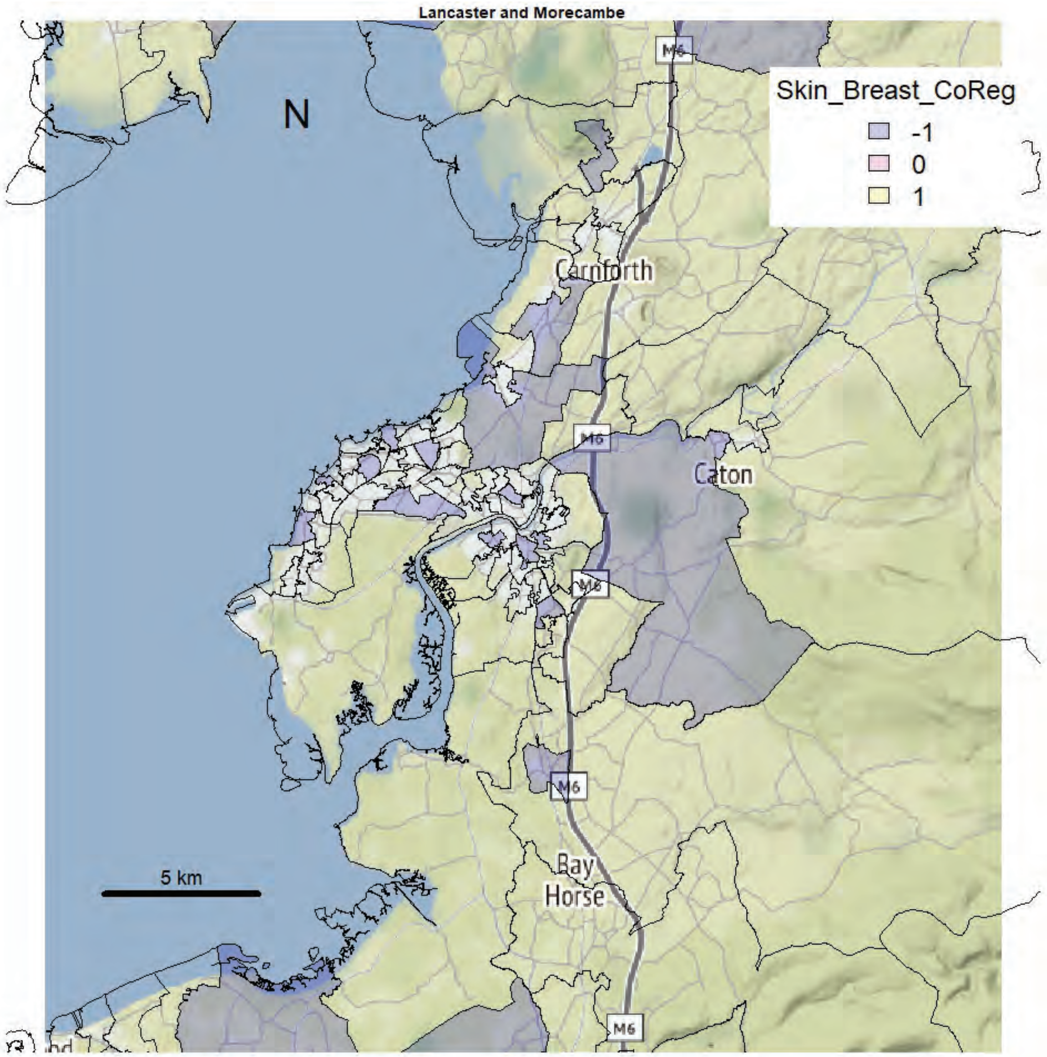
Lancaster and Morecambe
Skin_Breast_CoReg
-1
0
1
N
Carnforth
Caton
Bay Horse
M6
5 km

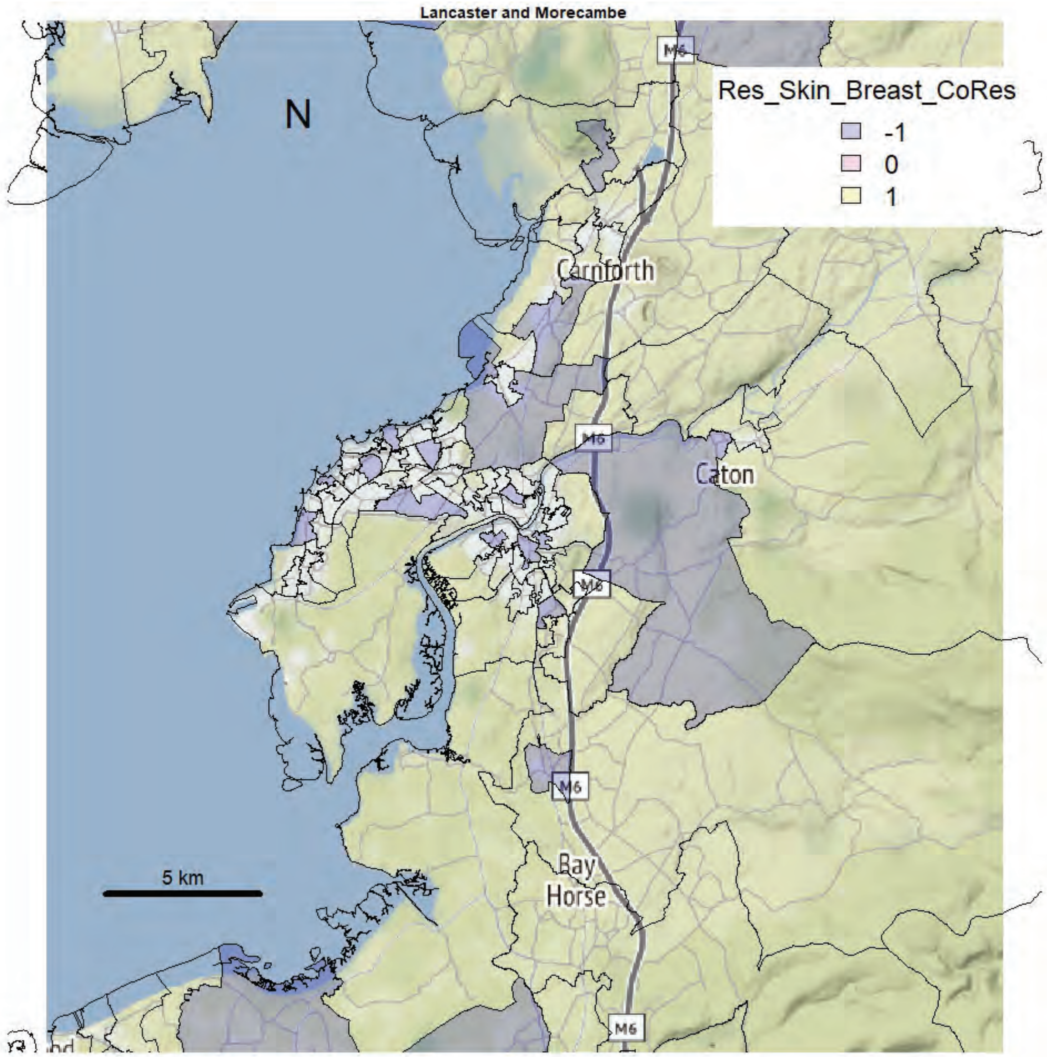
Lancaster and Morecambe
Res_Skin_Breast_CoRes
-1
0
1
N
Carnforth
Caton
Bay Horse
M6
5 km

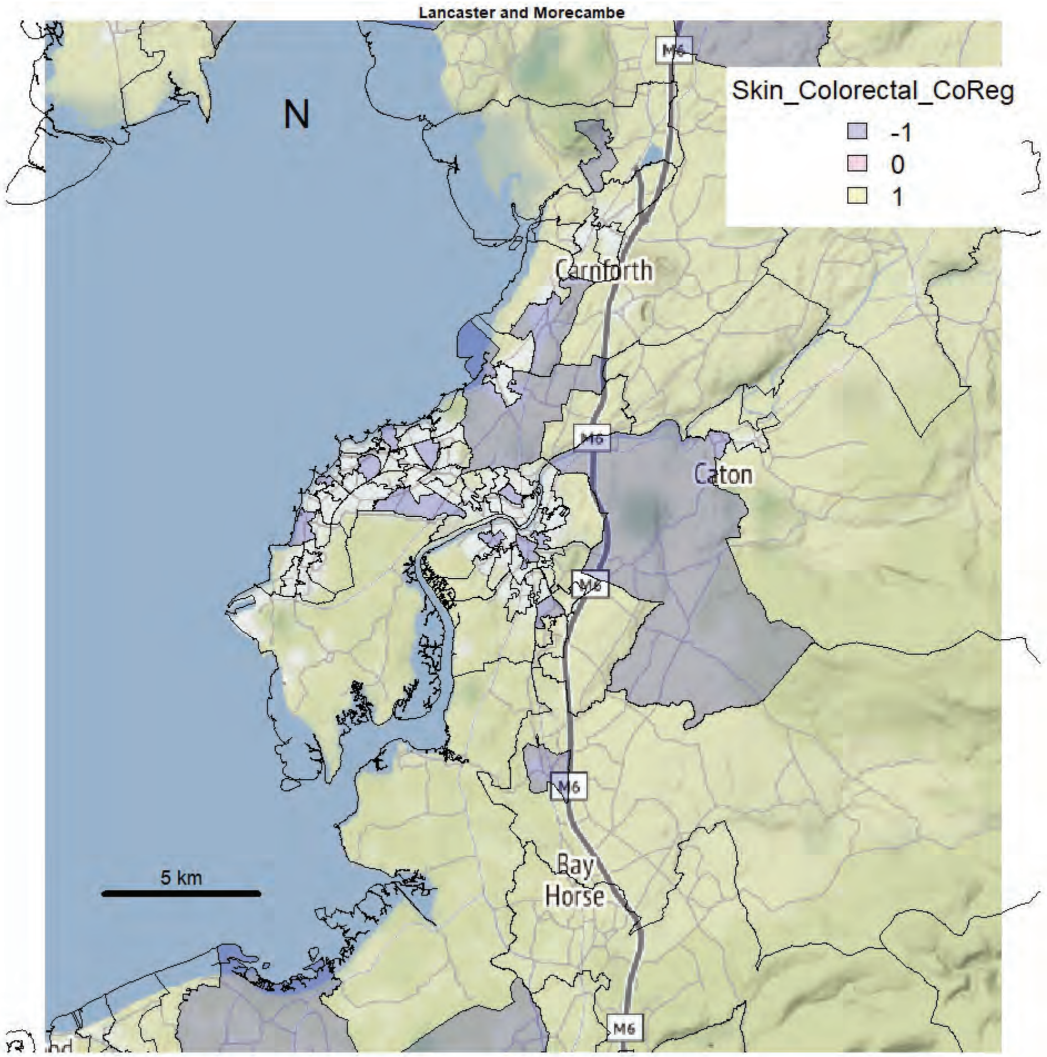
Lancaster and Morecambe
Skin_Colorectal_CoReg
-1
0
1
N
Carnforth
Caton
Bay
Horse
5 km
M6

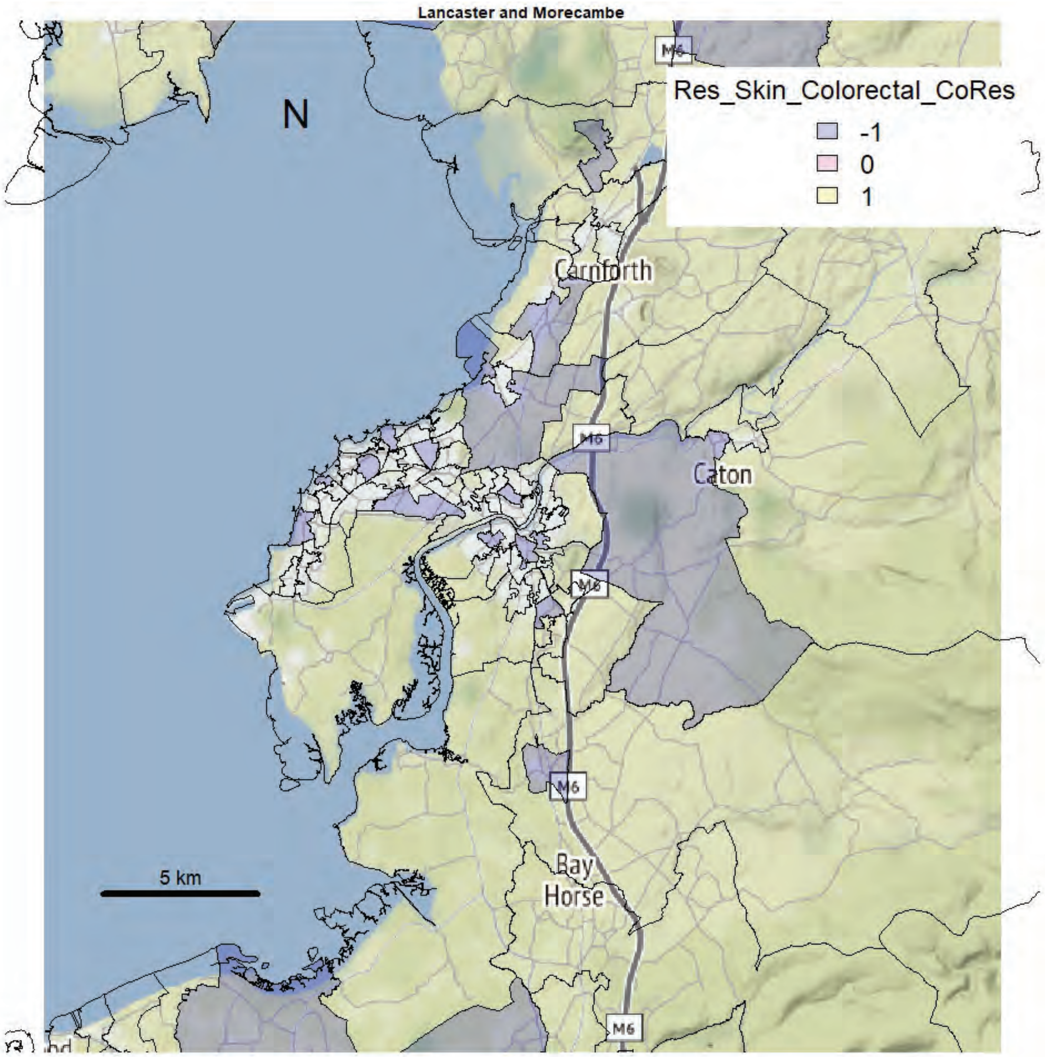
Lancaster and Morecambe
Res_Skin_Colorectal_CoRes
-1
0
1
N
Carnforth
Caton
Bay
Horse
5 km
M6

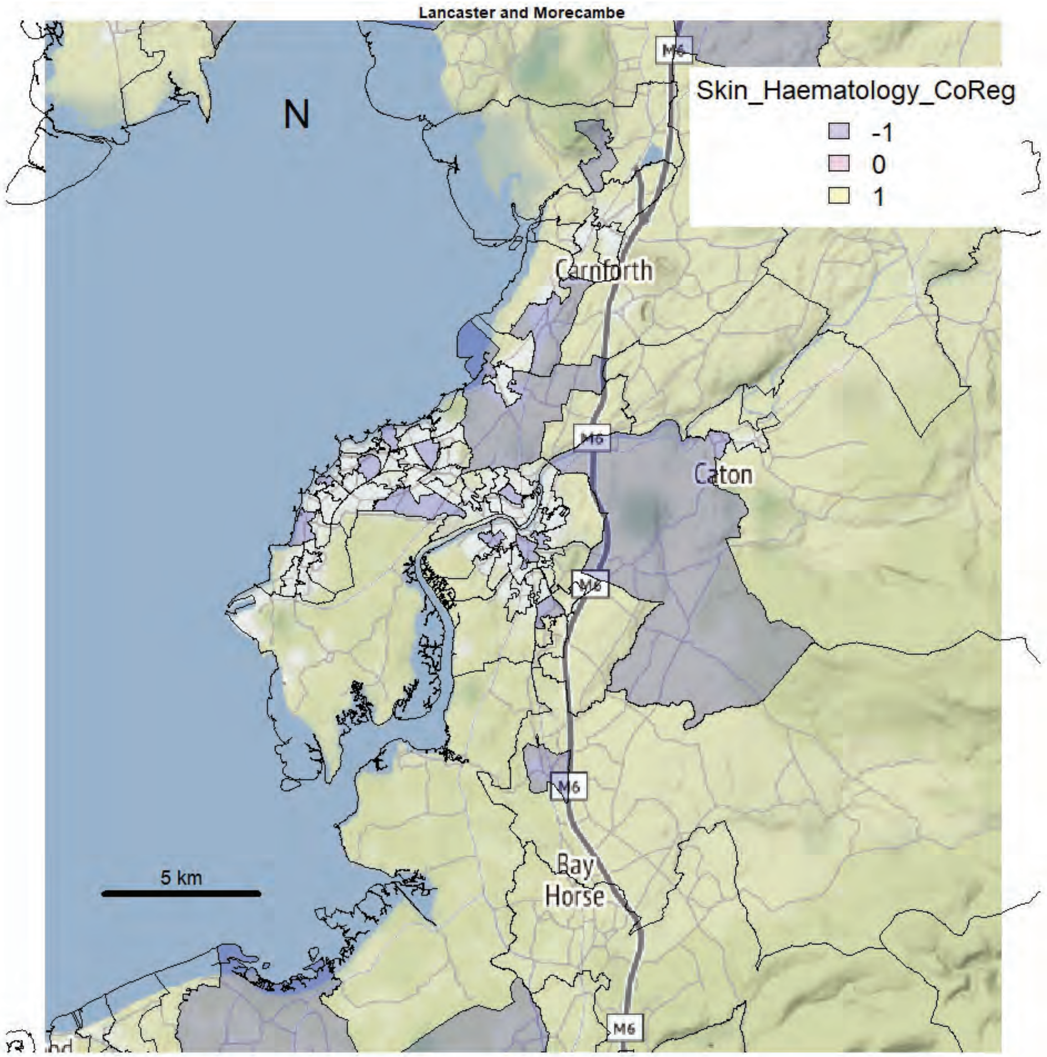
Lancaster and Morecambe
Skin_Haematology_CoReg
-1
0
1
N
Carnforth
Caton
Bay
Horse
M6
5 km

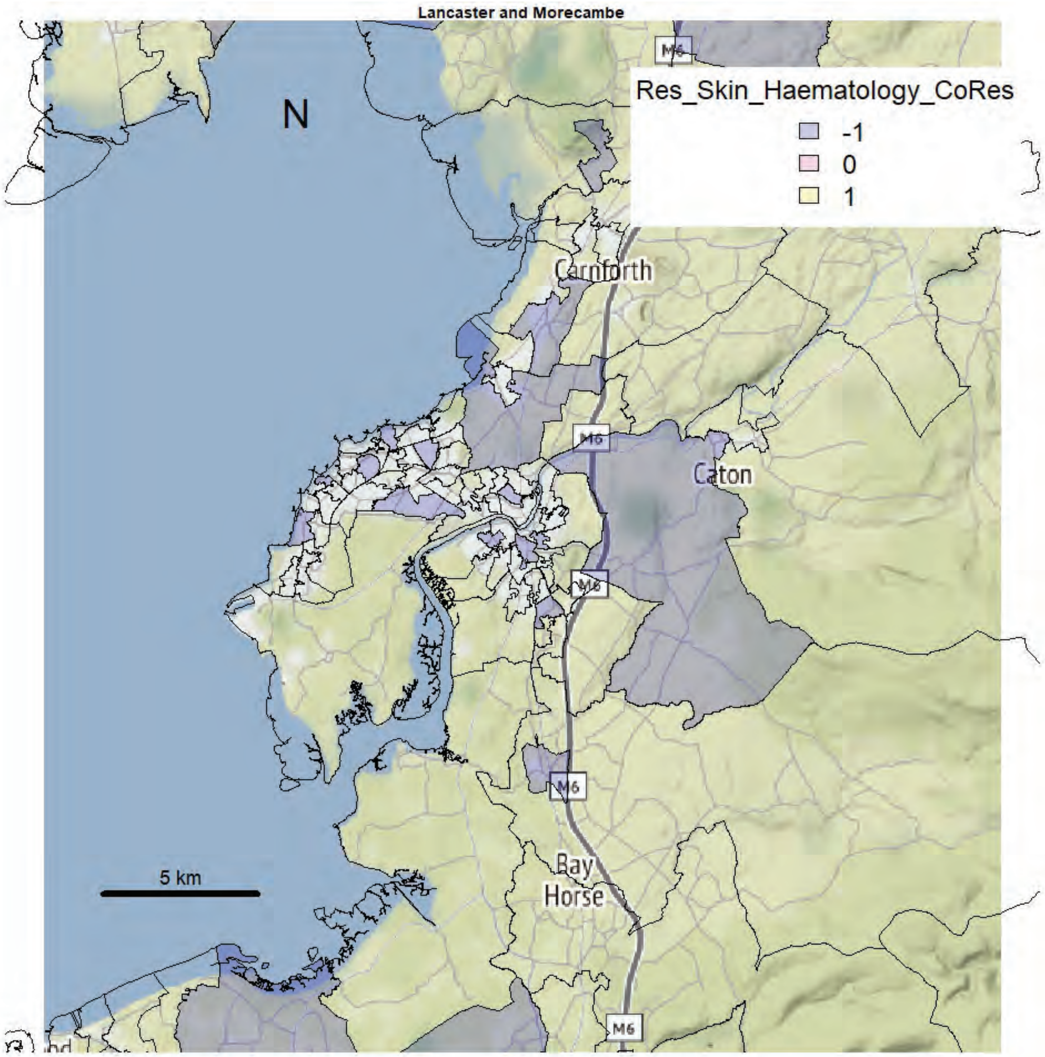
Lancaster and Morecambe
Res_Skin_Haematology_CoRes
-1
0
1
N
Carnforth
Caton
Bay
Horse
M6
5 km

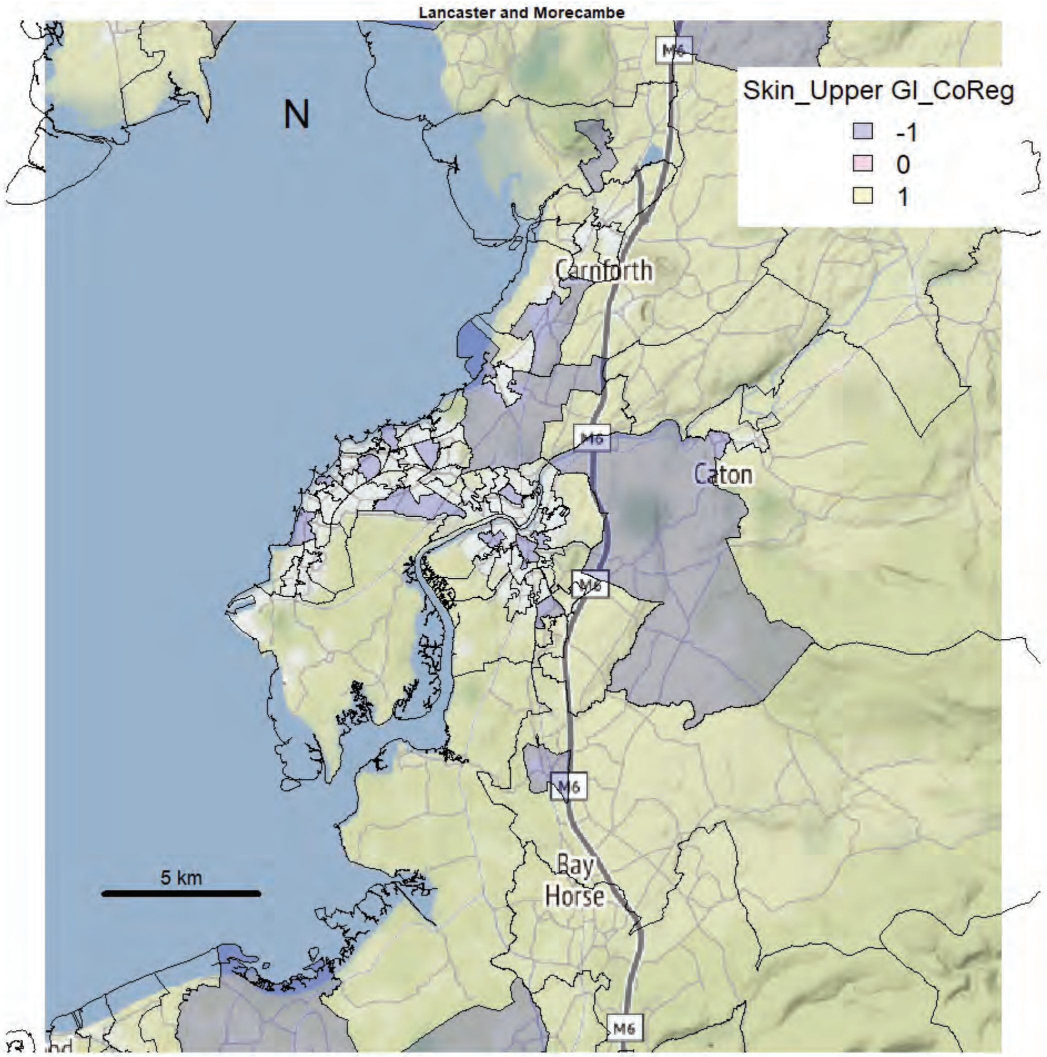
Lancaster and Morecambe
Skin_Upper GI_CoReg
-1
0
1
N
Carnforth
Caton
Bay
Horse
M6
5 km

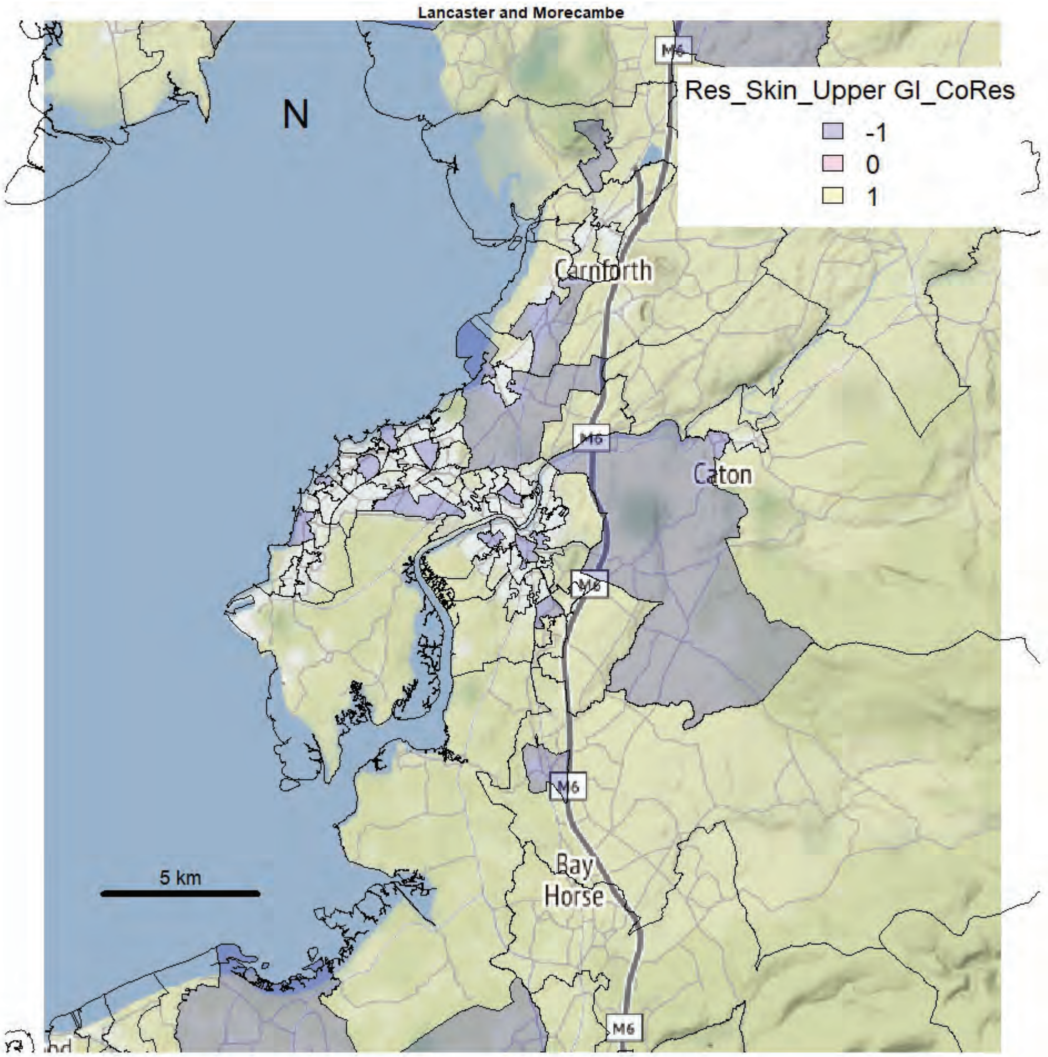
Lancaster and Morecambe
Res_Skin_Upper GI_CoRes
-1
0
1
N
Carnforth
Caton
Bay
Horse
M6
5 km

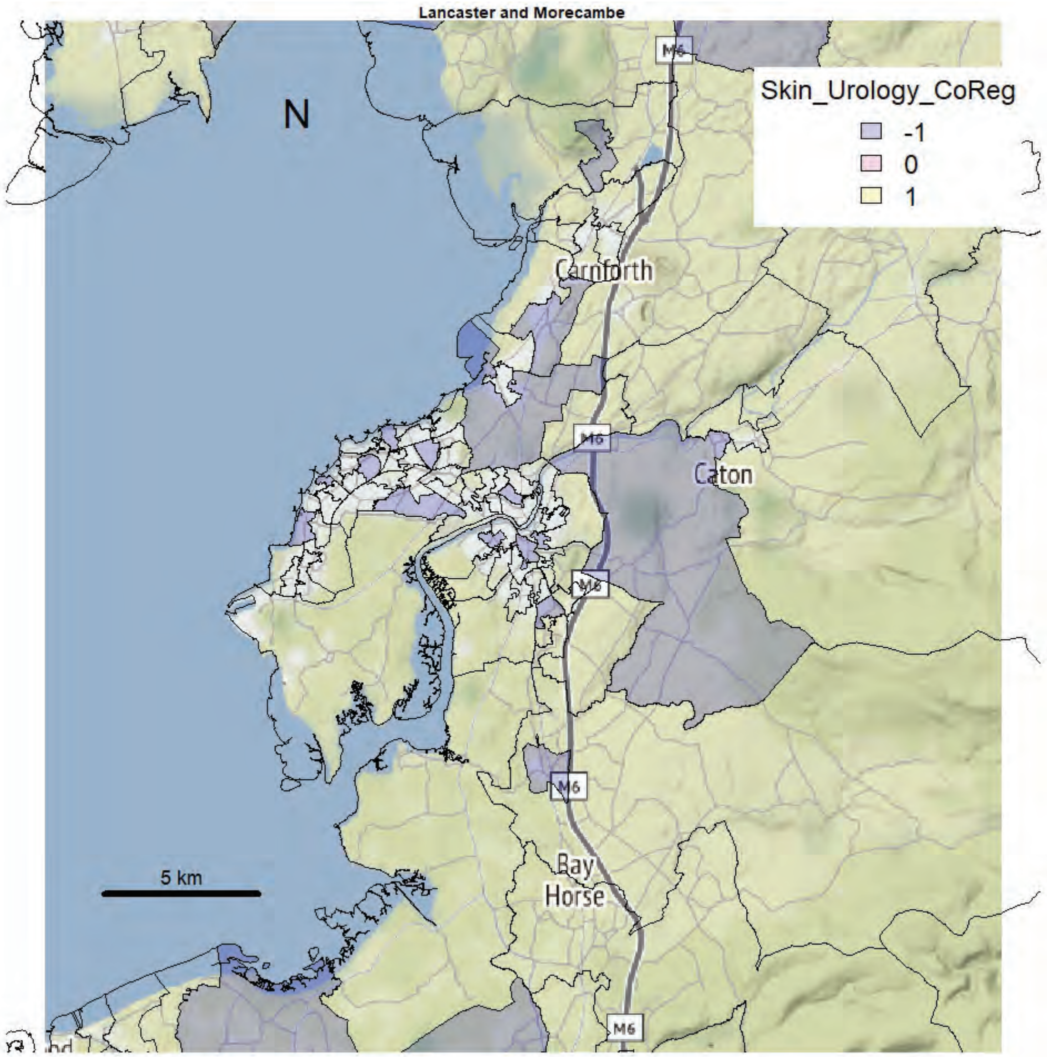

Lancaster and Morecambe
N
Skin_Urology_CoReg
-1
0
1
Carnforth
Caton
Bay Horse
M6
5 km

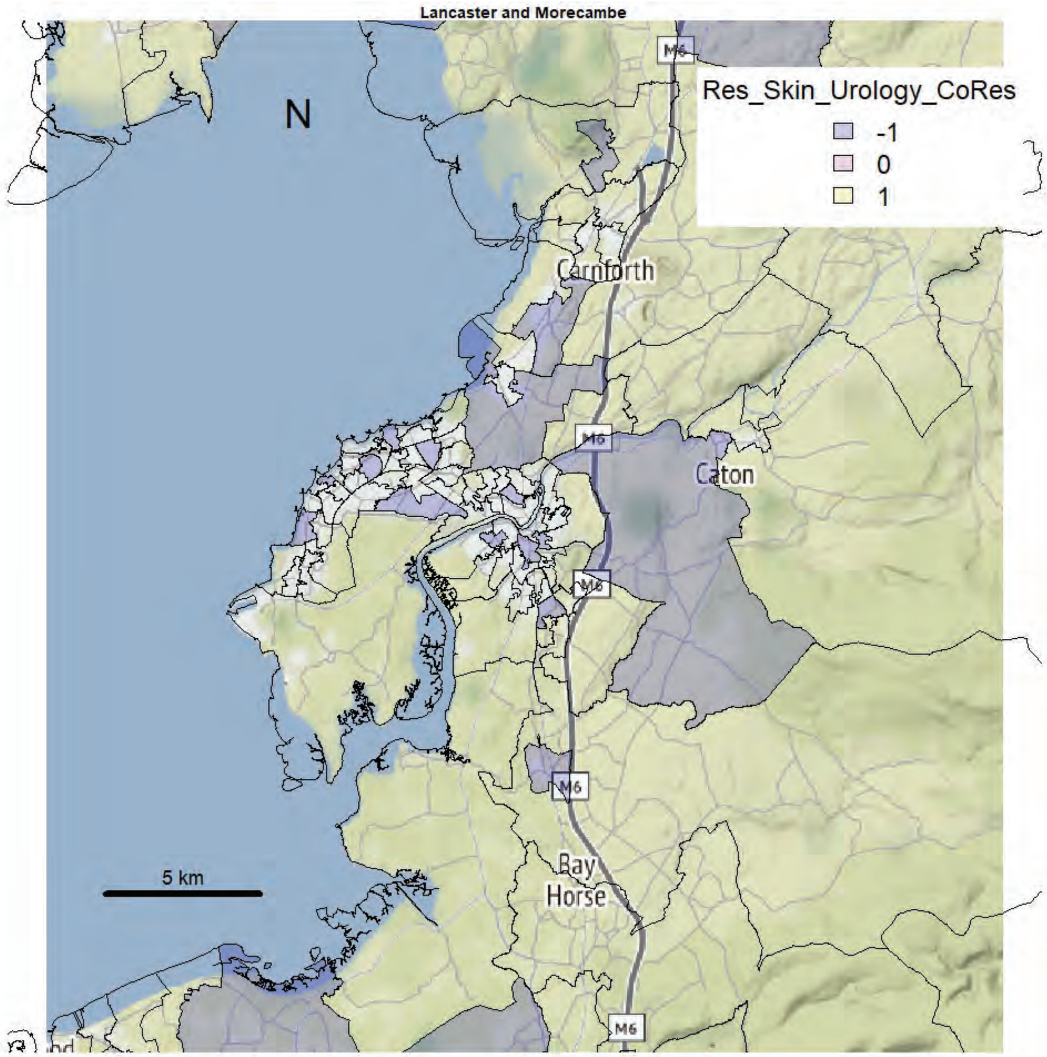

Lancaster and Morecambe
N
Res_Skin_Urology_CoRes
-1
0
1
Carnforth
Caton
Bay Horse
M6
5 km

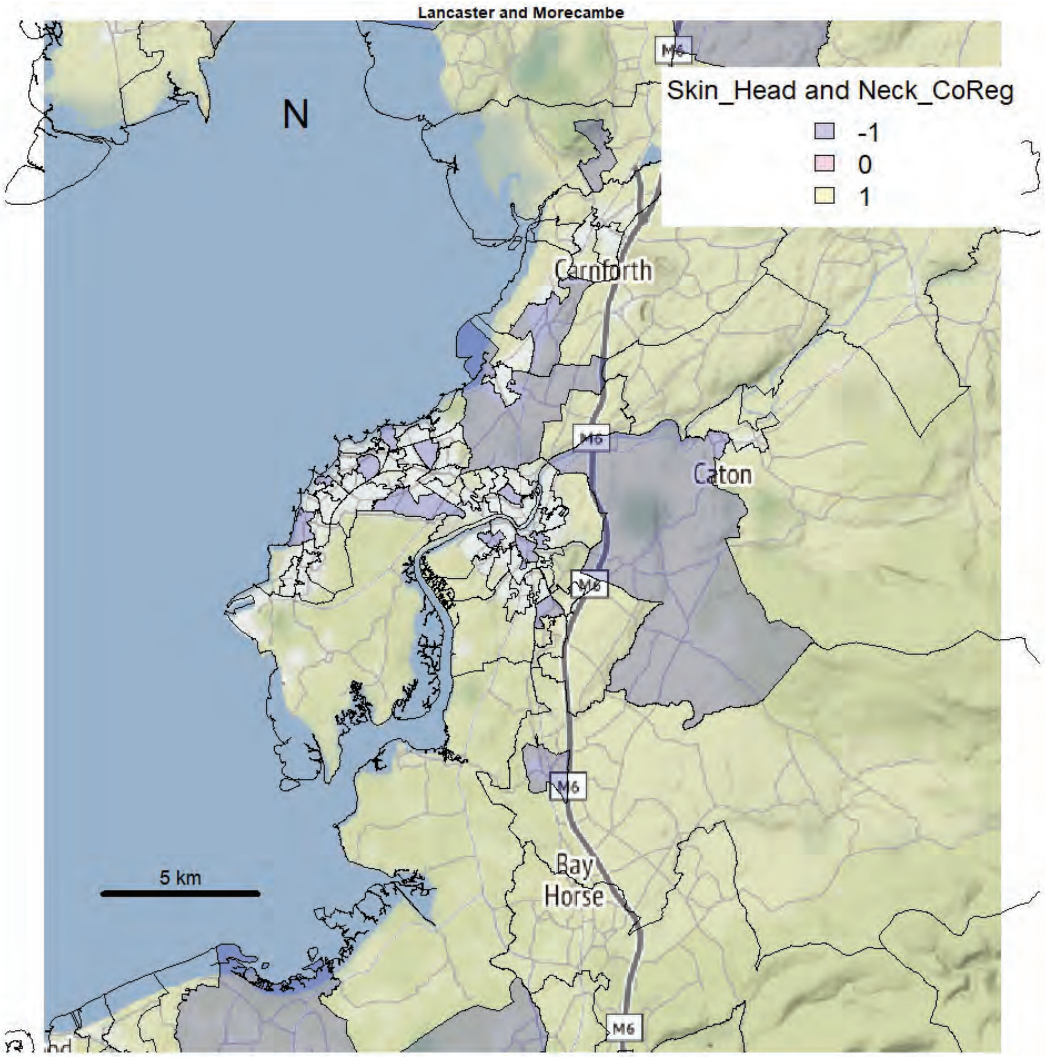
Lancaster and Morecambe
Skin_Head and Neck_CoReg
-1
0
1
N
Carnforth
Caton
Bay
Horse
M6
5 km

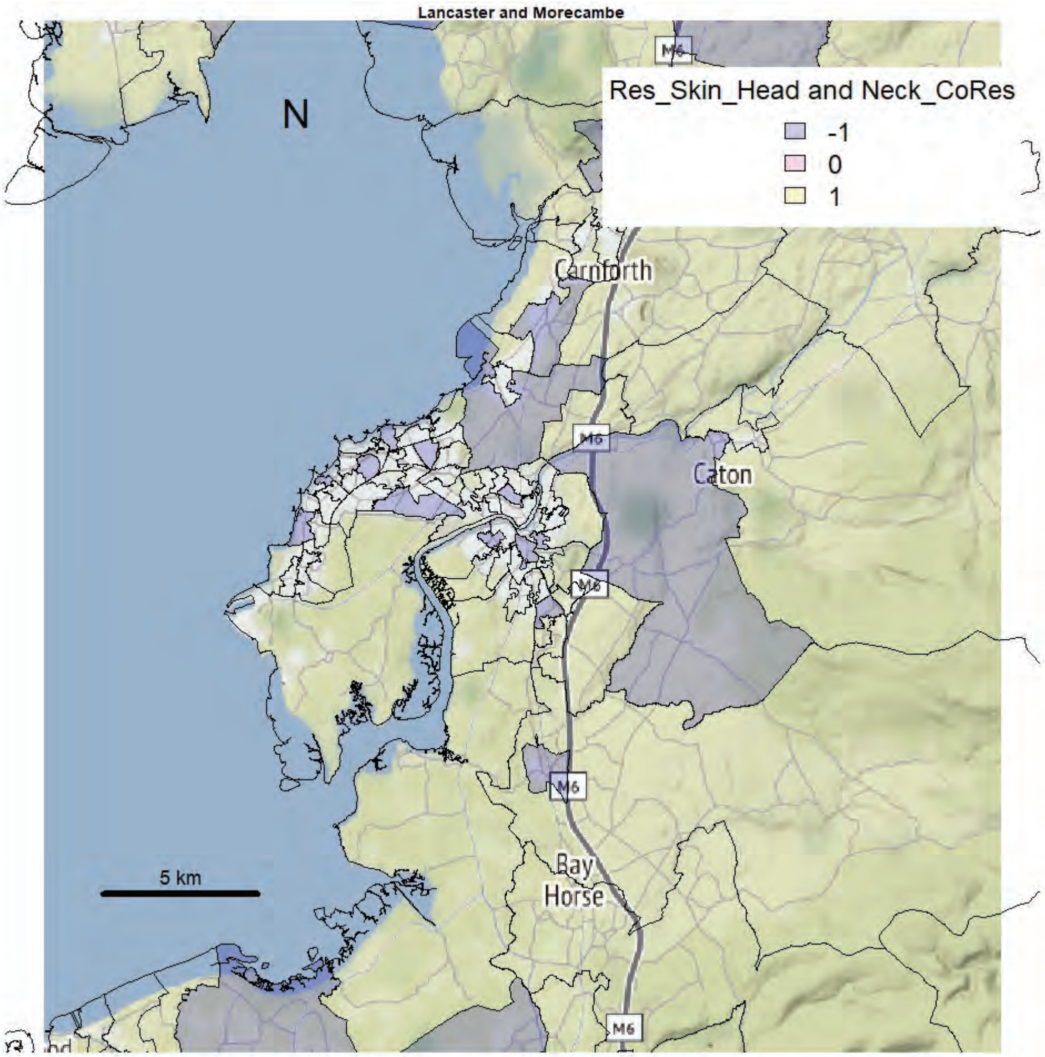
Lancaster and Morecambe
Res_Skin_Head and Neck_CoRes
-1
0
1
N
Carnforth
Caton
Bay
Horse
M6
5 km

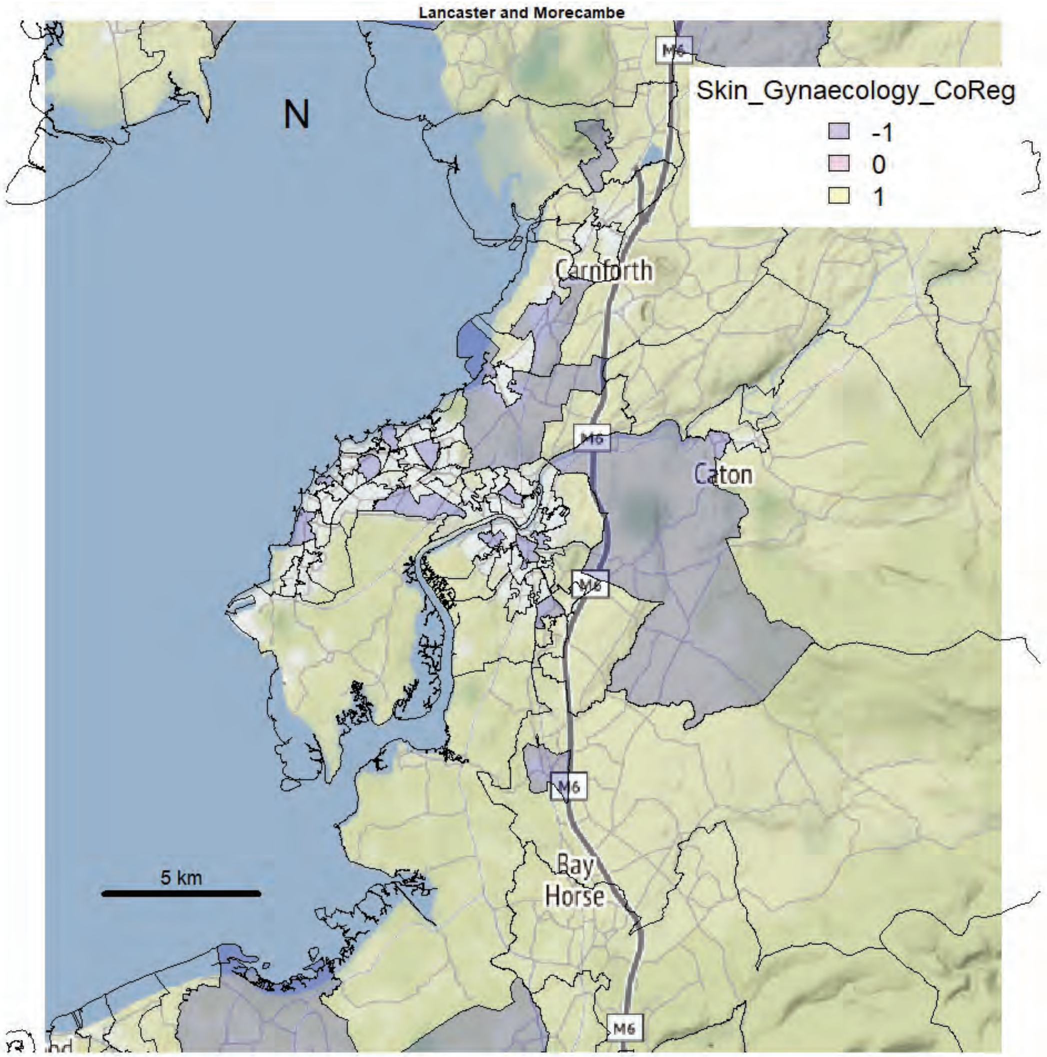
Lancaster and Morecambe
Skin_Gynaecology_CoReg
-1
0
1
N
Carnforth
Caton
Bay Horse
M6
5 km

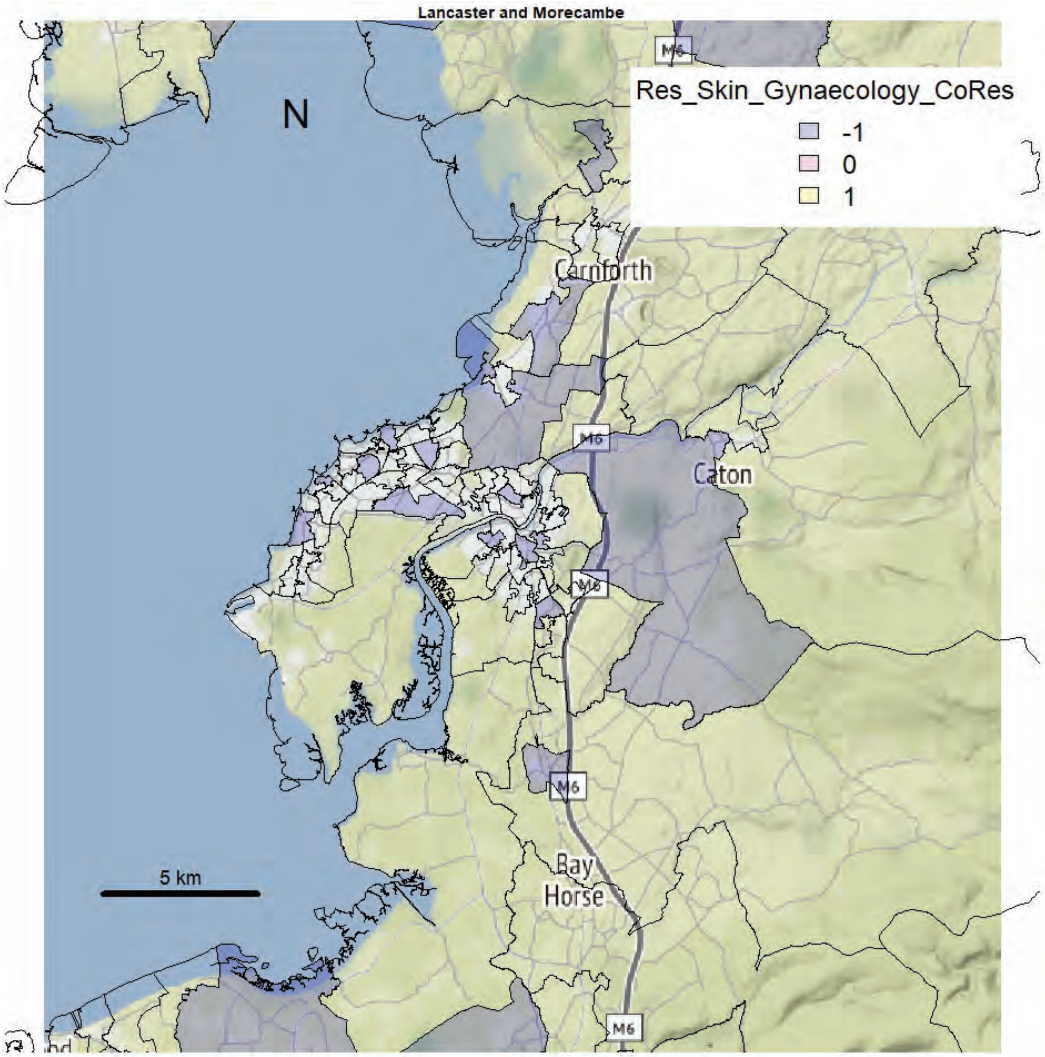
Lancaster and Morecambe
Res_Skin_Gynaecology_CoRes
-1
0
1
N
Carnforth
Caton
Bay Horse
M6
5 km

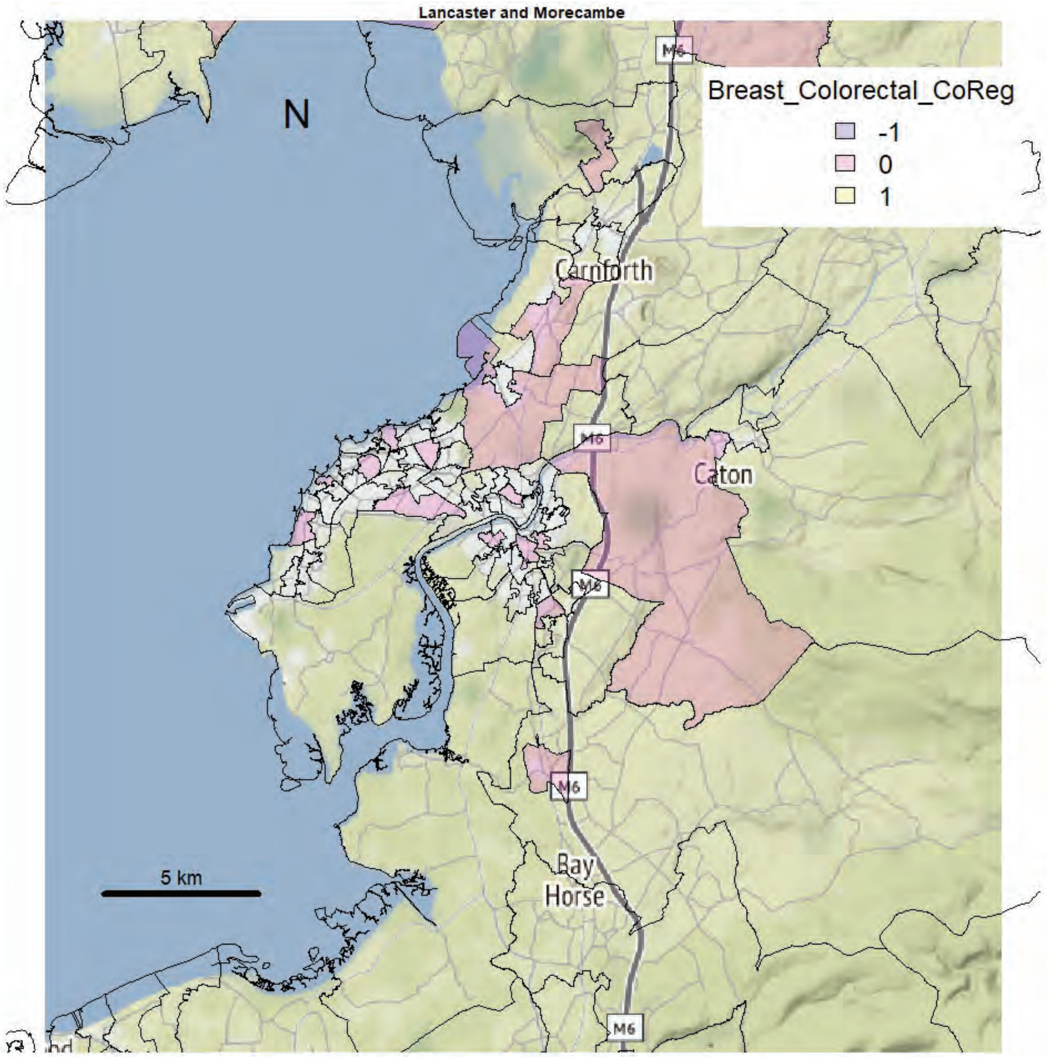
Lancaster and Morecambe
Breast_Colorectal_CoReg
-1
0
1
N
Carnforth
Caton
Bay
Horse
M6
5 km

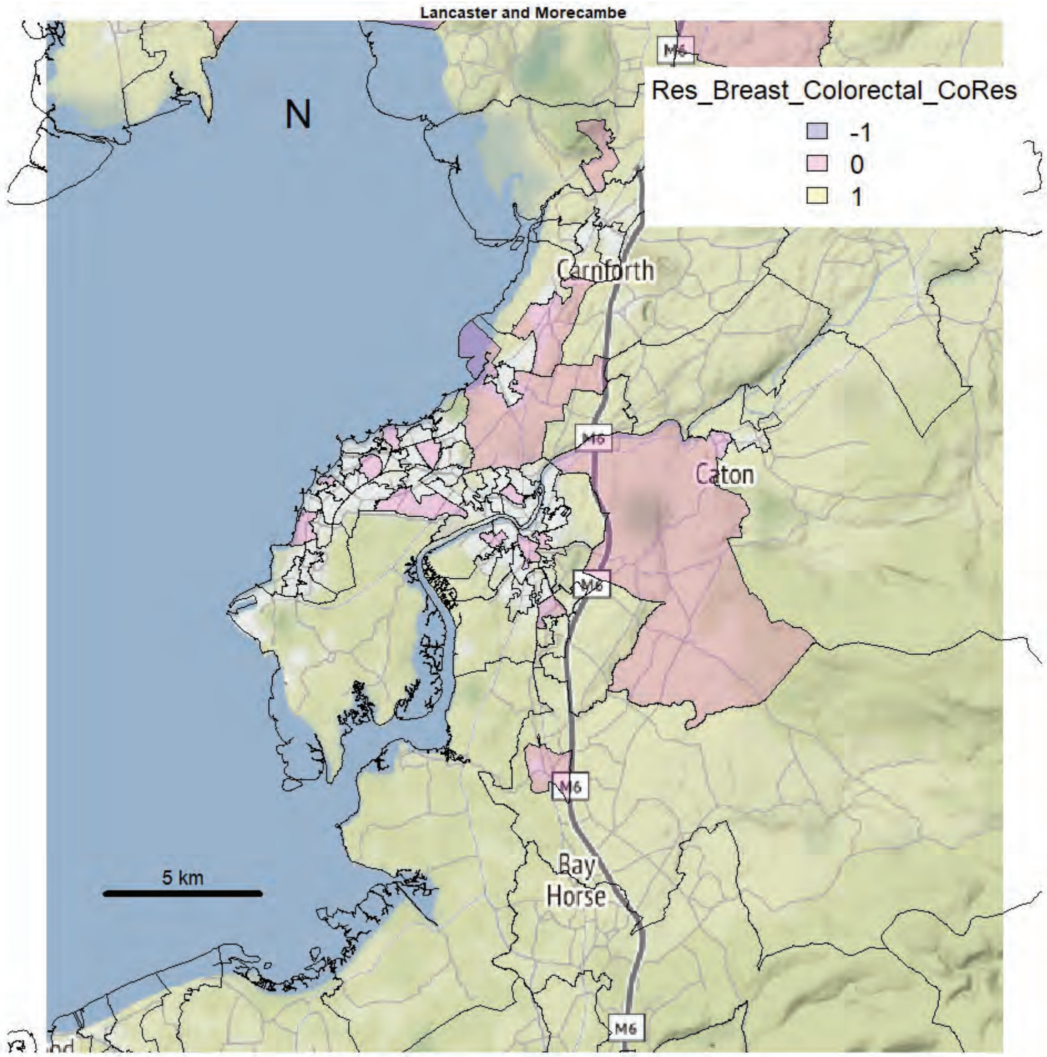
Lancaster and Morecambe
Res_Breast_Colorectal_CoRes
-1
0
1
N
Carnforth
Caton
Bay
Horse
M6
5 km

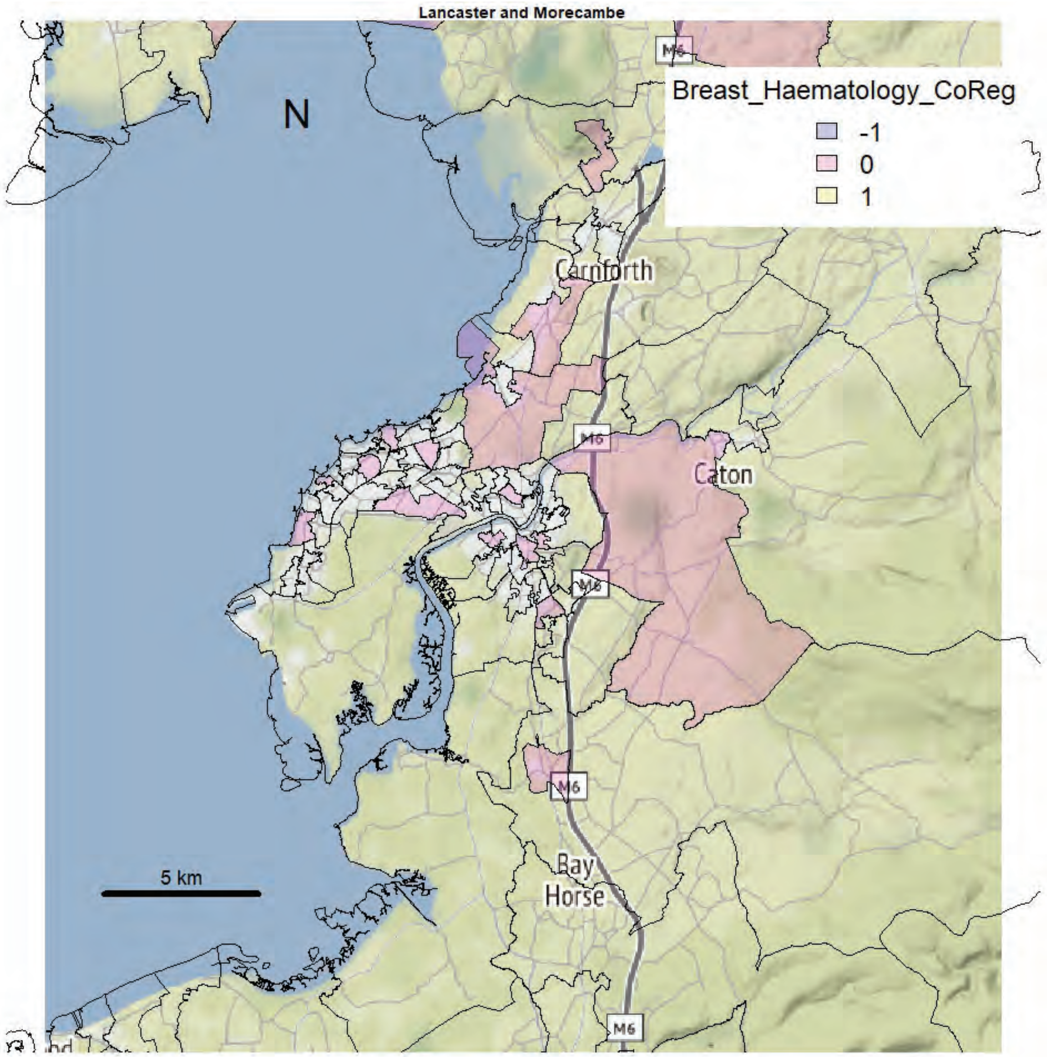
Lancaster and Morecambe
Breast_Haematology_CoReg
-1
0
1
N
Carnforth
Caton
M6
Bay
Horse
5 km

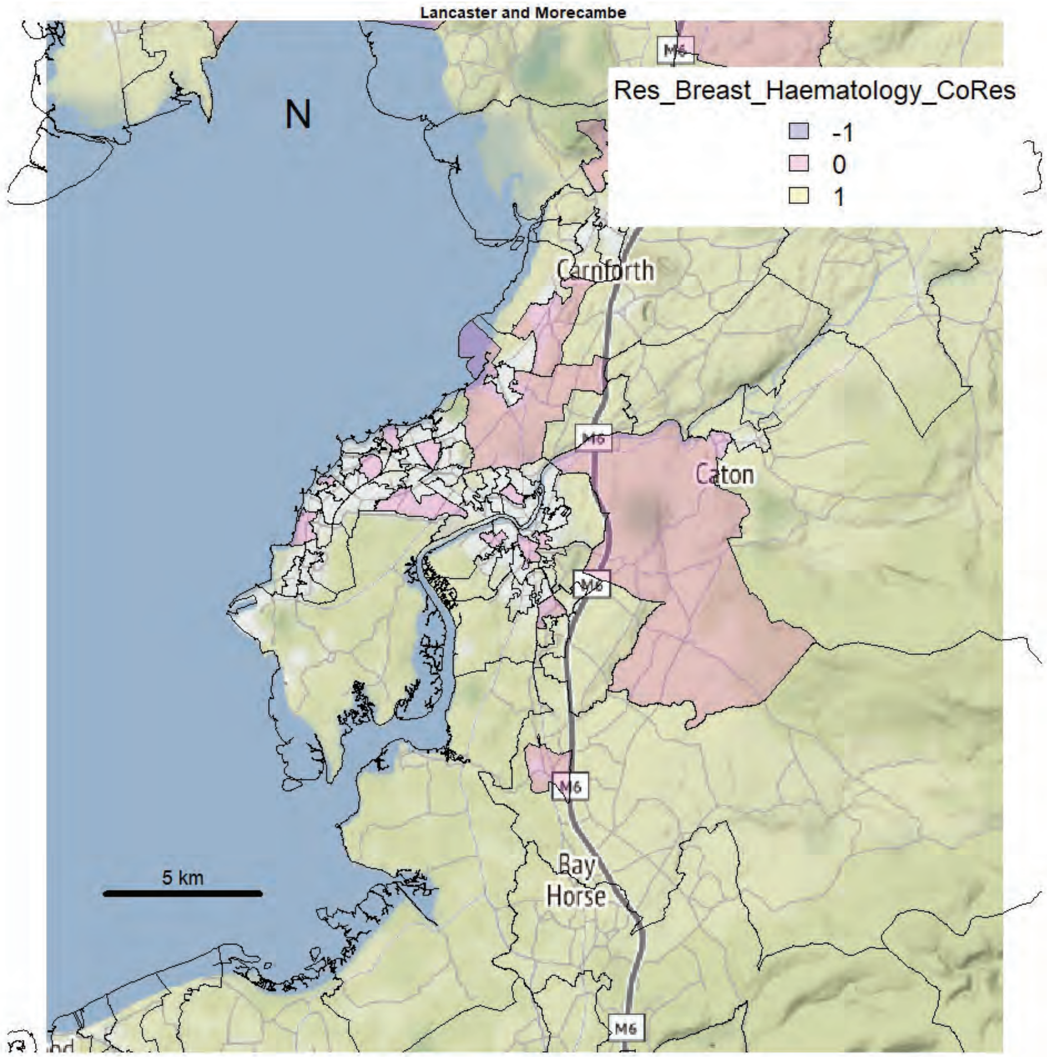
Lancaster and Morecambe
Res_Breast_Haematology_CoRes
-1
0
1
N
Carnforth
Caton
M6
Bay
Horse
5 km

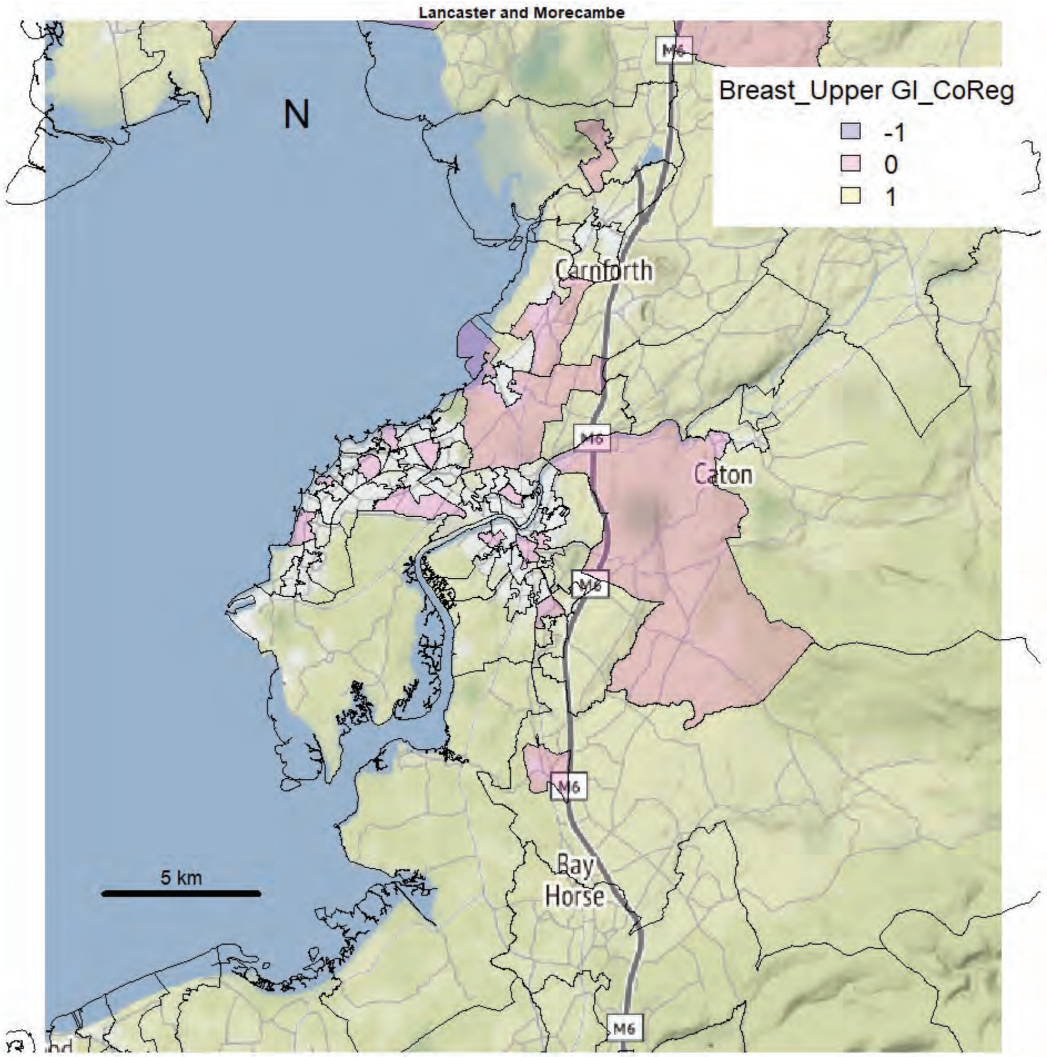
Lancaster and Morecambe
Breast_Upper GI_CoReg
-1
0
1
N
Carnforth
Caton
Bay
Horse
M6
5 km

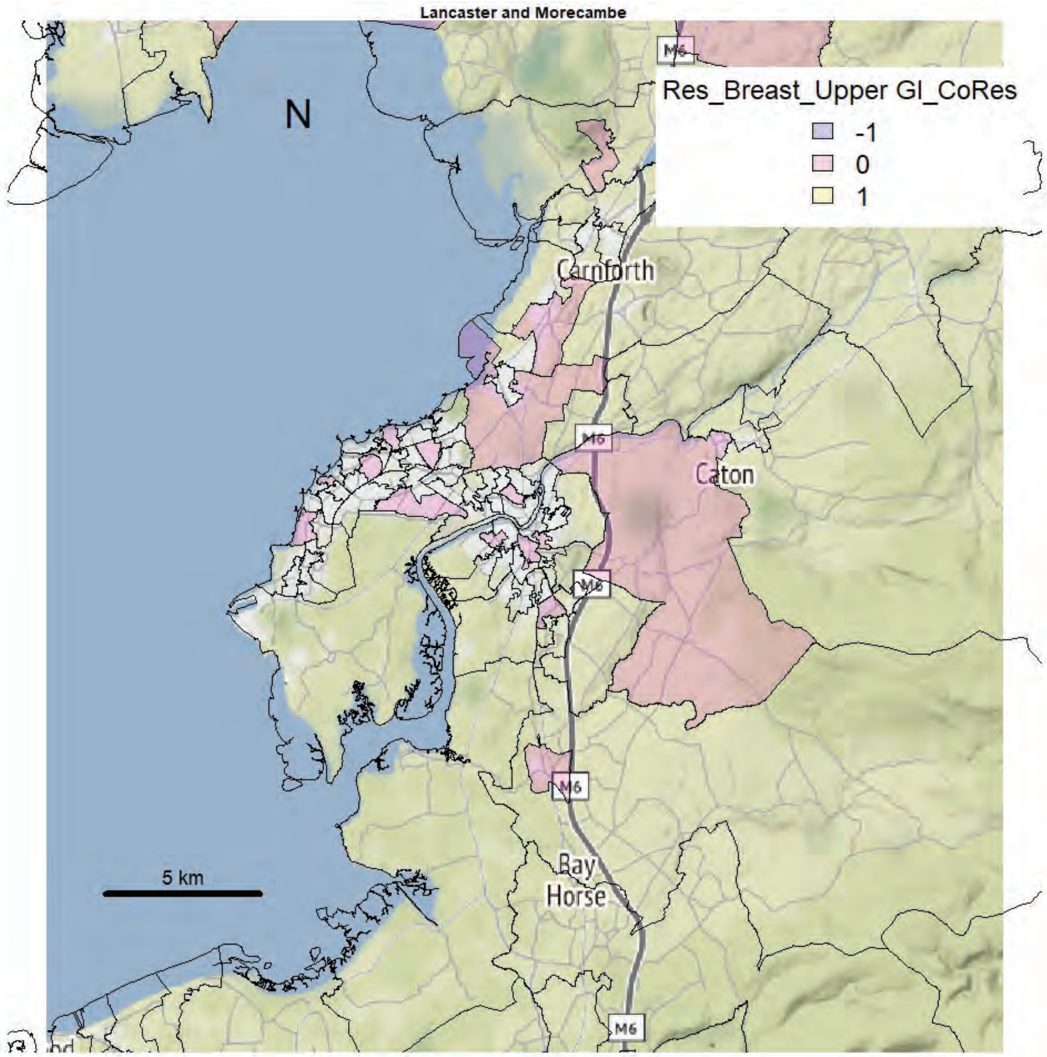
Lancaster and Morecambe
Res_Breast_Upper GI_CoRes
-1
0
1
N
Carnforth
Caton
Bay
Horse
M6
5 km

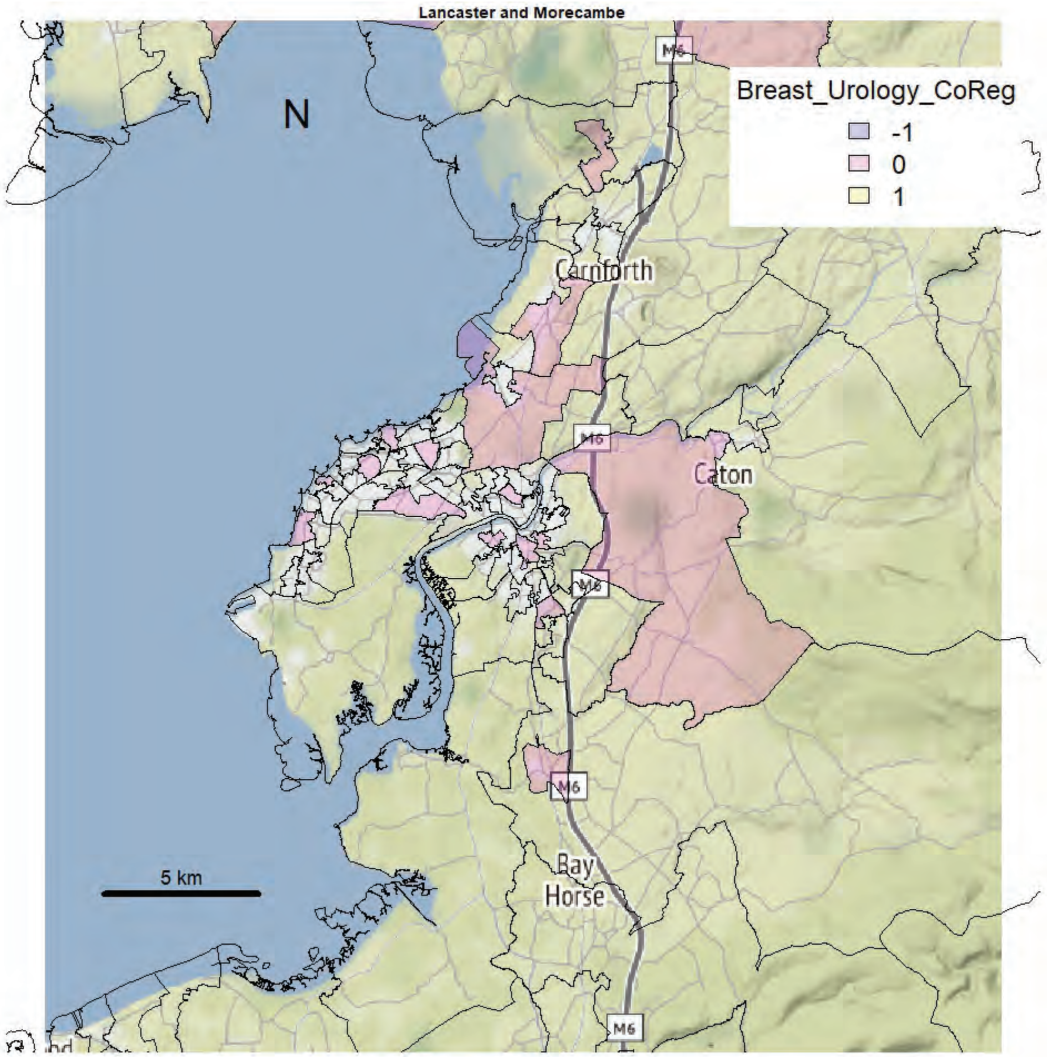
Lancaster and Morecambe
Breast_Urology_CoReg
-1
0
1
N
Carnforth
Caton
Bay
Horse
M6
5 km

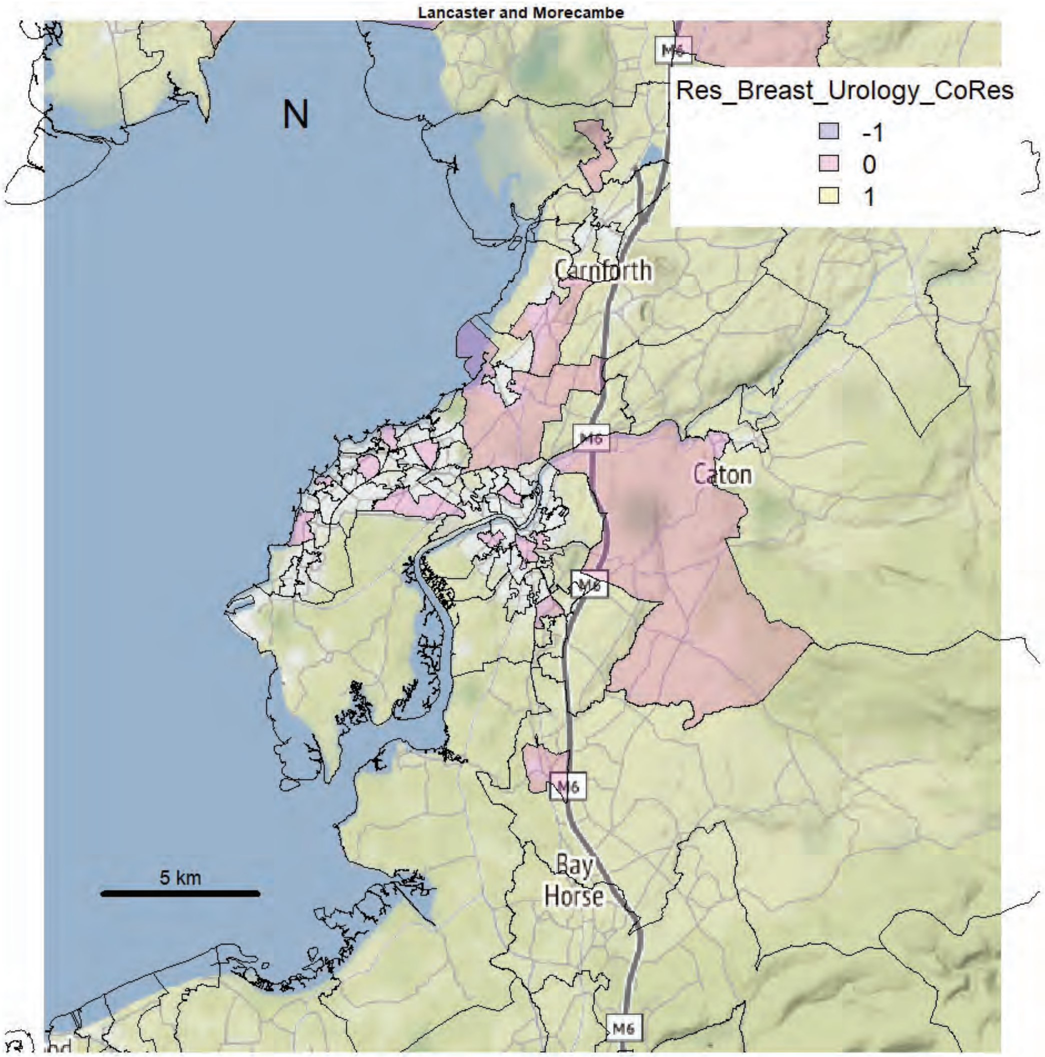
Lancaster and Morecambe
Res_Breast_Urology_CoRes
-1
0
1
N
Carnforth
Caton
Bay
Horse
M6
5 km

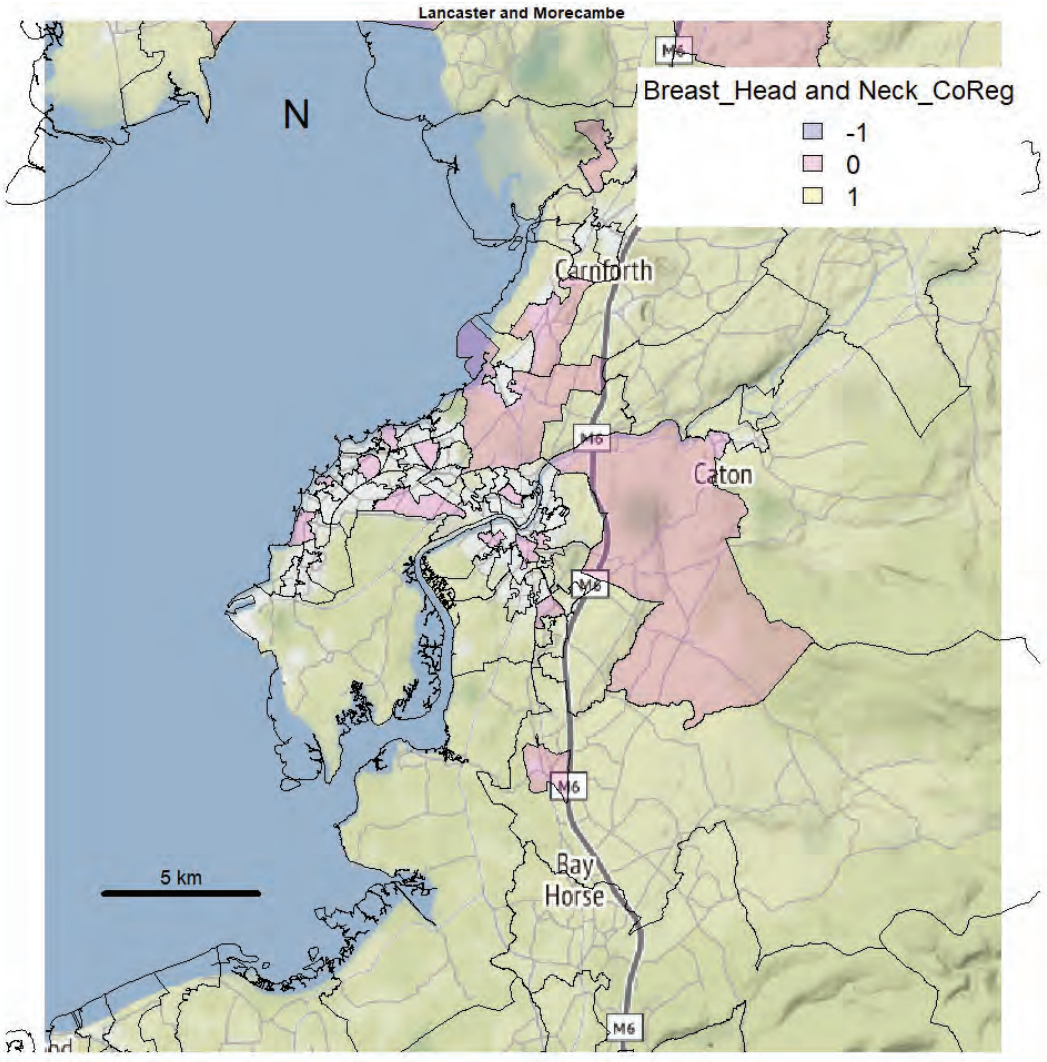
Lancaster and Morecambe
Breast_Head and Neck_CoReg
-1
0
1
N
Carnforth
Caton
Bay Horse
M6
5 km

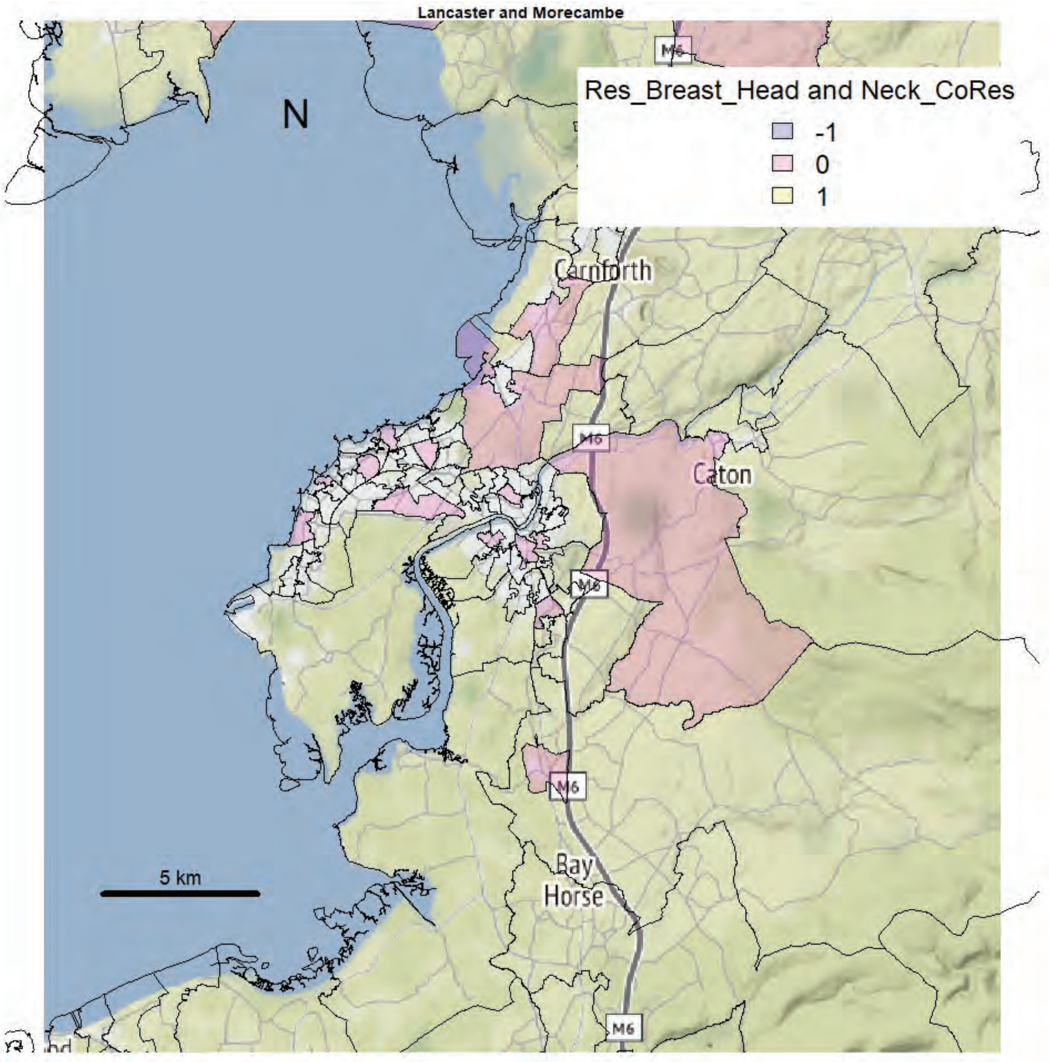
Lancaster and Morecambe
Res_Breast_Head and Neck_CoRes
-1
0
1
N
Carnforth
Caton
Bay Horse
M6
5 km

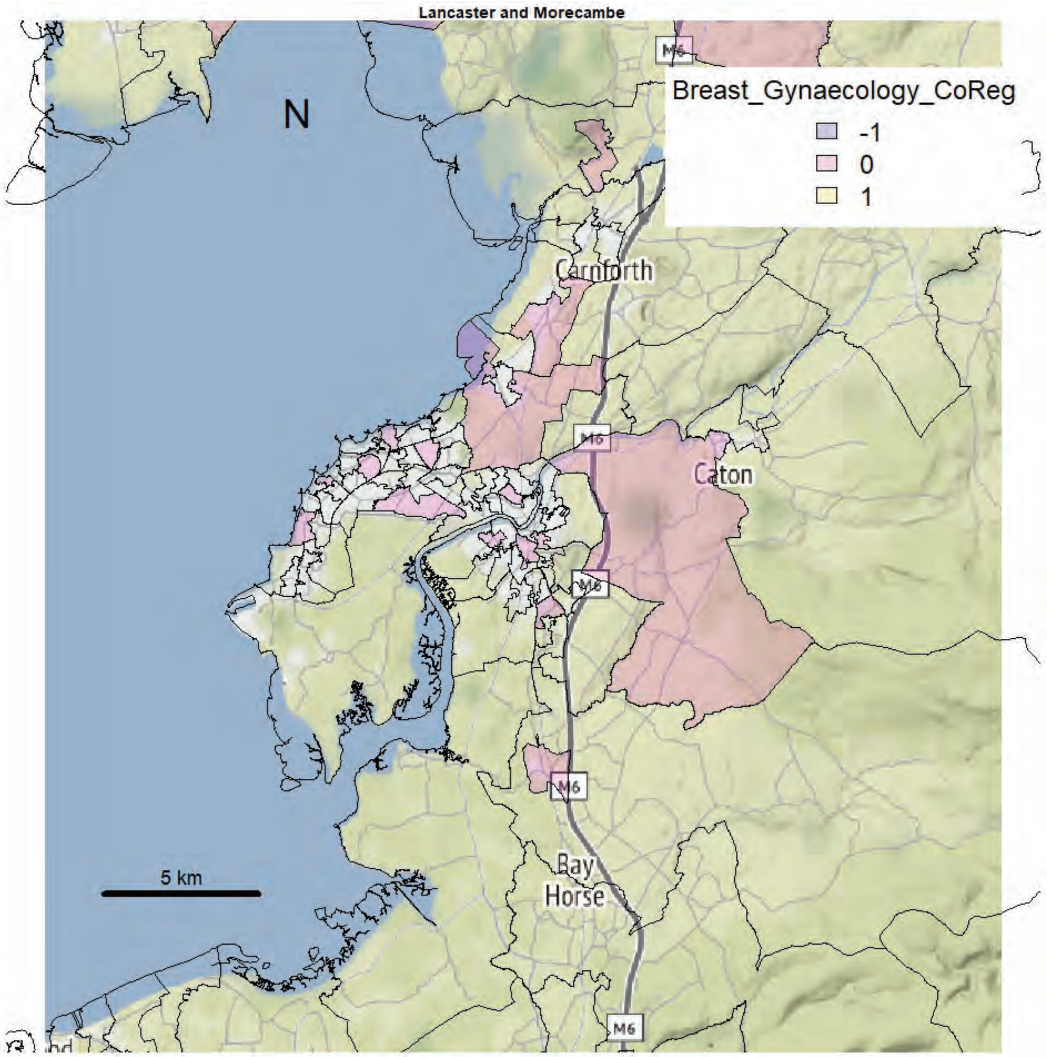
Lancaster and Morecambe
Breast_Gynaecology_CoReg
-1
0
1
N
Carnforth
Caton
Bay
Horse
M6
5 km

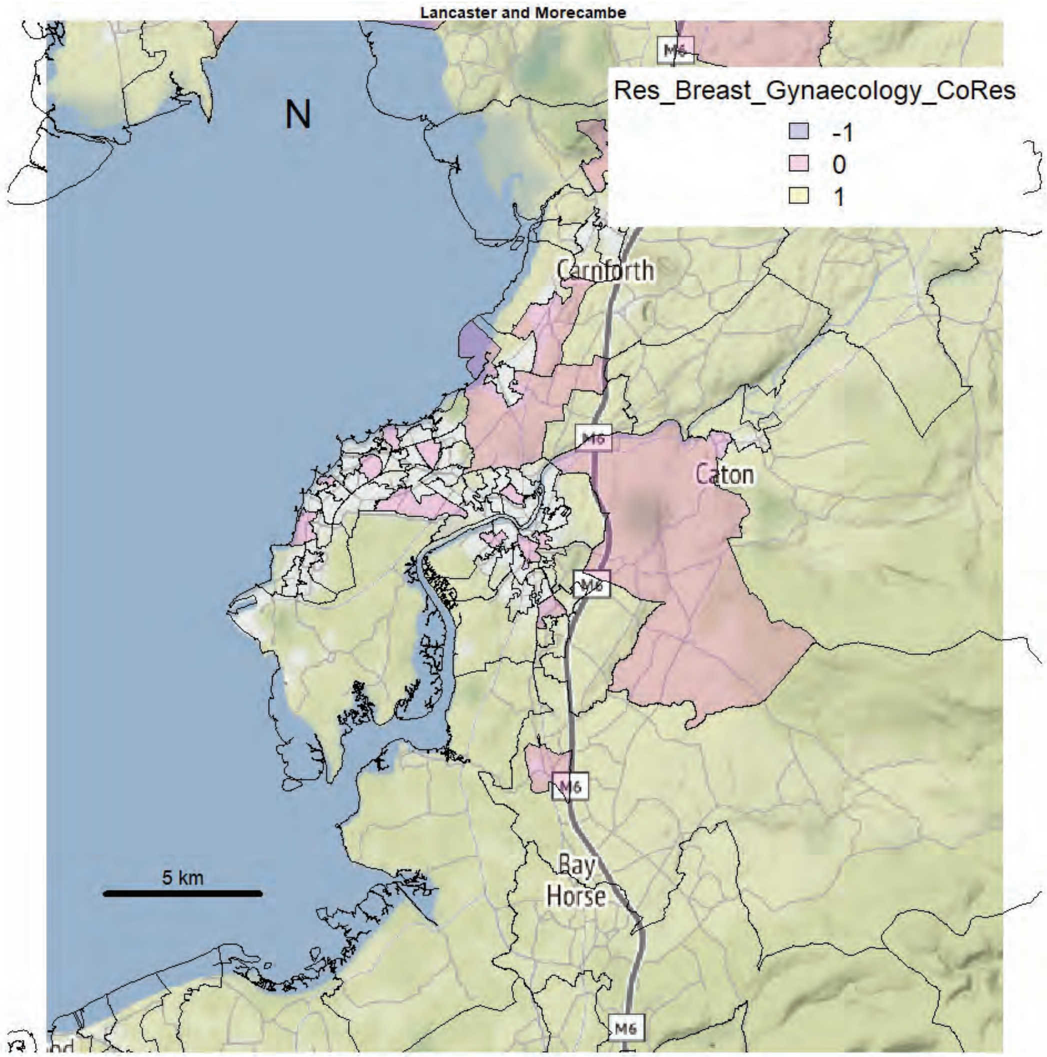
Lancaster and Morecambe
Res_Breast_Gynaecology_CoRes
-1
0
1
N
Carnforth
Caton
Bay
Horse
M6
5 km

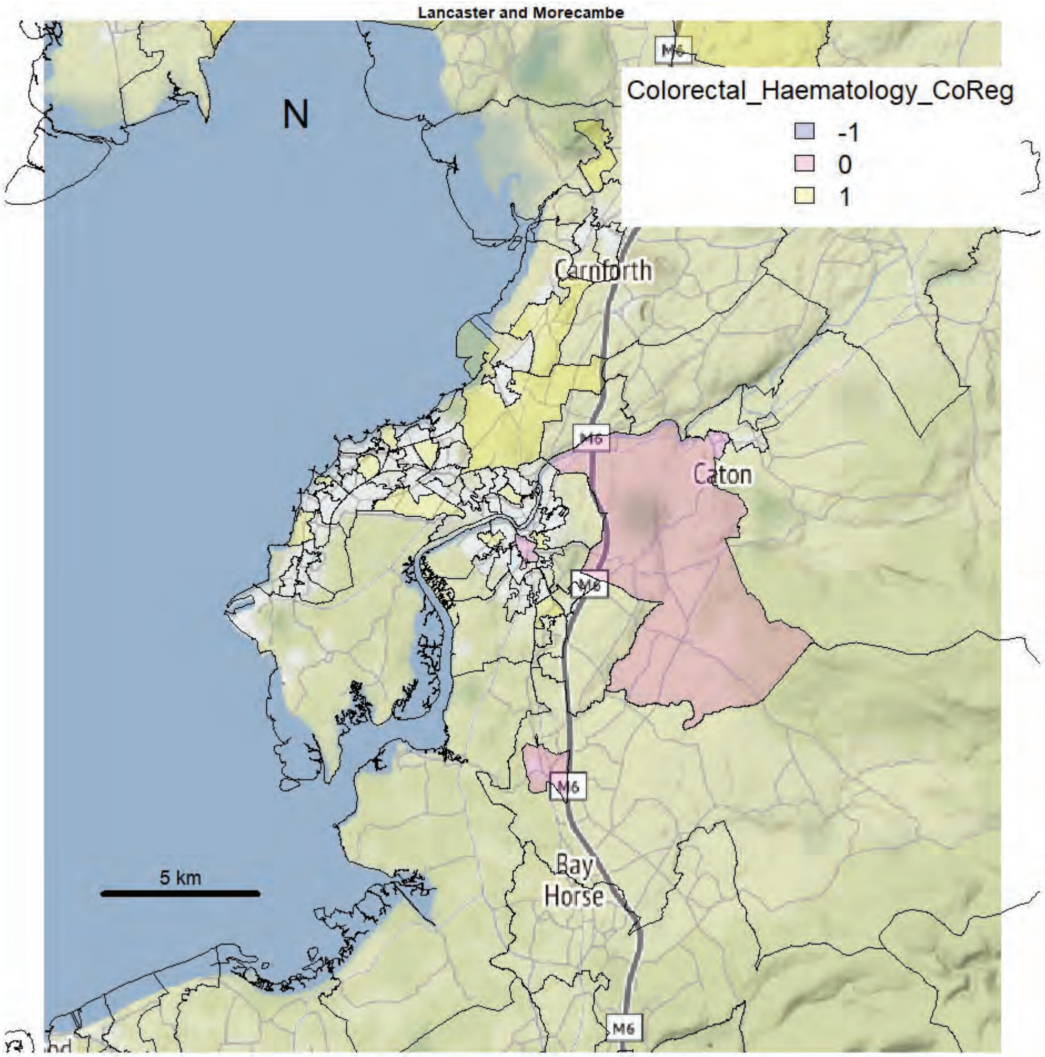
Lancaster and Morecambe
Colorectal_Haematology_CoReg
-1
0
1
N
Carnforth
Caton
Bay
Horse
M6
5 km
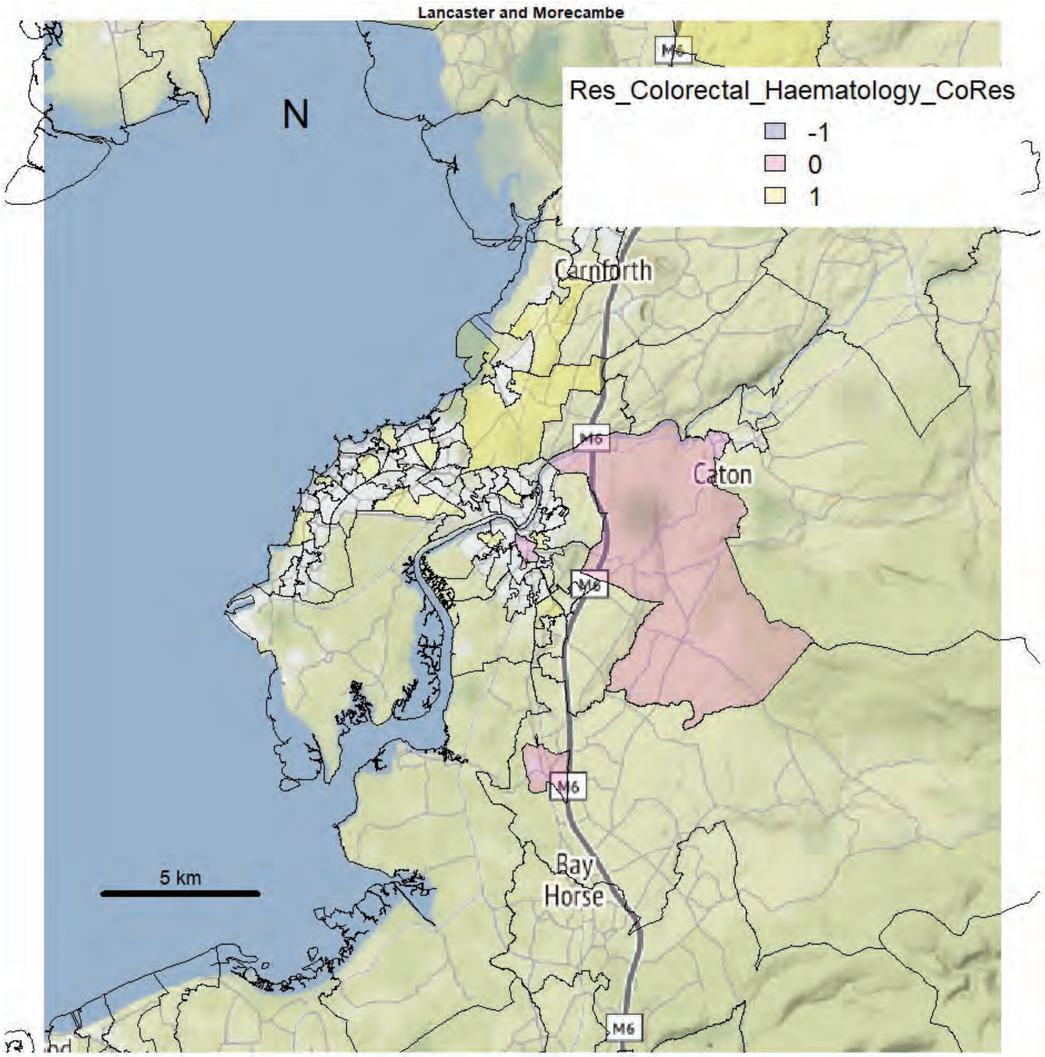
Lancaster and Morecambe
Res_Colorectal_Haematology_CoRes
-1
0
1
N
Carnforth
Caton
Bay
Horse
M6
5 km

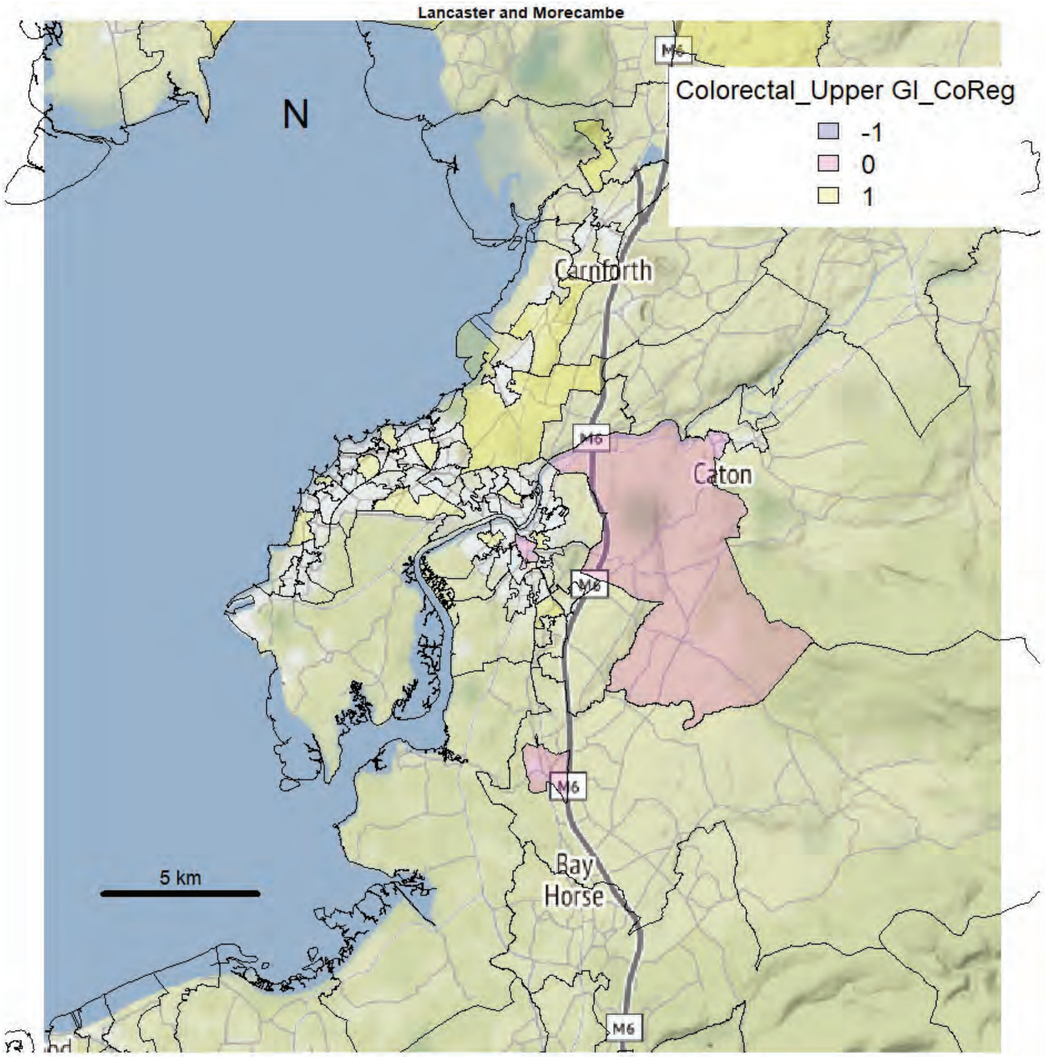
Lancaster and Morecambe
N
Colorectal_Upper GI_CoReg
-1
0
1
Carnforth
Caton
Bay
Horse
M6
5 km

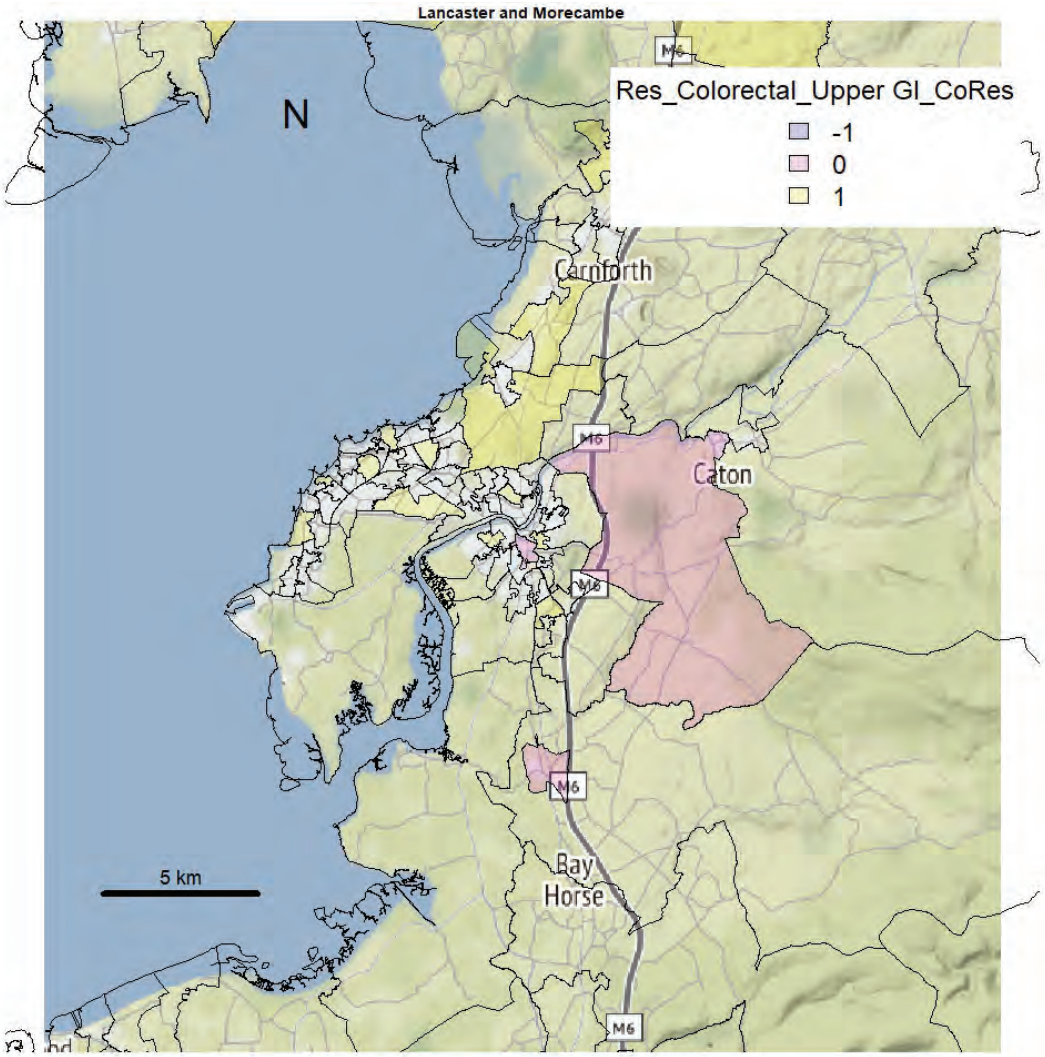
Lancaster and Morecambe
N
Res_Colorectal_Upper GI_CoRes
-1
0
1
Carnforth
Caton
Bay
Horse
M6
5 km

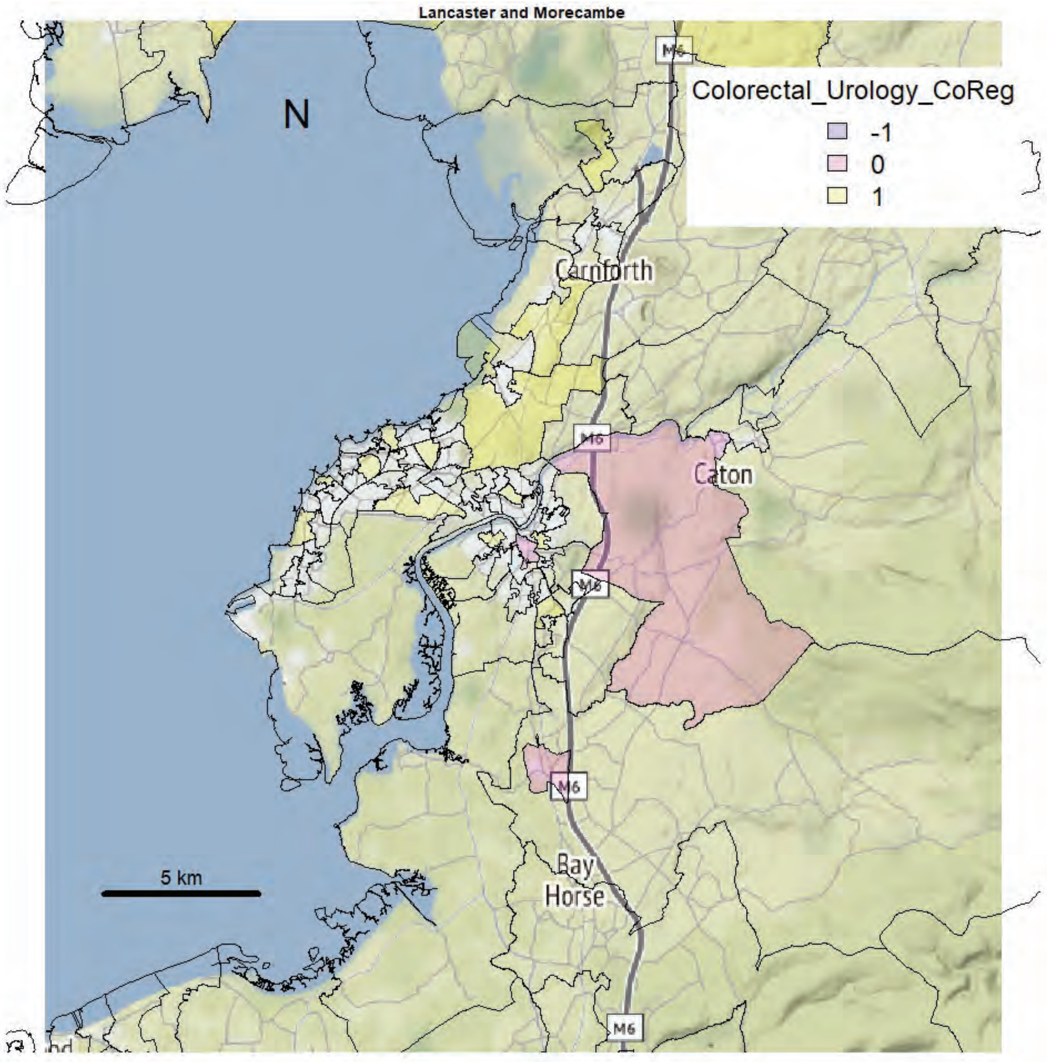
Lancaster and Morecambe
Colorectal_Urology_CoReg
-1
0
1
N
Carnforth
Caton
Bay
Horse
M6
5 km

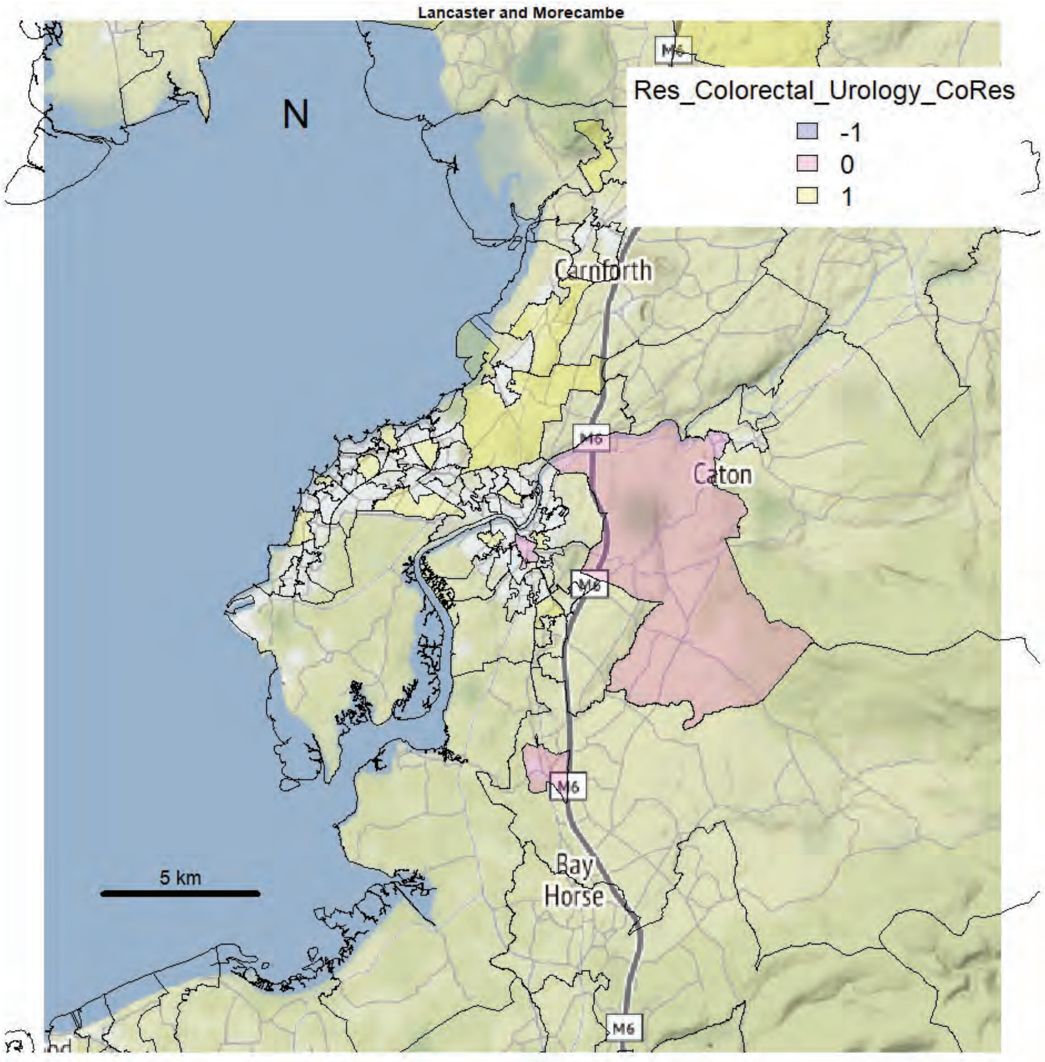
Lancaster and Morecambe
Res_Colorectal_Urology_CoRes
-1
0
1
N
Carnforth
Caton
Bay
Horse
M6
5 km

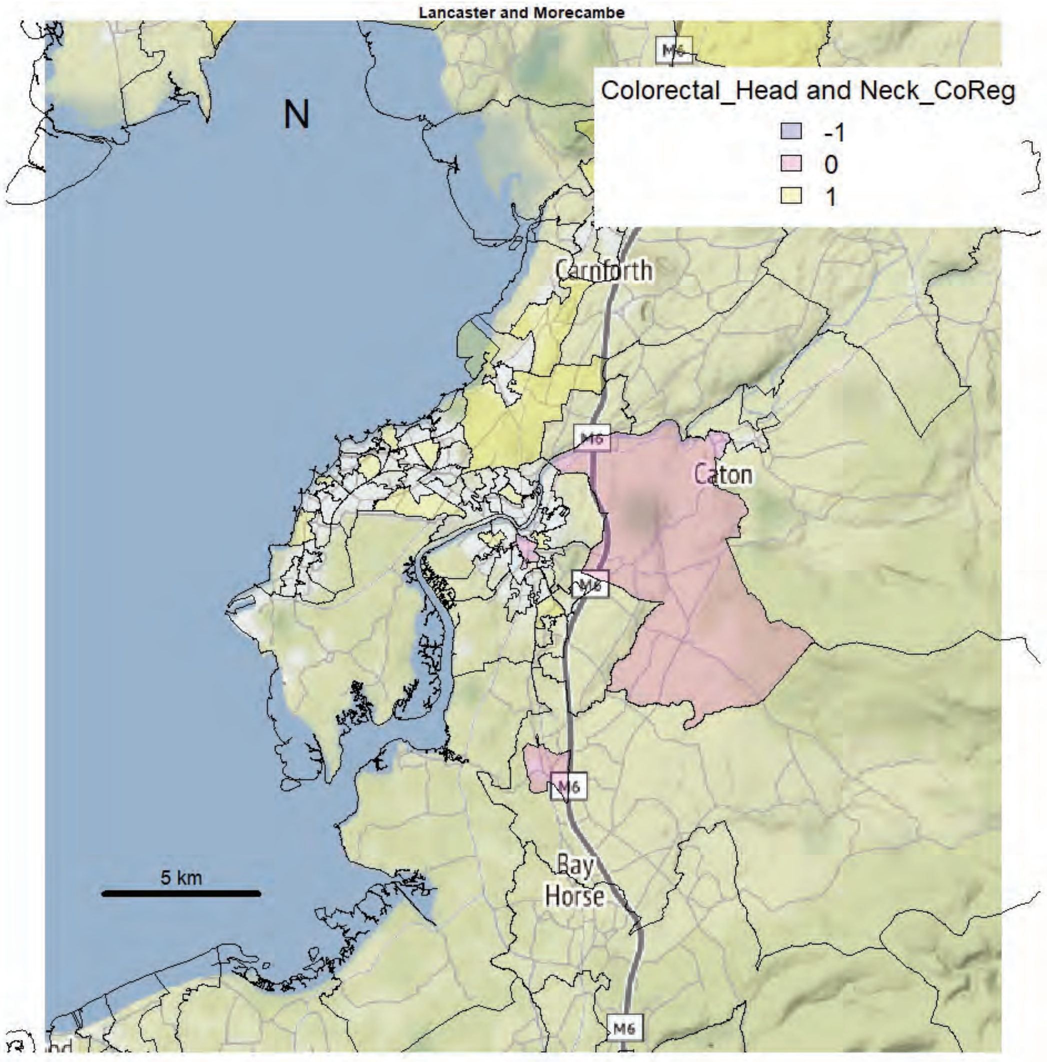
Lancaster and Morecambe
Colorectal_Head and Neck_CoReg
-1
0
1
N
Carnforth
Caton
M6
Bay
Horse
5 km

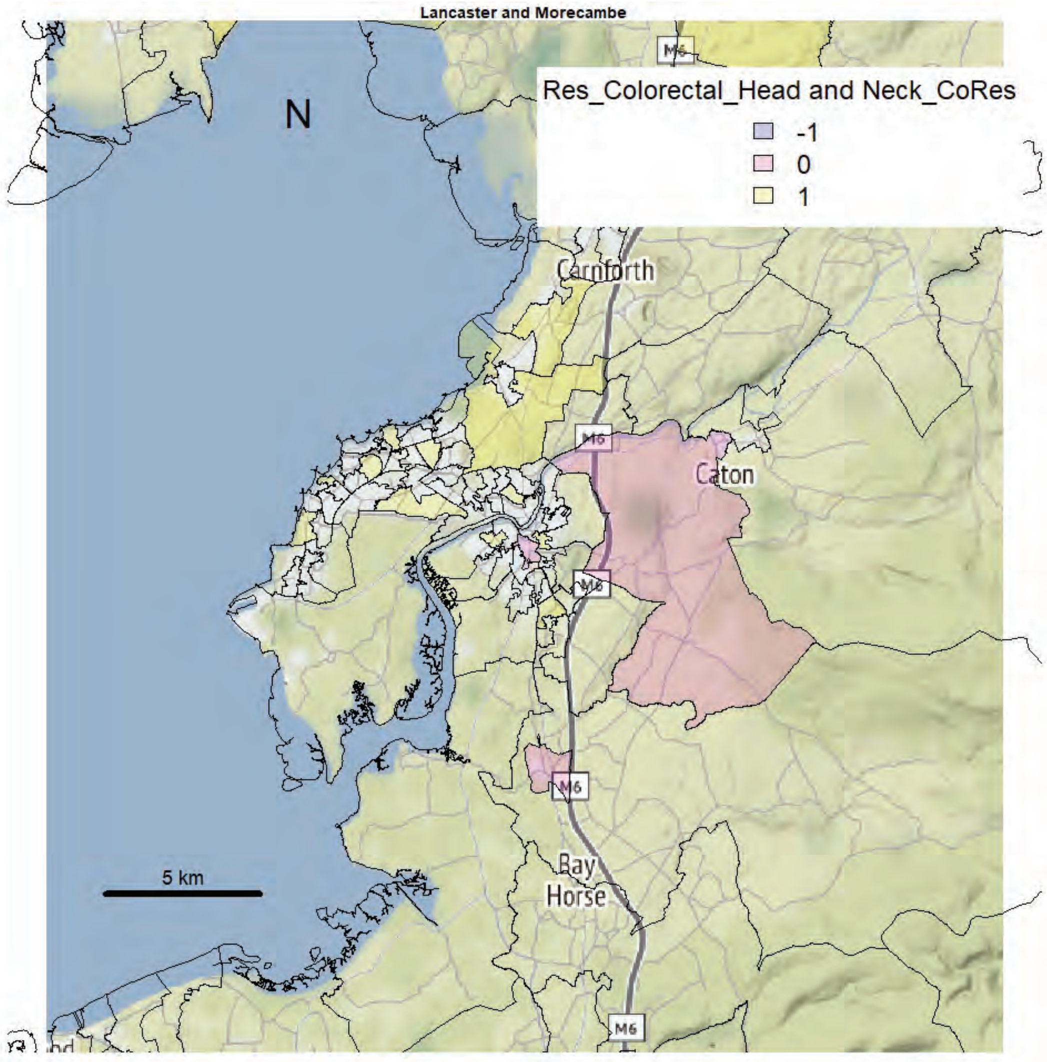
Lancaster and Morecambe
Res_Colorectal_Head and Neck_CoRes
-1
0
1
N
Carnforth
Caton
M6
Bay
Horse
5 km

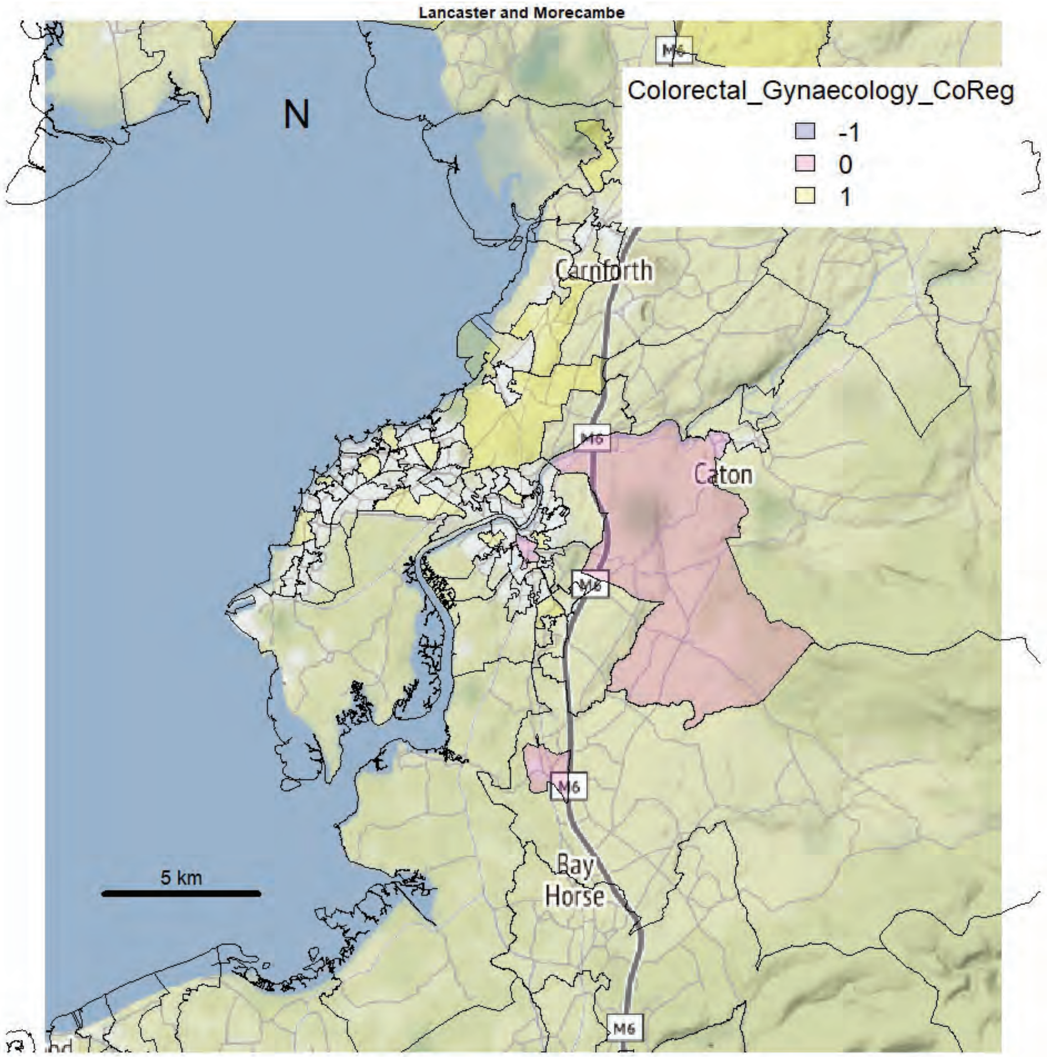
Lancaster and Morecambe
Colorectal_Gynaecology_CoReg
-1
0
1
N
Carnforth
Caton
Bay
Horse
M6
5 km

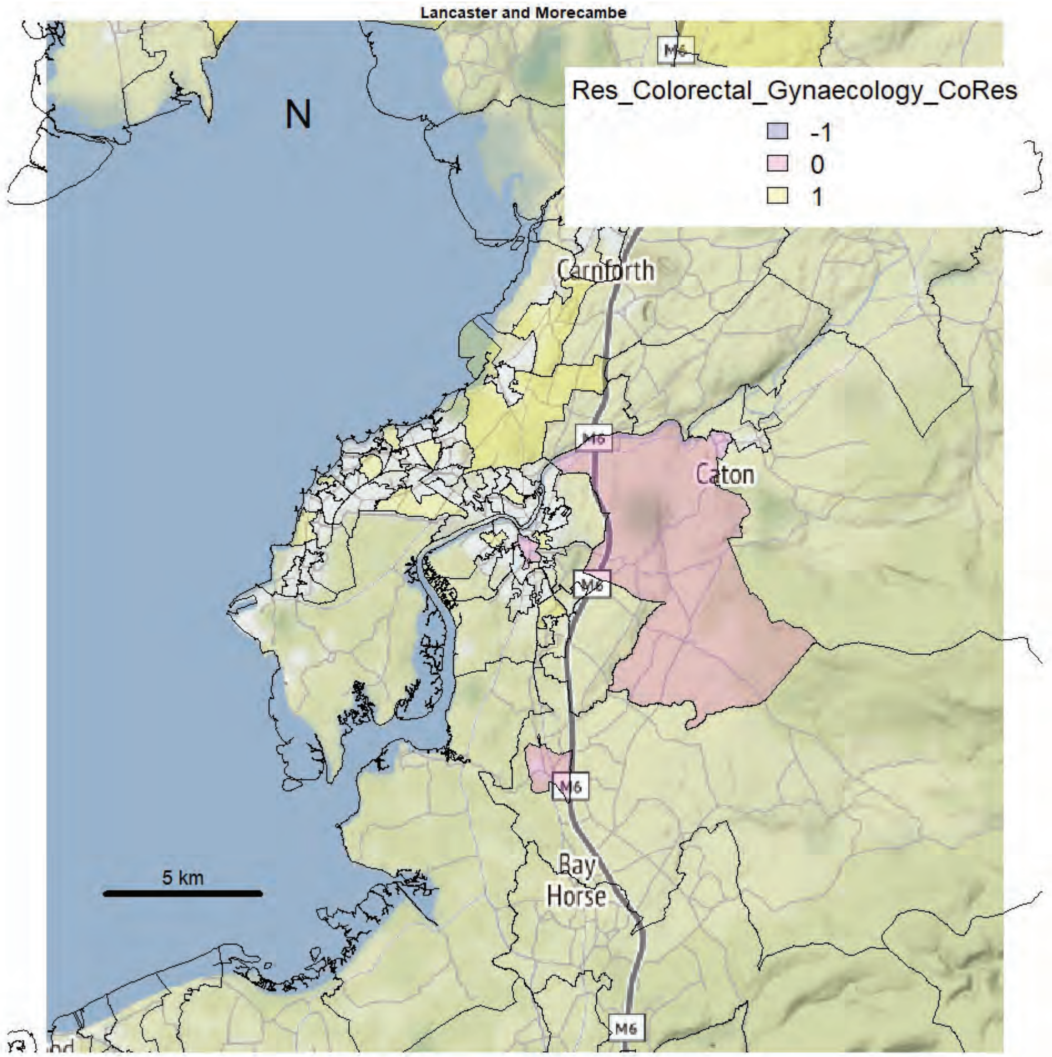
Lancaster and Morecambe
Res_Colorectal_Gynaecology_CoRes
-1
0
1
N
Carnforth
Caton
Bay
Horse
M6
5 km

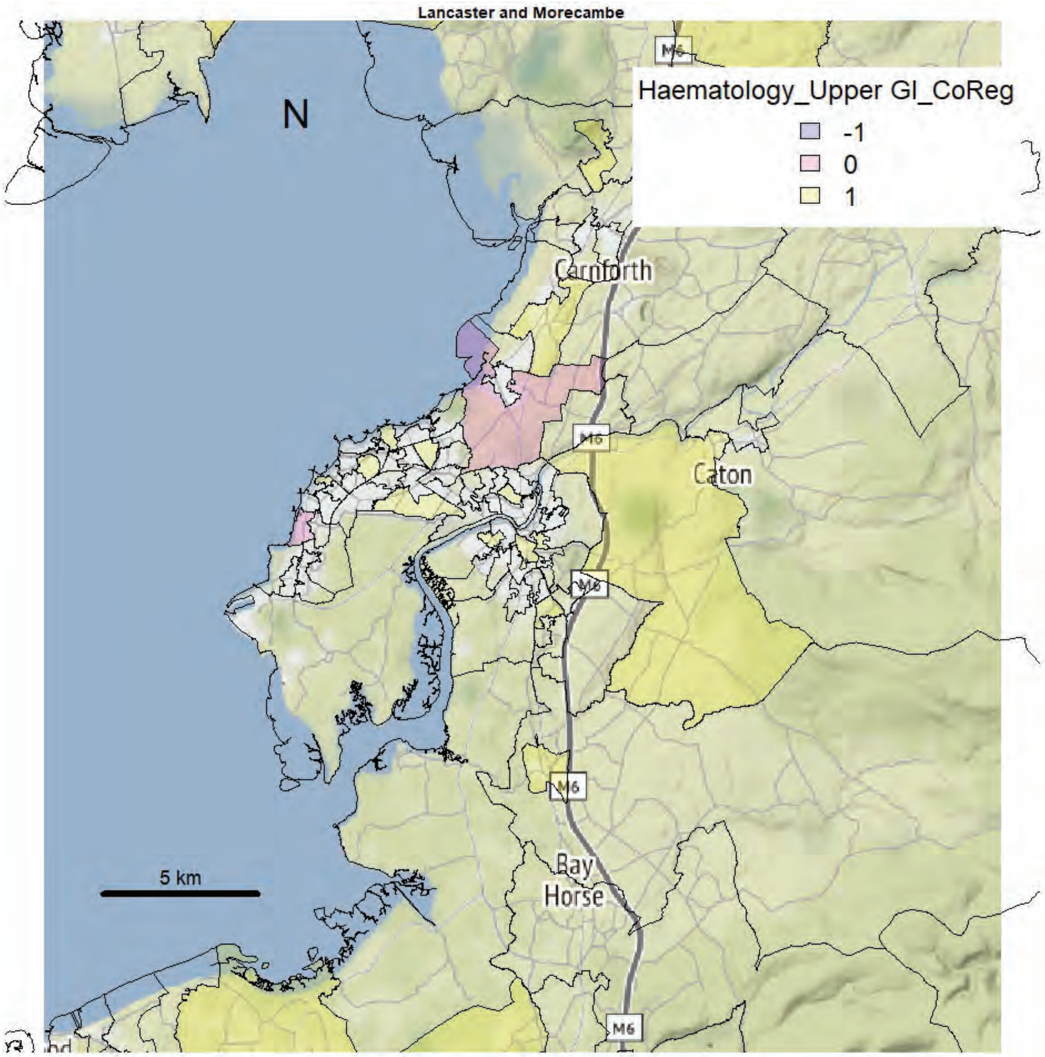
Lancaster and Morecambe
Haematology_Upper GI_CoReg
-1
0
1
N
Carnforth
Caton
Bay Horse
M6
5 km

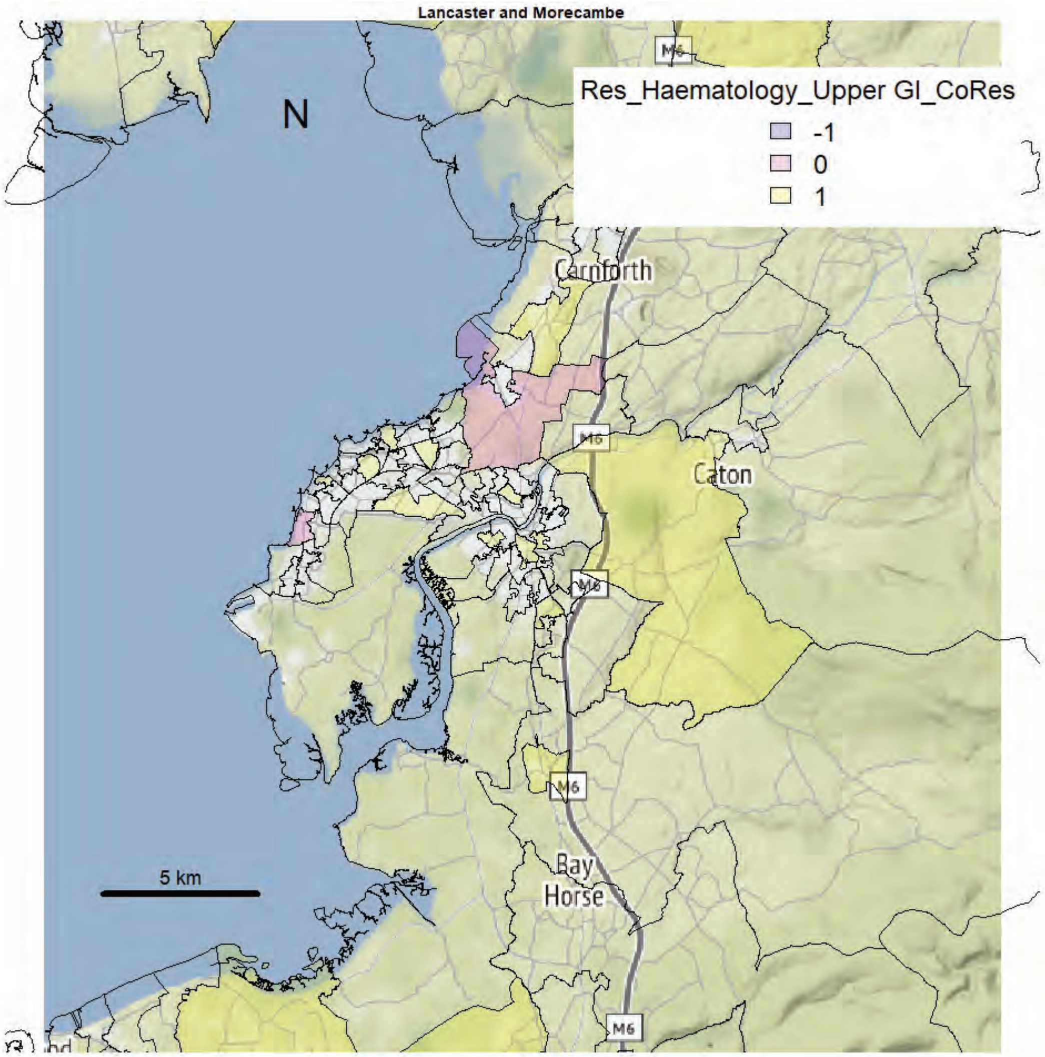
Lancaster and Morecambe
Res_Haematology_Upper GI_CoRes
-1
0
1
N
Carnforth
Caton
Bay Horse
M6
5 km

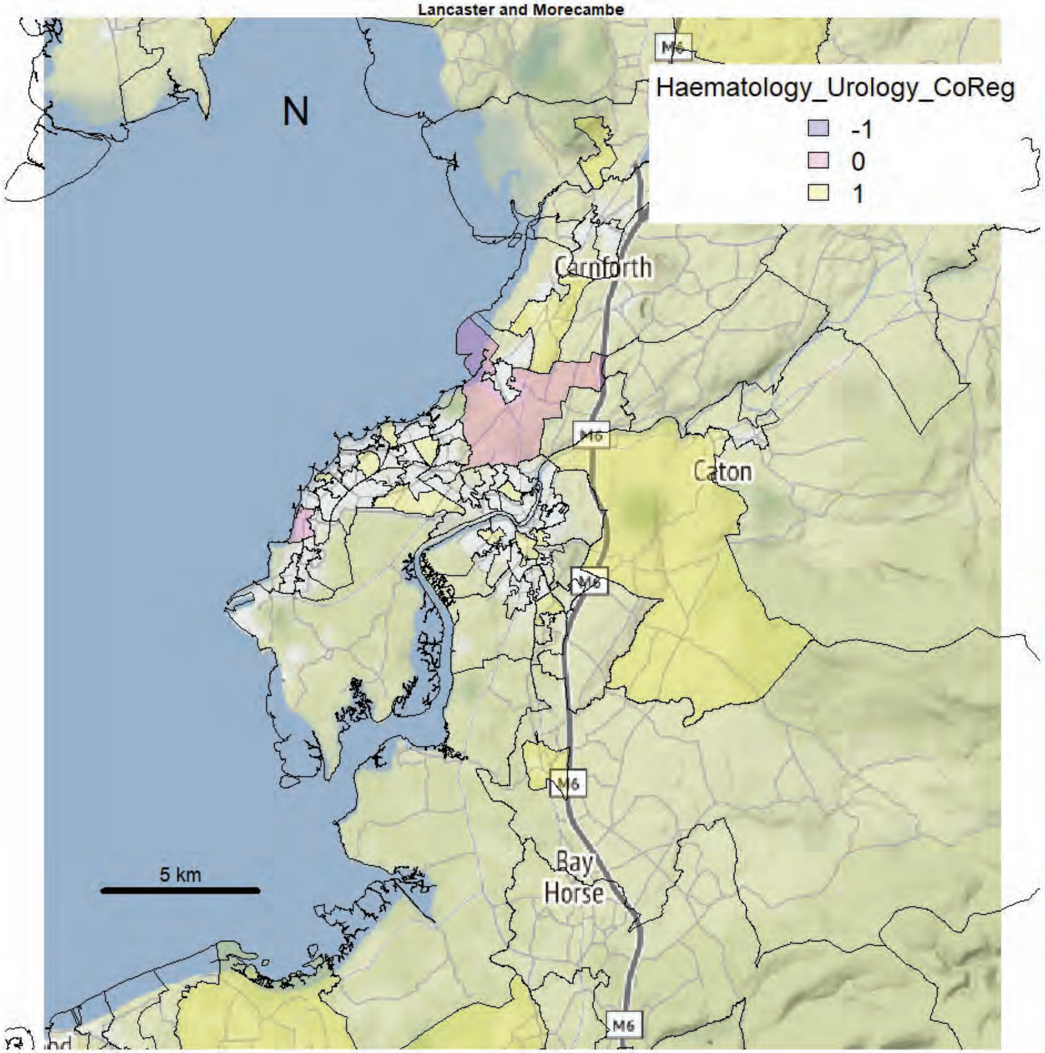
Lancaster and Morecambe
N
Haematology_Urology_CoReg
-1
0
1
Carnforth
Caton
Bay Horse
M6
5 km

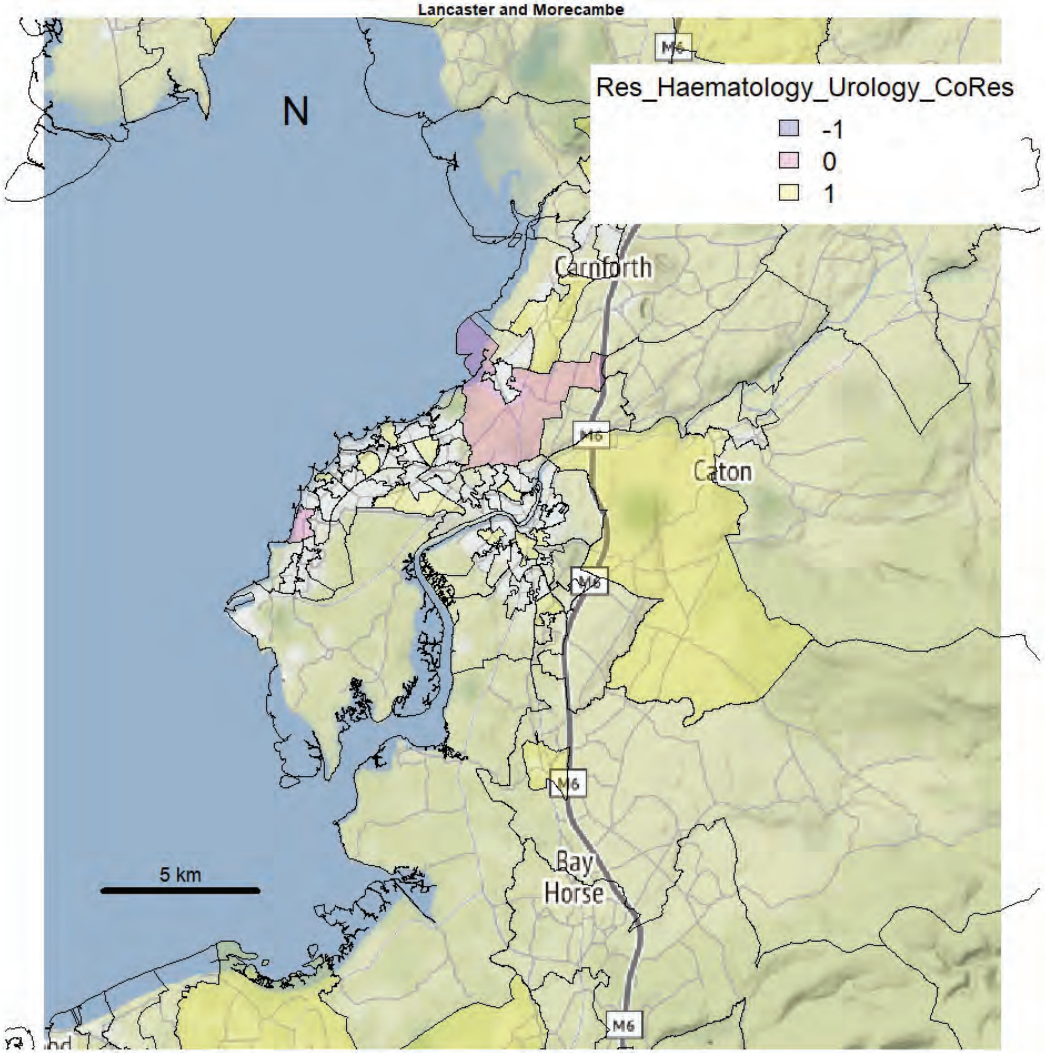
Lancaster and Morecambe
N
Res_Haematology_Urology_CoRes
-1
0
1
Carnforth
Caton
Bay Horse
M6
5 km

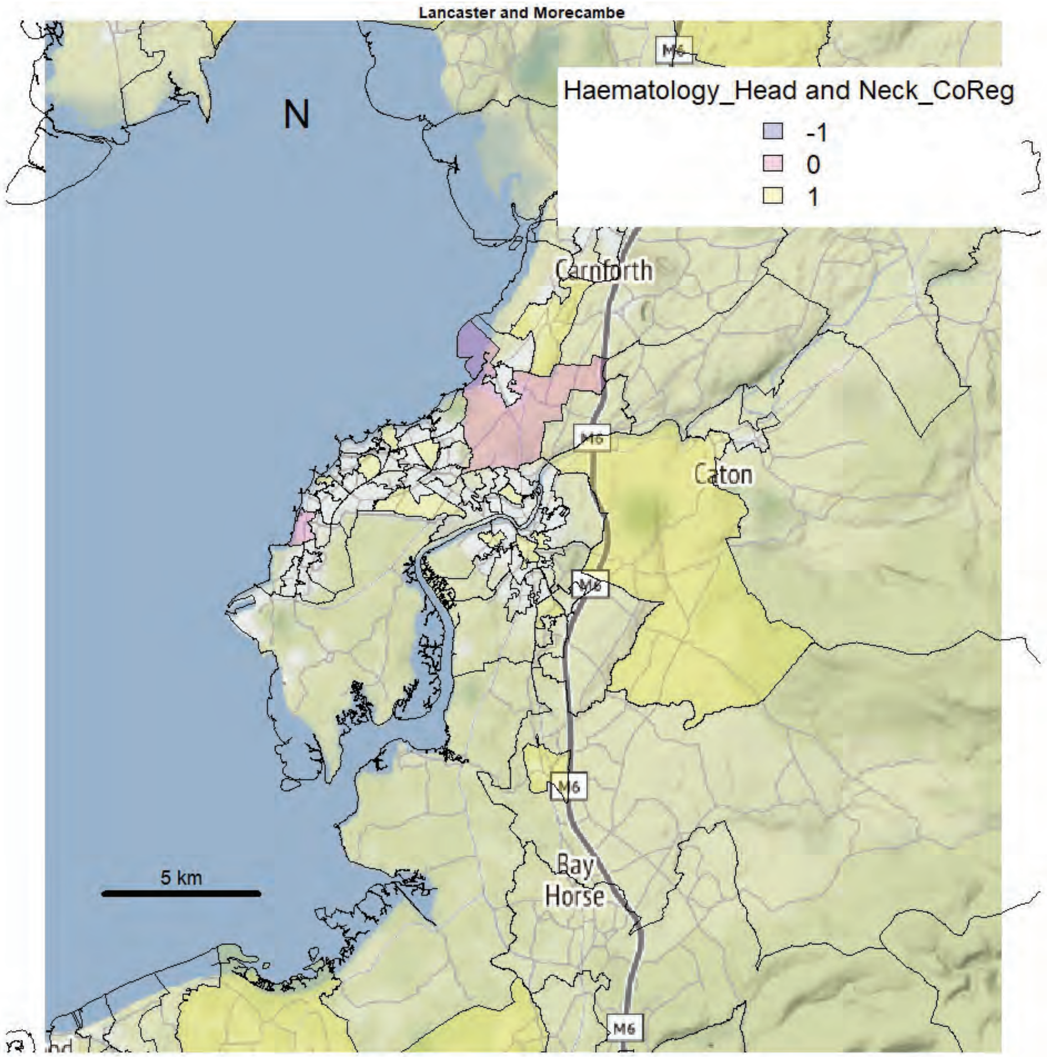
Lancaster and Morecambe
Haematology_Head and Neck_CoReg
-1
0
1
N
Carnforth
Caton
Bay
Horse
M6
5 km

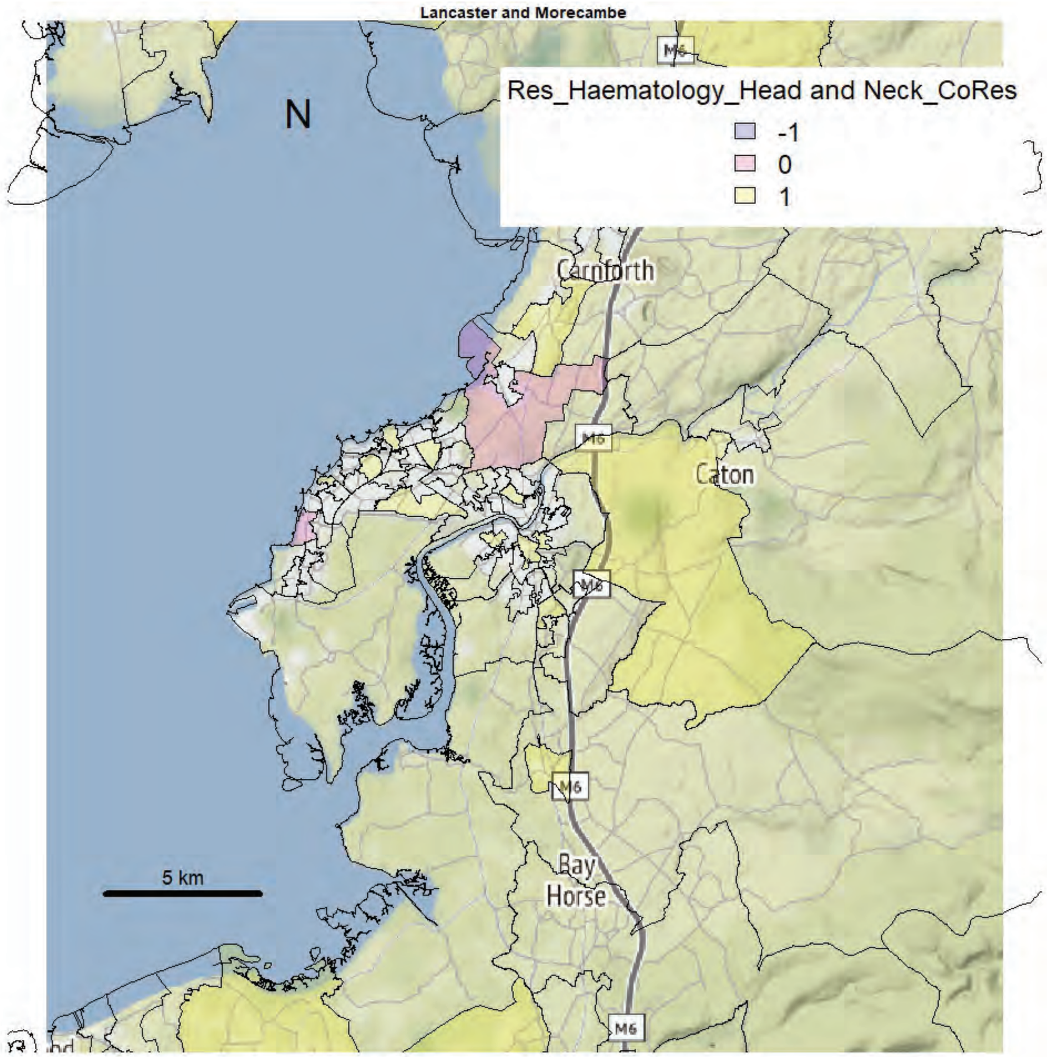
Lancaster and Morecambe
Res_Haematology_Head and Neck_CoRes
-1
0
1
N
Carnforth
Caton
Bay
Horse
M6
5 km

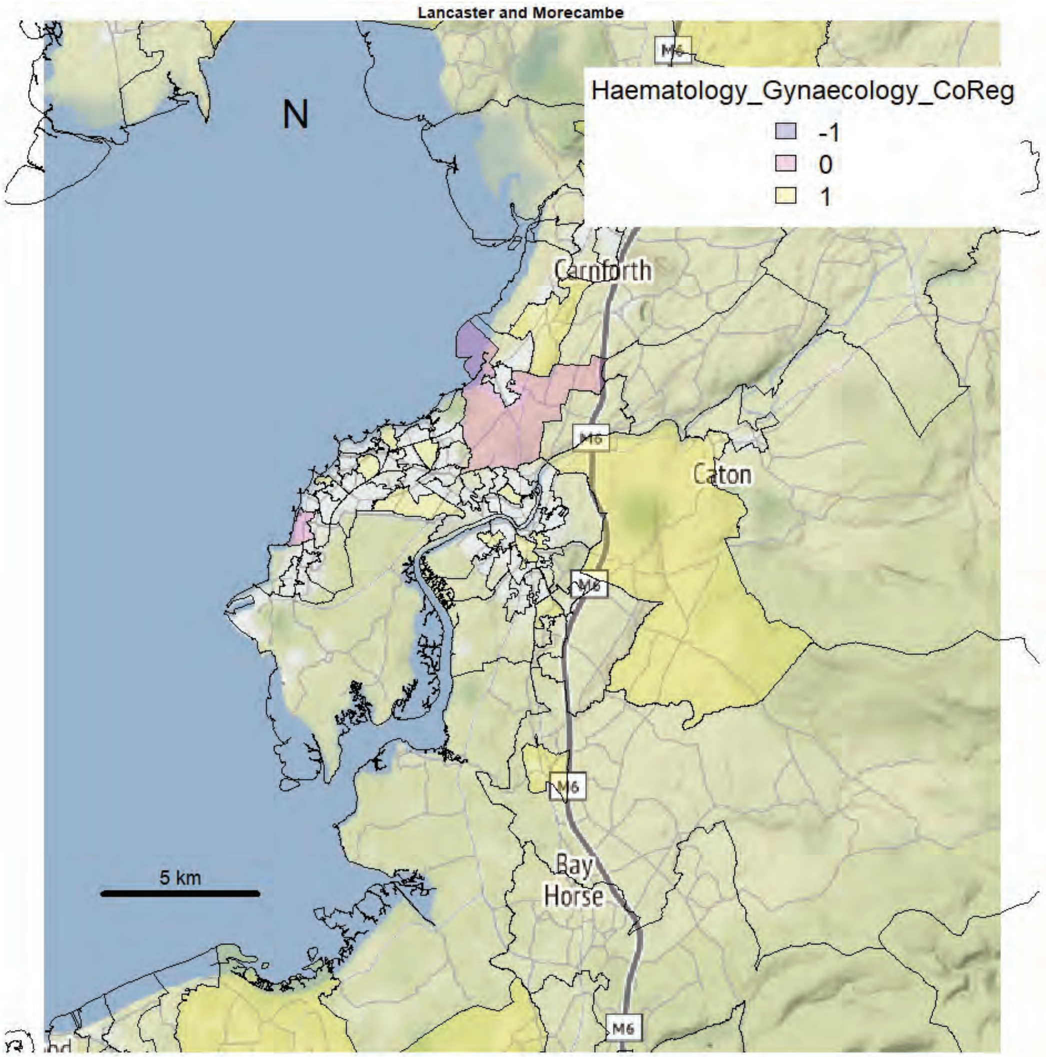
Lancaster and Morecambe
N
Haematology_Gynaecology_CoReg
-1
0
1
Carnforth
Caton
Bay
Horse
M6
5 km

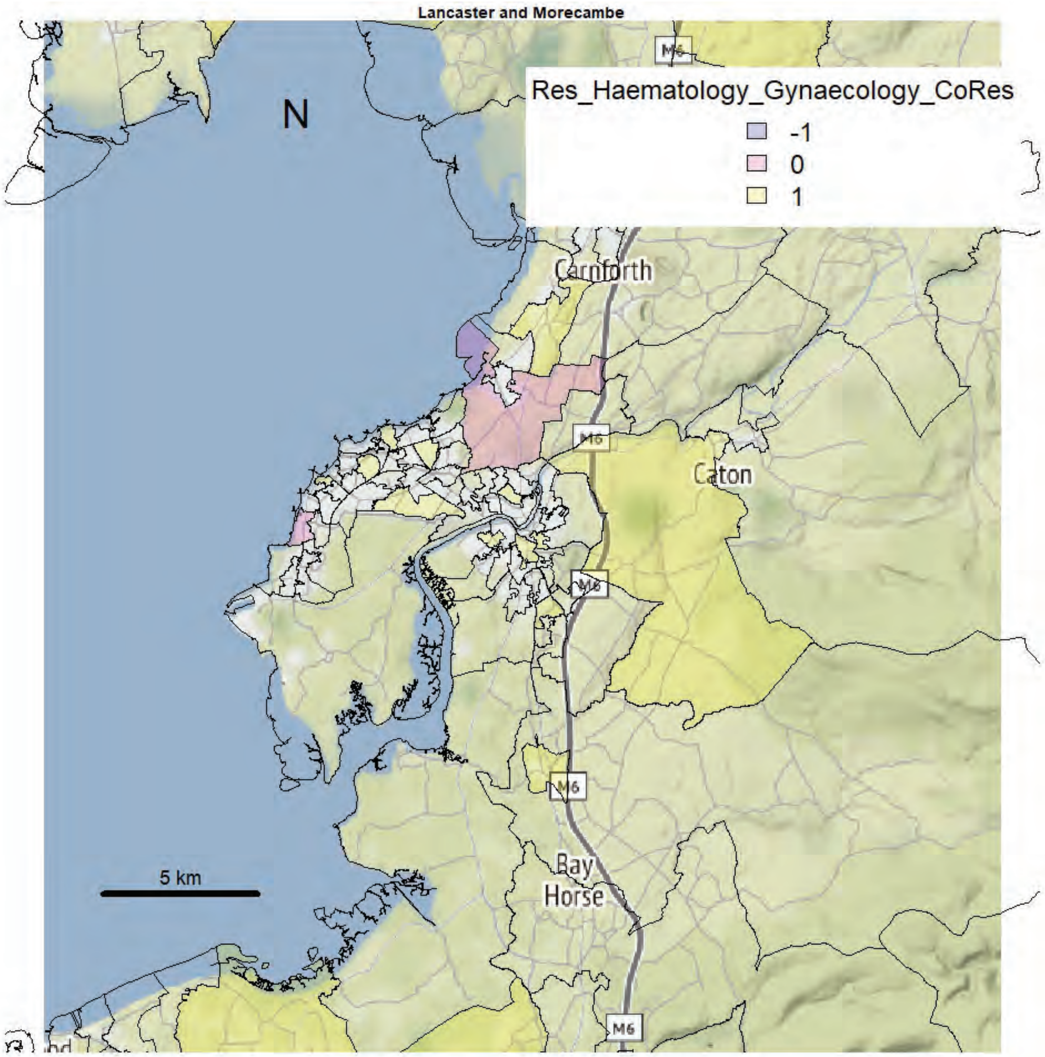
Lancaster and Morecambe
N
Res_Haematology_Gynaecology_CoRes
-1
0
1
Carnforth
Caton
Bay
Horse
M6
5 km

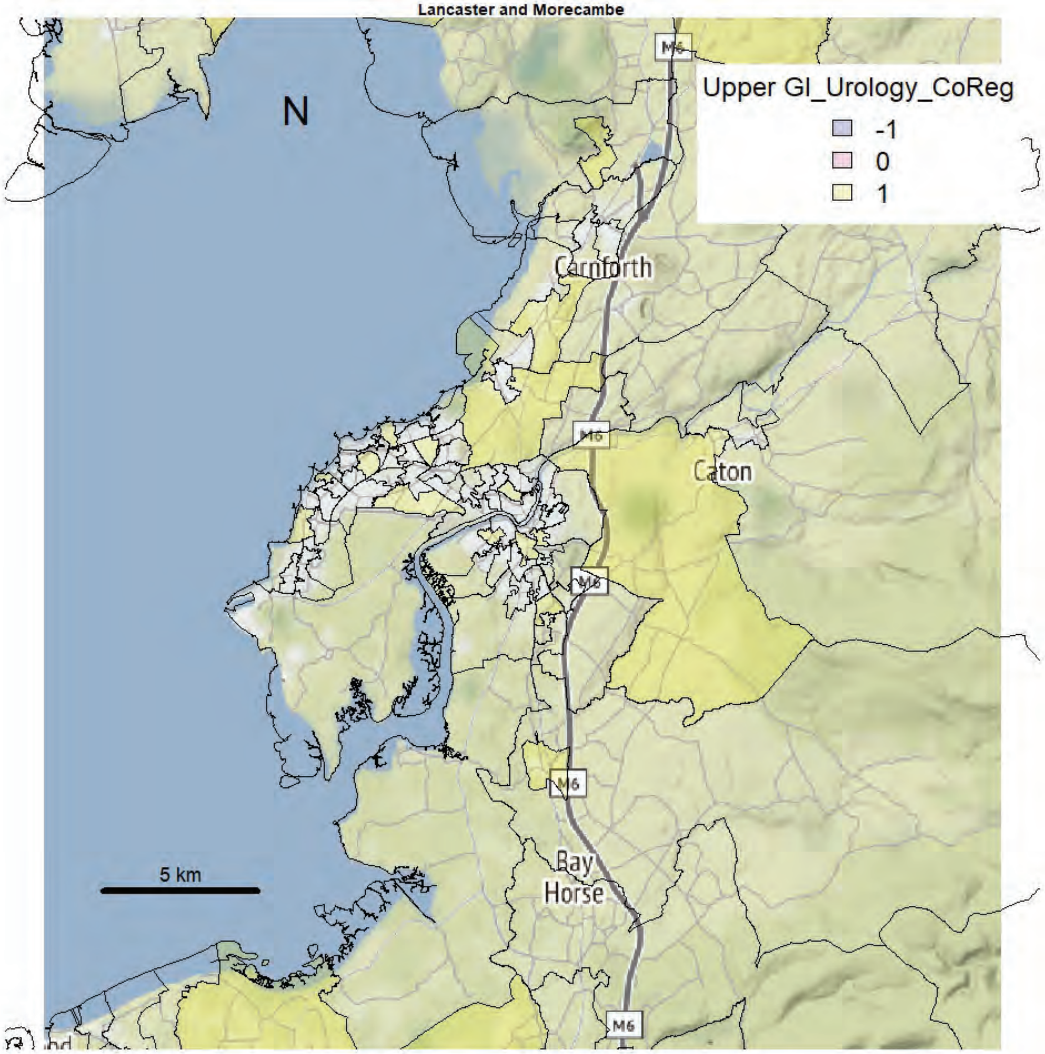

Lancaster and Morecambe
Upper GI_Urology_CoReg
-1
0
1
N
Carnforth
Caton
Bay
Horse
5 km
M6

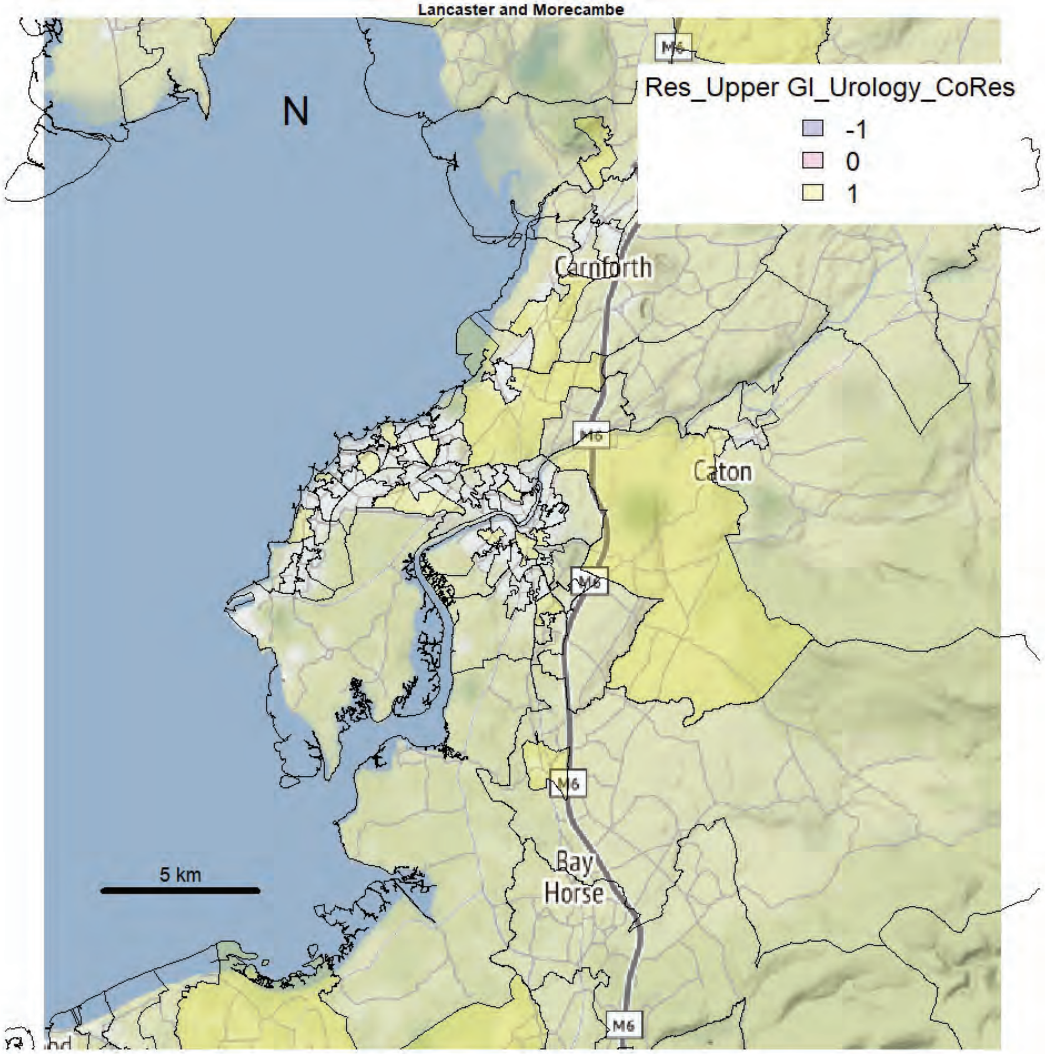

Lancaster and Morecambe
Res_Upper GI_Urology_CoRes
-1
0
1
N
Carnforth
Caton
Bay
Horse
5 km
M6

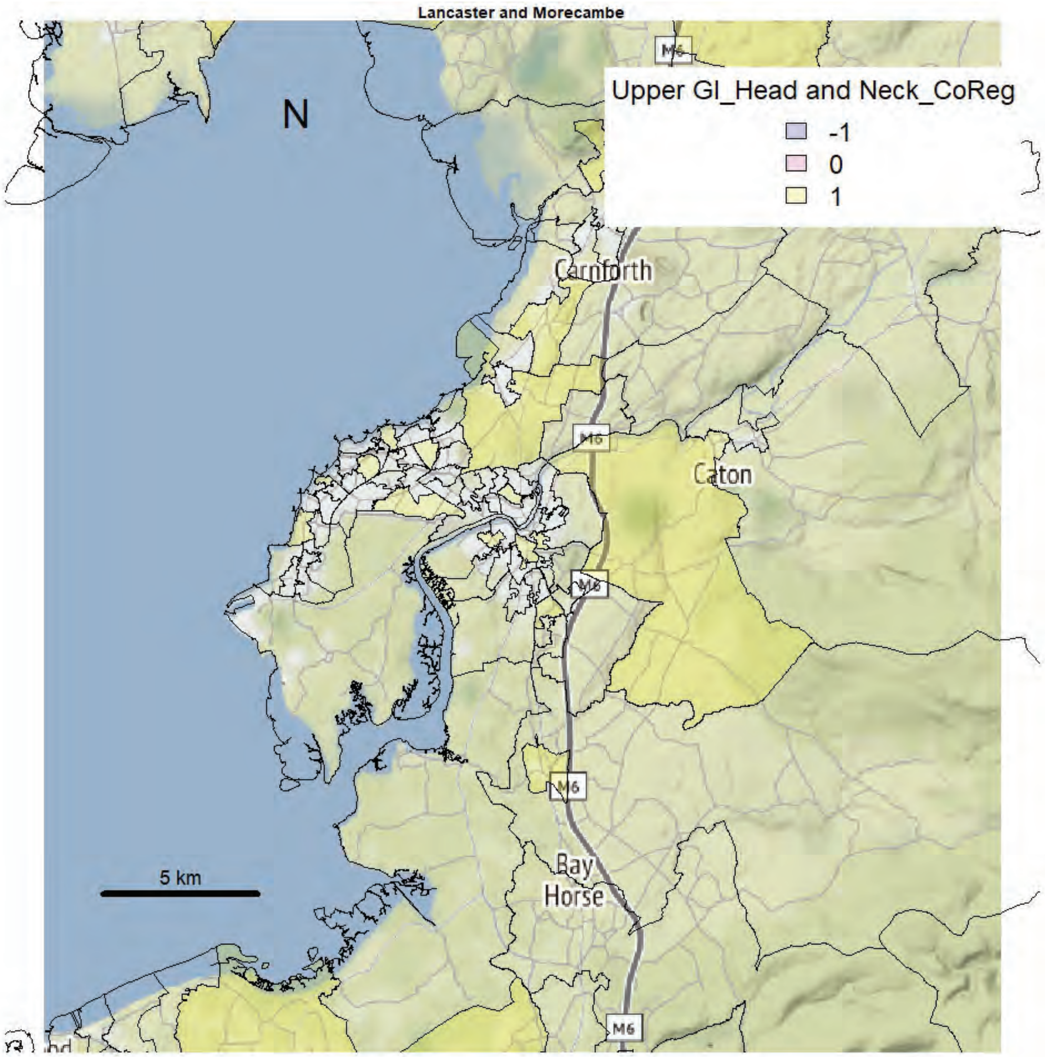
Lancaster and Morecambe
Upper GI_Head and Neck_CoReg
-1
0
1
N
Carnforth
Caton
Bay Horse
M6
5 km

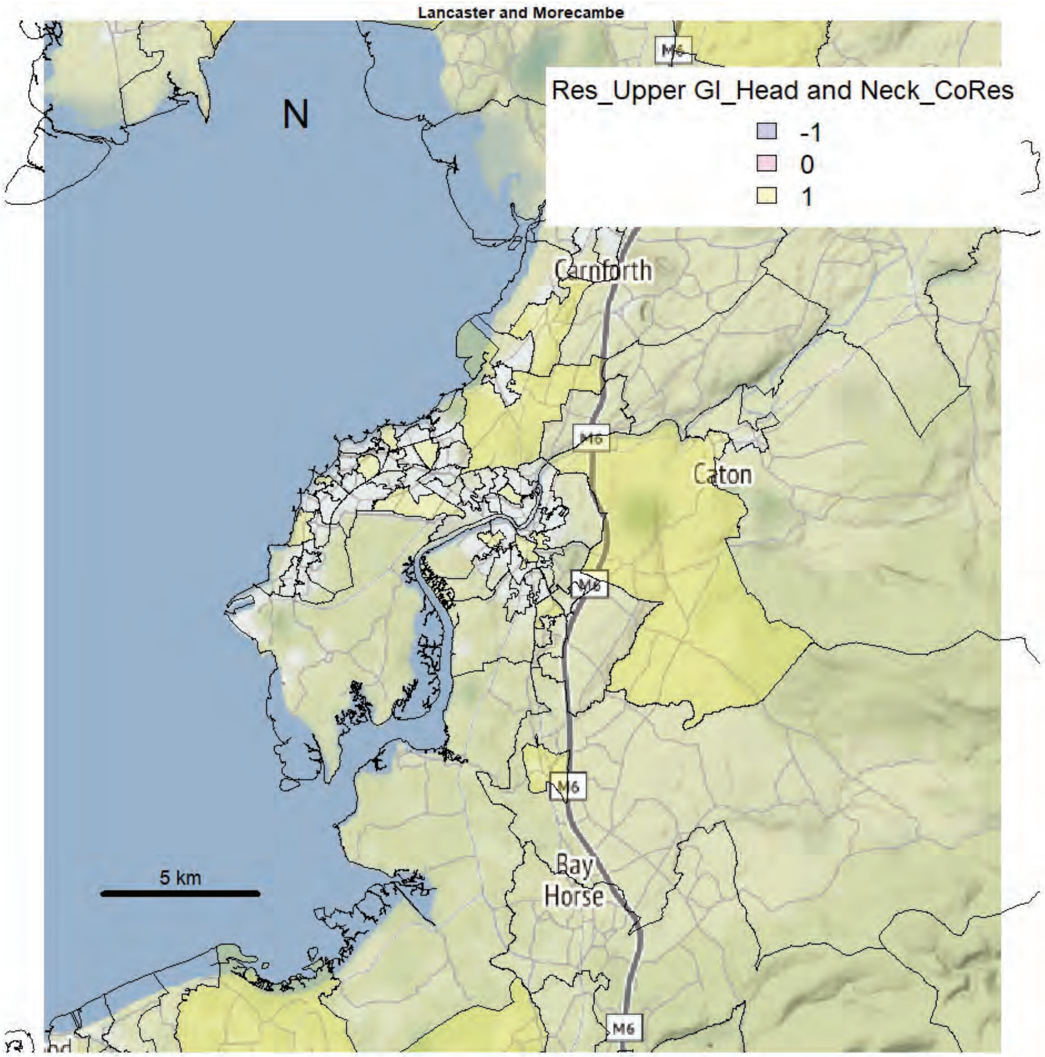
Lancaster and Morecambe
Res_Upper GI_Head and Neck_CoRes
-1
0
1
N
Carnforth
Caton
Bay Horse
M6
5 km

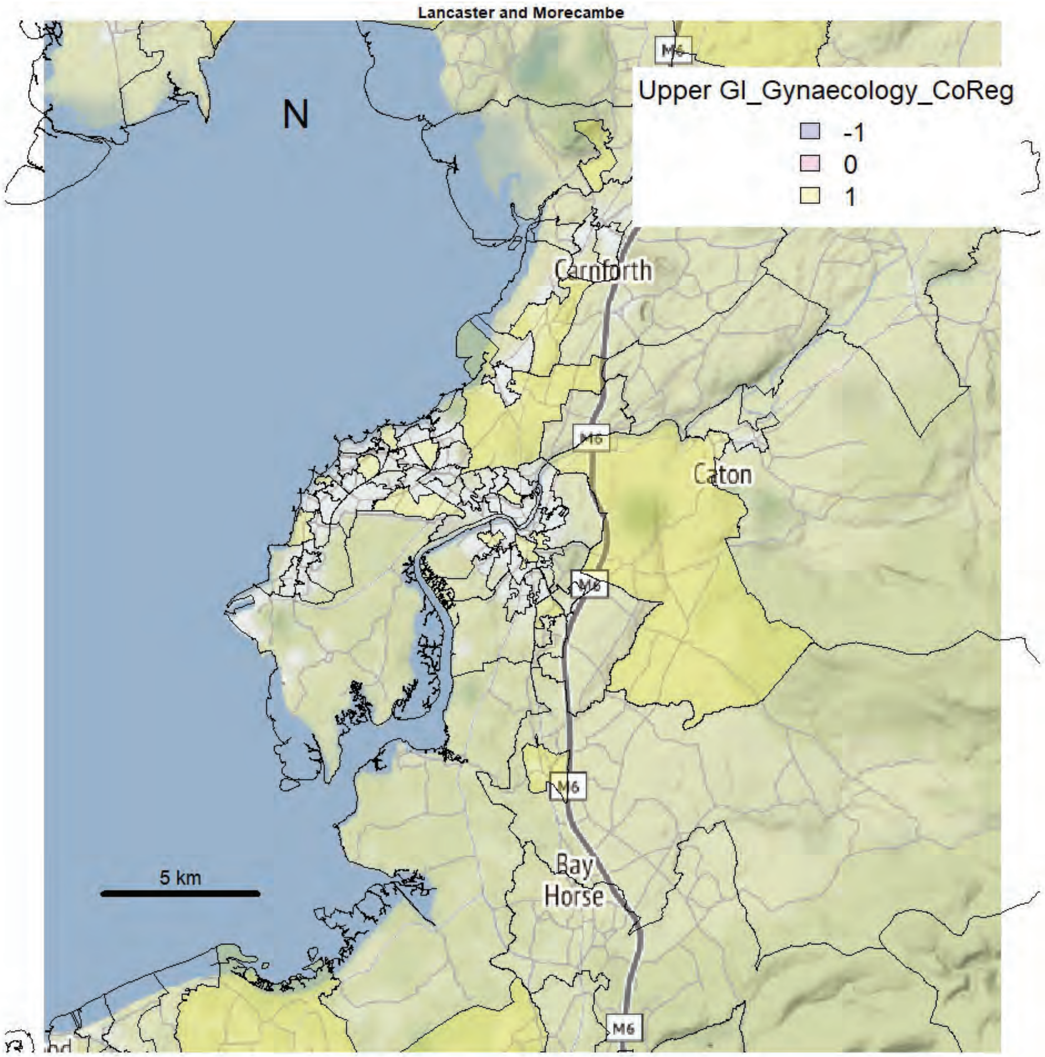
Lancaster and Morecambe
N
Upper GI_Gynaecology_CoReg
-1
0
1
Carnforth
M6
Caton
Bay
Horse
5 km

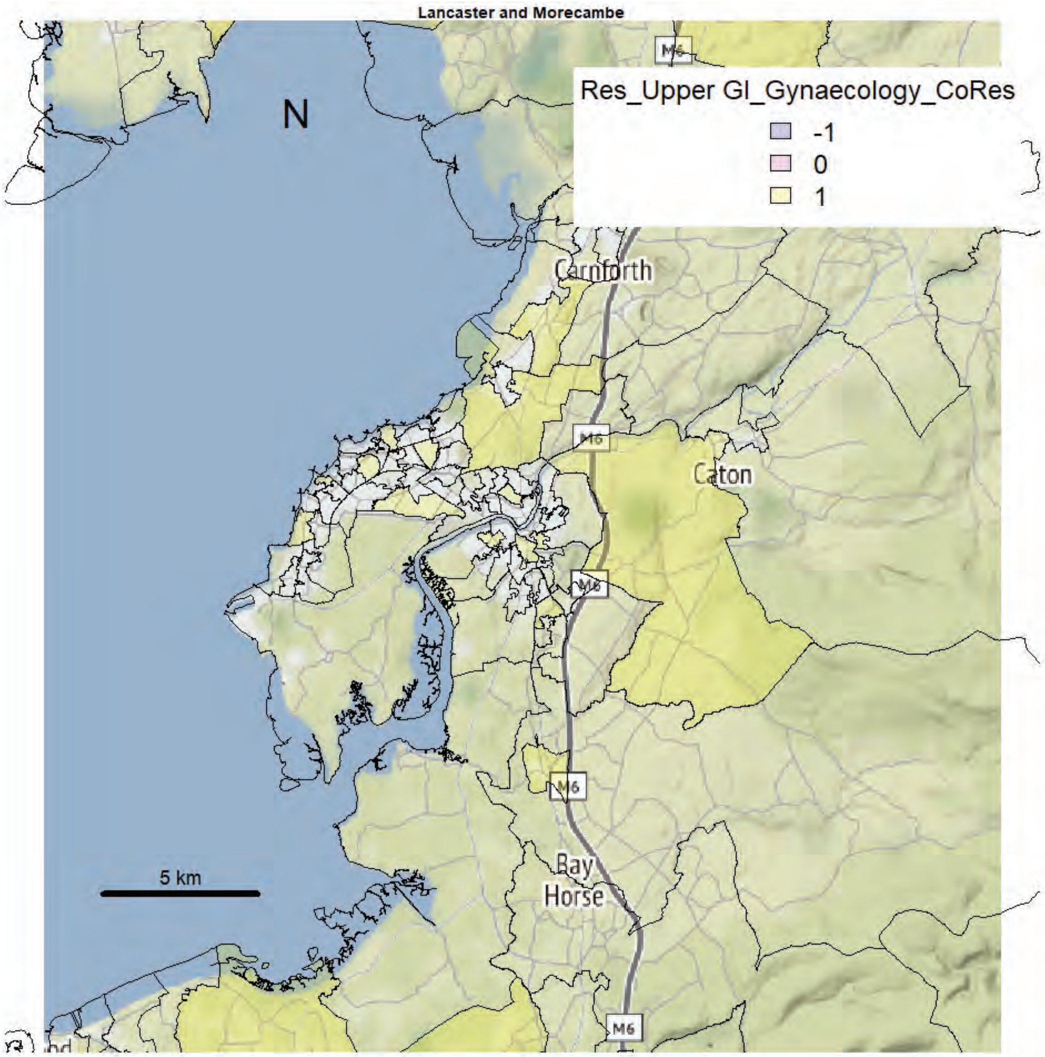
Lancaster and Morecambe
N
Res_Upper GI_Gynaecology_CoRes
-1
0
1
Carnforth
M6
Caton
Bay
Horse
5 km

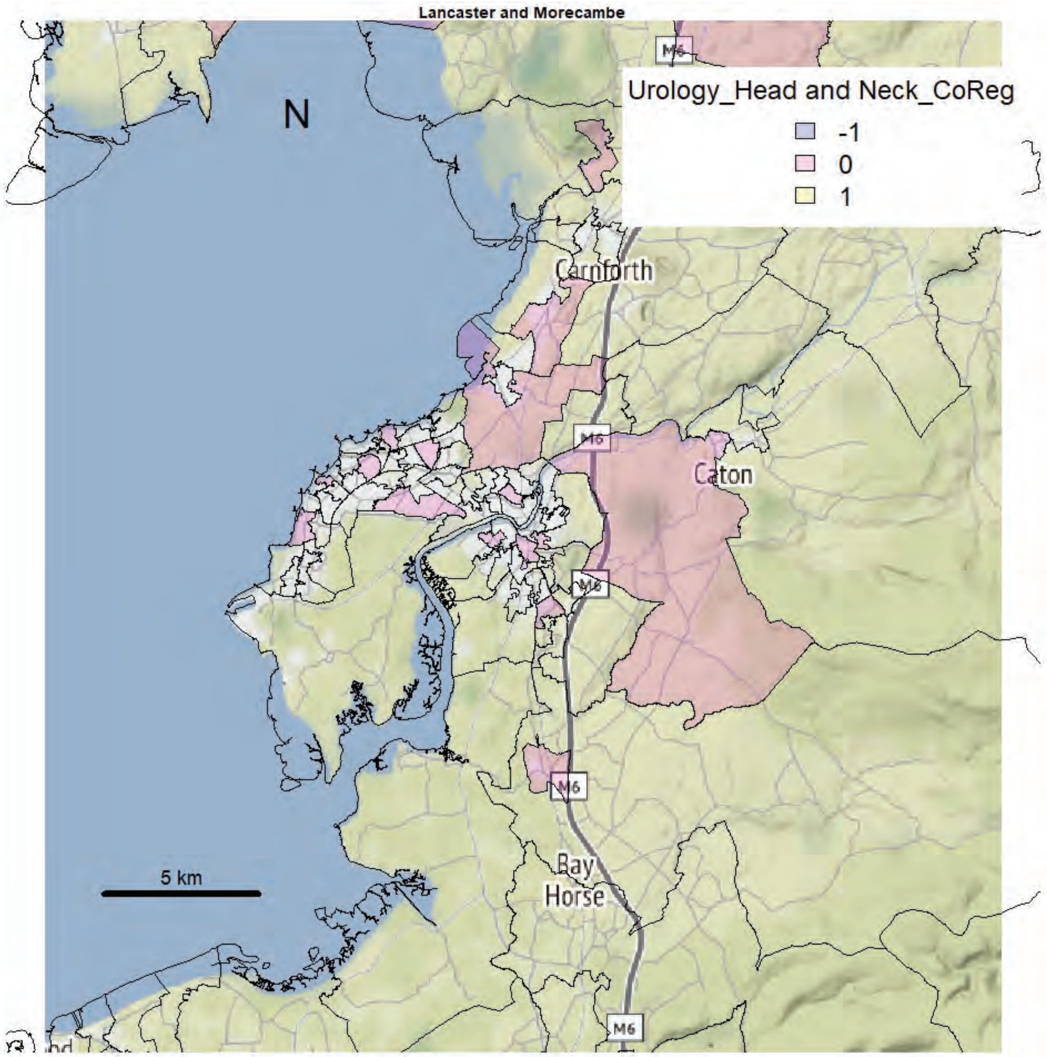
Lancaster and Morecambe
Urology_Head and Neck_CoReg
-1
0
1
N
Carnforth
Caton
Bay
Horse
M6
5 km

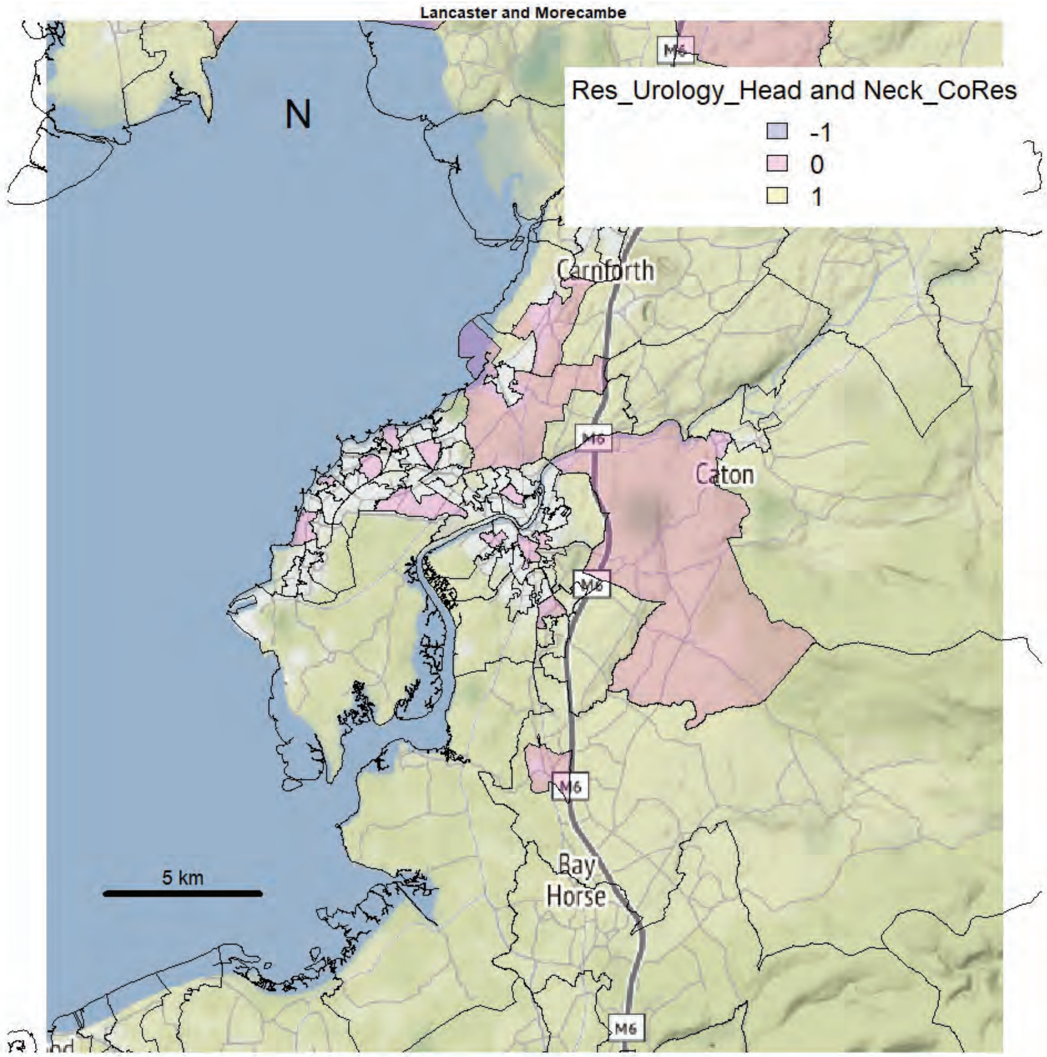
Lancaster and Morecambe
Res_Urology_Head and Neck_CoRes
-1
0
1
N
Carnforth
Caton
Bay
Horse
M6
5 km

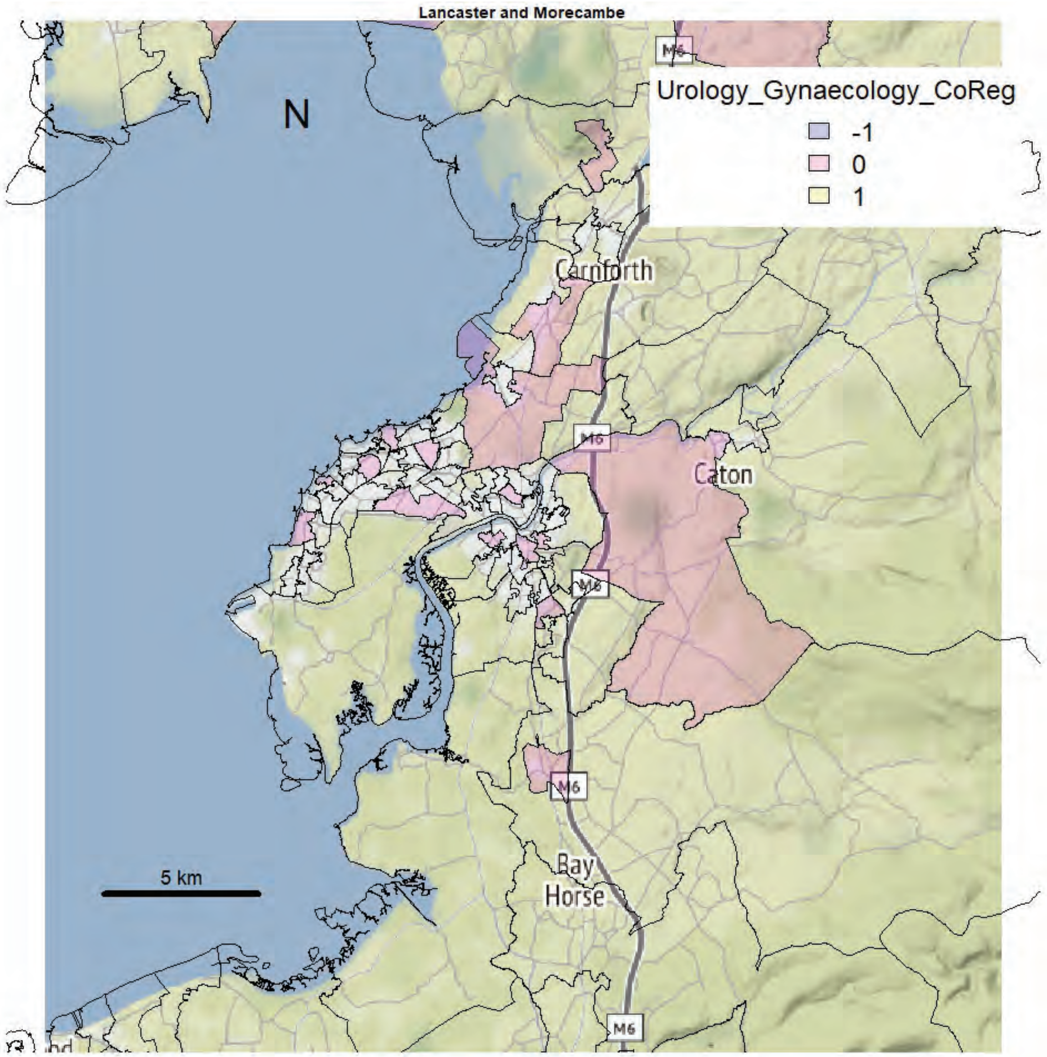
Lancaster and Morecambe
Urology_Gynaecology_CoReg
-1
0
1
N
Carnforth
Caton
Bay Horse
M6
5 km

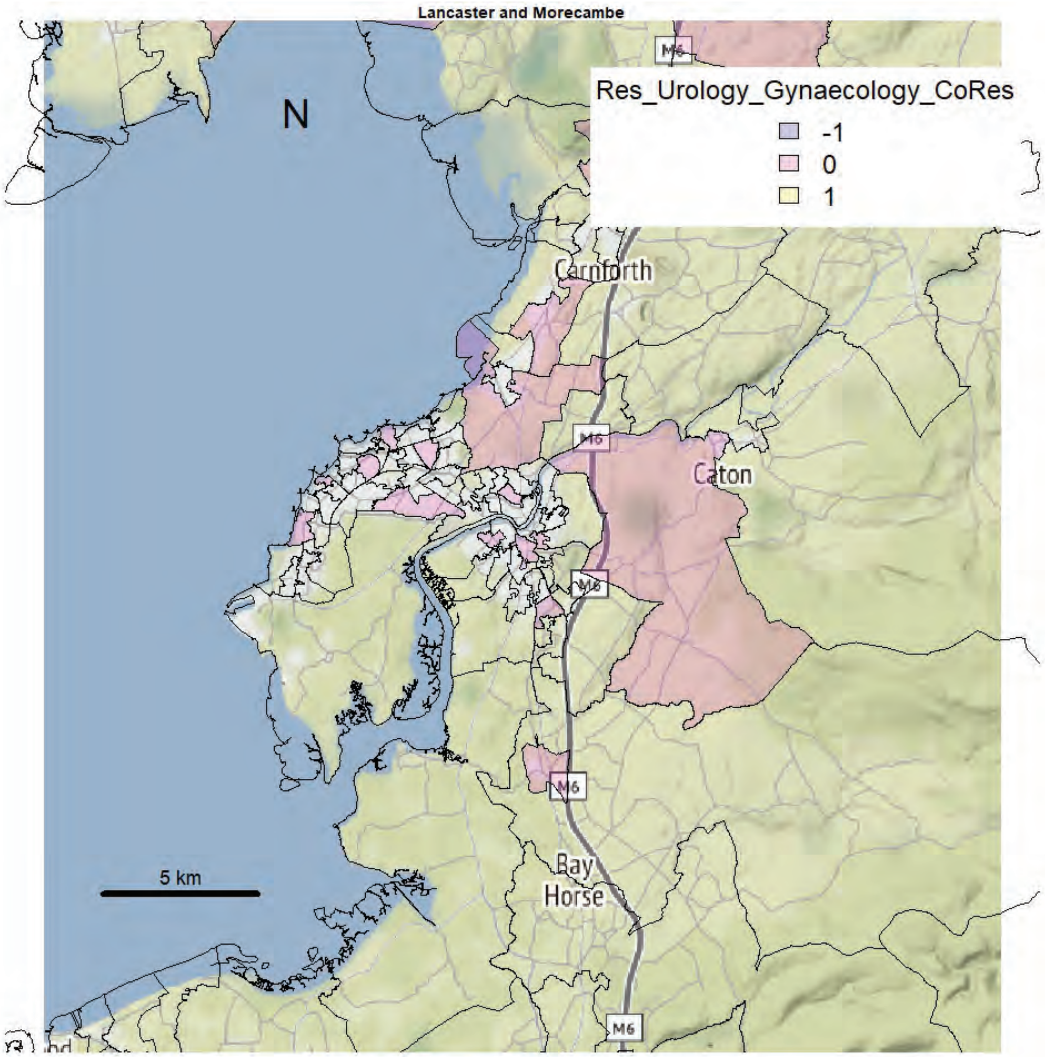
Lancaster and Morecambe
Res_Urology_Gynaecology_CoRes
-1
0
1
N
Carnforth
Caton
Bay Horse
M6
5 km

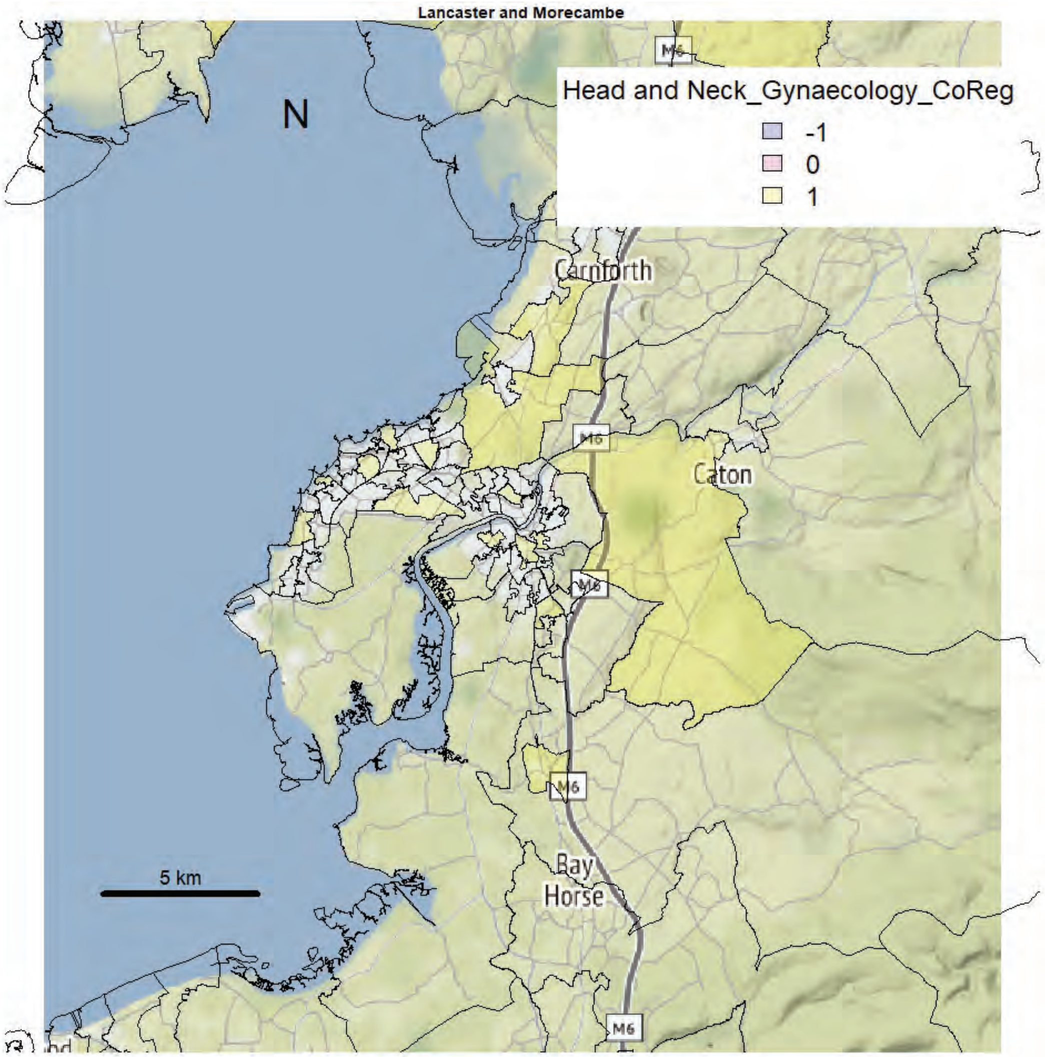

Lancaster and Morecambe
Head and Neck_Gynaecology_CoReg
-1
0
1
N
Carnforth
Caton
Bay Horse
M6
5 km

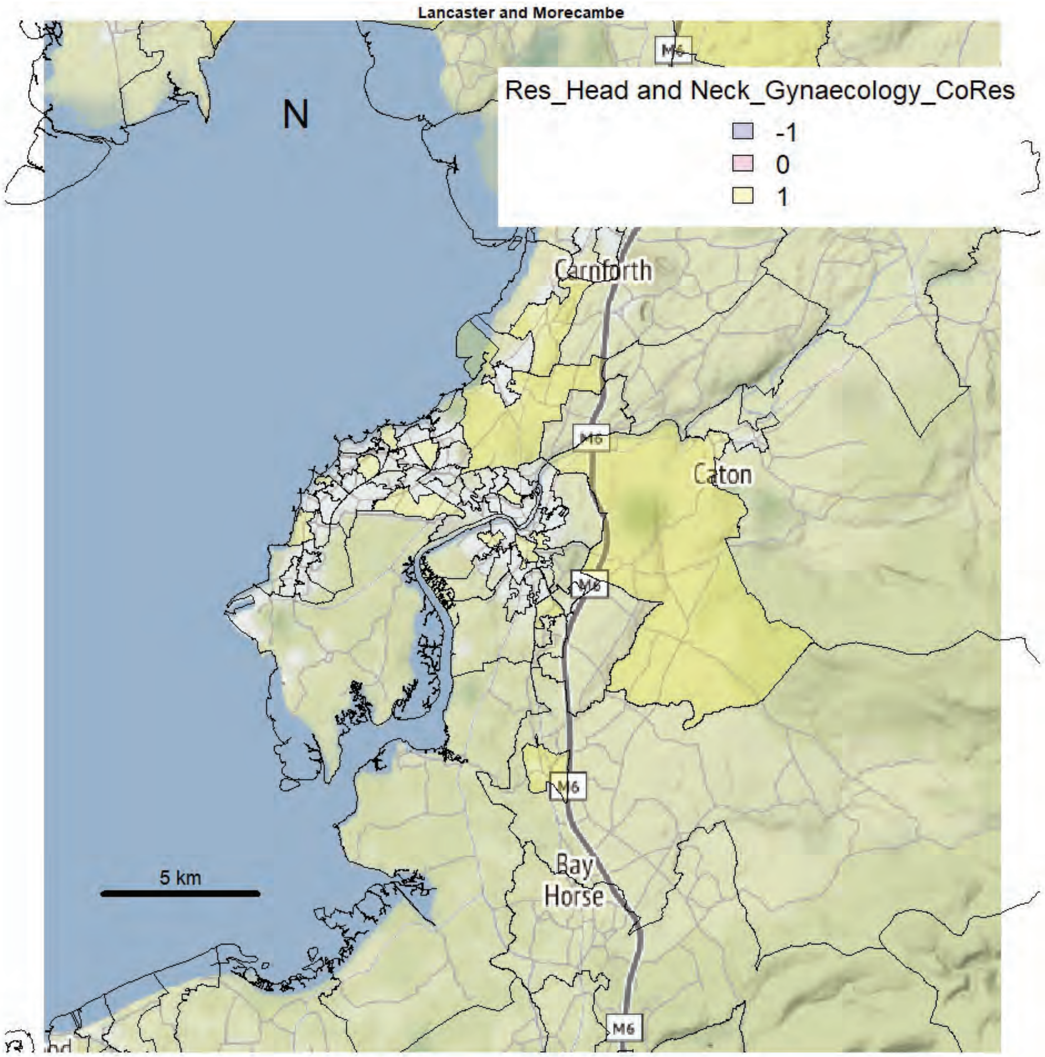

Lancaster and Morecambe
Res_Head and Neck_Gynaecology_CoRes
-1
0
1
N
Carnforth
Caton
Bay Horse
M6
5 km

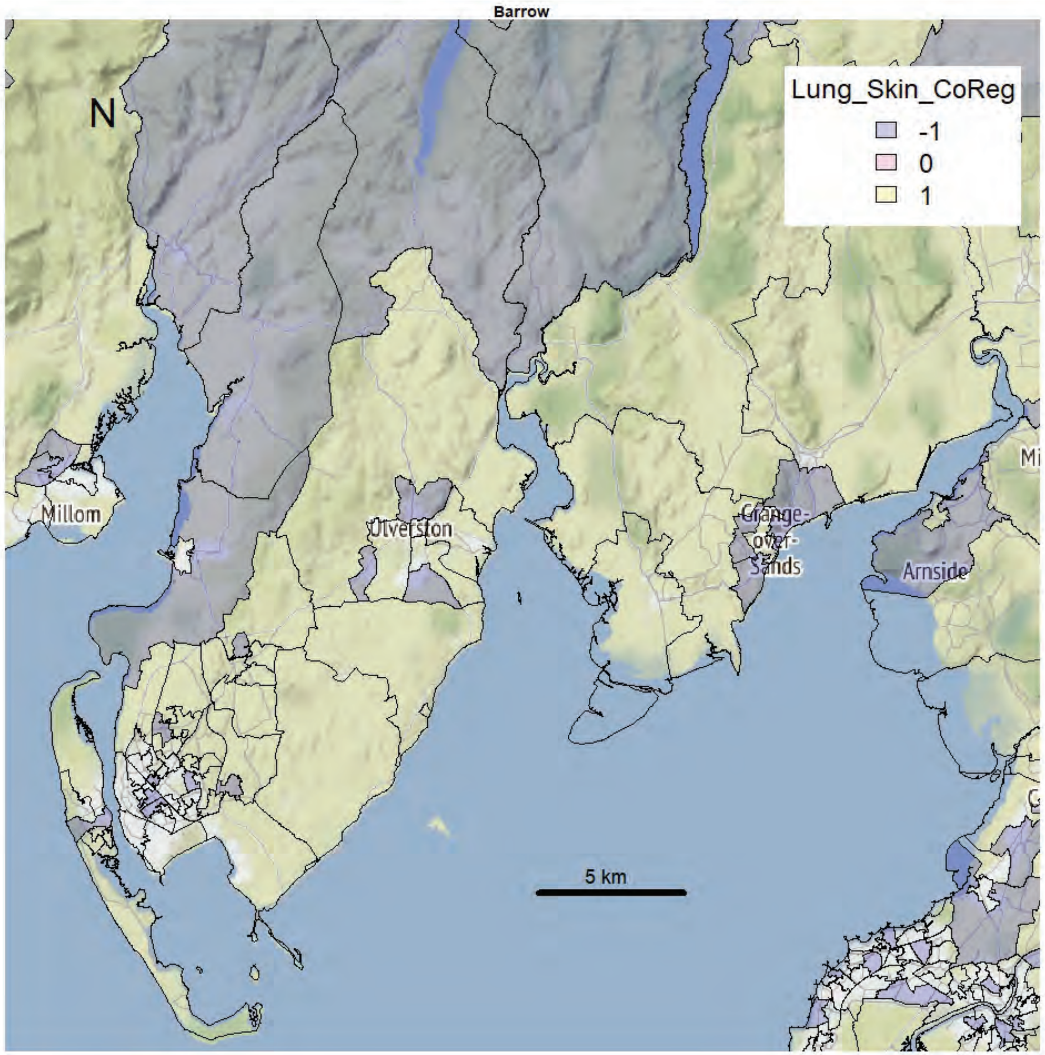
Barrow
N
Lung_Skin_CoReg
-1
0
1
Millom
Ulverston
Grange-over-Sands
Arnside
5 km

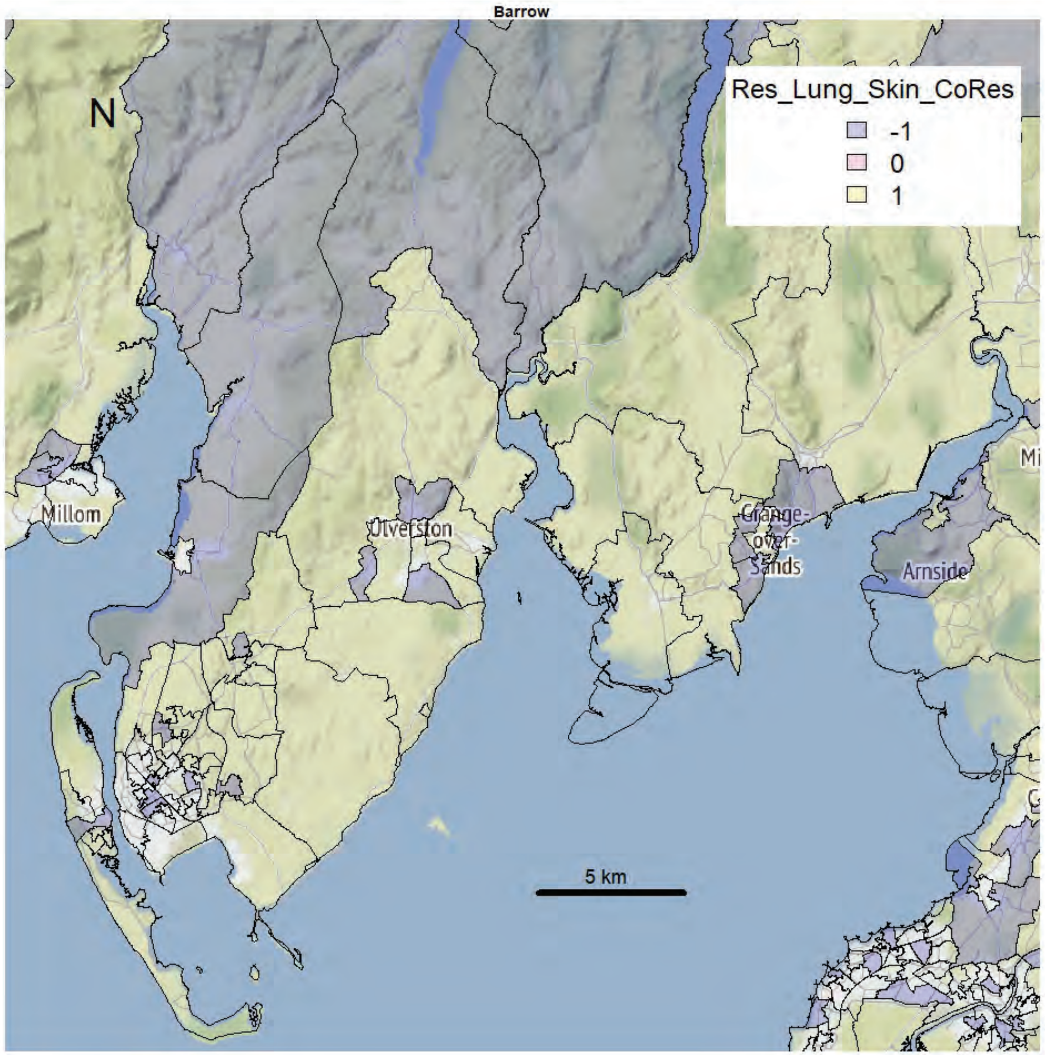
Barrow
N
Res_Lung_Skin_CoRes
-1
0
1
Millom
Ulverston
Grange-over-Sands
Arnside
5 km

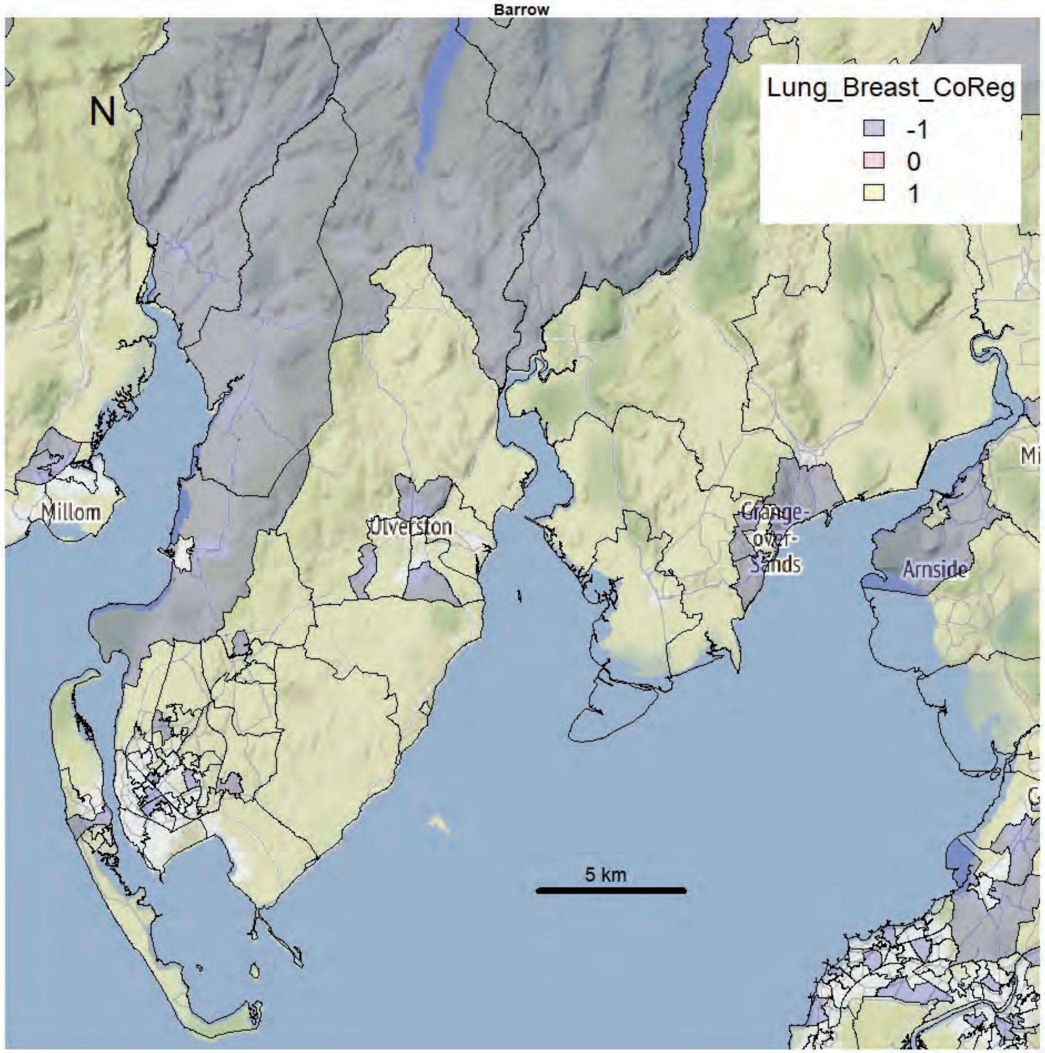
Barrow
N
Lung_Breast_CoReg
-1
0
1
Millom
Ulverston
Grange-over-Sands
Arnside
5 km

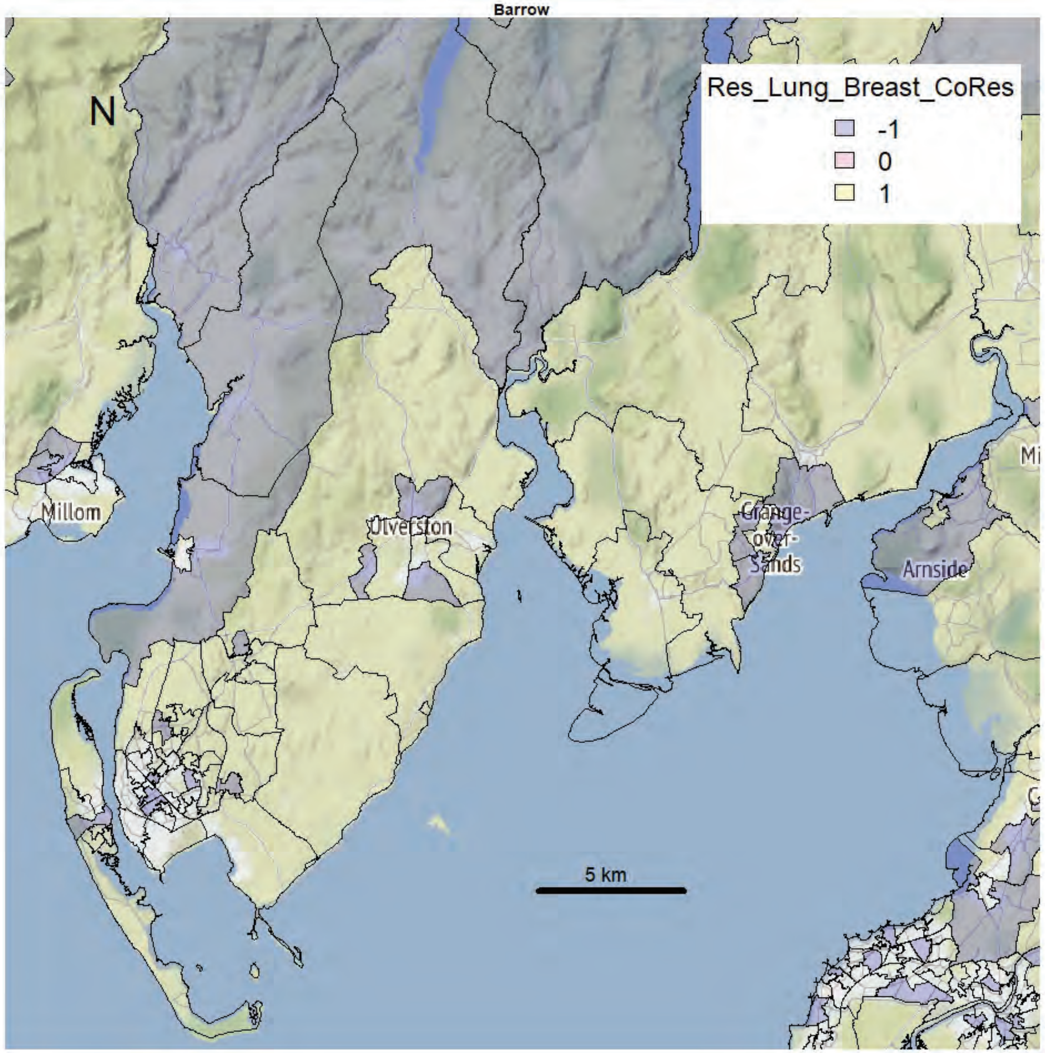
Barrow
N
Res_Lung_Breast_CoRes
-1
0
1
Millom
Ulverston
Grange-over-Sands
Arnside
5 km

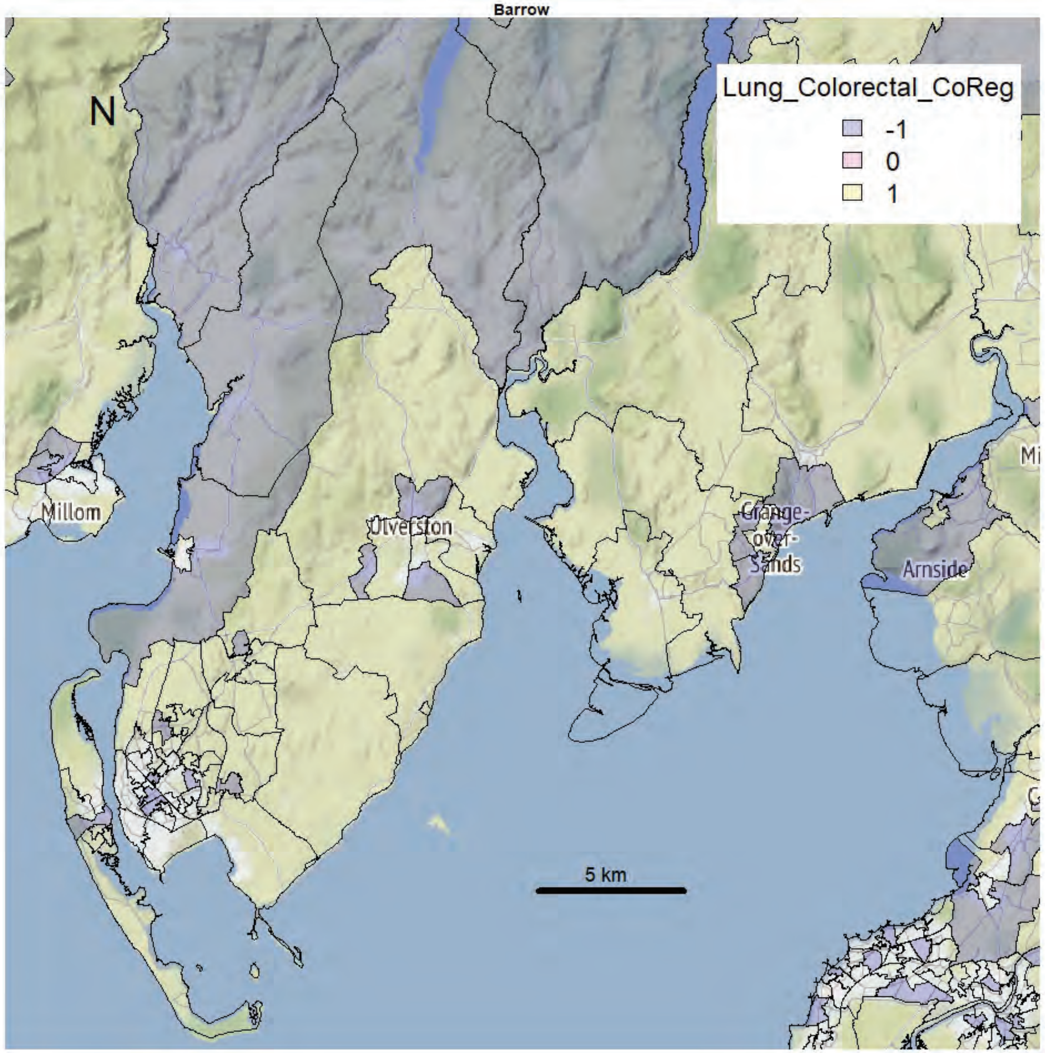

Barrow
N
Lung_Colorectal_CoReg
-1
0
1
Millom
Ulverston
Grange-
over-
Sands
Arnside
5 km

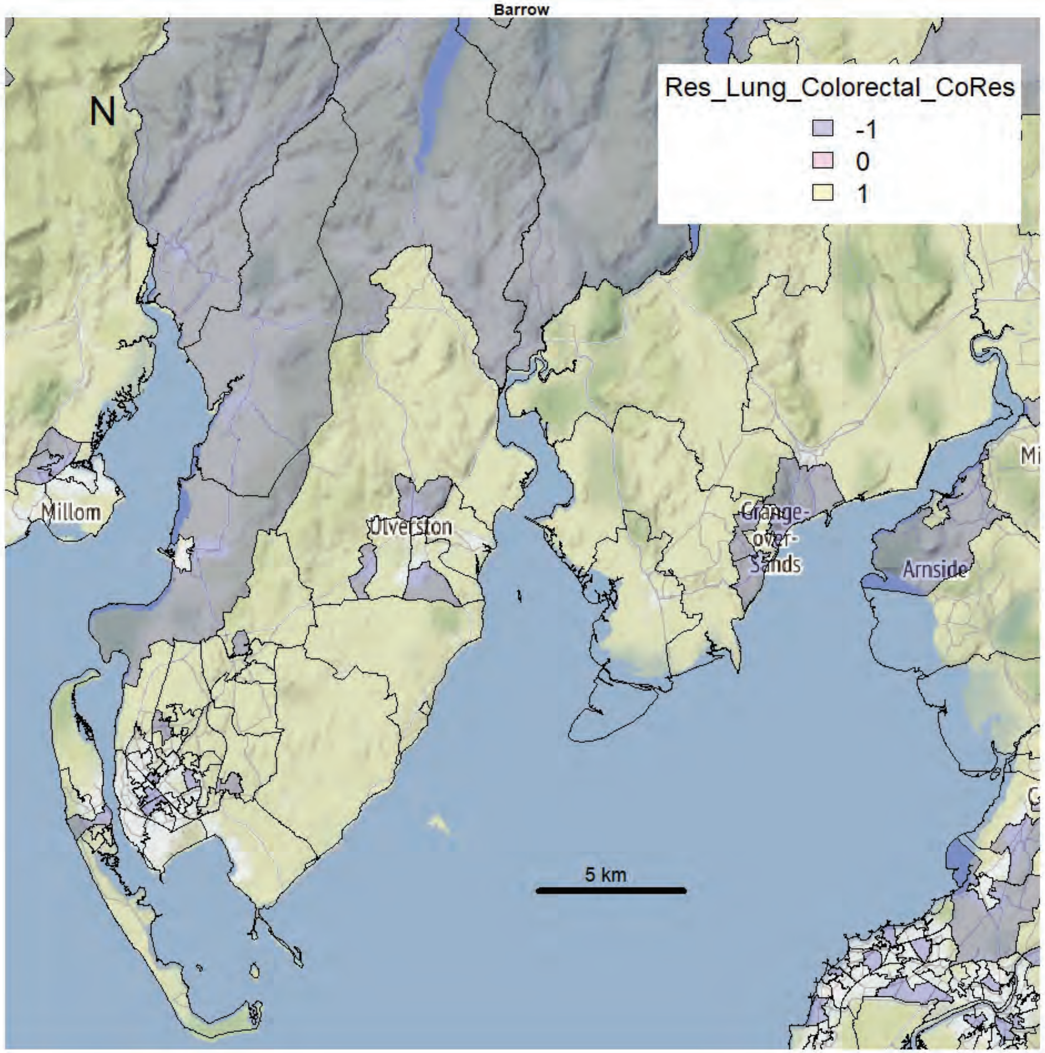

Barrow
N
Res_Lung_Colorectal_CoRes
-1
0
1
Millom
Ulverston
Grange-
over-
Sands
Arnside
5 km

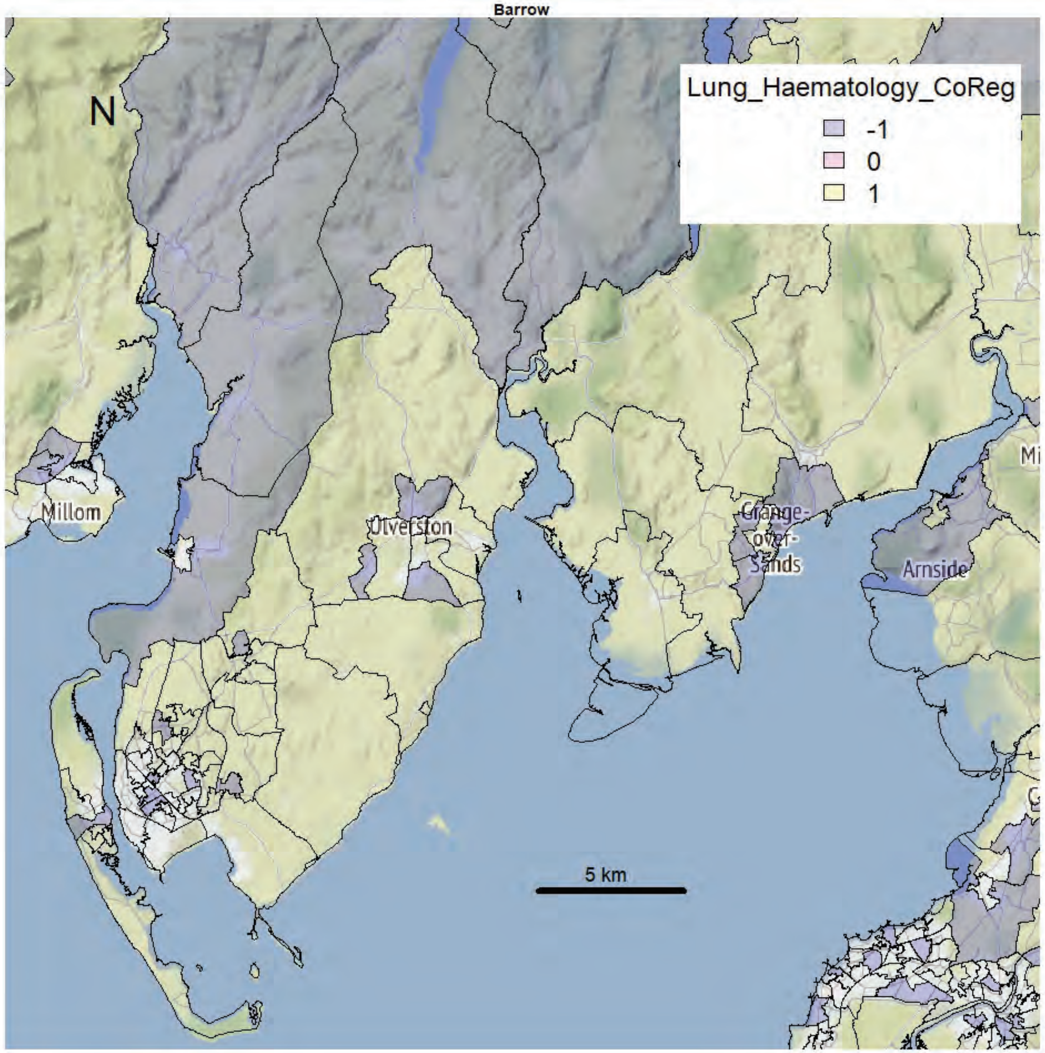
Barrow
N
Lung_Haematology_CoReg
-1
0
1
Millom
Ulverston
Grange-
over-
Sands
Arnside
5 km

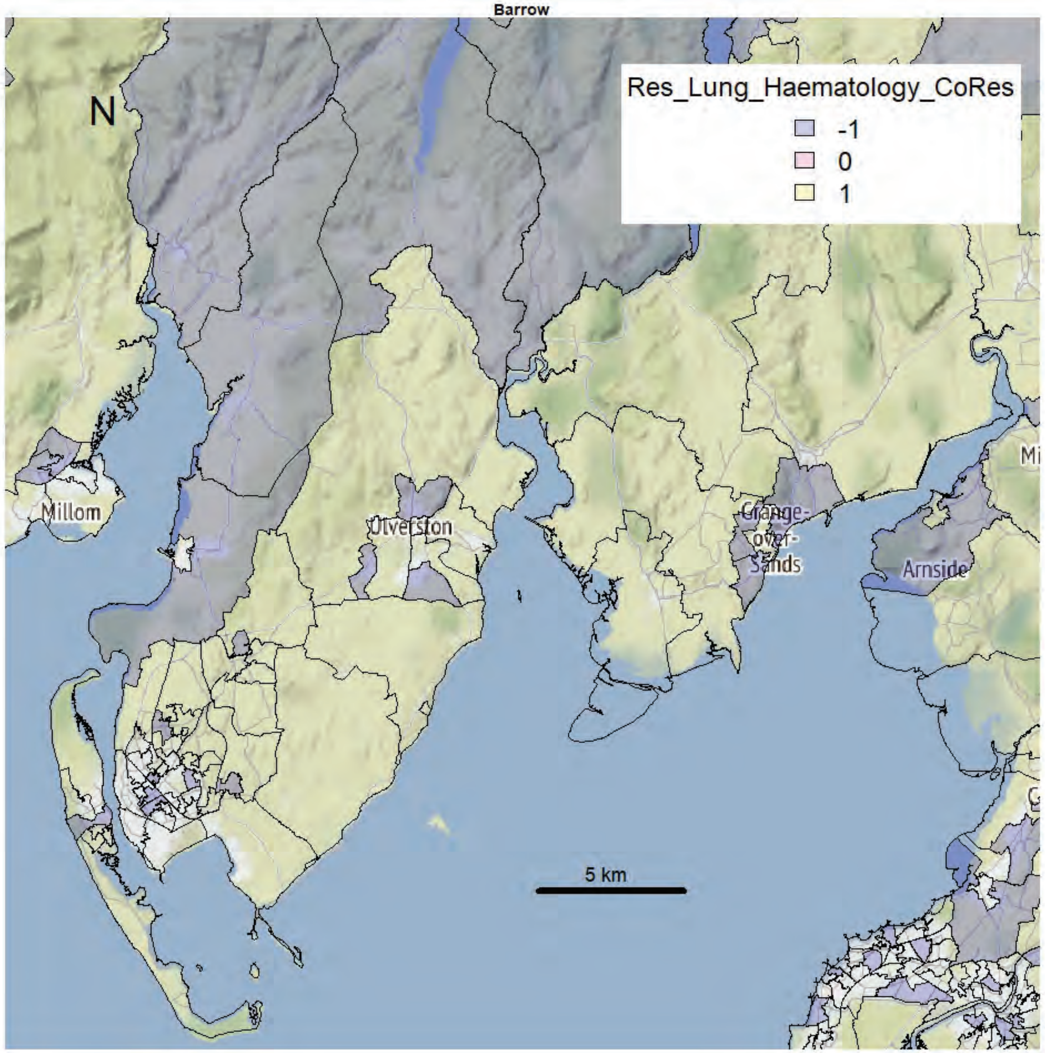
Barrow
N
Res_Lung_Haematology_CoRes
-1
0
1
Millom
Ulverston
Grange-
over-
Sands
Arnside
5 km

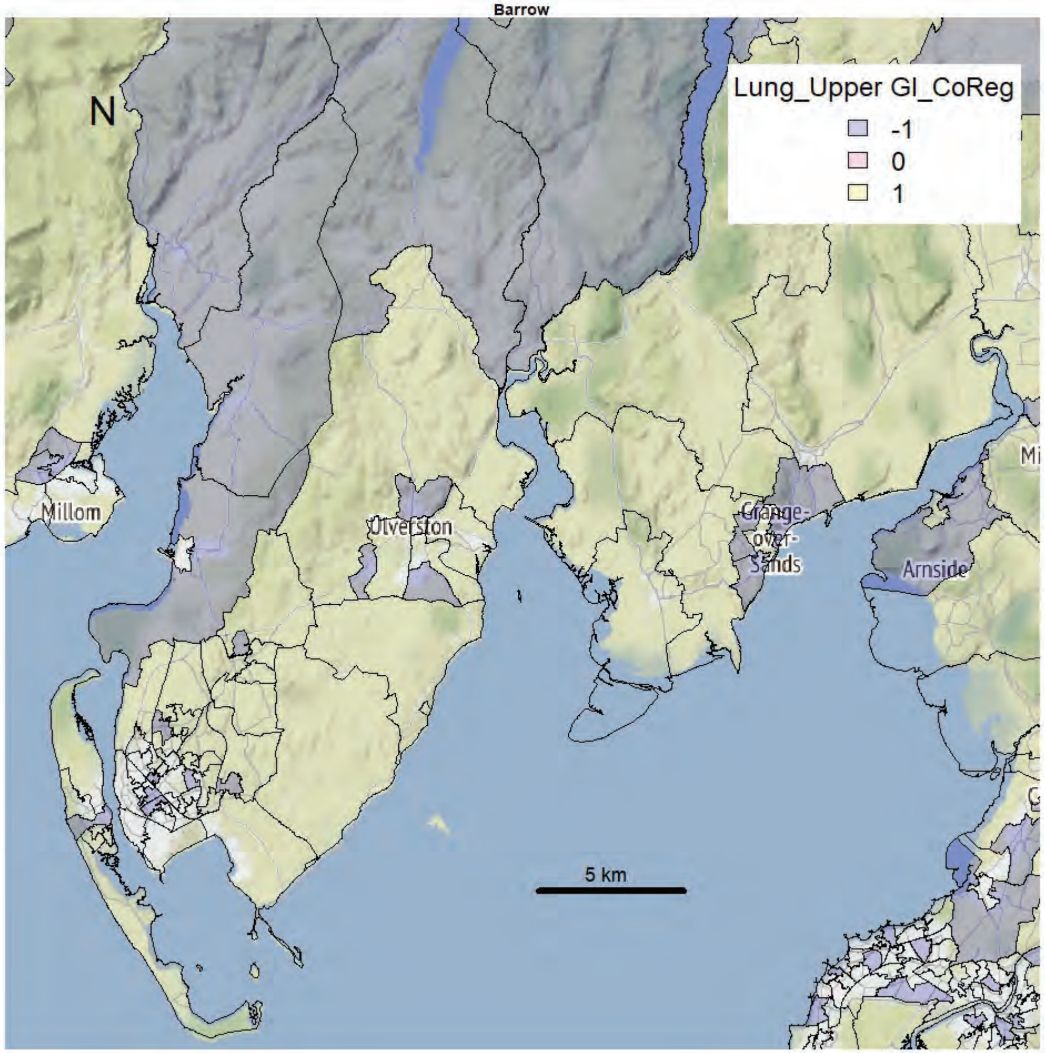
Barrow
N
Lung_Upper GI_CoReg
-1
0
1
Millom
Ulverston
Grange-over-Sands
Arnside
5 km

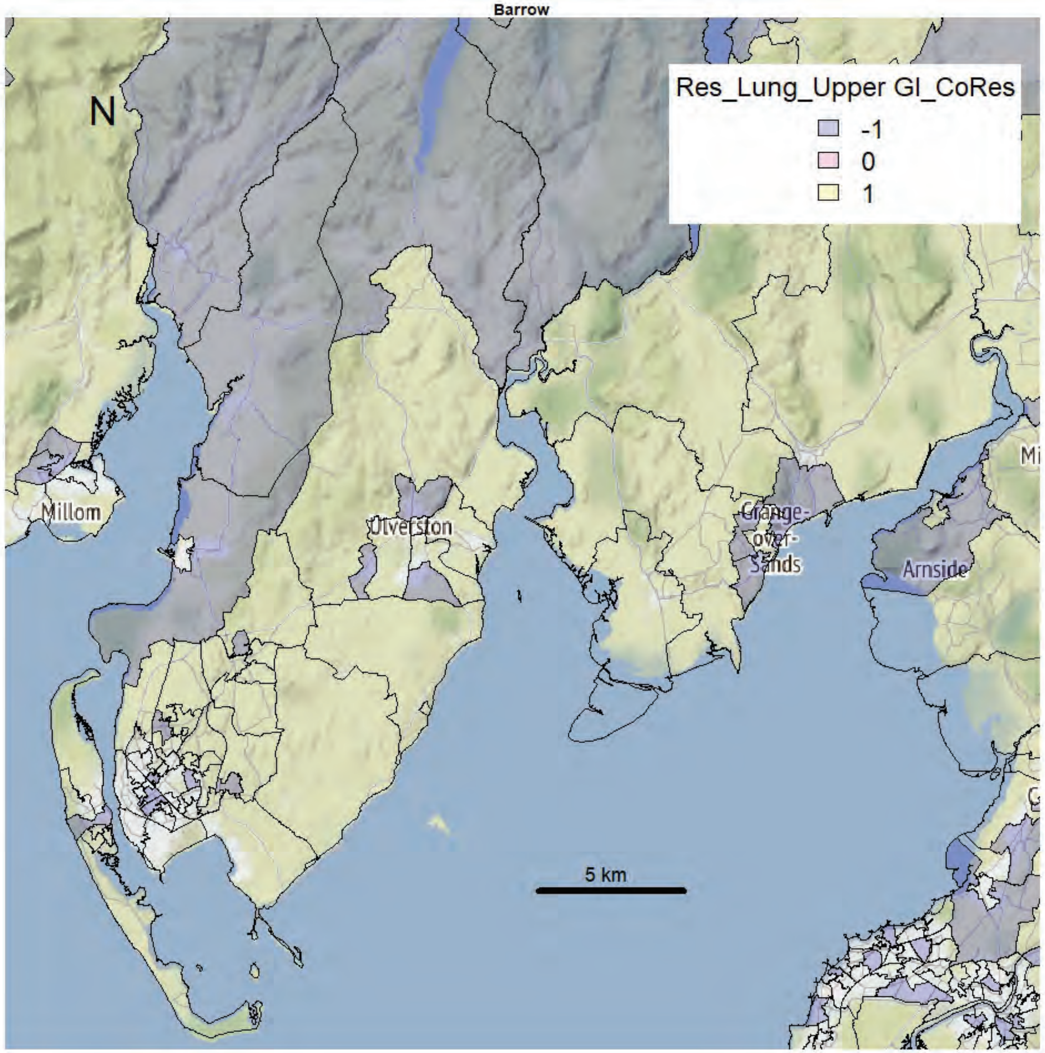
Barrow
N
Res_Lung_Upper GI_CoRes
-1
0
1
Millom
Ulverston
Grange-over-Sands
Arnside
5 km

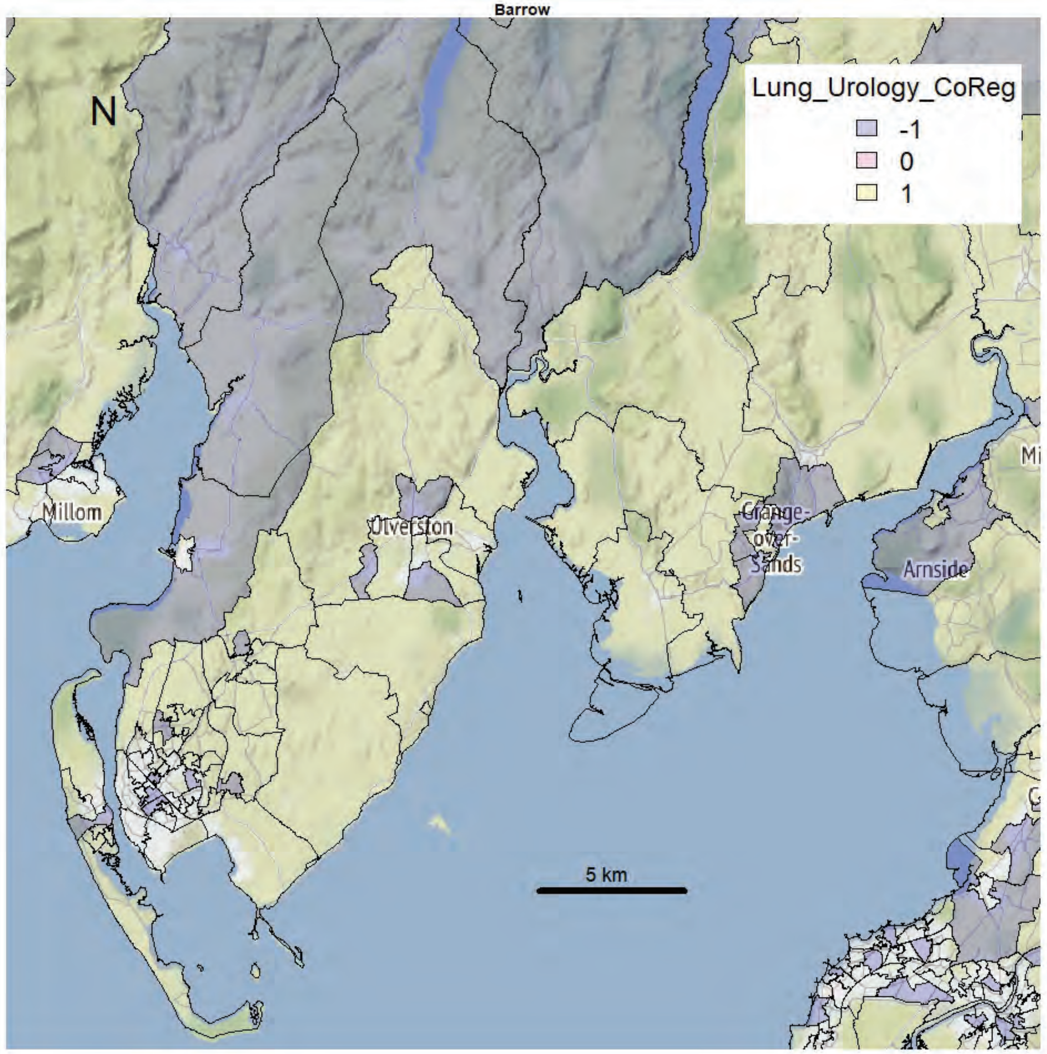
Barrow
N
Lung_Urology_CoReg
-1
0
1
Millom
Ulverston
Grange-over-Sands
Arnside
5 km

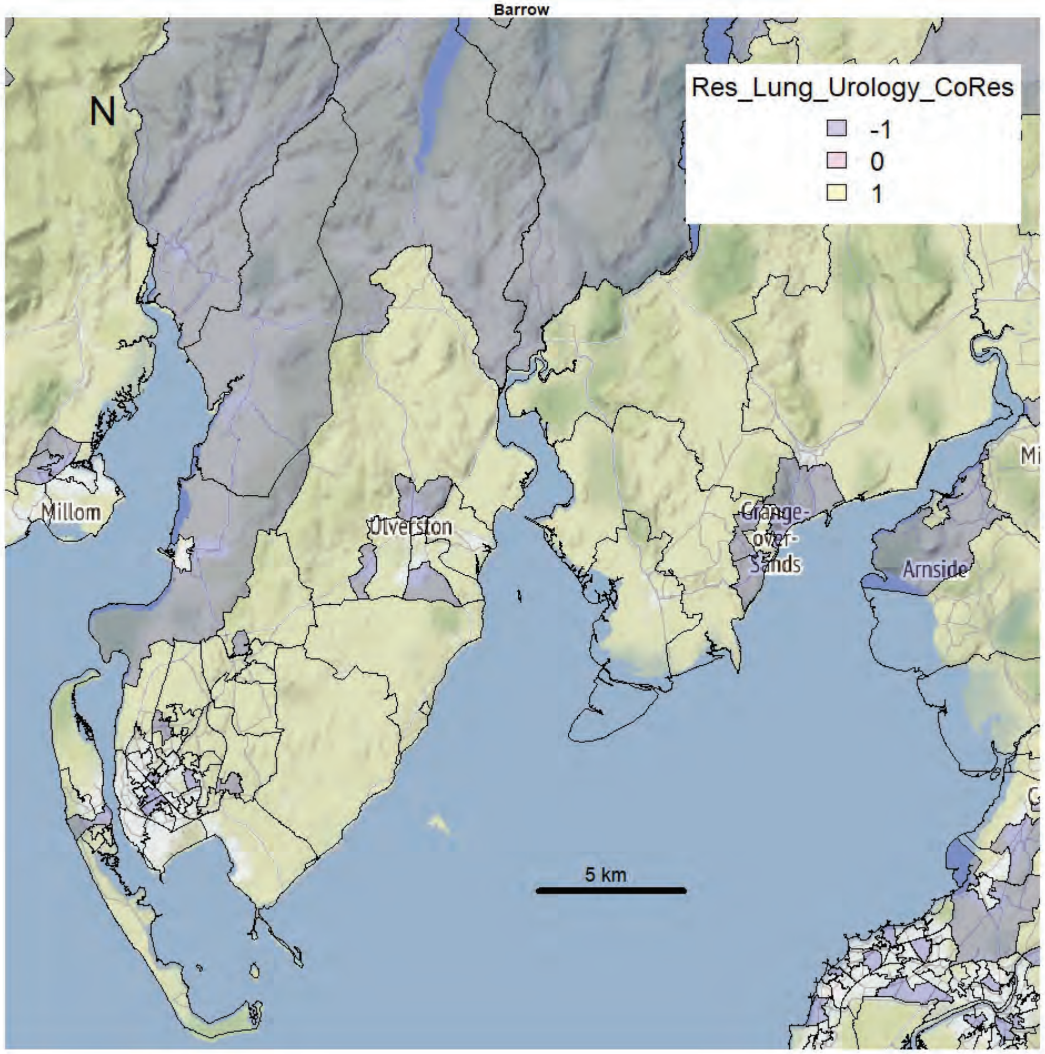
Barrow
N
Res_Lung_Urology_CoRes
-1
0
1
Millom
Ulverston
Grange-over-Sands
Arnside
5 km

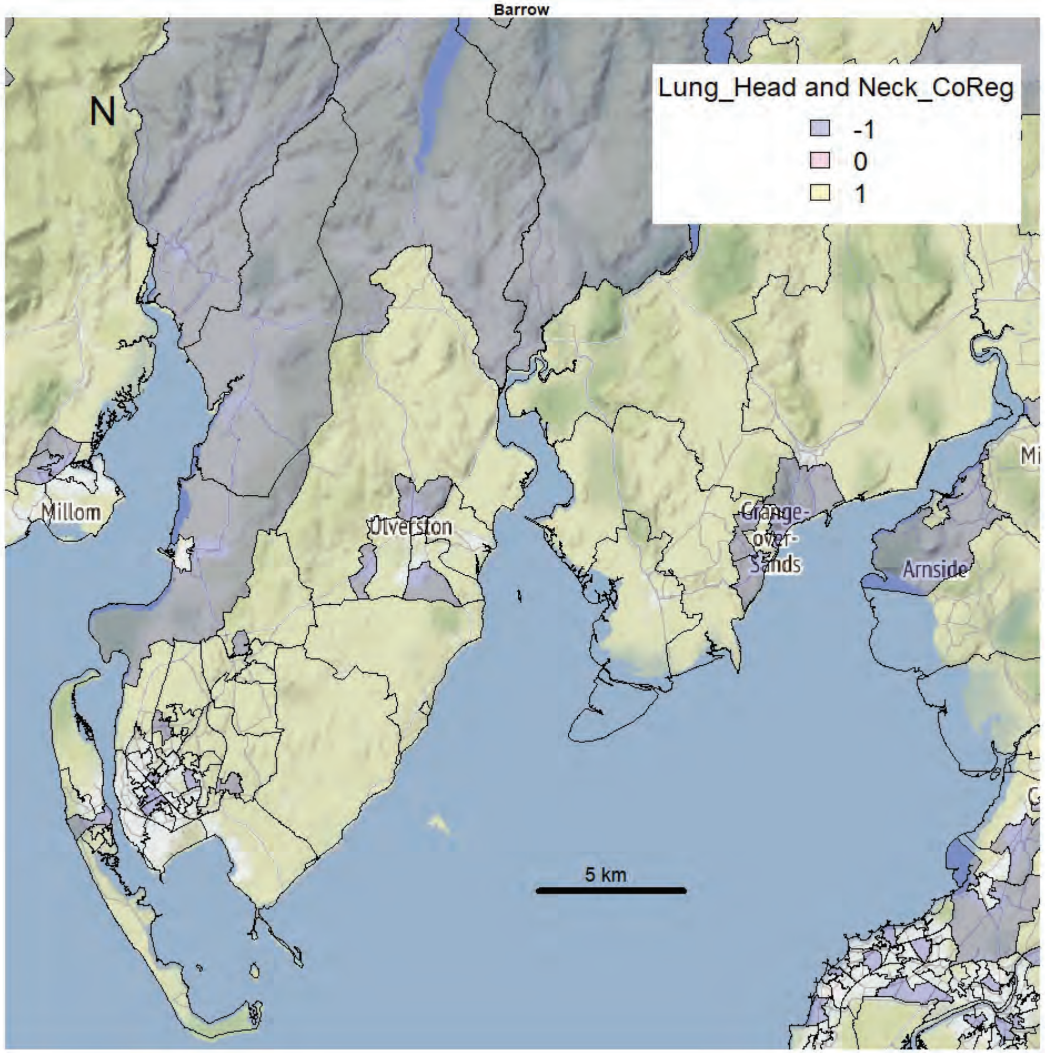
Barrow
Lung_Head and Neck_CoReg
-1
0
1
N
Millom
Ulverston
Grange-over-Sands
Arnside
5 km

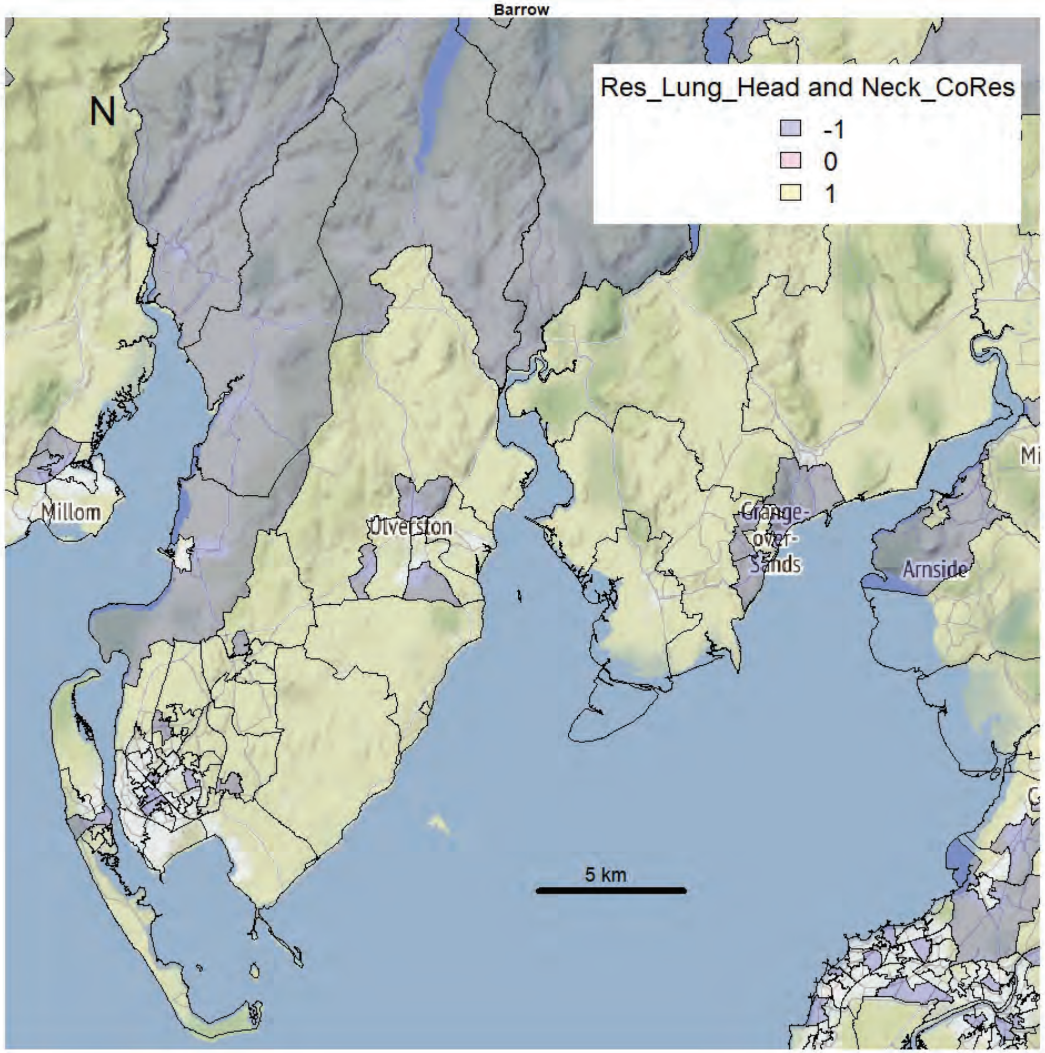
Barrow
Res_Lung_Head and Neck_CoRes
-1
0
1
N
Millom
Ulverston
Grange-over-Sands
Arnside
5 km

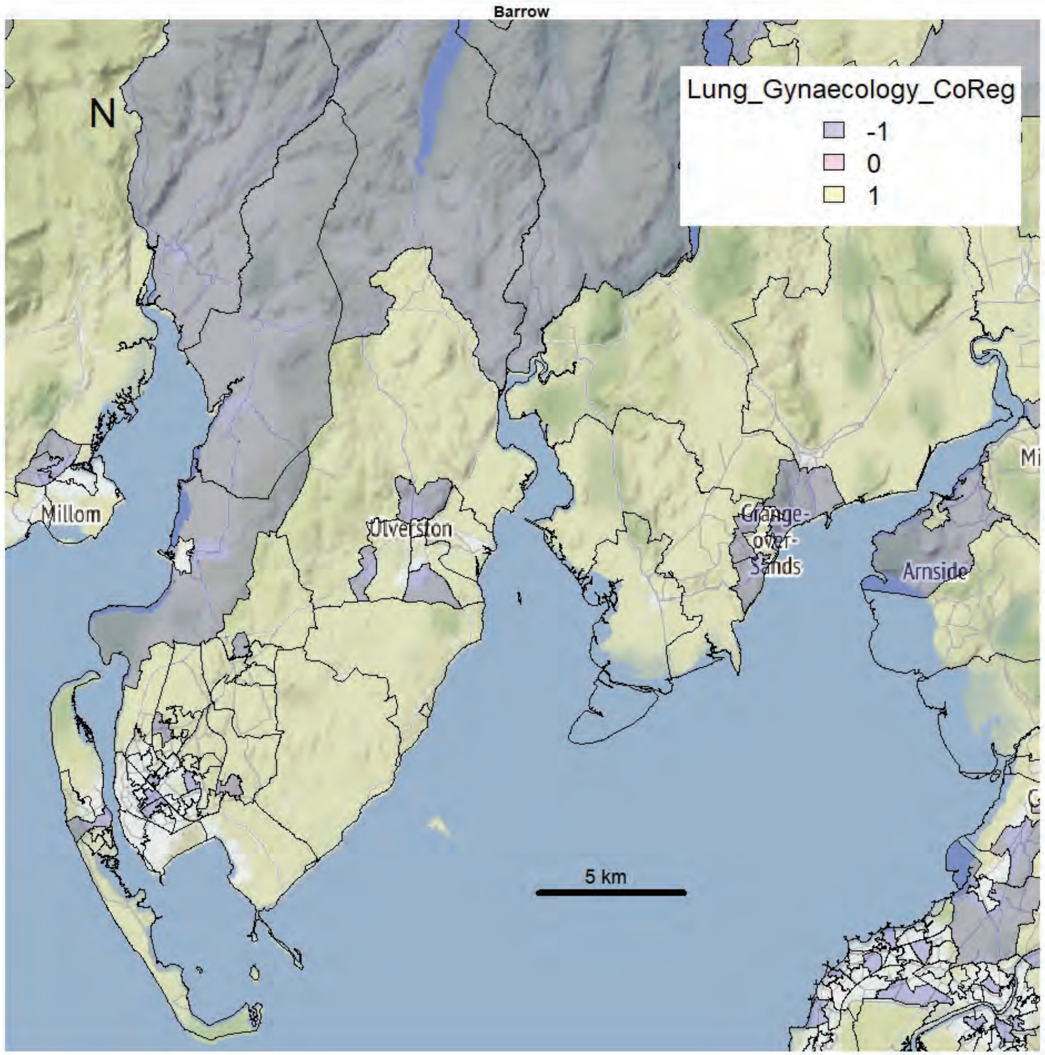
Barrow
N
Lung_Gynaecology_CoReg
-1
0
1
Millom
Ulverston
Grange-
over-
Sands
Arnside
5 km

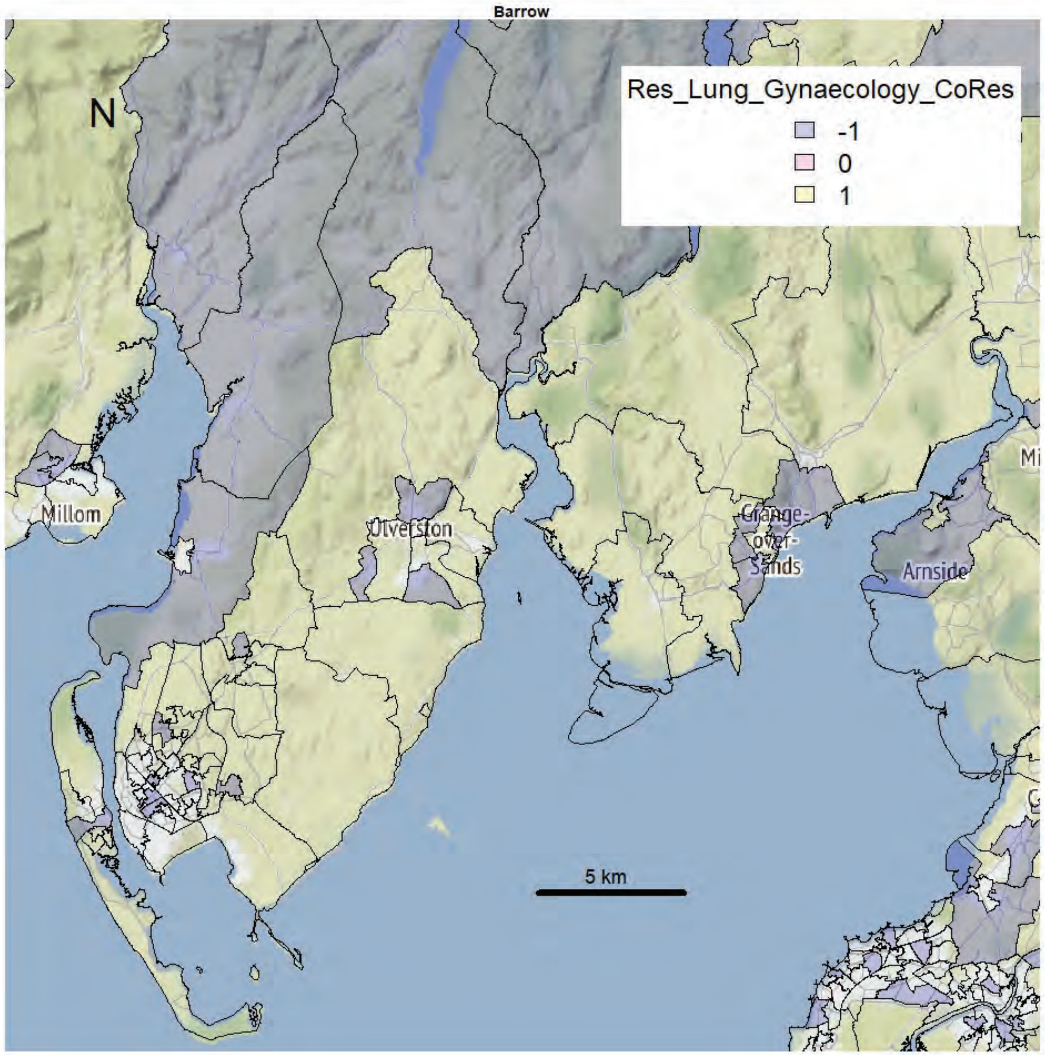
Barrow
N
Res_Lung_Gynaecology_CoRes
-1
0
1
Millom
Ulverston
Grange-
over-
Sands
Arnside
5 km

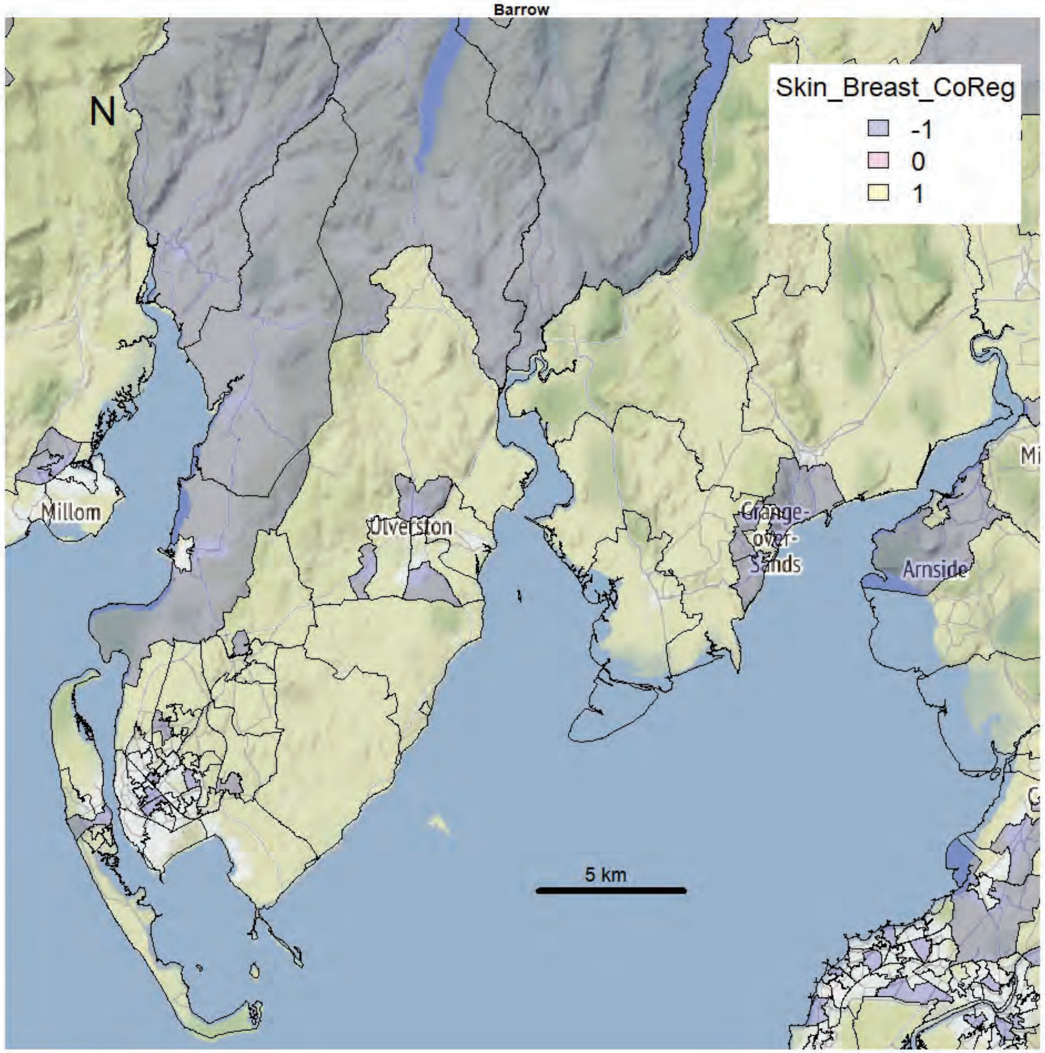
Barrow
N
Skin_Breast_CoReg
-1
0
1
Millom
Ulverston
Grange-over-Sands
Arnside
5 km

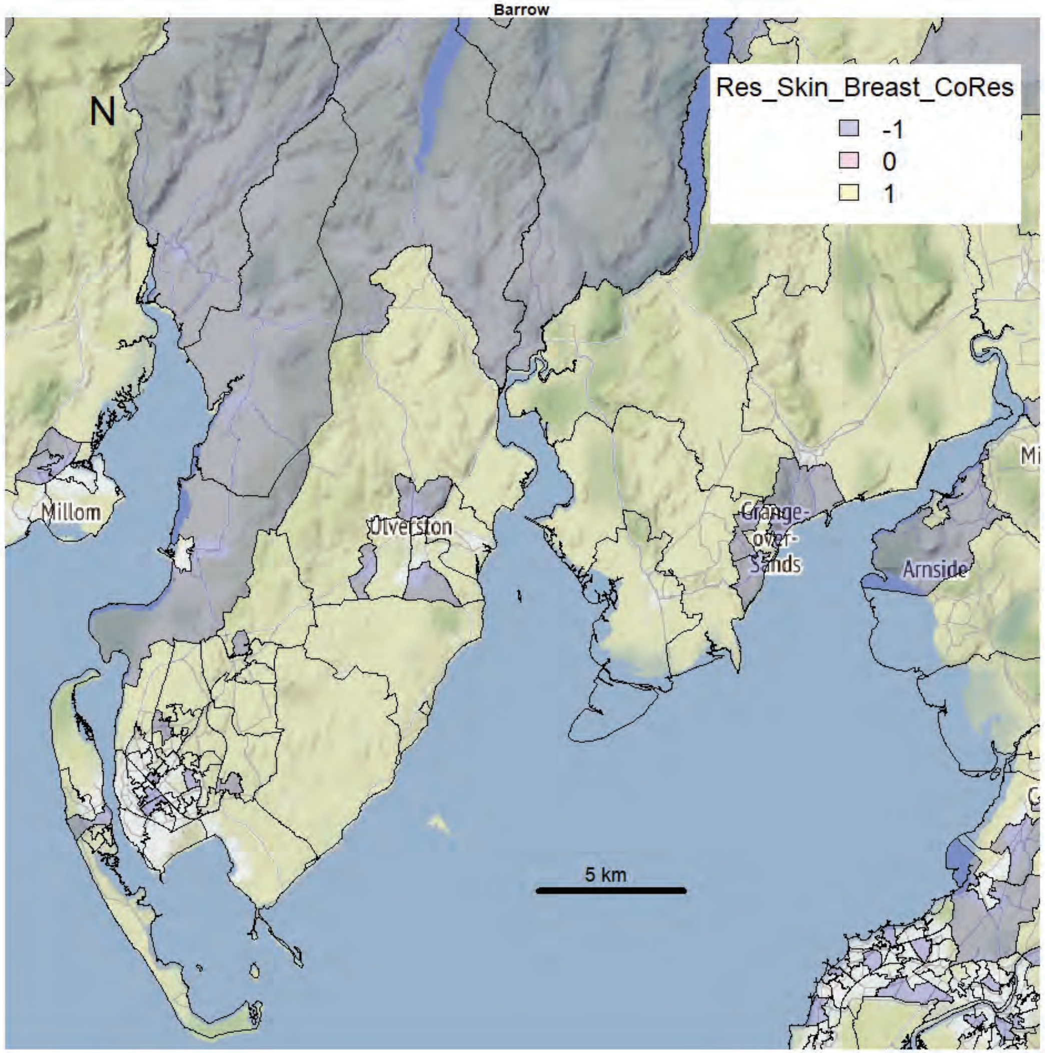
Barrow
N
Res_Skin_Breast_CoRes
-1
0
1
Millom
Ulverston
Grange-over-Sands
Arnside
5 km

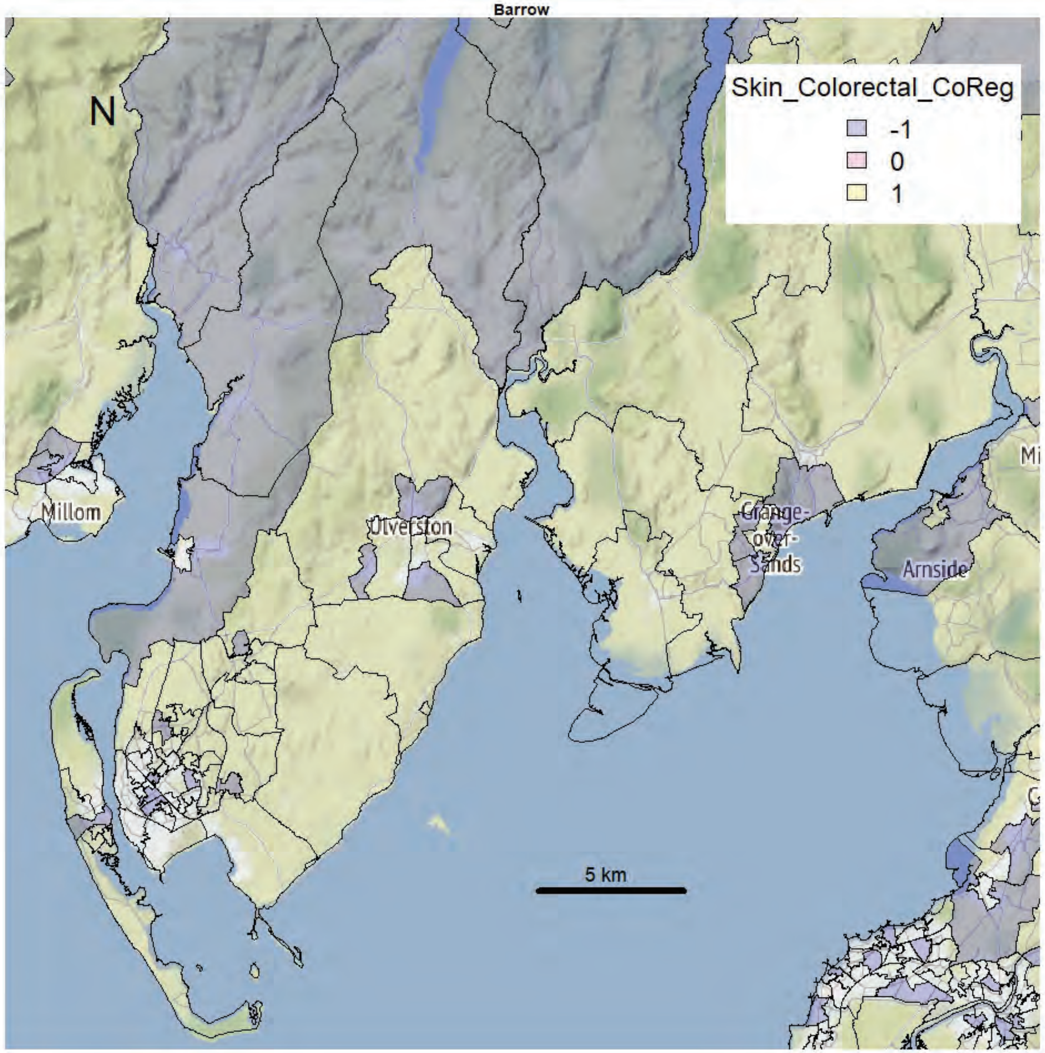
Barrow
N
Skin_Colorectal_CoReg
-1
0
1
Millom
Ulverston
Grange-over-Sands
Arnside
5 km

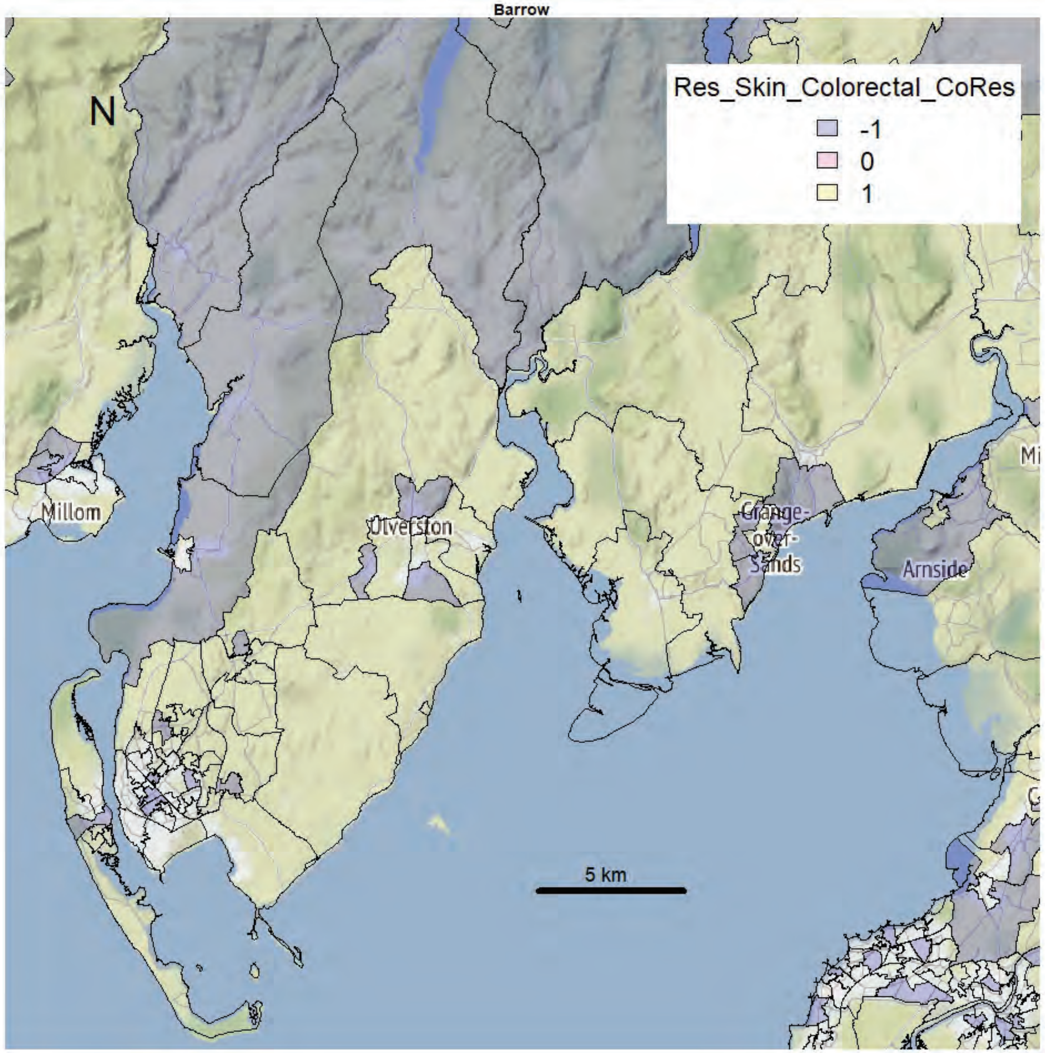
Barrow
N
Res_Skin_Colorectal_CoRes
-1
0
1
Millom
Ulverston
Grange-over-Sands
Arnside
5 km

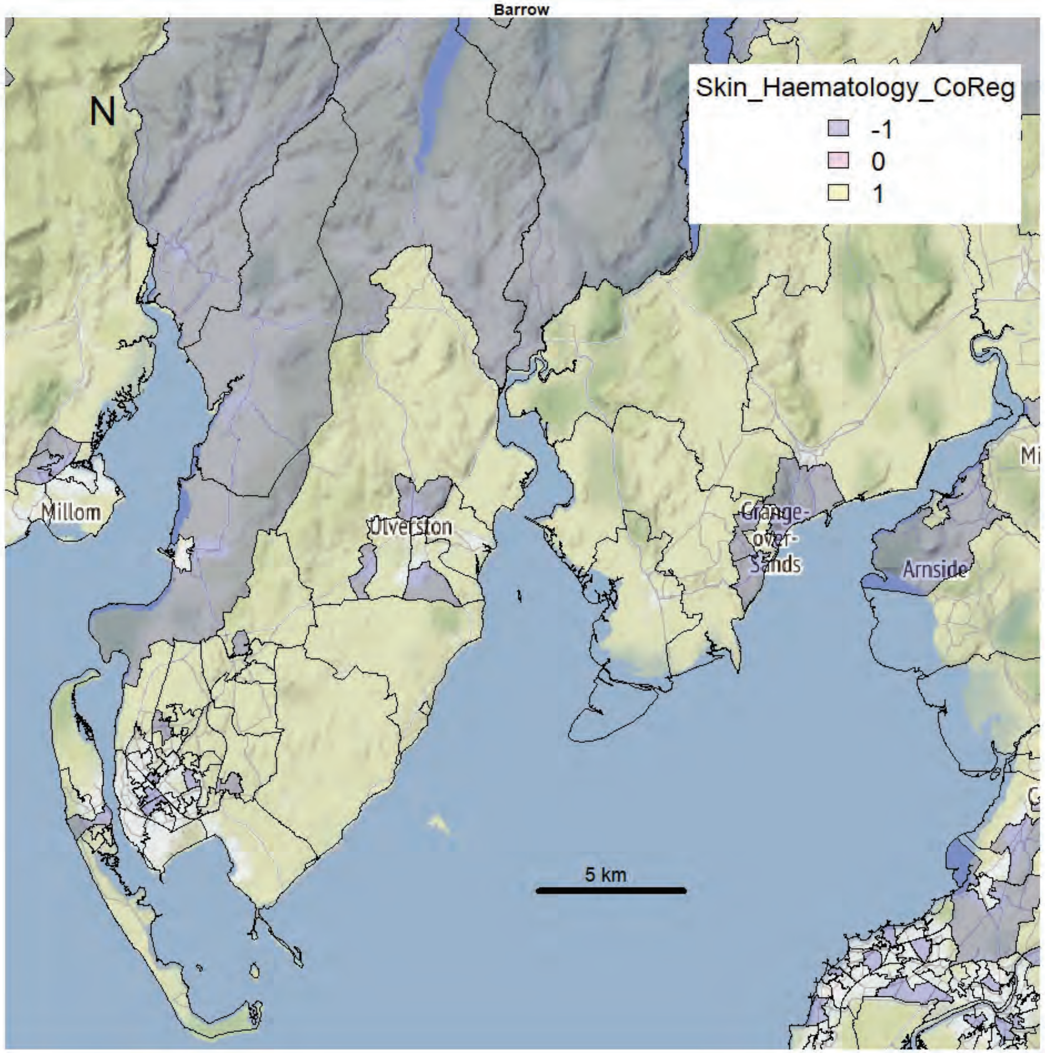
Barrow
N
Skin_Haematology_CoReg
-1
0
1
Millom
Ulverston
Grange-over-Sands
Arnside
5 km

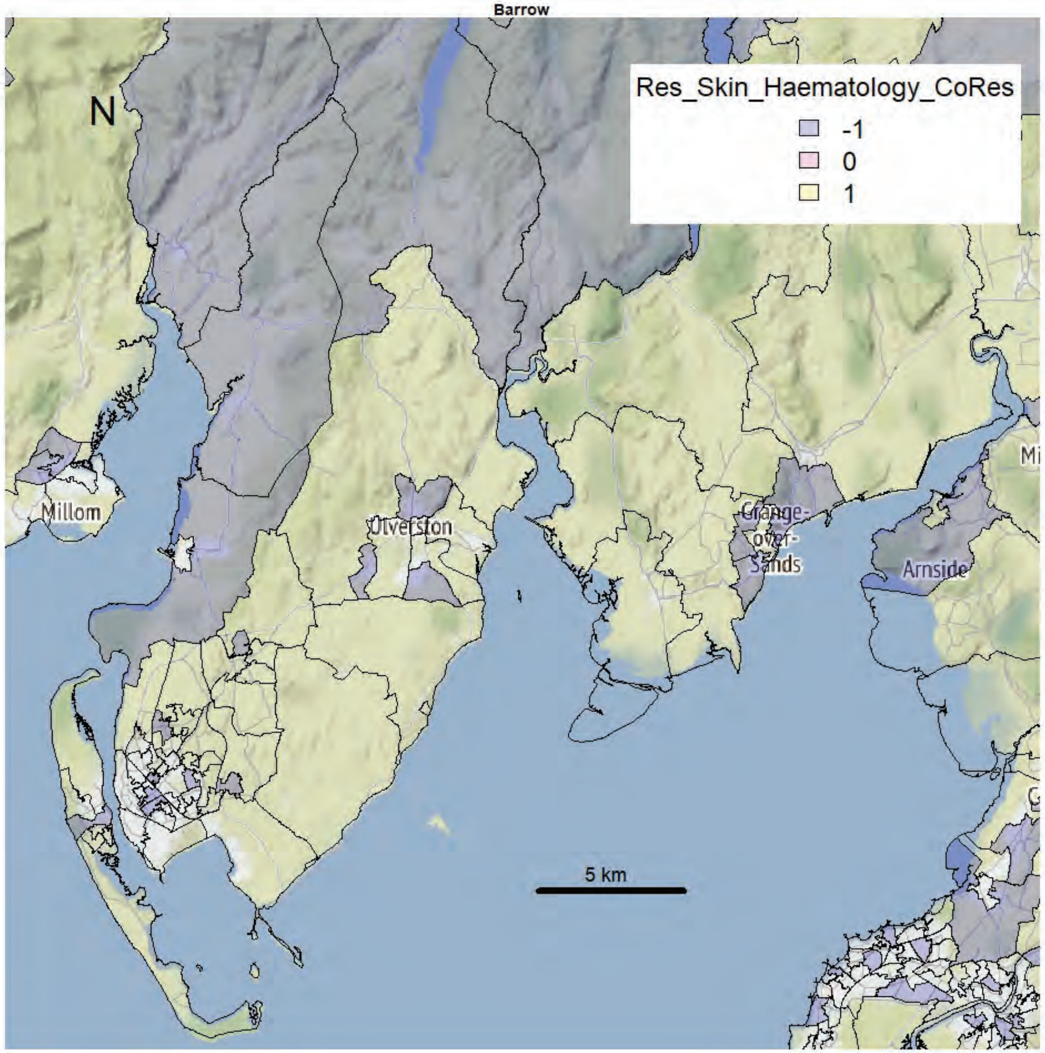
Barrow
N
Res_Skin_Haematology_CoRes
-1
0
1
Millom
Ulverston
Grange-over-Sands
Arnside
5 km

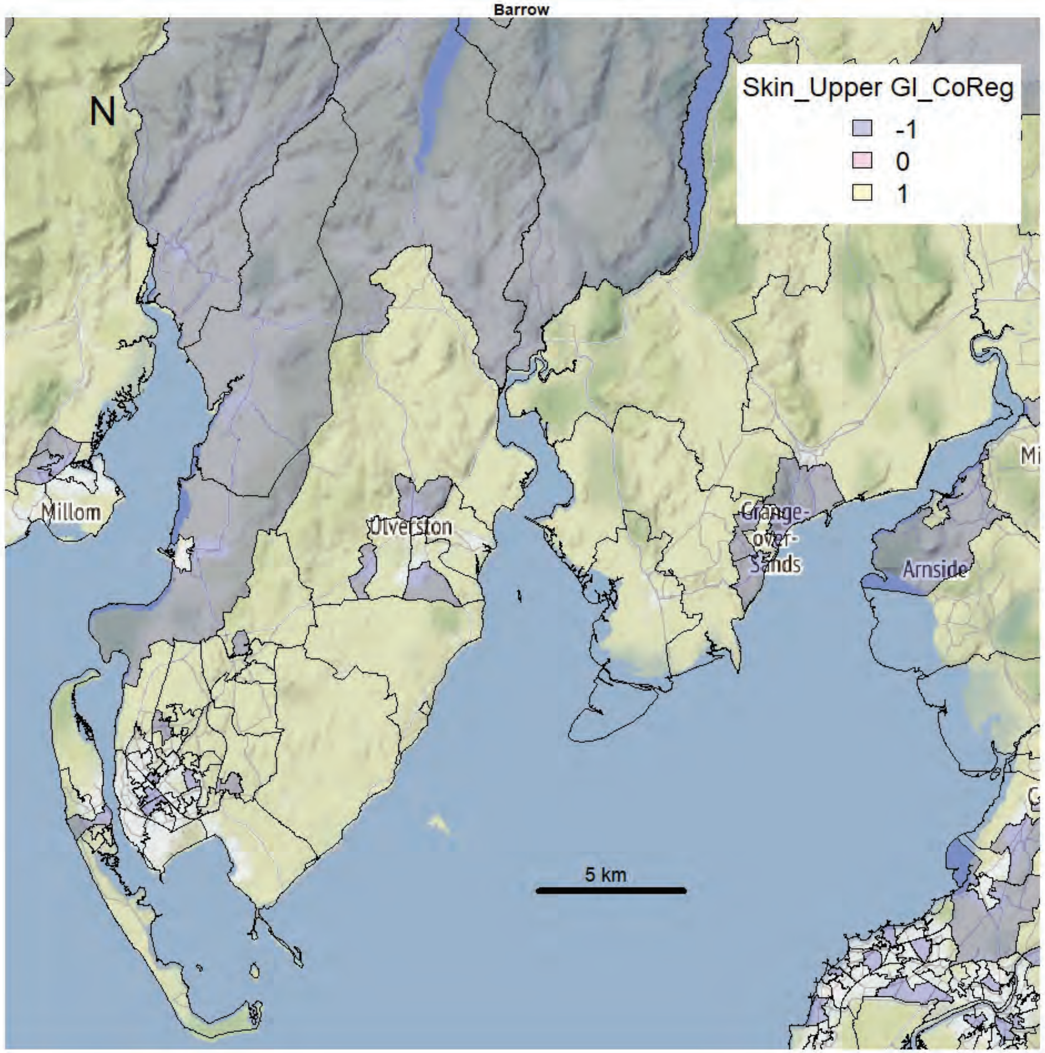

Barrow
N
Skin_Upper GI_CoReg
-1
0
1
Millom
Ulverston
Grange-over-Sands
Arnside
5 km

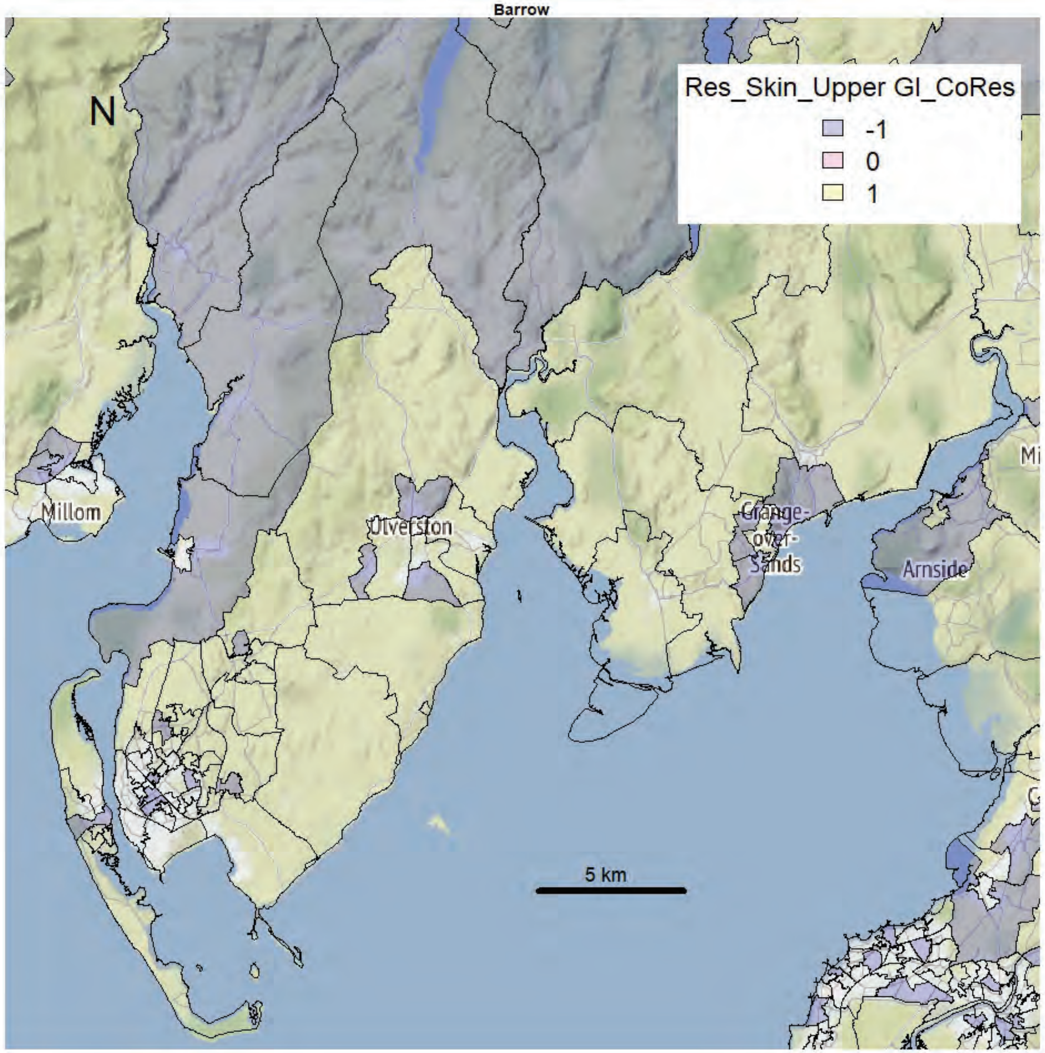

Barrow
N
Res_Skin_Upper GI_CoRes
-1
0
1
Millom
Ulverston
Grange-over-Sands
Arnside
5 km

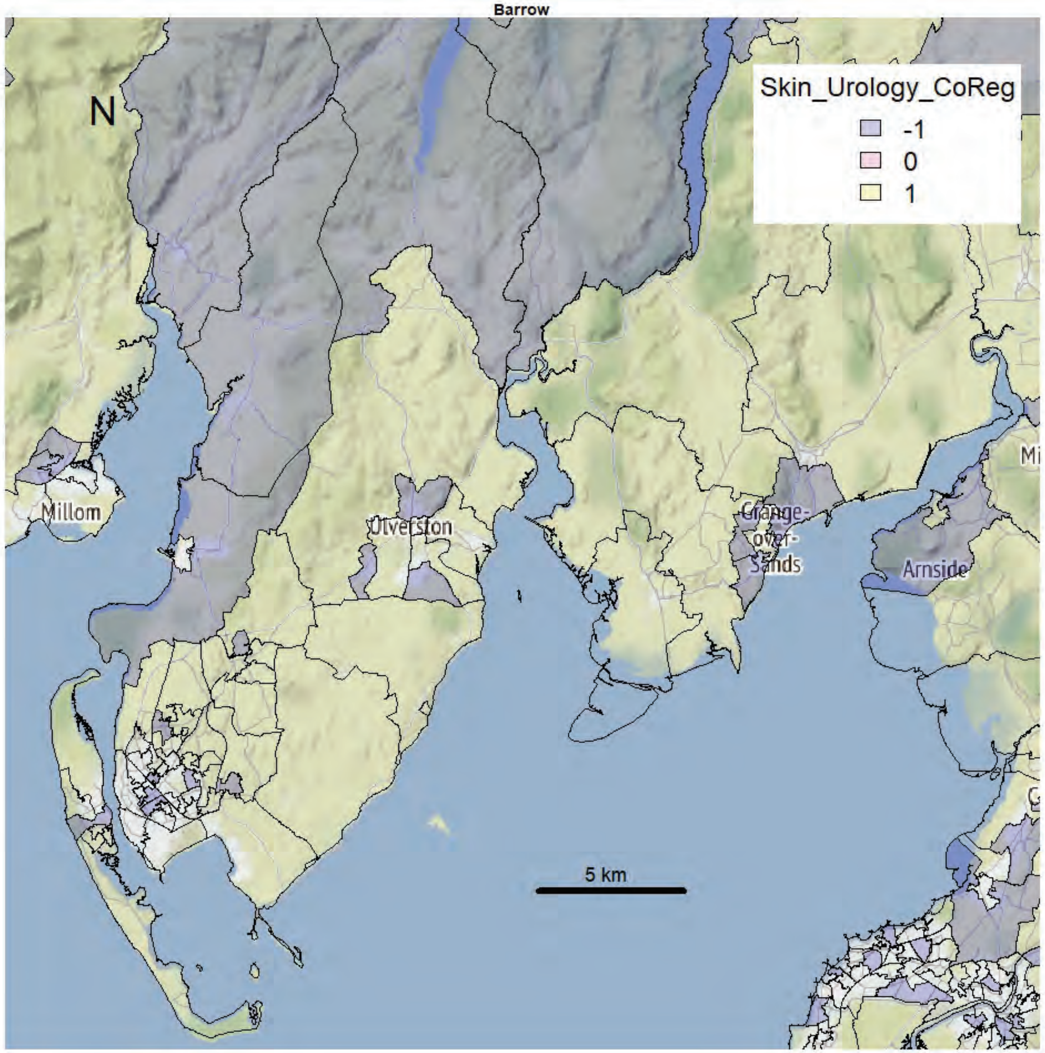
Barrow
N
Skin_Urology_CoReg
-1
0
1
Millom
Ulverston
Grange-
over-
Sands
Arnside
5 km

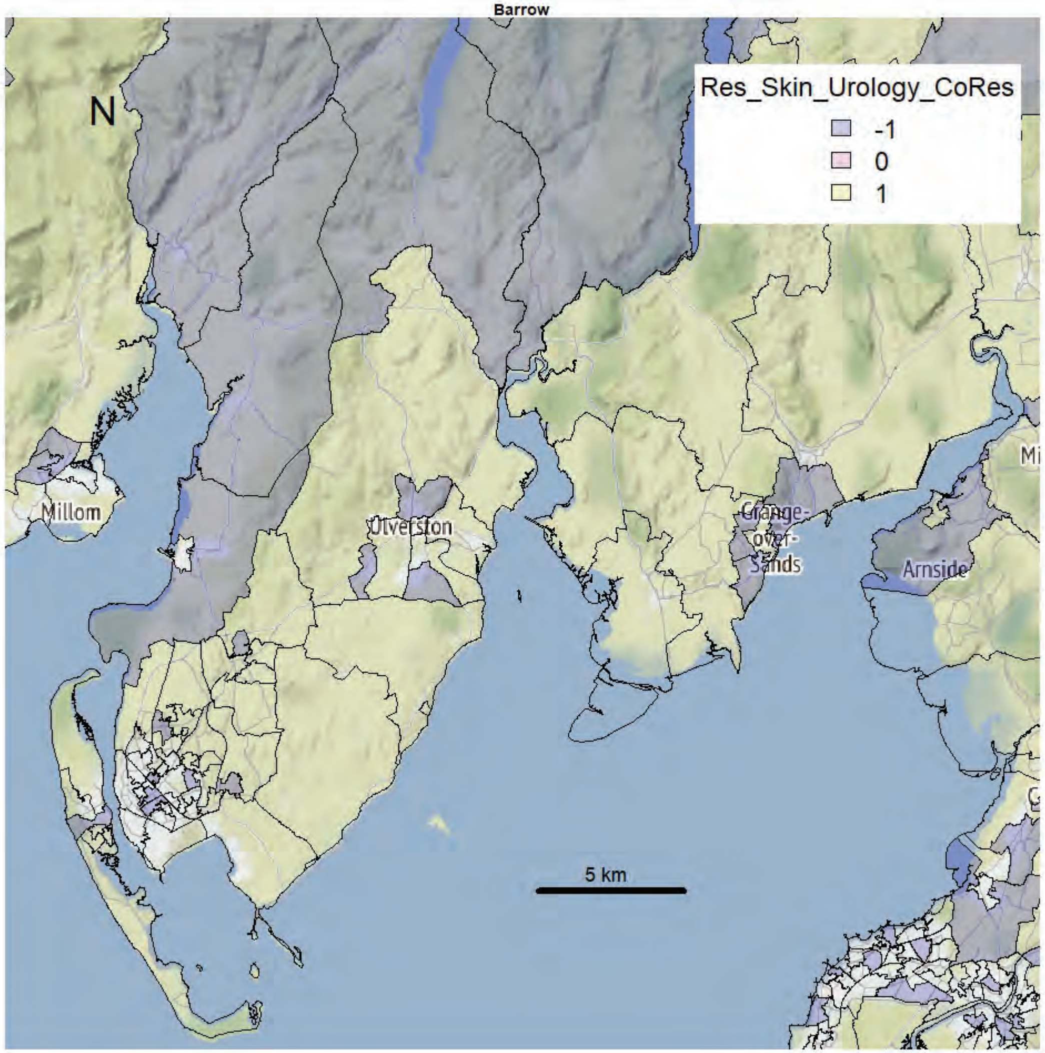
Barrow
N
Res_Skin_Urology_CoRes
-1
0
1
Millom
Ulverston
Grange-
over-
Sands
Arnside
5 km

Barrow

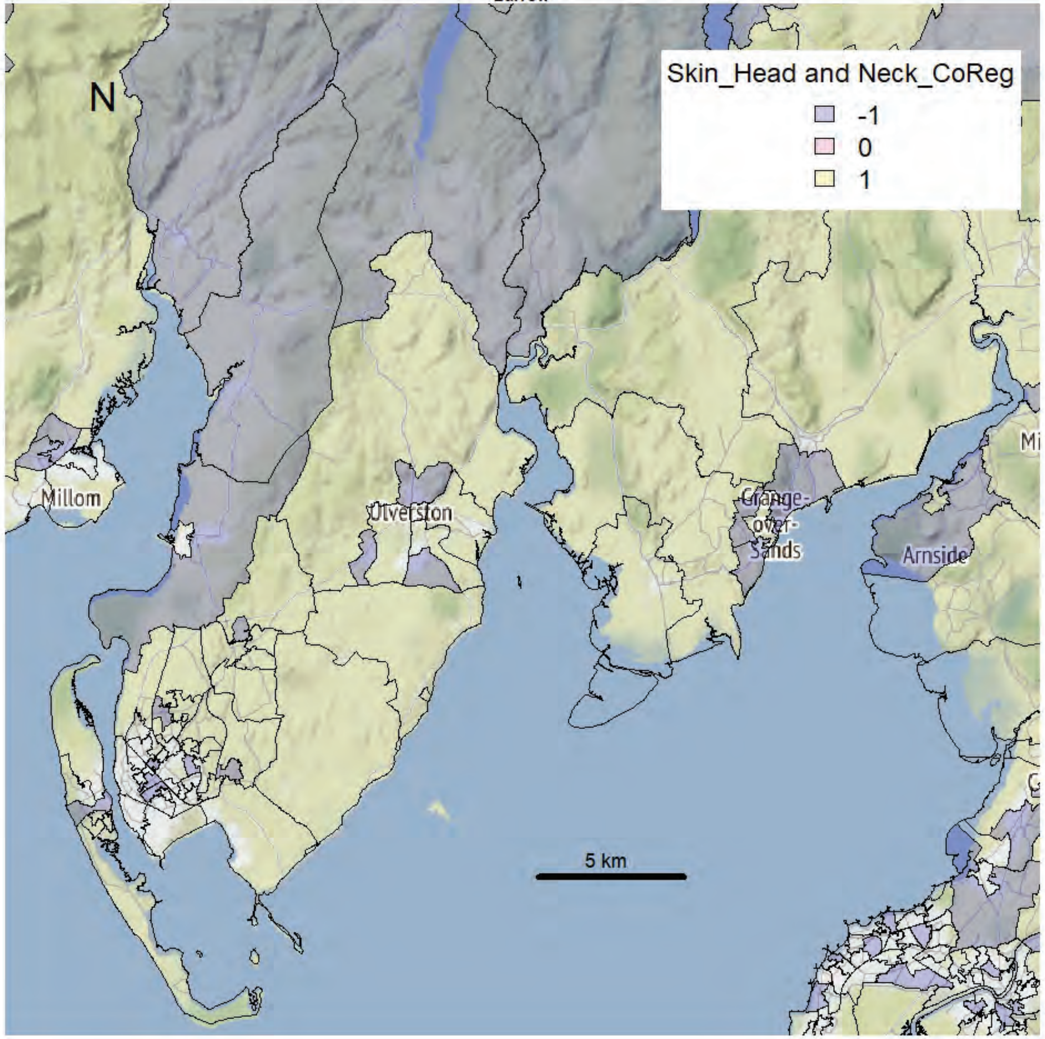

Barrow

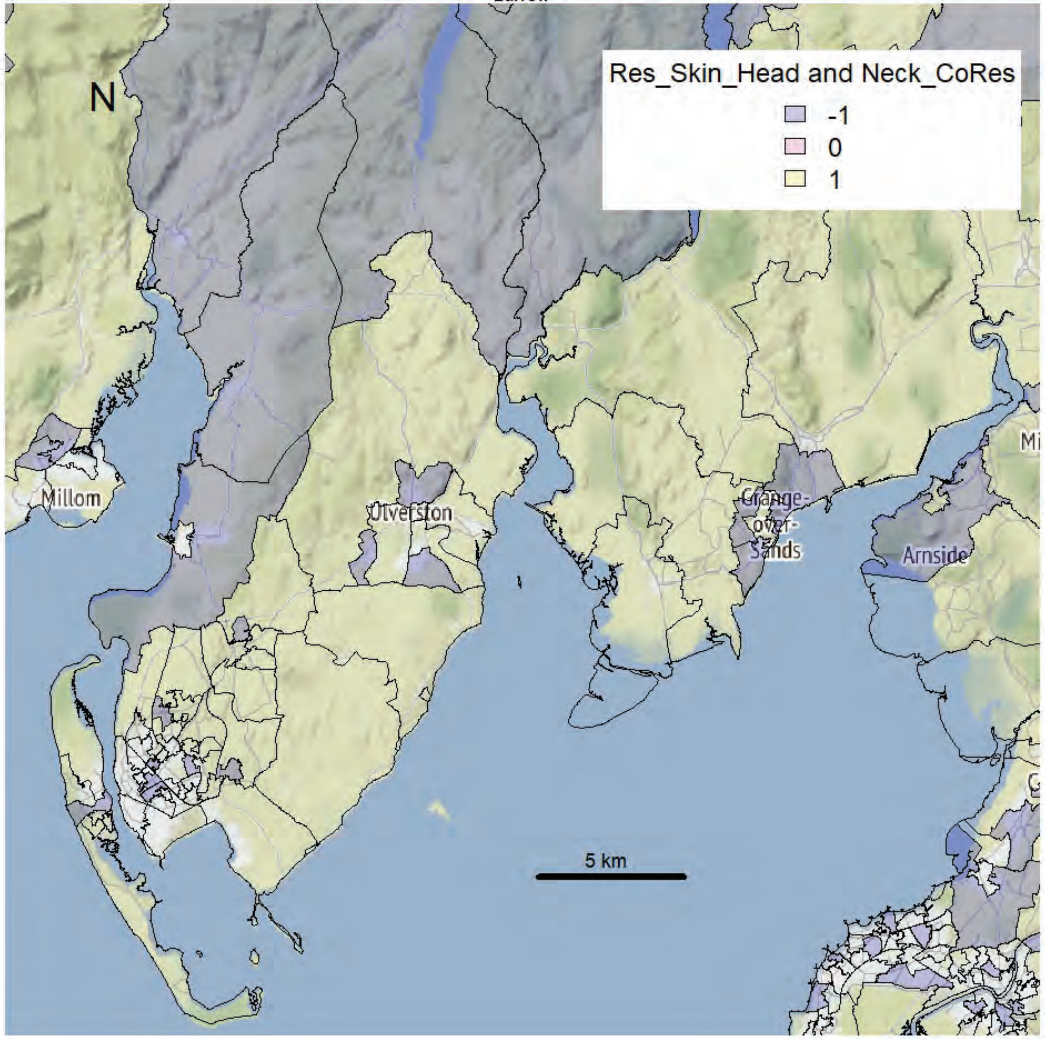

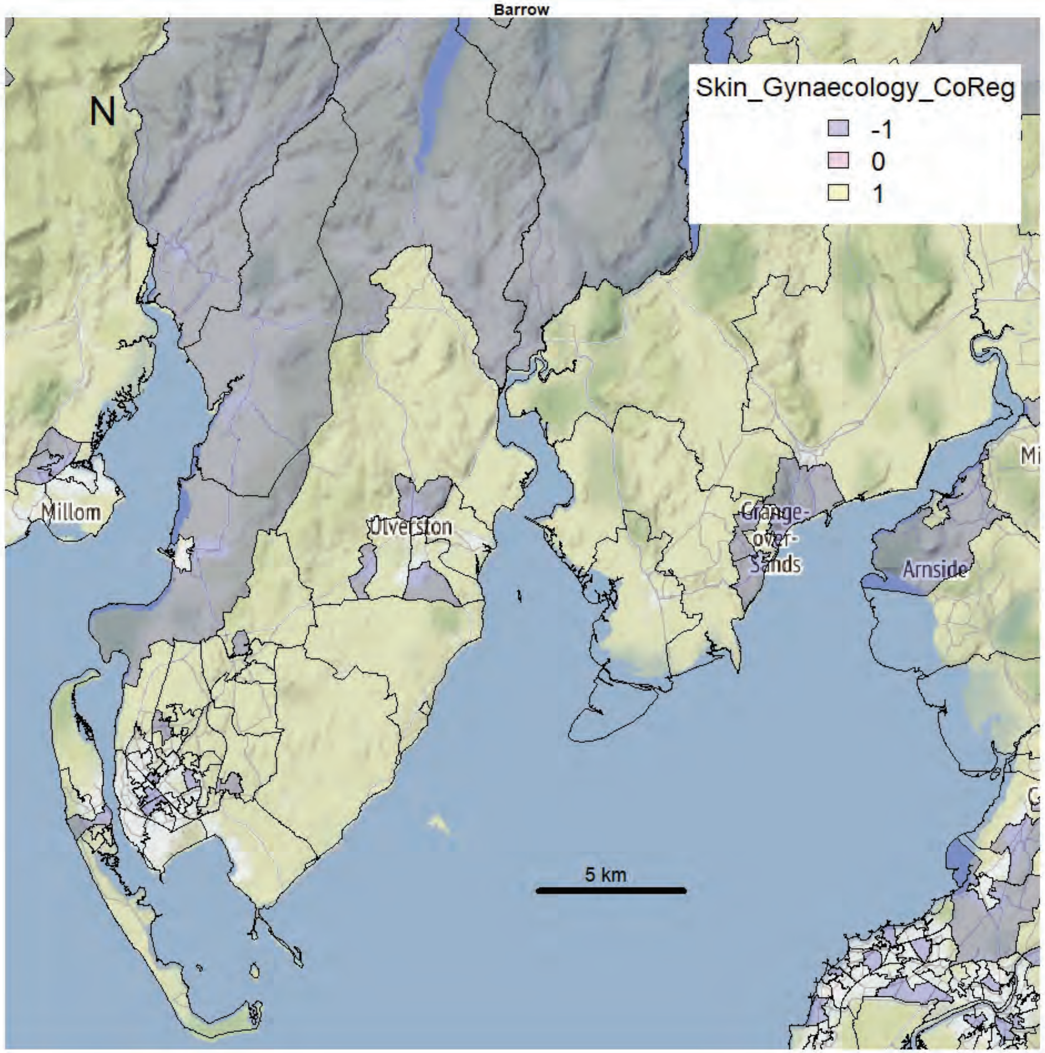
Barrow
N
Skin_Gynaecology_CoReg
-1
0
1
Millom
Ulverston
Grange-
over-
Sands
Arnside
5 km

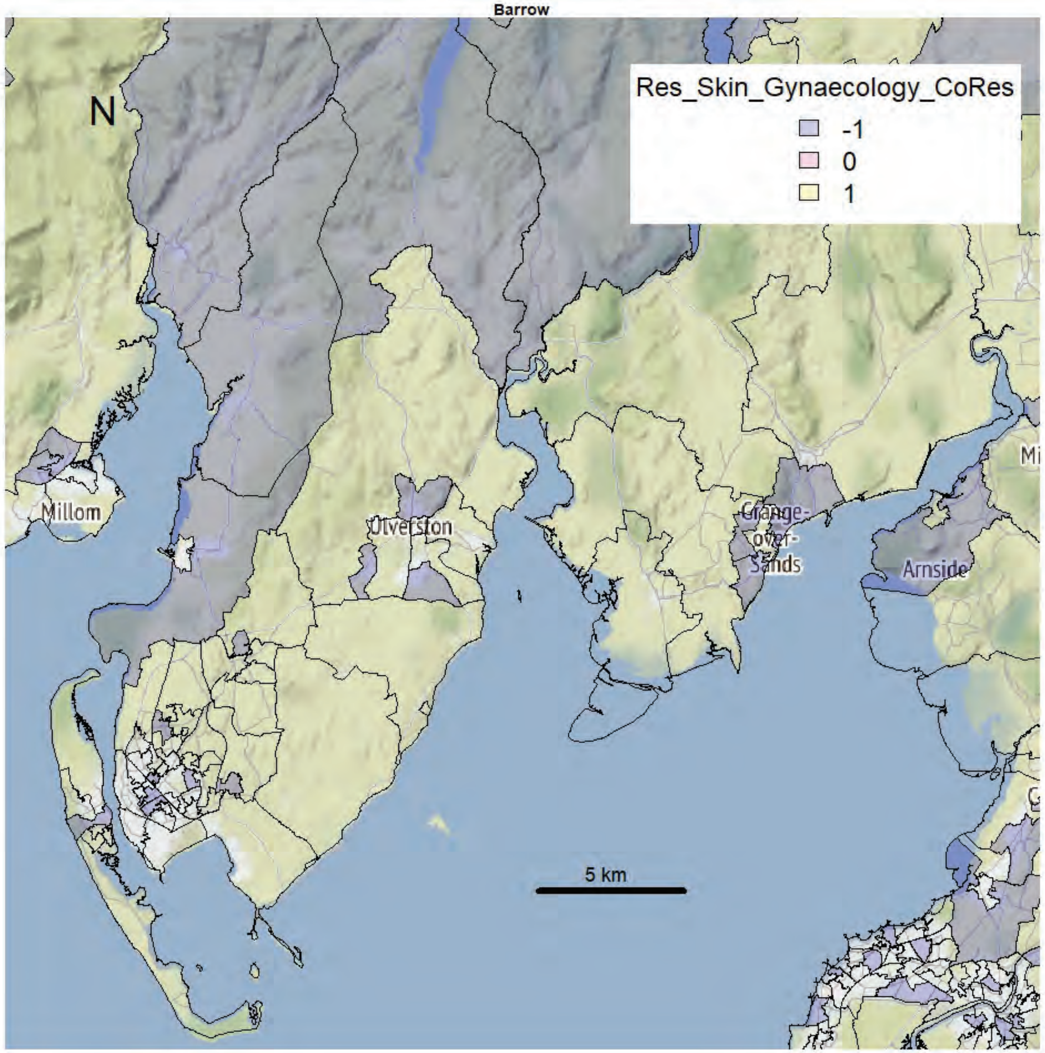
Barrow
N
Res_Skin_Gynaecology_CoRes
-1
0
1
Millom
Ulverston
Grange-
over-
Sands
Arnside
5 km

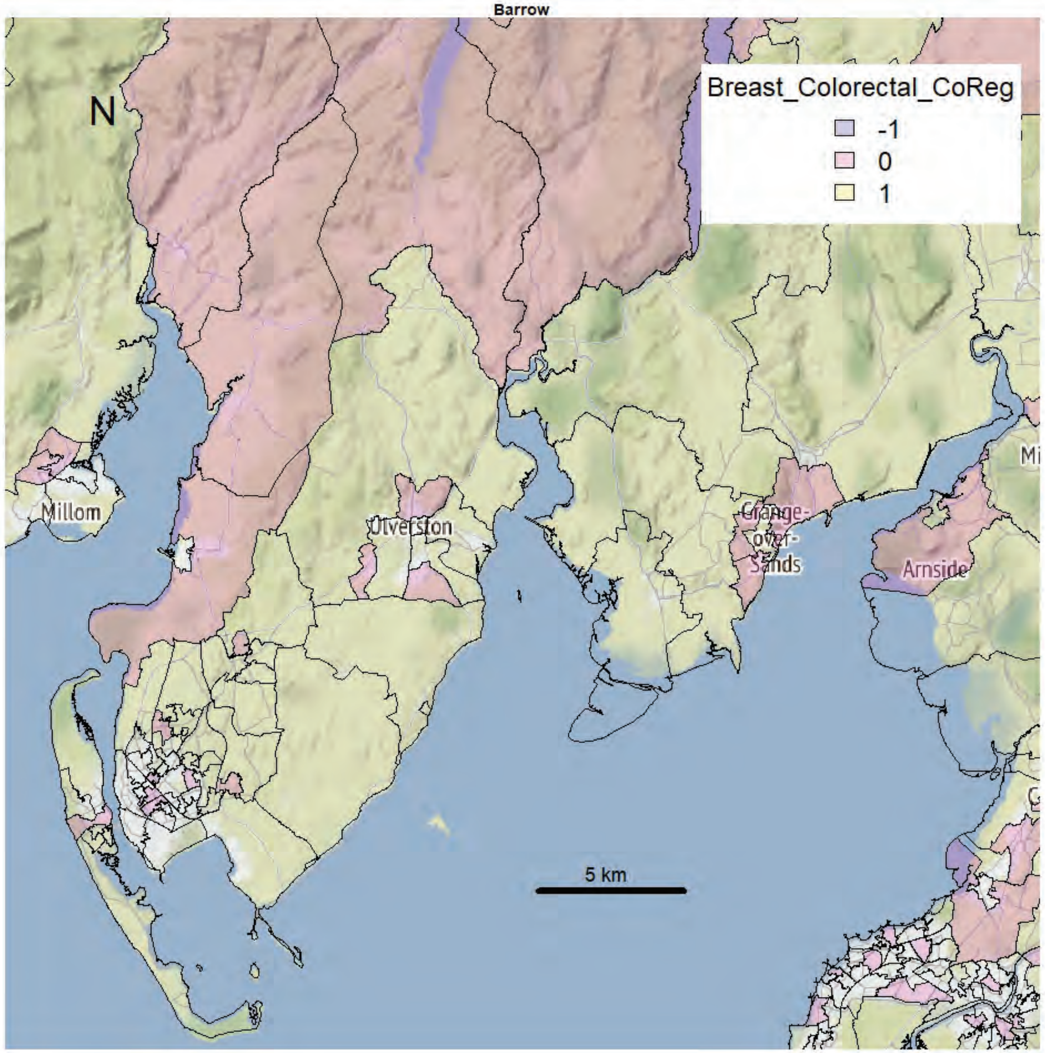
Barrow
N
Breast_Colorectal_CoReg
-1
0
1
Millom
Ulverston
Grange-
over-
Sands
Arnside
5 km

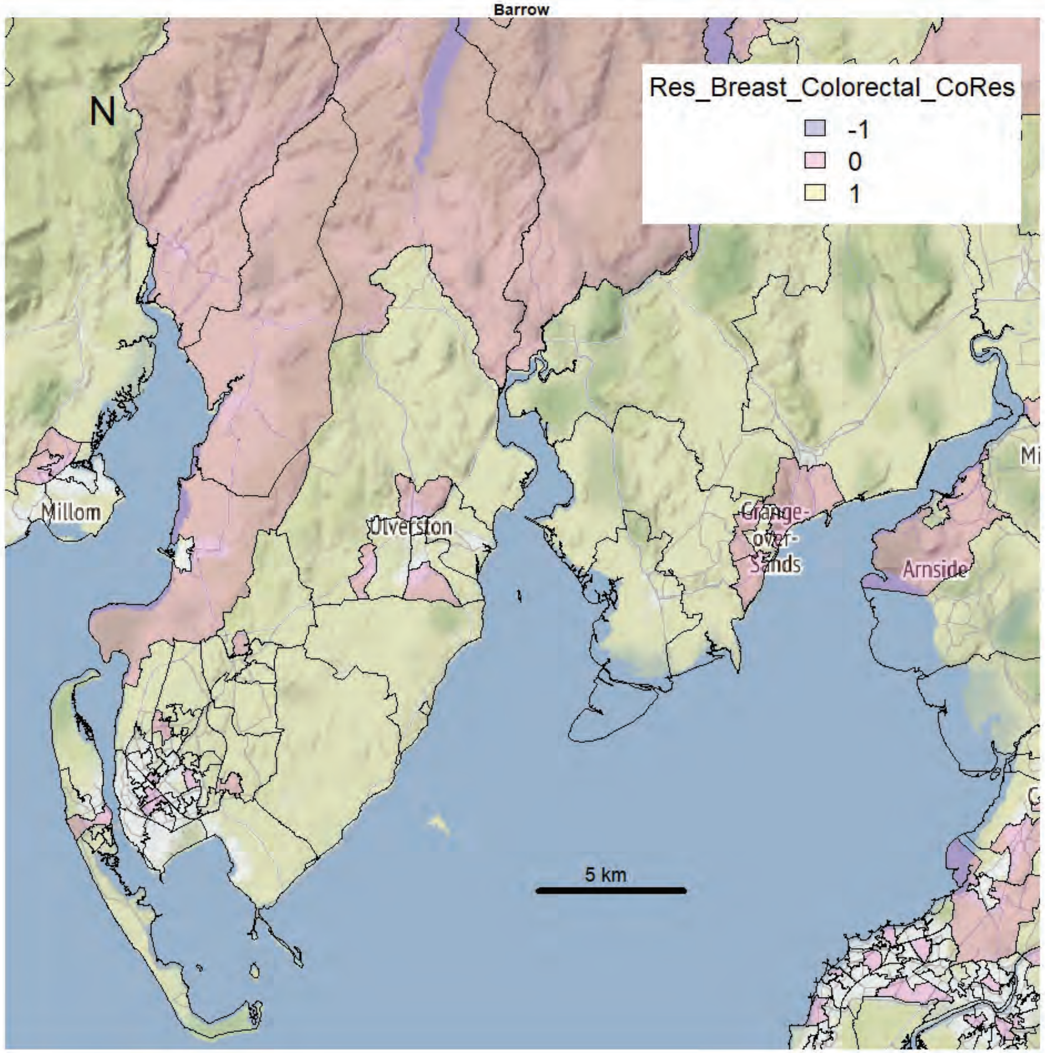
Barrow
N
Res_Breast_Colorectal_CoRes
-1
0
1
Millom
Ulverston
Grange-
over-
Sands
Arnside
5 km

Barrow

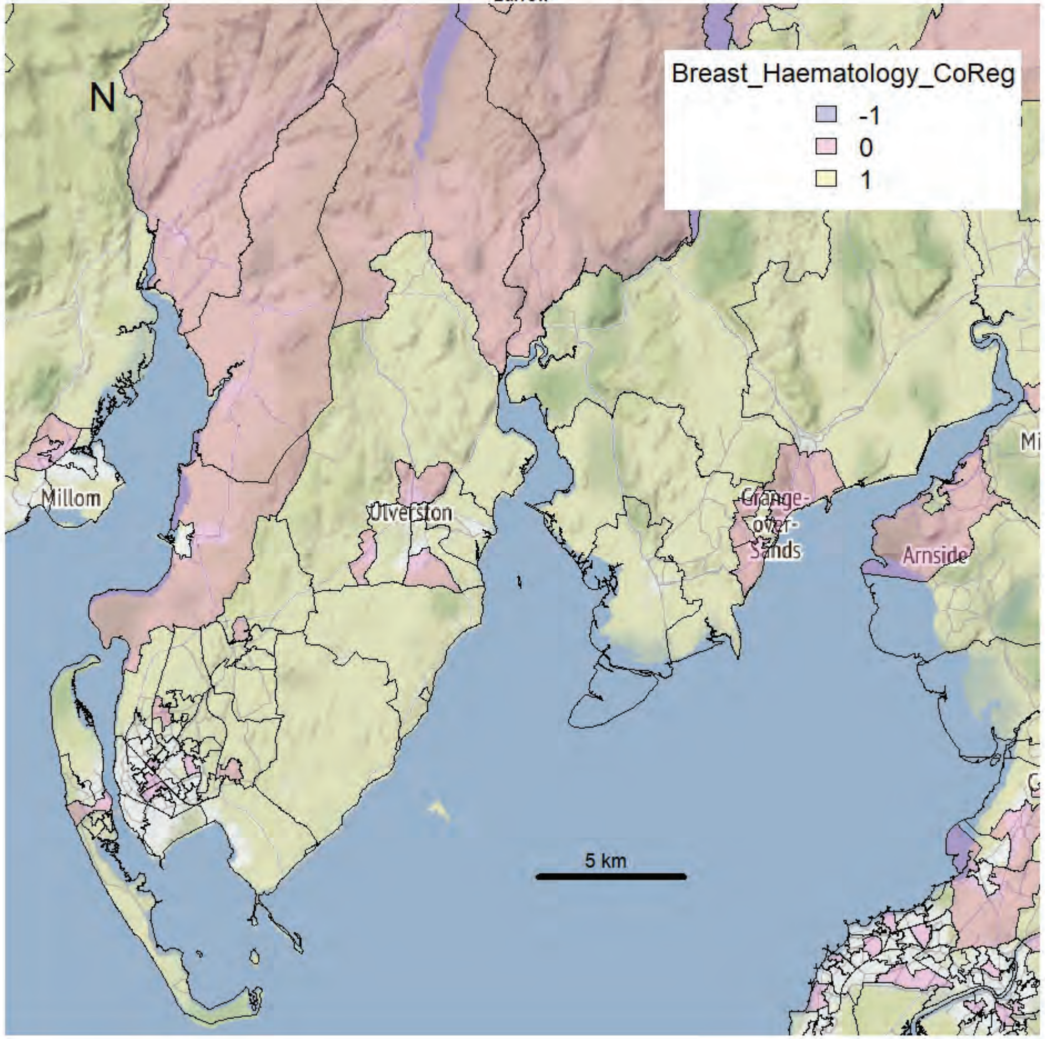

Barrow

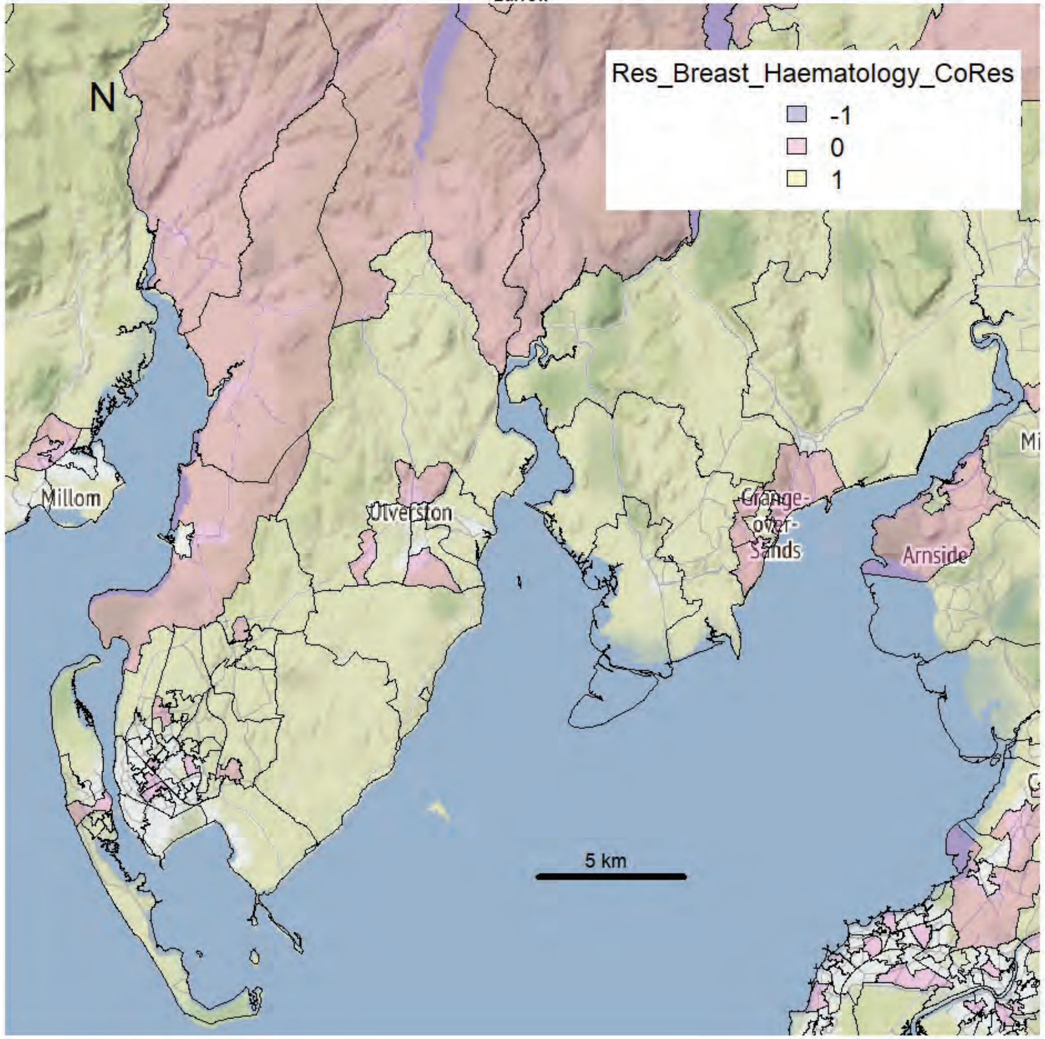

Barrow

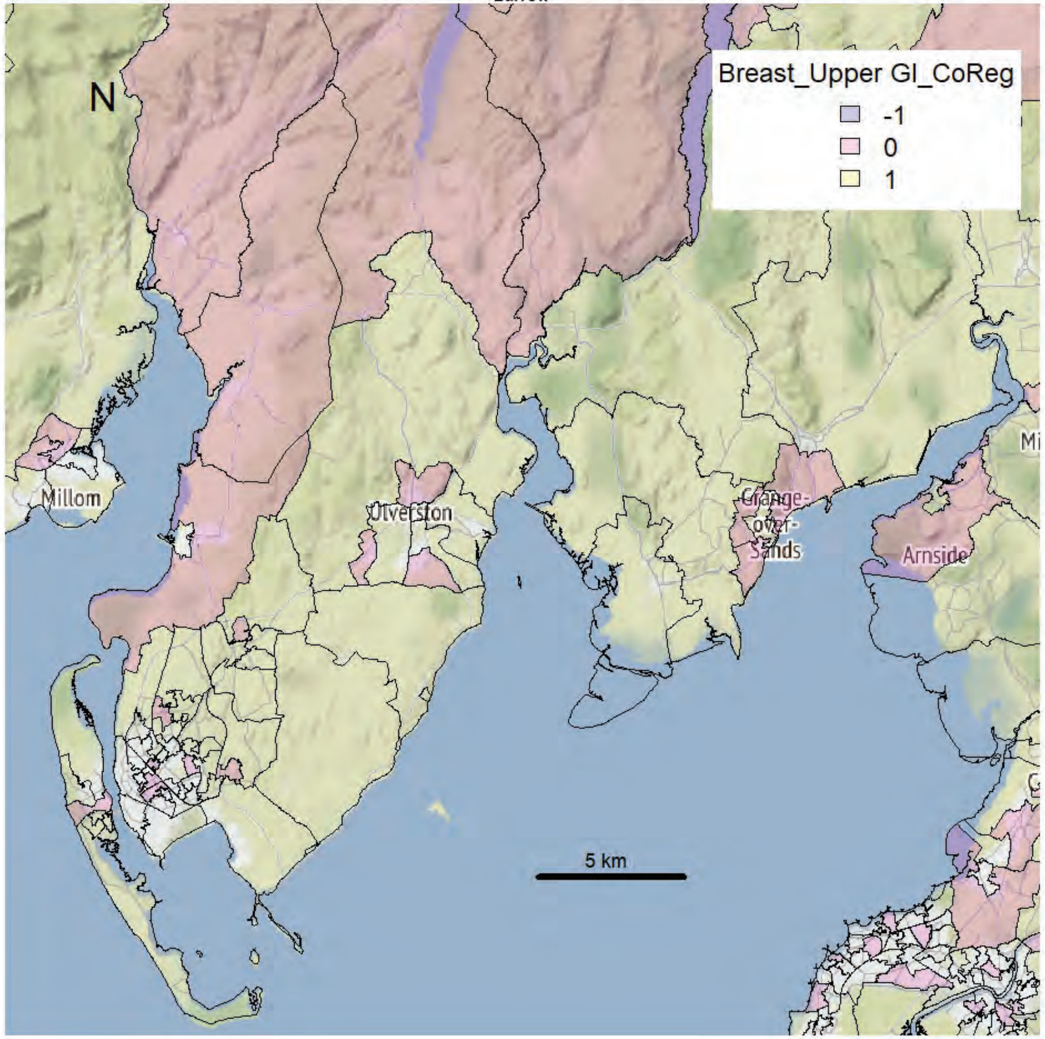

Barrow

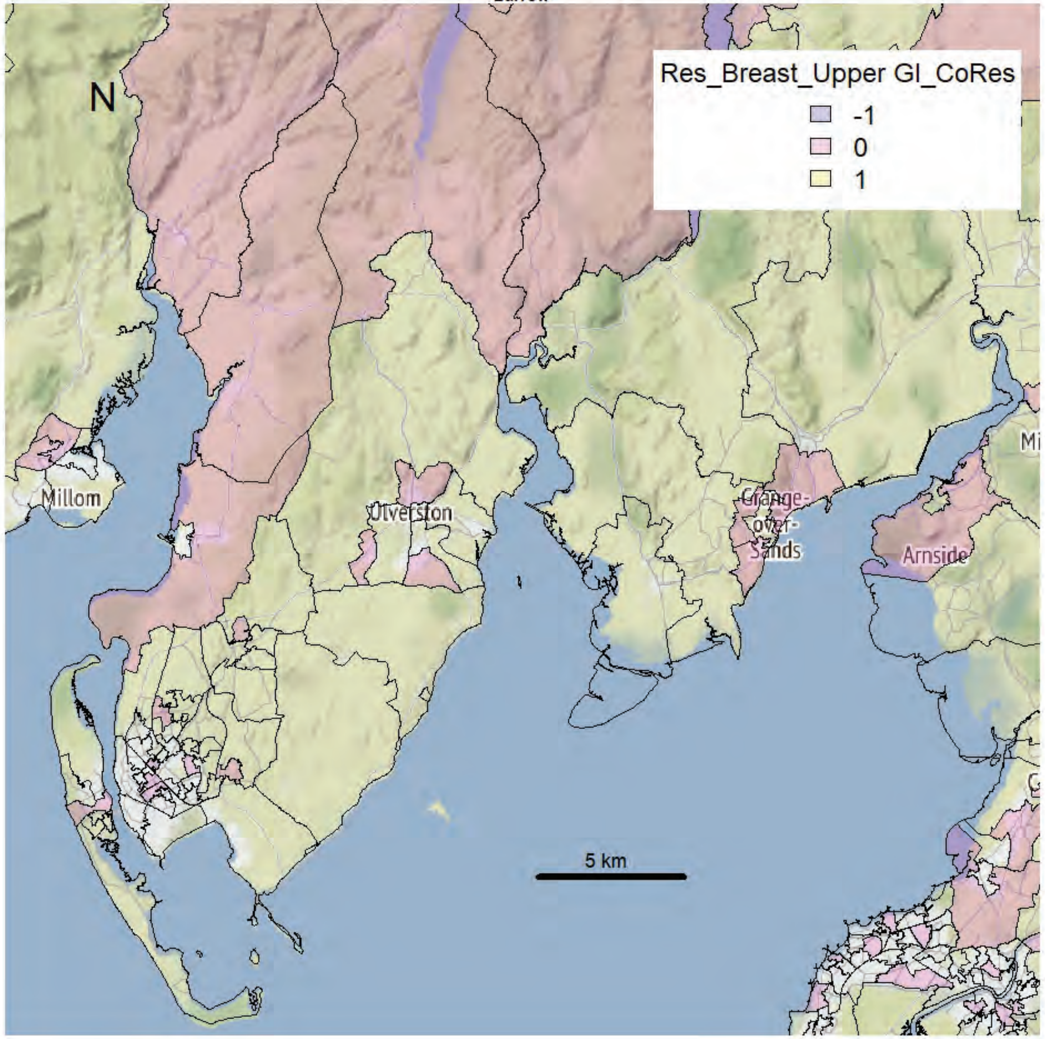

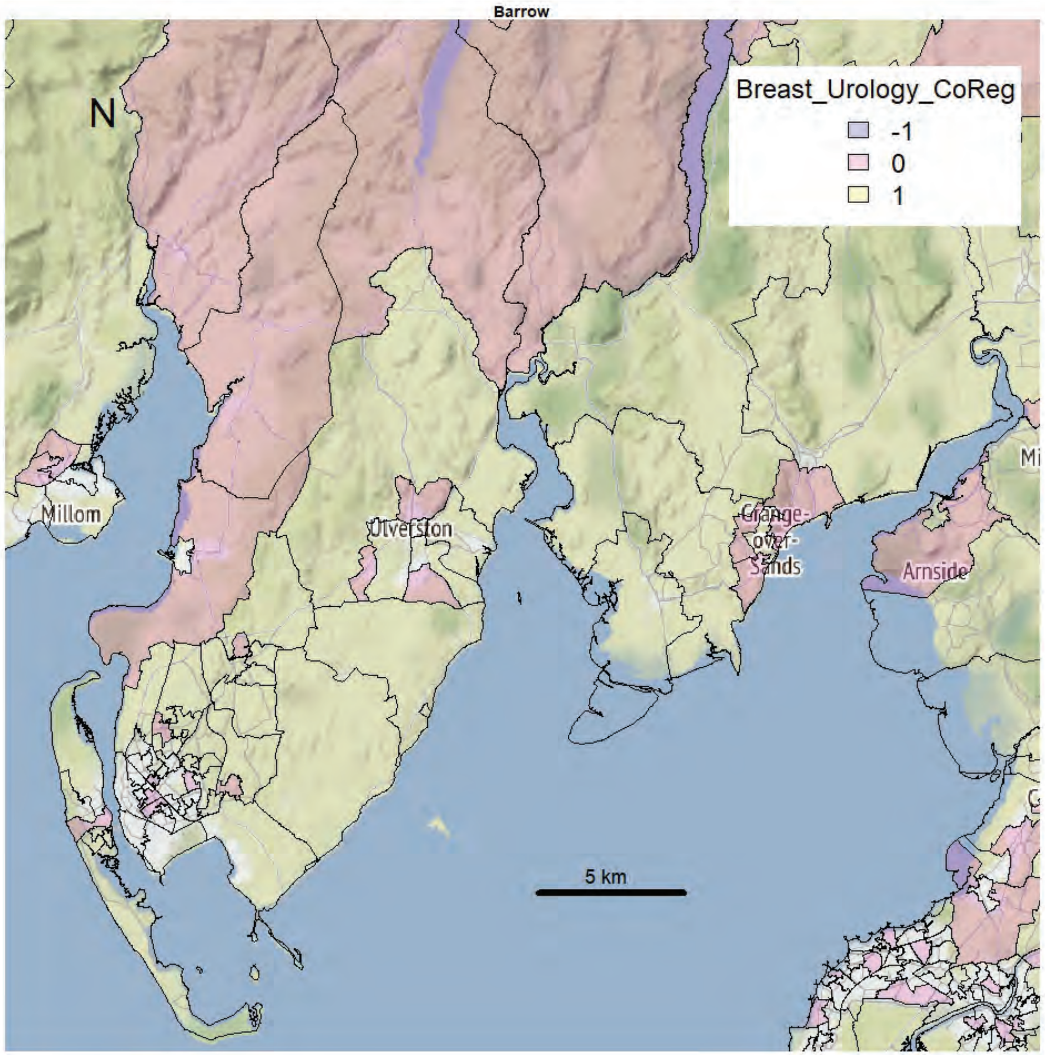
Barrow
Breast_Urology_CoReg
-1
0
1
N
Millom
Ulverston
Grange-
over-
Sands
Arnside
5 km

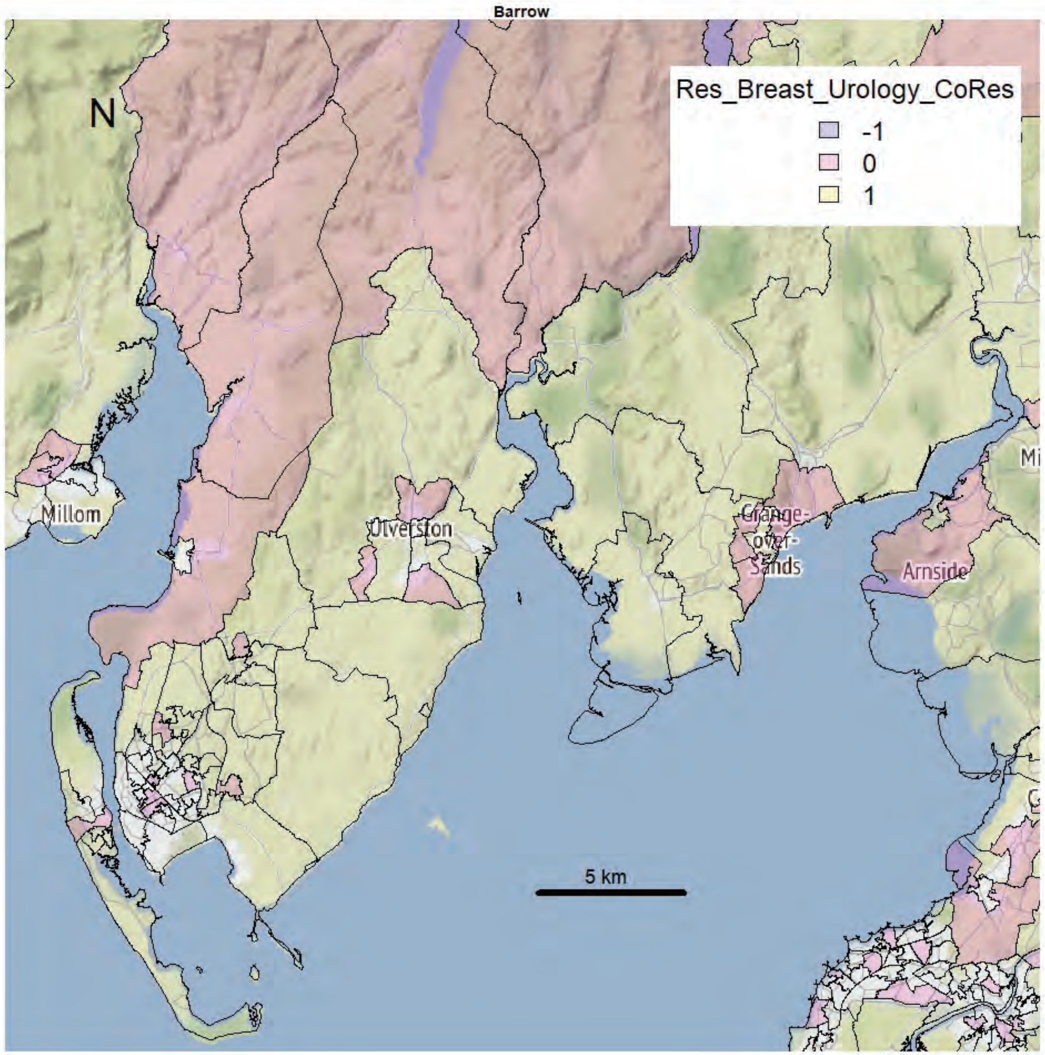
Barrow
Res_Breast_Urology_CoRes
-1
0
1
N
Millom
Ulverston
Grange-
over-
Sands
Arnside
5 km

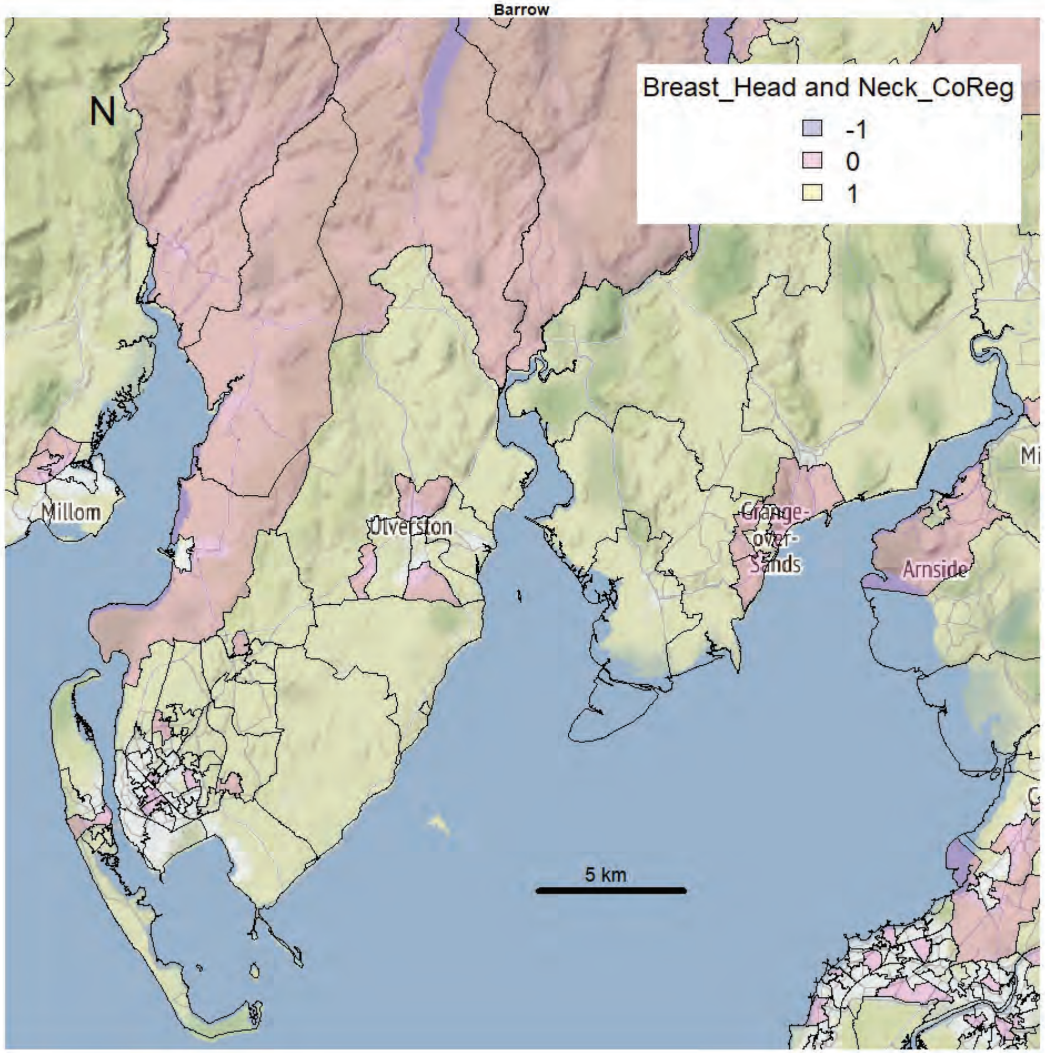

Barrow
N
Breast_Head and Neck_CoReg
-1
0
1
Millom
Ulverston
Grange-over-Sands
Arnside
5 km

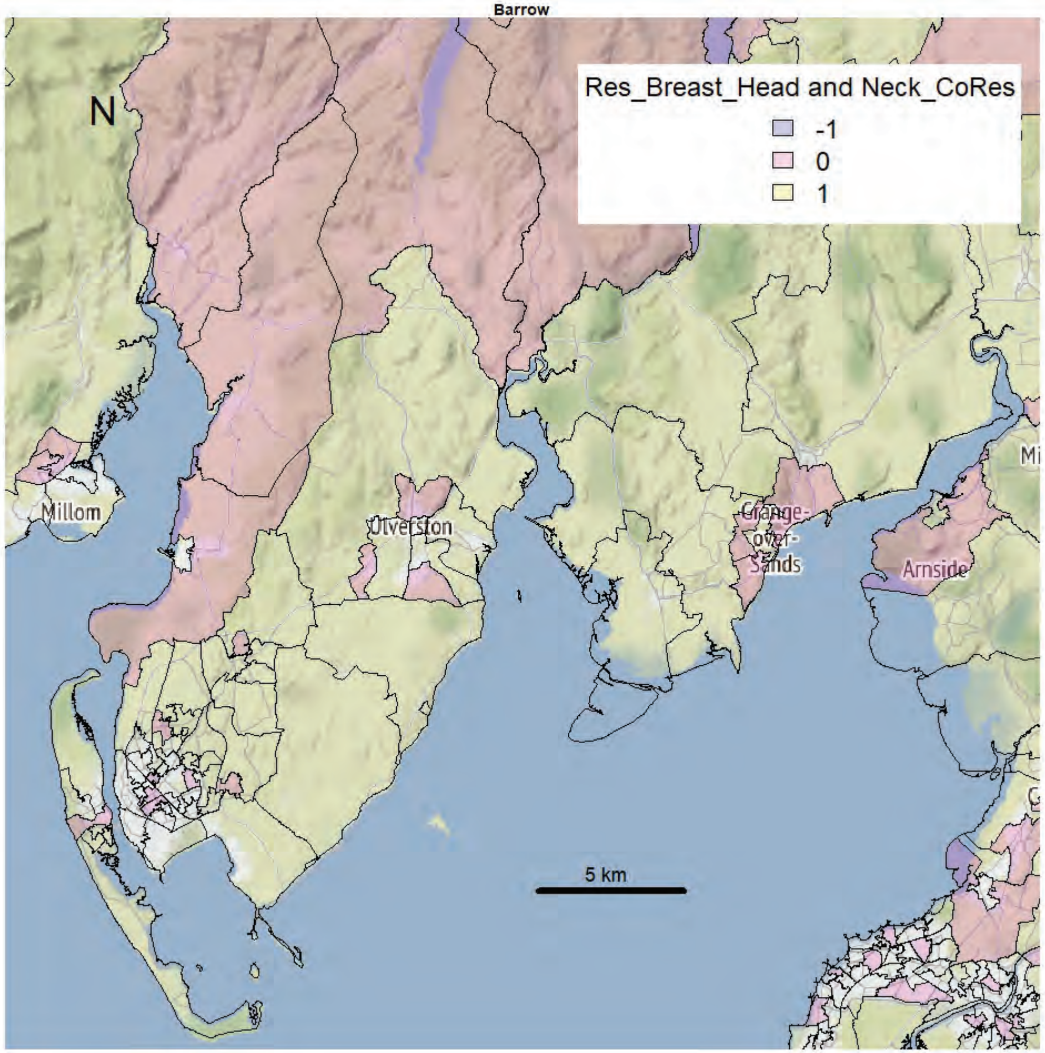

Barrow
N
Res_Breast_Head and Neck_CoRes
-1
0
1
Millom
Ulverston
Grange-over-Sands
Arnside
5 km

Barrow

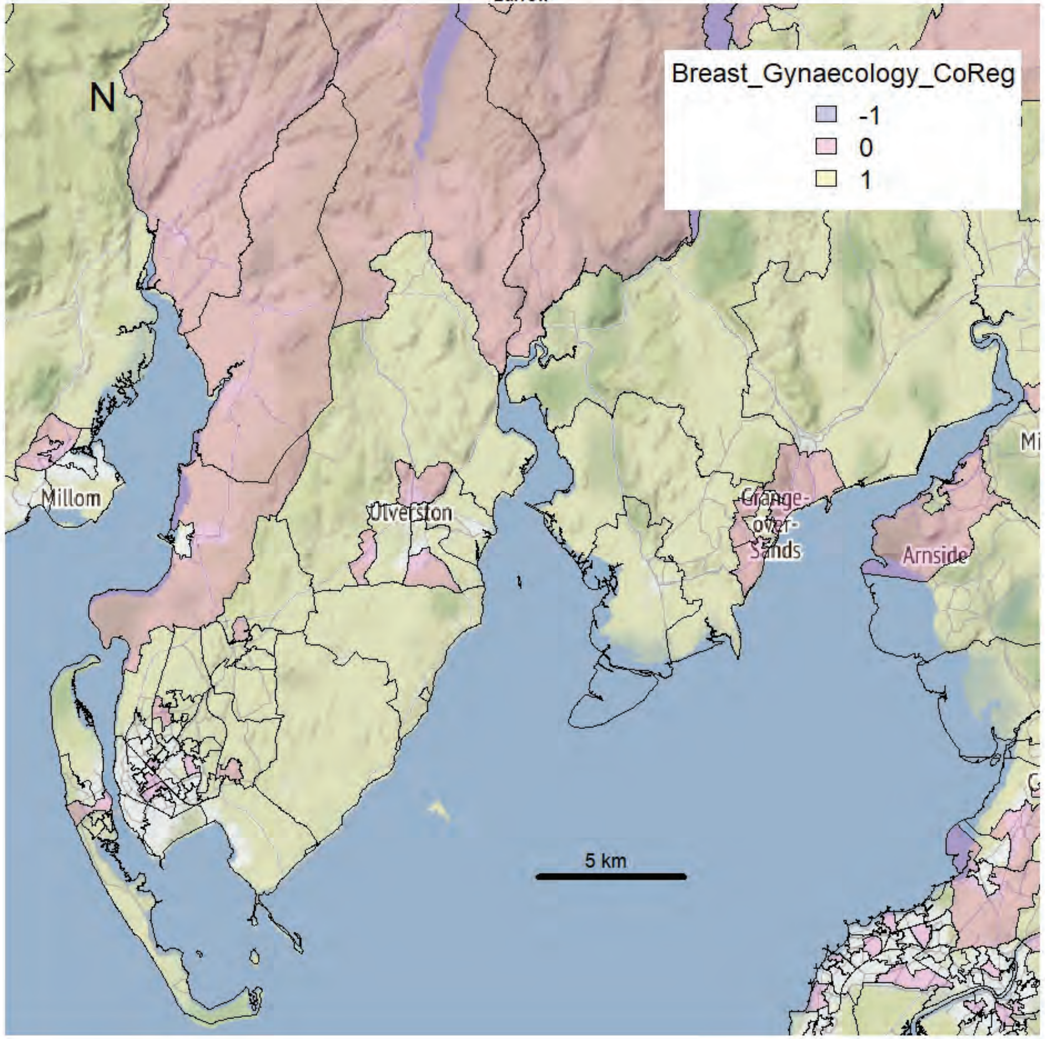

Barrow

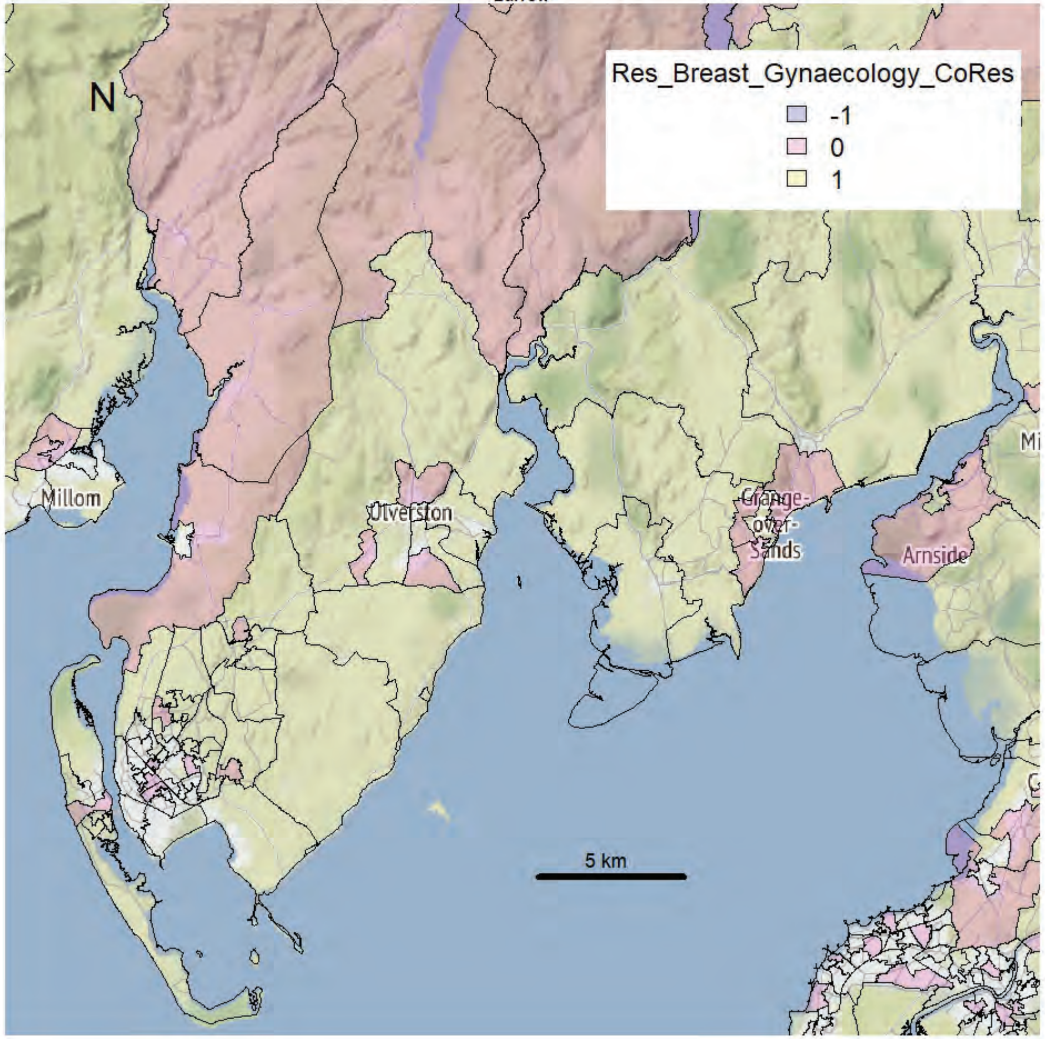

Barrow

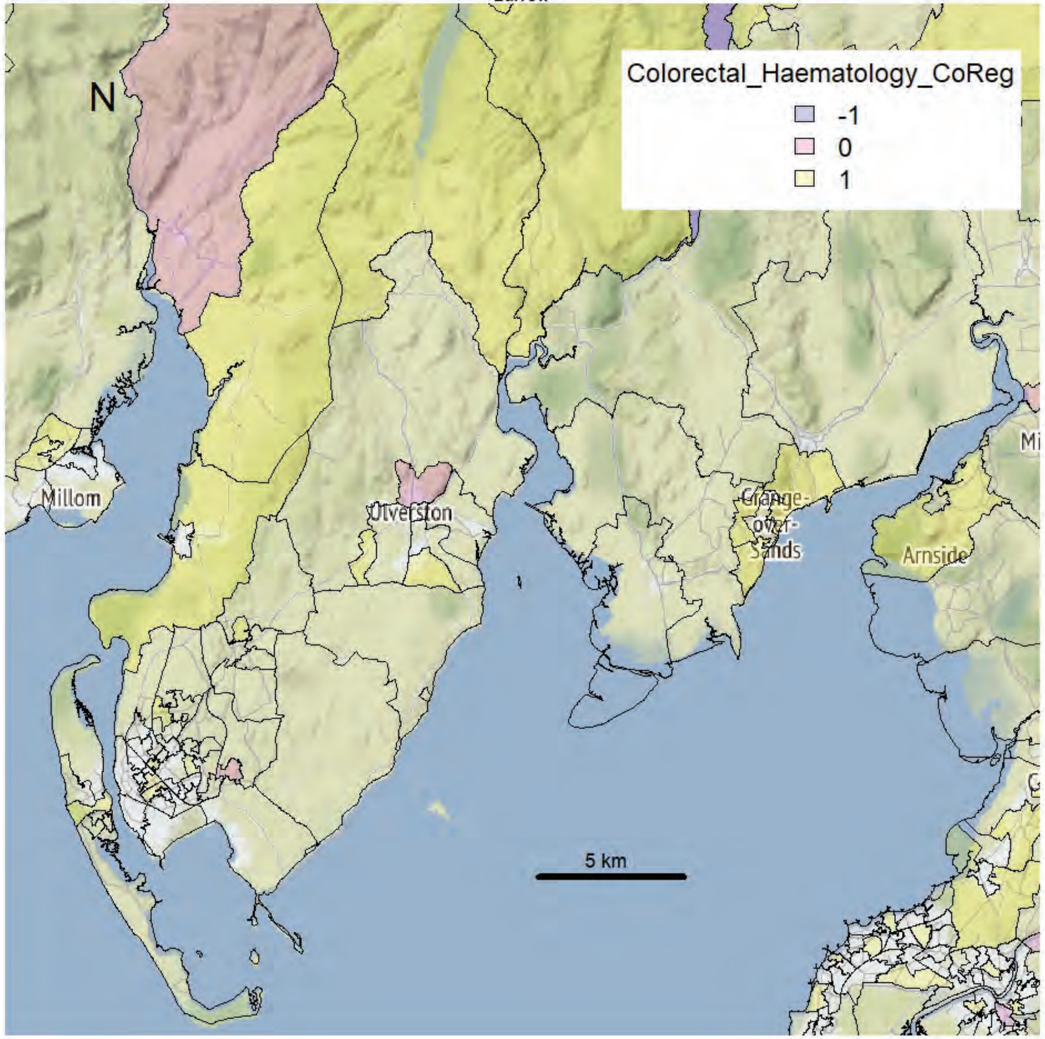

Barrow

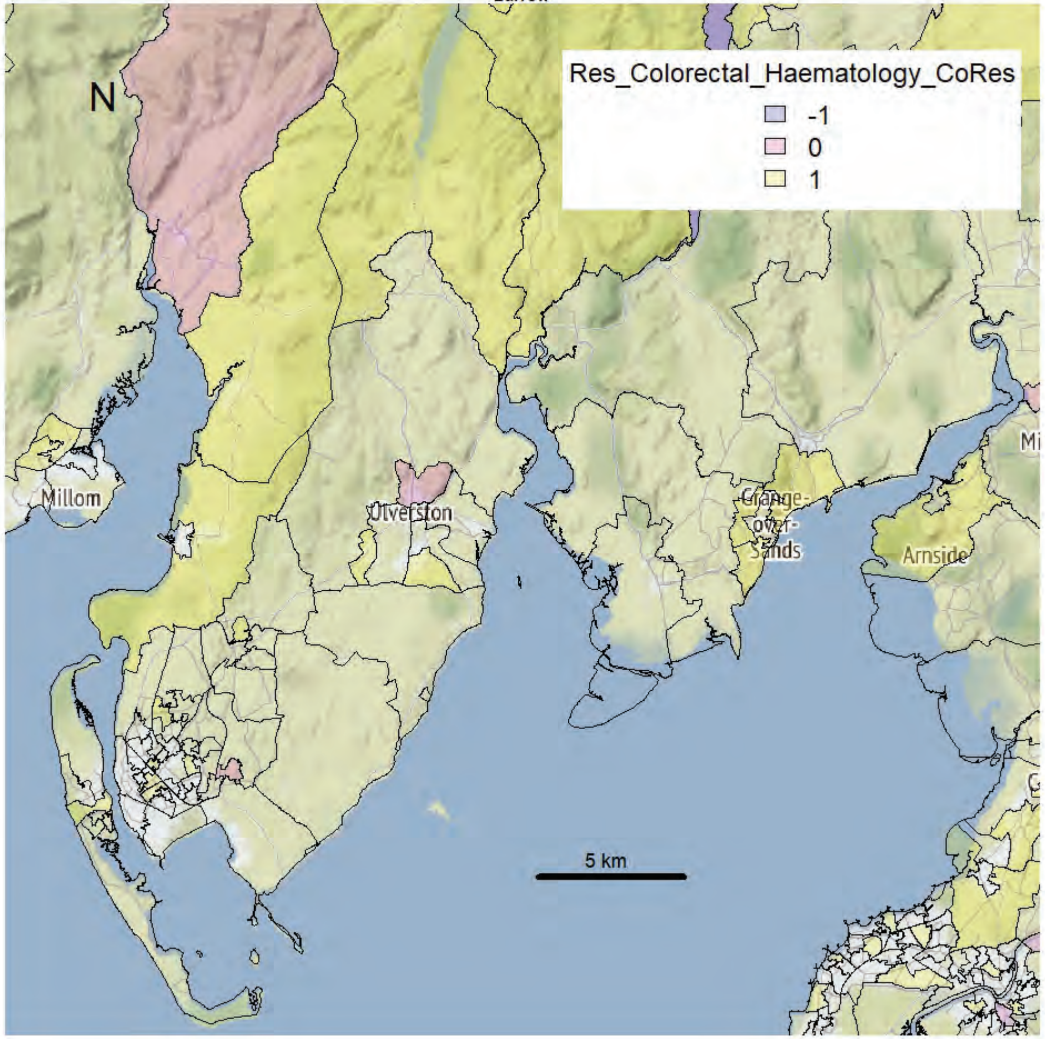

Barrow

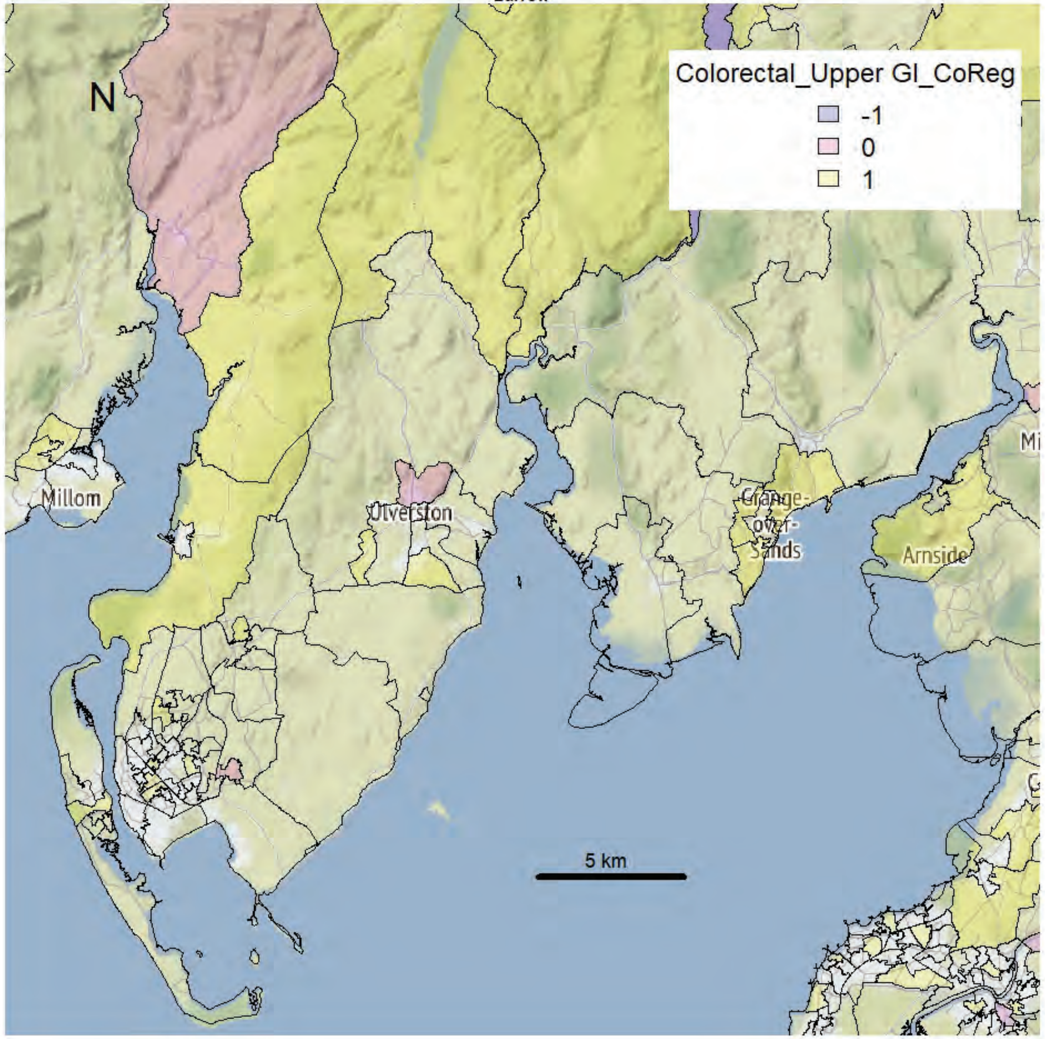

Barrow

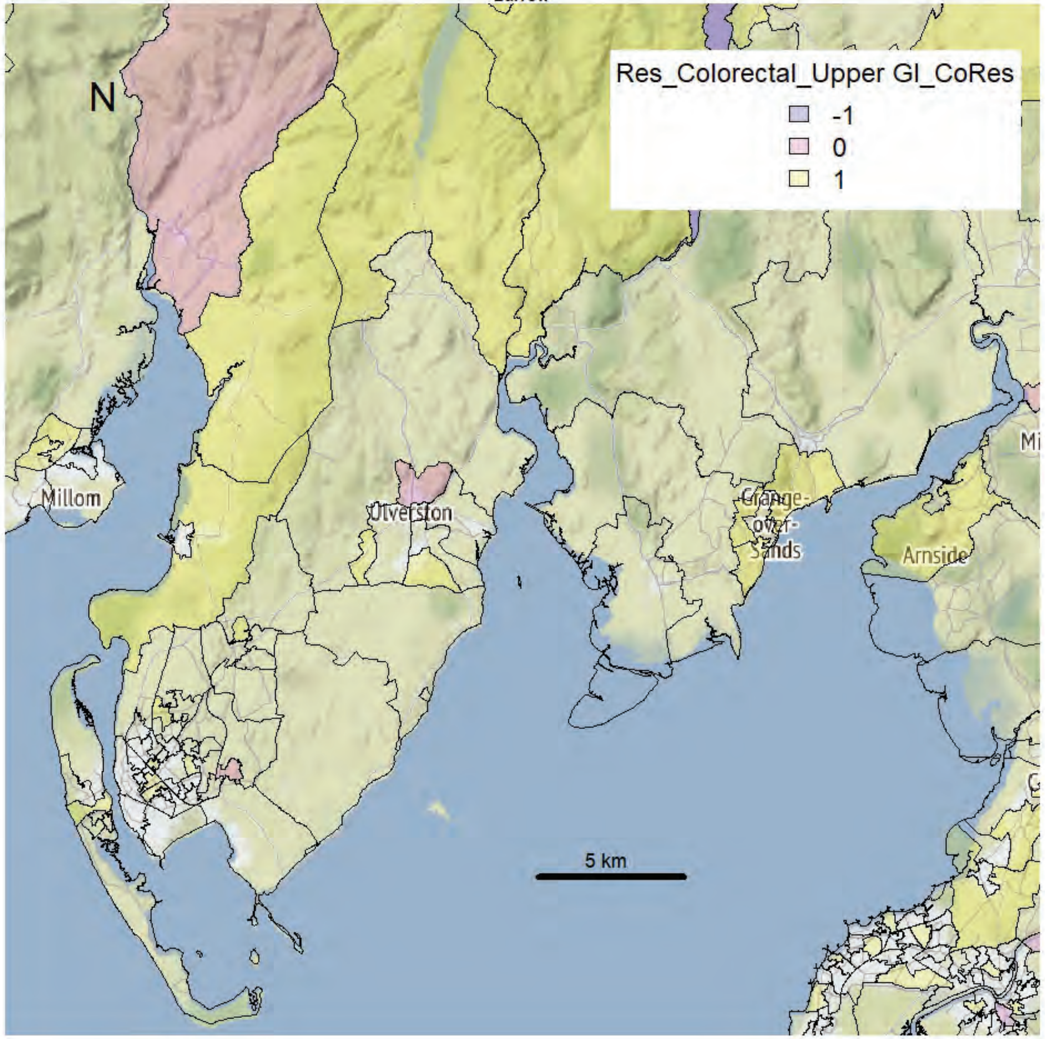

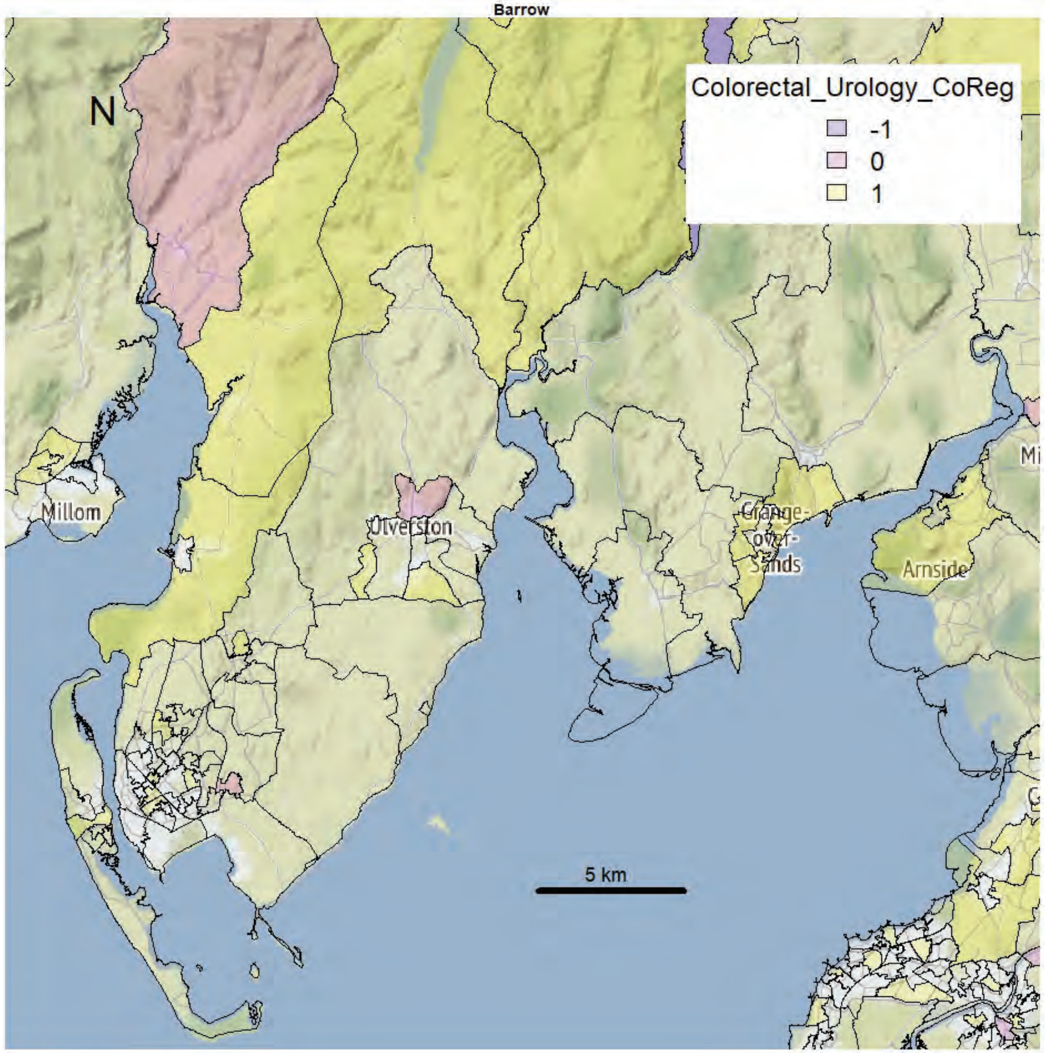
Barrow
N
Colorectal_Urology_CoReg
-1
0
1
Millom
Ulverston
Grange-over-Sands
Arnside
5 km

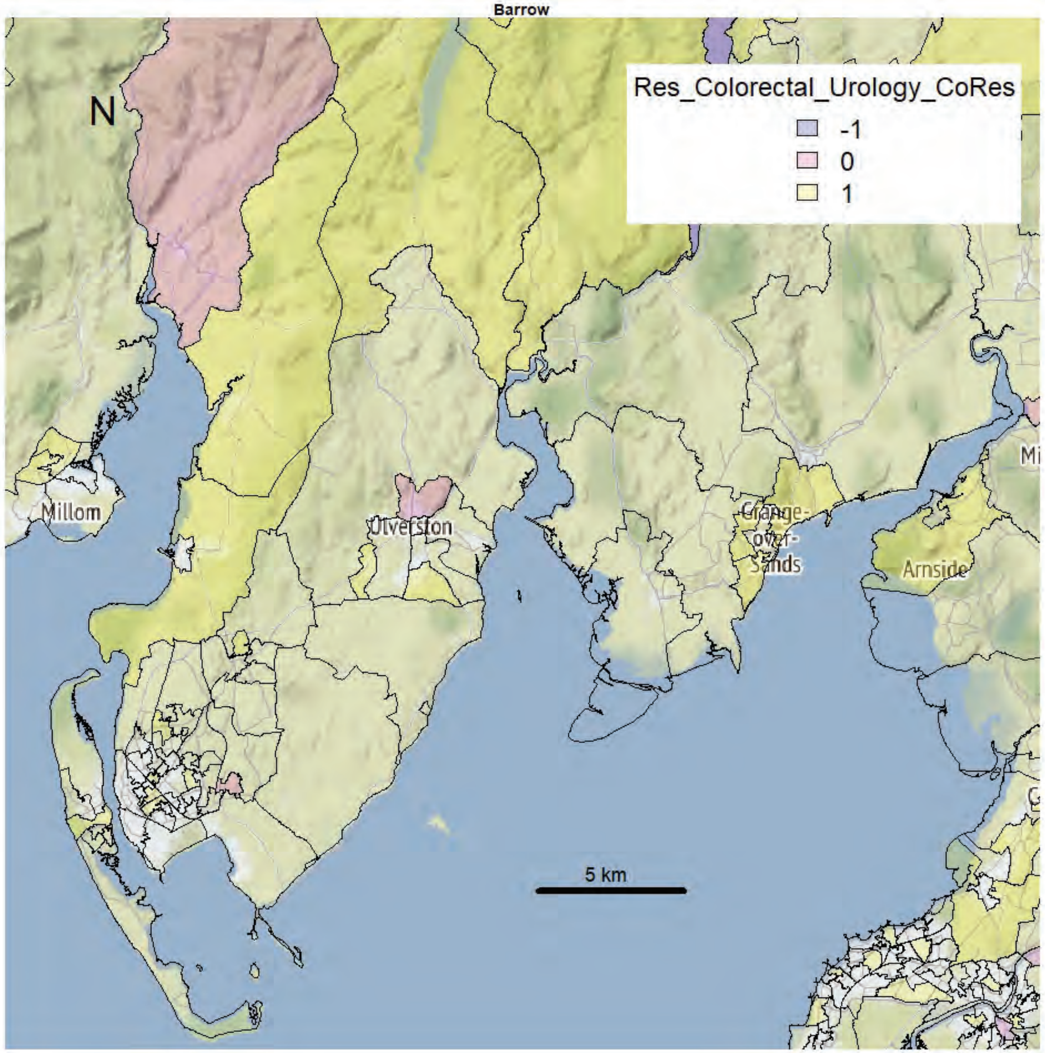
Barrow
N
Res_Colorectal_Urology_CoRes
-1
0
1
Millom
Ulverston
Grange-over-Sands
Arnside
5 km

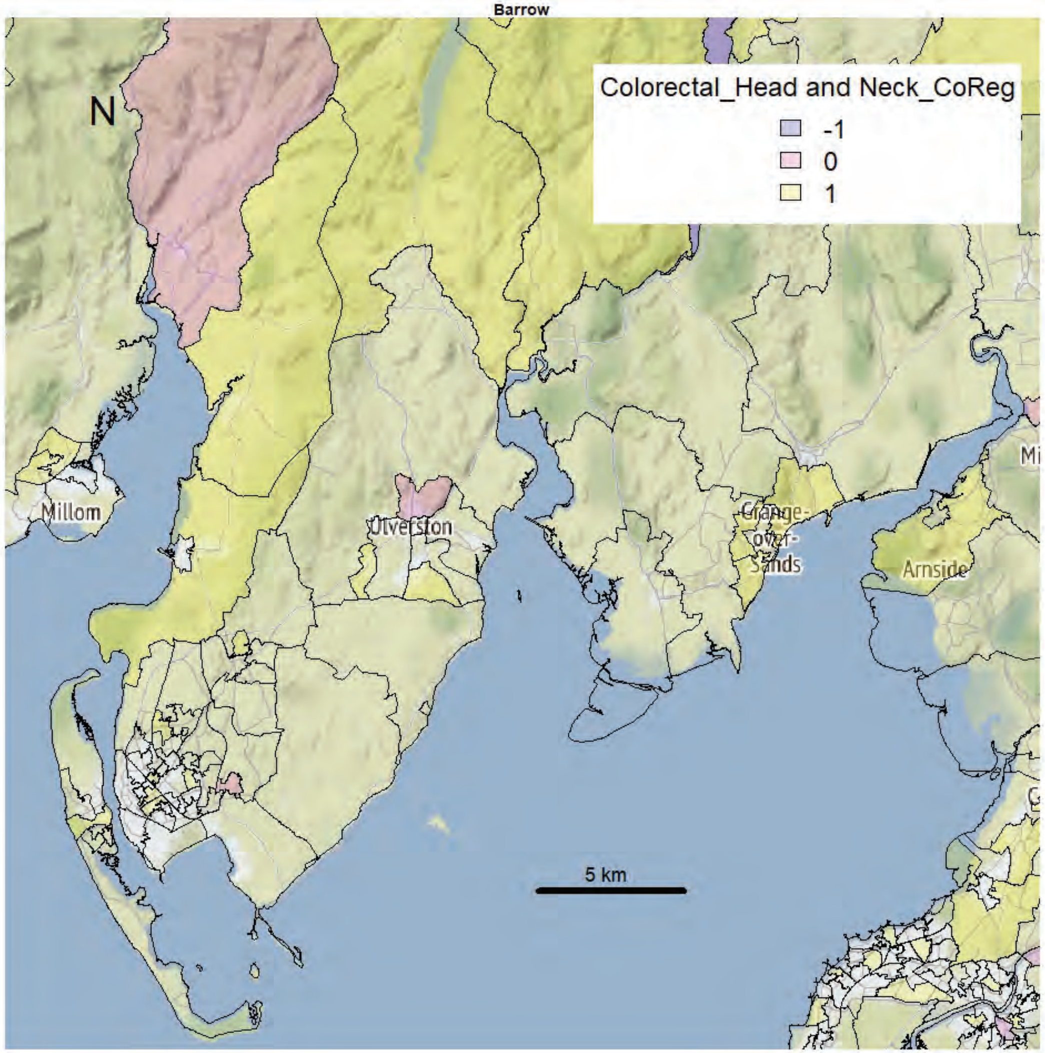
Barrow
N
Colorectal_Head and Neck_CoReg
-1
0
1
Millom
Ulverston
Grange-over-Sands
Arnside
5 km

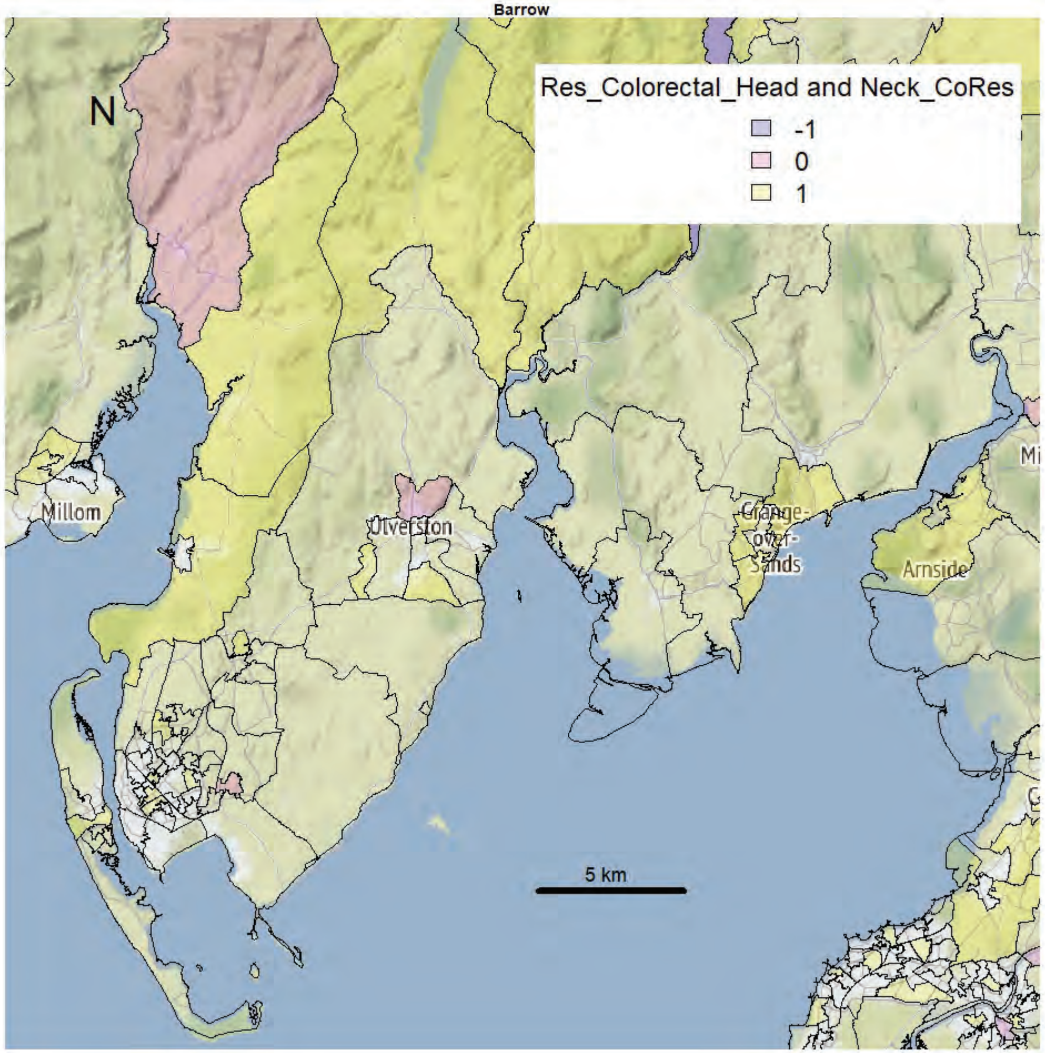
Barrow
N
Res_Colorectal_Head and Neck_CoRes
-1
0
1
Millom
Ulverston
Grange-over-Sands
Arnside
5 km

Barrow

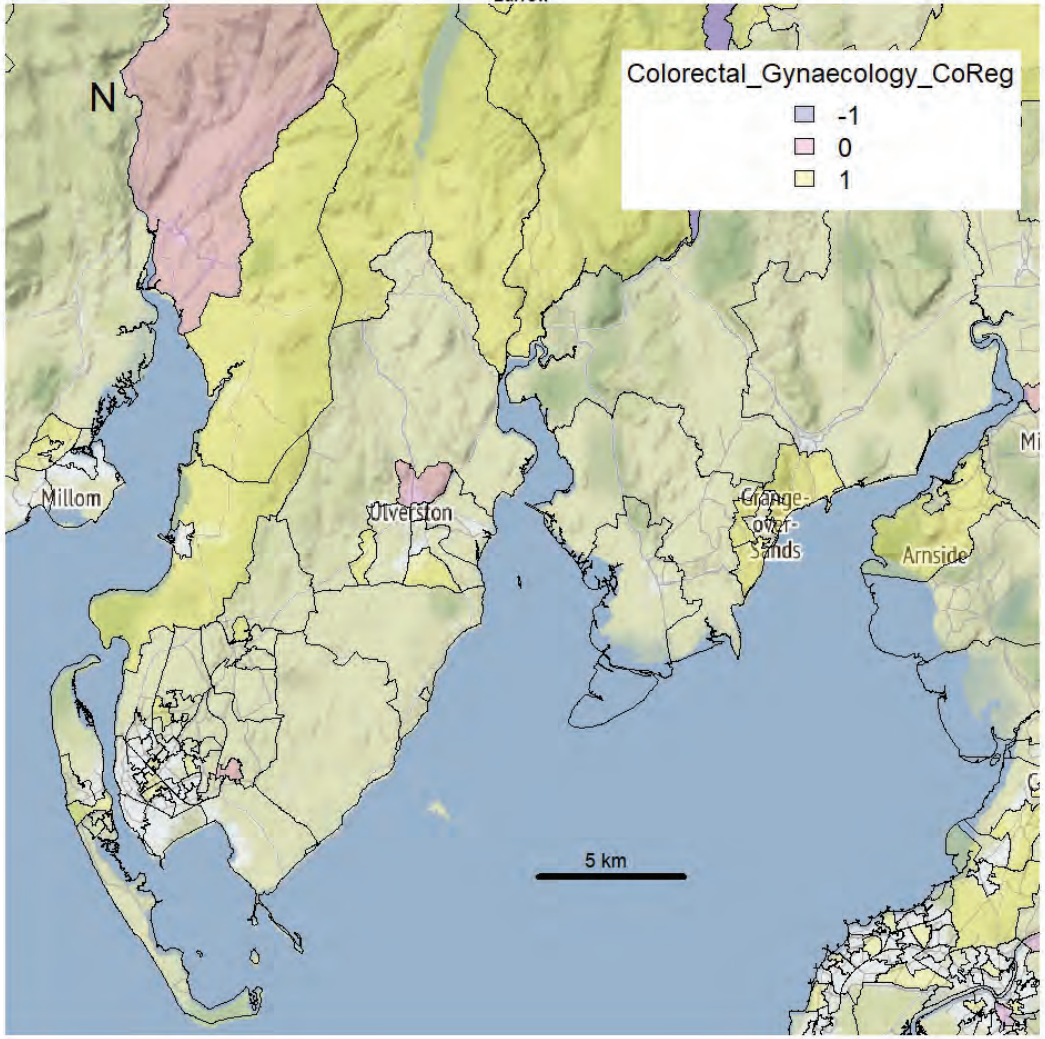

Barrow

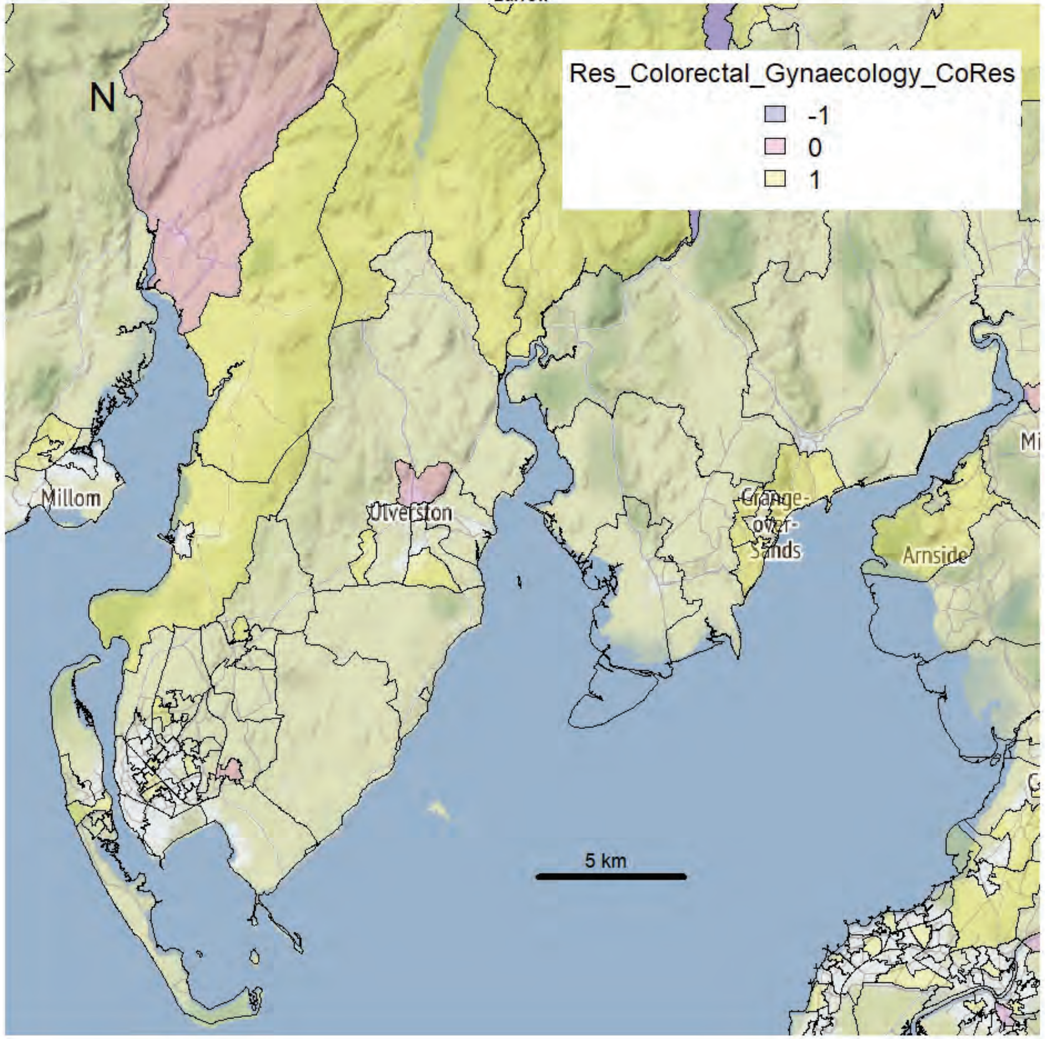

Barrow

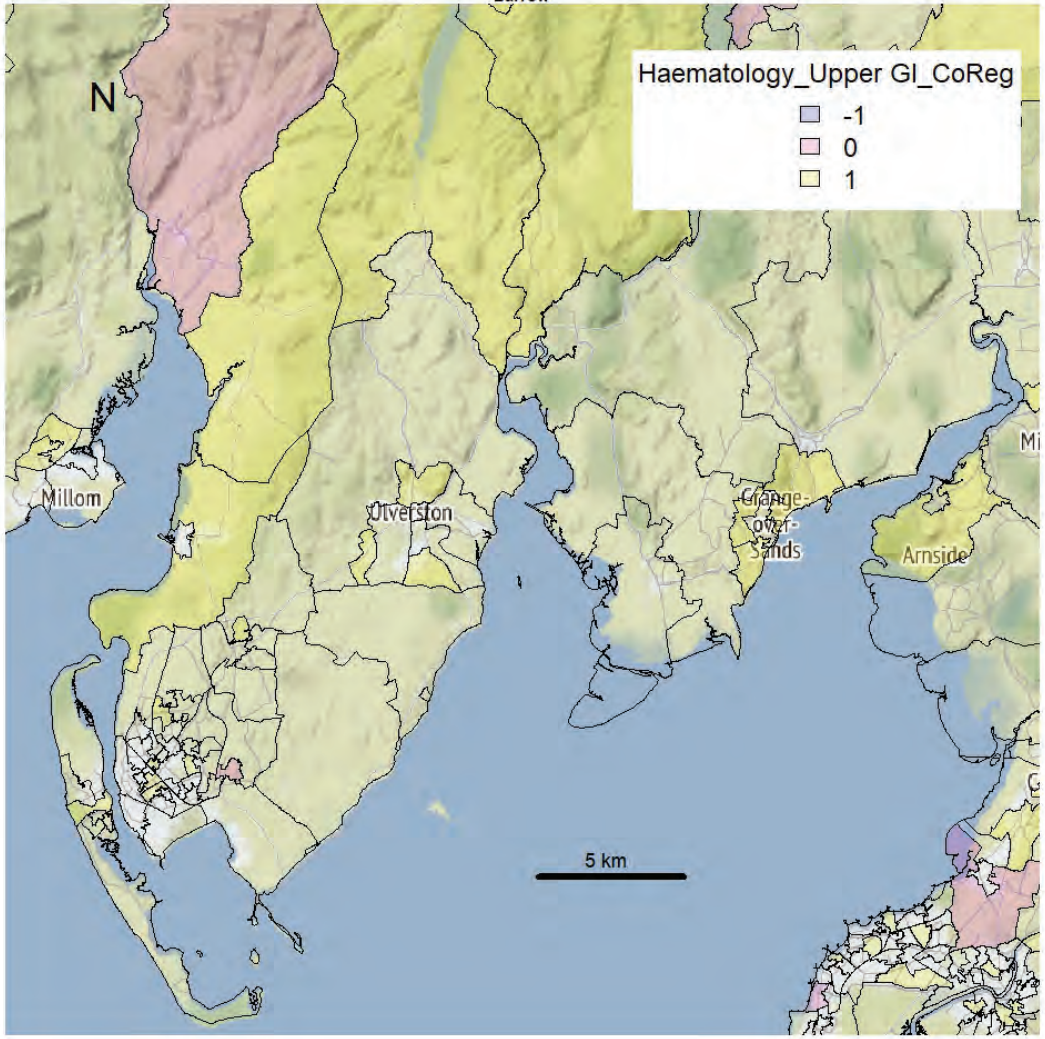

Barrow

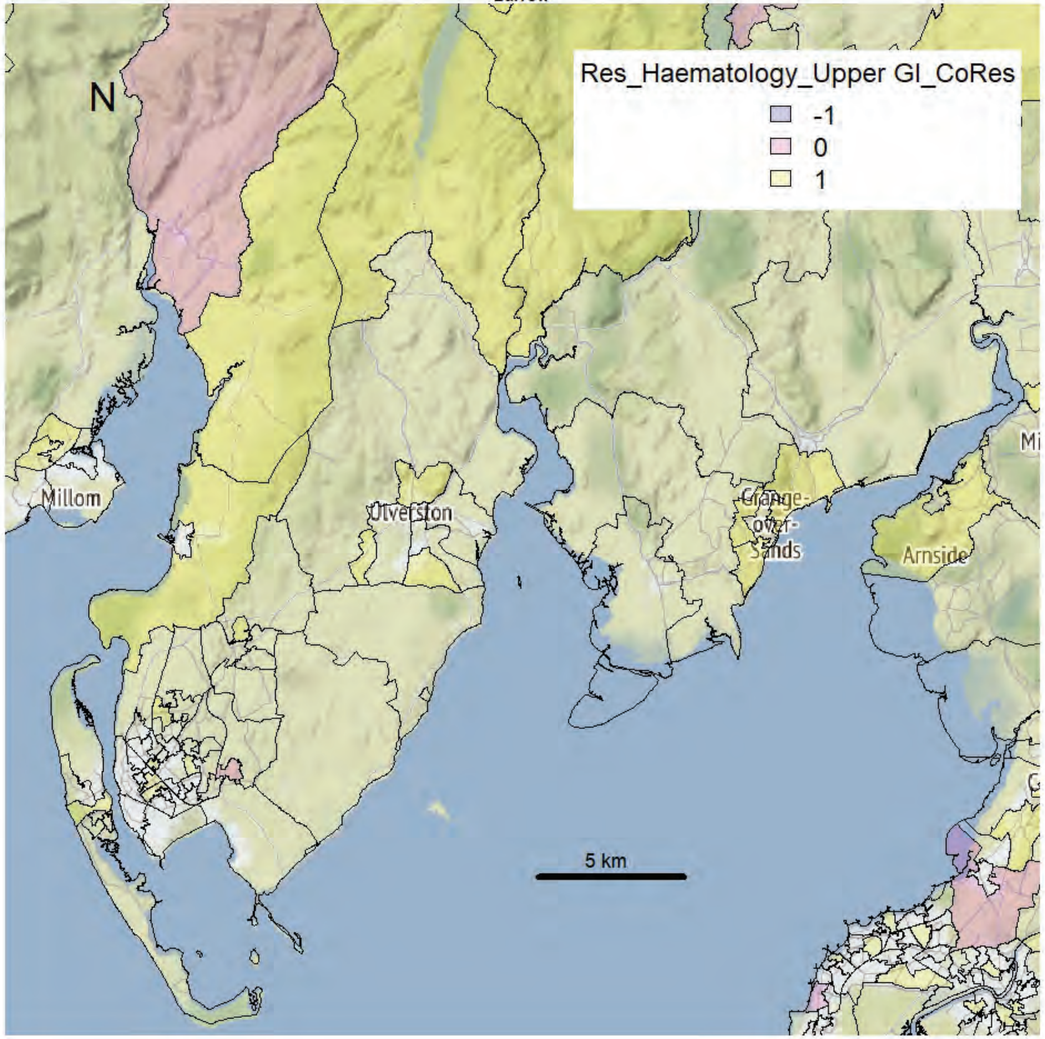

Barrow

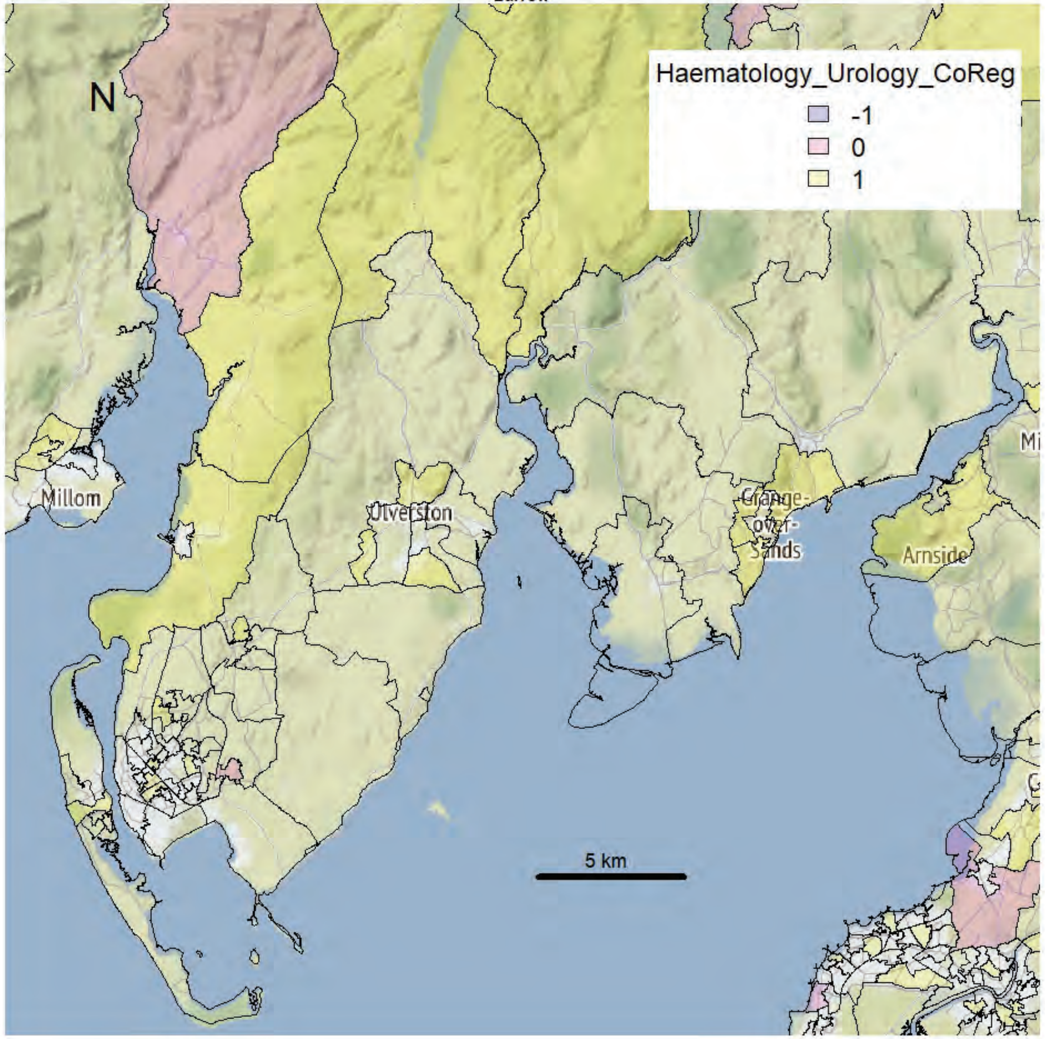

Barrow

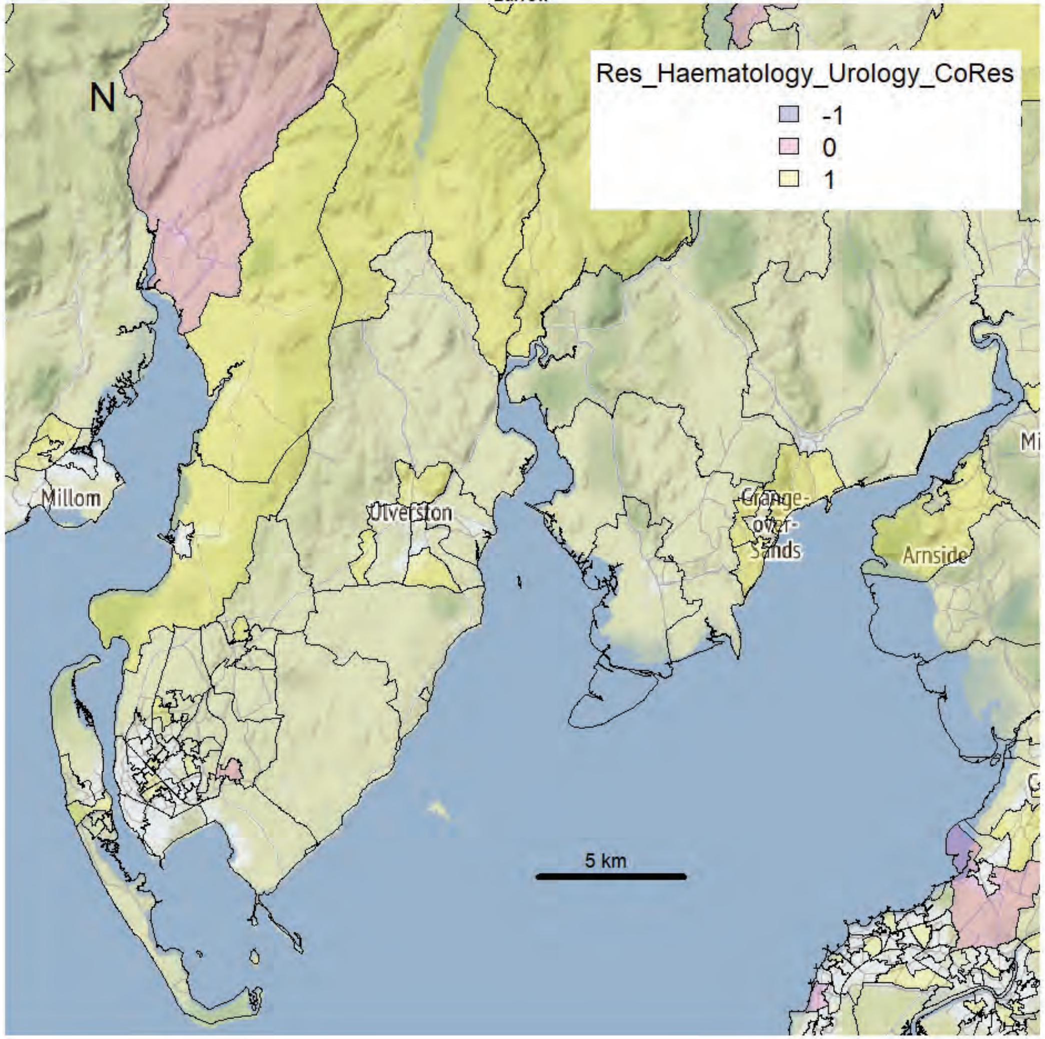

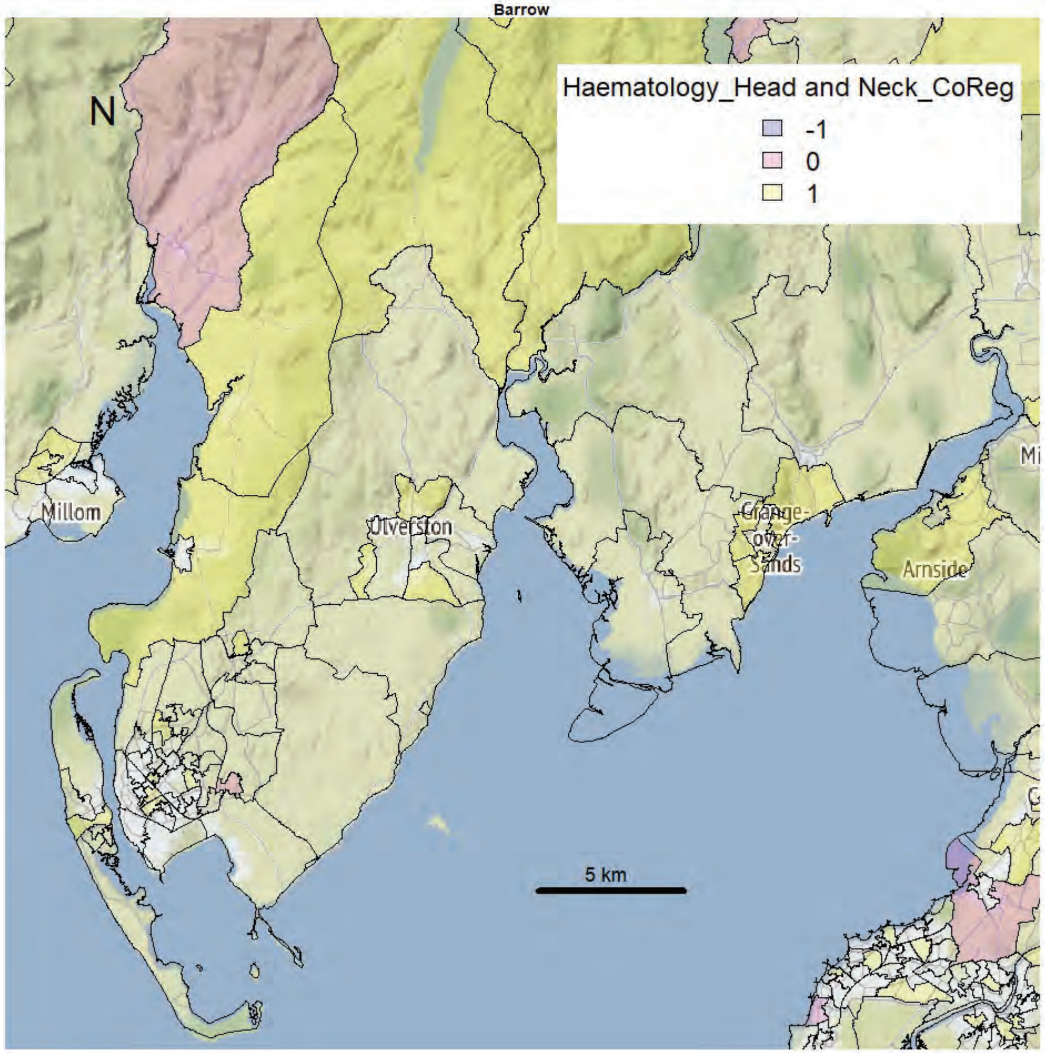
Barrow
Haematology_Head and Neck_CoReg
-1
0
1
N
Millom
Ulverston
Grange-over-Sands
Arnside
5 km

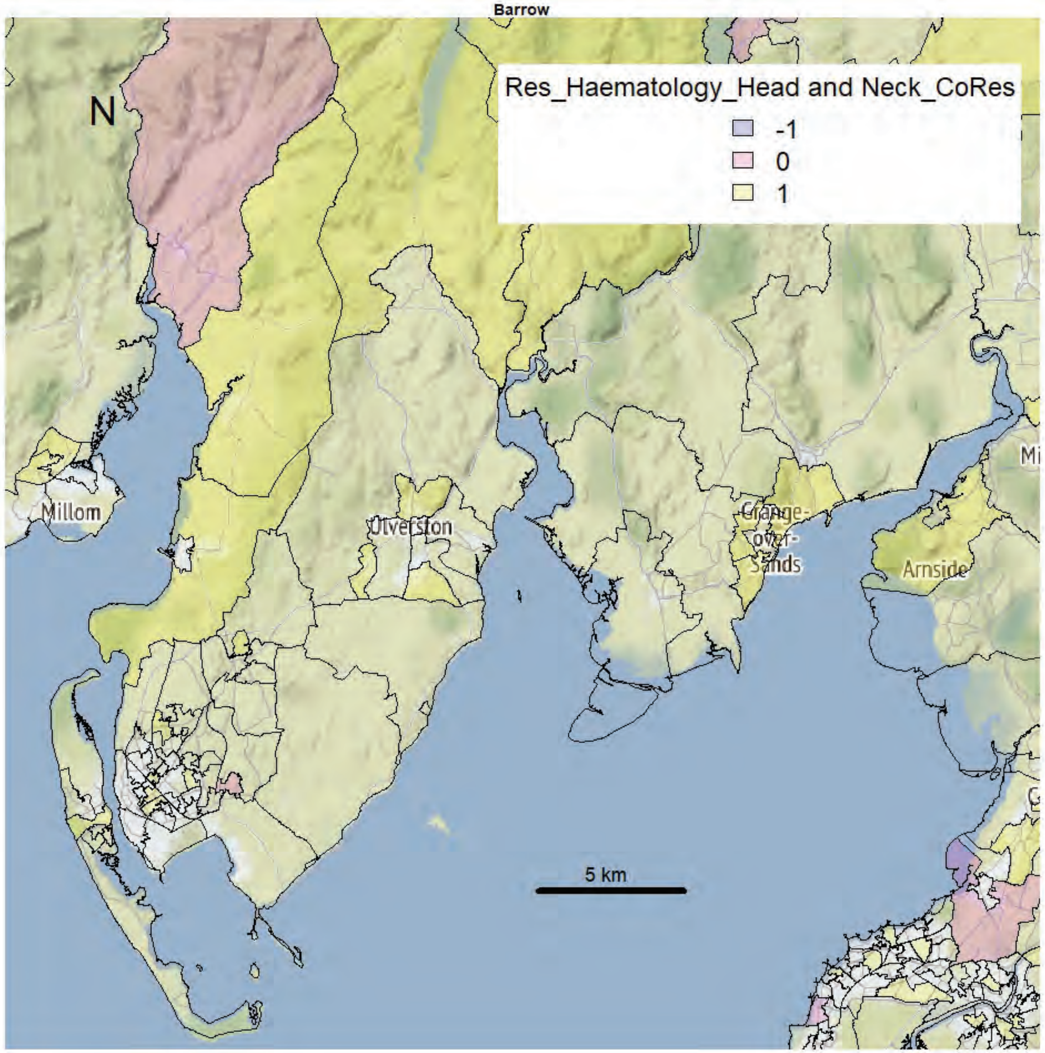
Barrow
Res_Haematology_Head and Neck_CoRes
-1
0
1
N
Millom
Ulverston
Grange-over-Sands
Arnside
5 km

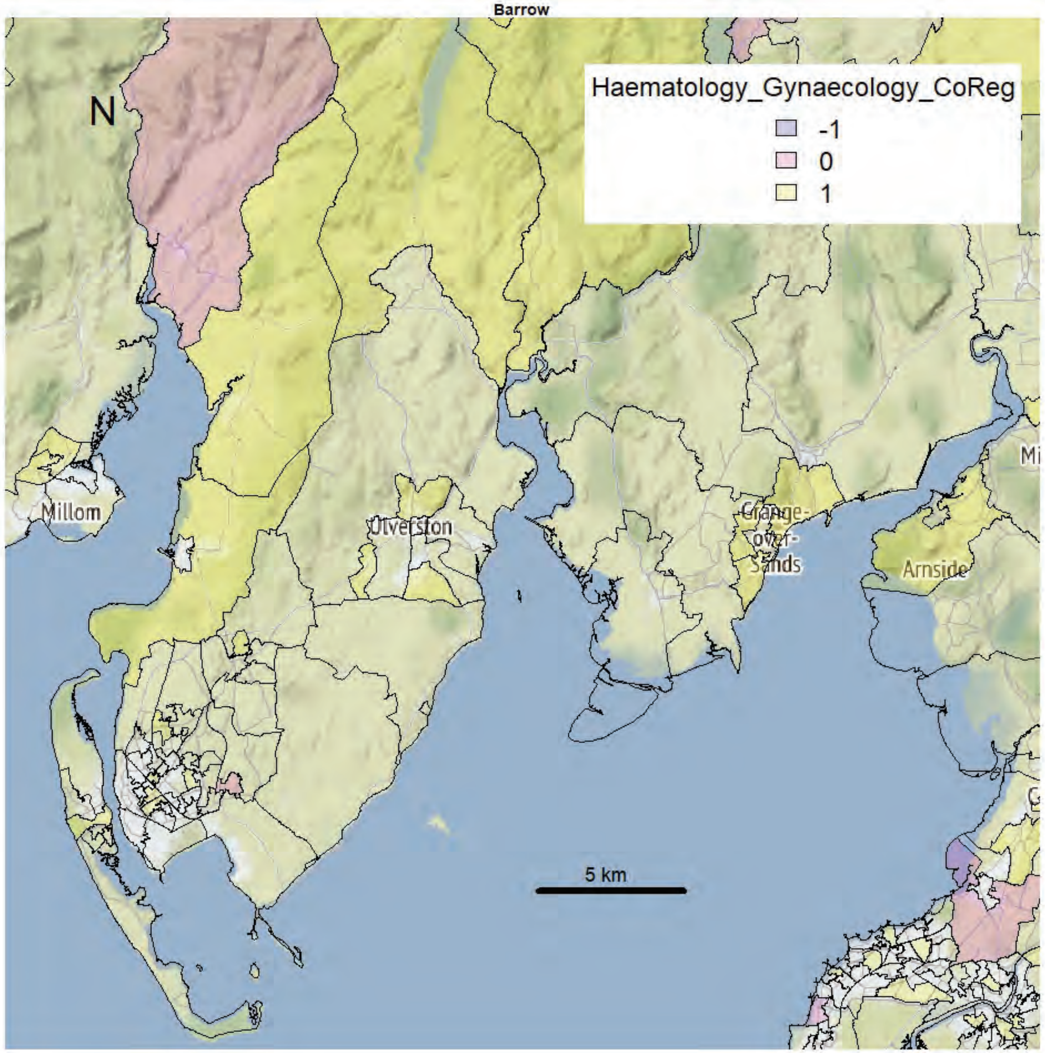
Barrow
N
Haematology_Gynaecology_CoReg
-1
0
1
Millom
Ulverston
Grange-over-Sands
Arnside
5 km

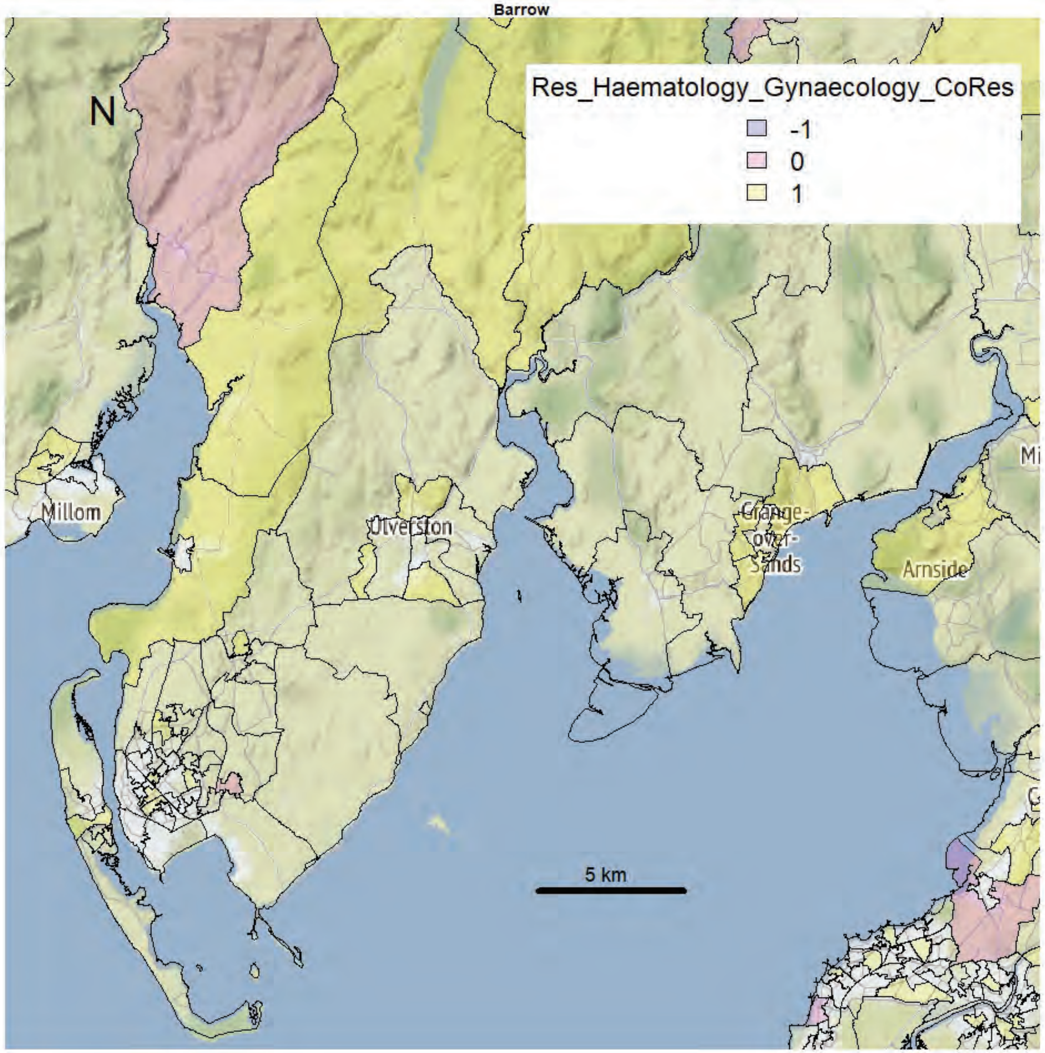
Barrow
N
Res_Haematology_Gynaecology_CoRes
-1
0
1
Millom
Ulverston
Grange-over-Sands
Arnside
5 km

Barrow

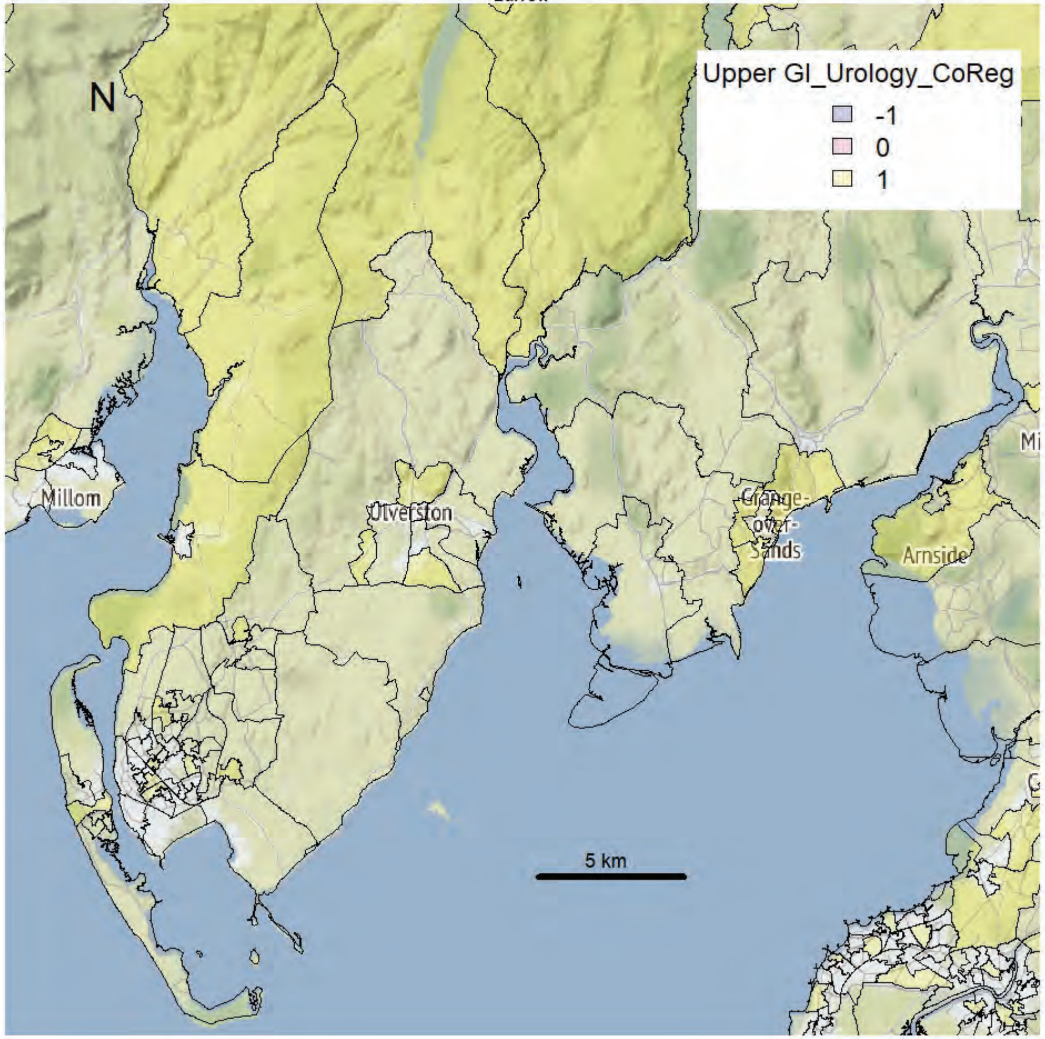

Barrow

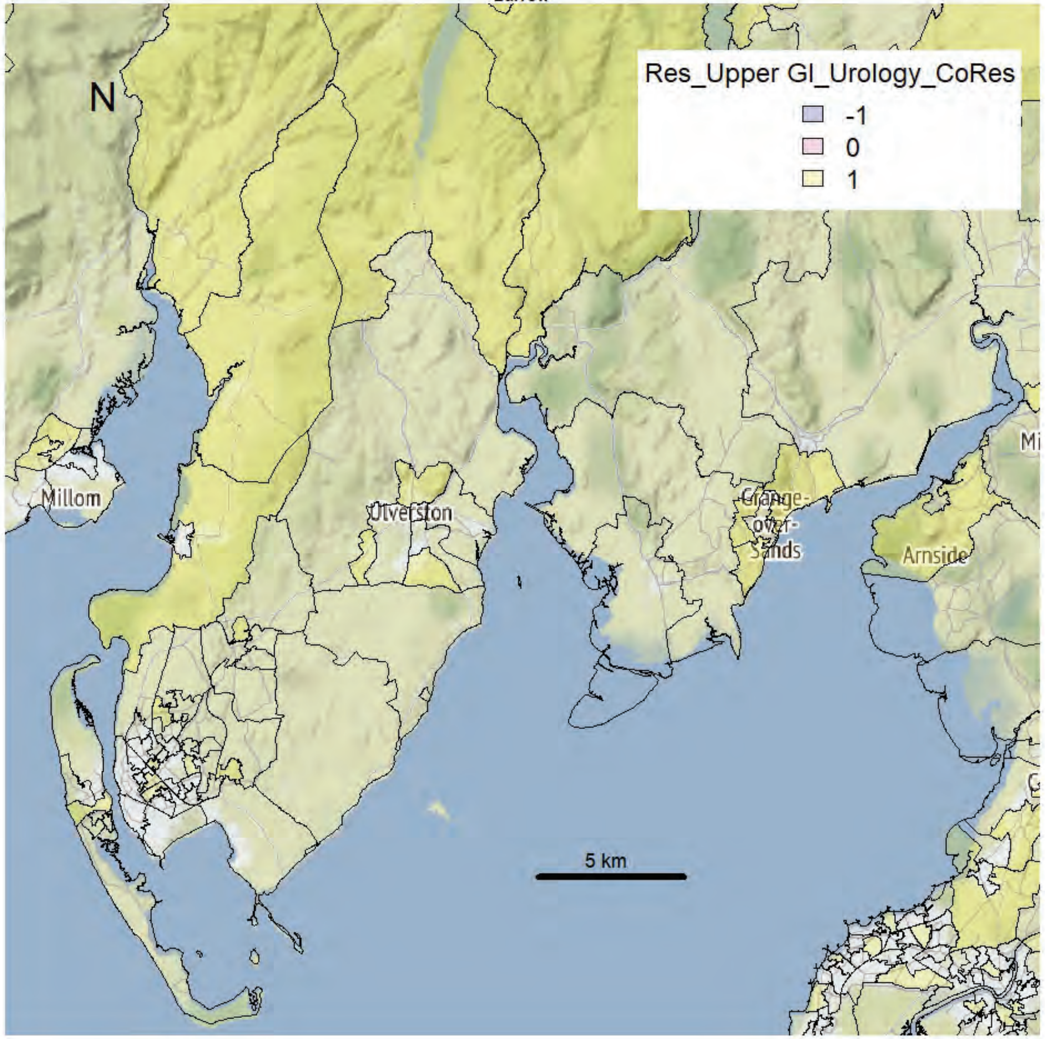

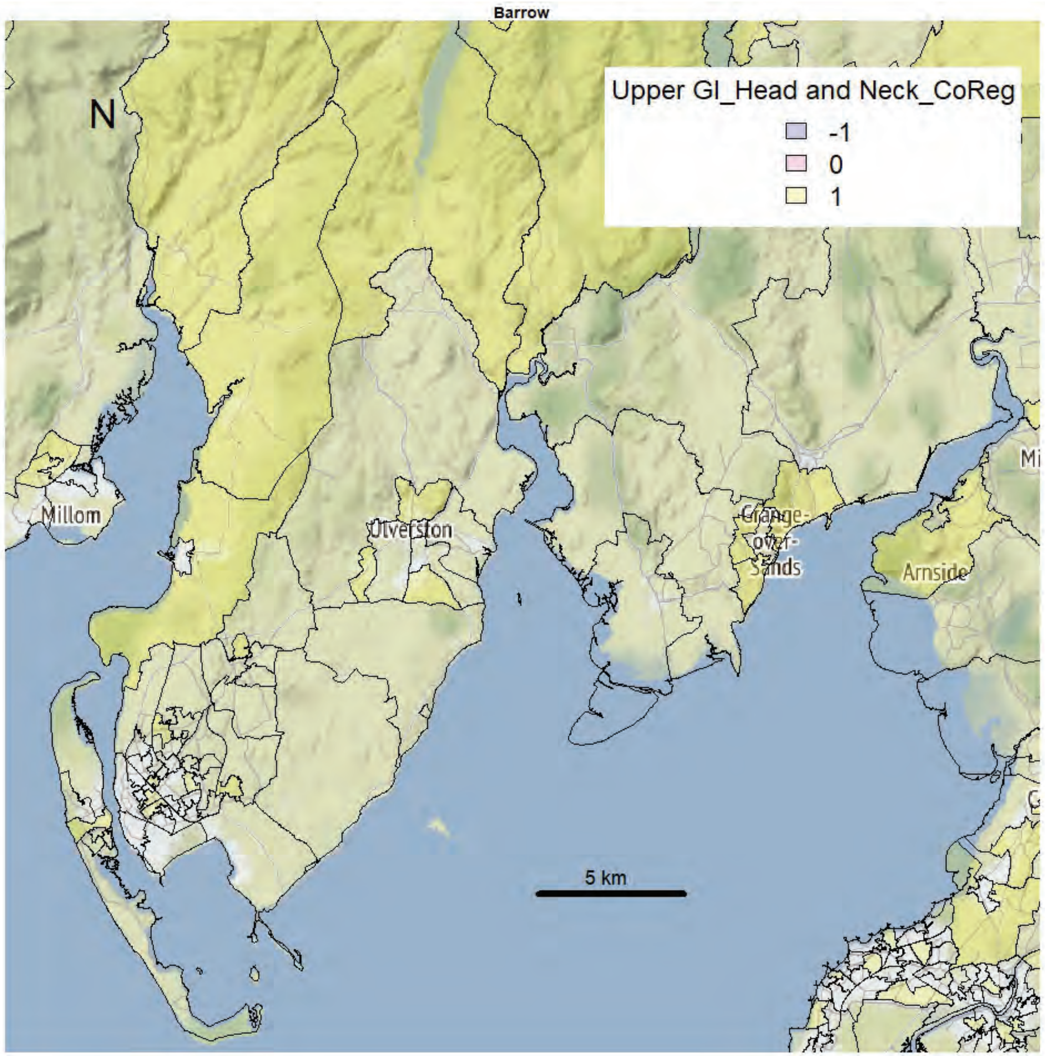
Barrow
N
Upper GI_Head and Neck_CoReg
-1
0
1
Millom
Ulverston
Grange-over-Sands
Arnside
5 km

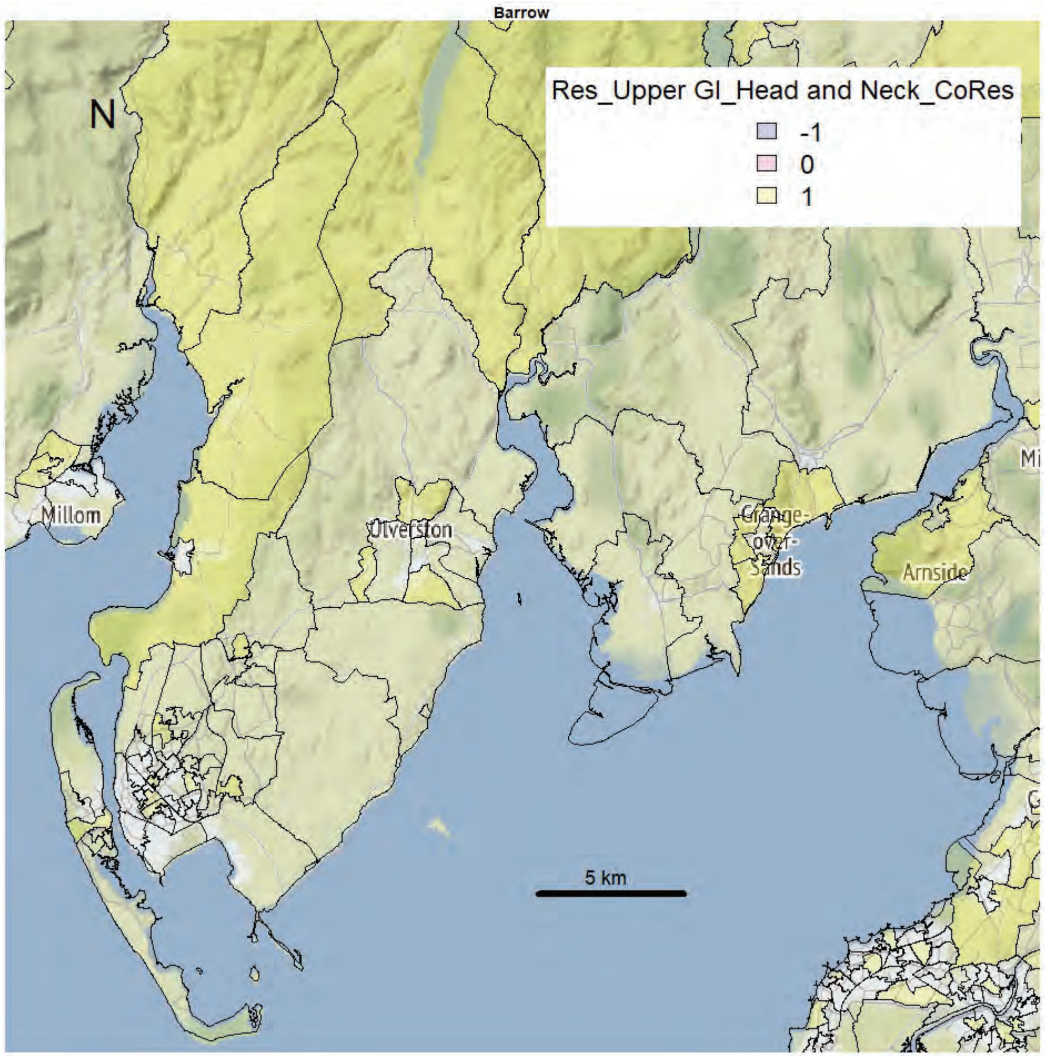
Barrow
N
Res_Upper GI_Head and Neck_CoRes
-1
0
1
Millom
Ulverston
Grange-over-Sands
Arnside
5 km

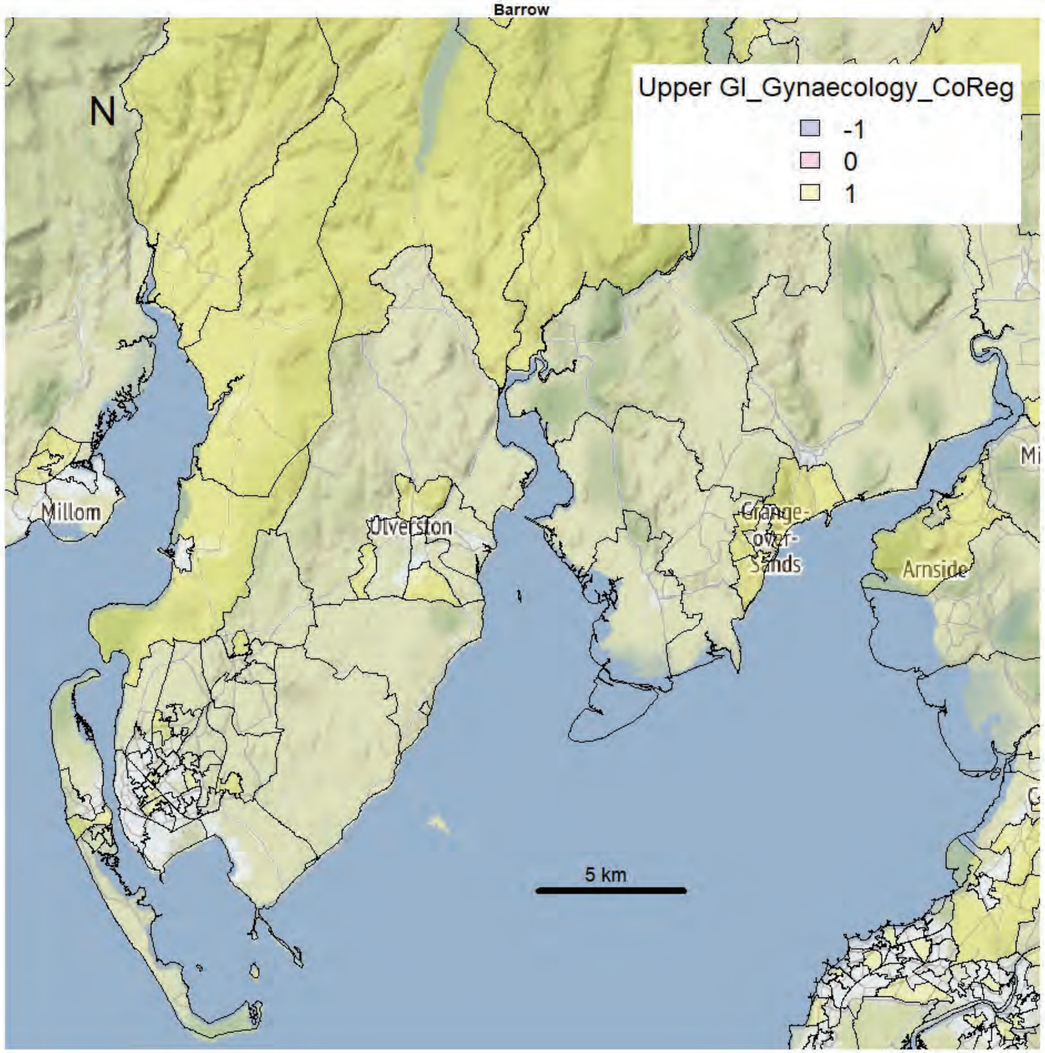
Barrow
N
Upper GI_Gynaecology_CoReg
-1
0
1
Millom
Ulverston
Grange-over-Sands
Arnside
5 km

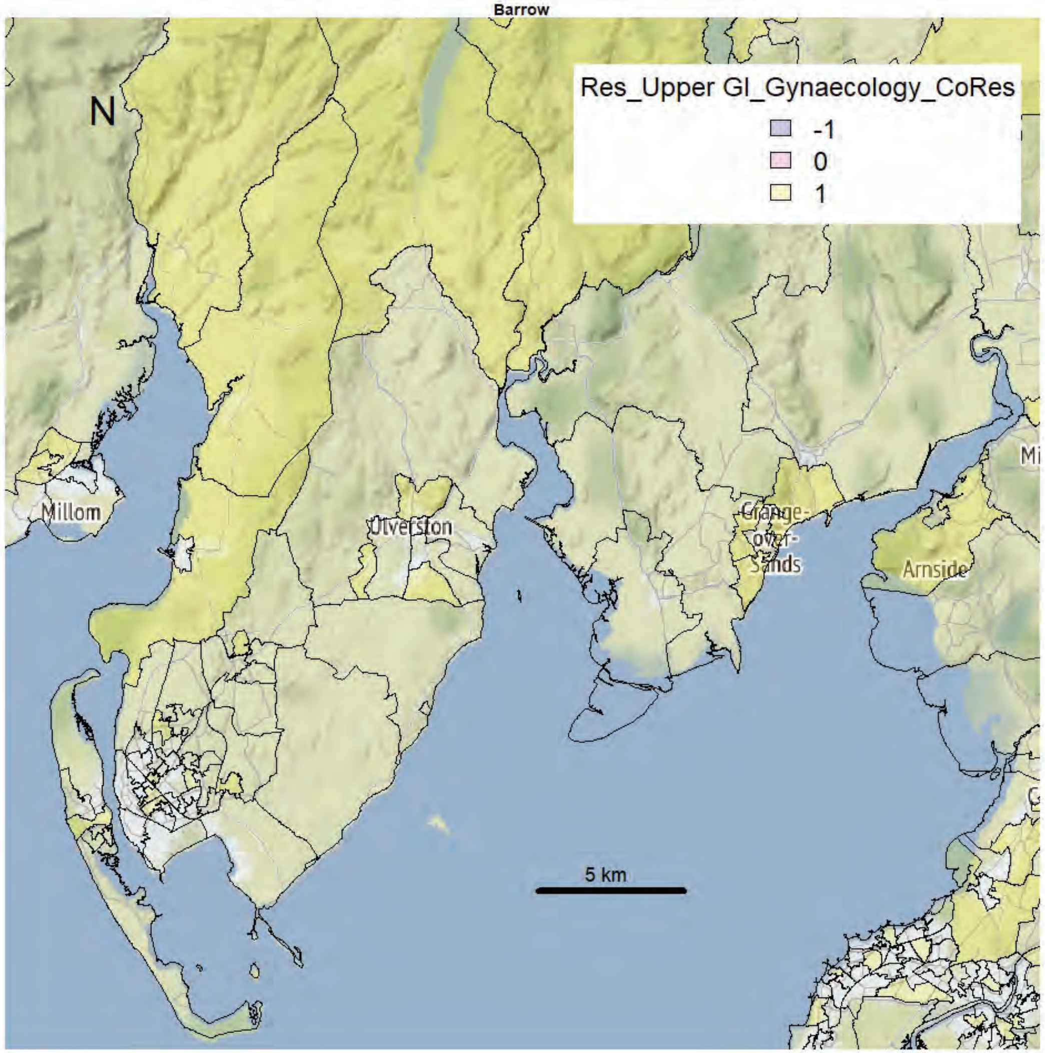
Barrow
N
Res_Upper GI_Gynaecology_CoRes
-1
0
1
Millom
Ulverston
Grange-over-Sands
Arnside
5 km

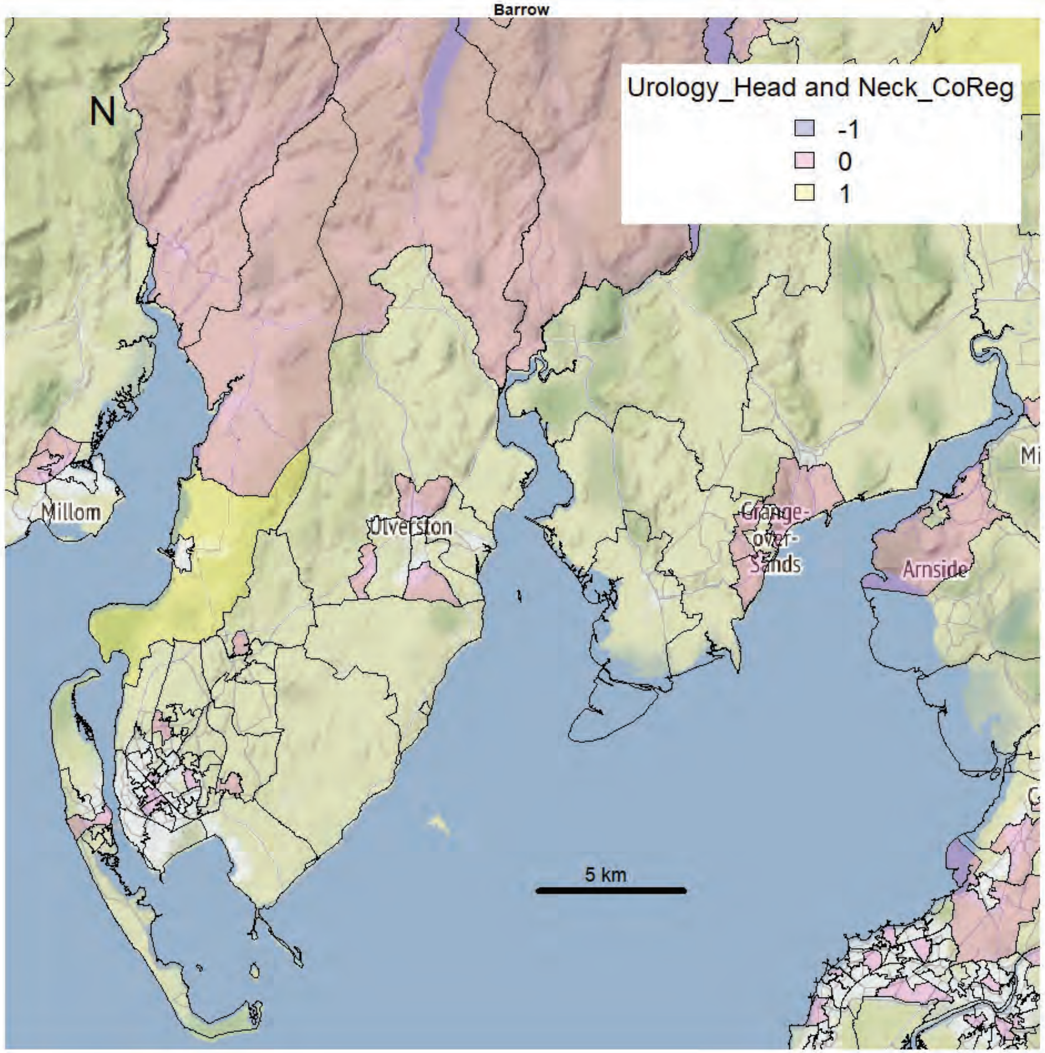
Barrow
N
Urology_Head and Neck_CoReg
-1
0
1
Millom
Ulverston
Grange-over-Sands
Arnside
5 km

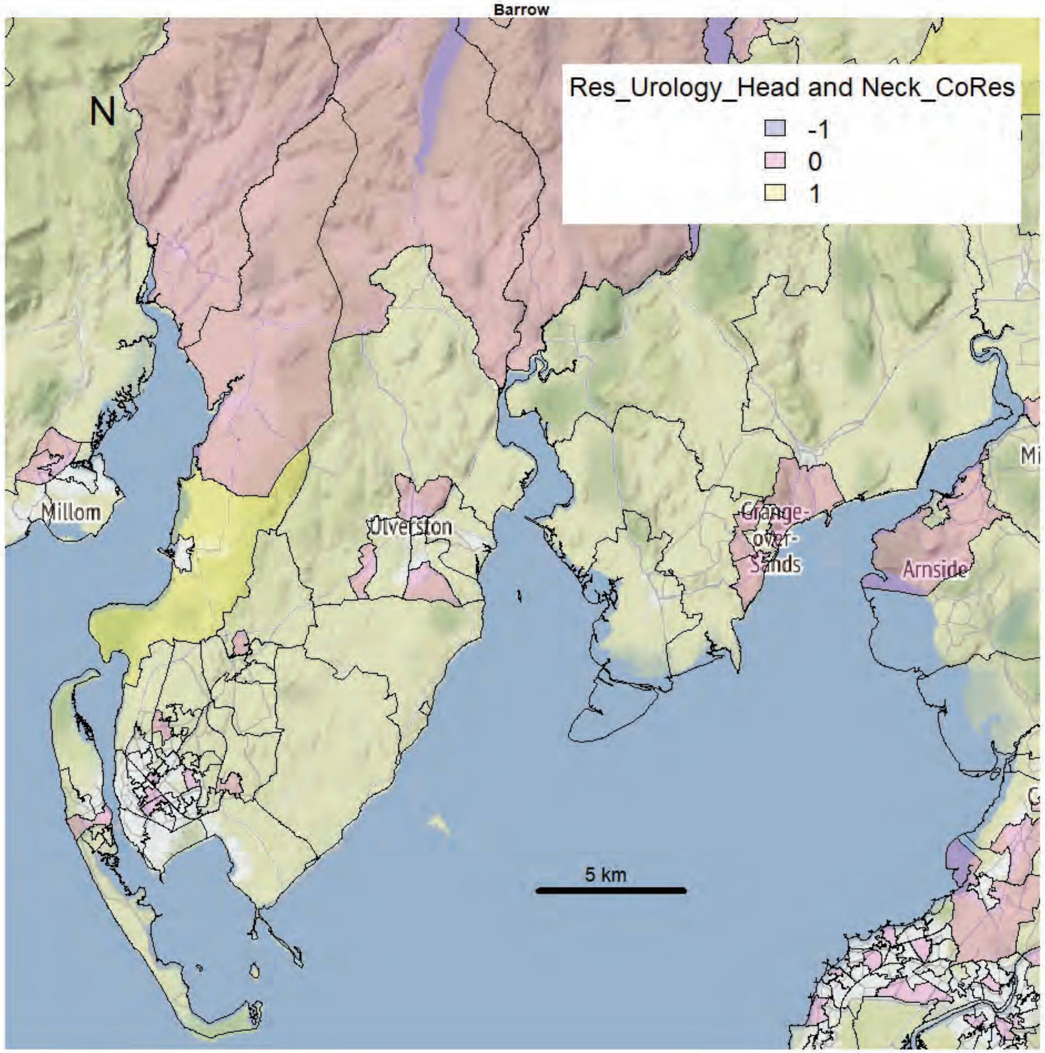
Barrow
N
Res_Urology_Head and Neck_CoRes
-1
0
1
Millom
Ulverston
Grange-over-Sands
Arnside
5 km

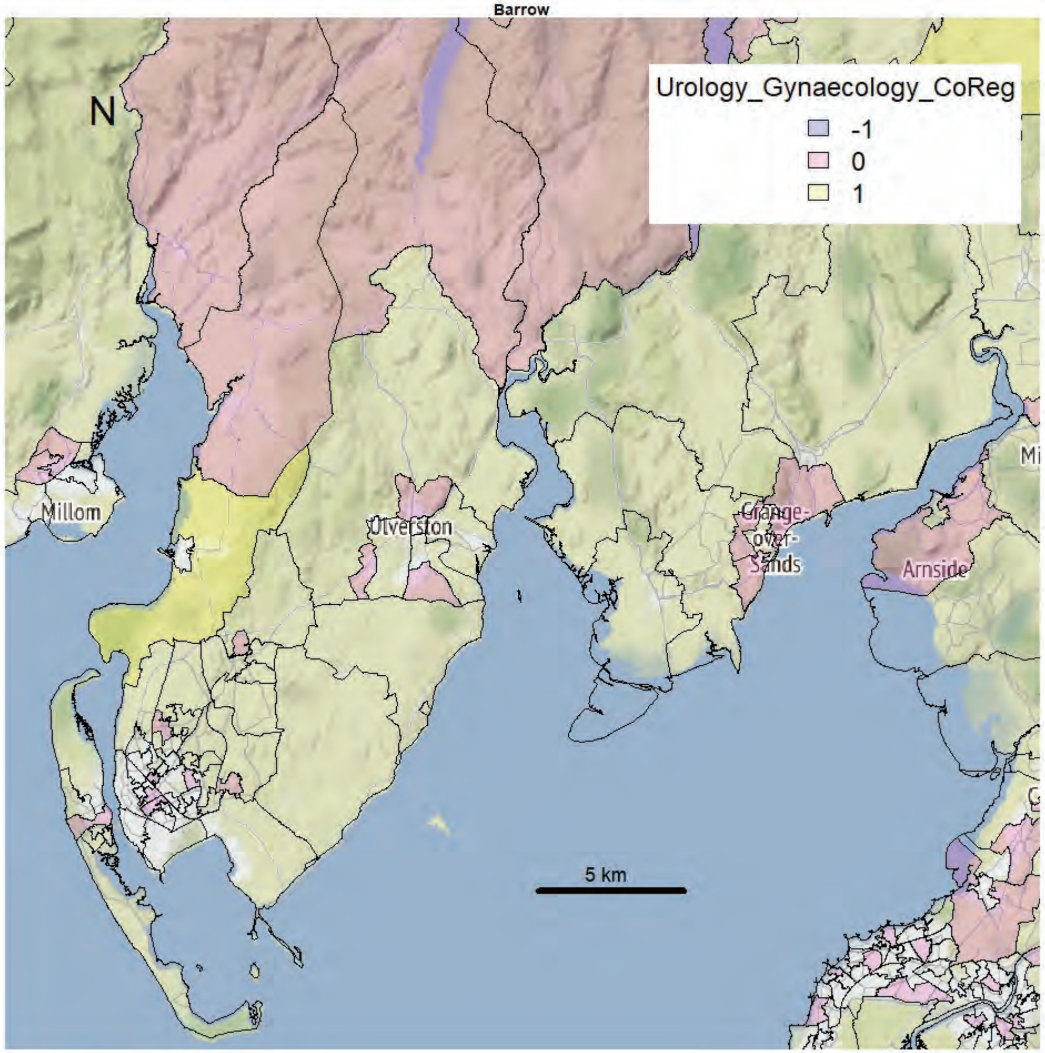
Barrow
N
Urology_Gynaecology_CoReg
-1
0
1
Millom
Ulverston
Grange-over-Sands
Arnside
5 km

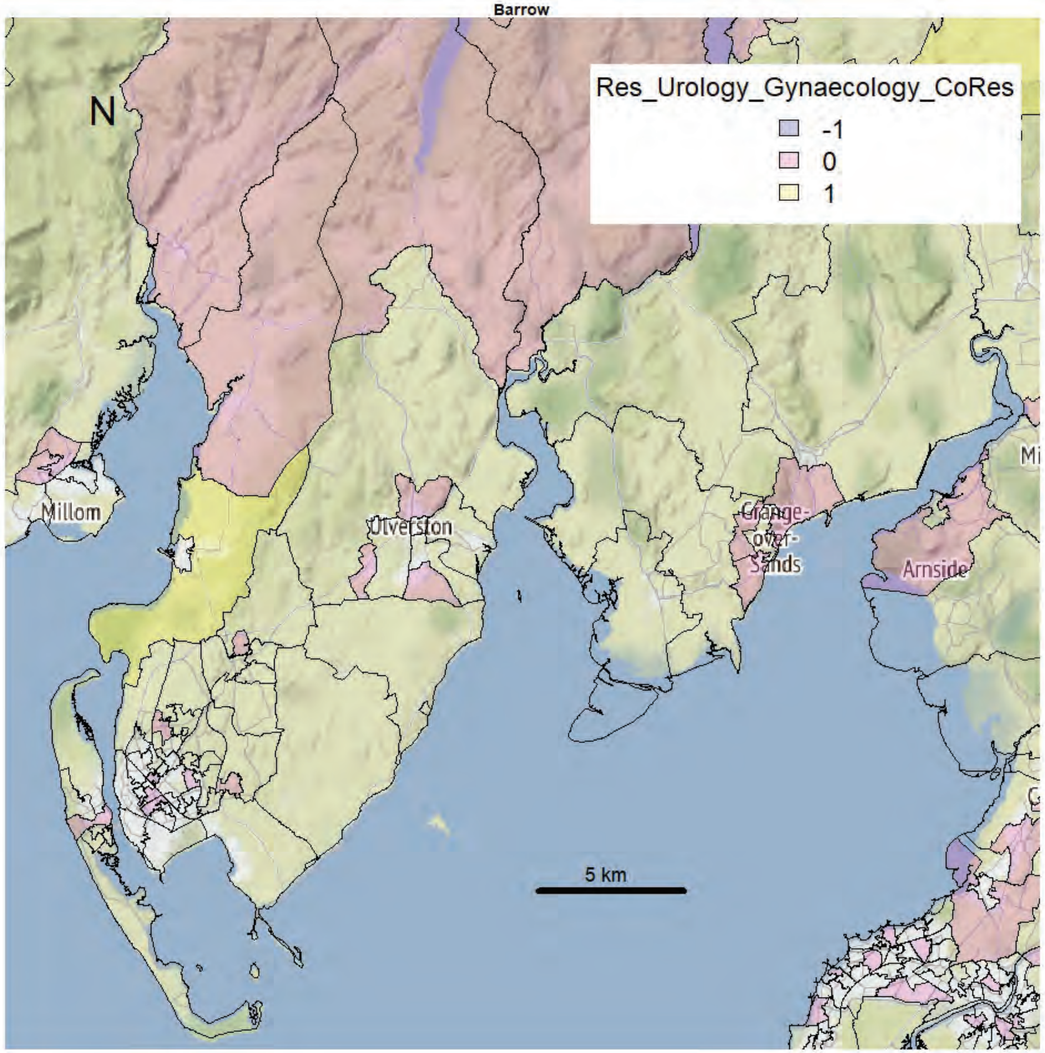
Barrow
N
Res_Urology_Gynaecology_CoRes
-1
0
1
Millom
Ulverston
Grange-over-Sands
Arnside
5 km

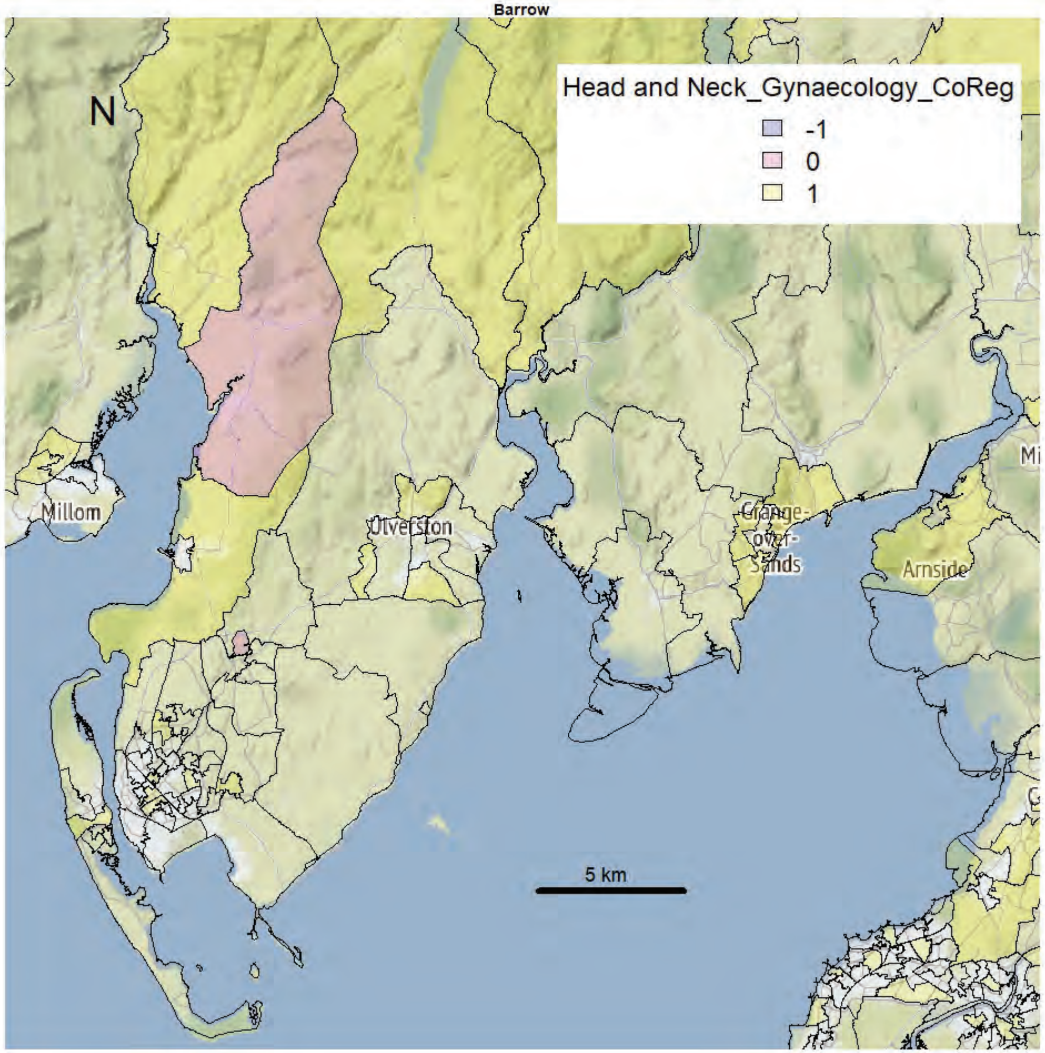
Barrow
Head and Neck_Gynaecology_CoReg
-1
0
1
N
Millom
Ulverston
Grange-over-Sands
Arnside
5 km

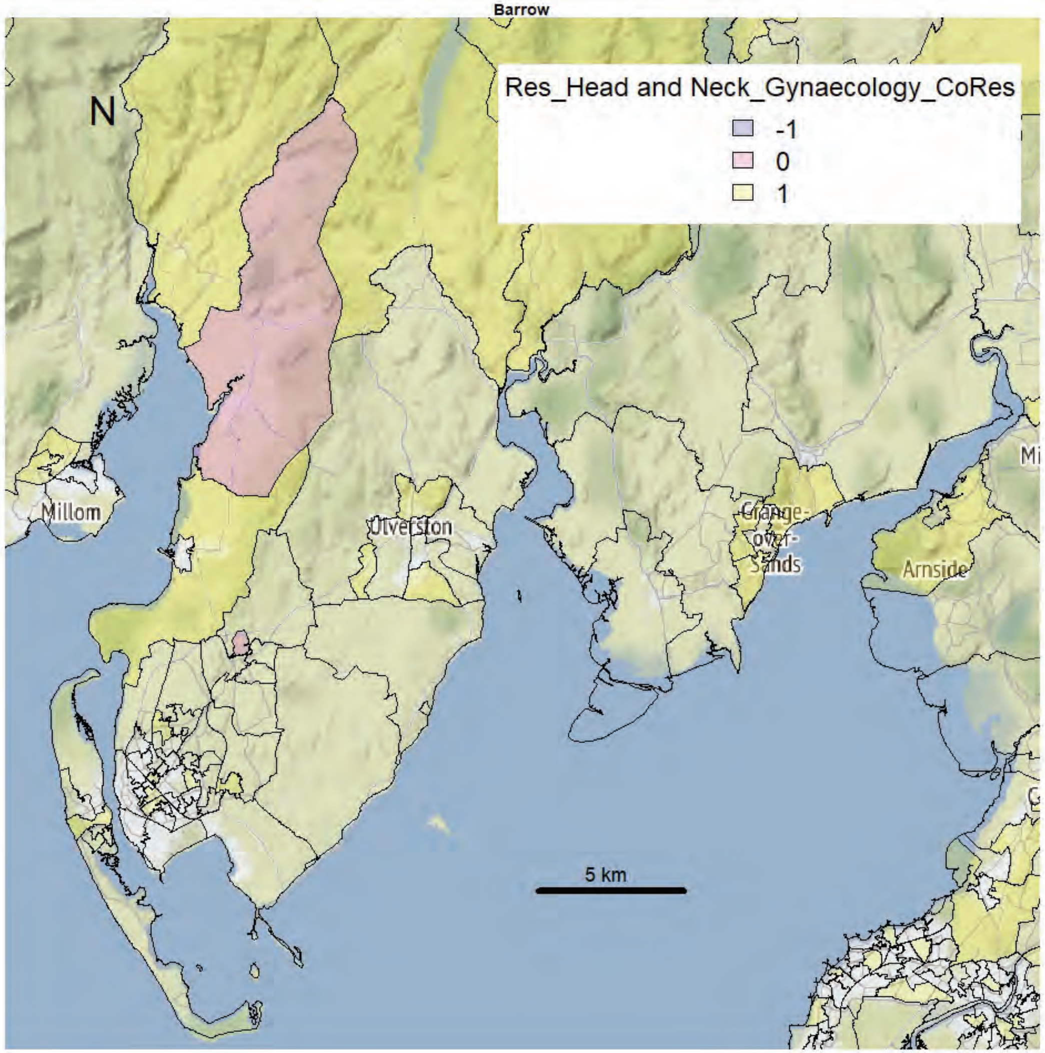
Barrow
Res_Head and Neck_Gynaecology_CoRes
-1
0
1
N
Millom
Ulverston
Grange-over-Sands
Arnside
5 km

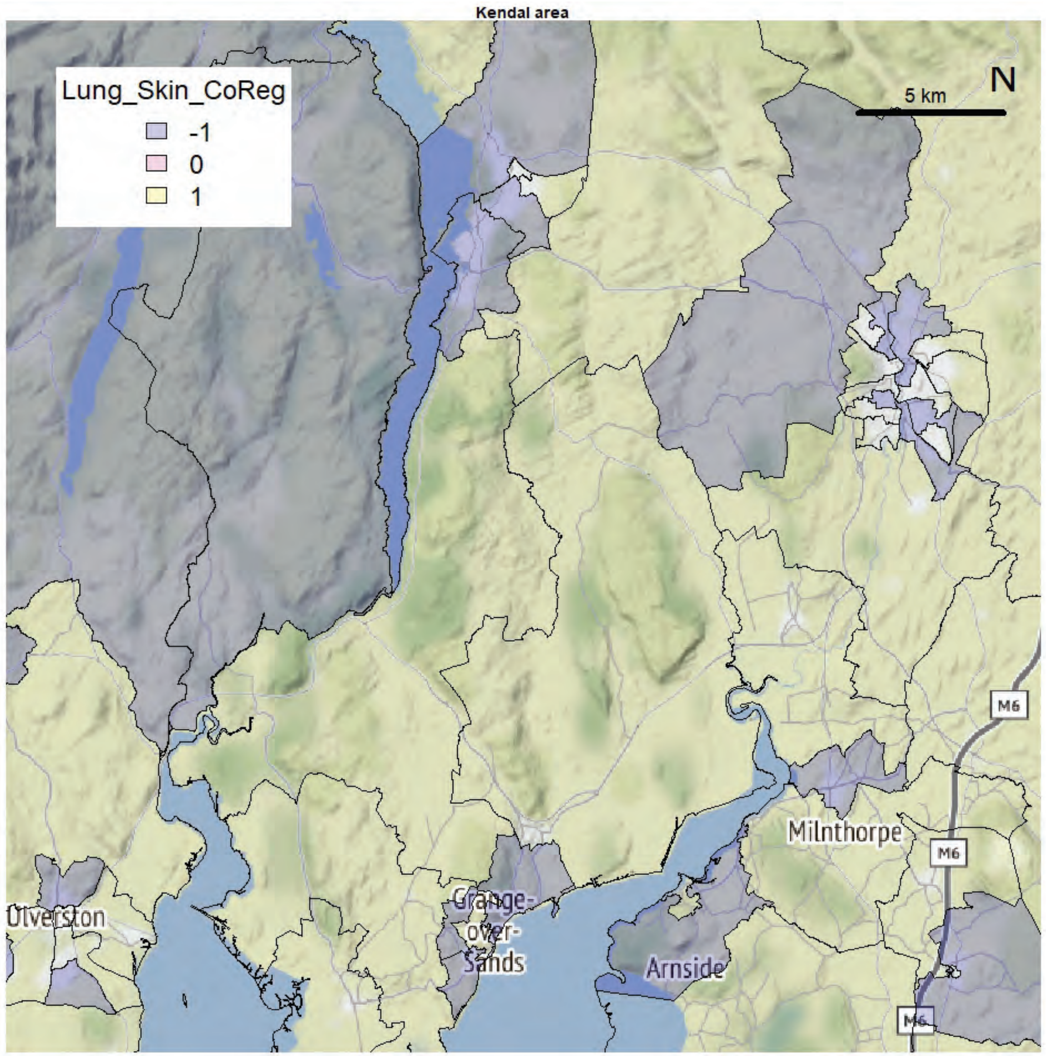
Kendal area
Lung_Skin_CoReg
-1
0
1
5 km
N
M6
Milnthorpe
M6
Ulverston
Grange-
over-
Sands
Arnside
M6

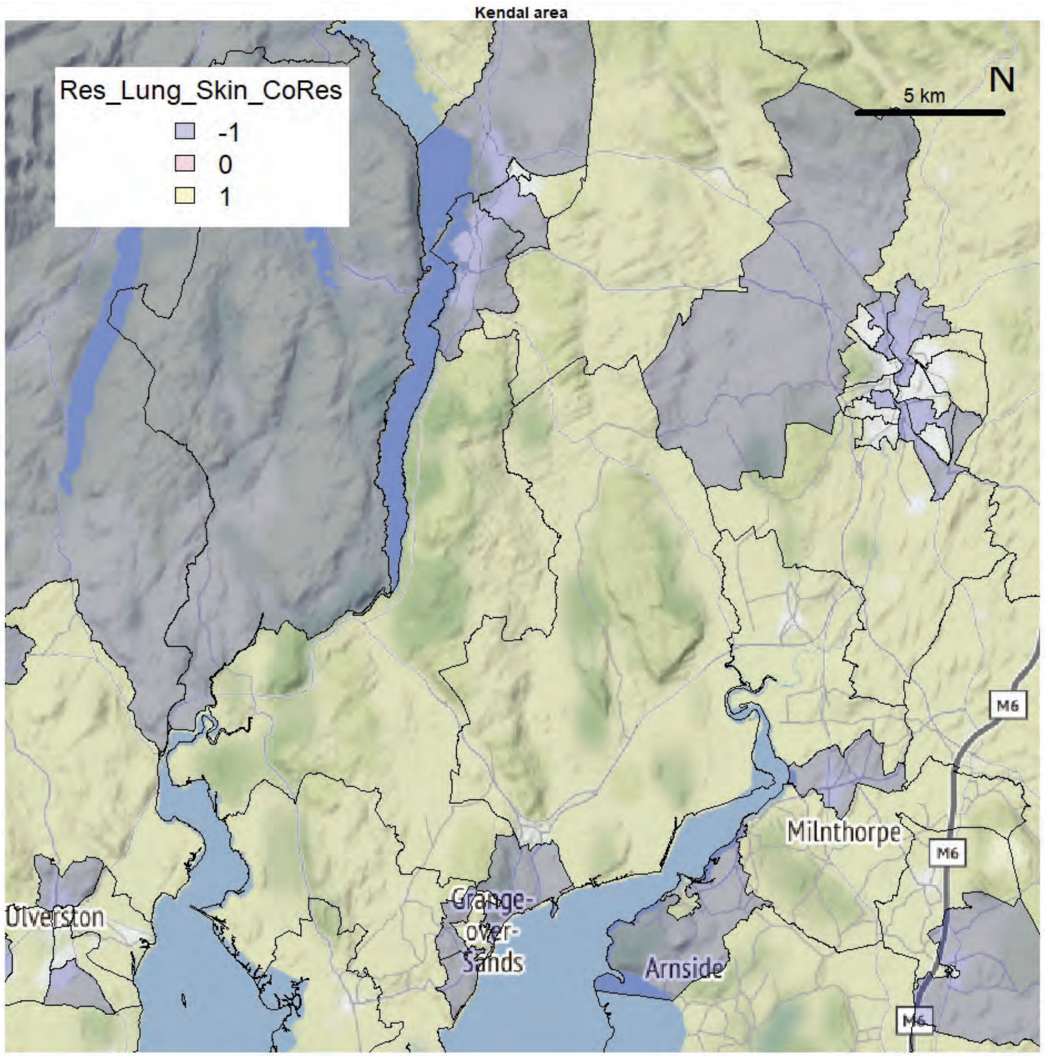
Kendal area
Res_Lung_Skin_CoRes
-1
0
1
5 km
N
M6
Milnthorpe
M6
Ulverston
Grange-
over-
Sands
Arnside
M6

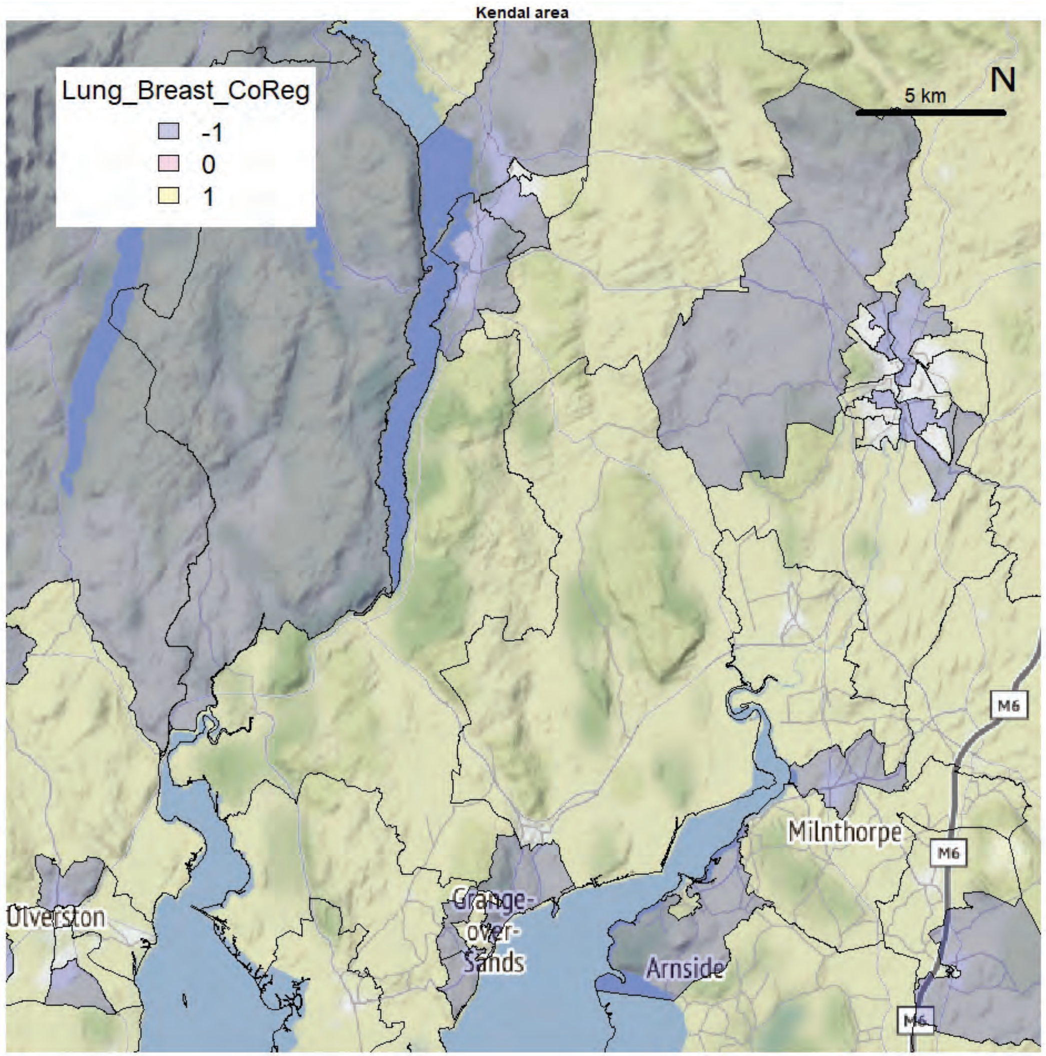
Kendal area
Lung_Breast_CoReg
-1
0
1
5 km
N
M6
Milnthorpe
M6
Ulverston
Grange-over-Sands
Arnside
M6

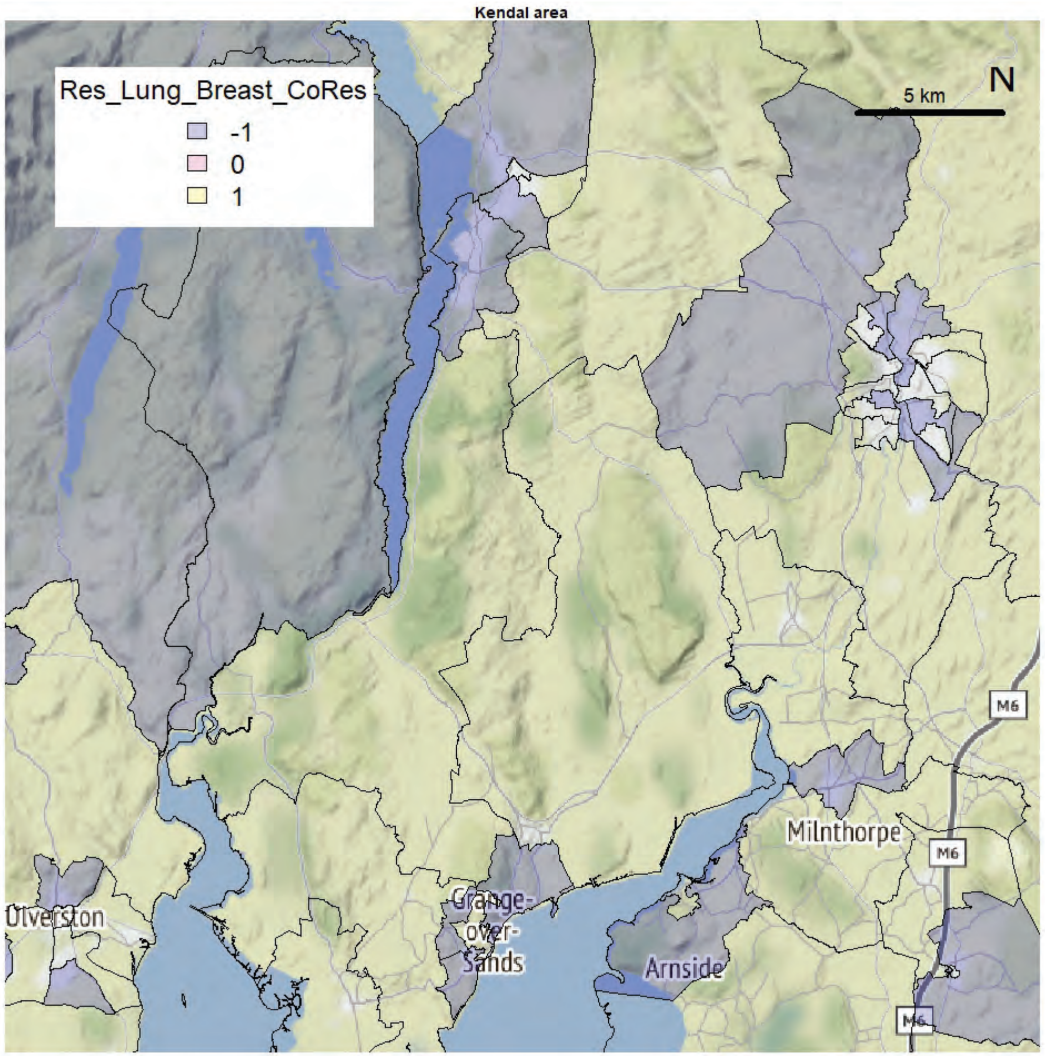
Kendal area
Res_Lung_Breast_CoRes
-1
0
1
5 km
N
M6
Milnthorpe
M6
Ulverston
Grange-over-Sands
Arnside
M6

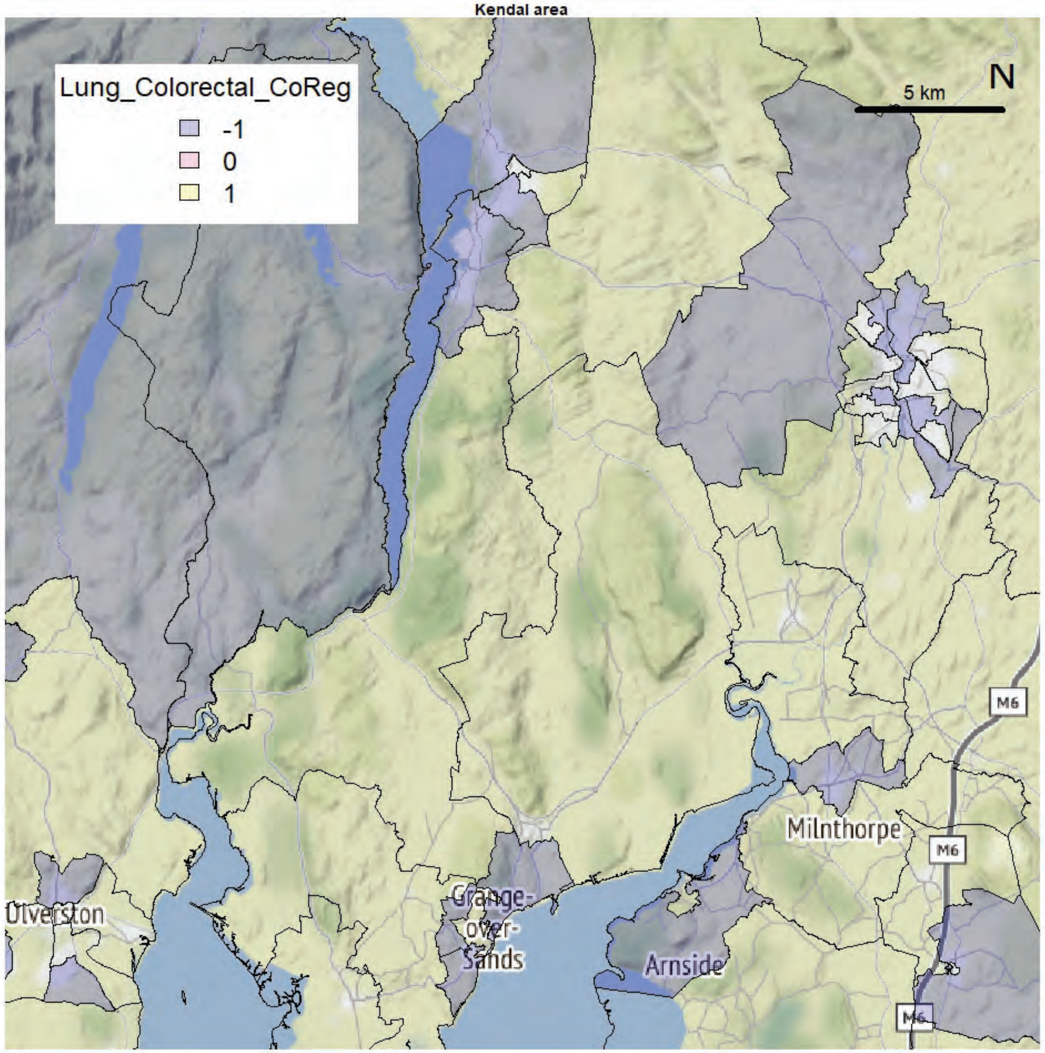
Kendal area
Lung_Colorectal_CoReg
-1
0
1
5 km
N
M6
Milnthorpe
M6
Ulverston
Grange-
over-
Sands
Arnside
M6

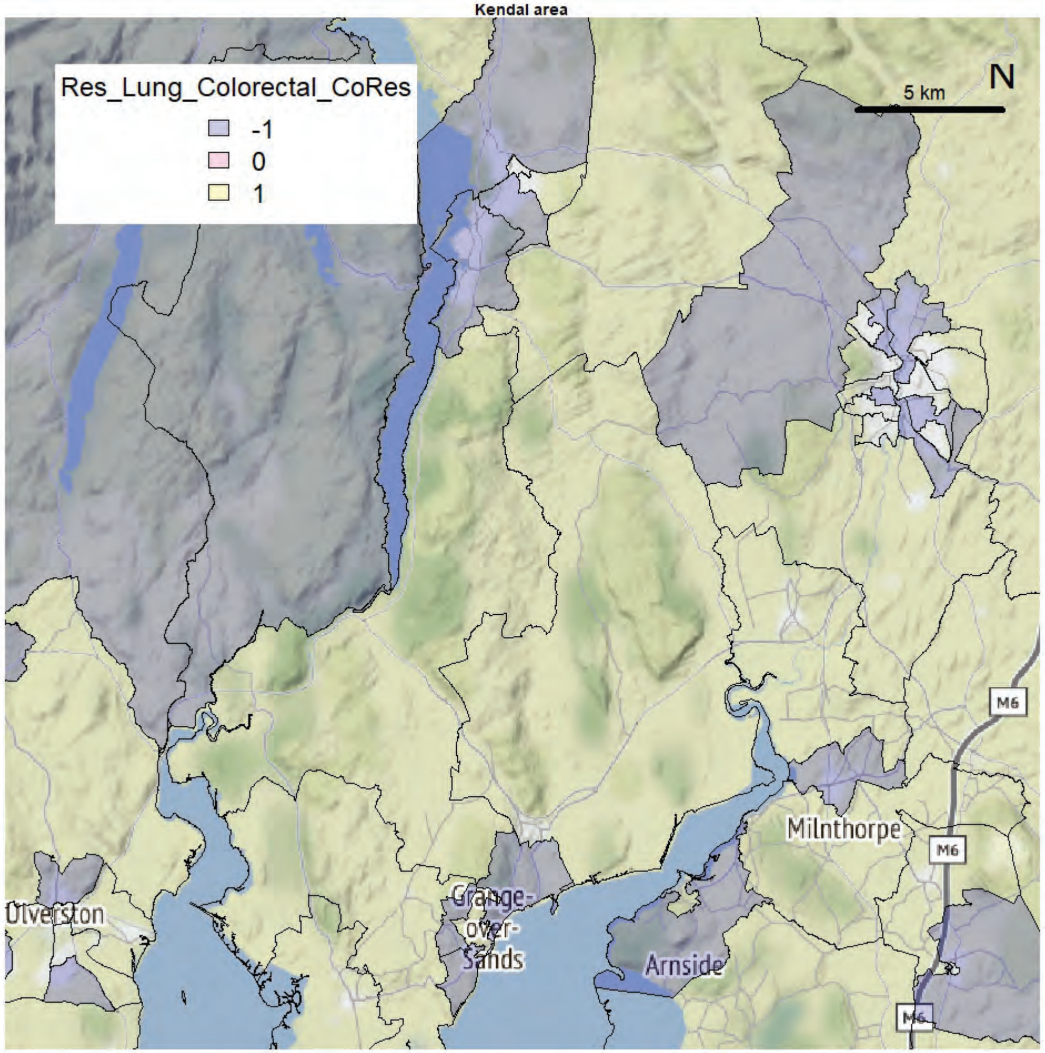
Kendal area
Res_Lung_Colorectal_CoRes
-1
0
1
5 km
N
M6
Milnthorpe
M6
Ulverston
Grange-
over-
Sands
Arnside
M6

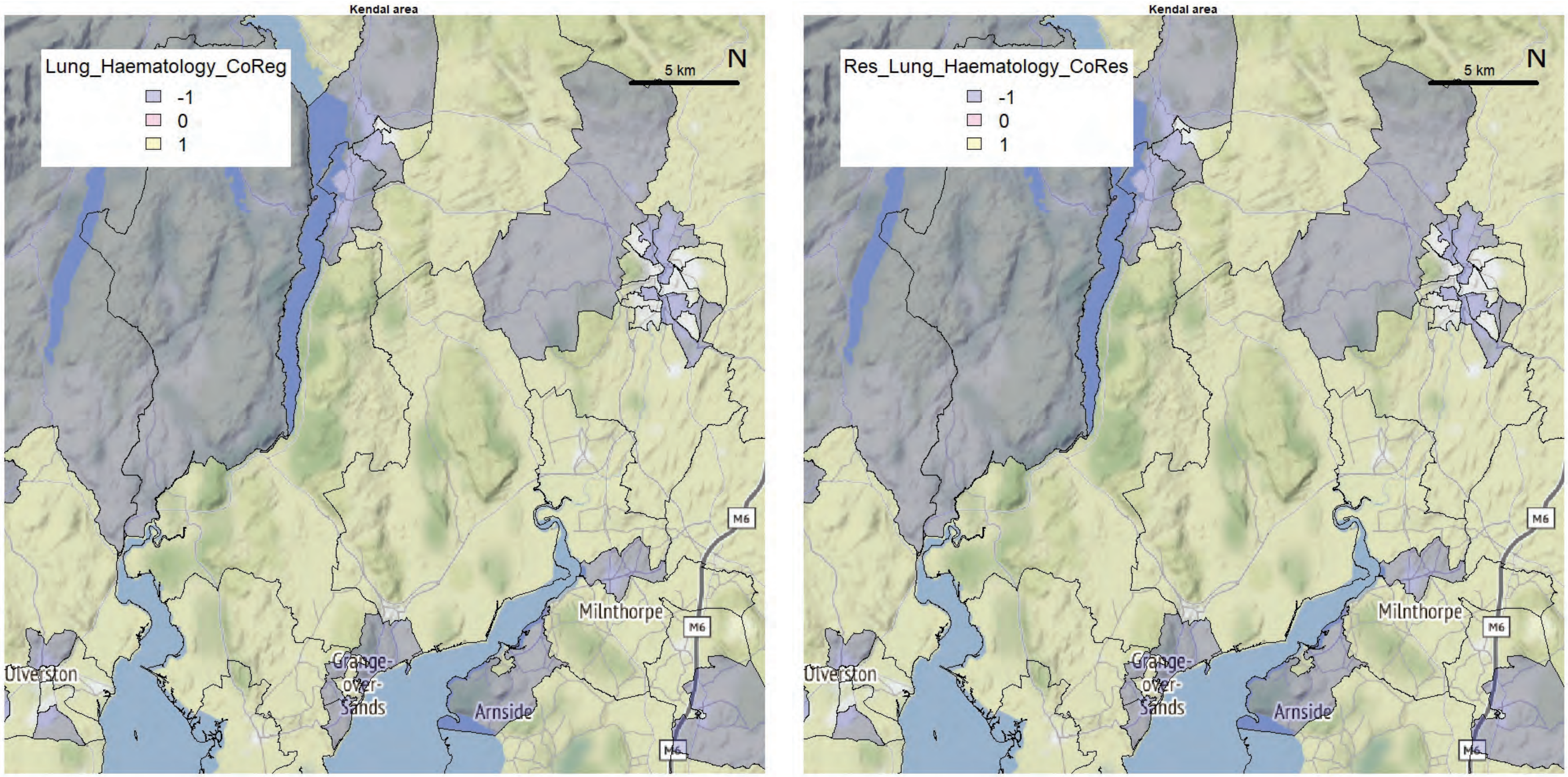

Kendal area
Lung_Haematology_CoReg
-1
0
1
5 km
N
M6
Milnthorpe
Ulverston
Grange-over-Sands
Arnside
Kendal area
Res_Lung_Haematology_CoRes
-1
0
1
5 km
N
M6
Milnthorpe
Ulverston
Grange-over-Sands
Arnside

Kendal area

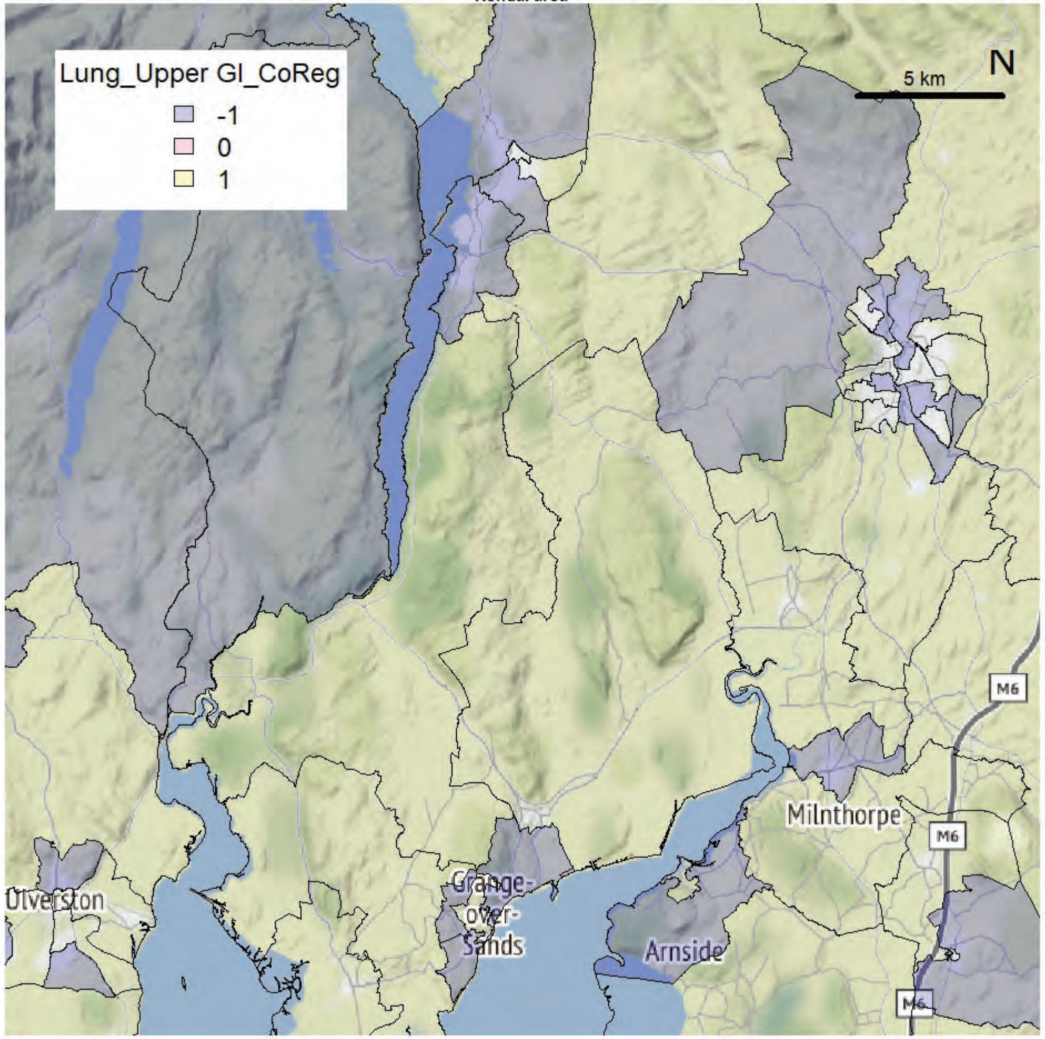

Kendal area

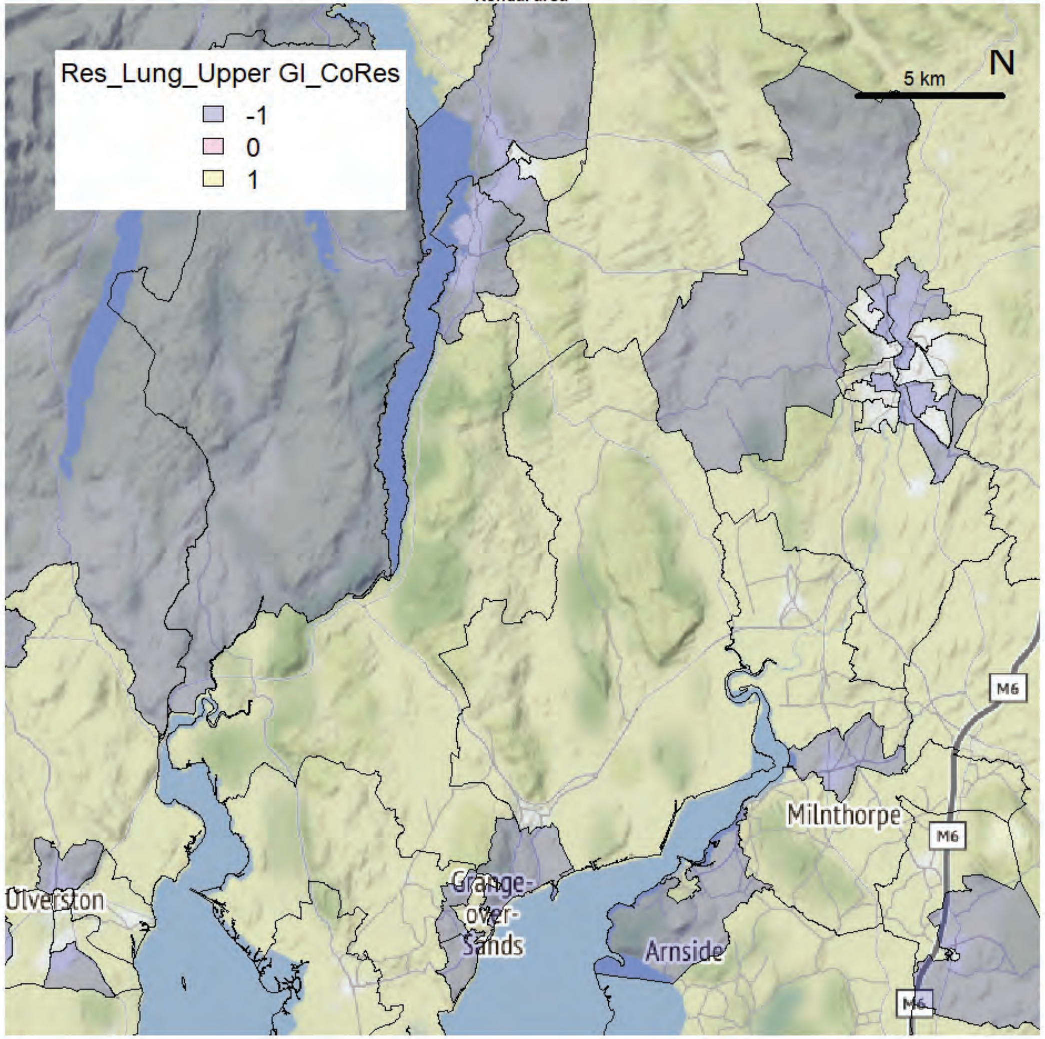

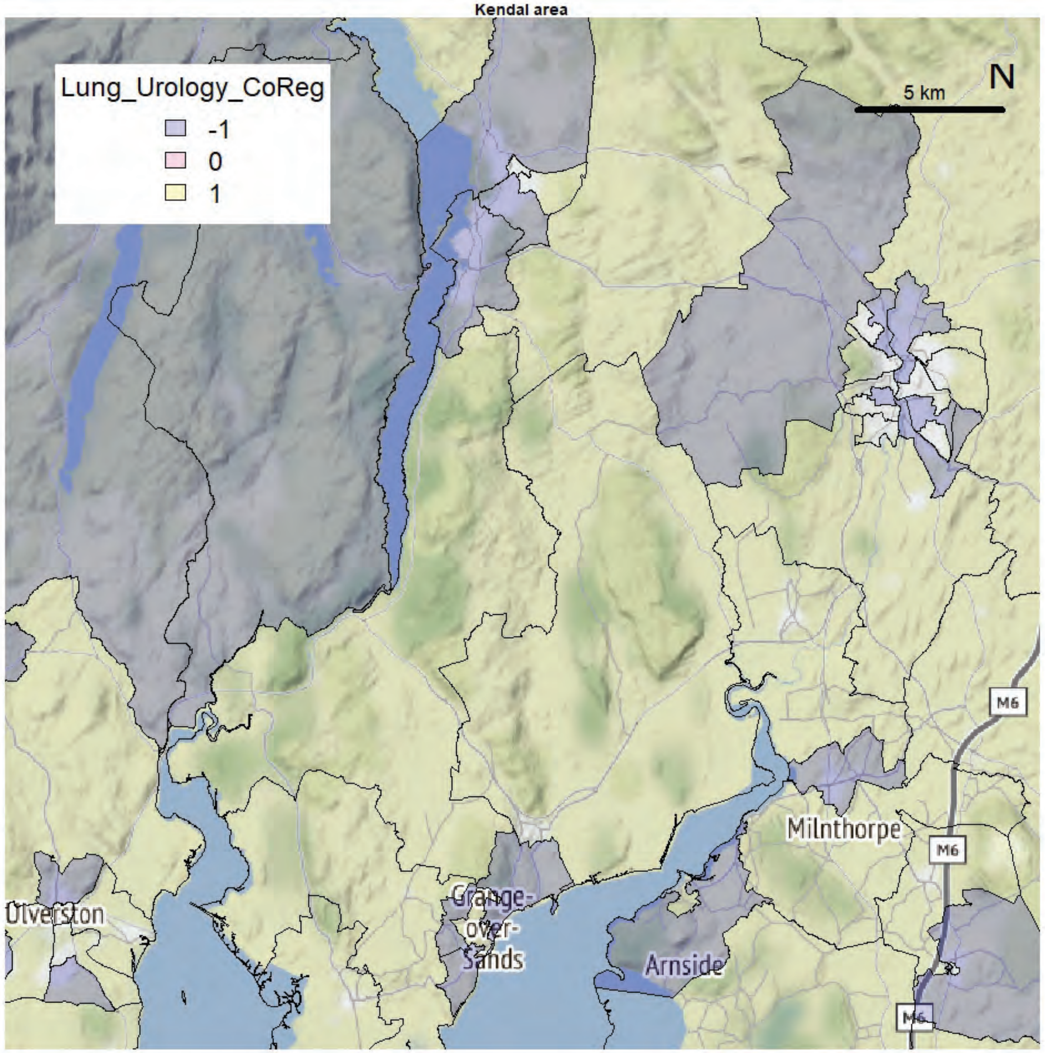
Kendal area
Lung_Urology_CoReg
-1
0
1
5 km
N
M6
Milnthorpe
M6
Ulverston
Grange-over-Sands
Arnside
M6

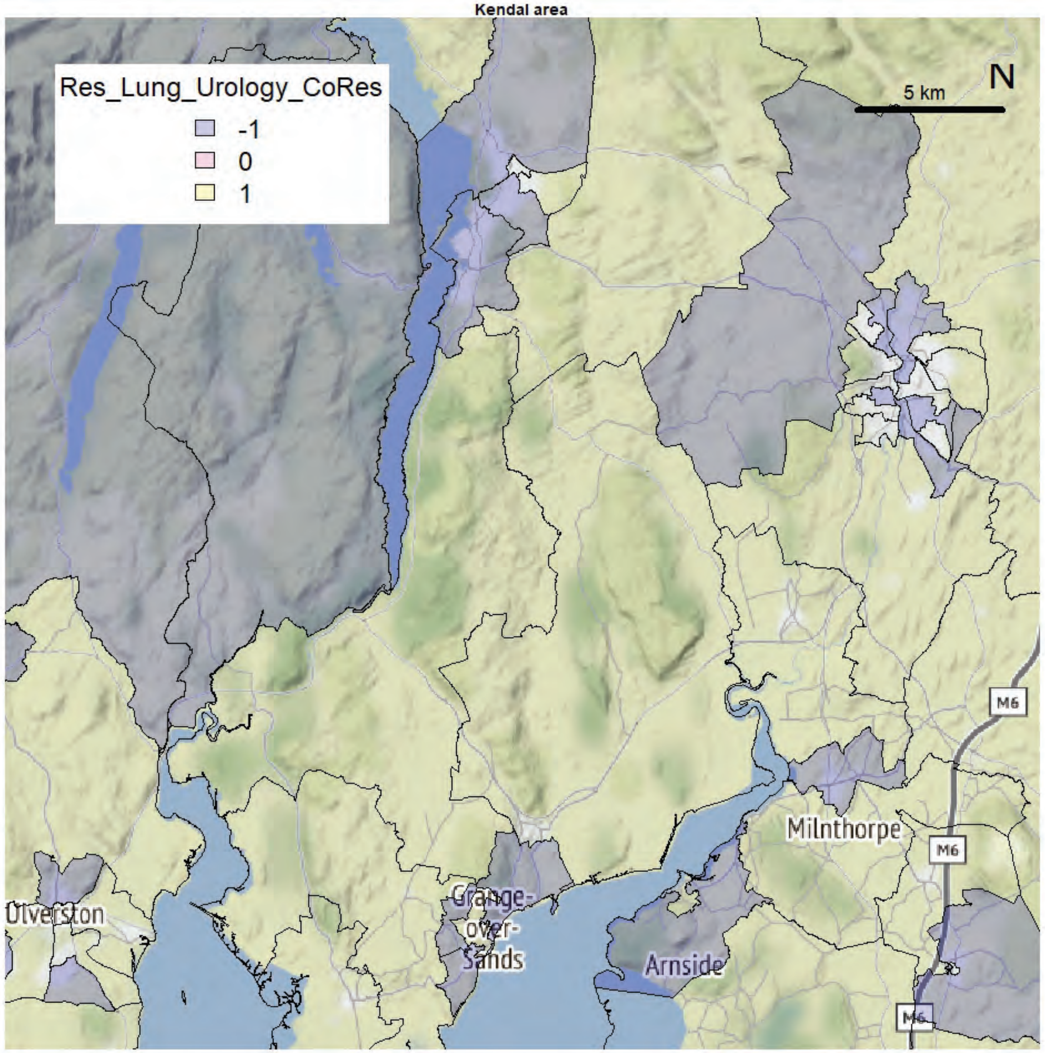
Kendal area
Res_Lung_Urology_CoRes
-1
0
1
5 km
N
M6
Milnthorpe
M6
Ulverston
Grange-over-Sands
Arnside
M6

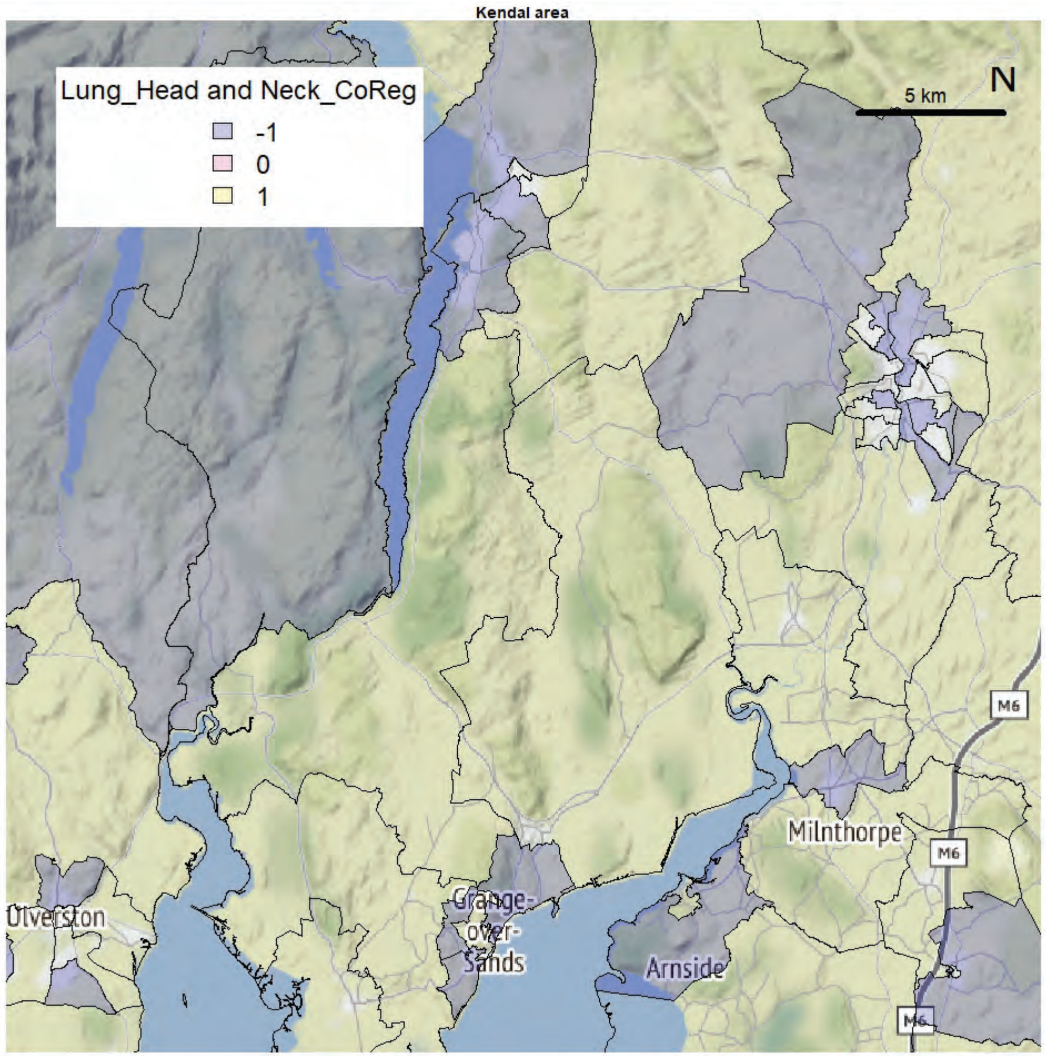
Kendal area
Lung_Head and Neck_CoReg
-1
0
1
5 km
N
M6
Milnthorpe
M6
Ulverston
Grange-
over-
Sands
Arnside
M6

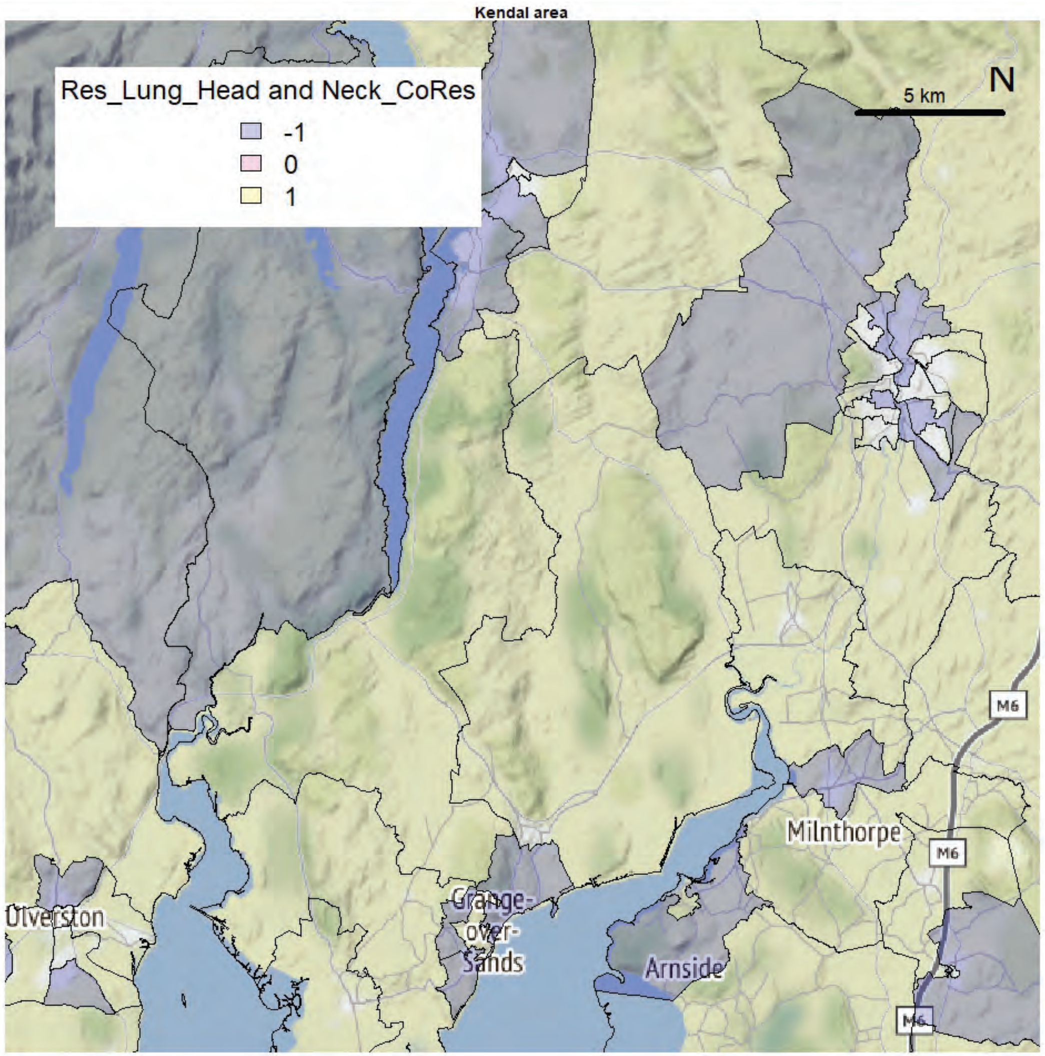
Kendal area
Res_Lung_Head and Neck_CoRes
-1
0
1
5 km
N
M6
Milnthorpe
M6
Ulverston
Grange-
over-
Sands
Arnside
M6

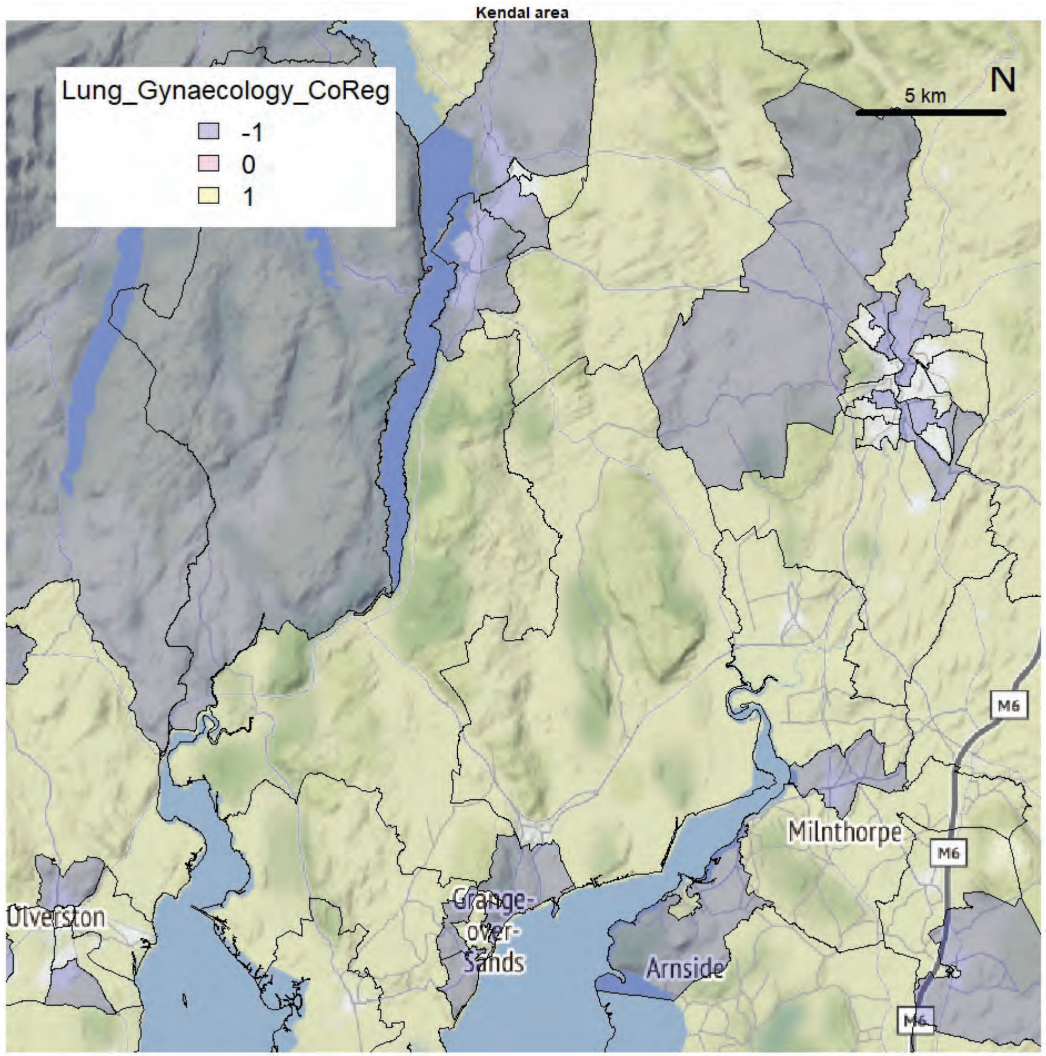
Kendal area
Lung_Gynaecology_CoReg
-1
0
1
5 km
N
M6
Milnthorpe
Ulverston
Grange-over-Sands
Arnside

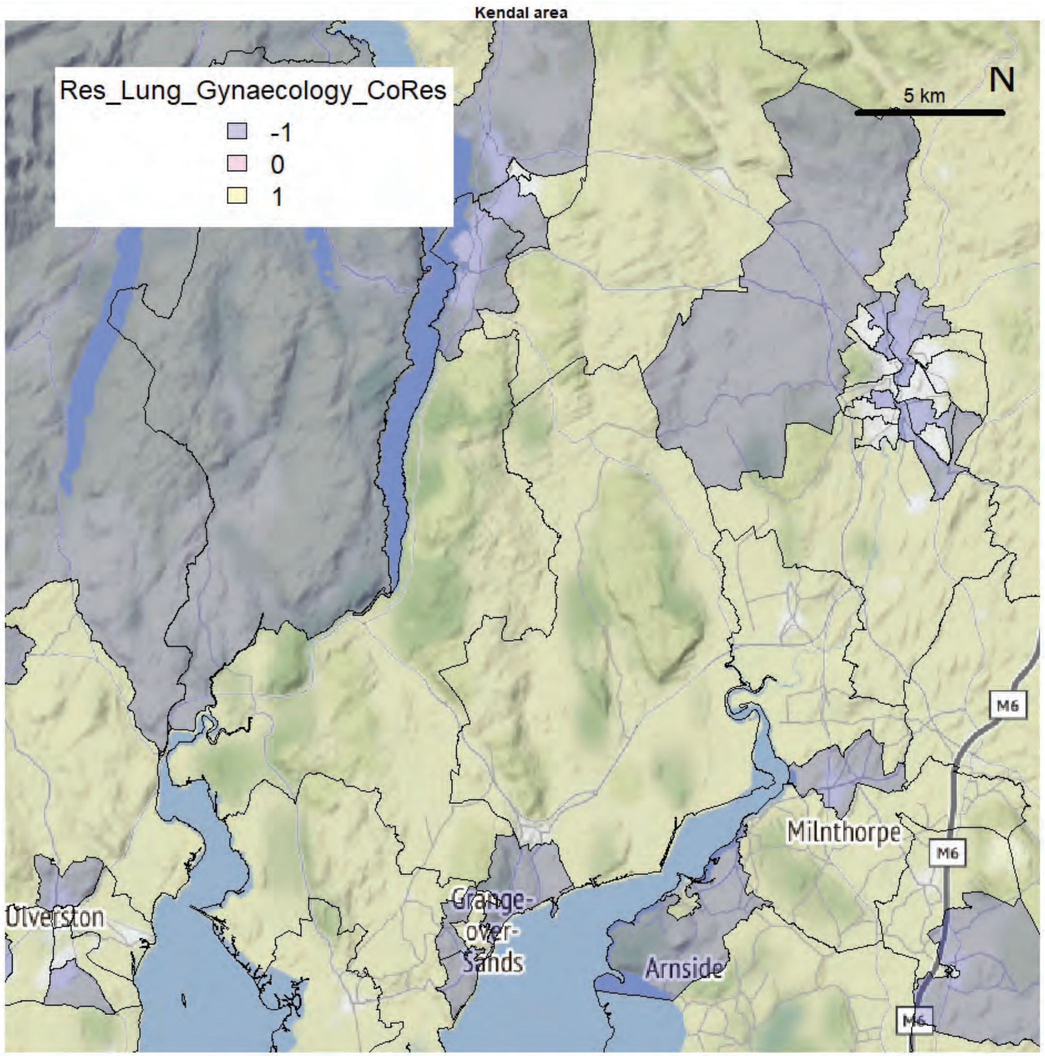
Kendal area
Res_Lung_Gynaecology_CoRes
-1
0
1
5 km
N
M6
Milnthorpe
Ulverston
Grange-over-Sands
Arnside

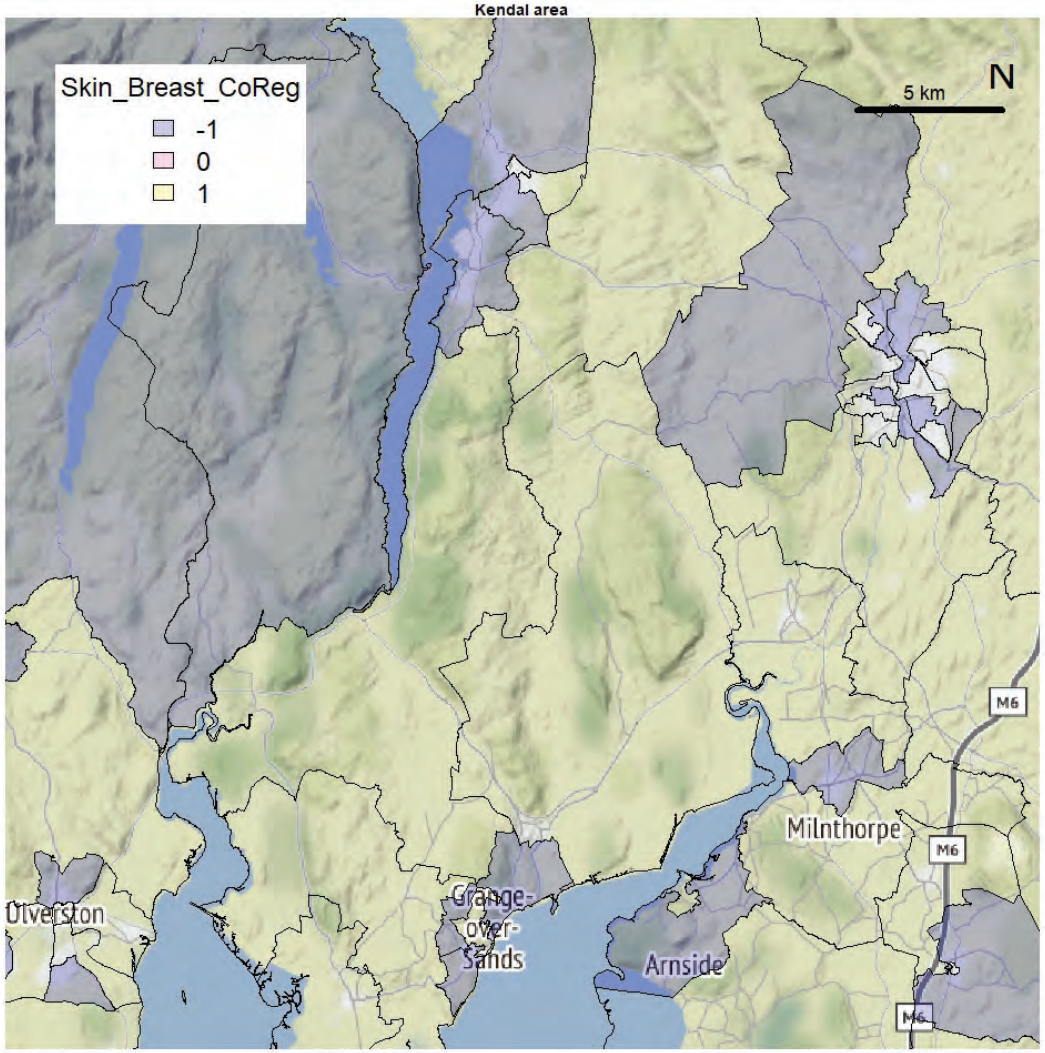
Kendal area
Skin_Breast_CoReg
-1
0
1
5 km
N
M6
Milnthorpe
M6
Ulverston
Grange-
over-
Sands
Arnside
M6

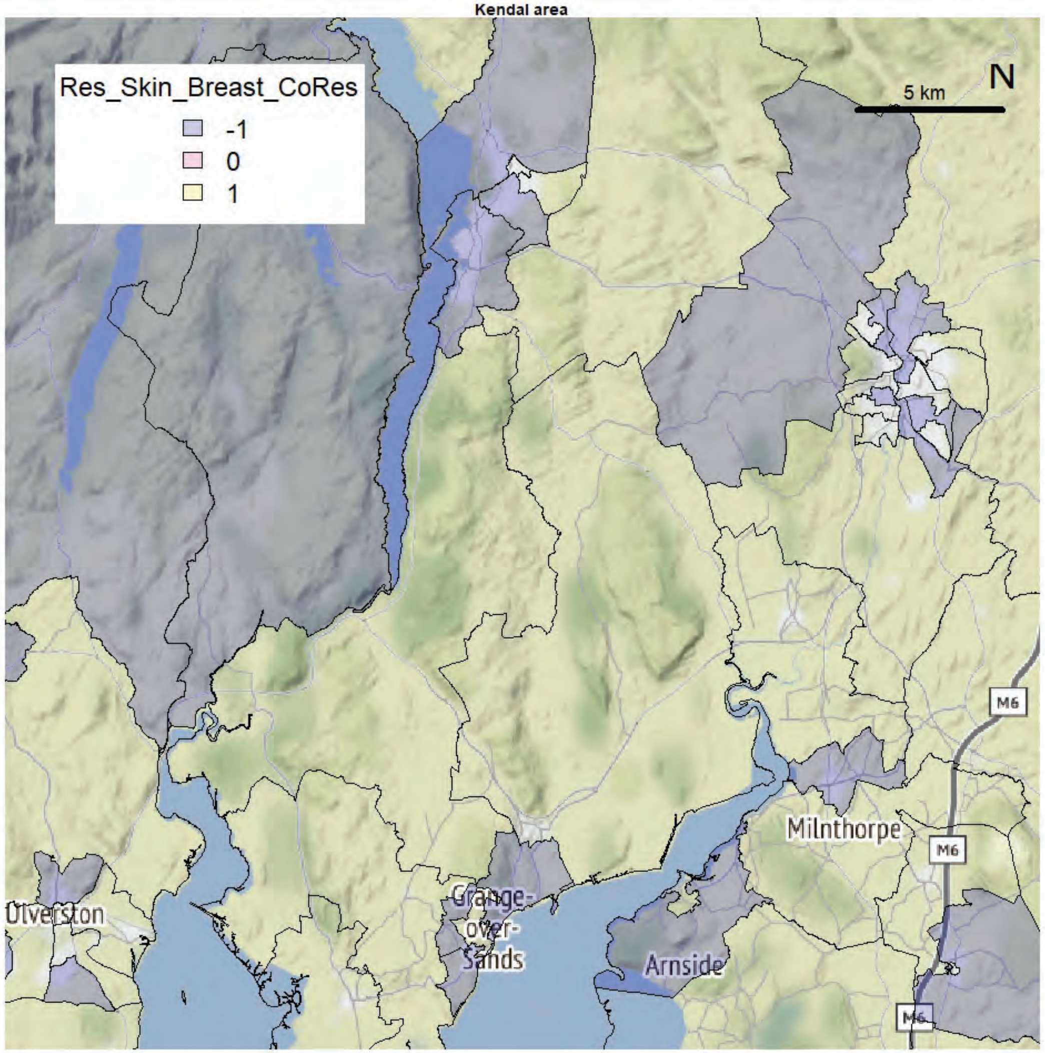
Kendal area
Res_Skin_Breast_CoRes
-1
0
1
5 km
N
M6
Milnthorpe
M6
Ulverston
Grange-
over-
Sands
Arnside
M6

Kendal area

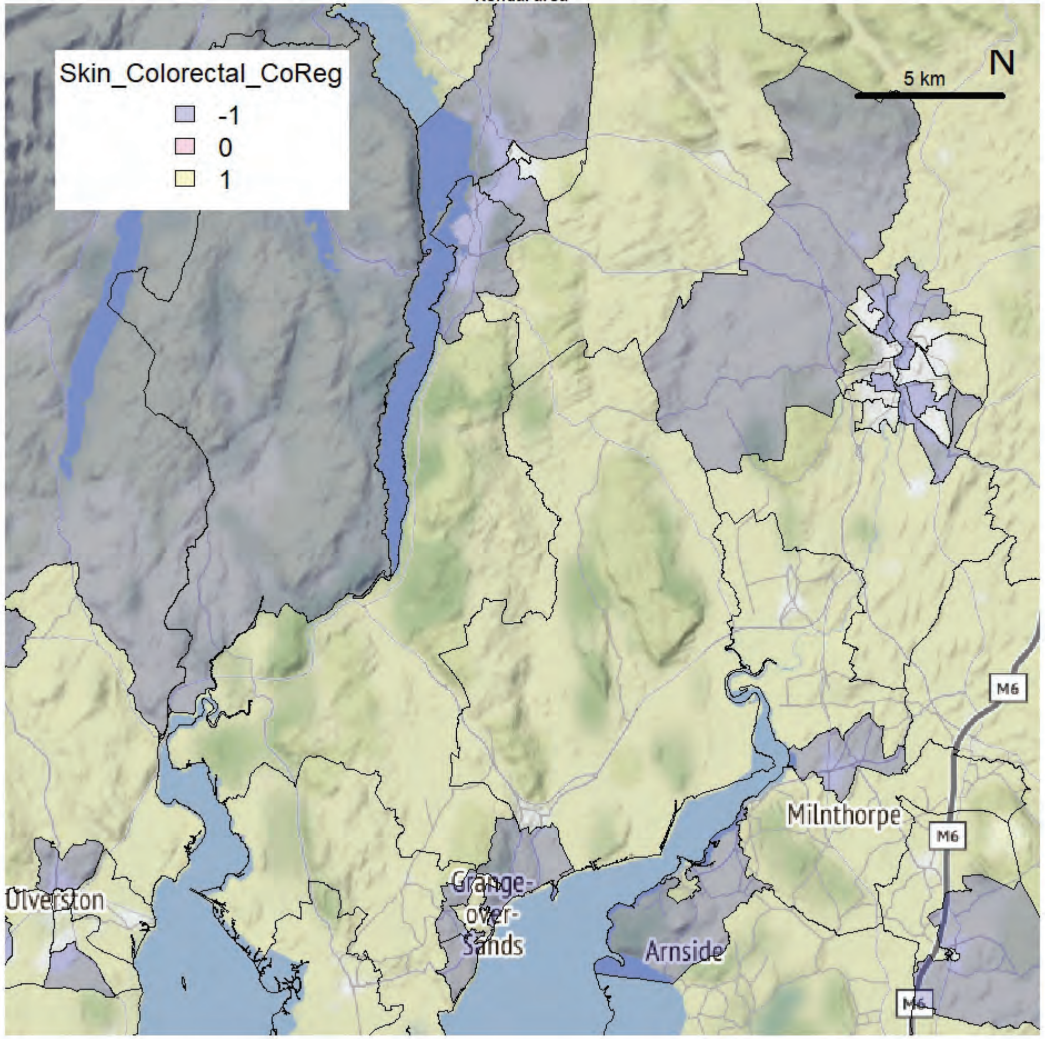

Kendal area

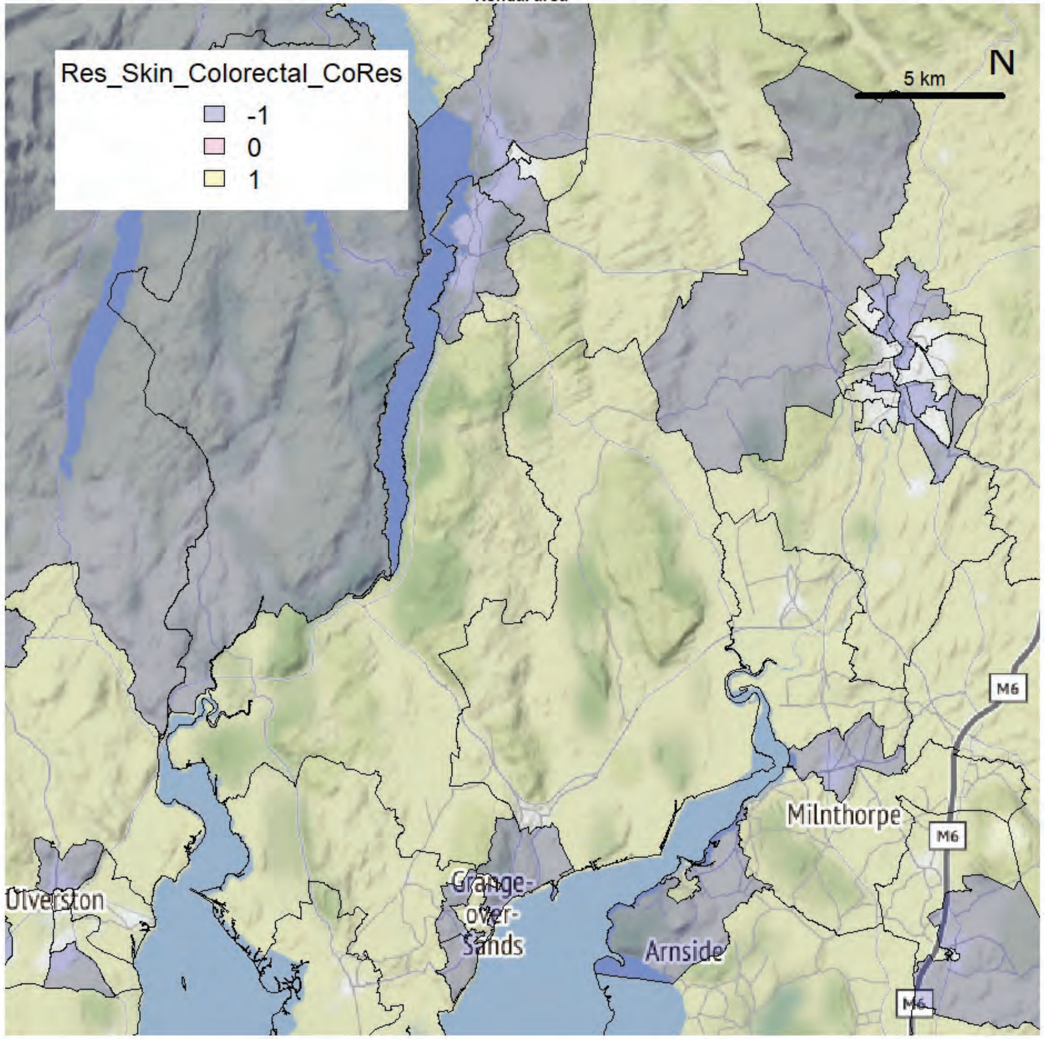

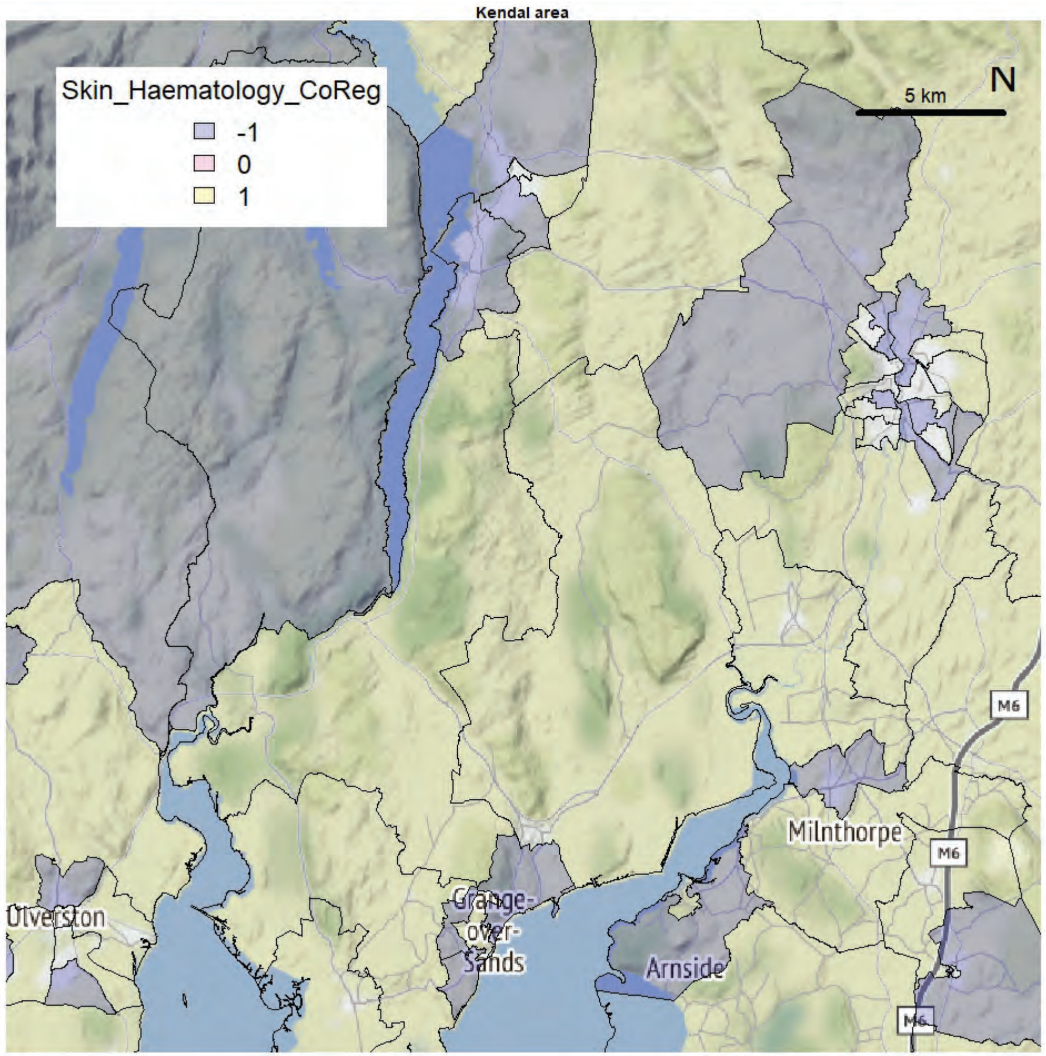

Kendal area
Skin_Haematology_CoReg
-1
0
1
5 km
N
M6
Milnthorpe
Ulverston
Grange-over-Sands
Arnside

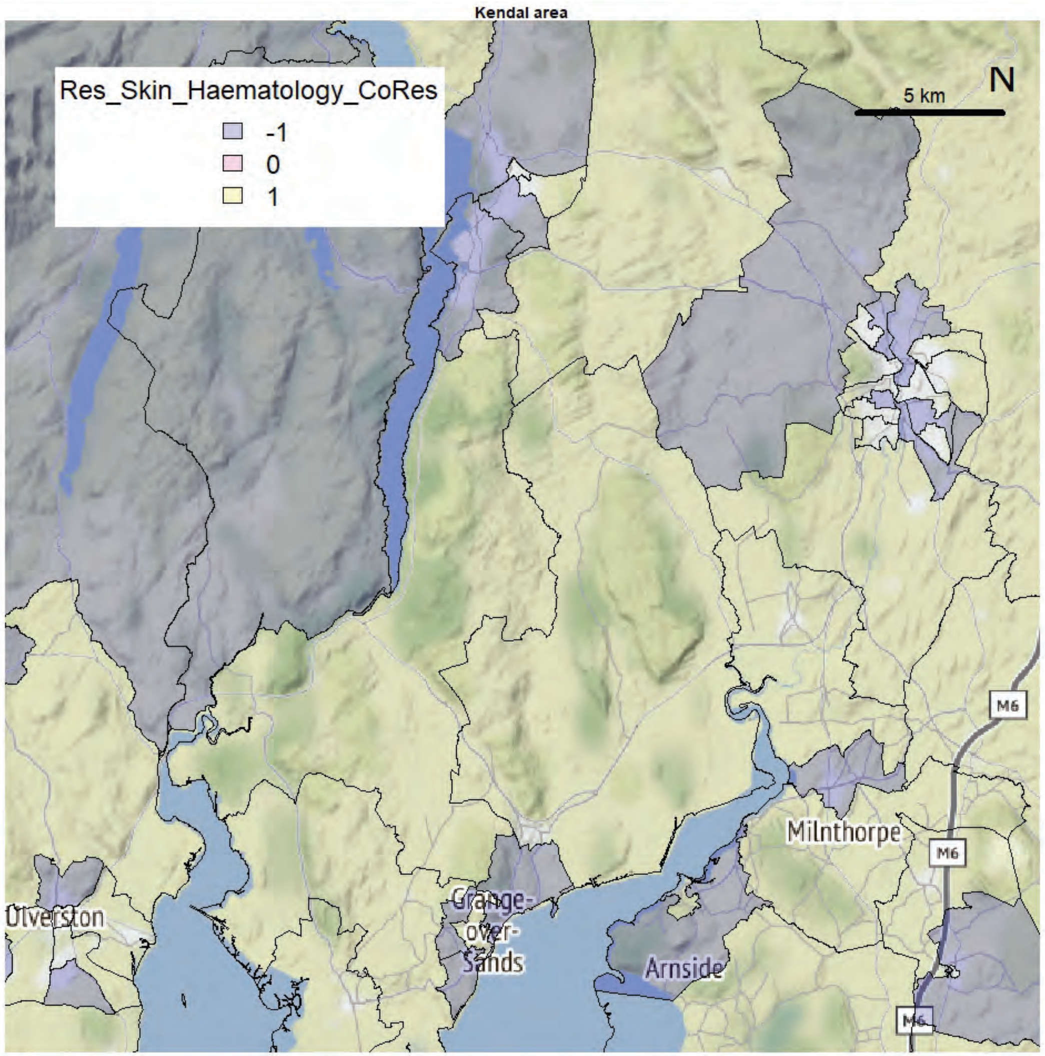

Kendal area
Res_Skin_Haematology_CoRes
-1
0
1
5 km
N
M6
Milnthorpe
Ulverston
Grange-over-Sands
Arnside

Kendal area

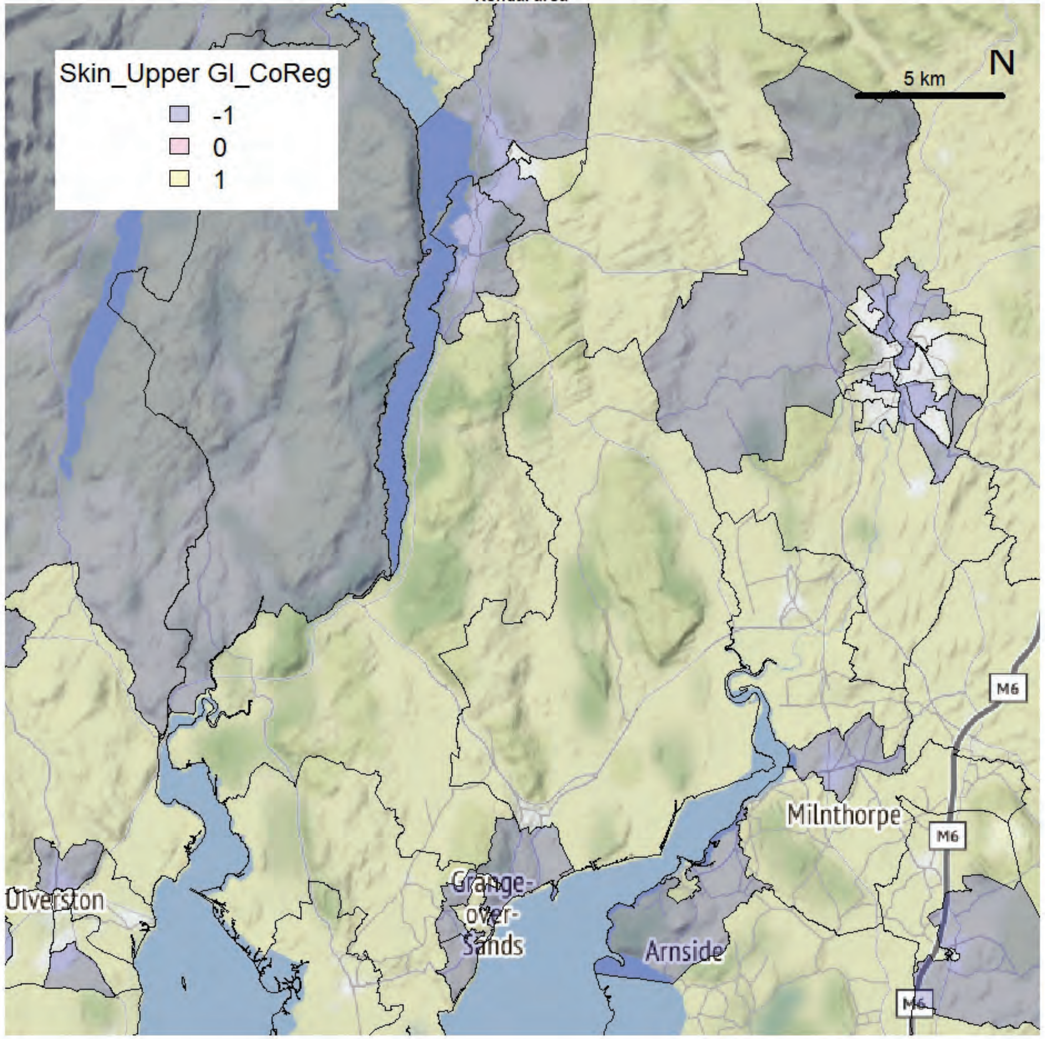

Kendal area

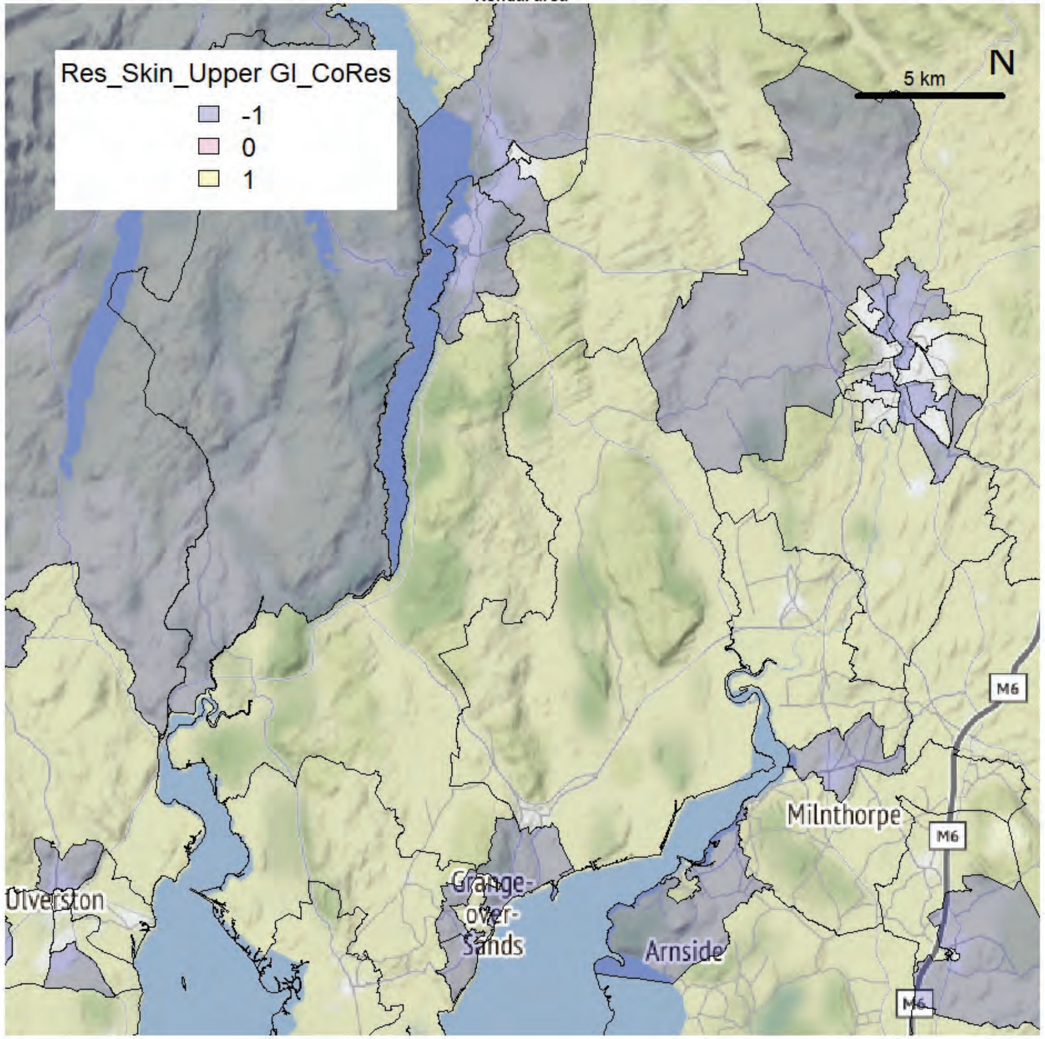

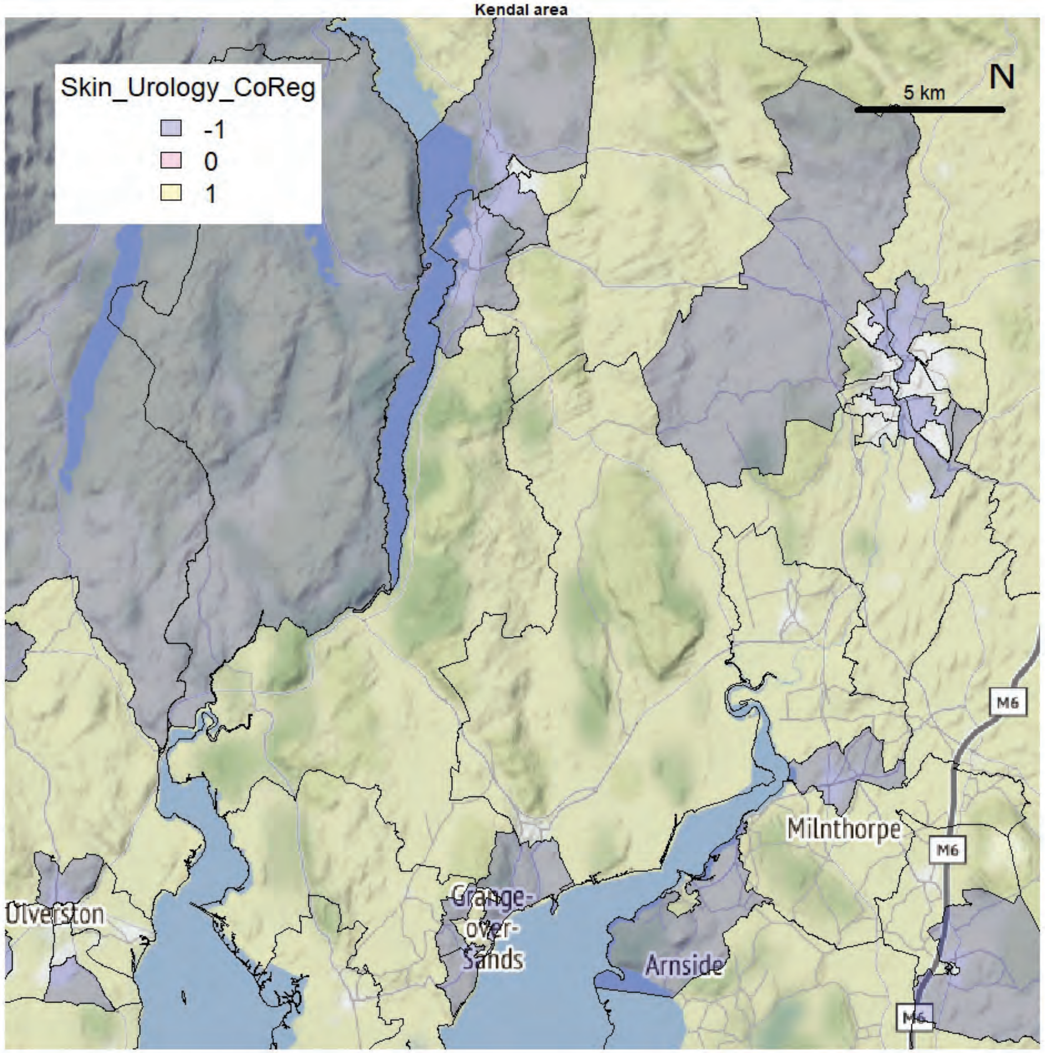
Kendal area
Skin_Urology_CoReg
-1
0
1
5 km
N
M6
Milnthorpe
M6
Ulverston
Grange-
over-
Sands
Arnside
M6

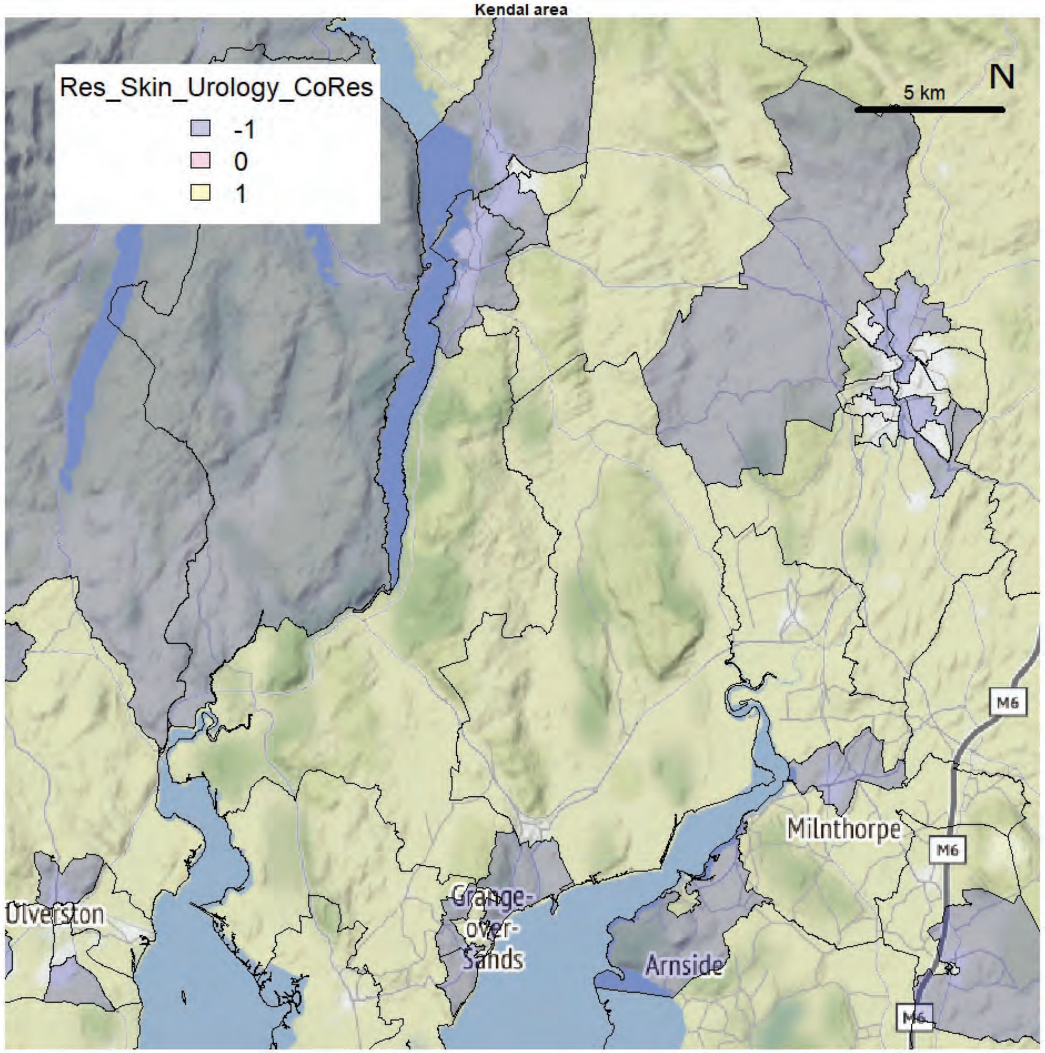
Kendal area
Res_Skin_Urology_CoRes
-1
0
1
5 km
N
M6
Milnthorpe
M6
Ulverston
Grange-
over-
Sands
Arnside
M6

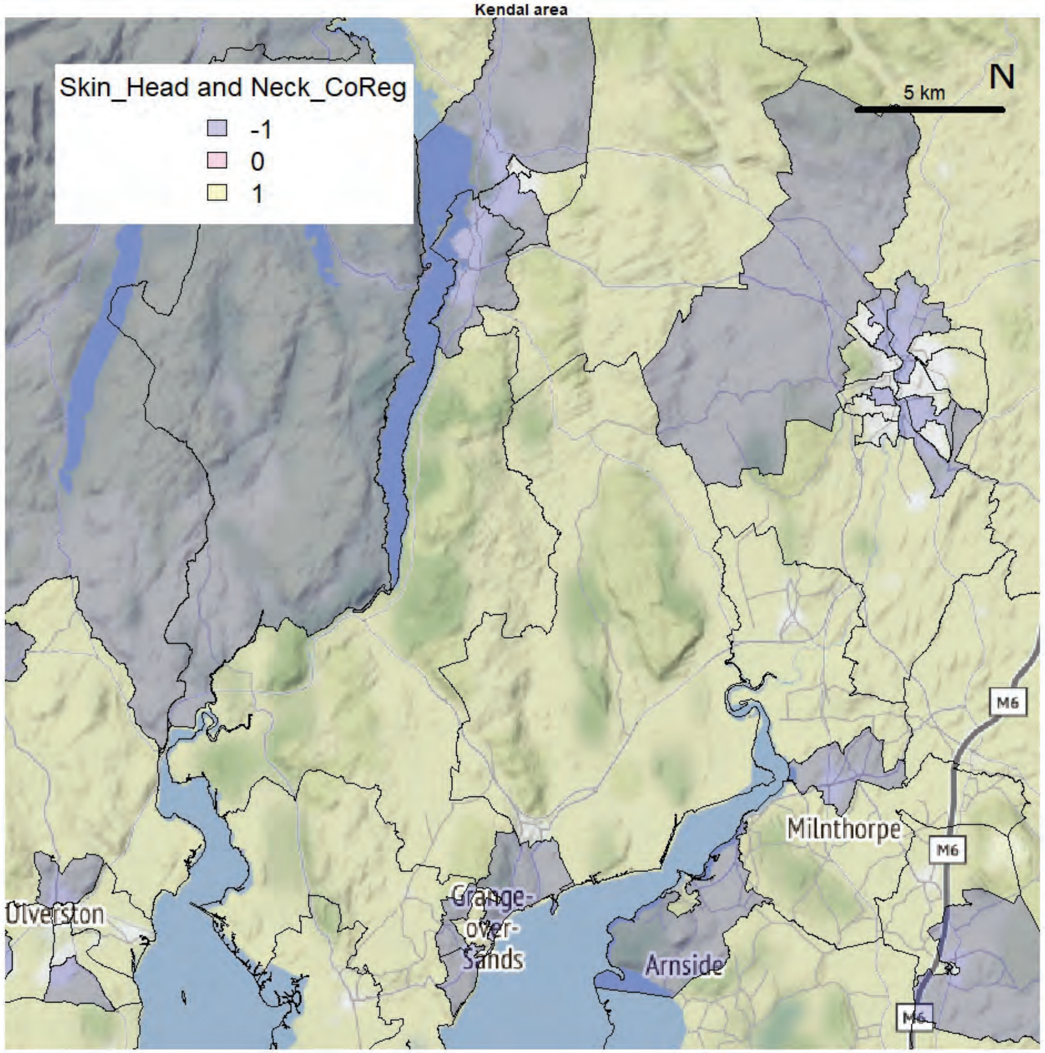
Kendal area
Skin_Head and Neck_CoReg
-1
0
1
5 km
N
M6
Milnthorpe
Ulverston
Grange-over-Sands
Arnside

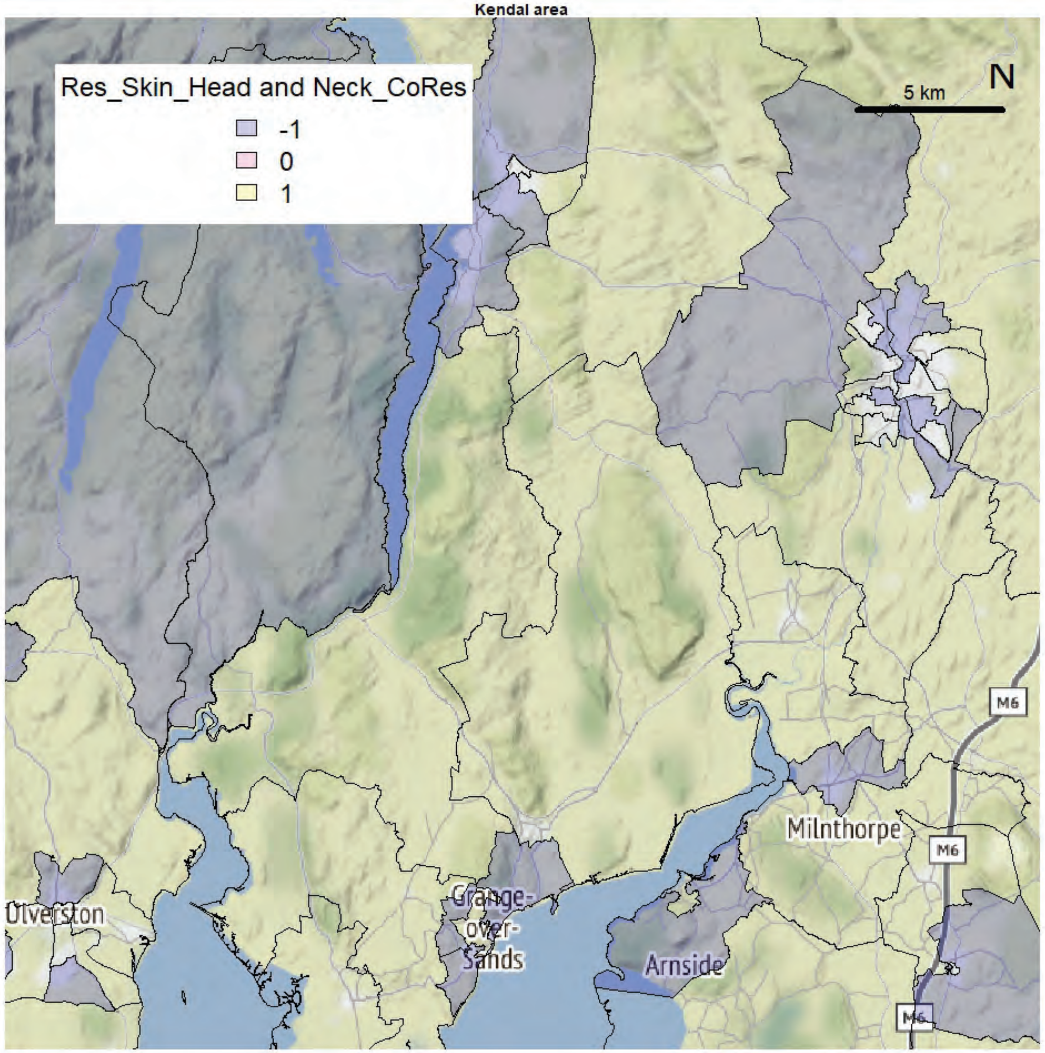
Kendal area
Res_Skin_Head and Neck_CoRes
-1
0
1
5 km
N
M6
Milnthorpe
Ulverston
Grange-over-Sands
Arnside

Kendal area

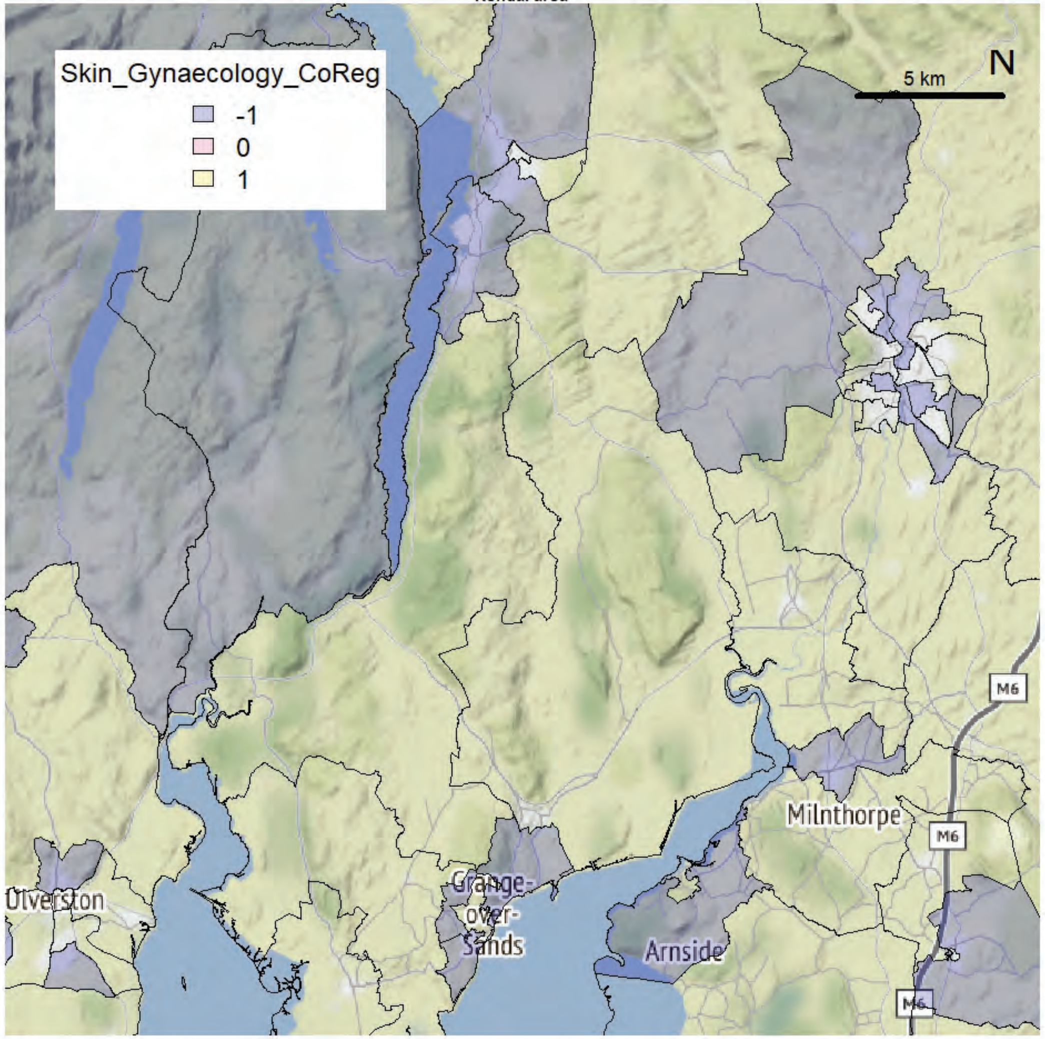

Kendal area

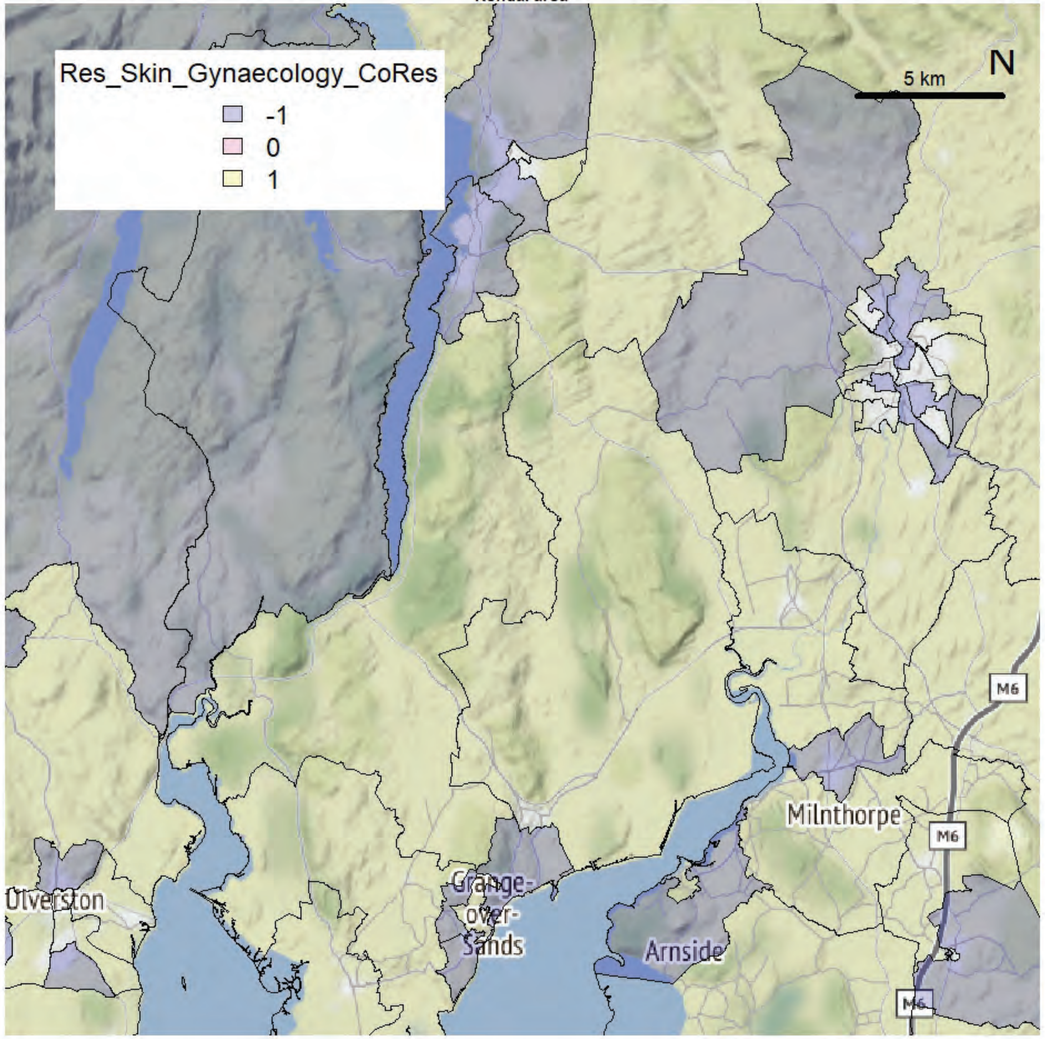

Kendal area

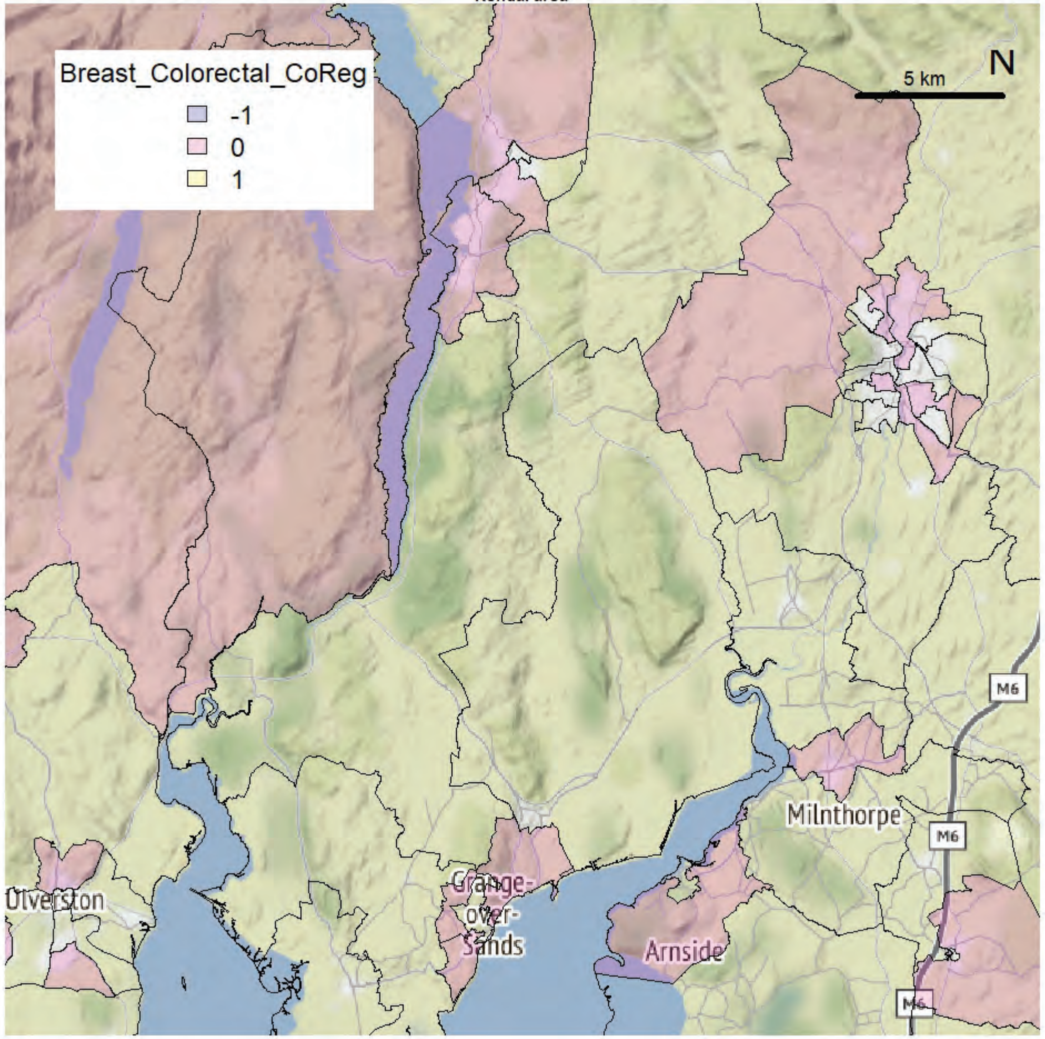

Kendal area

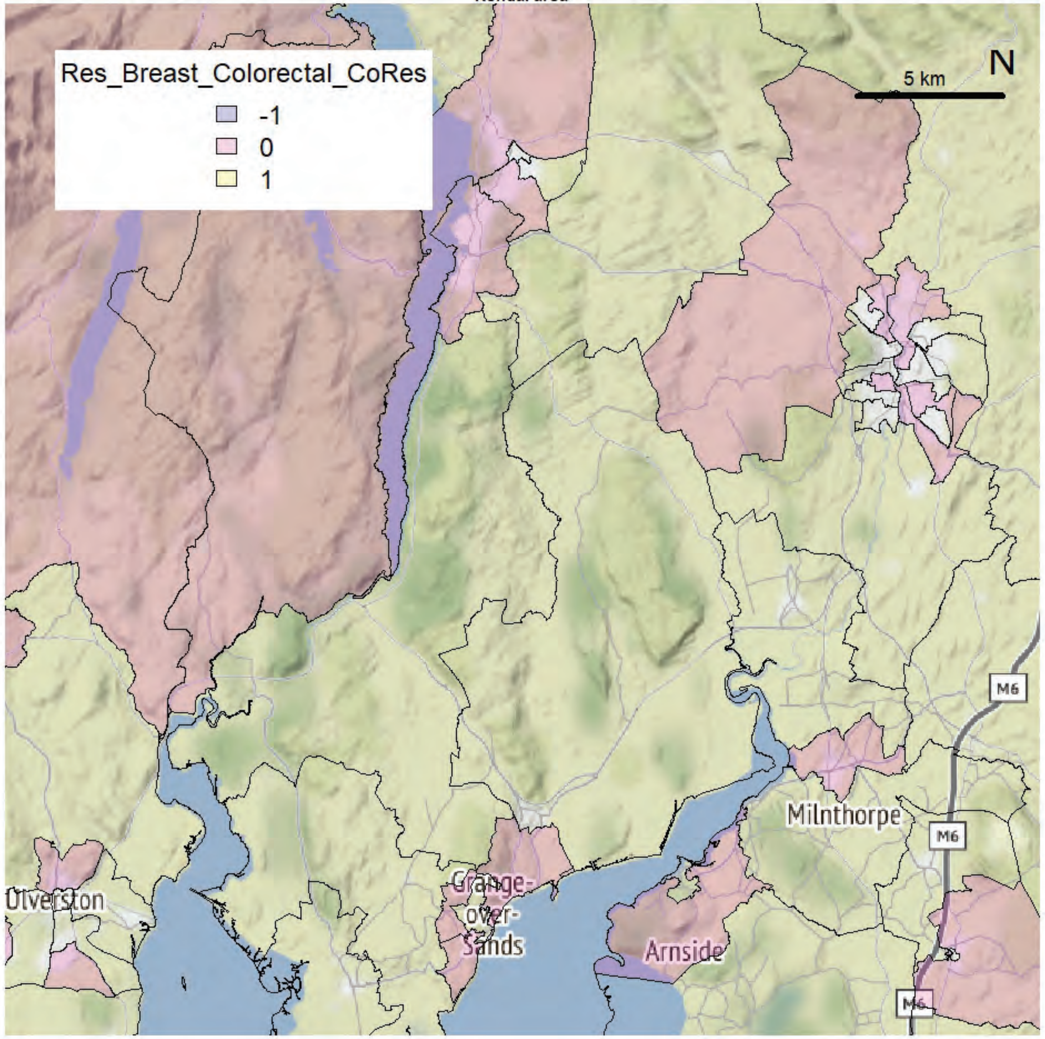

Kendal area

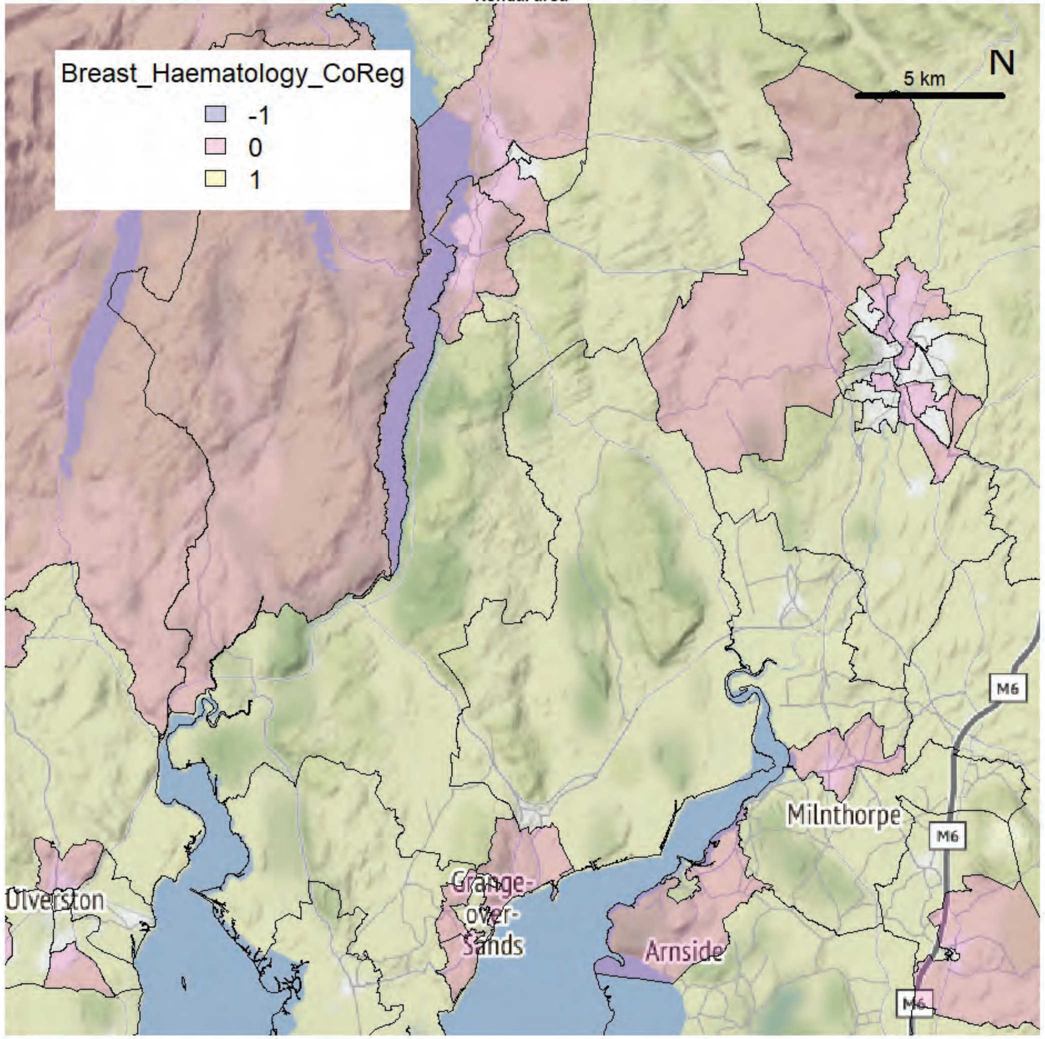

Kendal area

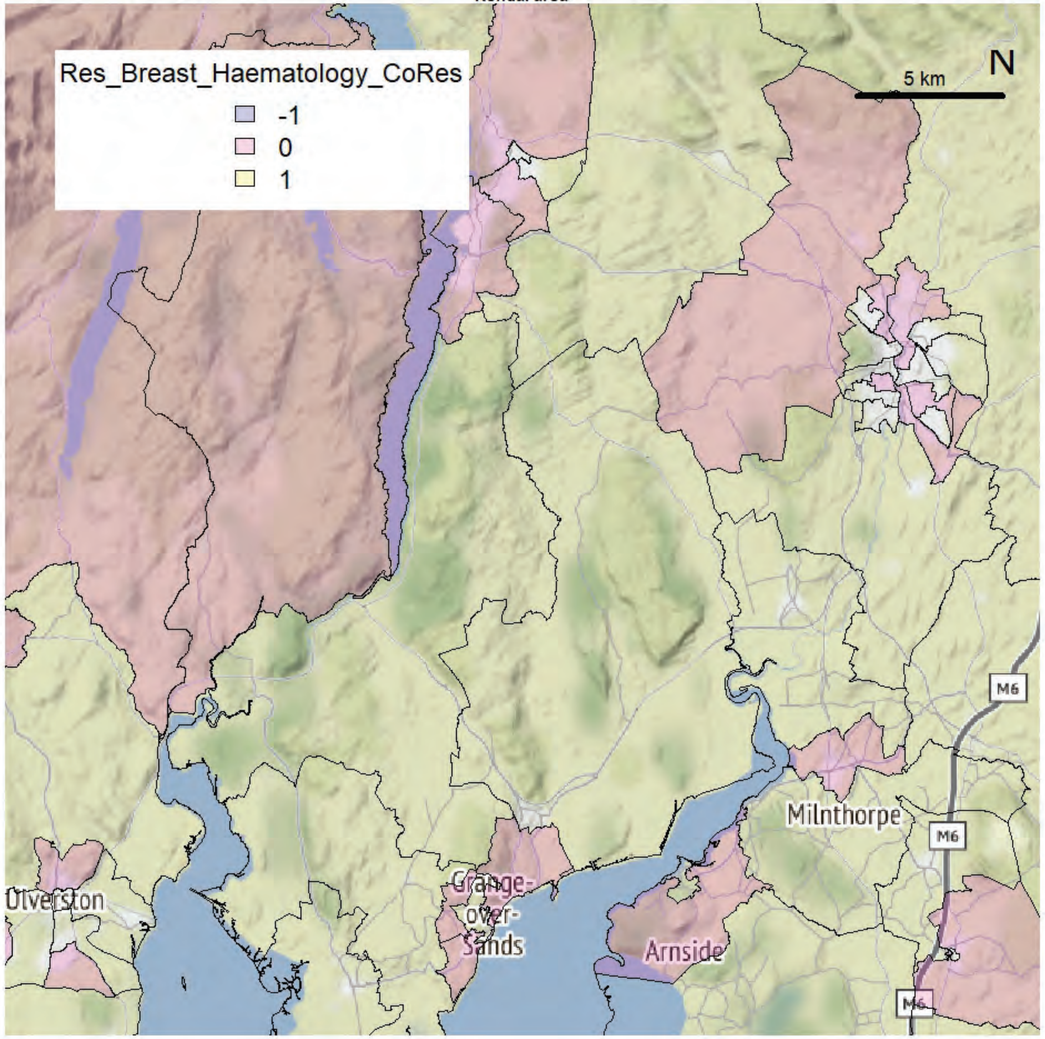

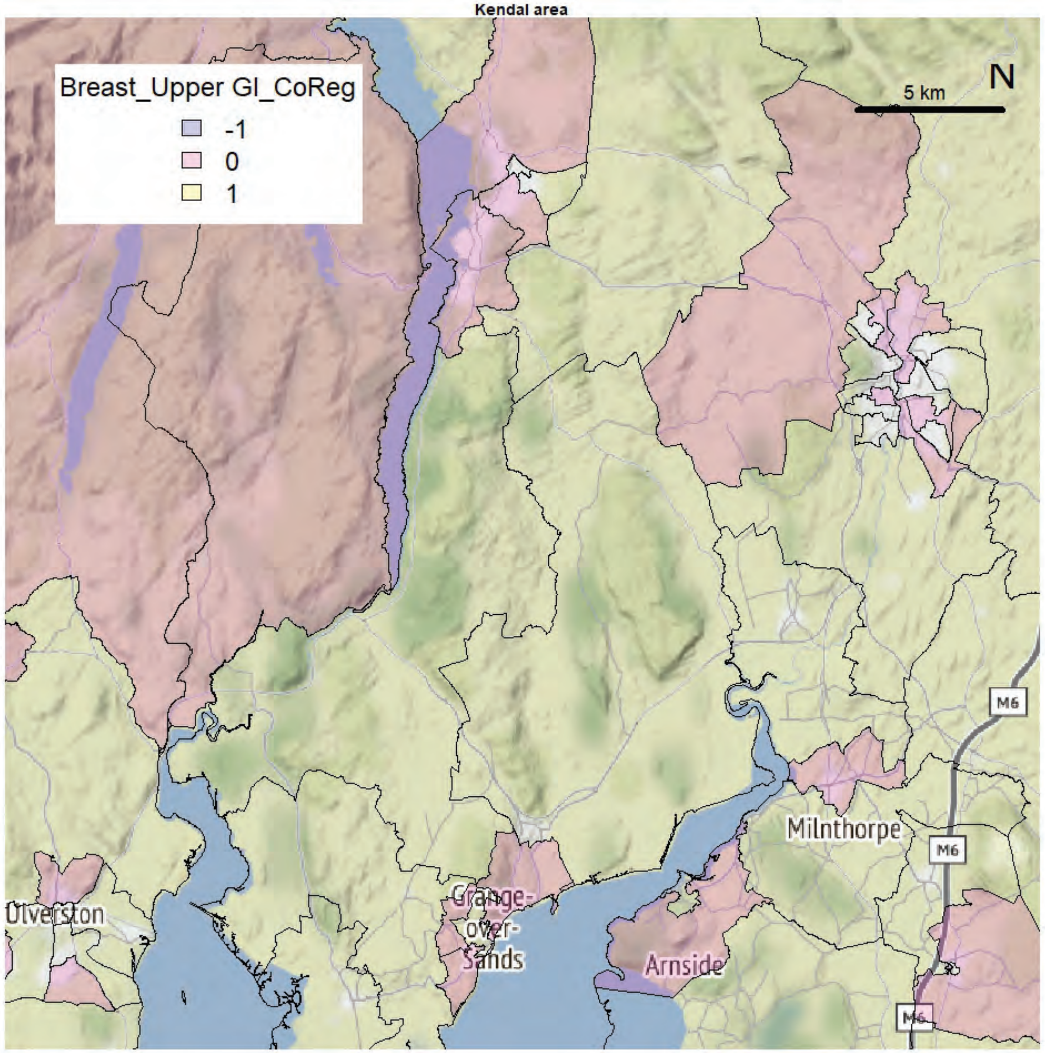
Kendal area
Breast_Upper GI_CoReg
-1
0
1
5 km
N
M6
Milnthorpe
M6
Ulverston
Grange-
over-
Sands
Arnside
M6

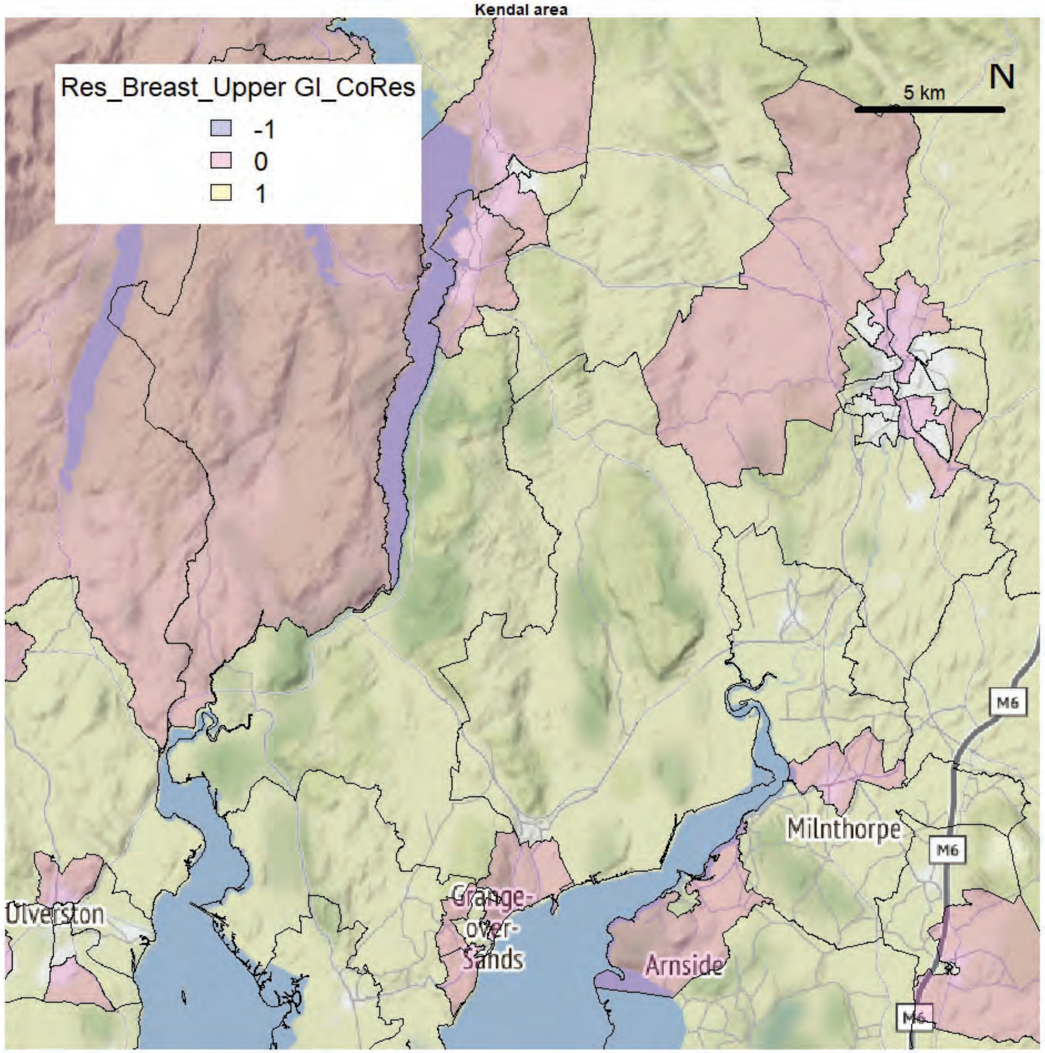
Kendal area
Res_Breast_Upper GI_CoRes
-1
0
1
5 km
N
M6
Milnthorpe
M6
Ulverston
Grange-
over-
Sands
Arnside
M6

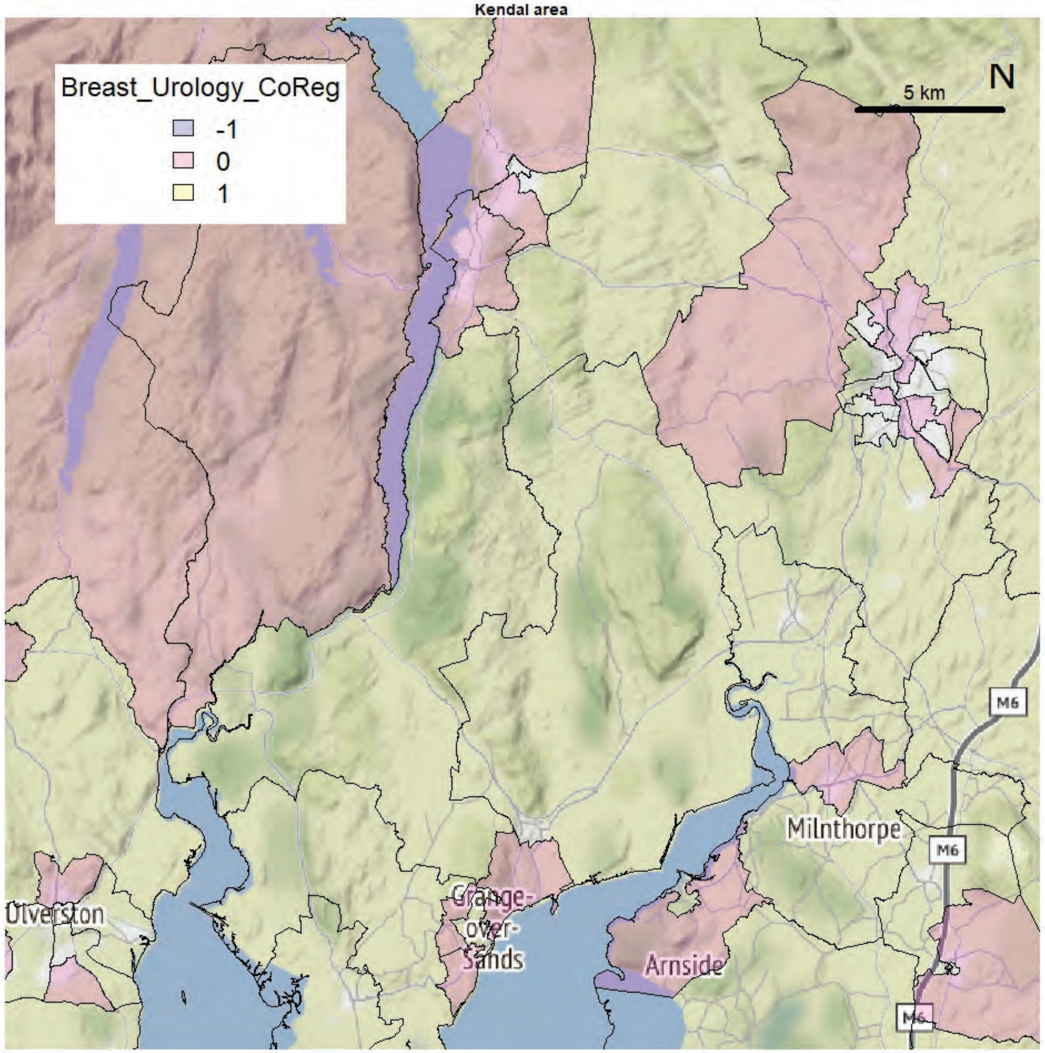
Kendal area
Breast_Urology_CoReg
-1
0
1
5 km
N
M6
Milnthorpe
Ulverston
Grange-over-Sands
Arnside

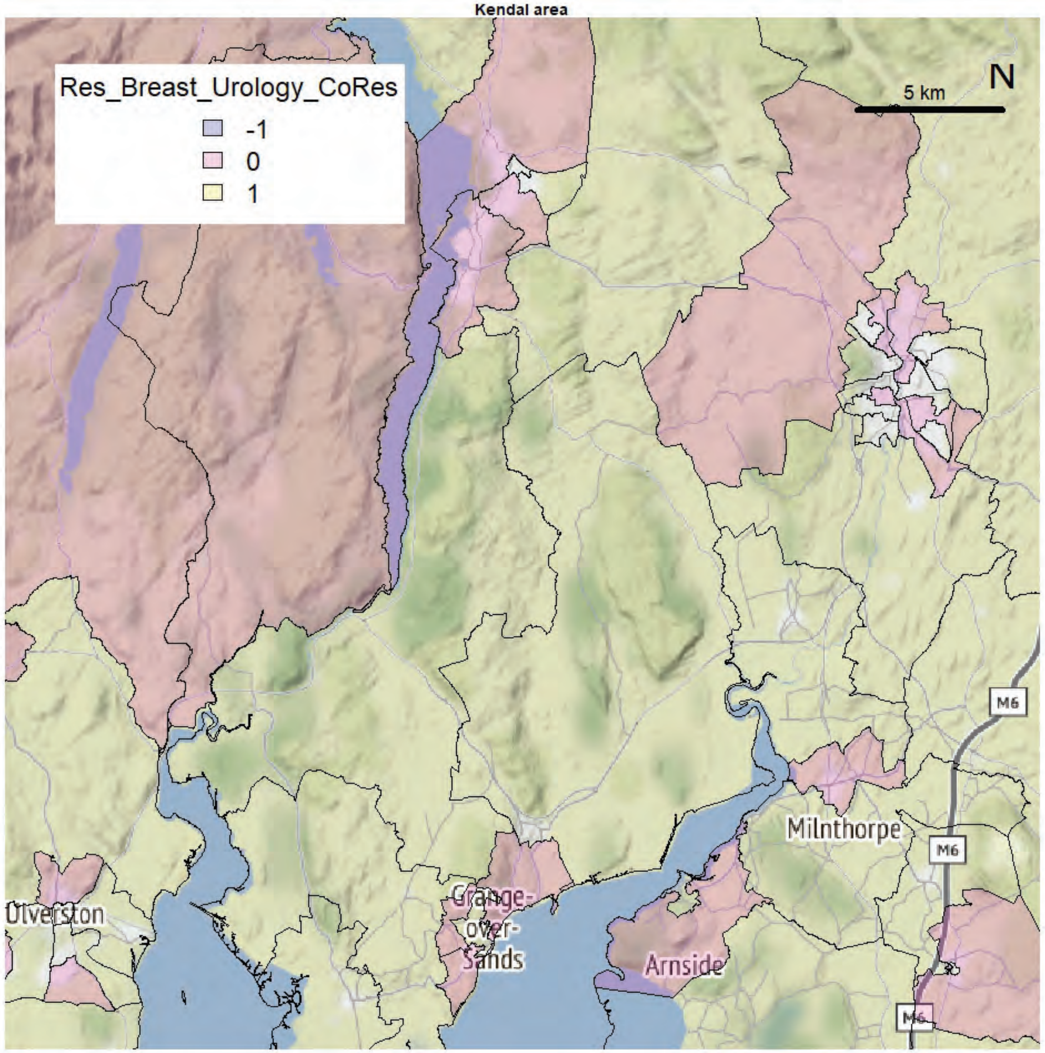
Kendal area
Res_Breast_Urology_CoRes
-1
0
1
5 km
N
M6
Milnthorpe
Ulverston
Grange-over-Sands
Arnside

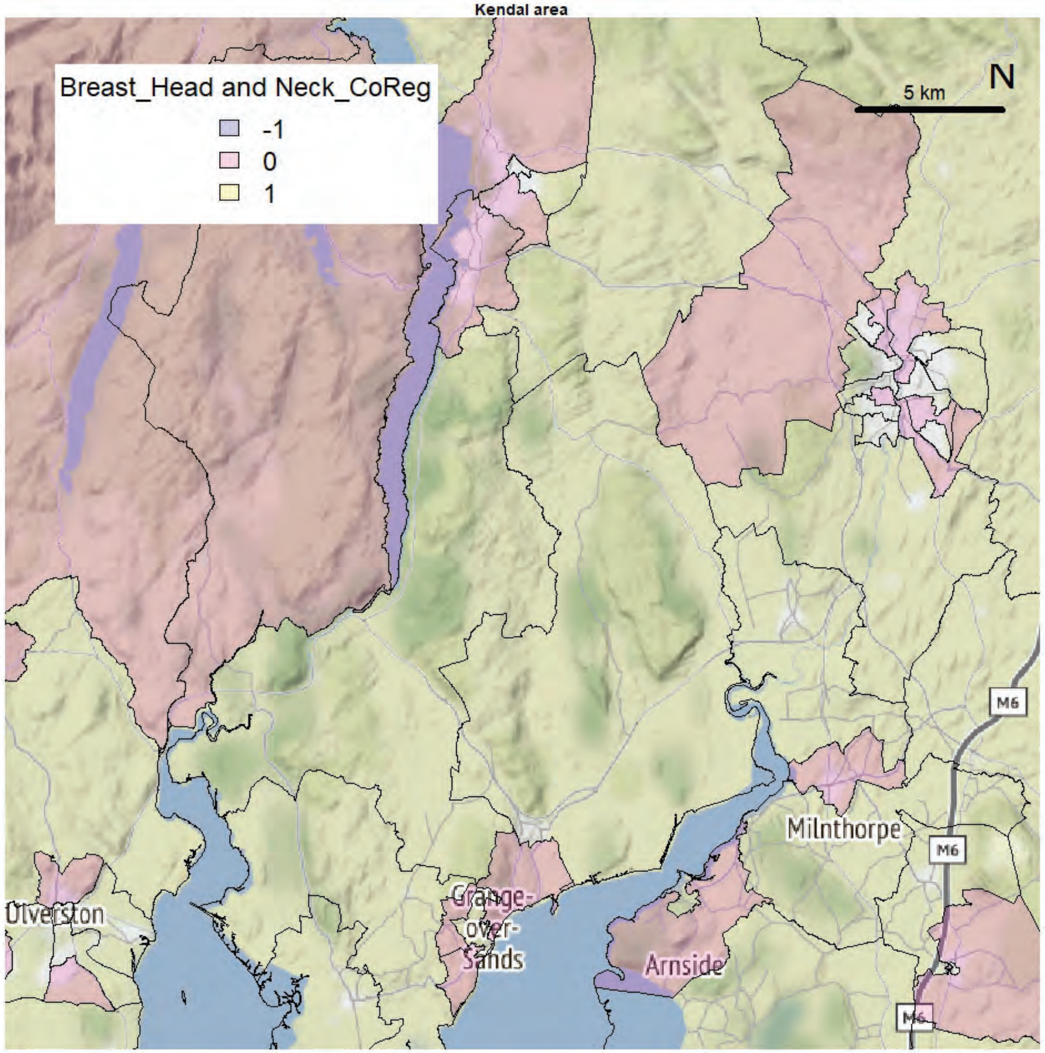
Kendal area
Breast_Head and Neck_CoReg
-1
0
1
5 km
N
M6
Milnthorpe
Ulverston
Grange-over-Sands
Arnside

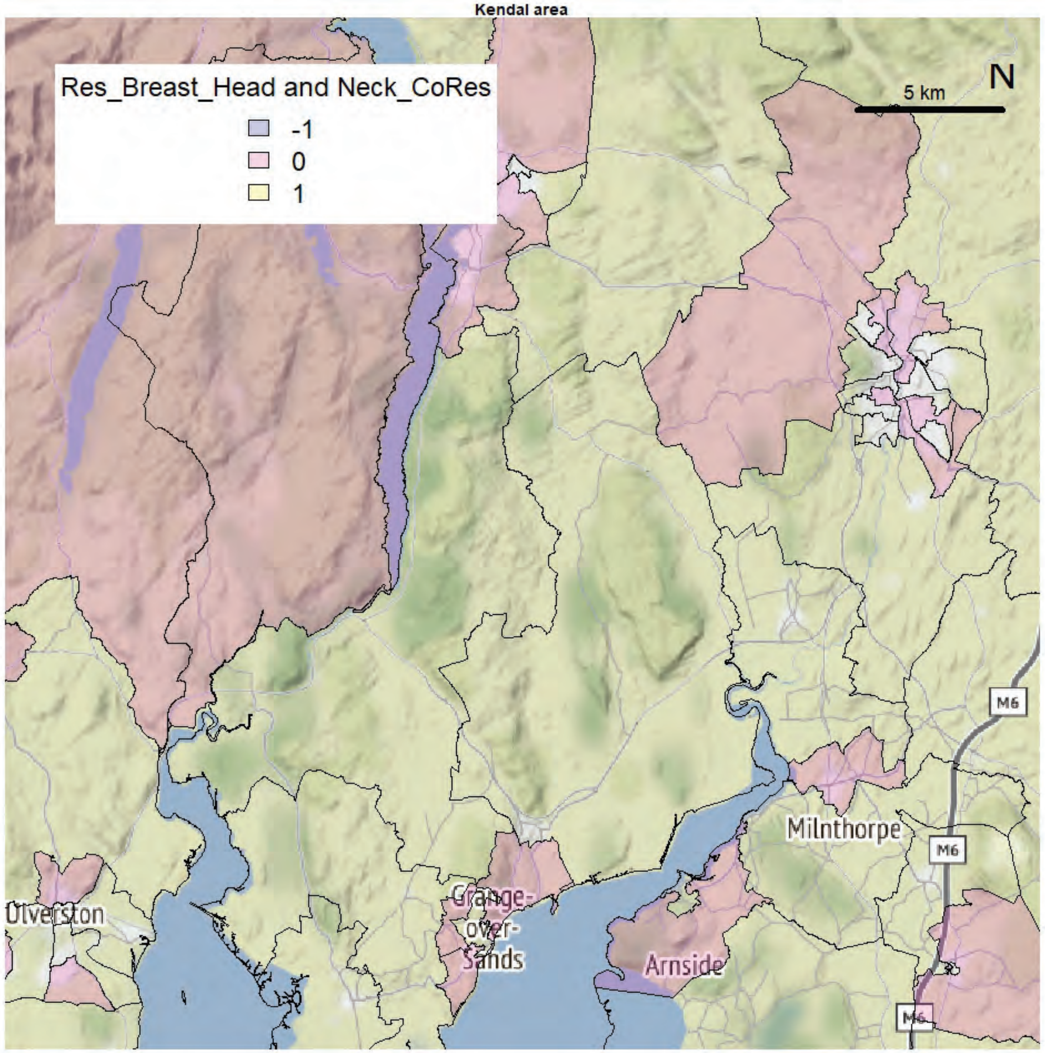
Kendal area
Res_Breast_Head and Neck_CoRes
-1
0
1
5 km
N
M6
Milnthorpe
Ulverston
Grange-over-Sands
Arnside

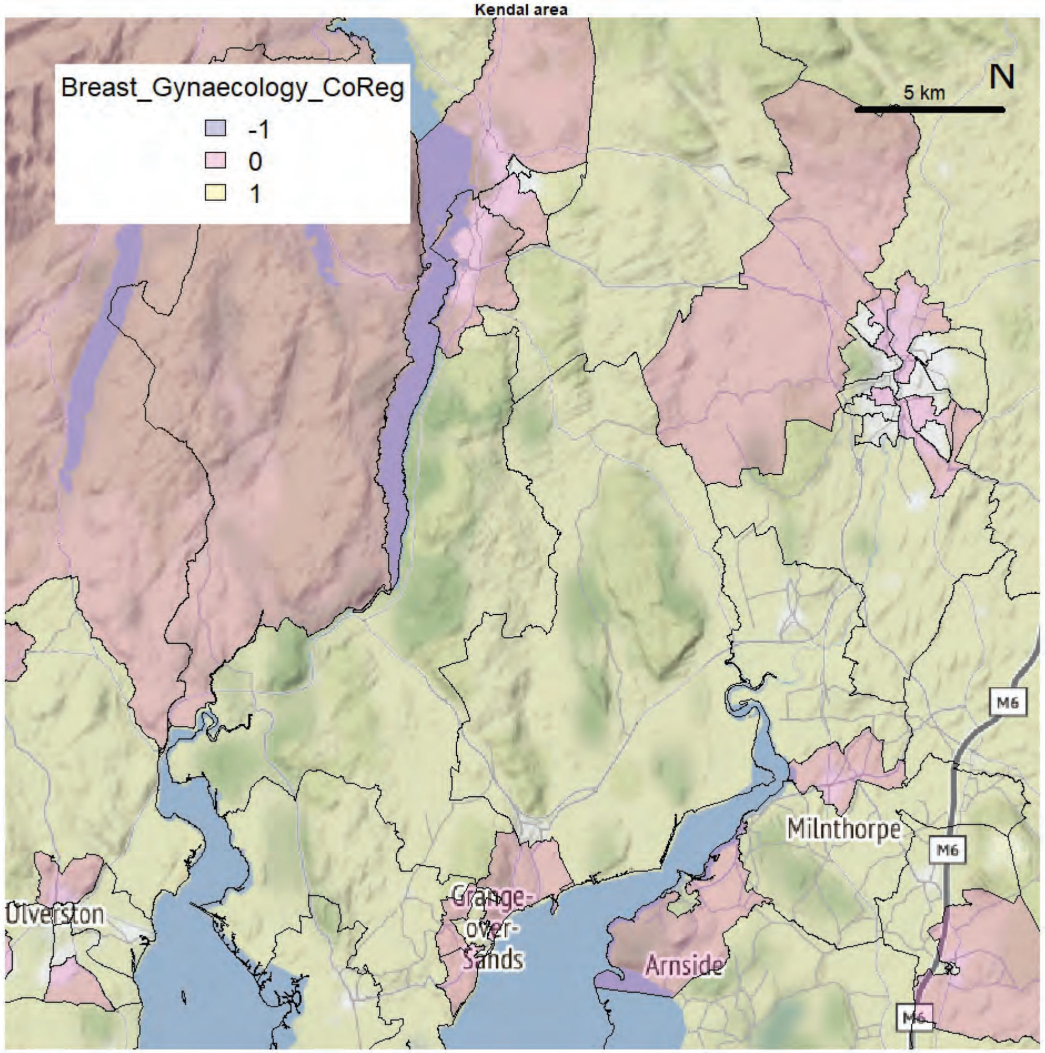

Kendal area
Breast_Gynaecology_CoReg
-1
0
1
5 km
N
M6
Milnthorpe
Ulverston
Grange-over-Sands
Arnside

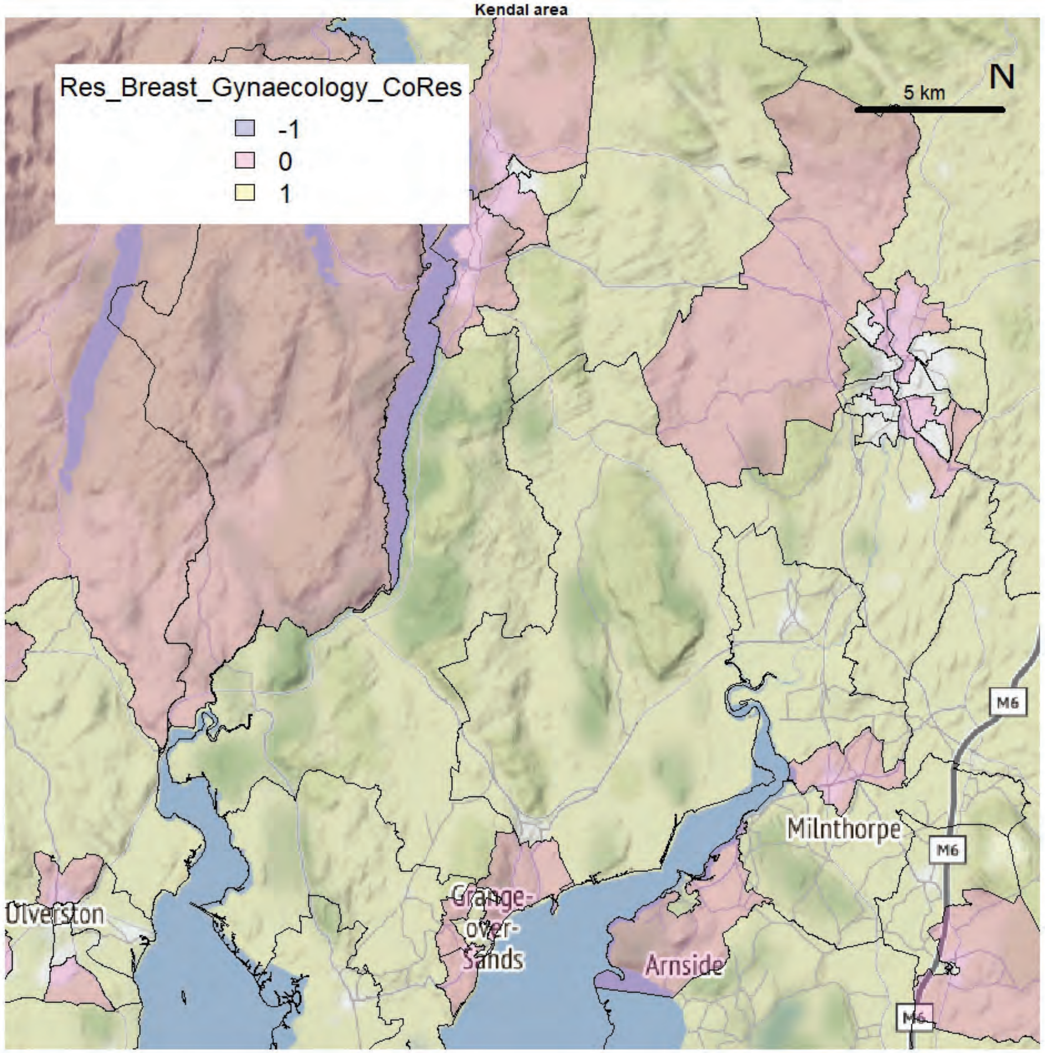

Kendal area
Res_Breast_Gynaecology_CoRes
-1
0
1
5 km
N
M6
Milnthorpe
Ulverston
Grange-over-Sands
Arnside

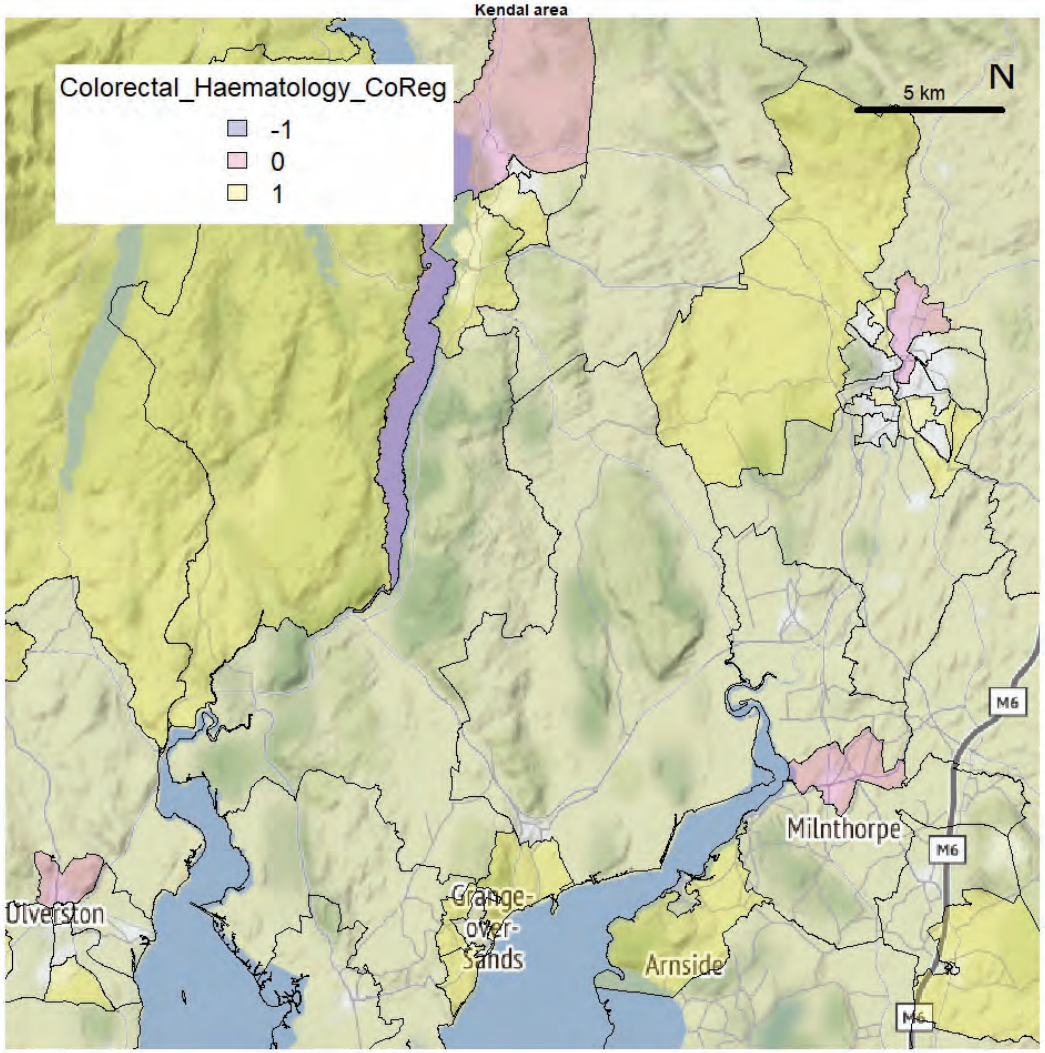

Kendal area
Colorectal_Haematology_CoReg
-1
0
1
5 km
N
Milnthorpe
Ulverston
Grange-over-Sands
Arnside
M6
M6
M6

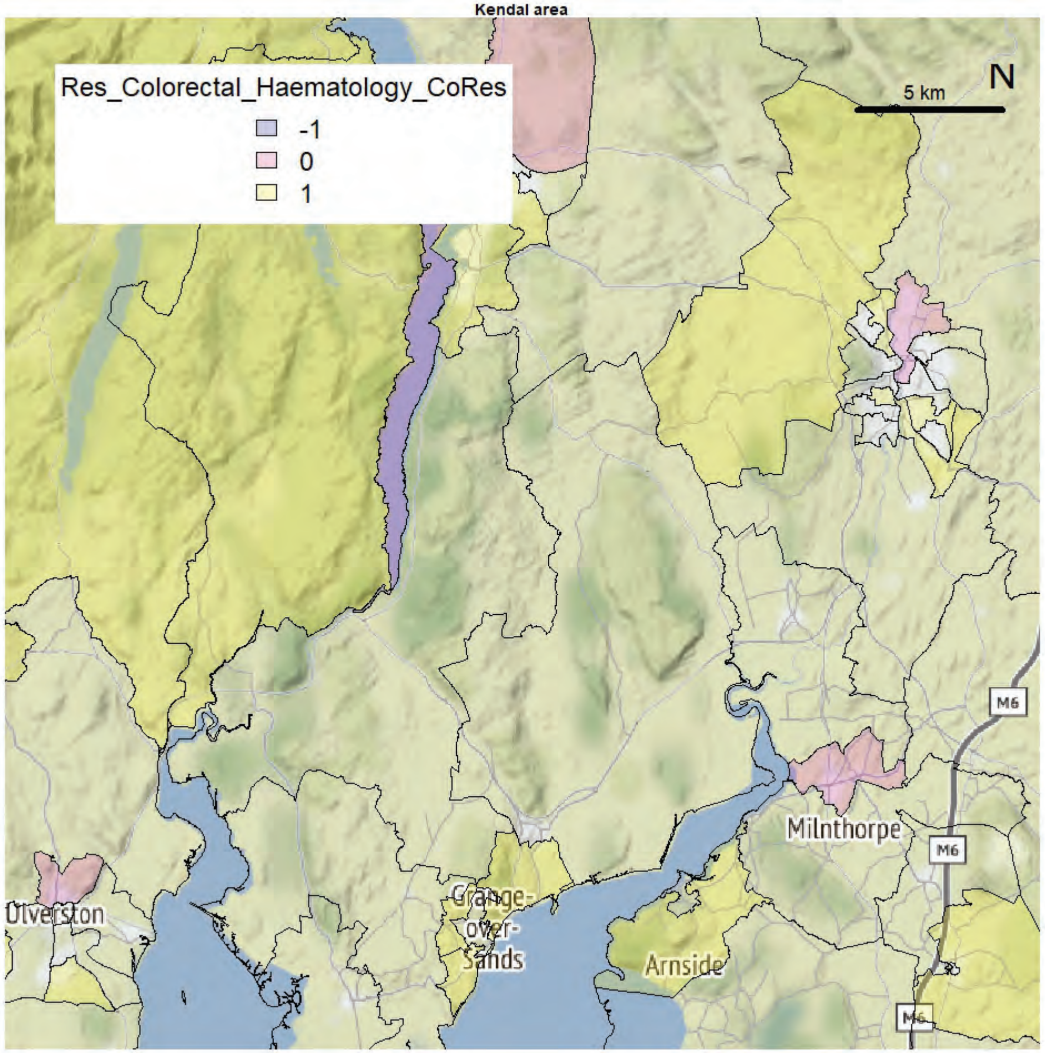

Kendal area
Res_Colorectal_Haematology_CoRes
-1
0
1
5 km
N
Milnthorpe
Ulverston
Grange-over-Sands
Arnside
M6
M6
M6

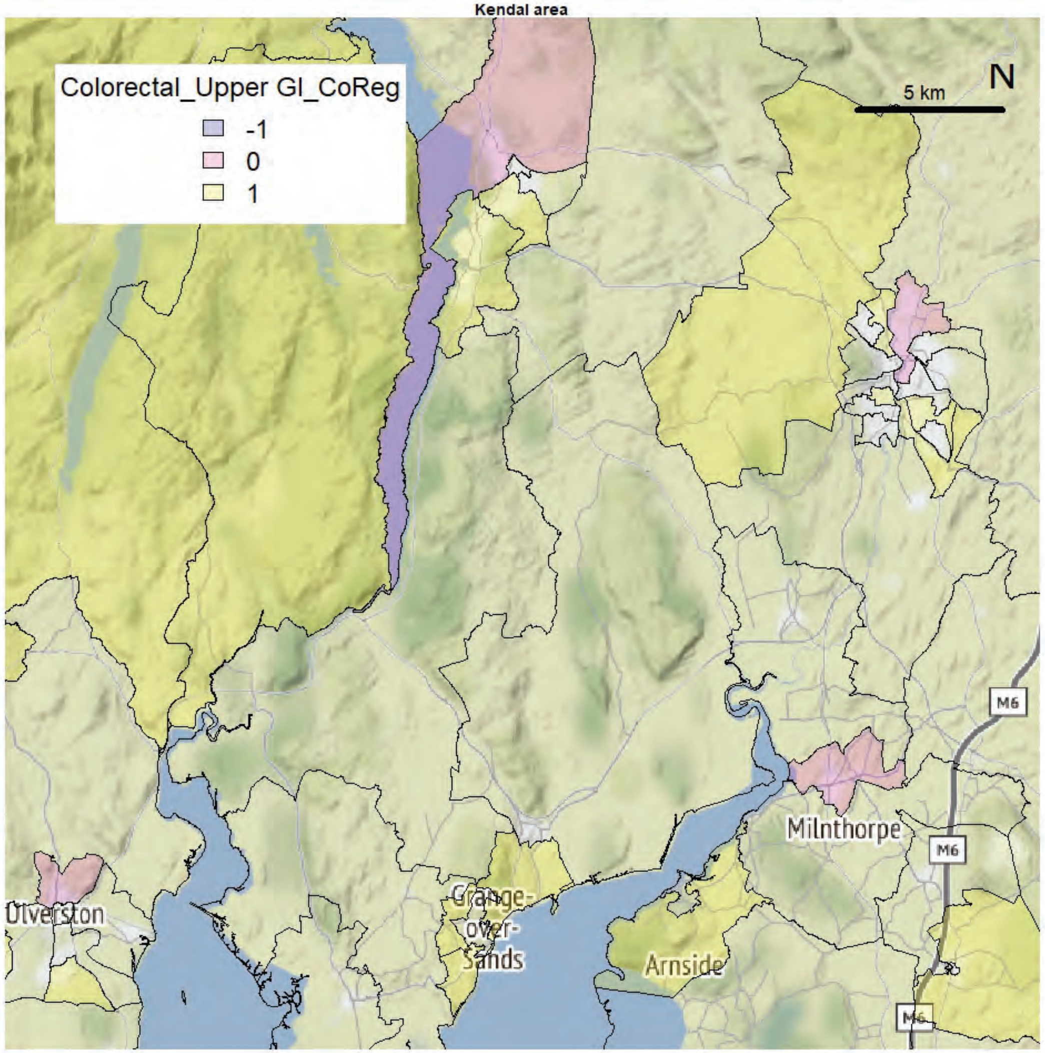
Kendal area
Colorectal_Upper GI_CoReg
-1
0
1
5 km
N
M6
Milnthorpe
Ulverston
Grange-
over-
Sands
Arnside

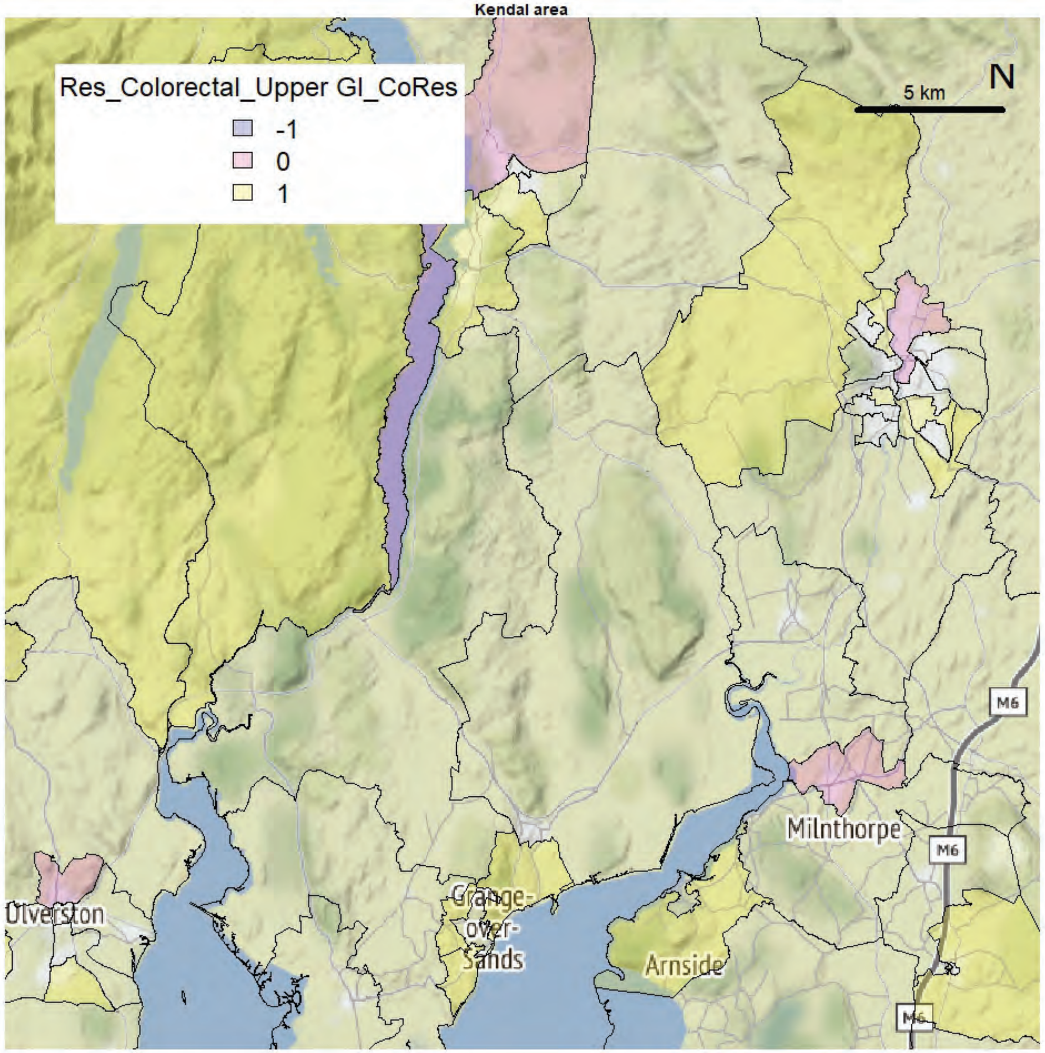
Kendal area
Res_Colorectal_Upper GI_CoRes
-1
0
1
5 km
N
M6
Milnthorpe
Ulverston
Grange-
over-
Sands
Arnside

Kendal area

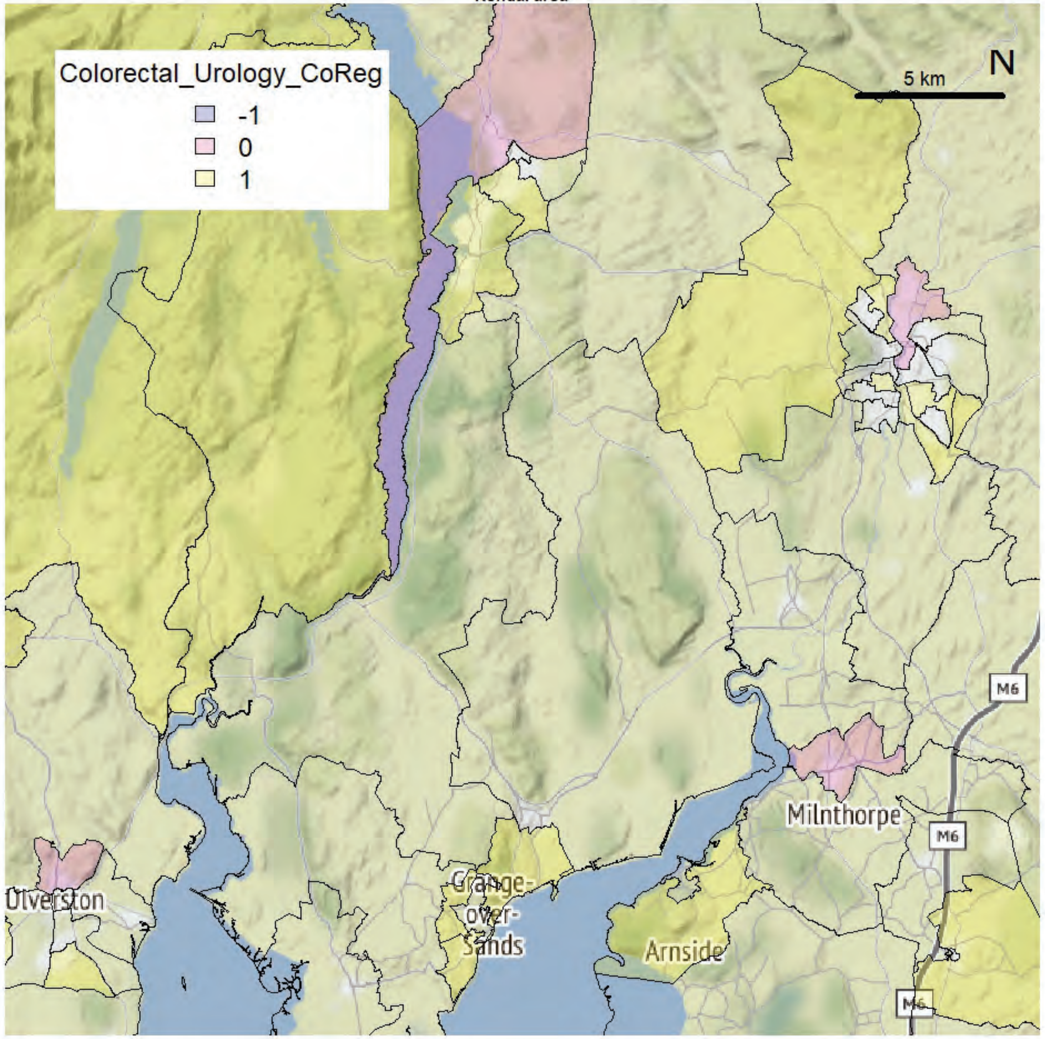

Kendal area

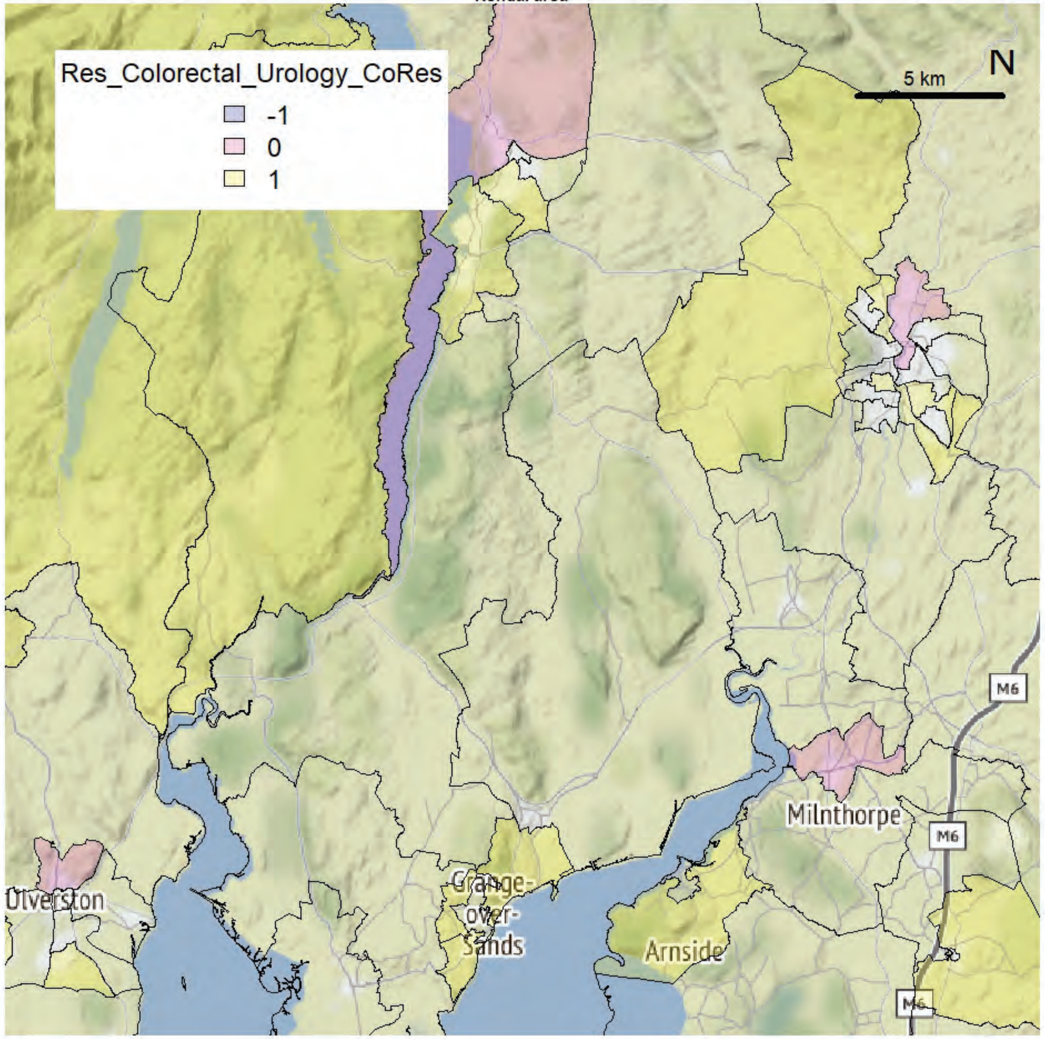

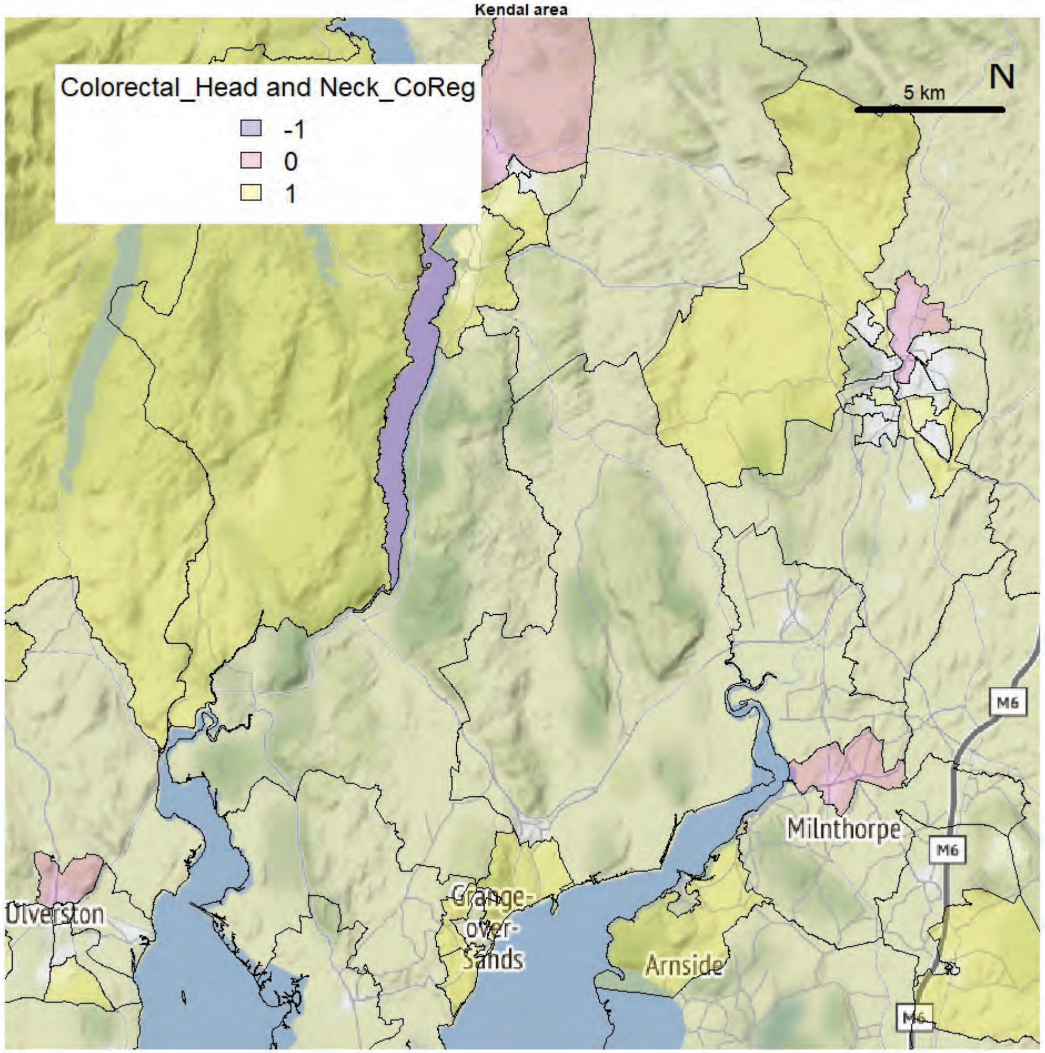
Kendal area
Colorectal_Head and Neck_CoReg
-1
0
1
5 km
N
M6
Milnthorpe
Ulverston
Grange-over-Sands
Arnside

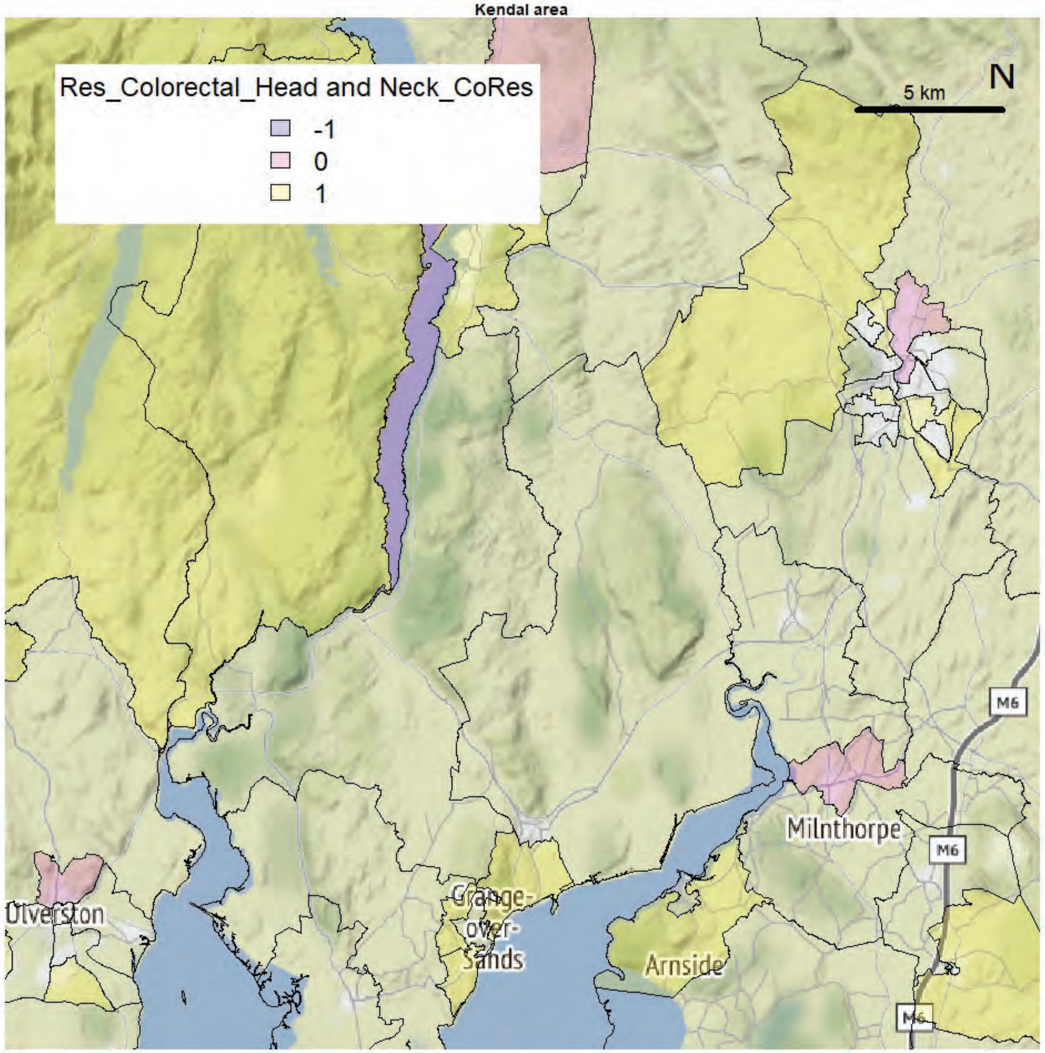
Kendal area
Res_Colorectal_Head and Neck_CoRes
-1
0
1
5 km
N
M6
Milnthorpe
Ulverston
Grange-over-Sands
Arnside

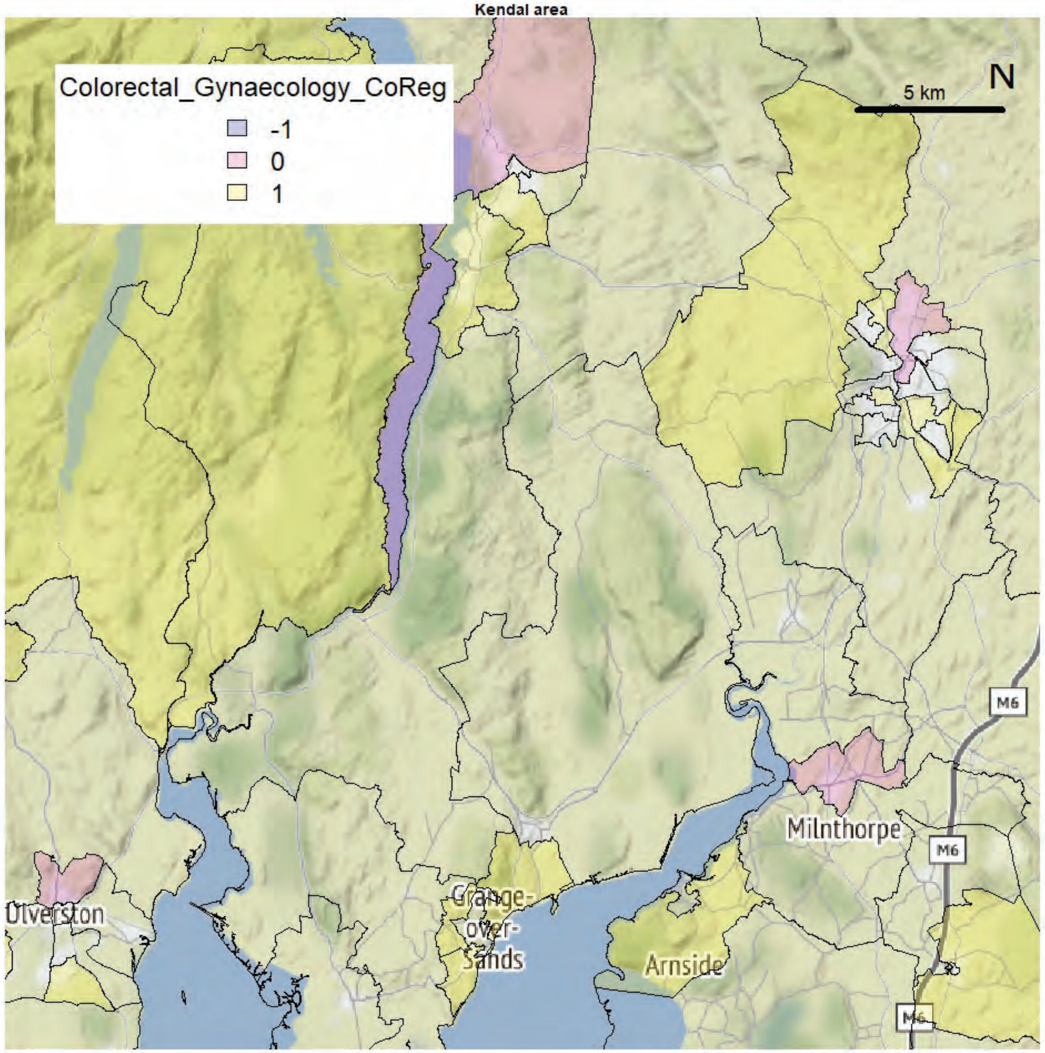
Kendal area
Colorectal_Gynaecology_CoReg
-1
0
1
5 km
N
M6
Milnthorpe
Ulverston
Grange-over-Sands
Arnside

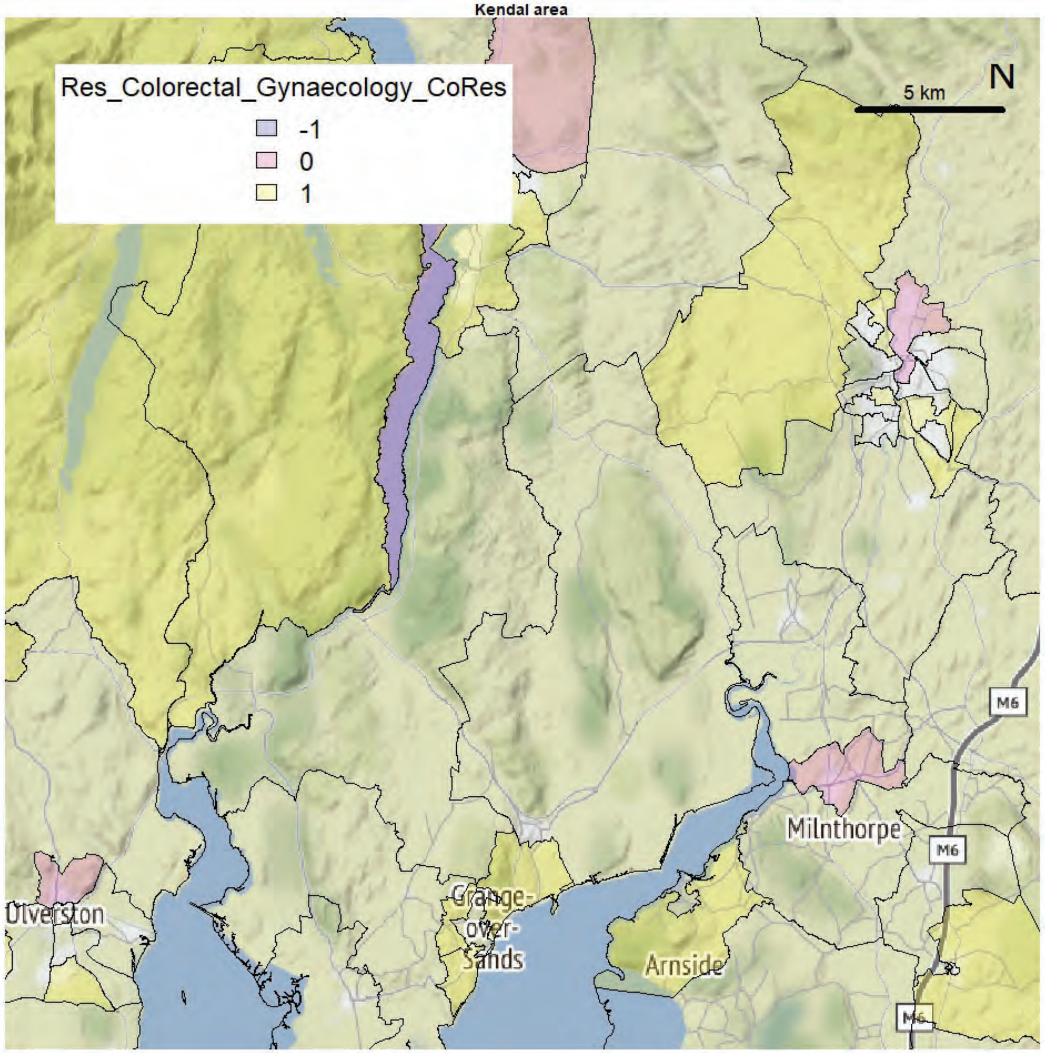
Kendal area
Res_Colorectal_Gynaecology_CoRes
-1
0
1
5 km
N
M6
Milnthorpe
Ulverston
Grange-over-Sands
Arnside

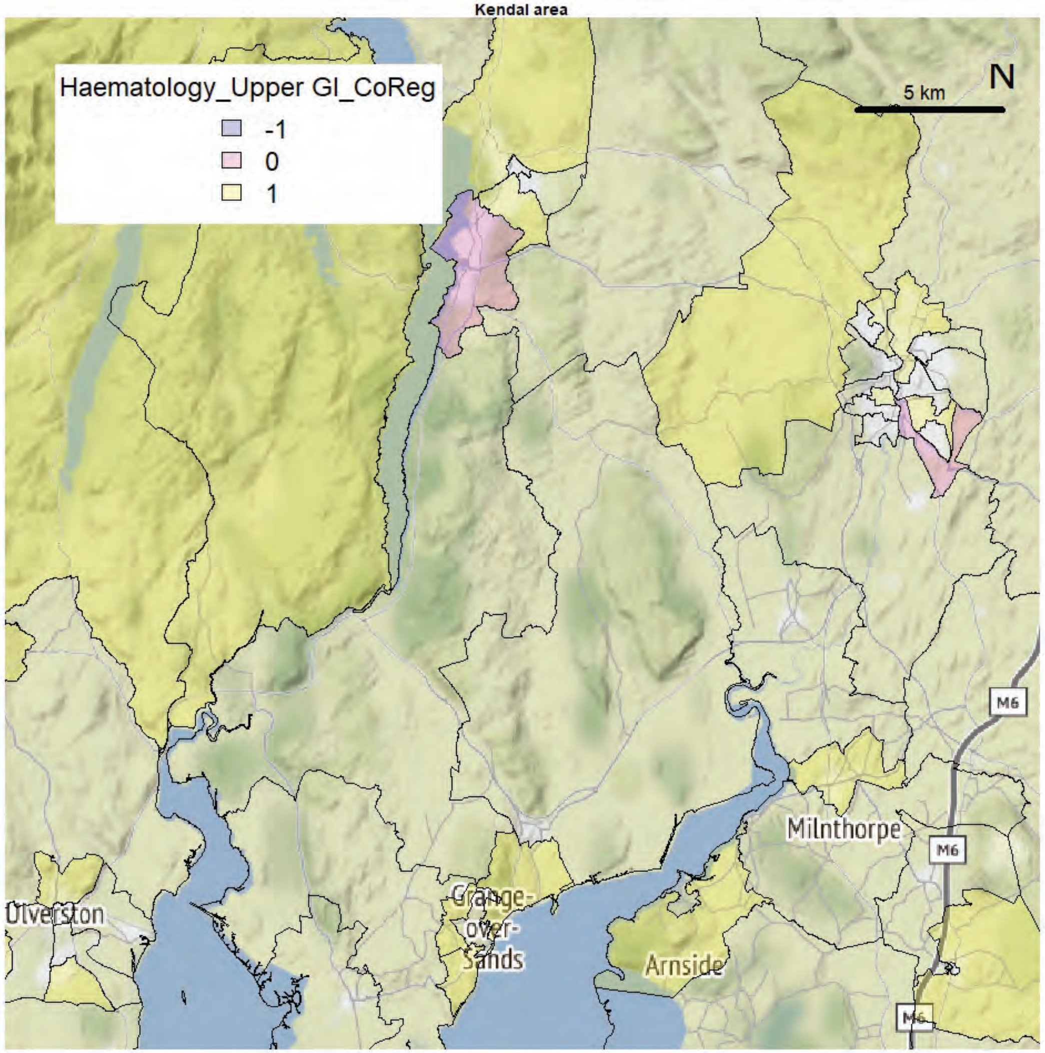
Kendal area
Haematology_Upper GI_CoReg
-1
0
1
5 km
N
M6
Milnthorpe
M6
Ulverston
Grange-over-Sands
Arnside
M6

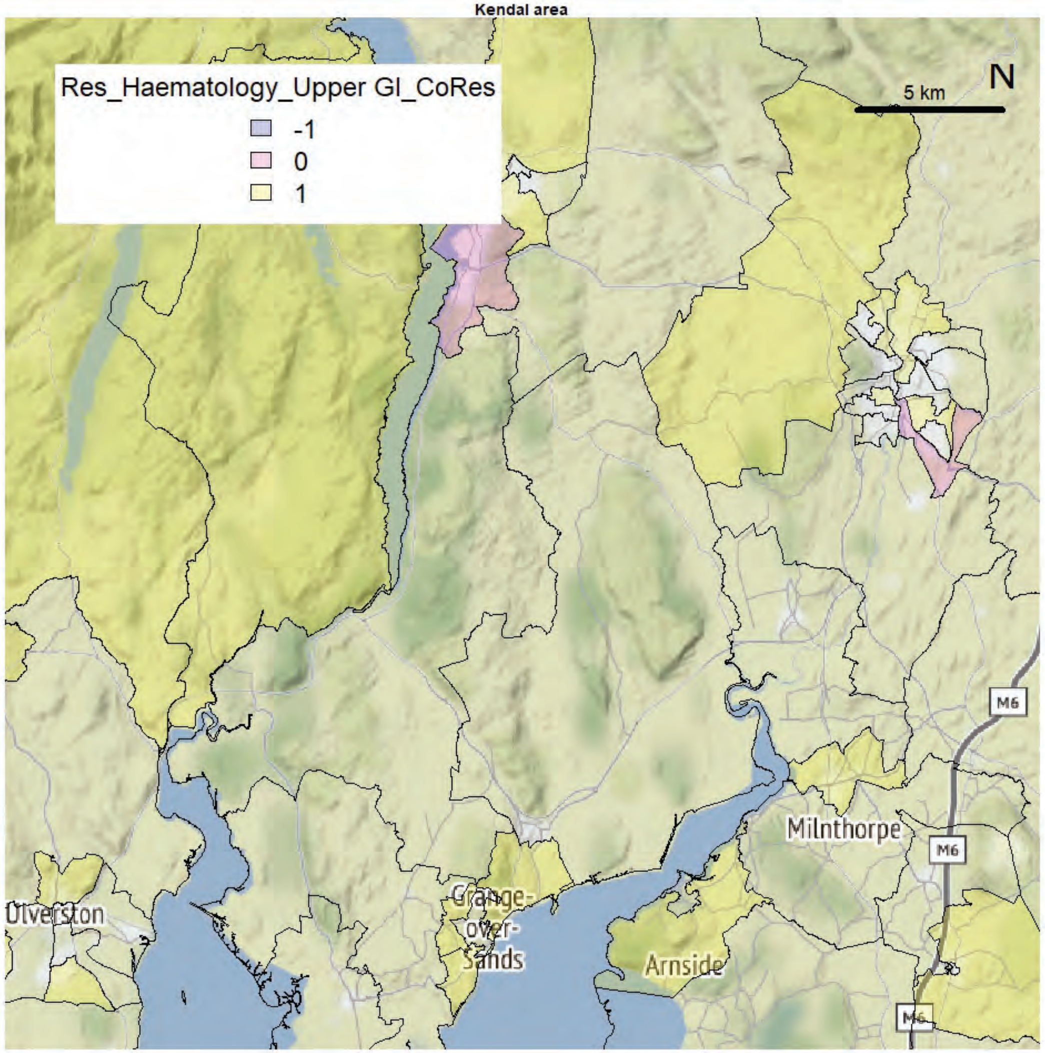
Kendal area
Res_Haematology_Upper GI_CoRes
-1
0
1
5 km
N
M6
Milnthorpe
M6
Ulverston
Grange-over-Sands
Arnside
M6

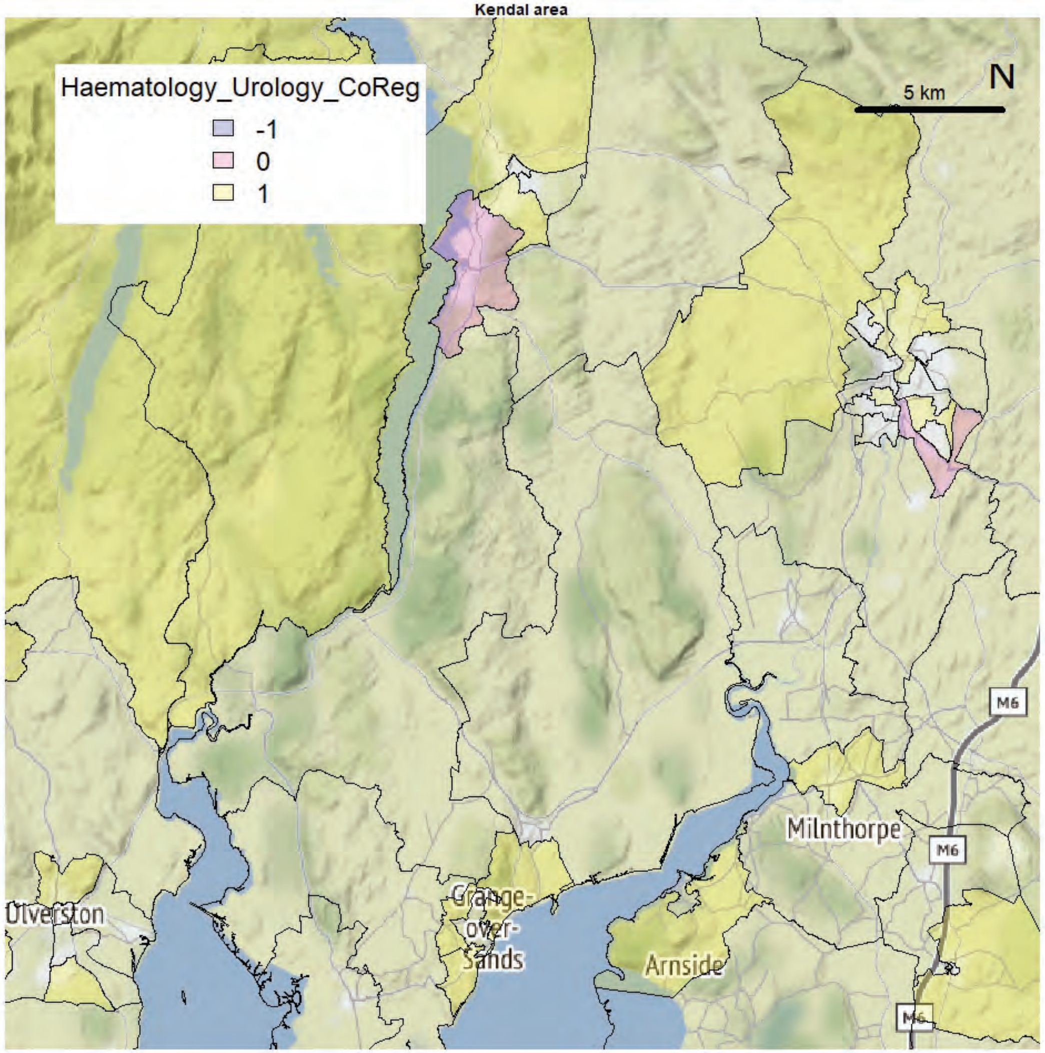
Kendal area
Haematology_Urology_CoReg
-1
0
1
5 km
N
M6
Milnthorpe
M6
Ulverston
Grange-over-Sands
Arnside
M6

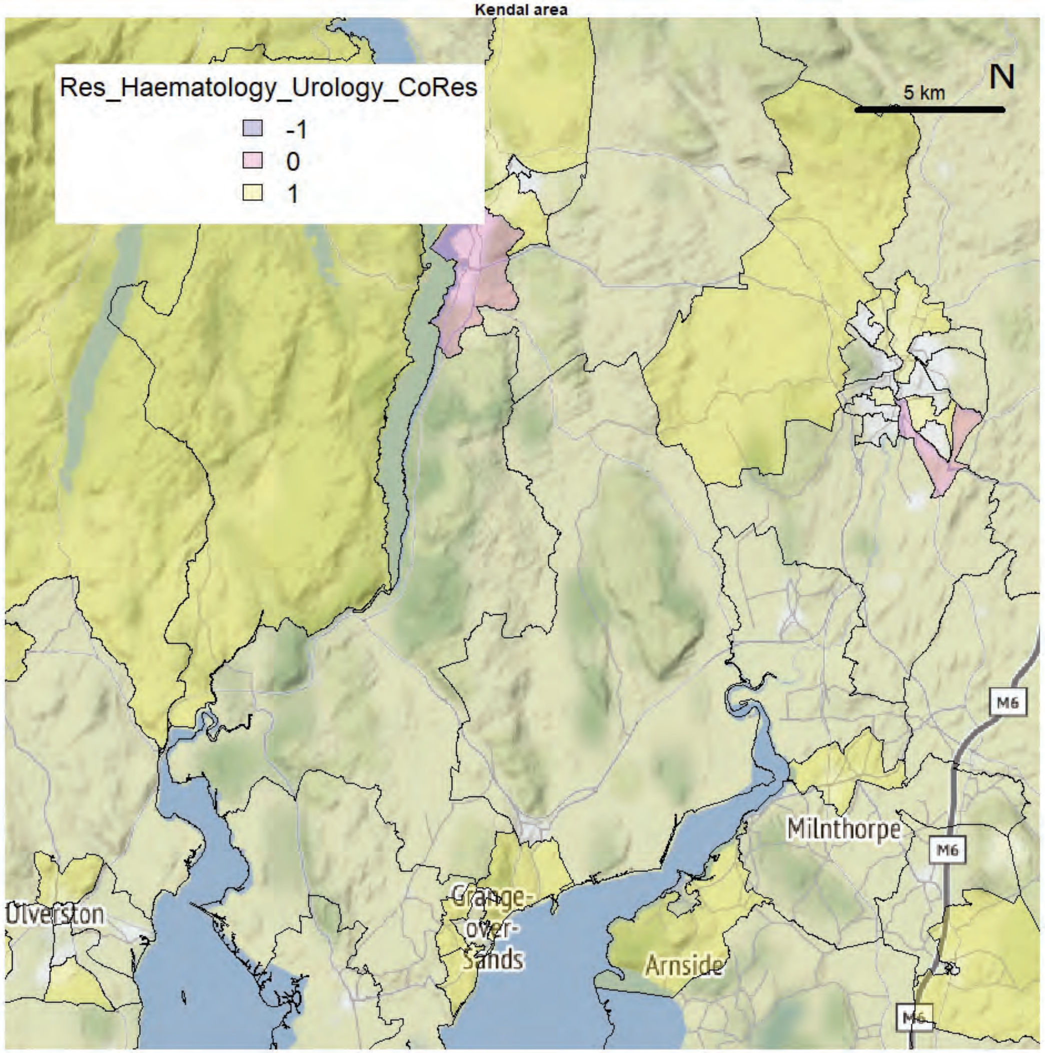
Kendal area
Res_Haematology_Urology_CoRes
-1
0
1
5 km
N
M6
Milnthorpe
M6
Ulverston
Grange-over-Sands
Arnside
M6

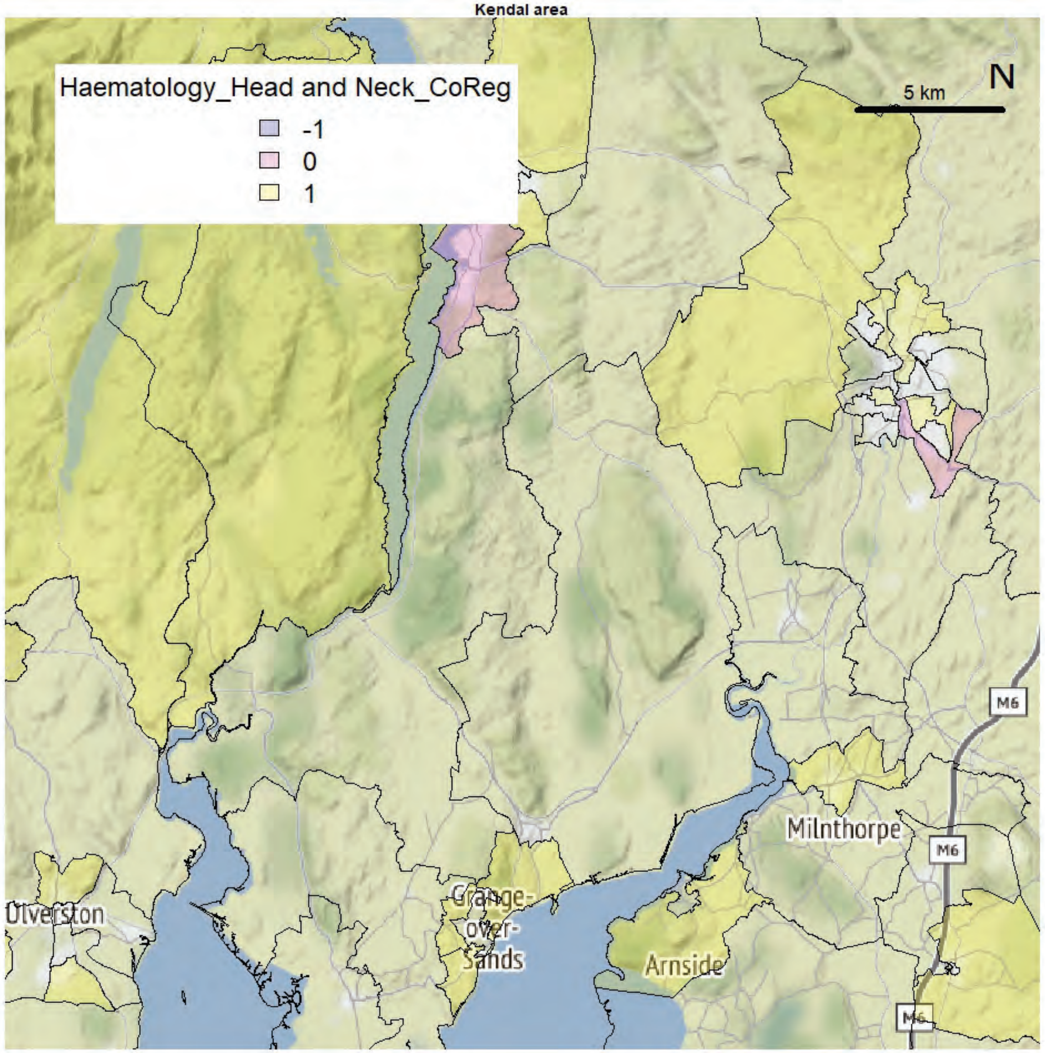
Kendal area
Haematology_Head and Neck_CoReg
-1
0
1
5 km
N
M6
Milnthorpe
Ulverston
Grange-over-Sands
Arnside

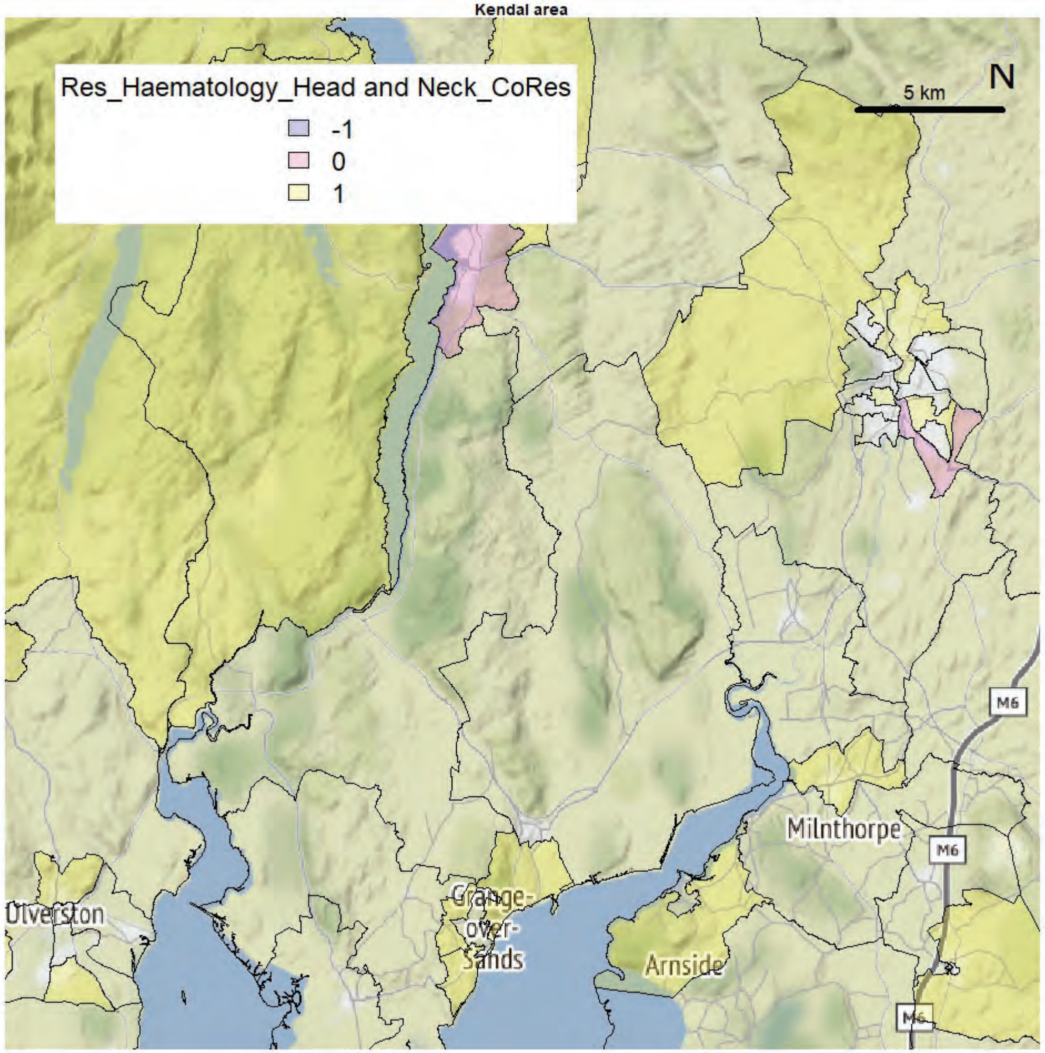
Kendal area
Res_Haematology_Head and Neck_CoRes
-1
0
1
5 km
N
M6
Milnthorpe
Ulverston
Grange-over-Sands
Arnside

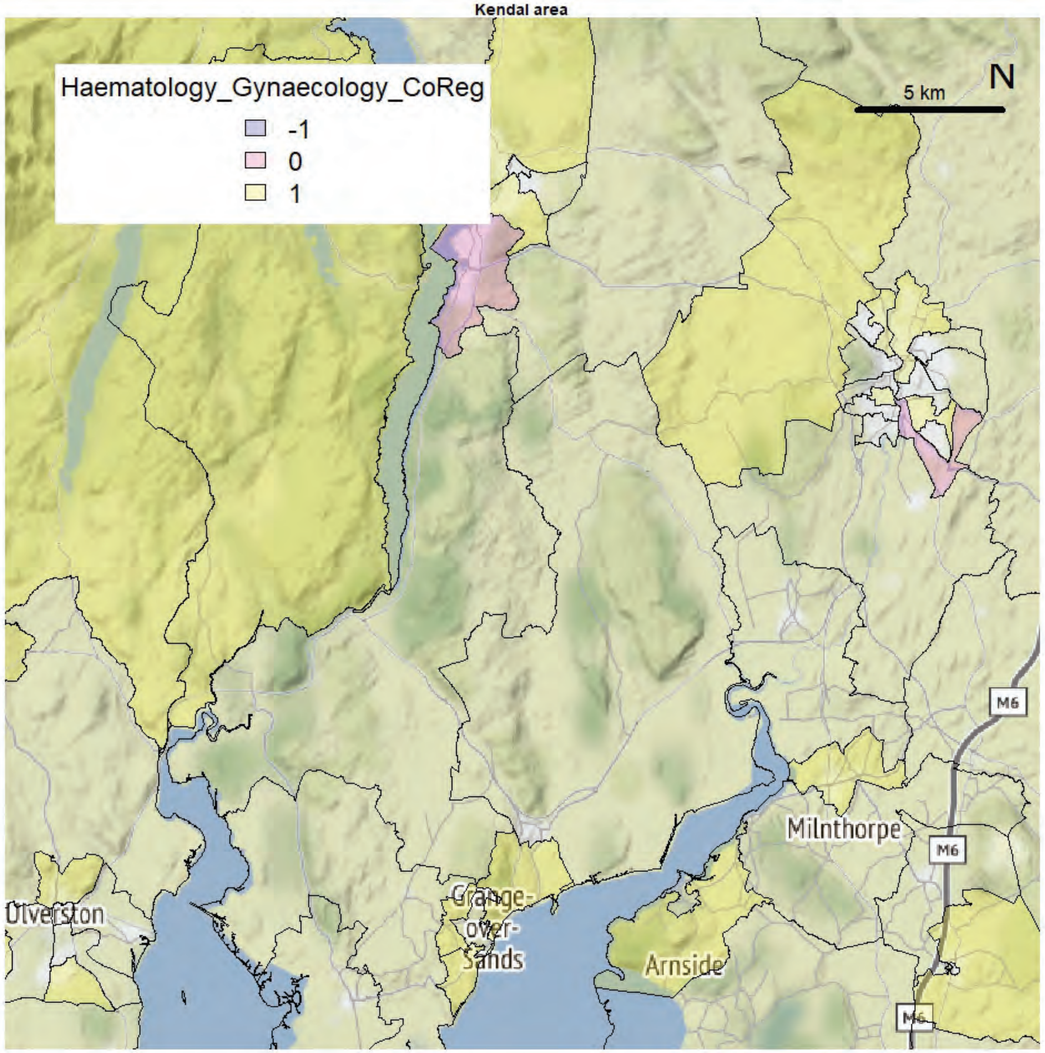
Kendal area
Haematology_Gynaecology_CoReg
-1
0
1
5 km
N
M6
Milnthorpe
Ulverston
Grange-over-Sands
Arnside

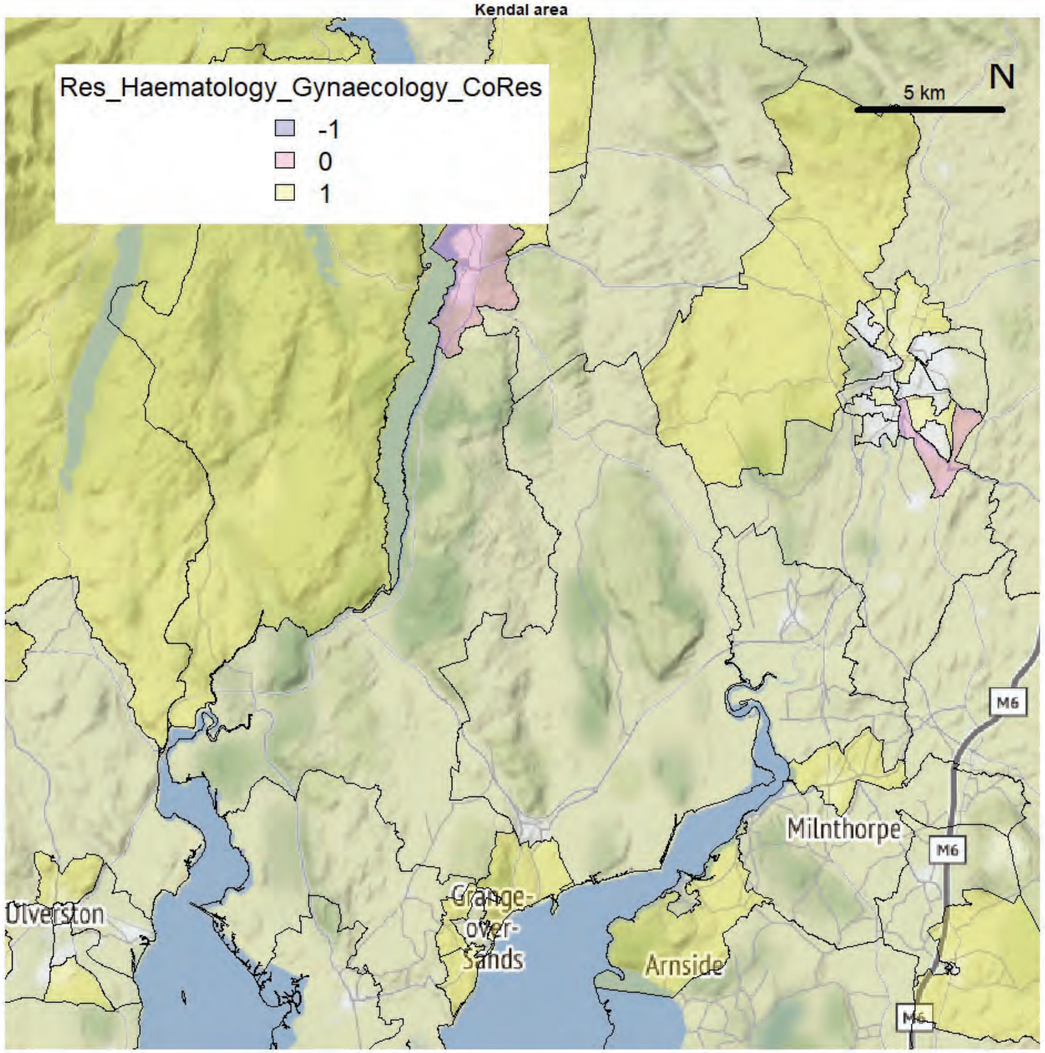
Kendal area
Res_Haematology_Gynaecology_CoRes
-1
0
1
5 km
N
M6
Milnthorpe
Ulverston
Grange-over-Sands
Arnside

Kendal area

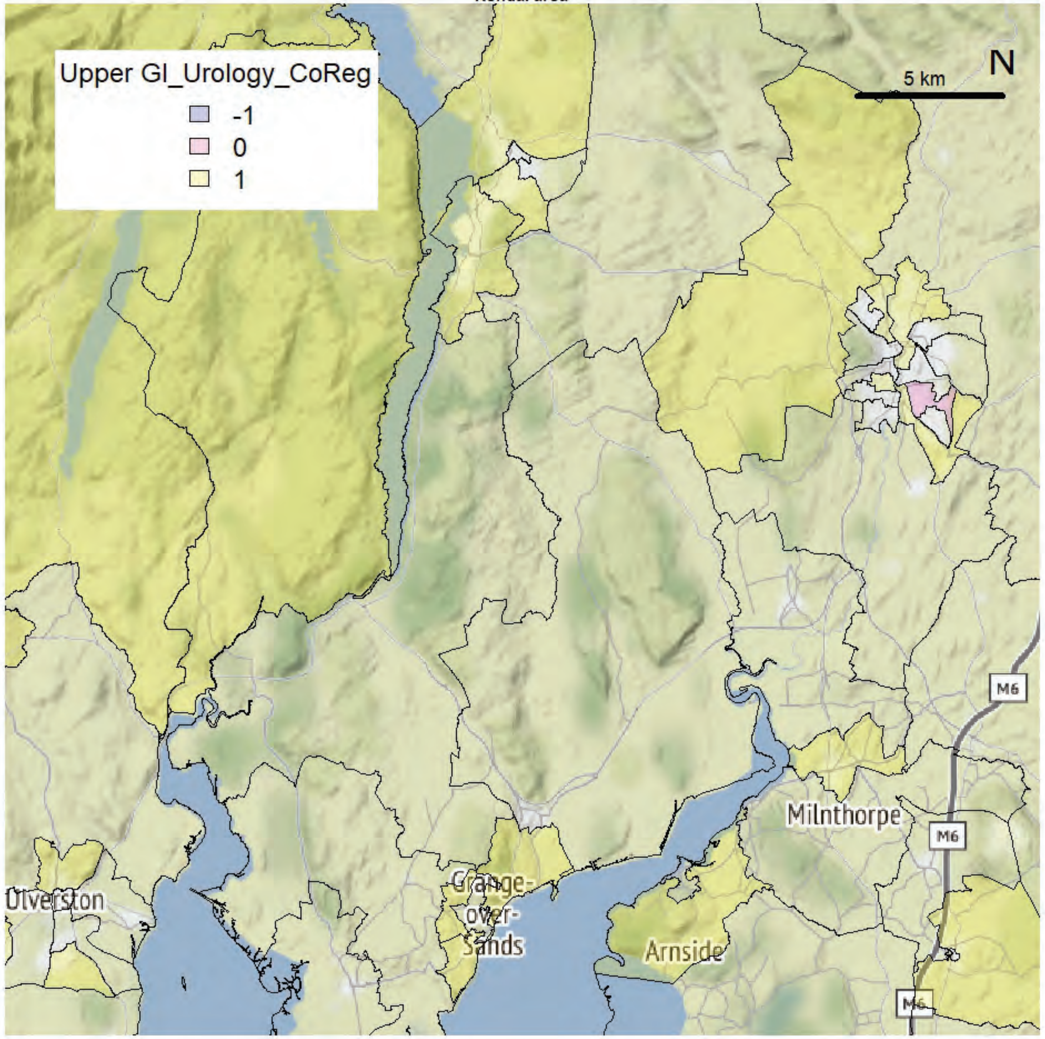

Kendal area

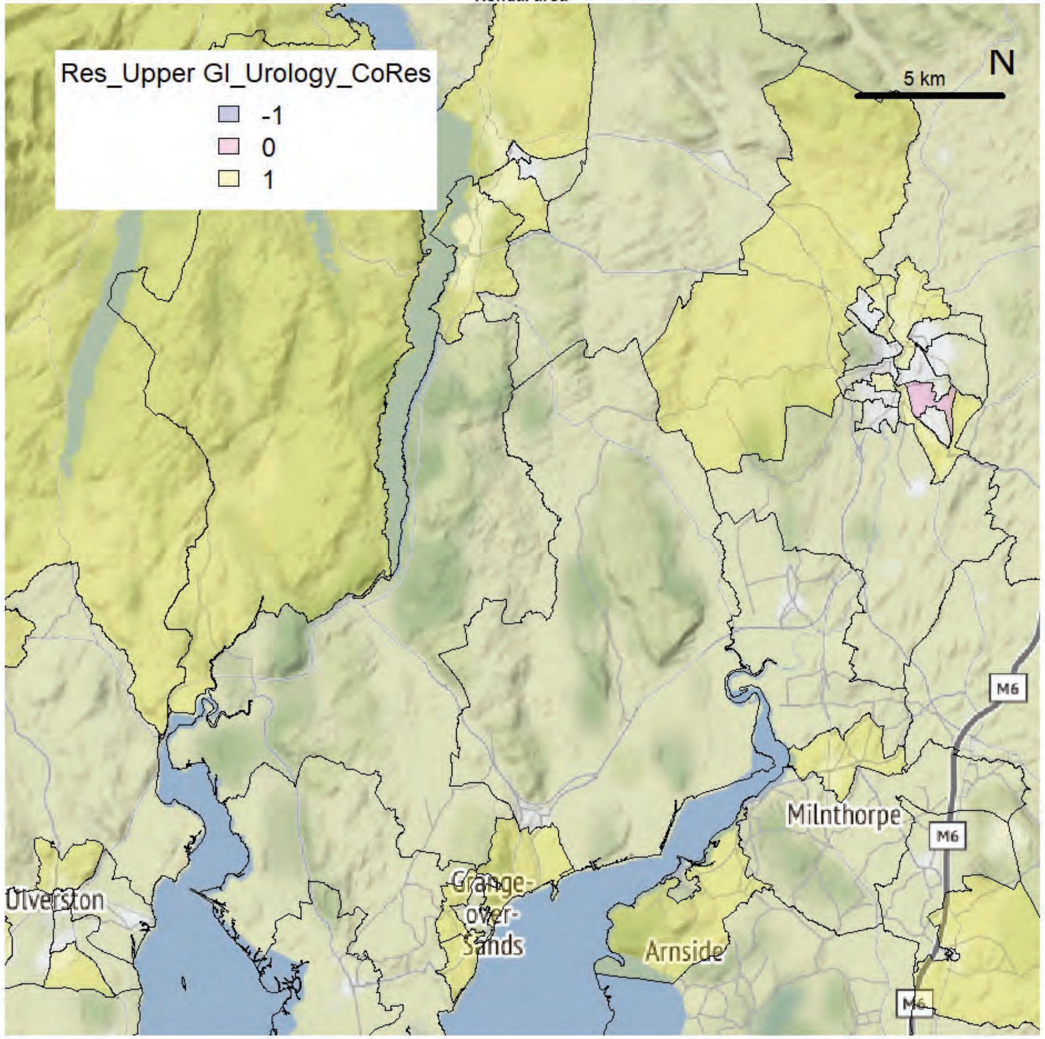

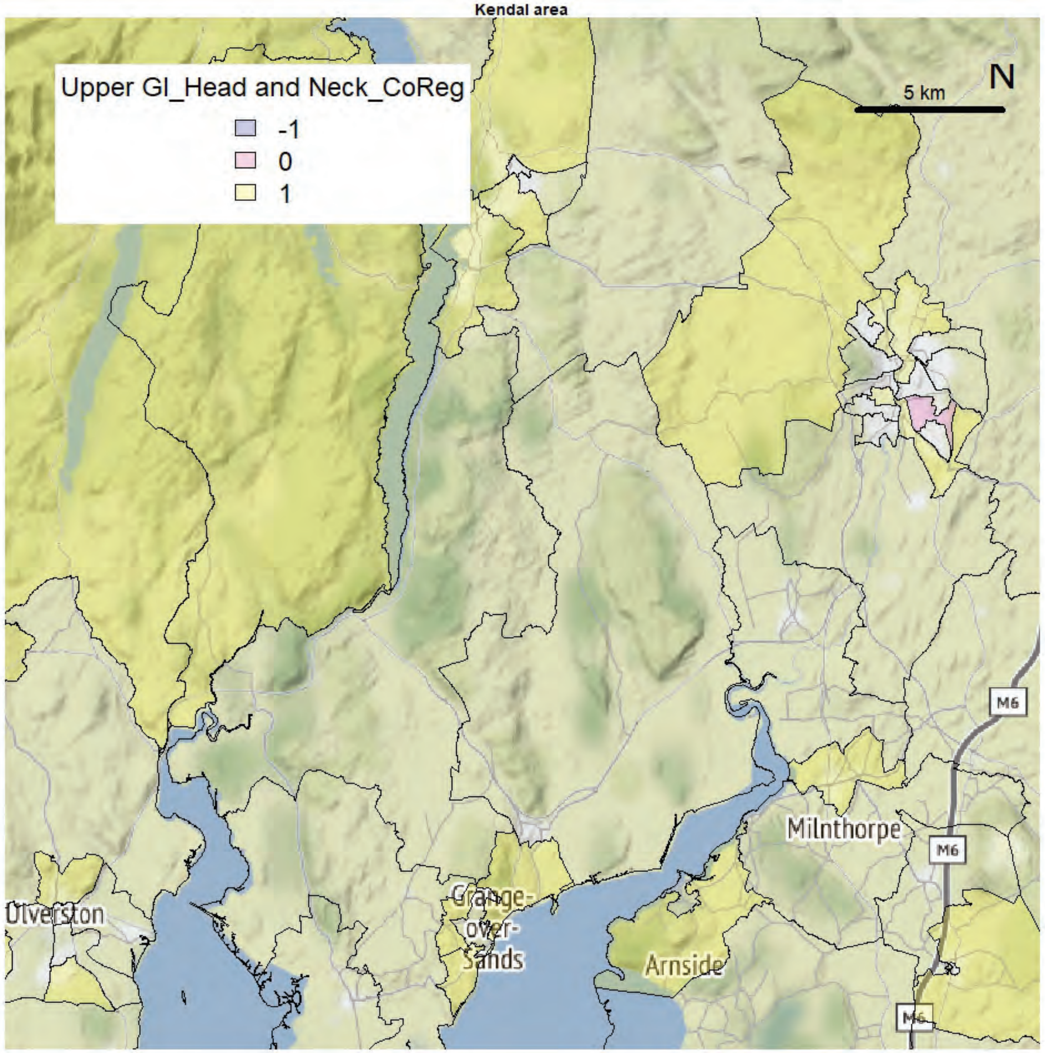
Kendal area
Upper GI_Head and Neck_CoReg
-1
0
1
5 km
N
M6
Milnthorpe
Ulverston
Grange-
over-
Sands
Arnside

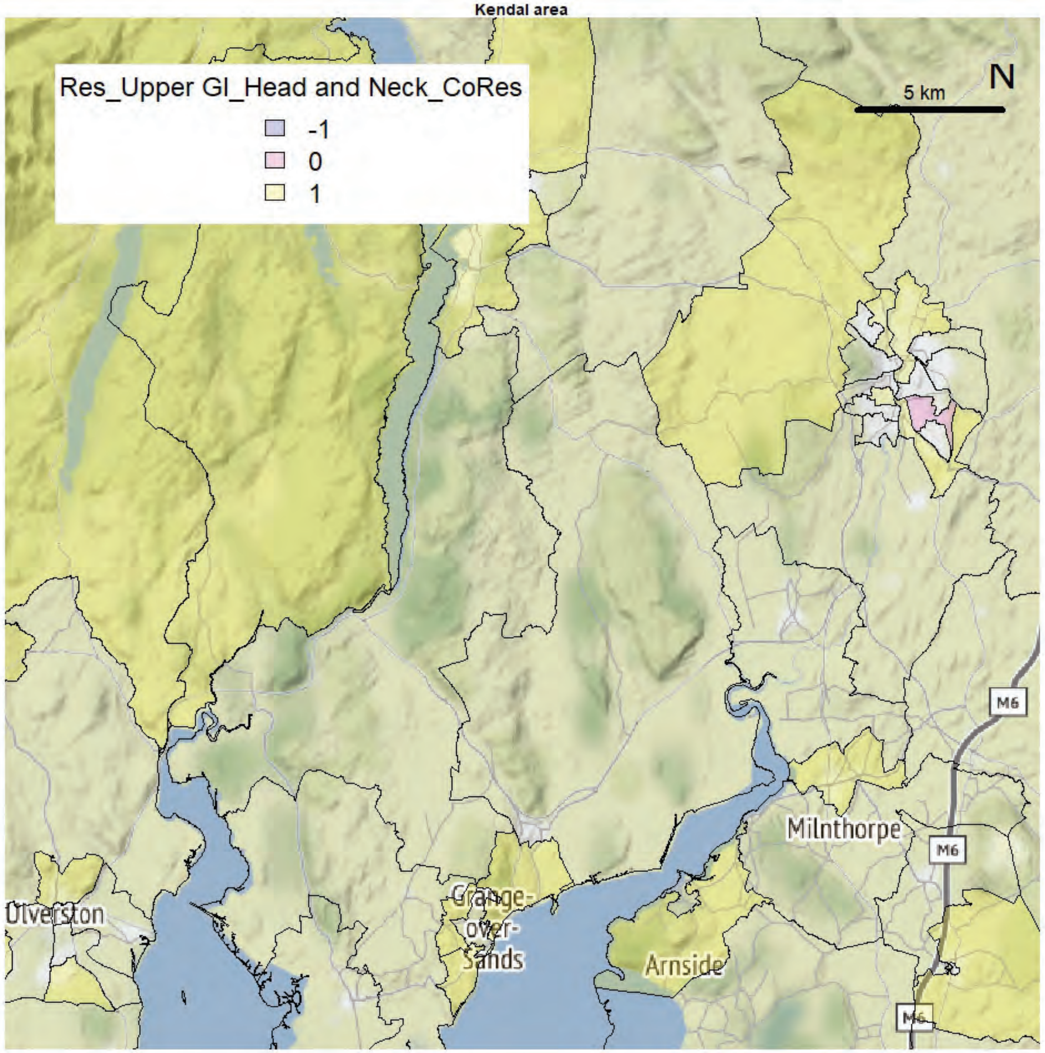
Kendal area
Res_Upper GI_Head and Neck_CoRes
-1
0
1
5 km
N
M6
Milnthorpe
Ulverston
Grange-
over-
Sands
Arnside

Kendal area

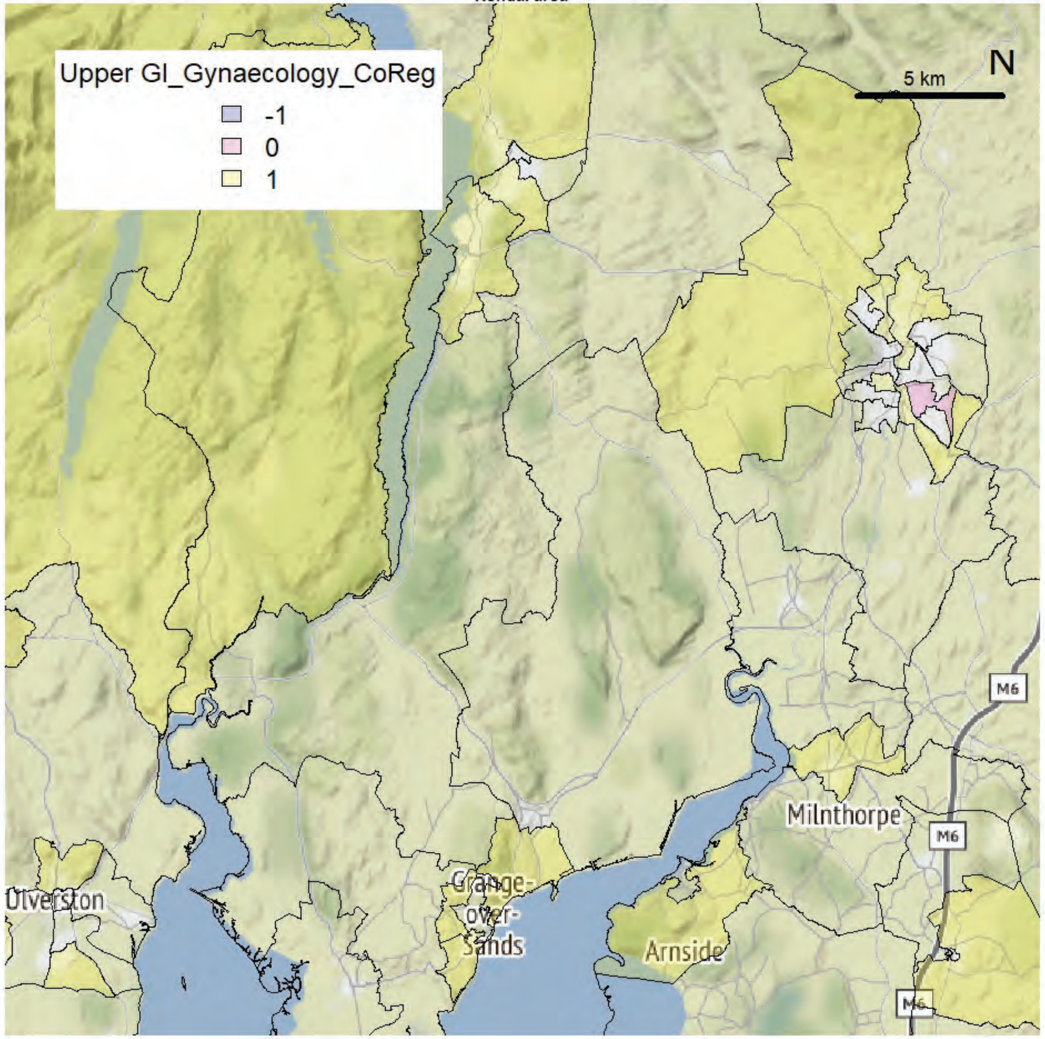

Kendal area

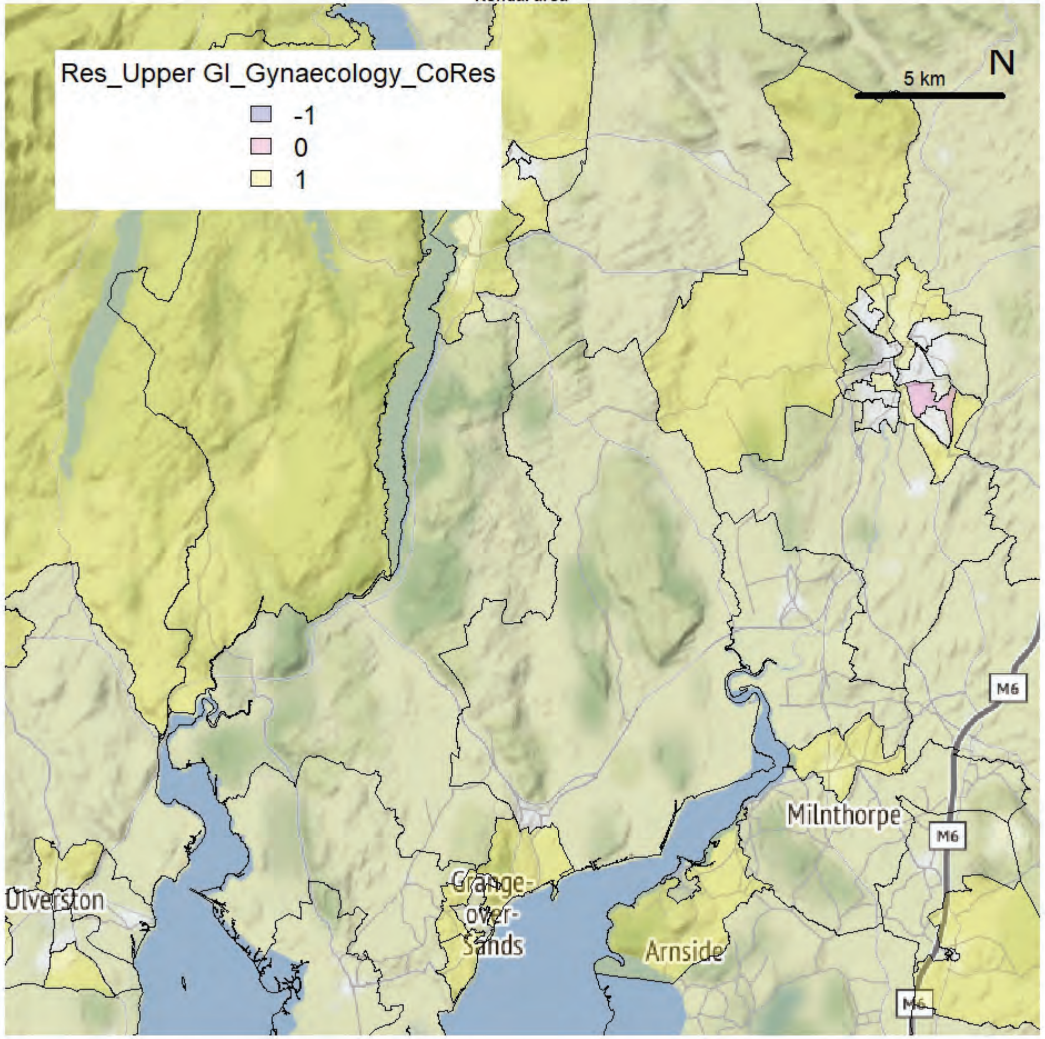

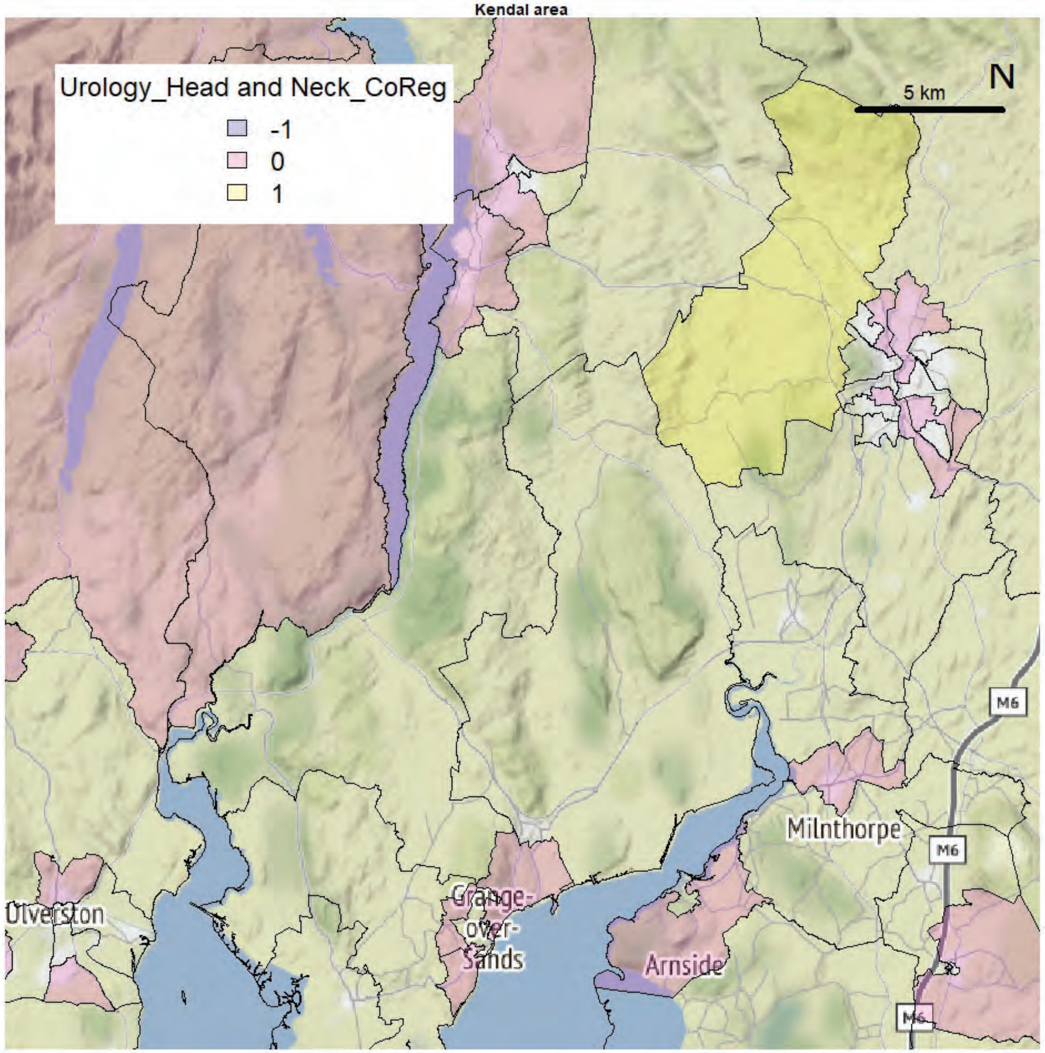
Kendal area
Urology_Head and Neck_CoReg
-1
0
1
5 km
N
M6
Milnthorpe
Ulverston
Grange-over-Sands
Arnside

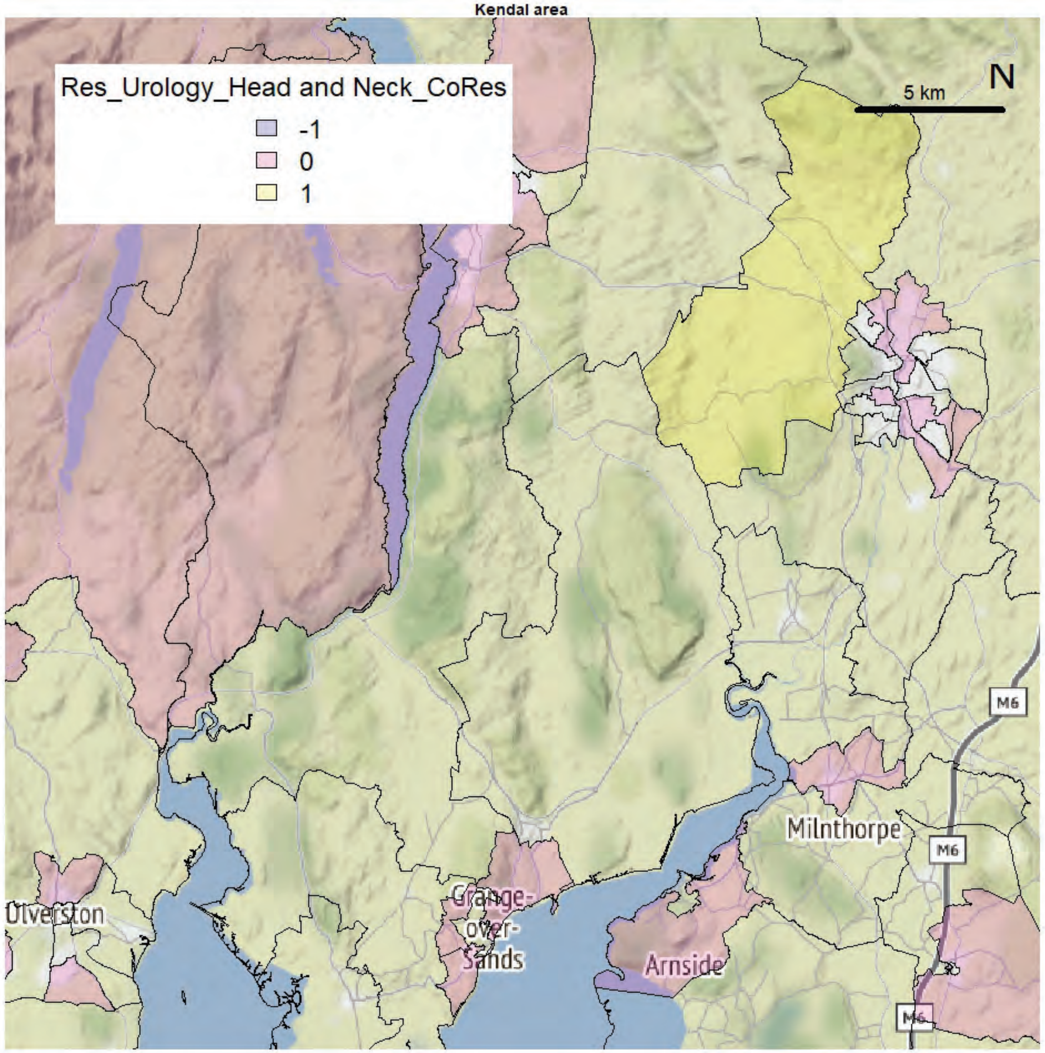
Kendal area
Res_Urology_Head and Neck_CoRes
-1
0
1
5 km
N
M6
Milnthorpe
Ulverston
Grange-over-Sands
Arnside

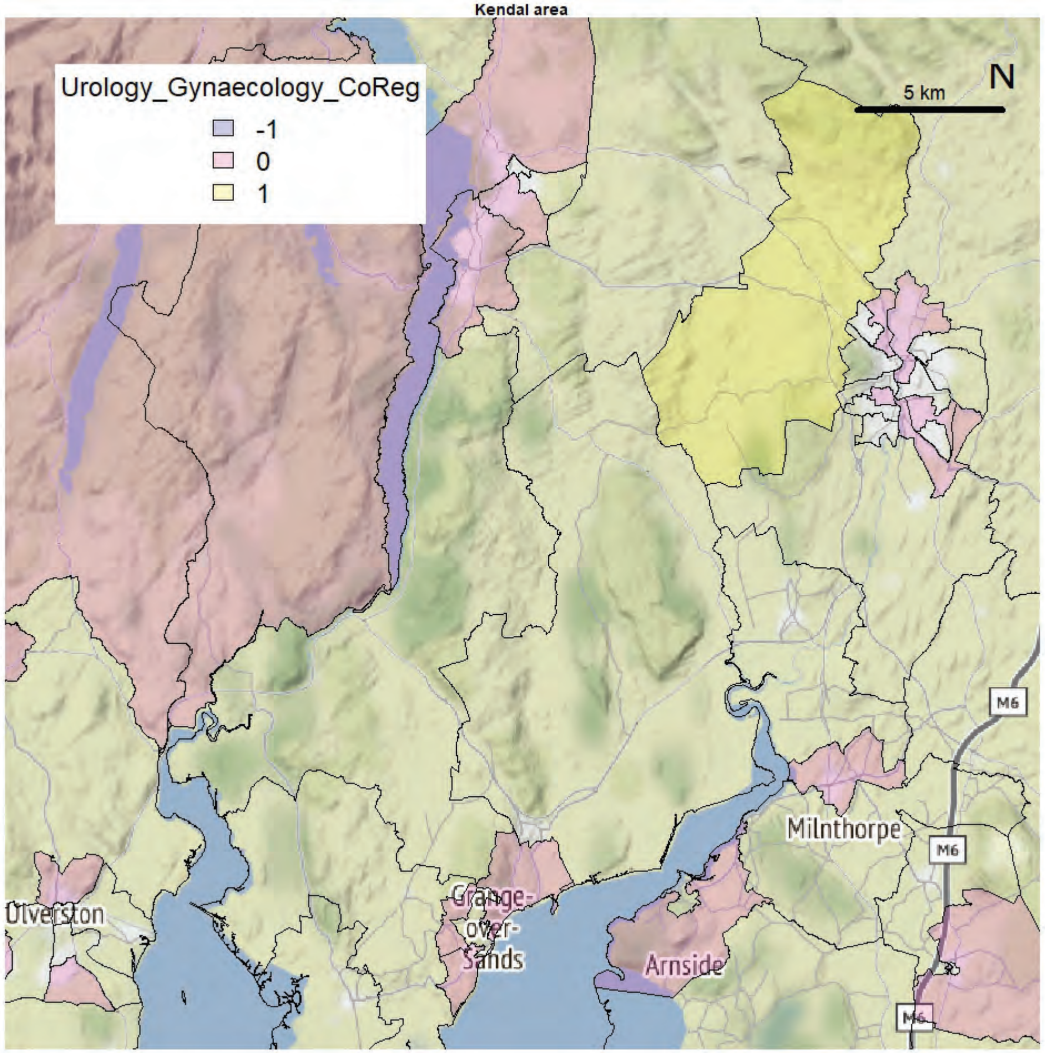
Kendal area
Urology_Gynaecology_CoReg
-1
0
1
5 km
N
M6
Milnthorpe
Ulverston
Grange-over-Sands
Arnside

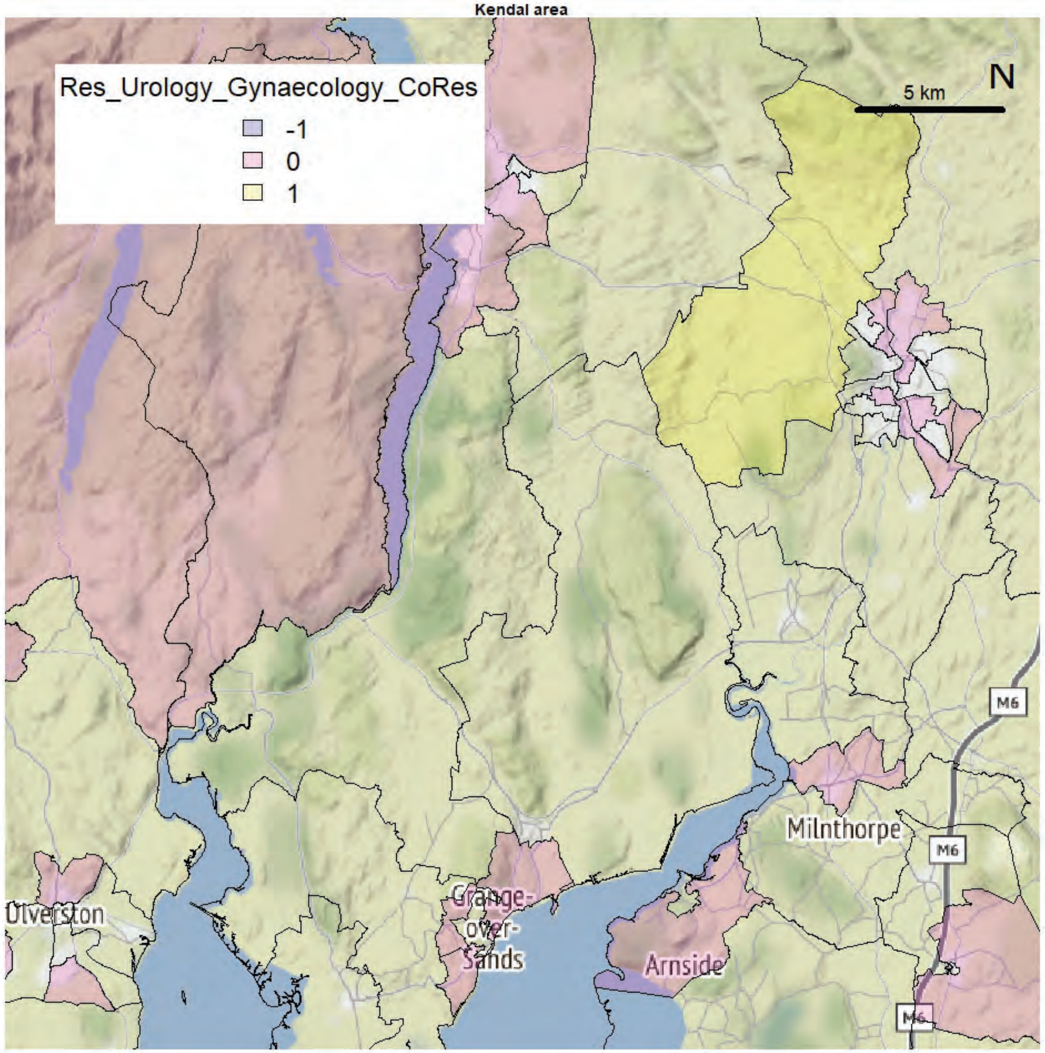
Kendal area
Res_Urology_Gynaecology_CoRes
-1
0
1
5 km
N
M6
Milnthorpe
Ulverston
Grange-over-Sands
Arnside

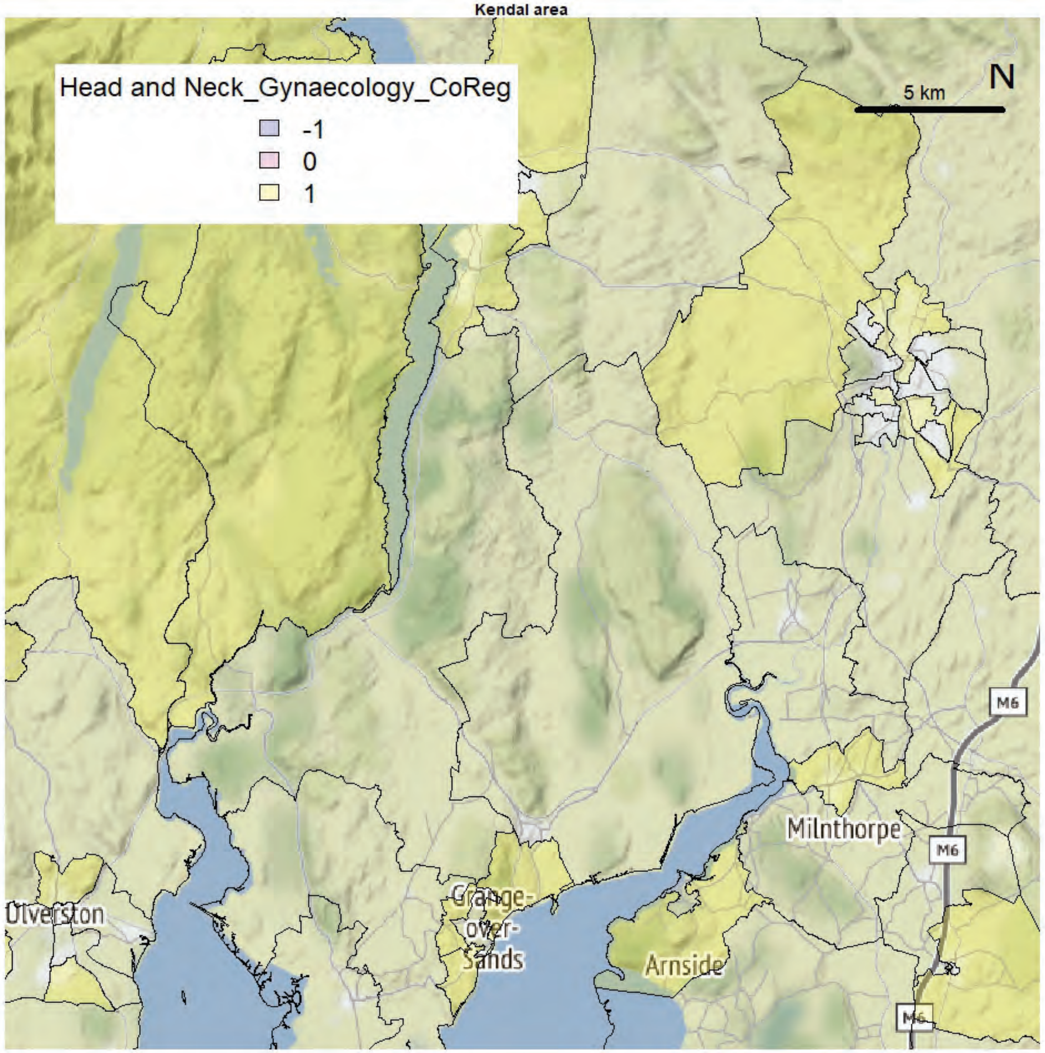
Kendal area
Head and Neck_Gynaecology_CoReg
-1
0
1
5 km
N
M6
Milnthorpe
Ulverston
Grange-over-Sands
Arnside

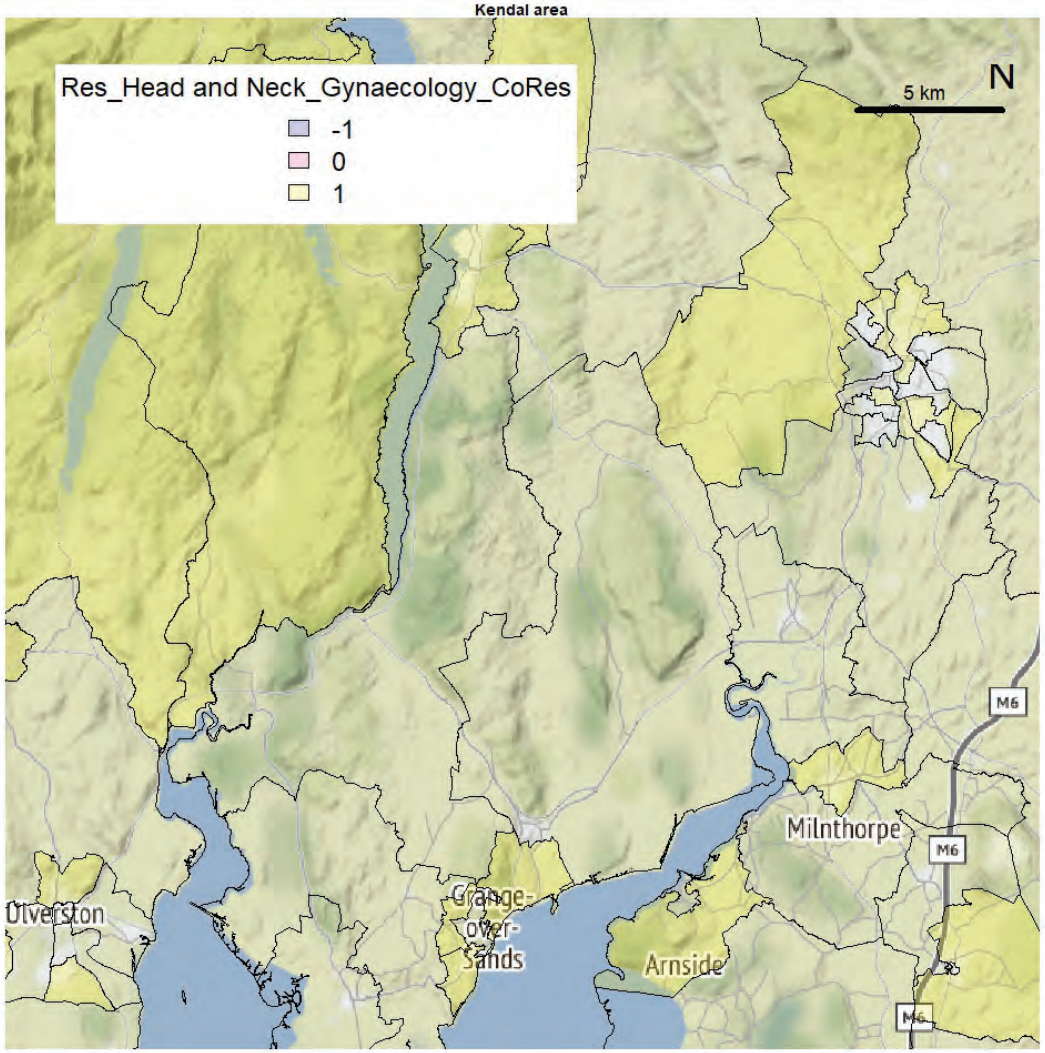
Kendal area
Res_Head and Neck_Gynaecology_CoRes
-1
0
1
5 km
N
M6
Milnthorpe
Ulverston
Grange-over-Sands
Arnside

Supplementary Information 12: Maps interpretation

Risk and relative standard errors of a cancer type.

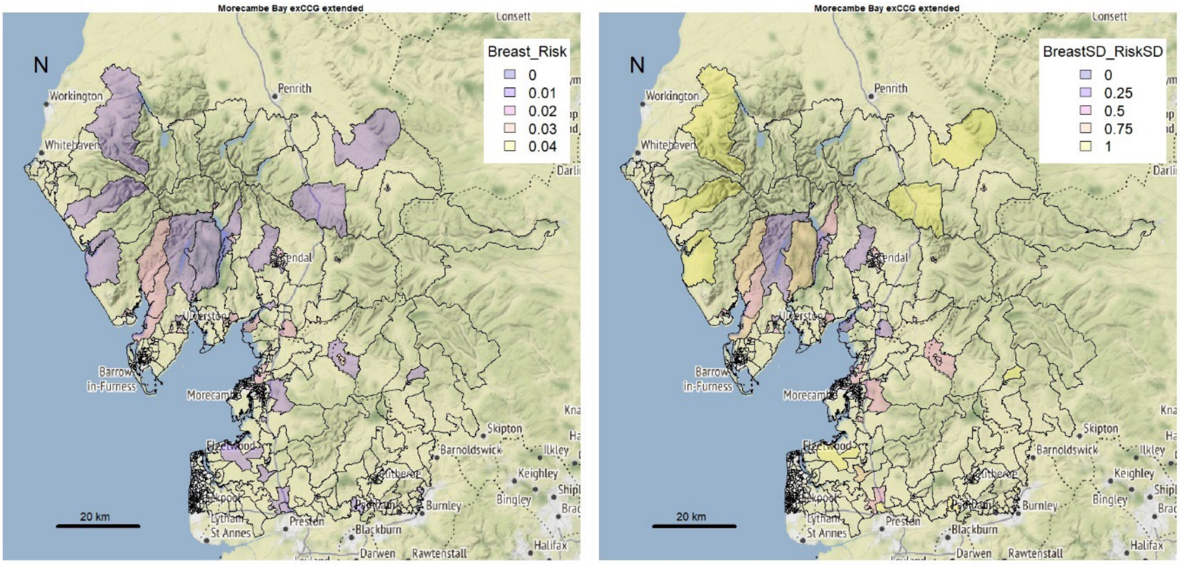

In this example for the Morecambe Bay ex CCG extended study area, on the left is shown the risk (in this case for breast cancer: Breast_Risk) and on the right the relative errors (BreastSD_RiskSD). Since the results are from a Bayesian model, the risk is represented by the average posterior incidence per LSOA unit (each polygon in the map is an LSOA unit). The errors representing the uncertainty in the data and model parameters is the posterior standard deviation of the incidence.

Left panel. Risk is measured as incidence, therefore 0.04 is equivalent to a model estimate of 4 cases of breast cancer any 100 women every 6 years for the LSOA area (each polygon is an LSOA unit).

Right panel. The standard errors are scaled on the overall uncertainty. Therefore a value of 1 is representing maximum uncertainty around the incidence value.

Age standardised risk of a cancer type.

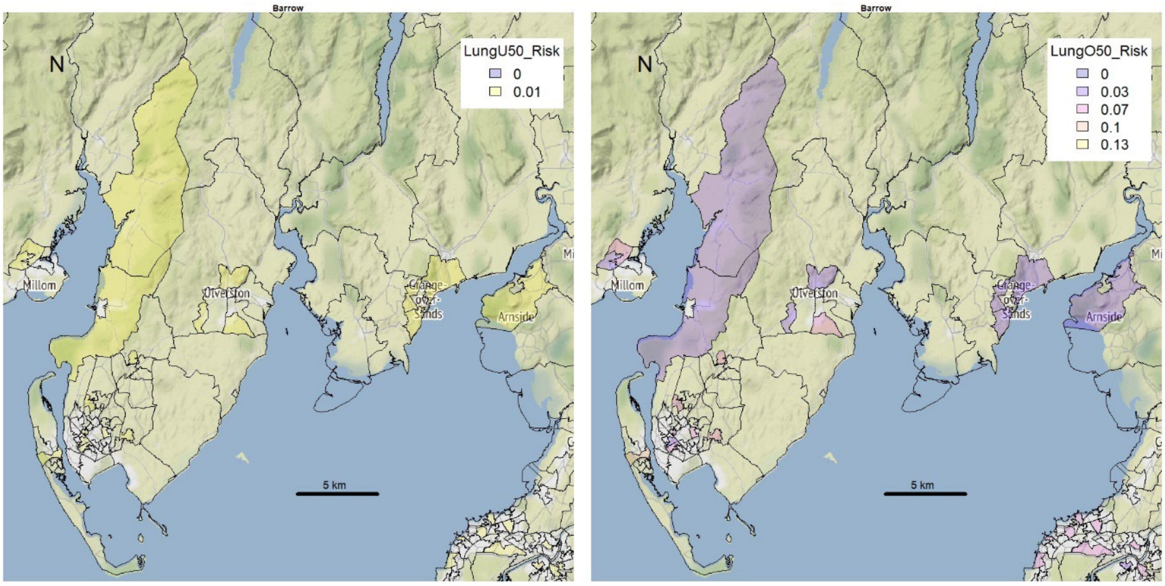

In this example for Barrow in Furness, on the left is shown the standardised risk for lung cancer in under 50 years old (LungU50_Risk) and on the right for the 50 years old and over (LungO50_Risk). Since the results are from a Bayesian model, the risk is represented by the average posterior incidence per LSOA unit (each polygon in the map is an LSOA unit).

Risk is measured as incidence. A value of 0.1 on the right panel means 1 case of lung cancer every 10 people of 50 years old and over every 6 years for the LSOA area (each polygon is an LSOA unit).

Spatial co-regionalisation between any two cancer types.

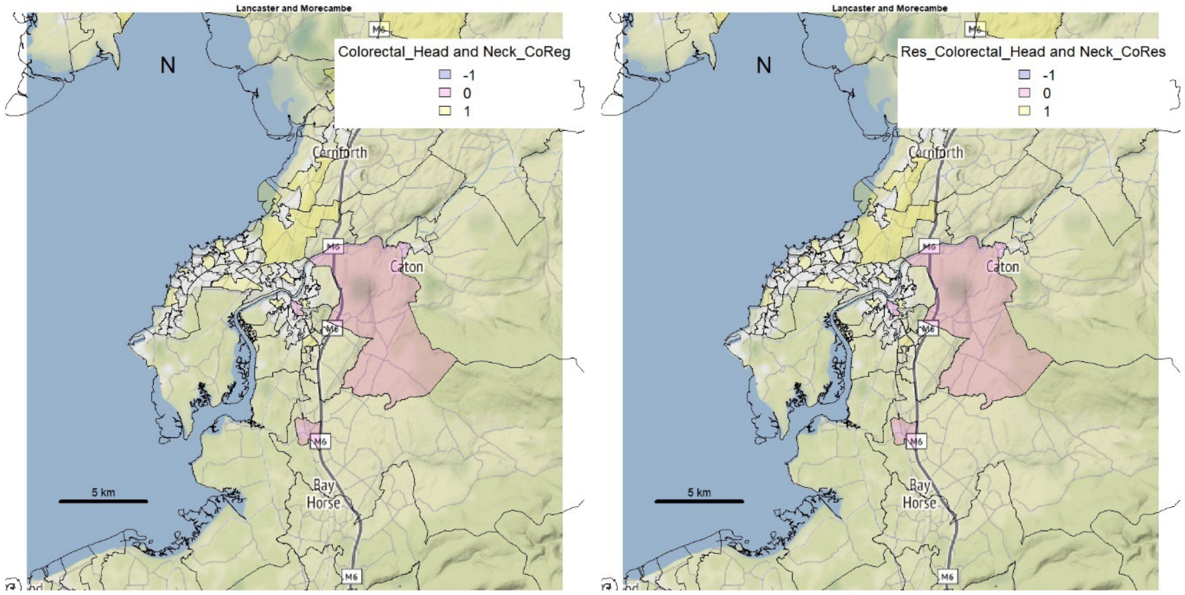

In this example for Morecambe and Lancaster, on the left is shown co-regionalisation between the risks of colorectal and head and neck cancer, while on the right are shown the co-regionalisation between the model's residuals between the same two cancer types.

Legend: -1, one cancer type has large incidence relatively to the low incidence of the other cancer; 0,no significant co-regionalisation between the two cancers; 1, the two cancer types have similar patterns in the high or low incidences.

The positive residual co-regionalisation (on the right panel) is indicating that the two cancers are likely sharing common factors (e.g. socioeconomic, demographic, environmental) that were not included in the model.

Supplementary Information S13: Model validation.

Model validation results indicate a precise joint model with an error of maximum of 2.5 cases on average per time period (Upper GI, mean squared error) (Table 4). Similar results were obtained when leaving out 10% of the data (root mean squared error and mean squared deviation ratio). The best fitting models based on the DIC and WAIC statistics were for the cancer types highly correlated to each other: gynaecology, haematology, head and neck and upper GI as shown elsewhere (1). The DIC and WAIC results were consistent, although for the latter upper GI was the best fitting model (instead of haematology for DIC).

Supplementary Table. Joint model validation statistics for all data and cross-validated data (indicated with *)

Cancer type	Mean error	Mean squared error	WAIC	DIC	Root Mean squared error*	Mean Squared Deviation Ratio*
Breast	-1	1	-116.19	224.96	1	0.22
Colorectal	-0.8	0.93	-98.17	79.36	0.96	0.55
Gynaecology	-0.8	1.03	-93.71	69.92	1.01	2.02
Haematology	-0.86	0.86	-97.34	68.17	0.93	1.34
Head and Neck	-0.58	0.89	-91.14	68.21	0.94	0.79
Lung	-0.66	0.8	-98.45	128.61	0.89	0.31
Skin	-1.4	2.33	-103.44	156.94	1.52	0.32
Upper GI	-0.46	2.46	-88.62	69.77	1.57	4.92
Urology	-1.05	1.48	-99.56	77.64	1.21	3.3

References

1. Held L, Natario I, Fenton SE, Rue H, Becker N. Towards joint disease mapping. Stat Methods Med Res. 2005;14(1):61-82.

Supplementary Information S14: Extended discussion on significant risk factors found in the study.

In the literature, socioeconomic differences in incidence and survival due to environment, lifestyle, biological effects, access to health care and health seeking behaviour, exist for different cancers even between neighbouring geographical areas (1) and in particular for breast, cervix, lung and malignant melanoma (2). However, this has not been established for childhood leukaemia prevalence in 1993–1994 in England, Wales, and Scotland (3), or colorectal cancer survival between 1991 and 1997 in Bolton (North West of England) (4), although other studies have shown an association with survival and uptake of screening (5, 6). An inverse socio-economic gradient was found for non-melanoma skin cancer rates in different studies covering Ireland (1994-2003) (7), England (2006-2008) (8), and the whole of the UK (2004-2010) (9), and for breast cancer incidence in Wales (1985-2012)(10). In addition, age is a common risk factor for cancers (11).

In this research, for cancer types where age was not found to be a statistically significant factor, frailty or comorbidities may have proxied the age effect. Apart from breast cancer type, at least one ethnic group was always associated to the rest of the cancer types. In terms of ethnicity, the present findings are in agreement with other research for the head and neck (12), and urology cancer types (13), although for other cancer types the present findings are novel. Smoking has been confirmed one of the major risk factors (14), and consistent with other research, for lung cancer (15) and head and neck cancer (16). Interestingly, smoking was found to be protective for the upper GI and gynaecology cancer types, both explained by frailty (that appeared only in these two cancers) which may have masked the effect of smoking (16). The presence of comorbidities as risk factor for most of the cancer types (but not for the skin cancer), has been described for different cancers elsewhere as potential diagnostic driver or because of the interlink between comorbidities, socio-economic status and behaviour (17). For example, diabetes may represent the effects of obesity (a variable not available in this study), which itself is linked to several comorbidities and cancers (18).

Only one socio-economic variable was selected among the many considered in this study (self employed with one parent working), but it was shown that most of the selected variables were correlated with other socio-economic and health factors (19). Deprivation was found to be not significant, in contrast with the large amount of literature linking deprivation or socio-economic status with cancer diagnosis and survival rates (1, 2, 11, 14, 16, 20-23).

Spatial heterogeneity has been observed in other studies where similar risk factors were considered. For example, age, socioeconomic deprivation, ethnicity, and geographical

region were all significantly associated with an incident diagnosis of liver cancer (from primary care data) at the population level (11, 24); for certain leukaemias and lymphomas in the North West (25); head and neck cancer in UK (16); and for joint modelling of lung, bowel and melanoma cancer (26).

The common spatial range for all cancer types was estimated by the joint model to be 15.2 km (95% CI, 14.6–15.8). This shows a relatively moderate range of influence of the shared spatial component. For example, it allows for statistical dependence (or influence) between cancer types in Lancaster and Morecambe with Arnside and Silverdale, but not between Lancaster and Morecambe and Barrow-in-Furness.

References

1. Phillips A, Kehoe S, Singh K, Elattar A, Nevin J, Balega J, et al. Socioeconomic differences impact overall survival in advanced ovarian cancer (AOC) prior to achievement of standard therapy. Arch Gynecol Obstet. 2019;300(5):1261-70.
2. Shack L, Jordan C, Thomson CS, Mak V, Moller H, Registries UKAoC. Variation in incidence of breast, lung and cervical cancer and malignant melanoma of skin by socioeconomic group in England. BMC Cancer. 2008;8:271.
3. Smith A, Roman E, Simpson J, Ansell P, Fear NT, Eden T. Childhood leukaemia and socioeconomic status: fact or artefact? A report from the United Kingdom childhood cancer study (UKCCS). Int J Epidemiol. 2006;35(6):1504-13.
4. Lyratzopoulos G, Sheridan GF, Michie HR, McElduff P, Hobbiss JH. Absence of socioeconomic variation in survival from colorectal cancer in patients receiving surgical treatment in one health district: cohort study. Colorectal Dis. 2004;6(6):512-7.
5. Mansouri D, McMillan DC, Grant Y, Crighton EM, Horgan PG. The impact of age, sex and socioeconomic deprivation on outcomes in a colorectal cancer screening programme. PLoS One. 2013;8(6):e66063.
6. McCaffery K, Wardle J, Nadel M, Atkin W. Socioeconomic variation in participation in colorectal cancer screening. J Med Screen. 2002;9(3):104-8.
7. Carsin AE, Sharp L, Comber H. Geographical, urban/rural and socioeconomic variations in nonmelanoma skin cancer incidence: a population-based study in Ireland. Br J Dermatol. 2011;164(4):822-9.
8. Wheeler BW, Kothencz G, Pollard AS. Geography of non-melanoma skin cancer and ecological associations with environmental risk factors in England. Br J Cancer. 2013;109(1):235-41.
9. Musah A, Gibson JE, Leonardi-Bee J, Cave MR, Ander EL, Bath-Hextall F. Regional variations of basal cell carcinoma incidence in the U.K. using The Health Improvement Network database (2004-10). Br J Dermatol. 2013;169(5):1093-9.
10. Abdulrahman GOJ. Breast cancer in Wales: time trends and geographical distribution. Gland Surg. 2014;3(4):237-42.
11. Liao W, Coupland CAC, Innes H, Jepsen P, Matthews PC, Campbell C, et al. Disparities in care and outcomes for primary liver cancer in England during 2008-2018: a cohort study of 8.52 million primary care population using the QResearch database. EClinicalMedicine. 2023;59:101969.
12. Moles DR, Fedele S, Speight PM, Porter SR, dos Santos Silva I. Oral and pharyngeal cancer in South Asians and non-South Asians in relation to socioeconomic deprivation in South East England. Br J Cancer. 2008;98(3):633-5.
13. DeChello LM, Gregorio DI, Samociuk H. Race-specific geography of prostate cancer incidence. Int J Health Geogr. 2006;5:59.

14. Edwards R, Pless-Mulloli T, Howel D, Chadwick T, Bhopal R, Harrison R, Gribbin H. Does living near heavy industry cause lung cancer in women? A case-control study using life grid interviews. Thorax. 2006;61(12):1076-82.
15. Tomintz M, Kosar B, Clarke G. smokeSALUD: exploring the effect of demographic change on the smoking prevalence at municipality level in Austria. Int J Health Geogr. 2016;15(1):36.
16. Taib BG, Oakley J, Dailey Y, Hodge I, Wright P, du Plessis R, et al. Socioeconomic deprivation and the burden of head and neck cancer-Regional variations of incidence and mortality in Merseyside and Cheshire, North West, England. Clin Otolaryngol. 2018;43(3):846-53.
17. Grose D, Morrison DS, Devereux G, Jones R, Sharma D, Selby C, et al. Comorbidities in lung cancer: prevalence, severity and links with socioeconomic status and treatment. Postgrad Med J. 2014;90(1064):305-10.
18. Ellaway A, Lamb KE, Ferguson NS, Ogilvie D. Associations between access to recreational physical activity facilities and body mass index in Scottish adults. BMC Public Health. 2016;16:756.
19. Chaturvedi N. Ethnicity as an epidemiological determinant—crudely racist or crucially important? International Journal of Epidemiology. 2001;30(5):925-7 %@ 0300-5771.
20. Bithell JF, Murphy MF, Stiller CA, Toumpakari E, Vincent T, Wakeford R. Leukaemia in young children in the vicinity of British nuclear power plants: a case-control study. Br J Cancer. 2013;109(11):2880-5.
21. Chambers AC, Dixon SW, White P, Williams AC, Thomas MG, Messenger DE. Demographic trends in the incidence of young-onset colorectal cancer: a population-based study. Br J Surg. 2020;107(5):595-605.
22. Rogers SN, Staunton A, Girach R, Langton S, Lowe D. Audit of the two-week pathway for patients with suspected cancer of the head and neck and the influence of socioeconomic status. Br J Oral Maxillofac Surg. 2019;57(5):419-24.
23. Rafiq M, Hayward A, Warren-Gash C, Denaxas S, Gonzalez-Izquierdo A, Lyratzopoulos G, Thomas S. Socioeconomic deprivation and regional variation in Hodgkin's lymphoma incidence in the UK: a population-based cohort study of 10 million individuals. BMJ Open. 2019;9(9):e029228.
24. Burton A, Balachandrakumar VK, Driver RJ, Tataru D, Paley L, Marshall A, et al. Regional variations in hepatocellular carcinoma incidence, routes to diagnosis, treatment and survival in England. Br J Cancer. 2022;126(5):804-14.
25. McNally RJ, Alston RD, Cairns DP, Eden OB, Birch JM. Geographical and ecological analyses of childhood acute leukaemias and lymphomas in north-west England. Br J Haematol. 2003;123(1):60-5.
26. Jahan F, Duncan EW, Cramb SM, Baade PD, Mengersen KL. Multivariate Bayesian meta-analysis: joint modelling of multiple cancer types using summary statistics. Int J Health Geogr. 2020;19(1):42.

Supplementary Table S8. Cancer screening in England, North West and Lancashire and relative cancer rates (sources: https://digital.nhs.uk/data-and-information/publications/statistical/breast-screening-programme; https://digital.nhs.uk/data-and-information/publications/statistical/cervical-screening-annual; https://www.gov.uk/government/publications/bowel-cancer-screening-annual-report-2021-to-2022#full-publication-update-history). Coverage expressed in % and rates in per 1,000 individuals.

**Region/year**	**Breast screening coverage[1]**	**Breast cancer rate[3]**	**% Breast non or micro invasive**	**Cervical cancer coverage[4]**	**Cervical cancer rate[5]**	**% Cervical low grade**	**Bowel cancer coverage[6]**	**Bowel cancer rate[6]**
England								
2016-17	75.4	8.4	47.6	21.3	3.5	39.7	NA	NA
2017-18	74.9	8.4	48.9	21.0	3.3	39.5	NA	NA
2018-19	74.6	8.8	48.8	22.2	3.3	40.3	NA	NA
2019-20	74.2	8.4	49.4	20.5	3.3	45.2	NA	NA
2020-21	64.2	9.1	51.4	19.2	3.5	49.7	NA	NA
2021-22	65.3	9.2	52.5	21.8	3.6	51.7	70.3	2.0
2022-23	66.4	8.7	52.7	21.0	3.6	51.5	NA	NA
North West								
2016-17	72.5	8.6	48.0	21.2	3.9	42.0	NA	NA
2017-18	72.8	8.6	49.1	21.1	3.8	41.7	NA	NA
2018-19	73.2	9.0	48.8	22.9	3.6	44.4	NA	NA
2019-20	72.3	8.2	50.4	21.2	3.2	49.4	NA	NA
2020-21	62.0	9.3	54.9	19.5	3.5	49.4	NA	NA
2021-22	62.8	9.2	52.9	21.8	3.4	50.7	68.0	2.3
2022-23	65.4	8.7	53.0	21.1	3.0	52.5	NA	NA
Lancashire[2]								

2016-17	74.1	9.1	52.0	21.0	3.4	42.7	NA	NA
2017-18	73.9	9.2	47.3	21.0	3.6	44.2	NA	NA
2018-19	75.0	8.8	42.6	22.7	2.6	45.1	NA	NA
2019-20	74.1	8.3	45.4	21.0	2.7	48.8	NA	NA
2020-21	60.6	10.2	44.2	19.2	3.3	48.9	NA	NA
2021-22	63.5	9.6	57.3	21.8	3.3	48.0	NA	NA
2022-23	68.5	9.8	50.7	21.2	3.1	52.4	NA	NA

[1] Relative to 53-70 age group. [2] Breast cancer rates are relative to North Lancashire and South Cumbria. [3] Rates for 45 years old and over, considering invasive and non-invasive breast cancer. [4] 25-64 age group. [5] Dyskaryosis - low to severe. [6] data relative to 2020-2022 period for age 50 to 74.
